# Exportin-5 binding precedes 5′- and 3′-end processing of tRNA precursors in *Drosophila*

**DOI:** 10.1016/j.jbc.2024.107632

**Published:** 2024-08-02

**Authors:** Ze Li, Junko Iida, Masami Shiimori, Katsutomo Okamura

**Affiliations:** 1 Graduate School of Science and Technology, Nara Institute of Science and Technology, Ikoma, Nara, Japan; 2 Temasek Life Sciences Laboratory, National University of Singapore, Singapore, Singapore

**Keywords:** RNA export, Drosophila, exportin-5, tRNA processing, CLIP, intronless gene

## Abstract

Exportin5 (Exp5) is the major miRNA nuclear export factor and recognizes structural features of pre-miRNA hairpins, while it also exports other minihelix-containing RNAs. In *Drosophila,* Exp5 is suggested to play a major role in tRNA export because the gene encoding the canonical tRNA export factor Exportin-t is missing in its genome. To understand molecular functions of fly Exp5, we studied the Exp5/RNA interactome in the cell line S2R + using the crosslinking and immunoprecipitation (CLIP) technology. The CLIP experiment captured known substrates such as tRNAs and miRNAs and detected candidates of novel Exp5 substrates including various mRNAs and long non-coding RNAs (lncRNAs). Some mRNAs and lncRNAs enriched PAR-CLIP tags compared to their expression levels, suggesting selective binding of Exp5 to them. Intronless mRNAs tended to enrich PAR-CLIP tags; therefore, we proposed that Exp5 might play a role in the export of specific classes of mRNAs/lncRNAs. This result suggested that *Drosophila* Exp5 might have a wider variety of substrates than initially thought. Surprisingly, Exp5 CLIP reads often contained sequences corresponding to the flanking 5′-leaders and 3′-trailers of tRNAs, which were thought to be removed prior to nuclear export. In fact, we found pre-tRNAs before end-processing were present in the cytoplasm, supporting the idea that tRNA end-processing is a cytoplasmic event. In summary, our results provide a genome-wide list of Exp5 substrate candidates and suggest that flies may lack a mechanism to distinguish pre-tRNAs with or without the flanking sequences.

Compartmentalization of eukaryotic cells by various membranes necessitates mechanisms that transport biological materials from the sites of production to the sites of usage. Transcripts encoded by the nuclear genome need to be exported from the nucleus following the completion of nuclear processing steps. To export various classes of RNAs, eukaryotes have multiple transport machineries, each of which is specialized in transporting specific classes of cargo RNAs by recognizing molecular features that define the classes (1).

Most mRNAs are exported following nuclear processing including 5′-capping, splicing, and polyadenylation, through binding by the transcription-export (TREX) complex and NXF1 (2). TREX is recruited to mRNA precursors during splicing by the spliceosome with help of the cap-binding protein and therefore distinguishes mRNAs from other classes by the presence of exon-intron structures and the 5′-cap.

Nonprotein coding RNAs (ncRNAs) of different classes are also exported by specific machineries that recognize their molecular features. With the exception of 5S rRNA, rRNAs are transcribed as a polycistronic rRNA precursor, which is processed into individual mature rRNAs during the ribosome assembly (3). Mature rRNAs are exported to the cytoplasm as a part of mature ribosomes by the CRM1-dependent pathway, a major protein nuclear export pathway, while the involvement of another export receptor Exportin-5 (Exp5) is also suggested (4).

In yeast and mammals, tRNAs are believed to be mainly exported by an export factor Exportin-t (Exp-t) although Exp5 also plays a role (5, 6, 7, 8, 9, 10). Exp-t has a binding pocket that specifically recognizes the CCA-tail of tRNA molecules, which are the hallmark of processed tRNA molecules. The 5′-leader and 3′-trailer sequences of pre-tRNAs are removed by RNase P and RNase Z, respectively, and then the CCA-tail is added to the 3′-end (6, 8, 9, 10, 11, 12). The degree of contribution to tRNA export by Exp5 appears to vary depending on the organism, ranging from no detectable contribution in plants (13) to strong dependence in *Drosophila*, where Exp-t ortholog is missing in the genome (14).

The best studied cargoes of Exp5 would be miRNA precursors. The short hairpin processing intermediates of miRNAs produced by the nuclear RNase III enzyme Drosha needs to be exported to the cytoplasm for the subsequent processing step by the cytoplasmic RNase III Dicer (2). The hallmarks of Drosha products (hairpin-like structure with ∼20 nt stem and 2nt-3′ overhang) are recognized by the baseball mitt-like structure of the Exp5 to selectively export the pre-miRNAs in a RanGTP-dependent manner (15). The binding of Exp5 appears to precede processing by Drosha, as the sequences flanking the hairpin are also bound by Exp5 in a RanGTP-independent manner to facilitate miRNA processing in human cells (16). On the other hand, Exp5 is not essential for miRNA production as demonstrated by Exp5 knockout, while strong reductions of mature miRNA species were seen, which may imply the presence of an alternative export mechanism (16, 17).

Exp5 is also involved in the export of various types of RNAs including 7SL RNA and adenovirus VA1 RNA by recognizing their characteristic structures (16, 18, 19, 20, 21, 22, 23). The crystal structure of human Exp5 complexed with a pre-miRNA hairpin and the GTP-bound form of Ran GTPase clarified the geometry of the binding (15). The stem region of the pre-miRNA hairpin is held by the baseball mitt-like structure consisting of 20 repeats of the HEAT domain, resembling the structures of other importin ß family proteins. The 3′ overhang is recognized by a tunnel-like structure formed by two of the HEAT repeats, consistent with the binding preference of Exp5 for RNA molecules with short dsRNA regions with 3′ overhangs (19).

Although Exp5 and Exp-t share very similar substrate preferences (12, 15), they still exhibit clear differences. Exp5 facilitates nuclear export of eEF1A, a factor that delivers aminoacylated tRNAs to ribosome, presumably through the formation of a complex containing Exp5-tRNA-eEF1A, but Exp-t does not because Exp-t specifically binds the free CCA-tail that is not bound by eEF1A (24, 25).

Intrigued by the absence of an Exp-t ortholog in the *Drosophila* genome, we investigated the breadth of binding specificity of fly Exp5 using PAR-CLIP (26). We detected PAR-CLIP tags from known substrates including miRNAs, tRNAs, and 7SL RNA, confirming published results. Interestingly, PAR-CLIP tags from tRNA loci were predominantly derived from tRNA precursor sequences rather than the mature tRNAs, challenging the common notion that only end-processed tRNAs are recognized by the export factors at least in *Drosophila*. Indeed, we found that pre-tRNAs were exported to the cytoplasm prior to their end-processing. We also found that our PAR-CLIP library was dominated by tags from a variety of mRNAs and long ncRNAs (lncRNAs), suggesting broader roles of Exp5 in *Drosophila* than previously anticipated.

## Results

### Exp5 PAR-CLIP reveals a large repertoire of Exp5 substrates

**Figure 1:**
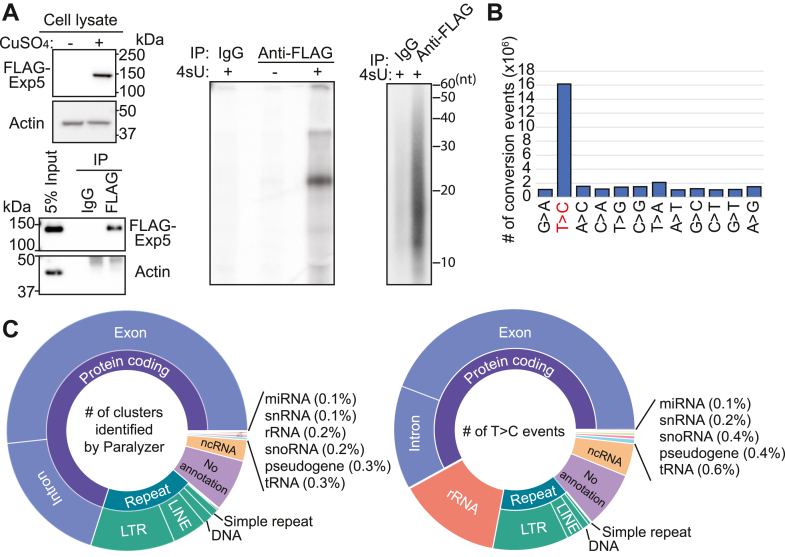
**Overview of Exp5 PAR-CLIP results.***A*, expression of FLAG-tagged Exp5 in S2-R+ cells. S2-R+ cells were stably transfected with a plasmid containing a FLAG-tagged Exp5 coding sequence after the metallothionein promoter (clone LD26789). Total cell extracts were prepared from cells incubated in the presence or absence of 1 mM CuSO_4_ to induce the expression of Exp5-FLAG. The protein sample was run on a 10% SDS-PAGE gel and probed with an anti-FLAG and actin antibodies (*Upper left* panels). Immunoprecipitation of FLAG-Exp5 was verified by analyzing the input and immunoprecipitated samples by Western blotting using anti-FLAG and actin antibodies (*Lower left* panels). Immunoprecipitated Exp5 that was crosslinked with RNA bearing radioactive 5′-phosphate was analyzed on 10% SDS-PAGE and visualized by autoradiography (*Middle panel*). A strong band was only seen when cells were incubated with 4sU and irradiated with 365 nm UV light. The third gel picture shows the autoradiogram of the 15% denaturing Urea-PAGE that was used to extract RNA fragments eluted from the excised SDS-PAGE gel piece (*Right panel*). *B*, frequency of nucleotide change events. Each type of nucleotide substitution was counted on PAR-CLIP reads as compared with the reference sequence where PAR-CLIP reads were mapped and shown in the bar chart. Note that T-to-C substitution events dominated the PAR-CLIP library, indicating successful enrichment of RNA fragments crosslinked with Exp5 peptides. *C*, pie charts of genomic locations where PARalyzer-identified clusters (*left*) or T-to-C substitution events (*right*) were mapped. miRNA constituted a small fraction of the PAR-CLIP library.

To comprehensively catalog the Exp5 substrates in *Drosophila*, we constructed a PAR-CLIP library from S2-R+ cells. We used cells stably transfected with a plasmid encoding FLAG-HA-tagged full-length Exp5 under the control of the metallothionein promoter, whose activity could be controlled by various divalent cations (Fig. 1*A*, upper left) (27). The expressed protein could be efficiently purified by the anti-FLAG antibody (Fig. 1*A*, lower left). To perform PAR-CLIP, cells expressing FLAG-HA-Exp5 were incubated in the presence of 2 mM 4-thiouridine (4sU) for 48 h and then irradiated with 0.15 J/cm^2^ 365 nm UV. Strong radioactive signals were observed in a manner dependent on the precipitation of tagged Exp5 and the incubation with 4sU, indicating the successful isolation of RNA fragments covalently linked to Exp5 (Fig. 1*A*, middle). RNA fragments were released from proteins by proteinase K treatment and their size was ∼15-35 nt as estimated on a denaturing gel, presumably reflecting the size of Exp5 footprints on the substrate RNA molecules (Fig. 1*A* right).

Illumina sequencing of the resulting PAR-CLIP library yielded ∼47 million reads mappable to the dm6 genome allowing up to two mismatches (Table S1). Most frequent conversions were confirmed to be T-to-C, which is the hallmark of cDNA molecules that were reverse-transcribed through a 4sU-modified nucleotide crosslinked with a peptide (26). These results suggested that our PAR-CLIP library successfully enriched RNA tags directly bound by Exp5 (Fig. 1*B*). The Paralyzer pipeline (28) detected ∼112 thousand clusters derived from various RNA molecules belonging to a wide range of RNA classes including mRNAs, lncRNAs, miRNAs, and tRNAs (Fig. 1*C*, left; Table S2). Although the best studied substrates were pre-miRNAs, we found that the clusters were mostly located in coding mRNA sequences and a very small fraction (0.01%) of clusters corresponded to miRNA regions. It was possible that fewer clusters were detected for miRNA genes because there were a smaller number of miRNA genes in the genome and the abundance of PAR-CLIP reads supporting each cluster was not considered in this analysis. Therefore, we counted the T-to-C conversion events within clusters in each category, but the proportions of the categories remained similar, with the exception of rRNAs (Fig. 1*C* right).

The results suggested that Exp5 might have a broader substrate specificity and be involved in the export of a variety of molecules. However, we also note that the quantity of the PAR-CLIP reads could be strongly affected by multiple factors including accessibility of 4sU and adjacent amino acids for UV crosslinking, presumably reflecting the geometry of the protein–RNA complexes (29).

### Binding of Exp5 to miRNA precursors

**Figure 2:**
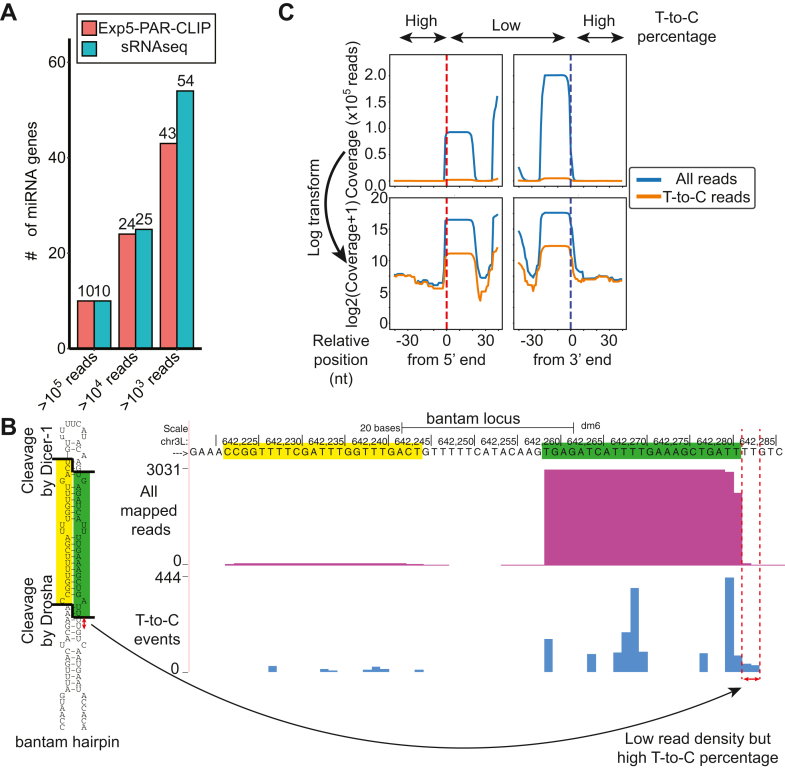
**PAR-CLIP reads from miRNA loci.***A*, PAR-CLIP reads were recovered from most of the abundantly expressed miRNAs in S2-R+ cells. miRNA genes were binned based on the read counts in the Exp5 PAR-CLIP or regular sRNAseq libraries and the number of miRNAs in each bin was plotted. *B*, representative example of PAR-CLIP read mapping patterns at miRNA loci. The bantam locus is shown. The y-axes of the first and second tracks show the normalized (reads per million mapped reads) read density and raw T-to-C event count, respectively. The majority of reads corresponded to their mature 5p and 3p species with very low T-to-C frequencies. On the other hand, reads extending to outside of the hairpin at the 3′ end had relatively high T-to-C frequencies, suggesting that reads that did not correspond to the abundant mature species could be more confidently considered as genuine PAR-CLIP tags copurified with Exp5. *C*, metagene plots of 50 most abundantly expressed miRNAs. The genes were selected based on expression levels in the regular sRNAseq libraries and those flanked by mRNA exons or rRNA precursors were removed. Reads were aligned at the 5′ or 3′ end of the pre-miRNA hairpin and the sum of the read densities is shown. Charts are shown with or without the logarithmic Y-scale. Density plots for individual genes are shown in Supplementary PDF1. Consistent with (*B*), high peaks were present at the mature miRNA positions, but the T-to-C substitution frequency was very low in these regions. On the other hand, much higher T-to-C substitution frequencies were seen in flanking regions although read frequencies were low.

Although Exp5 was expected to bind pre-miRNAs only after their processing by Drosha, a previous CLIP study of human Exp5 recovered reads derived from the regions flanking the pre-miRNA hairpins, suggesting that binding of Exp5 to miRNA hairpins precedes cleavage by Drosha (16). In our PAR-CLIP library, 83% (43 out of 52) of miRNA genes expressed well in S2-R+ cells (1000 reads in the sRNA library) produced PAR-CLIP reads (Fig. 2*A*). A closer look at individual miRNA loci (Supplementary PDF1) revealed that most reads corresponded to the processed 5p- and 3p-species, and the trend was also apparent in the metagene plot (Fig. 2, *B* and *C*). These reads showed very low T-to-C conversion rates, suggesting that these were contaminants of processed small RNAs not bound by Exp5. Although not very abundant, we detected reads extending beyond the cleavage sites by Drosha with a high percentage of T-to-C conversions (Fig. 2*C*), consistent with the notion that the Exp5 binding to primary miRNA transcripts before cleavage as suggested in a previous study (16).

Our results supported the notion that the binding of Exp5 to pre-miRNAs before the cleavage by Drosha and suggested that the phenomenon is conserved in *Drosophila*.

### Fly Exp5 binds 7SL RNA

**Figure 3:**
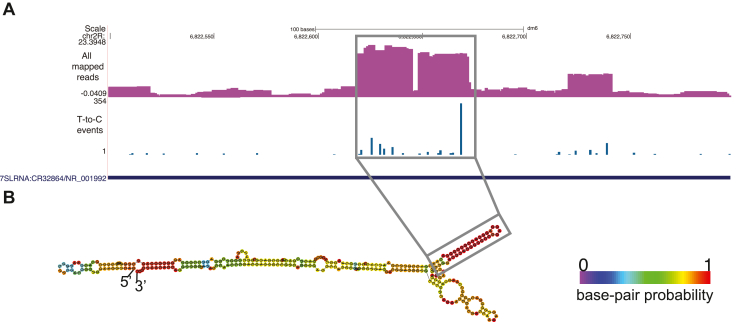
**Exp5 binding to 7SL RNA is conserved in *Drosophila*.***A*, UCSC screenshot of the 7SL RNA locus. The *upper* and *lower* tracks show the normalized (reads per million mapped reads) read density of all reads and the row count of T-to-C substitution events at each T-nucleotide, respectively. *B*, predicted secondary structure of the *Drosophila* 7SL RNA. The structure was predicted by the Vienna RNAfold server, and the color shows the base-paring probability. The *rectangle* shows the hairpin with a high frequency of T-to-C substitutions, suggesting interactions with Exp5.

Previous studies uncovered 7SL RNA to be a substrate of Exp5 in humans (16, 23). We were curious to see whether this binding was conserved in *Drosophila*. Paralyzer detected several clusters across the 7SL RNA locus (Fig. 3*A*), suggesting that 7SL RNA was also bound by Exp5 in *Drosophila*. The conversion events were concentrated at the helix 6 near the conserved SRP19-binding site (30, 31), which was unexpected since the Exp5 prefers structured RNA molecules with 3′ overhangs (15, 18, 23) (Fig. 3*B*). However, we note that accurate mapping of binding sites between RNA molecules and RNA-binding proteins is difficult to achieve by CLIP analysis and requires independent assays such as structural or mutational analysis. The geometry of Exp5–RNA complexes has been studied with only a small number of substrates, and the finding that PAR-CLIP signals were mapped to locations distant from the 3′ end prompts further investigation.

### Fly Exp5 binds various mRNAs and mRNA-like ncRNAs

**Figure 4:**
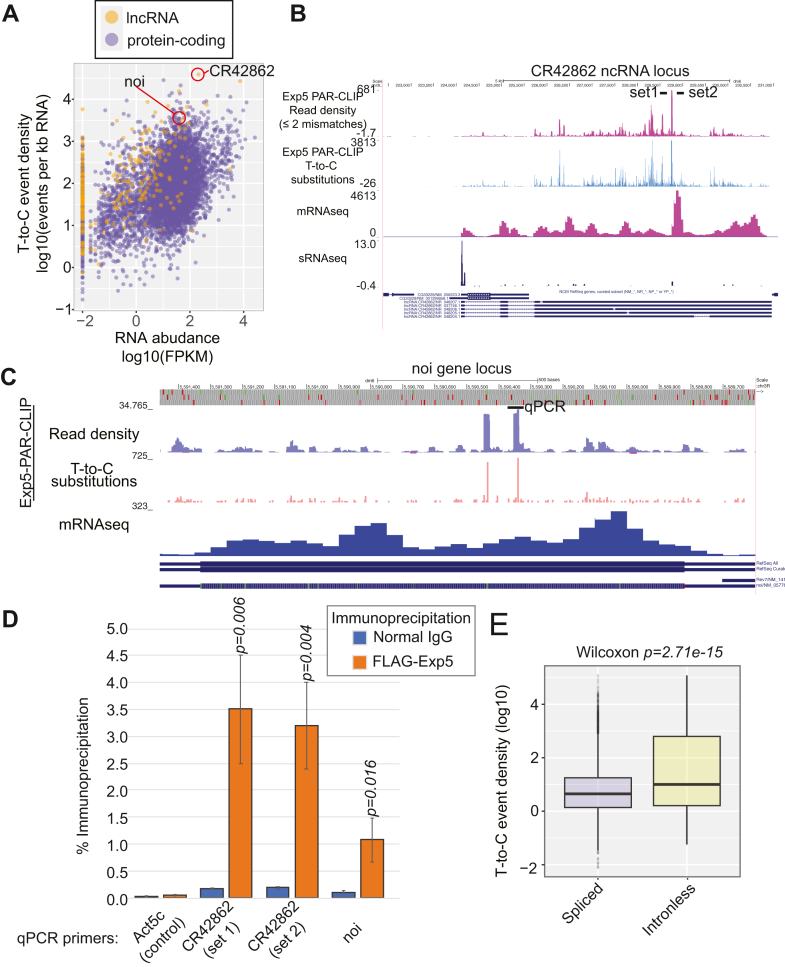
**Exp5 PAR-CLIP tags from mRNAs and lncRNAs.***A*, subsets of mRNAs and lncRNAs enriched PAR-CLIP reads. Counts of PAR-CLIP reads containing T-to-C substitutions in PARalyzer-detected clusters that fell within annotated exons were counted and normalized for the effective transcript size estimated by the published mRNAseq data from S2-R+ cells (33) (T-to-C event counts per kb mRNA, Table S4). The T-to-C event density was plotted against the transcript abundance estimated by the mRNAseq dataset. mRNAs and lncRNAs are shown as *purple* and *orange dots*, respectively. The list of genes and values can be found in Table S5. *B*, UCSC screenshot of the CR42862 lncRNA locus. *C*, UCSC screenshot of the noi intronless gene locus. *D*, the FLAG-Exp5 complex was purified from S2-R+ cells expressing FLAG-Exp5 using a FLAG-antibody and RNA was extracted from the purified complexes and the input sample. The RNA samples were reverse-transcribed, and the targets were quantified by qPCR. The positions of the primer targets used for CR42862 and noi are shown in panels B and C. Act5C mRNA, which showed a very low T-to-C density/FPKM value (Table S4), was used as a negative control. The immunoprecipitation rate (%) was calculated using Cq values from the input and IP samples for each primer set, and values of six replicates from two biological replicate samples were used to calculate averages and SDs (error bars). The *p*-values were calculated by comparing the precipitation efficiency of noi or CR42862 in the FLAG-IP and that of Act5C by using *t* test (unpaired, two-tailed). The TPM and T-to-C density values of the three genes (noi, CR42862, and Act5C) are highlighted with *red* letters in Table S5. *E*, PAR-CLIP reads are enriched in intronless genes. The ratio between T-to-C substitution density and FPKM of mRNAseq data was calculated for each gene, and their distributions were shown for intronless genes (517 genes, Table S5) and spliced protein coding genes (5722 genes). The *p*-value was calculated by the Wilcoxon test. qPCR, quantitative polymerase chain reaction.

While the main role for Exp5 known so far is nuclear export of structured short RNA species, Exp5 has also been suggested to play a role in mRNA transport (32). As we detected a large number of CLIP tags from mRNAs and mRNA-like ncRNAs, we were interested to test whether the tags were preferentially derived from certain genes or they were proportional to the expression levels of the cognate transcripts. When the number of PAR-CLIP reads were plotted against the abundance of transcripts estimated by a published mRNAseq study (33), some genes showed higher PAR-CLIP tag densities than the average (Fig. 4*A*. For full gene names and values, see Table S4 and for mapping data, use the UCSC genome browser tracks.). Figure 4*B* shows the CR42862 locus as an example of a nuclear ncRNA highly expressed in amnioserosa cells in developing fly embryos (34). The CLIP tags were distributed across the length of transcripts (Fig. 4*B*), and the patterns were not consistent with the hypothesis that Exp5 interacts only with structured regions. The protein-coding gene noi also caught our attention because of its high density of T-to-C substitution events (Fig. 4, *A* and *C*). To verify these results by an independent method, coprecipitation of these transcripts was quantified by reverse transcription-quantitative polymerase chain reaction in the immunoprecipitated FLAG–Exp5 complex (Fig. 4*D*). Both CR42862 and noi transcripts, but not the control transcript Act5c mRNA, were specifically enriched in the immunoprecipitations using FLAG-antibody compared to that with normal IgG, validating the PAR-CLIP results. The noi gene lacked introns therefore conceivable to depend on a noncanonical mRNA export pathway (35). We compared the CLIP-tag enrichment factors between intronless genes (Table S5) and spliced genes and found that intronless genes tended to enrich more CLIP tags (Fig. 4*E*).

The results suggested a specialized export machinery involving Exp5 that might play a role in intronless mRNA biogenesis, but further investigation will be needed to test such a hypothesis.

### Binding of fly Exp5 to tRNA precursors

tRNAs have been recognized as a class of Exp5 substrates, although Exp-t appears to play the major role in exporting tRNAs in many species (36). As the gene encoding Exp-t ortholog is missing in *Drosophila*, we were interested to study how *Drosophila* Exp5 recognizes tRNA molecules. Consistent with the idea that Exp5 is a major tRNA export receptor, we detected 343 PAR-CLIP clusters identified by Paralyzer at 295 tRNA loci (Table S2).

**Figure 5:**
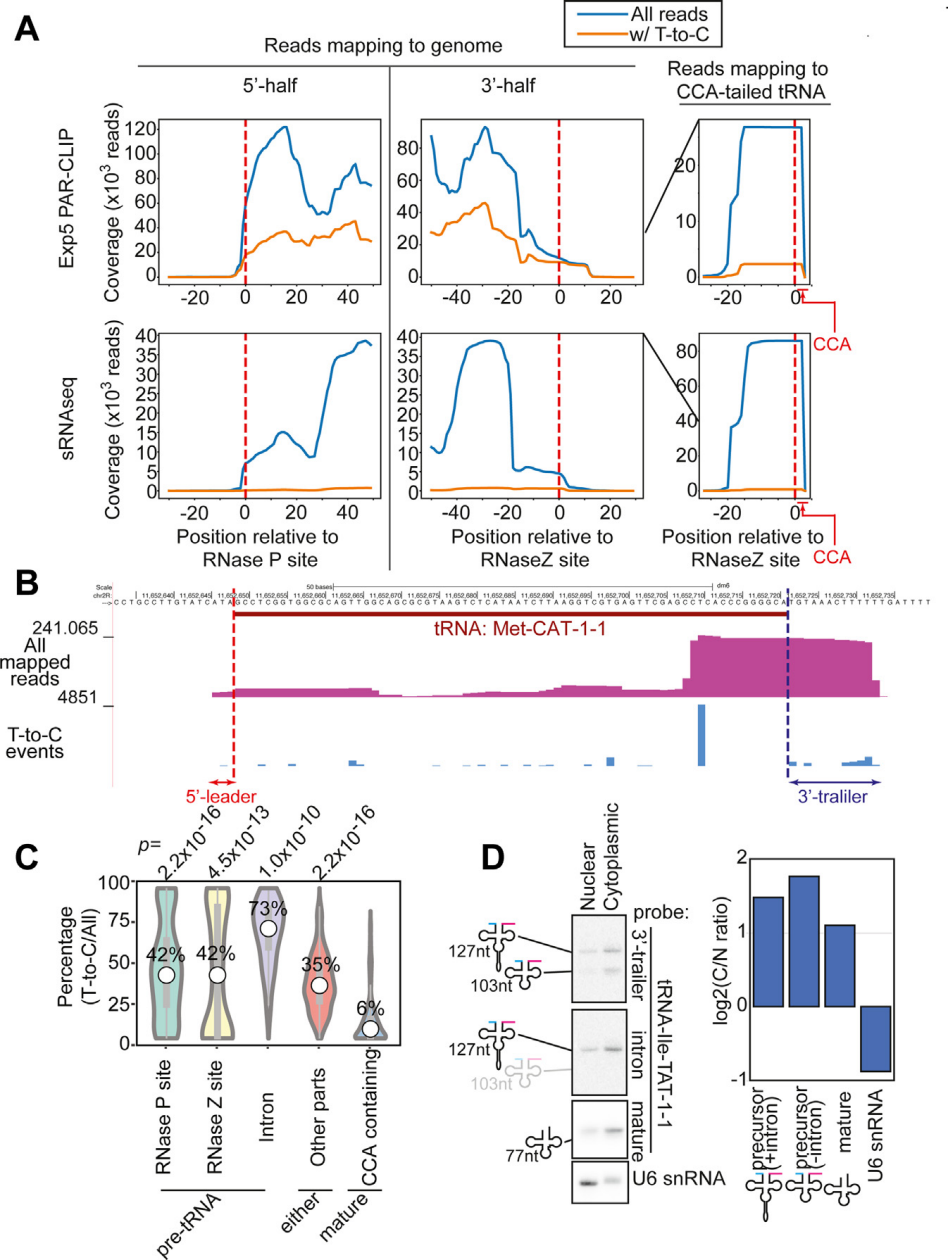
**pre-tRNAs are bound by Exp5.***A*, metagene plots of PAR-CLIP reads (*upper*) or small RNAseq reads (*lower*; data from SRR013547 and SRR013548 combined) at tRNA loci. Only intronless tRNA genes were considered. Reads mapped to tRNA genes were aligned at the 5′ or 3′ end of the mature tRNA and sum of the read densities are shown. To analyze reads derived from end-processed tRNAs, reads mapping to tRNA sequences followed by a 3′-CCA tail sequence were also analyzed by mapping reads ending with CCA to the tRNA + CCA reference sequences. Density plots for individual genes are shown in Supplementary PDF2. *B*, representative tRNA locus. The tRNA-Met-CAT-1-1 locus is shown. The y-axes of the first and second tracks show the normalized (reads per million mapped reads) read density and raw T-to-C event count, respectively. *C*, the percentages of T-to-C containing reads were calculated for each category (spanning RNase Z, RNase P or splice site, inside of tRNA gene body, or containing 3′-CCA tail) for individual genes, and their distributions are shown in the Violin plot. The percentages were significantly higher with reads spanning RNase Z, RNase P, or splice site than with those containing 3′-CCA tail, suggesting that Exp5-PAR-CLIP reads were preferentially produced from unprocessed tRNA precursors. The numbers in the chart are the medians of T-to-C containing read percentages. The *p*-values are Wilcoxon test *p* values compared to the T-to-C percentages of CCA-containing reads. *D*, detection of mature and precursor tRNAs in the cytoplasmic and nuclear fractions. RNA samples from fractions obtained from 1 × 10ˆ6 cells were loaded and bands were detected by probes against the 3′-trailer, intron, and mature sequences of tRNA-Ile-TAT-1-1. U6 snRNA was used as a control of nuclear RNA. The enrichment factors were calculated by dividing the band intensity in the cytoplasmic fraction by that in the nuclear fraction.

To gain insights into the mode of binding between Exp5 and tRNAs, metagene analysis was performed by aligning genes with respect to the mature tRNA 5′- and 3′-ends (Fig. 5*A* upper panel). Unexpectedly, we observed ∼50,000 and ∼10,000 reads extending to the leader and trailer sequences, respectively, raising the possibility that fly Exp5 binding precedes the removal of leader and trailer sequences (Fig. 5*B*). Approximately twenty five thousand reads were found to contain the CCA-tail sequence but they tended to lack the T-to-C substitutions, suggesting that they might be contaminants produced from abundant mature tRNA molecules (Fig. 5*A* upper panel, Table S6).

The percentages of reads containing T-to-C substitutions were similar between reads overlapping the RNase P/Z site (derived from precursors before end processing), those at the intronic regions and those from the tRNA gene body (can be derived from either precursor or mature tRNAs) (Fig. 5*C*, Table S6). On the other hand, lower percentages were seen with reads containing the CCA-tail, suggesting that many of the CCA-containing reads were derived from RNA fragments not bound by Exp5 rather than genuine RNA molecules directly crosslinked to Exp5. tRNAs contain nucleotide modifications at various positions, which could interfere with reverse transcription, and as a result, such modifications might enrich pre-tRNA molecules with no or fewer chemical modifications (37). If this was true, we would find similar read mapping patterns in regular sRNAseq libraries. However, CCA-containing reads were much more abundant (∼80,000) than the genome-mapping reads spanning the RNase Z site (>10,000 reads) in the sRNA libraries, suggesting that such potential random degradation products were mainly derived from the mature tRNA species (Fig. 5*A*, lower panel) and excluding the possibility that the enrichment of pre-tRNA–derived sequences in the Exp5 PAR-CLIP library was a mere artifact (Fig. 5*C*). We also attempted to test this directly by detecting pre- and mature-tRNA species by Northern blotting in Exp5 immunoprecipitations (Fig. S1). We found that pre-tRNA species that contained both trailer and intronic regions were more strongly enriched than the spliced precursor still containing the trailer, consistent with high T-to-C ratio of reads from intronic regions (Fig. 5*C*). Unexpectedly, mature tRNA species were enriched in the Exp5 complex at a similar enrichment factor as the pre-tRNAs before splicing and end-processing (Fig. S1). The difference may stem from the fact that tRNA molecules are known to shuttle between the nucleus and the cytoplasm (5), and the regular IP and PAR-CLIP procedures may capture distinct states of tRNA–Exp5 interactions. The binding dynamics and roles for fly Exp5 in tRNA export needs to be studied further. However, similar enrichment of mature tRNAs and pre-tRNAs again highlights the mechanistic difference from the yeast Los1p, which is selective for end-processed tRNAs (12).

The binding of pre-tRNAs before end-processing to Exp5 predicts that cytoplasmic processing occurs in the cytoplasm in contrast to the established notion (38, 39). In the IP experiment, we also found that intron splicing occurs prior to end-processing although both processes were believed to occur in the nucleus at least in frog eggs (40). Therefore, we sought evidence that these processing events occur in the cytoplasm in fly cells by detecting precursor and mature tRNAs in nuclear and cytoplasmic fractions (Fig. 5*D*). We found both the pre-tRNA with/without the intron and the mature tRNA were similarly enriched in the cytoplasm in contrast to the strong nuclear enrichment of U6 snRNA, supporting the prediction that tRNA splicing and end-processing are cytoplasmic events in *Drosophila*.

In summary, we identified tRNA precursors containing 5′- and 3′-flanking sequences as a class of Exp5 substrates. In contrast to tRNA binding to yeast Exp-t (12), our PAR-CLIP results suggested that fly Exp5 could bind pre-tRNAs before end-processing. Such a preference of Exp5 may be reasonable if RNase P and RNase Z are localized in the cytoplasm in fly cells, similar to those enzymes that were recently reported to localize in the cytoplasm in mammalian cells (41). Indeed, we show that tRNA splicing and end-processing occur in the cytoplasm.

## Discussions

Among the importin-ß superfamily receptors, Exp5 has been characterized as a receptor for short structured RNA molecules (18). The best studied class of substrates are miRNA precursors (16, 20, 21, 22), while it was later shown that Exp5 KO cell lines still produced mature miRNAs at least at some levels (17). In addition, another closely related export factor Exp-t is conserved in many organisms to specifically export CCA-tailed tRNAs, although the gene is lost in *Drosophila* (14). Therefore, we were curious to test the extent of evolutionary conservation of Exp5 functions by studying the substrate specificity of *Drosophila* Exp5.

Our PAR-CLIP results confirmed that previously identified substrates of mammalian Exp5 were bound by fly Exp5, including pre-/pri-miRNAs, 7SL RNA, and tRNAs (14, 16, 23). In addition, we found a variety of mRNAs and mRNA-like noncoding RNAs to be bound by fly Exp5, suggesting that the role of Exp5 might be broader than previously recognized (Figs. 1 and 4). Select mRNAs and lncRNAs showed high enrichments in the PAR-CLIP read density, including those mRNAs that lack introns (Fig. 4), suggesting that intronless mRNAs might have a distinct export machinery involving Exp5. mRNA processing and nuclear export are closely tied processes, and the TREX complex, which is deposited to mRNA precursors during the splicing reaction, facilitates mRNA nuclear export (42). Intronless mRNAs are proposed to transit through the nuclear membraneless structure, nuclear speckle, *via* the binding of SR proteins to the exonic splicing enhancer motifs found on intronless mRNAs, and the deposition of the TREX complex is facilitated by SR proteins in mammals (43, 44). In *Drosophila*, the essential splicing factor dU2AF^50^ binds intronless mRNAs to promote their nuclear export (35). The noi mRNA was found to be bound by dU2AF^50^ while our Exp5-PAR-CLIP also identified strong peaks on the noi mRNA (Fig. 4*C*). We note that our PAR-CLIP and IP-qPCR experiments were performed with overexpressed Exp5 in a cultured cell line. Whether endogenous Exp5 binds intronless mRNAs *in vivo* in flies, whether the binding is conserved in mammals, and whether *Drosophila* Exp5 and dU2AF^50^ cooperate or independently work should be studied in future, to understand the molecular mechanisms of intronless mRNA export.

Another notable class was represented by tRNA precursors. Although the CLIP analysis in theory does not separate the binding events occurring with tRNAs and tRNA fragments (tRFs), a recently discovered family of small regulatory RNAs, we believe the majority of the CLIP-tags to be derived from tRNAs because the IP-Northern blot experiment detected bands corresponding to mature and pre-tRNA lengths as major bands. Therefore, we considered that the CLIP-tags from tRNA loci mainly represent binding between Exp5 and tRNAs, not tRFs, although possible contributions of tRFs should not be excluded. The lack of the Exp-t ortholog in the fly genome raises a question about nuclear export of tRNAs, and a previous study proposed that Exp5 is the major export receptor for tRNAs (14). On the other hand, Exp5 is suggested to bind tRNA molecules in a geometry that is different from that of tRNA binding to Exp-t based on its ability to simultaneously bind tRNA and eEF1a (24, 25). The lack of the CCA-binding pocket supports the notion that Exp5 is unable to distinguish processed tRNAs from their precursors (12, 14). The initial steps of tRNA processing such as the removal of 5′-leaders and 3′-trailers are believed to occur in the nucleus (38, 39). If so, how does the fly cell ensure efficient processing of tRNAs without an export factor distinguishing pre-tRNAs and mature tRNAs? A clue may have arisen from a recent study proposing a new model of tRNA processing in mammals (41). The study showed that RNase P and RNase Z were mainly localized in the cytoplasm and the processing events occurred in the cytoplasm. This challenges the previously proposed model in which these cleavage events occur in the nucleus, a notion mostly based on the results from yeast and *Xenopus* oocyte injection experiments. In addition, because the enzymes involved in tRNA biogenesis also participate in other biological processes that occur in multiple cellular compartments, interpreting the protein/RNA localization results is not a simple task (45).

Taken together, we propose a revised model for the role of Exp5 in fly tRNA biogenesis. In *Drosophila*, knockdown of Exp5 is shown to reduce the tRNA abundance, indicating its roles in tRNA export (14). There may be a quality control mechanism to degrade tRNAs retaining in the nucleus similar to the yeast TRAMP complex–nuclear exosome axis (46, 47), explaining the reduction of tRNA abundance upon Exp5 knockdown. The subcellular localization of RNase P and RNase Z in fly cells needs to be clarified in future experiments, but we speculate the role of fly Exp5 in tRNA biogenesis to be facilitating the evasion of tRNA precursors from the nuclear quality control system by exporting tRNA molecules regardless of the processing status or preferentially binding pre-tRNAs. The exported tRNAs may shuttle back to the nucleus for further processing. By this way, tRNA molecules undergo normal processing and chemical modifications that occur in both nucleus and cytoplasm, while ill-formed tRNA precursors that are not exported properly are targeted by the quality control system. Thus, strict discrimination between CCA-tailed species and tRNA precursors is not essential for efficient tRNA production, as the role for export factors is to facilitate the shuttling of tRNA molecules between the nucleus and the cytoplasm to evade nuclear degradation, rather than ensuring only CCA-tailed species to be exported for the sequential processing in an orderly fashion. This may also explain why Exp-t is not an essential gene even in yeast and why *Drosophila* could lose the Exp-t ortholog without a problem with tRNA processing.

Further studies will be needed to clarify the localization of tRNA processing factors in *Drosophila* in their native states and test the hypothesis regarding the role of Exp5 in tRNA biogenesis. Such studies will shed light on the evolution of tRNA biogenesis and the biological significance of the dual export system involving Exp5 and Exp-t in many species.

## Experimental procedures

### Plasmids and cell lines

S2-R+ cells (DGRC Stock 150; https://dgrc.bio.indiana.edu//stock/150; RRID:CVCL_Z831) were cultured in Schneider’s *Drosophila* medium containing 10% fetal bovine serum and 1% penicillin/streptomycin at 25 °C. The plasmid for expression of FLAG-HA-tagged Exp5 under the control of the metallothionein promoter (FMO06236) was obtained from DGRC (LD26789; DGRC Stock 6165; https://dgrc.bio.indiana.edu//stock/6165; RRID:DGRC_6165), and the plasmid was used for stable transfection of S2-R+ cells. Transfected cells were selected by culturing in the medium containing 150 μg/ml hygromycin. Expression of Exp5 was induced by 1 mM CuSO_4_ for 48 h before cells were harvested.

### PAR-CLIP

PAR-CLIP protocol was adapted from (26). S2-R+ cells stably transfected with the FLAG-HA tagged Exp5 construct were grown in 20 × 15 cm cell culture dishes in 20 ml in the presence of 1 mM CuSO_4_ and 100 μM 4-thiouridine, respectively, and incubated for 48 h at 25 °C. The medium was then discarded and the cells were irradiated with 0.15 J/cm^2^ 365 nm UV in a UV crosslinker (Stratagene). After washing cells with ice-cold PBS, cells were lysed in RIPA buffer (1× PBS, 0.1% SDS, 0.5% deoxycholate, 0.5% NP-40 with Complete EDTA-free protease inhibitor (Roche)). The lysate was passed through a 25G needle four times and precleared by centrifugation at 13,000*g* for 10 min at 4 °C. One microliter of 1000 U/μl RNase T1 was added per 1 ml of lysate and incubated for 15 min in a 22 °C water bath. Six hundred seventy-five microliters of Dynabeads Protein G (Invitrogen) was washed thrice using citrate-phosphate buffer (0.47% citric acid, 0.92% Na_2_HPO_4_, pH 5.0), then bound with 180 μg of FLAG antibody (Wako) for 40 min on a rotator at room temperature. Antibody-bound beads were then washed thrice using citrate-phosphate buffer and twice using RIPA buffer, then incubated with 1 ml lysate for 1 h at 4 °C on a rotator. Beads were washed thrice using IP wash buffer (50 mM Hepes–KOH pH 7.5, 300 mM KCl, 2 mM EDTA, 1 mM NaF, 0.05% NP-40, 0.5 mM DTT, with Complete EDTA-free protease inhibitor (Roche)) and resuspended in one volume of IP buffer, then 1 μl of 1000U/μl RNase T1 was added per μl of IP buffer and incubated for 15 min on a 22 °C water bath. The beads were washed thrice using high salt wash buffer (50 mM Hepes-KOH pH 7.5, 500 mM KCl, 2 mM EDTA, 1 mM NaF, 0.05% NP-40, 0.5 mM DTT with Complete EDTA-free protease inhibitor (Roche)) and once using dephosphorylation buffer (100 mM NaCl, 50 mM Tris–HCl, 10 mM MgCl_2_, 1 mM DTT, pH 7.9). To remove phosphate groups at the 5′ ends of RNA molecules, 60 μl of dephosphorylation buffer and 3 μl of CIP was added and the tubes were incubated for 10 min at 37 °C. The beads were washed twice with phosphatase wash buffer (50 mM Tris–HCl pH 7.5, 20 mM EGTA, 0.5% NP-40) and twice with 1× PNK buffer (50 mM Tris–HCl pH 7.5, 50 mM NaCl, 10 mM MgCl_2_, 5 mM DTT). Beads were resuspended in 80 μl 1× PNK buffer and labeled with 1 μl 6000 Ci/mol γ-ATP and 2 μl PNK for 30 min at 37 °C. Hundred micromolars of ATP was then added to ensure complete phosphorylation of RNA fragments and incubated at 37 °C for 5 min, then beads were washed for 5 times with 1× PNK buffer, and resuspended in 70 μl SDS-PAGE buffer (4% SDS, 250 mM Tris pH 6.8, 16% β-mercaptoethanol, 30% glycerol, 0.006% bromophenol blue). Samples were boiled for 5 min at 95 °C, then the samples were separated on a 6% SDS-PAGE gel. The radioactive Exp5 band was excised and placed in a dialysis tube, and RNAs from the gel piece was eluted for 1 h at 100V in a submarine gel box. 1/10 eluate volume of 10× proteinase K buffer was and 1.2 mg/ml proteinase K were added and incubated for 30 min at 55 °C. RNA was extracted using phenol-chloroform extraction followed by ethanol precipitation and used for library cloning.

To produce deep sequencing libraries, RNA was mixed with equal volumes of 2× RNA loading buffer (Ambion) onto a 10% denaturing polyacrylamide urea gel and the gel fragment in the ∼20-40 nt size range was excised. The gel fragment was crushed using a pipette tip and RNA was eluted from the gel fragment in 400 μl of 0.4 M NaCl overnight at room temperature on a rotator. After the eluted RNA was precipitated and resuspended in water, 3′ sequencing linker (RA3) was ligated to the 3′ end of the RNA and RNA marker using T4 RNA ligase 2, truncated K227Q at room temperature for 4 h. Ligated RNA was then size-separated on a 10% denaturing polyacrylamide urea gel and the gel fragments corresponding to the molecular weight of the ligated RNA marker was excised and eluted. After precipitation and resuspension of the ligated product, 5′ sequencing linker (RA5) was ligated to the 5′ ligated RNA product and RNA marker using T4 RNA ligase at 37 °C for 2 h. Ligated RNA was then size-separated on a 6% denaturing polyacrylamide urea gel and the gel fragment corresponding to the molecular weight of the 5′- and 3′-ligated RNA marker was excised from the gel. The ligated product was then eluted and reverse transcribed with RT primer and Superscript III reverse transcriptase (Invitrogen). PCR amplification was performed on the reverse-transcribed DNA using RP1 forward primer and a barcoded RP reverse primer. The resulting ∼140 nt PCR product was gel-purified on a 6% native TBE PAGE gel and then sent for sequencing at the Duke-NUS core facility.

### Immunoprecipitation and quantification of co-precipitated RNA

Immunoprecipitation was done using S2-R+ cells stably transfected with the CuSO_4_-inducible FLAG-Exp5 plasmid. One millimolar of CuSO4 was added to the medium for 48 h before harvesting cells. Cells were lysed in Hepes-NP40 buffer (30 mM Hepes–KOH [pH 7.3], 150 mM potassium acetate [KOAc], 2 mM magnesium acetate [Mg(OAc)2], 5 mM DTT, 0.1% NP-40, 1 × Complete EDTA-free protease inhibitor (Roche)) and then immunoprecipitated with normal IgG or anti-FLAG-antibody (Fuji Film). The samples were separated for RNA extraction by Trizol-LS (Thermo) and protein extraction by 2xSDS-PAGE sample buffer. Extracted RNA was either directly used for reverse transcription-quantitative polymerase chain reaction using iScript Reverse Transcription Supermix (BioRad) and PowerTrack SYBR Green Master Mix (Thermo) or loaded on a 10% Sequagel (National Diagnostics). Quantitative polymerase chain reaction primers and Northern blotting probes are described in Table S3.

### Nuclear-cytoplasmic fractionation

Nuclear-cytoplasmic fractionation of S2-R+ cells was done according to a method described in a previous study (48). In brief, 2 × 10ˆ7 cells were collected by a centrifugation at 500*g* for 5 minutes, then washed once with PBS. The pellet was resuspended in 500 μl hypotonic buffer (10 mM Hepes, pH 7.9, 1.5 mM MgCl2, 10 mM KCl, 0.5 mM DTT) and kept on ice for 15 min. Then the suspension was centrifuged at 700*g* for 8 min at 4 °C. The pellet and supernatant were used as nuclear and cytoplasmic fractions. RNA was extracted from each fraction by Trizol-LS (Thermo).

### Bioinformatics analysis

The bioinformatics tools and their references are summarized in Table S3. The public libraries of S2-R+ cells (mRNAseq data SRR070266, sRNAseq data SRP000598) made by the modENCODE consortium (33, 49) were downloaded. The Fastq file of the PAR-CLIP library was subjected to pre-processing where the linker sequence (CTGTAGGCACCATCAATC) was clipped and reads shorter than 18 nucleotides were removed using fastx_clipper and then converted to a fasta file using fastq_to_fasta. We first removed reads derived from the stably transfected vector sequence (FMO06236) by collecting reads unmapped to the FMO06236 sequence by bowtie (50) without allowing mismatches because a large number of reads were derived from the Exp5 expression plasmid sequence, which is presumably the contamination of transgene-derived endo-siRNAs (51). After the removal, reads were mapped to the dm6 genome http://www.ncbi.nlm.nih.gov/genome/47. Only the assembled chromosomes were used. Mapping was done by allowing up to two mismatches and up to 10 mapping locations with the bowtie options --best --strata -v 2 -m 10. Using the resulting sam file, Paralyzer analysis was performed with default settings (28). Metagene plots and density plots for individual genes were drawn using the HTseq package (52). mRNAseq data from S2-R+ cells (SRR070266) were obtained from the modENCODE website http://data.modencode.org, and mapped to the dm6 genome by STAR, and the RSEM normalized data were used. The T-to-C density values used for the scatter plots were calculated by dividing T-to-C conversion counts by the effective length of the transcript (kb). A pseudocount of 0.01 was added to all values before log transformation. To count T-to-C substitution events in exons, dm6.ensGene.gtf was downloaded from the UCSC Genome Browser website and used bedtools <https://bedtools.readthedocs.io> (53) to intersect exonic sequences with PARalyzer-identified clusters. To normalize the read counts for the transcript size, the effective sizes estimated by the RSEM package were used. To identify intronless genes, we chose genes whose last exons were annotated exon 1, and then mitochondrial genes were removed. For tRNA analysis, we found that CCA-tailed species were sometimes mapped to the genome sequence by allowing two mismatches. To avoid this, we first isolated reads containing CCA at their 3′ ends and mapped them to the last 30 nt of mature tRNA sequences followed by CCA, and these were used as “CCA-containing” reads. Unmapped reads were combined with the non-CCA reads and then used for genome mapping. Reads corresponding to tRNA genes were then selected and counted by bedtools.

## Data availability

The PAR-CLIP sequencing data are deposited in SRA under the BioProject ID PRJNA1092371.

## Supporting information

This article contains supporting information.

## Conflicts of interest

The authors declare that they have no conflcits of interest with the contents of this article.

## Supporting information

Supplementary Information Summary

Table S6

UCSC Tracks

## References

[1] Williams T., Ngo L.H., Wickramasinghe V.O. (2018). Nuclear export of RNA: different sizes, shapes and functions. Semin. Cell Dev. Biol..

[2] Köhler A., Hurt E. (2007). Exporting RNA from the nucleus to the cytoplasm. Nat. Rev. Mol. Cell Biol..

[3] Bohnsack K.E., Bohnsack M.T. (2019). Uncovering the assembly pathway of human ribosomes and its emerging links to disease. EMBO J..

[4] Wild T., Horvath P., Wyler E., Widmann B., Badertscher L., Zemp I. (2010). A protein inventory of human ribosome biogenesis reveals an essential function of exportin 5 in 60S subunit export. PLoS Biol..

[5] Takano A., Endo T., Yoshihisa T. (2005). tRNA actively shuttles between the nucleus and cytosol in yeast. Science.

[6] Arts G.-J. (1998). The role of exportin-t in selective nuclear export of mature tRNAs. EMBO J..

[7] Arts G.-J., Fornerod M., Mattaj L.W. (1998). Identification of a nuclear export receptor for tRNA. Curr. Biol..

[8] Kutay U., Lipowsky G., Izaurralde E., Bischoff F.R., Schwarzmaier P., Hartmann E. (1998). Identification of a tRNA-specific nuclear export receptor. Mol. Cell..

[9] LIPOWSKY G., BISCHOFF F.R., IZAURRALDE E., KUTAY U., SCHÄFER S., GROSS H.J. (1999). Coordination of tRNA nuclear export with processing of tRNA. RNA.

[10] Hellmuth K., Lau D.M., Bischoff F.R., Künzler M., Hurt E., Simos G. (1998). Yeast Los1p has properties of an exportin-like nucleocytoplasmic transport factor for tRNA. Mol. Cell. Biol..

[11] Lu S., Cullen B.R. (2004). Adenovirus VA1 noncoding RNA can inhibit small interfering RNA and MicroRNA biogenesis. J. Virol..

[12] Cook A.G., Fukuhara N., Jinek M., Conti E. (2009). Structures of the tRNA export factor in the nuclear and cytosolic states. Nature.

[13] Park M.Y., Wu G., Gonzalez-Sulser A., Vaucheret H., Poethig R.S. (2005). Nuclear processing and export of microRNAs in Arabidopsis. Proc. Natl. Acad. Sci. U. S. A..

[14] Shibata S., Sasaki M., Miki T., Shimamoto A., Furuichi Y., Katahira J. (2006). Exportin-5 orthologues are functionally divergent among species. Nucleic Acids Res..

[15] Okada C., Yamashita E., Lee S.J., Shibata S., Katahira J., Nakagawa A. (2009). A high-resolution structure of the pre-microRNA nuclear export machinery. Science.

[16] Wang J., Lee J.E., Riemondy K., Yu Y., Marquez S.M., Lai E.C. (2020). XPO5 promotes primary miRNA processing independently of RanGTP. Nat. Commun..

[17] Kim Y.K., Kim B., Kim V.N. (2016). Re-evaluation of the roles of DROSHA, Exportin 5, and DICER in microRNA biogenesis. Proc. Natl. Acad. Sci. U. S. A..

[18] Gwizdek C., Ossareh-Nazari B., Brownawell A.M., Doglio A., Bertrand E., Macara I.G. (2003). Exportin-5 mediates nuclear export of minihelix-containing RNAs. J. Biol. Chem..

[19] Gwizdek C., Bertrand E., Dargemont C., Lefebvre J.-C., Blanchard J.-M., Singer R.H. (2001). Terminal minihelix, a novel RNA motif that directs polymerase III transcripts to the cell cytoplasm. J. Biol. Chem..

[20] Bohnsack M.T., Czaplinski K., Gorlich D. (2004). Exportin 5 is a RanGTP-dependent dsRNA-binding protein that mediates nuclear export of pre-miRNAs. Rna..

[21] Lund E., Guttinger S., Calado A., Dahlberg J.E., Kutay U. (2004). Nuclear export of microRNA precursors. Science.

[22] Yi R., Qin Y., Macara I.G., Cullen B.R. (2003). Exportin-5 mediates the nuclear export of pre-microRNAs and short hairpin RNAs. Genes Dev..

[23] Takeiwa T., Taniguchi I., Ohno M. (2015). Exportin-5 mediates nuclear export of SRP RNA in vertebrates. Genes Cells.

[24] Calado A., Treichel N., Müller E.-C., Otto A., Kutay U. (2002). Exportin-5-mediated nuclear export of eukaryotic elongation factor 1A and tRNA. EMBO J..

[25] Bohnsack M.T., Regener K., Schwappach B., Saffrich R., Paraskeva E., Hartmann E. (2002). Exp5 exports eEF1A via tRNA from nuclei and synergizes with other transport pathways to confine translation to the cytoplasm. EMBO J..

[26] Hafner M., Landthaler M., Burger L., Khorshid M., Hausser J., Berninger P. (2010). Transcriptome-wide identification of RNA-binding protein and microRNA target sites by PAR-CLIP. Cell.

[27] Bunch T.A., Grinblat Y., Goldstein L.S.B. (1988). Characterization and use of the Drosophila metallothionein promoter in cultured Drosophila melanogaster cells. Nucleic Acids Res..

[28] Corcoran D.L., Georgiev S., Mukherjee N., Gottwein E., Skalsky R.L., Keene J.D. (2011). PARalyzer: definition of RNA binding sites from PAR-CLIP short-read sequence data. Genome Biol..

[29] Darnell R.B. (2010). HITS-CLIP: panoramic views of protein–RNA regulation in living cells. Wiley Interdiscip. Rev. RNA.

[30] Oubridge C., Kuglstatter A., Jovine L., Nagai K. (2002). Crystal structure of SRP19 in complex with the S domain of SRP RNA and its implication for the assembly of the signal recognition particle. Mol. Cell..

[31] Sakamoto T., Morita S., Tabata K., Nakamura K., Kawai G. (2002). Solution structure of a SRP19 binding domain in human SRP RNA. J. Biochem..

[32] Bennasser Y., Chable-Bessia C., Triboulet R., Gibbings D., Gwizdek C., Dargemont C. (2011). Competition for XPO5 binding between Dicer mRNA, pre-miRNA and viral RNA regulates human Dicer levels. Nat. Struct. Mol. Biol..

[33] Cherbas L., Willingham A., Zhang D., Yang L., Zou Y., Eads B.D. (2011). The transcriptional diversity of 25 Drosophila cell lines. Genome Res..

[34] Wilk R., Hu J., Blotsky D., Krause H.M. (2016). Diverse and pervasive subcellular distributions for both coding and long noncoding RNAs. Genes Dev..

[35] Blanchette M., Labourier E., Green R.E., Brenner S.E., Rio D.C. (2004). Genome-wide analysis reveals an unexpected function for the Drosophila splicing factor U2AF50 in the nuclear export of intronless mRNAs. Mol. Cell..

[36] Chatterjee K., Nostramo R.T., Wan Y., Hopper A.K. (2018). tRNA dynamics between the nucleus, cytoplasm and mitochondrial surface: location, location, location. Biochim. Biophys. Acta.

[37] Suzuki T. (2021). The expanding world of tRNA modifications and their disease relevance. Nat. Rev. Mol. Cell Biol..

[38] Phizicky E.M., Hopper A.K. (2010). tRNA biology charges to the front. Genes Dev..

[39] Kessler A.C., Silveira d’Almeida G., Alfonzo J.D. (2018). The role of intracellular compartmentalization on tRNA processing and modification. RNA Biol..

[40] De Robertis E.M., Black P., Nishikura K. (1981). Intranuclear location of the tRNA splicing enzymes. Cell.

[41] Akiyama Y., Lyons S.M., Takaaki A., Paul J., Anderson P.I. (2022). Cytoplasmic processing of human transfer RNAs. bioRxiv.

[42] Khan M., Hou S., Chen M., Lei H. (2023). Mechanisms of RNA export and nuclear retention. Wiley Interdiscip. Rev. RNA.

[43] Wang K., Wang L., Wang J., Chen S., Shi M., Cheng H. (2018). Intronless mRNAs transit through nuclear speckles to gain export competence. J. Cell Biol..

[44] Lei H., Dias A.P., Reed R. (2011). Export and stability of naturally intronless mRNAs require specific coding region sequences and the TREX mRNA export complex. Proc. Natl. Acad. Sci. U. S. A..

[45] Hopper A.K., Phizicky E.M. (2003). tRNA transfers to the limelight. Genes Dev..

[46] Kadaba S., Krueger A., Trice T., Krecic A.M., Hinnebusch A.G., Anderson J. (2004). Nuclear surveillance and degradation of hypomodified initiator tRNA Met in S. cerevisiae. Genes Dev..

[47] Wang X., Jia H., Jankowsky E., Anderson J.T. (2008). Degradation of hypomodified tRNA i Met in vivo involves RNA-dependent ATPase activity of the DExH helicase Mtr4p. RNA.

[48] Herold A. (2003). Genome-wide analysis of nuclear mRNA export pathways in Drosophila. EMBO J..

[49] Berezikov E., Robine N., Samsonova A., Westholm J.O., Naqvi A., Hung J.H. (2011). Deep annotation of Drosophila melanogaster microRNAs yields insights into their processing, modification, and emergence. Genome Res..

[50] Langmead B., Trapnell C., Pop M., Salzberg S.L. (2009). Ultrafast and memory-efficient alignment of short DNA sequences to the human genome. Genome Biol..

[51] Hartig J.V., Esslinger S., Bottcher R., Saito K., Forstemann K. (2009). Endo-siRNAs depend on a new isoform of loquacious and target artificially introduced, high-copy sequences. EMBO J..

[52] Putri G.H., Anders S., Pyl P.T., Pimanda J.E., Zanini F. (2022). Analysing high-throughput sequencing data in Python with HTSeq 2.0. Bioinformatics.

[53] Quinlan A.R., Hall I.M. (2010). BEDTools: a flexible suite of utilities for comparing genomic features. Bioinformatics.

