# Supplementary material for: Exportin-5 binding precedes 5′- and 3′-end processing of tRNA precursors in Drosophila
Source: J Biol Chem. 2024 Aug 2;300(9):107632. doi: 10.1016/j.jbc.2024.107632 (PMC11402290; doi:10.1016/j.jbc.2024.107632)
Supplement: Supplementary Information Summary [file mmc1.docx]

**Supplementary information for**

**Li et al. “Exportin-5 binding precedes 5’- and 3’-end processing of tRNA precursors in *Drosophila*.”**

**Supplementary figure**

Figure S1 Pre- and mature tRNA detection in FLAG-Exp5 immunoprecipitations.

**Supplementary tables**

Table S1. Statistics of the PAR-CLIP library

Table S2. Read mapping information for each gene category

Table S3. List of materials and bioinformatics resources

Table S4. Statistics of PAR-CLIP signals from annotated mRNAs

Table S5. List of intronless protein coding genes

Table S6. Counts of tRNA-mapping reads (Sheet 1: PAR-CLIP library, Sheet2: sRNA library)

**Supplementary Data**

Supplementary PDF1. Read density maps of individual miRNA genes

Each page contains read density maps of PAR-CLIP reads (all), PAR-CLIP reads (T-to-C), SRR013547 (sRNA library; all reads), SRR013548 (sRNA library; all reads) for a miRNA gene.

Supplementary PDF2. Read density maps of individual tRNA genes

Each page contains read density maps of sRNA library (SRR013547 and SRR013548 combined) in the upper row, and PAR-CLIP library in the lower row for a tRNA gene. The left panels show genome mapping reads and the right panels show reads mapping to the CCA-modified tRNA 3’ end. Blue and yellow lines show read densities of all reads and T-to-C reads, respectively.

UCSC tracks: dm6 tracks used in this manuscript.
