# Supplementary material for: Exportin-5 binding precedes 5′- and 3′-end processing of tRNA precursors in Drosophila
Source: J Biol Chem. 2024 Aug 2;300(9):107632. doi: 10.1016/j.jbc.2024.107632 (PMC11402290; doi:10.1016/j.jbc.2024.107632)
Supplement: Supplementary Material 1 [file mmc2.pdf]

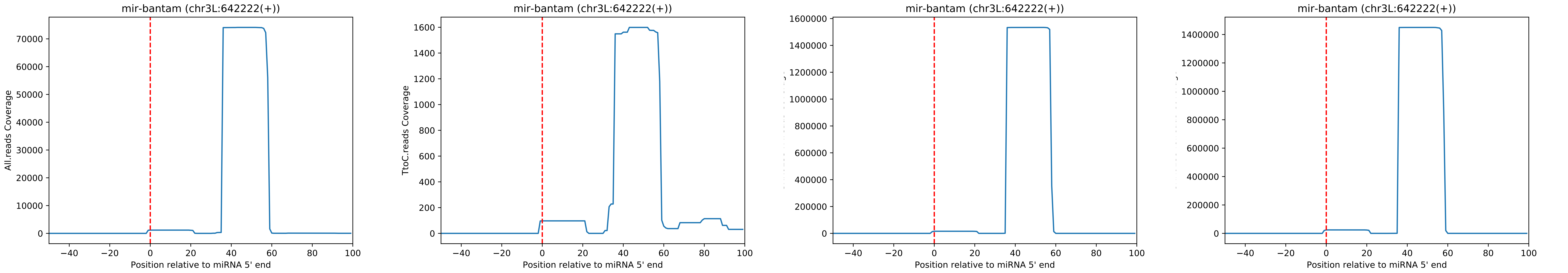
mir-bantam (chr3L:642222(+))
All.reads Coverage
0
10000
20000
30000
40000
50000
60000
70000
−40
−20
0
20
40
60
80
100
Position relative to miRNA 5' end
mir-bantam (chr3L:642222(+))
TtoC.reads Coverage
0
200
400
600
800
1000
1200
1400
1600
−40
−20
0
20
40
60
80
100
Position relative to miRNA 5' end
mir-bantam (chr3L:642222(+))
0
200000
400000
600000
800000
1000000
1200000
1400000
1600000
−40
−20
0
20
40
60
80
100
Position relative to miRNA 5' end
mir-bantam (chr3L:642222(+))
0
200000
400000
600000
800000
1000000
1200000
1400000
−40
−20
0
20
40
60
80
100
Position relative to miRNA 5' end

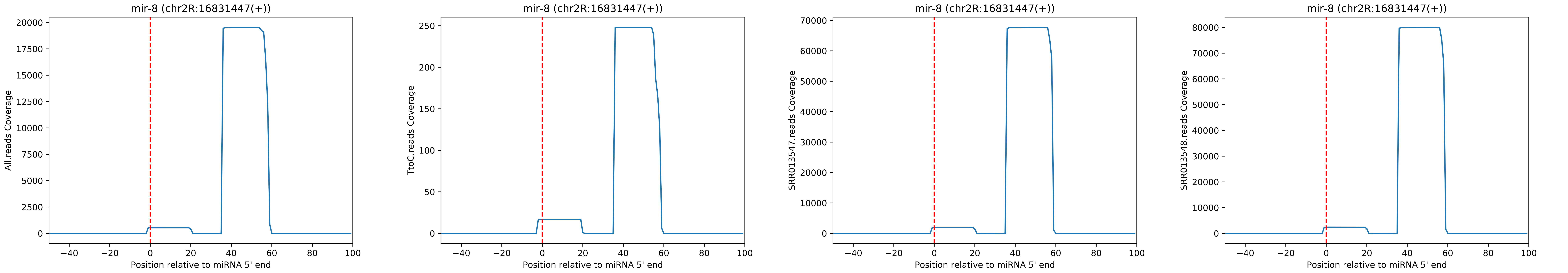
mir-8 (chr2R:16831447(+))
All.reads Coverage
Position relative to miRNA 5' end
mir-8 (chr2R:16831447(+))
TtoC.reads Coverage
Position relative to miRNA 5' end
mir-8 (chr2R:16831447(+))
SRR013547.reads Coverage
Position relative to miRNA 5' end
mir-8 (chr2R:16831447(+))
SRR013548.reads Coverage
Position relative to miRNA 5' end

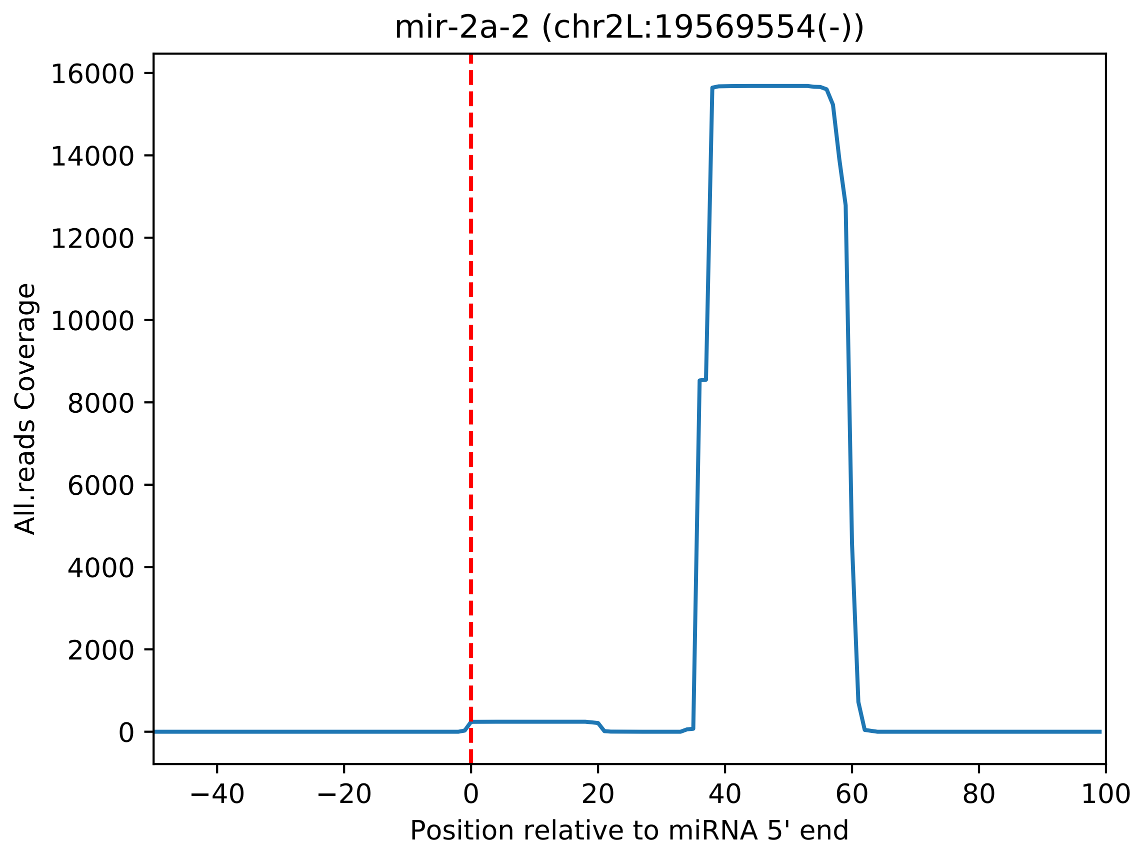
mir-2a-2 (chr2L:19569554(-))
All.reads Coverage
Position relative to miRNA 5' end

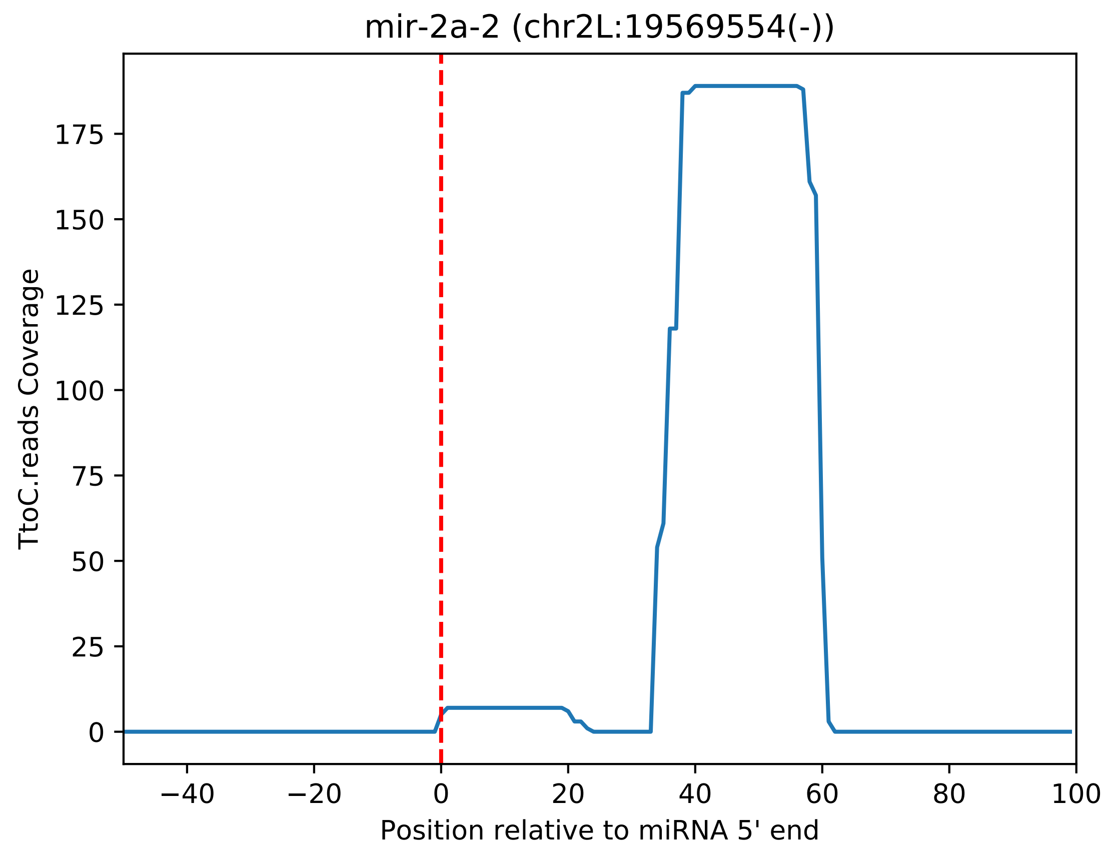
mir-2a-2 (chr2L:19569554(-))
TtoC.reads Coverage
Position relative to miRNA 5' end

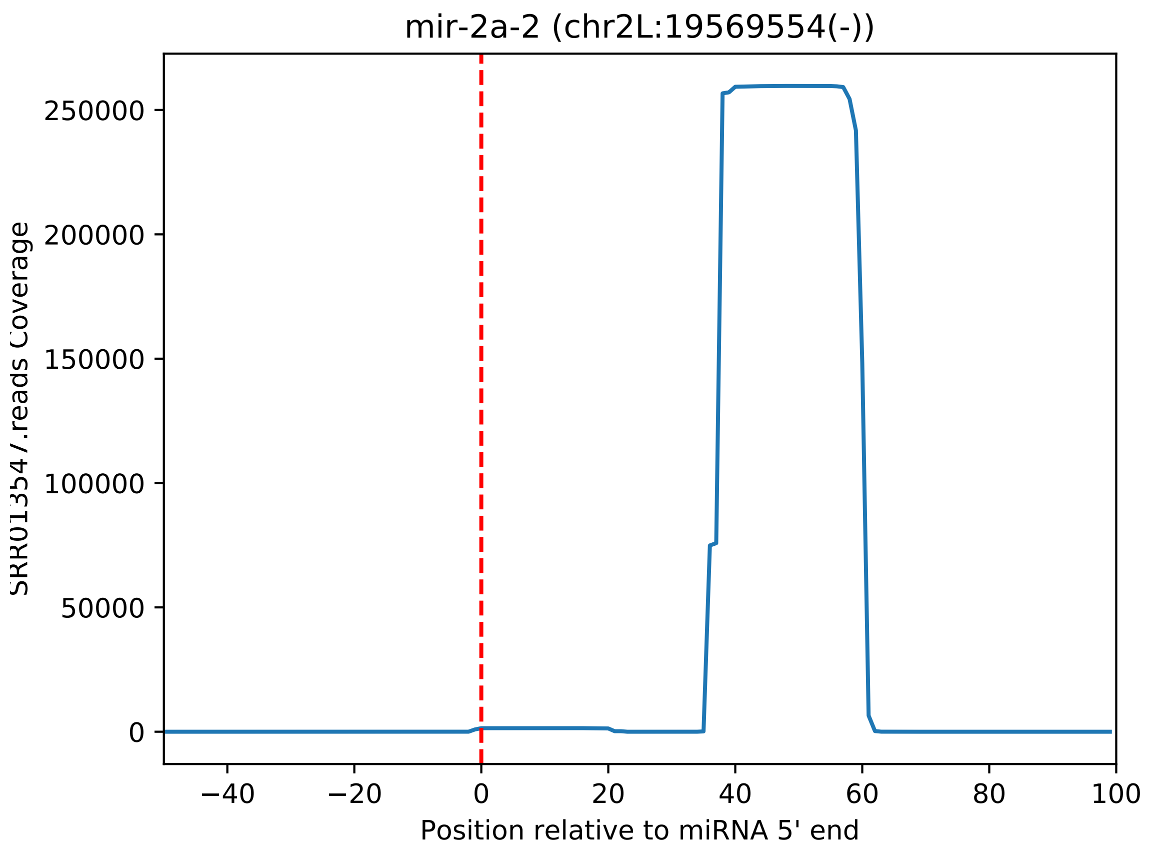
mir-2a-2 (chr2L:19569554(-))
SRR013547.reads Coverage
Position relative to miRNA 5' end

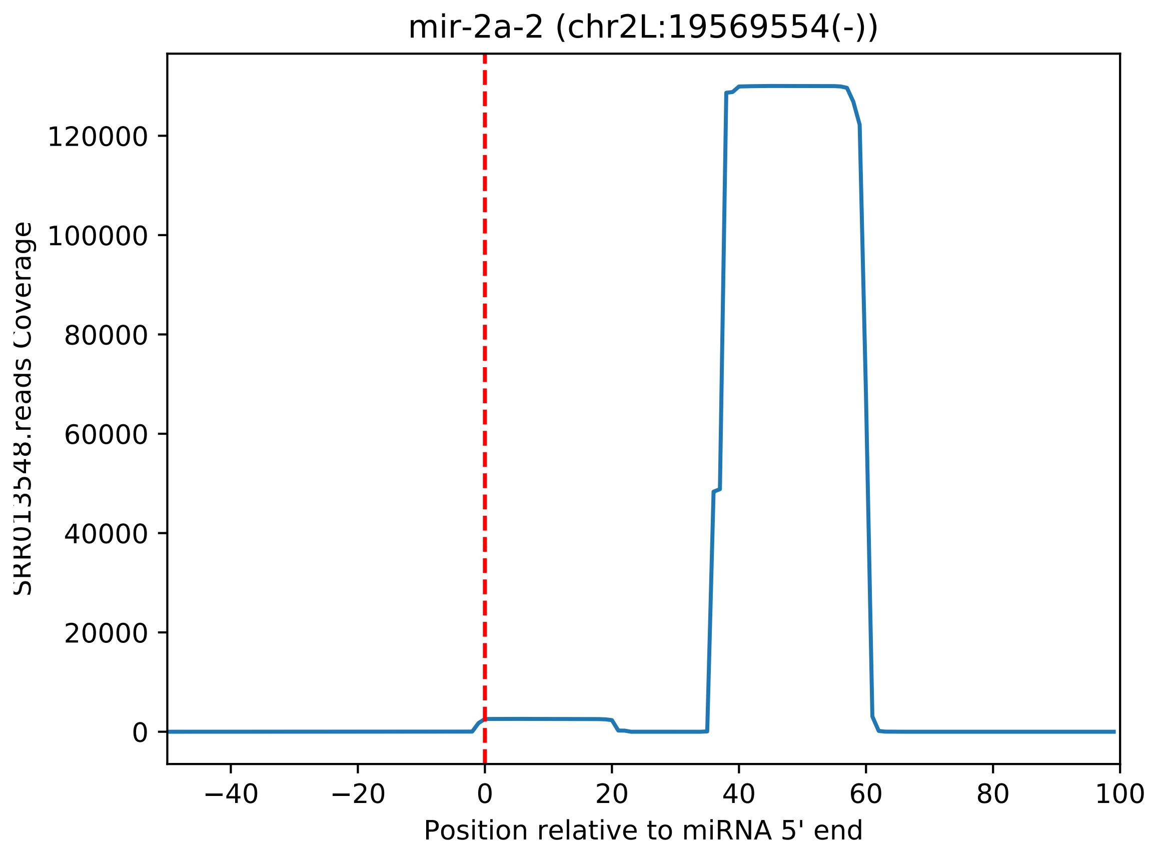
mir-2a-2 (chr2L:19569554(-))
SRR013548.reads Coverage
Position relative to miRNA 5' end

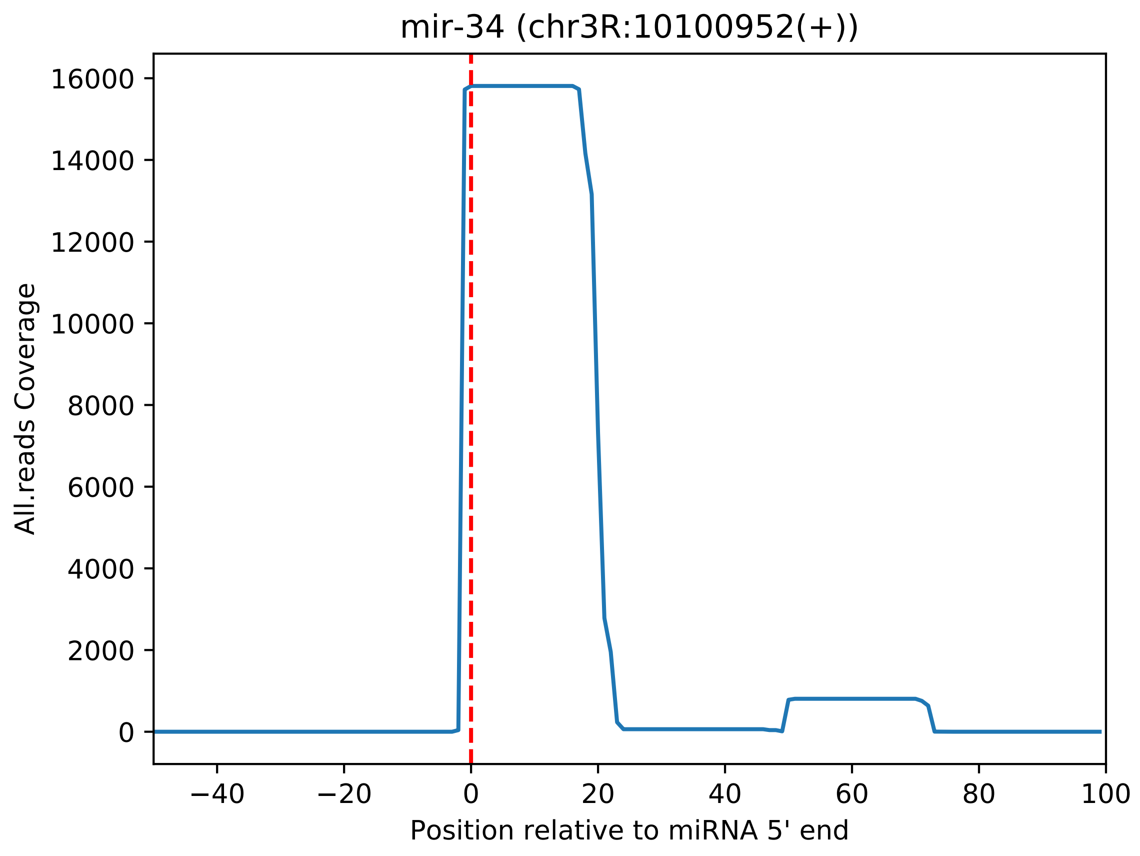
mir-34 (chr3R:10100952(+))
All.reads Coverage
Position relative to miRNA 5' end
0
2000
4000
6000
8000
10000
12000
14000
16000
−40
−20
0
20
40
60
80
100

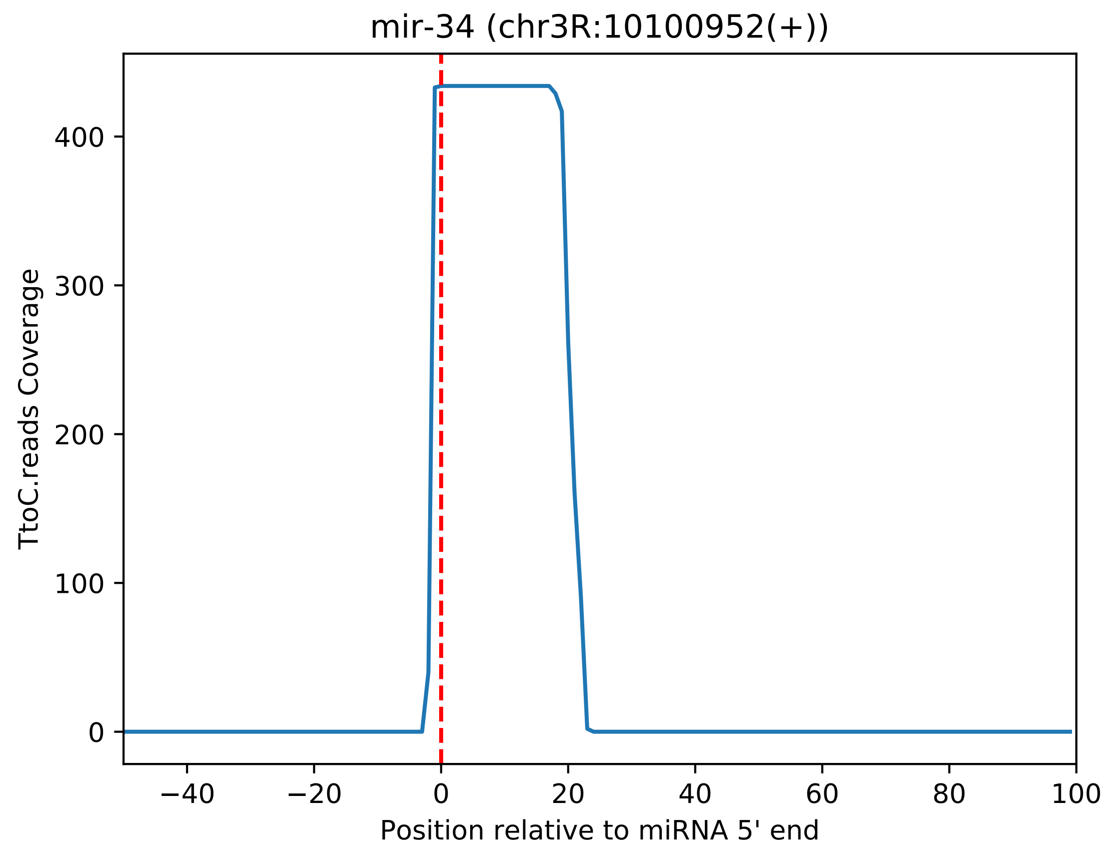
mir-34 (chr3R:10100952(+))
TtoC.reads Coverage
Position relative to miRNA 5' end
0
100
200
300
400
−40
−20
0
20
40
60
80
100

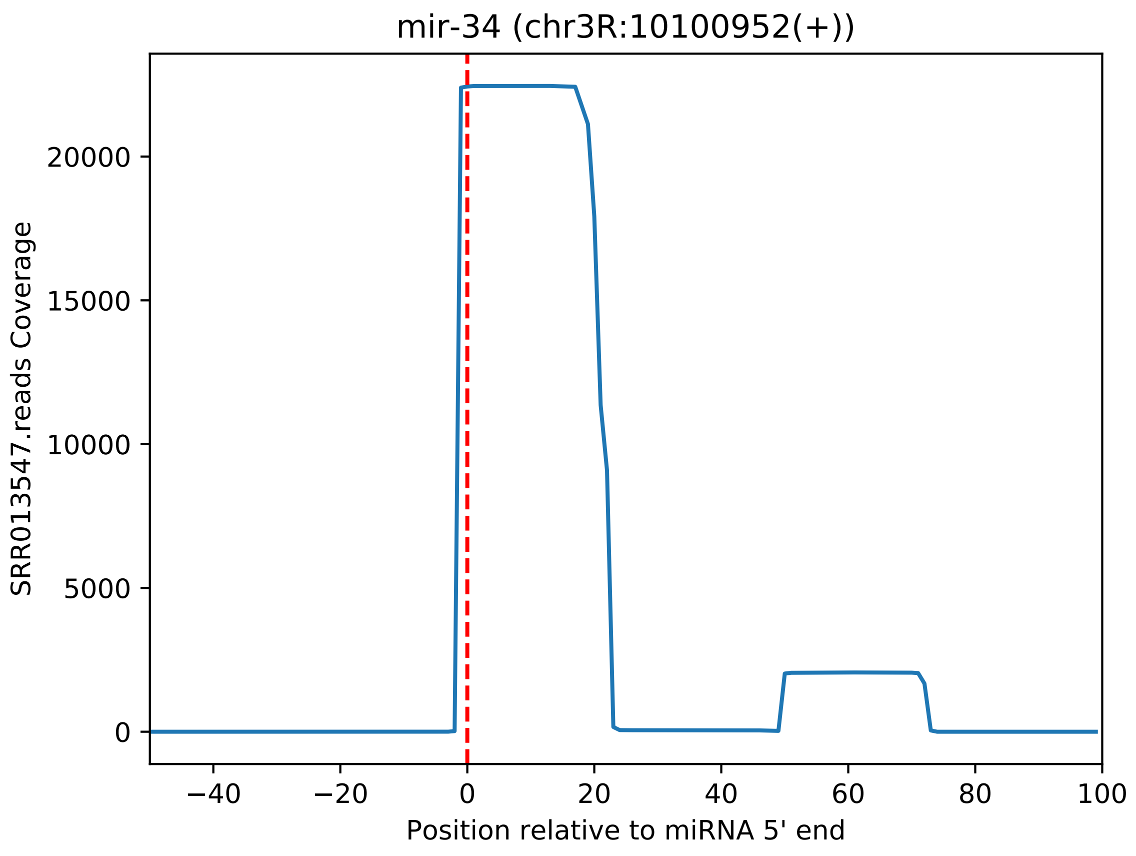
mir-34 (chr3R:10100952(+))
SRR013547.reads Coverage
Position relative to miRNA 5' end
0
5000
10000
15000
20000
−40
−20
0
20
40
60
80
100

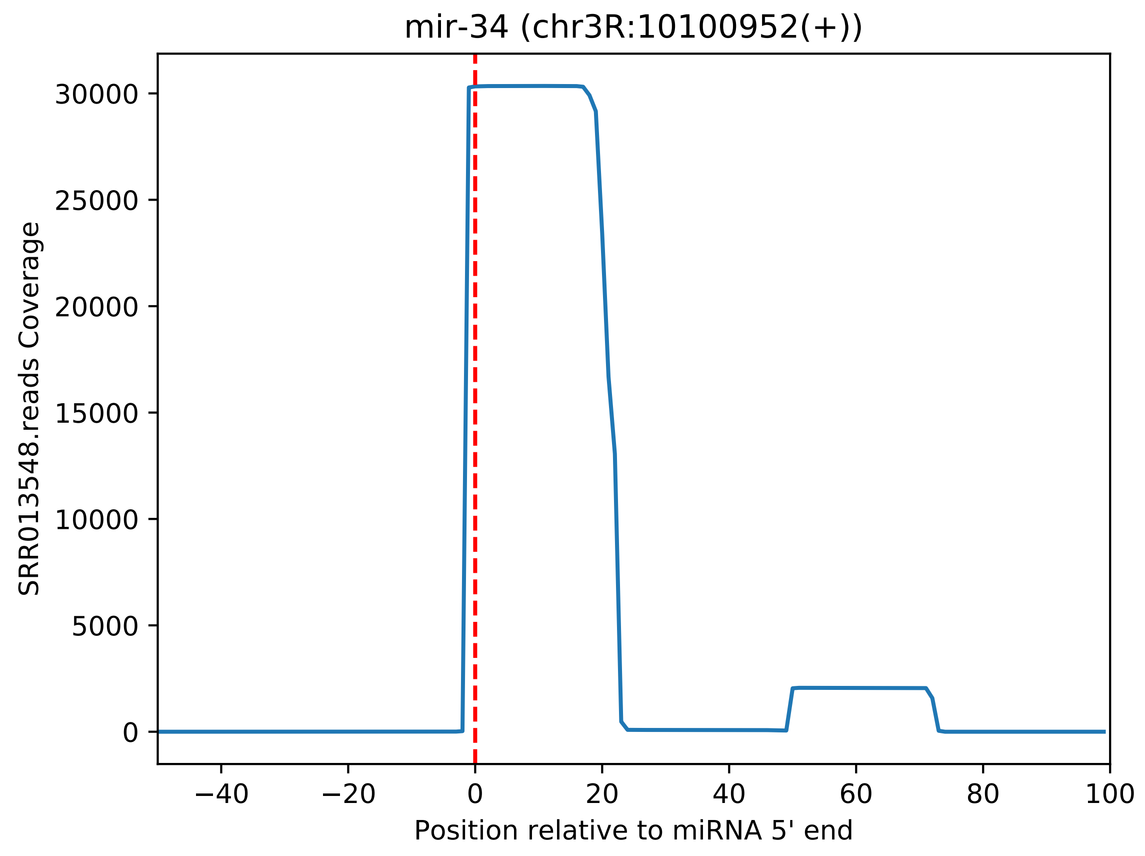
mir-34 (chr3R:10100952(+))
SRR013548.reads Coverage
Position relative to miRNA 5' end
0
5000
10000
15000
20000
25000
30000
−40
−20
0
20
40
60
80
100

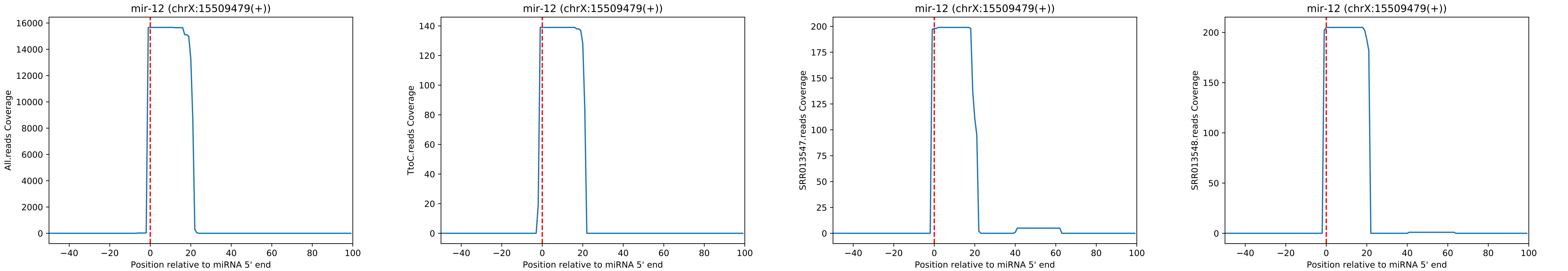
mir-12 (chrX:15509479(+))
All.reads Coverage
Position relative to miRNA 5' end
mir-12 (chrX:15509479(+))
TtoC.reads Coverage
Position relative to miRNA 5' end
mir-12 (chrX:15509479(+))
SRR013547.reads Coverage
Position relative to miRNA 5' end
mir-12 (chrX:15509479(+))
SRR013548.reads Coverage
Position relative to miRNA 5' end

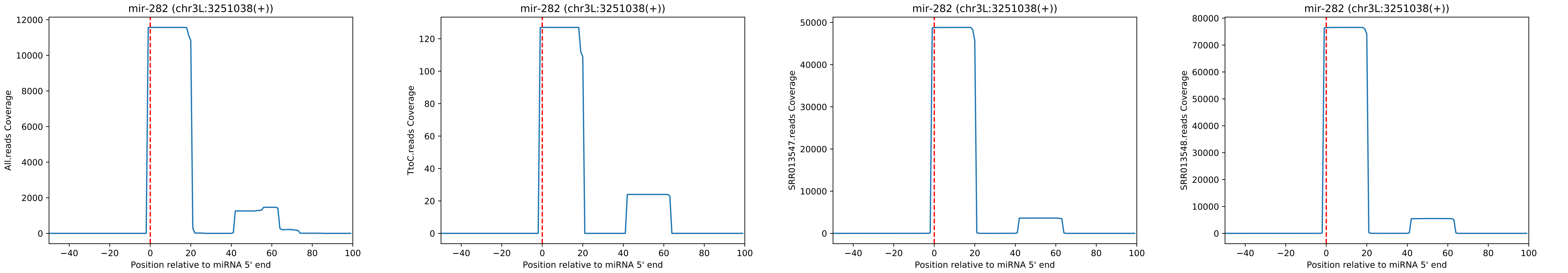
mir-282 (chr3L:3251038(+))
All.reads Coverage
Position relative to miRNA 5' end
mir-282 (chr3L:3251038(+))
TtoC.reads Coverage
Position relative to miRNA 5' end
mir-282 (chr3L:3251038(+))
SRR013547.reads Coverage
Position relative to miRNA 5' end
mir-282 (chr3L:3251038(+))
SRR013548.reads Coverage
Position relative to miRNA 5' end

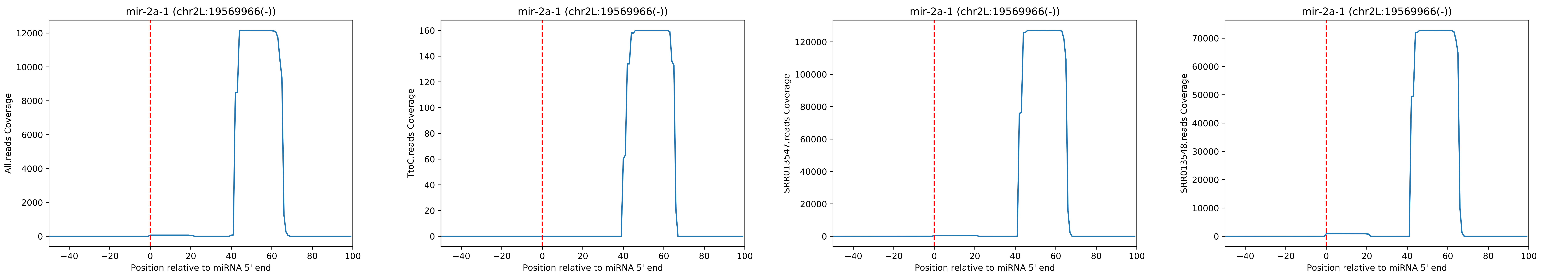
mir-2a-1 (chr2L:19569966(-))
All.reads Coverage
Position relative to miRNA 5' end
mir-2a-1 (chr2L:19569966(-))
TtoC.reads Coverage
Position relative to miRNA 5' end
mir-2a-1 (chr2L:19569966(-))
Position relative to miRNA 5' end
mir-2a-1 (chr2L:19569966(-))
SRR013548.reads Coverage
Position relative to miRNA 5' end

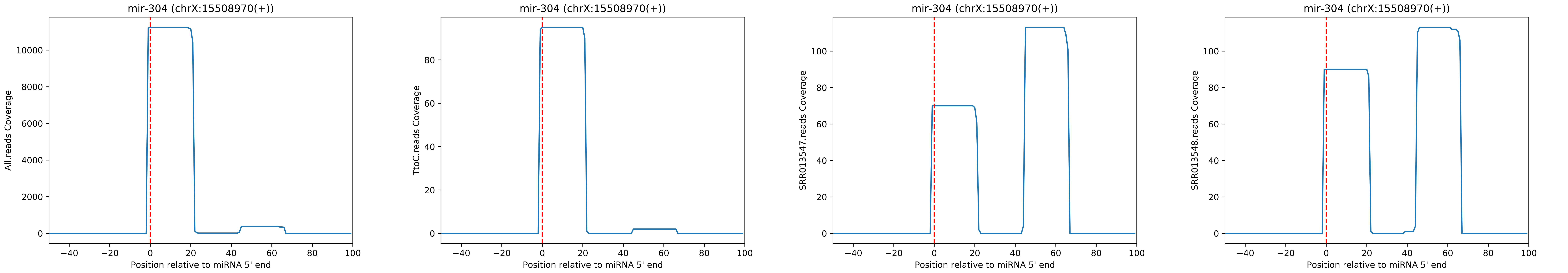
mir-304 (chrX:15508970(+))
All.reads Coverage
Position relative to miRNA 5' end
mir-304 (chrX:15508970(+))
TtoC.reads Coverage
Position relative to miRNA 5' end
mir-304 (chrX:15508970(+))
SRR013547.reads Coverage
Position relative to miRNA 5' end
mir-304 (chrX:15508970(+))
SRR013548.reads Coverage
Position relative to miRNA 5' end

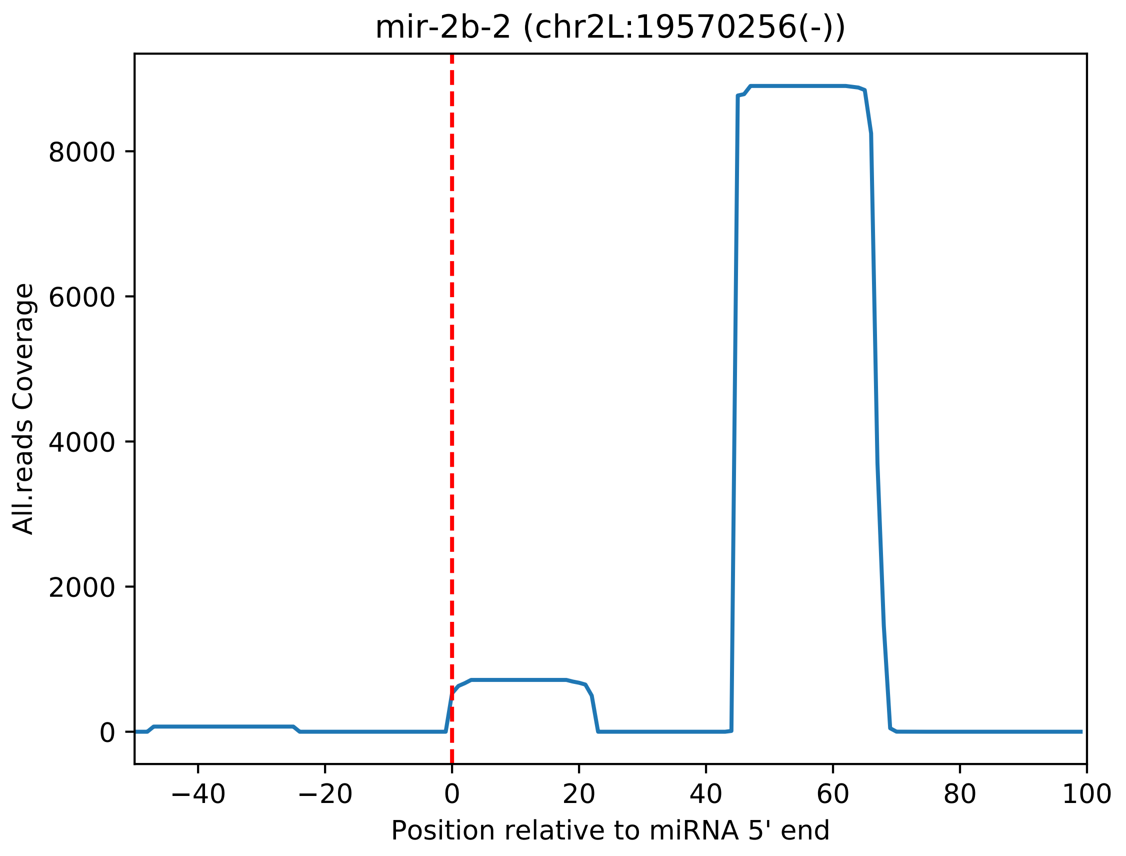
mir-2b-2 (chr2L:19570256(-))
All.reads Coverage
Position relative to miRNA 5' end
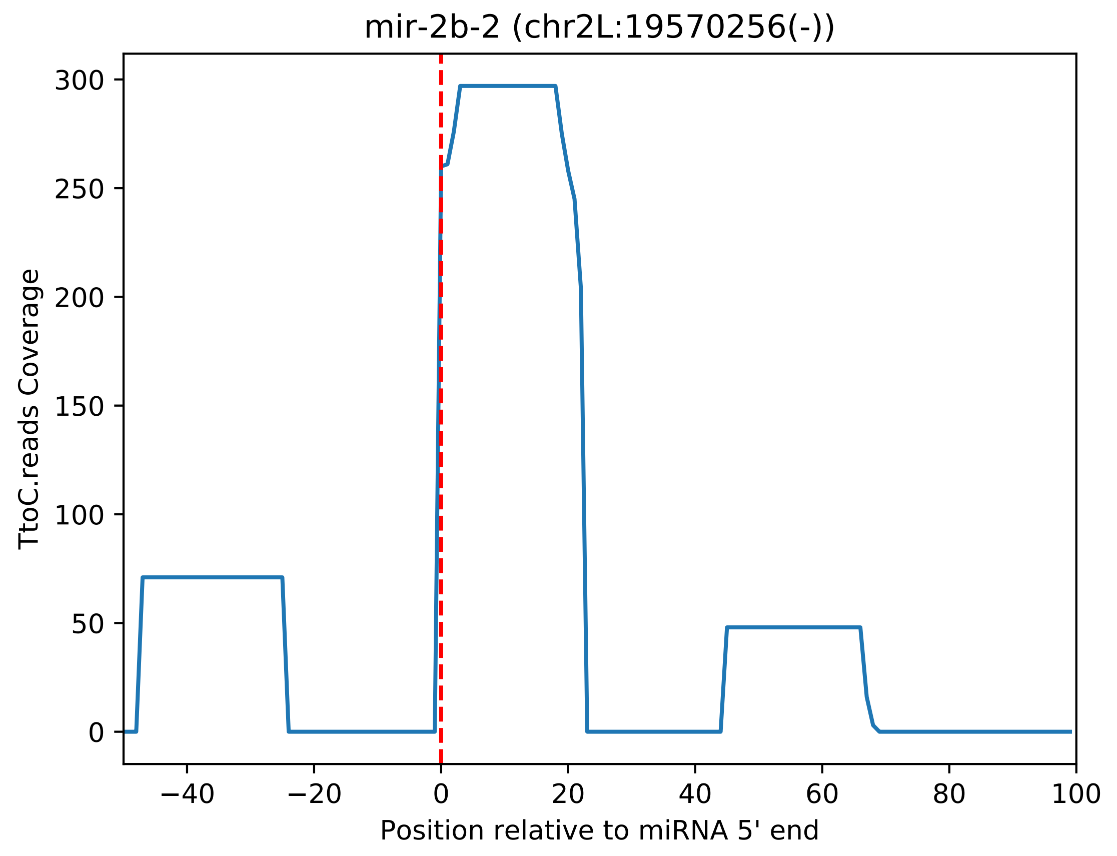
mir-2b-2 (chr2L:19570256(-))
TtoC.reads Coverage
Position relative to miRNA 5' end
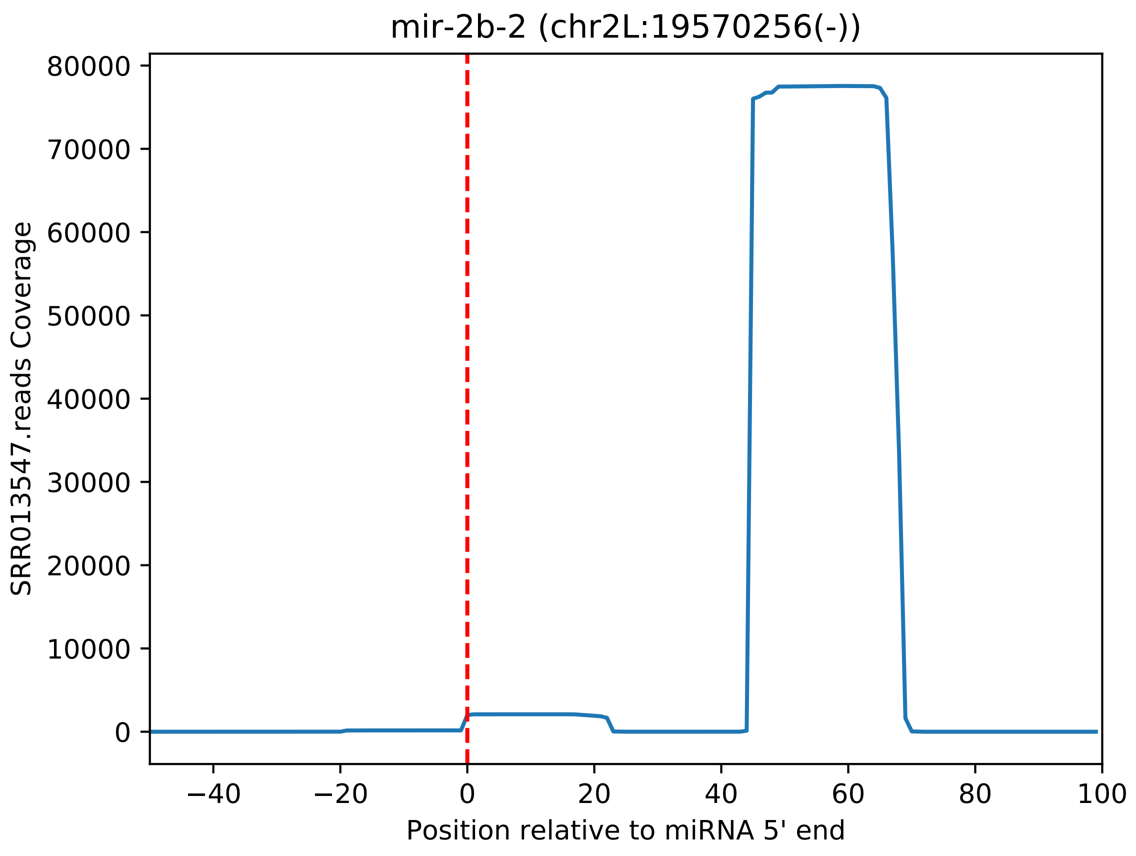
mir-2b-2 (chr2L:19570256(-))
SRR013547.reads Coverage
Position relative to miRNA 5' end
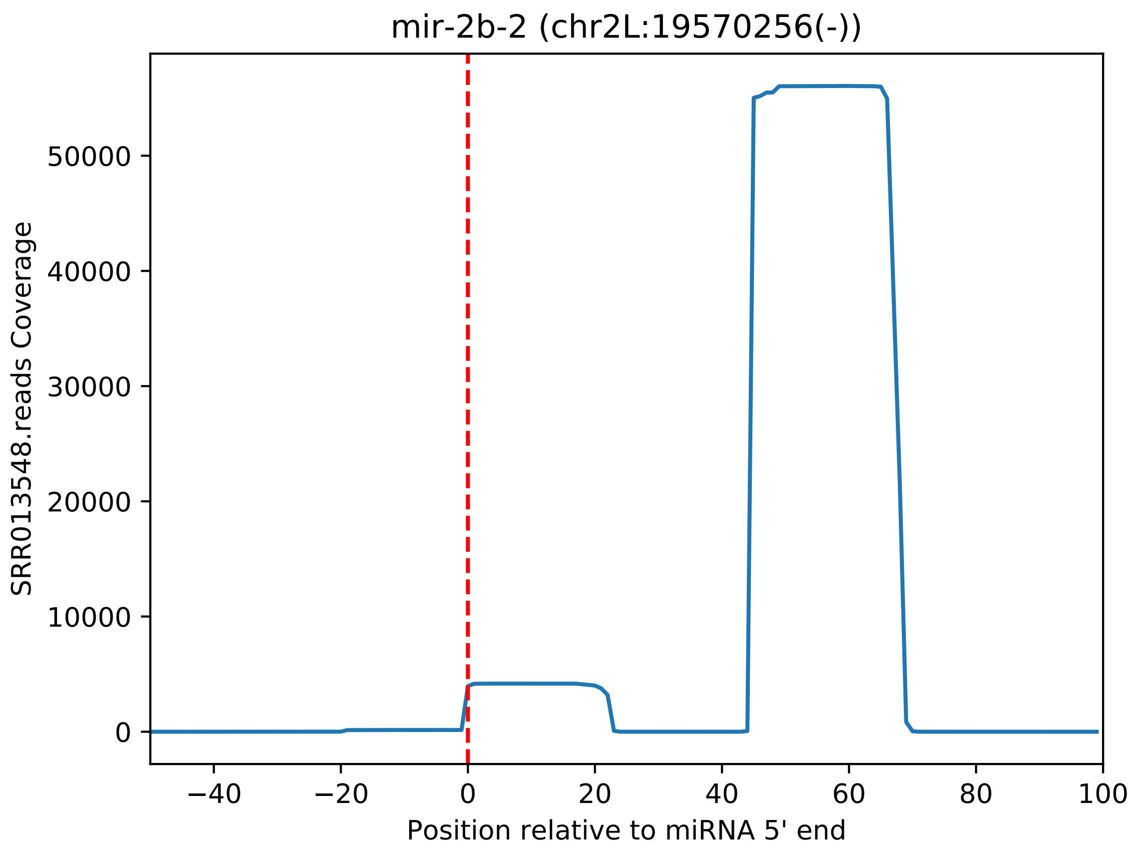
mir-2b-2 (chr2L:19570256(-))
SRR013548.reads Coverage
Position relative to miRNA 5' end

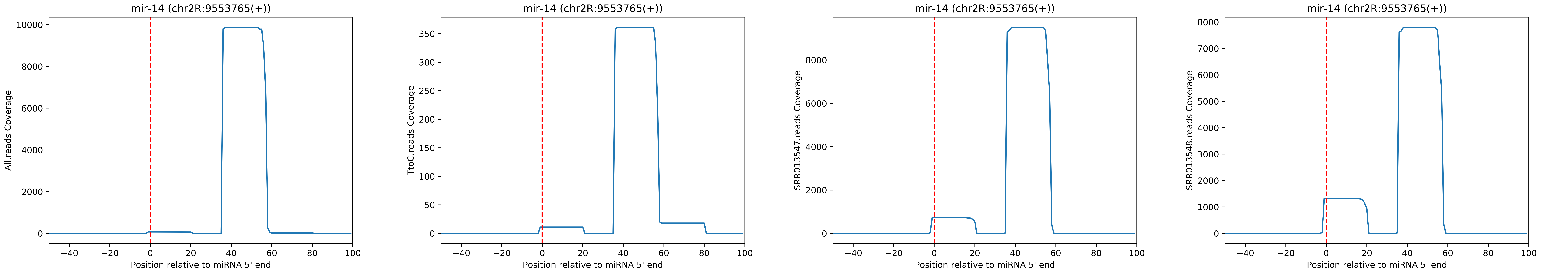

mir-14 (chr2R:9553765(+))
All.reads Coverage
Position relative to miRNA 5' end
mir-14 (chr2R:9553765(+))
TtoC.reads Coverage
Position relative to miRNA 5' end
mir-14 (chr2R:9553765(+))
SRR013547.reads Coverage
Position relative to miRNA 5' end
mir-14 (chr2R:9553765(+))
SRR013548.reads Coverage
Position relative to miRNA 5' end

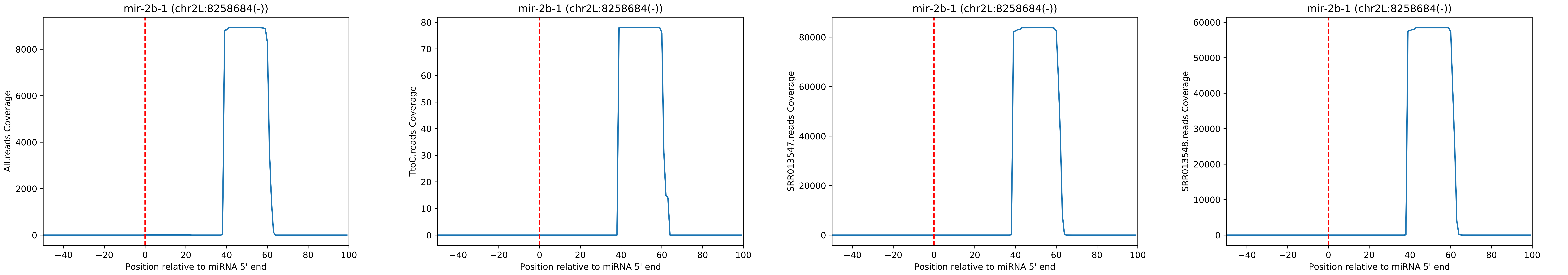
mir-2b-1 (chr2L:8258684(-))
All.reads Coverage
0
2000
4000
6000
8000
−40
−20
0
20
40
60
80
100
Position relative to miRNA 5' end
mir-2b-1 (chr2L:8258684(-))
TtoC.reads Coverage
0
10
20
30
40
50
60
70
80
−40
−20
0
20
40
60
80
100
Position relative to miRNA 5' end
mir-2b-1 (chr2L:8258684(-))
SRR013547.reads Coverage
0
20000
40000
60000
80000
−40
−20
0
20
40
60
80
100
Position relative to miRNA 5' end
mir-2b-1 (chr2L:8258684(-))
SRR013548.reads Coverage
0
10000
20000
30000
40000
50000
60000
−40
−20
0
20
40
60
80
100
Position relative to miRNA 5' end

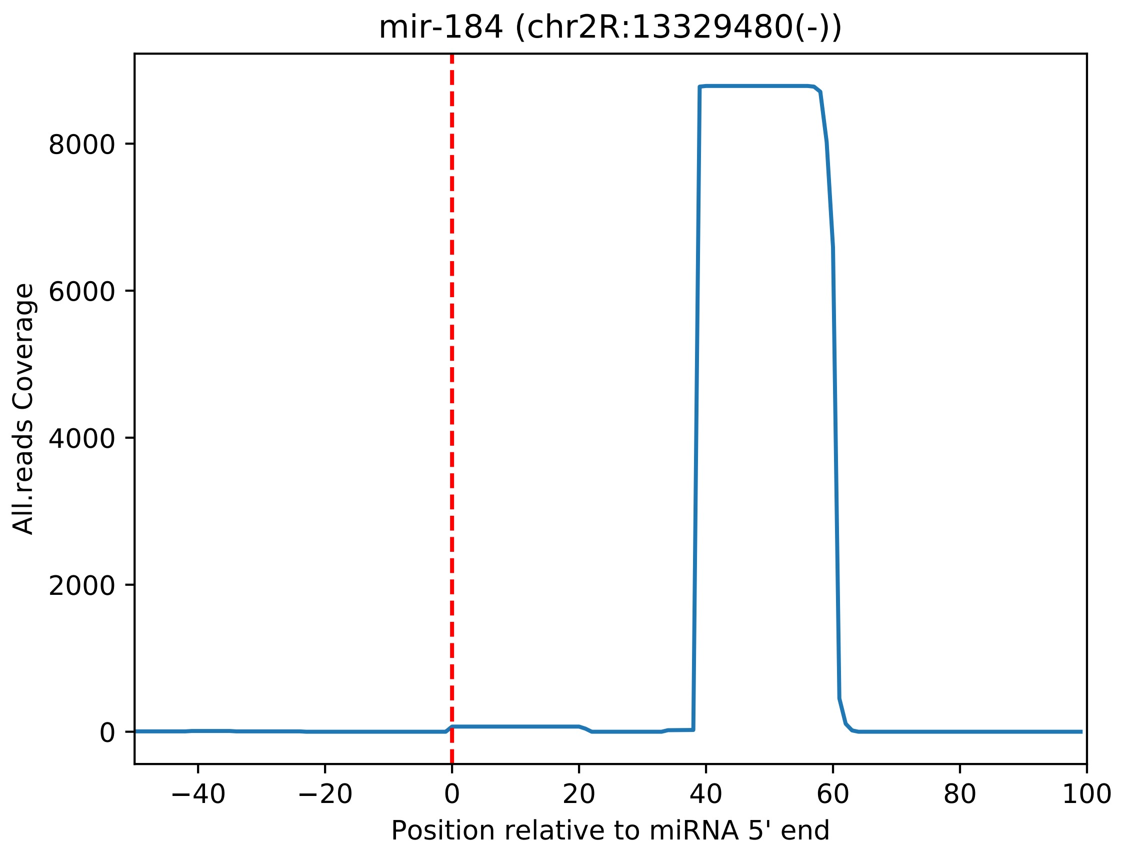
mir-184 (chr2R:13329480(-))
All.reads Coverage
Position relative to miRNA 5' end
0
2000
4000
6000
8000
−40
−20
0
20
40
60
80
100

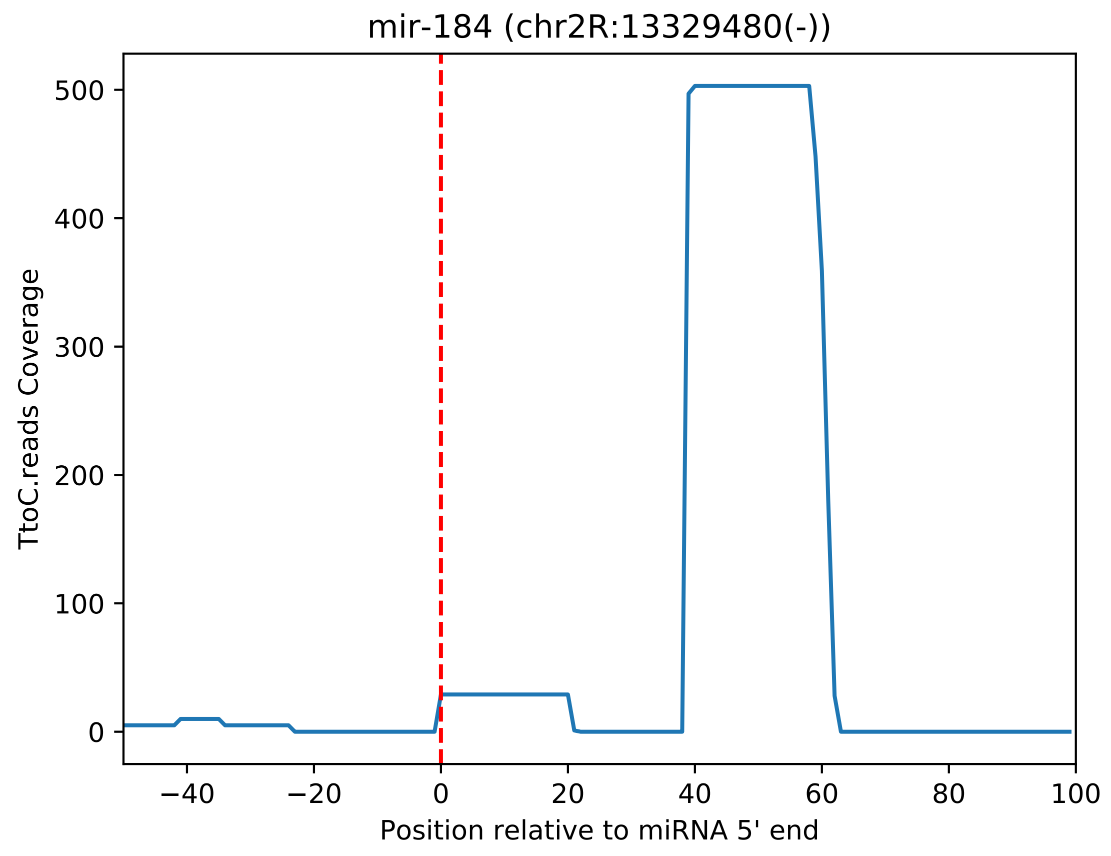
mir-184 (chr2R:13329480(-))
TtoC.reads Coverage
Position relative to miRNA 5' end
0
100
200
300
400
500
−40
−20
0
20
40
60
80
100

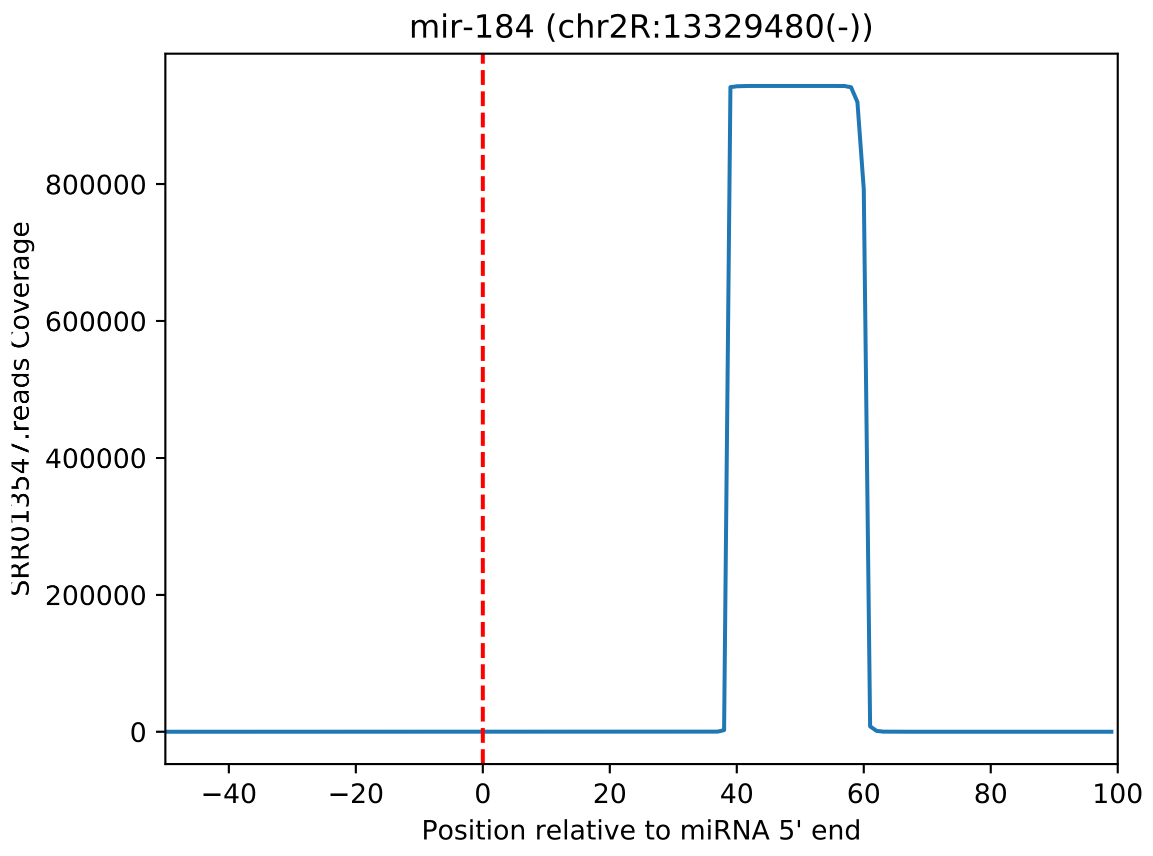
mir-184 (chr2R:13329480(-))
.reads Coverage
Position relative to miRNA 5' end
0
200000
400000
600000
800000
−40
−20
0
20
40
60
80
100

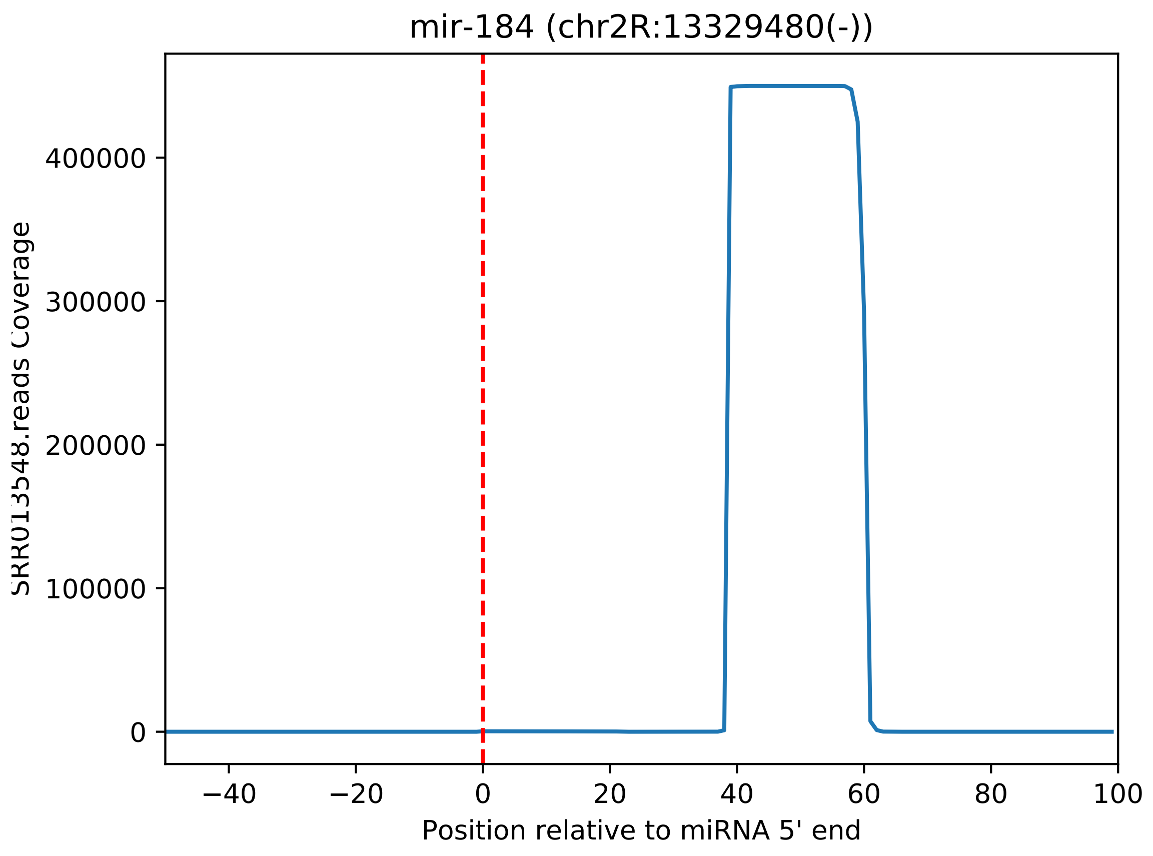
mir-184 (chr2R:13329480(-))
.reads Coverage
Position relative to miRNA 5' end
0
100000
200000
300000
400000
−40
−20
0
20
40
60
80
100

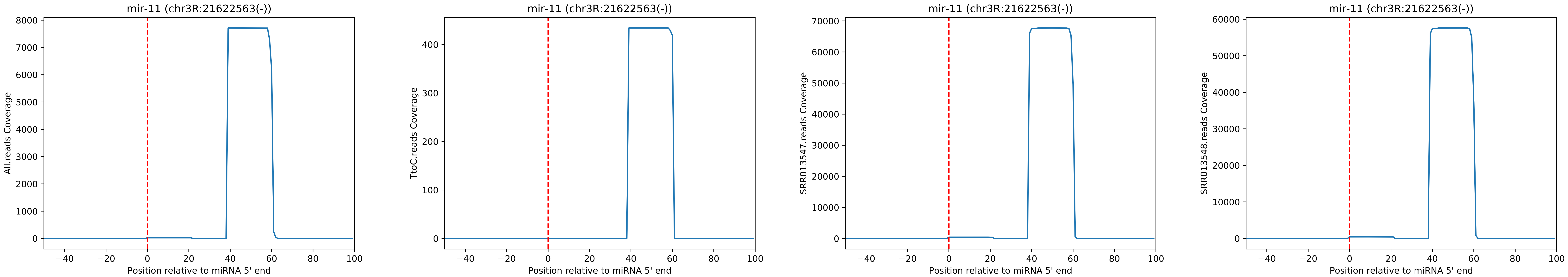

mir-11 (chr3R:21622563(-))
All.reads Coverage
Position relative to miRNA 5' end
mir-11 (chr3R:21622563(-))
TtoC.reads Coverage
Position relative to miRNA 5' end
mir-11 (chr3R:21622563(-))
SRR013547.reads Coverage
Position relative to miRNA 5' end
mir-11 (chr3R:21622563(-))
SRR013548.reads Coverage
Position relative to miRNA 5' end

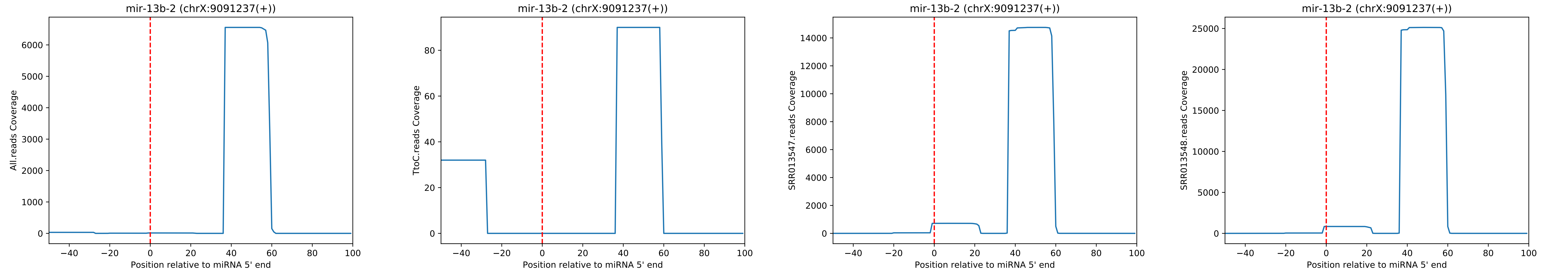
mir-13b-2 (chrX:9091237(+))
All.reads Coverage
0
1000
2000
3000
4000
5000
6000
Position relative to miRNA 5' end
−40
−20
0
20
40
60
80
100
mir-13b-2 (chrX:9091237(+))
TtoC.reads Coverage
0
20
40
60
80
Position relative to miRNA 5' end
mir-13b-2 (chrX:9091237(+))
SRR013547.reads Coverage
0
2000
4000
6000
8000
10000
12000
14000
Position relative to miRNA 5' end
mir-13b-2 (chrX:9091237(+))
SRR013548.reads Coverage
0
5000
10000
15000
20000
25000
Position relative to miRNA 5' end

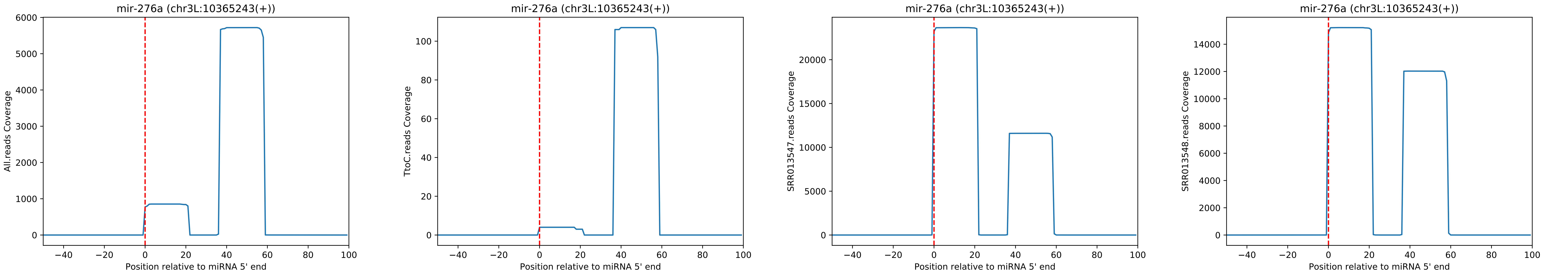

mir-276a (chr3L:10365243(+))
All.reads Coverage
TtoC.reads Coverage
SRR013547.reads Coverage
SRR013548.reads Coverage
Position relative to miRNA 5' end

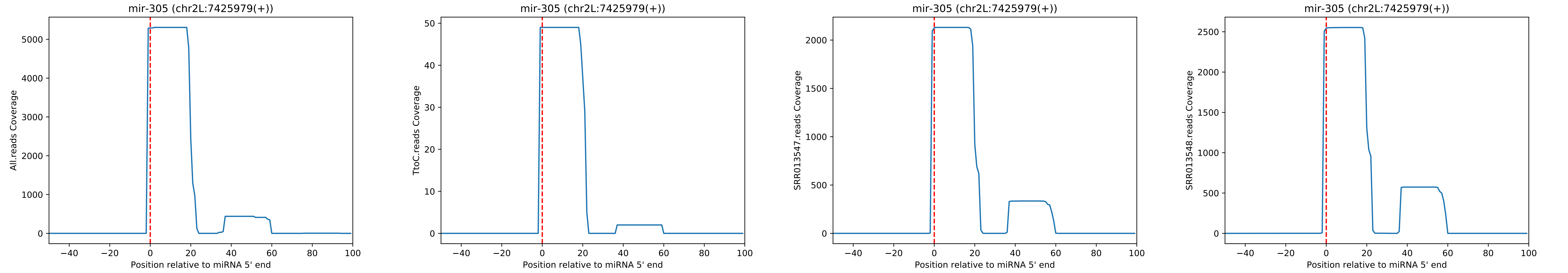
mir-305 (chr2L:7425979(+))
All.reads Coverage
Position relative to miRNA 5' end
mir-305 (chr2L:7425979(+))
TtoC.reads Coverage
Position relative to miRNA 5' end
mir-305 (chr2L:7425979(+))
SRR013547.reads Coverage
Position relative to miRNA 5' end
mir-305 (chr2L:7425979(+))
SRR013548.reads Coverage
Position relative to miRNA 5' end

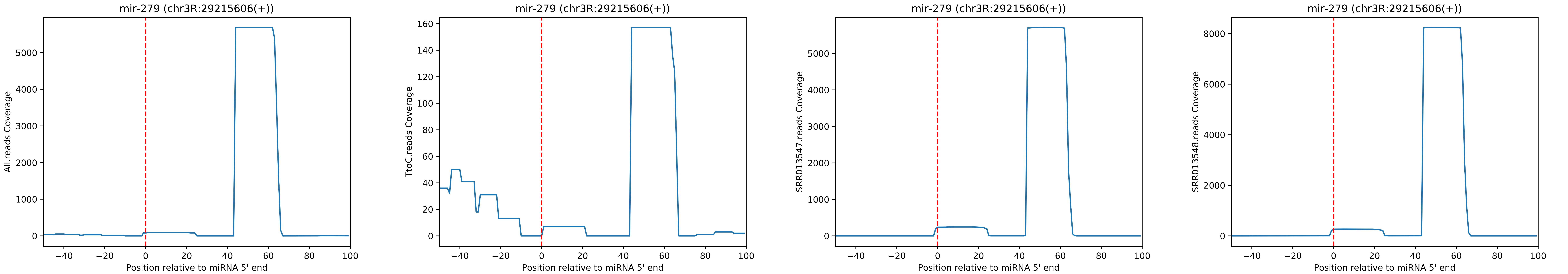
mir-279 (chr3R:29215606(+))
All.reads Coverage
Position relative to miRNA 5' end
mir-279 (chr3R:29215606(+))
TtoC.reads Coverage
Position relative to miRNA 5' end
mir-279 (chr3R:29215606(+))
SRR013547.reads Coverage
Position relative to miRNA 5' end
mir-279 (chr3R:29215606(+))
SRR013548.reads Coverage
Position relative to miRNA 5' end

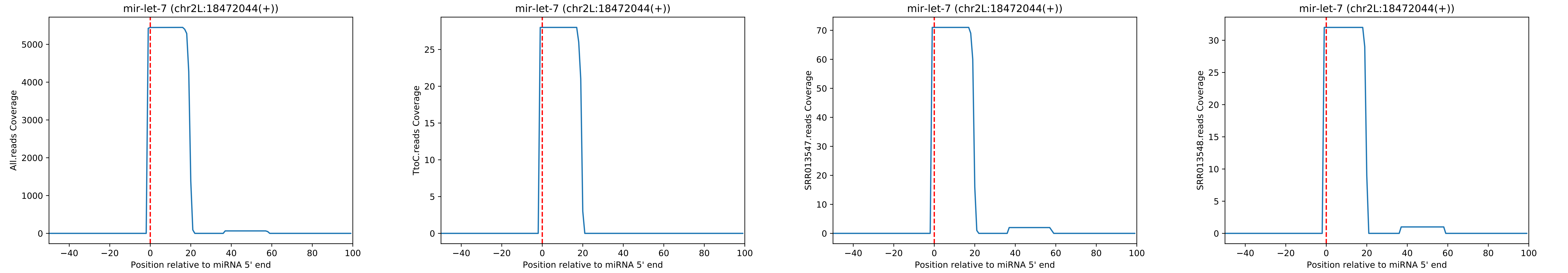
mir-let-7 (chr2L:18472044(+))
All.reads Coverage
Position relative to miRNA 5' end
mir-let-7 (chr2L:18472044(+))
TtoC.reads Coverage
Position relative to miRNA 5' end
mir-let-7 (chr2L:18472044(+))
SRR013547.reads Coverage
Position relative to miRNA 5' end
mir-let-7 (chr2L:18472044(+))
SRR013548.reads Coverage
Position relative to miRNA 5' end

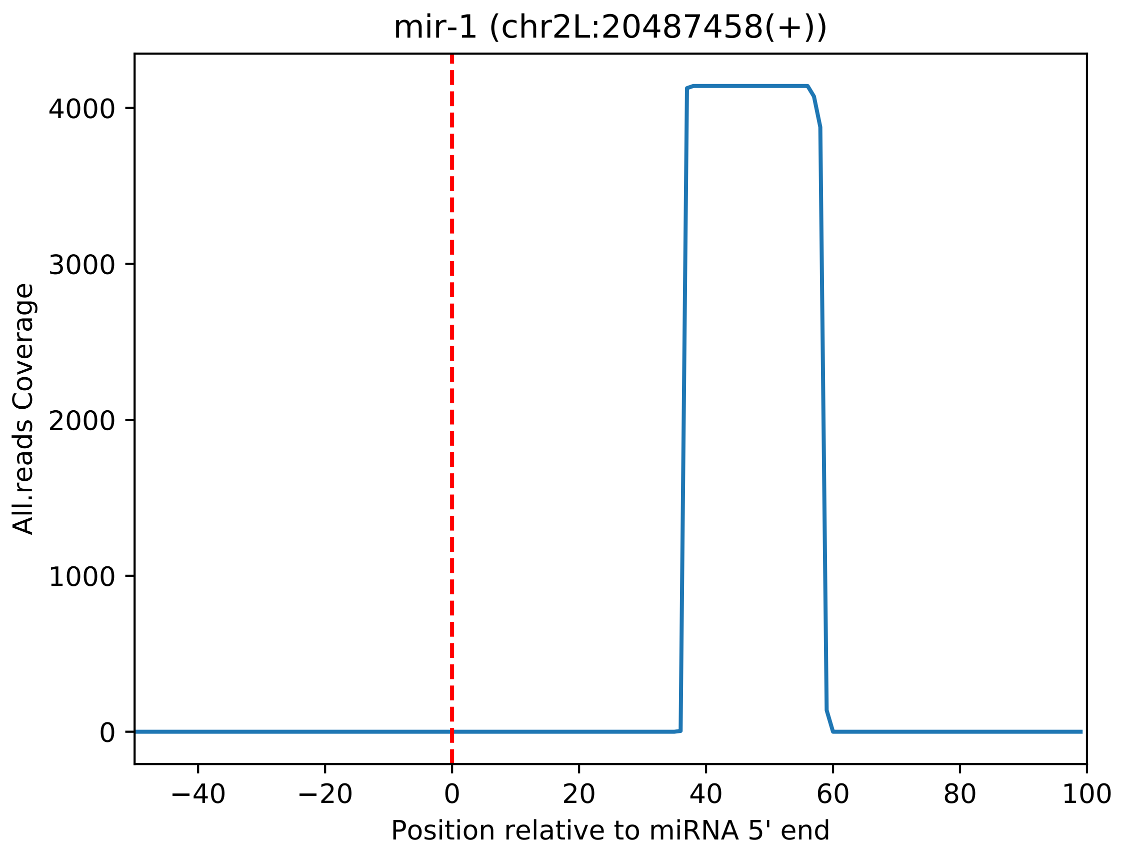
mir-1 (chr2L:20487458(+))
All.reads Coverage
Position relative to miRNA 5' end
0
1000
2000
3000
4000
−40
−20
0
20
40
60
80
100

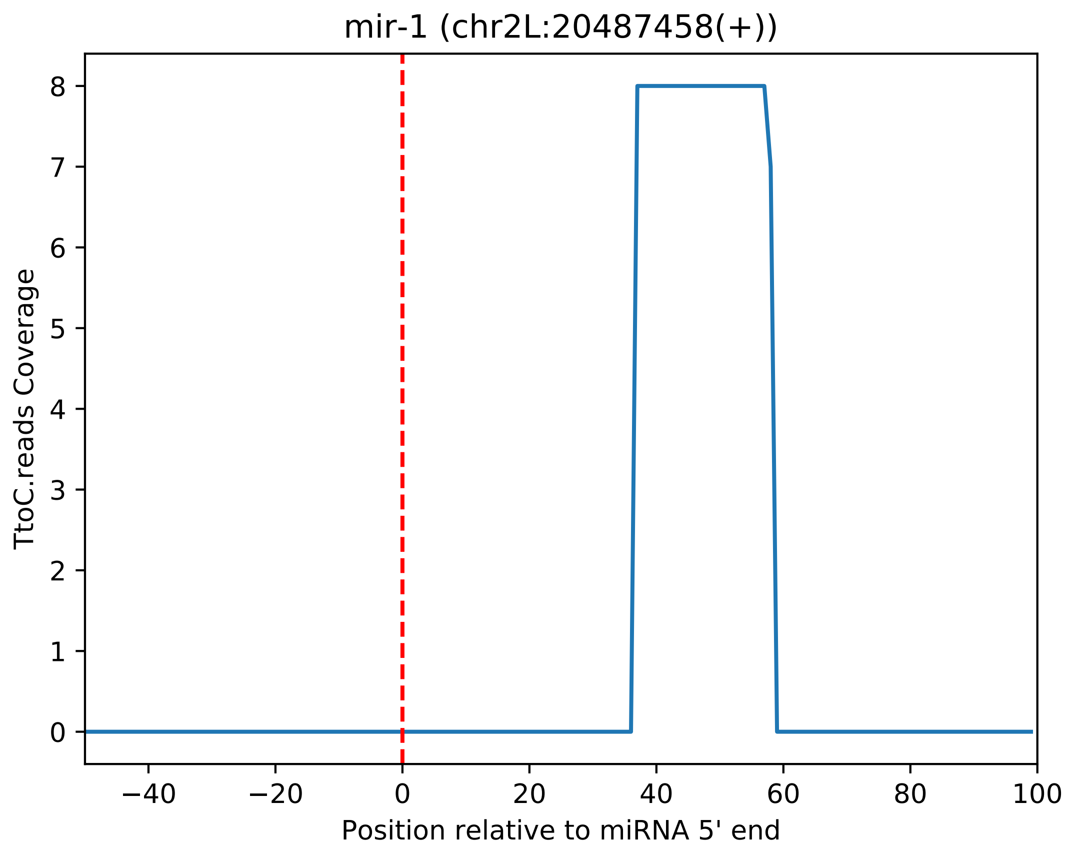
mir-1 (chr2L:20487458(+))
TtoC.reads Coverage
Position relative to miRNA 5' end
0
1
2
3
4
5
6
7
8
−40
−20
0
20
40
60
80
100

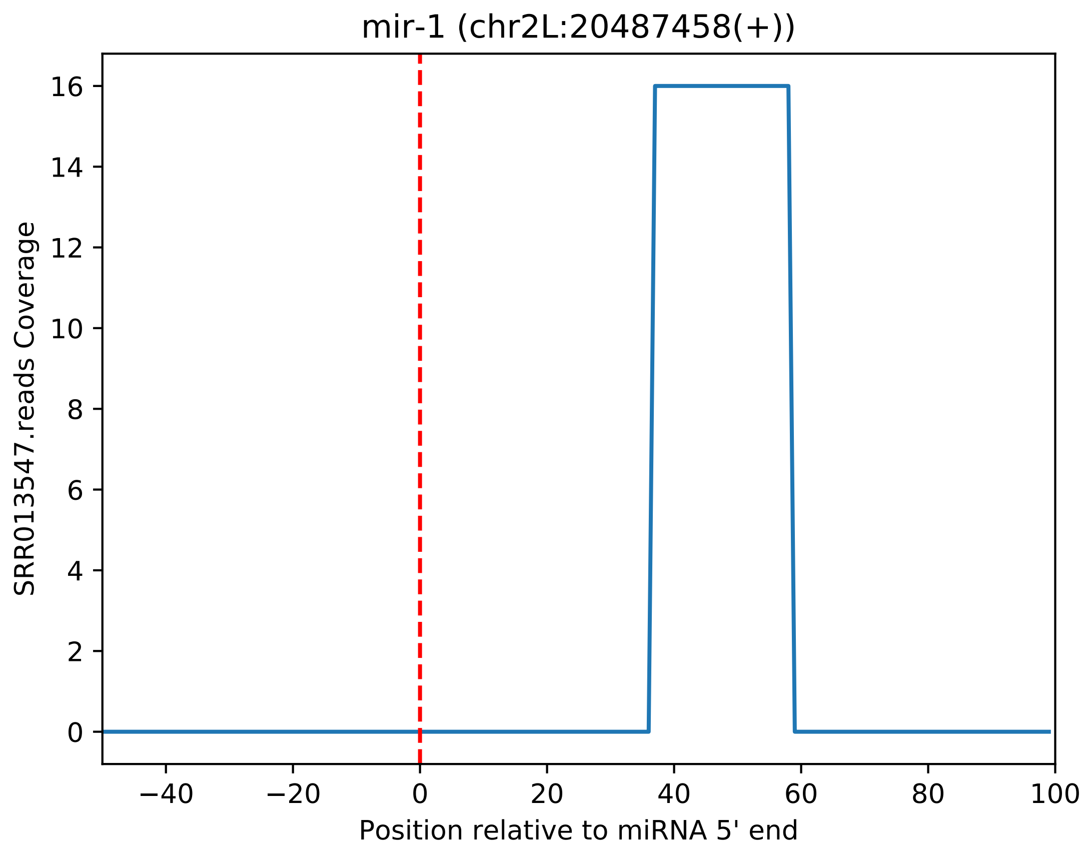
mir-1 (chr2L:20487458(+))
SRR013547.reads Coverage
Position relative to miRNA 5' end
0
2
4
6
8
10
12
14
16
−40
−20
0
20
40
60
80
100

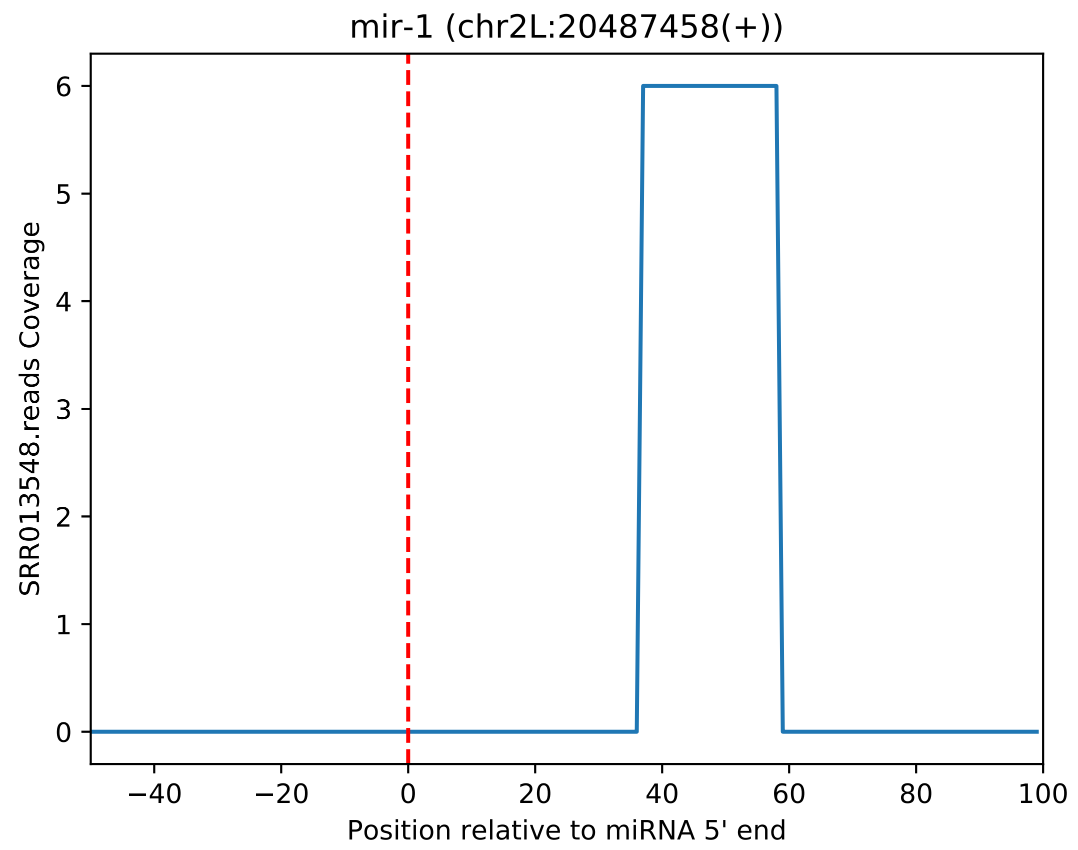
mir-1 (chr2L:20487458(+))
SRR013548.reads Coverage
Position relative to miRNA 5' end
0
1
2
3
4
5
6
−40
−20
0
20
40
60
80
100

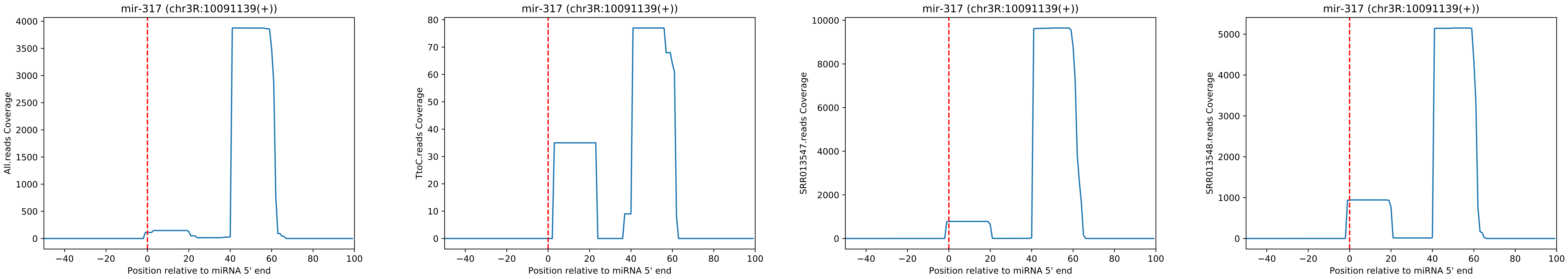

mir-317 (chr3R:10091139(+))
All.reads Coverage
Position relative to miRNA 5' end
mir-317 (chr3R:10091139(+))
TtoC.reads Coverage
Position relative to miRNA 5' end
mir-317 (chr3R:10091139(+))
SRR013547.reads Coverage
Position relative to miRNA 5' end
mir-317 (chr3R:10091139(+))
SRR013548.reads Coverage
Position relative to miRNA 5' end

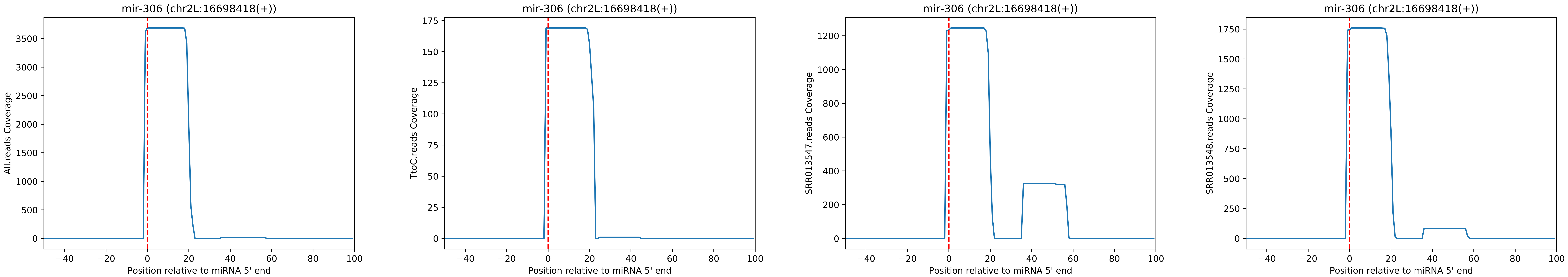
mir-306 (chr2L:16698418(+))
All.reads Coverage
Position relative to miRNA 5' end
mir-306 (chr2L:16698418(+))
TtoC.reads Coverage
Position relative to miRNA 5' end
mir-306 (chr2L:16698418(+))
SRR013547.reads Coverage
Position relative to miRNA 5' end
mir-306 (chr2L:16698418(+))
SRR013548.reads Coverage
Position relative to miRNA 5' end

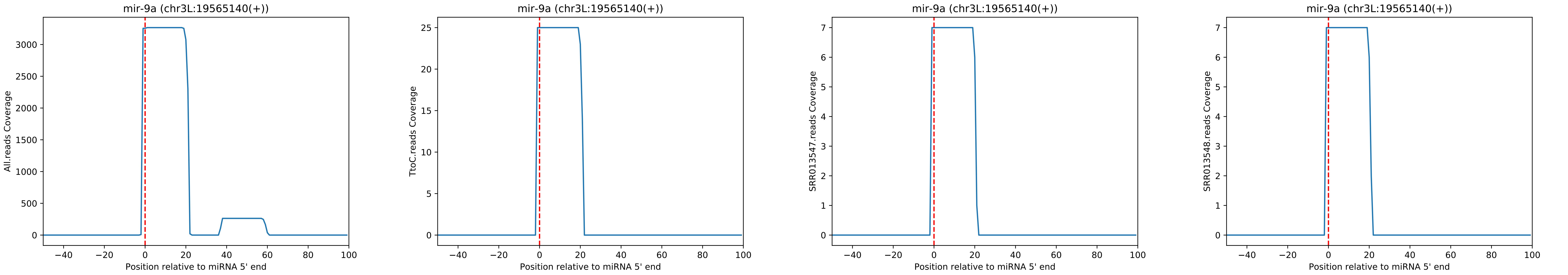
mir-9a (chr3L:19565140(+))
All.reads Coverage
Position relative to miRNA 5' end
mir-9a (chr3L:19565140(+))
TtoC.reads Coverage
Position relative to miRNA 5' end
mir-9a (chr3L:19565140(+))
SRR013547.reads Coverage
Position relative to miRNA 5' end
mir-9a (chr3L:19565140(+))
SRR013548.reads Coverage
Position relative to miRNA 5' end

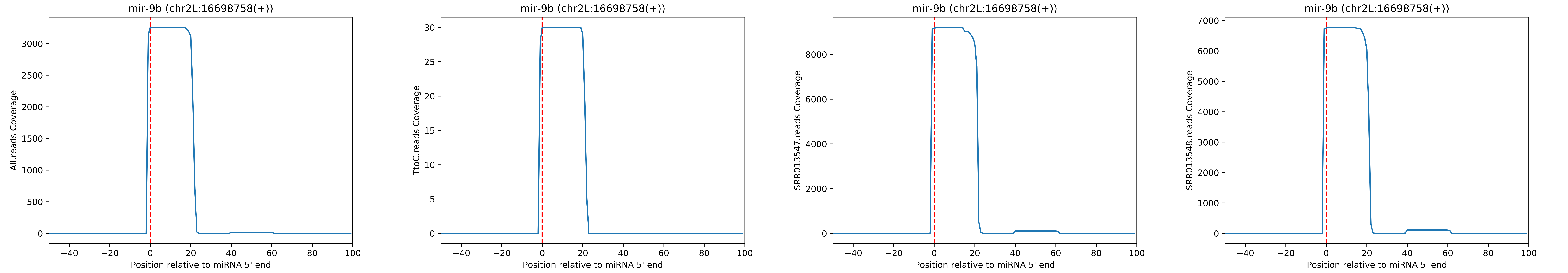

mir-9b (chr2L:16698758(+))
All.reads Coverage
0
500
1000
1500
2000
2500
3000
Position relative to miRNA 5' end
−40
−20
0
20
40
60
80
100
mir-9b (chr2L:16698758(+))
TtoC.reads Coverage
0
5
10
15
20
25
30
Position relative to miRNA 5' end
−40
−20
0
20
40
60
80
100
mir-9b (chr2L:16698758(+))
SRR013547.reads Coverage
0
2000
4000
6000
8000
Position relative to miRNA 5' end
−40
−20
0
20
40
60
80
100
mir-9b (chr2L:16698758(+))
SRR013548.reads Coverage
0
1000
2000
3000
4000
5000
6000
7000
Position relative to miRNA 5' end
−40
−20
0
20
40
60
80
100

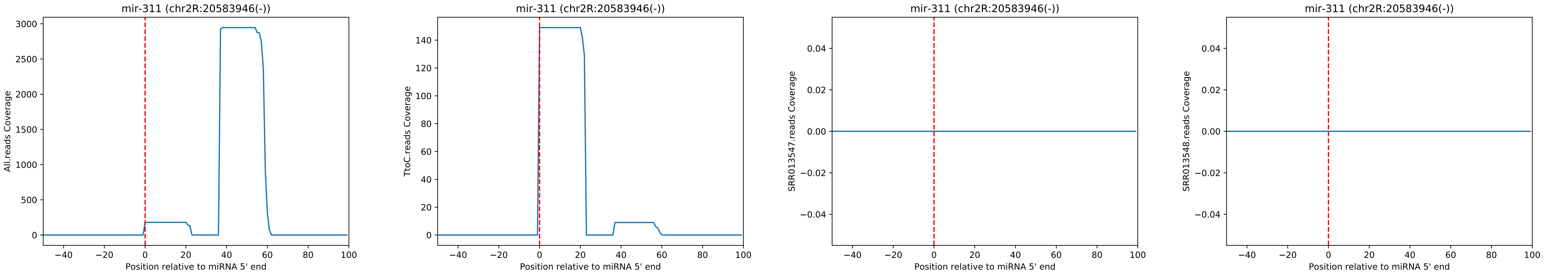
mir-311 (chr2R:20583946(-))
All.reads Coverage
Position relative to miRNA 5' end
mir-311 (chr2R:20583946(-))
TtoC.reads Coverage
Position relative to miRNA 5' end
mir-311 (chr2R:20583946(-))
SRR013547.reads Coverage
Position relative to miRNA 5' end
mir-311 (chr2R:20583946(-))
SRR013548.reads Coverage
Position relative to miRNA 5' end

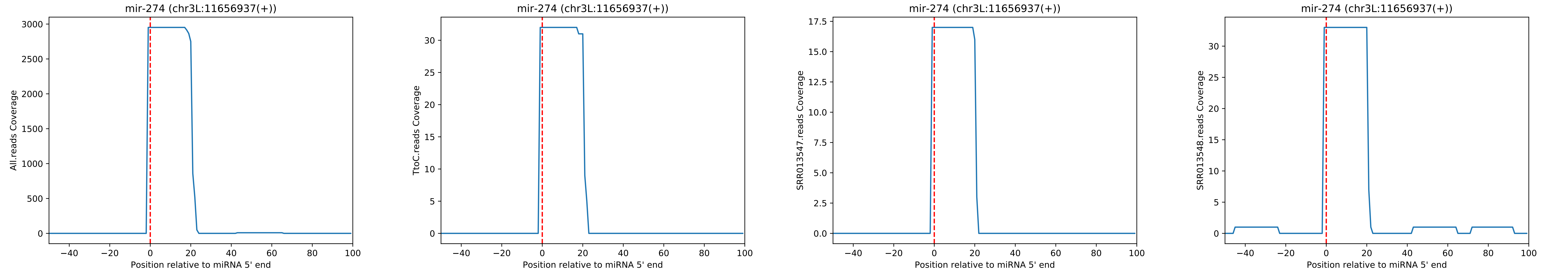

mir-274 (chr3L:11656937(+))
All.reads Coverage
0
500
1000
1500
2000
2500
3000
−40
−20
0
20
40
60
80
100
Position relative to miRNA 5' end
mir-274 (chr3L:11656937(+))
TtoC.reads Coverage
0
5
10
15
20
25
30
−40
−20
0
20
40
60
80
100
Position relative to miRNA 5' end
mir-274 (chr3L:11656937(+))
SRR013547.reads Coverage
0.0
2.5
5.0
7.5
10.0
12.5
15.0
17.5
−40
−20
0
20
40
60
80
100
Position relative to miRNA 5' end
mir-274 (chr3L:11656937(+))
SRR013548.reads Coverage
0
5
10
15
20
25
30
−40
−20
0
20
40
60
80
100
Position relative to miRNA 5' end

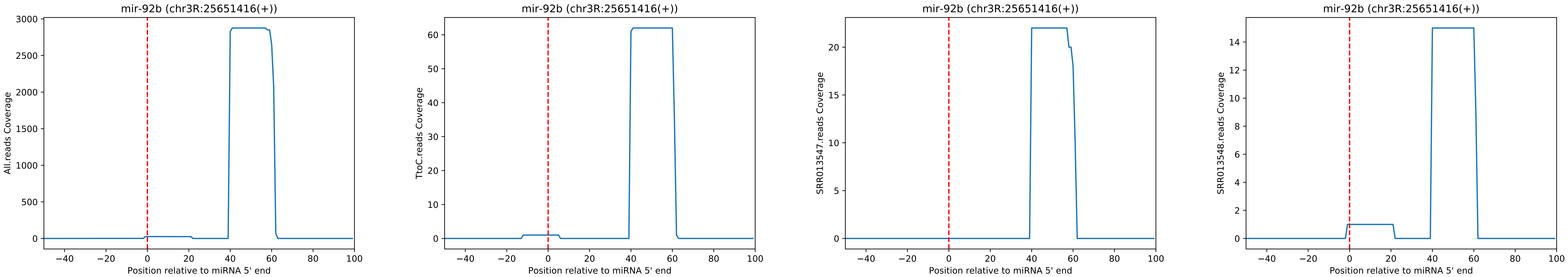
mir-92b (chr3R:25651416(+))
All.reads Coverage
Position relative to miRNA 5' end
mir-92b (chr3R:25651416(+))
TtoC.reads Coverage
Position relative to miRNA 5' end
mir-92b (chr3R:25651416(+))
SRR013547.reads Coverage
Position relative to miRNA 5' end
mir-92b (chr3R:25651416(+))
SRR013548.reads Coverage
Position relative to miRNA 5' end

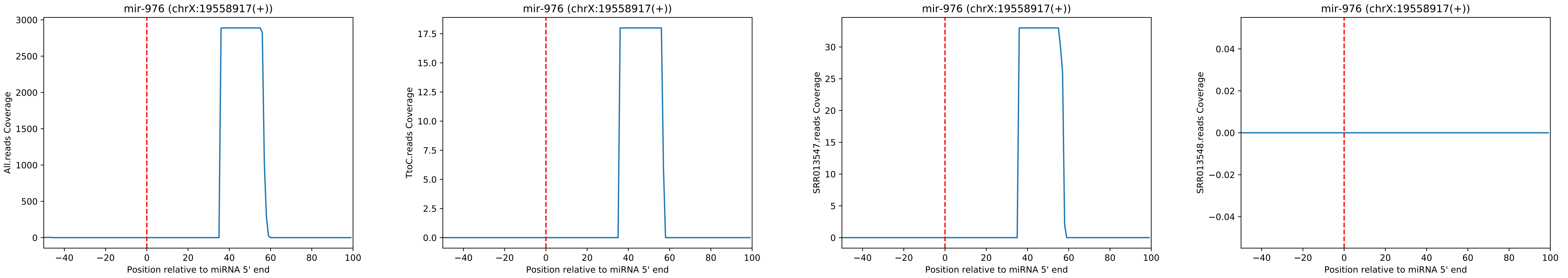
mir-976 (chrX:19558917(+))
All.reads Coverage
Position relative to miRNA 5' end
mir-976 (chrX:19558917(+))
TtoC.reads Coverage
Position relative to miRNA 5' end
mir-976 (chrX:19558917(+))
SRR013547.reads Coverage
Position relative to miRNA 5' end
mir-976 (chrX:19558917(+))
SRR013548.reads Coverage
Position relative to miRNA 5' end

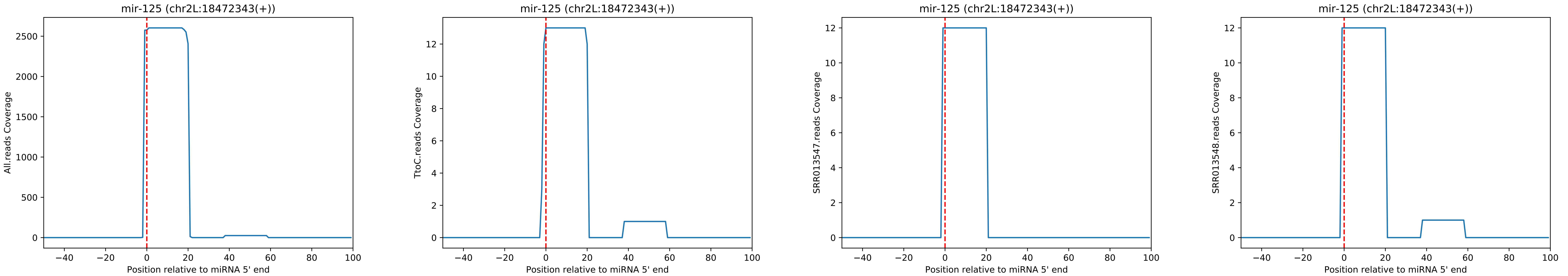

mir-125 (chr2L:18472343(+))
All.reads Coverage
Position relative to miRNA 5' end
mir-125 (chr2L:18472343(+))
TtoC.reads Coverage
Position relative to miRNA 5' end
mir-125 (chr2L:18472343(+))
SRR013547.reads Coverage
Position relative to miRNA 5' end
mir-125 (chr2L:18472343(+))
SRR013548.reads Coverage
Position relative to miRNA 5' end

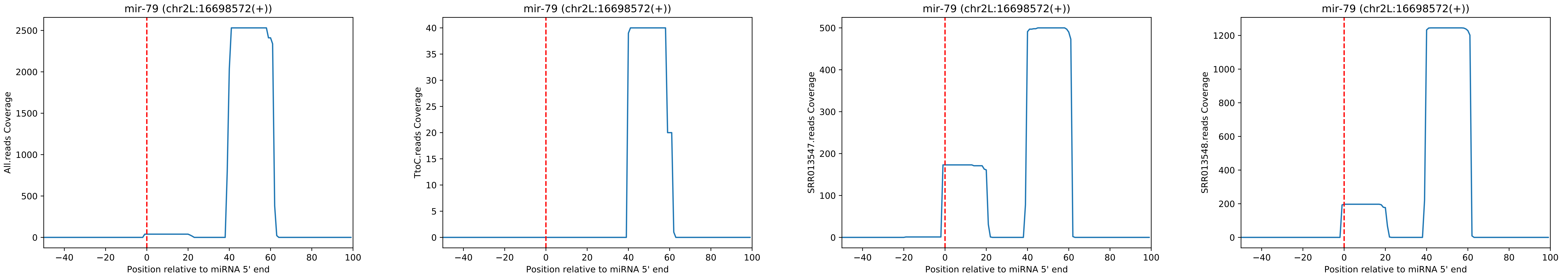

mir-79 (chr2L:16698572(+))
All.reads Coverage
Position relative to miRNA 5' end
mir-79 (chr2L:16698572(+))
TtoC.reads Coverage
Position relative to miRNA 5' end
mir-79 (chr2L:16698572(+))
SRR013547.reads Coverage
Position relative to miRNA 5' end
mir-79 (chr2L:16698572(+))
SRR013548.reads Coverage
Position relative to miRNA 5' end

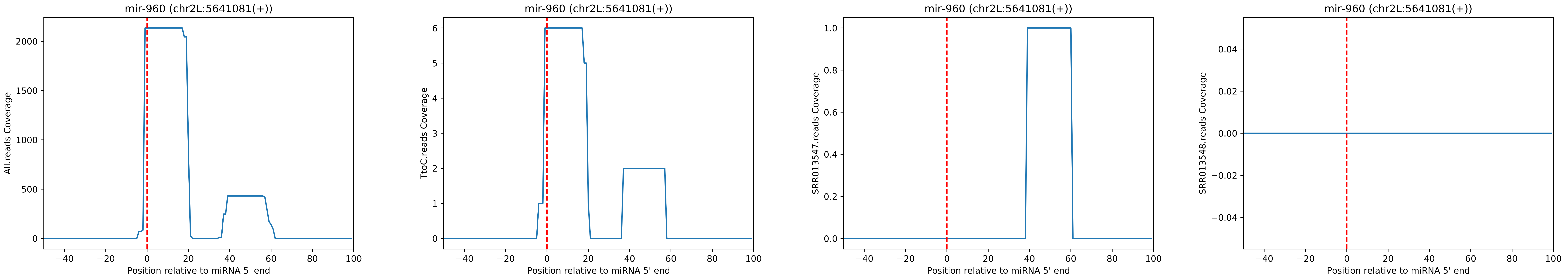

mir-960 (chr2L:5641081(+))
All.reads Coverage
Position relative to miRNA 5' end
mir-960 (chr2L:5641081(+))
TtoC.reads Coverage
Position relative to miRNA 5' end
mir-960 (chr2L:5641081(+))
SRR013547.reads Coverage
Position relative to miRNA 5' end
mir-960 (chr2L:5641081(+))
SRR013548.reads Coverage
Position relative to miRNA 5' end

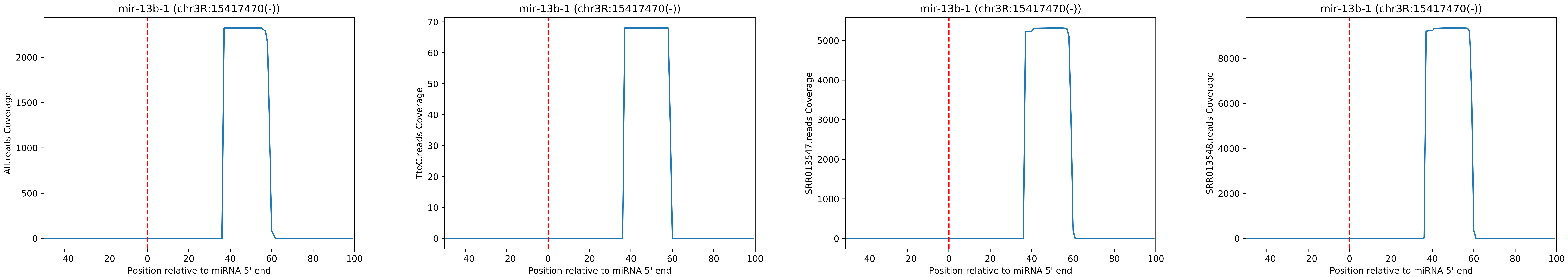
mir-13b-1 (chr3R:15417470(-))
All.reads Coverage
Position relative to miRNA 5' end
mir-13b-1 (chr3R:15417470(-))
TtoC.reads Coverage
Position relative to miRNA 5' end
mir-13b-1 (chr3R:15417470(-))
SRR013547.reads Coverage
Position relative to miRNA 5' end
mir-13b-1 (chr3R:15417470(-))
SRR013548.reads Coverage
Position relative to miRNA 5' end

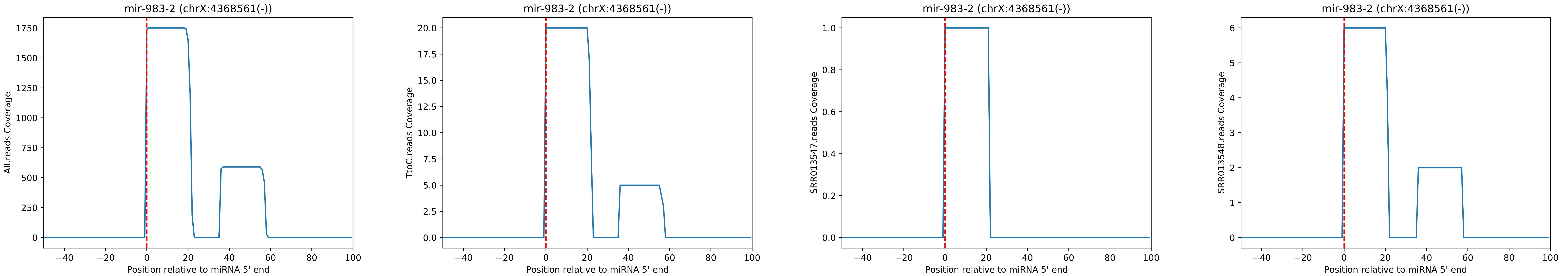
mir-983-2 (chrX:4368561(-))
All.reads Coverage
TtoC.reads Coverage
SRR013547.reads Coverage
SRR013548.reads Coverage
Position relative to miRNA 5' end

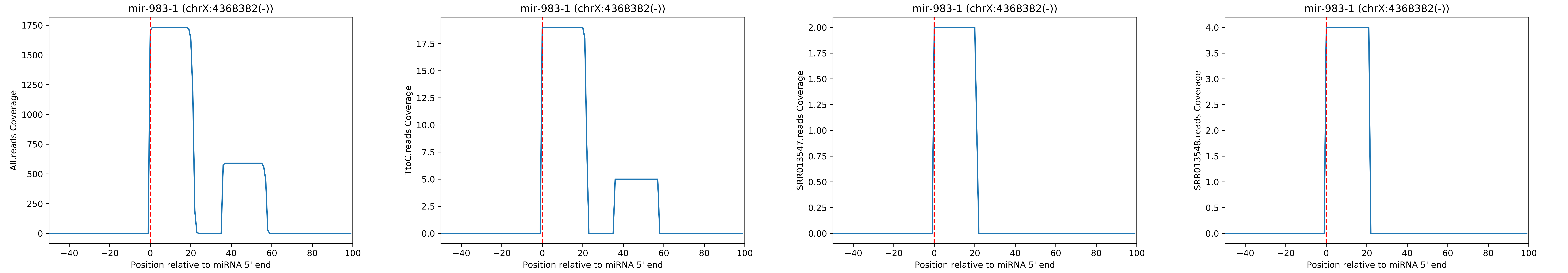
mir-983-1 (chrX:4368382(-))
All.reads Coverage
Position relative to miRNA 5' end
mir-983-1 (chrX:4368382(-))
TtoC.reads Coverage
Position relative to miRNA 5' end
mir-983-1 (chrX:4368382(-))
SRR013547.reads Coverage
Position relative to miRNA 5' end
mir-983-1 (chrX:4368382(-))
SRR013548.reads Coverage
Position relative to miRNA 5' end

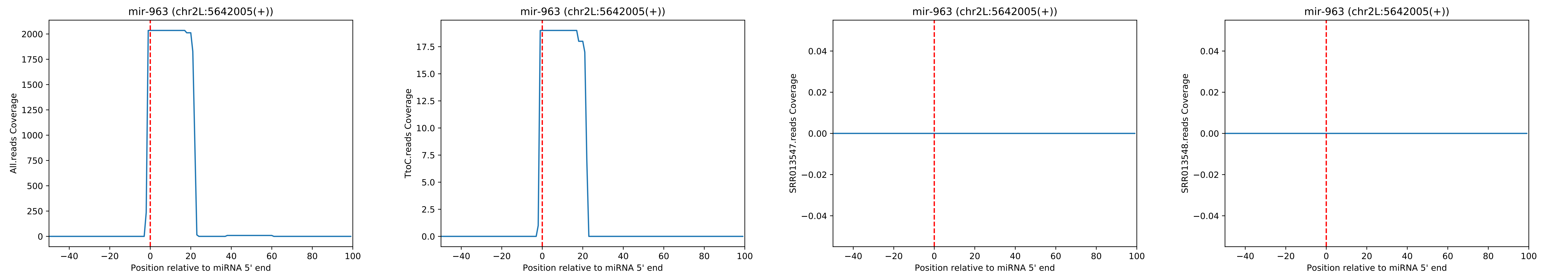
mir-963 (chr2L:5642005(+))
All.reads Coverage
Position relative to miRNA 5' end
mir-963 (chr2L:5642005(+))
TtoC.reads Coverage
Position relative to miRNA 5' end
mir-963 (chr2L:5642005(+))
SRR013547.reads Coverage
Position relative to miRNA 5' end
mir-963 (chr2L:5642005(+))
SRR013548.reads Coverage
Position relative to miRNA 5' end

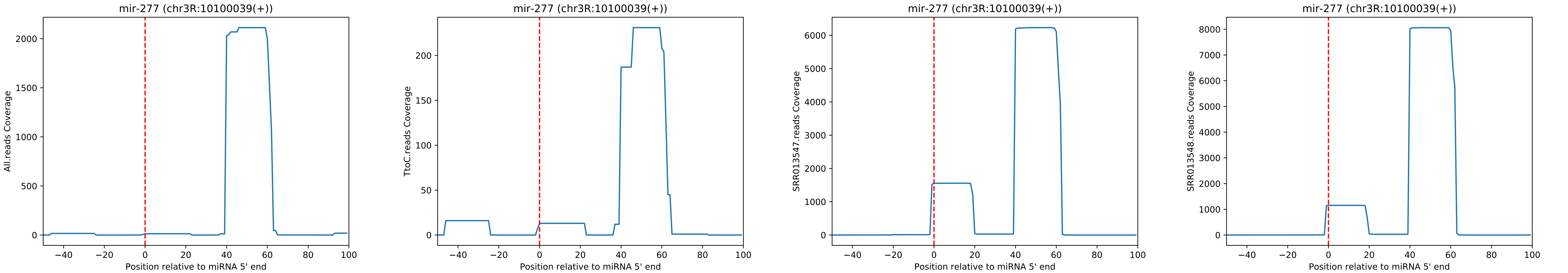
mir-277 (chr3R:10100039(+))
All.reads Coverage
Position relative to miRNA 5' end
mir-277 (chr3R:10100039(+))
TtoC.reads Coverage
Position relative to miRNA 5' end
mir-277 (chr3R:10100039(+))
SRR013547.reads Coverage
Position relative to miRNA 5' end
mir-277 (chr3R:10100039(+))
SRR013548.reads Coverage
Position relative to miRNA 5' end

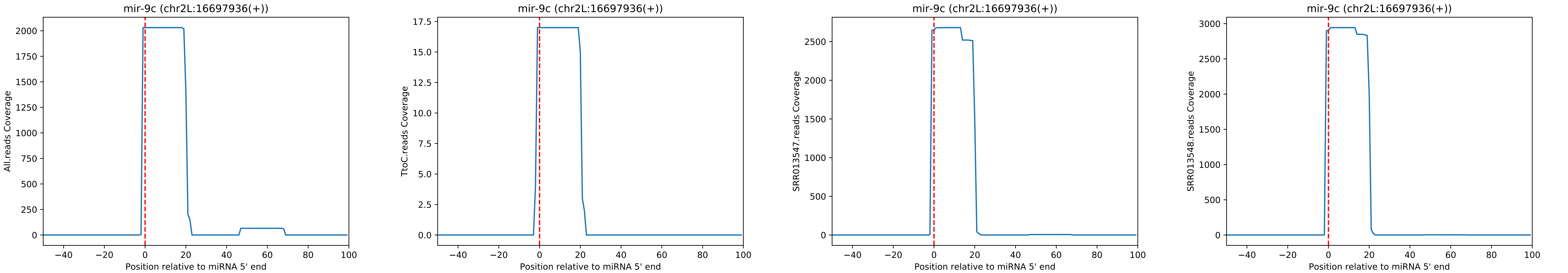

mir-9c (chr2L:16697936(+))
All.reads Coverage
Position relative to miRNA 5' end
mir-9c (chr2L:16697936(+))
TtoC.reads Coverage
Position relative to miRNA 5' end
mir-9c (chr2L:16697936(+))
SRR013547.reads Coverage
Position relative to miRNA 5' end
mir-9c (chr2L:16697936(+))
SRR013548.reads Coverage
Position relative to miRNA 5' end

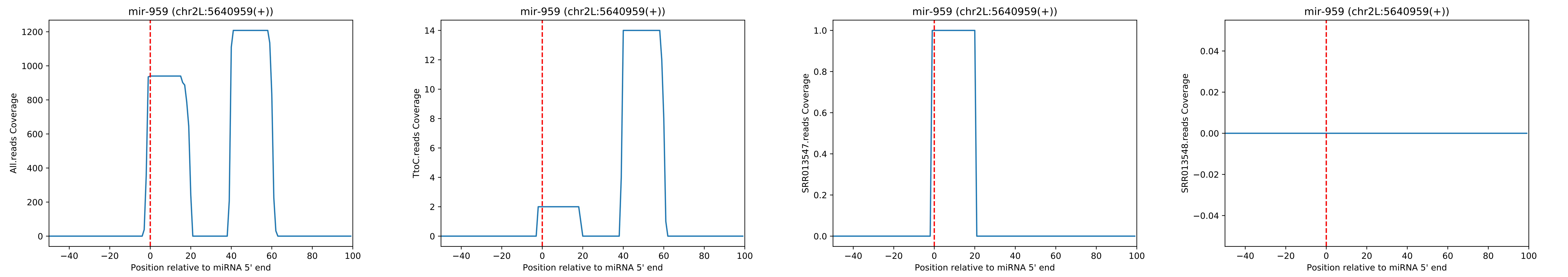
mir-959 (chr2L:5640959(+))
All.reads Coverage
Position relative to miRNA 5' end
mir-959 (chr2L:5640959(+))
TtoC.reads Coverage
Position relative to miRNA 5' end
mir-959 (chr2L:5640959(+))
SRR013547.reads Coverage
Position relative to miRNA 5' end
mir-959 (chr2L:5640959(+))
SRR013548.reads Coverage
Position relative to miRNA 5' end

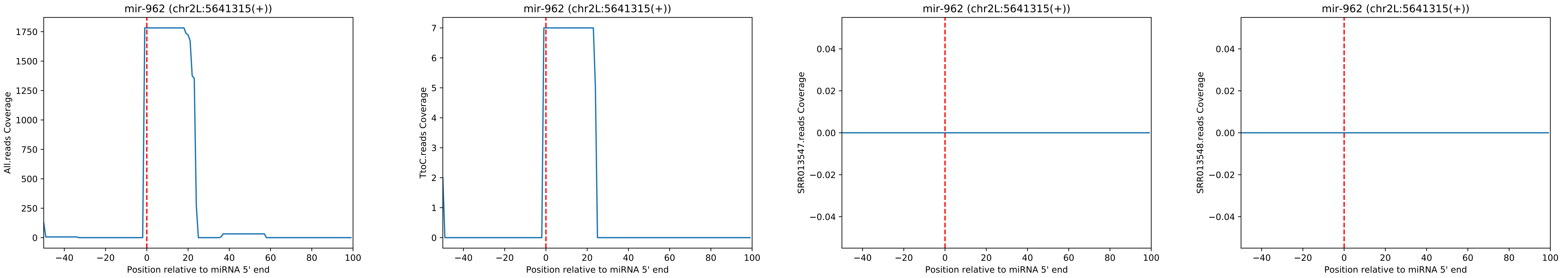
mir-962 (chr2L:5641315(+))
All.reads Coverage
Position relative to miRNA 5' end
mir-962 (chr2L:5641315(+))
TtoC.reads Coverage
Position relative to miRNA 5' end
mir-962 (chr2L:5641315(+))
SRR013547.reads Coverage
Position relative to miRNA 5' end
mir-962 (chr2L:5641315(+))
SRR013548.reads Coverage
Position relative to miRNA 5' end

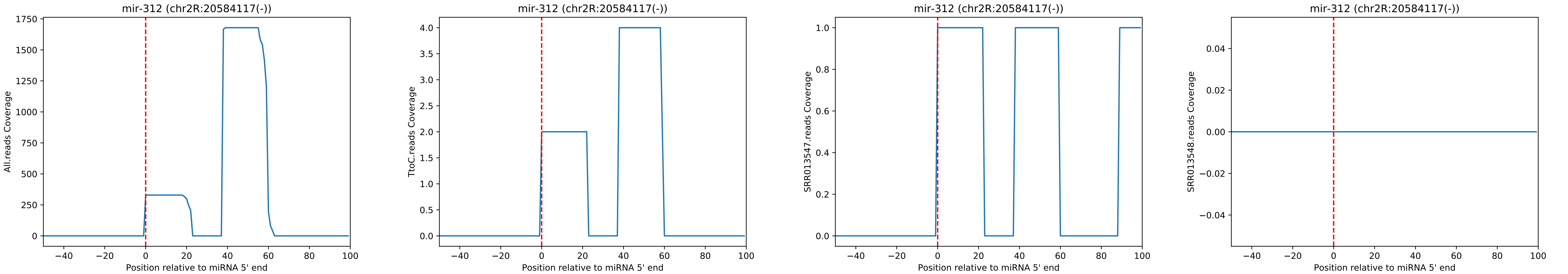
mir-312 (chr2R:20584117(-))
All.reads Coverage
TtoC.reads Coverage
SRR013547.reads Coverage
SRR013548.reads Coverage
Position relative to miRNA 5' end

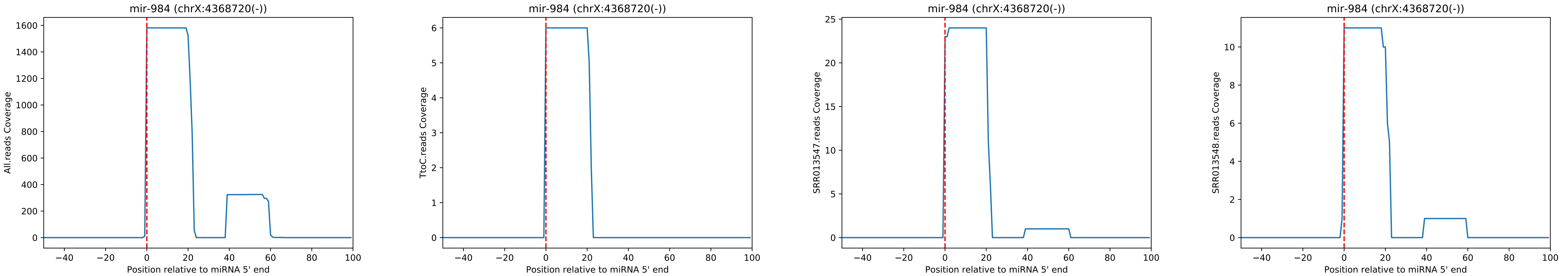
mir-984 (chrX:4368720(-))
All.reads Coverage
Position relative to miRNA 5' end
mir-984 (chrX:4368720(-))
TtoC.reads Coverage
Position relative to miRNA 5' end
mir-984 (chrX:4368720(-))
SRR013547.reads Coverage
Position relative to miRNA 5' end
mir-984 (chrX:4368720(-))
SRR013548.reads Coverage
Position relative to miRNA 5' end

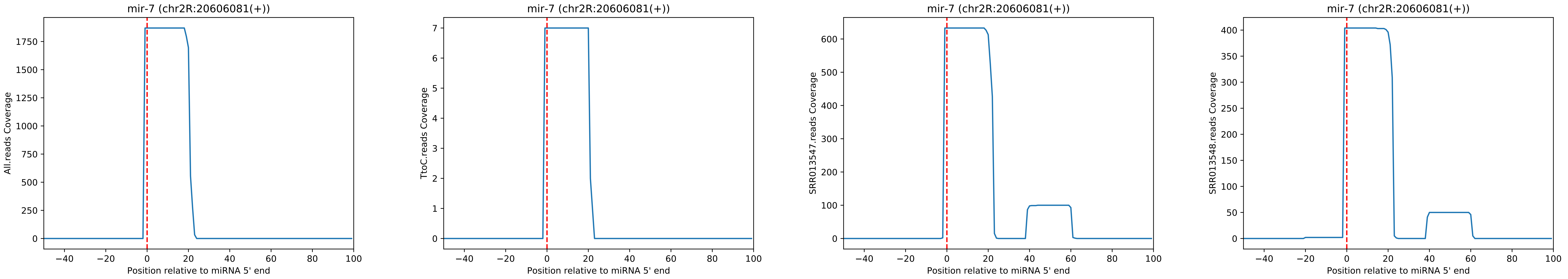
mir-7 (chr2R:20606081(+))
All.reads Coverage
Position relative to miRNA 5' end
mir-7 (chr2R:20606081(+))
TtoC.reads Coverage
Position relative to miRNA 5' end
mir-7 (chr2R:20606081(+))
SRR013547.reads Coverage
Position relative to miRNA 5' end
mir-7 (chr2R:20606081(+))
SRR013548.reads Coverage
Position relative to miRNA 5' end

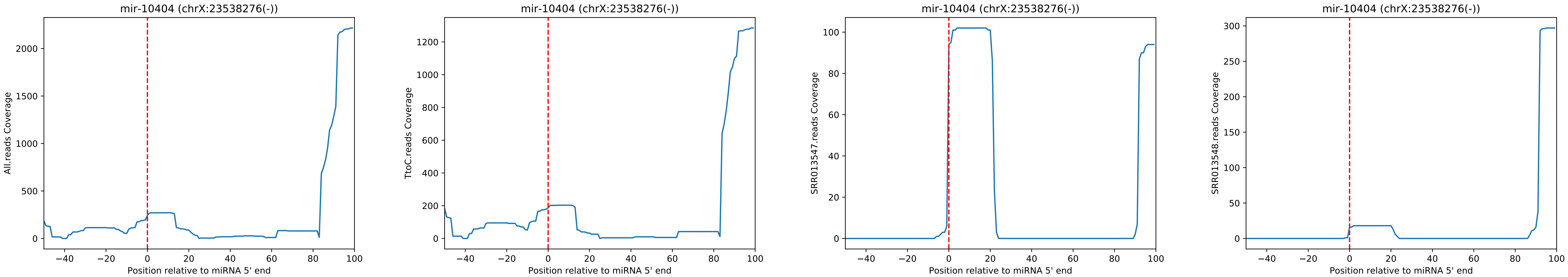
mir-10404 (chrX:23538276(-))
All.reads Coverage
Position relative to miRNA 5' end
mir-10404 (chrX:23538276(-))
TtoC.reads Coverage
Position relative to miRNA 5' end
mir-10404 (chrX:23538276(-))
SRR013547.reads Coverage
Position relative to miRNA 5' end
mir-10404 (chrX:23538276(-))
SRR013548.reads Coverage
Position relative to miRNA 5' end

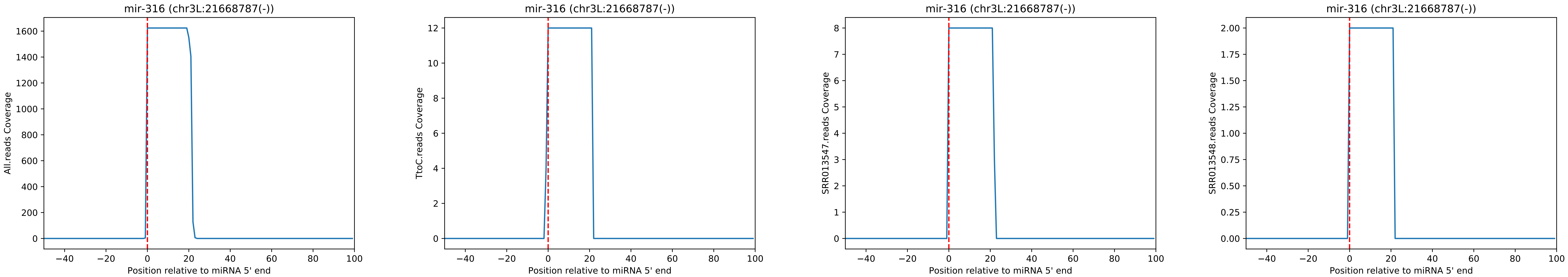

mir-316 (chr3L:21668787(-))
All.reads Coverage
Position relative to miRNA 5' end
mir-316 (chr3L:21668787(-))
TtoC.reads Coverage
Position relative to miRNA 5' end
mir-316 (chr3L:21668787(-))
SRR013547.reads Coverage
Position relative to miRNA 5' end
mir-316 (chr3L:21668787(-))
SRR013548.reads Coverage
Position relative to miRNA 5' end

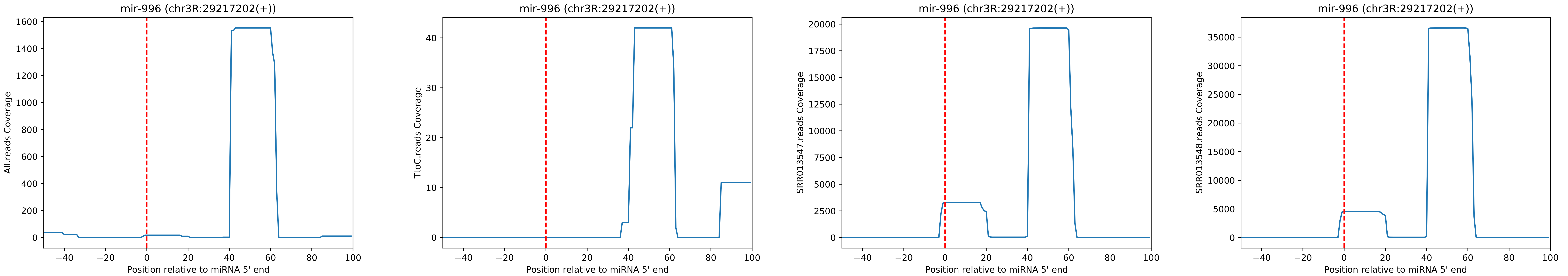
mir-996 (chr3R:29217202(+))
All.reads Coverage
Position relative to miRNA 5' end
mir-996 (chr3R:29217202(+))
TtoC.reads Coverage
Position relative to miRNA 5' end
mir-996 (chr3R:29217202(+))
SRR013547.reads Coverage
Position relative to miRNA 5' end
mir-996 (chr3R:29217202(+))
SRR013548.reads Coverage
Position relative to miRNA 5' end

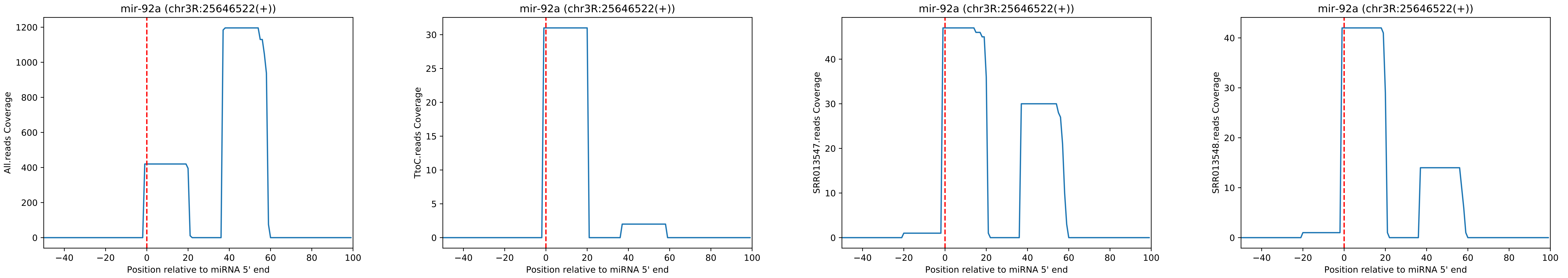
mir-92a (chr3R:25646522(+))
All.reads Coverage
Position relative to miRNA 5' end
mir-92a (chr3R:25646522(+))
TtoC.reads Coverage
Position relative to miRNA 5' end
mir-92a (chr3R:25646522(+))
SRR013547.reads Coverage
Position relative to miRNA 5' end
mir-92a (chr3R:25646522(+))
SRR013548.reads Coverage
Position relative to miRNA 5' end

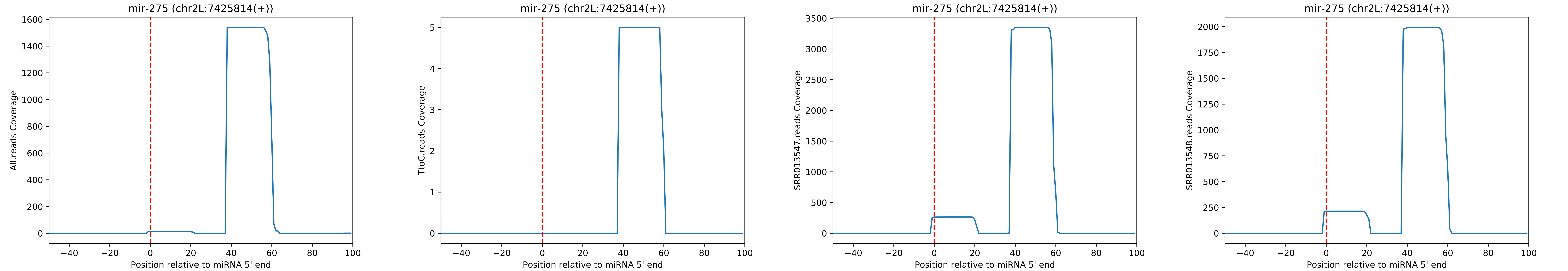

mir-275 (chr2L:7425814(+))
All.reads Coverage
Position relative to miRNA 5' end
mir-275 (chr2L:7425814(+))
TtoC.reads Coverage
Position relative to miRNA 5' end
mir-275 (chr2L:7425814(+))
SRR013547.reads Coverage
Position relative to miRNA 5' end
mir-275 (chr2L:7425814(+))
SRR013548.reads Coverage
Position relative to miRNA 5' end

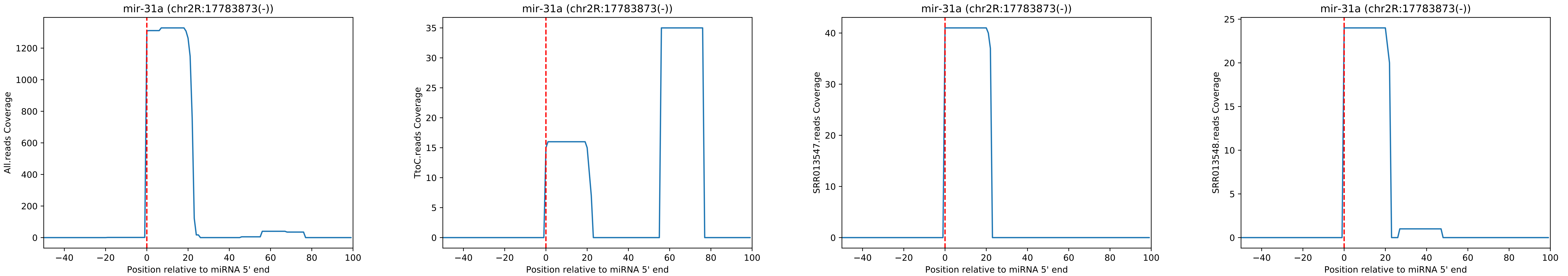
mir-31a (chr2R:17783873(-))
All.reads Coverage
Position relative to miRNA 5' end
mir-31a (chr2R:17783873(-))
TtoC.reads Coverage
Position relative to miRNA 5' end
mir-31a (chr2R:17783873(-))
SRR013547.reads Coverage
Position relative to miRNA 5' end
mir-31a (chr2R:17783873(-))
SRR013548.reads Coverage
Position relative to miRNA 5' end

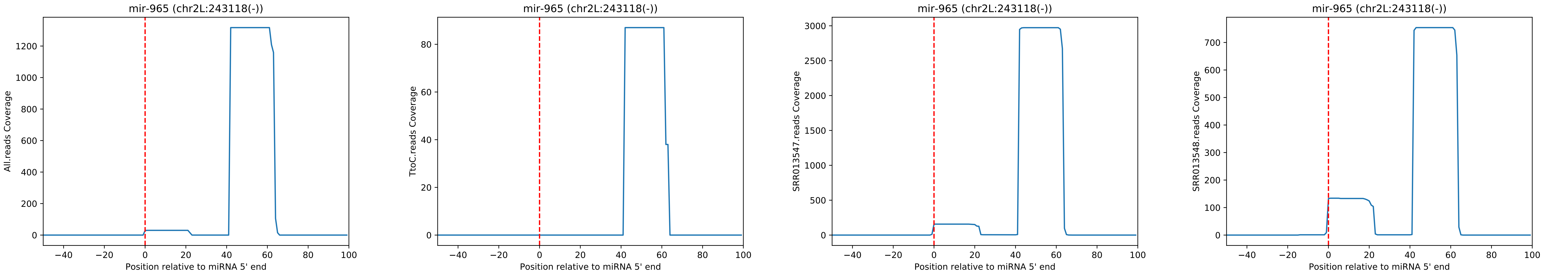
mir-965 (chr2L:243118(-))
All.reads Coverage
Position relative to miRNA 5' end
mir-965 (chr2L:243118(-))
TtoC.reads Coverage
Position relative to miRNA 5' end
mir-965 (chr2L:243118(-))
SRR013547.reads Coverage
Position relative to miRNA 5' end
mir-965 (chr2L:243118(-))
SRR013548.reads Coverage
Position relative to miRNA 5' end

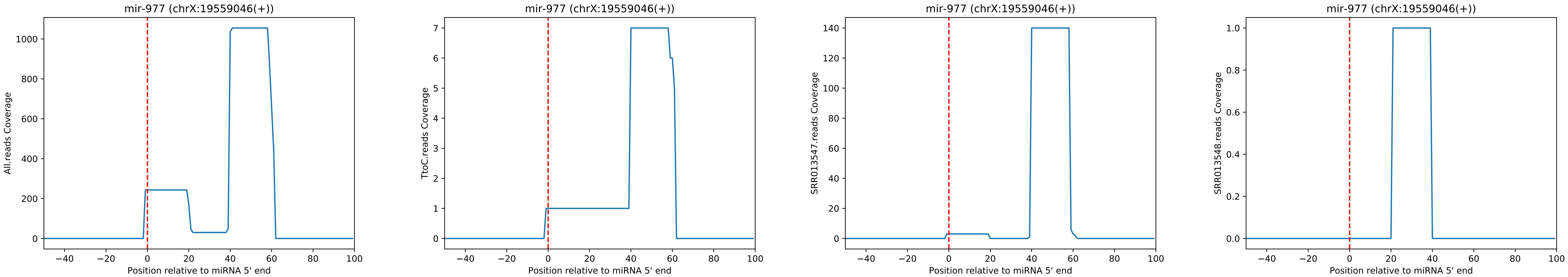
mir-977 (chrX:19559046(+))
All.reads Coverage
Position relative to miRNA 5' end
mir-977 (chrX:19559046(+))
TtoC.reads Coverage
Position relative to miRNA 5' end
mir-977 (chrX:19559046(+))
SRR013547.reads Coverage
Position relative to miRNA 5' end
mir-977 (chrX:19559046(+))
SRR013548.reads Coverage
Position relative to miRNA 5' end

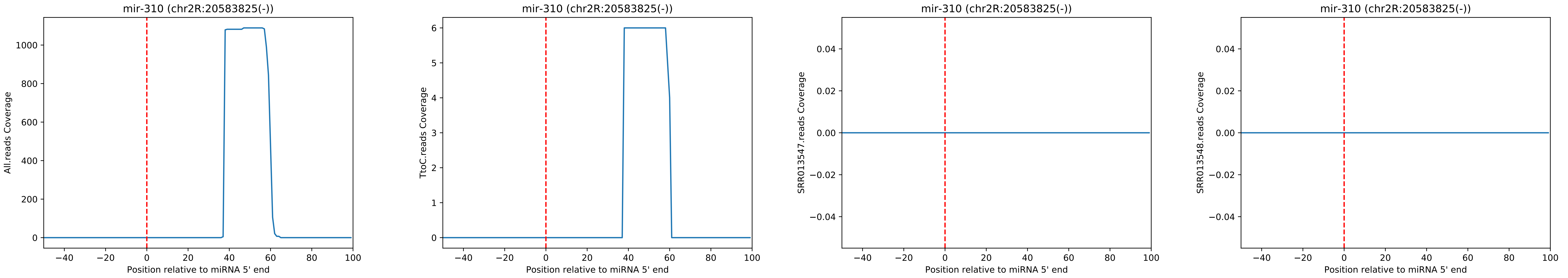
mir-310 (chr2R:20583825(-))
All.reads Coverage
TtoC.reads Coverage
SRR013547.reads Coverage
SRR013548.reads Coverage
Position relative to miRNA 5' end

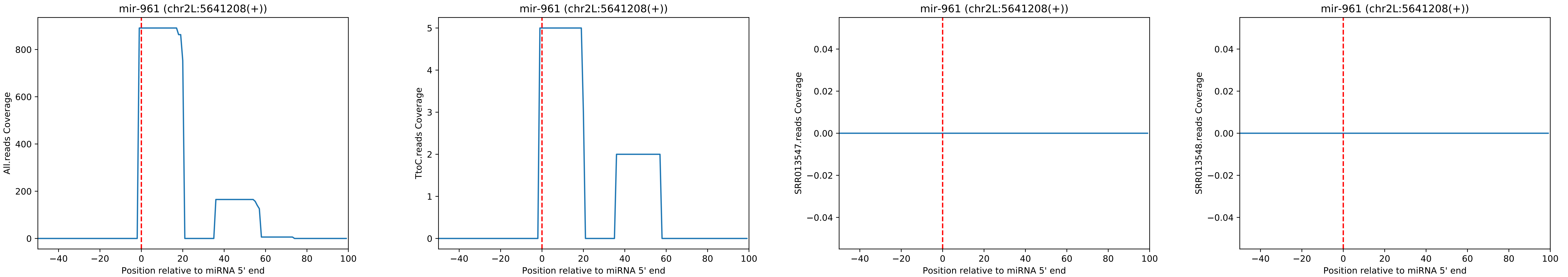
mir-961 (chr2L:5641208(+))
All.reads Coverage
Position relative to miRNA 5' end
mir-961 (chr2L:5641208(+))
TtoC.reads Coverage
Position relative to miRNA 5' end
mir-961 (chr2L:5641208(+))
SRR013547.reads Coverage
Position relative to miRNA 5' end
mir-961 (chr2L:5641208(+))
SRR013548.reads Coverage
Position relative to miRNA 5' end

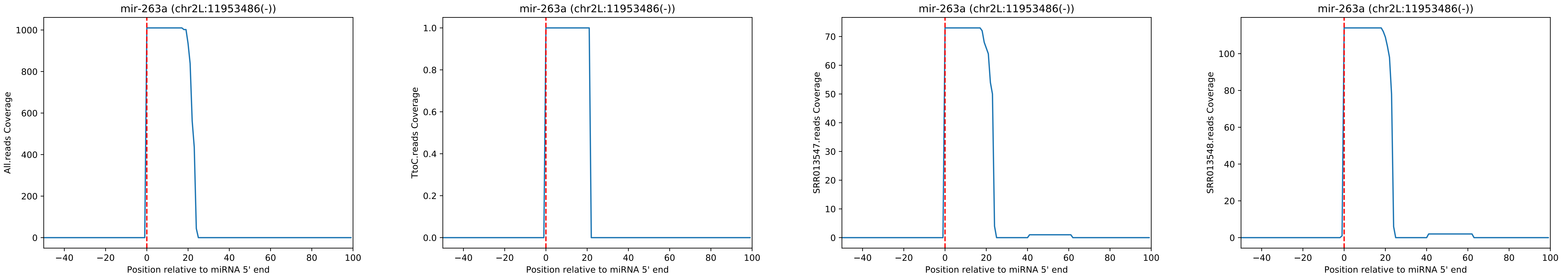

mir-263a (chr2L:11953486(-))
All.reads Coverage
Position relative to miRNA 5' end
mir-263a (chr2L:11953486(-))
TtoC.reads Coverage
Position relative to miRNA 5' end
mir-263a (chr2L:11953486(-))
SRR013547.reads Coverage
Position relative to miRNA 5' end
mir-263a (chr2L:11953486(-))
SRR013548.reads Coverage
Position relative to miRNA 5' end

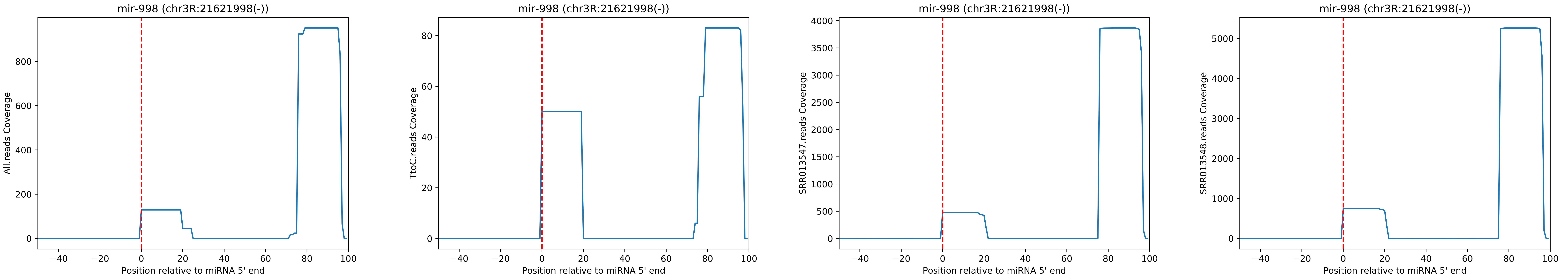

mir-998 (chr3R:21621998(-))
All.reads Coverage
TtoC.reads Coverage
SRR013547.reads Coverage
SRR013548.reads Coverage
Position relative to miRNA 5' end

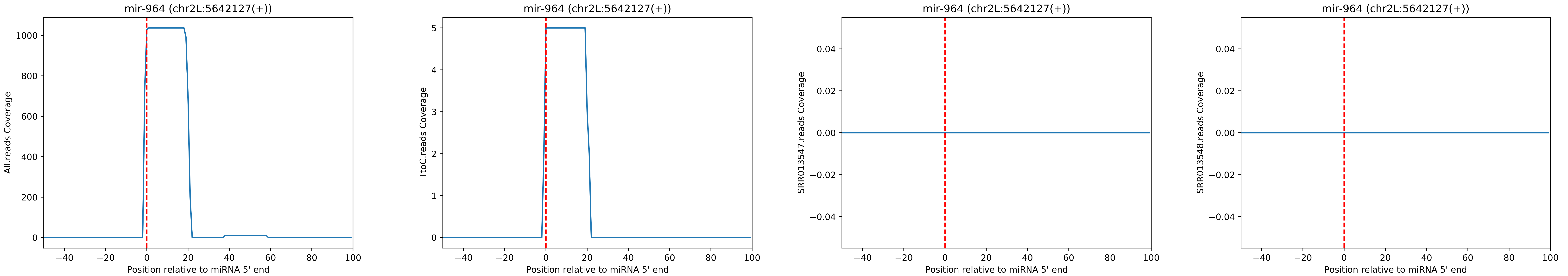
mir-964 (chr2L:5642127(+))
All.reads Coverage
TtoC.reads Coverage
SRR013547.reads Coverage
SRR013548.reads Coverage
Position relative to miRNA 5' end

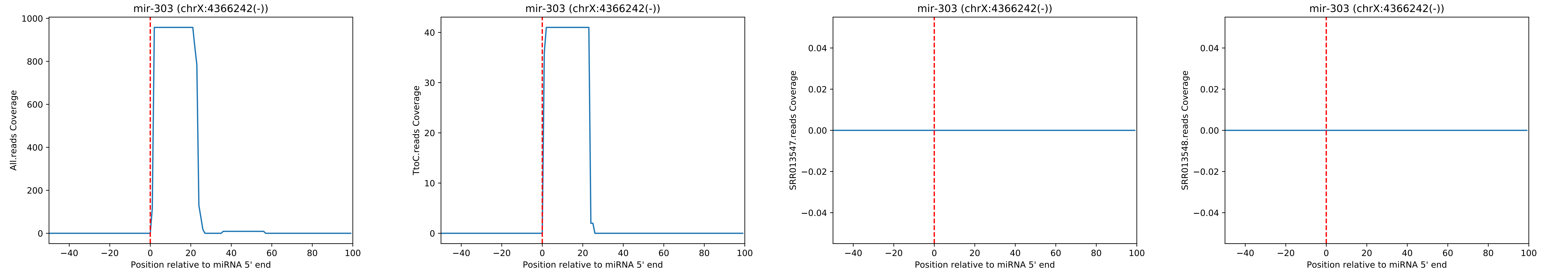

mir-303 (chrX:4366242(-))
All.reads Coverage
0
200
400
600
800
1000
Position relative to miRNA 5' end
−40
−20
0
20
40
60
80
100
mir-303 (chrX:4366242(-))
TtoC.reads Coverage
0
10
20
30
40
Position relative to miRNA 5' end
mir-303 (chrX:4366242(-))
SRR013547.reads Coverage
−0.04
−0.02
0.00
0.02
0.04
Position relative to miRNA 5' end
mir-303 (chrX:4366242(-))
SRR013548.reads Coverage
Position relative to miRNA 5' end

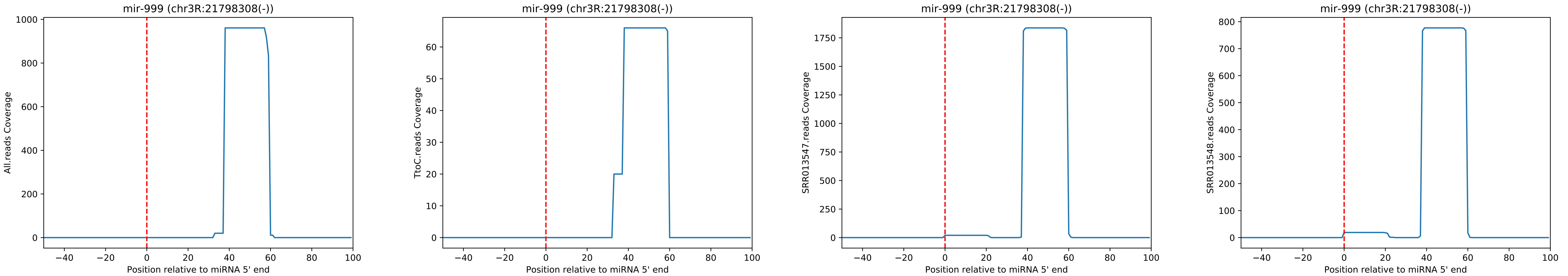
mir-999 (chr3R:21798308(-))
All.reads Coverage
Position relative to miRNA 5' end
mir-999 (chr3R:21798308(-))
TtoC.reads Coverage
Position relative to miRNA 5' end
mir-999 (chr3R:21798308(-))
SRR013547.reads Coverage
Position relative to miRNA 5' end
mir-999 (chr3R:21798308(-))
SRR013548.reads Coverage
Position relative to miRNA 5' end

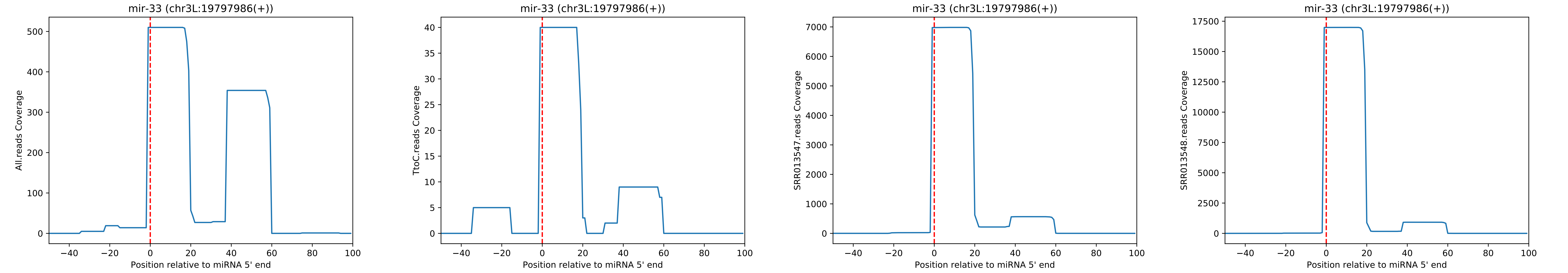
mir-33 (chr3L:19797986(+))
All.reads Coverage
Position relative to miRNA 5' end
mir-33 (chr3L:19797986(+))
TtoC.reads Coverage
Position relative to miRNA 5' end
mir-33 (chr3L:19797986(+))
SRR013547.reads Coverage
Position relative to miRNA 5' end
mir-33 (chr3L:19797986(+))
SRR013548.reads Coverage
Position relative to miRNA 5' end

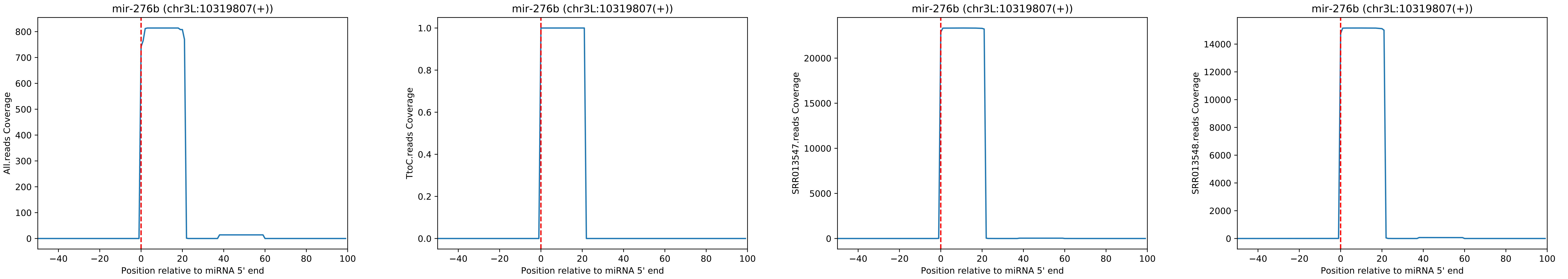
mir-276b (chr3L:10319807(+))
All.reads Coverage
0
100
200
300
400
500
600
700
800
Position relative to miRNA 5' end
−40
−20
0
20
40
60
80
100
mir-276b (chr3L:10319807(+))
TtoC.reads Coverage
0.0
0.2
0.4
0.6
0.8
1.0
Position relative to miRNA 5' end
−40
−20
0
20
40
60
80
100
mir-276b (chr3L:10319807(+))
SRR013547.reads Coverage
0
5000
10000
15000
20000
Position relative to miRNA 5' end
−40
−20
0
20
40
60
80
100
mir-276b (chr3L:10319807(+))
SRR013548.reads Coverage
0
2000
4000
6000
8000
10000
12000
14000
Position relative to miRNA 5' end
−40
−20
0
20
40
60
80
100

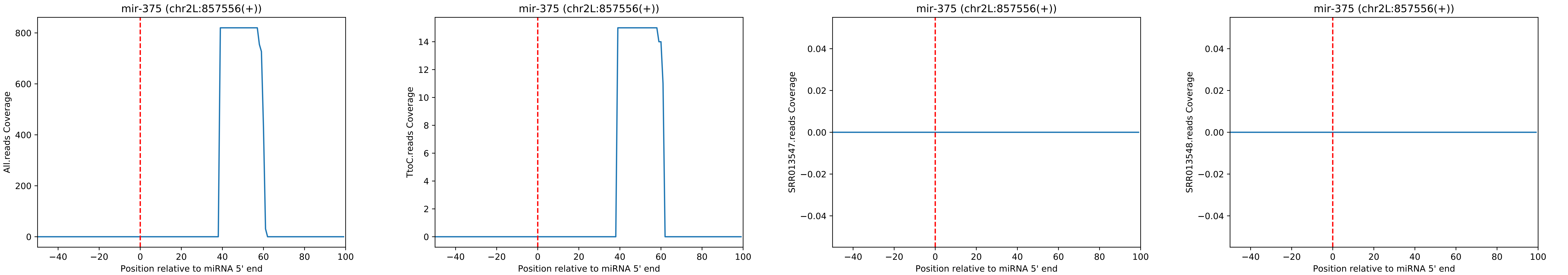

mir-375 (chr2L:857556(+))
All.reads Coverage
Position relative to miRNA 5' end
mir-375 (chr2L:857556(+))
TtoC.reads Coverage
Position relative to miRNA 5' end
mir-375 (chr2L:857556(+))
SRR013547.reads Coverage
Position relative to miRNA 5' end
mir-375 (chr2L:857556(+))
SRR013548.reads Coverage
Position relative to miRNA 5' end

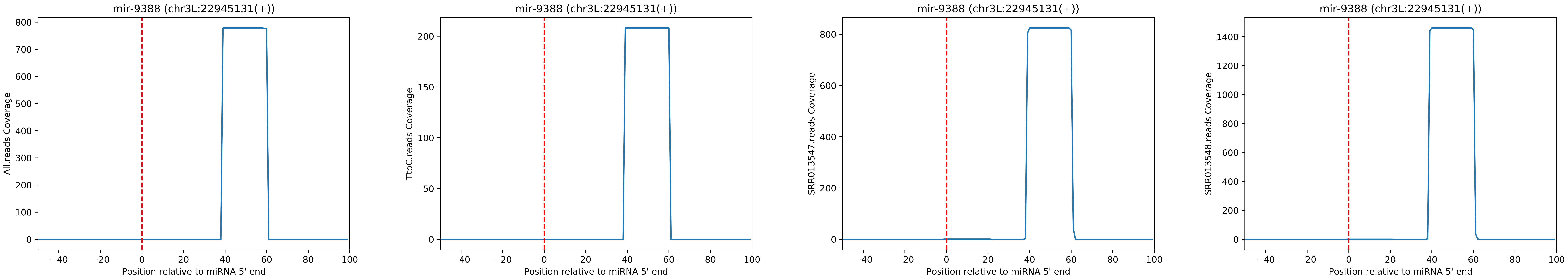
mir-9388 (chr3L:22945131(+))
All.reads Coverage
Position relative to miRNA 5' end
mir-9388 (chr3L:22945131(+))
TtoC.reads Coverage
Position relative to miRNA 5' end
mir-9388 (chr3L:22945131(+))
SRR013547.reads Coverage
Position relative to miRNA 5' end
mir-9388 (chr3L:22945131(+))
SRR013548.reads Coverage
Position relative to miRNA 5' end

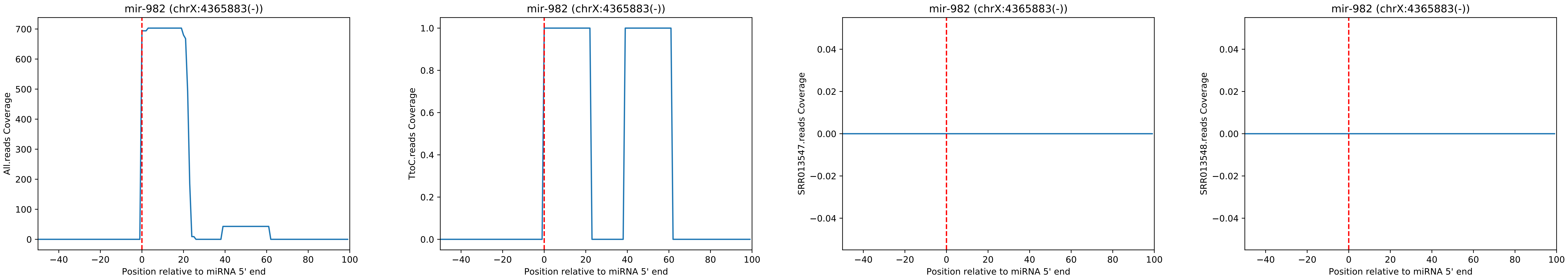
mir-982 (chrX:4365883(-))
All.reads Coverage
Position relative to miRNA 5' end
mir-982 (chrX:4365883(-))
TtoC.reads Coverage
Position relative to miRNA 5' end
mir-982 (chrX:4365883(-))
SRR013547.reads Coverage
Position relative to miRNA 5' end
mir-982 (chrX:4365883(-))
SRR013548.reads Coverage
Position relative to miRNA 5' end

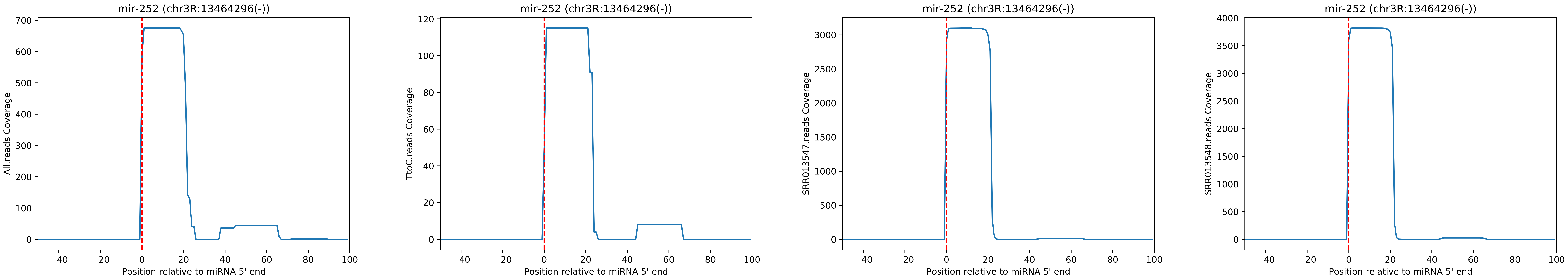
mir-252 (chr3R:13464296(-))
All.reads Coverage
Position relative to miRNA 5' end
mir-252 (chr3R:13464296(-))
TtoC.reads Coverage
Position relative to miRNA 5' end
mir-252 (chr3R:13464296(-))
SRR013547.reads Coverage
Position relative to miRNA 5' end
mir-252 (chr3R:13464296(-))
SRR013548.reads Coverage
Position relative to miRNA 5' end

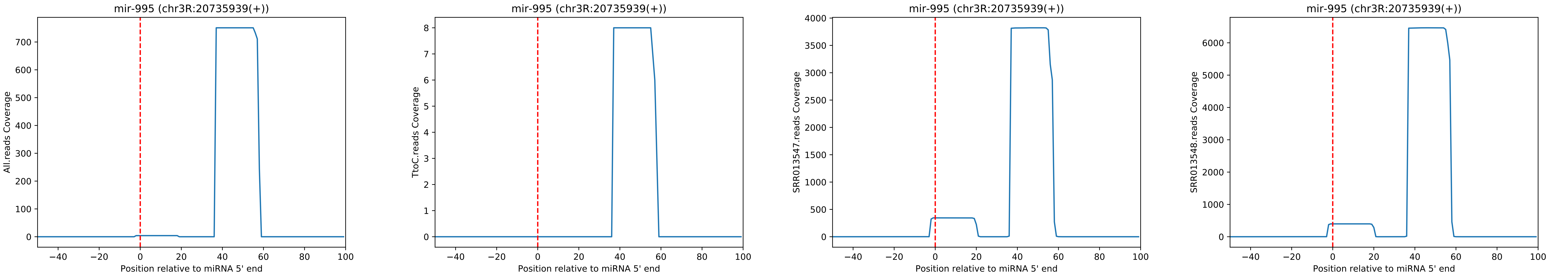

mir-995 (chr3R:20735939(+))
All.reads Coverage
Position relative to miRNA 5' end
mir-995 (chr3R:20735939(+))
TtoC.reads Coverage
Position relative to miRNA 5' end
mir-995 (chr3R:20735939(+))
SRR013547.reads Coverage
Position relative to miRNA 5' end
mir-995 (chr3R:20735939(+))
SRR013548.reads Coverage
Position relative to miRNA 5' end

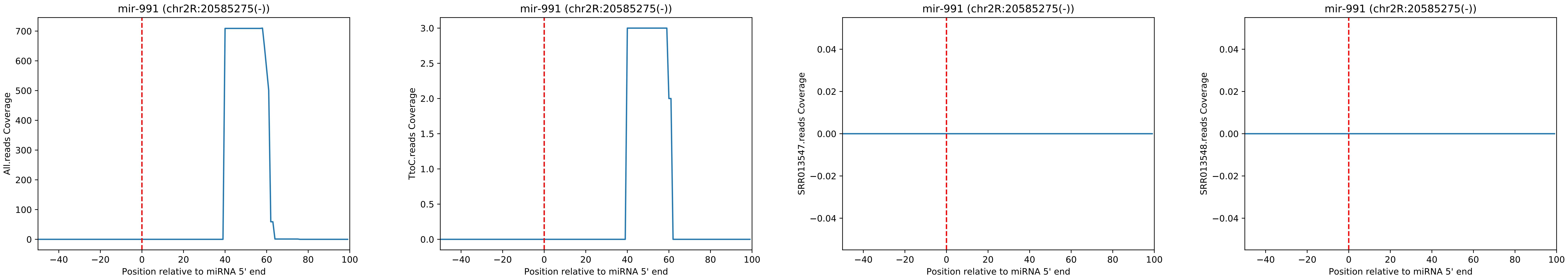
mir-991 (chr2R:20585275(-))
All.reads Coverage
Position relative to miRNA 5' end
mir-991 (chr2R:20585275(-))
TtoC.reads Coverage
Position relative to miRNA 5' end
mir-991 (chr2R:20585275(-))
SRR013547.reads Coverage
Position relative to miRNA 5' end
mir-991 (chr2R:20585275(-))
SRR013548.reads Coverage
Position relative to miRNA 5' end

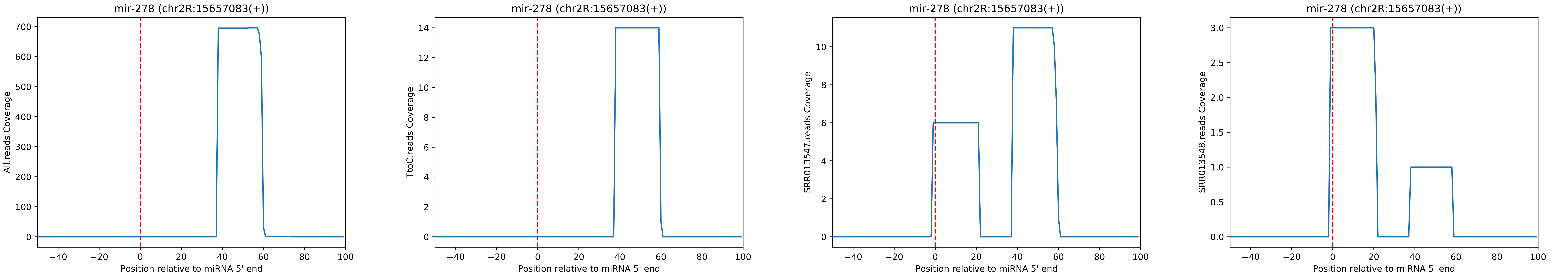
mir-278 (chr2R:15657083(+))
All.reads Coverage
Position relative to miRNA 5' end
mir-278 (chr2R:15657083(+))
TtoC.reads Coverage
Position relative to miRNA 5' end
mir-278 (chr2R:15657083(+))
SRR013547.reads Coverage
Position relative to miRNA 5' end
mir-278 (chr2R:15657083(+))
SRR013548.reads Coverage
Position relative to miRNA 5' end

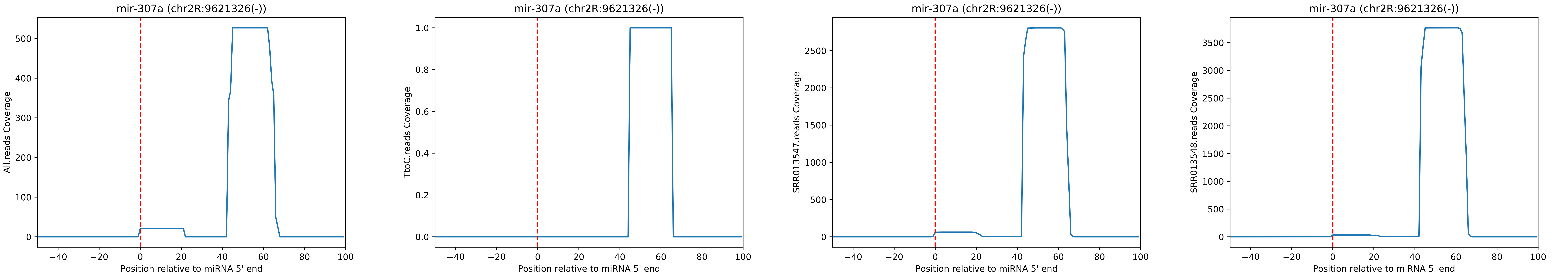

mir-307a (chr2R:9621326(-))
All.reads Coverage
Position relative to miRNA 5' end
mir-307a (chr2R:9621326(-))
TtoC.reads Coverage
Position relative to miRNA 5' end
mir-307a (chr2R:9621326(-))
SRR013547.reads Coverage
Position relative to miRNA 5' end
mir-307a (chr2R:9621326(-))
SRR013548.reads Coverage
Position relative to miRNA 5' end

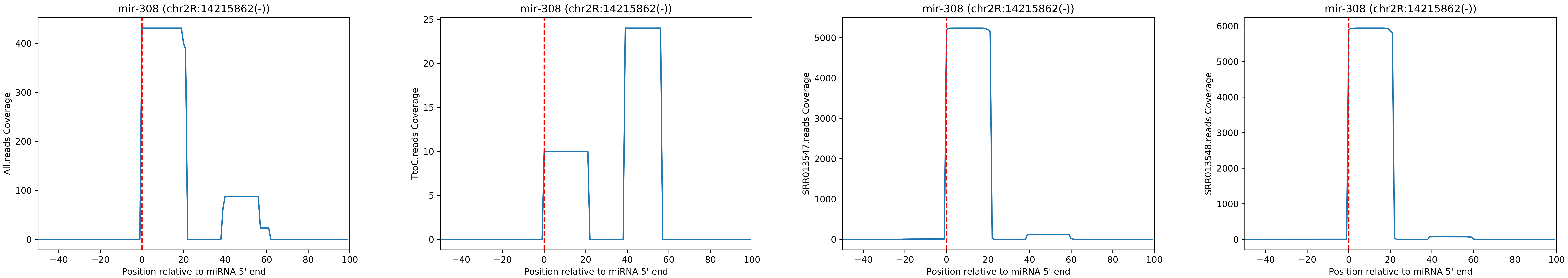
mir-308 (chr2R:14215862(-))
All.reads Coverage
Position relative to miRNA 5' end
mir-308 (chr2R:14215862(-))
TtoC.reads Coverage
Position relative to miRNA 5' end
mir-308 (chr2R:14215862(-))
SRR013547.reads Coverage
Position relative to miRNA 5' end
mir-308 (chr2R:14215862(-))
SRR013548.reads Coverage
Position relative to miRNA 5' end

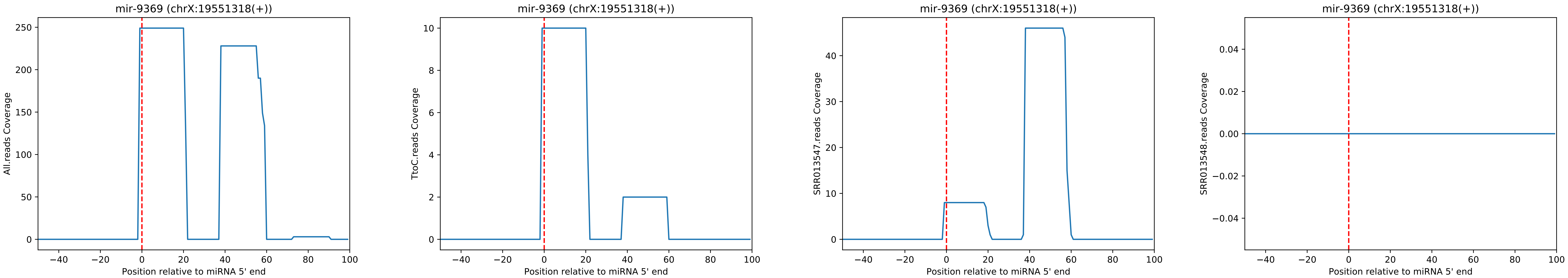

mir-9369 (chrX:19551318(+))
All.reads Coverage
Position relative to miRNA 5' end
mir-9369 (chrX:19551318(+))
TtoC.reads Coverage
Position relative to miRNA 5' end
mir-9369 (chrX:19551318(+))
SRR013547.reads Coverage
Position relative to miRNA 5' end
mir-9369 (chrX:19551318(+))
SRR013548.reads Coverage
Position relative to miRNA 5' end

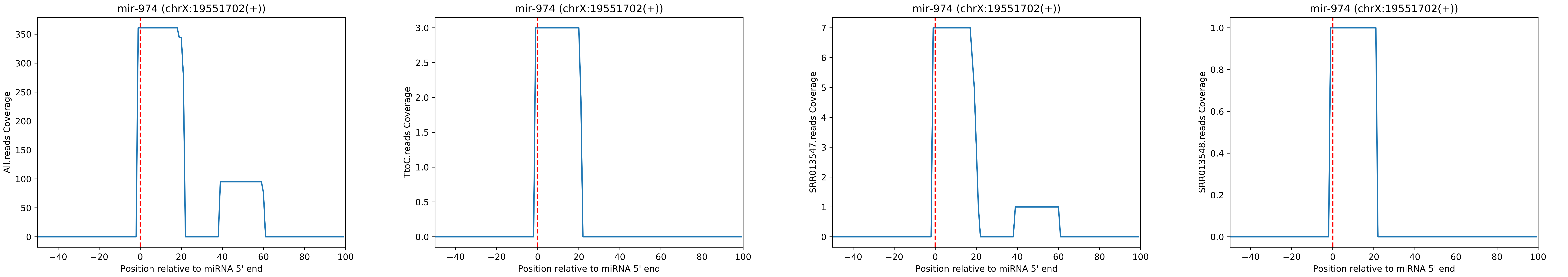
mir-974 (chrX:19551702(+))
All.reads Coverage
Position relative to miRNA 5' end
mir-974 (chrX:19551702(+))
TtoC.reads Coverage
Position relative to miRNA 5' end
mir-974 (chrX:19551702(+))
SRR013547.reads Coverage
Position relative to miRNA 5' end
mir-974 (chrX:19551702(+))
SRR013548.reads Coverage
Position relative to miRNA 5' end

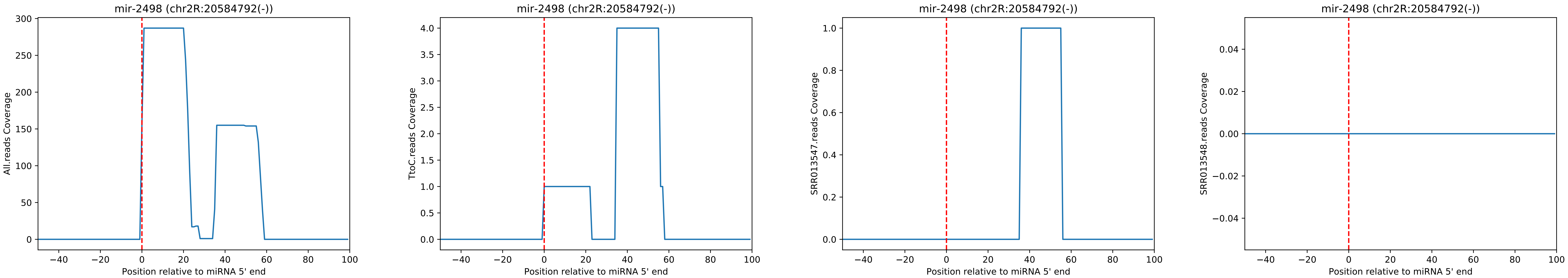
mir-2498 (chr2R:20584792(-))
All.reads Coverage
TtoC.reads Coverage
SRR013547.reads Coverage
SRR013548.reads Coverage
Position relative to miRNA 5' end

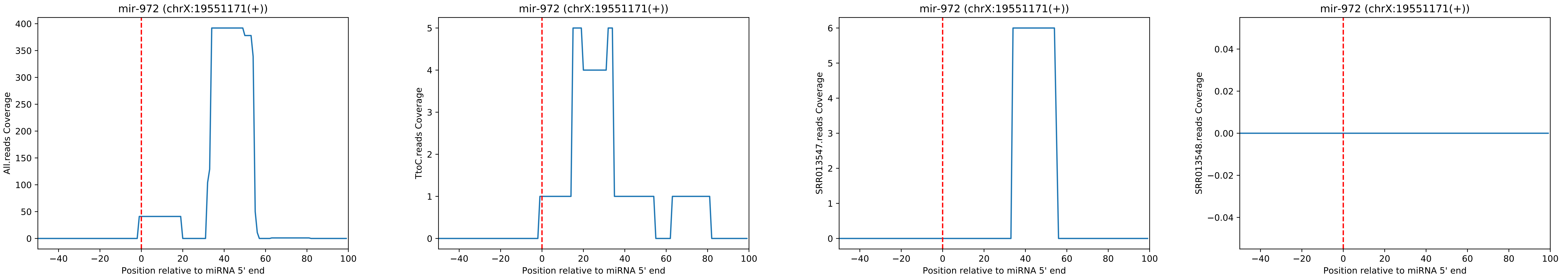
mir-972 (chrX:19551171(+))
All.reads Coverage
Position relative to miRNA 5' end
mir-972 (chrX:19551171(+))
TtoC.reads Coverage
Position relative to miRNA 5' end
mir-972 (chrX:19551171(+))
SRR013547.reads Coverage
Position relative to miRNA 5' end
mir-972 (chrX:19551171(+))
SRR013548.reads Coverage
Position relative to miRNA 5' end

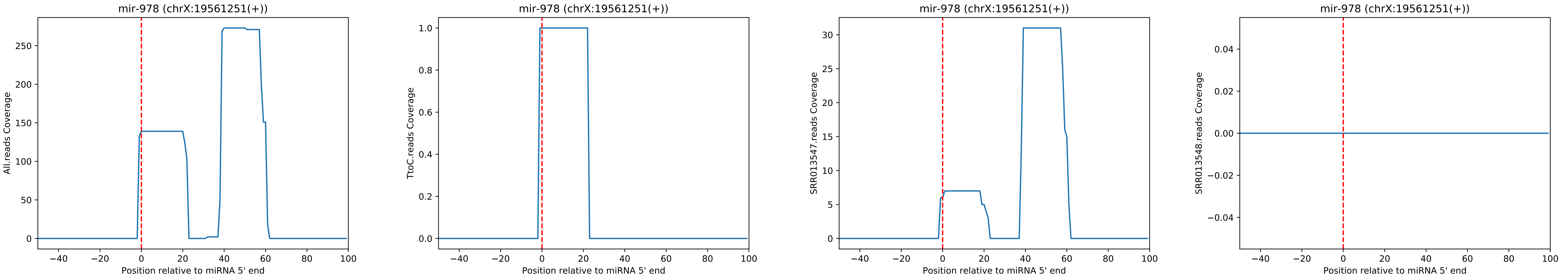

mir-978 (chrX:19561251(+))
All.reads Coverage
Position relative to miRNA 5' end
mir-978 (chrX:19561251(+))
TtoC.reads Coverage
Position relative to miRNA 5' end
mir-978 (chrX:19561251(+))
SRR013547.reads Coverage
Position relative to miRNA 5' end
mir-978 (chrX:19561251(+))
SRR013548.reads Coverage
Position relative to miRNA 5' end

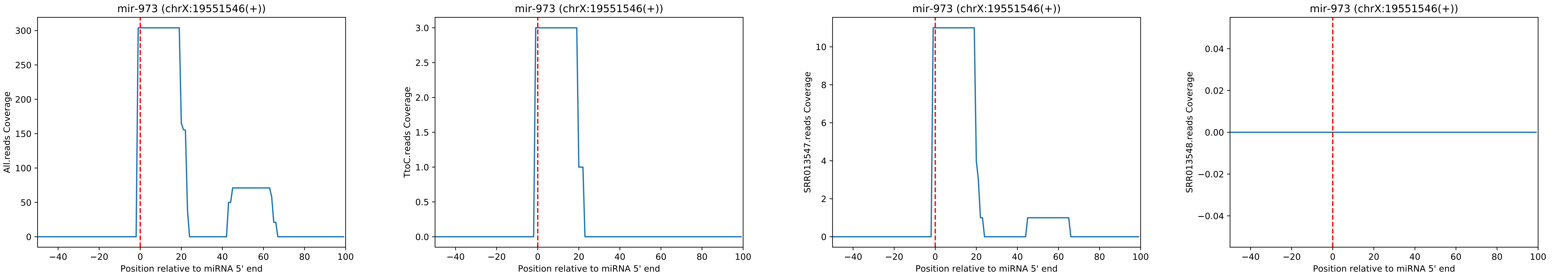
mir-973 (chrX:19551546(+))
All.reads Coverage
Position relative to miRNA 5' end
mir-973 (chrX:19551546(+))
TtoC.reads Coverage
Position relative to miRNA 5' end
mir-973 (chrX:19551546(+))
SRR013547.reads Coverage
Position relative to miRNA 5' end
mir-973 (chrX:19551546(+))
SRR013548.reads Coverage
Position relative to miRNA 5' end

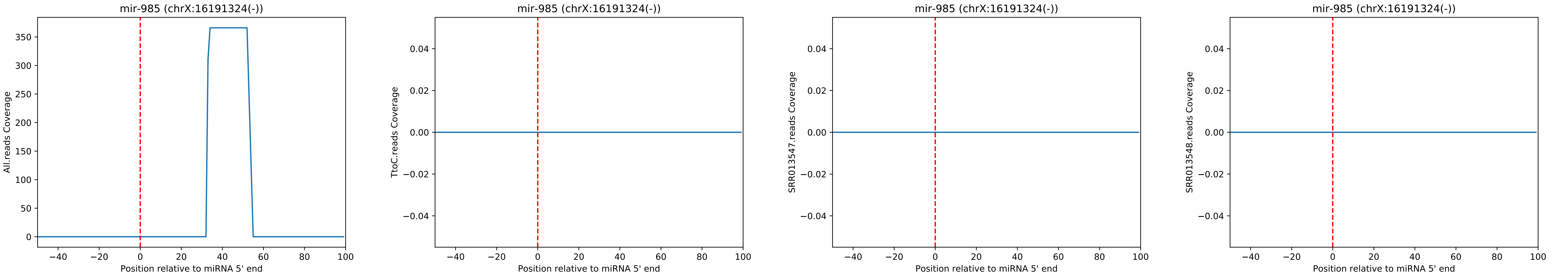

mir-985 (chrX:16191324(-))
All.reads Coverage
TtoC.reads Coverage
SRR013547.reads Coverage
SRR013548.reads Coverage
Position relative to miRNA 5' end

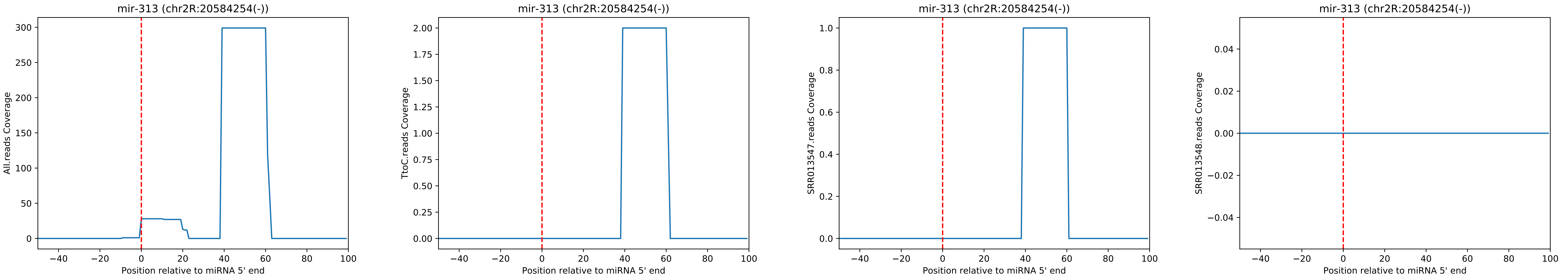

mir-313 (chr2R:20584254(-))
All.reads Coverage
TtoC.reads Coverage
SRR013547.reads Coverage
SRR013548.reads Coverage
Position relative to miRNA 5' end

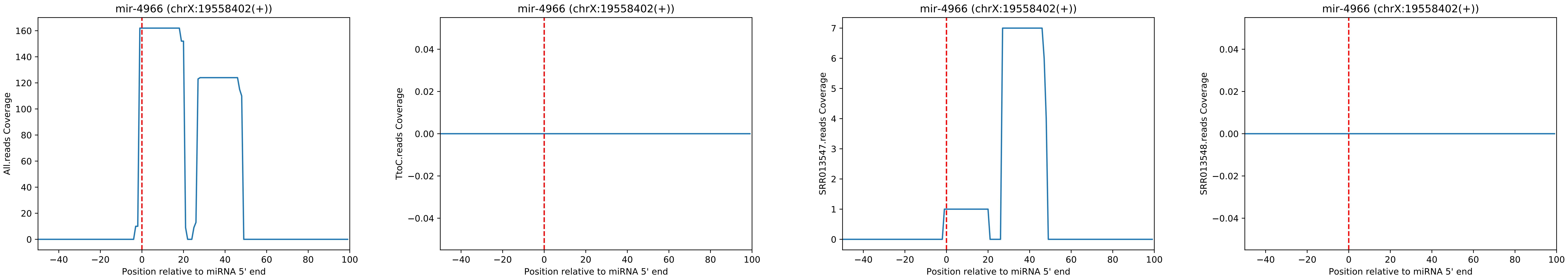
mir-4966 (chrX:19558402(+))
All.reads Coverage
Position relative to miRNA 5' end
mir-4966 (chrX:19558402(+))
TtoC.reads Coverage
Position relative to miRNA 5' end
mir-4966 (chrX:19558402(+))
SRR013547.reads Coverage
Position relative to miRNA 5' end
mir-4966 (chrX:19558402(+))
SRR013548.reads Coverage
Position relative to miRNA 5' end

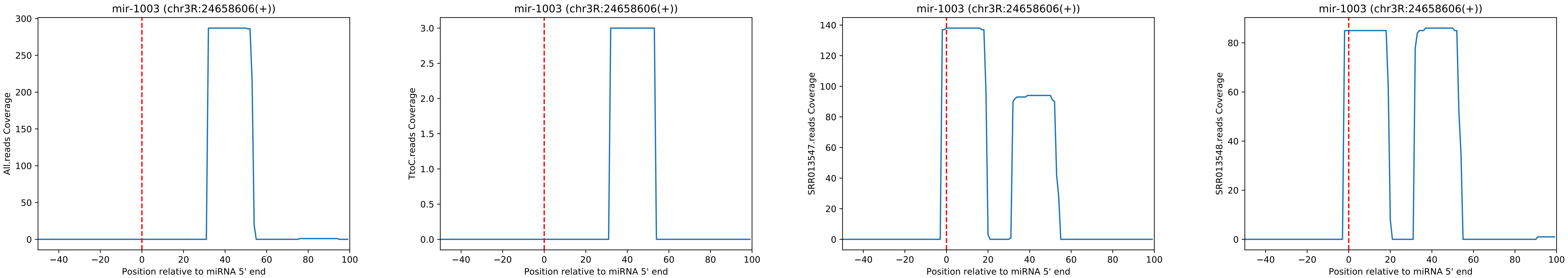
mir-1003 (chr3R:24658606(+))
All.reads Coverage
TtoC.reads Coverage
SRR013547.reads Coverage
SRR013548.reads Coverage
Position relative to miRNA 5' end

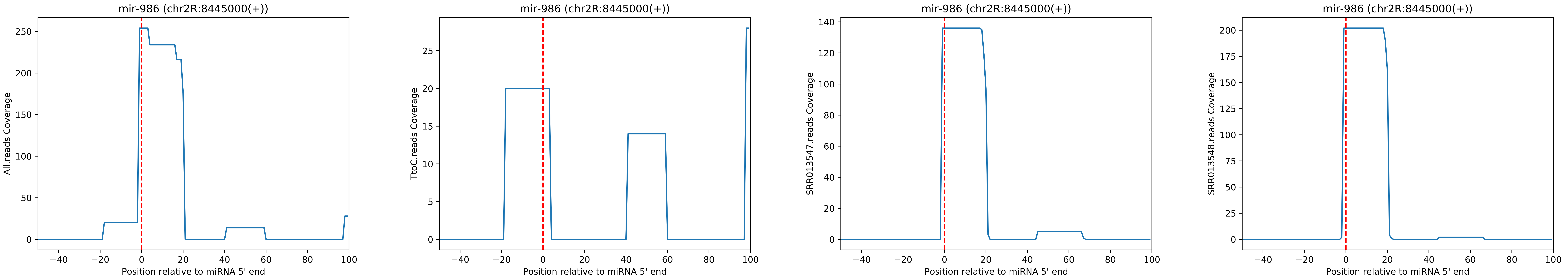
mir-986 (chr2R:8445000(+))
All.reads Coverage
Position relative to miRNA 5' end
mir-986 (chr2R:8445000(+))
TtoC.reads Coverage
Position relative to miRNA 5' end
mir-986 (chr2R:8445000(+))
SRR013547.reads Coverage
Position relative to miRNA 5' end
mir-986 (chr2R:8445000(+))
SRR013548.reads Coverage
Position relative to miRNA 5' end

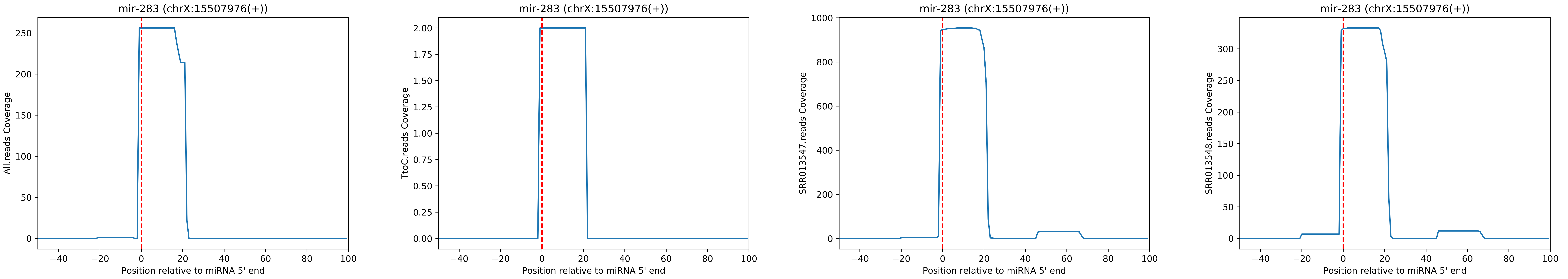

mir-283 (chrX:15507976(+))
All.reads Coverage
Position relative to miRNA 5' end
mir-283 (chrX:15507976(+))
TtoC.reads Coverage
Position relative to miRNA 5' end
mir-283 (chrX:15507976(+))
SRR013547.reads Coverage
Position relative to miRNA 5' end
mir-283 (chrX:15507976(+))
SRR013548.reads Coverage
Position relative to miRNA 5' end

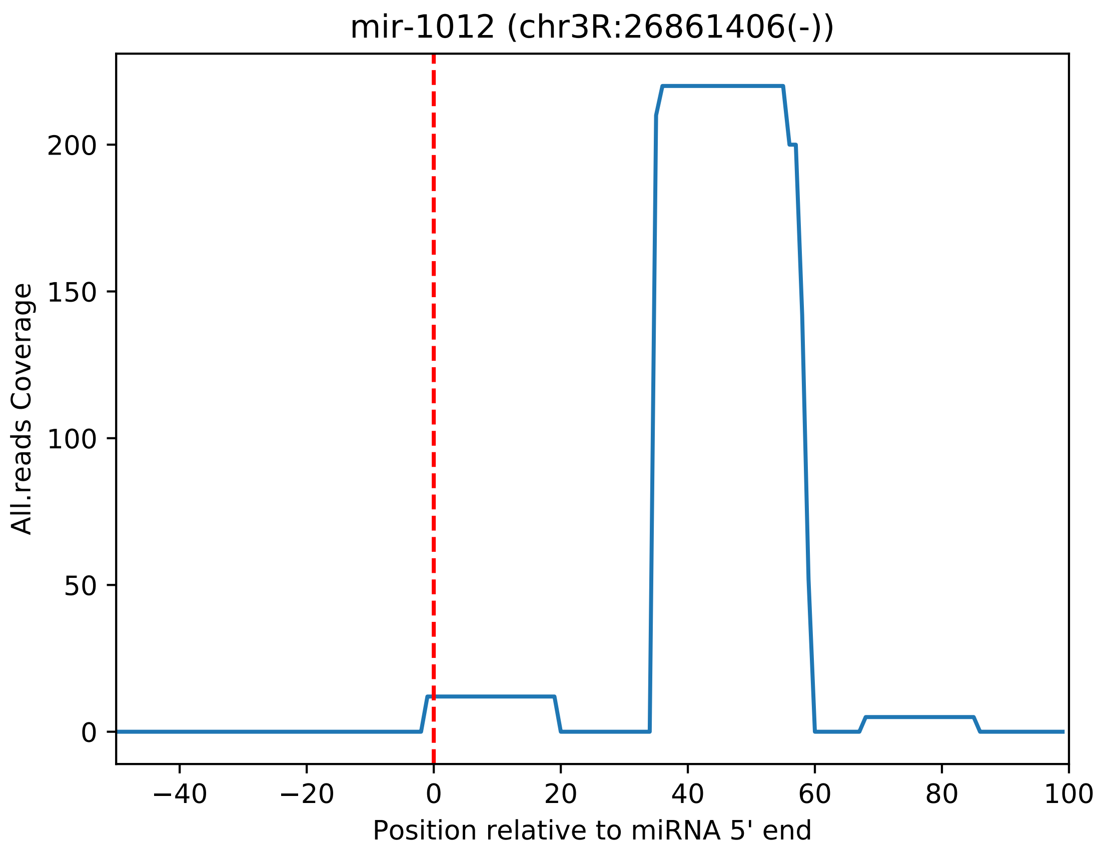
mir-1012 (chr3R:26861406(-))
All.reads Coverage
0
50
100
150
200
−40
−20
0
20
40
60
80
100
Position relative to miRNA 5' end
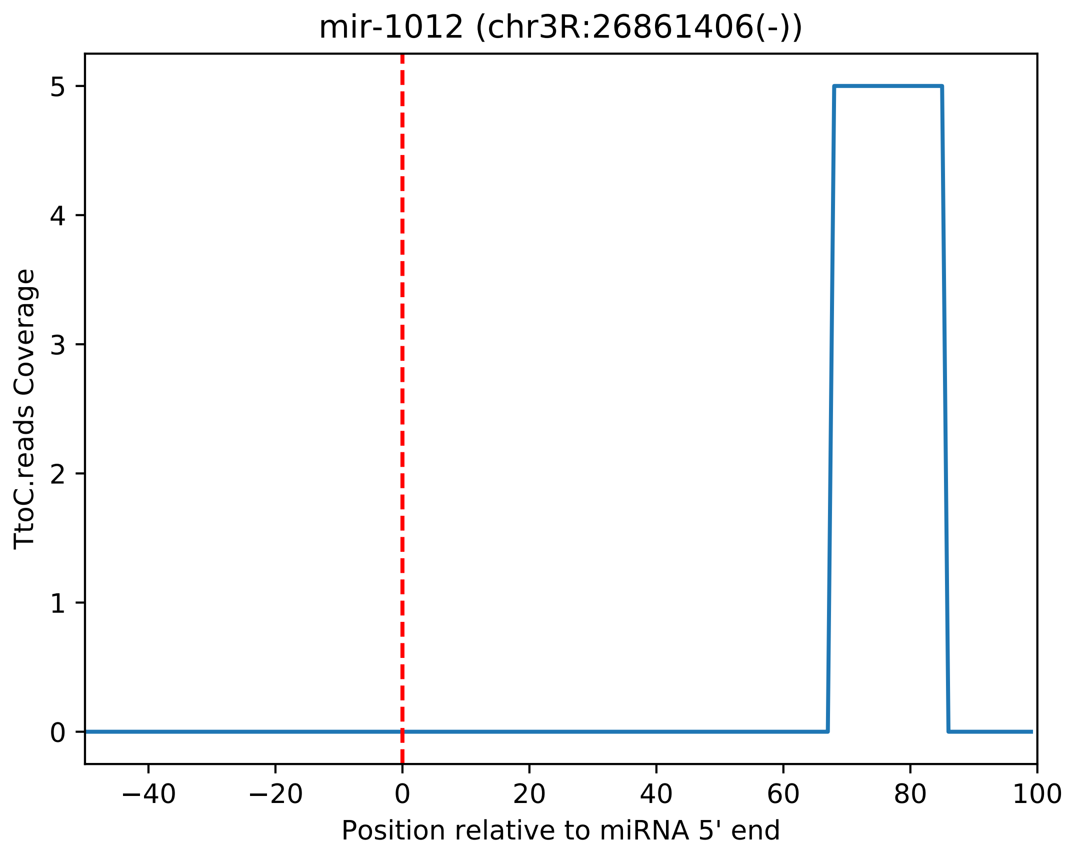
mir-1012 (chr3R:26861406(-))
TtoC.reads Coverage
0
1
2
3
4
5
−40
−20
0
20
40
60
80
100
Position relative to miRNA 5' end
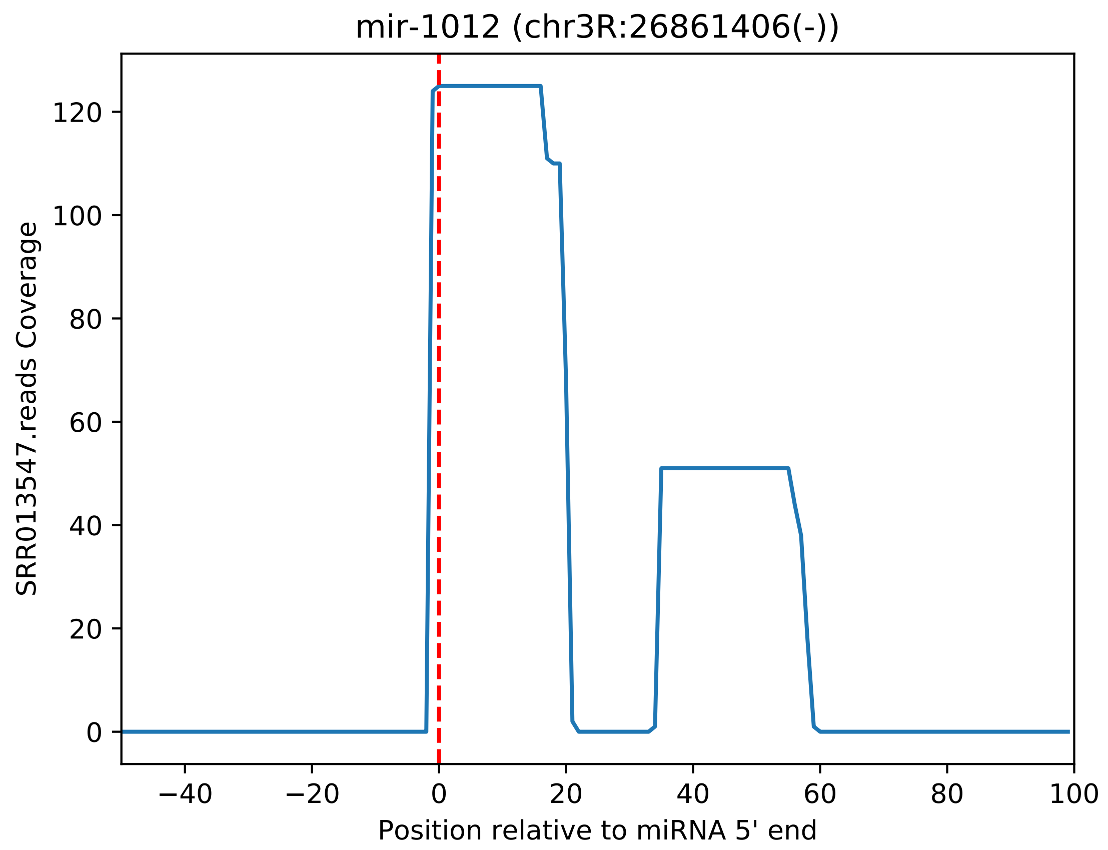
mir-1012 (chr3R:26861406(-))
SRR013547.reads Coverage
0
20
40
60
80
100
120
−40
−20
0
20
40
60
80
100
Position relative to miRNA 5' end
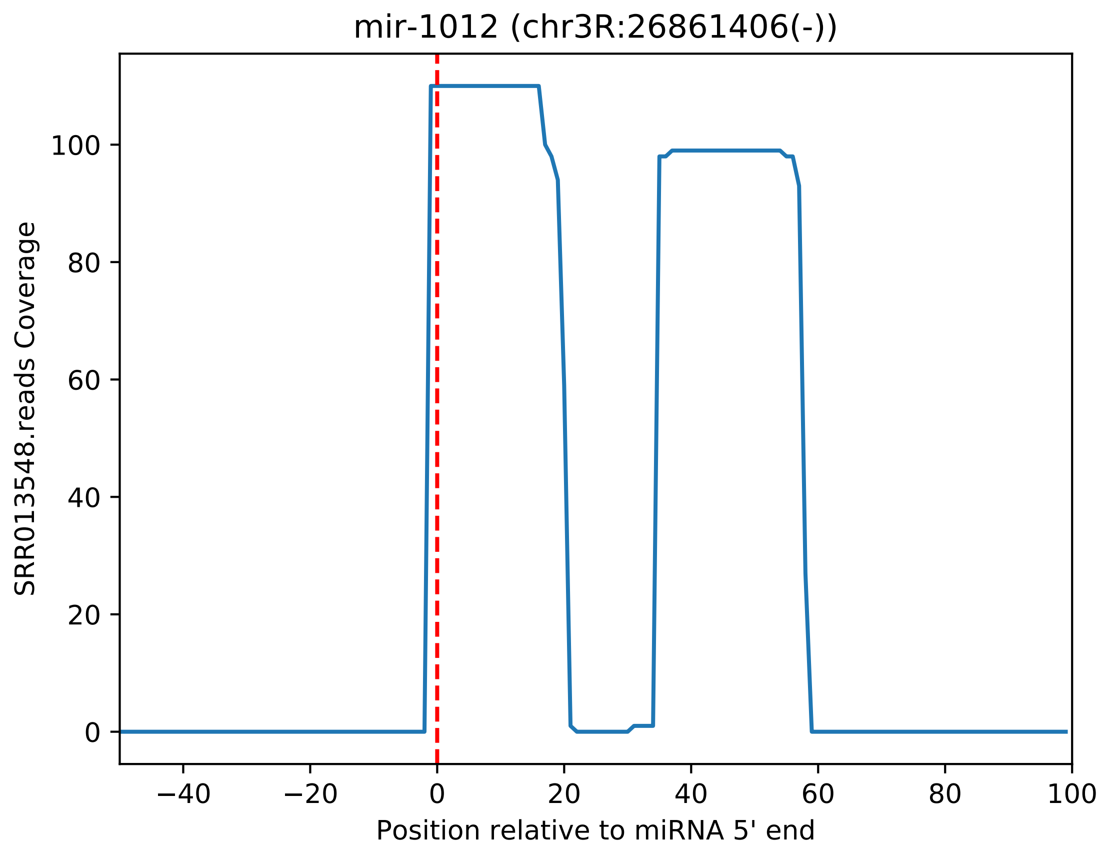
mir-1012 (chr3R:26861406(-))
SRR013548.reads Coverage
0
20
40
60
80
100
−40
−20
0
20
40
60
80
100
Position relative to miRNA 5' end

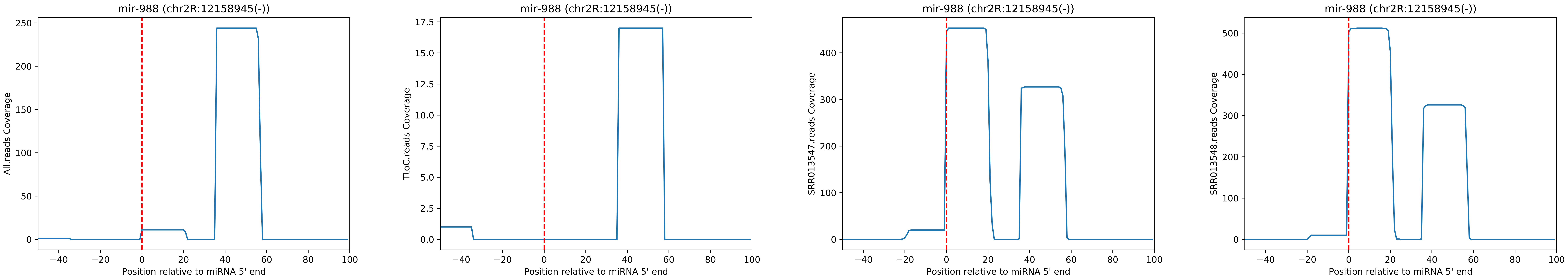
mir-988 (chr2R:12158945(-))
All.reads Coverage
Position relative to miRNA 5' end
0
50
100
150
200
250
-40
-20
0
20
40
60
80
100
mir-988 (chr2R:12158945(-))
TtoC.reads Coverage
Position relative to miRNA 5' end
0.0
2.5
5.0
7.5
10.0
12.5
15.0
17.5
-40
-20
0
20
40
60
80
100
mir-988 (chr2R:12158945(-))
SRR013547.reads Coverage
Position relative to miRNA 5' end
0
100
200
300
400
-40
-20
0
20
40
60
80
100
mir-988 (chr2R:12158945(-))
SRR013548.reads Coverage
Position relative to miRNA 5' end
0
100
200
300
400
500
-40
-20
0
20
40
60
80
100

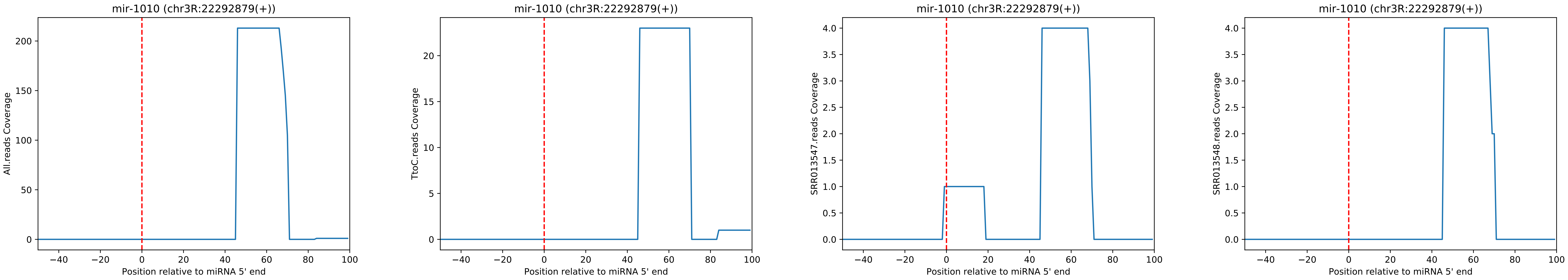
mir-1010 (chr3R:22292879(+))
All.reads Coverage
Position relative to miRNA 5' end
mir-1010 (chr3R:22292879(+))
TtoC.reads Coverage
Position relative to miRNA 5' end
mir-1010 (chr3R:22292879(+))
SRR013547.reads Coverage
Position relative to miRNA 5' end
mir-1010 (chr3R:22292879(+))
SRR013548.reads Coverage
Position relative to miRNA 5' end

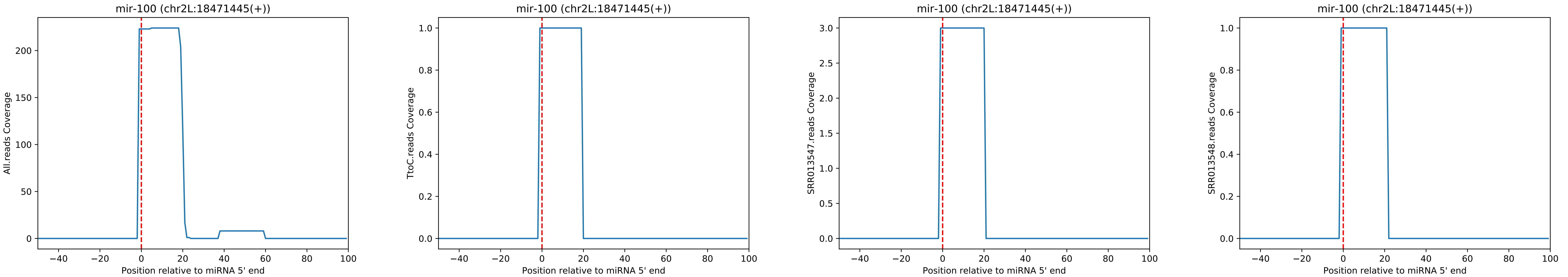
mir-100 (chr2L:18471445(+))
All.reads Coverage
Position relative to miRNA 5' end
mir-100 (chr2L:18471445(+))
TtoC.reads Coverage
Position relative to miRNA 5' end
mir-100 (chr2L:18471445(+))
SRR013547.reads Coverage
Position relative to miRNA 5' end
mir-100 (chr2L:18471445(+))
SRR013548.reads Coverage
Position relative to miRNA 5' end

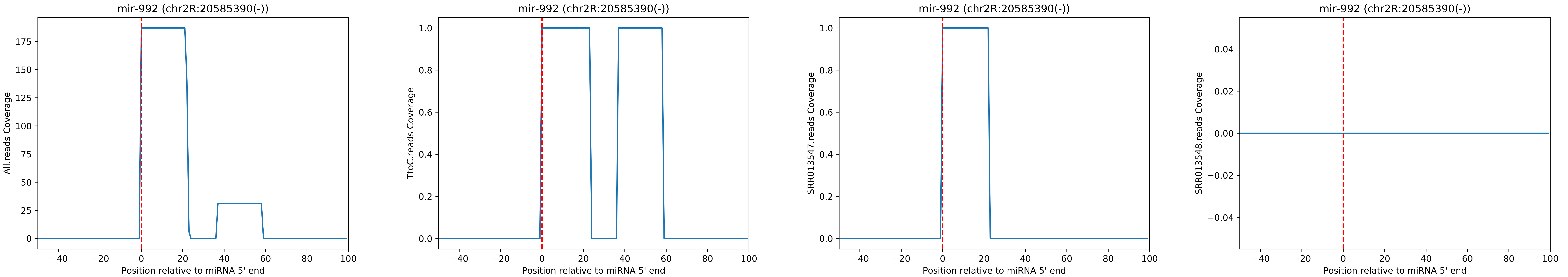

mir-992 (chr2R:20585390(-))
All.reads Coverage
TtoC.reads Coverage
SRR013547.reads Coverage
SRR013548.reads Coverage
Position relative to miRNA 5' end

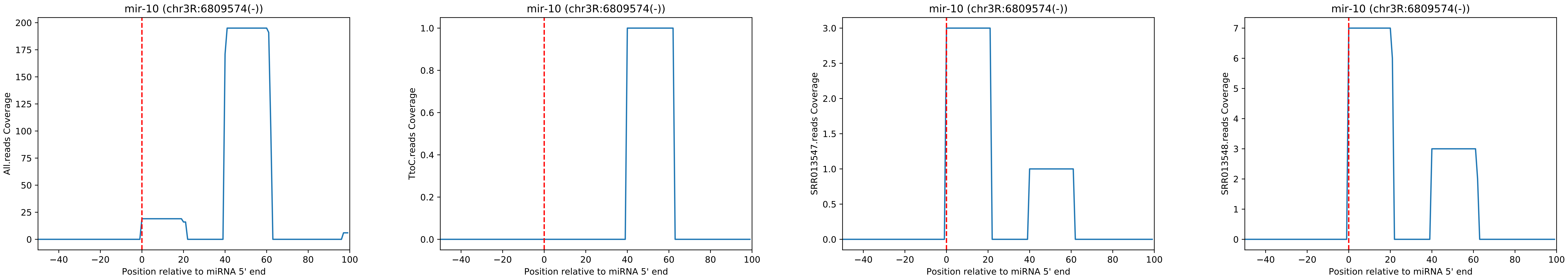
mir-10 (chr3R:6809574(-))
All.reads Coverage
Position relative to miRNA 5' end
mir-10 (chr3R:6809574(-))
TtoC.reads Coverage
Position relative to miRNA 5' end
mir-10 (chr3R:6809574(-))
SRR013547.reads Coverage
Position relative to miRNA 5' end
mir-10 (chr3R:6809574(-))
SRR013548.reads Coverage
Position relative to miRNA 5' end

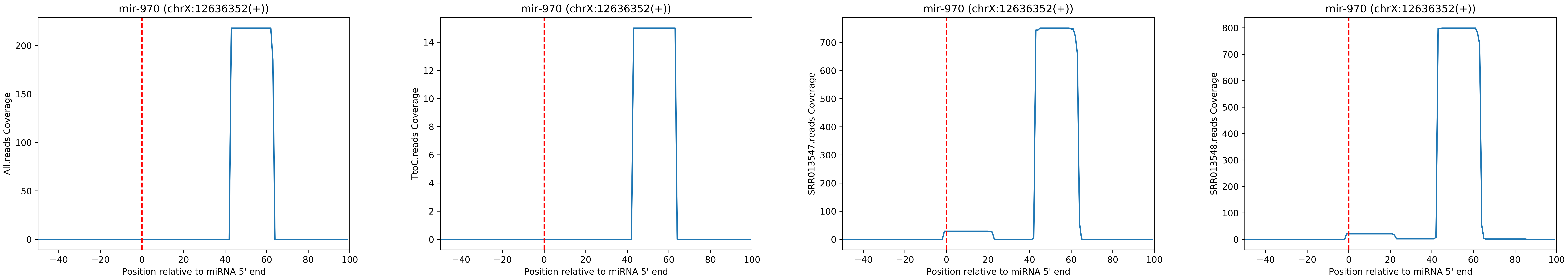
mir-970 (chrX:12636352(+))
All.reads Coverage
Position relative to miRNA 5' end
mir-970 (chrX:12636352(+))
TtoC.reads Coverage
Position relative to miRNA 5' end
mir-970 (chrX:12636352(+))
SRR013547.reads Coverage
Position relative to miRNA 5' end
mir-970 (chrX:12636352(+))
SRR013548.reads Coverage
Position relative to miRNA 5' end

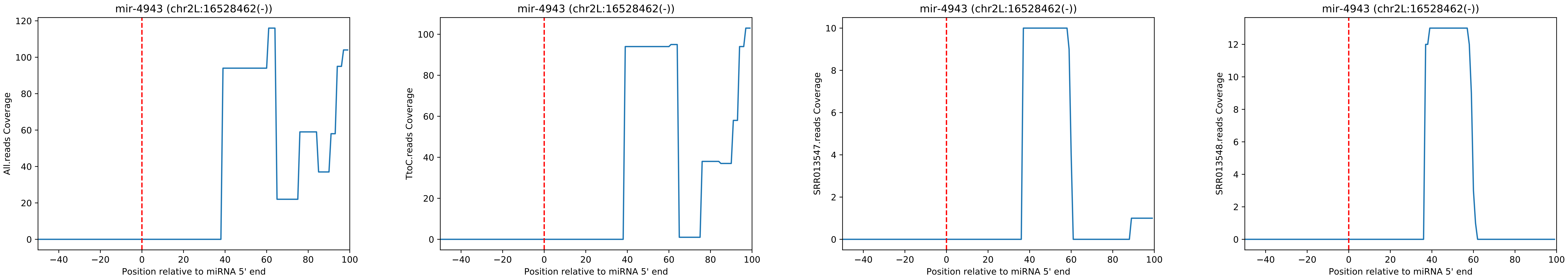
mir-4943 (chr2L:16528462(-))
All.reads Coverage
Position relative to miRNA 5' end
mir-4943 (chr2L:16528462(-))
TtoC.reads Coverage
Position relative to miRNA 5' end
mir-4943 (chr2L:16528462(-))
SRR013547.reads Coverage
Position relative to miRNA 5' end
mir-4943 (chr2L:16528462(-))
SRR013548.reads Coverage
Position relative to miRNA 5' end

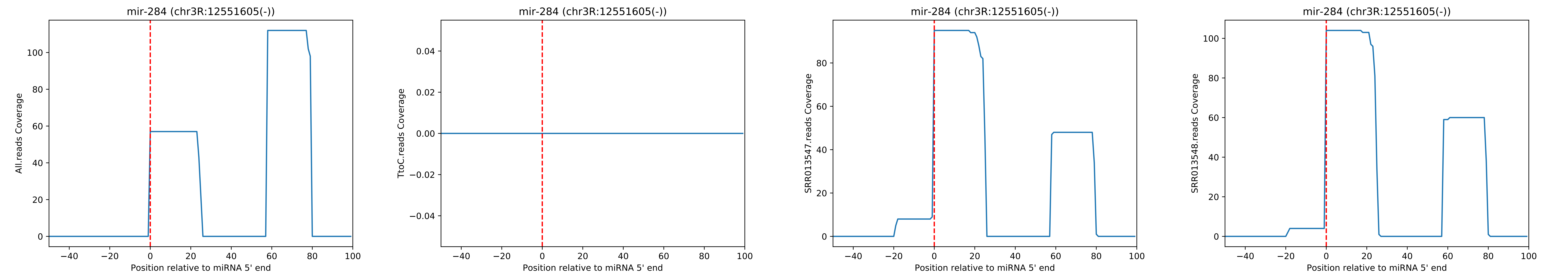
mir-284 (chr3R:12551605(-))
All.reads Coverage
Position relative to miRNA 5' end
mir-284 (chr3R:12551605(-))
TtoC.reads Coverage
Position relative to miRNA 5' end
mir-284 (chr3R:12551605(-))
SRR013547.reads Coverage
Position relative to miRNA 5' end
mir-284 (chr3R:12551605(-))
SRR013548.reads Coverage
Position relative to miRNA 5' end

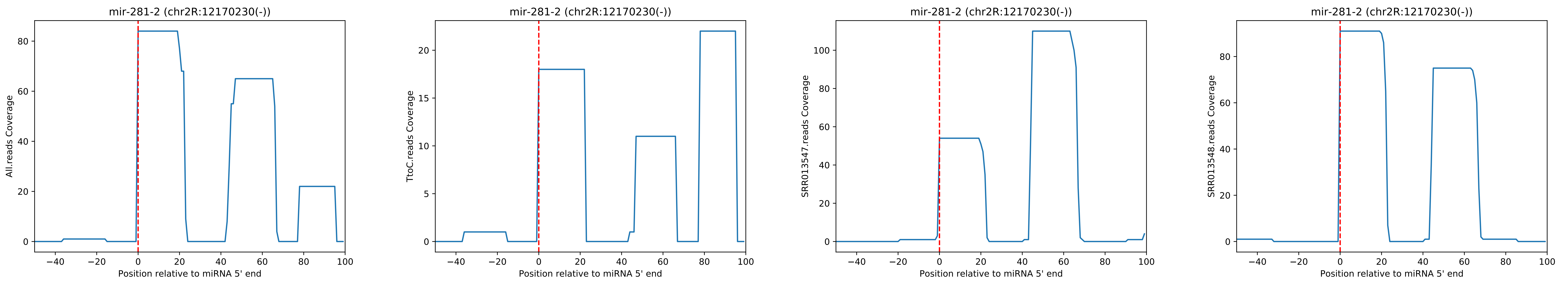

mir-281-2 (chr2R:12170230(-))
All.reads Coverage
Position relative to miRNA 5' end
mir-281-2 (chr2R:12170230(-))
TtoC.reads Coverage
Position relative to miRNA 5' end
mir-281-2 (chr2R:12170230(-))
SRR013547.reads Coverage
Position relative to miRNA 5' end
mir-281-2 (chr2R:12170230(-))
SRR013548.reads Coverage
Position relative to miRNA 5' end

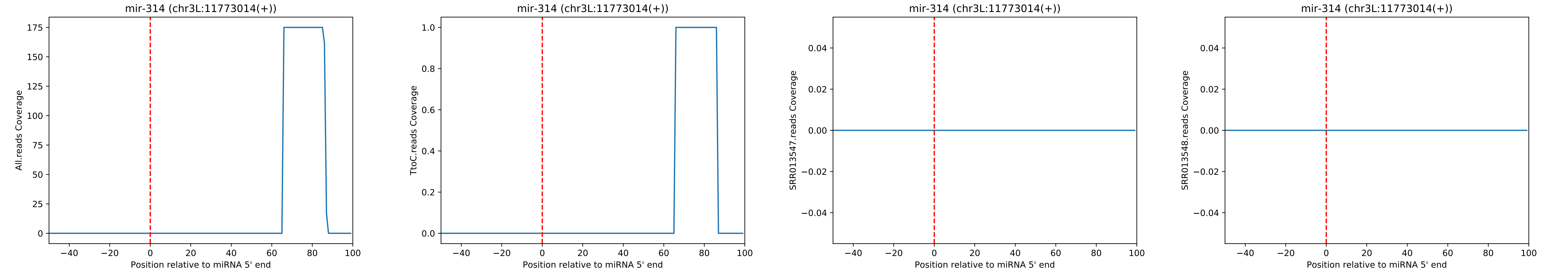
mir-314 (chr3L:11773014(+))
All.reads Coverage
Position relative to miRNA 5' end
mir-314 (chr3L:11773014(+))
TtoC.reads Coverage
Position relative to miRNA 5' end
mir-314 (chr3L:11773014(+))
SRR013547.reads Coverage
Position relative to miRNA 5' end
mir-314 (chr3L:11773014(+))
SRR013548.reads Coverage
Position relative to miRNA 5' end

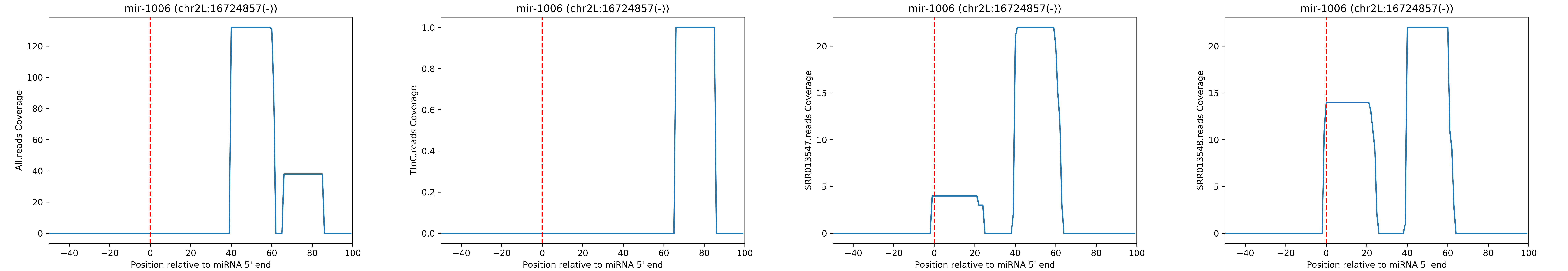
mir-1006 (chr2L:16724857(-))
All.reads Coverage
Position relative to miRNA 5' end
mir-1006 (chr2L:16724857(-))
TtoC.reads Coverage
Position relative to miRNA 5' end
mir-1006 (chr2L:16724857(-))
SRR013547.reads Coverage
Position relative to miRNA 5' end
mir-1006 (chr2L:16724857(-))
SRR013548.reads Coverage
Position relative to miRNA 5' end

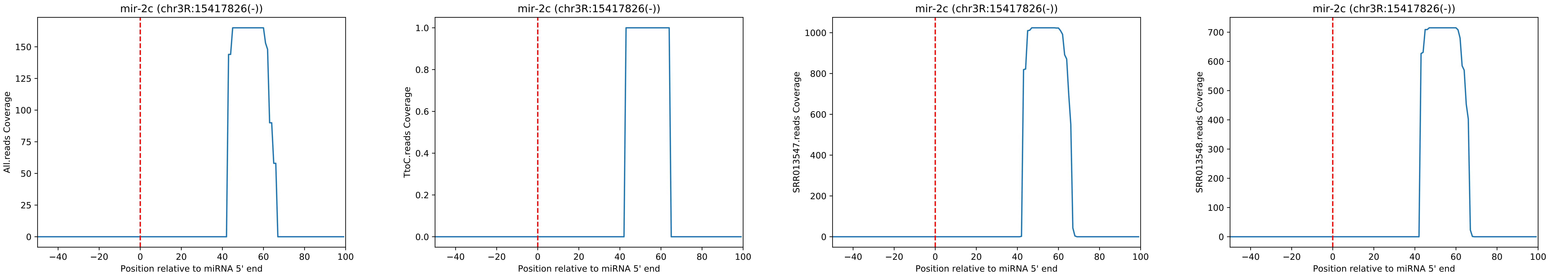

mir-2c (chr3R:15417826(-))
All.reads Coverage
Position relative to miRNA 5' end
mir-2c (chr3R:15417826(-))
TtoC.reads Coverage
Position relative to miRNA 5' end
mir-2c (chr3R:15417826(-))
SRR013547.reads Coverage
Position relative to miRNA 5' end
mir-2c (chr3R:15417826(-))
SRR013548.reads Coverage
Position relative to miRNA 5' end

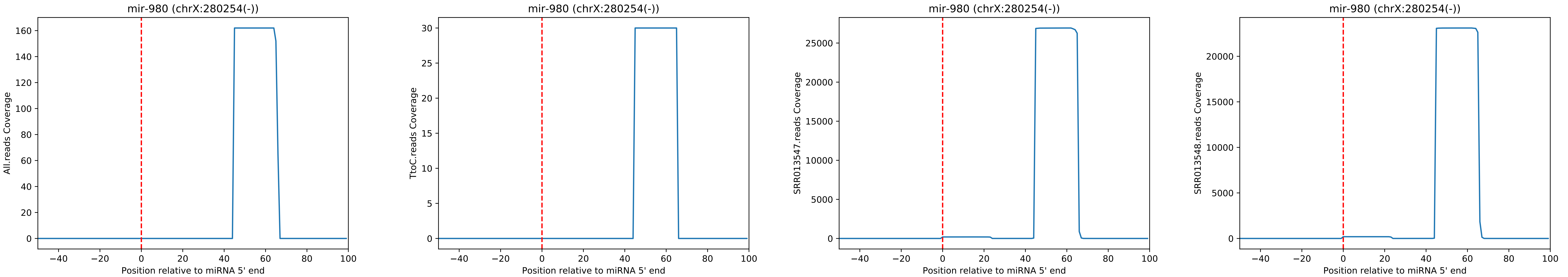
mir-980 (chrX:280254(-))
All.reads Coverage
Position relative to miRNA 5' end
mir-980 (chrX:280254(-))
TtoC.reads Coverage
Position relative to miRNA 5' end
mir-980 (chrX:280254(-))
SRR013547.reads Coverage
Position relative to miRNA 5' end
mir-980 (chrX:280254(-))
SRR013548.reads Coverage
Position relative to miRNA 5' end

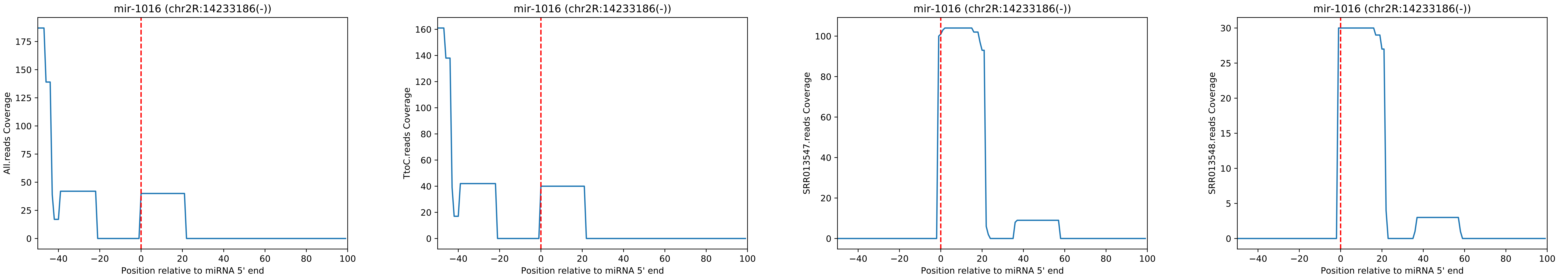
mir-1016 (chr2R:14233186(-))
All.reads Coverage
Position relative to miRNA 5' end
mir-1016 (chr2R:14233186(-))
TtoC.reads Coverage
Position relative to miRNA 5' end
mir-1016 (chr2R:14233186(-))
SRR013547.reads Coverage
Position relative to miRNA 5' end
mir-1016 (chr2R:14233186(-))
SRR013548.reads Coverage
Position relative to miRNA 5' end

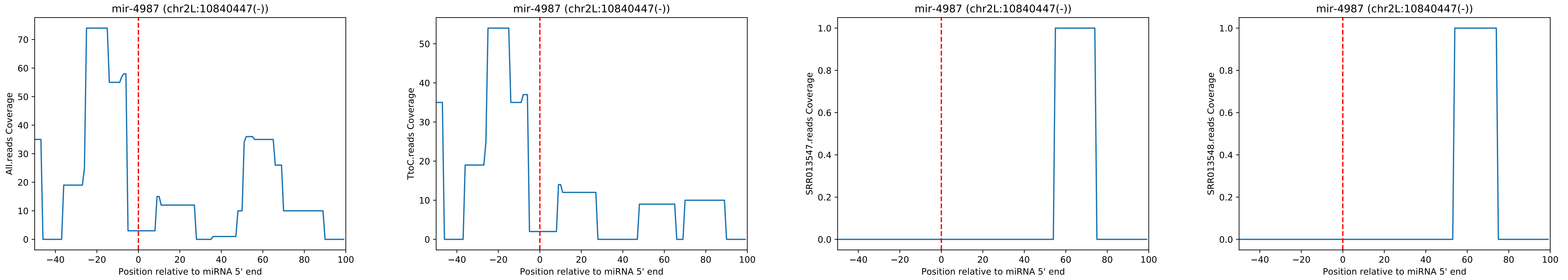

mir-4987 (chr2L:10840447(-))
All.reads Coverage
Position relative to miRNA 5' end
mir-4987 (chr2L:10840447(-))
TtoC.reads Coverage
Position relative to miRNA 5' end
mir-4987 (chr2L:10840447(-))
SRR013547.reads Coverage
Position relative to miRNA 5' end
mir-4987 (chr2L:10840447(-))
SRR013548.reads Coverage
Position relative to miRNA 5' end

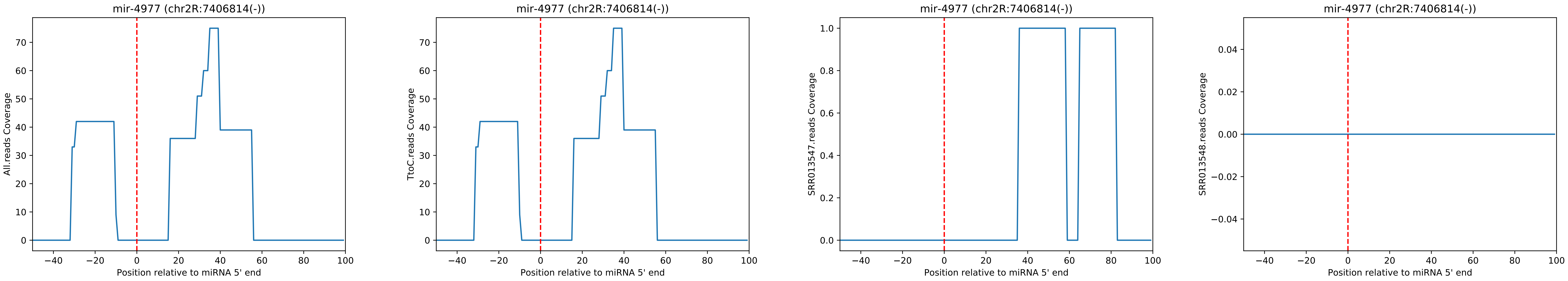
mir-4977 (chr2R:7406814(-))
All.reads Coverage
Position relative to miRNA 5' end
mir-4977 (chr2R:7406814(-))
TtoC.reads Coverage
Position relative to miRNA 5' end
mir-4977 (chr2R:7406814(-))
SRR013547.reads Coverage
Position relative to miRNA 5' end
mir-4977 (chr2R:7406814(-))
SRR013548.reads Coverage
Position relative to miRNA 5' end

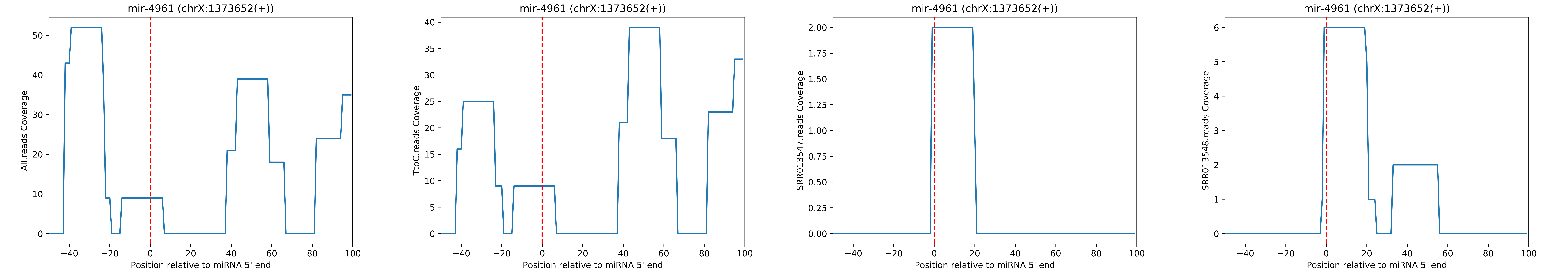
mir-4961 (chrX:1373652(+))
All.reads Coverage
Position relative to miRNA 5' end
mir-4961 (chrX:1373652(+))
TtoC.reads Coverage
Position relative to miRNA 5' end
mir-4961 (chrX:1373652(+))
SRR013547.reads Coverage
Position relative to miRNA 5' end
mir-4961 (chrX:1373652(+))
SRR013548.reads Coverage
Position relative to miRNA 5' end

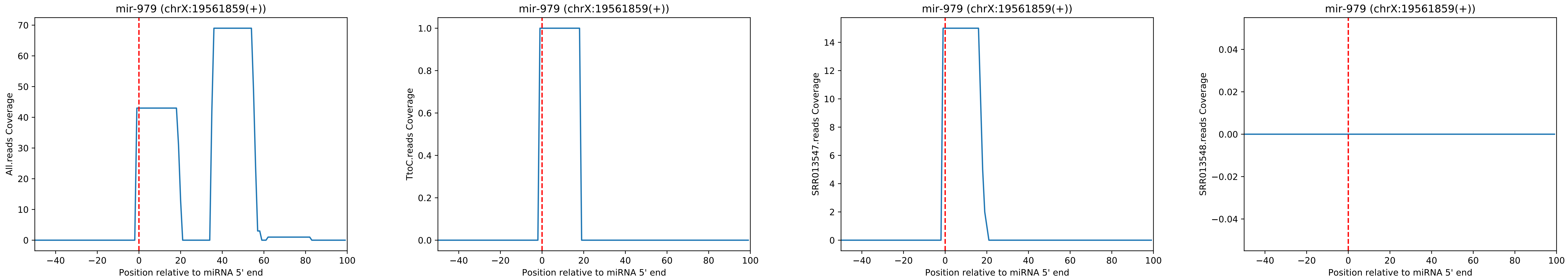

mir-979 (chrX:19561859(+))
All.reads Coverage
Position relative to miRNA 5' end
mir-979 (chrX:19561859(+))
TtoC.reads Coverage
Position relative to miRNA 5' end
mir-979 (chrX:19561859(+))
SRR013547.reads Coverage
Position relative to miRNA 5' end
mir-979 (chrX:19561859(+))
SRR013548.reads Coverage
Position relative to miRNA 5' end

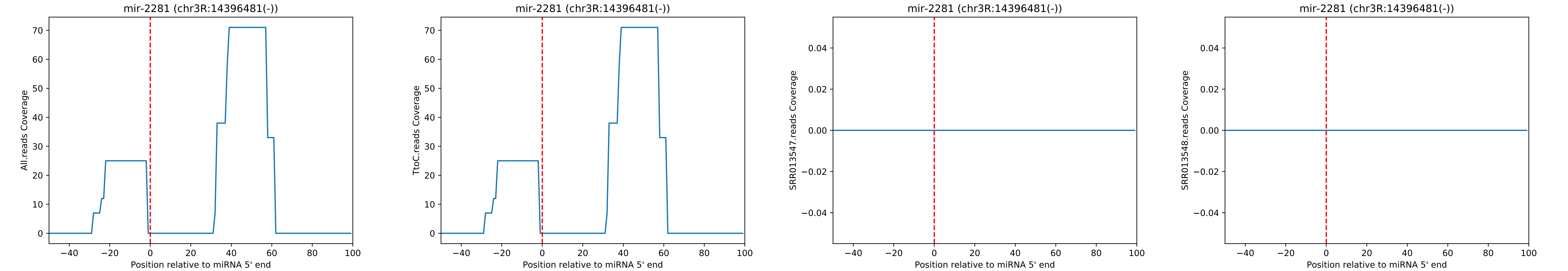

mir-2281 (chr3R:14396481(-))
All.reads Coverage
Position relative to miRNA 5' end
mir-2281 (chr3R:14396481(-))
TtoC.reads Coverage
Position relative to miRNA 5' end
mir-2281 (chr3R:14396481(-))
SRR013547.reads Coverage
Position relative to miRNA 5' end
mir-2281 (chr3R:14396481(-))
SRR013548.reads Coverage
Position relative to miRNA 5' end

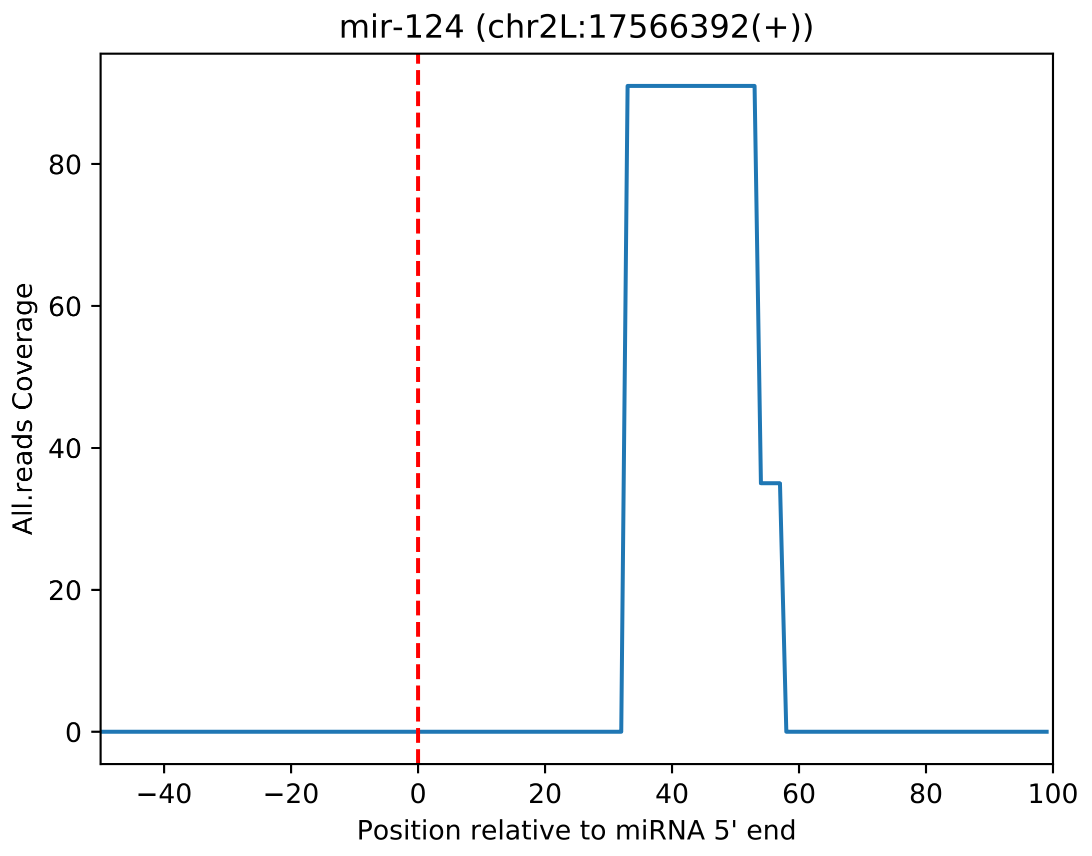
mir-124 (chr2L:17566392(+))
All.reads Coverage
Position relative to miRNA 5' end
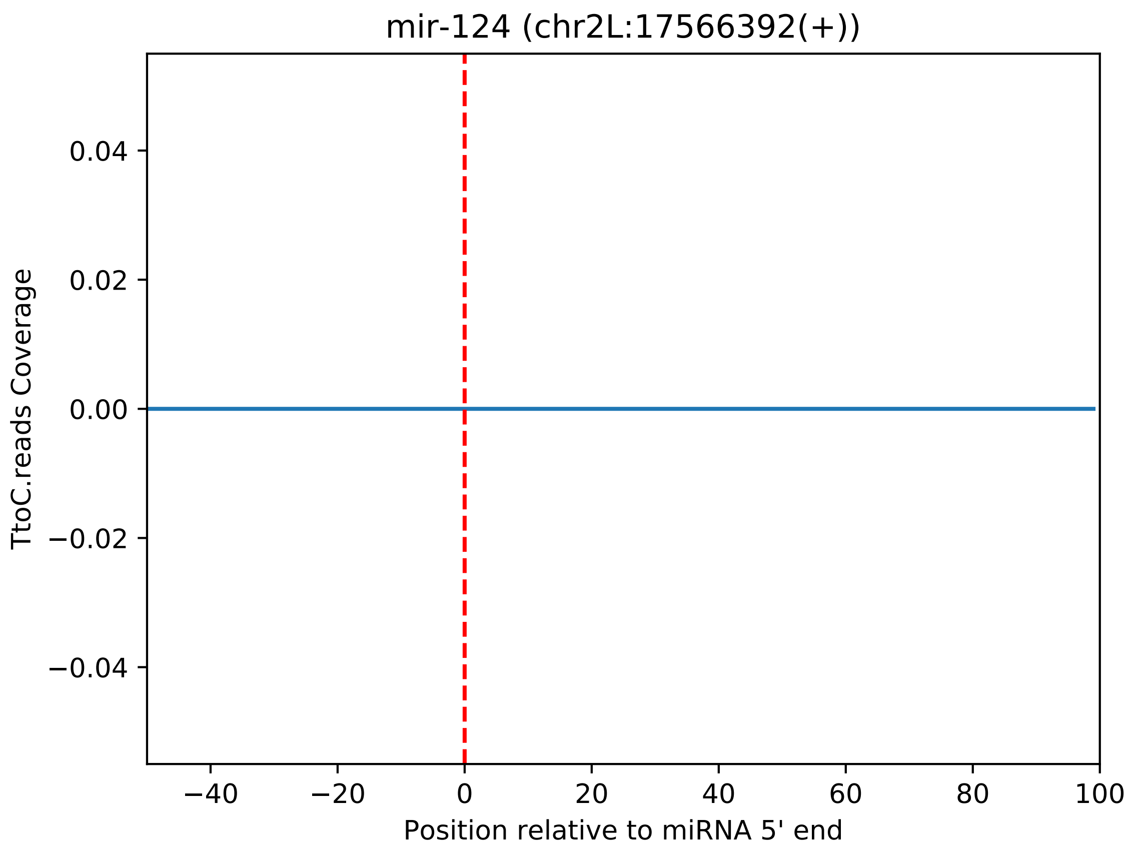
mir-124 (chr2L:17566392(+))
TtoC.reads Coverage
Position relative to miRNA 5' end
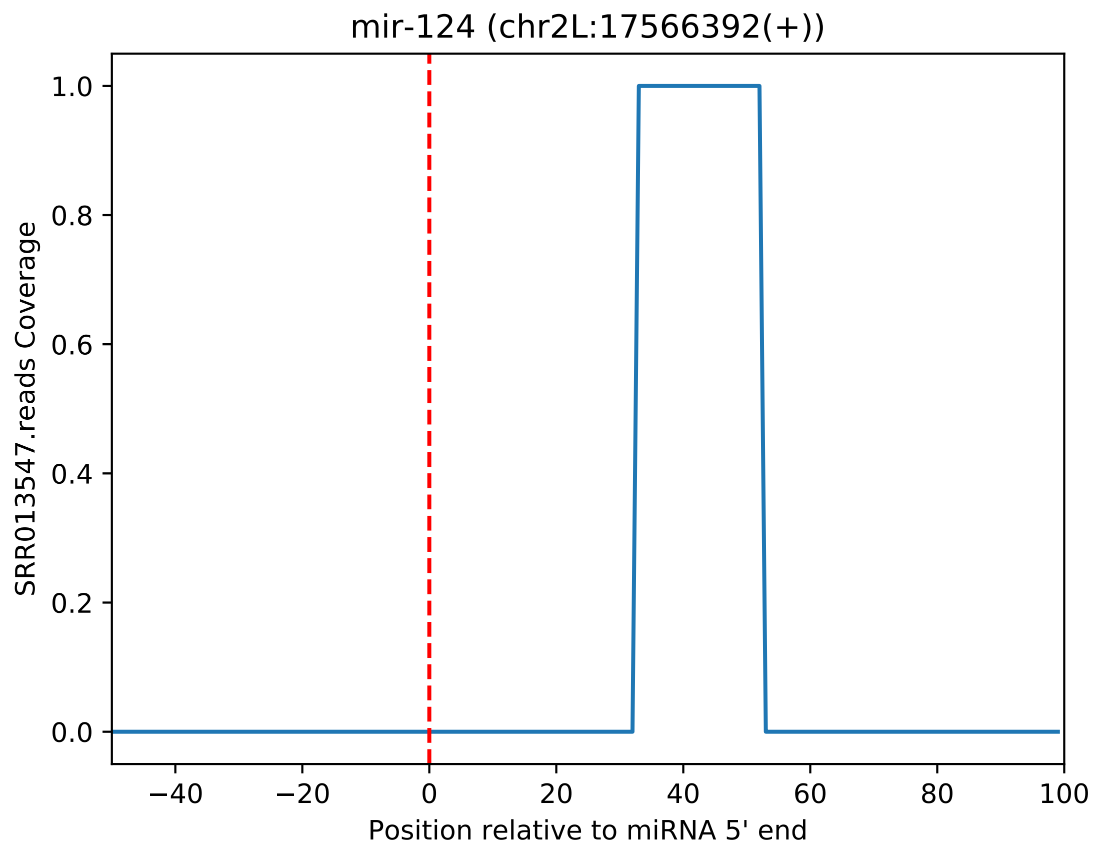
mir-124 (chr2L:17566392(+))
SRR013547.reads Coverage
Position relative to miRNA 5' end
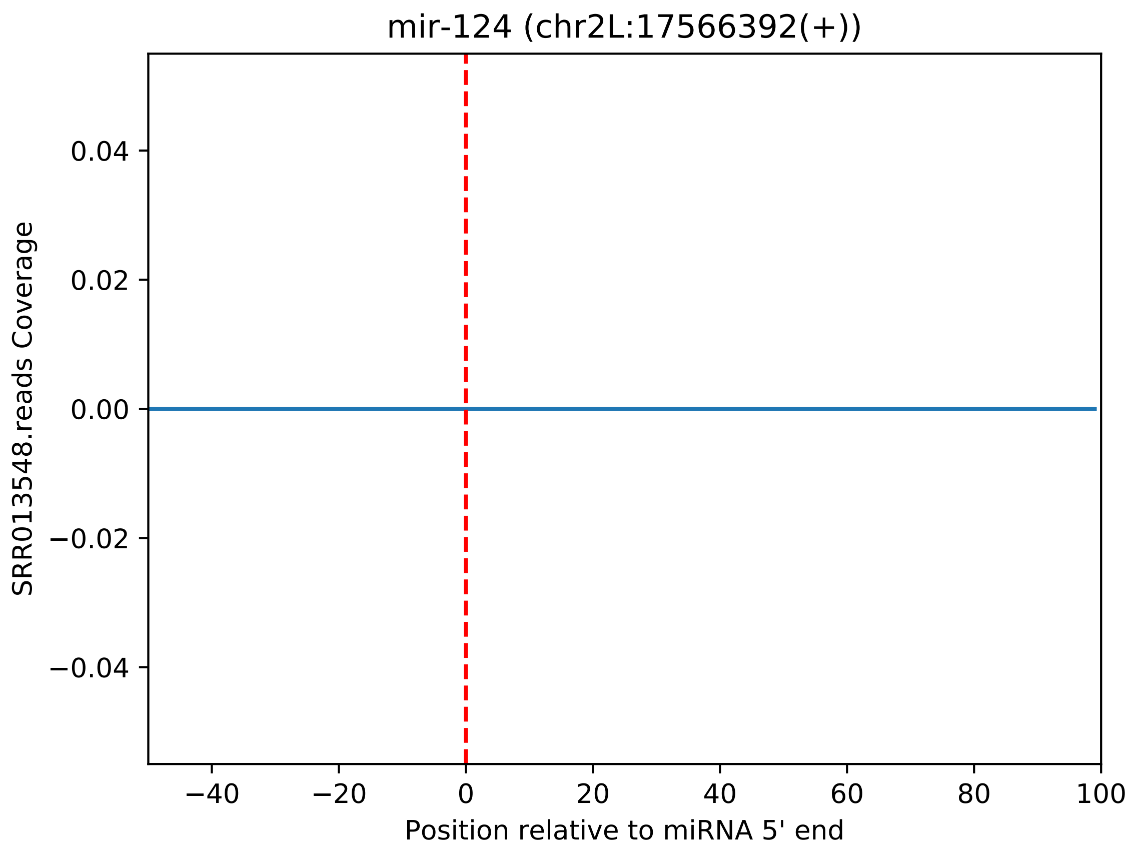
mir-124 (chr2L:17566392(+))
SRR013548.reads Coverage
Position relative to miRNA 5' end

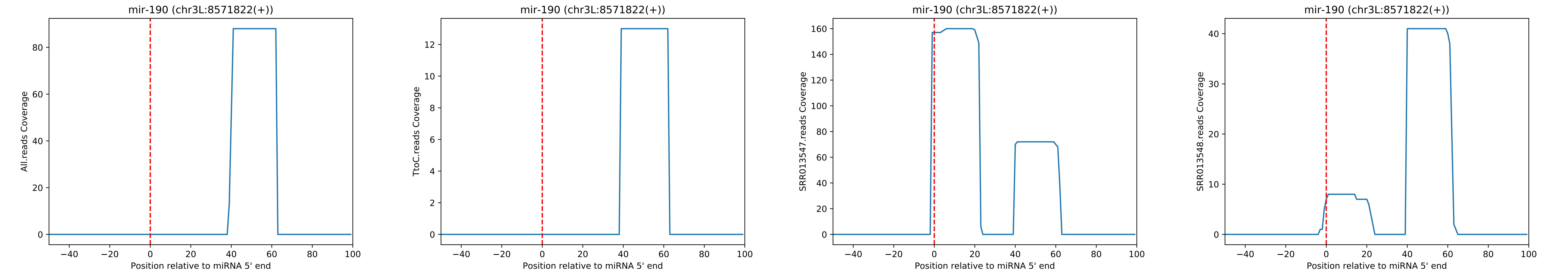

mir-190 (chr3L:8571822(+))
All.reads Coverage
Position relative to miRNA 5' end
mir-190 (chr3L:8571822(+))
TtoC.reads Coverage
Position relative to miRNA 5' end
mir-190 (chr3L:8571822(+))
SRR013547.reads Coverage
Position relative to miRNA 5' end
mir-190 (chr3L:8571822(+))
SRR013548.reads Coverage
Position relative to miRNA 5' end

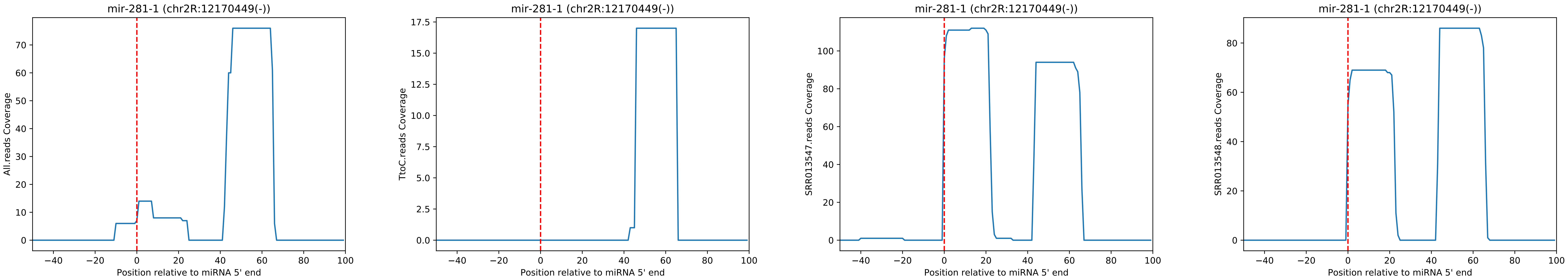

mir-281-1 (chr2R:12170449(-))
All.reads Coverage
Position relative to miRNA 5' end
mir-281-1 (chr2R:12170449(-))
TtoC.reads Coverage
Position relative to miRNA 5' end
mir-281-1 (chr2R:12170449(-))
SRR013547.reads Coverage
Position relative to miRNA 5' end
mir-281-1 (chr2R:12170449(-))
SRR013548.reads Coverage
Position relative to miRNA 5' end

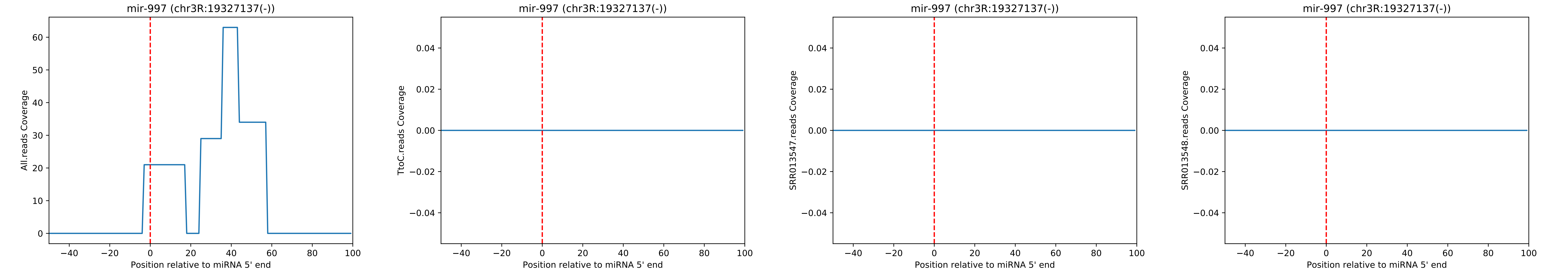
mir-997 (chr3R:19327137(-))
All.reads Coverage
0
10
20
30
40
50
60
−40
−20
0
20
40
60
80
100
Position relative to miRNA 5' end
mir-997 (chr3R:19327137(-))
TtoC.reads Coverage
0.04
0.02
0.00
−0.02
−0.04
Position relative to miRNA 5' end
mir-997 (chr3R:19327137(-))
SRR013547.reads Coverage
Position relative to miRNA 5' end
mir-997 (chr3R:19327137(-))
SRR013548.reads Coverage
Position relative to miRNA 5' end

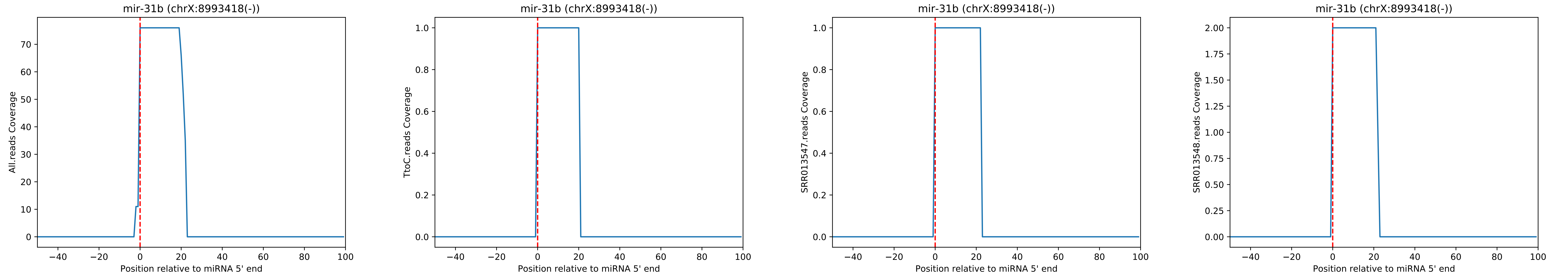
mir-31b (chrX:8993418(-))
All.reads Coverage
Position relative to miRNA 5' end
mir-31b (chrX:8993418(-))
TtoC.reads Coverage
Position relative to miRNA 5' end
mir-31b (chrX:8993418(-))
SRR013547.reads Coverage
Position relative to miRNA 5' end
mir-31b (chrX:8993418(-))
SRR013548.reads Coverage
Position relative to miRNA 5' end

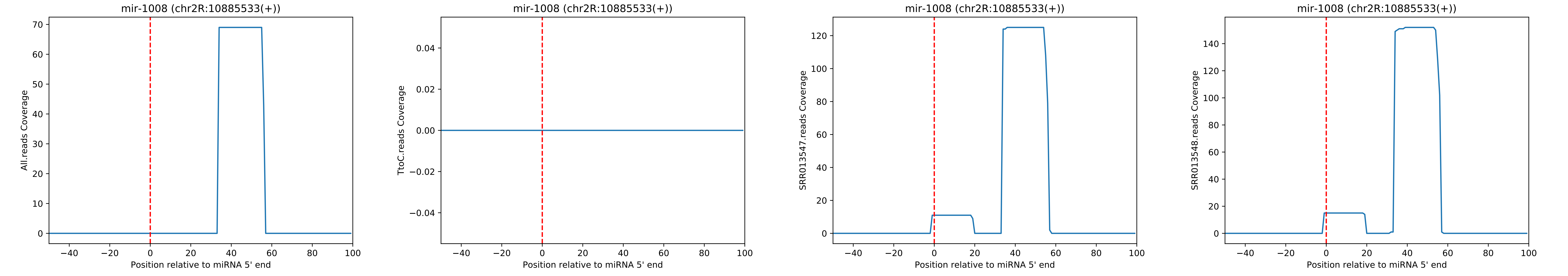

mir-1008 (chr2R:10885533(+))
All.reads Coverage
Position relative to miRNA 5' end
mir-1008 (chr2R:10885533(+))
TtoC.reads Coverage
Position relative to miRNA 5' end
mir-1008 (chr2R:10885533(+))
SRR013547.reads Coverage
Position relative to miRNA 5' end
mir-1008 (chr2R:10885533(+))
SRR013548.reads Coverage
Position relative to miRNA 5' end

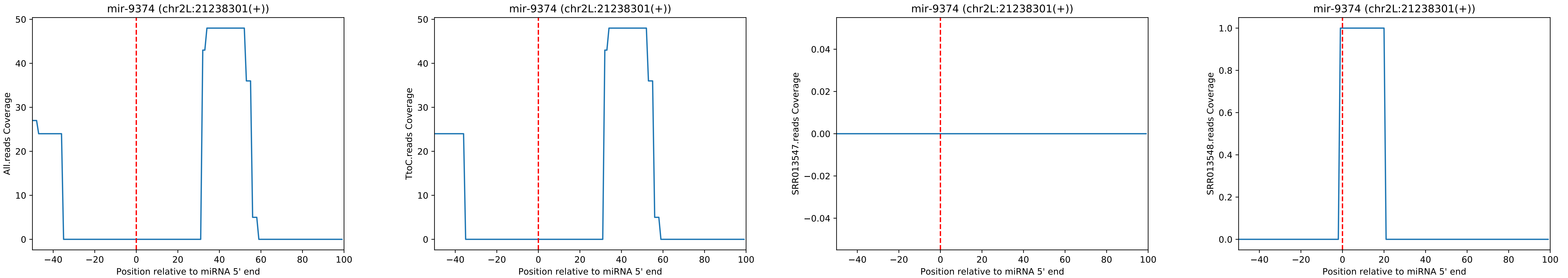

mir-9374 (chr2L:21238301(+))
All.reads Coverage
Position relative to miRNA 5' end
mir-9374 (chr2L:21238301(+))
TtoC.reads Coverage
Position relative to miRNA 5' end
mir-9374 (chr2L:21238301(+))
SRR013547.reads Coverage
Position relative to miRNA 5' end
mir-9374 (chr2L:21238301(+))
SRR013548.reads Coverage
Position relative to miRNA 5' end

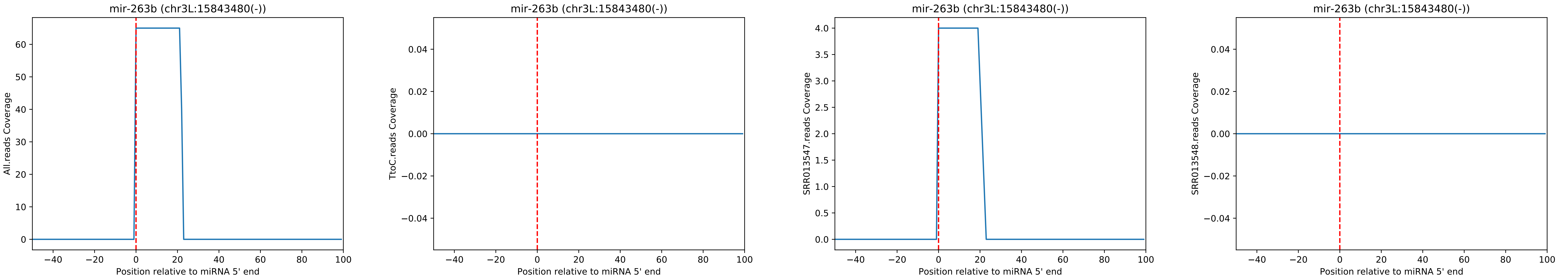
mir-263b (chr3L:15843480(-))
All.reads Coverage
Position relative to miRNA 5' end
mir-263b (chr3L:15843480(-))
TtoC.reads Coverage
Position relative to miRNA 5' end
mir-263b (chr3L:15843480(-))
SRR013547.reads Coverage
Position relative to miRNA 5' end
mir-263b (chr3L:15843480(-))
SRR013548.reads Coverage
Position relative to miRNA 5' end

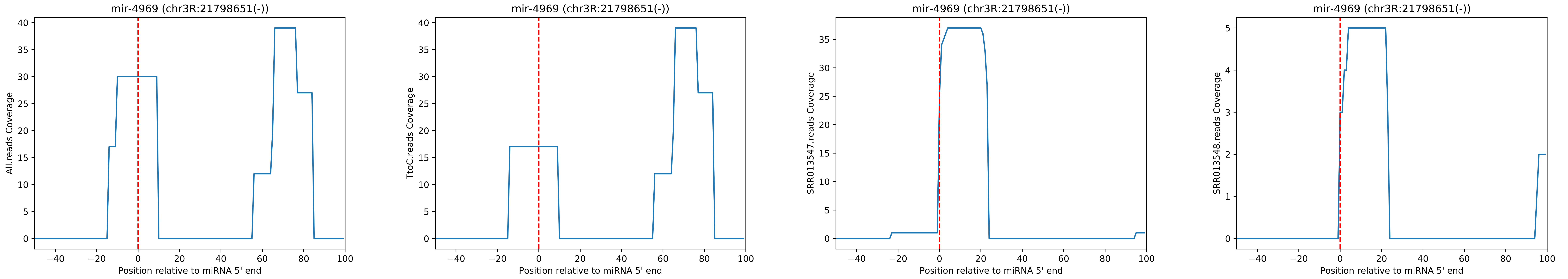
mir-4969 (chr3R:21798651(-))
All.reads Coverage
TtoC.reads Coverage
SRR013547.reads Coverage
SRR013548.reads Coverage
Position relative to miRNA 5' end
−40
−20
0
20
40
60
80
100
0
5
10
15
20
25
30
35
40
1
2
3
4

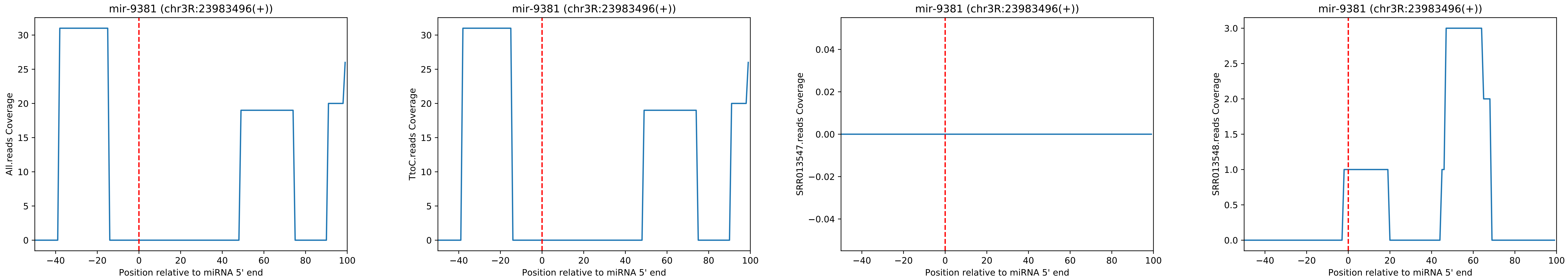

mir-9381 (chr3R:23983496(+))
All.reads Coverage
0
5
10
15
20
25
30
−40
−20
0
20
40
60
80
100
Position relative to miRNA 5' end
mir-9381 (chr3R:23983496(+))
TtoC.reads Coverage
0
5
10
15
20
25
30
−40
−20
0
20
40
60
80
100
Position relative to miRNA 5' end
mir-9381 (chr3R:23983496(+))
SRR013547.reads Coverage
0.04
0.02
0.00
−0.02
−0.04
−40
−20
0
20
40
60
80
100
Position relative to miRNA 5' end
mir-9381 (chr3R:23983496(+))
SRR013548.reads Coverage
0.0
0.5
1.0
1.5
2.0
2.5
3.0
−40
−20
0
20
40
60
80
100
Position relative to miRNA 5' end

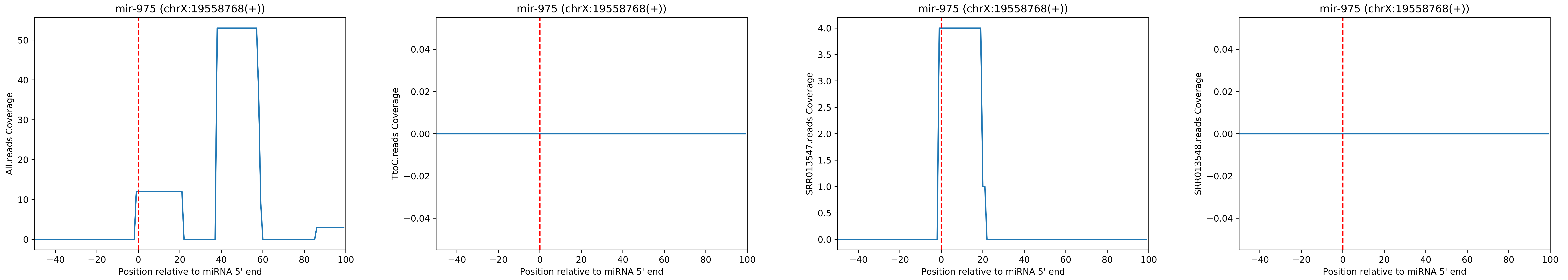

mir-975 (chrX:19558768(+))
All.reads Coverage
TtoC.reads Coverage
SRR013547.reads Coverage
SRR013548.reads Coverage
Position relative to miRNA 5' end

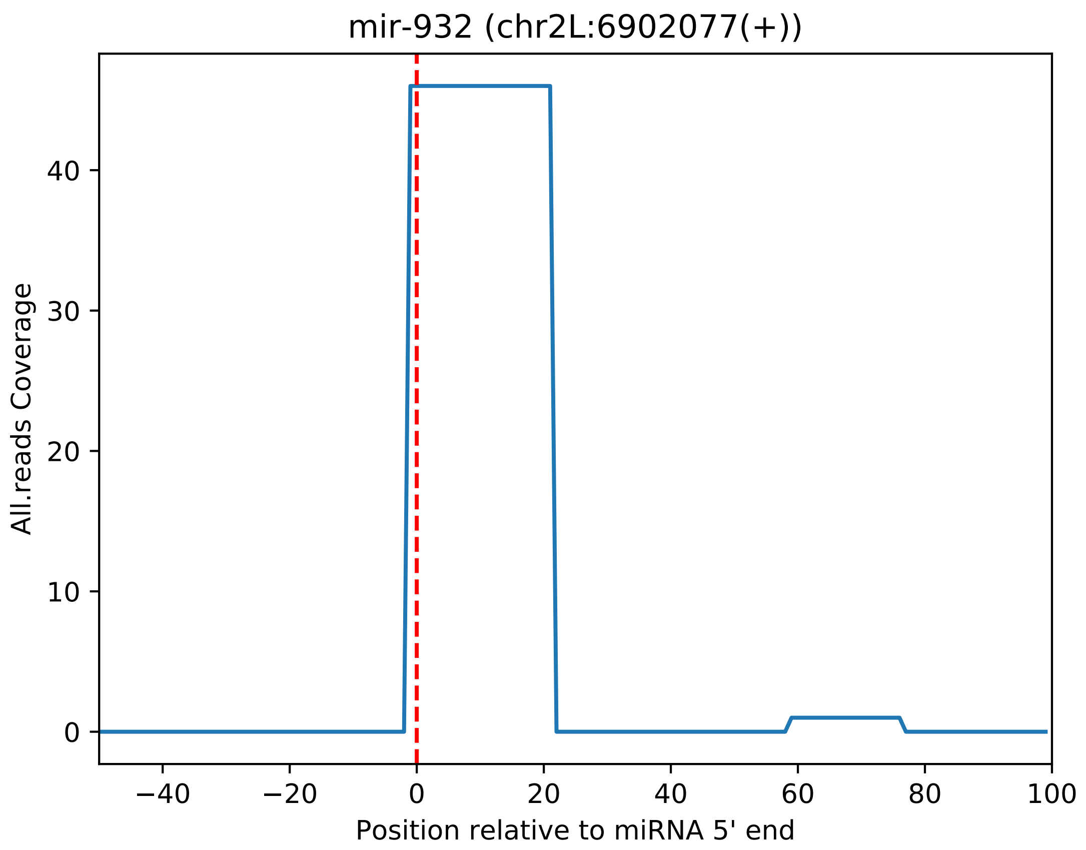
mir-932 (chr2L:6902077(+))
All.reads Coverage
0
10
20
30
40
−40
−20
0
20
40
60
80
100
Position relative to miRNA 5' end

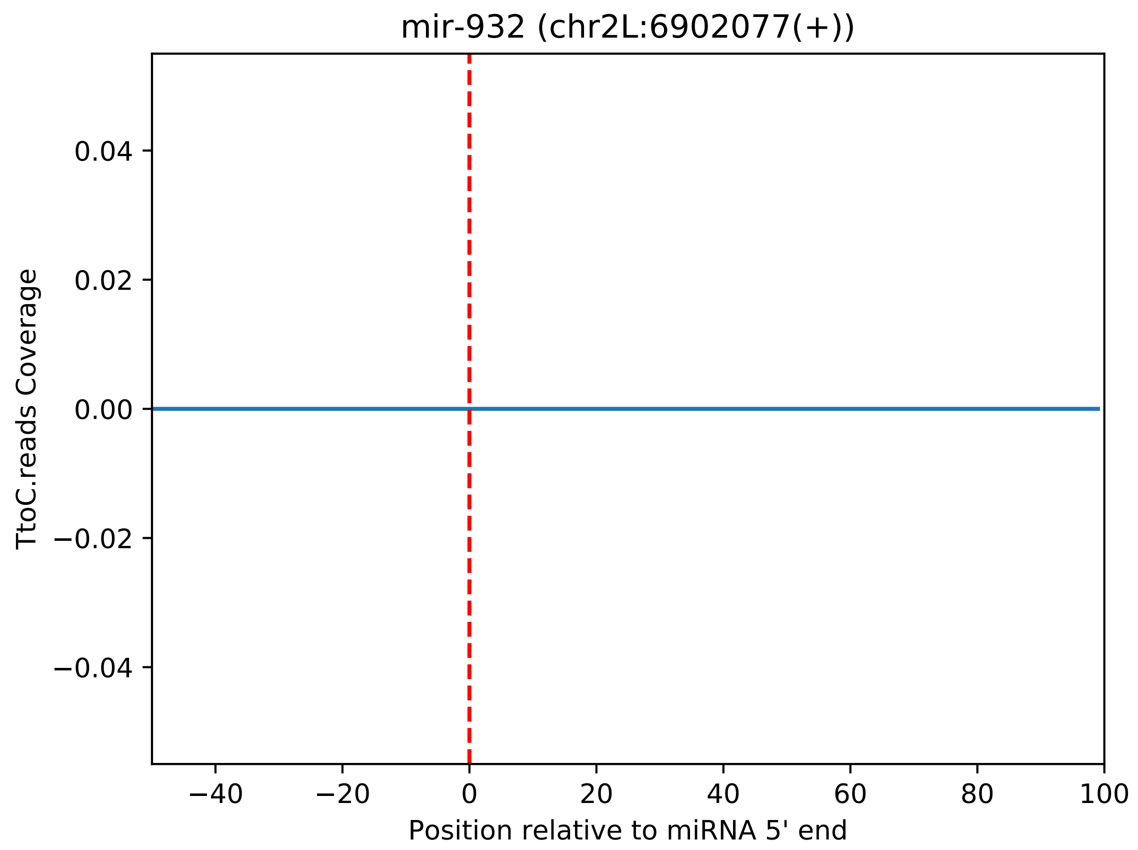
mir-932 (chr2L:6902077(+))
TtoC.reads Coverage
0.04
0.02
0.00
−0.02
−0.04
−40
−20
0
20
40
60
80
100
Position relative to miRNA 5' end

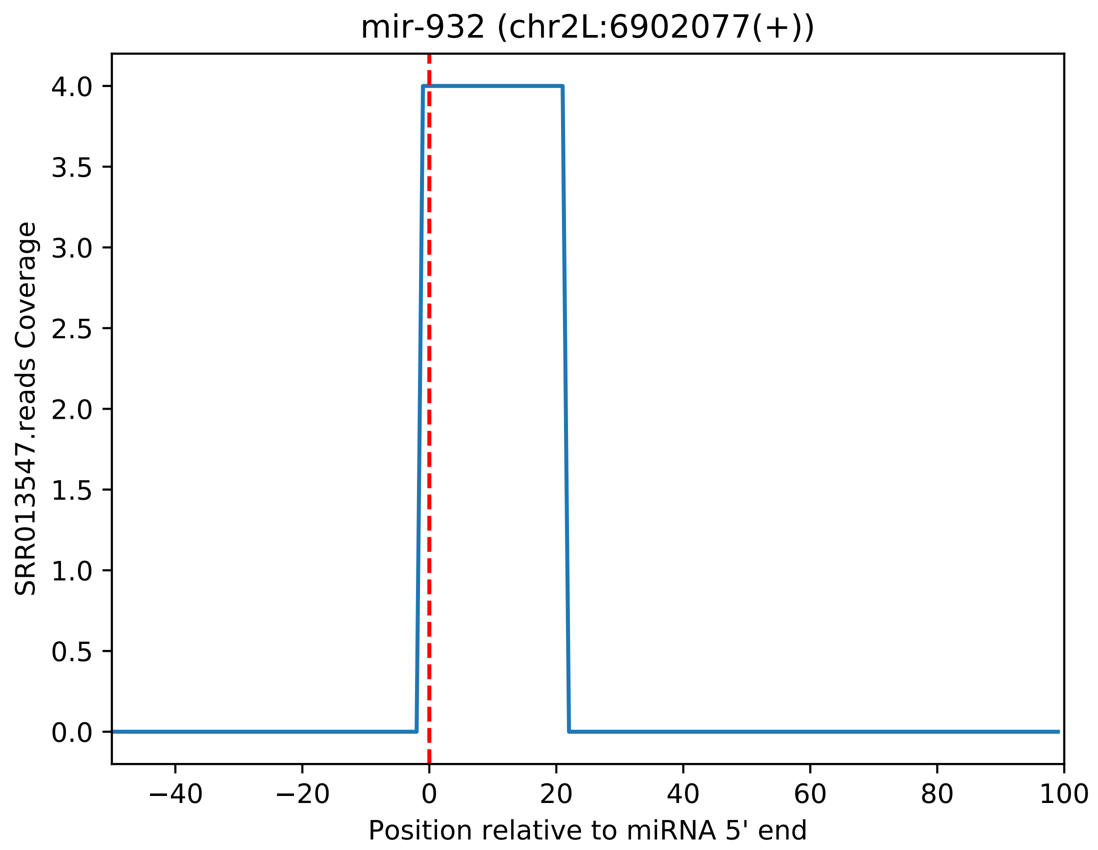
mir-932 (chr2L:6902077(+))
SRR013547.reads Coverage
4.0
3.5
3.0
2.5
2.0
1.5
1.0
0.5
0.0
−40
−20
0
20
40
60
80
100
Position relative to miRNA 5' end

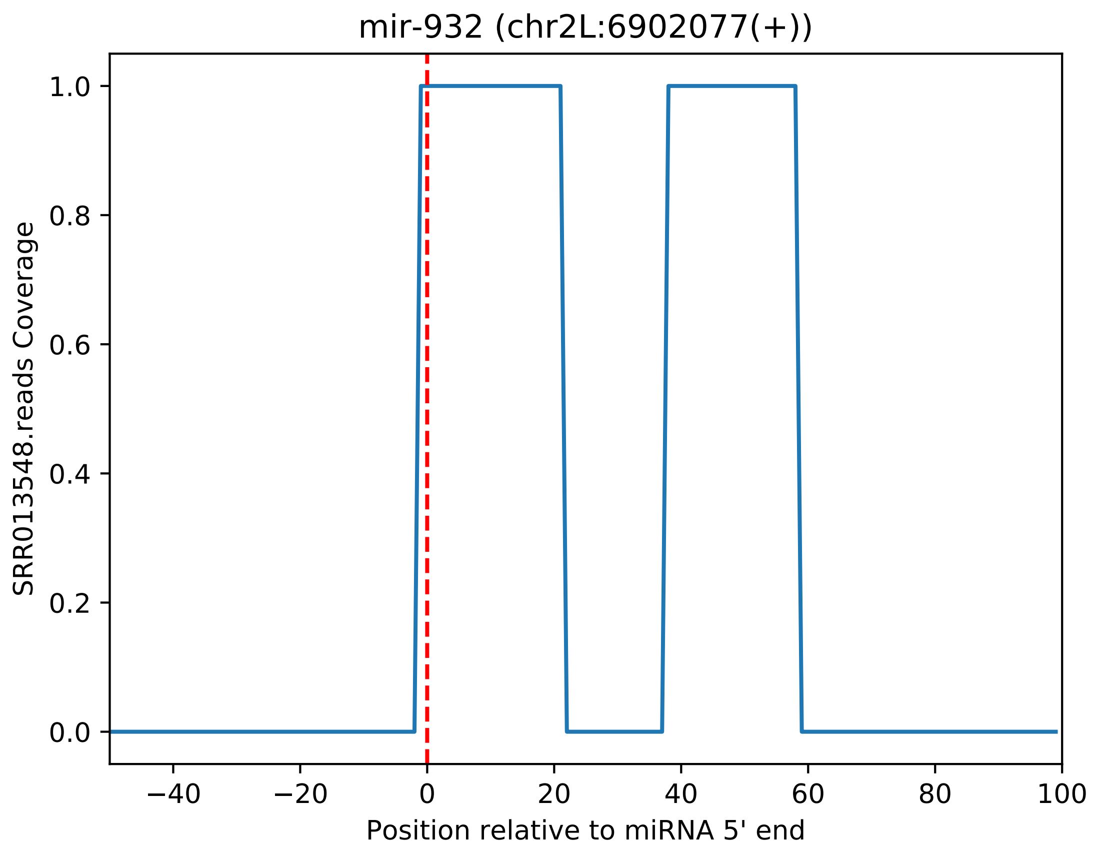
mir-932 (chr2L:6902077(+))
SRR013548.reads Coverage
1.0
0.8
0.6
0.4
0.2
0.0
−40
−20
0
20
40
60
80
100
Position relative to miRNA 5' end

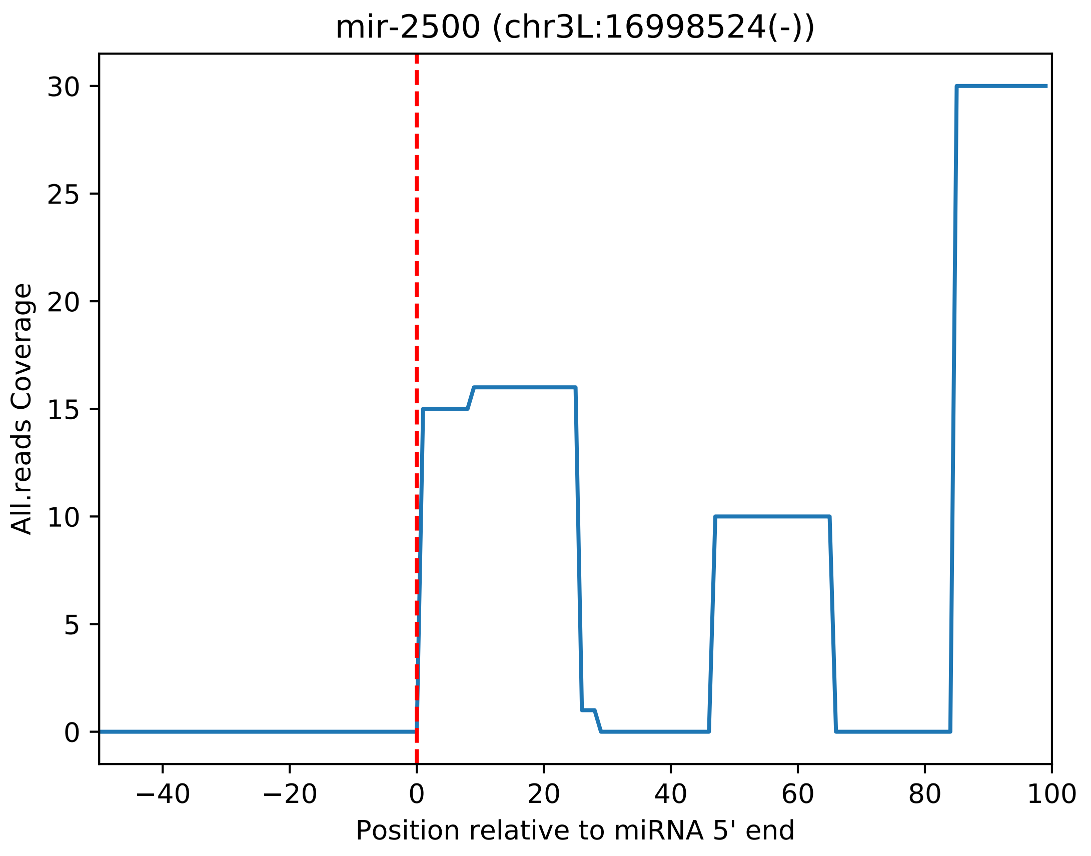
mir-2500 (chr3L:16998524(-))
All.reads Coverage
Position relative to miRNA 5' end

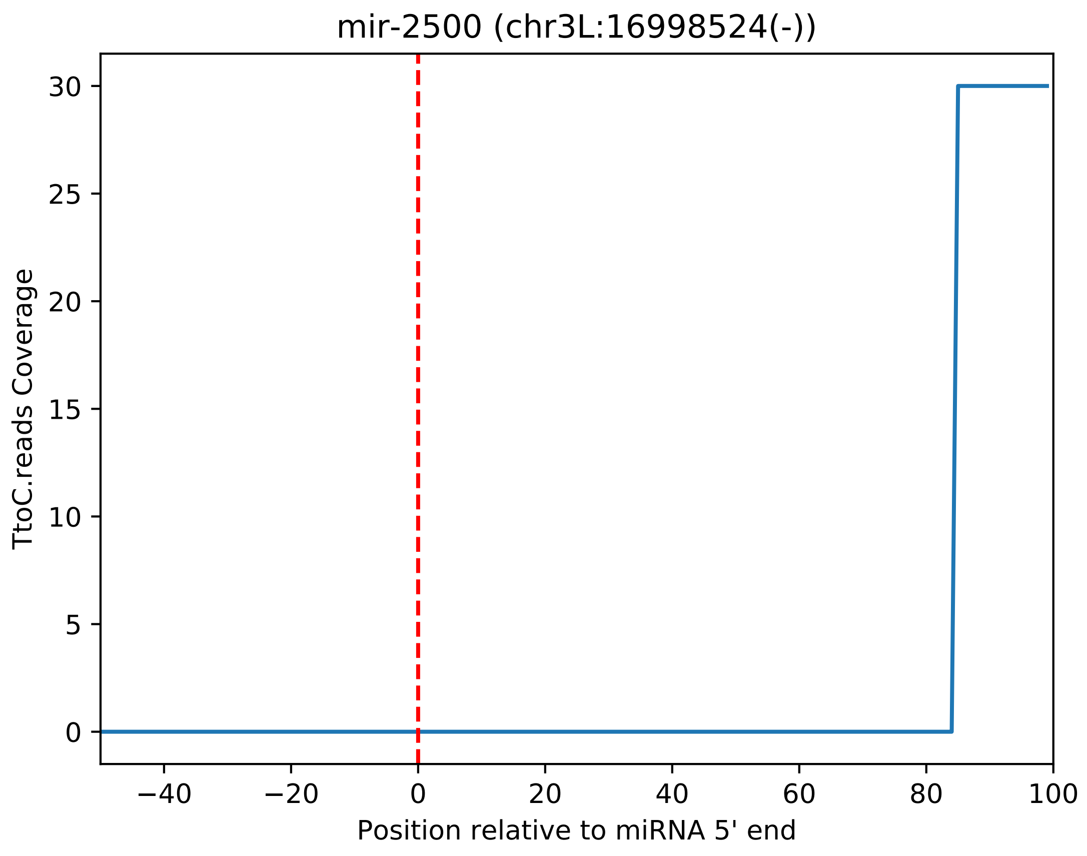
mir-2500 (chr3L:16998524(-))
TtoC.reads Coverage
Position relative to miRNA 5' end

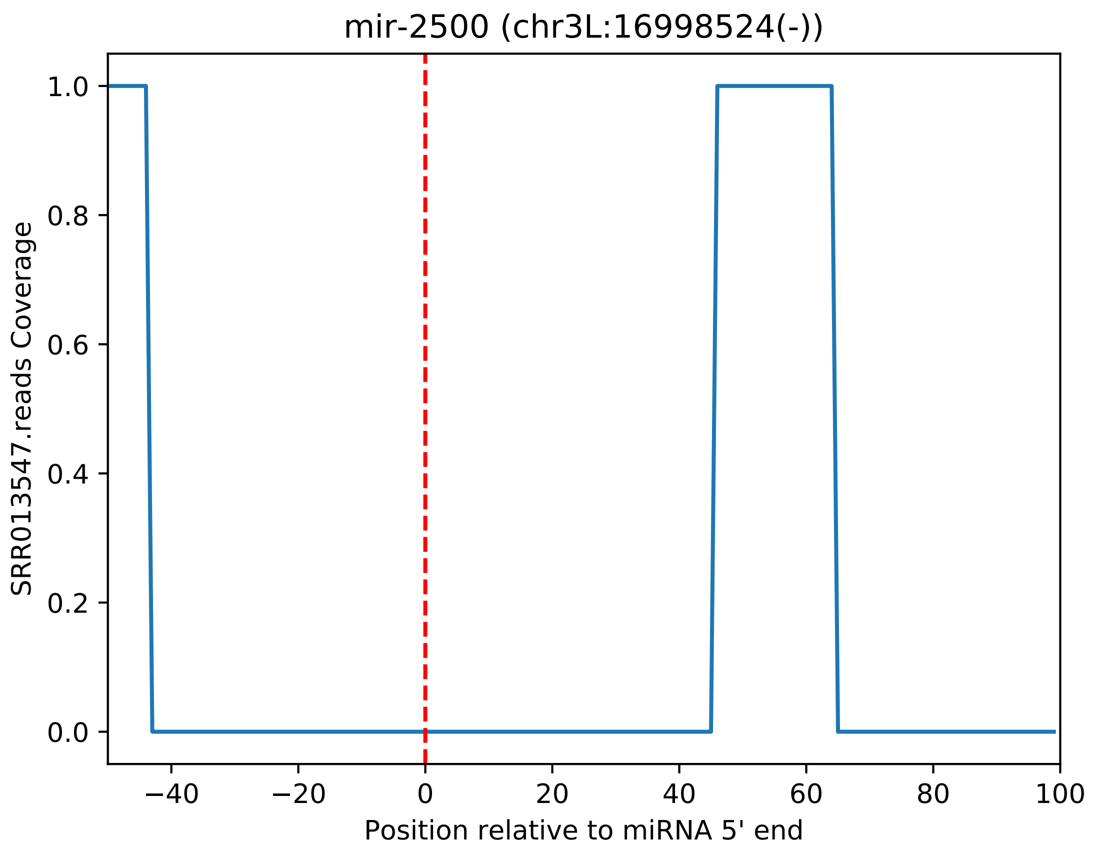
mir-2500 (chr3L:16998524(-))
SRR013547.reads Coverage
Position relative to miRNA 5' end

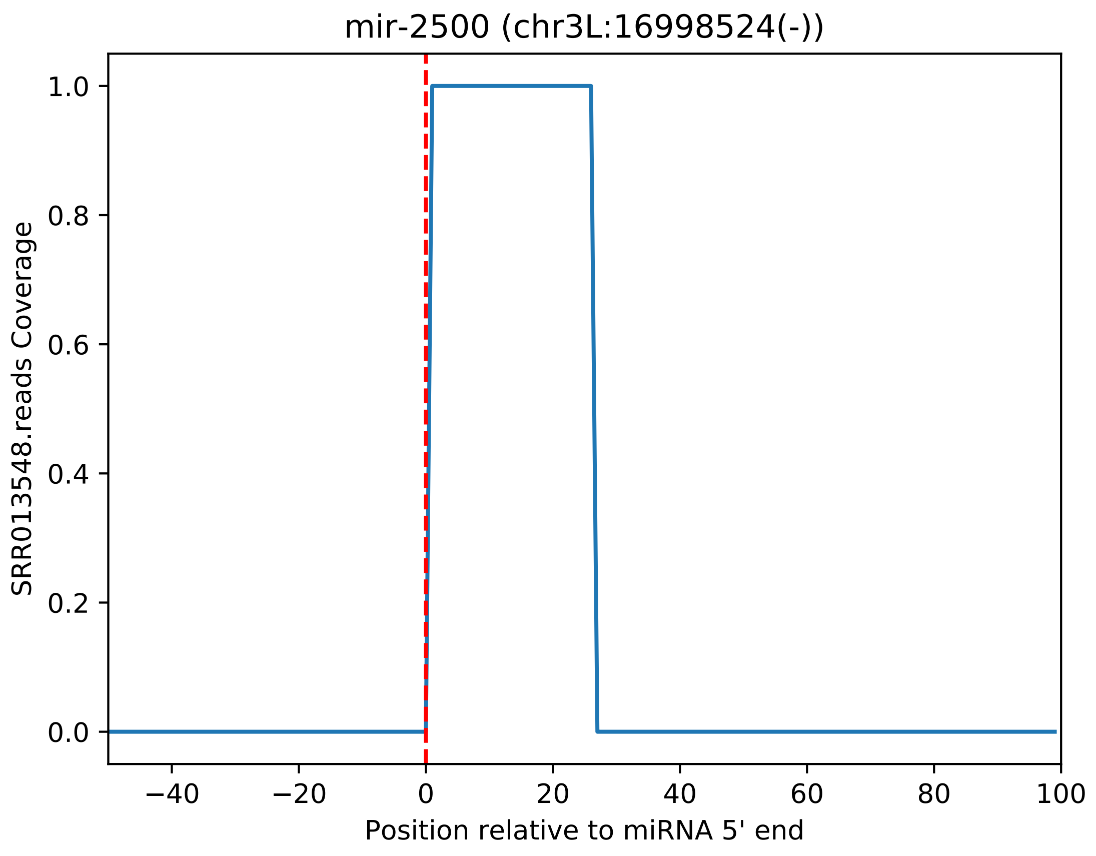
mir-2500 (chr3L:16998524(-))
SRR013548.reads Coverage
Position relative to miRNA 5' end

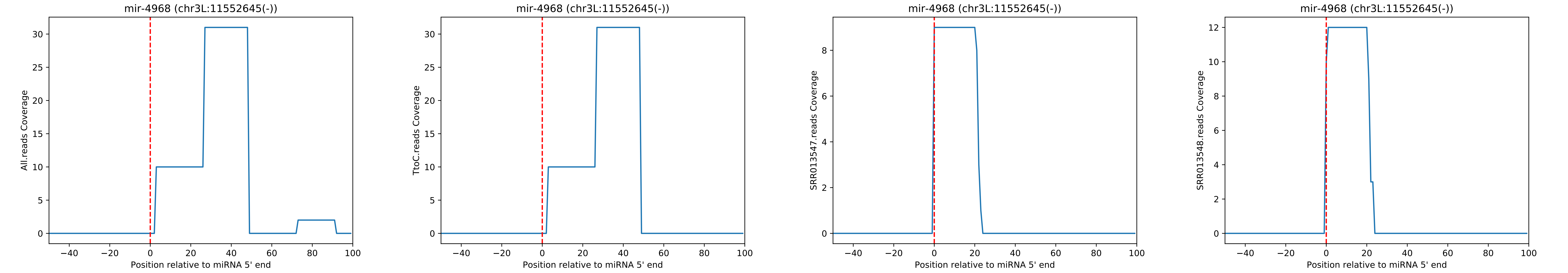
mir-4968 (chr3L:11552645(-))
All.reads Coverage
Position relative to miRNA 5' end
mir-4968 (chr3L:11552645(-))
TtoC.reads Coverage
Position relative to miRNA 5' end
mir-4968 (chr3L:11552645(-))
SRR013547.reads Coverage
Position relative to miRNA 5' end
mir-4968 (chr3L:11552645(-))
SRR013548.reads Coverage
Position relative to miRNA 5' end

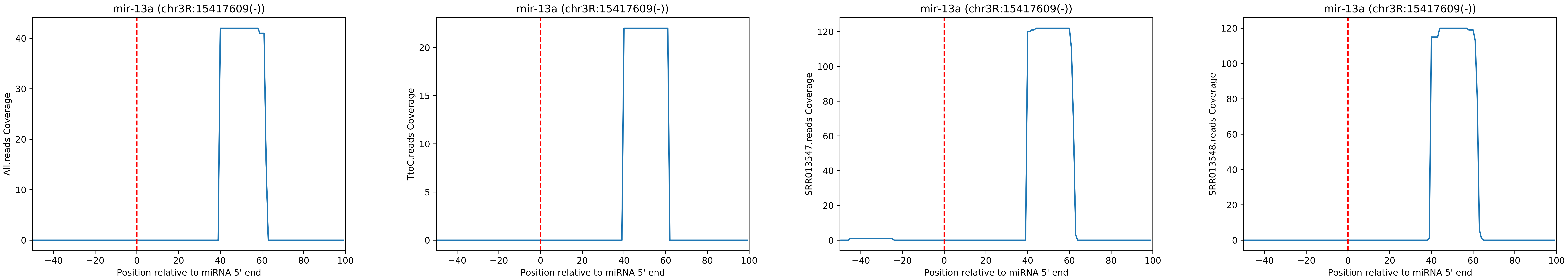

mir-13a (chr3R:15417609(-))
All.reads Coverage
TtoC.reads Coverage
SRR013547.reads Coverage
SRR013548.reads Coverage
Position relative to miRNA 5' end

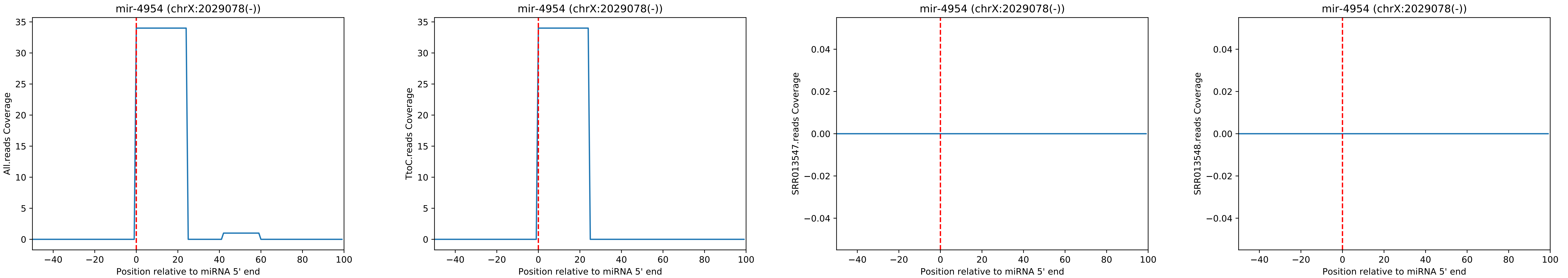
mir-4954 (chrX:2029078(-))
All.reads Coverage
TtoC.reads Coverage
SRR013547.reads Coverage
SRR013548.reads Coverage
Position relative to miRNA 5' end

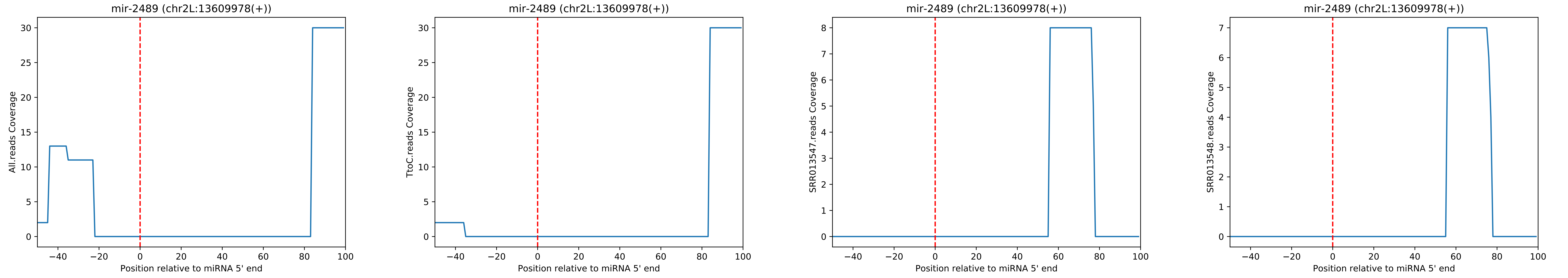
mir-2489 (chr2L:13609978(+))
All.reads Coverage
Position relative to miRNA 5' end
mir-2489 (chr2L:13609978(+))
TtoC.reads Coverage
Position relative to miRNA 5' end
mir-2489 (chr2L:13609978(+))
SRR013547.reads Coverage
Position relative to miRNA 5' end
mir-2489 (chr2L:13609978(+))
SRR013548.reads Coverage
Position relative to miRNA 5' end

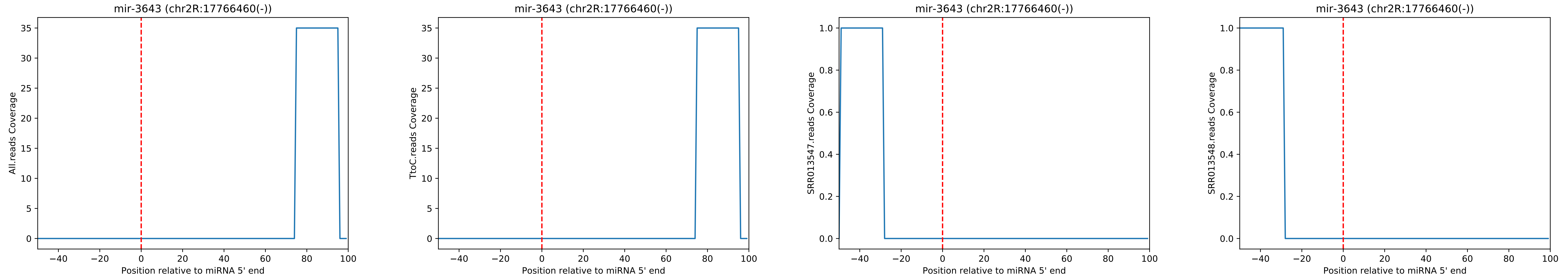

mir-3643 (chr2R:17766460(-))
All.reads Coverage
Position relative to miRNA 5' end
mir-3643 (chr2R:17766460(-))
TtoC.reads Coverage
Position relative to miRNA 5' end
mir-3643 (chr2R:17766460(-))
SRR013547.reads Coverage
Position relative to miRNA 5' end
mir-3643 (chr2R:17766460(-))
SRR013548.reads Coverage
Position relative to miRNA 5' end

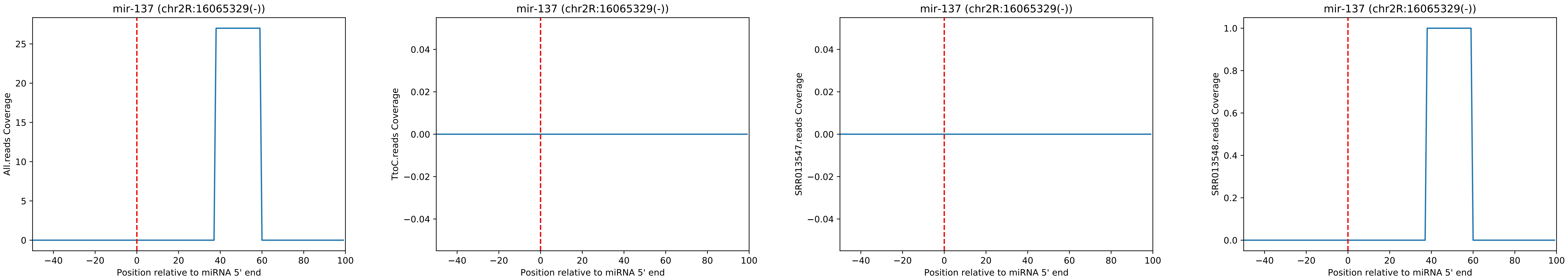
mir-137 (chr2R:16065329(-))
All.reads Coverage
Position relative to miRNA 5' end
mir-137 (chr2R:16065329(-))
TtoC.reads Coverage
Position relative to miRNA 5' end
mir-137 (chr2R:16065329(-))
SRR013547.reads Coverage
Position relative to miRNA 5' end
mir-137 (chr2R:16065329(-))
SRR013548.reads Coverage
Position relative to miRNA 5' end

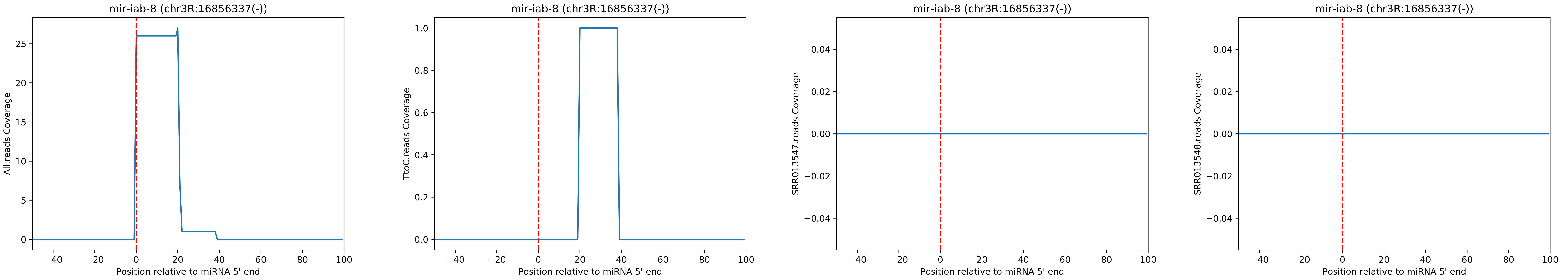
mir-iab-8 (chr3R:16856337(-))
All.reads Coverage
TtoC.reads Coverage
SRR013547.reads Coverage
SRR013548.reads Coverage
Position relative to miRNA 5' end

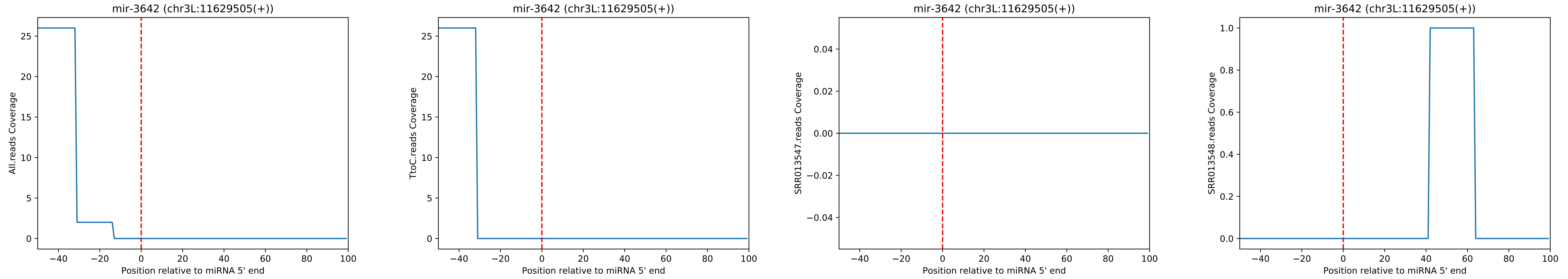
mir-3642 (chr3L:11629505(+))
All.reads Coverage
Position relative to miRNA 5' end
mir-3642 (chr3L:11629505(+))
TtoC.reads Coverage
Position relative to miRNA 5' end
mir-3642 (chr3L:11629505(+))
SRR013547.reads Coverage
Position relative to miRNA 5' end
mir-3642 (chr3L:11629505(+))
SRR013548.reads Coverage
Position relative to miRNA 5' end

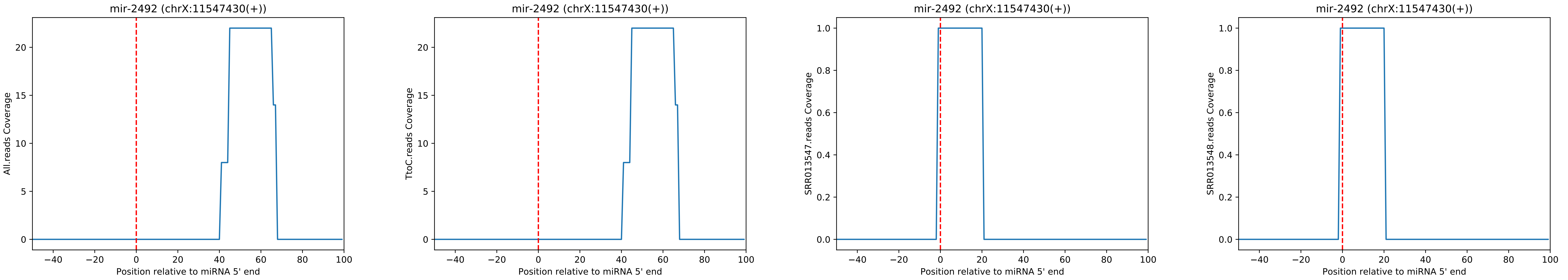
mir-2492 (chrX:11547430(+))
All.reads Coverage
Position relative to miRNA 5' end
mir-2492 (chrX:11547430(+))
TtoC.reads Coverage
Position relative to miRNA 5' end
mir-2492 (chrX:11547430(+))
SRR013547.reads Coverage
Position relative to miRNA 5' end
mir-2492 (chrX:11547430(+))
SRR013548.reads Coverage
Position relative to miRNA 5' end

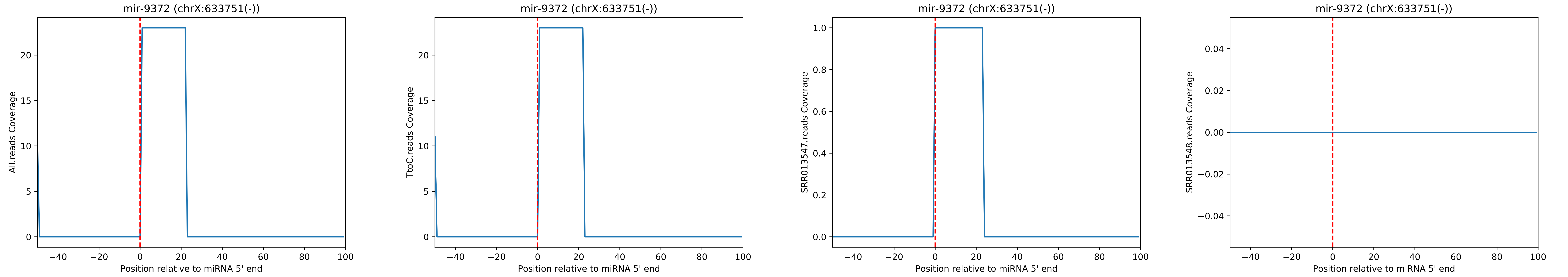
mir-9372 (chrX:633751(-))
All.reads Coverage
Position relative to miRNA 5' end
mir-9372 (chrX:633751(-))
TtoC.reads Coverage
Position relative to miRNA 5' end
mir-9372 (chrX:633751(-))
SRR013547.reads Coverage
Position relative to miRNA 5' end
mir-9372 (chrX:633751(-))
SRR013548.reads Coverage
Position relative to miRNA 5' end

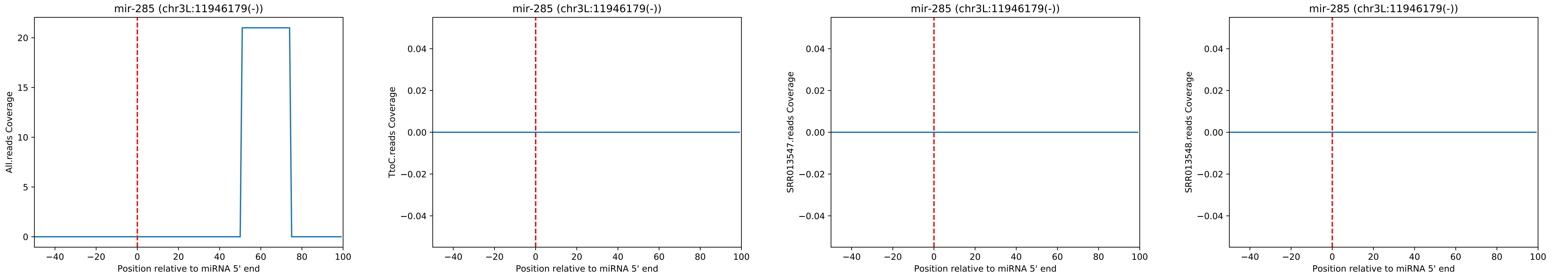
mir-285 (chr3L:11946179(-))
All.reads Coverage
TtoC.reads Coverage
SRR013547.reads Coverage
SRR013548.reads Coverage
Position relative to miRNA 5' end

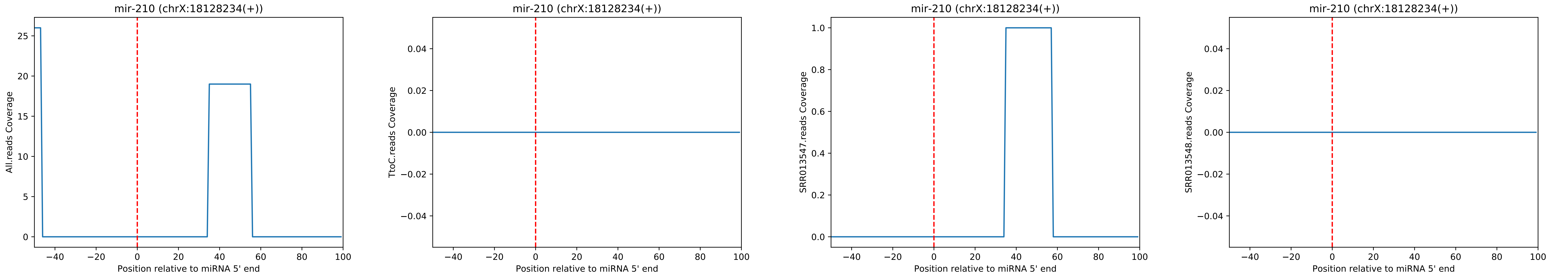

mir-210 (chrX:18128234(+))
All.reads Coverage
Position relative to miRNA 5' end
mir-210 (chrX:18128234(+))
TtoC.reads Coverage
Position relative to miRNA 5' end
mir-210 (chrX:18128234(+))
SRR013547.reads Coverage
Position relative to miRNA 5' end
mir-210 (chrX:18128234(+))
SRR013548.reads Coverage
Position relative to miRNA 5' end

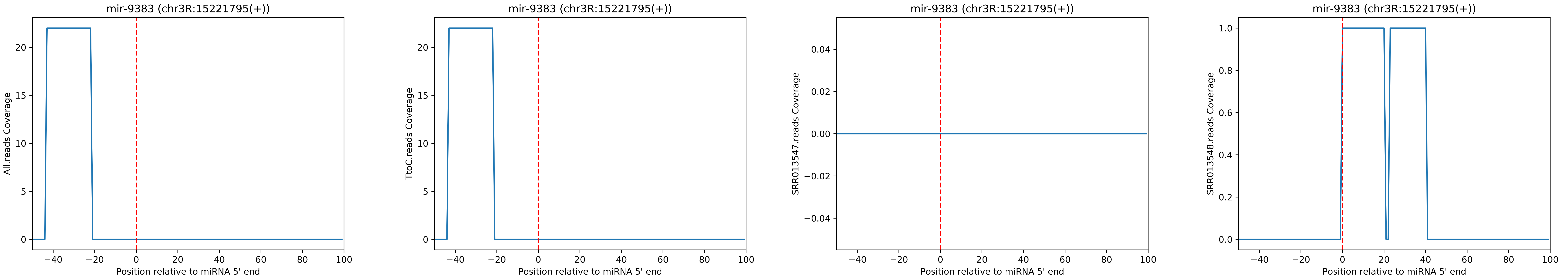

mir-9383 (chr3R:15221795(+))
All.reads Coverage
Position relative to miRNA 5' end
mir-9383 (chr3R:15221795(+))
TtoC.reads Coverage
Position relative to miRNA 5' end
mir-9383 (chr3R:15221795(+))
SRR013547.reads Coverage
Position relative to miRNA 5' end
mir-9383 (chr3R:15221795(+))
SRR013548.reads Coverage
Position relative to miRNA 5' end

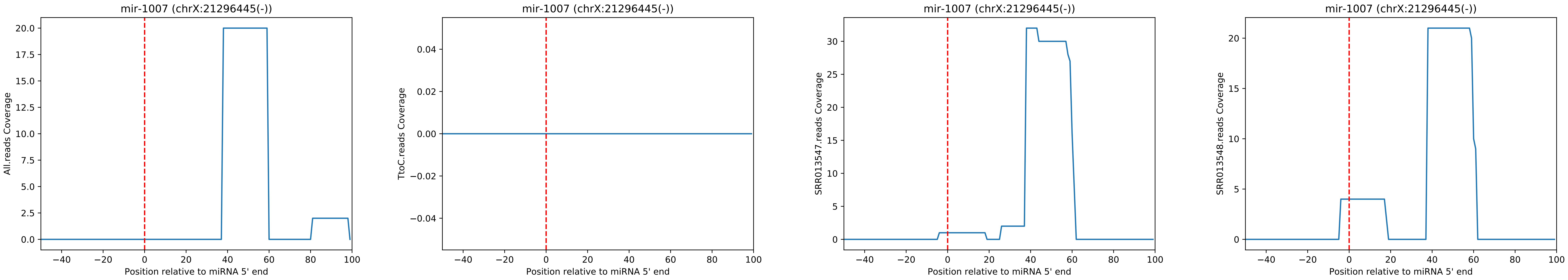
mir-1007 (chrX:21296445(-))
All.reads Coverage
Position relative to miRNA 5' end
mir-1007 (chrX:21296445(-))
TtoC.reads Coverage
Position relative to miRNA 5' end
mir-1007 (chrX:21296445(-))
SRR013547.reads Coverage
Position relative to miRNA 5' end
mir-1007 (chrX:21296445(-))
SRR013548.reads Coverage
Position relative to miRNA 5' end

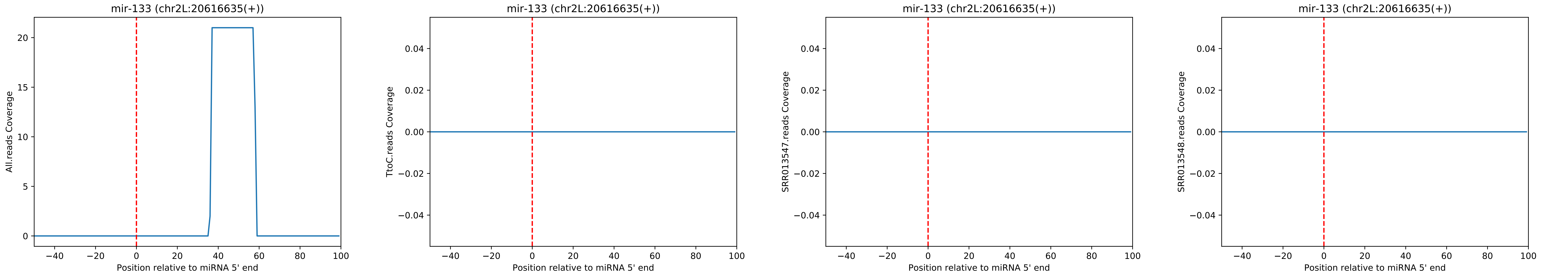

mir-133 (chr2L:20616635(+))
All.reads Coverage
Position relative to miRNA 5' end
mir-133 (chr2L:20616635(+))
TtoC.reads Coverage
Position relative to miRNA 5' end
mir-133 (chr2L:20616635(+))
SRR013547.reads Coverage
Position relative to miRNA 5' end
mir-133 (chr2L:20616635(+))
SRR013548.reads Coverage
Position relative to miRNA 5' end

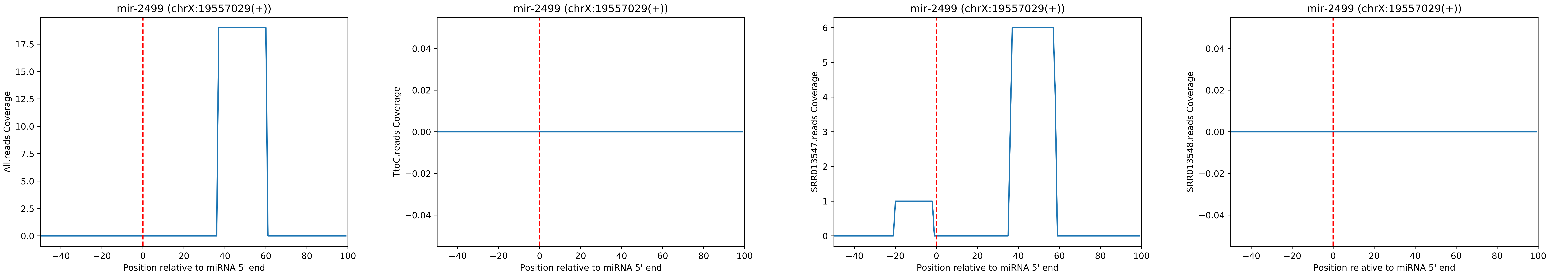

mir-2499 (chrX:19557029(+))
All.reads Coverage
TtoC.reads Coverage
SRR013547.reads Coverage
SRR013548.reads Coverage
Position relative to miRNA 5' end

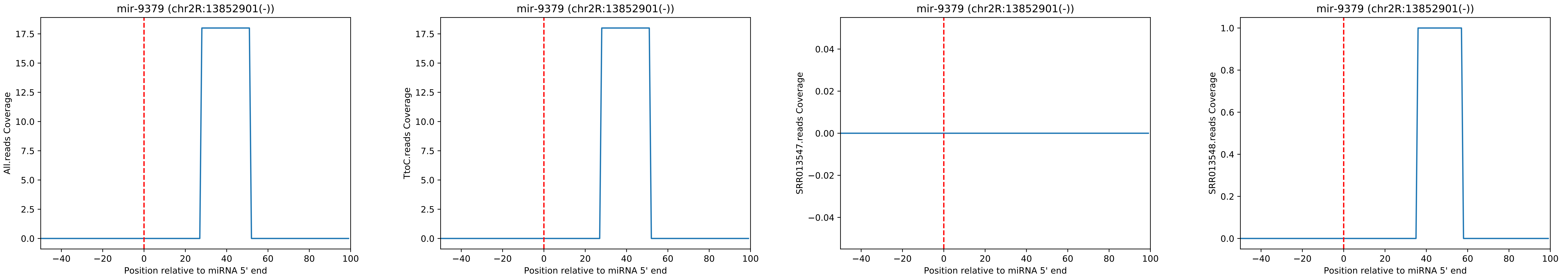
mir-9379 (chr2R:13852901(-))
All.reads Coverage
Position relative to miRNA 5' end
mir-9379 (chr2R:13852901(-))
TtoC.reads Coverage
Position relative to miRNA 5' end
mir-9379 (chr2R:13852901(-))
SRR013547.reads Coverage
Position relative to miRNA 5' end
mir-9379 (chr2R:13852901(-))
SRR013548.reads Coverage
Position relative to miRNA 5' end

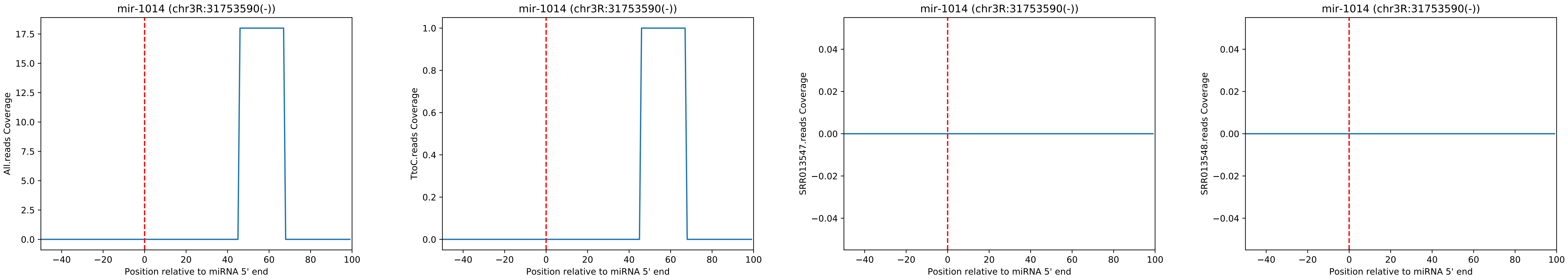
mir-1014 (chr3R:31753590(-))
All.reads Coverage
TtoC.reads Coverage
SRR013547.reads Coverage
SRR013548.reads Coverage
Position relative to miRNA 5' end

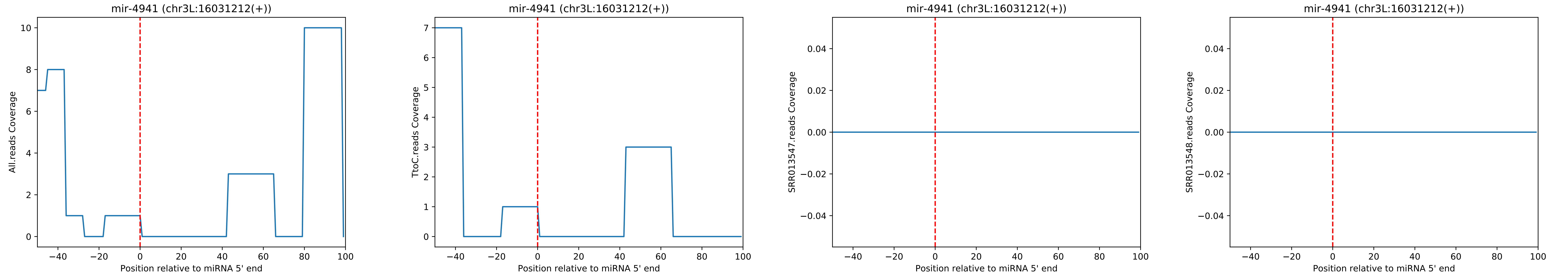
mir-4941 (chr3L:16031212(+))
All.reads Coverage
Position relative to miRNA 5' end
mir-4941 (chr3L:16031212(+))
TtoC.reads Coverage
Position relative to miRNA 5' end
mir-4941 (chr3L:16031212(+))
SRR013547.reads Coverage
Position relative to miRNA 5' end
mir-4941 (chr3L:16031212(+))
SRR013548.reads Coverage
Position relative to miRNA 5' end

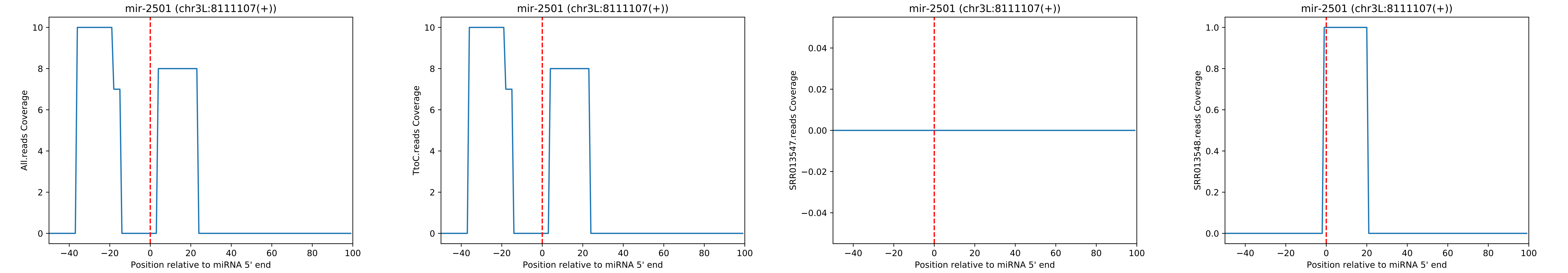
mir-2501 (chr3L:8111107(+))
All.reads Coverage
Position relative to miRNA 5' end
mir-2501 (chr3L:8111107(+))
TtoC.reads Coverage
Position relative to miRNA 5' end
mir-2501 (chr3L:8111107(+))
SRR013547.reads Coverage
Position relative to miRNA 5' end
mir-2501 (chr3L:8111107(+))
SRR013548.reads Coverage
Position relative to miRNA 5' end

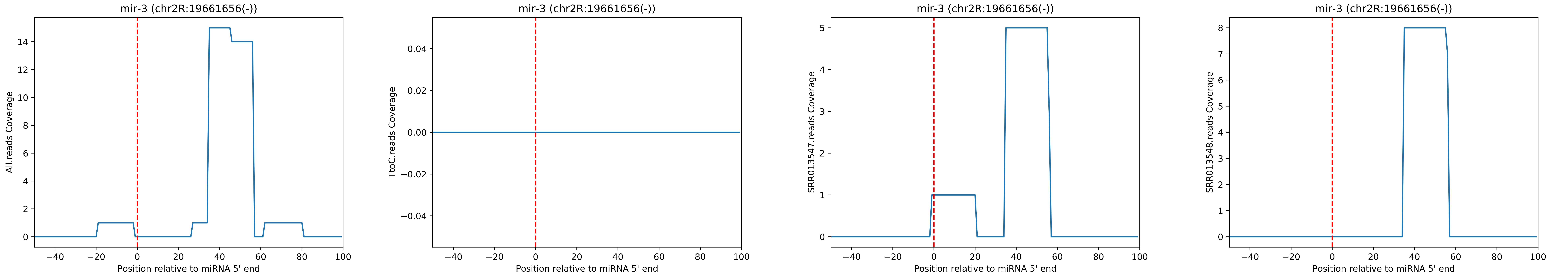

mir-3 (chr2R:19661656(-))
All.reads Coverage
TtoC.reads Coverage
SRR013547.reads Coverage
SRR013548.reads Coverage
Position relative to miRNA 5' end

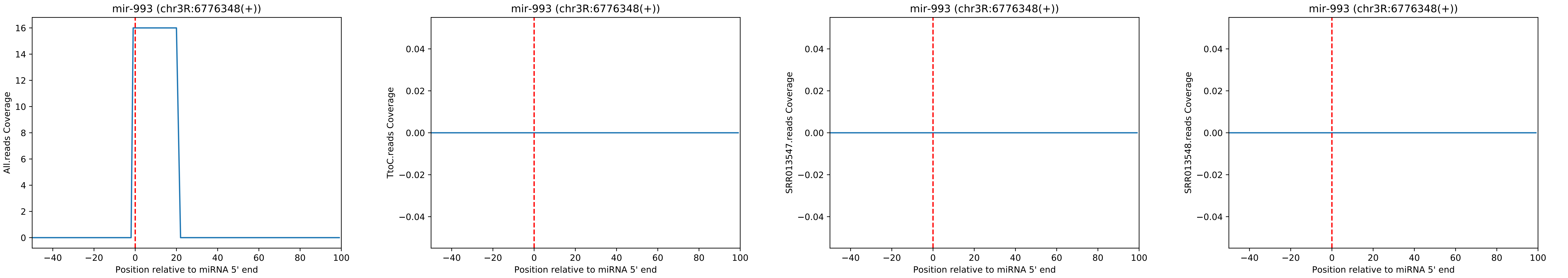
mir-993 (chr3R:6776348(+))
All.reads Coverage
TtoC.reads Coverage
SRR013547.reads Coverage
SRR013548.reads Coverage
Position relative to miRNA 5' end

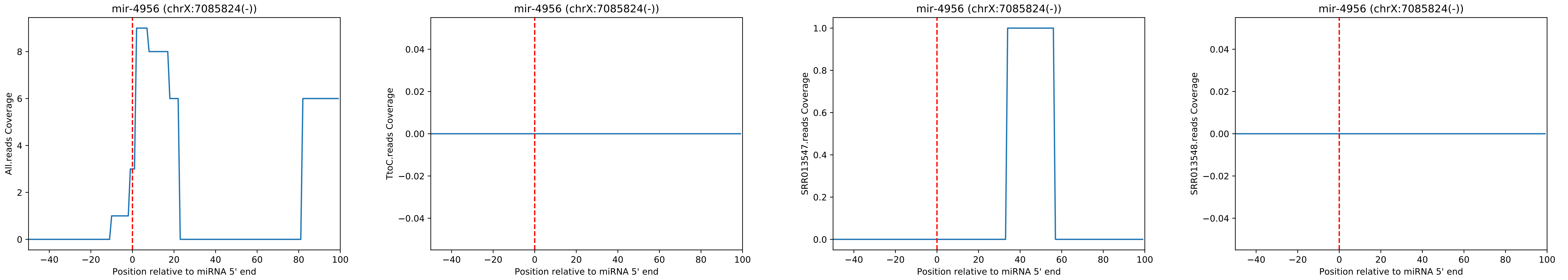

mir-4956 (chrX:7085824(-))
All.reads Coverage
Position relative to miRNA 5' end
mir-4956 (chrX:7085824(-))
TtoC.reads Coverage
Position relative to miRNA 5' end
mir-4956 (chrX:7085824(-))
SRR013547.reads Coverage
Position relative to miRNA 5' end
mir-4956 (chrX:7085824(-))
SRR013548.reads Coverage
Position relative to miRNA 5' end

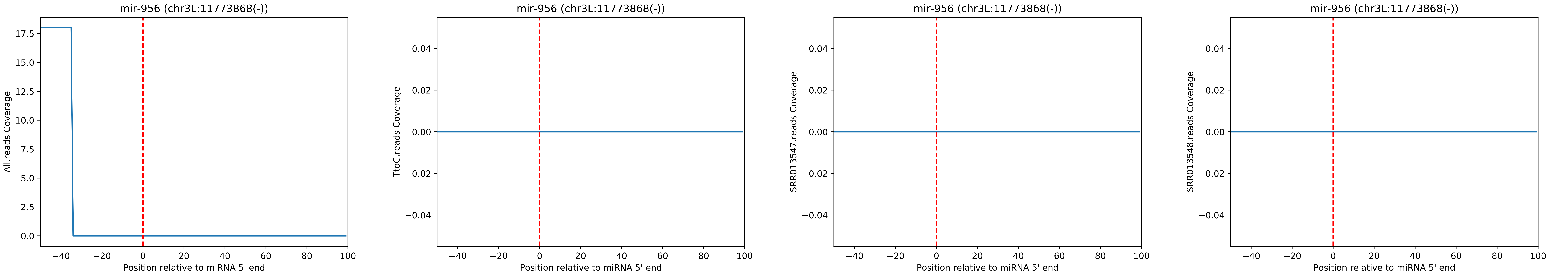
mir-956 (chr3L:11773868(-))
All.reads Coverage
TtoC.reads Coverage
SRR013547.reads Coverage
SRR013548.reads Coverage
Position relative to miRNA 5' end

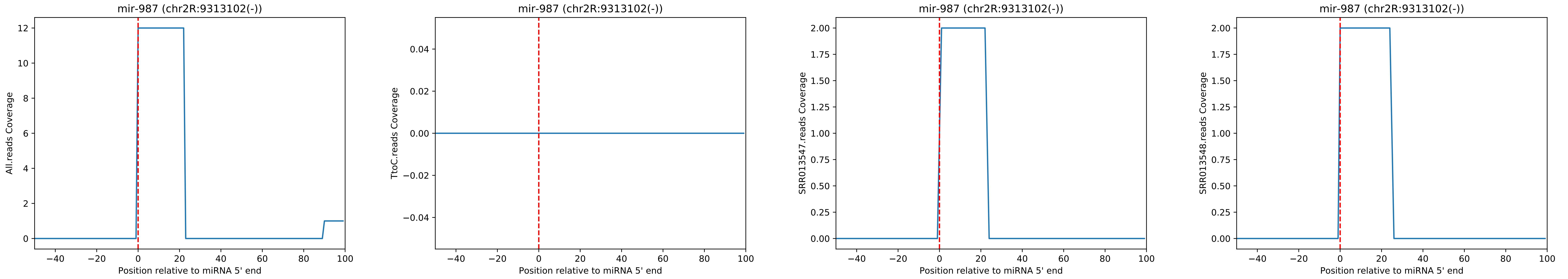
mir-987 (chr2R:9313102(-))
All.reads Coverage
Position relative to miRNA 5' end
mir-987 (chr2R:9313102(-))
TtoC.reads Coverage
Position relative to miRNA 5' end
mir-987 (chr2R:9313102(-))
SRR013547.reads Coverage
Position relative to miRNA 5' end
mir-987 (chr2R:9313102(-))
SRR013548.reads Coverage
Position relative to miRNA 5' end

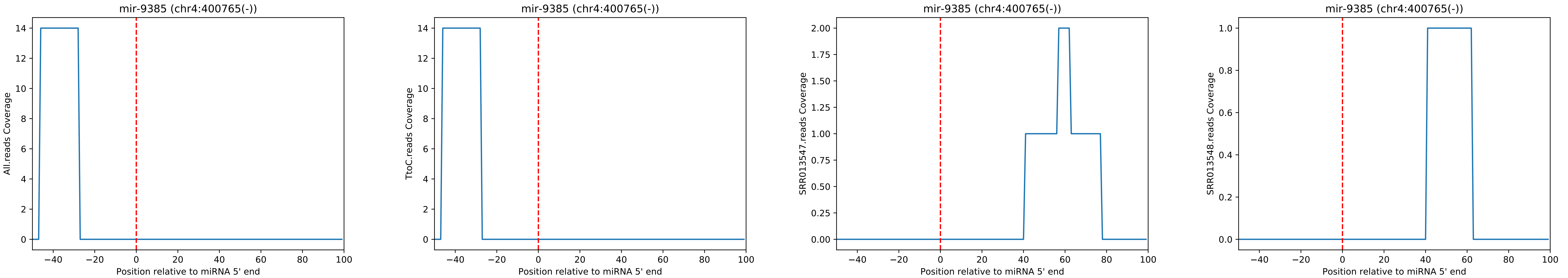
mir-9385 (chr4:400765(-))
All.reads Coverage
Position relative to miRNA 5' end
mir-9385 (chr4:400765(-))
TtoC.reads Coverage
Position relative to miRNA 5' end
mir-9385 (chr4:400765(-))
SRR013547.reads Coverage
Position relative to miRNA 5' end
mir-9385 (chr4:400765(-))
SRR013548.reads Coverage
Position relative to miRNA 5' end

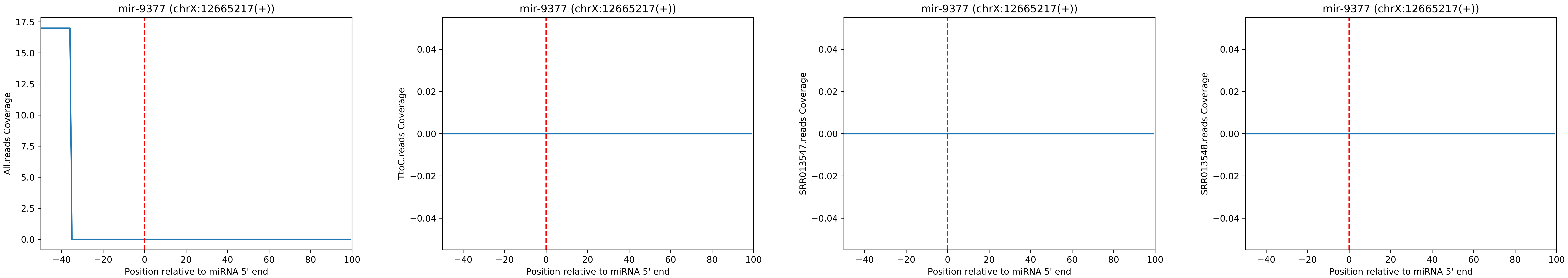
mir-9377 (chrX:12665217(+))
All.reads Coverage
TtoC.reads Coverage
SRR013547.reads Coverage
SRR013548.reads Coverage
Position relative to miRNA 5' end

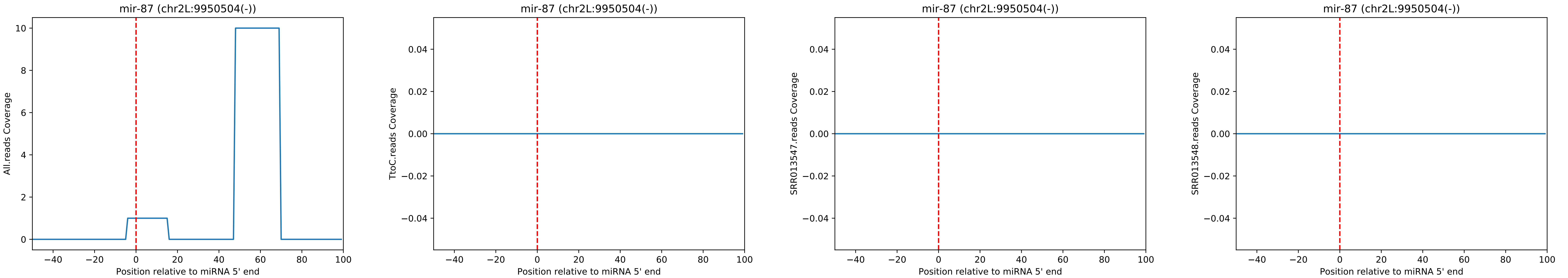

mir-87 (chr2L:9950504(-))
All.reads Coverage
TtoC.reads Coverage
SRR013547.reads Coverage
SRR013548.reads Coverage
Position relative to miRNA 5' end

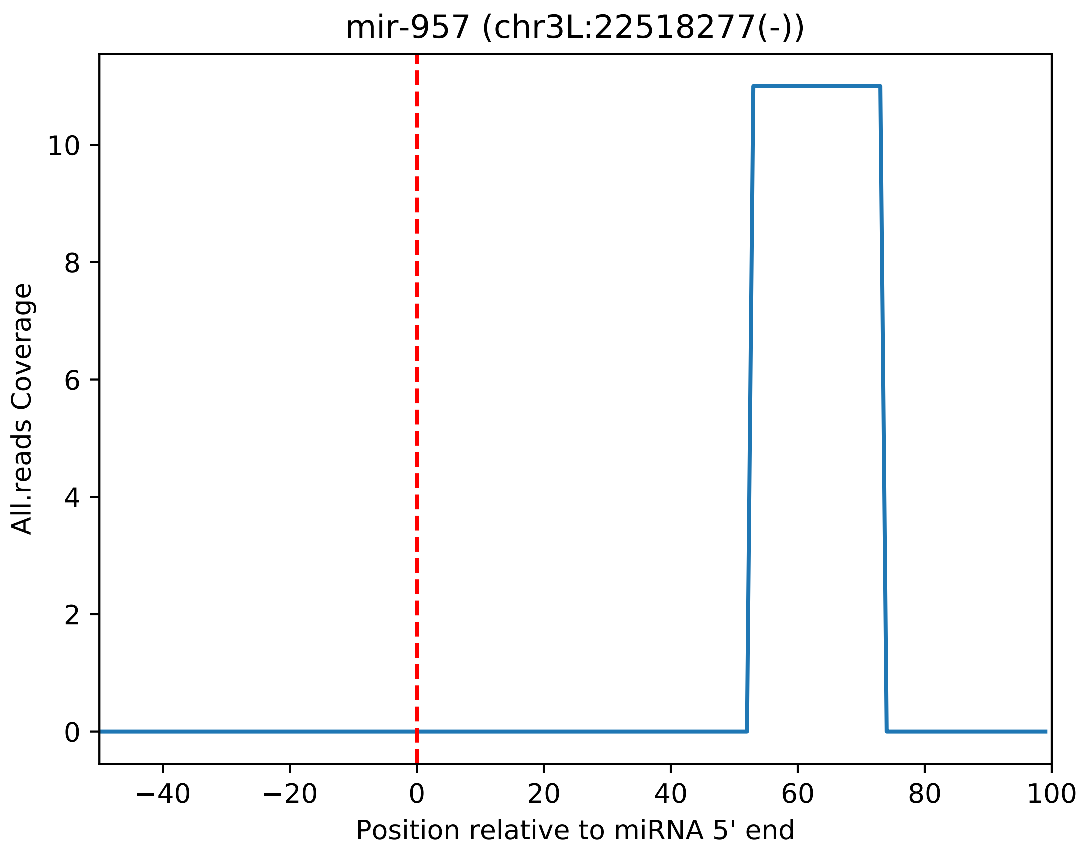

mir-957 (chr3L:22518277(-))
All.reads Coverage
Position relative to miRNA 5' end

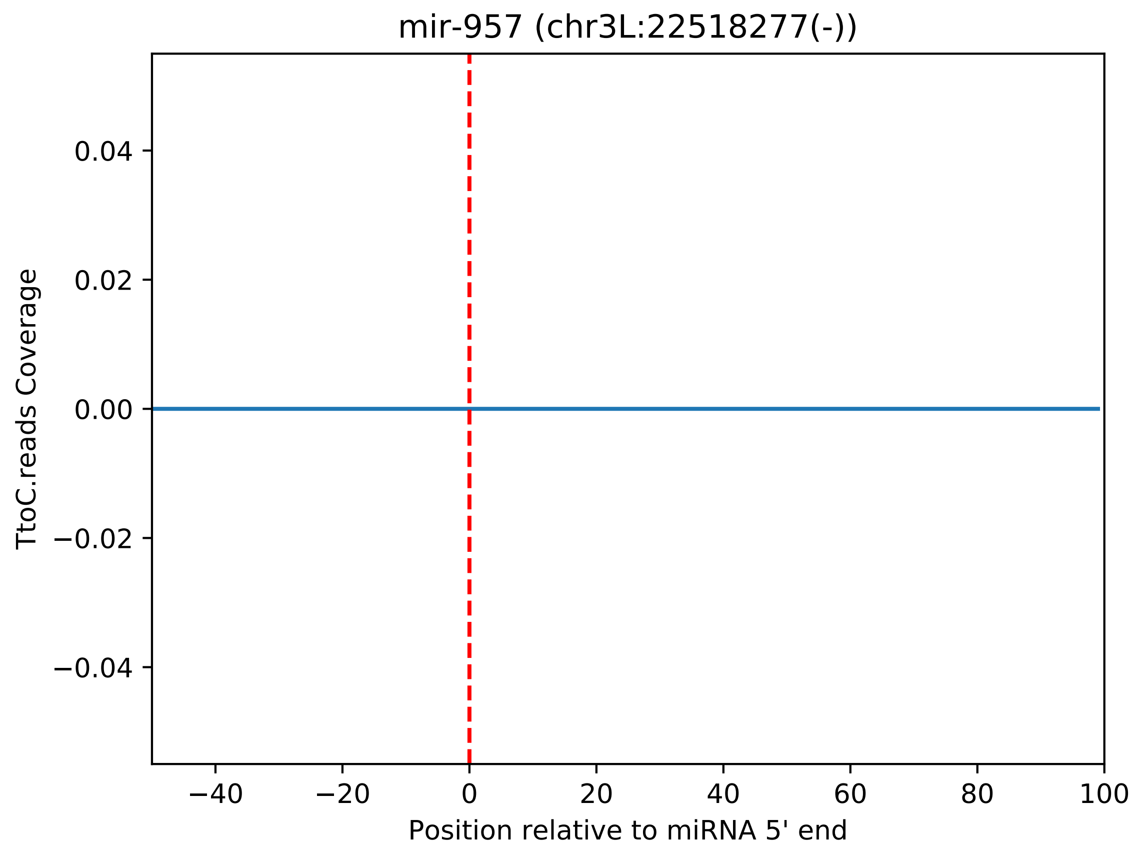

mir-957 (chr3L:22518277(-))
TtoC.reads Coverage
Position relative to miRNA 5' end

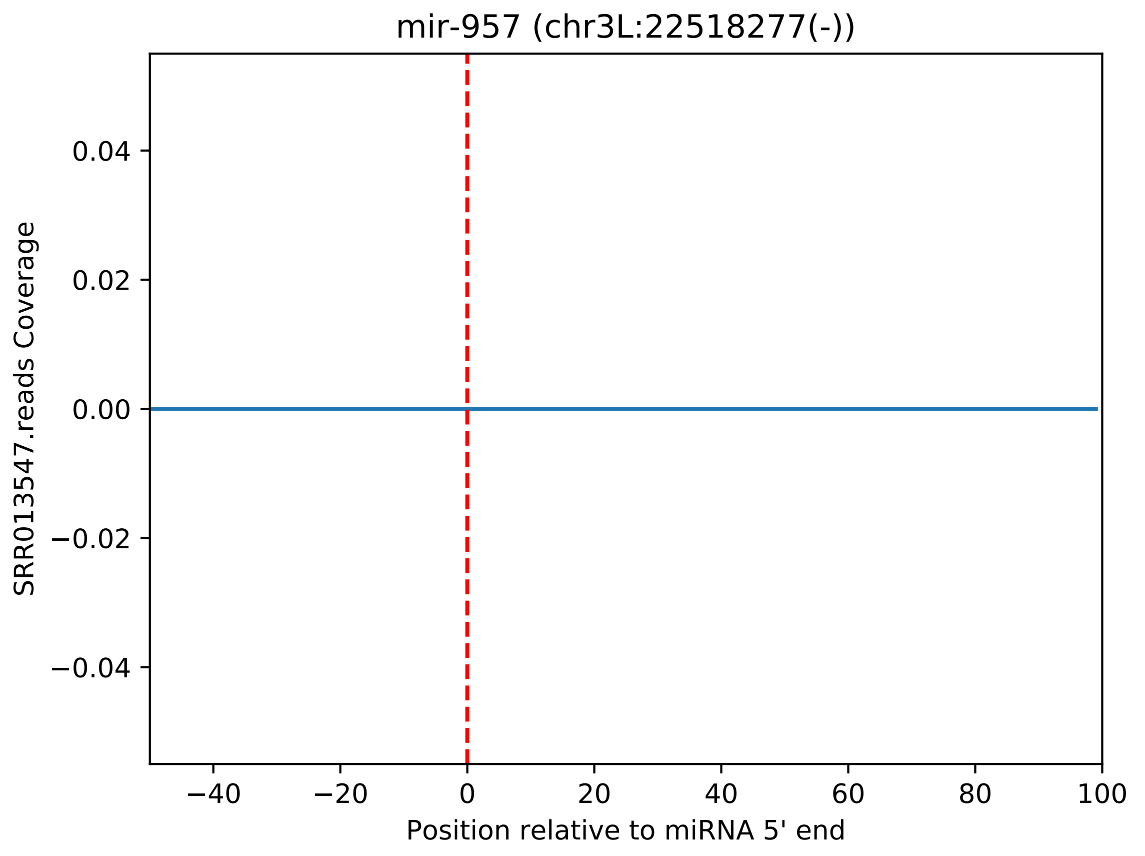

mir-957 (chr3L:22518277(-))
SRR013547.reads Coverage
Position relative to miRNA 5' end

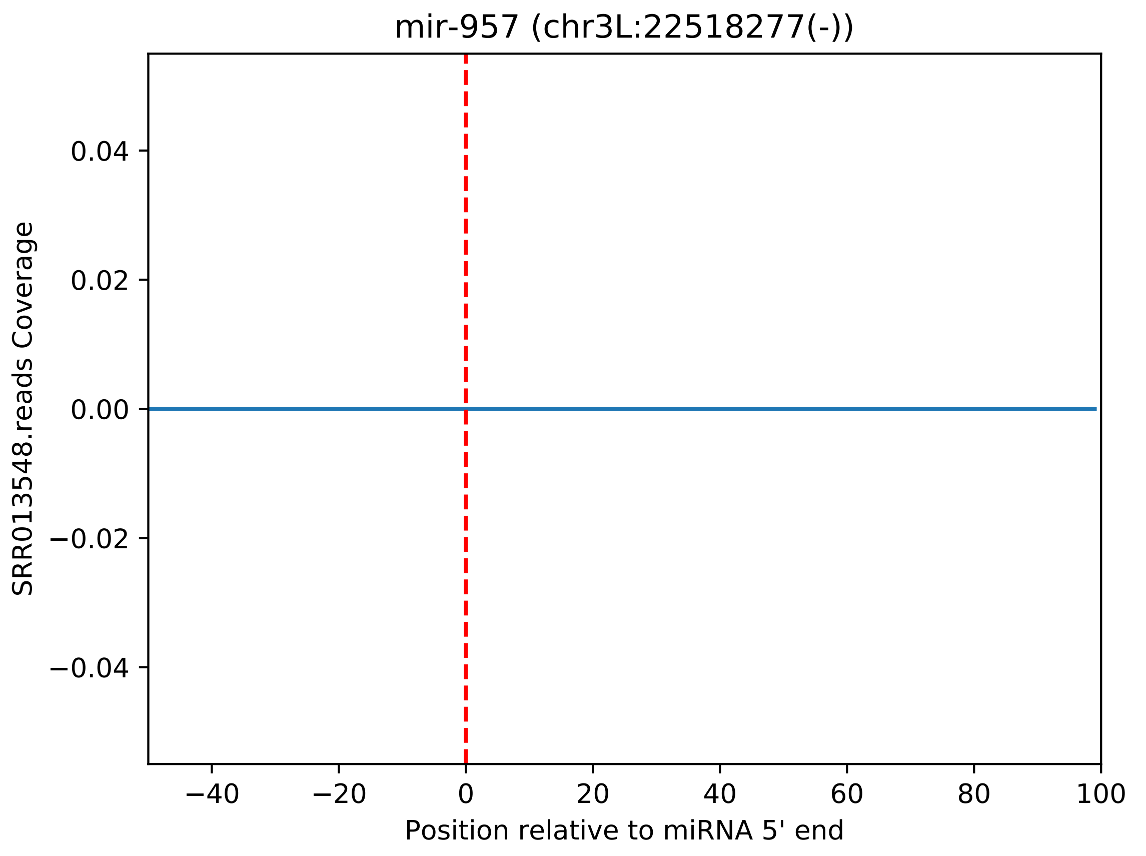

mir-957 (chr3L:22518277(-))
SRR013548.reads Coverage
Position relative to miRNA 5' end

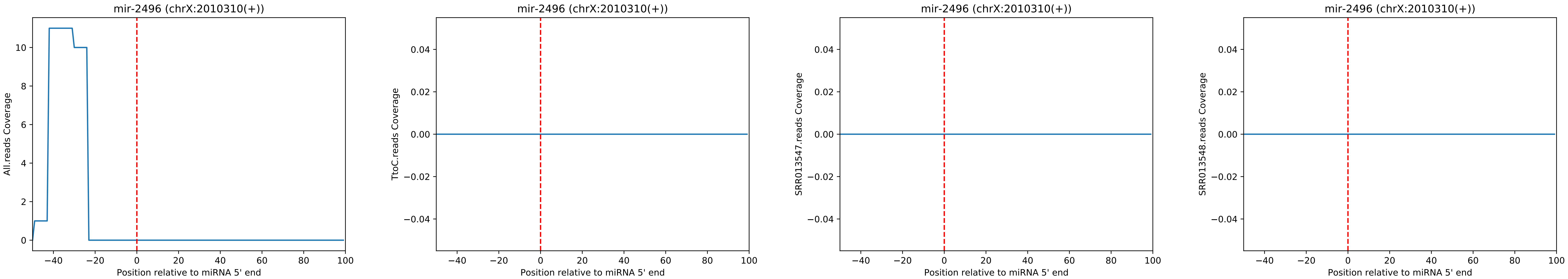

mir-2496 (chrX:2010310(+))
All.reads Coverage
Position relative to miRNA 5' end
mir-2496 (chrX:2010310(+))
TtoC.reads Coverage
Position relative to miRNA 5' end
mir-2496 (chrX:2010310(+))
SRR013547.reads Coverage
Position relative to miRNA 5' end
mir-2496 (chrX:2010310(+))
SRR013548.reads Coverage
Position relative to miRNA 5' end

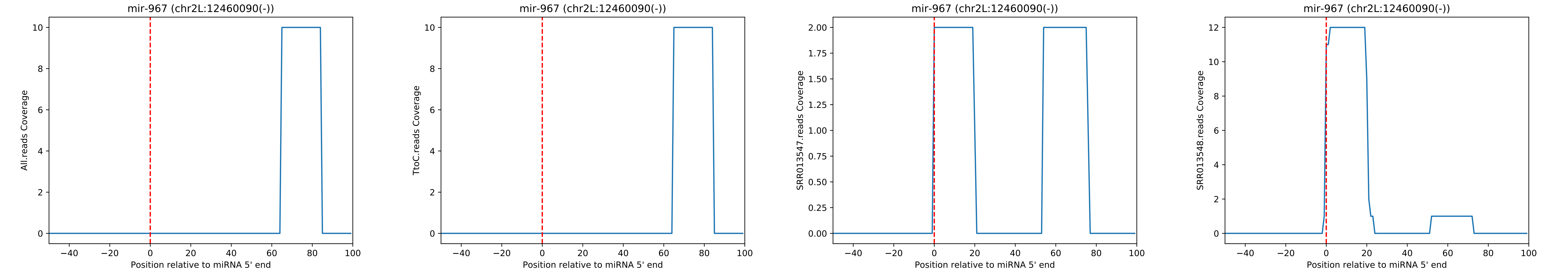
mir-967 (chr2L:12460090(-))
All.reads Coverage
Position relative to miRNA 5' end
mir-967 (chr2L:12460090(-))
TtoC.reads Coverage
Position relative to miRNA 5' end
mir-967 (chr2L:12460090(-))
SRR013547.reads Coverage
Position relative to miRNA 5' end
mir-967 (chr2L:12460090(-))
SRR013548.reads Coverage
Position relative to miRNA 5' end
−40
−20
0
20
40
60
80
100
0
2
4
6
8
10
12
0.00
0.25
0.50
0.75
1.00
1.25
1.50
1.75
2.00

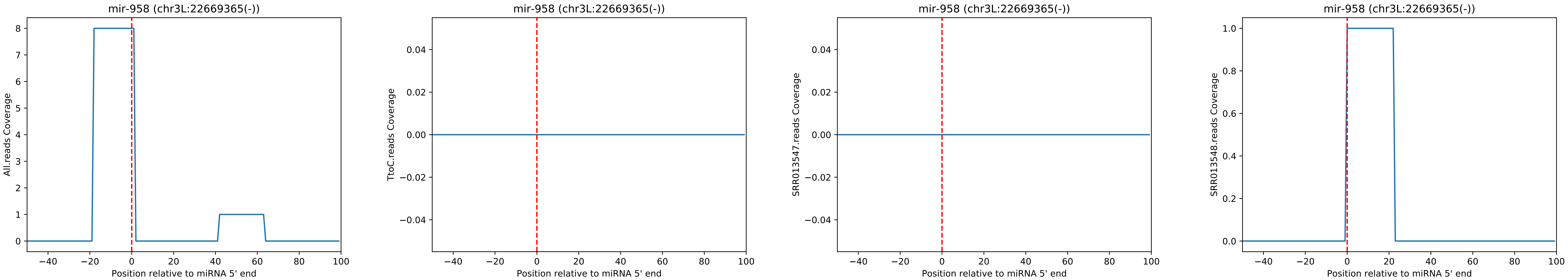

mir-958 (chr3L:22669365(-))
All.reads Coverage
Position relative to miRNA 5' end
mir-958 (chr3L:22669365(-))
TtoC.reads Coverage
Position relative to miRNA 5' end
mir-958 (chr3L:22669365(-))
SRR013547.reads Coverage
Position relative to miRNA 5' end
mir-958 (chr3L:22669365(-))
SRR013548.reads Coverage
Position relative to miRNA 5' end

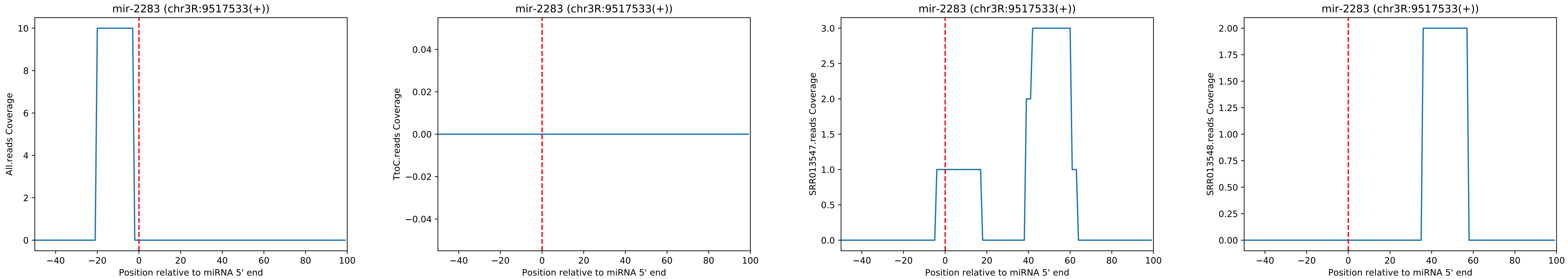
mir-2283 (chr3R:9517533(+))
All.reads Coverage
TtoC.reads Coverage
SRR013547.reads Coverage
SRR013548.reads Coverage
Position relative to miRNA 5' end

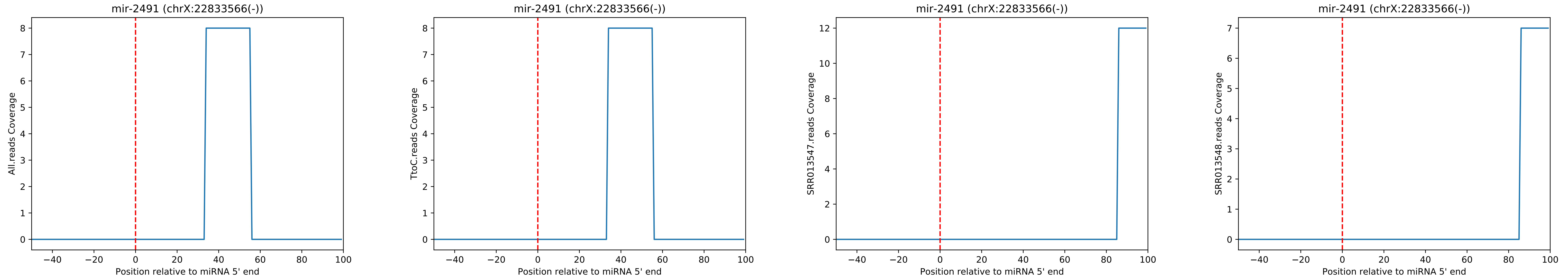

mir-2491 (chrX:22833566(-))
All.reads Coverage
Position relative to miRNA 5' end
mir-2491 (chrX:22833566(-))
TtoC.reads Coverage
Position relative to miRNA 5' end
mir-2491 (chrX:22833566(-))
SRR013547.reads Coverage
Position relative to miRNA 5' end
mir-2491 (chrX:22833566(-))
SRR013548.reads Coverage
Position relative to miRNA 5' end

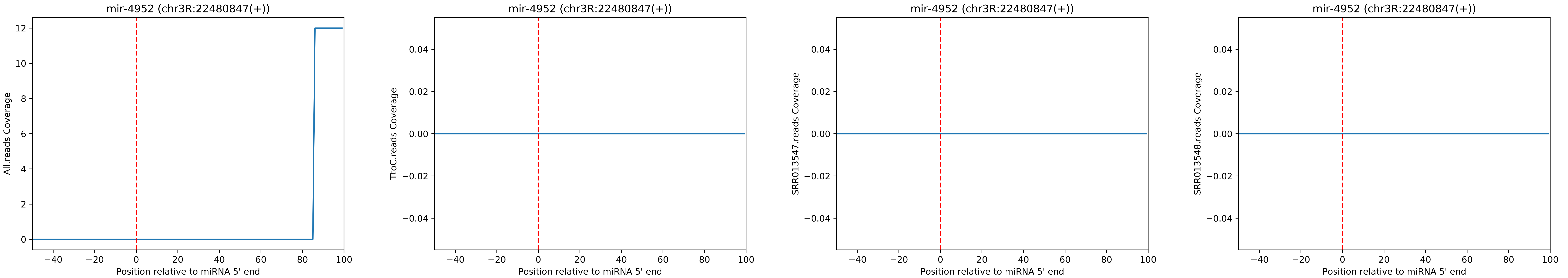
mir-4952 (chr3R:22480847(+))
All.reads Coverage
Position relative to miRNA 5' end
mir-4952 (chr3R:22480847(+))
TtoC.reads Coverage
Position relative to miRNA 5' end
mir-4952 (chr3R:22480847(+))
SRR013547.reads Coverage
Position relative to miRNA 5' end
mir-4952 (chr3R:22480847(+))
SRR013548.reads Coverage
Position relative to miRNA 5' end

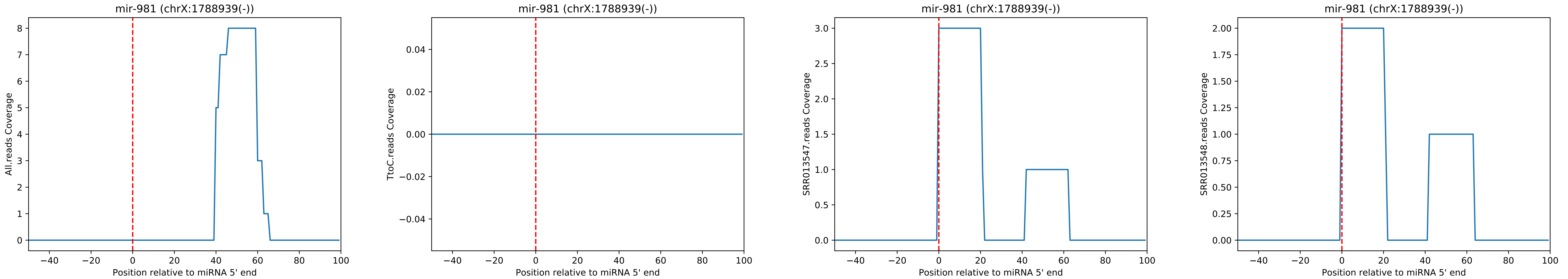
mir-981 (chrX:1788939(-))
All.reads Coverage
Position relative to miRNA 5' end
mir-981 (chrX:1788939(-))
TtoC.reads Coverage
Position relative to miRNA 5' end
mir-981 (chrX:1788939(-))
SRR013547.reads Coverage
Position relative to miRNA 5' end
mir-981 (chrX:1788939(-))
SRR013548.reads Coverage
Position relative to miRNA 5' end

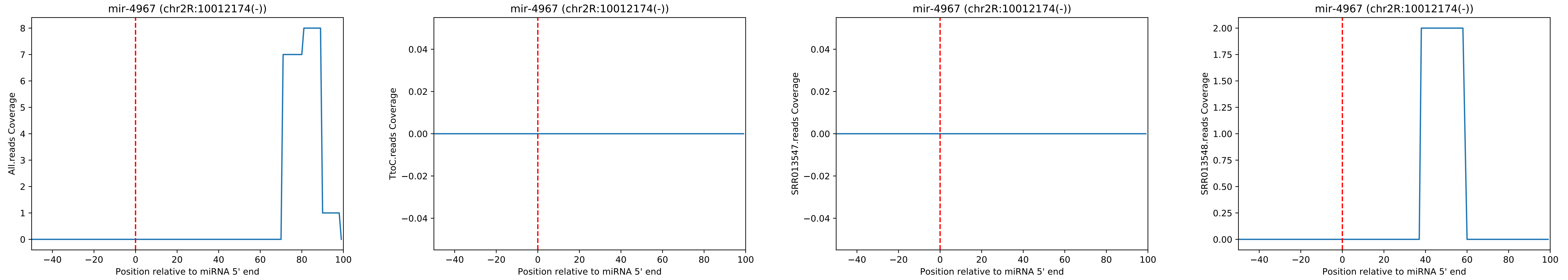
mir-4967 (chr2R:10012174(-))
All.reads Coverage
Position relative to miRNA 5' end
mir-4967 (chr2R:10012174(-))
TtoC.reads Coverage
Position relative to miRNA 5' end
mir-4967 (chr2R:10012174(-))
SRR013547.reads Coverage
Position relative to miRNA 5' end
mir-4967 (chr2R:10012174(-))
SRR013548.reads Coverage
Position relative to miRNA 5' end

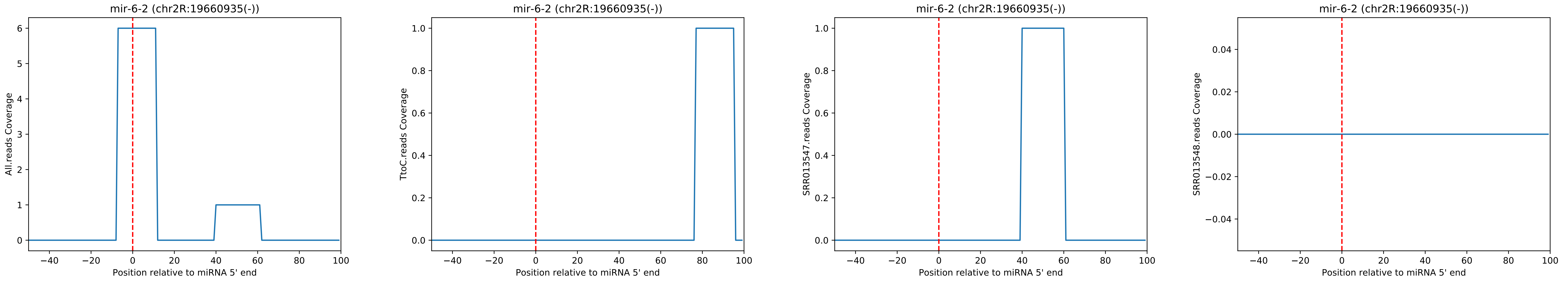
mir-6-2 (chr2R:19660935(-))
All.reads Coverage
Position relative to miRNA 5' end
mir-6-2 (chr2R:19660935(-))
TtoC.reads Coverage
Position relative to miRNA 5' end
mir-6-2 (chr2R:19660935(-))
SRR013547.reads Coverage
Position relative to miRNA 5' end
mir-6-2 (chr2R:19660935(-))
SRR013548.reads Coverage
Position relative to miRNA 5' end

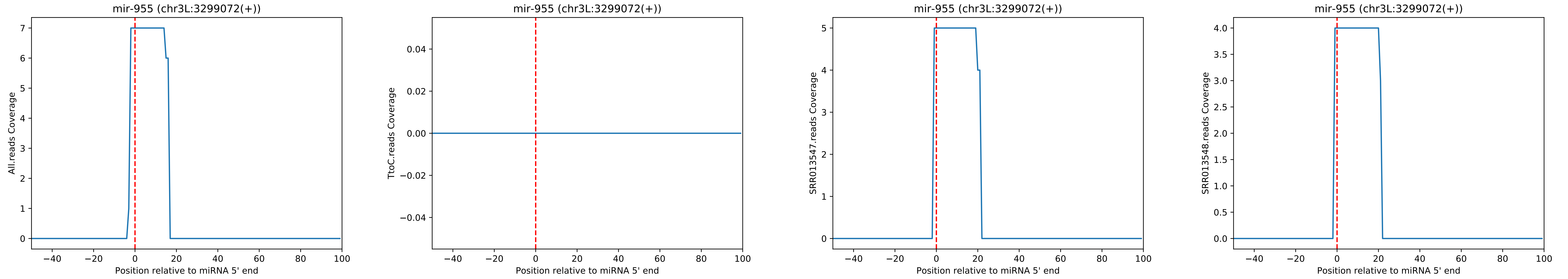
mir-955 (chr3L:3299072(+))
All.reads Coverage
TtoC.reads Coverage
SRR013547.reads Coverage
SRR013548.reads Coverage
Position relative to miRNA 5' end

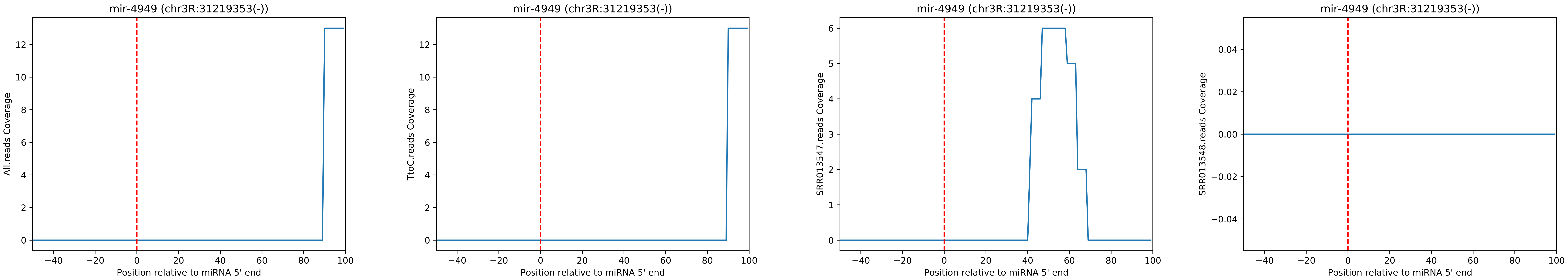
mir-4949 (chr3R:31219353(-))
All.reads Coverage
Position relative to miRNA 5' end
mir-4949 (chr3R:31219353(-))
TtoC.reads Coverage
Position relative to miRNA 5' end
mir-4949 (chr3R:31219353(-))
SRR013547.reads Coverage
Position relative to miRNA 5' end
mir-4949 (chr3R:31219353(-))
SRR013548.reads Coverage
Position relative to miRNA 5' end

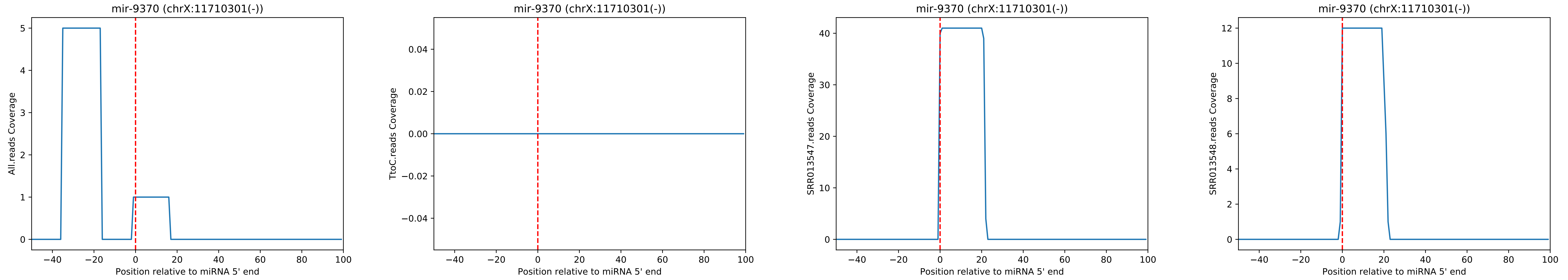

mir-9370 (chrX:11710301(-))
All.reads Coverage
Position relative to miRNA 5' end
mir-9370 (chrX:11710301(-))
TtoC.reads Coverage
Position relative to miRNA 5' end
mir-9370 (chrX:11710301(-))
SRR013547.reads Coverage
Position relative to miRNA 5' end
mir-9370 (chrX:11710301(-))
SRR013548.reads Coverage
Position relative to miRNA 5' end

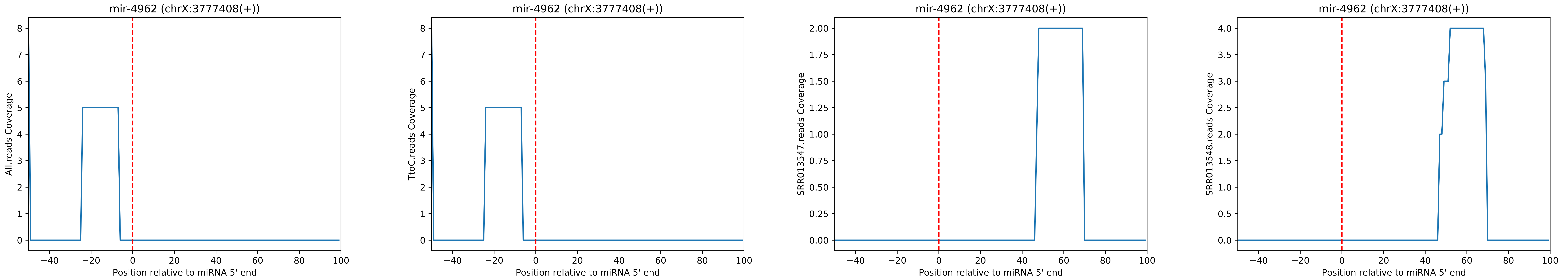
mir-4962 (chrX:3777408(+))
All.reads Coverage
0
1
2
3
4
5
6
7
8
−40
−20
0
20
40
60
80
100
Position relative to miRNA 5' end
mir-4962 (chrX:3777408(+))
TtoC.reads Coverage
0
1
2
3
4
5
6
7
8
−40
−20
0
20
40
60
80
100
Position relative to miRNA 5' end
mir-4962 (chrX:3777408(+))
SRR013547.reads Coverage
0.00
0.25
0.50
0.75
1.00
1.25
1.50
1.75
2.00
−40
−20
0
20
40
60
80
100
Position relative to miRNA 5' end
mir-4962 (chrX:3777408(+))
SRR013548.reads Coverage
0.0
0.5
1.0
1.5
2.0
2.5
3.0
3.5
4.0
−40
−20
0
20
40
60
80
100
Position relative to miRNA 5' end

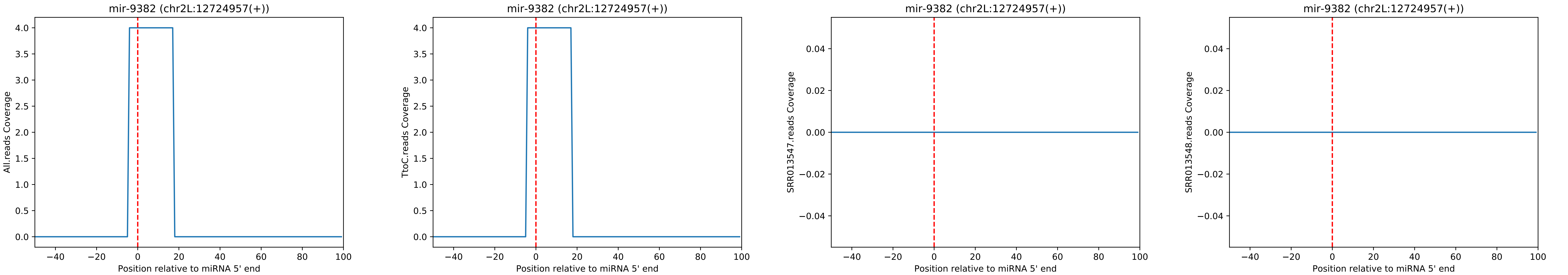
mir-9382 (chr2L:12724957(+))
All.reads Coverage
Position relative to miRNA 5' end
mir-9382 (chr2L:12724957(+))
TtoC.reads Coverage
Position relative to miRNA 5' end
mir-9382 (chr2L:12724957(+))
SRR013547.reads Coverage
Position relative to miRNA 5' end
mir-9382 (chr2L:12724957(+))
SRR013548.reads Coverage
Position relative to miRNA 5' end

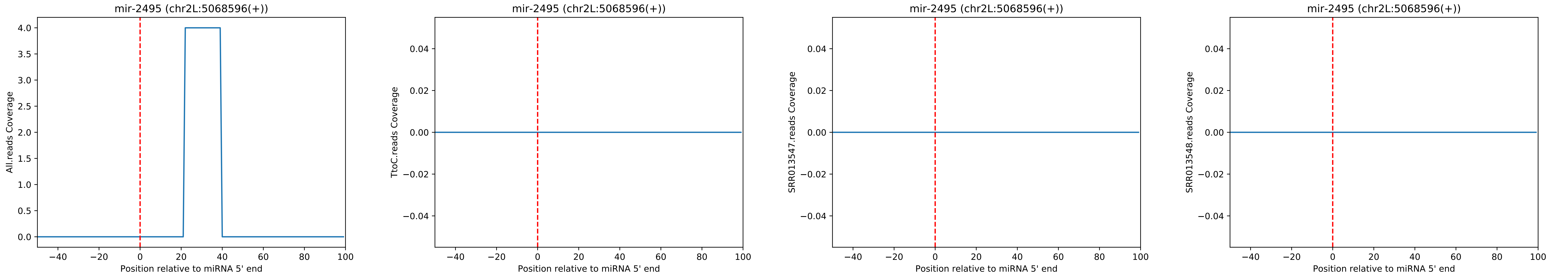
mir-2495 (chr2L:5068596(+))
All.reads Coverage
TtoC.reads Coverage
SRR013547.reads Coverage
SRR013548.reads Coverage
Position relative to miRNA 5' end

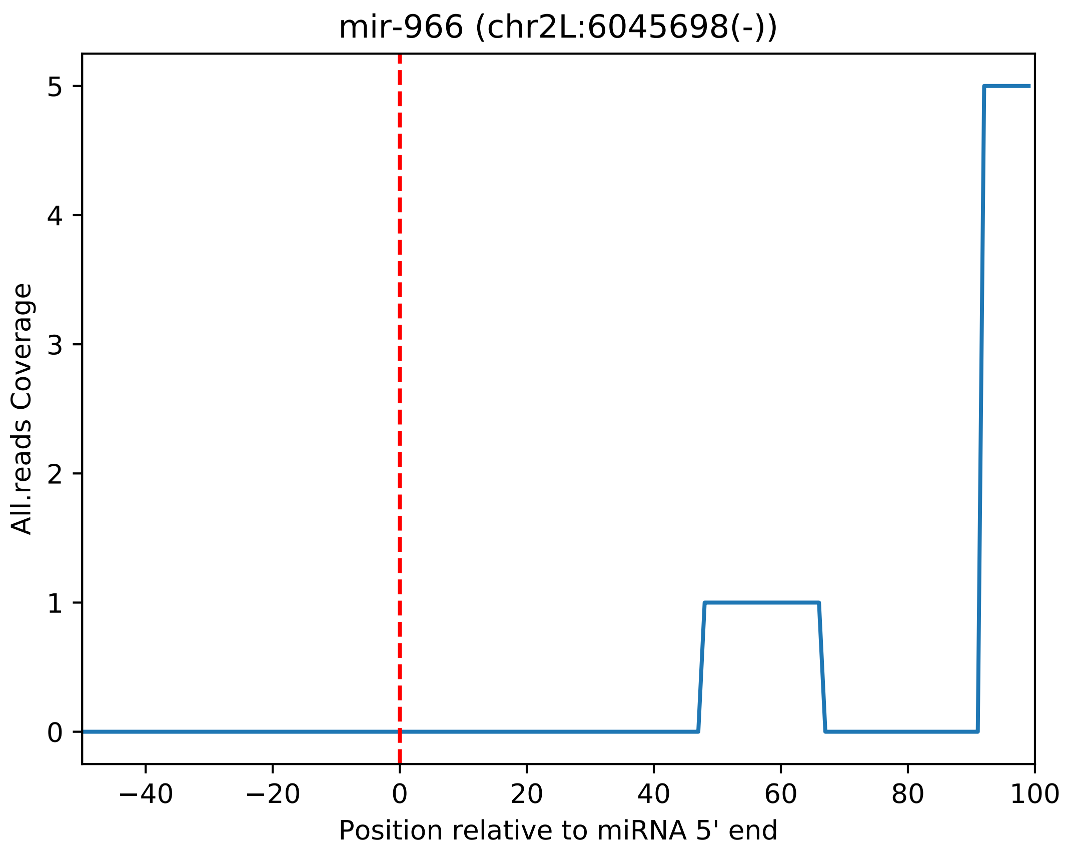
mir-966 (chr2L:6045698(-))
All.reads Coverage
Position relative to miRNA 5' end

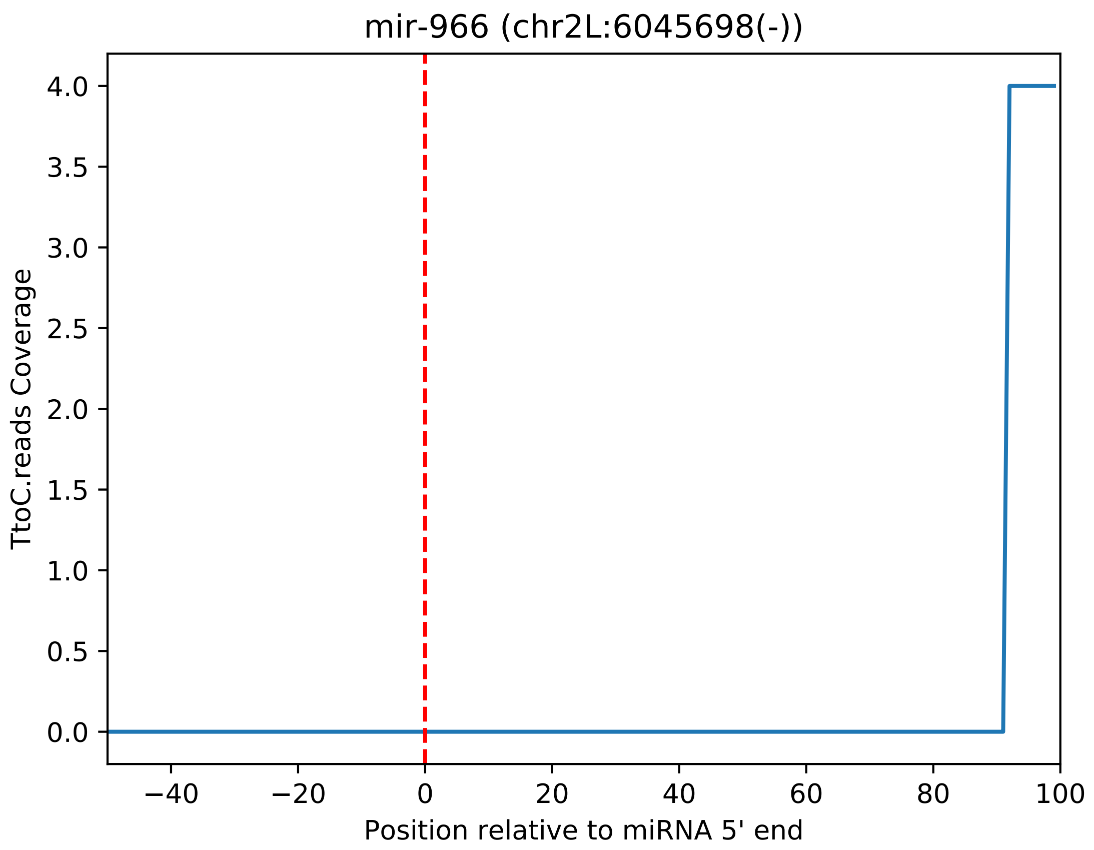
mir-966 (chr2L:6045698(-))
TtoC.reads Coverage
Position relative to miRNA 5' end

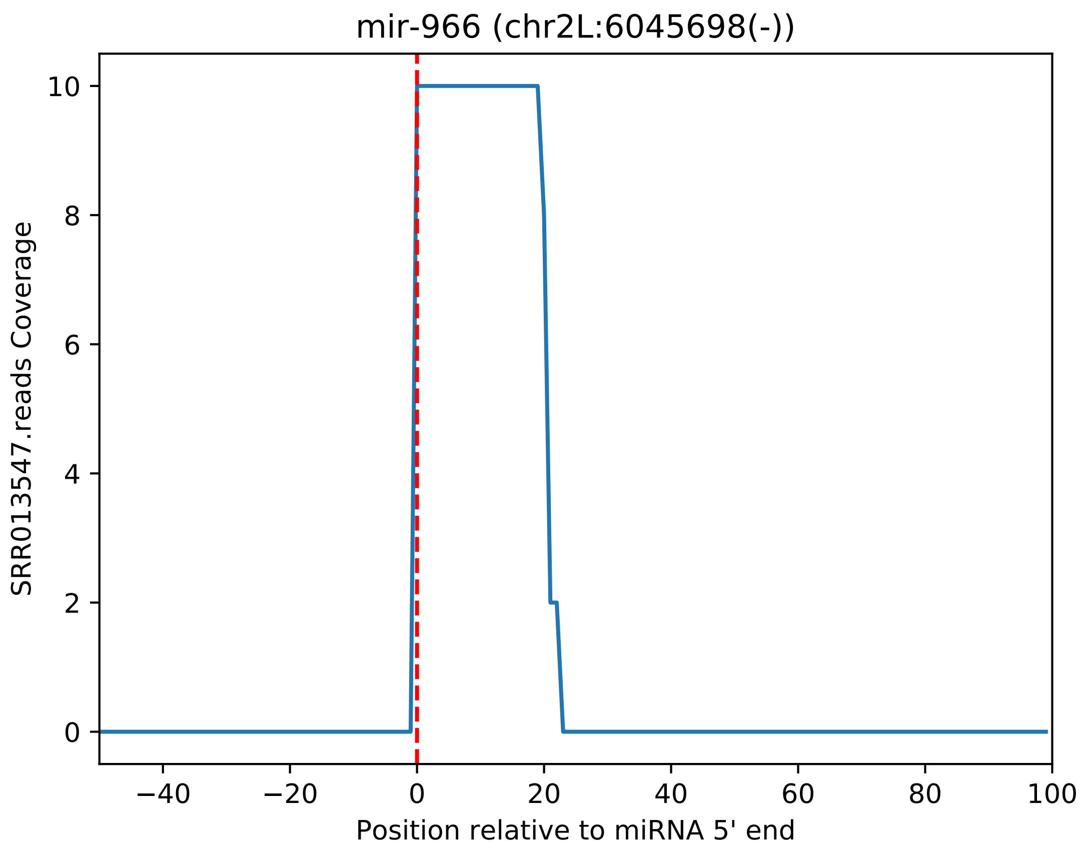
mir-966 (chr2L:6045698(-))
SRR013547.reads Coverage
Position relative to miRNA 5' end

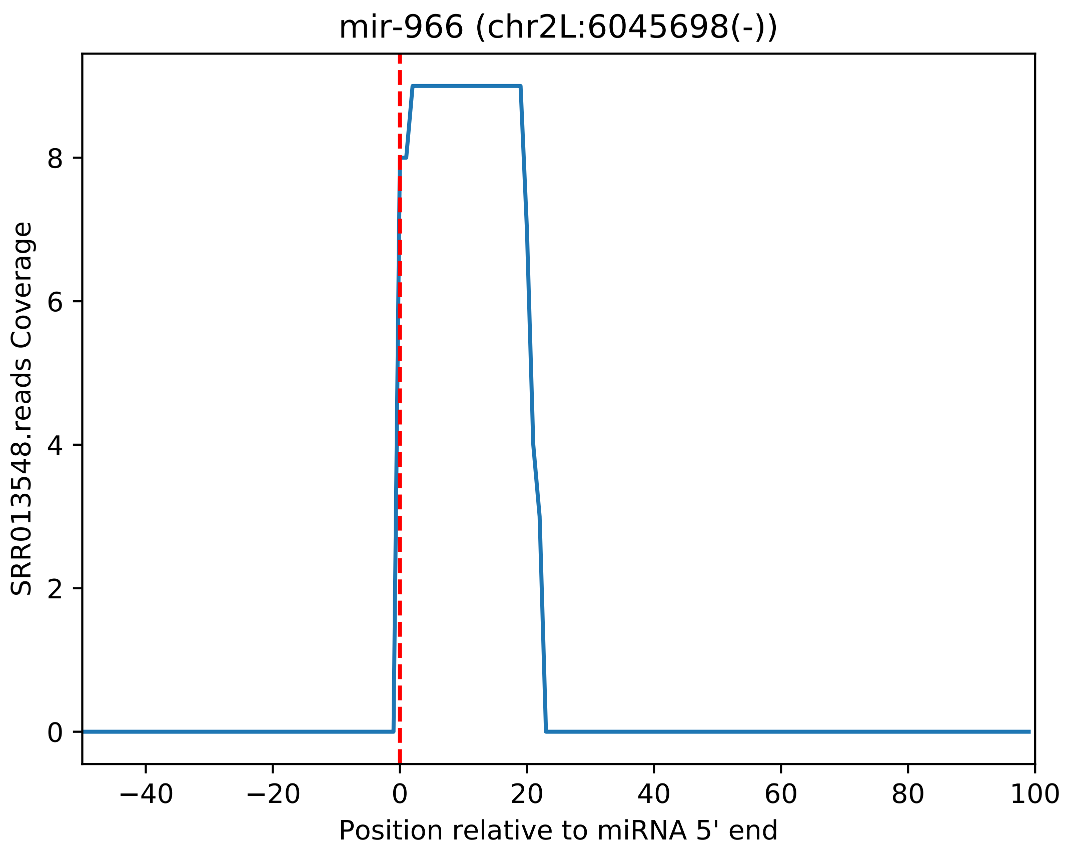
mir-966 (chr2L:6045698(-))
SRR013548.reads Coverage
Position relative to miRNA 5' end

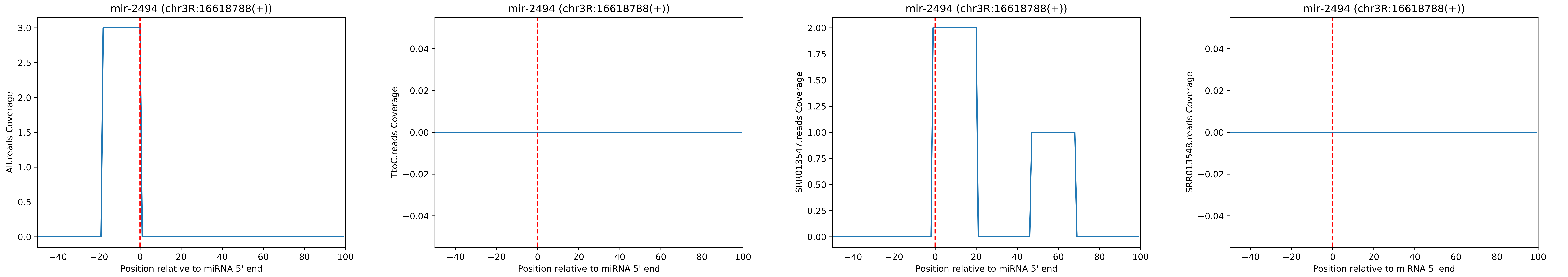

mir-2494 (chr3R:16618788(+))
All.reads Coverage
mir-2494 (chr3R:16618788(+))
TtoC.reads Coverage
mir-2494 (chr3R:16618788(+))
SRR013547.reads Coverage
mir-2494 (chr3R:16618788(+))
SRR013548.reads Coverage
Position relative to miRNA 5' end

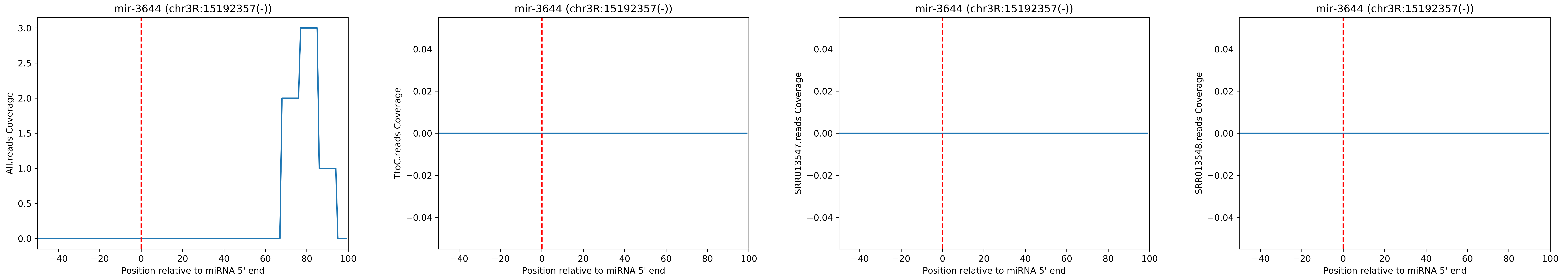

mir-3644 (chr3R:15192357(-))
All.reads Coverage
Position relative to miRNA 5' end
mir-3644 (chr3R:15192357(-))
TtoC.reads Coverage
Position relative to miRNA 5' end
mir-3644 (chr3R:15192357(-))
SRR013547.reads Coverage
Position relative to miRNA 5' end
mir-3644 (chr3R:15192357(-))
SRR013548.reads Coverage
Position relative to miRNA 5' end

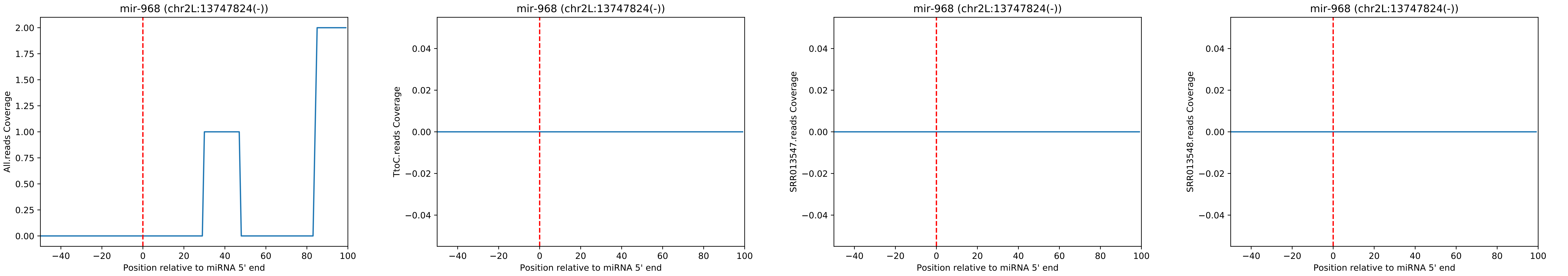

mir-968 (chr2L:13747824(-))
All.reads Coverage
Position relative to miRNA 5' end
mir-968 (chr2L:13747824(-))
TtoC.reads Coverage
Position relative to miRNA 5' end
mir-968 (chr2L:13747824(-))
SRR013547.reads Coverage
Position relative to miRNA 5' end
mir-968 (chr2L:13747824(-))
SRR013548.reads Coverage
Position relative to miRNA 5' end

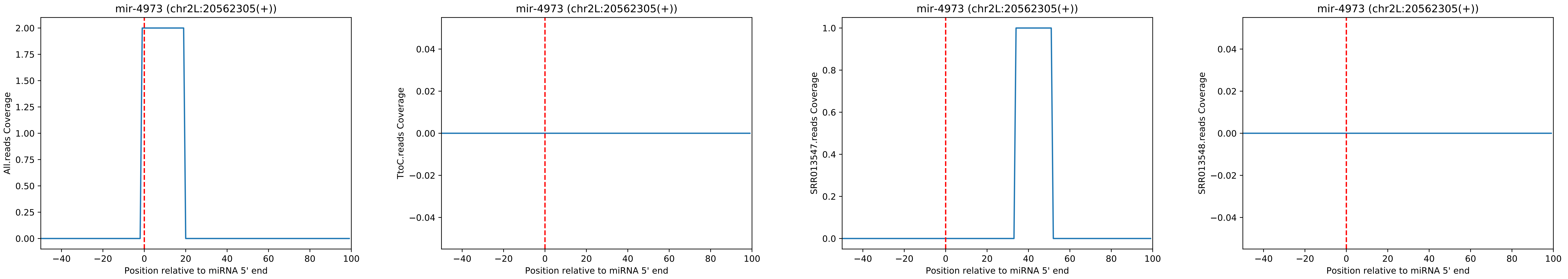

mir-4973 (chr2L:20562305(+))
All.reads Coverage
Position relative to miRNA 5' end
mir-4973 (chr2L:20562305(+))
TtoC.reads Coverage
Position relative to miRNA 5' end
mir-4973 (chr2L:20562305(+))
SRR013547.reads Coverage
Position relative to miRNA 5' end
mir-4973 (chr2L:20562305(+))
SRR013548.reads Coverage
Position relative to miRNA 5' end

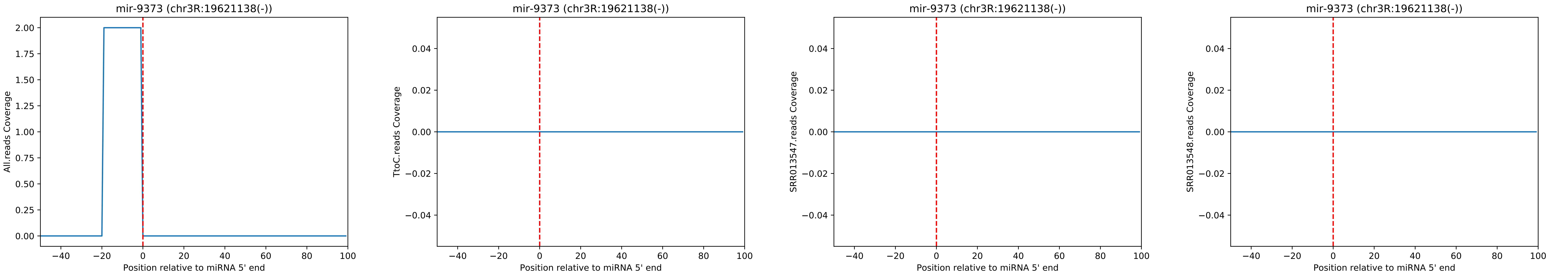
mir-9373 (chr3R:19621138(-))
All.reads Coverage
TtoC.reads Coverage
SRR013547.reads Coverage
SRR013548.reads Coverage
Position relative to miRNA 5' end

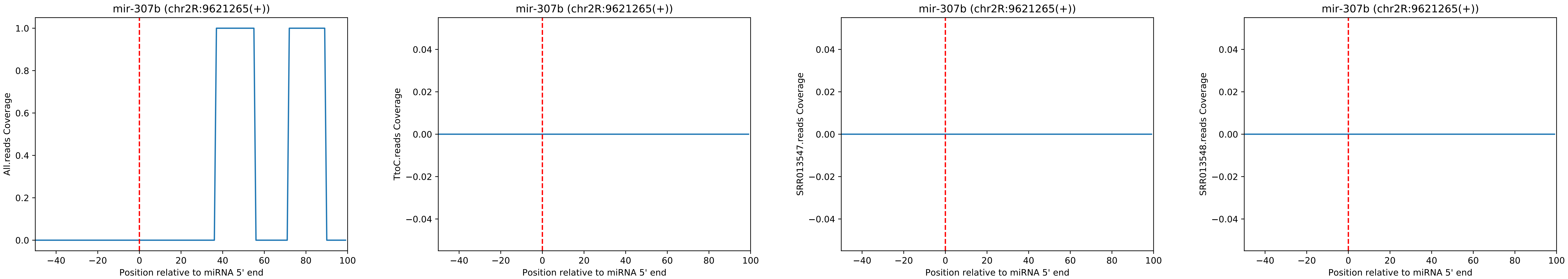
mir-307b (chr2R:9621265(+))
All.reads Coverage
TtoC.reads Coverage
SRR013547.reads Coverage
SRR013548.reads Coverage
Position relative to miRNA 5' end

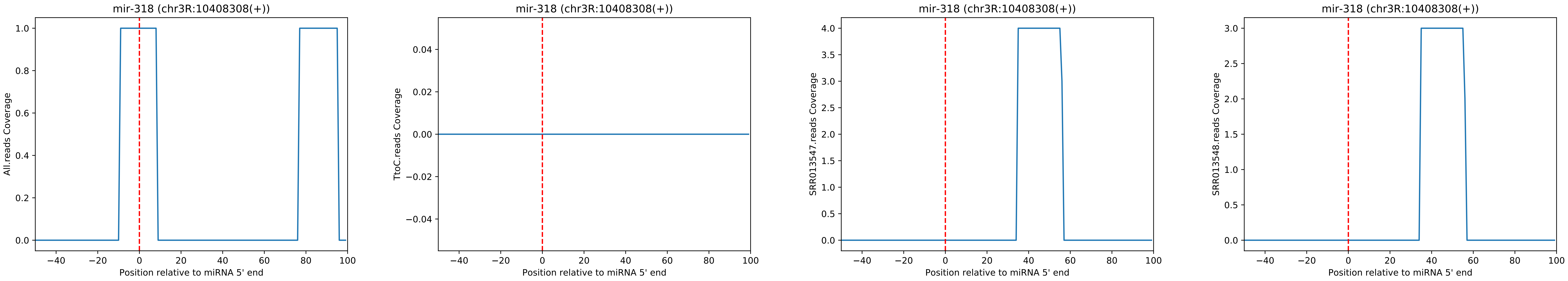
mir-318 (chr3R:10408308(+))
All.reads Coverage
Position relative to miRNA 5' end
mir-318 (chr3R:10408308(+))
TtoC.reads Coverage
Position relative to miRNA 5' end
mir-318 (chr3R:10408308(+))
SRR013547.reads Coverage
Position relative to miRNA 5' end
mir-318 (chr3R:10408308(+))
SRR013548.reads Coverage
Position relative to miRNA 5' end

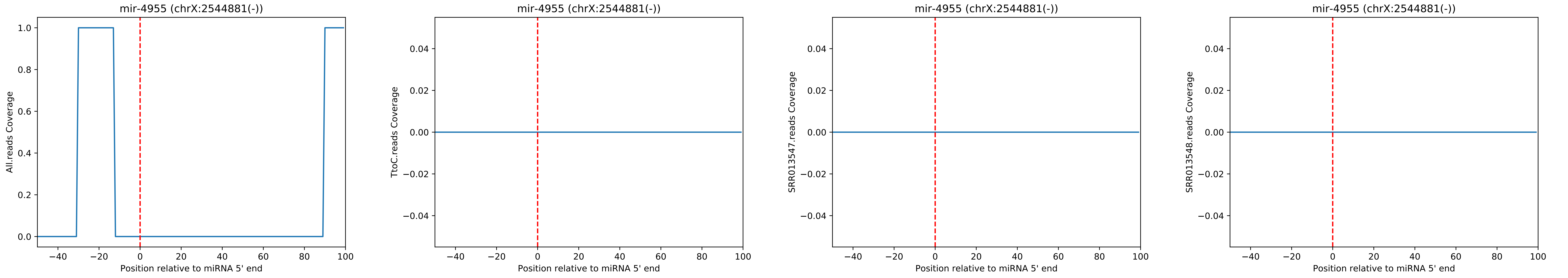
mir-4955 (chrX:2544881(-))
All.reads Coverage
Position relative to miRNA 5' end
mir-4955 (chrX:2544881(-))
TtoC.reads Coverage
Position relative to miRNA 5' end
mir-4955 (chrX:2544881(-))
SRR013547.reads Coverage
Position relative to miRNA 5' end
mir-4955 (chrX:2544881(-))
SRR013548.reads Coverage
Position relative to miRNA 5' end

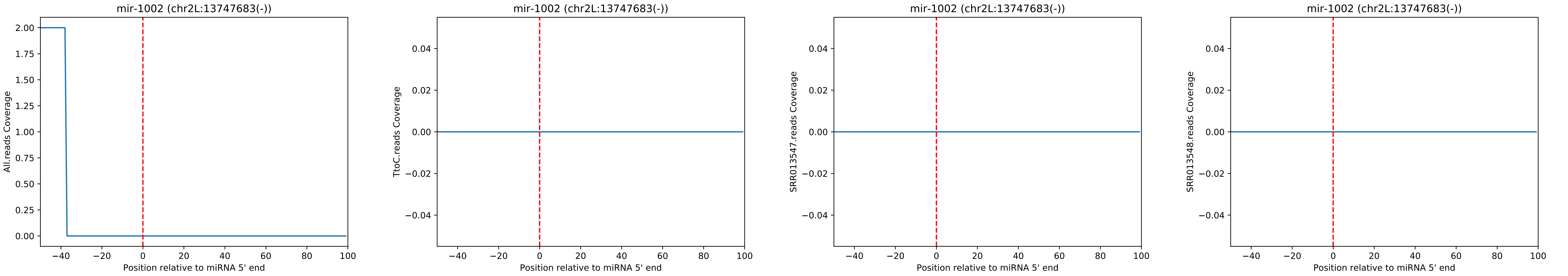

mir-1002 (chr2L:13747683(-))
All.reads Coverage
2.00
1.75
1.50
1.25
1.00
0.75
0.50
0.25
0.00
−40
−20
0
20
40
60
80
100
Position relative to miRNA 5' end
mir-1002 (chr2L:13747683(-))
TtoC.reads Coverage
0.04
0.02
0.00
−0.02
−0.04
Position relative to miRNA 5' end
mir-1002 (chr2L:13747683(-))
SRR013547.reads Coverage
Position relative to miRNA 5' end
mir-1002 (chr2L:13747683(-))
SRR013548.reads Coverage
Position relative to miRNA 5' end

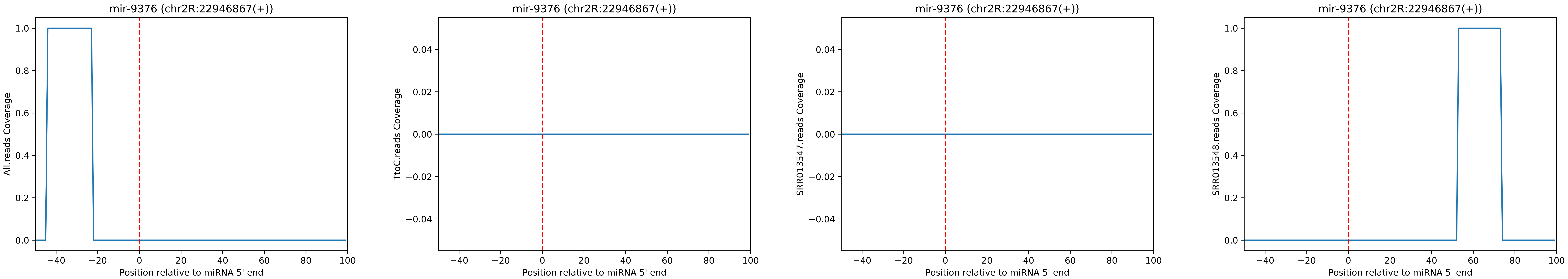
mir-9376 (chr2R:22946867(+))
All.reads Coverage
TtoC.reads Coverage
SRR013547.reads Coverage
SRR013548.reads Coverage
Position relative to miRNA 5' end

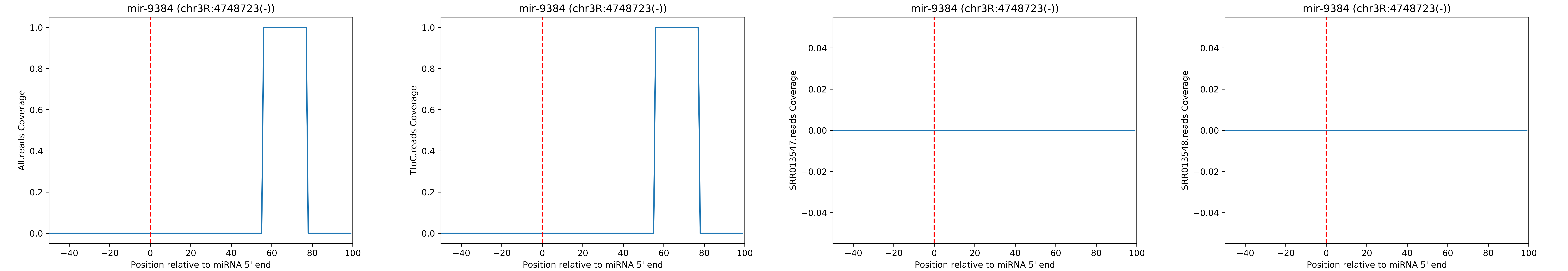
mir-9384 (chr3R:4748723(-))
All.reads Coverage
Position relative to miRNA 5' end
mir-9384 (chr3R:4748723(-))
TtoC.reads Coverage
Position relative to miRNA 5' end
mir-9384 (chr3R:4748723(-))
SRR013547.reads Coverage
Position relative to miRNA 5' end
mir-9384 (chr3R:4748723(-))
SRR013548.reads Coverage
Position relative to miRNA 5' end

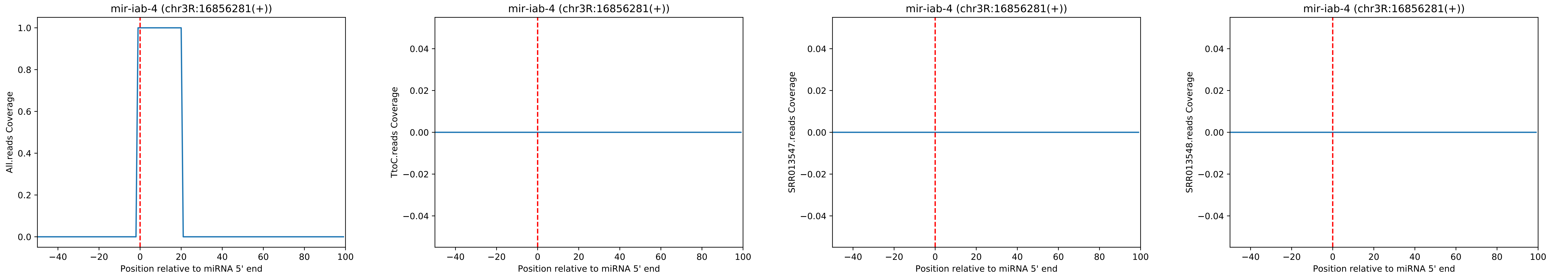

mir-iab-4 (chr3R:16856281(+))
All.reads Coverage
Position relative to miRNA 5' end
mir-iab-4 (chr3R:16856281(+))
TtoC.reads Coverage
Position relative to miRNA 5' end
mir-iab-4 (chr3R:16856281(+))
SRR013547.reads Coverage
Position relative to miRNA 5' end
mir-iab-4 (chr3R:16856281(+))
SRR013548.reads Coverage
Position relative to miRNA 5' end

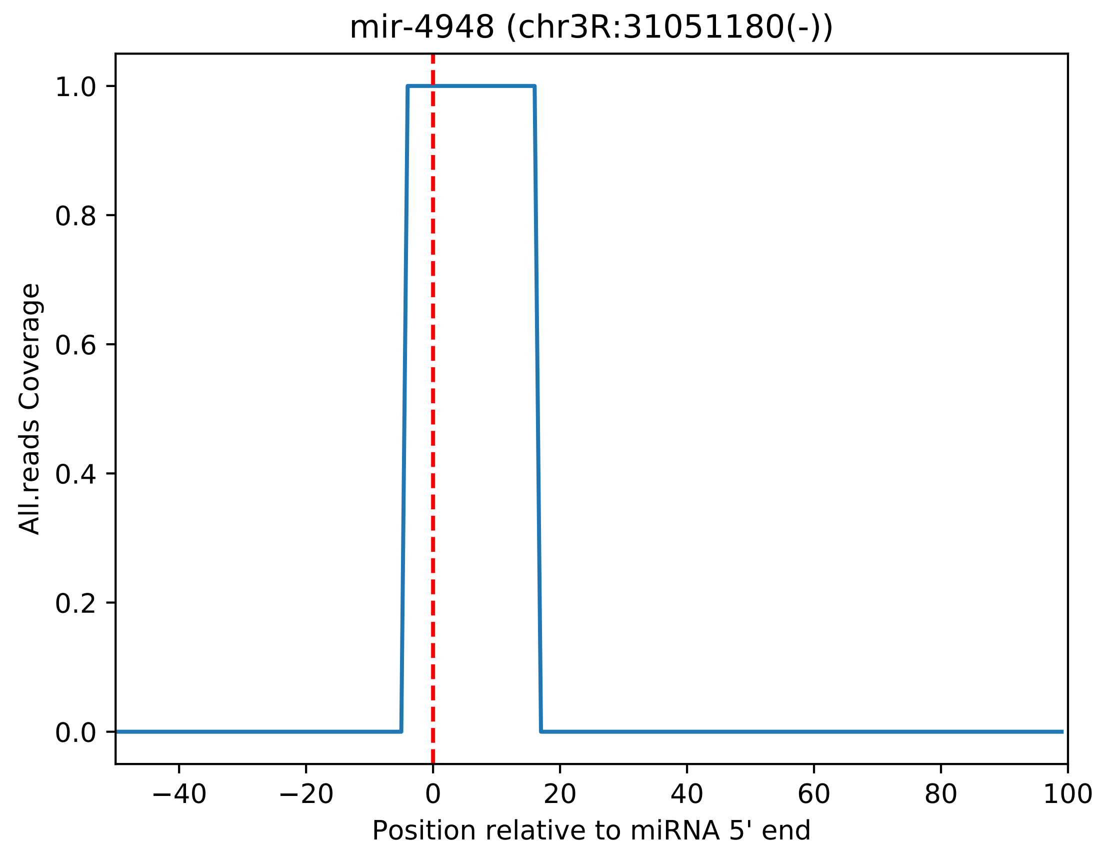
mir-4948 (chr3R:31051180(-))
All.reads Coverage
Position relative to miRNA 5' end

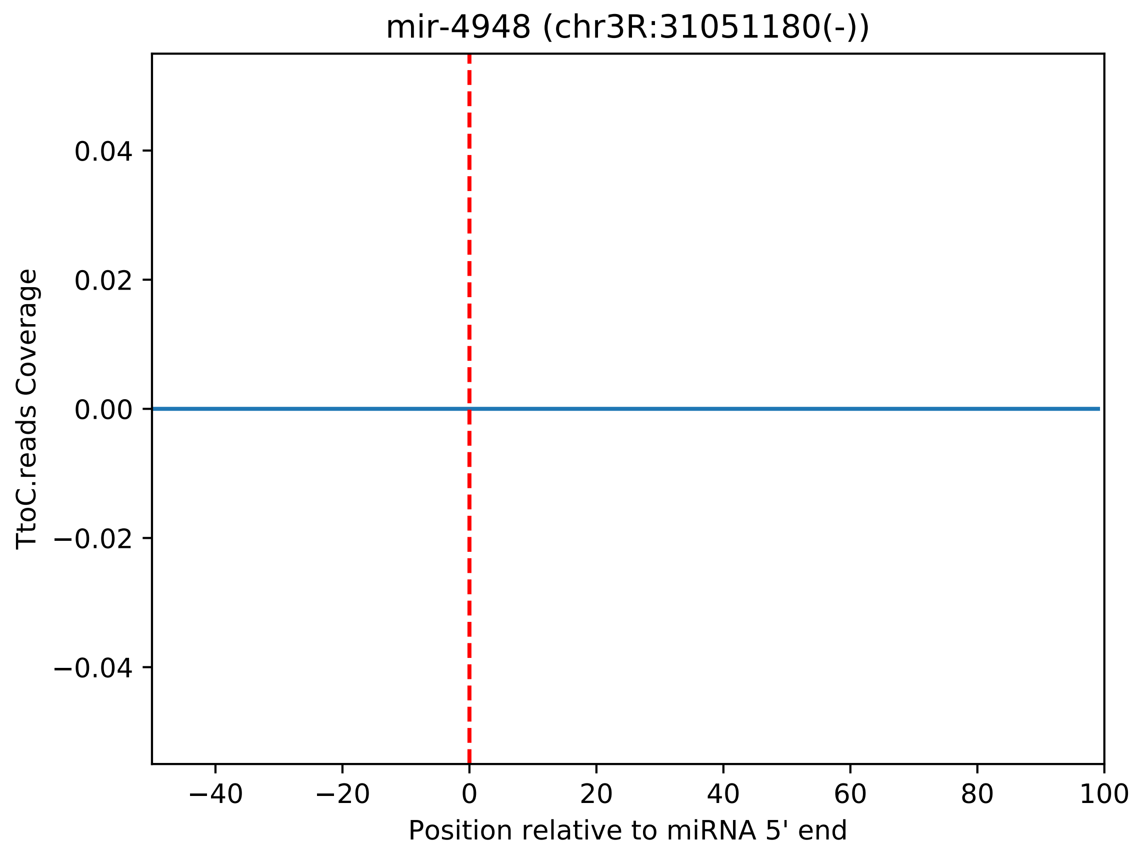
mir-4948 (chr3R:31051180(-))
TtoC.reads Coverage
Position relative to miRNA 5' end

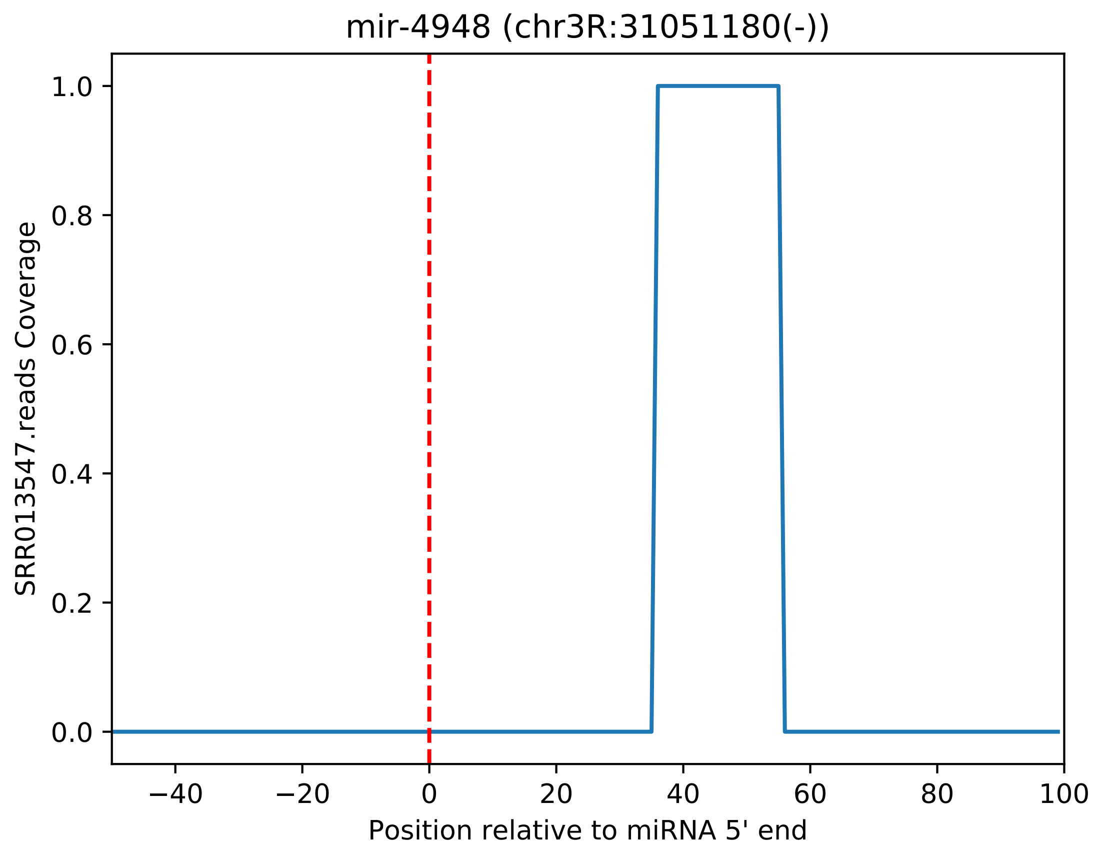
mir-4948 (chr3R:31051180(-))
SRR013547.reads Coverage
Position relative to miRNA 5' end

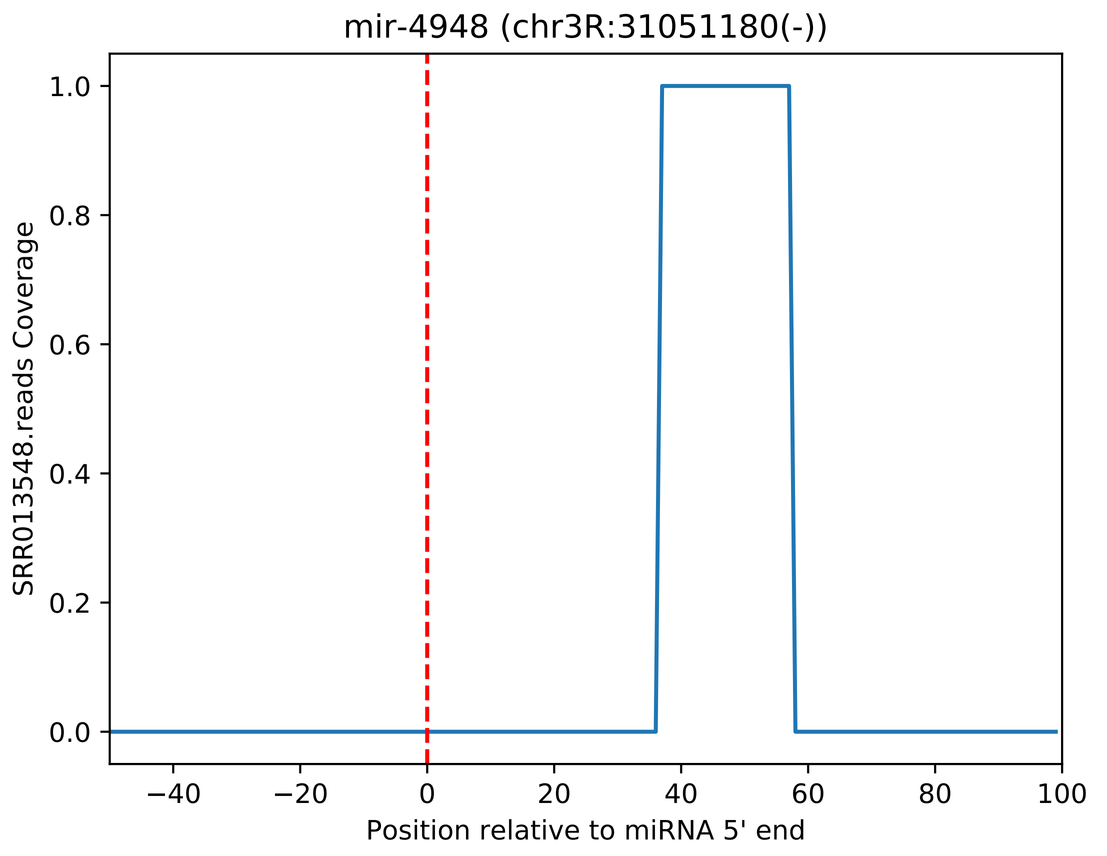
mir-4948 (chr3R:31051180(-))
SRR013548.reads Coverage
Position relative to miRNA 5' end

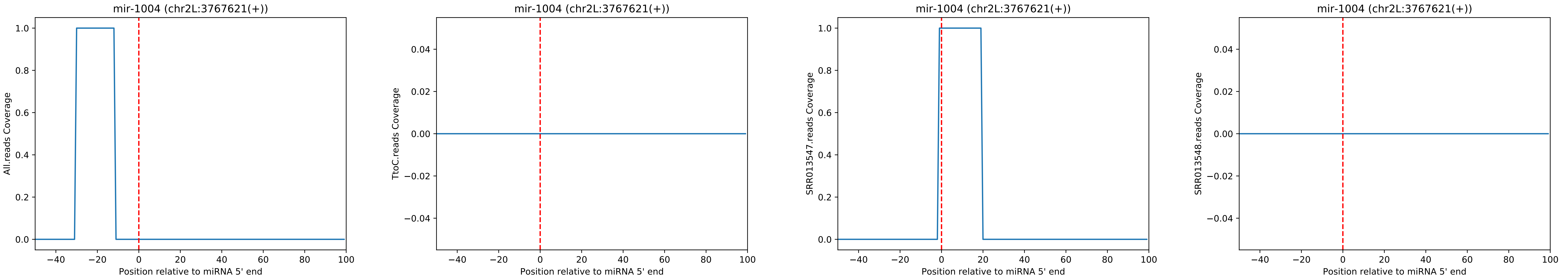

mir-1004 (chr2L:3767621(+))
All.reads Coverage
Position relative to miRNA 5' end
mir-1004 (chr2L:3767621(+))
TtoC.reads Coverage
Position relative to miRNA 5' end
mir-1004 (chr2L:3767621(+))
SRR013547.reads Coverage
Position relative to miRNA 5' end
mir-1004 (chr2L:3767621(+))
SRR013548.reads Coverage
Position relative to miRNA 5' end

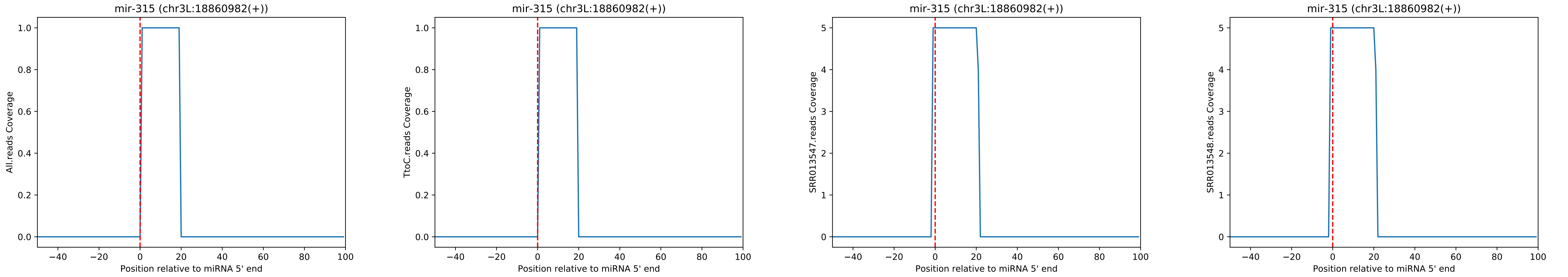
mir-315 (chr3L:18860982(+))
All.reads Coverage
Position relative to miRNA 5' end
mir-315 (chr3L:18860982(+))
TtoC.reads Coverage
Position relative to miRNA 5' end
mir-315 (chr3L:18860982(+))
SRR013547.reads Coverage
Position relative to miRNA 5' end
mir-315 (chr3L:18860982(+))
SRR013548.reads Coverage
Position relative to miRNA 5' end

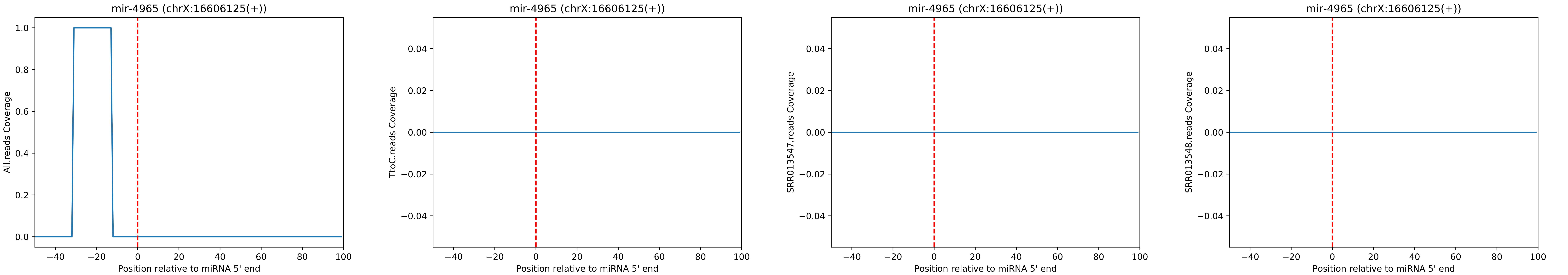

mir-4965 (chrX:16606125(+))
All.reads Coverage
TtoC.reads Coverage
SRR013547.reads Coverage
SRR013548.reads Coverage
Position relative to miRNA 5' end

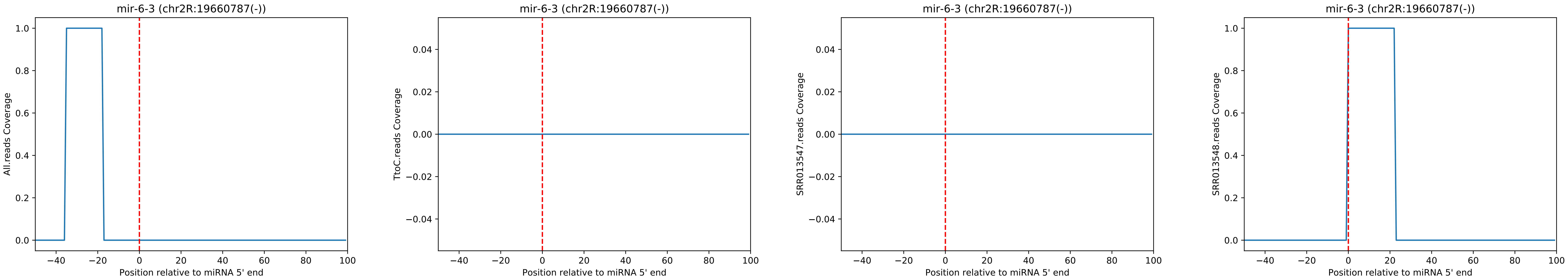
mir-6-3 (chr2R:19660787(-))
All.reads Coverage
Position relative to miRNA 5' end
mir-6-3 (chr2R:19660787(-))
TtoC.reads Coverage
Position relative to miRNA 5' end
mir-6-3 (chr2R:19660787(-))
SRR013547.reads Coverage
Position relative to miRNA 5' end
mir-6-3 (chr2R:19660787(-))
SRR013548.reads Coverage
Position relative to miRNA 5' end

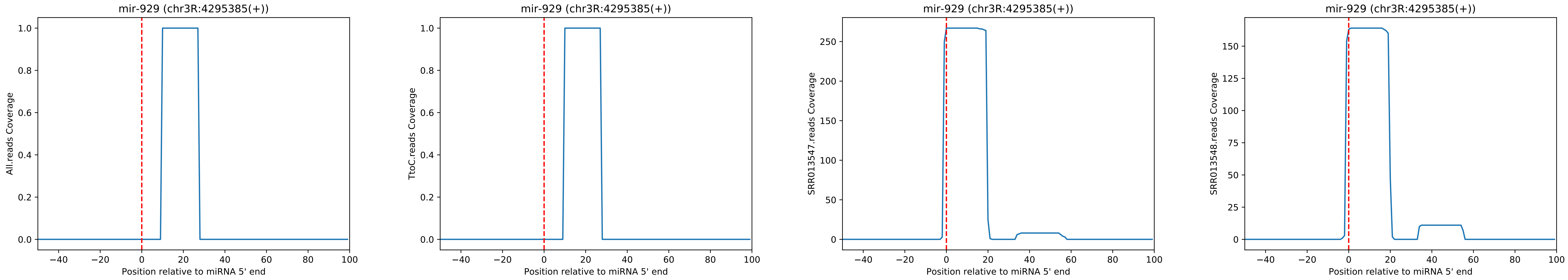
mir-929 (chr3R:4295385(+))
All.reads Coverage
Position relative to miRNA 5' end
mir-929 (chr3R:4295385(+))
TtoC.reads Coverage
Position relative to miRNA 5' end
mir-929 (chr3R:4295385(+))
SRR013547.reads Coverage
Position relative to miRNA 5' end
mir-929 (chr3R:4295385(+))
SRR013548.reads Coverage
Position relative to miRNA 5' end

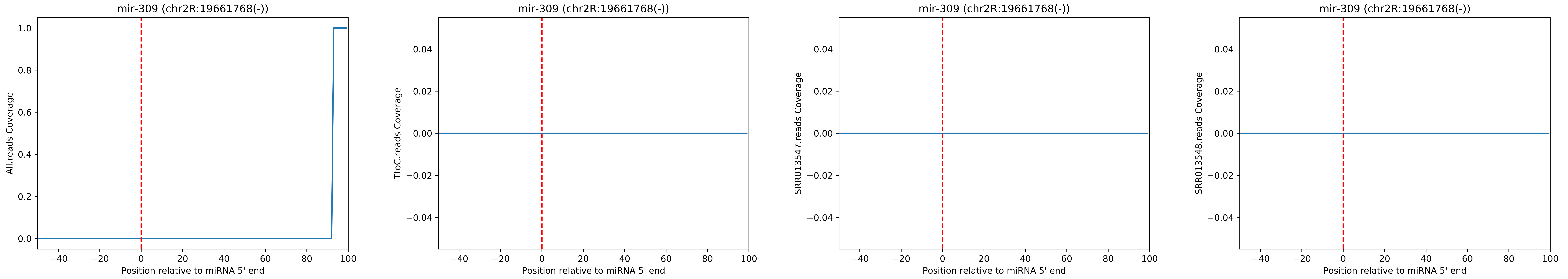
mir-309 (chr2R:19661768(-))
All.reads Coverage
Position relative to miRNA 5' end
mir-309 (chr2R:19661768(-))
TtoC.reads Coverage
Position relative to miRNA 5' end
mir-309 (chr2R:19661768(-))
SRR013547.reads Coverage
Position relative to miRNA 5' end
mir-309 (chr2R:19661768(-))
SRR013548.reads Coverage
Position relative to miRNA 5' end

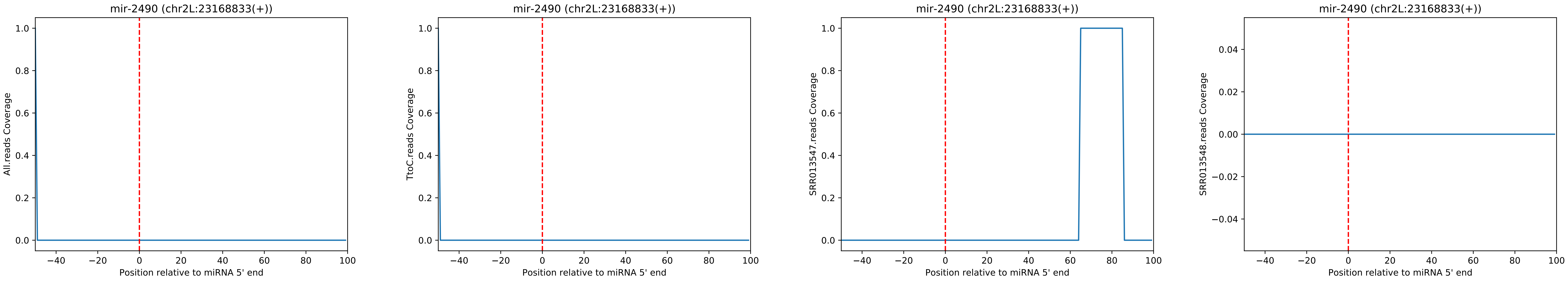
mir-2490 (chr2L:23168833(+))
All.reads Coverage
Position relative to miRNA 5' end
mir-2490 (chr2L:23168833(+))
TtoC.reads Coverage
Position relative to miRNA 5' end
mir-2490 (chr2L:23168833(+))
SRR013547.reads Coverage
Position relative to miRNA 5' end
mir-2490 (chr2L:23168833(+))
SRR013548.reads Coverage
Position relative to miRNA 5' end

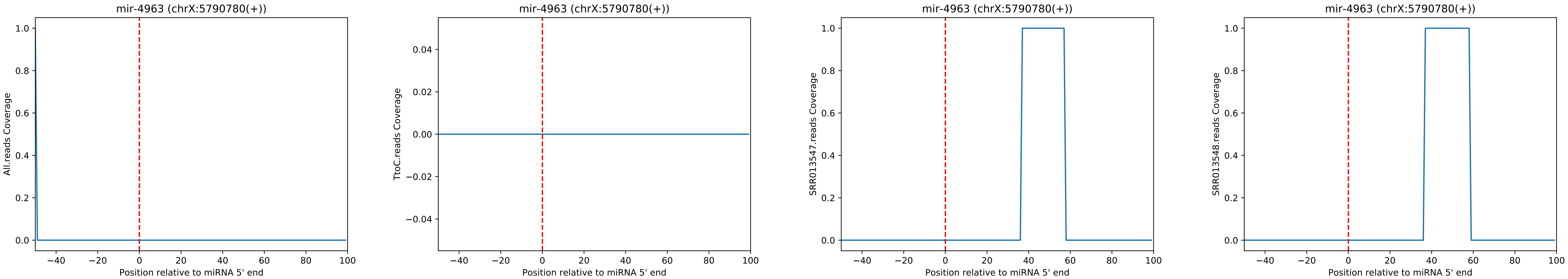

mir-4963 (chrX:5790780(+))
All.reads Coverage
Position relative to miRNA 5' end
mir-4963 (chrX:5790780(+))
TtoC.reads Coverage
Position relative to miRNA 5' end
mir-4963 (chrX:5790780(+))
SRR013547.reads Coverage
Position relative to miRNA 5' end
mir-4963 (chrX:5790780(+))
SRR013548.reads Coverage
Position relative to miRNA 5' end

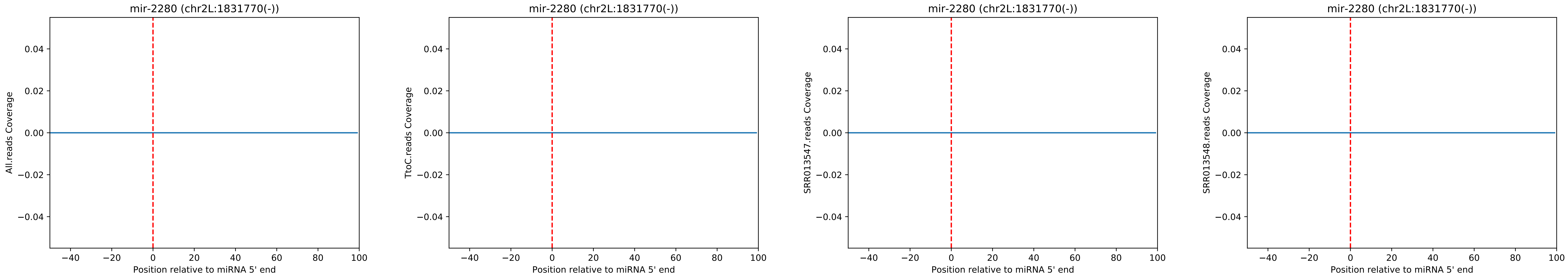
mir-2280 (chr2L:1831770(-))
All.reads Coverage
TtoC.reads Coverage
SRR013547.reads Coverage
SRR013548.reads Coverage
0.04
0.02
0.00
−0.02
−0.04
−40
−20
0
20
40
60
80
100
Position relative to miRNA 5' end

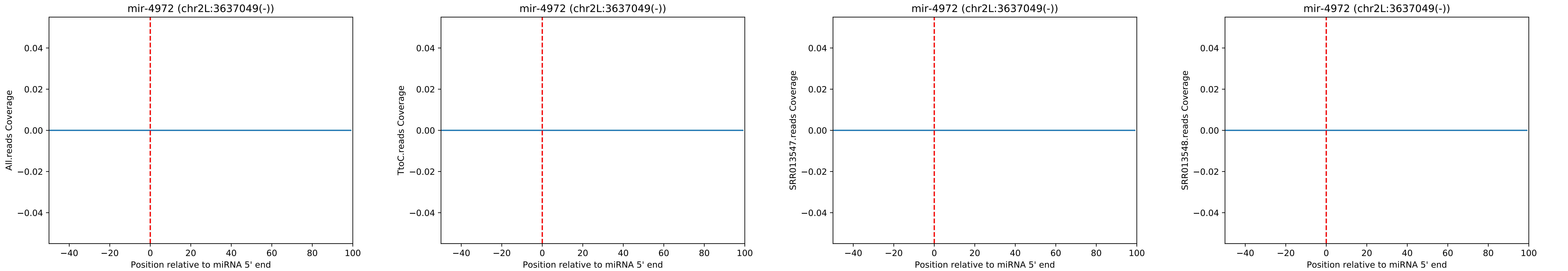

mir-4972 (chr2L:3637049(-))
All.reads Coverage
TtoC.reads Coverage
SRR013547.reads Coverage
SRR013548.reads Coverage
0.04
0.02
0.00
−0.02
−0.04
−40
−20
0
20
40
60
80
100
Position relative to miRNA 5' end

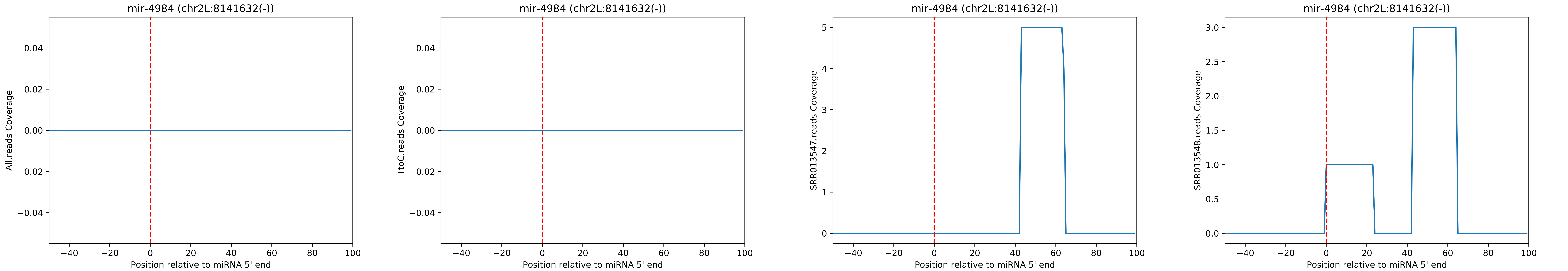
mir-4984 (chr2L:8141632(-))
All.reads Coverage
Position relative to miRNA 5' end
mir-4984 (chr2L:8141632(-))
TtoC.reads Coverage
Position relative to miRNA 5' end
mir-4984 (chr2L:8141632(-))
SRR013547.reads Coverage
Position relative to miRNA 5' end
mir-4984 (chr2L:8141632(-))
SRR013548.reads Coverage
Position relative to miRNA 5' end

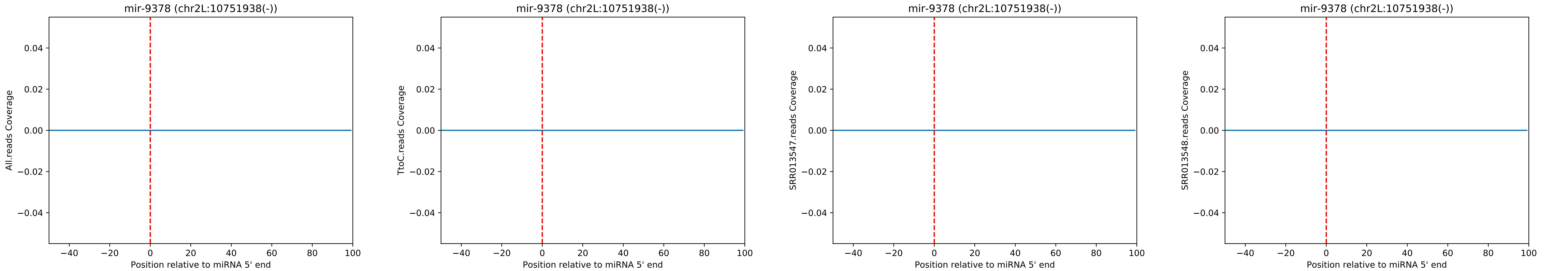
mir-9378 (chr2L:10751938(-))
All.reads Coverage
Position relative to miRNA 5' end
mir-9378 (chr2L:10751938(-))
TtoC.reads Coverage
Position relative to miRNA 5' end
mir-9378 (chr2L:10751938(-))
SRR013547.reads Coverage
Position relative to miRNA 5' end
mir-9378 (chr2L:10751938(-))
SRR013548.reads Coverage
Position relative to miRNA 5' end

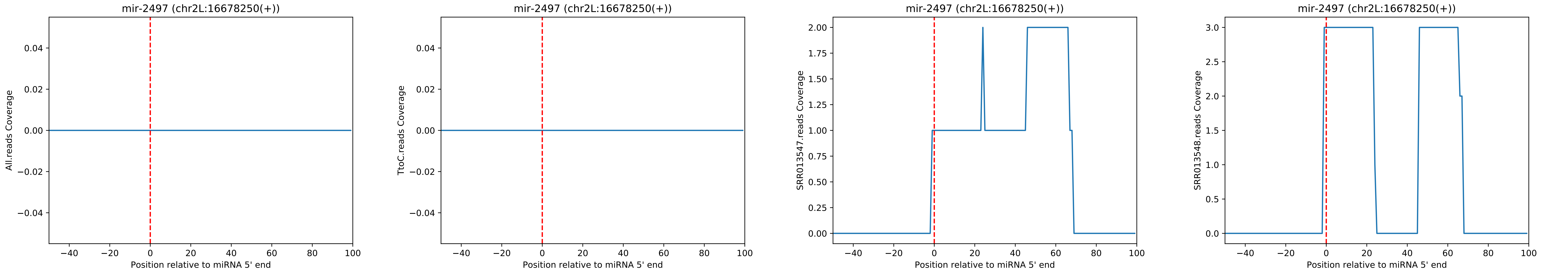
mir-2497 (chr2L:16678250(+))
All.reads Coverage
TtoC.reads Coverage
SRR013547.reads Coverage
SRR013548.reads Coverage
Position relative to miRNA 5' end

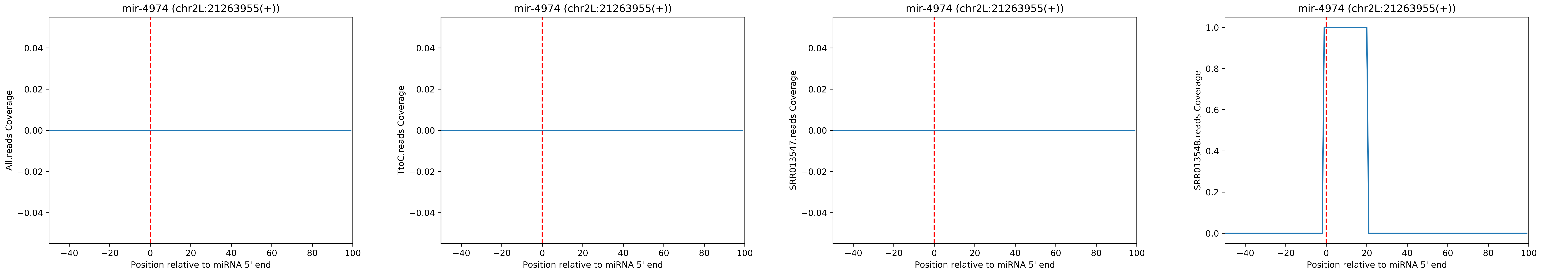
mir-4974 (chr2L:21263955(+))
All.reads Coverage
0.04
0.02
0.00
−0.02
−0.04
Position relative to miRNA 5' end
−40
−20
0
20
40
60
80
100
mir-4974 (chr2L:21263955(+))
TtoC.reads Coverage
Position relative to miRNA 5' end
mir-4974 (chr2L:21263955(+))
SRR013547.reads Coverage
Position relative to miRNA 5' end
mir-4974 (chr2L:21263955(+))
SRR013548.reads Coverage
1.0
0.8
0.6
0.4
0.2
0.0
Position relative to miRNA 5' end

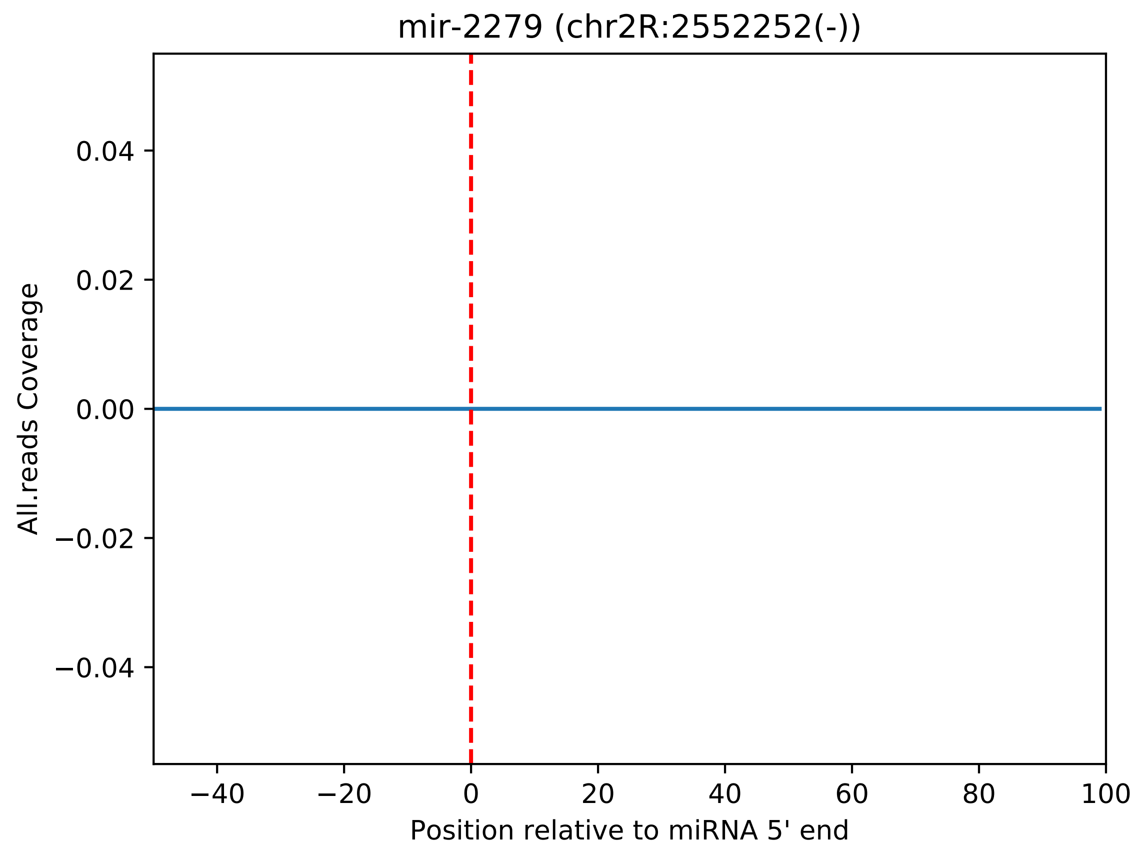
mir-2279 (chr2R:2552252(-))
All.reads Coverage
0.04
0.02
0.00
−0.02
−0.04
−40
−20
0
20
40
60
80
100
Position relative to miRNA 5' end

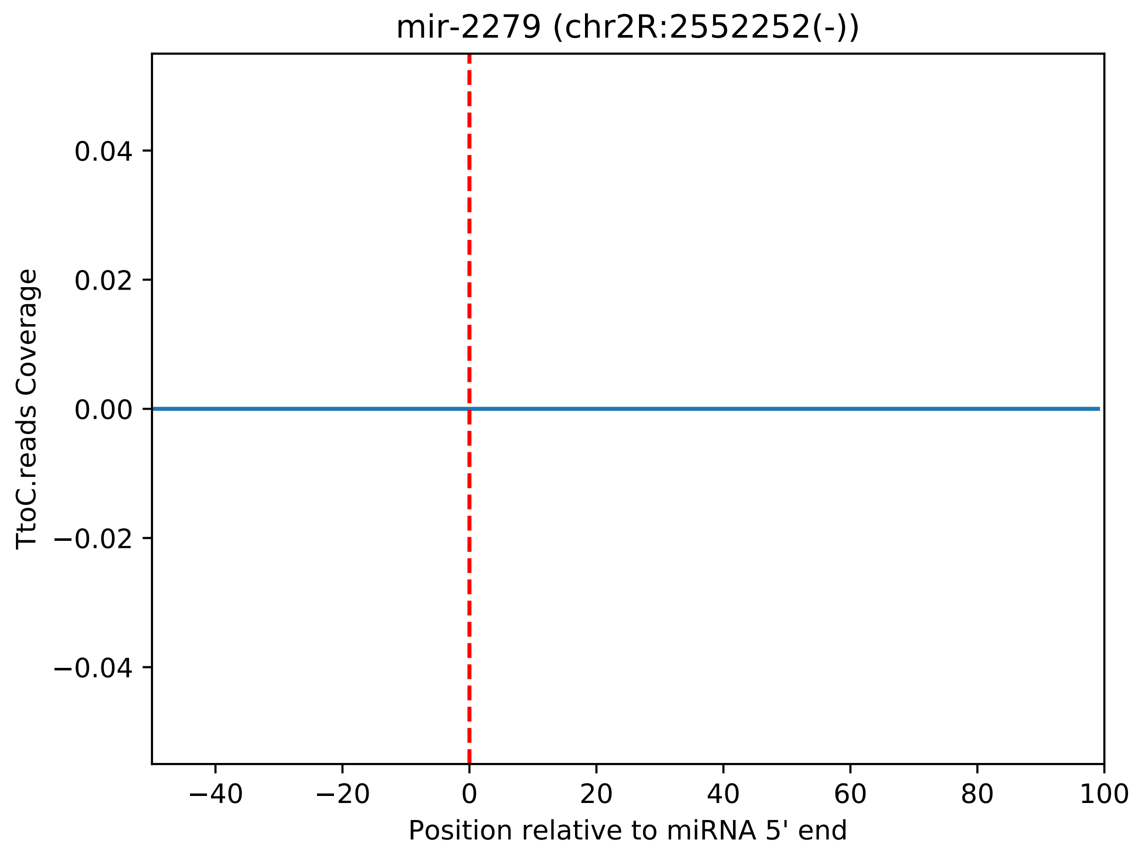
mir-2279 (chr2R:2552252(-))
TtoC.reads Coverage
0.04
0.02
0.00
−0.02
−0.04
−40
−20
0
20
40
60
80
100
Position relative to miRNA 5' end

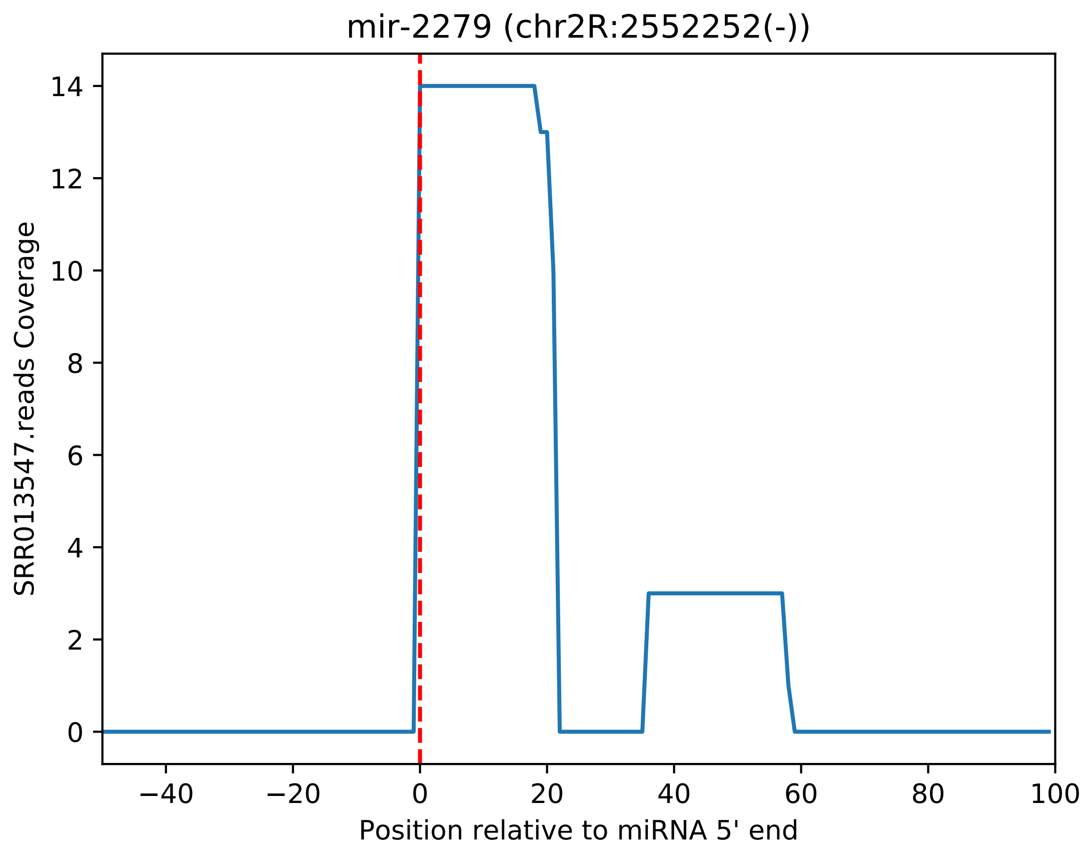
mir-2279 (chr2R:2552252(-))
SRR013547.reads Coverage
14
12
10
8
6
4
2
0
−40
−20
0
20
40
60
80
100
Position relative to miRNA 5' end

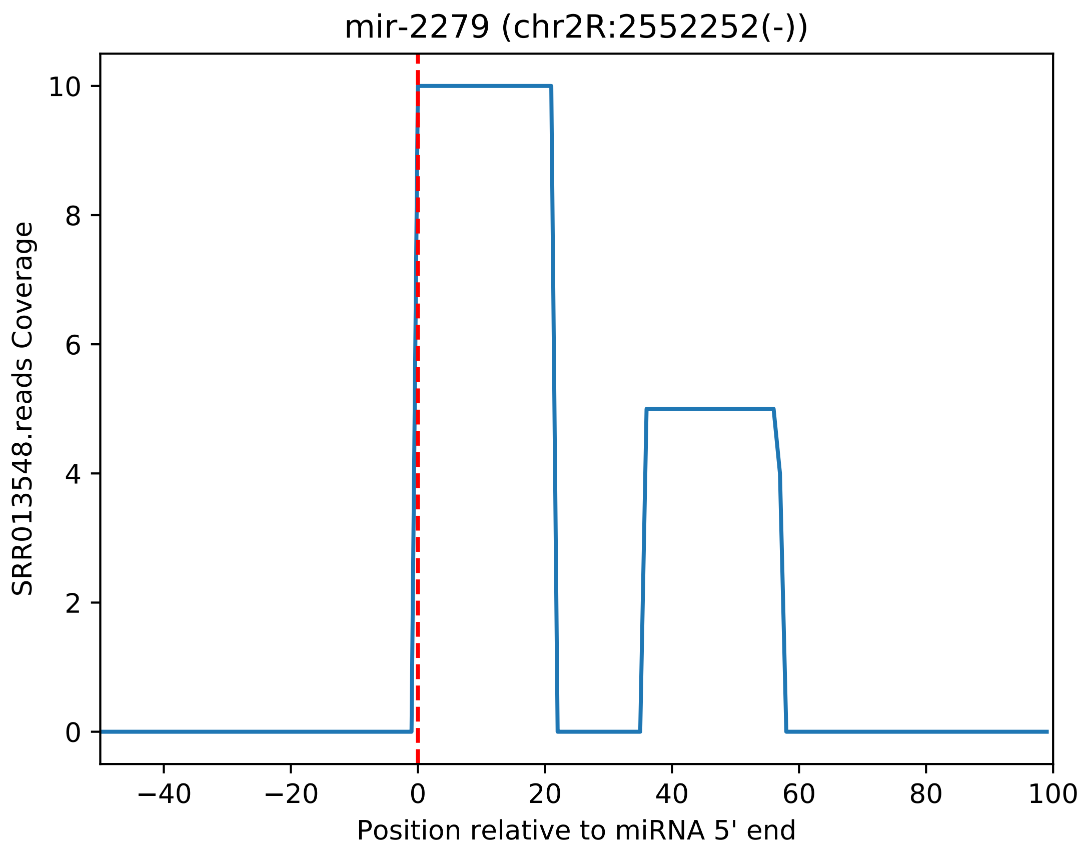
mir-2279 (chr2R:2552252(-))
SRR013548.reads Coverage
10
8
6
4
2
0
−40
−20
0
20
40
60
80
100
Position relative to miRNA 5' end

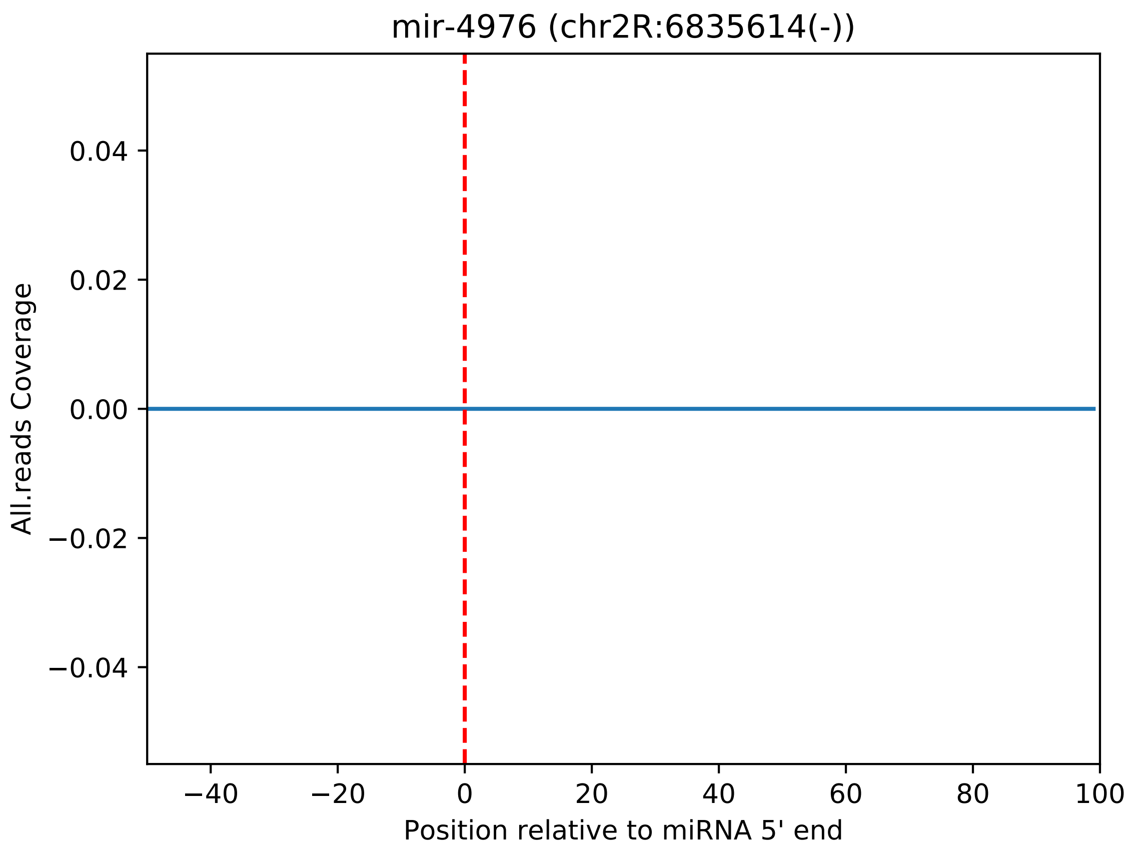
mir-4976 (chr2R:6835614(-))
All.reads Coverage
0.04
0.02
0.00
−0.02
−0.04
−40
−20
0
20
40
60
80
100
Position relative to miRNA 5' end

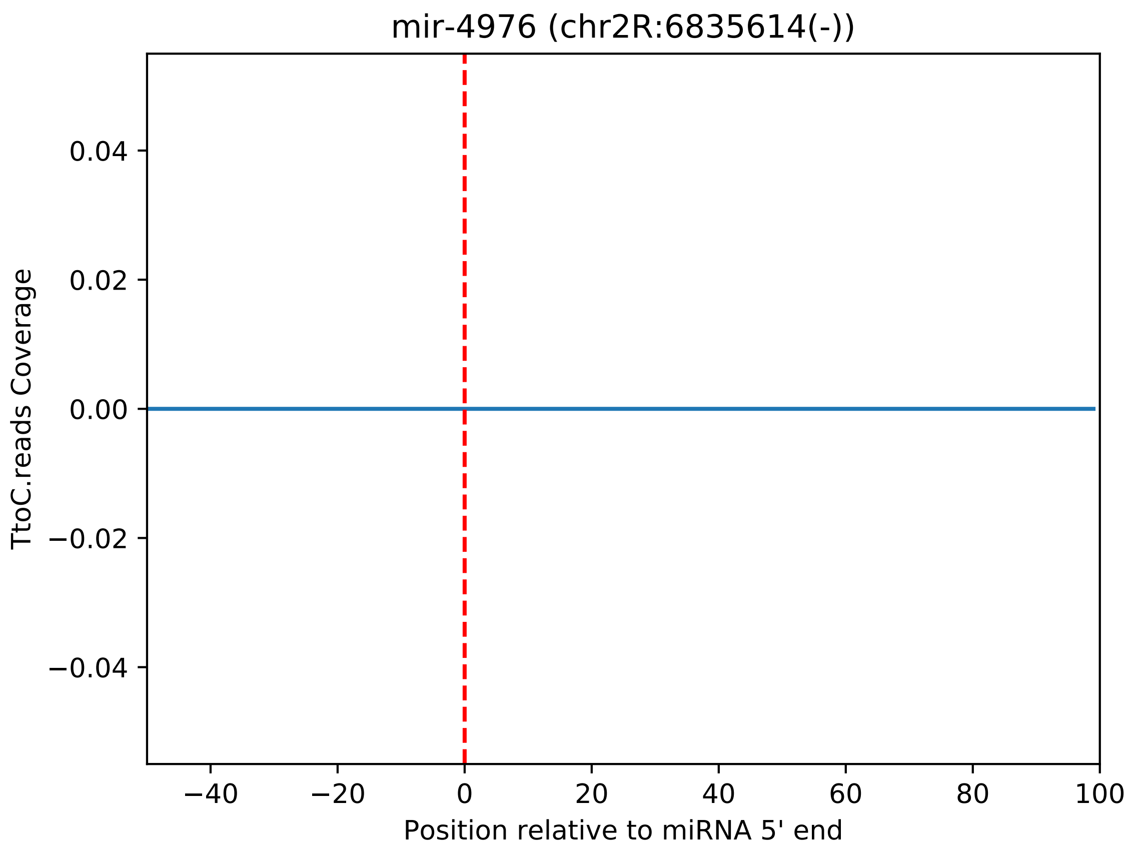
mir-4976 (chr2R:6835614(-))
TtoC.reads Coverage
0.04
0.02
0.00
−0.02
−0.04
−40
−20
0
20
40
60
80
100
Position relative to miRNA 5' end

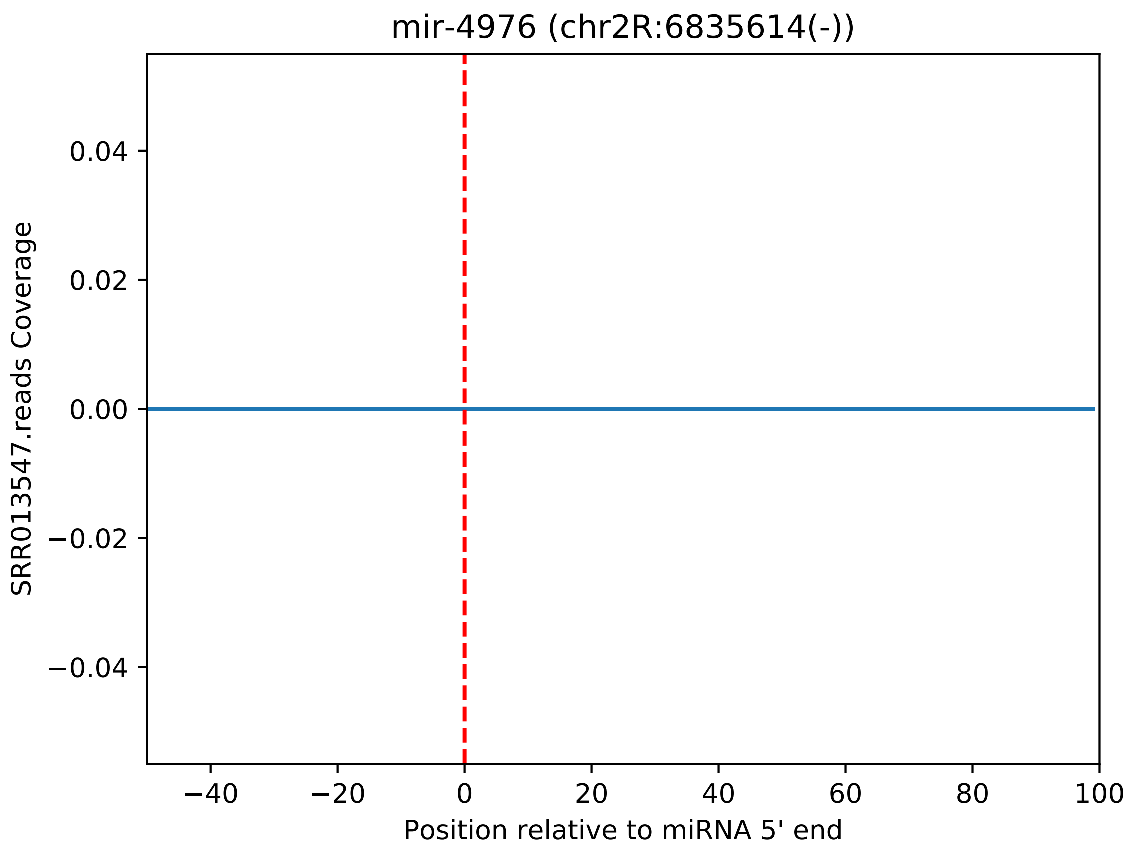
mir-4976 (chr2R:6835614(-))
SRR013547.reads Coverage
0.04
0.02
0.00
−0.02
−0.04
−40
−20
0
20
40
60
80
100
Position relative to miRNA 5' end

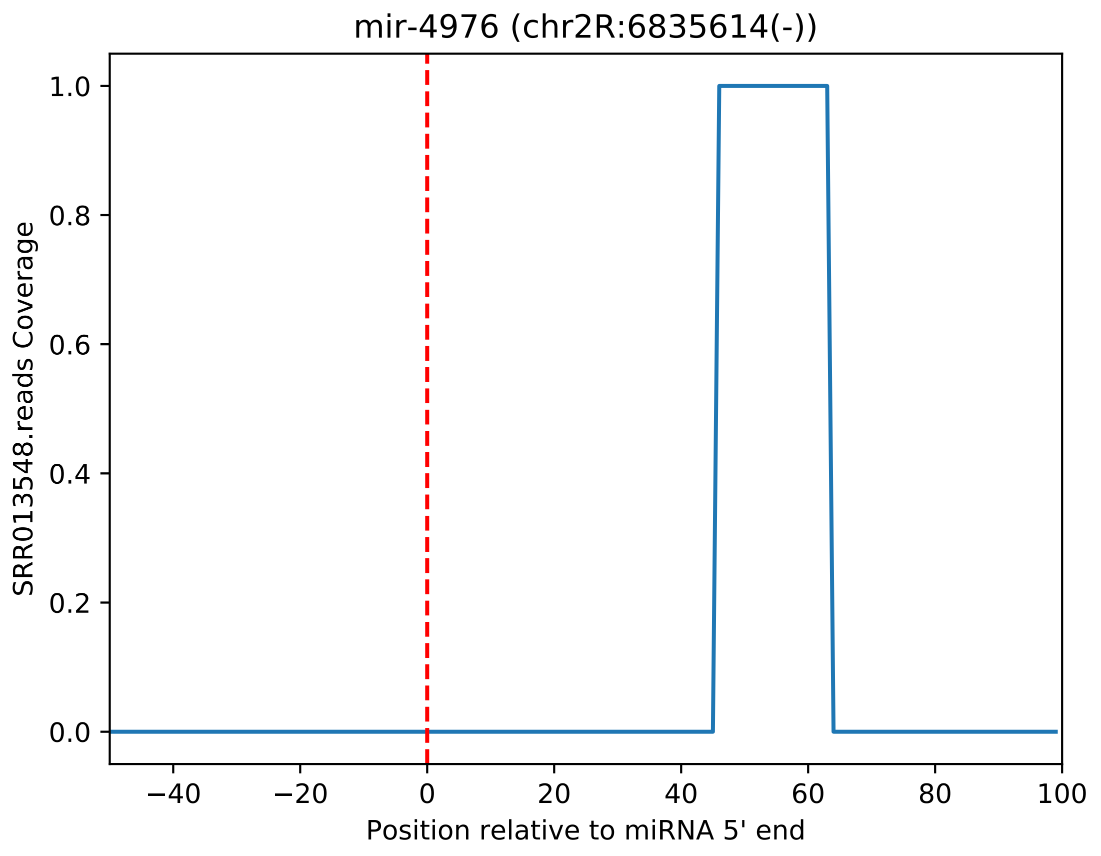
mir-4976 (chr2R:6835614(-))
SRR013548.reads Coverage
1.0
0.8
0.6
0.4
0.2
0.0
−40
−20
0
20
40
60
80
100
Position relative to miRNA 5' end

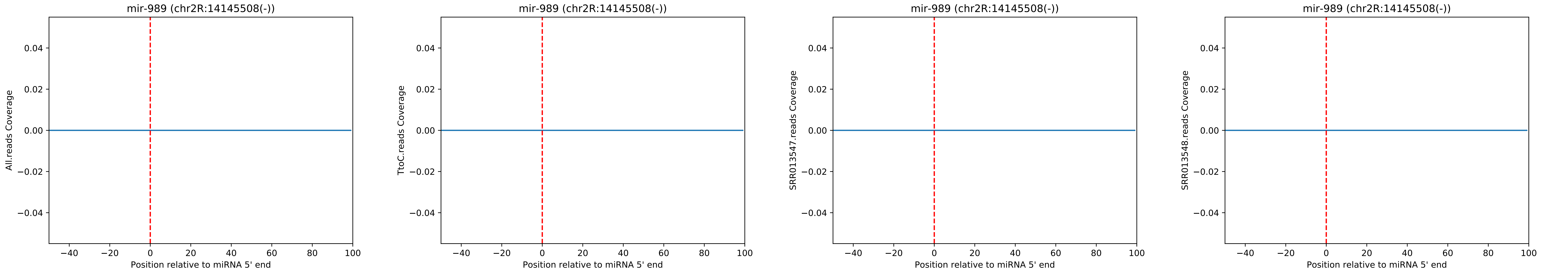
mir-989 (chr2R:14145508(-))
All.reads Coverage
TtoC.reads Coverage
SRR013547.reads Coverage
SRR013548.reads Coverage
0.04
0.02
0.00
−0.02
−0.04
−40
−20
0
20
40
60
80
100
Position relative to miRNA 5' end

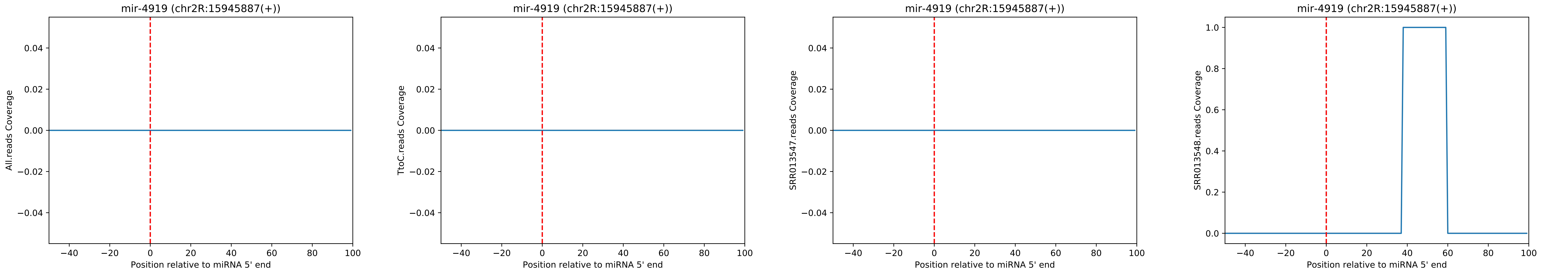

mir-4919 (chr2R:15945887(+))
All.reads Coverage
Position relative to miRNA 5' end
mir-4919 (chr2R:15945887(+))
TtoC.reads Coverage
Position relative to miRNA 5' end
mir-4919 (chr2R:15945887(+))
SRR013547.reads Coverage
Position relative to miRNA 5' end
mir-4919 (chr2R:15945887(+))
SRR013548.reads Coverage
Position relative to miRNA 5' end

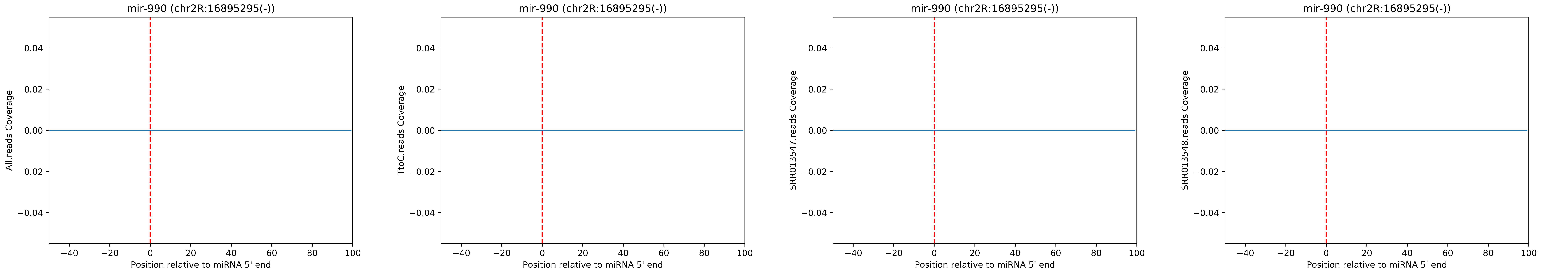
mir-990 (chr2R:16895295(-))
All.reads Coverage
TtoC.reads Coverage
SRR013547.reads Coverage
SRR013548.reads Coverage
Position relative to miRNA 5' end
0.04
0.02
0.00
−0.02
−0.04
−40
−20
0
20
40
60
80
100

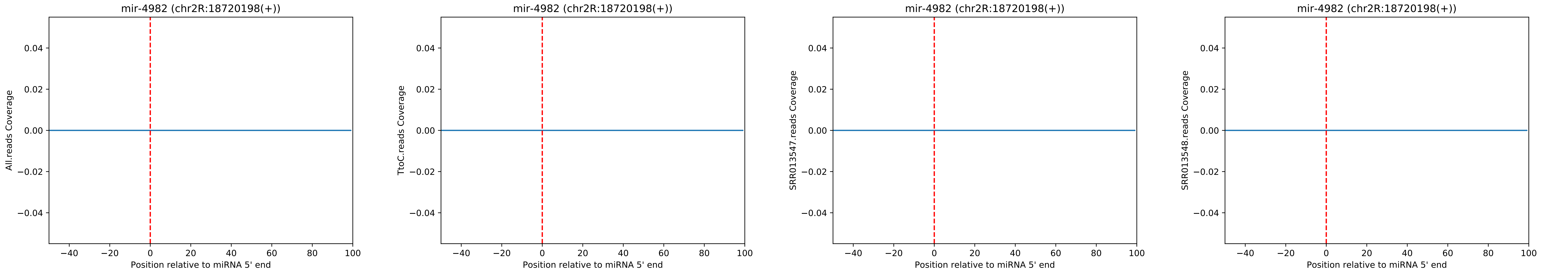

mir-4982 (chr2R:18720198(+))
All.reads Coverage
TtoC.reads Coverage
SRR013547.reads Coverage
SRR013548.reads Coverage
Position relative to miRNA 5' end

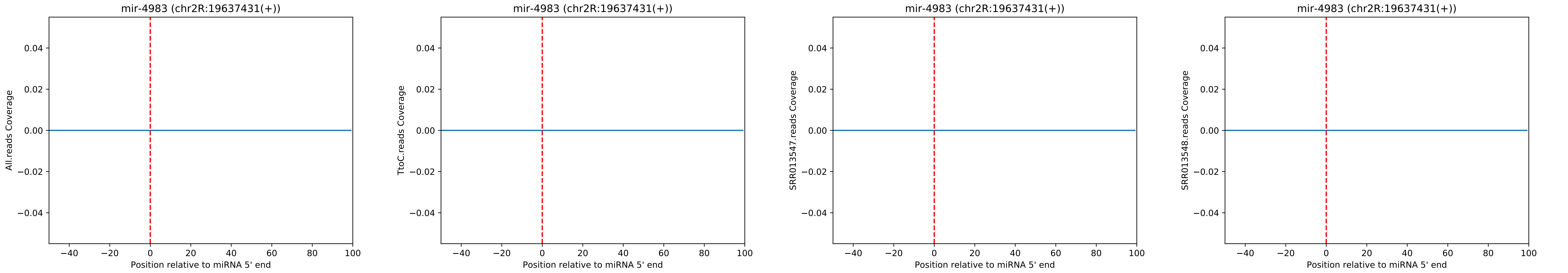
mir-4983 (chr2R:19637431(+))
All.reads Coverage
TtoC.reads Coverage
SRR013547.reads Coverage
SRR013548.reads Coverage
Position relative to miRNA 5' end
0.04
0.02
0.00
−0.02
−0.04
−40
−20
0
20
40
60
80
100

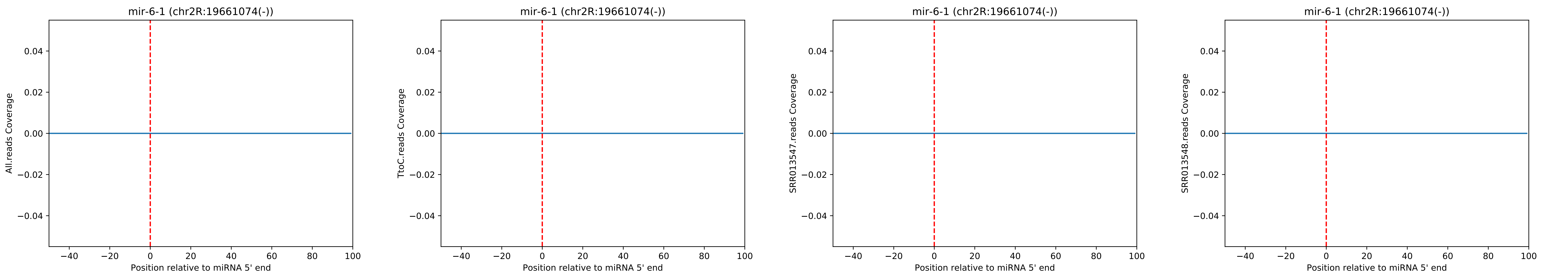

mir-6-1 (chr2R:19661074(-))
All.reads Coverage
Position relative to miRNA 5' end
mir-6-1 (chr2R:19661074(-))
TtoC.reads Coverage
Position relative to miRNA 5' end
mir-6-1 (chr2R:19661074(-))
SRR013547.reads Coverage
Position relative to miRNA 5' end
mir-6-1 (chr2R:19661074(-))
SRR013548.reads Coverage
Position relative to miRNA 5' end

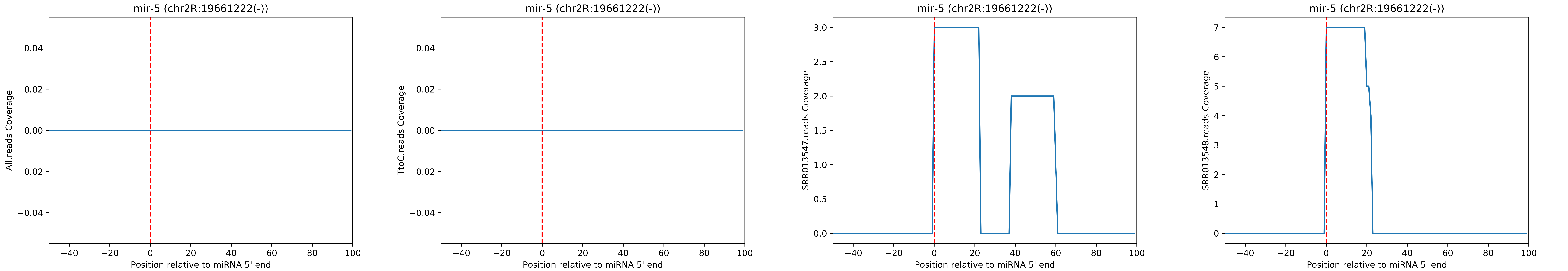
mir-5 (chr2R:19661222(-))
All.reads Coverage
Position relative to miRNA 5' end
mir-5 (chr2R:19661222(-))
TtoC.reads Coverage
Position relative to miRNA 5' end
mir-5 (chr2R:19661222(-))
SRR013547.reads Coverage
Position relative to miRNA 5' end
mir-5 (chr2R:19661222(-))
SRR013548.reads Coverage
Position relative to miRNA 5' end

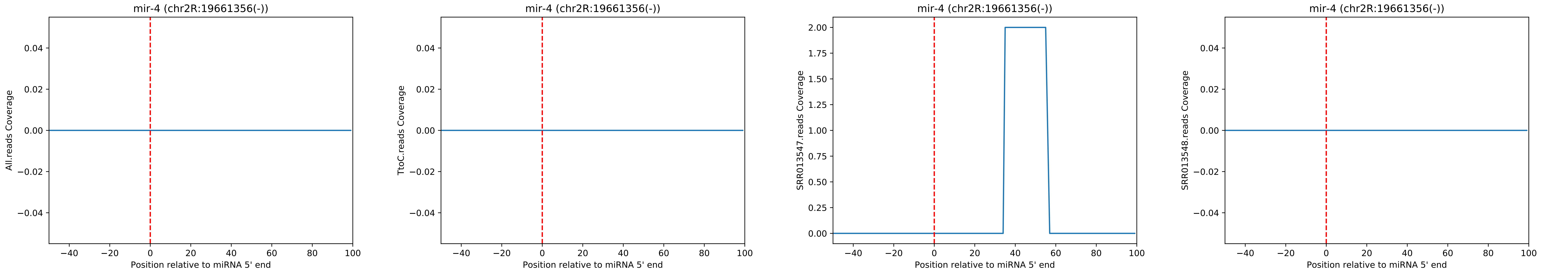
mir-4 (chr2R:19661356(-))
All.reads Coverage
TtoC.reads Coverage
SRR013547.reads Coverage
SRR013548.reads Coverage
Position relative to miRNA 5' end
0.04
0.02
0.00
−0.02
−0.04
2.00
1.75
1.50
1.25
1.00
0.75
0.50
0.25
0.00
−40
−20
0
20
40
60
80
100

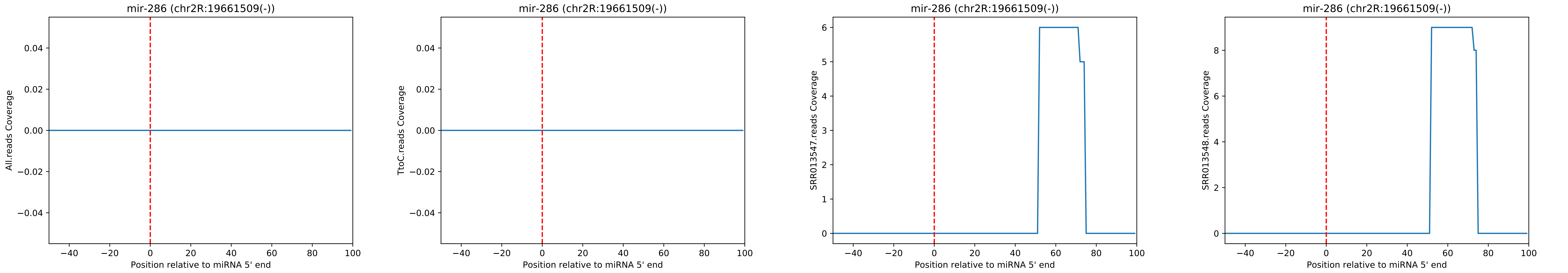

mir-286 (chr2R:19661509(-))
All.reads Coverage
TtoC.reads Coverage
SRR013547.reads Coverage
SRR013548.reads Coverage
Position relative to miRNA 5' end

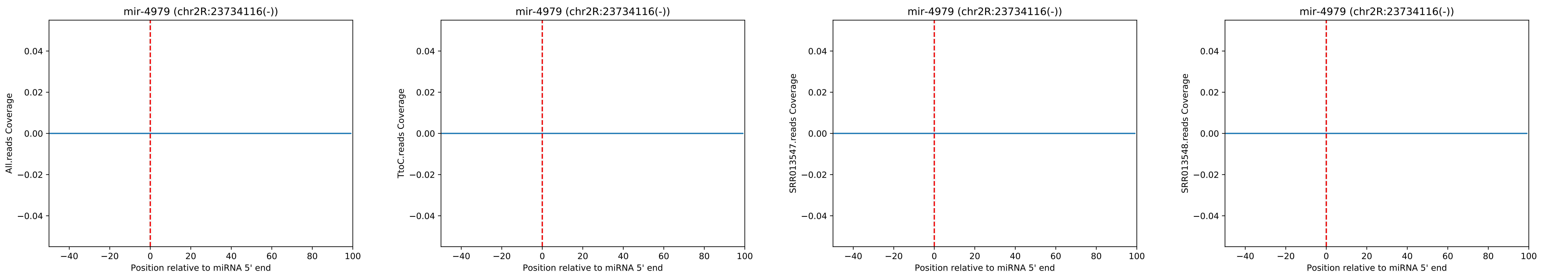
mir-4979 (chr2R:23734116(-))
All.reads Coverage
TtoC.reads Coverage
SRR013547.reads Coverage
SRR013548.reads Coverage
Position relative to miRNA 5' end

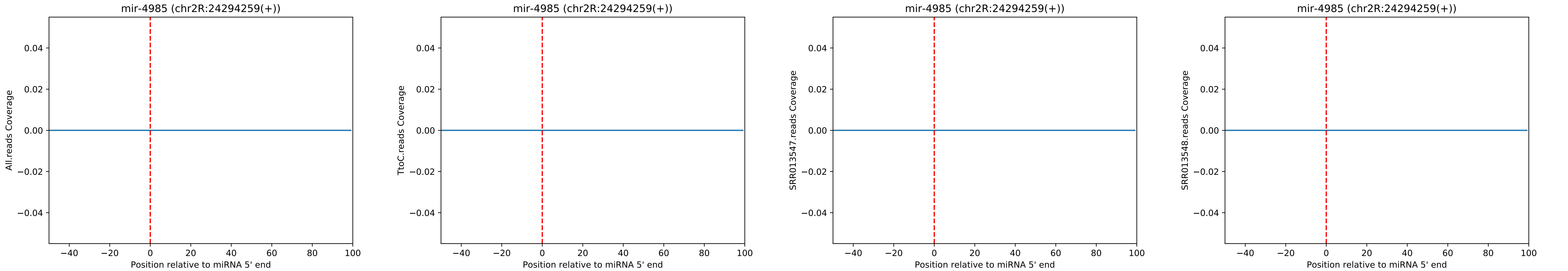

mir-4985 (chr2R:24294259(+))
All.reads Coverage
Position relative to miRNA 5' end
mir-4985 (chr2R:24294259(+))
TtoC.reads Coverage
Position relative to miRNA 5' end
mir-4985 (chr2R:24294259(+))
SRR013547.reads Coverage
Position relative to miRNA 5' end
mir-4985 (chr2R:24294259(+))
SRR013548.reads Coverage
Position relative to miRNA 5' end

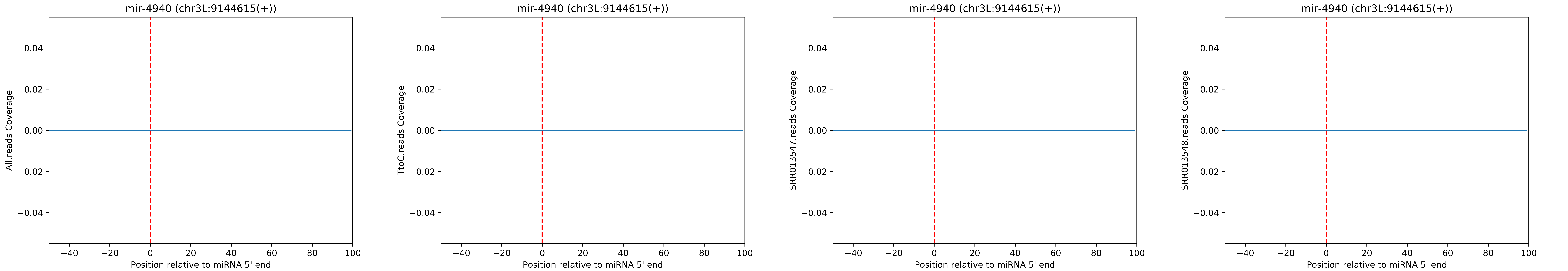
mir-4940 (chr3L:9144615(+))
All.reads Coverage
TtoC.reads Coverage
SRR013547.reads Coverage
SRR013548.reads Coverage
Position relative to miRNA 5' end

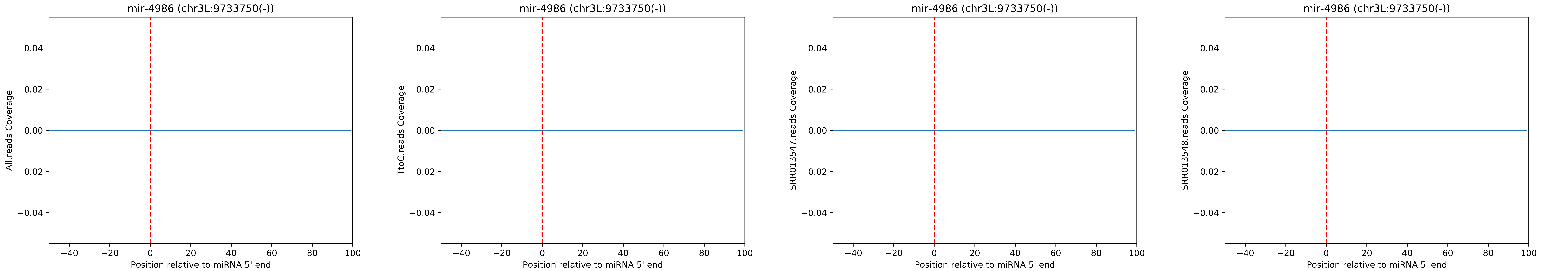

mir-4986 (chr3L:9733750(-))
All.reads Coverage
TtoC.reads Coverage
SRR013547.reads Coverage
SRR013548.reads Coverage
0.04
0.02
0.00
−0.02
−0.04
−40
−20
0
20
40
60
80
100
Position relative to miRNA 5' end

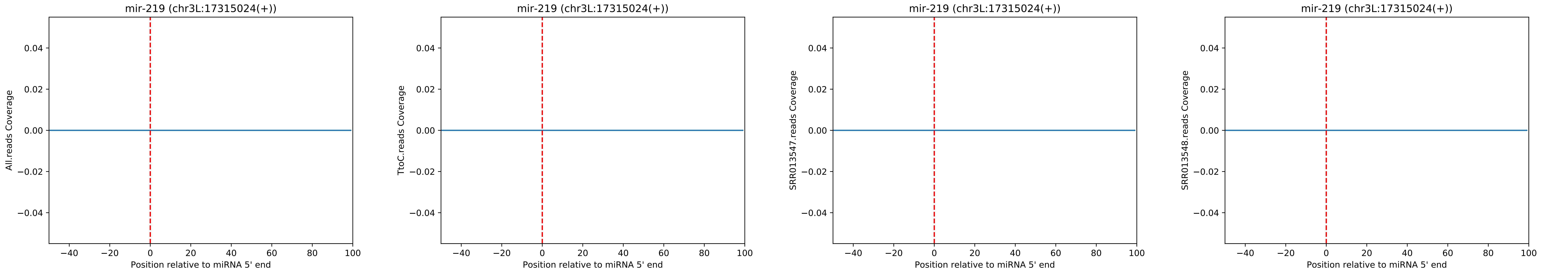
mir-219 (chr3L:17315024(+))
All.reads Coverage
TtoC.reads Coverage
SRR013547.reads Coverage
SRR013548.reads Coverage
0.04
0.02
0.00
−0.02
−0.04
−40
−20
0
20
40
60
80
100
Position relative to miRNA 5' end

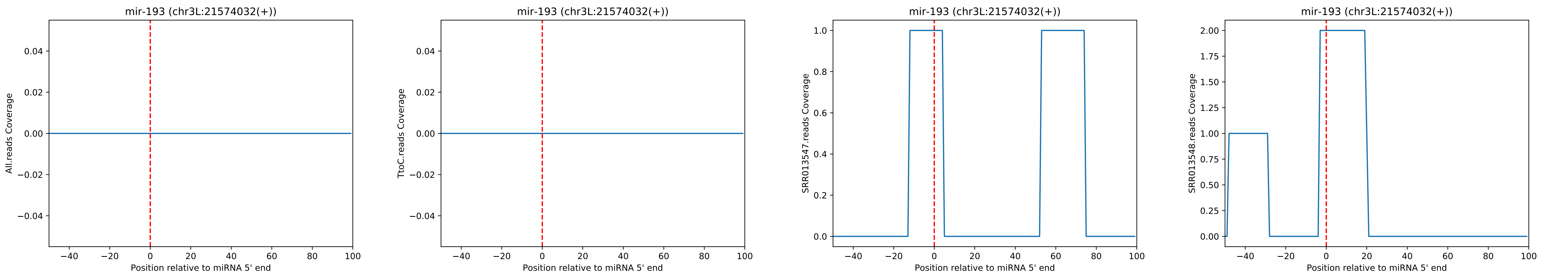

mir-193 (chr3L:21574032(+))
All.reads Coverage
Position relative to miRNA 5' end
mir-193 (chr3L:21574032(+))
TtoC.reads Coverage
Position relative to miRNA 5' end
mir-193 (chr3L:21574032(+))
SRR013547.reads Coverage
Position relative to miRNA 5' end
mir-193 (chr3L:21574032(+))
SRR013548.reads Coverage
Position relative to miRNA 5' end

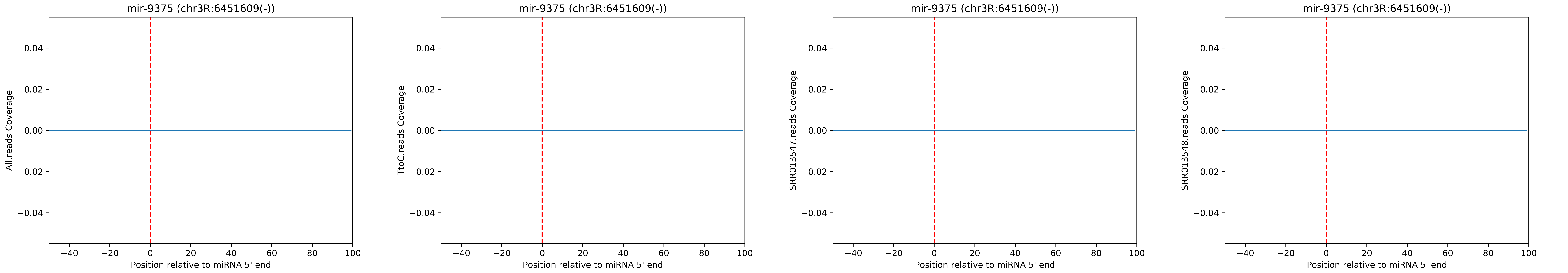
mir-9375 (chr3R:6451609(-))
All.reads Coverage
Position relative to miRNA 5' end
mir-9375 (chr3R:6451609(-))
TtoC.reads Coverage
Position relative to miRNA 5' end
mir-9375 (chr3R:6451609(-))
SRR013547.reads Coverage
Position relative to miRNA 5' end
mir-9375 (chr3R:6451609(-))
SRR013548.reads Coverage
Position relative to miRNA 5' end

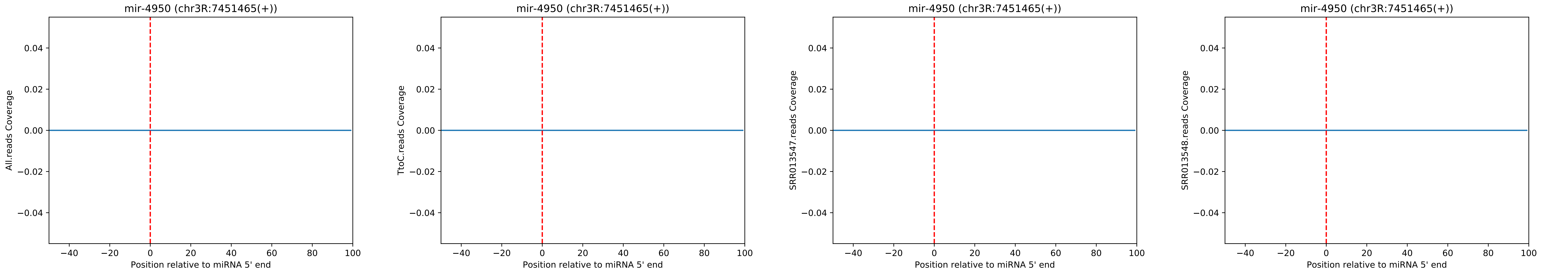
mir-4950 (chr3R:7451465(+))
All.reads Coverage
TtoC.reads Coverage
SRR013547.reads Coverage
SRR013548.reads Coverage
0.04
0.02
0.00
−0.02
−0.04
−40
−20
0
20
40
60
80
100
Position relative to miRNA 5' end

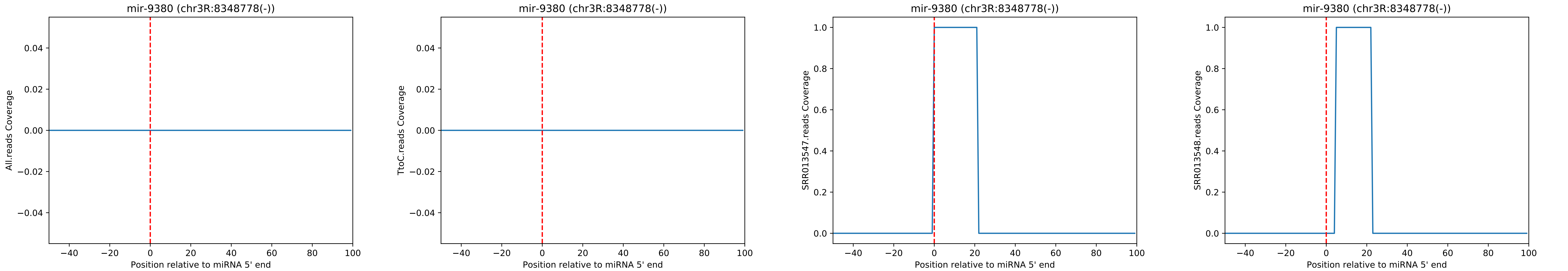
mir-9380 (chr3R:8348778(-))
All.reads Coverage
TtoC.reads Coverage
SRR013547.reads Coverage
SRR013548.reads Coverage
Position relative to miRNA 5' end
0.04
0.02
0.00
−0.02
−0.04
1.0
0.8
0.6
0.4
0.2
0.0
−40
−20
0
20
40
60
80
100

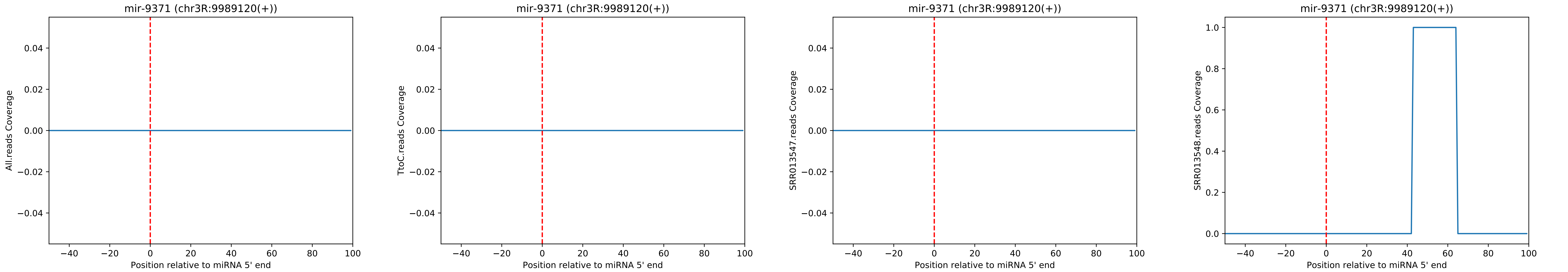

mir-9371 (chr3R:9989120(+))
All.reads Coverage
Position relative to miRNA 5' end
mir-9371 (chr3R:9989120(+))
TtoC.reads Coverage
Position relative to miRNA 5' end
mir-9371 (chr3R:9989120(+))
SRR013547.reads Coverage
Position relative to miRNA 5' end
mir-9371 (chr3R:9989120(+))
SRR013548.reads Coverage
Position relative to miRNA 5' end

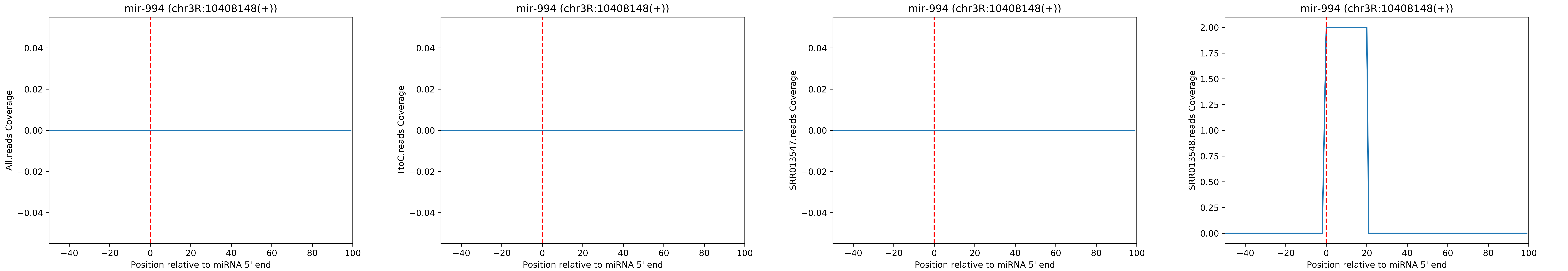
mir-994 (chr3R:10408148(+))
All.reads Coverage
Position relative to miRNA 5' end
mir-994 (chr3R:10408148(+))
TtoC.reads Coverage
Position relative to miRNA 5' end
mir-994 (chr3R:10408148(+))
SRR013547.reads Coverage
Position relative to miRNA 5' end
mir-994 (chr3R:10408148(+))
SRR013548.reads Coverage
Position relative to miRNA 5' end

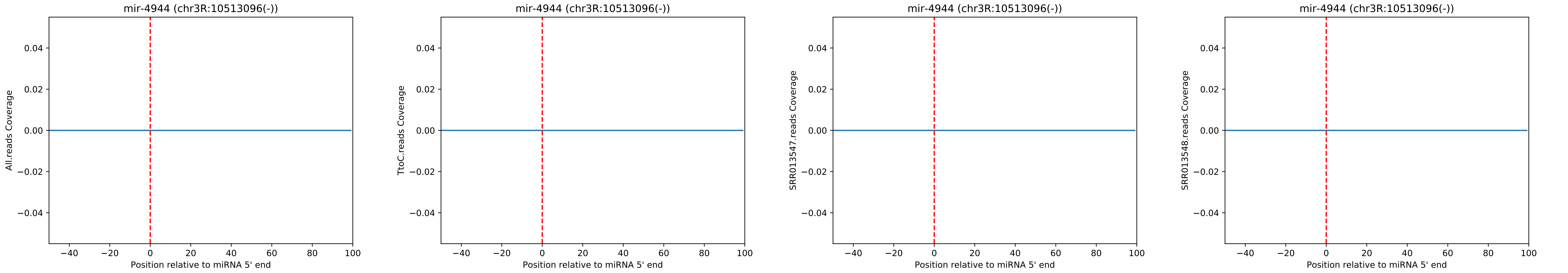

mir-4944 (chr3R:10513096(-))
All.reads Coverage
Position relative to miRNA 5' end
mir-4944 (chr3R:10513096(-))
TtoC.reads Coverage
Position relative to miRNA 5' end
mir-4944 (chr3R:10513096(-))
SRR013547.reads Coverage
Position relative to miRNA 5' end
mir-4944 (chr3R:10513096(-))
SRR013548.reads Coverage
Position relative to miRNA 5' end

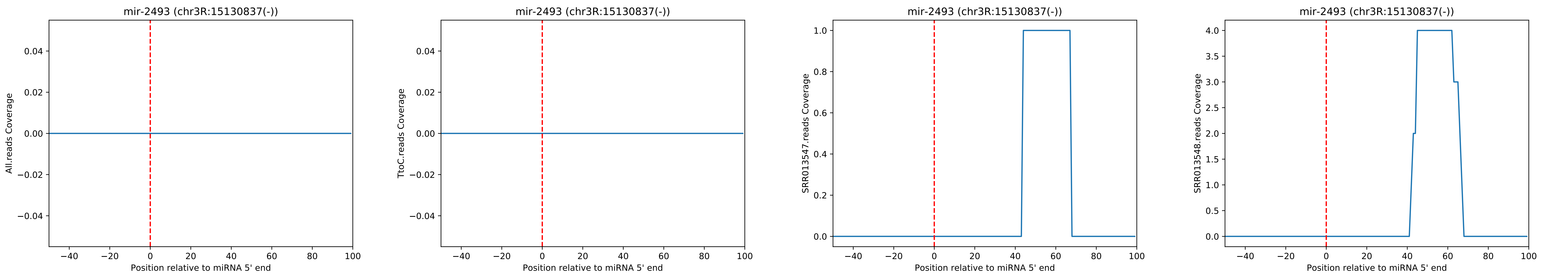
mir-2493 (chr3R:15130837(-))
All.reads Coverage
Position relative to miRNA 5' end
mir-2493 (chr3R:15130837(-))
TtoC.reads Coverage
Position relative to miRNA 5' end
mir-2493 (chr3R:15130837(-))
SRR013547.reads Coverage
Position relative to miRNA 5' end
mir-2493 (chr3R:15130837(-))
SRR013548.reads Coverage
Position relative to miRNA 5' end

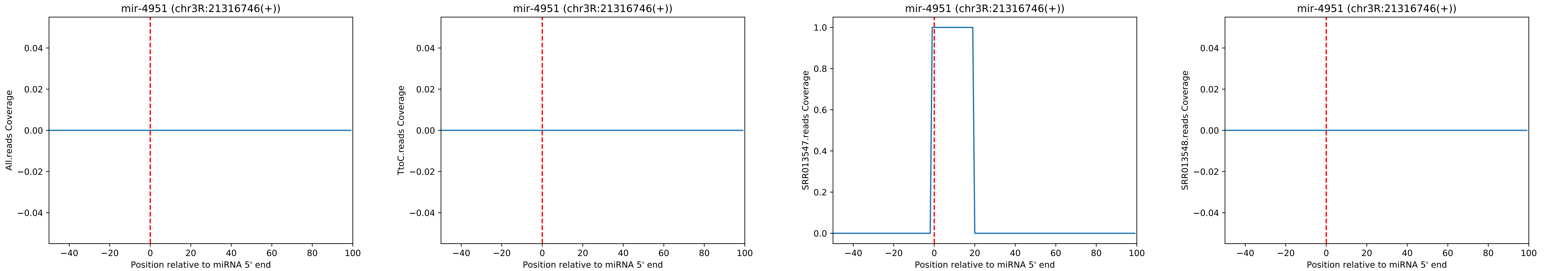

mir-4951 (chr3R:21316746(+))
All.reads Coverage
0.04
0.02
0.00
−0.02
−0.04
−40
−20
0
20
40
60
80
100
Position relative to miRNA 5' end
mir-4951 (chr3R:21316746(+))
TtoC.reads Coverage
Position relative to miRNA 5' end
mir-4951 (chr3R:21316746(+))
SRR013547.reads Coverage
1.0
0.8
0.6
0.4
0.2
0.0
Position relative to miRNA 5' end
mir-4951 (chr3R:21316746(+))
SRR013548.reads Coverage
Position relative to miRNA 5' end

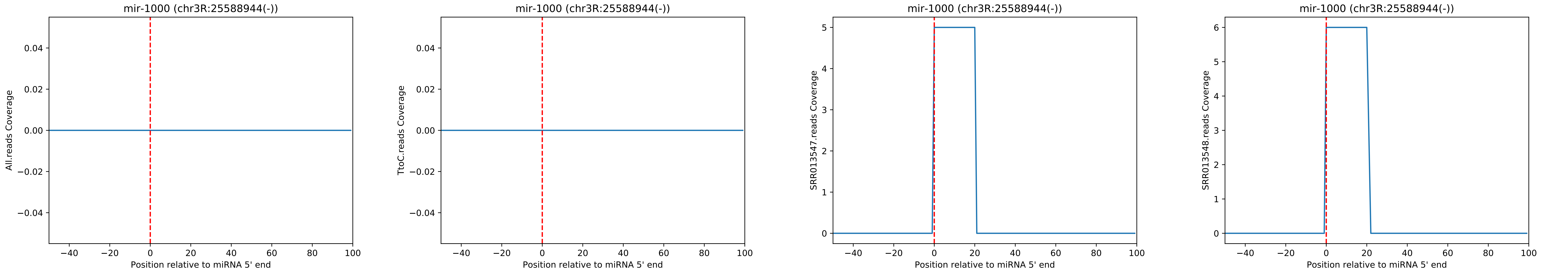

mir-1000 (chr3R:25588944(-))
All.reads Coverage
TtoC.reads Coverage
SRR013547.reads Coverage
SRR013548.reads Coverage
Position relative to miRNA 5' end

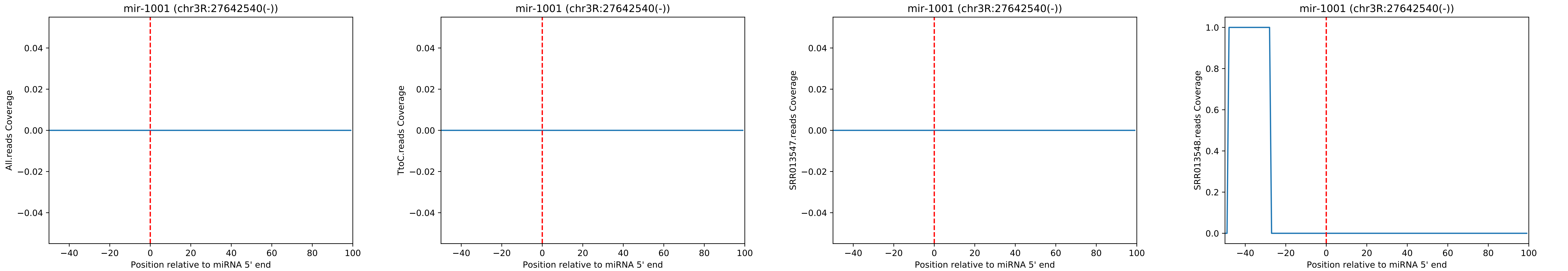

mir-1001 (chr3R:27642540(-))
All.reads Coverage
TtoC.reads Coverage
SRR013547.reads Coverage
SRR013548.reads Coverage
Position relative to miRNA 5' end

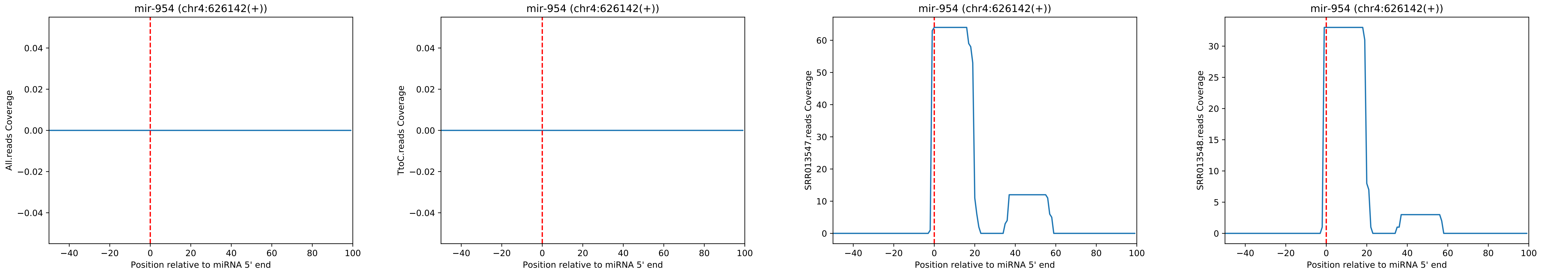
mir-954 (chr4:626142(+))
All.reads Coverage
Position relative to miRNA 5' end
mir-954 (chr4:626142(+))
TtoC.reads Coverage
Position relative to miRNA 5' end
mir-954 (chr4:626142(+))
SRR013547.reads Coverage
Position relative to miRNA 5' end
mir-954 (chr4:626142(+))
SRR013548.reads Coverage
Position relative to miRNA 5' end

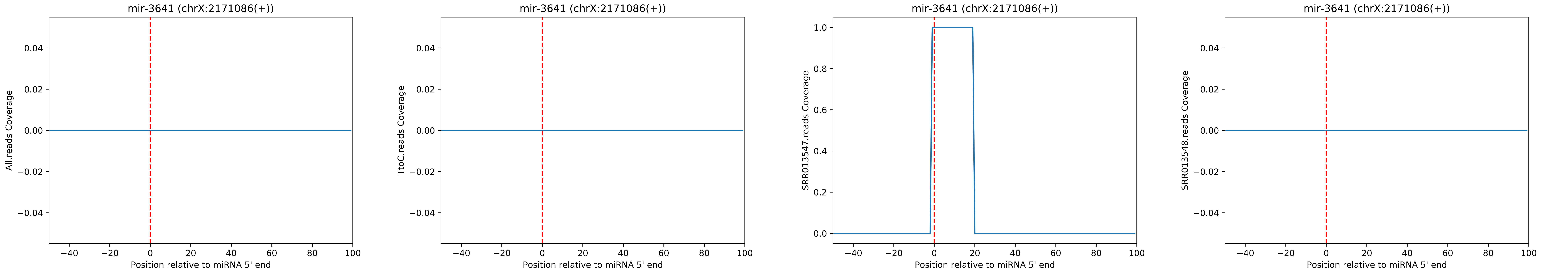
mir-3641 (chrX:2171086(+))
All.reads Coverage
TtoC.reads Coverage
SRR013547.reads Coverage
SRR013548.reads Coverage
Position relative to miRNA 5' end

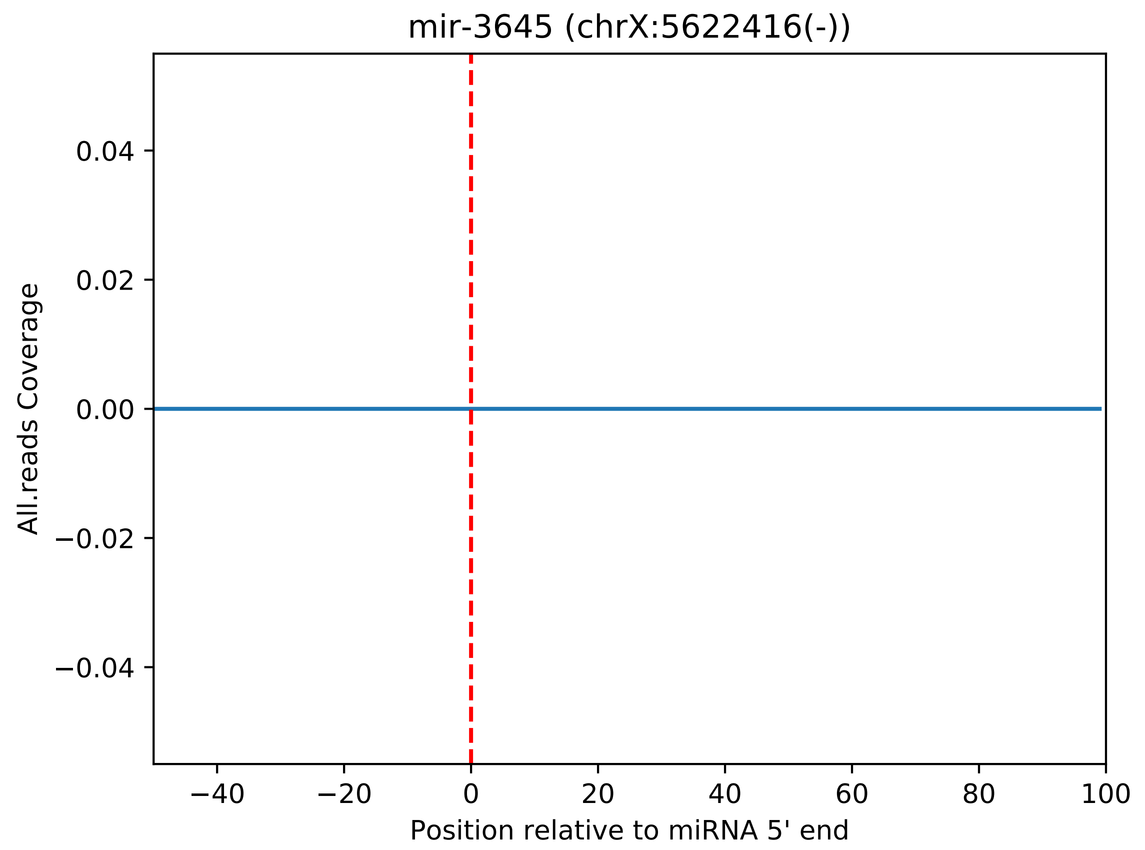
mir-3645 (chrX:5622416(-))
All.reads Coverage
0.04
0.02
0.00
−0.02
−0.04
−40
−20
0
20
40
60
80
100
Position relative to miRNA 5' end

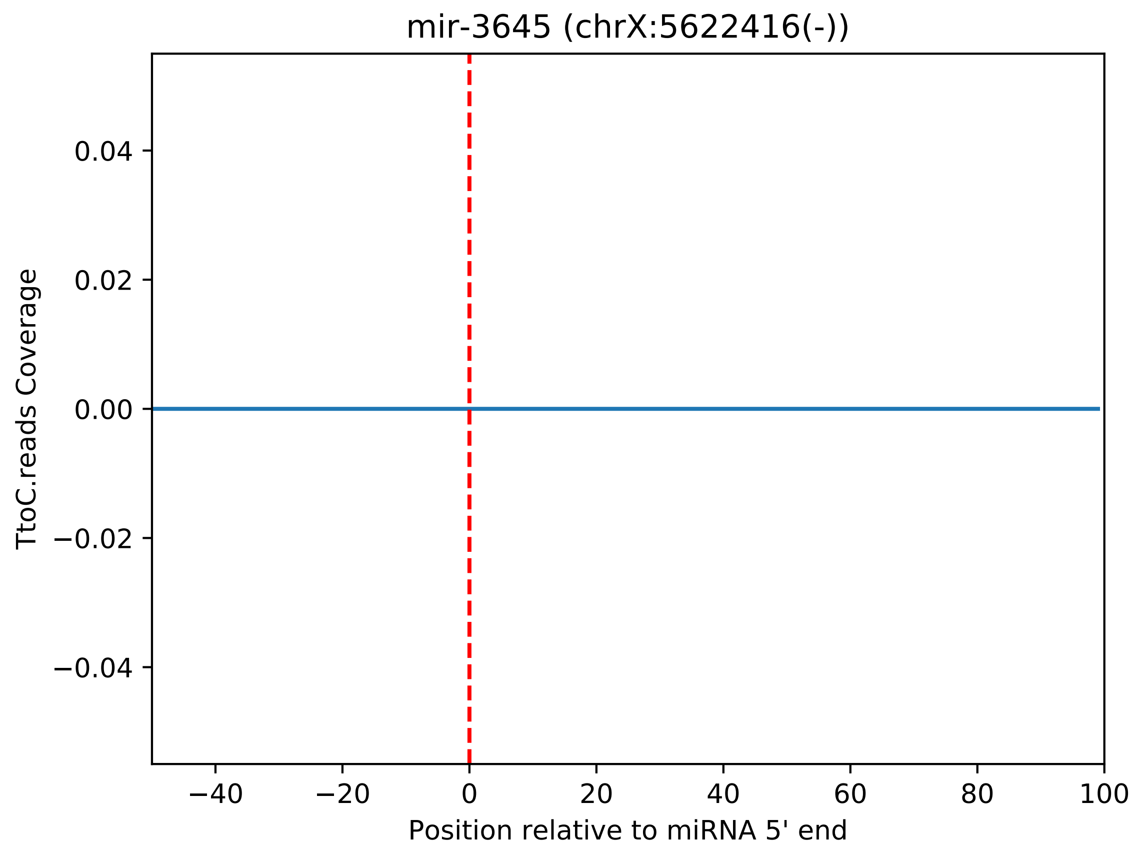
mir-3645 (chrX:5622416(-))
TtoC.reads Coverage
0.04
0.02
0.00
−0.02
−0.04
−40
−20
0
20
40
60
80
100
Position relative to miRNA 5' end

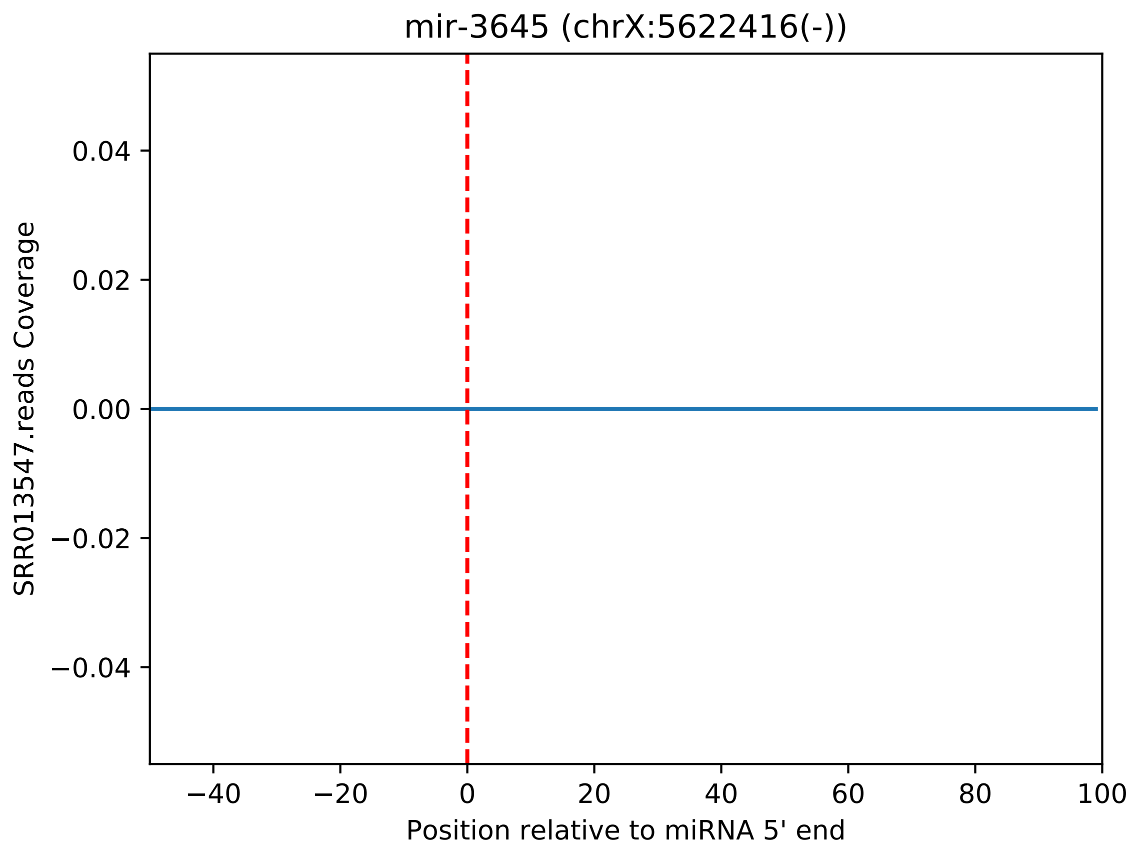
mir-3645 (chrX:5622416(-))
SRR013547.reads Coverage
0.04
0.02
0.00
−0.02
−0.04
−40
−20
0
20
40
60
80
100
Position relative to miRNA 5' end

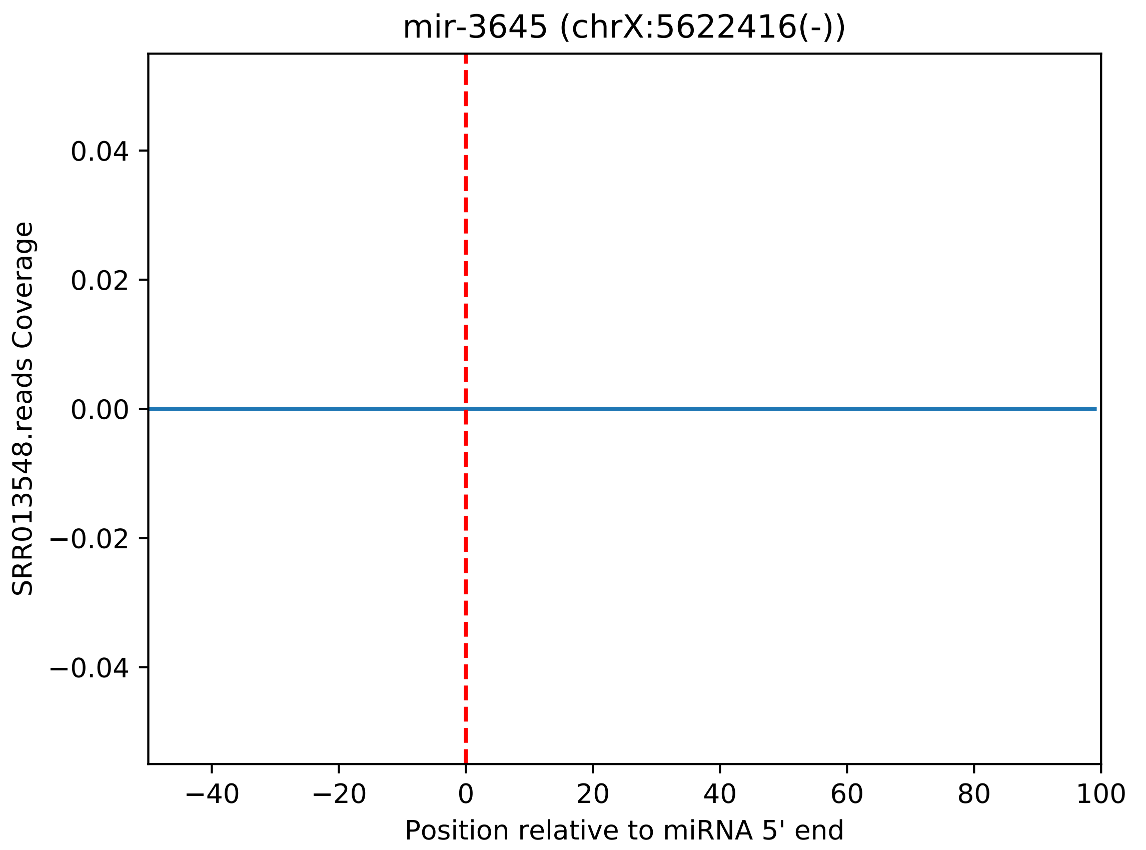
mir-3645 (chrX:5622416(-))
SRR013548.reads Coverage
0.04
0.02
0.00
−0.02
−0.04
−40
−20
0
20
40
60
80
100
Position relative to miRNA 5' end

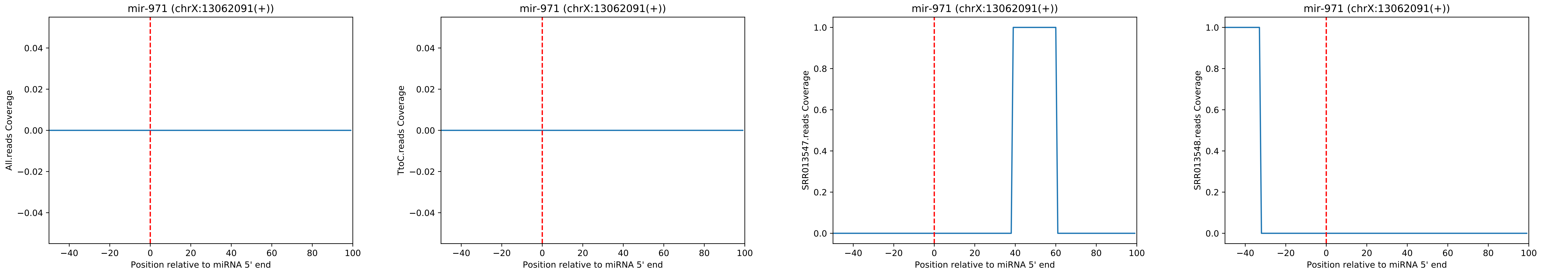

mir-971 (chrX:13062091(+))
All.reads Coverage
Position relative to miRNA 5' end
mir-971 (chrX:13062091(+))
TtoC.reads Coverage
Position relative to miRNA 5' end
mir-971 (chrX:13062091(+))
SRR013547.reads Coverage
Position relative to miRNA 5' end
mir-971 (chrX:13062091(+))
SRR013548.reads Coverage
Position relative to miRNA 5' end

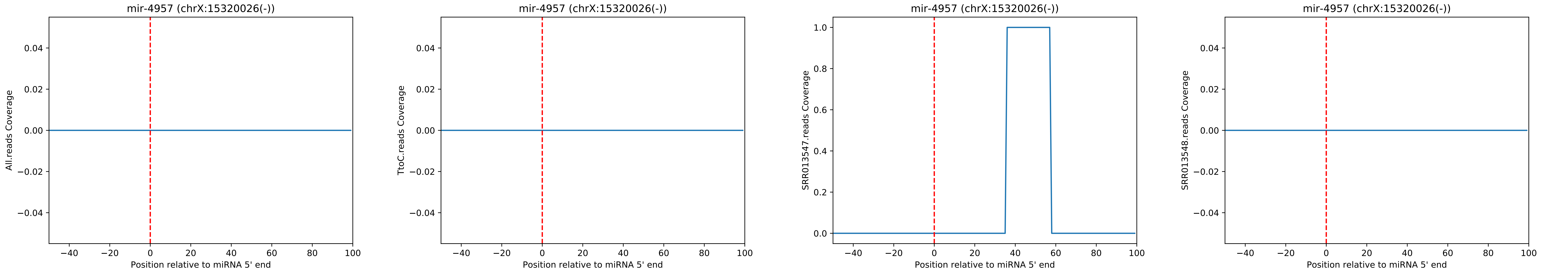

mir-4957 (chrX:15320026(-))
All.reads Coverage
Position relative to miRNA 5' end
mir-4957 (chrX:15320026(-))
TtoC.reads Coverage
Position relative to miRNA 5' end
mir-4957 (chrX:15320026(-))
SRR013547.reads Coverage
Position relative to miRNA 5' end
mir-4957 (chrX:15320026(-))
SRR013548.reads Coverage
Position relative to miRNA 5' end

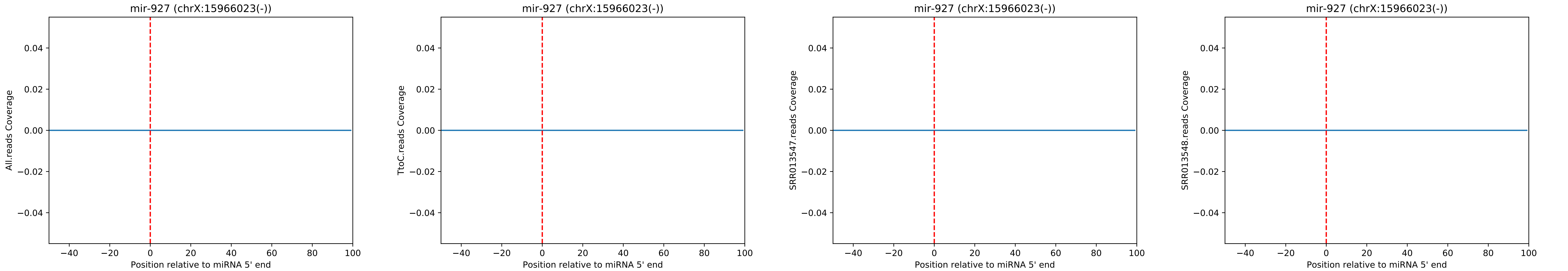
mir-927 (chrX:15966023(-))
All.reads Coverage
Position relative to miRNA 5' end
mir-927 (chrX:15966023(-))
TtoC.reads Coverage
Position relative to miRNA 5' end
mir-927 (chrX:15966023(-))
SRR013547.reads Coverage
Position relative to miRNA 5' end
mir-927 (chrX:15966023(-))
SRR013548.reads Coverage
Position relative to miRNA 5' end

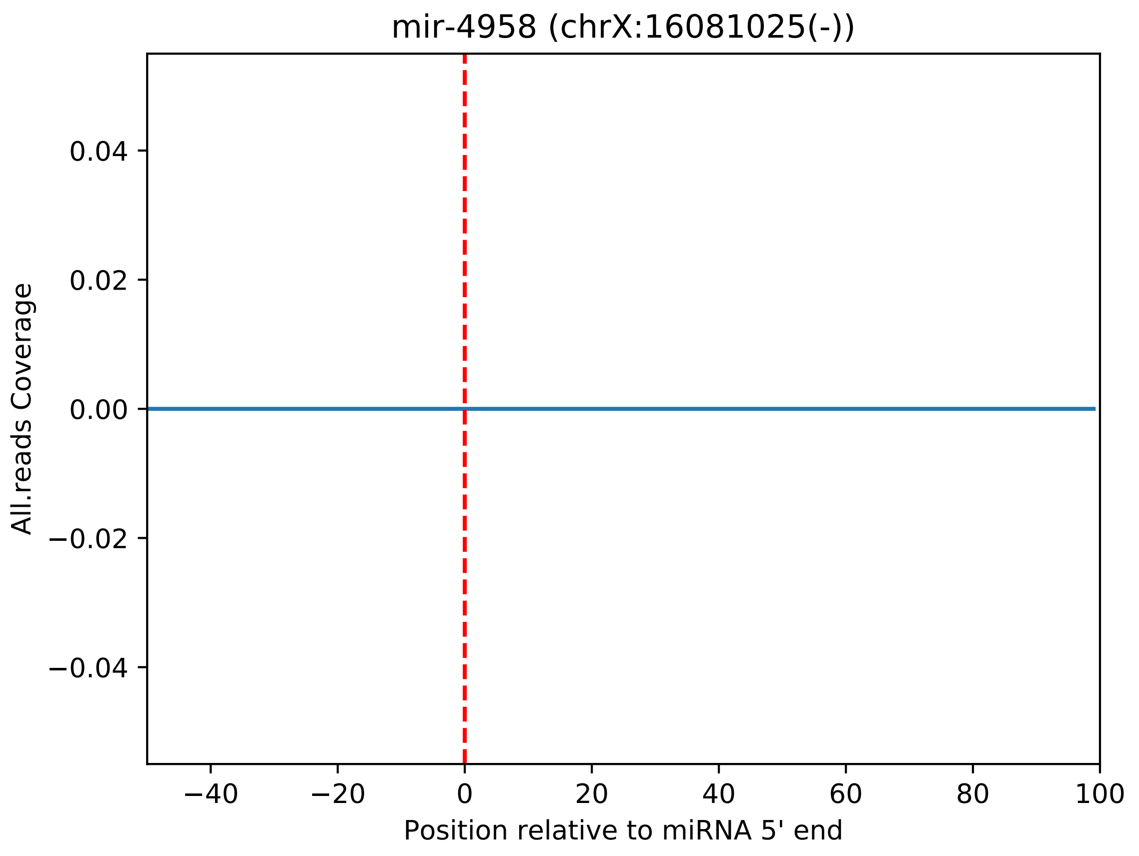

mir-4958 (chrX:16081025(-))
All.reads Coverage
Position relative to miRNA 5' end


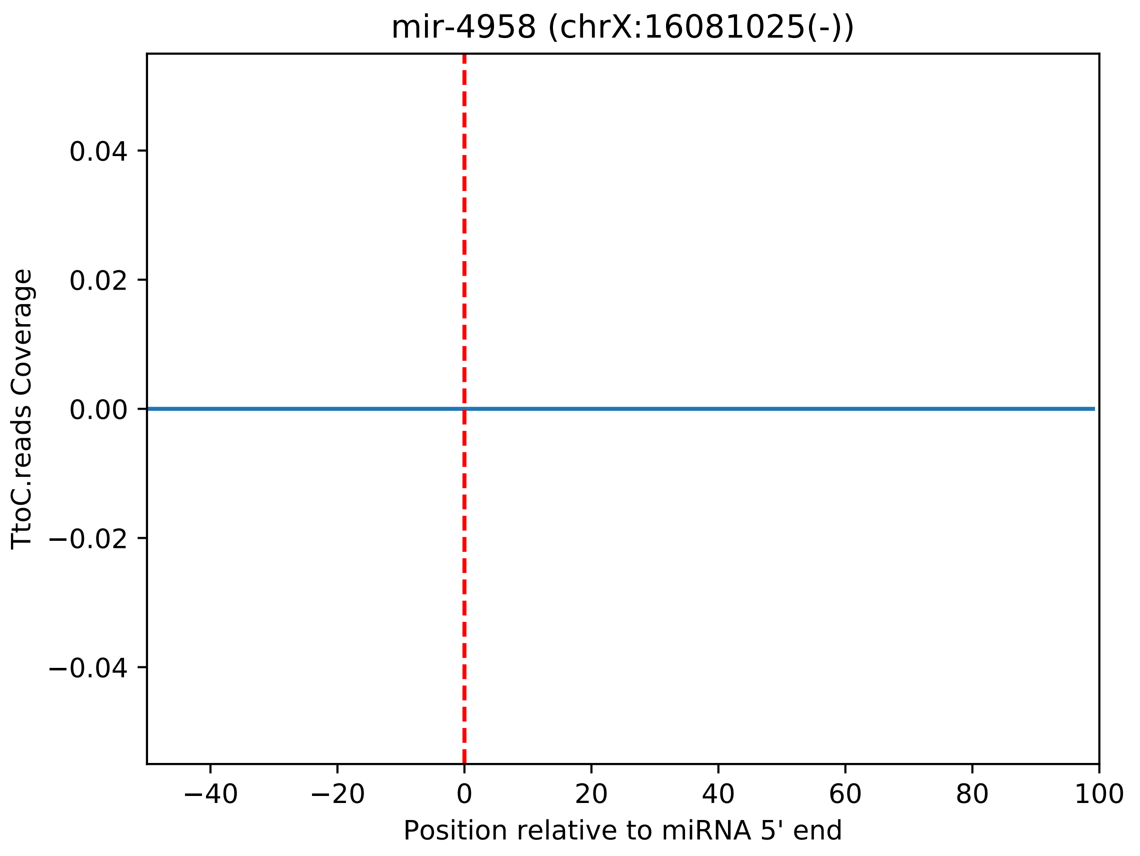

mir-4958 (chrX:16081025(-))
TtoC.reads Coverage
Position relative to miRNA 5' end


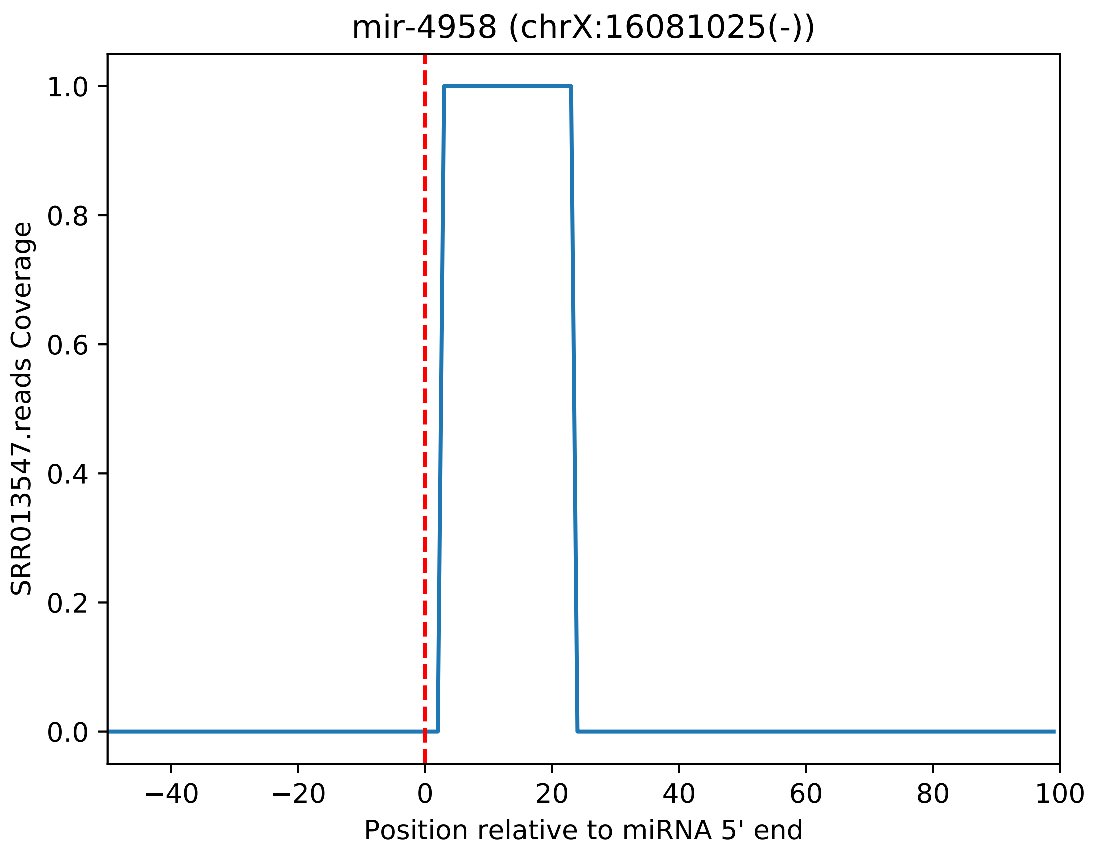

mir-4958 (chrX:16081025(-))
SRR013547.reads Coverage
Position relative to miRNA 5' end


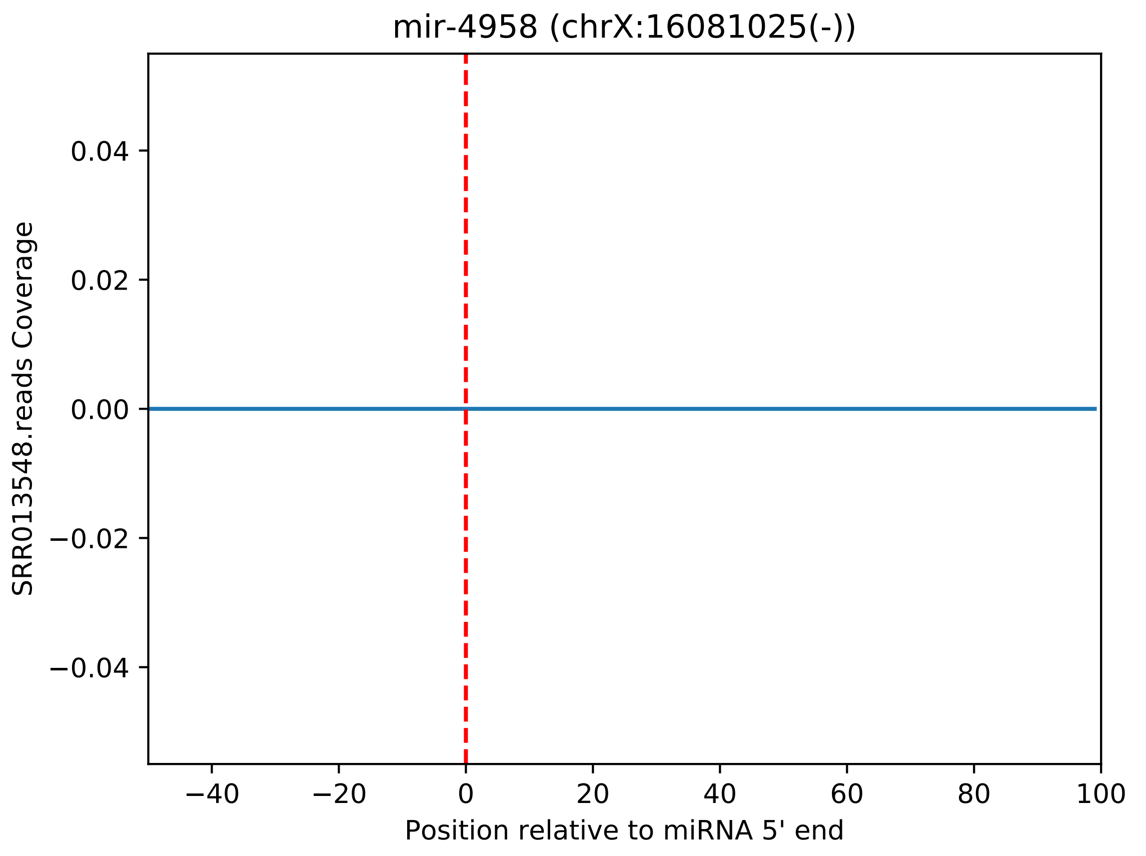

mir-4958 (chrX:16081025(-))
SRR013548.reads Coverage
Position relative to miRNA 5' end

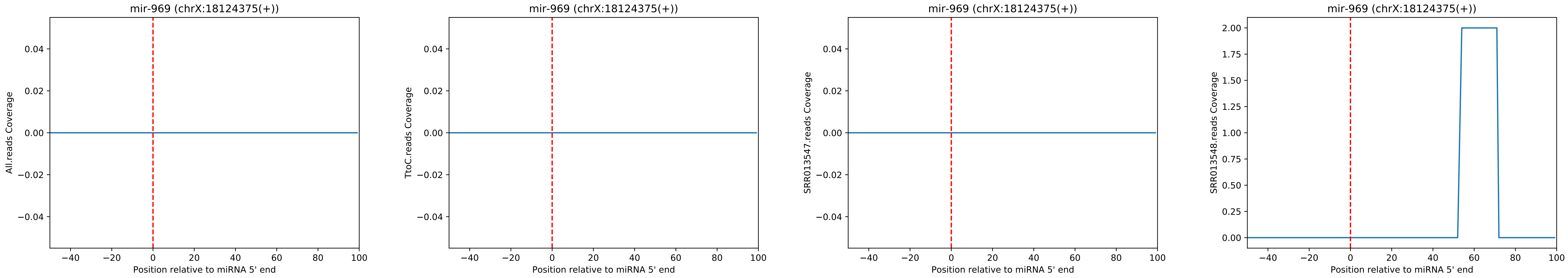
mir-969 (chrX:18124375(+))
All.reads Coverage
TtoC.reads Coverage
SRR013547.reads Coverage
SRR013548.reads Coverage
0.04
0.02
0.00
−0.02
−0.04
2.00
1.75
1.50
1.25
1.00
0.75
0.50
0.25
−40
−20
0
20
40
60
80
100
Position relative to miRNA 5' end
