# Supplementary material for: Exportin-5 binding precedes 5′- and 3′-end processing of tRNA precursors in Drosophila
Source: J Biol Chem. 2024 Aug 2;300(9):107632. doi: 10.1016/j.jbc.2024.107632 (PMC11402290; doi:10.1016/j.jbc.2024.107632)

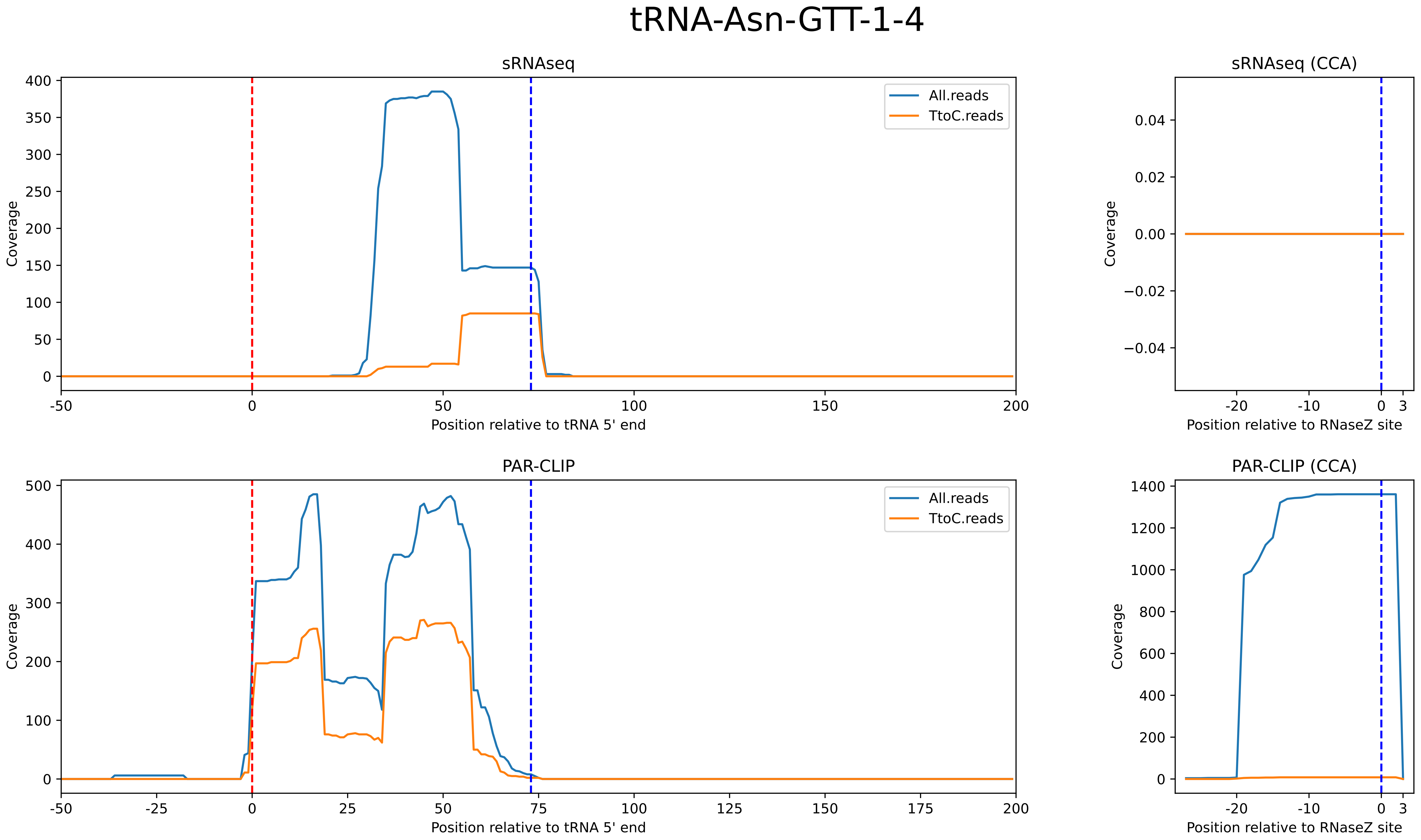
tRNA-Asn-GTT-1-4
sRNAseq
All.reads
TtoC.reads
Coverage
Position relative to tRNA 5' end
sRNAseq (CCA)
Position relative to RNaseZ site
PAR-CLIP
All.reads
TtoC.reads
Coverage
Position relative to tRNA 5' end
PAR-CLIP (CCA)
Coverage
Position relative to RNaseZ site

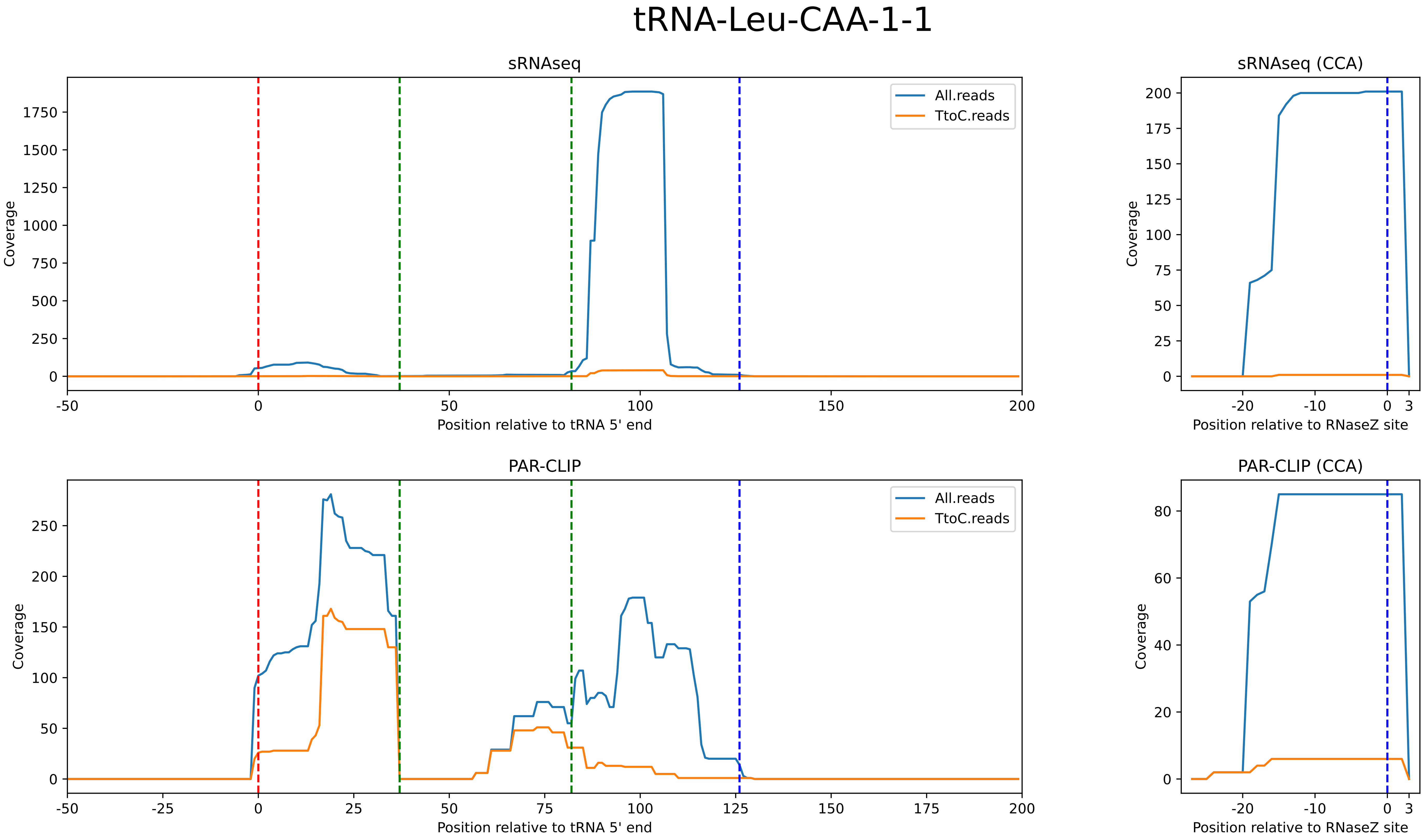

tRNA-Leu-CAA-1-1
sRNAseq
sRNAseq (CCA)
PAR-CLIP
PAR-CLIP (CCA)
All.reads
TtoC.reads
Coverage
Position relative to tRNA 5' end
Position relative to RNaseZ site

# tRNA-Ser-AGA-3-1

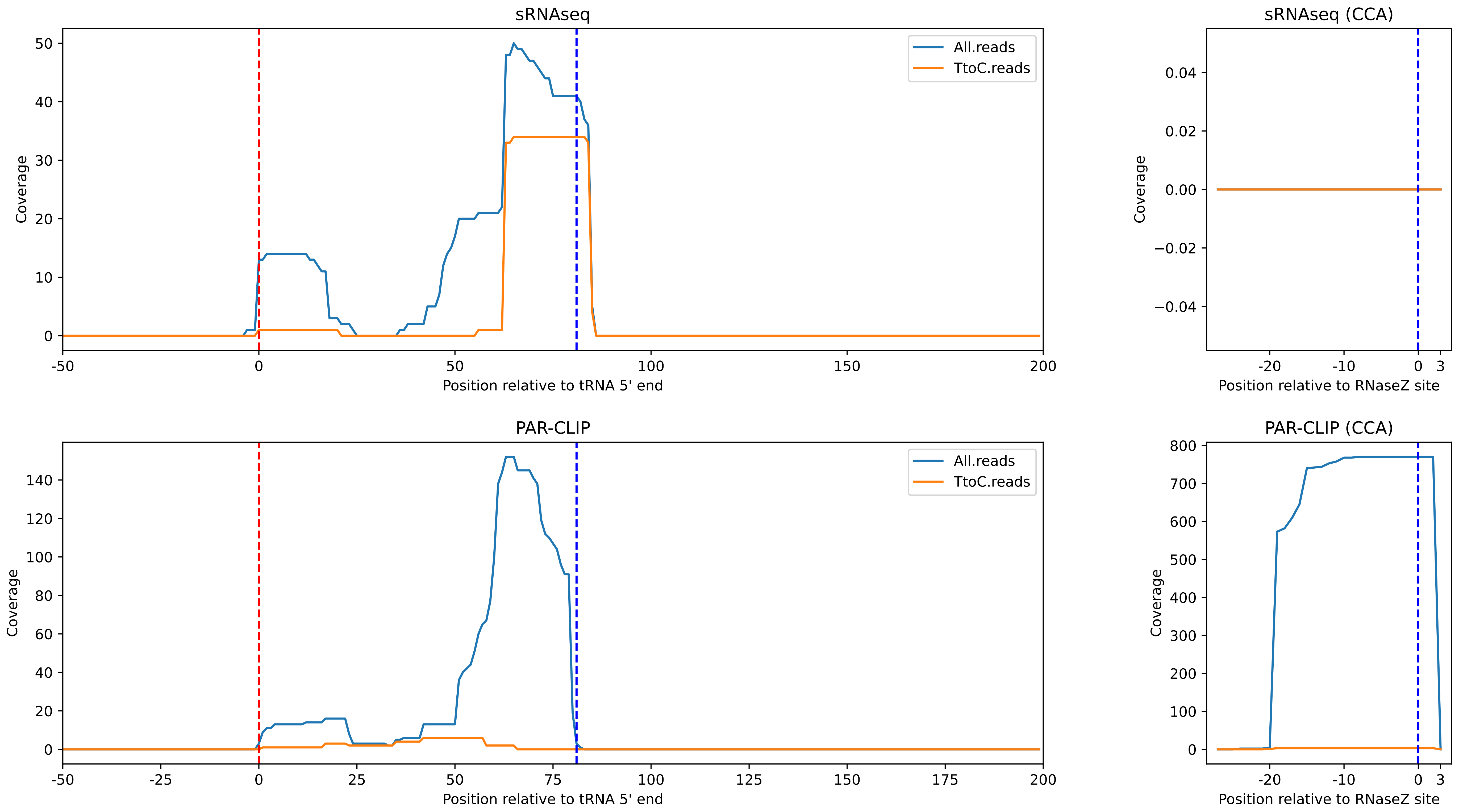

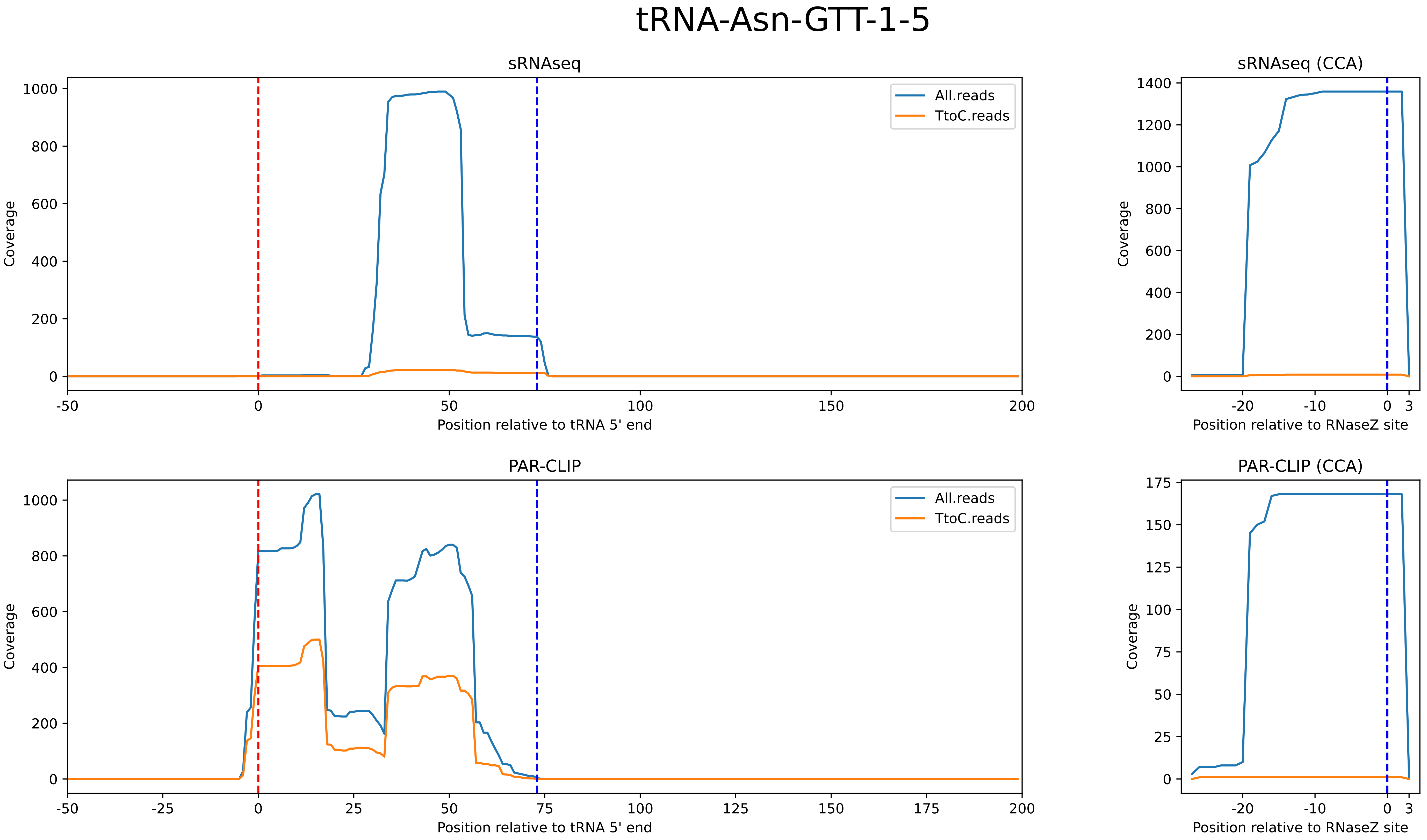

tRNA-Asn-GTT-1-5
sRNAseq
sRNAseq (CCA)
PAR-CLIP
PAR-CLIP (CCA)
All.reads
TtoC.reads
Coverage
Position relative to tRNA 5' end
Position relative to RNaseZ site

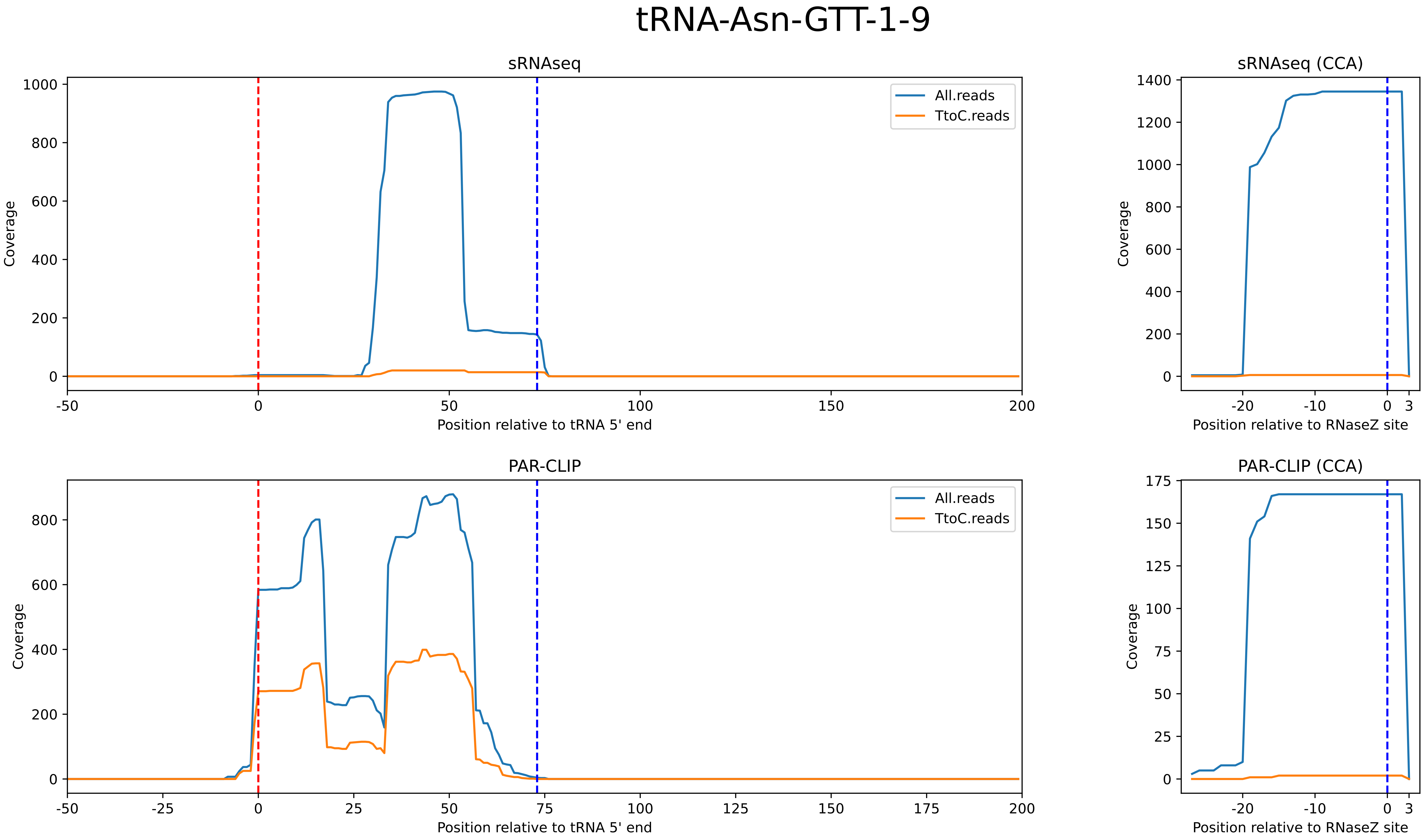

tRNA-Asn-GTT-1-9
sRNAseq
All.reads
TtoC.reads
Coverage
Position relative to tRNA 5' end
sRNAseq (CCA)
Position relative to RNaseZ site
PAR-CLIP
PAR-CLIP (CCA)

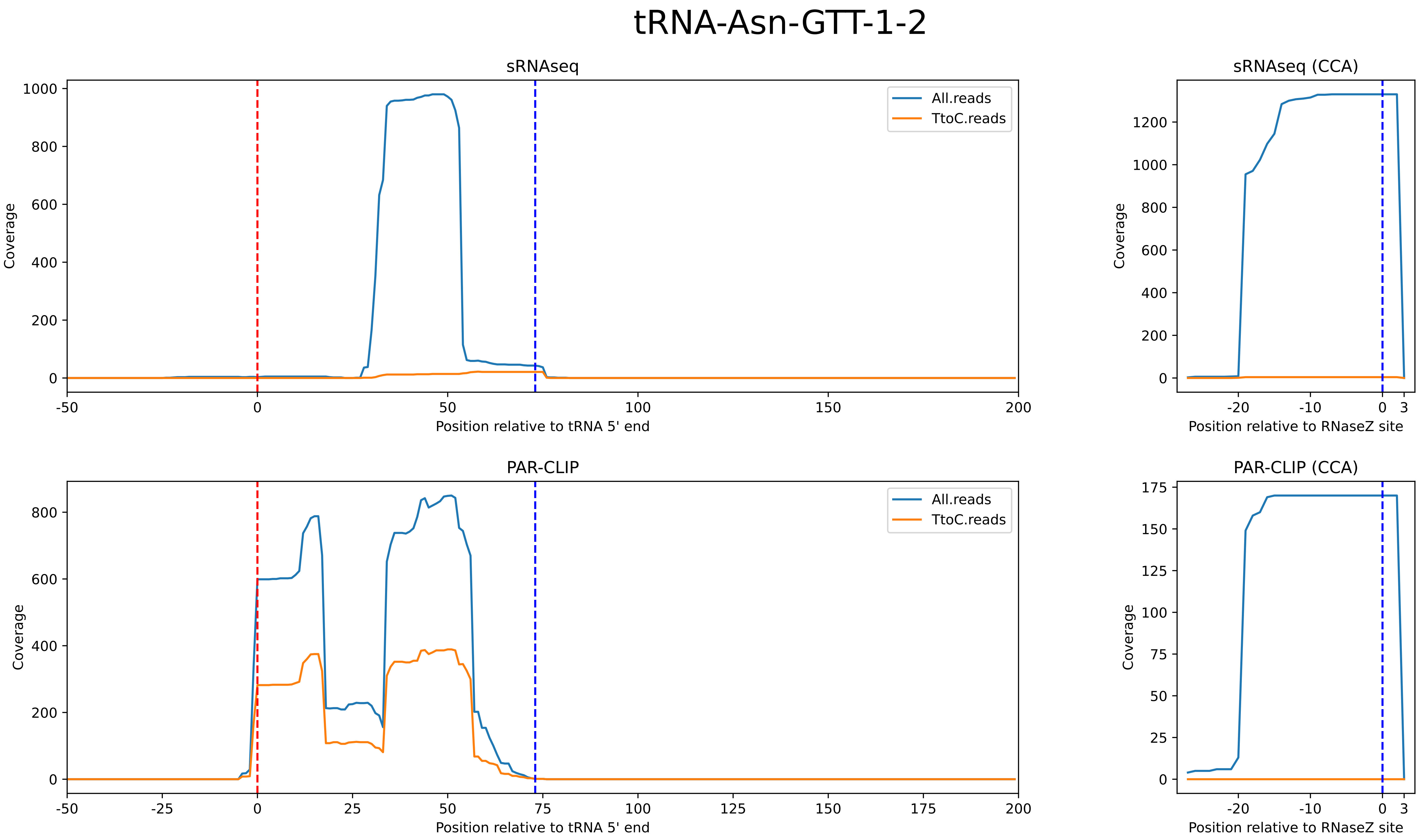

tRNA-Asn-GTT-1-2
sRNAseq
sRNAseq (CCA)
PAR-CLIP
PAR-CLIP (CCA)
All.reads
TtoC.reads
Coverage
Position relative to tRNA 5' end
Position relative to RNaseZ site

# tRNA-Arg-ACG-1-7

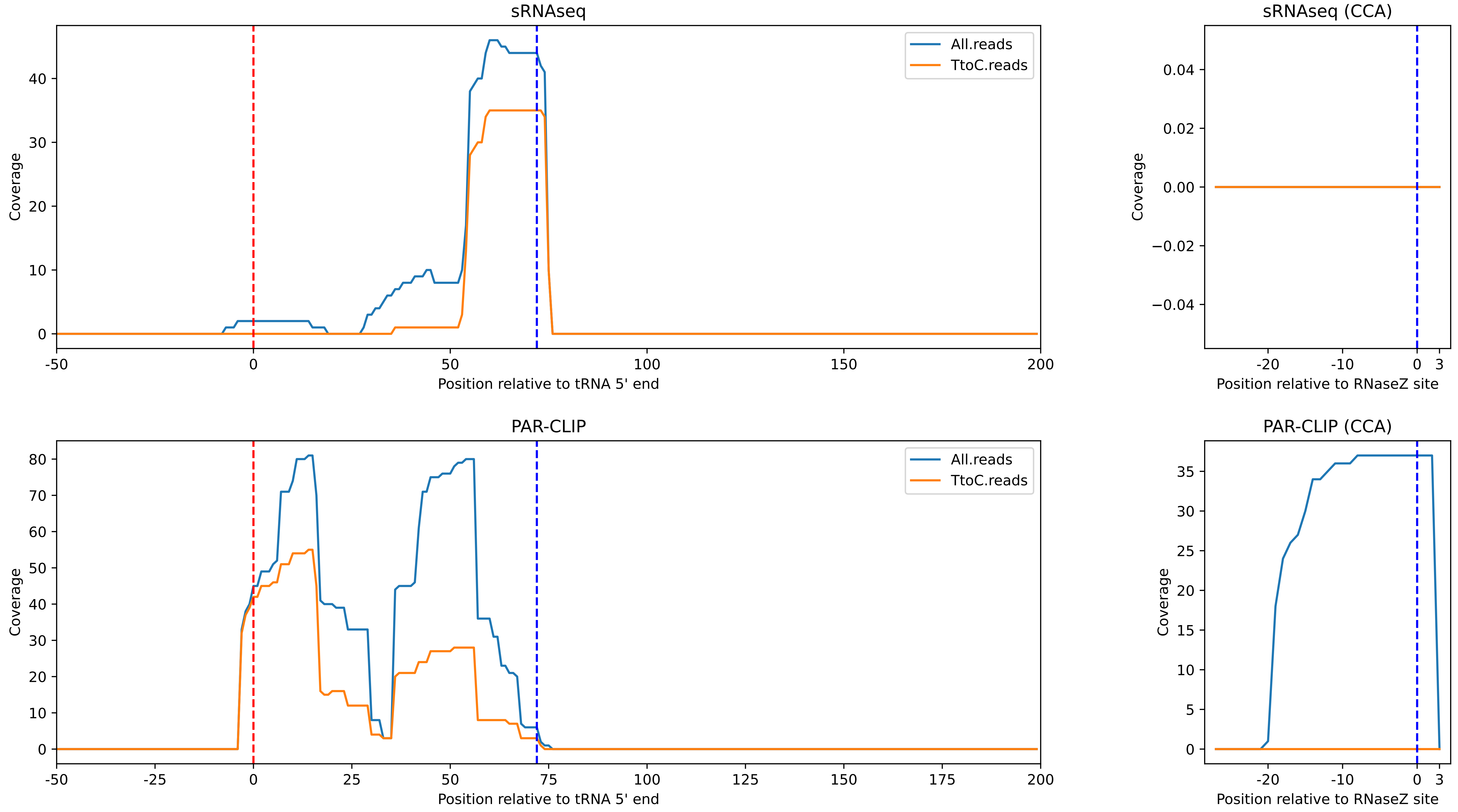

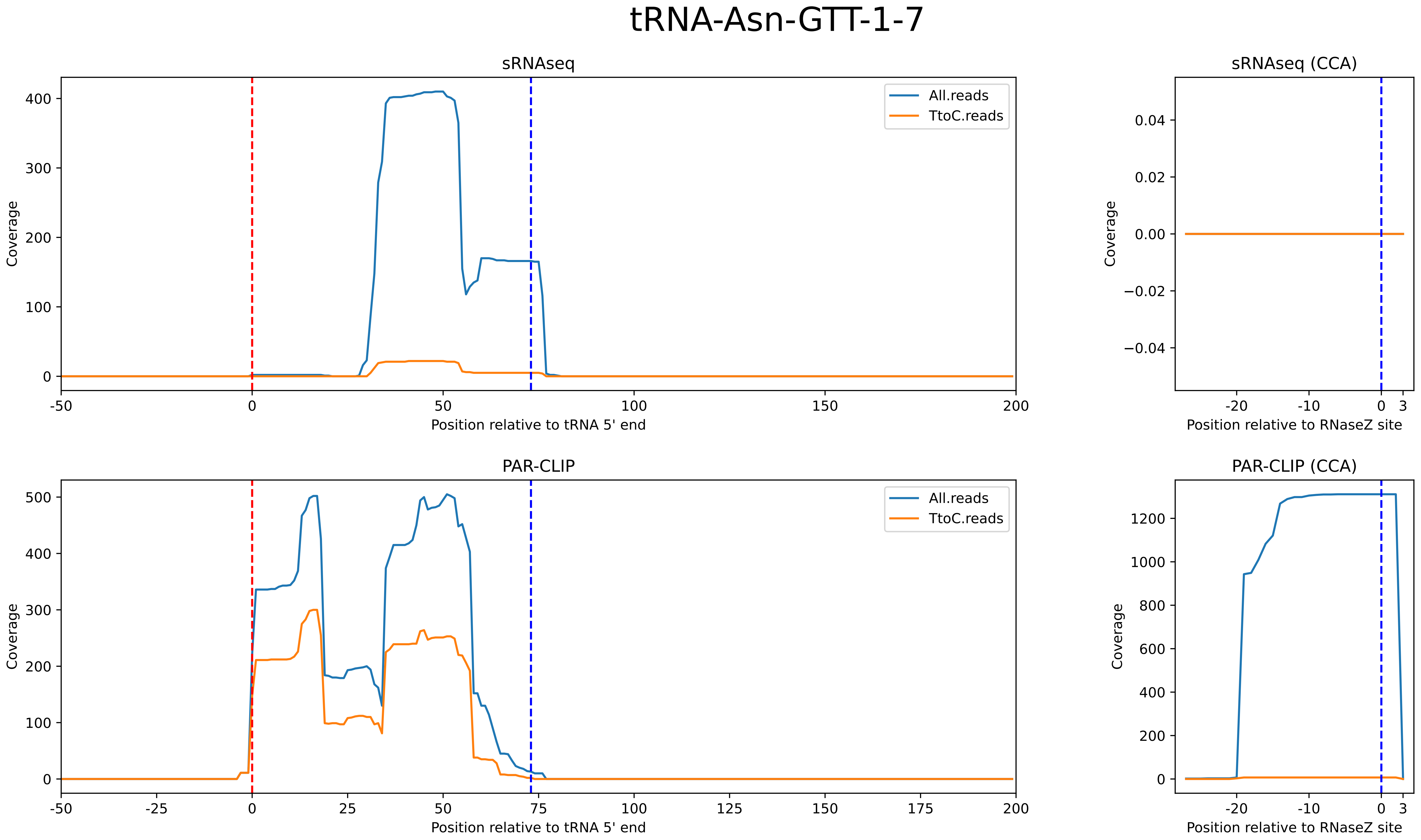
tRNA-Asn-GTT-1-7
sRNAseq
sRNAseq (CCA)
PAR-CLIP
PAR-CLIP (CCA)
All.reads
TtoC.reads
Coverage
Position relative to tRNA 5' end
Position relative to RNaseZ site

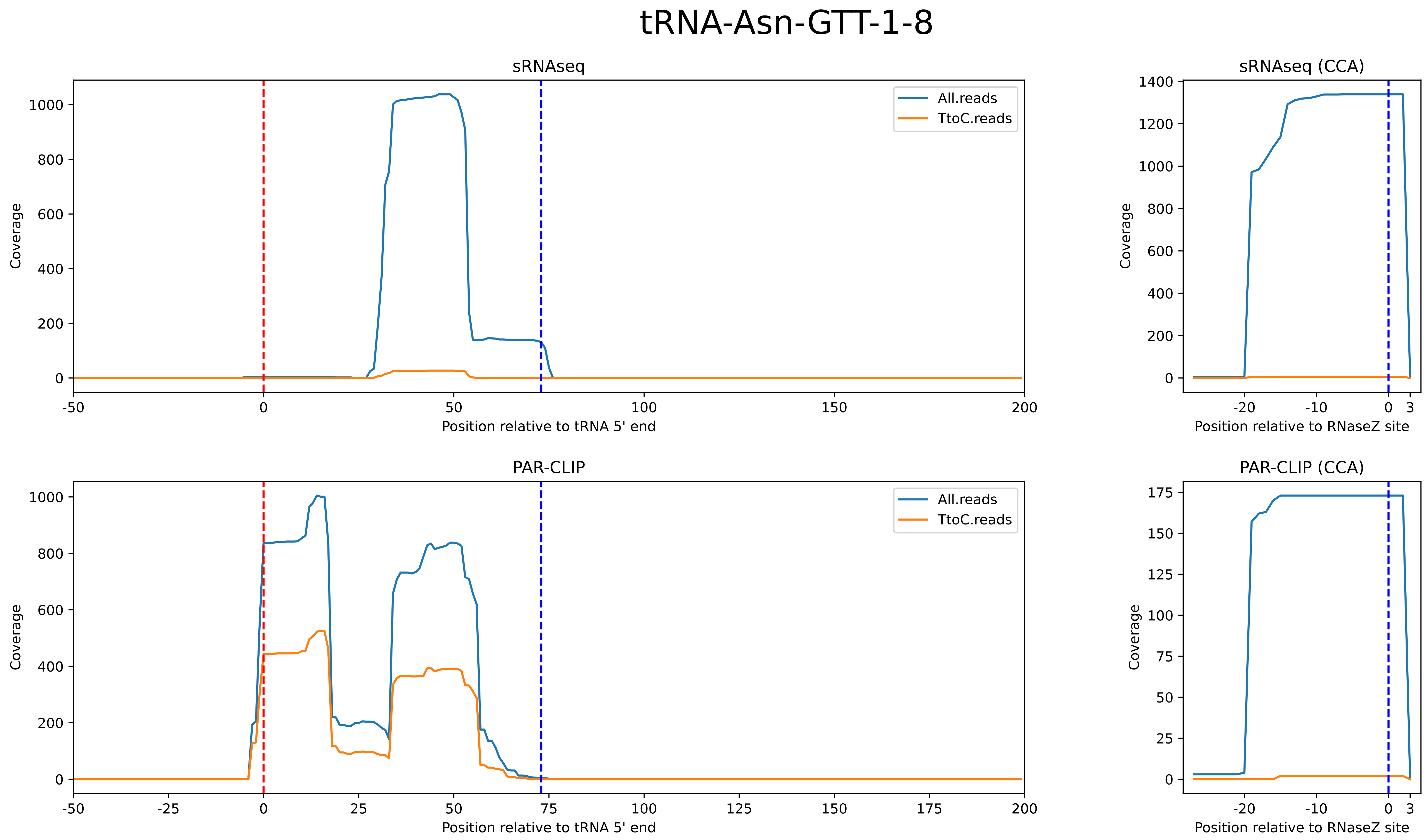
tRNA-Asn-GTT-1-8
sRNAseq
All.reads
TtoC.reads
Coverage
Position relative to tRNA 5' end
sRNAseq (CCA)
Position relative to RNaseZ site
PAR-CLIP
PAR-CLIP (CCA)
-50
-25
0
25
50
75
100
125
150
175
200
-20
-10
3
1400
1200
1000
800
600
400

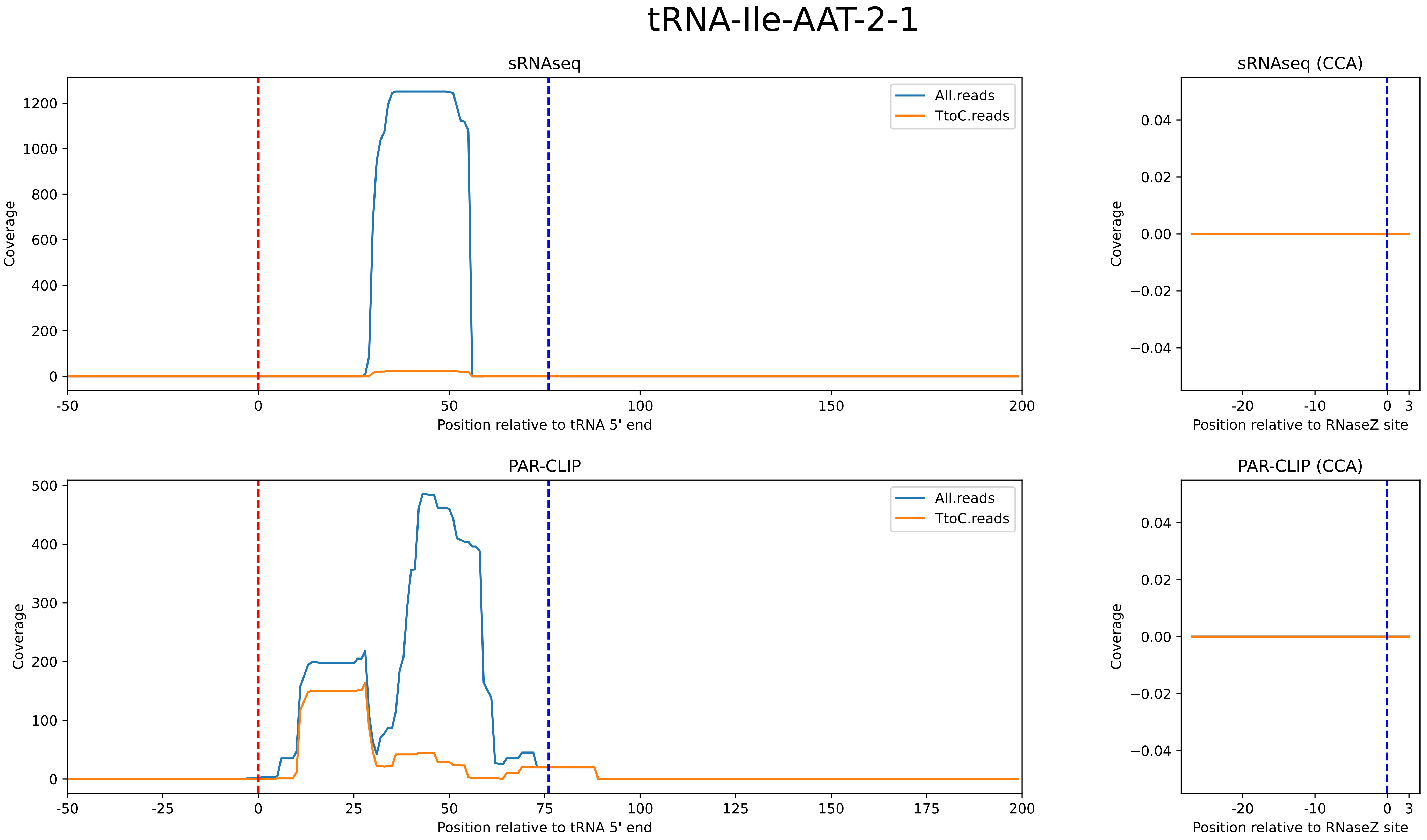

tRNA-Ile-AAT-2-1
sRNAseq
sRNAseq (CCA)
PAR-CLIP
PAR-CLIP (CCA)
All.reads
TtoC.reads
Coverage
Position relative to tRNA 5' end
Position relative to RNaseZ site

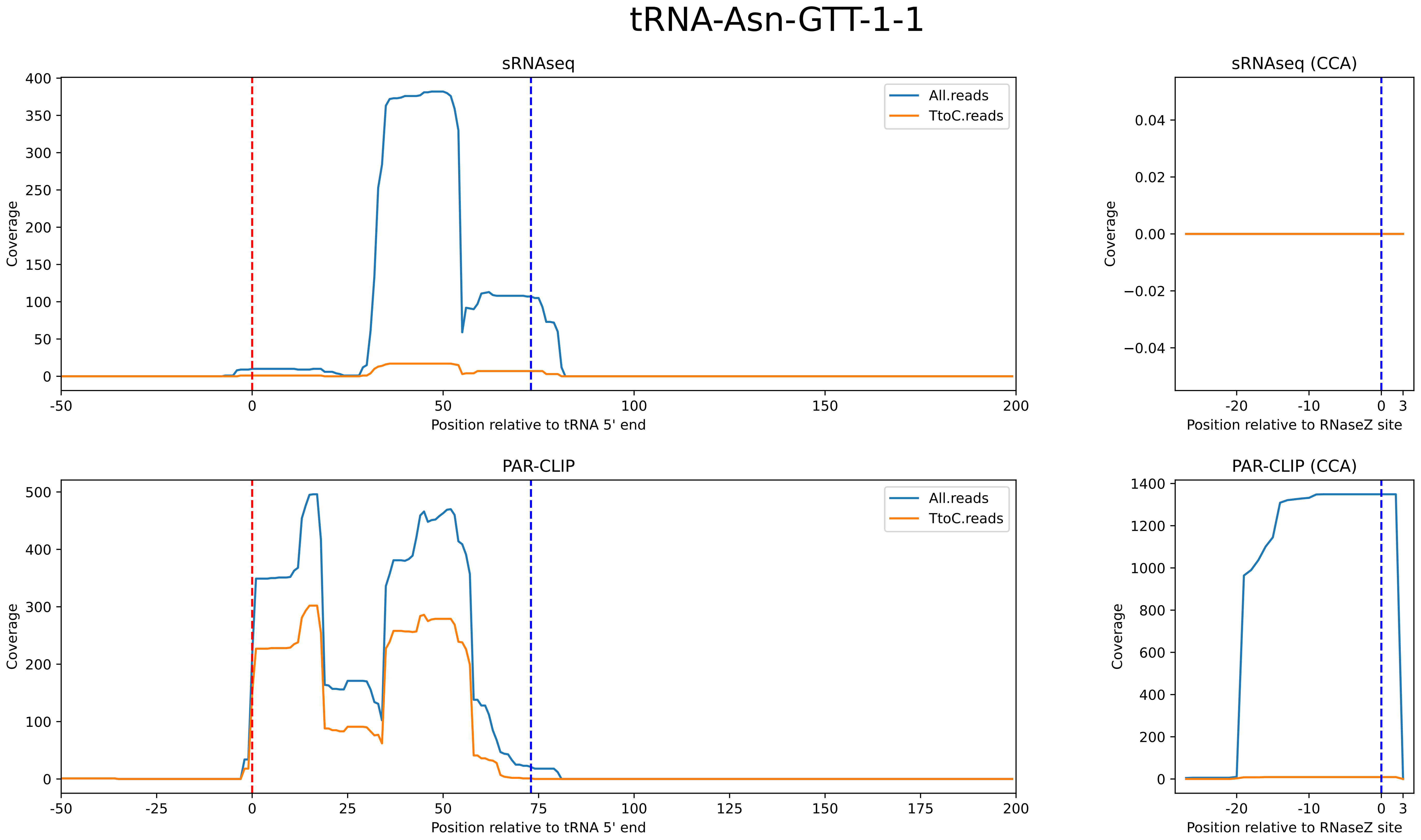

tRNA-Asn-GTT-1-1
sRNAseq
All.reads
TtoC.reads
Coverage
Position relative to tRNA 5' end
sRNAseq (CCA)
Position relative to RNaseZ site
PAR-CLIP
PAR-CLIP (CCA)

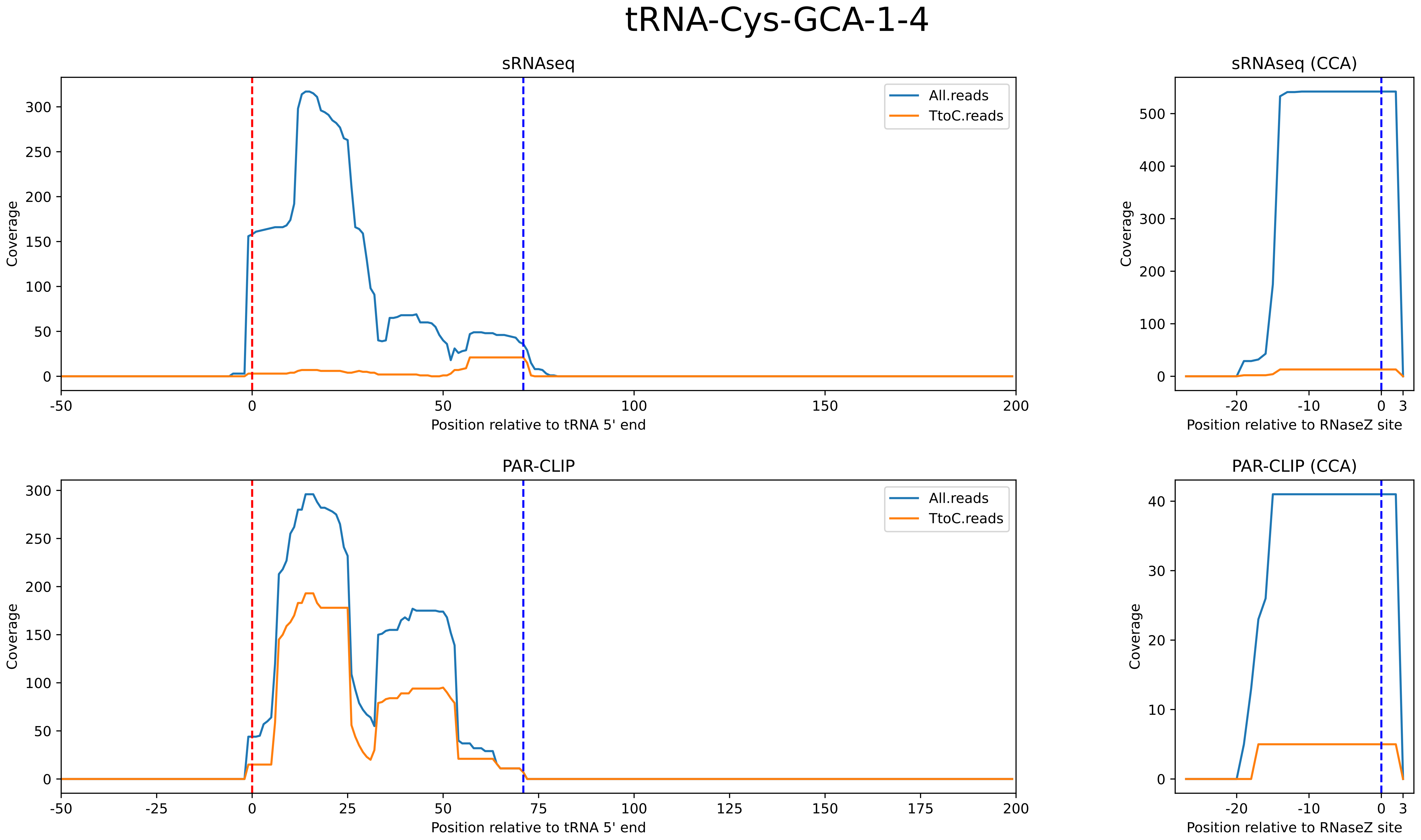

tRNA-Cys-GCA-1-4
sRNAseq
Coverage
Position relative to tRNA 5' end
All.reads
TtoC.reads
sRNAseq (CCA)
Position relative to RNaseZ site
PAR-CLIP
PAR-CLIP (CCA)

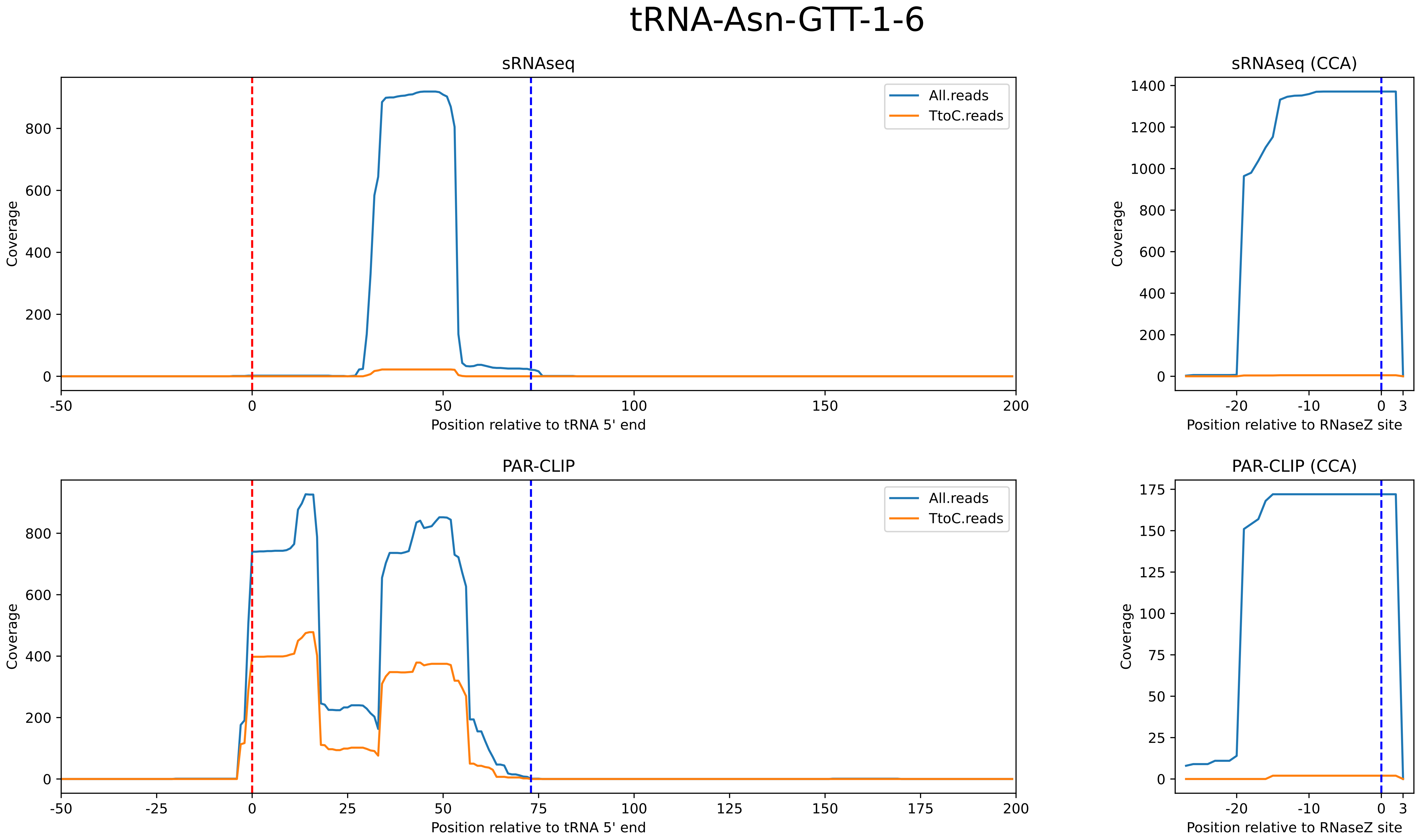
tRNA-Asn-GTT-1-6
sRNAseq
All.reads
TtoC.reads
Coverage
Position relative to tRNA 5' end
sRNAseq (CCA)
Position relative to RNaseZ site
PAR-CLIP
PAR-CLIP (CCA)

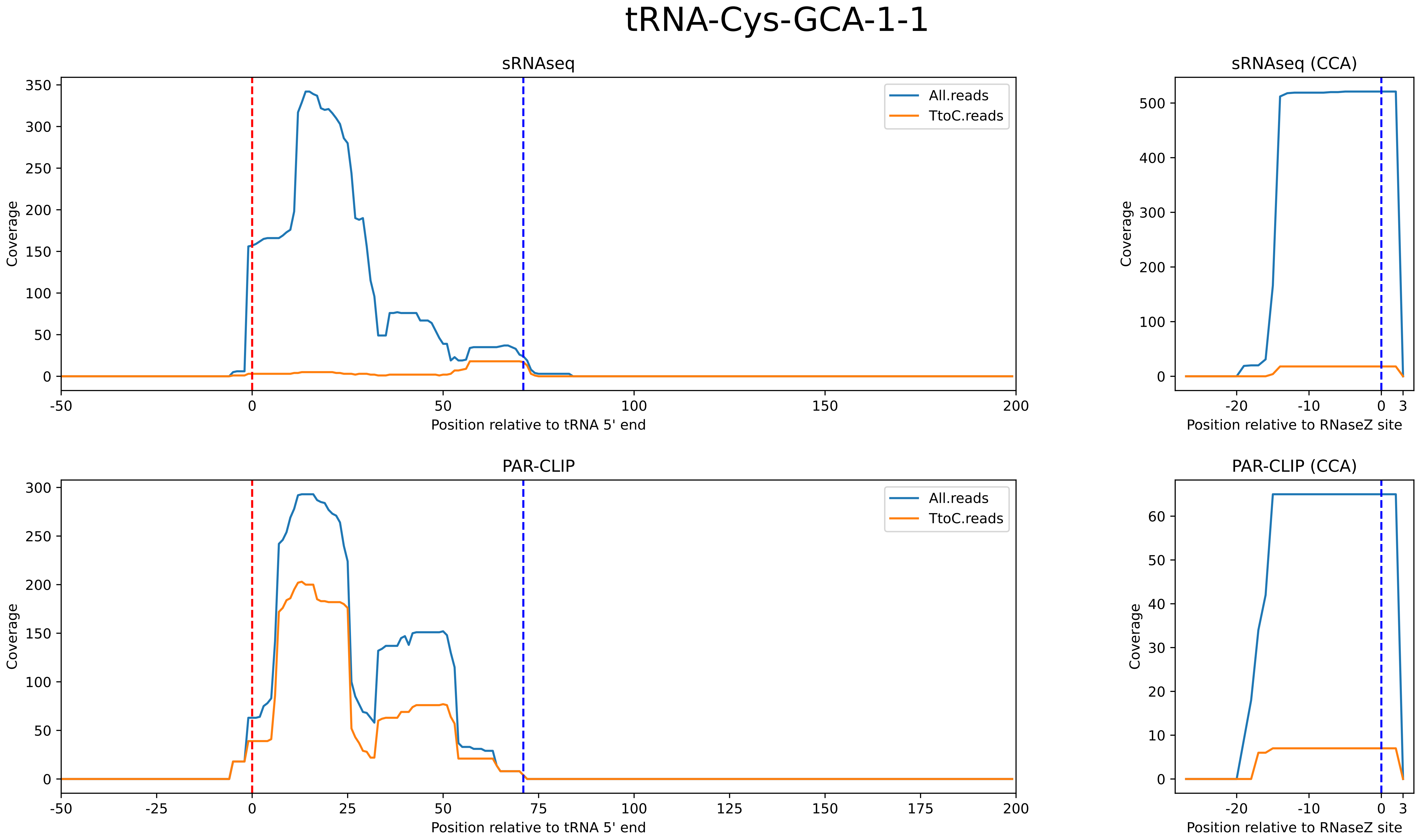
tRNA-Cys-GCA-1-1
sRNAseq
All.reads
TtoC.reads
Coverage
Position relative to tRNA 5' end
sRNAseq (CCA)
Position relative to RNaseZ site
PAR-CLIP
PAR-CLIP (CCA)

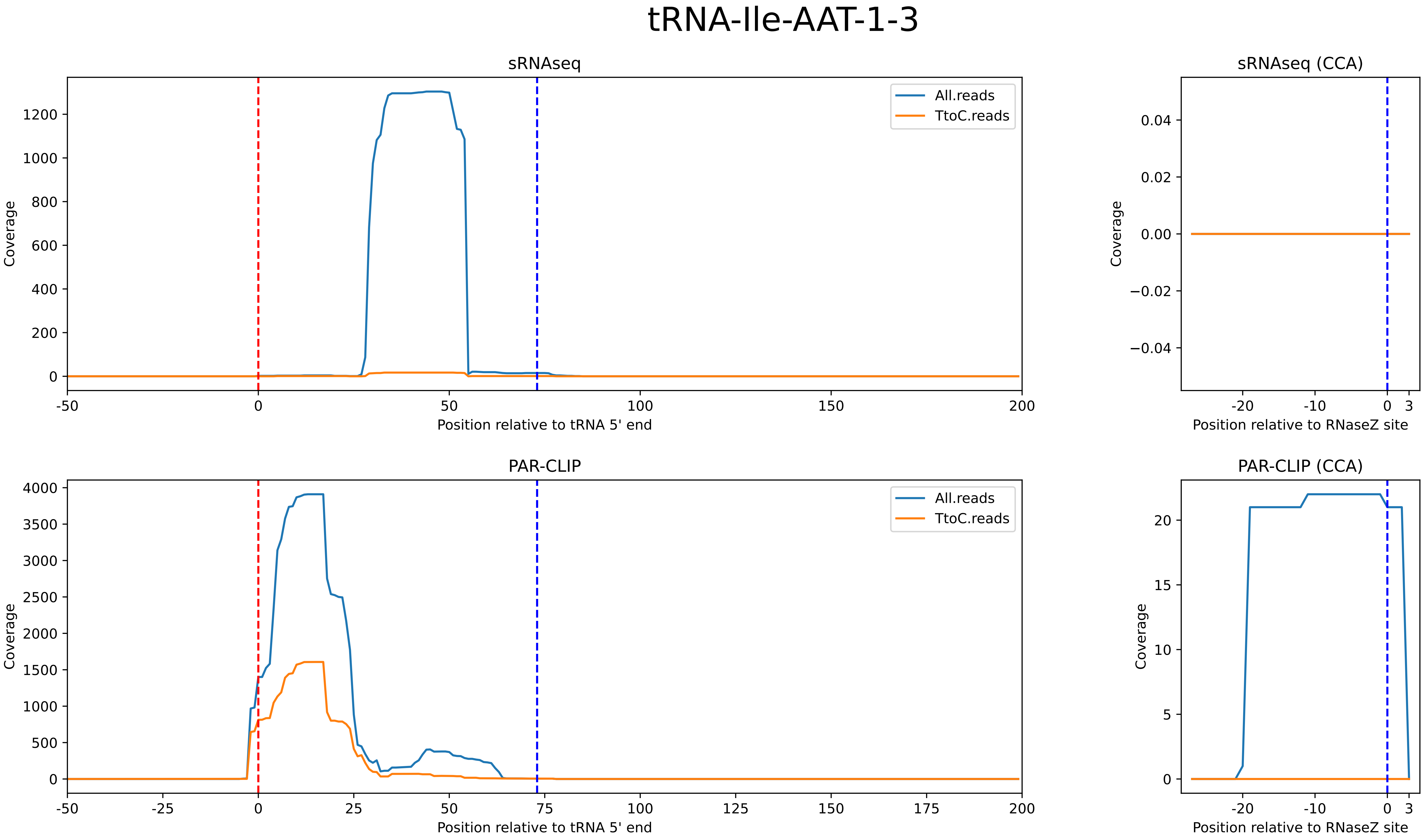

tRNA-Ile-AAT-1-3
sRNAseq
All.reads
TtoC.reads
Coverage
Position relative to tRNA 5' end
sRNAseq (CCA)
Position relative to RNaseZ site
PAR-CLIP
PAR-CLIP (CCA)

# tRNA-Asn-GTT-1-3

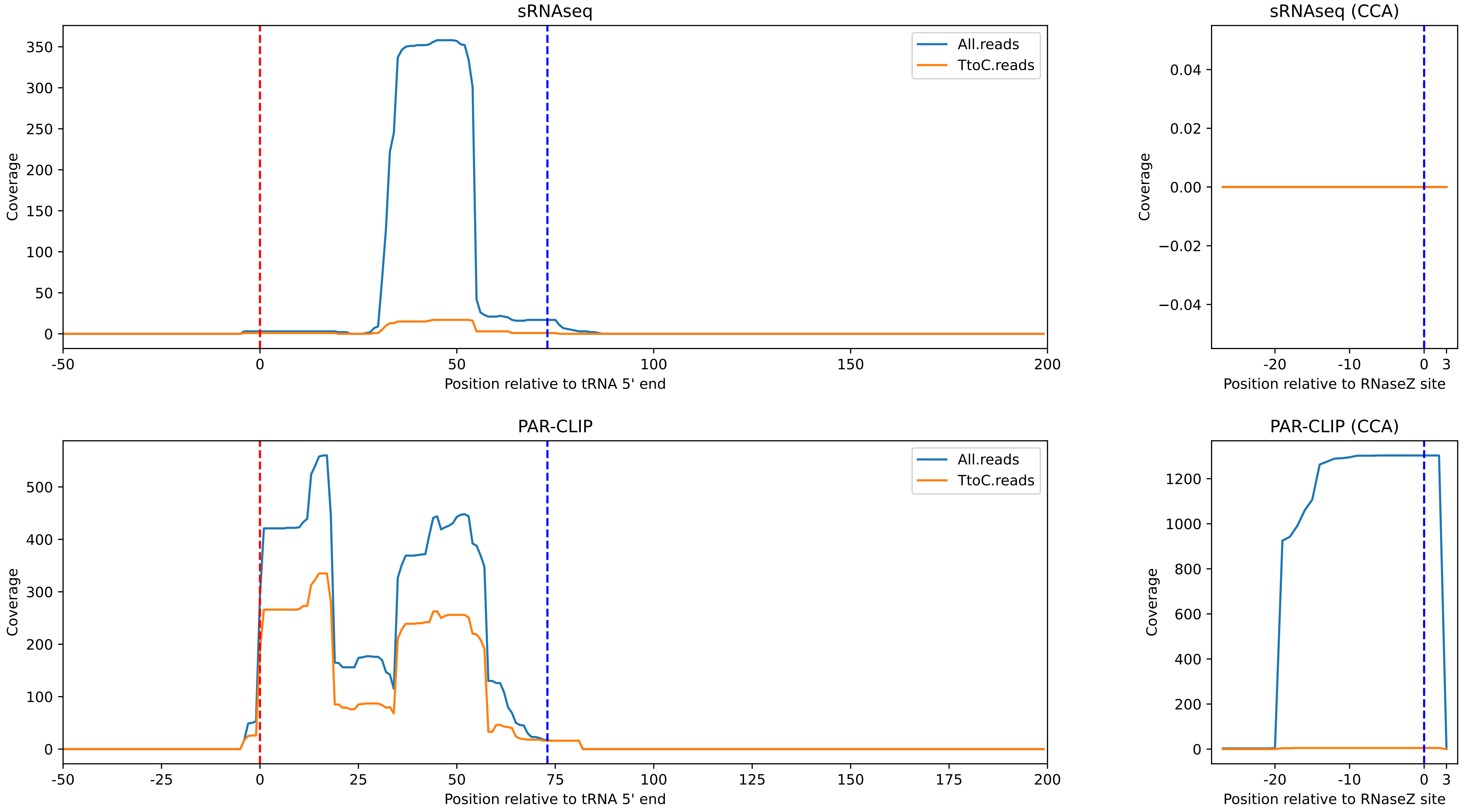

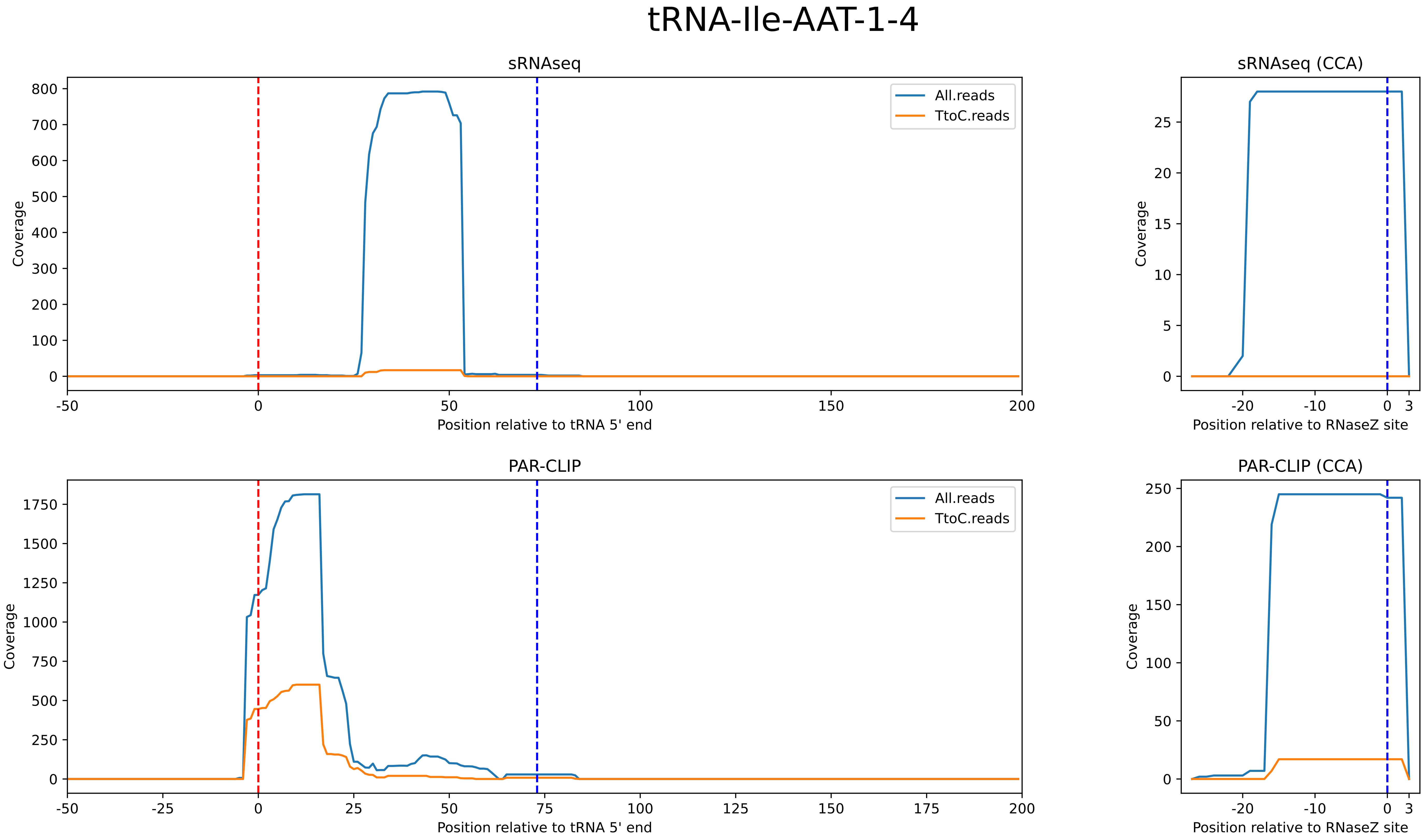

tRNA-Ile-AAT-1-4
sRNAseq
All.reads
TtoC.reads
Coverage
Position relative to tRNA 5' end
sRNAseq (CCA)
Position relative to RNaseZ site
PAR-CLIP
PAR-CLIP (CCA)

# tRNA-Phe-GAA-1-8

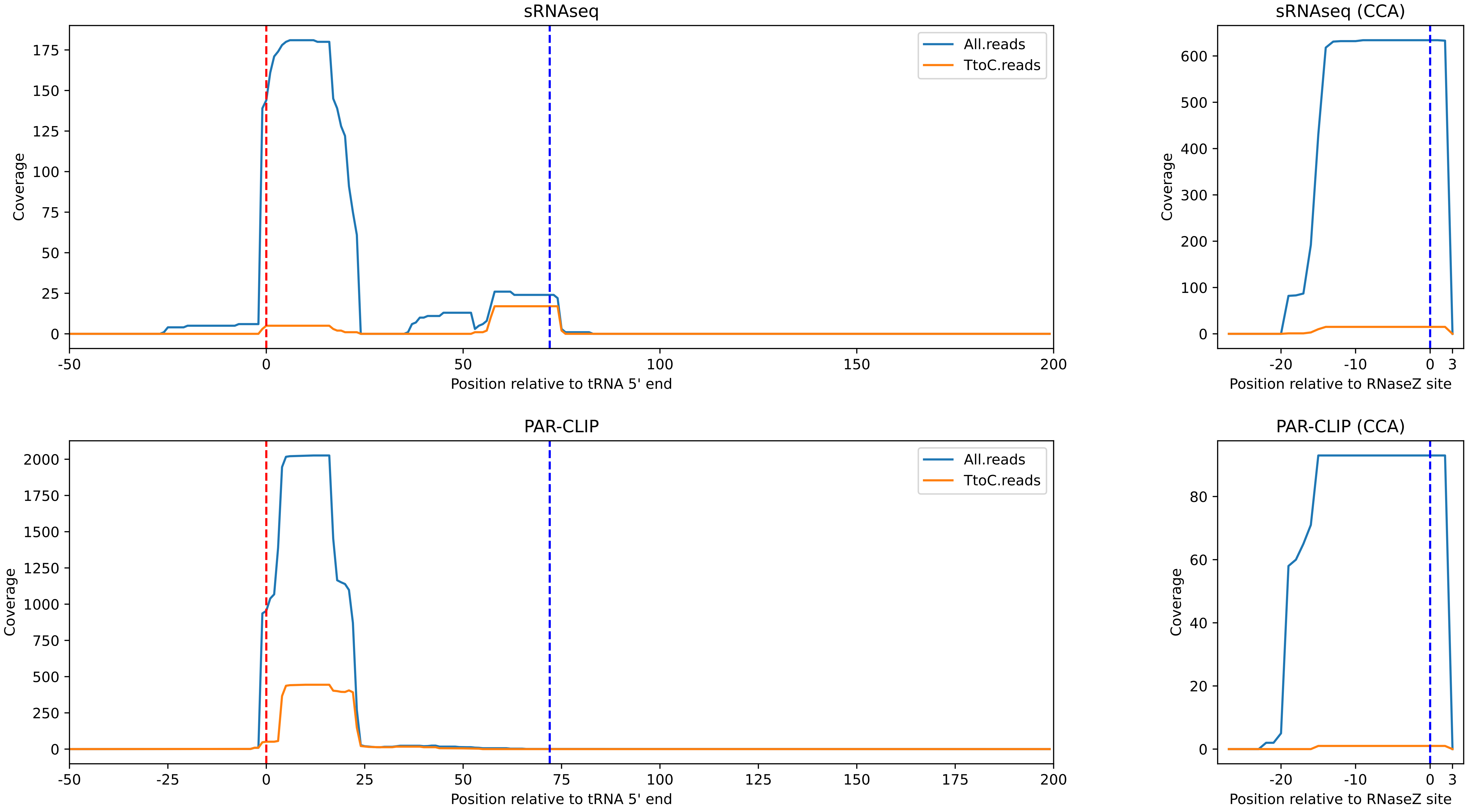

# tRNA-Asn-GTT-1-10

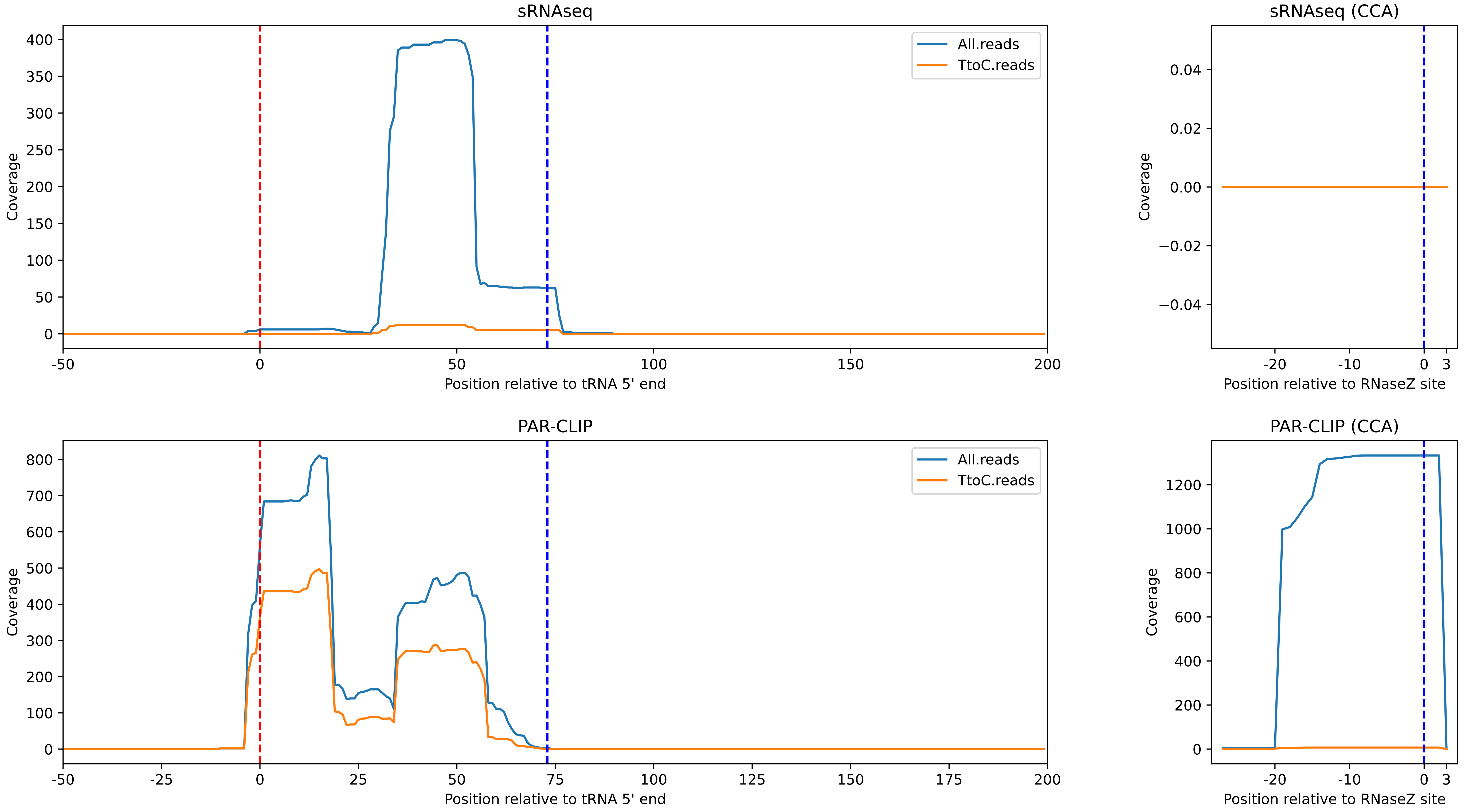

# tRNA-Cys-GCA-1-3

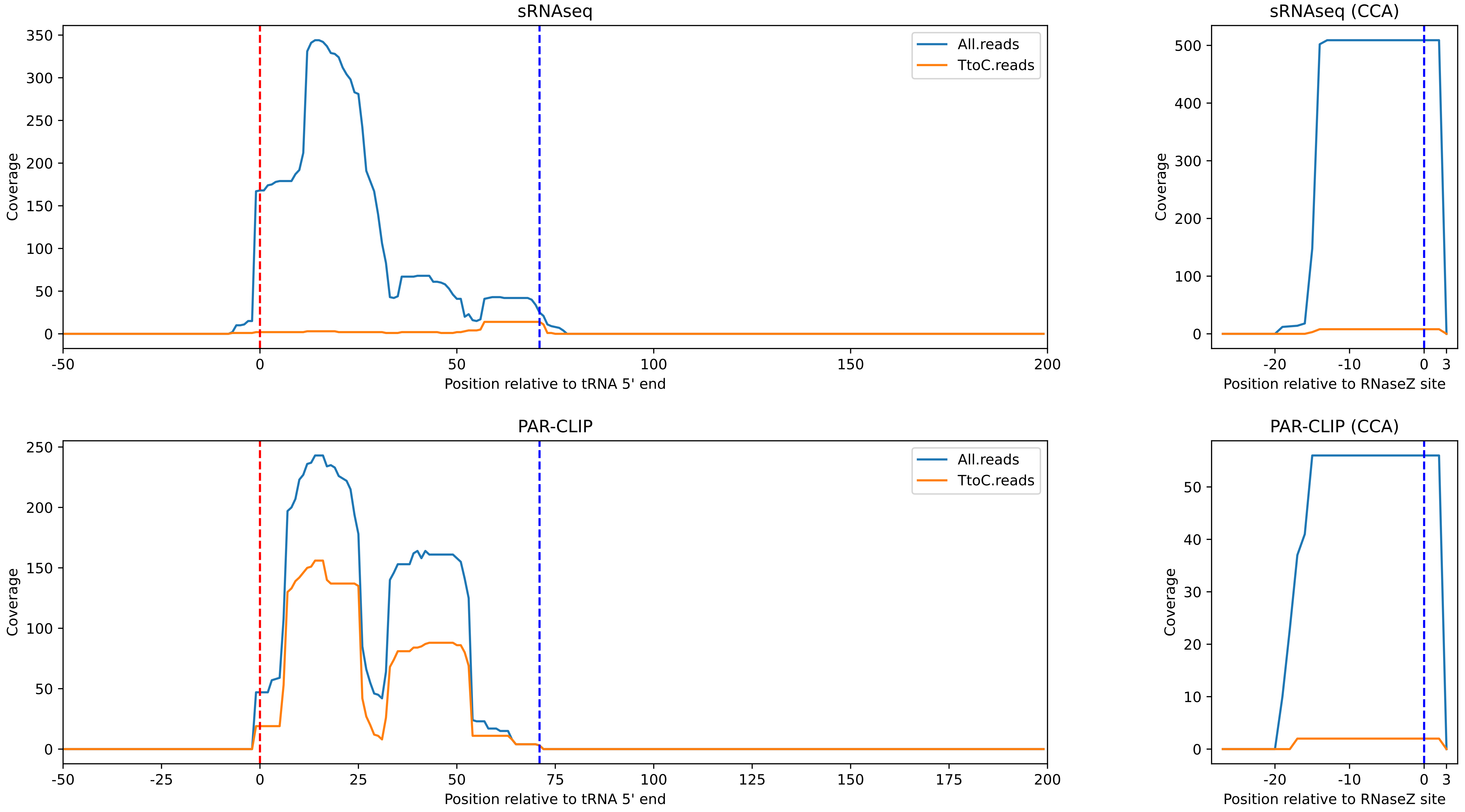

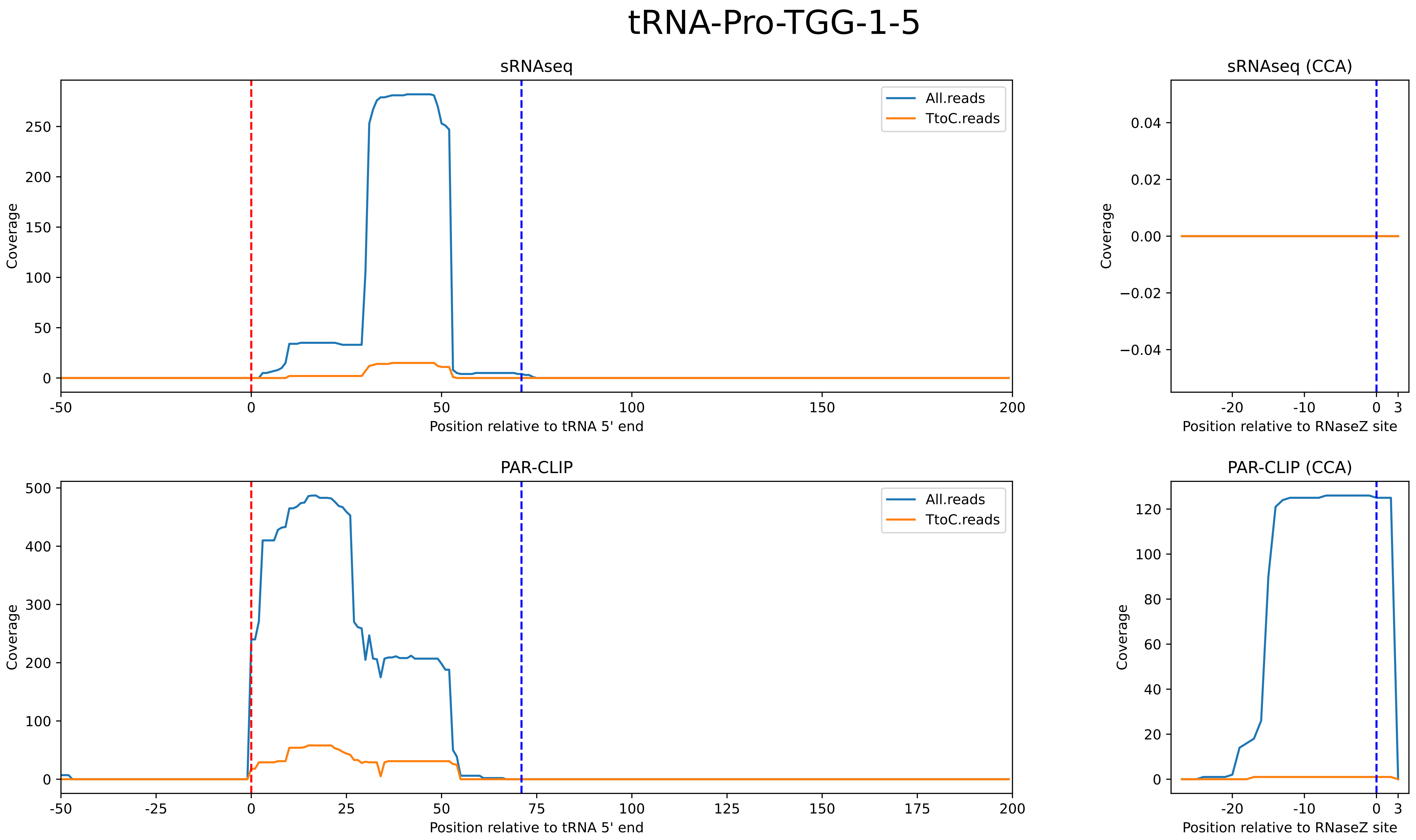

tRNA-Pro-TGG-1-5
sRNAseq
All.reads
TtoC.reads
Coverage
Position relative to tRNA 5' end
sRNAseq (CCA)
Position relative to RNaseZ site
PAR-CLIP
PAR-CLIP (CCA)

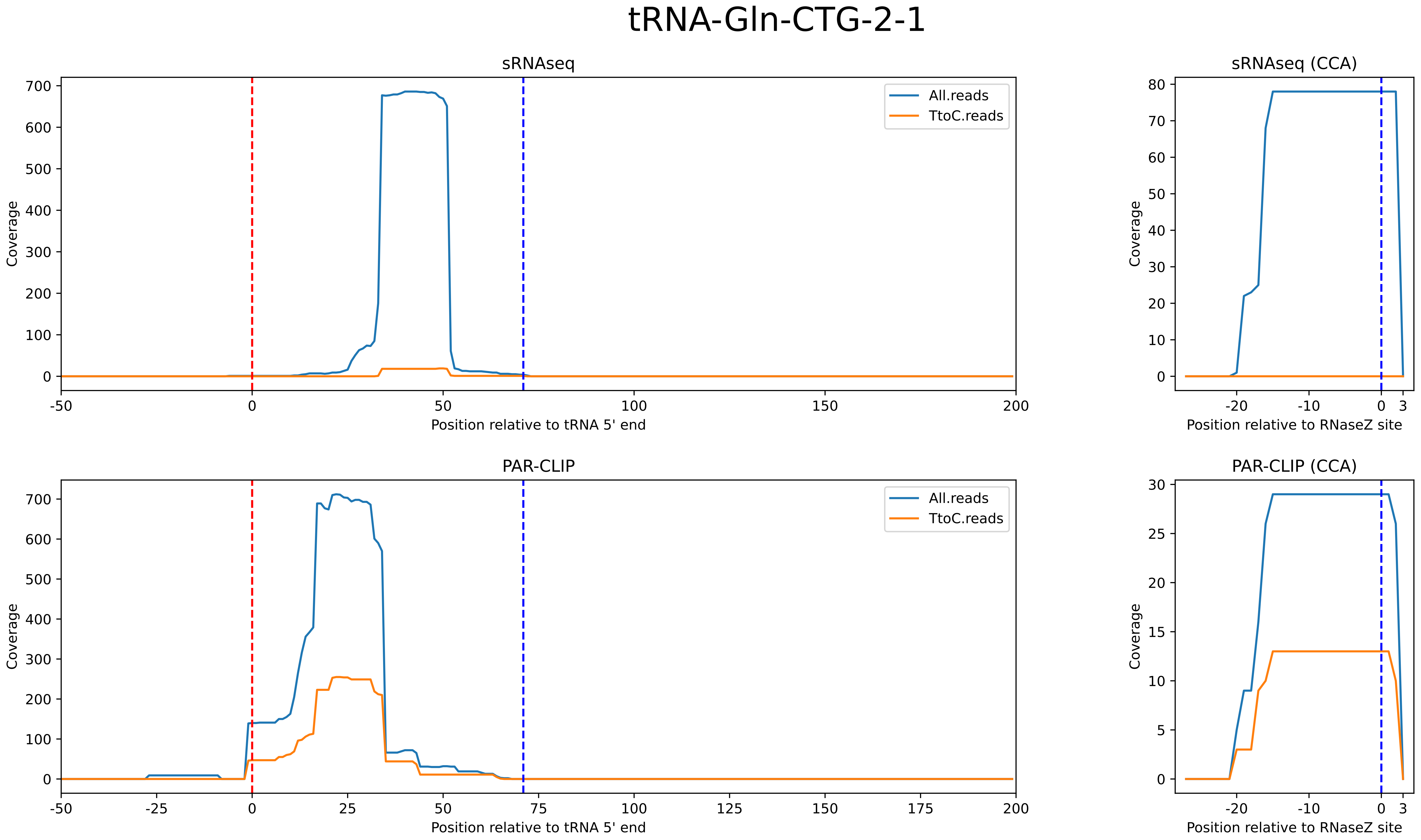

tRNA-Gln-CTG-2-1
sRNAseq
sRNAseq (CCA)
PAR-CLIP
PAR-CLIP (CCA)
All.reads
TtoC.reads
Coverage
Position relative to tRNA 5' end
Position relative to RNaseZ site

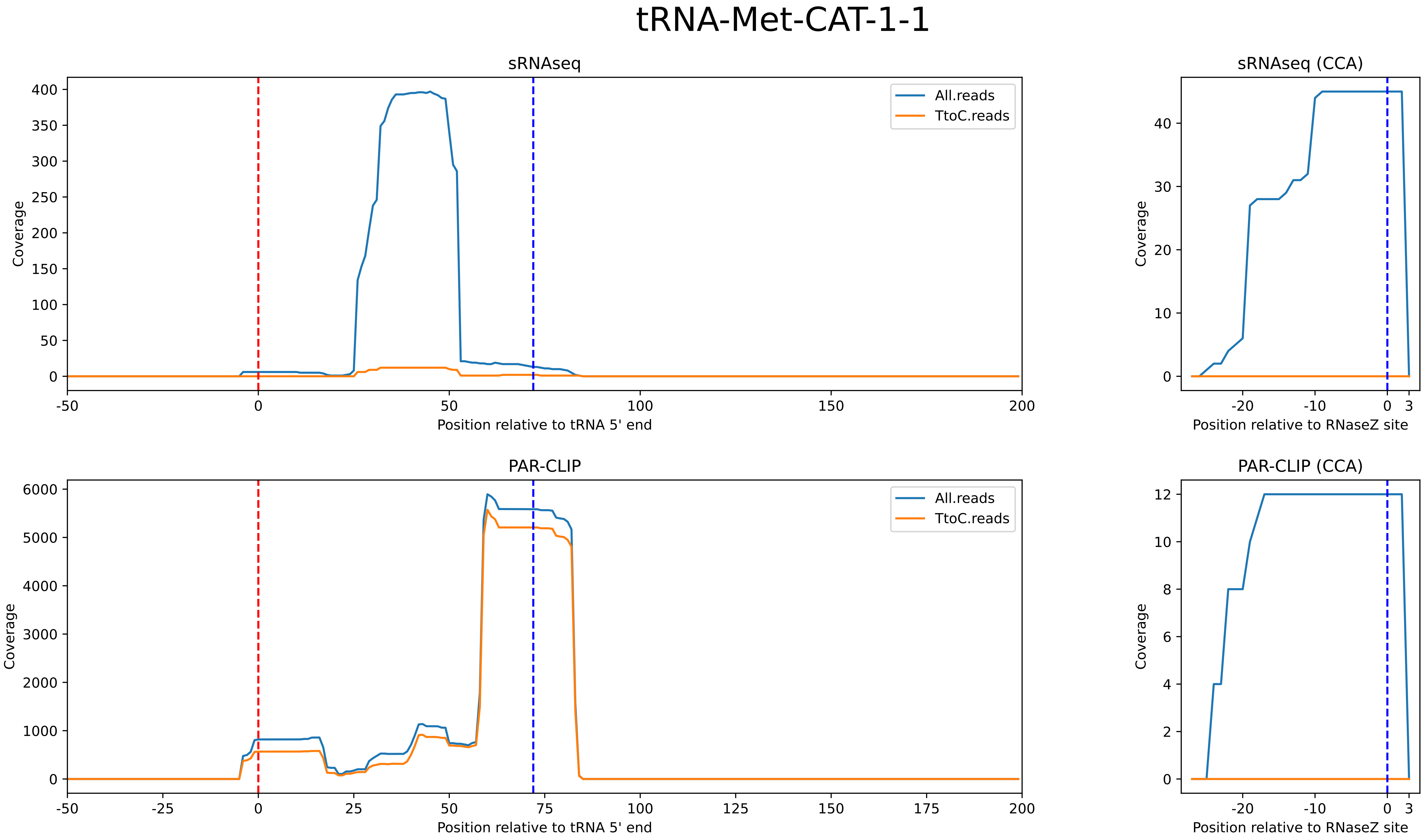

tRNA-Met-CAT-1-1
sRNAseq
sRNAseq (CCA)
PAR-CLIP
PAR-CLIP (CCA)
All.reads
TtoC.reads
Coverage
Position relative to tRNA 5' end
Position relative to RNaseZ site

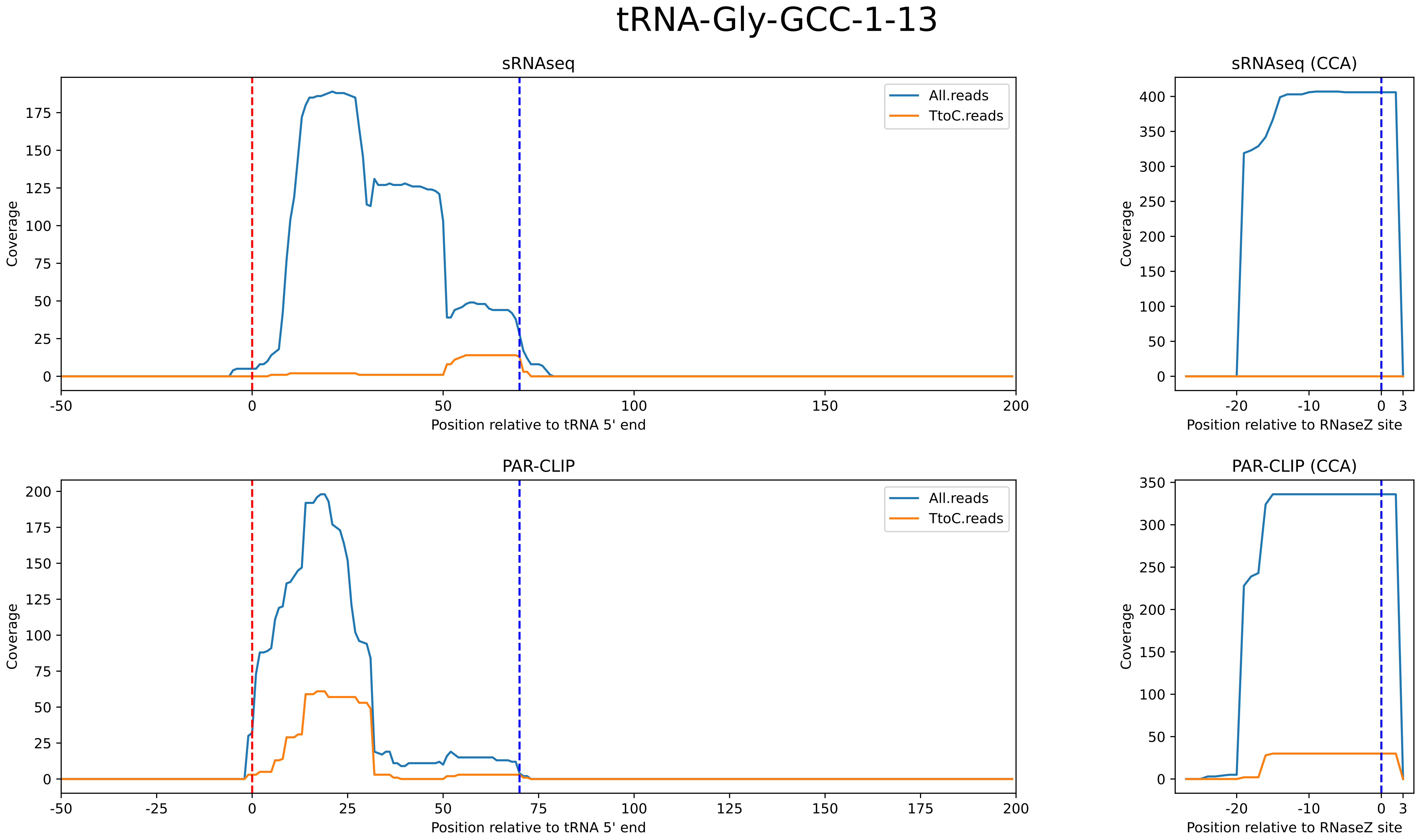
tRNA-Gly-GCC-1-13
sRNAseq
sRNAseq (CCA)
PAR-CLIP
PAR-CLIP (CCA)
All.reads
TtoC.reads
Coverage
Position relative to tRNA 5' end
Position relative to RNaseZ site

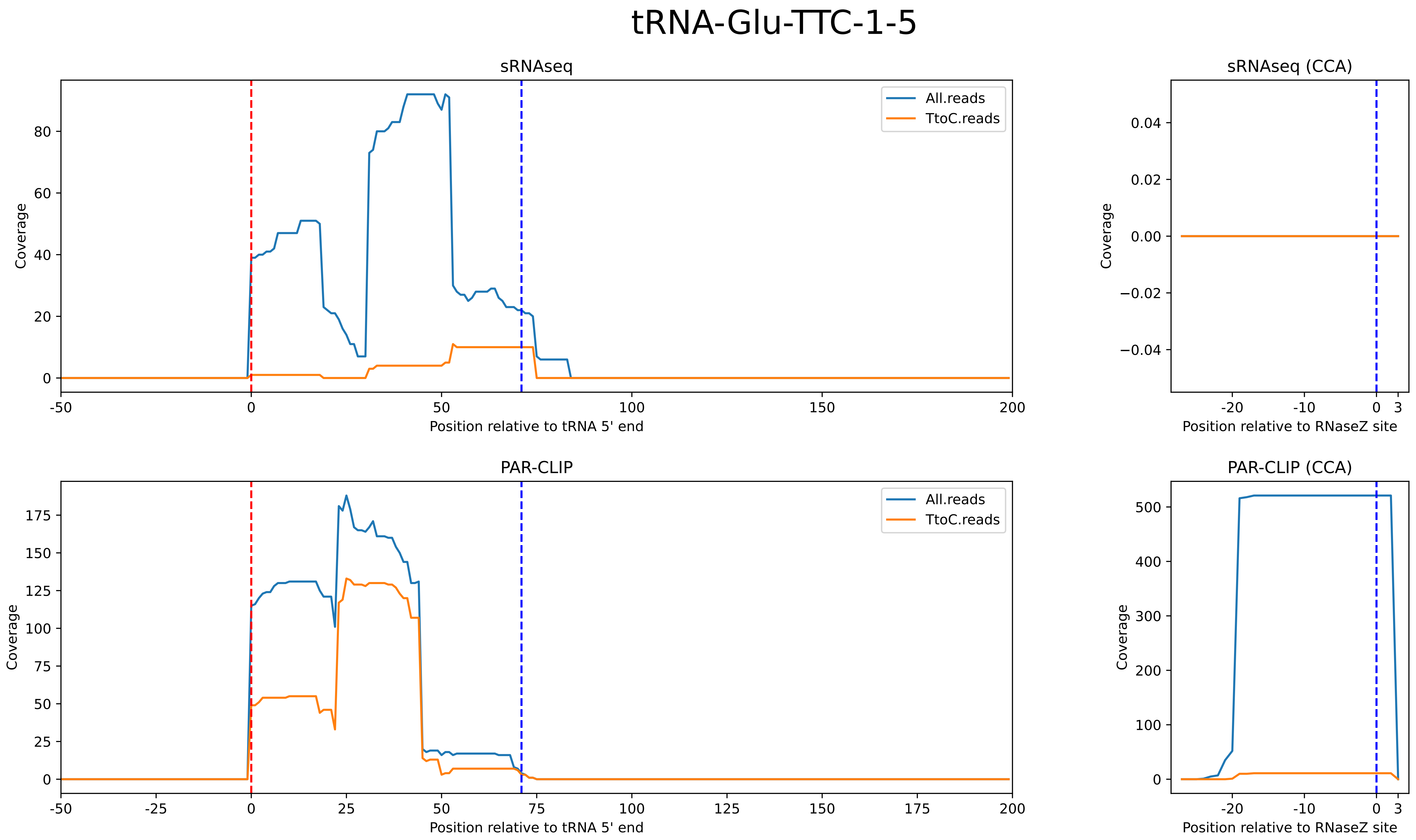

tRNA-Glu-TTC-1-5
sRNAseq
sRNAseq (CCA)
PAR-CLIP
PAR-CLIP (CCA)
All.reads
TtoC.reads
Coverage
Position relative to tRNA 5' end
Position relative to RNaseZ site

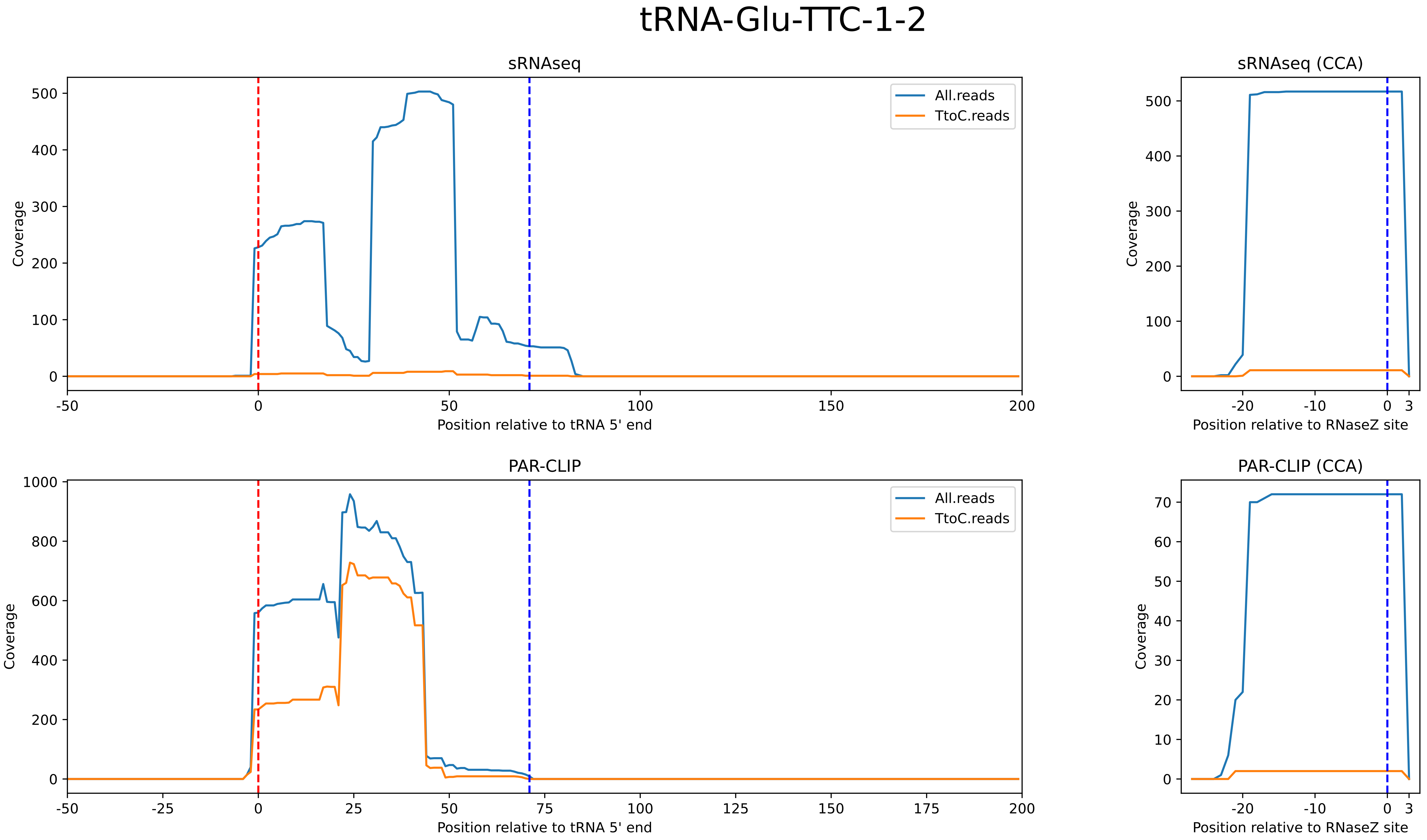
tRNA-Glu-TTC-1-2
sRNAseq
All.reads
TtoC.reads
Coverage
Position relative to tRNA 5' end
sRNAseq (CCA)
Position relative to RNaseZ site
PAR-CLIP
PAR-CLIP (CCA)

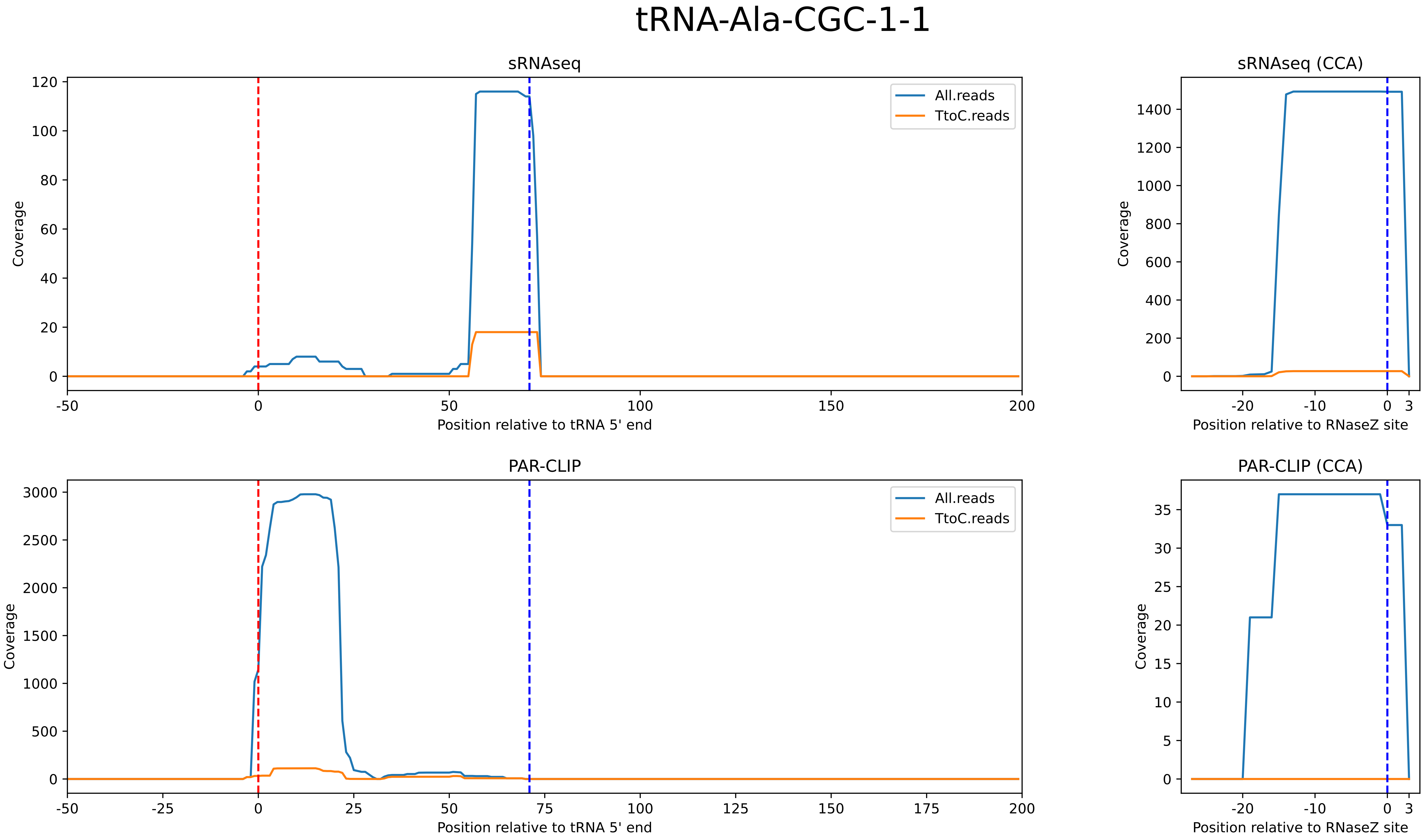

tRNA-Ala-CGC-1-1
sRNAseq
All.reads
TtoC.reads
Coverage
Position relative to tRNA 5' end
sRNAseq (CCA)
Position relative to RNaseZ site
PAR-CLIP
PAR-CLIP (CCA)

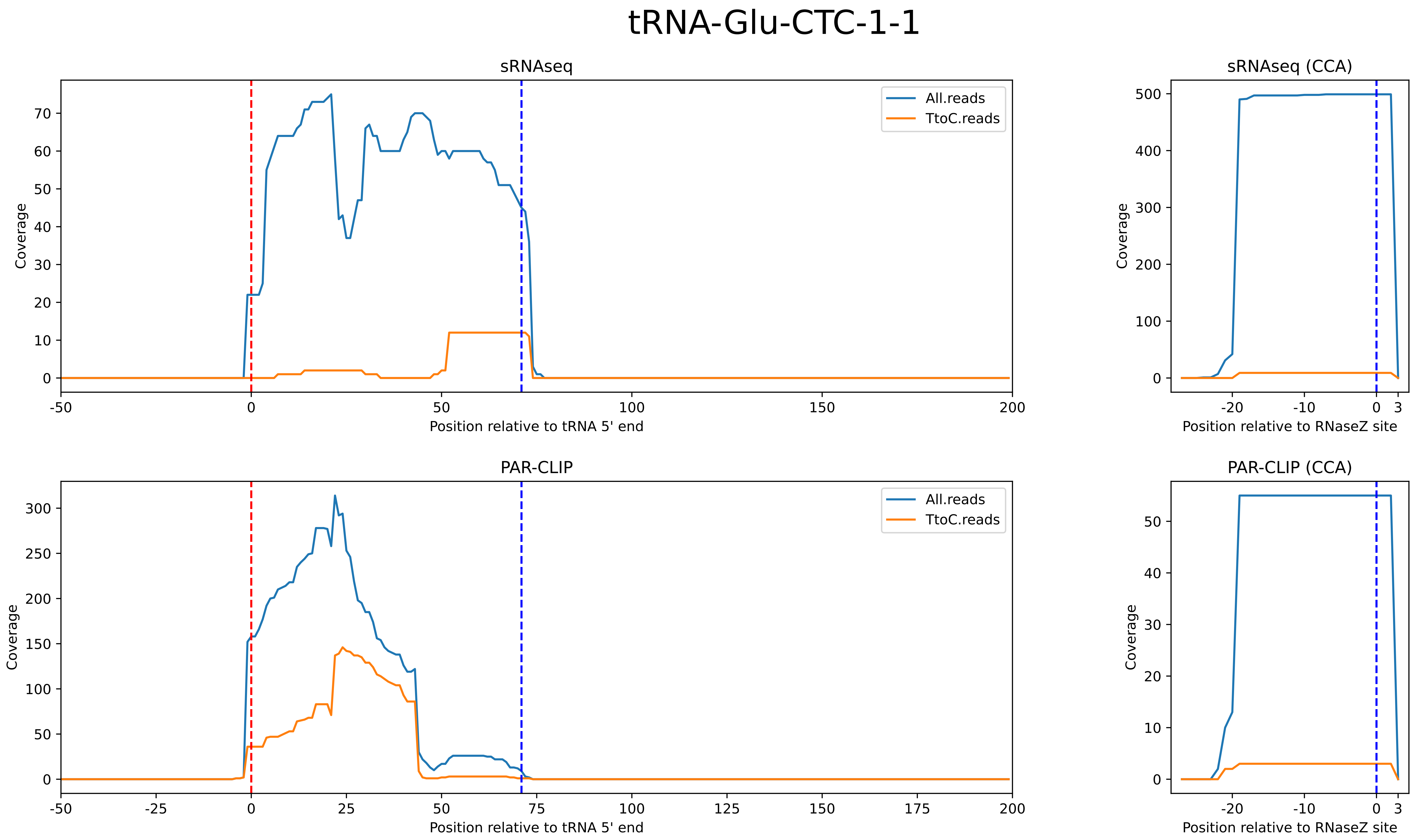
tRNA-Glu-CTC-1-1
sRNAseq
All.reads
TtoC.reads
Coverage
Position relative to tRNA 5' end
sRNAseq (CCA)
Position relative to RNaseZ site
PAR-CLIP
All.reads
TtoC.reads
Coverage
Position relative to tRNA 5' end
PAR-CLIP (CCA)
Coverage
Position relative to RNaseZ site

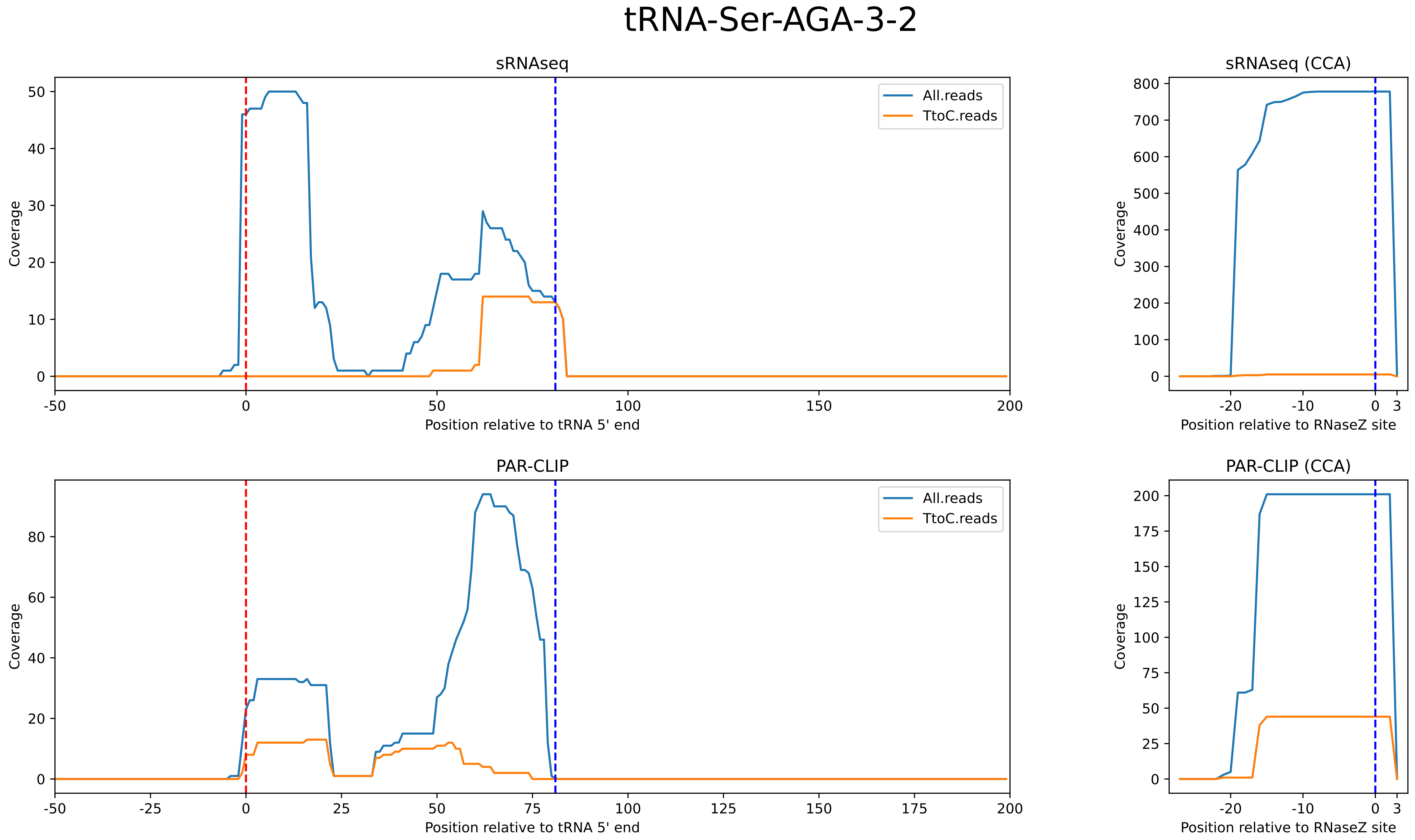

tRNA-Ser-AGA-3-2
sRNAseq
sRNAseq (CCA)
PAR-CLIP
PAR-CLIP (CCA)
All.reads
TtoC.reads
Coverage
Position relative to tRNA 5' end
Position relative to RNaseZ site

# tRNA-Phe-GAA-1-3

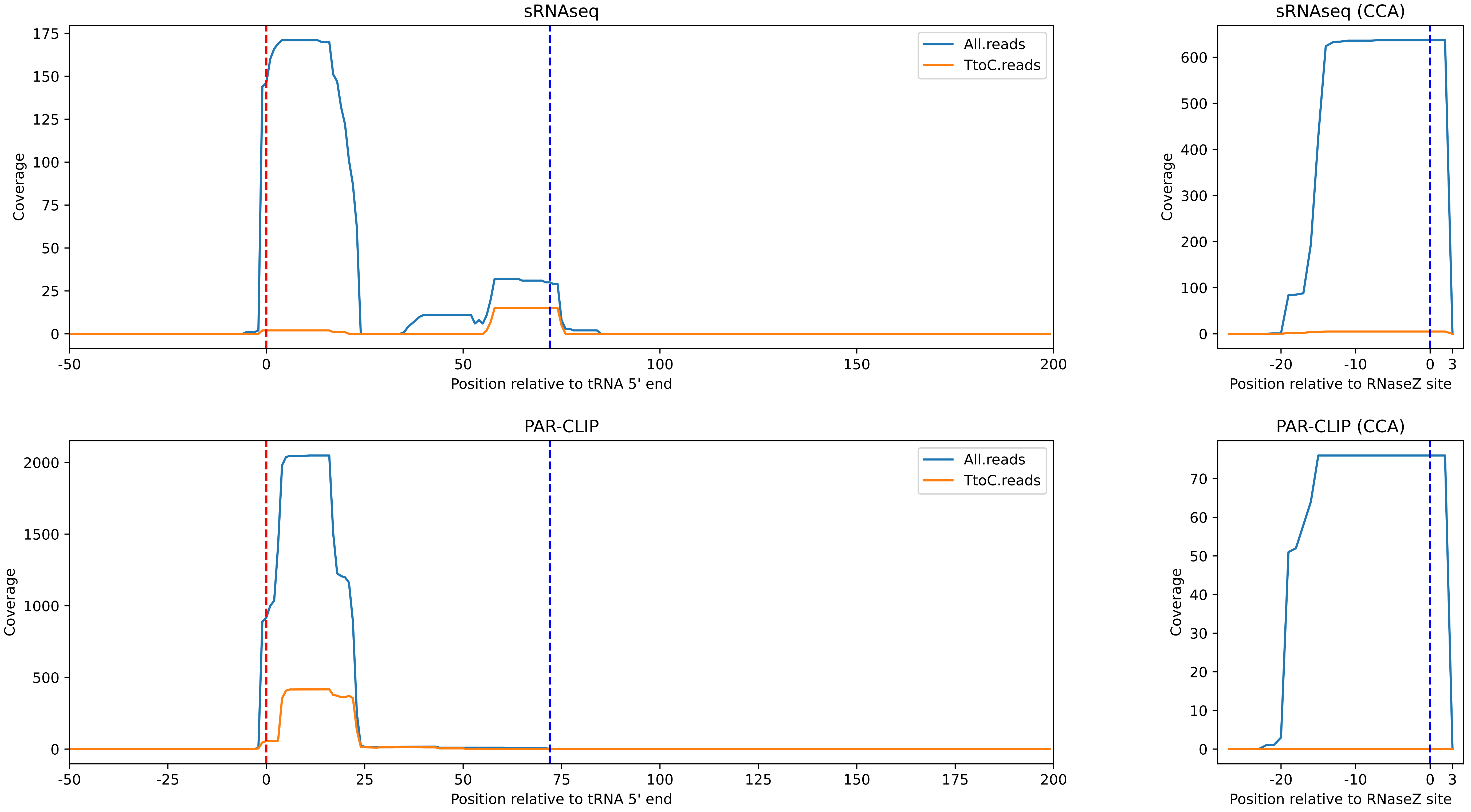

# tRNA-Lys-CTT-1-12

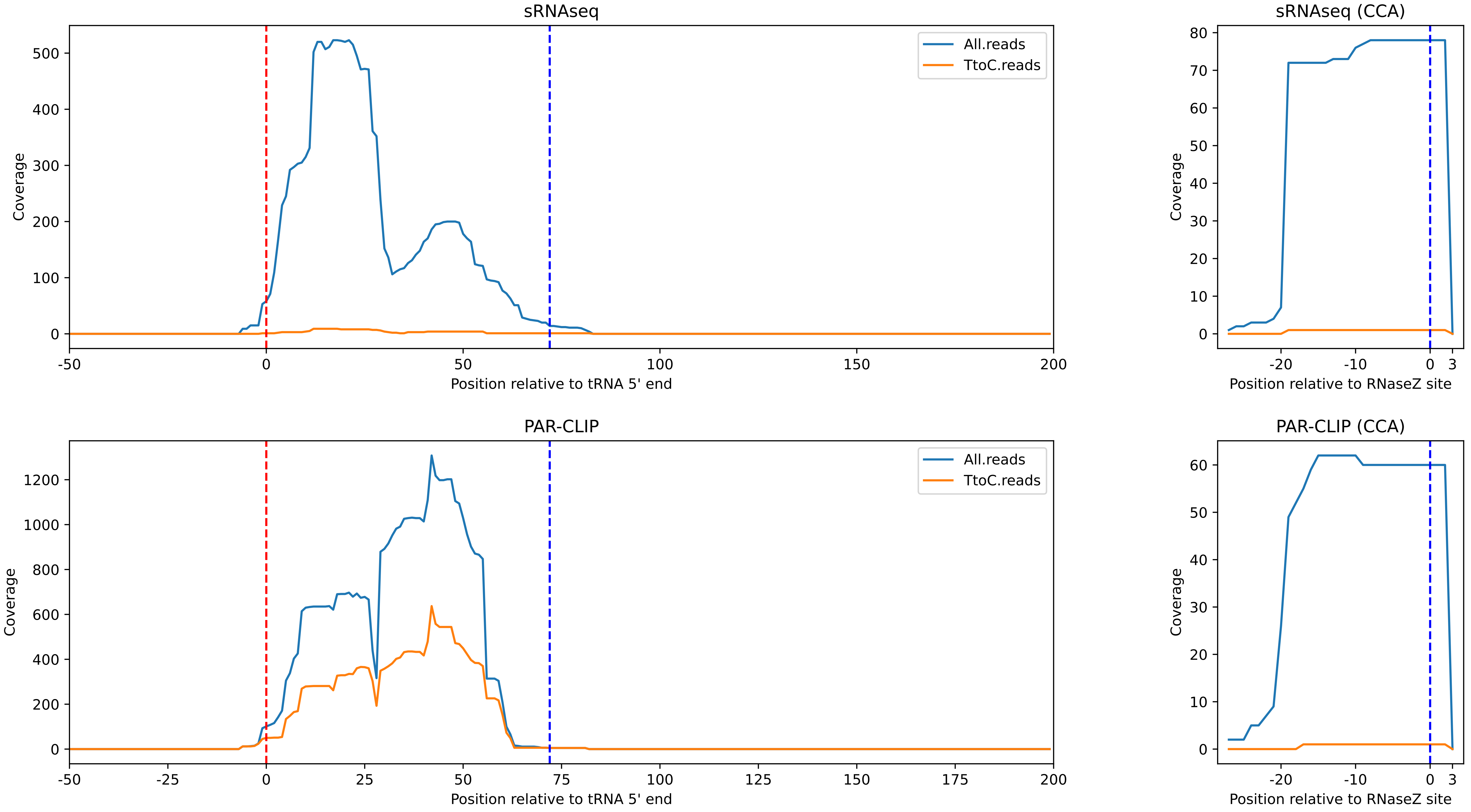

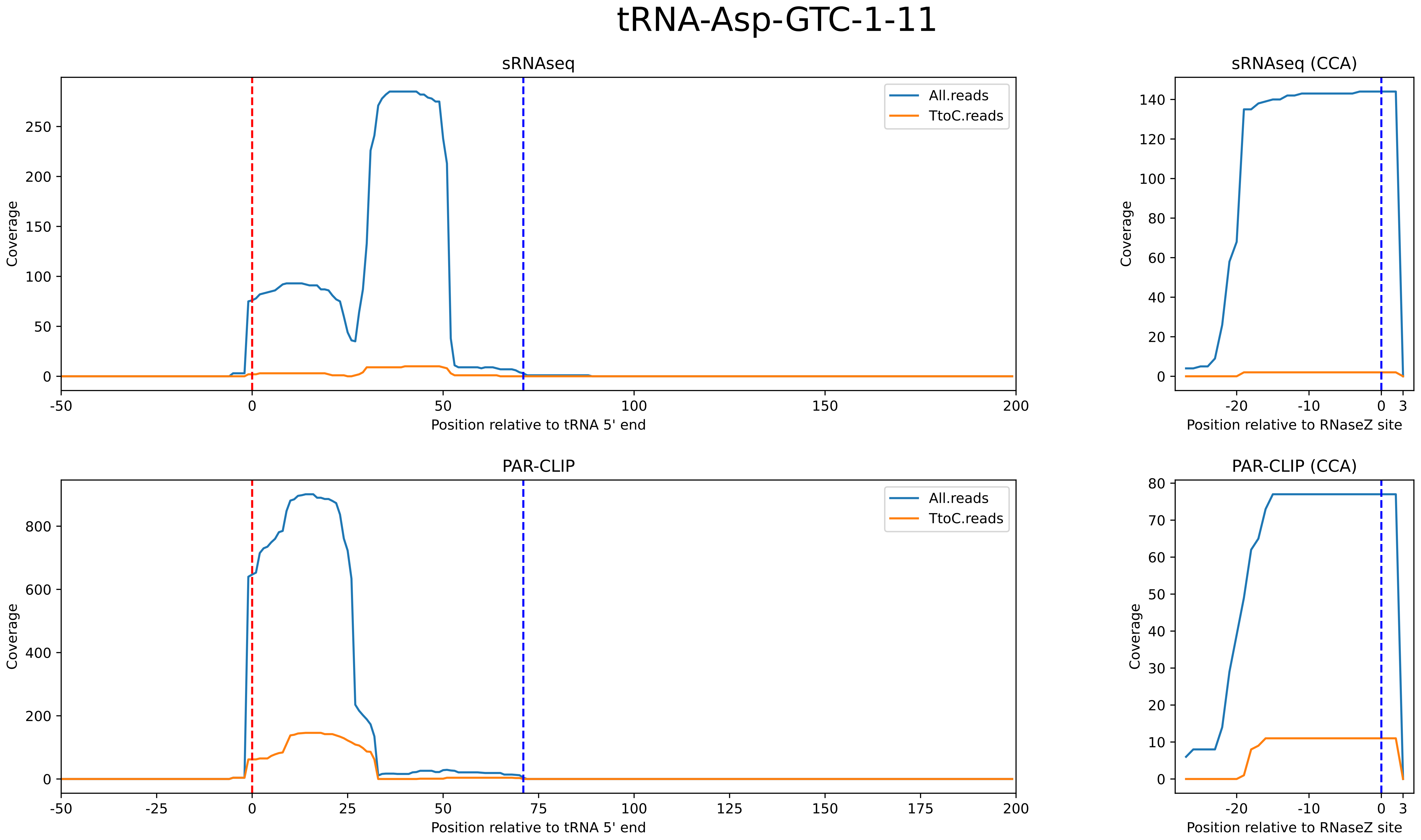

tRNA-Asp-GTC-1-11
sRNAseq
All.reads
TtoC.reads
Coverage
Position relative to tRNA 5' end
sRNAseq (CCA)
Position relative to RNaseZ site
PAR-CLIP
PAR-CLIP (CCA)

# tRNA-Lys-CTT-1-4

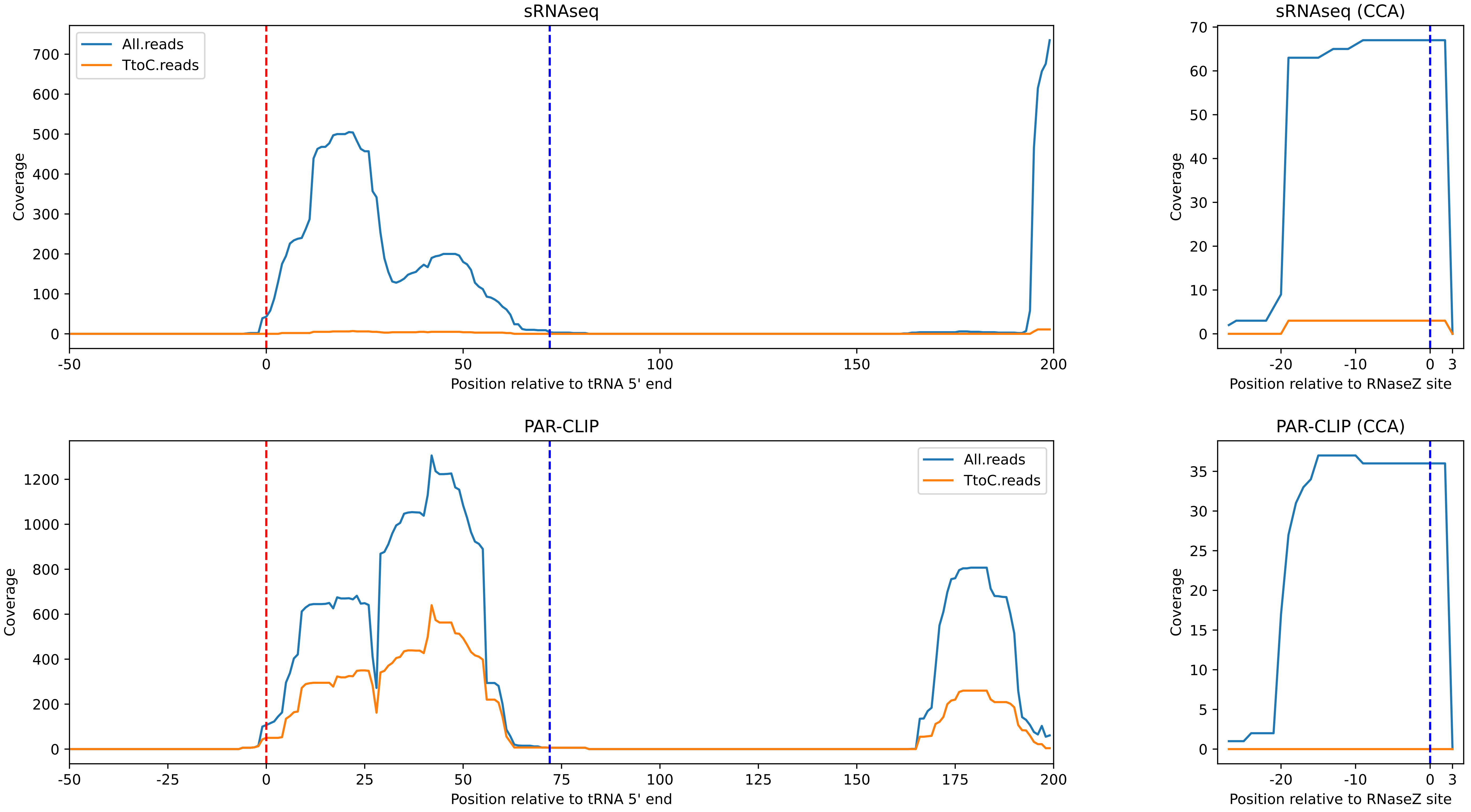

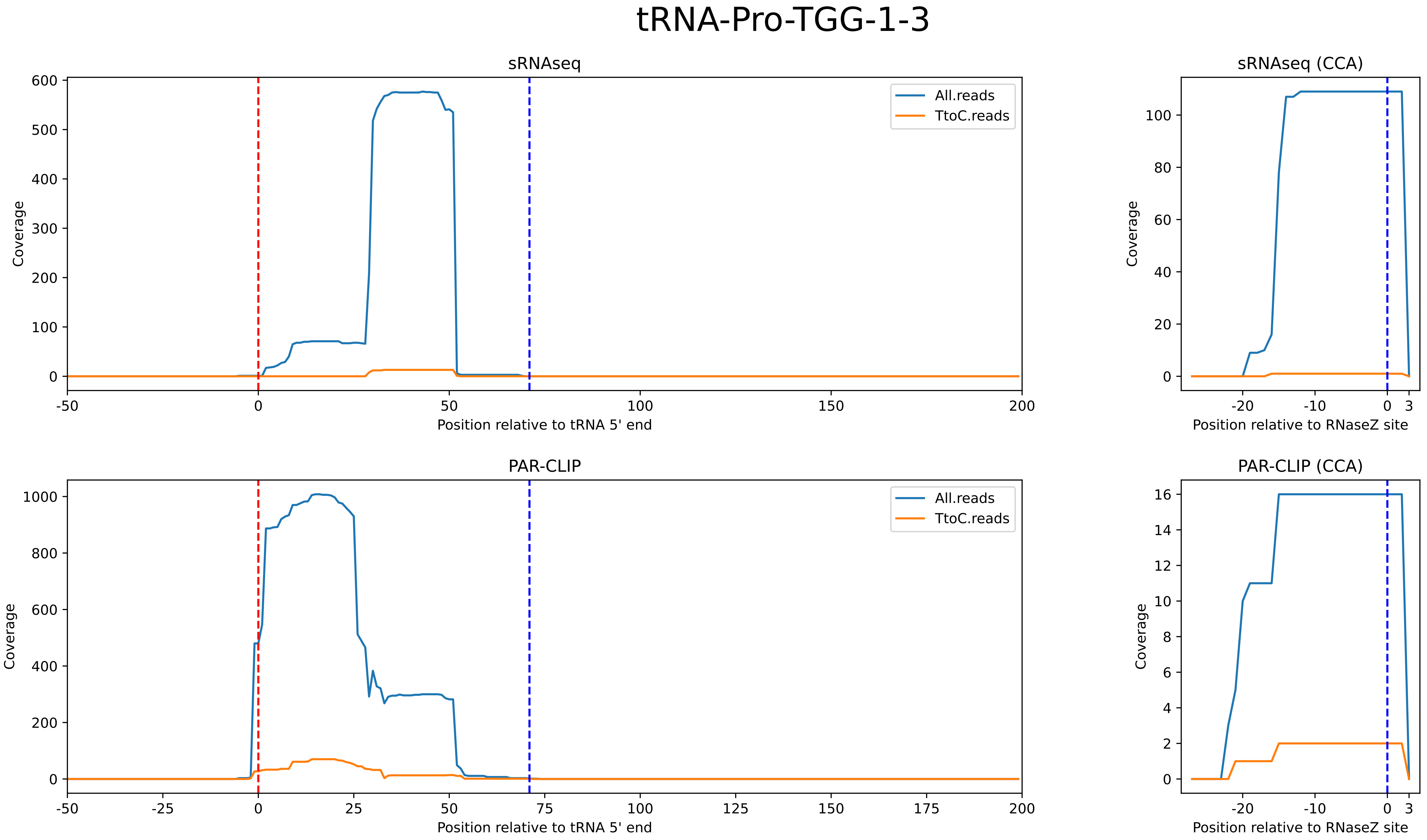

tRNA-Pro-TGG-1-3
sRNAseq
All.reads
TtoC.reads
Coverage
Position relative to tRNA 5' end
sRNAseq (CCA)
Position relative to RNaseZ site
PAR-CLIP
PAR-CLIP (CCA)

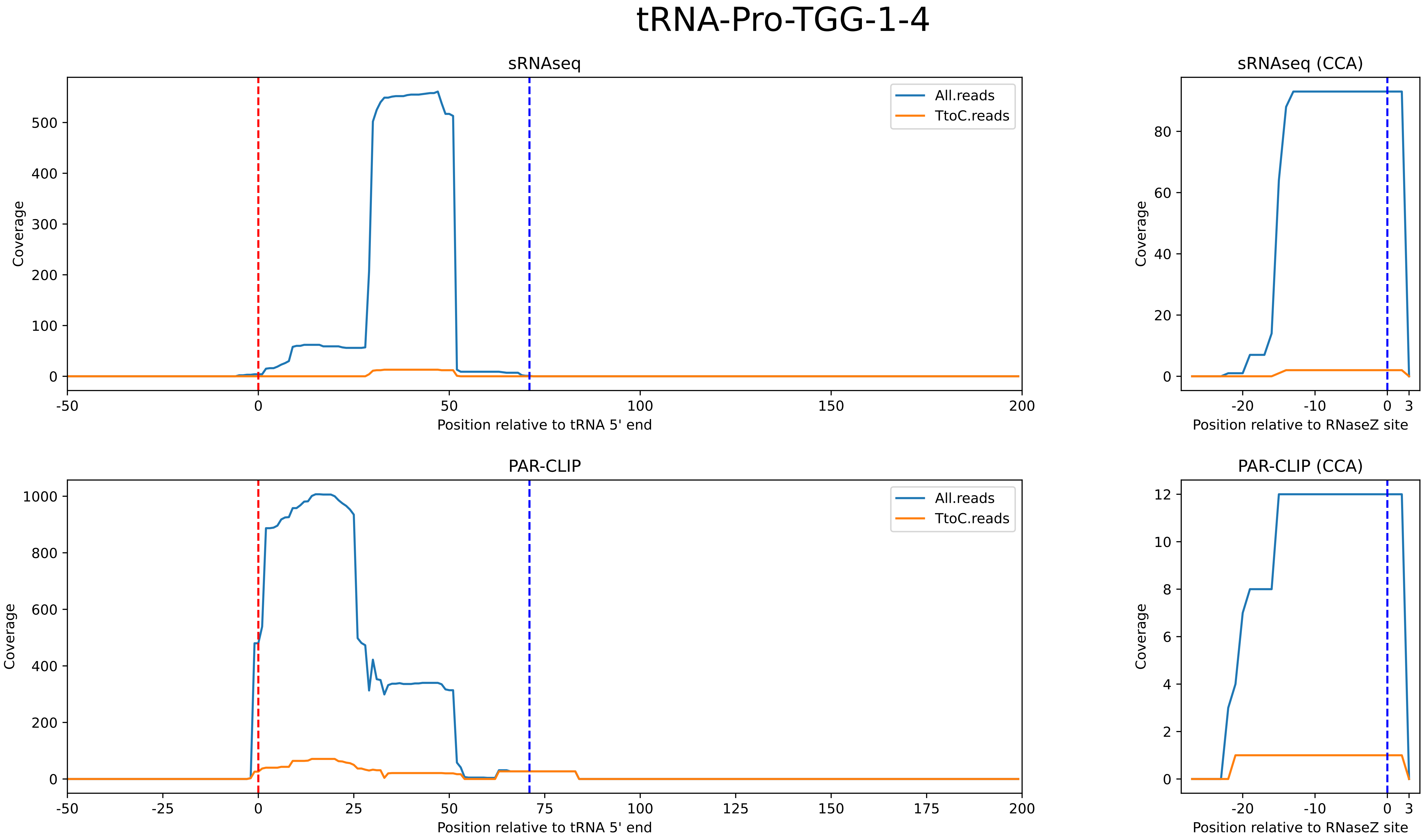
tRNA-Pro-TGG-1-4
sRNAseq
All.reads
TtoC.reads
Coverage
Position relative to tRNA 5' end
sRNAseq (CCA)
Position relative to RNaseZ site
PAR-CLIP
PAR-CLIP (CCA)

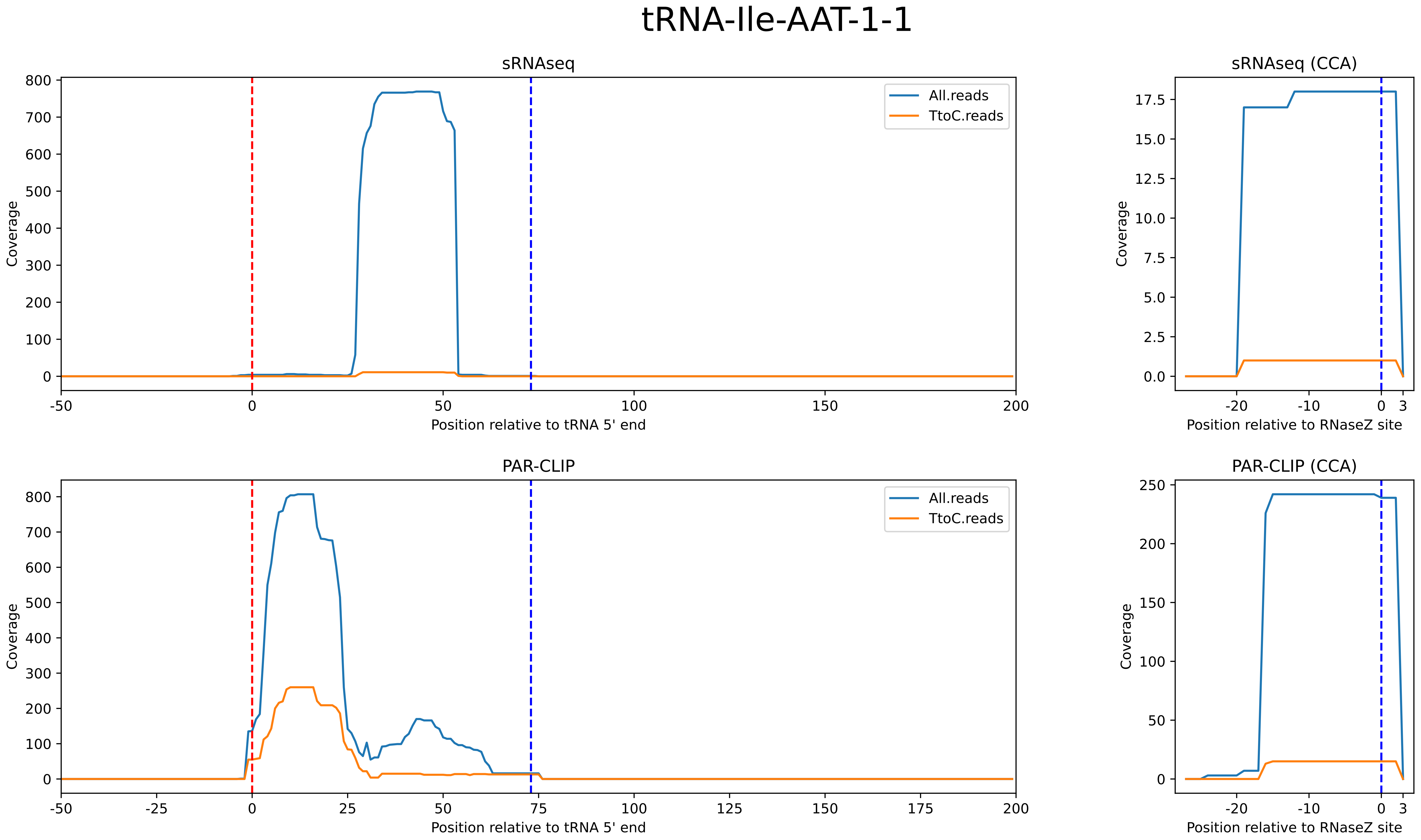

tRNA-Ile-AAT-1-1
sRNAseq
sRNAseq (CCA)
PAR-CLIP
PAR-CLIP (CCA)
All.reads
TtoC.reads
Coverage
Position relative to tRNA 5' end
Position relative to RNaseZ site

# tRNA-Leu-TAA-1-1

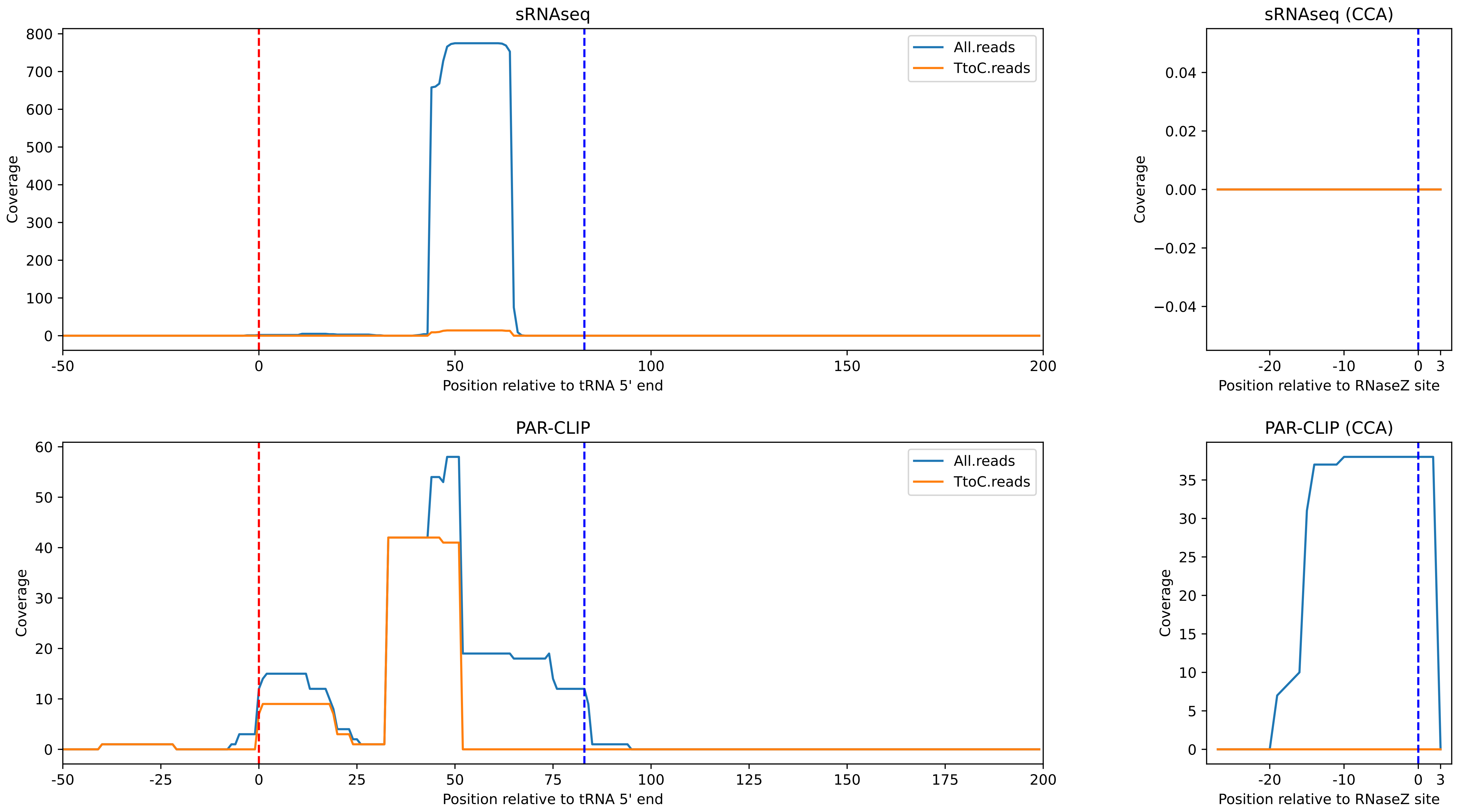

# tRNA-Cys-GCA-4-1

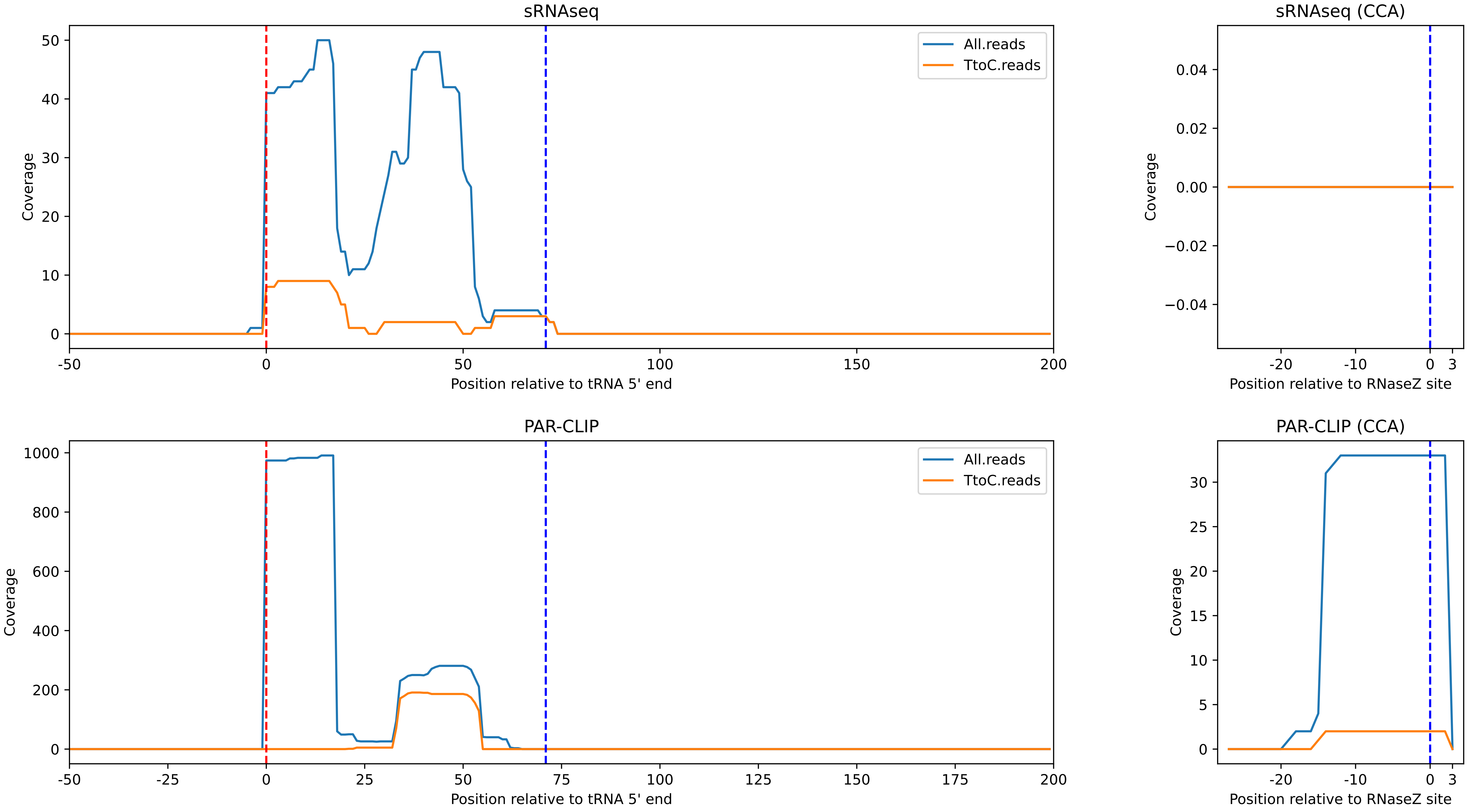

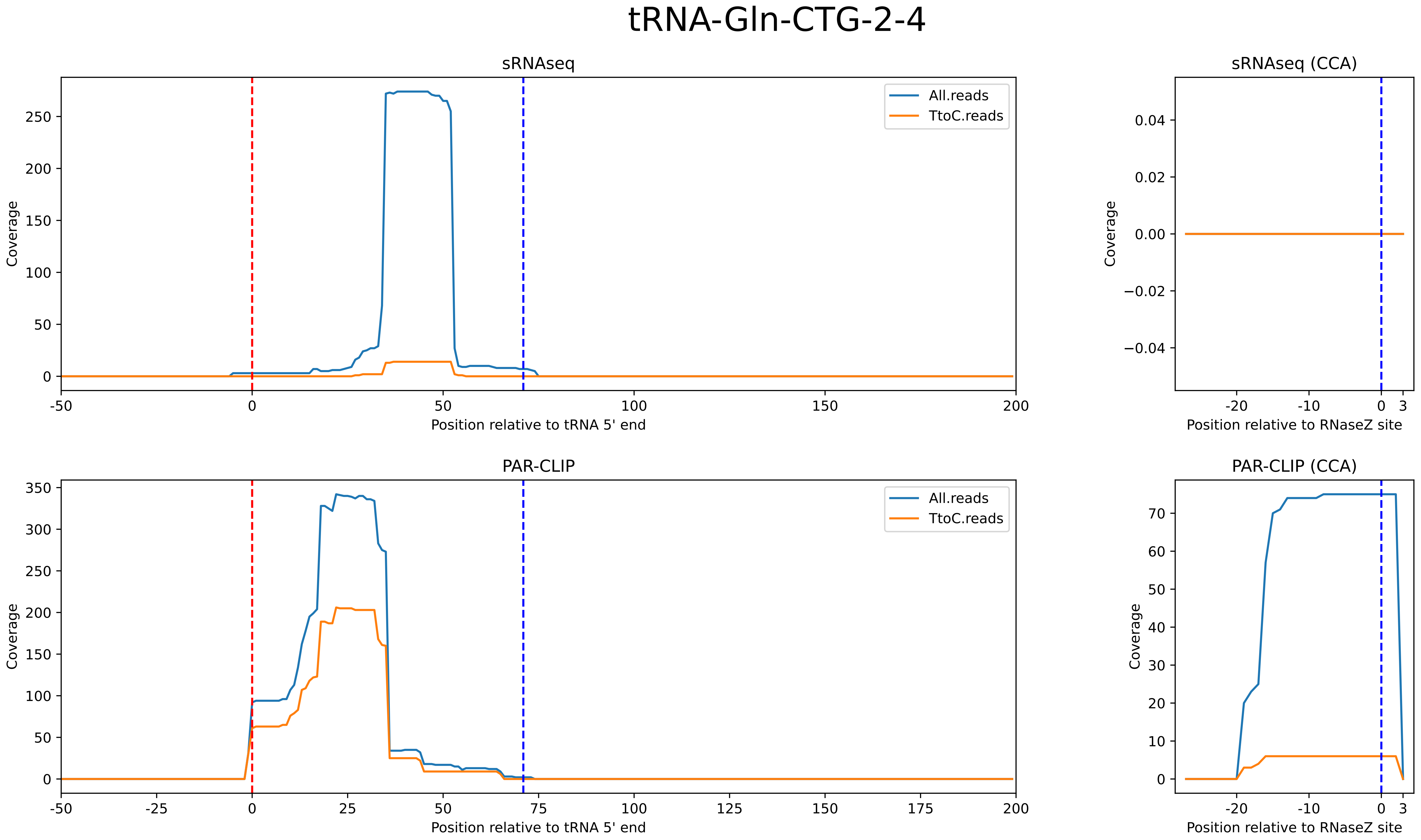
tRNA-Gln-CTG-2-4
sRNAseq
All.reads
TtoC.reads
Coverage
Position relative to tRNA 5' end
sRNAseq (CCA)
Position relative to RNaseZ site
PAR-CLIP
PAR-CLIP (CCA)

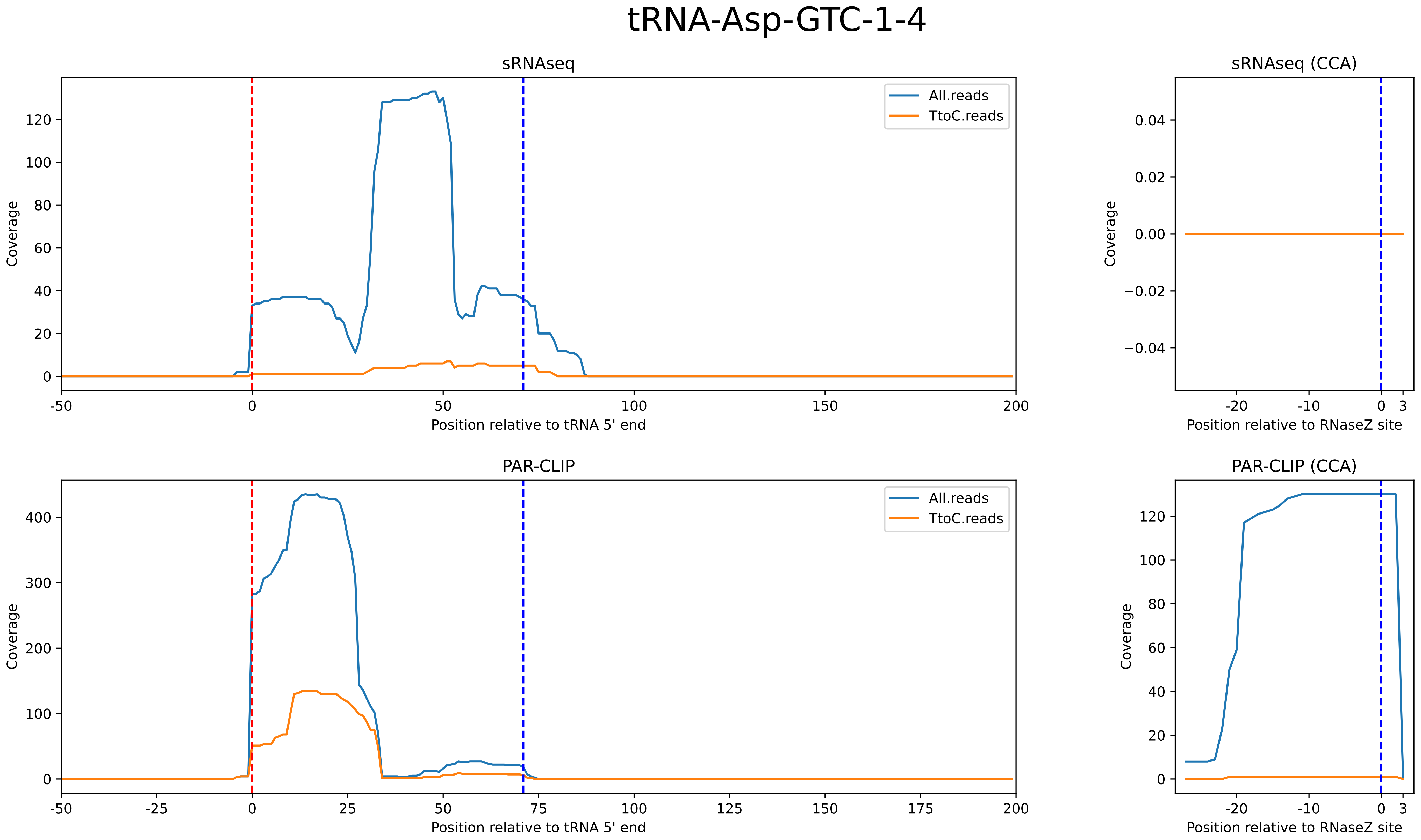

tRNA-Asp-GTC-1-4
sRNAseq
sRNAseq (CCA)
PAR-CLIP
PAR-CLIP (CCA)
All.reads
TtoC.reads
Coverage
Position relative to tRNA 5' end
Position relative to RNaseZ site

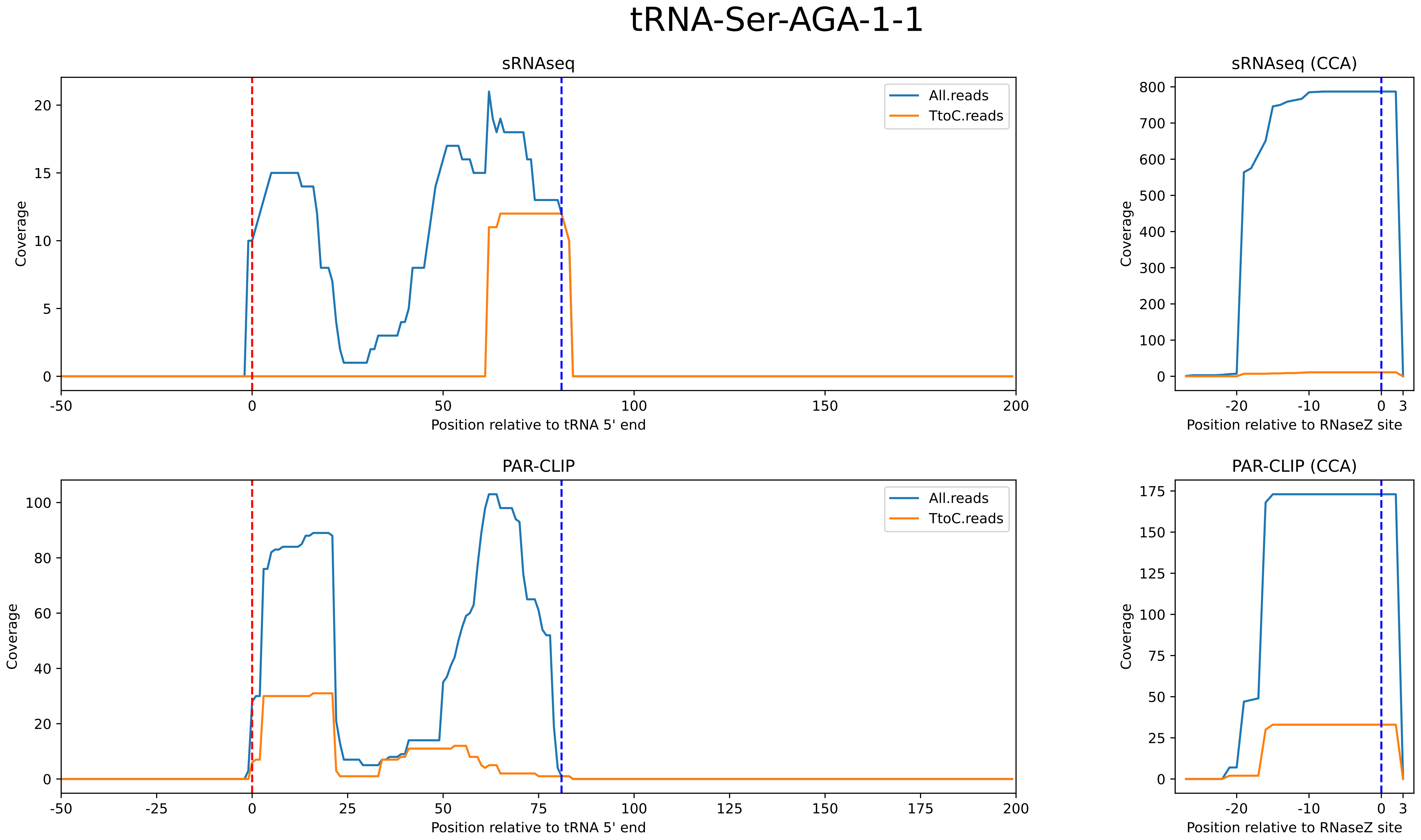
tRNA-Ser-AGA-1-1
sRNAseq
All.reads
TtoC.reads
Coverage
Position relative to tRNA 5' end
sRNAseq (CCA)
Position relative to RNaseZ site
PAR-CLIP
PAR-CLIP (CCA)

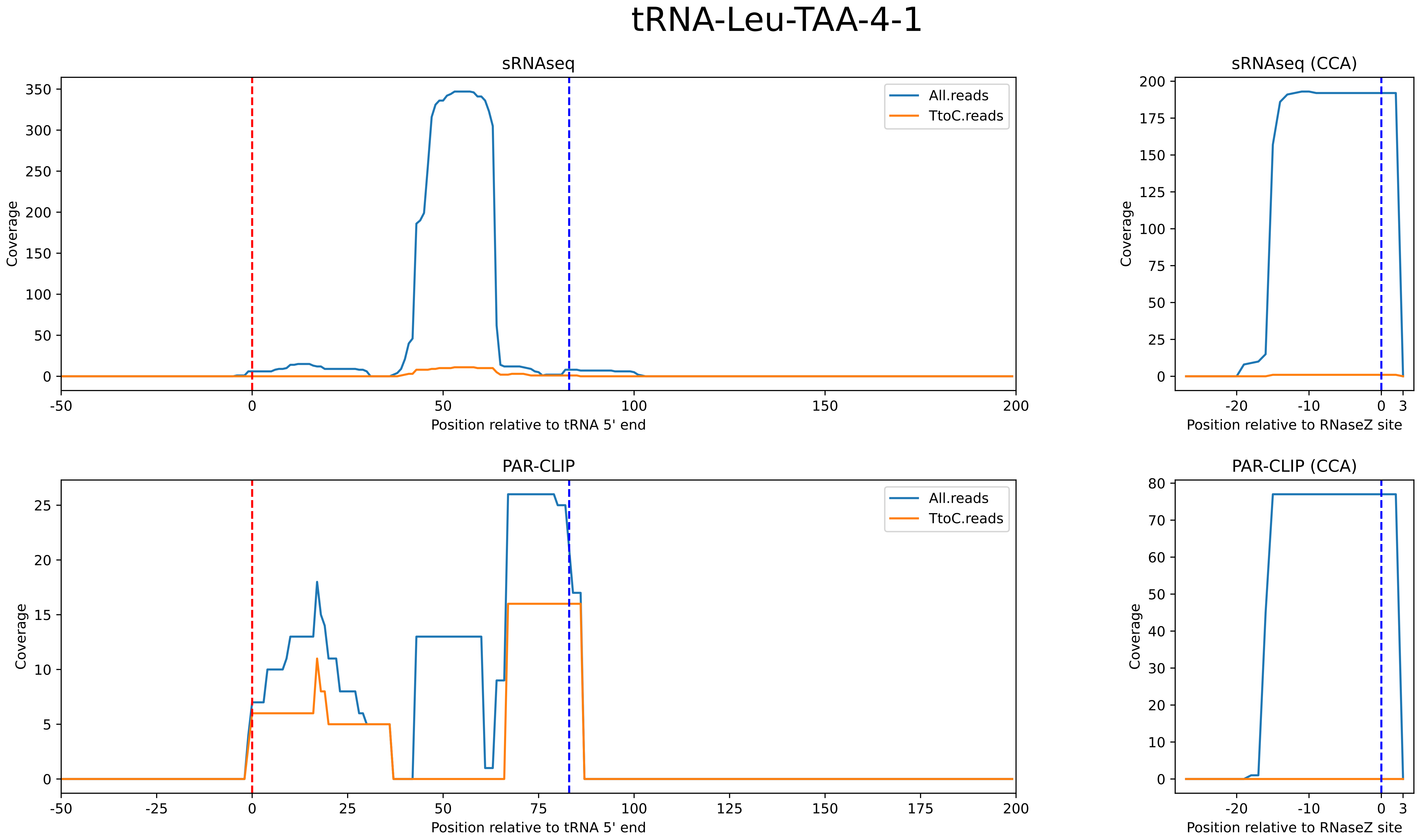

tRNA-Leu-TAA-4-1
sRNAseq
sRNAseq (CCA)
PAR-CLIP
PAR-CLIP (CCA)
All.reads
TtoC.reads
Coverage
Position relative to tRNA 5' end
Position relative to RNaseZ site

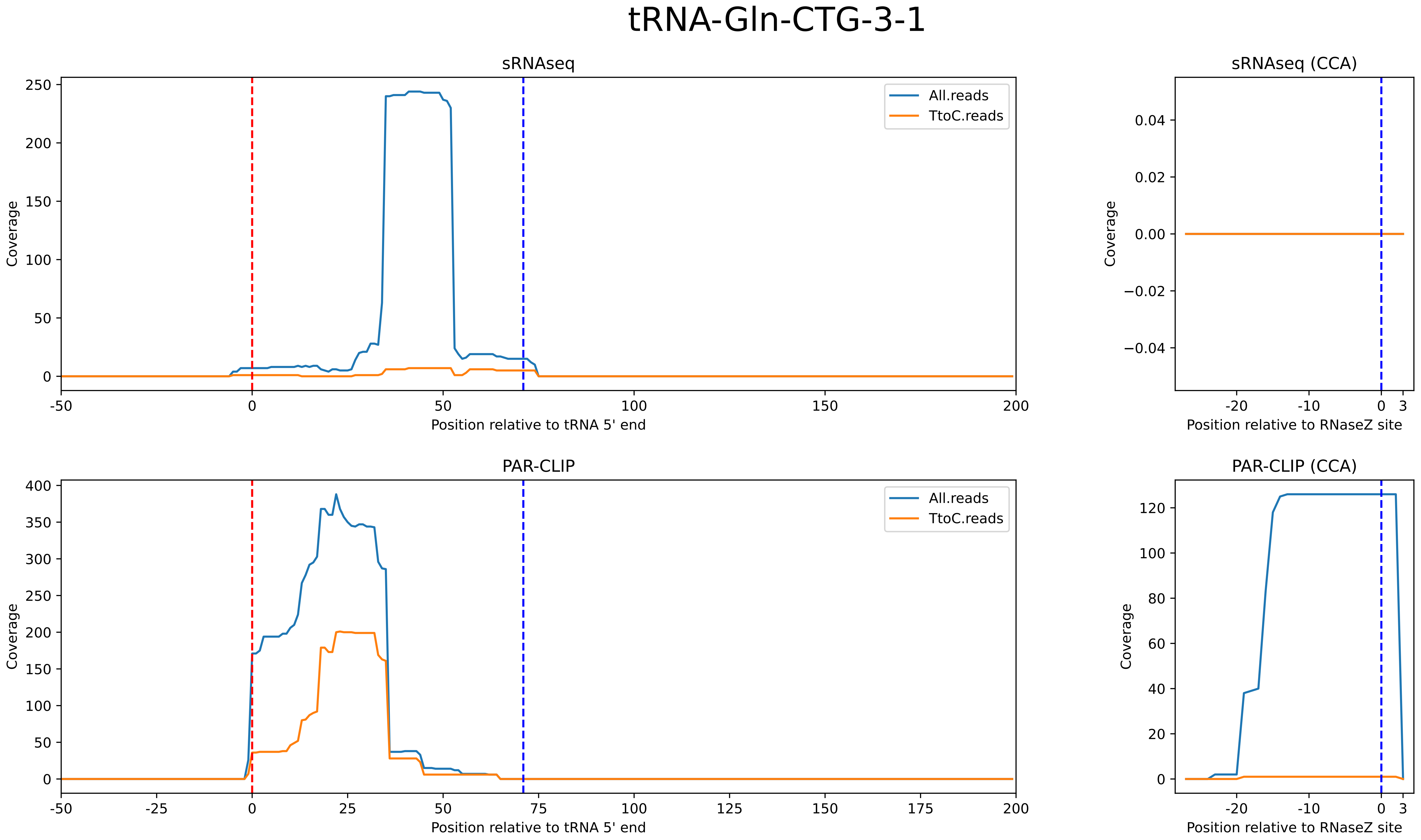
tRNA-Gln-CTG-3-1
sRNAseq
sRNAseq (CCA)
PAR-CLIP
PAR-CLIP (CCA)
All.reads
TtoC.reads
Coverage
Position relative to tRNA 5' end
Position relative to RNaseZ site

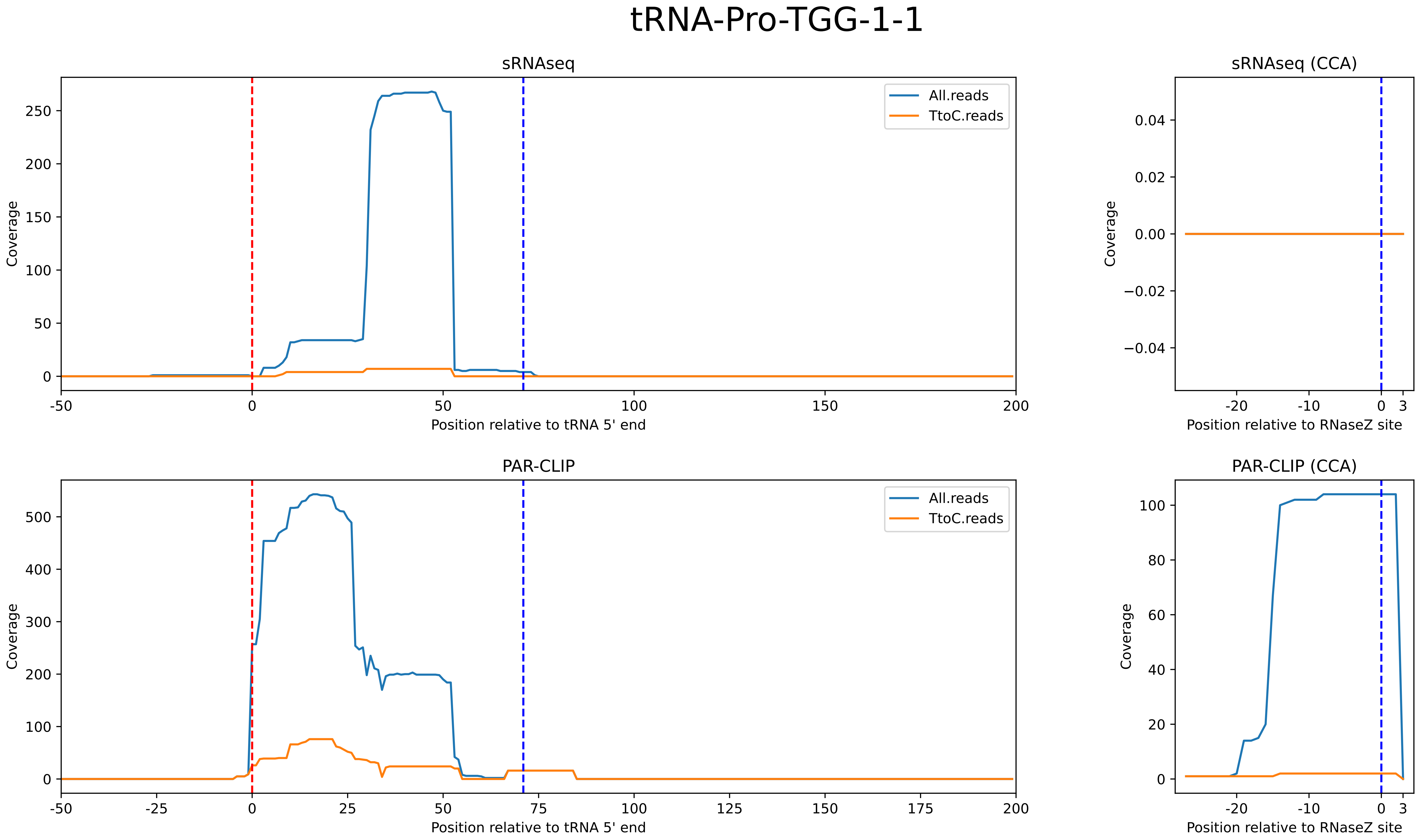
tRNA-Pro-TGG-1-1
sRNAseq
sRNAseq (CCA)
PAR-CLIP
PAR-CLIP (CCA)
All.reads
TtoC.reads
Coverage
Position relative to tRNA 5' end
Position relative to RNaseZ site

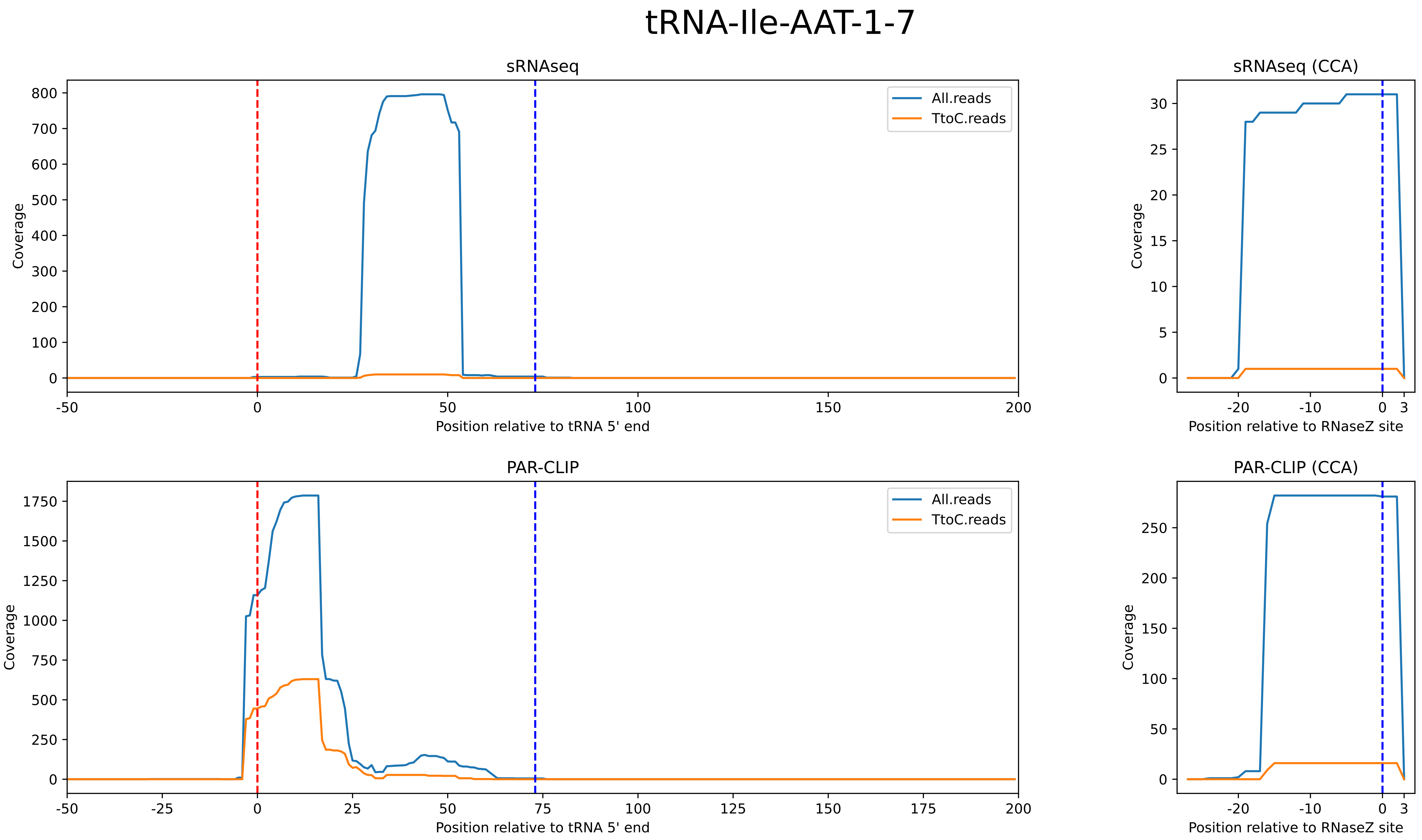
tRNA-Ile-AAT-1-7
sRNAseq
sRNAseq (CCA)
PAR-CLIP
PAR-CLIP (CCA)
All.reads
TtoC.reads
Coverage
Position relative to tRNA 5' end
Position relative to RNaseZ site

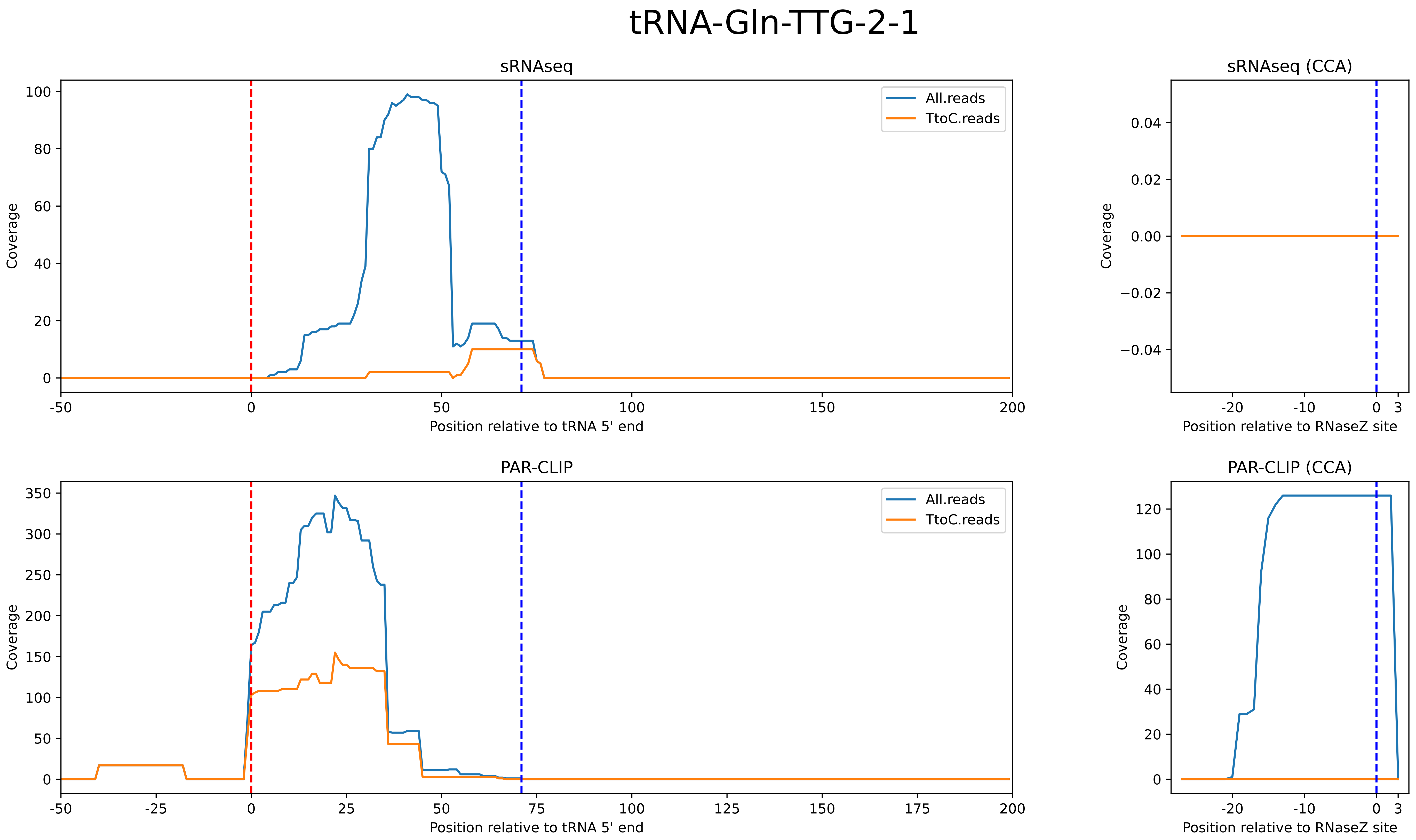
tRNA-Gln-TTG-2-1
sRNAseq
All.reads
TtoC.reads
Coverage
Position relative to tRNA 5' end
sRNAseq (CCA)
Position relative to RNaseZ site
PAR-CLIP
PAR-CLIP (CCA)

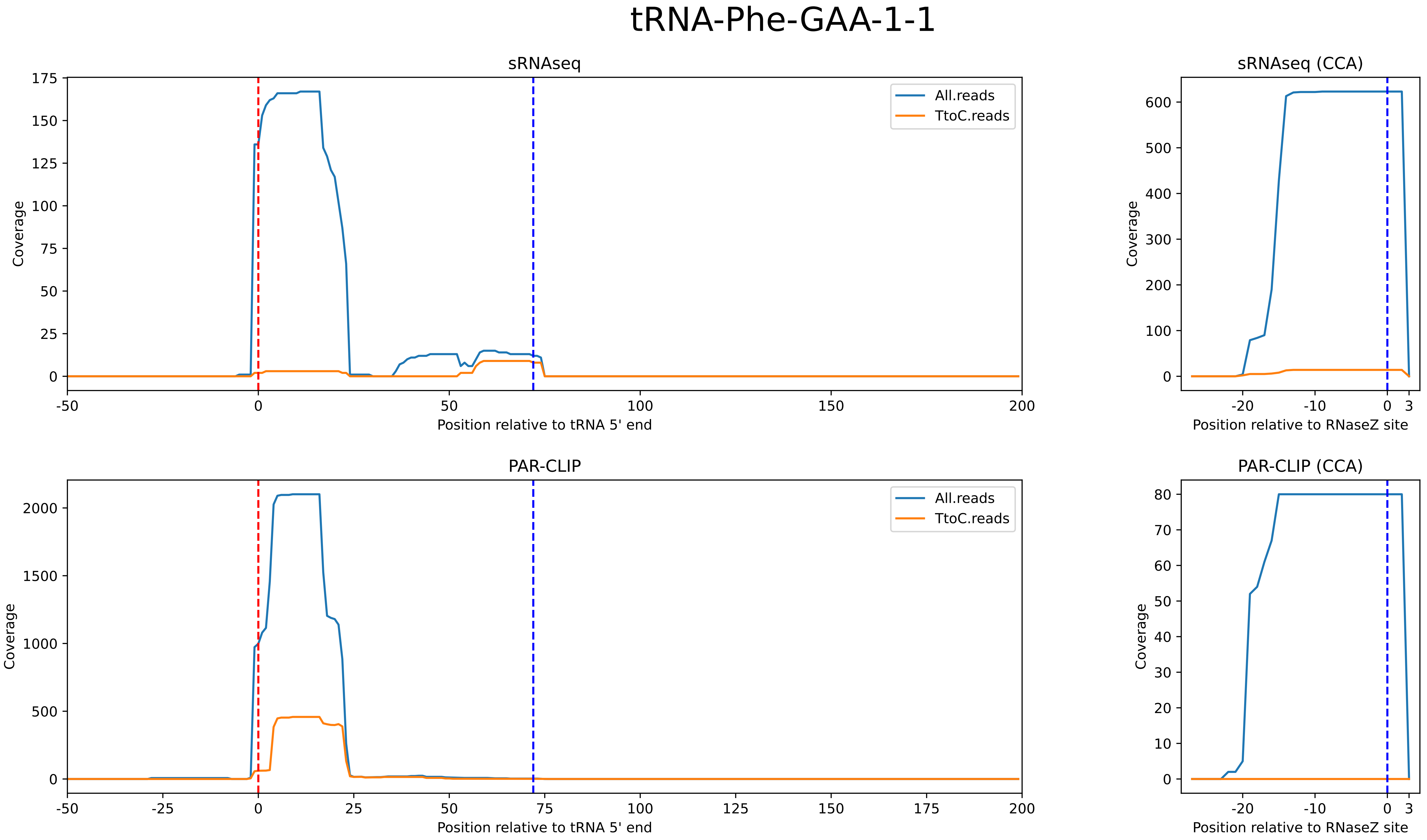
tRNA-Phe-GAA-1-1
sRNAseq
All.reads
TtoC.reads
Coverage
Position relative to tRNA 5' end
sRNAseq (CCA)
Position relative to RNaseZ site
PAR-CLIP
PAR-CLIP (CCA)

# tRNA-Ile-AAT-1-9

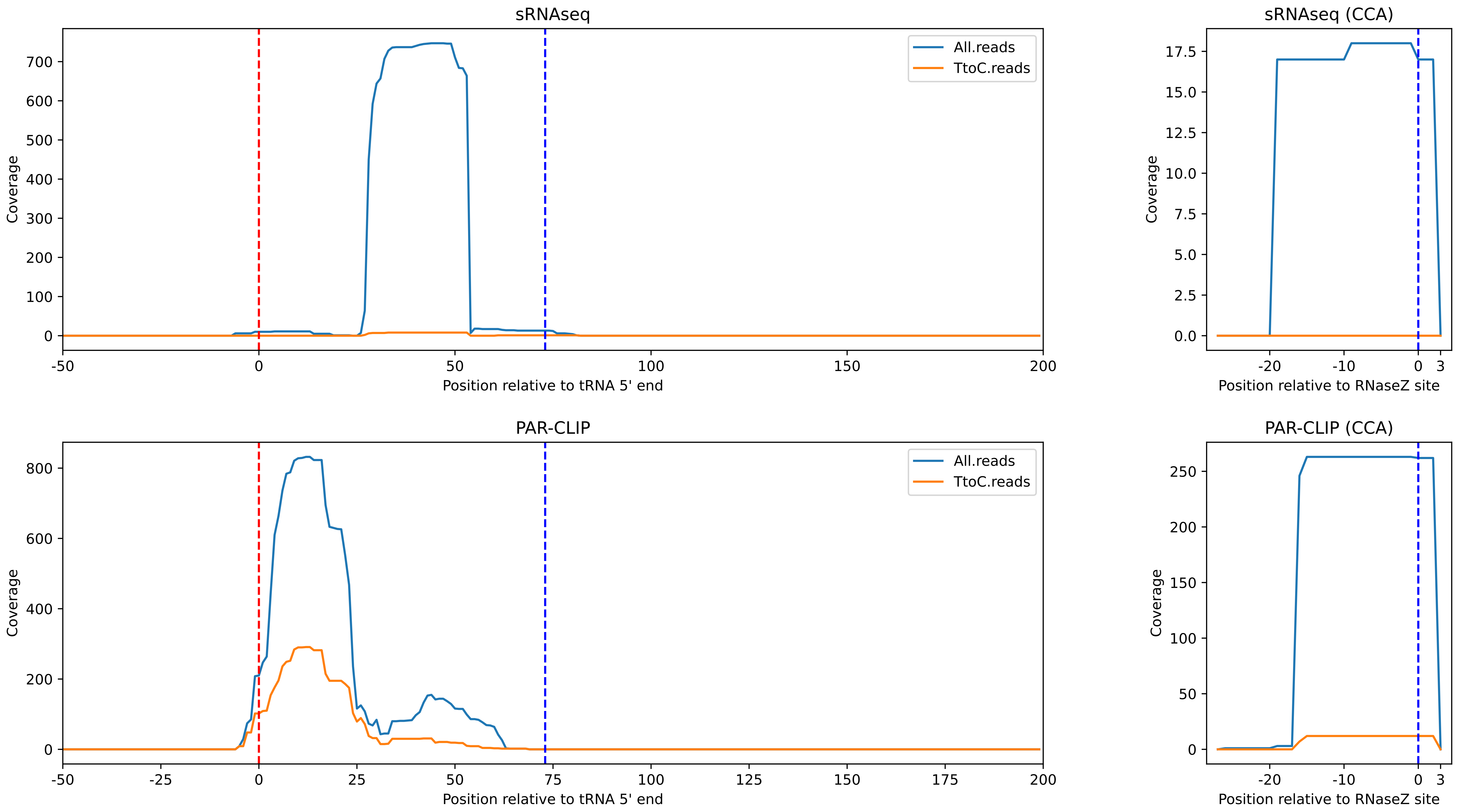

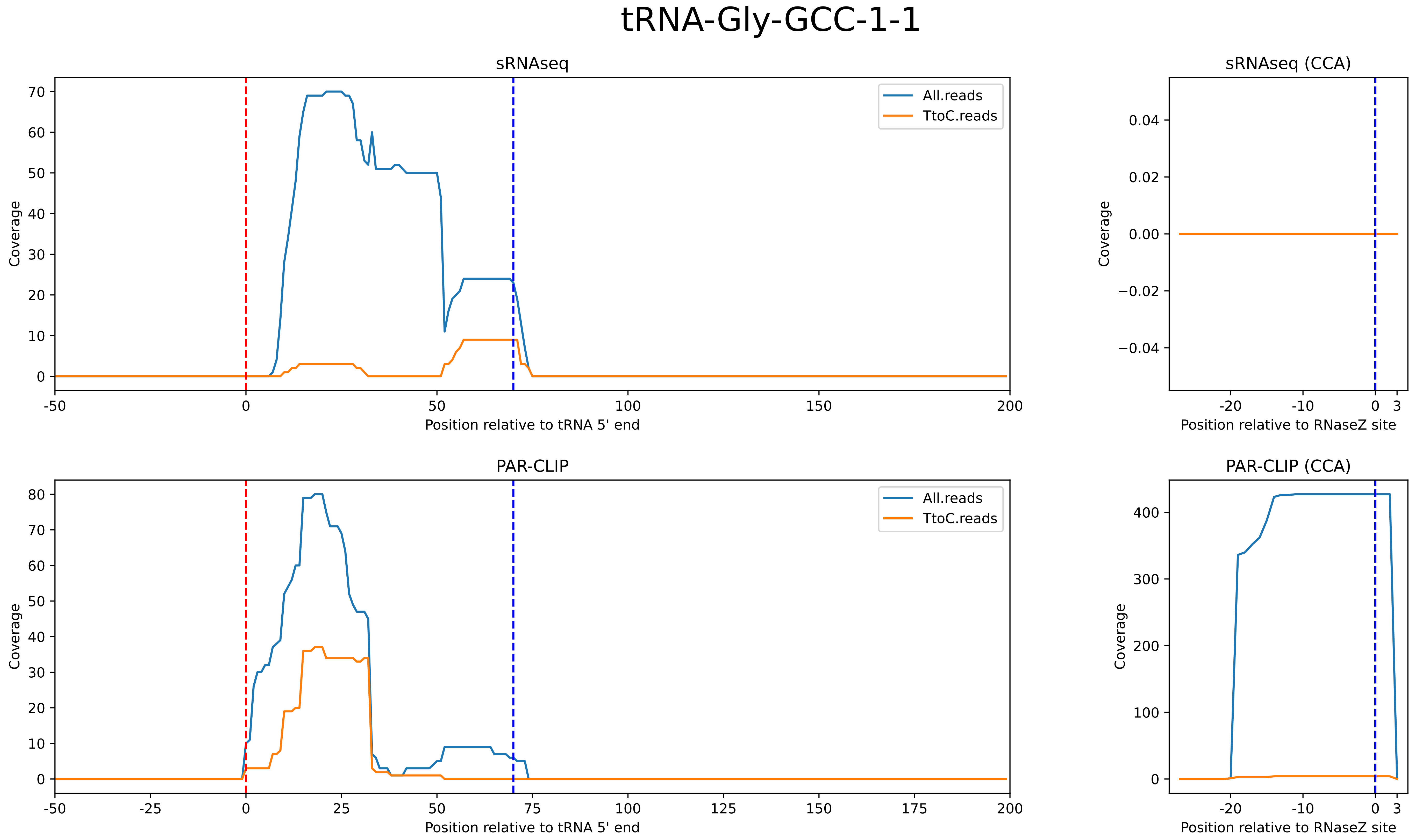
tRNA-Gly-GCC-1-1
sRNAseq
sRNAseq (CCA)
PAR-CLIP
PAR-CLIP (CCA)
All.reads
TtoC.reads
Coverage
Position relative to tRNA 5' end
Position relative to RNaseZ site

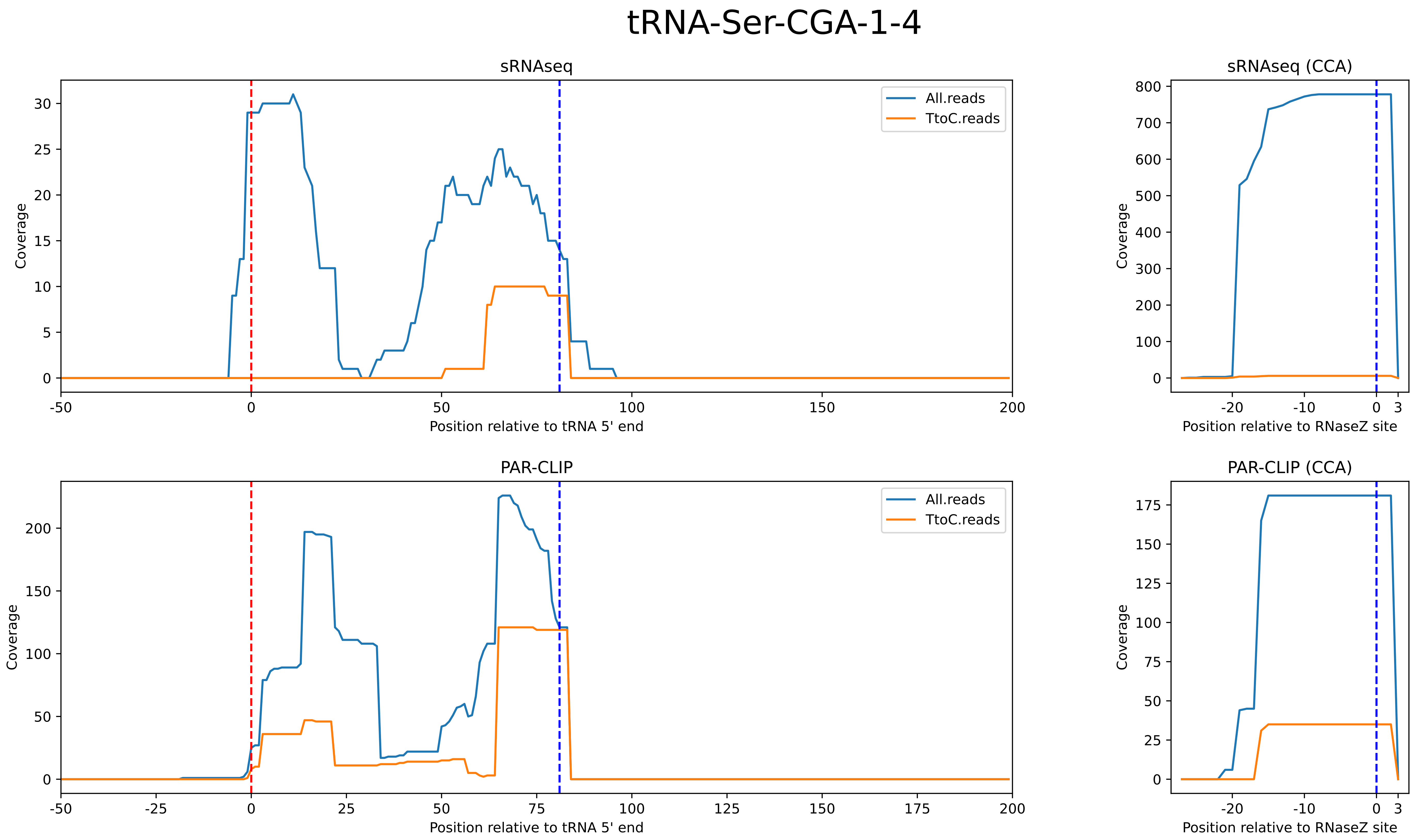
tRNA-Ser-CGA-1-4
sRNAseq
All.reads
TtoC.reads
Coverage
Position relative to tRNA 5' end
sRNAseq (CCA)
Position relative to RNaseZ site
PAR-CLIP
PAR-CLIP (CCA)

# tRNA-Val-AAC-2-1

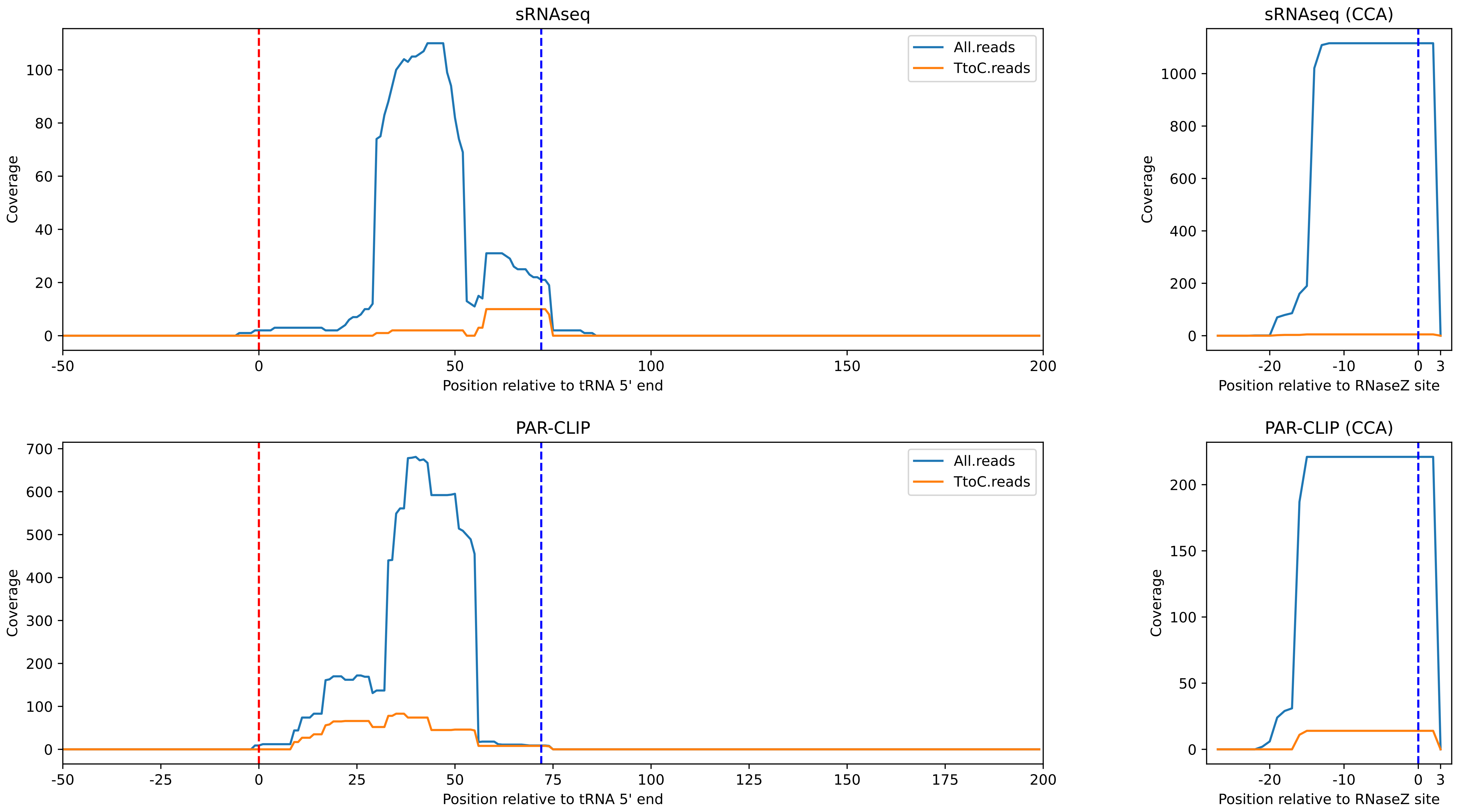

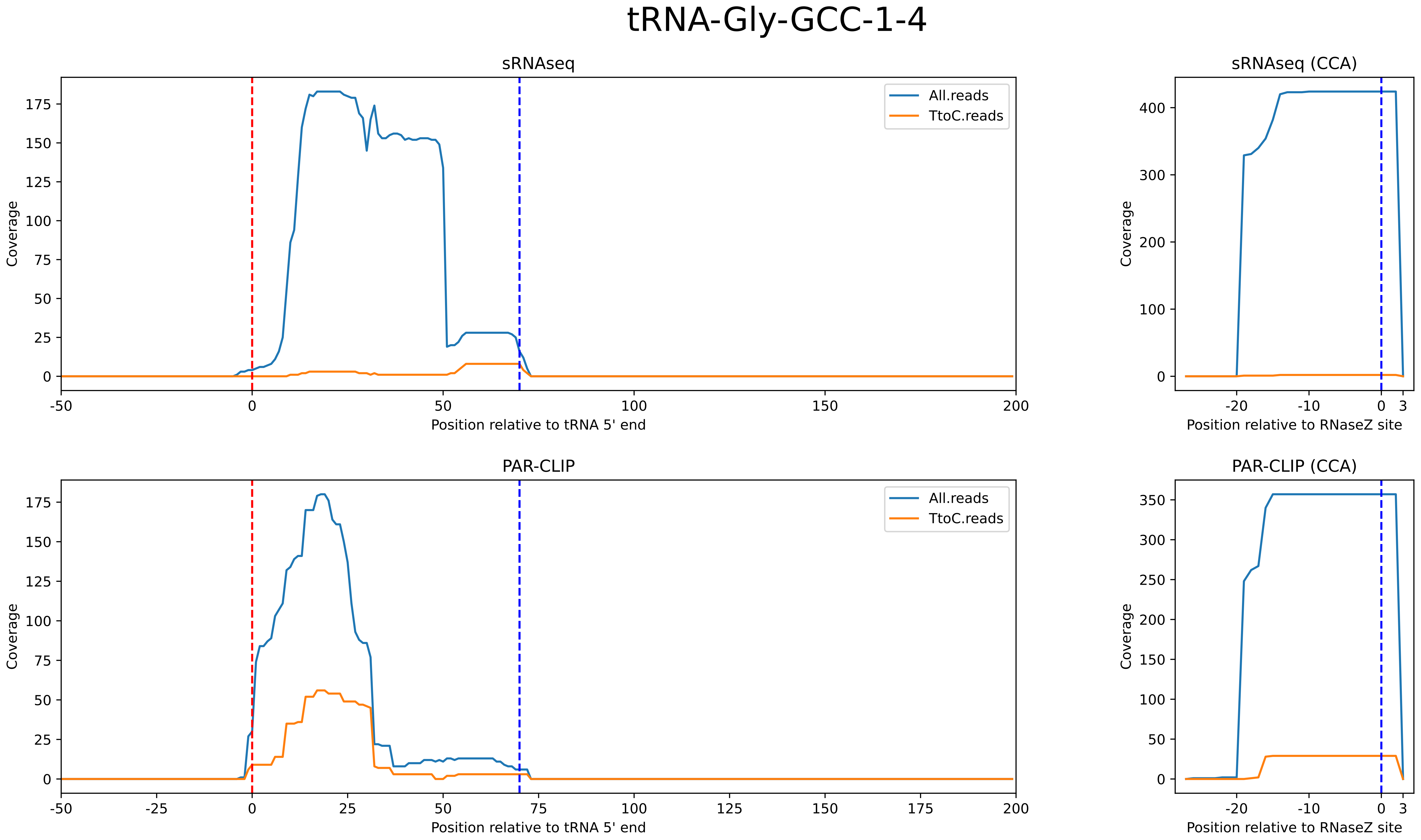
tRNA-Gly-GCC-1-4
sRNAseq
sRNAseq (CCA)
PAR-CLIP
PAR-CLIP (CCA)
All.reads
TtoC.reads
Coverage
Position relative to tRNA 5' end
Position relative to RNaseZ site

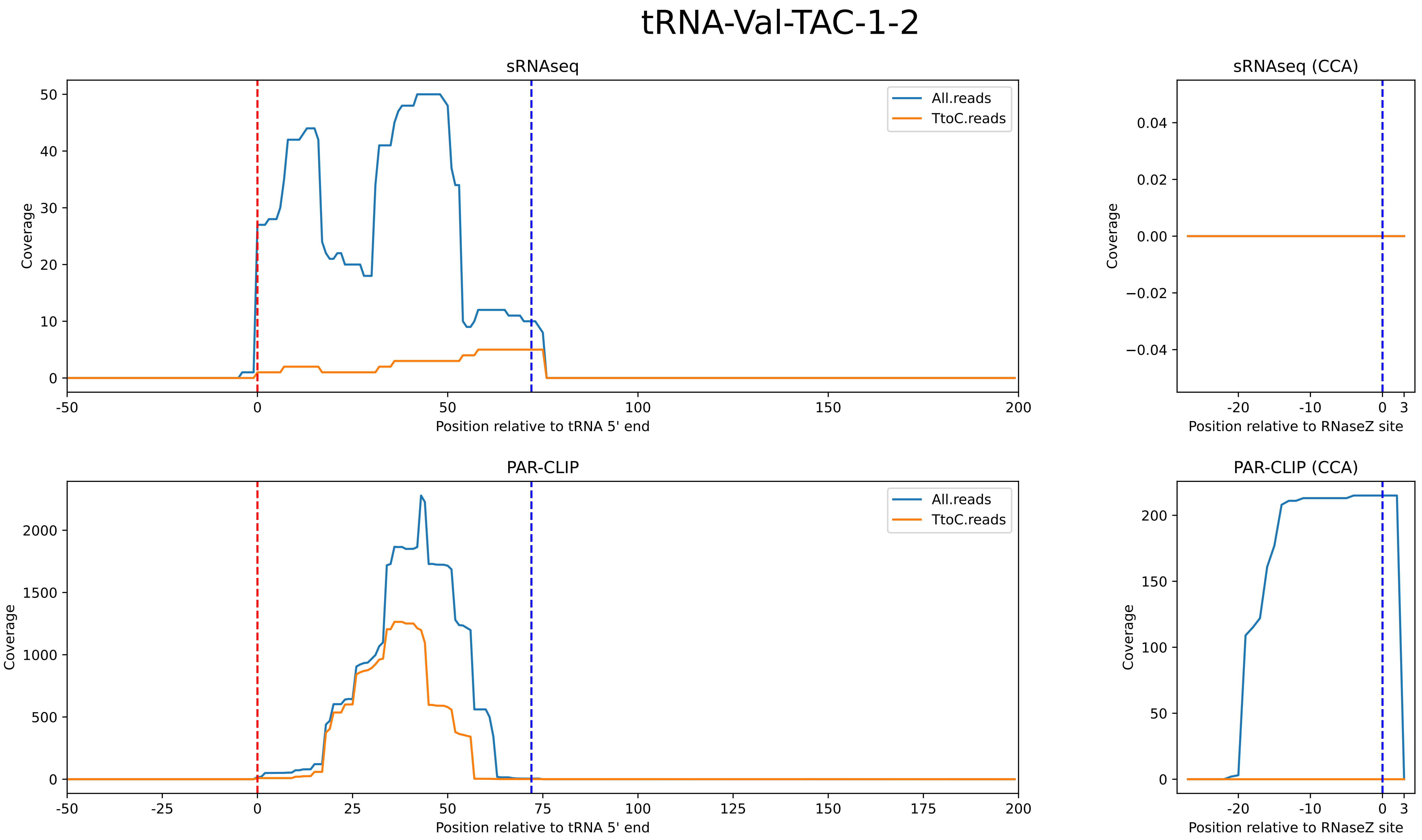

tRNA-Val-TAC-1-2
sRNAseq
All.reads
TtoC.reads
Coverage
Position relative to tRNA 5' end
sRNAseq (CCA)
Position relative to RNaseZ site
PAR-CLIP
PAR-CLIP (CCA)

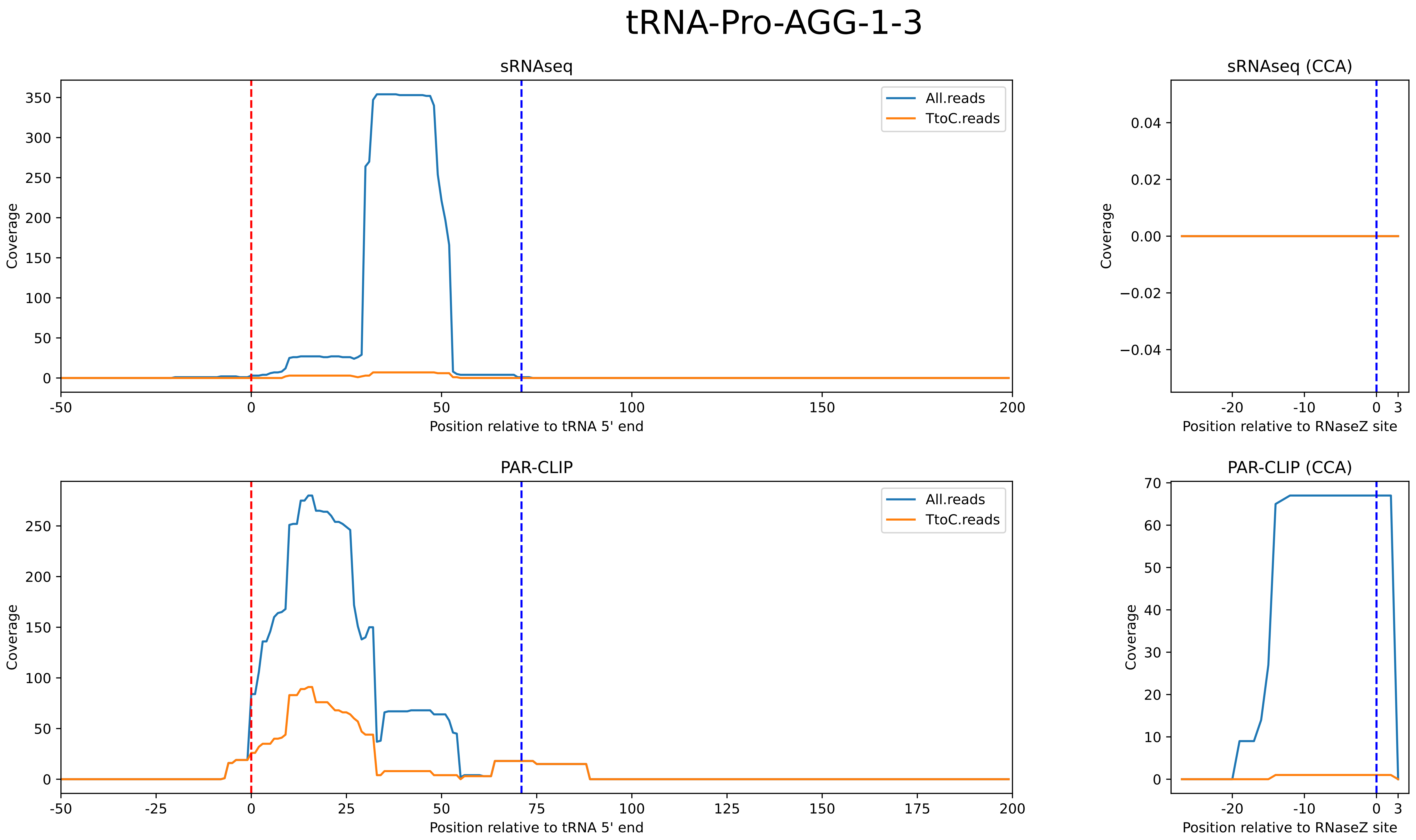

tRNA-Pro-AGG-1-3
sRNAseq
All.reads
TtoC.reads
Coverage
Position relative to tRNA 5' end
sRNAseq (CCA)
Position relative to RNaseZ site
PAR-CLIP
PAR-CLIP (CCA)

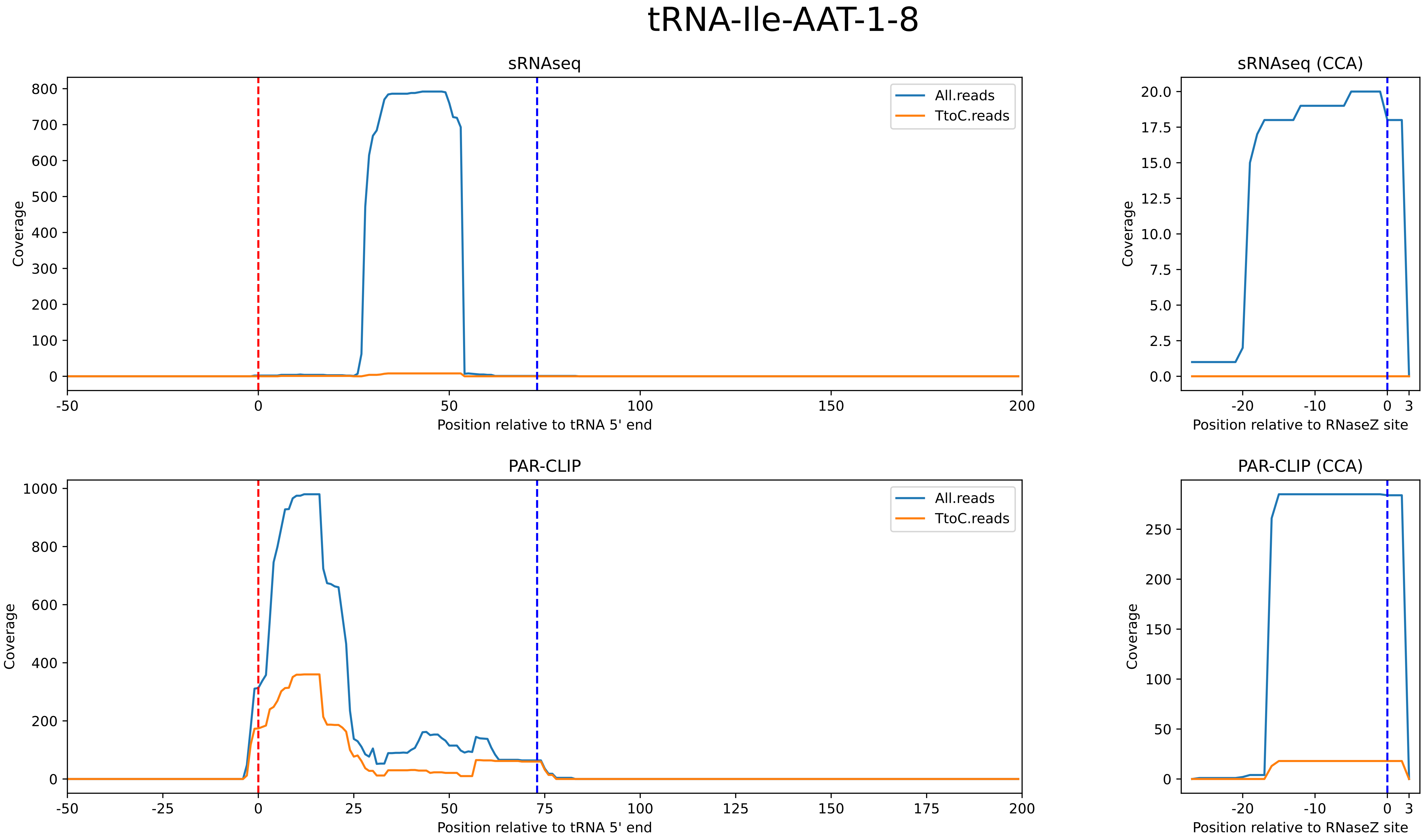

tRNA-Ile-AAT-1-8
sRNAseq
sRNAseq (CCA)
PAR-CLIP
PAR-CLIP (CCA)
All.reads
TtoC.reads
Coverage
Position relative to tRNA 5' end
Position relative to RNaseZ site

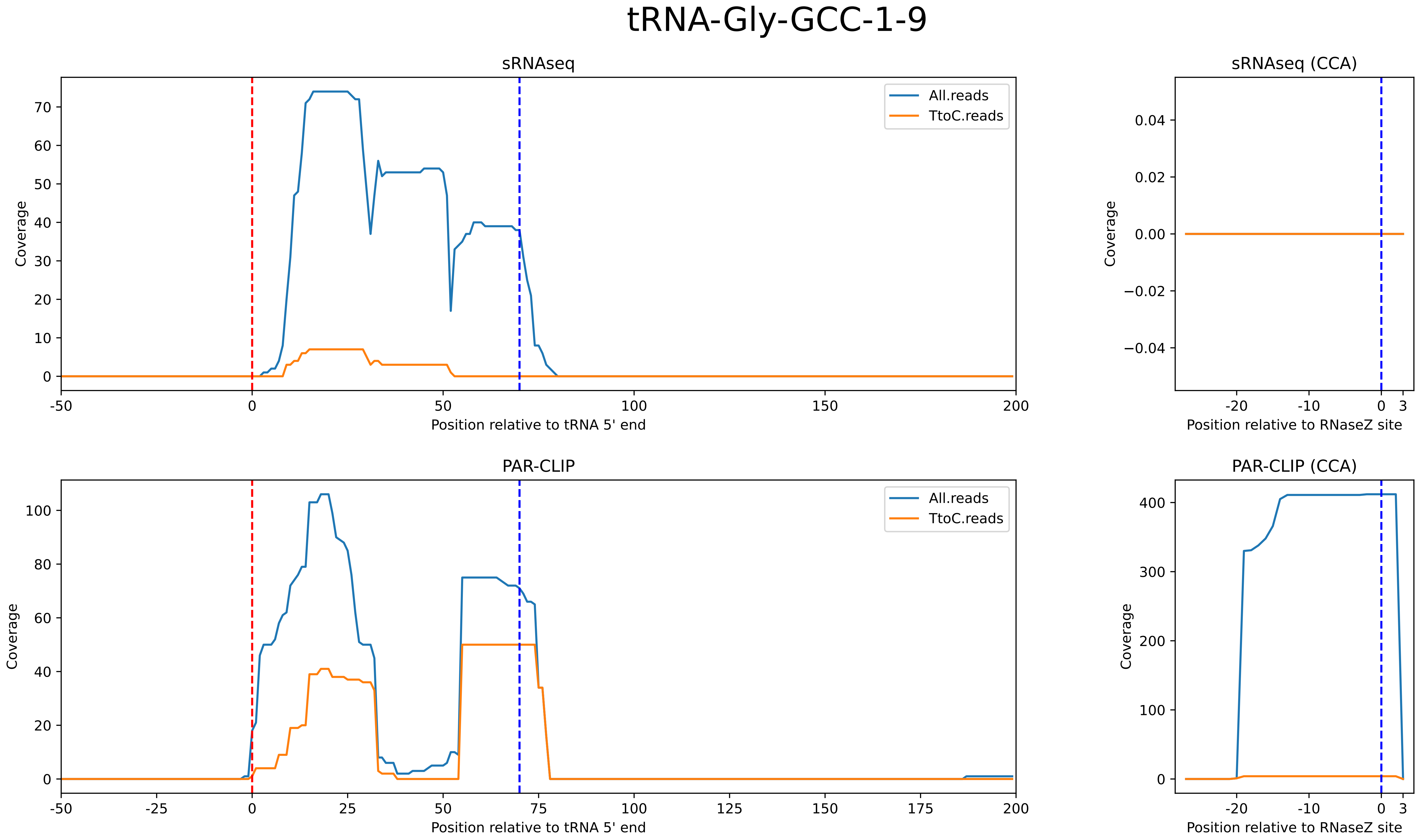

tRNA-Gly-GCC-1-9
sRNAseq
sRNAseq (CCA)
PAR-CLIP
PAR-CLIP (CCA)
All.reads
TtoC.reads
Coverage
Position relative to tRNA 5' end
Position relative to RNaseZ site

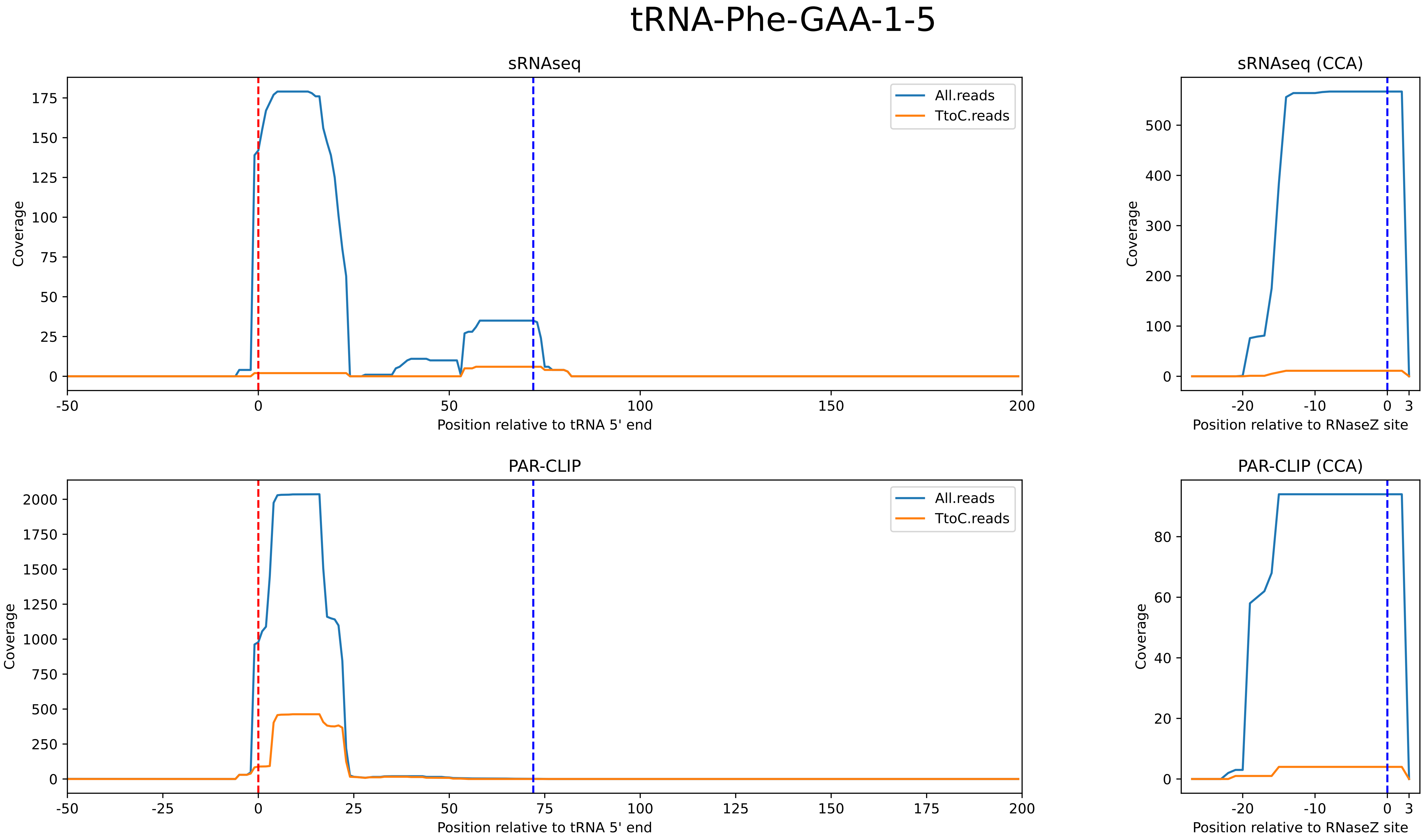
tRNA-Phe-GAA-1-5
sRNAseq
sRNAseq (CCA)
PAR-CLIP
PAR-CLIP (CCA)
All.reads
TtoC.reads
Coverage
Position relative to tRNA 5' end
Position relative to RNaseZ site

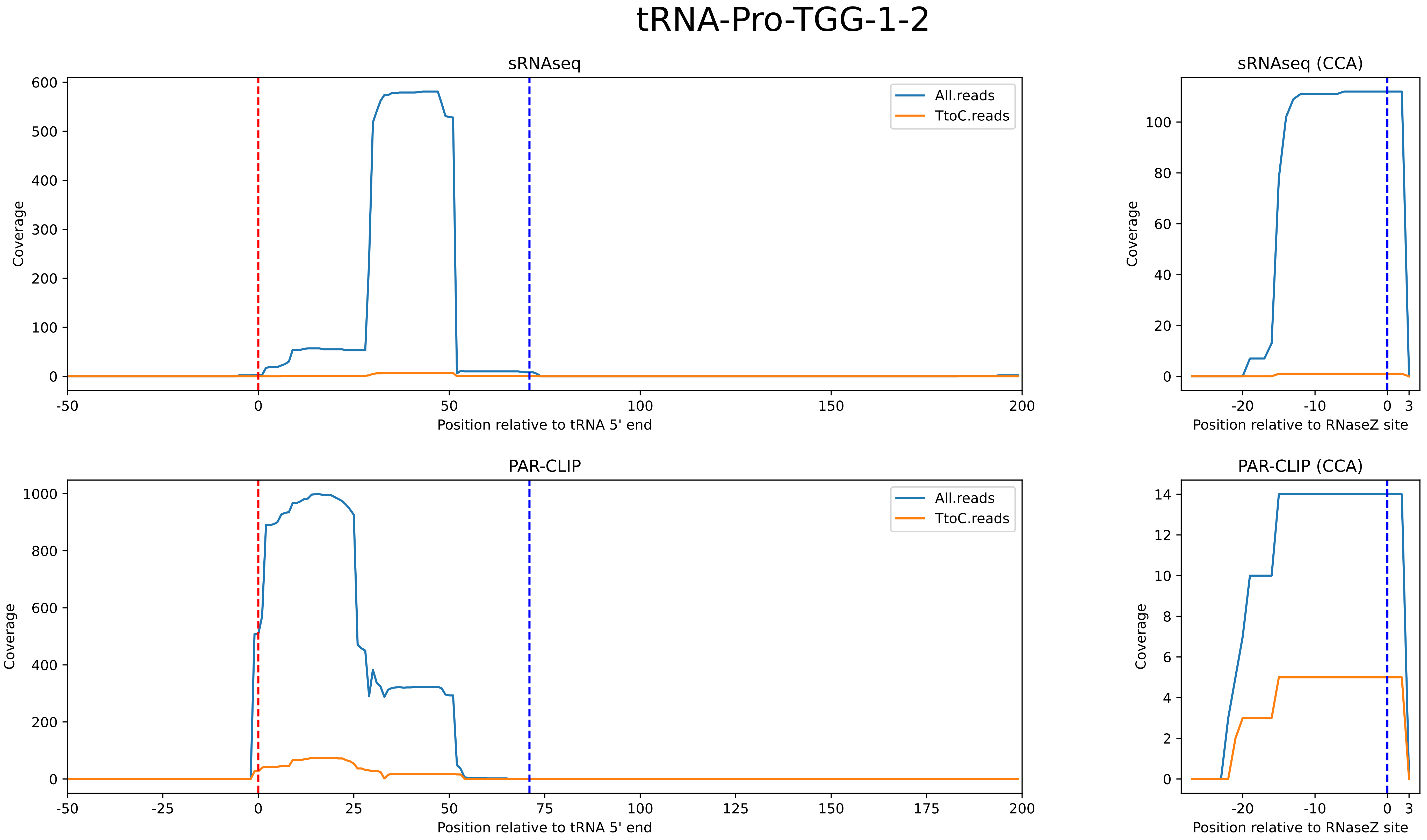
tRNA-Pro-TGG-1-2
sRNAseq
All.reads
TtoC.reads
Coverage
Position relative to tRNA 5' end
sRNAseq (CCA)
Coverage
Position relative to RNaseZ site
PAR-CLIP
All.reads
TtoC.reads
Coverage
Position relative to tRNA 5' end
PAR-CLIP (CCA)
Coverage
Position relative to RNaseZ site

# tRNA-Phe-GAA-1-7

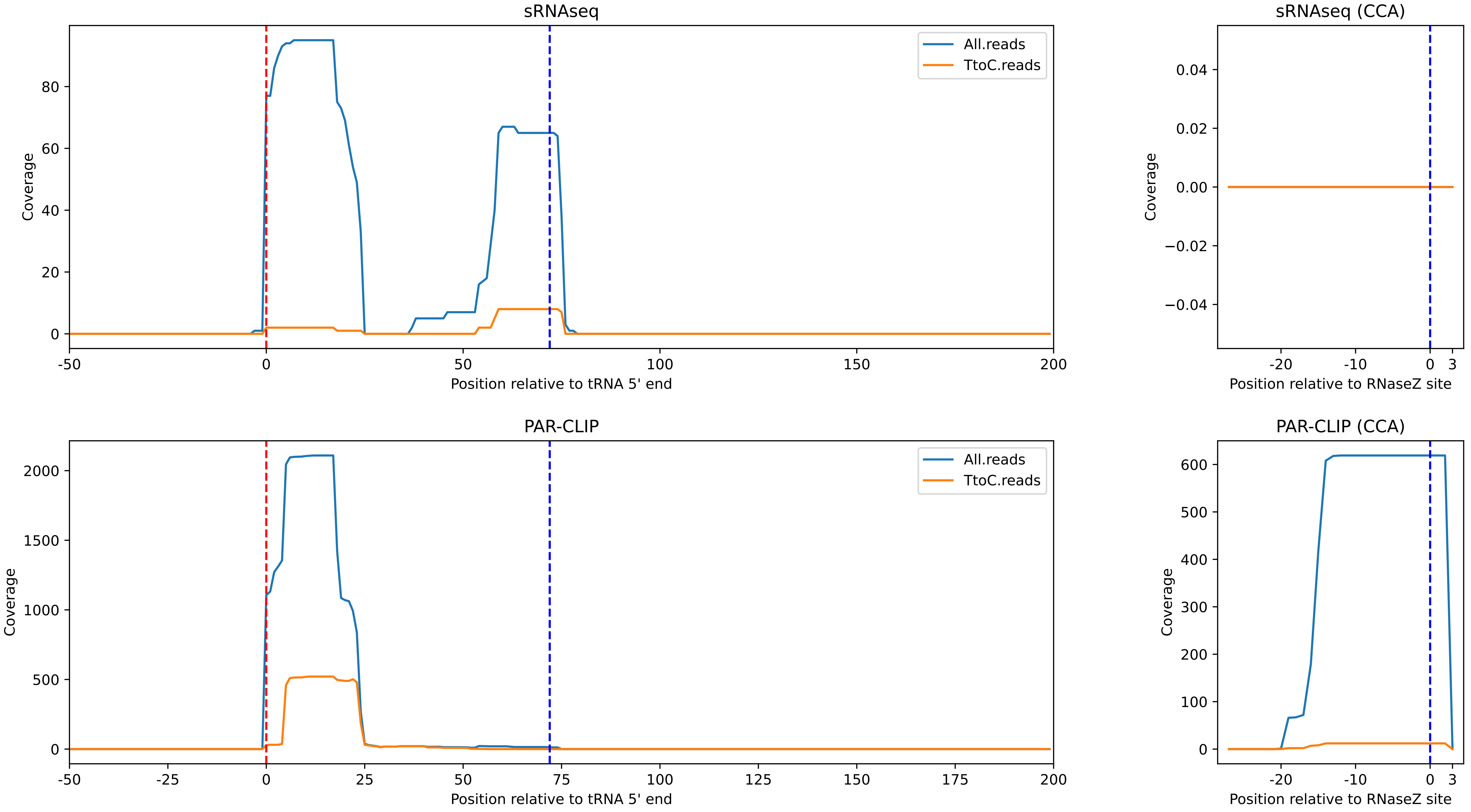

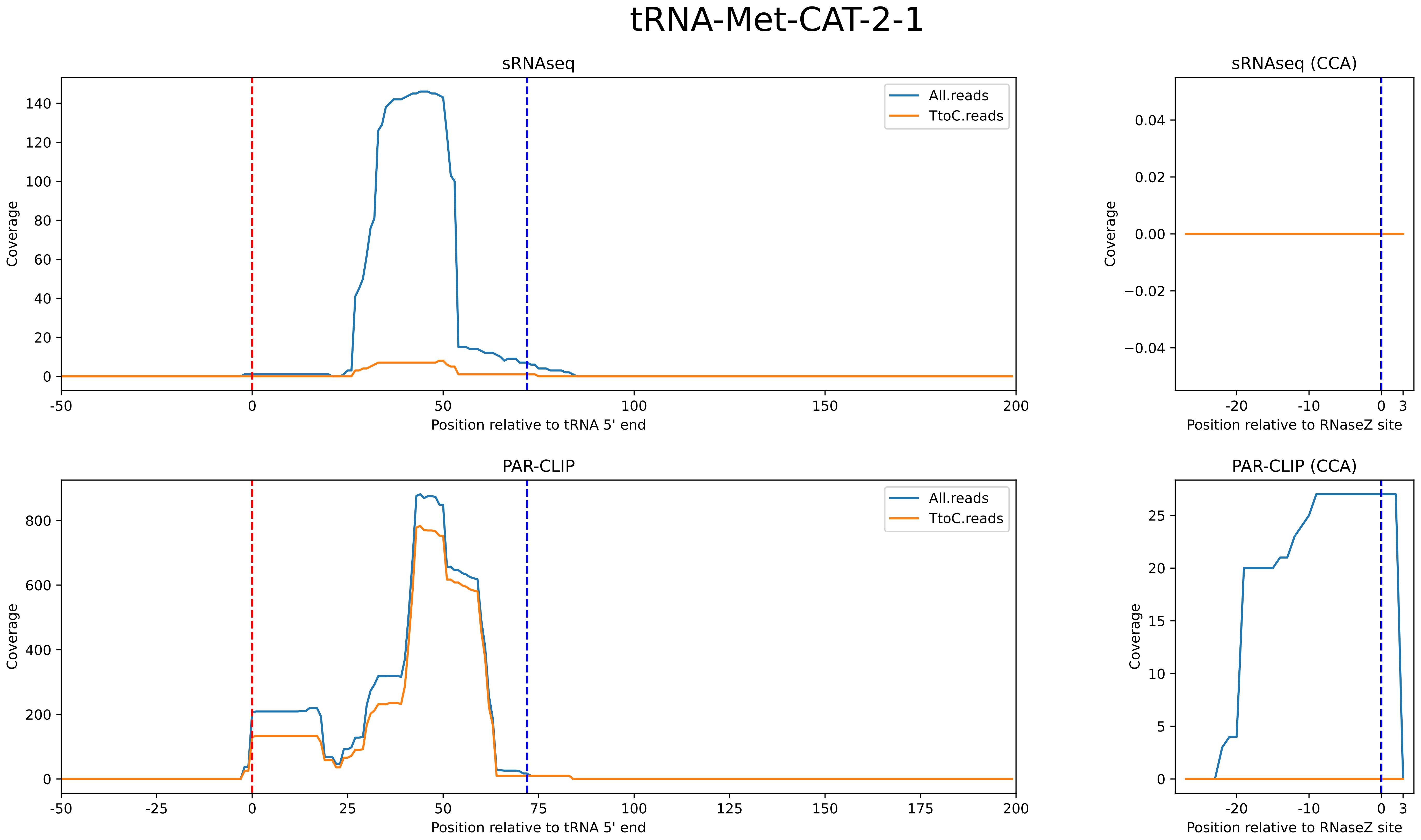
tRNA-Met-CAT-2-1
sRNAseq
All.reads
TtoC.reads
Coverage
Position relative to tRNA 5' end
sRNAseq (CCA)
Position relative to RNaseZ site
PAR-CLIP
PAR-CLIP (CCA)

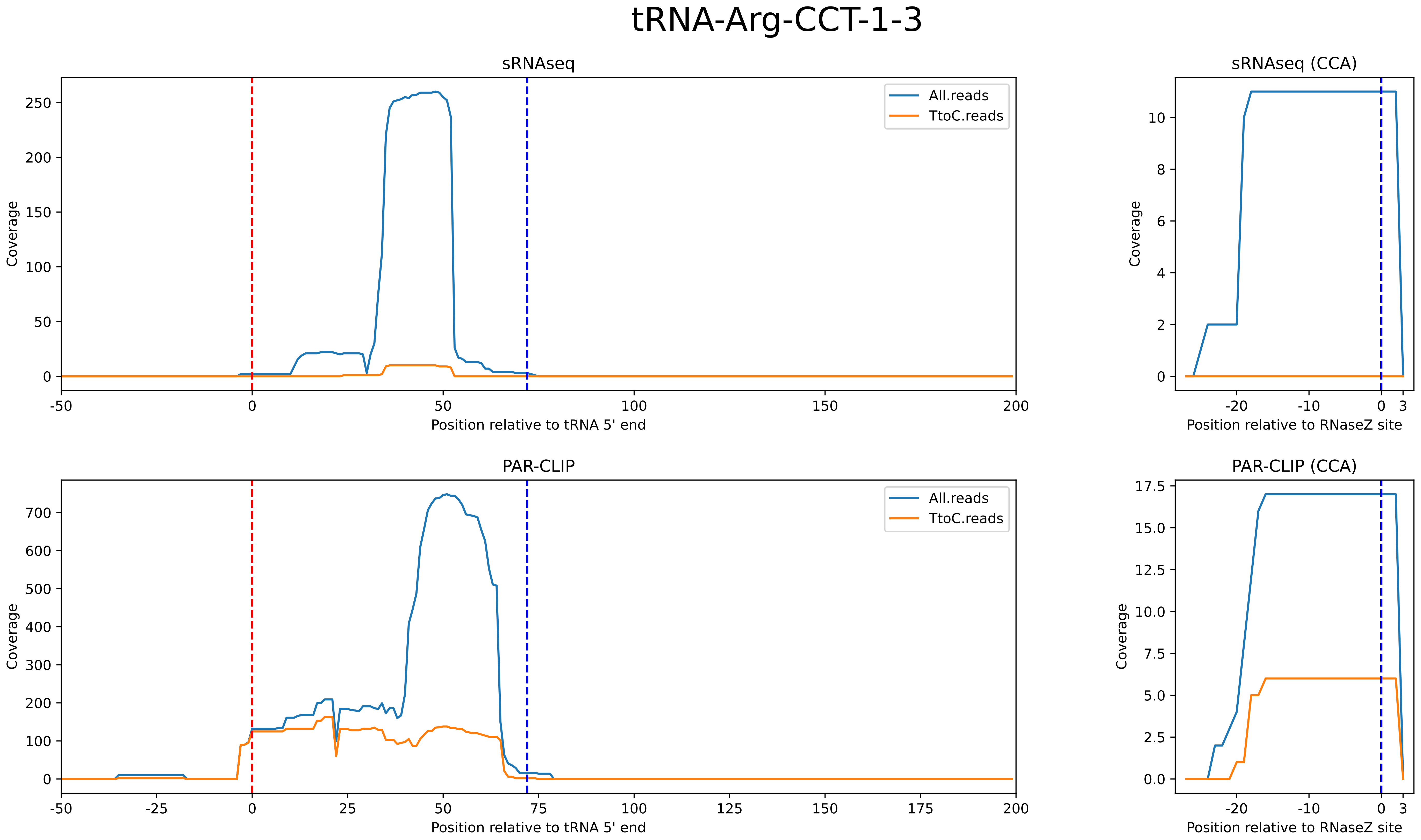

tRNA-Arg-CCT-1-3
sRNAseq
sRNAseq (CCA)
PAR-CLIP
PAR-CLIP (CCA)
All.reads
TtoC.reads
Coverage
Position relative to tRNA 5' end
Position relative to RNaseZ site

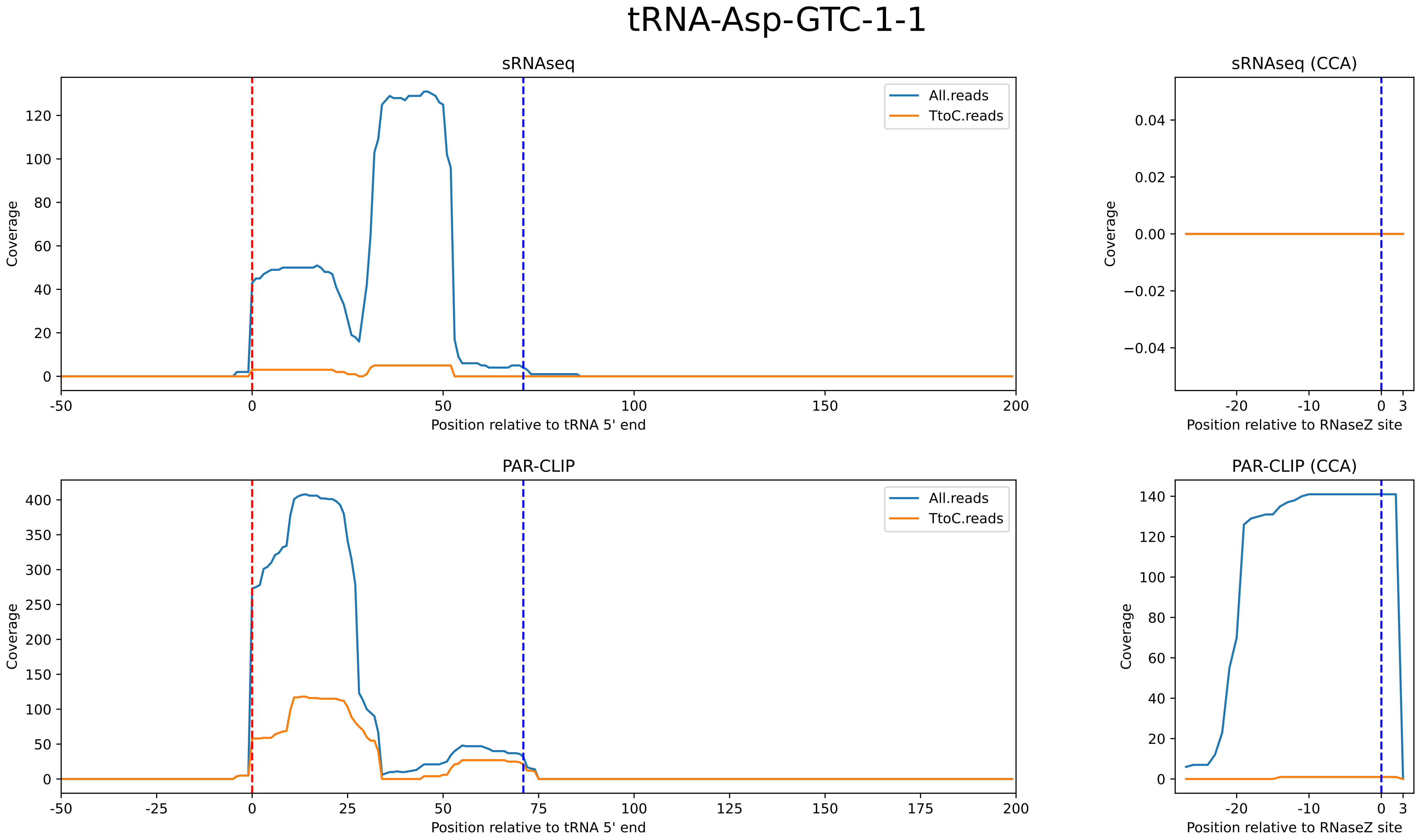

tRNA-Asp-GTC-1-1
sRNAseq
All.reads
TtoC.reads
Coverage
Position relative to tRNA 5' end
sRNAseq (CCA)
Position relative to RNaseZ site
PAR-CLIP
PAR-CLIP (CCA)

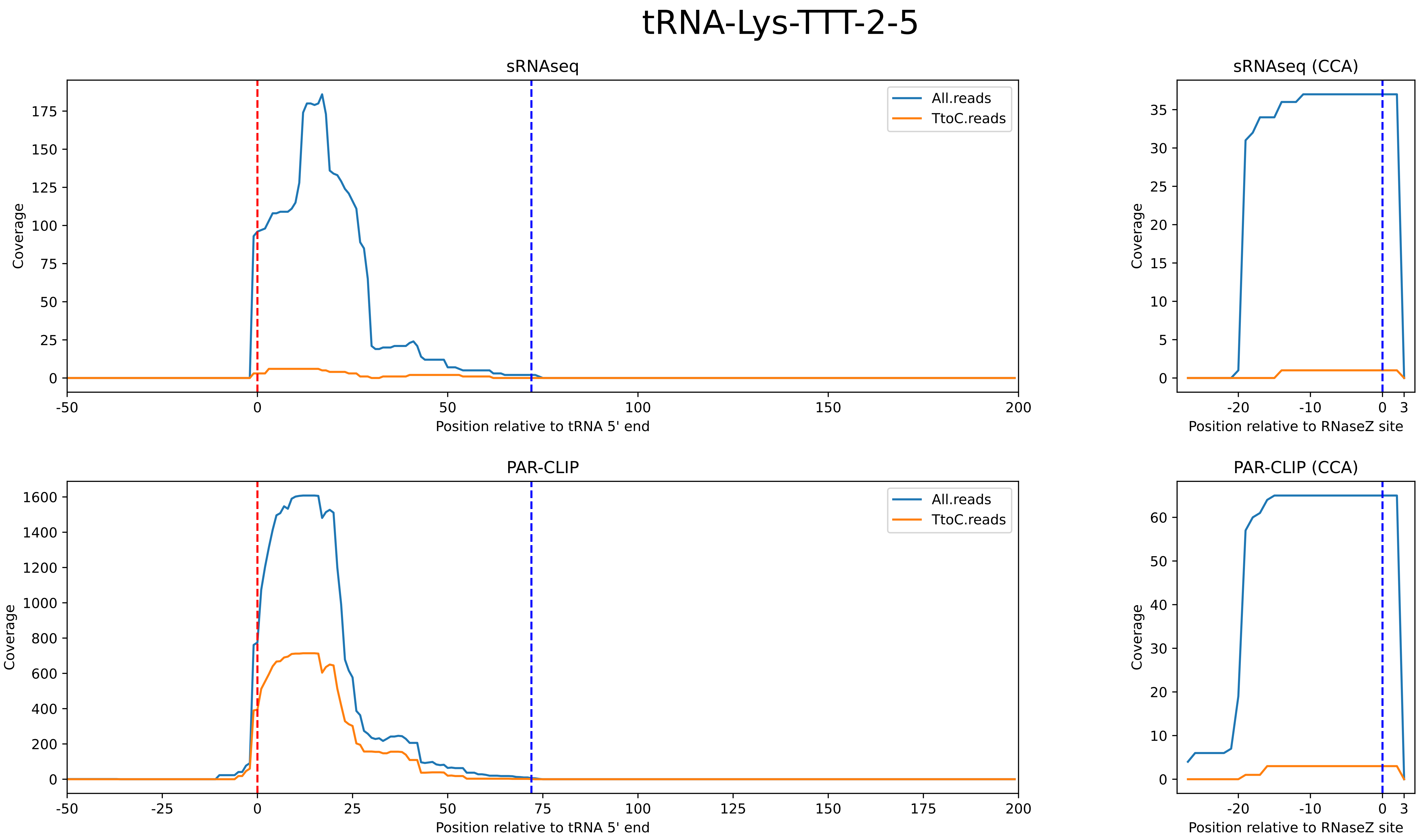
tRNA-Lys-TTT-2-5
sRNAseq
All.reads
TtoC.reads
Coverage
Position relative to tRNA 5' end
sRNAseq (CCA)
Position relative to RNaseZ site
PAR-CLIP
PAR-CLIP (CCA)

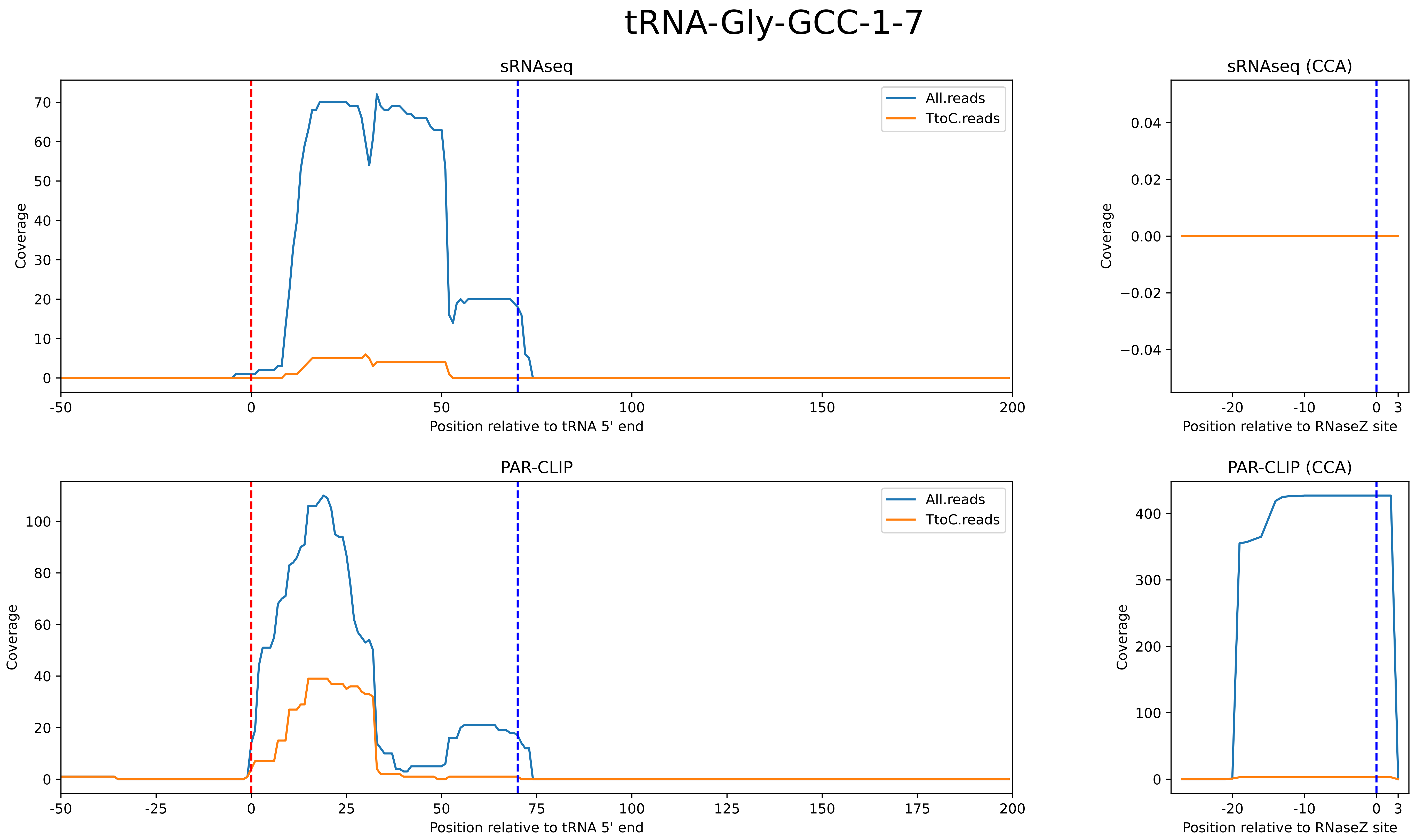
tRNA-Gly-GCC-1-7
sRNAseq
sRNAseq (CCA)
PAR-CLIP
PAR-CLIP (CCA)
All.reads
TtoC.reads
Coverage
Position relative to tRNA 5' end
Position relative to RNaseZ site

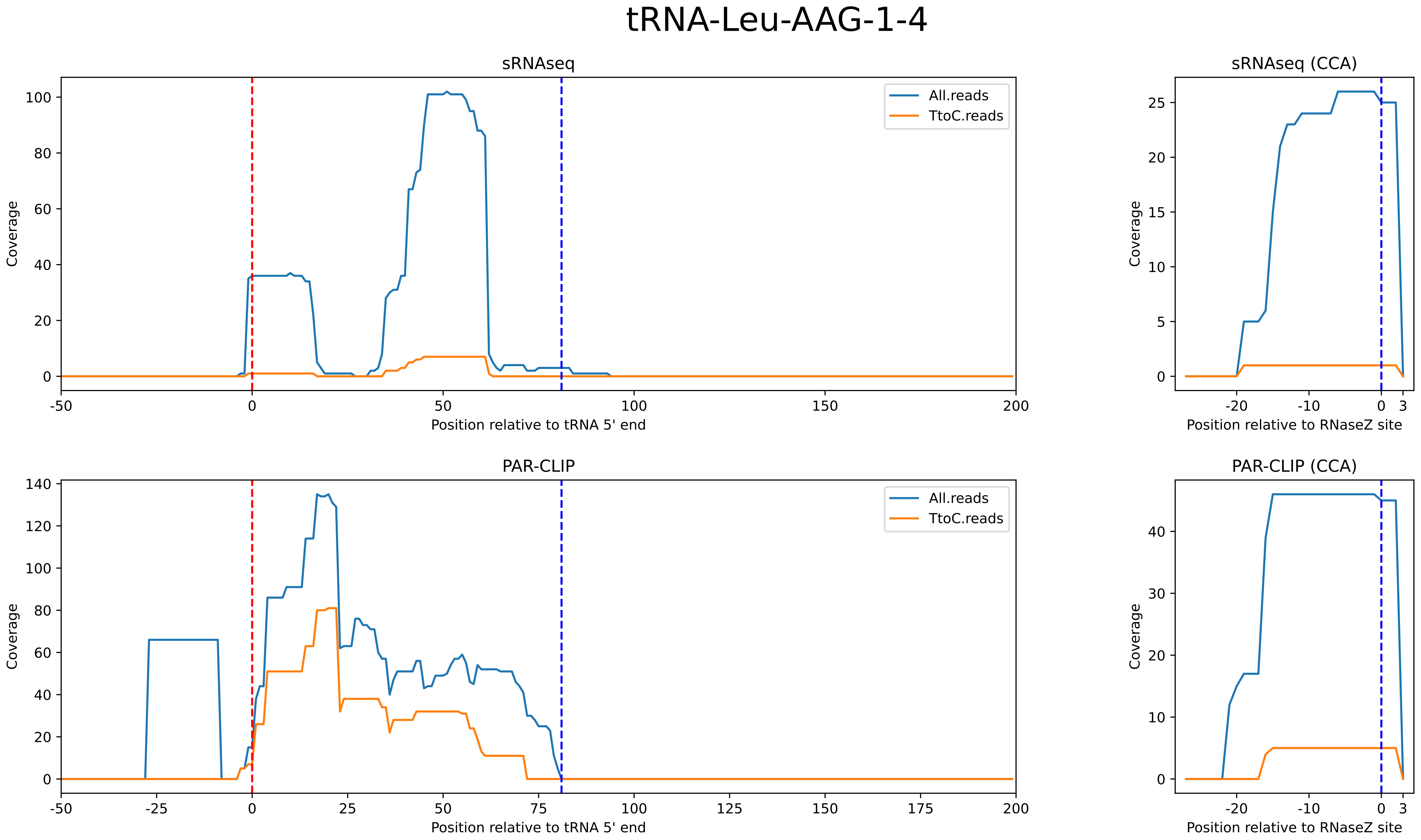
tRNA-Leu-AAG-1-4
sRNAseq
All.reads
TtoC.reads
Coverage
Position relative to tRNA 5' end
sRNAseq (CCA)
Position relative to RNaseZ site
PAR-CLIP
PAR-CLIP (CCA)

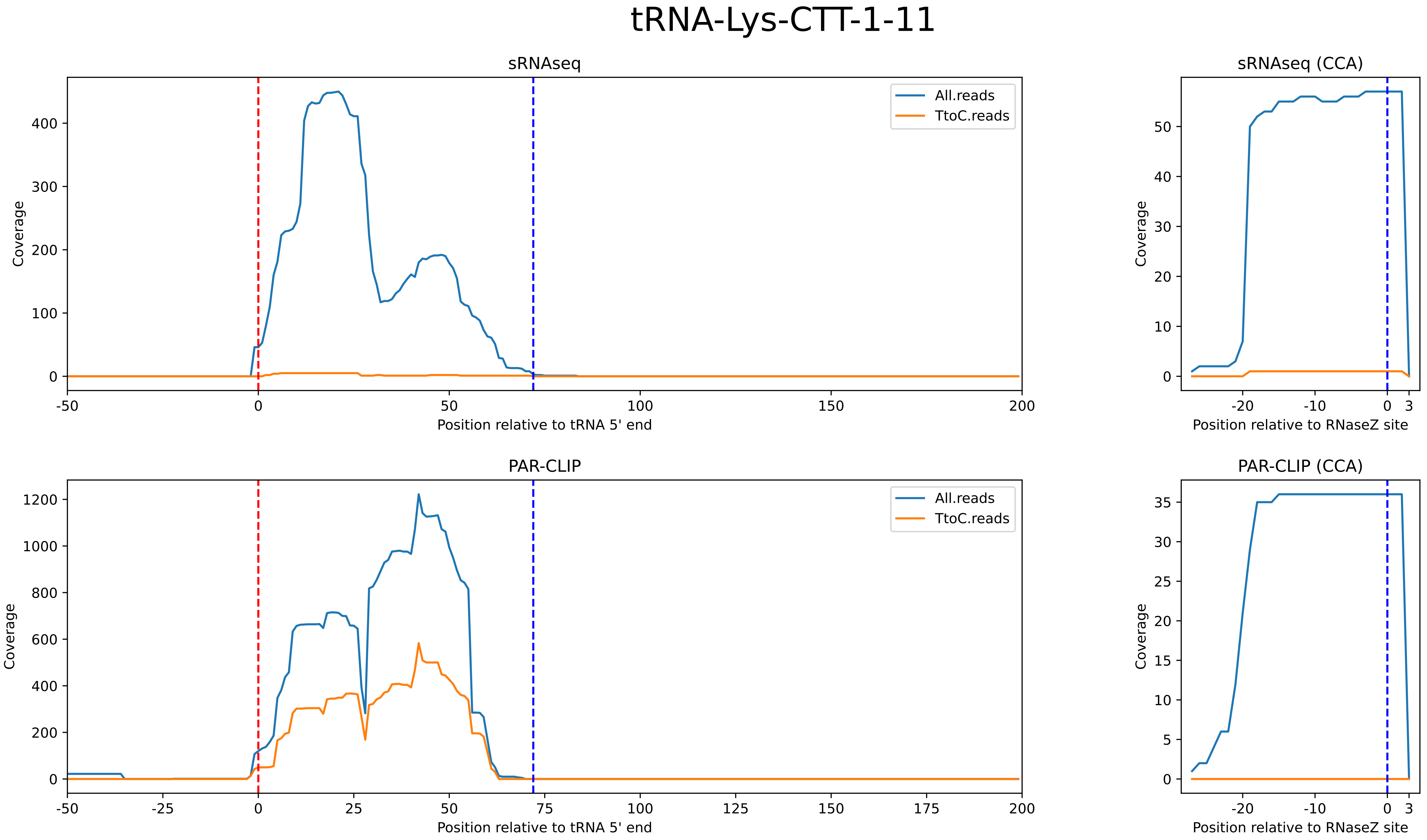

tRNA-Lys-CTT-1-11
sRNAseq
sRNAseq (CCA)
PAR-CLIP
PAR-CLIP (CCA)
All.reads
TtoC.reads
Coverage
Position relative to tRNA 5' end
Position relative to RNaseZ site

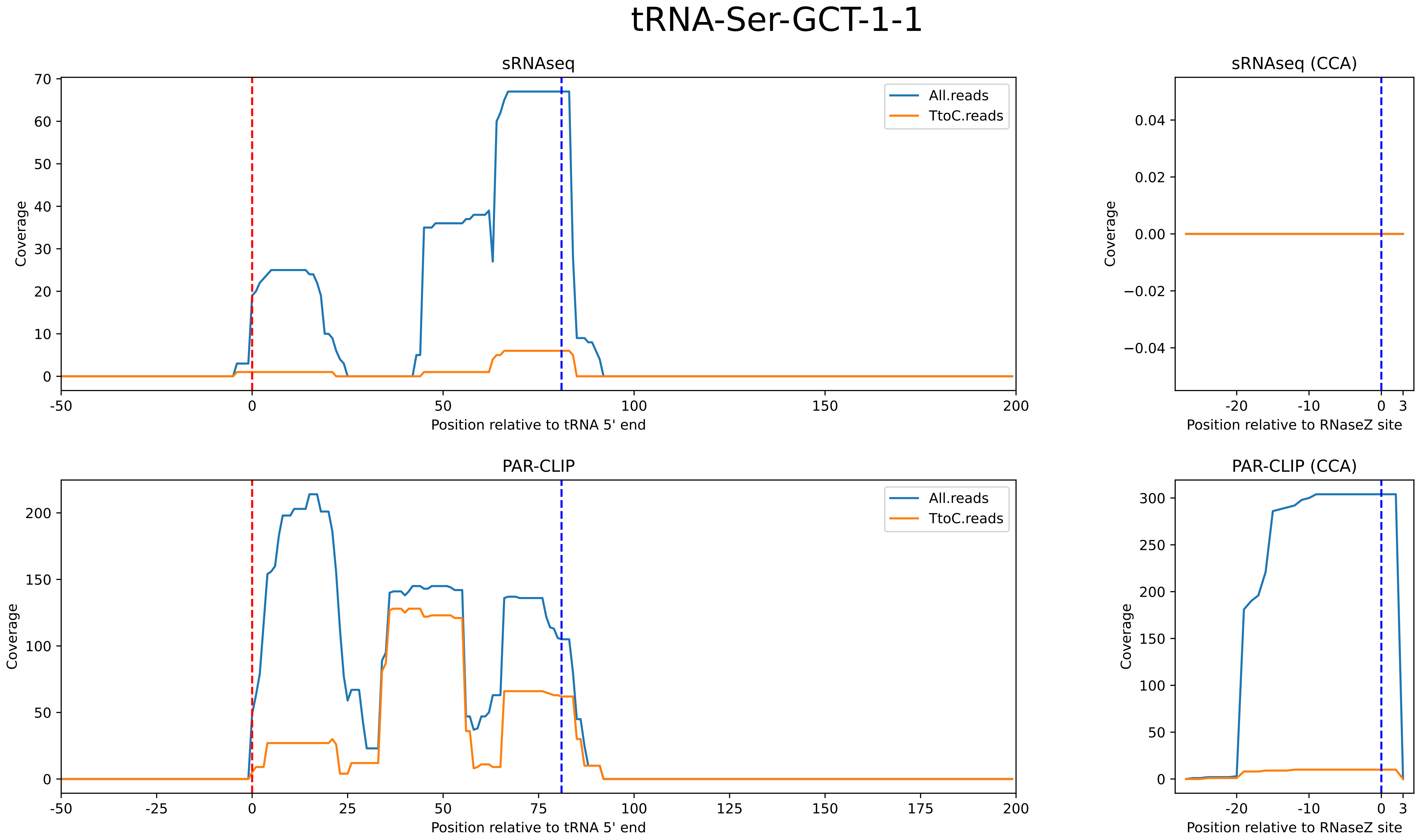
tRNA-Ser-GCT-1-1
sRNAseq
sRNAseq (CCA)
PAR-CLIP
PAR-CLIP (CCA)
All.reads
TtoC.reads
Coverage
Position relative to tRNA 5' end
Position relative to RNaseZ site

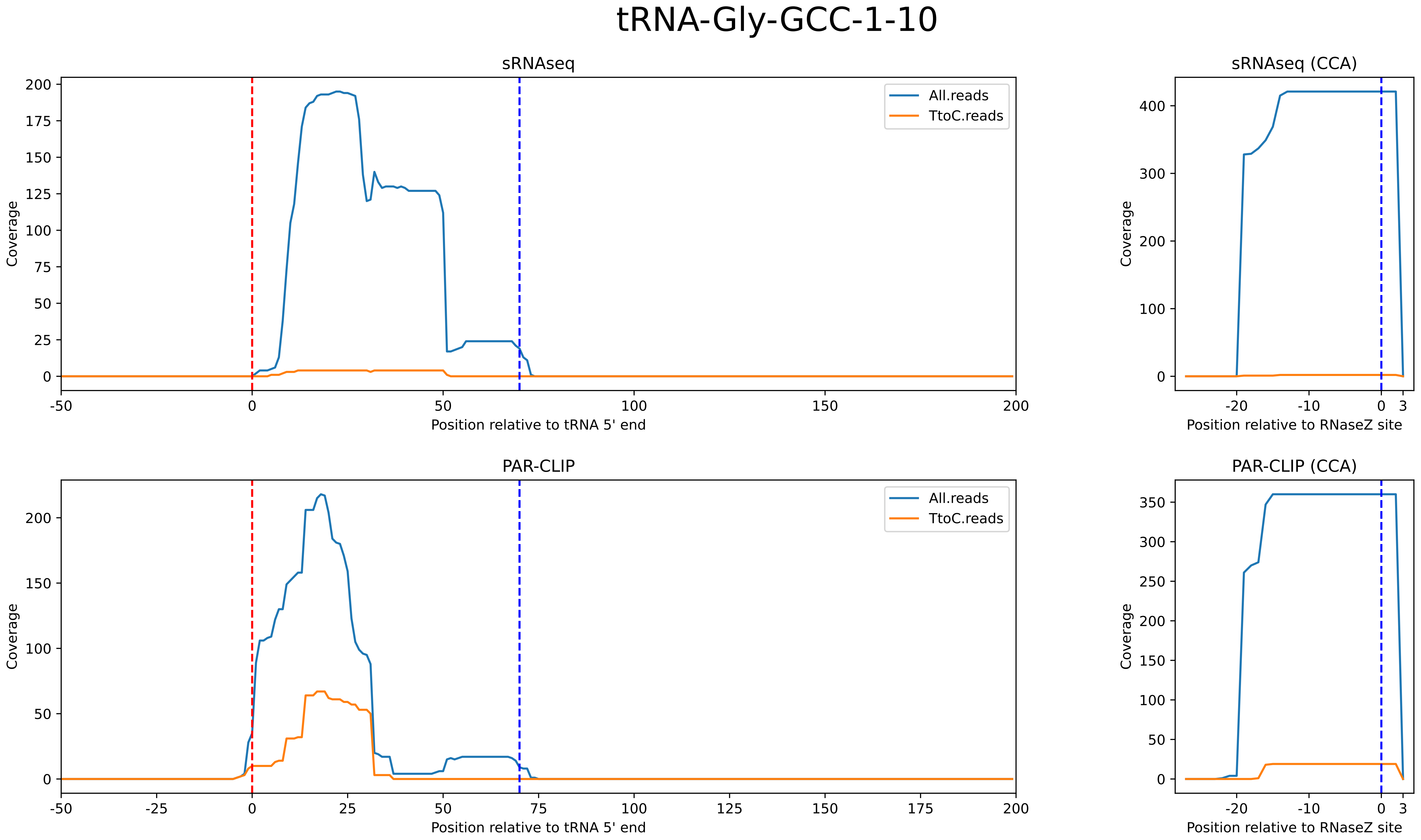

tRNA-Gly-GCC-1-10
sRNAseq
All.reads
TtoC.reads
Coverage
Position relative to tRNA 5' end
sRNAseq (CCA)
Position relative to RNaseZ site
PAR-CLIP
PAR-CLIP (CCA)

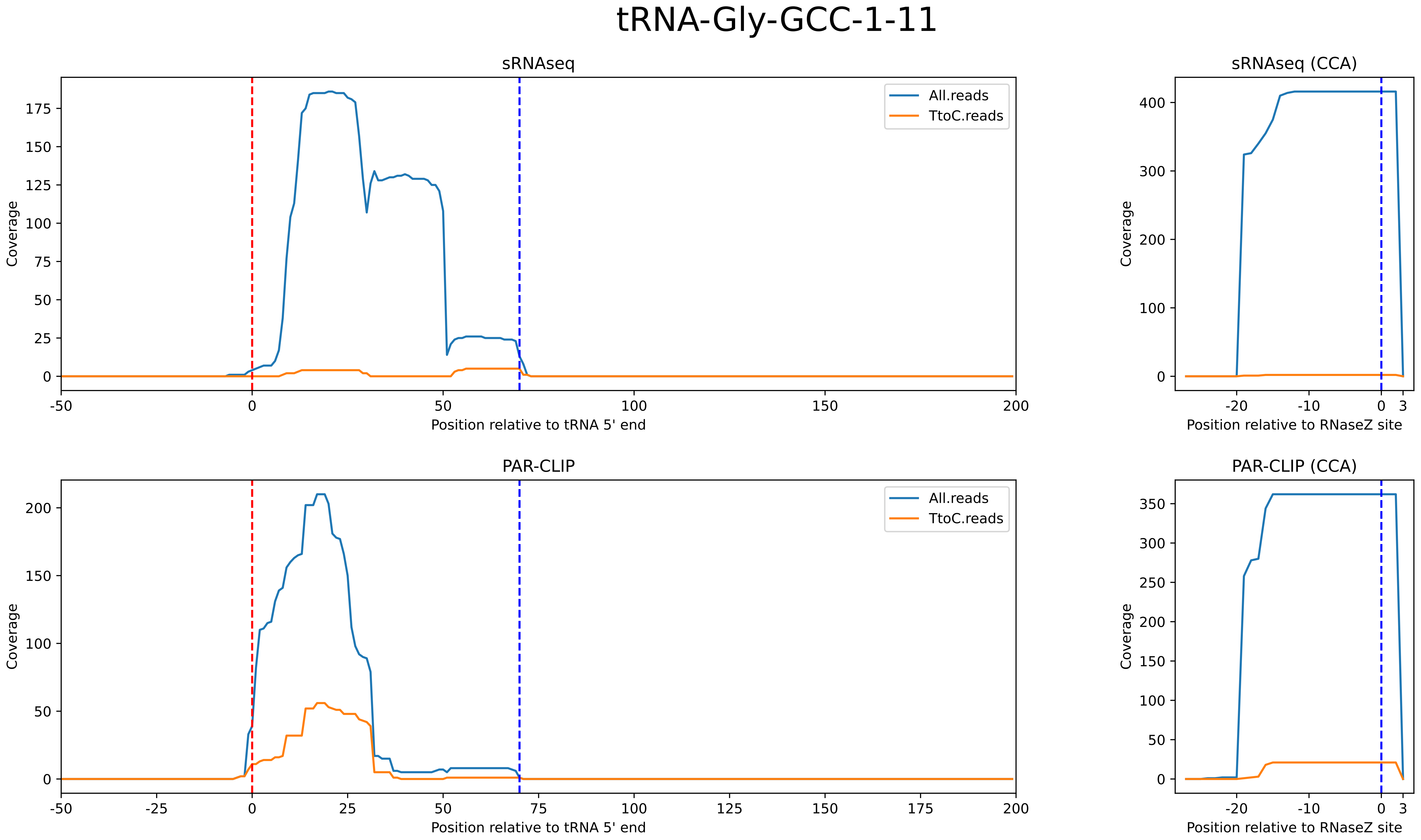

tRNA-Gly-GCC-1-11
sRNAseq
All.reads
TtoC.reads
Coverage
Position relative to tRNA 5' end
sRNAseq (CCA)
Position relative to RNaseZ site
PAR-CLIP
PAR-CLIP (CCA)

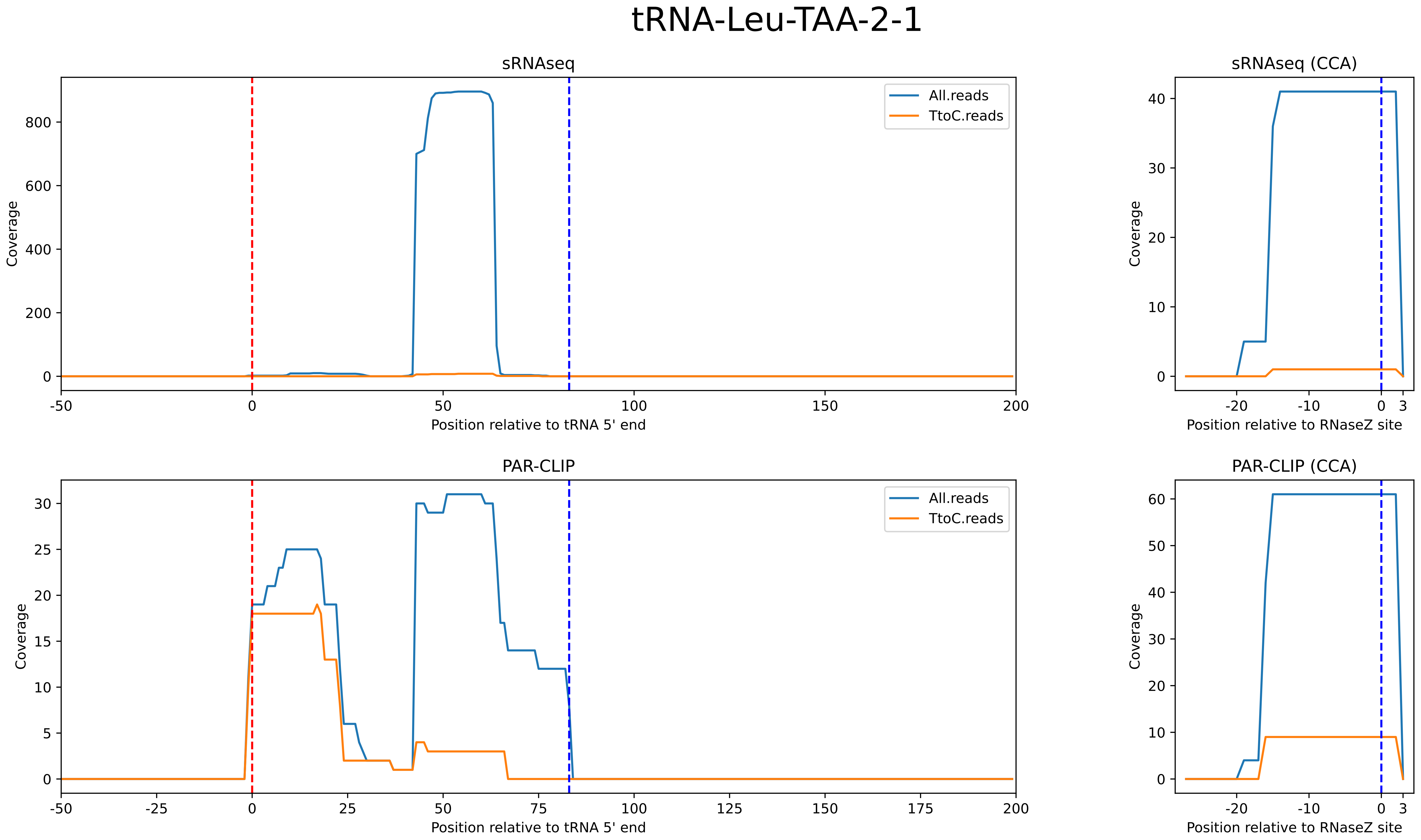

tRNA-Leu-TAA-2-1
sRNAseq
All.reads
TtoC.reads
Coverage
Position relative to tRNA 5' end
sRNAseq (CCA)
Position relative to RNaseZ site
PAR-CLIP
PAR-CLIP (CCA)

# tRNA-Lys-CTT-1-10

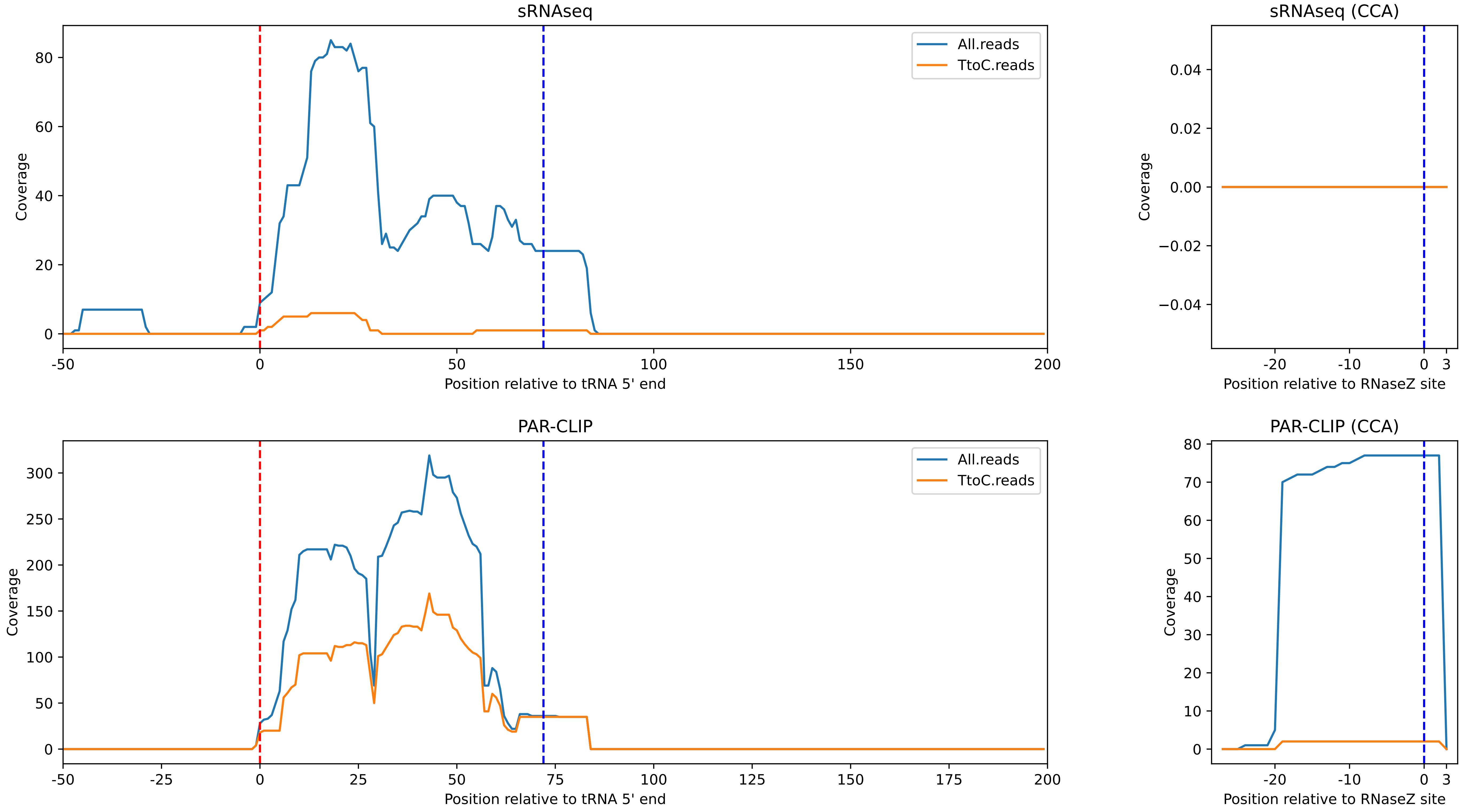

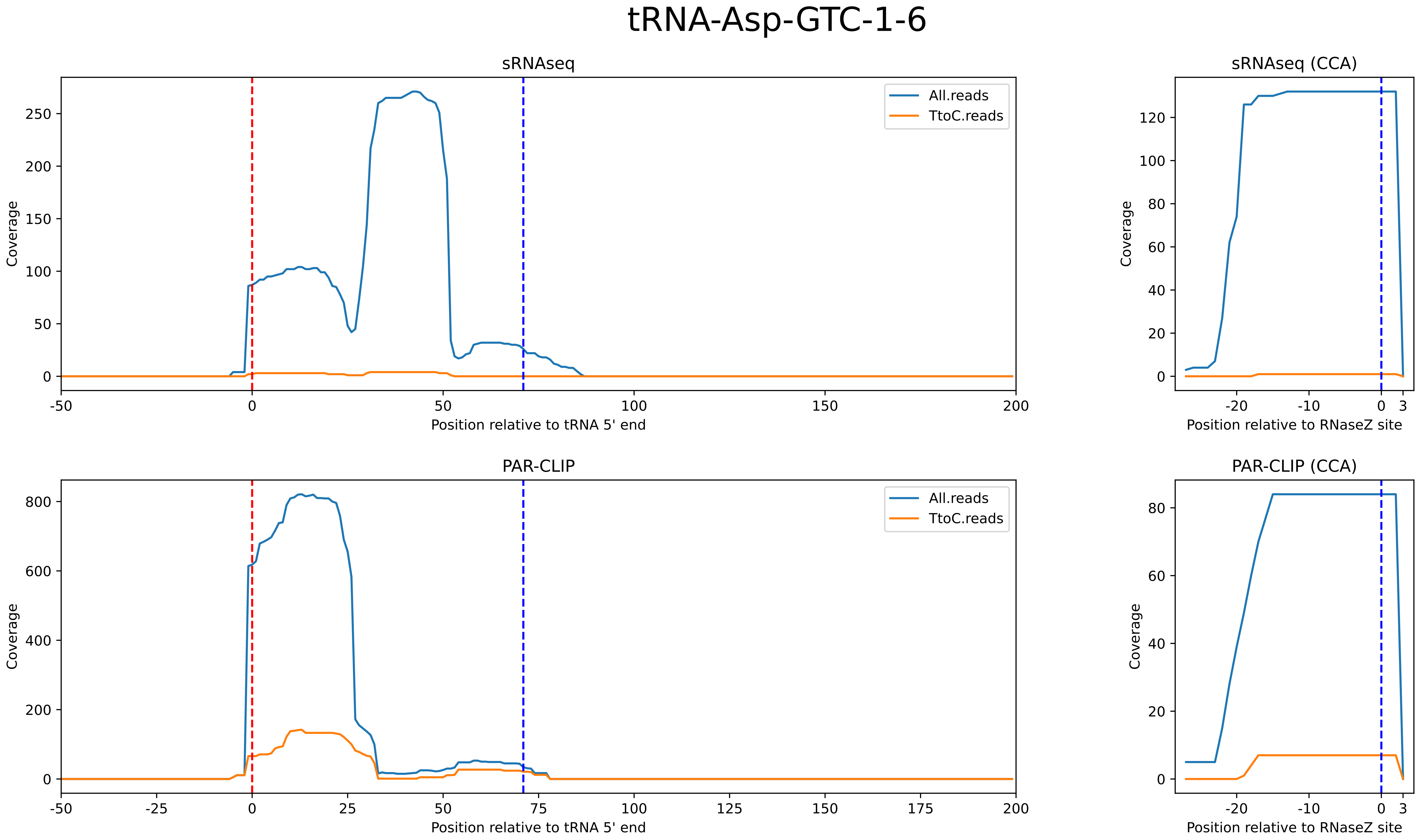

tRNA-Asp-GTC-1-6
sRNAseq
sRNAseq (CCA)
PAR-CLIP
PAR-CLIP (CCA)
All.reads
TtoC.reads
Coverage
Position relative to tRNA 5' end
Position relative to RNaseZ site

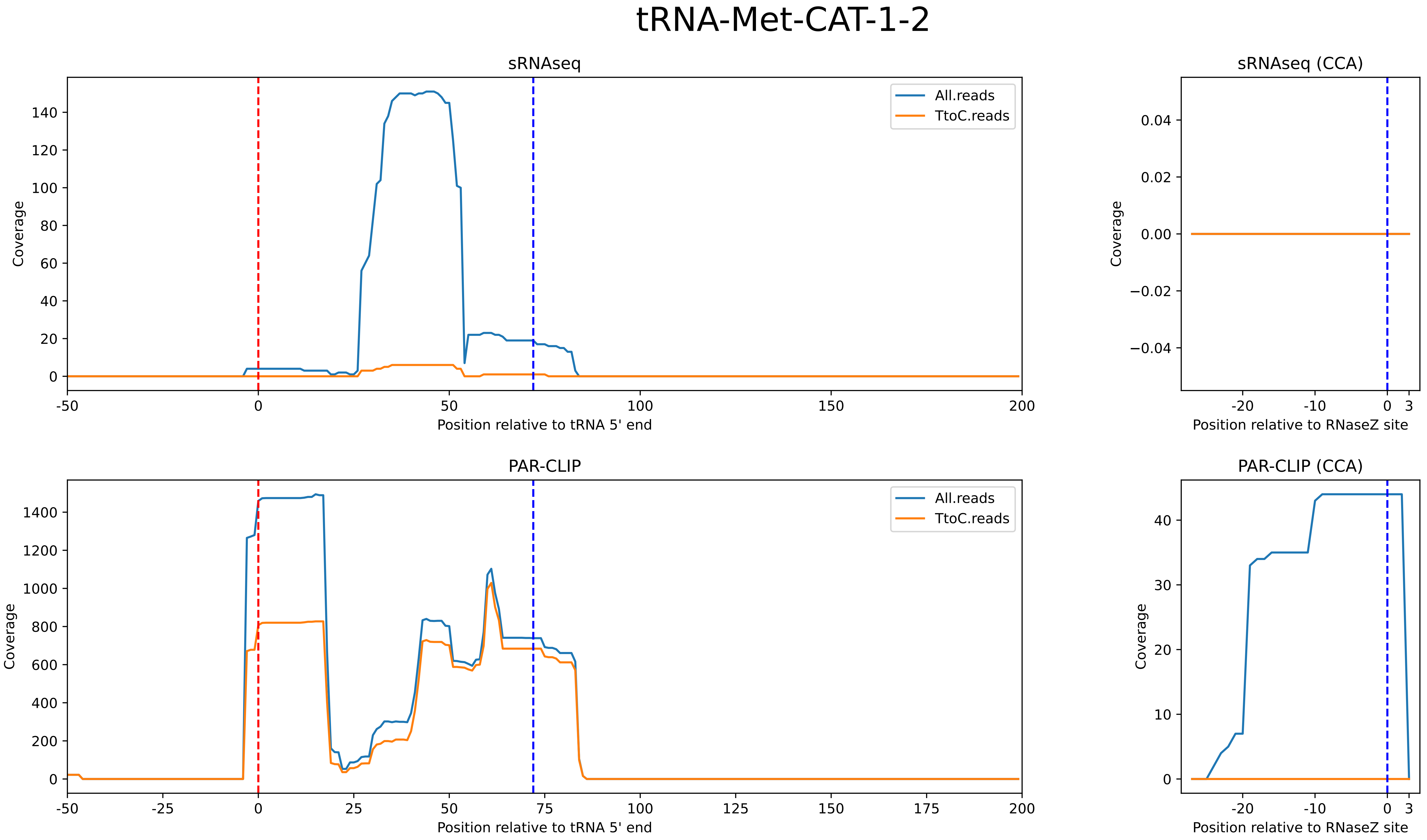
tRNA-Met-CAT-1-2
sRNAseq
All.reads
TtoC.reads
Coverage
Position relative to tRNA 5' end
sRNAseq (CCA)
Position relative to RNaseZ site
PAR-CLIP
PAR-CLIP (CCA)

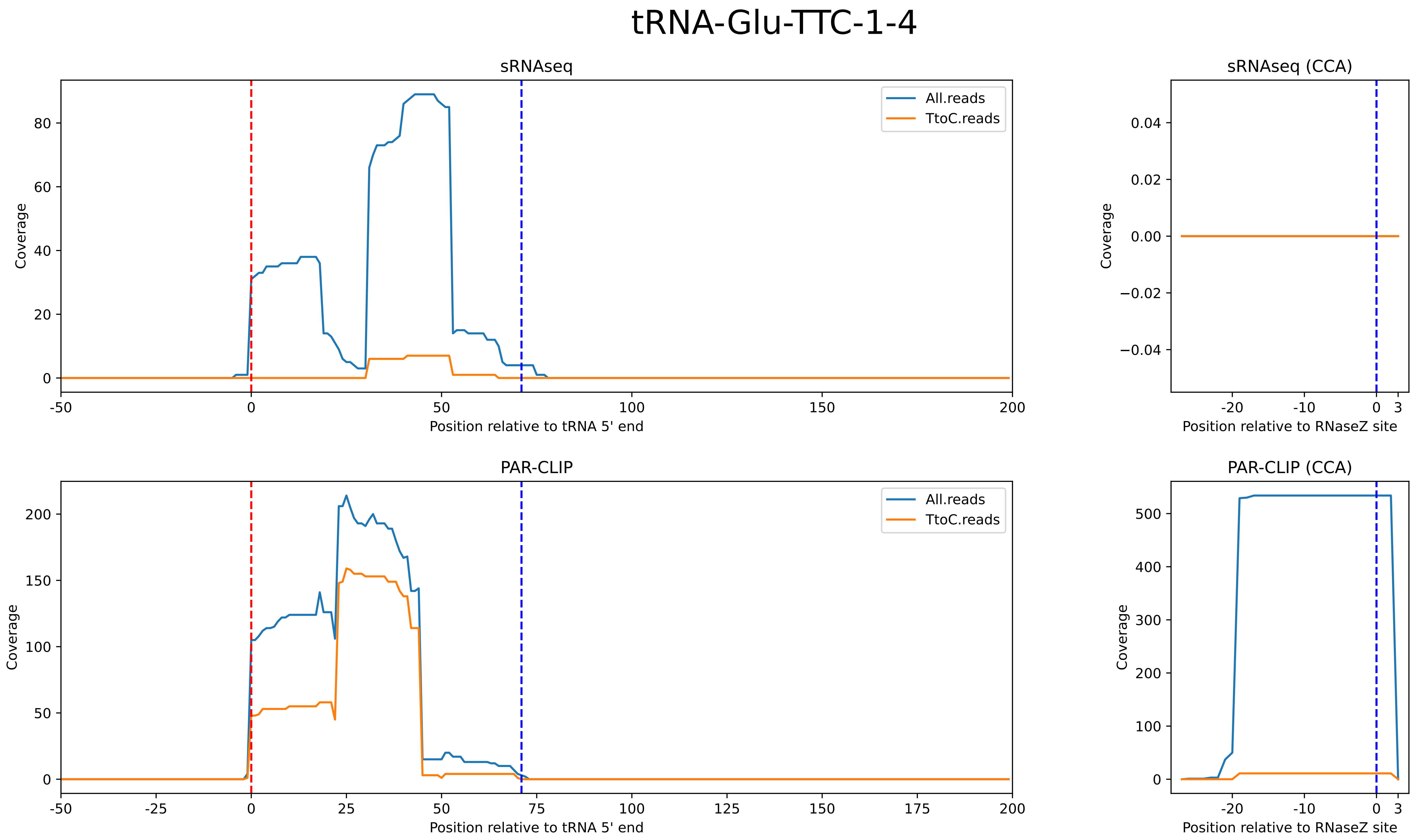
tRNA-Glu-TTC-1-4
sRNAseq
All.reads
TtoC.reads
Coverage
Position relative to tRNA 5' end
sRNAseq (CCA)
Position relative to RNaseZ site
PAR-CLIP
PAR-CLIP (CCA)

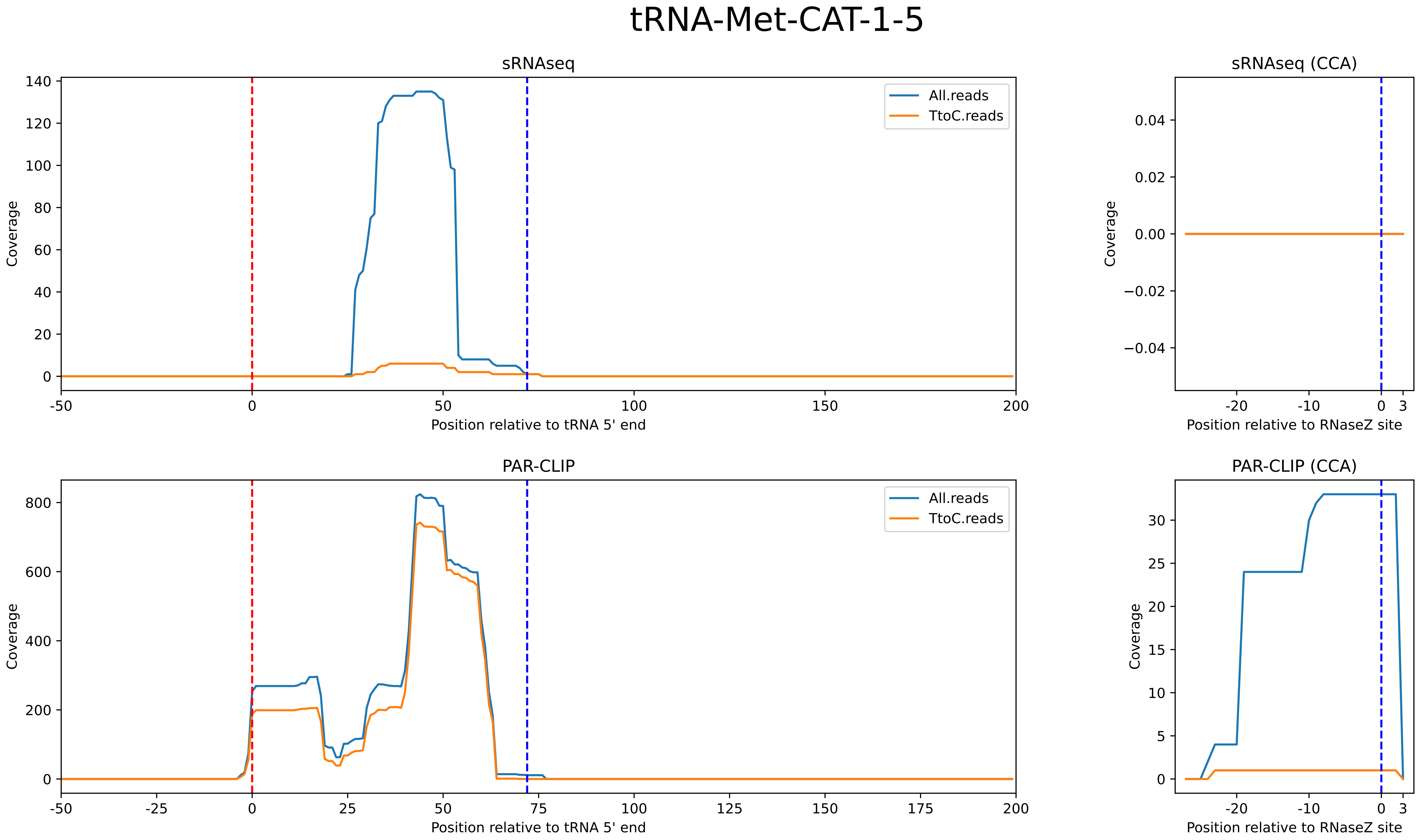

tRNA-Met-CAT-1-5
sRNAseq
sRNAseq (CCA)
PAR-CLIP
PAR-CLIP (CCA)
All.reads
TtoC.reads
Coverage
Position relative to tRNA 5' end
Position relative to RNaseZ site

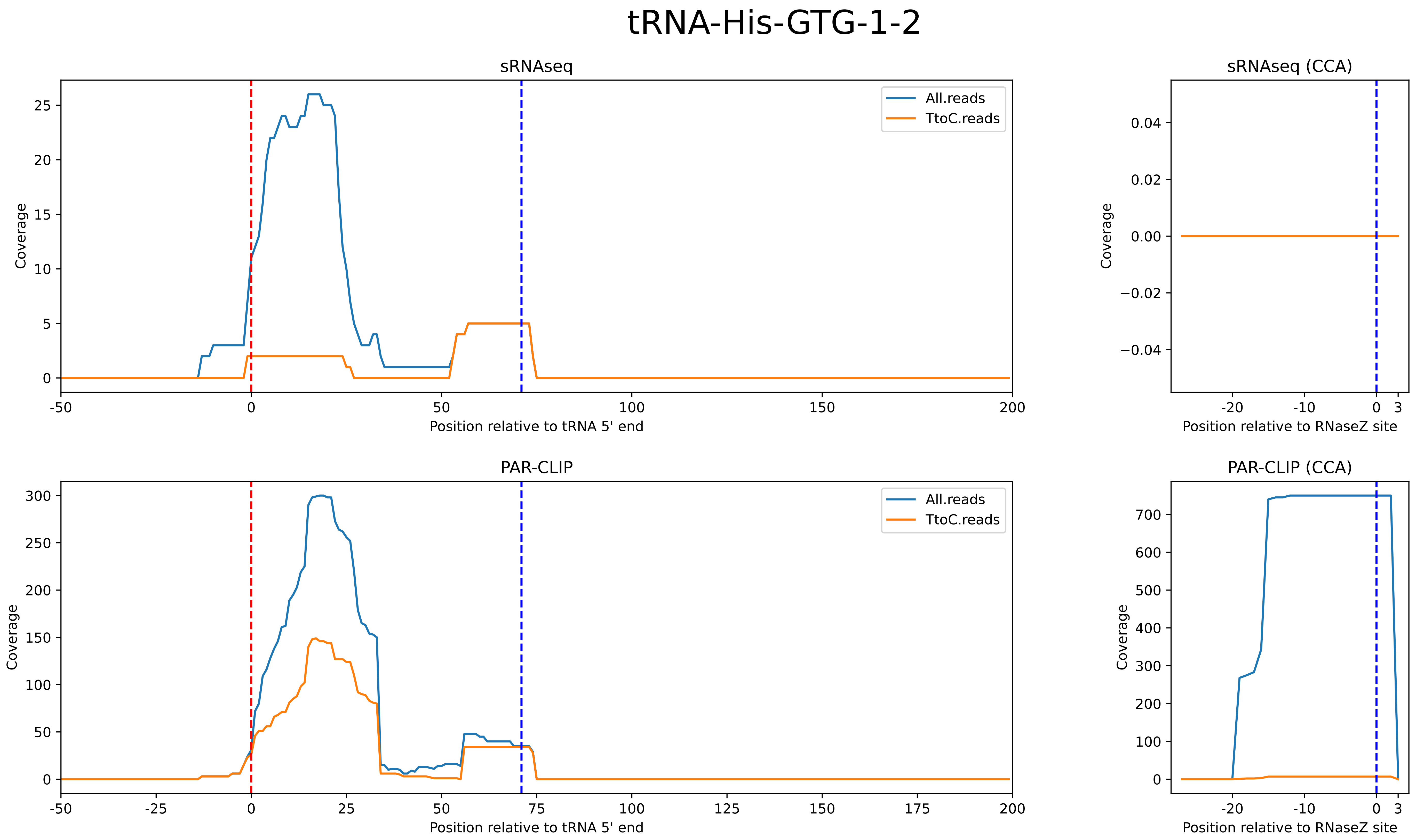
tRNA-His-GTG-1-2
sRNAseq
All.reads
TtoC.reads
Coverage
Position relative to tRNA 5' end
sRNAseq (CCA)
Position relative to RNaseZ site
PAR-CLIP
PAR-CLIP (CCA)

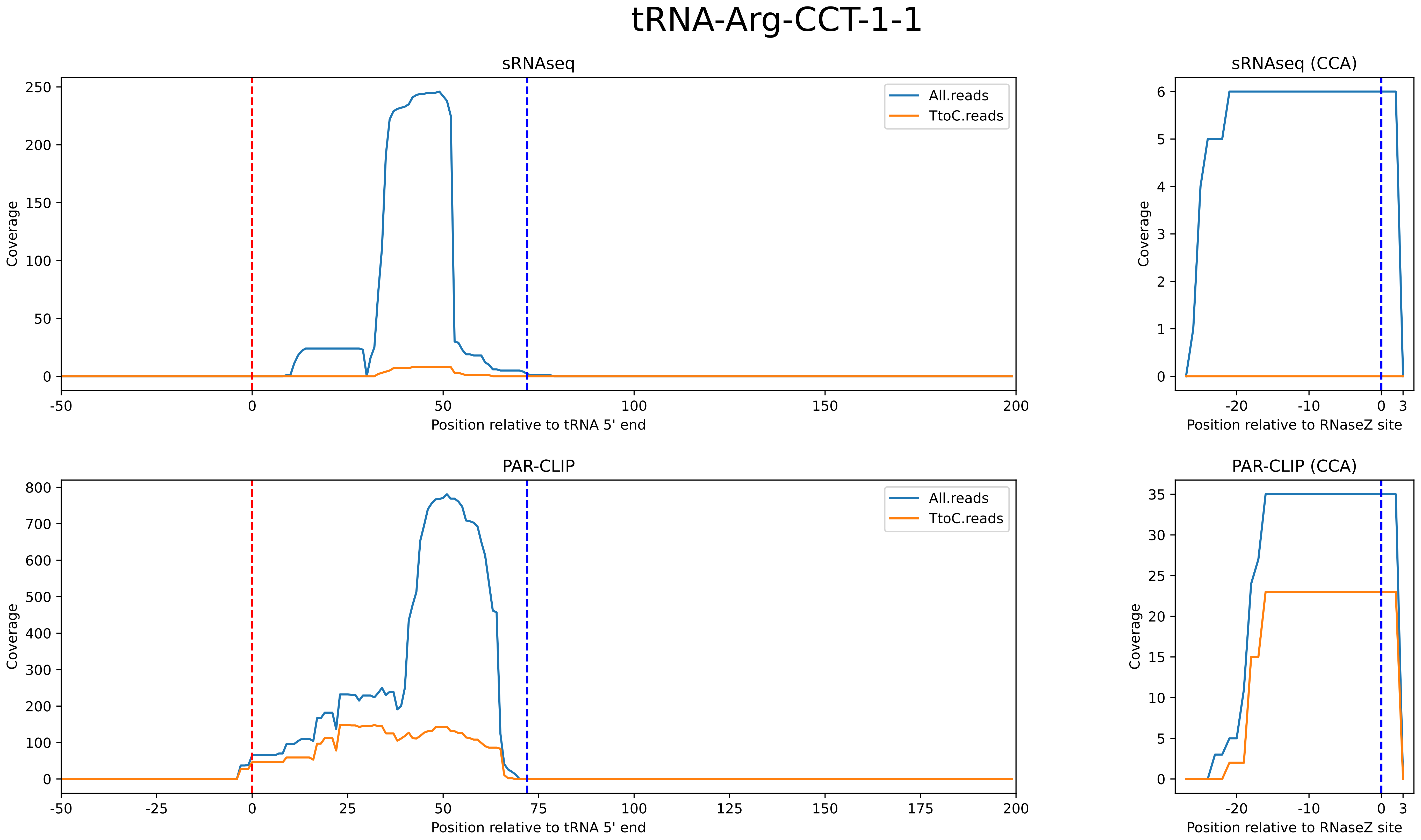
tRNA-Arg-CCT-1-1
sRNAseq
sRNAseq (CCA)
PAR-CLIP
PAR-CLIP (CCA)
All.reads
TtoC.reads
Coverage
Position relative to tRNA 5' end
Position relative to RNaseZ site

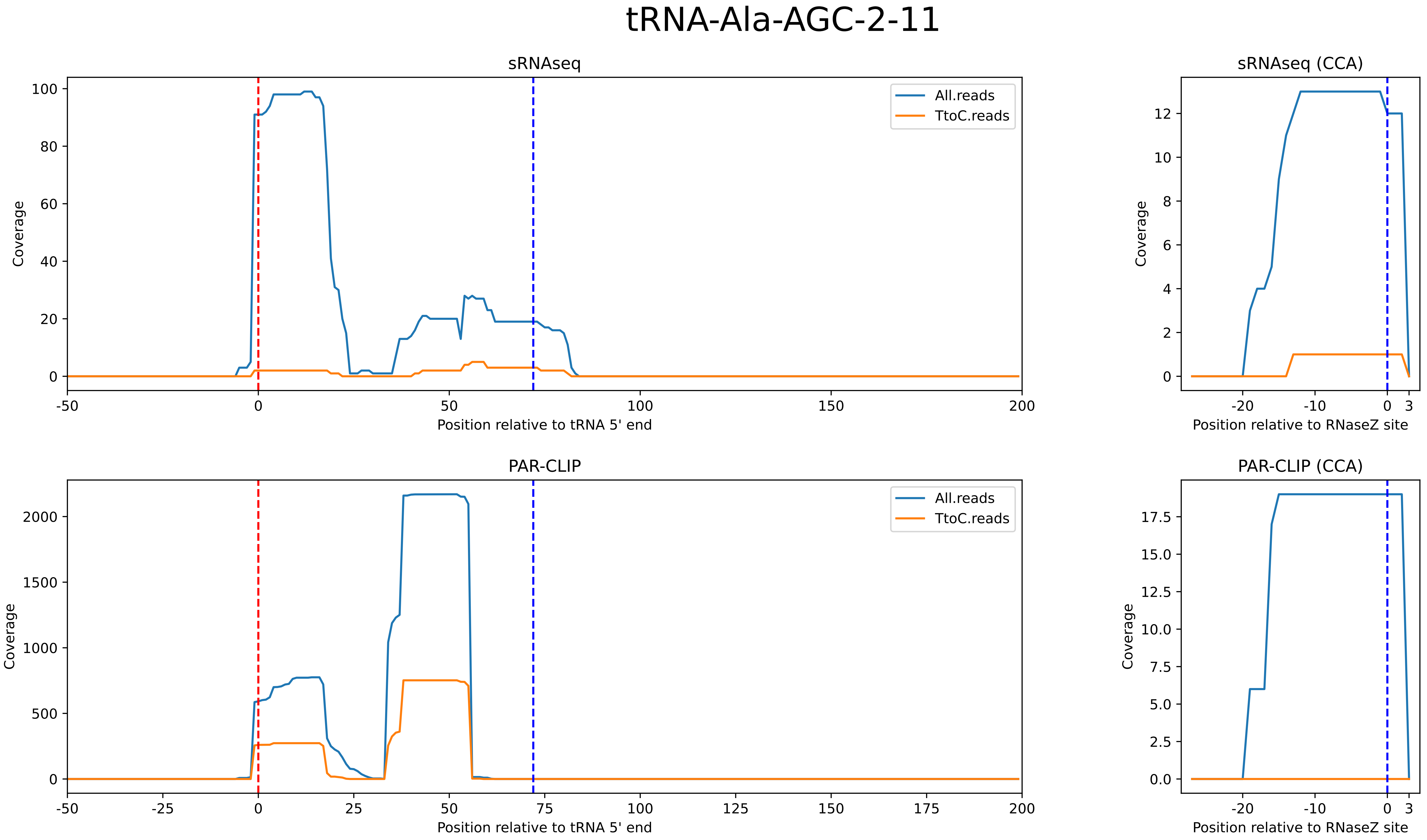

tRNA-Ala-AGC-2-11
sRNAseq
All.reads
TtoC.reads
Coverage
Position relative to tRNA 5' end
sRNAseq (CCA)
Position relative to RNaseZ site
PAR-CLIP
PAR-CLIP (CCA)

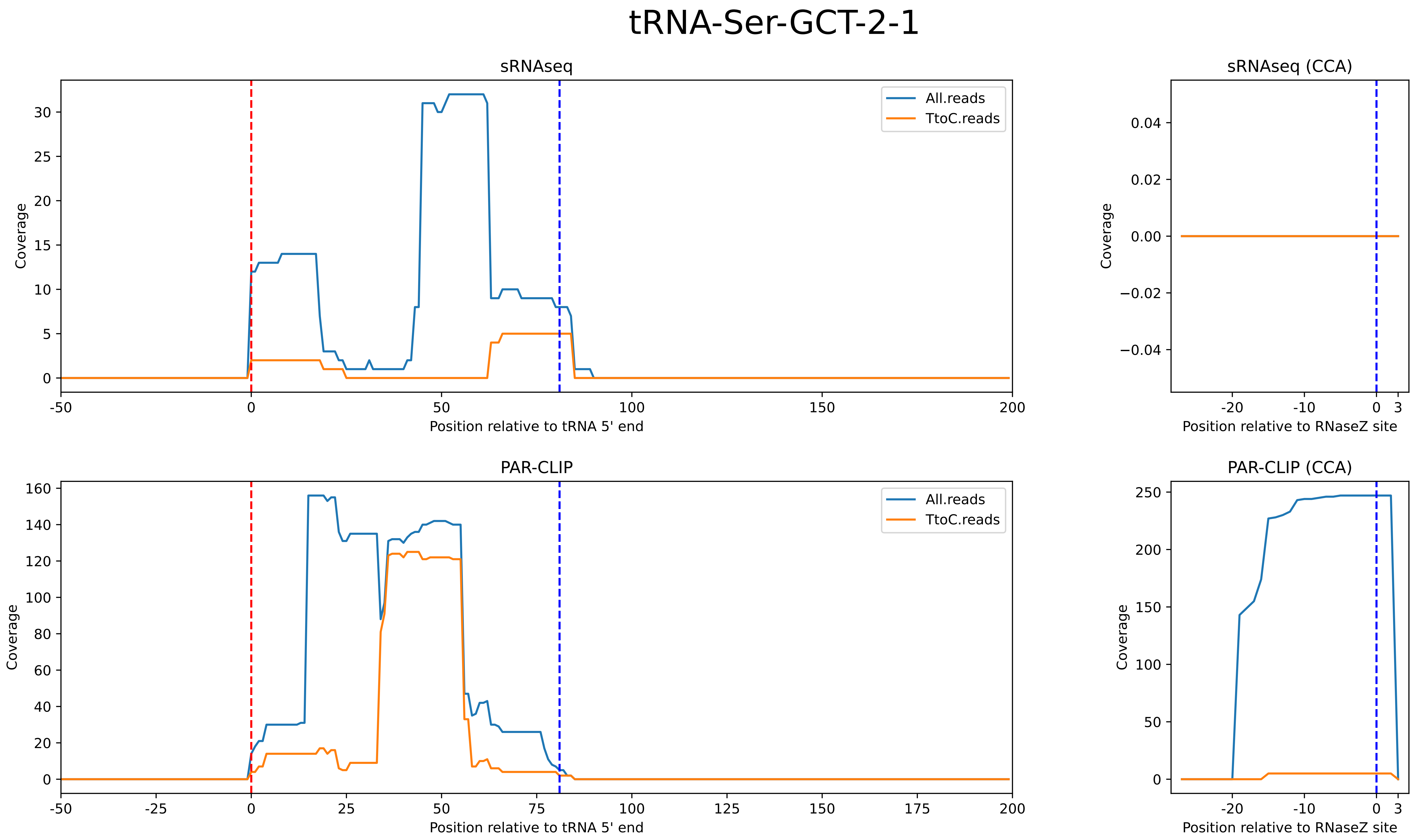
tRNA-Ser-GCT-2-1
sRNAseq
All.reads
TtoC.reads
Coverage
Position relative to tRNA 5' end
sRNAseq (CCA)
Position relative to RNaseZ site
PAR-CLIP
PAR-CLIP (CCA)

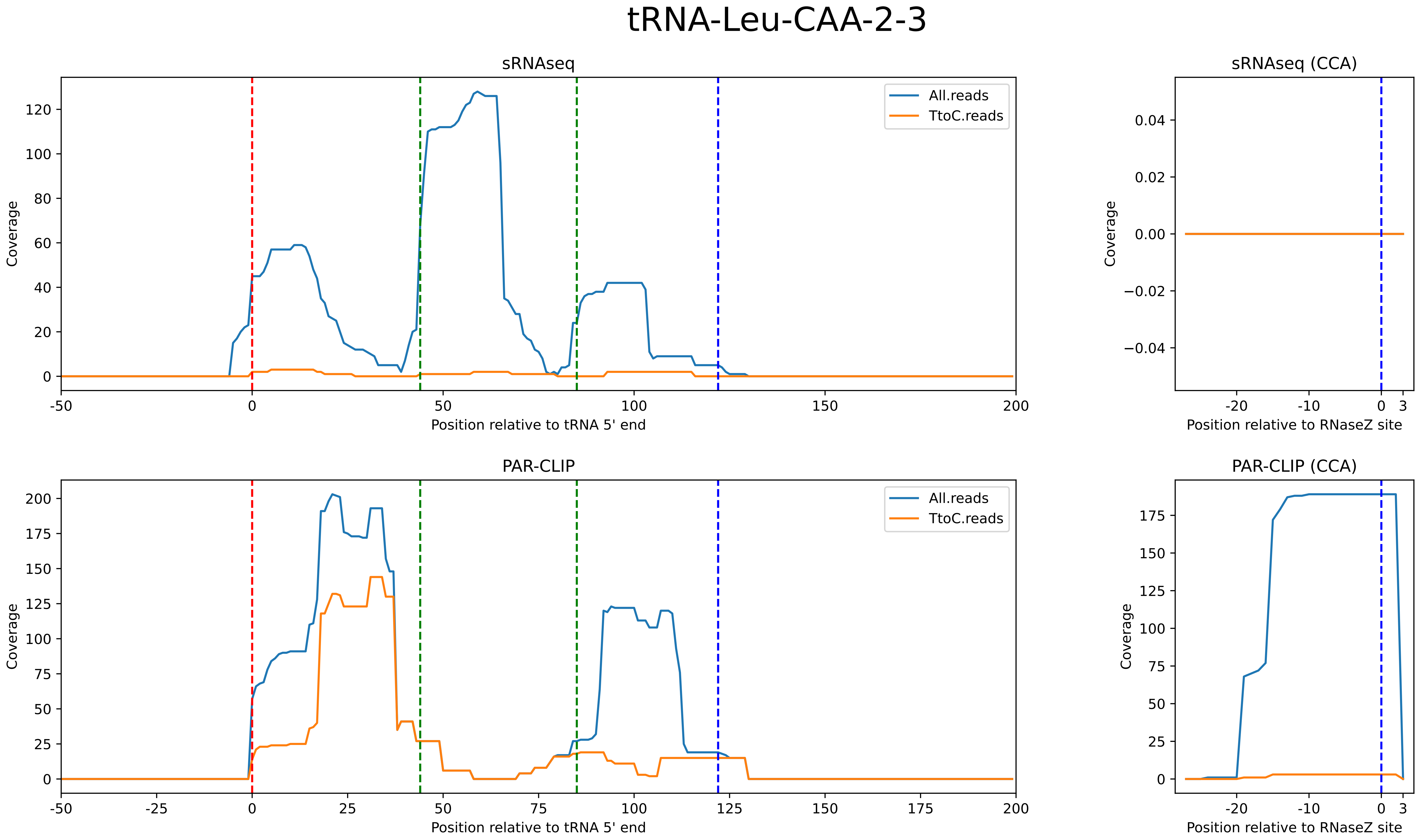

tRNA-Leu-CAA-2-3
sRNAseq
All.reads
TtoC.reads
Coverage
Position relative to tRNA 5' end
sRNAseq (CCA)
Position relative to RNaseZ site
PAR-CLIP
PAR-CLIP (CCA)

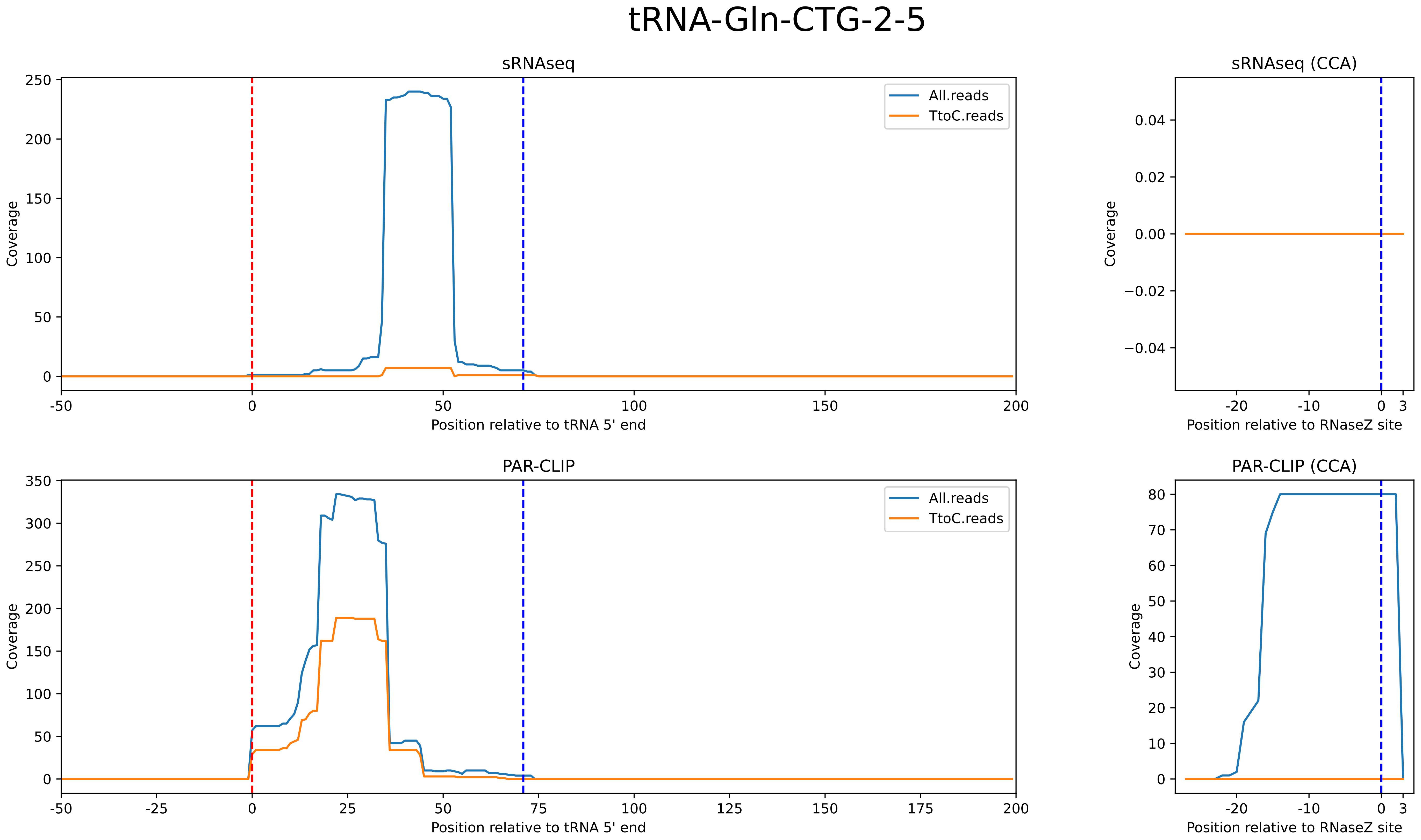
tRNA-Gln-CTG-2-5
sRNAseq
sRNAseq (CCA)
PAR-CLIP
PAR-CLIP (CCA)
All.reads
TtoC.reads
Coverage
Position relative to tRNA 5' end
Position relative to RNaseZ site

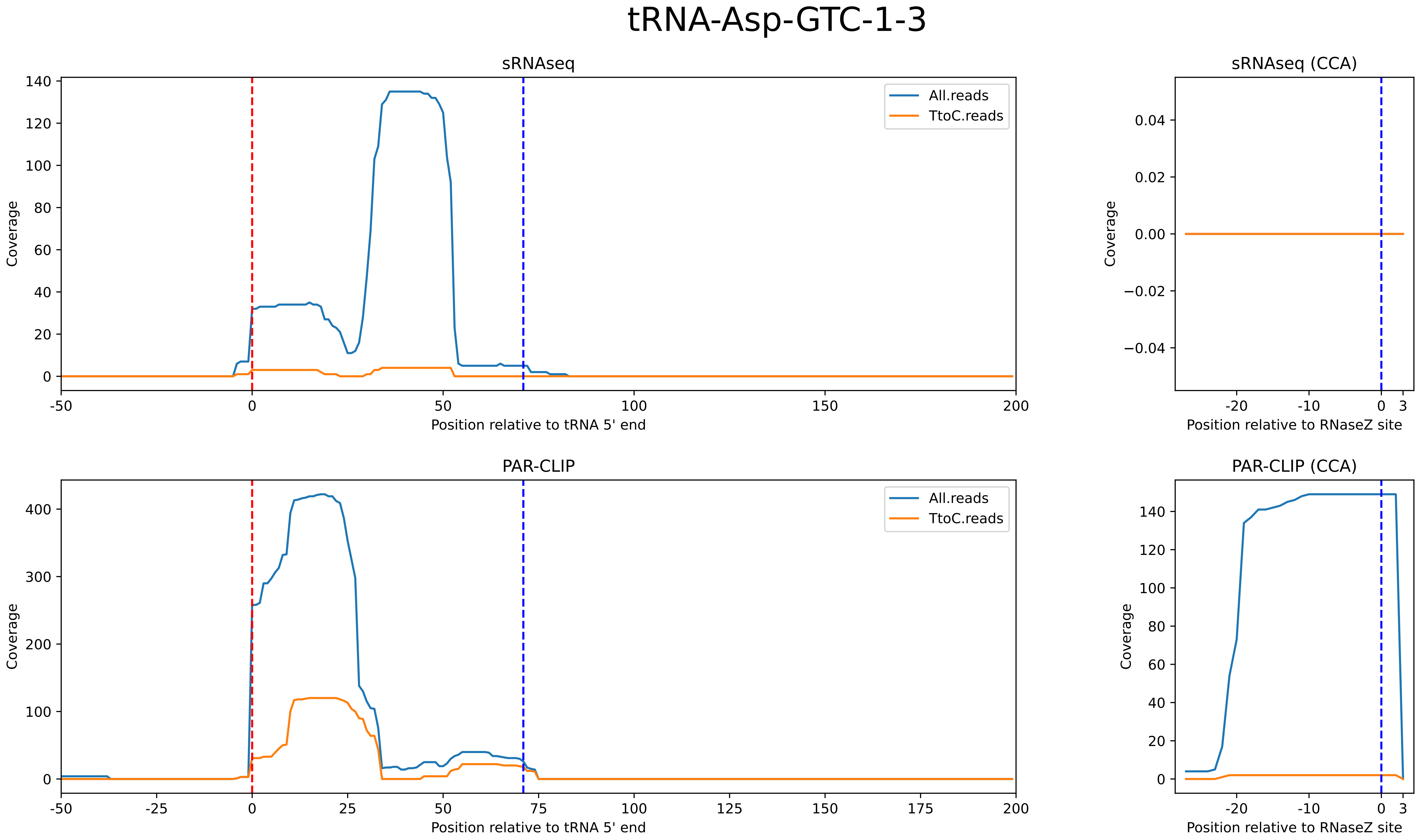
tRNA-Asp-GTC-1-3
sRNAseq
All.reads
TtoC.reads
Coverage
Position relative to tRNA 5' end
sRNAseq (CCA)
Position relative to RNaseZ site
PAR-CLIP
PAR-CLIP (CCA)

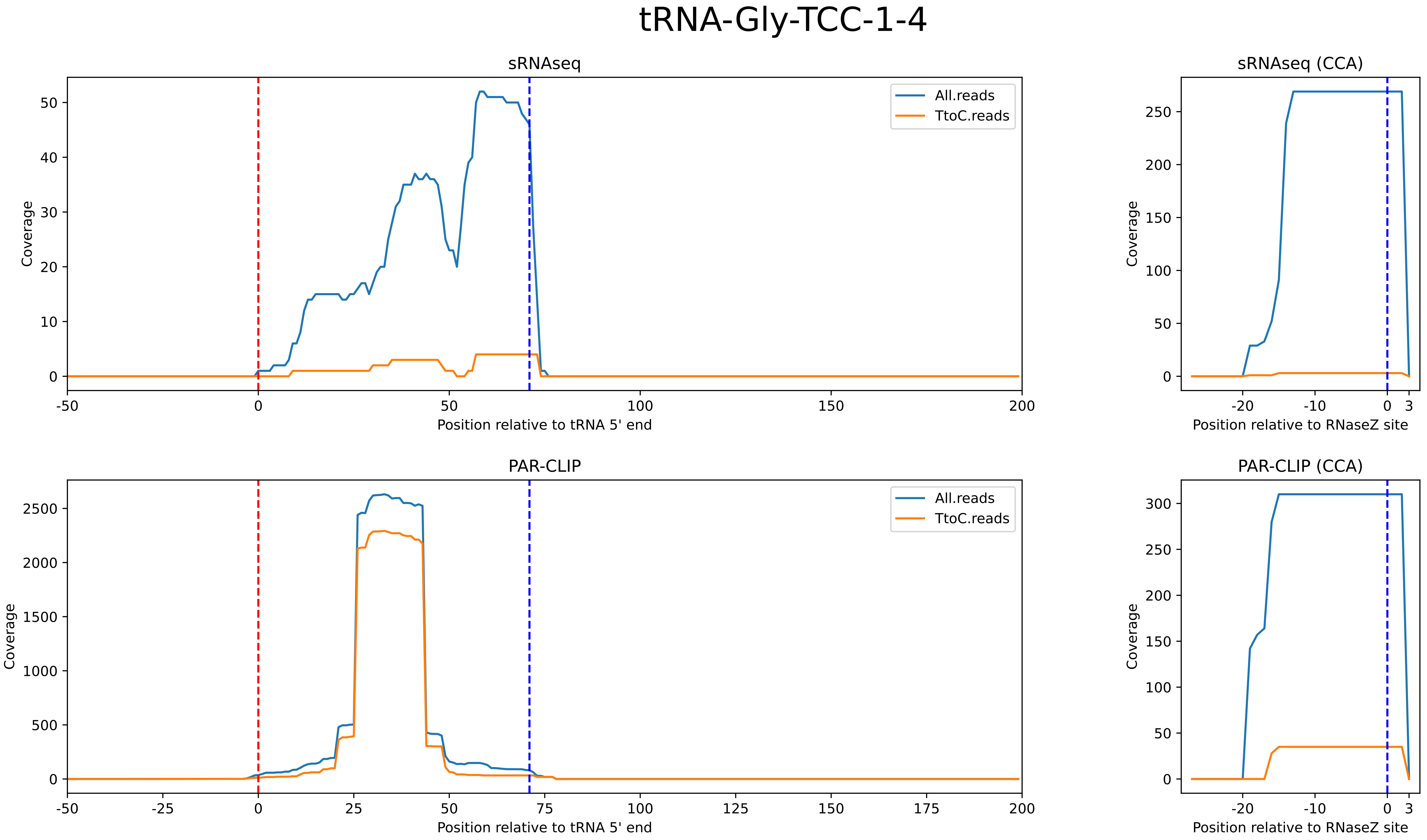
tRNA-Gly-TCC-1-4
sRNAseq
All.reads
TtoC.reads
Coverage
Position relative to tRNA 5' end
sRNAseq (CCA)
Position relative to RNaseZ site
PAR-CLIP
PAR-CLIP (CCA)

# tRNA-Leu-TAA-3-1

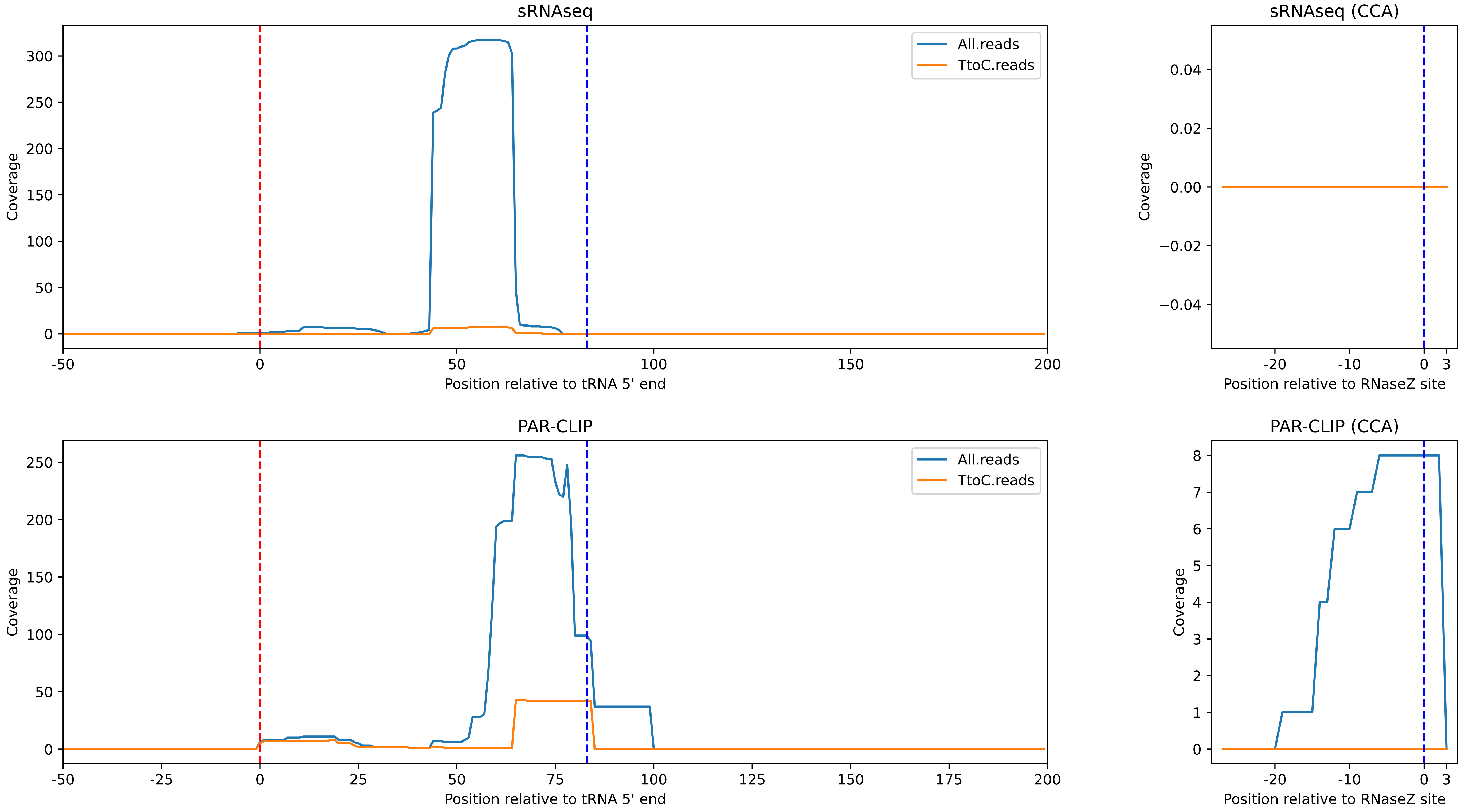

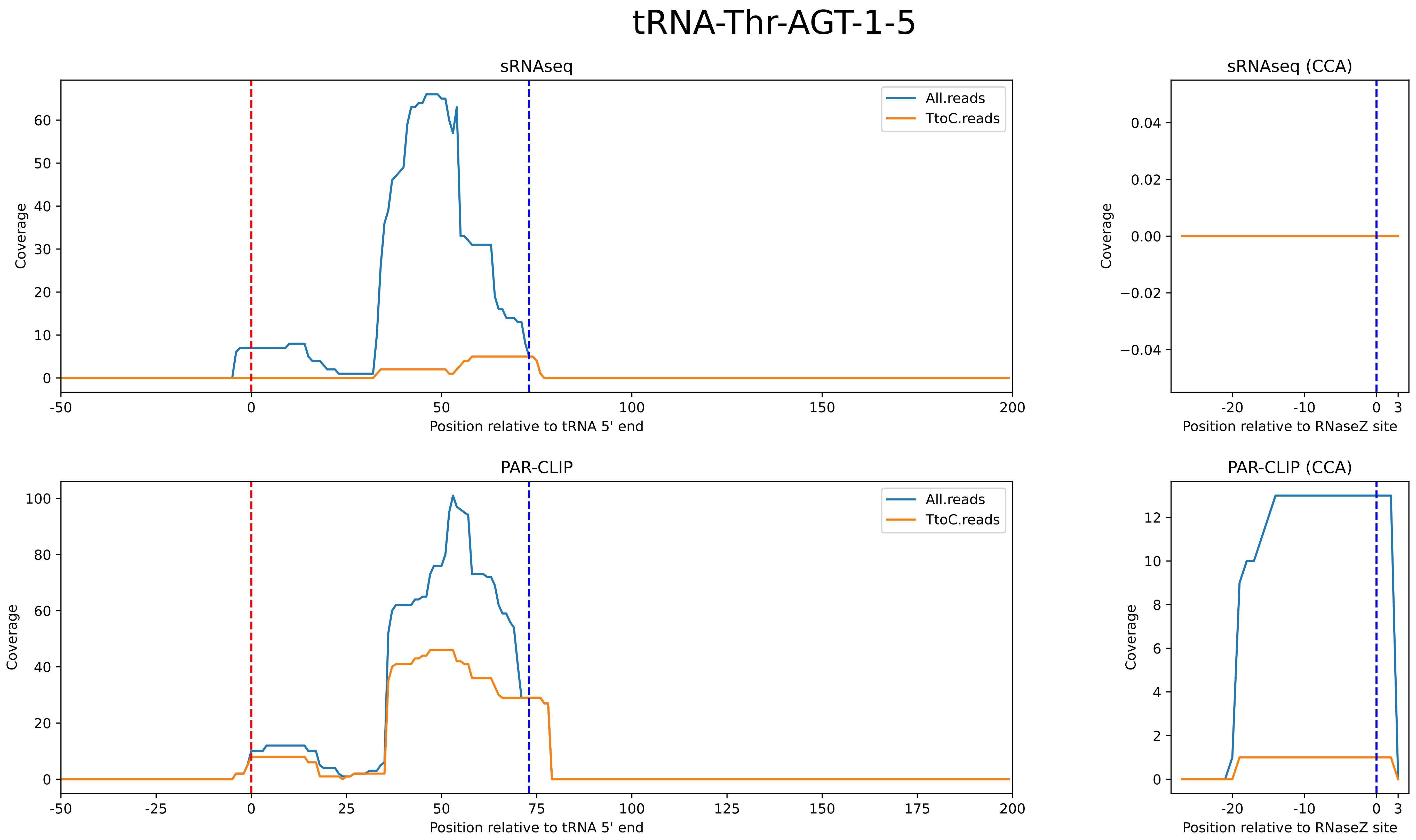

tRNA-Thr-AGT-1-5
sRNAseq
All.reads
TtoC.reads
Coverage
Position relative to tRNA 5' end
sRNAseq (CCA)
Position relative to RNaseZ site
PAR-CLIP
PAR-CLIP (CCA)

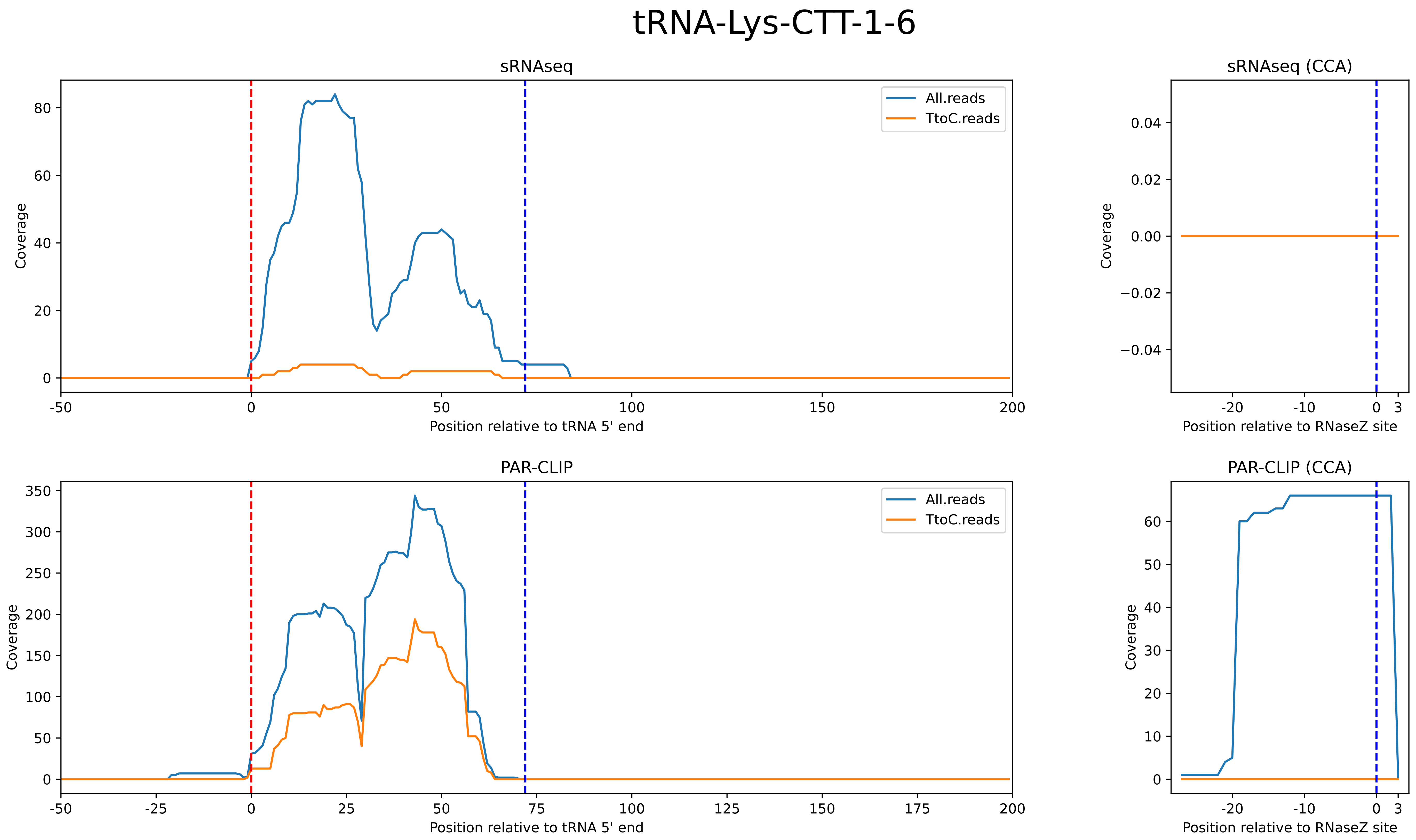
tRNA-Lys-CTT-1-6
sRNAseq
sRNAseq (CCA)
PAR-CLIP
PAR-CLIP (CCA)
All.reads
TtoC.reads
Coverage
Position relative to tRNA 5' end
Position relative to RNaseZ site

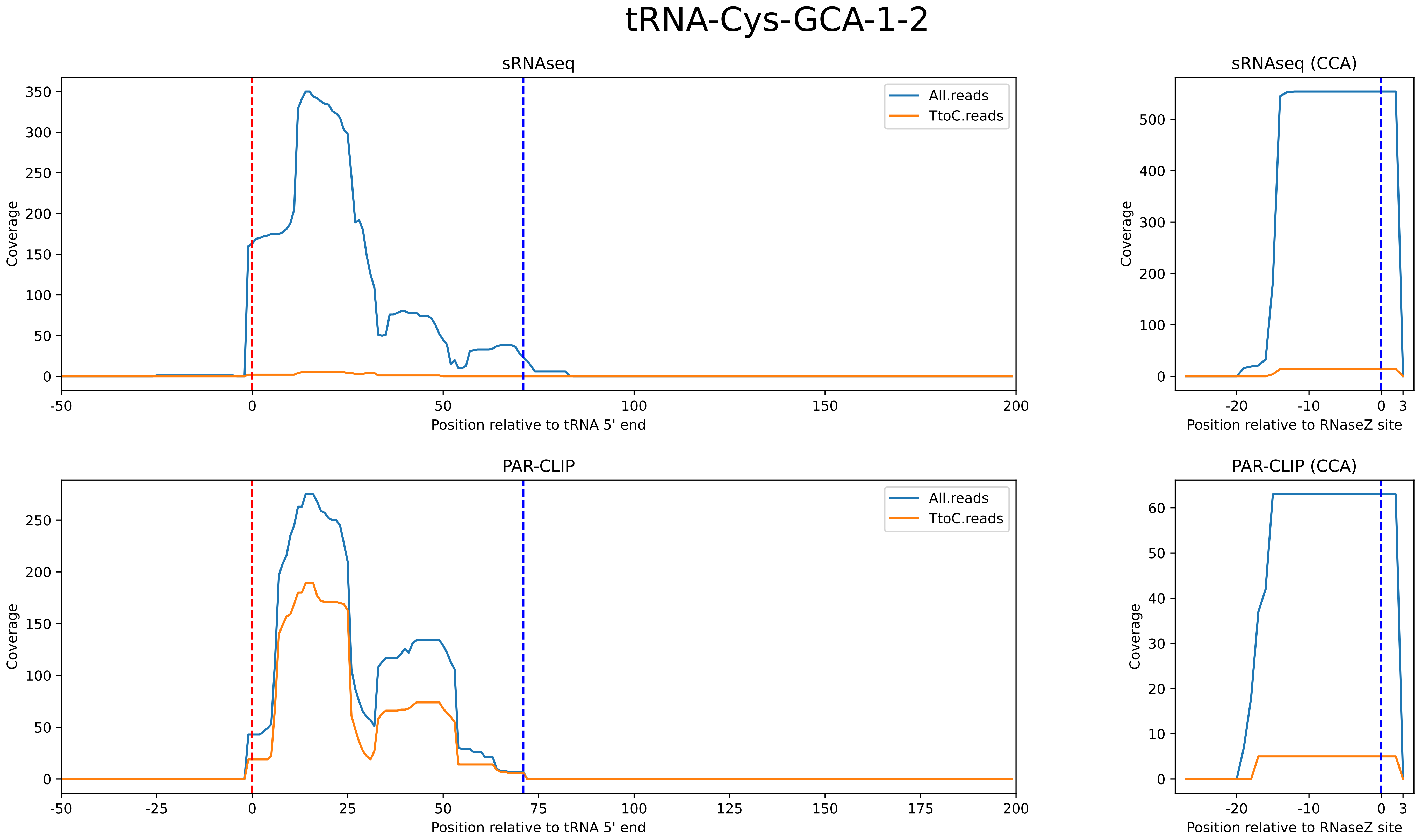

tRNA-Cys-GCA-1-2
sRNAseq
All.reads
TtoC.reads
Coverage
Position relative to tRNA 5' end
sRNAseq (CCA)
Position relative to RNaseZ site
PAR-CLIP
PAR-CLIP (CCA)

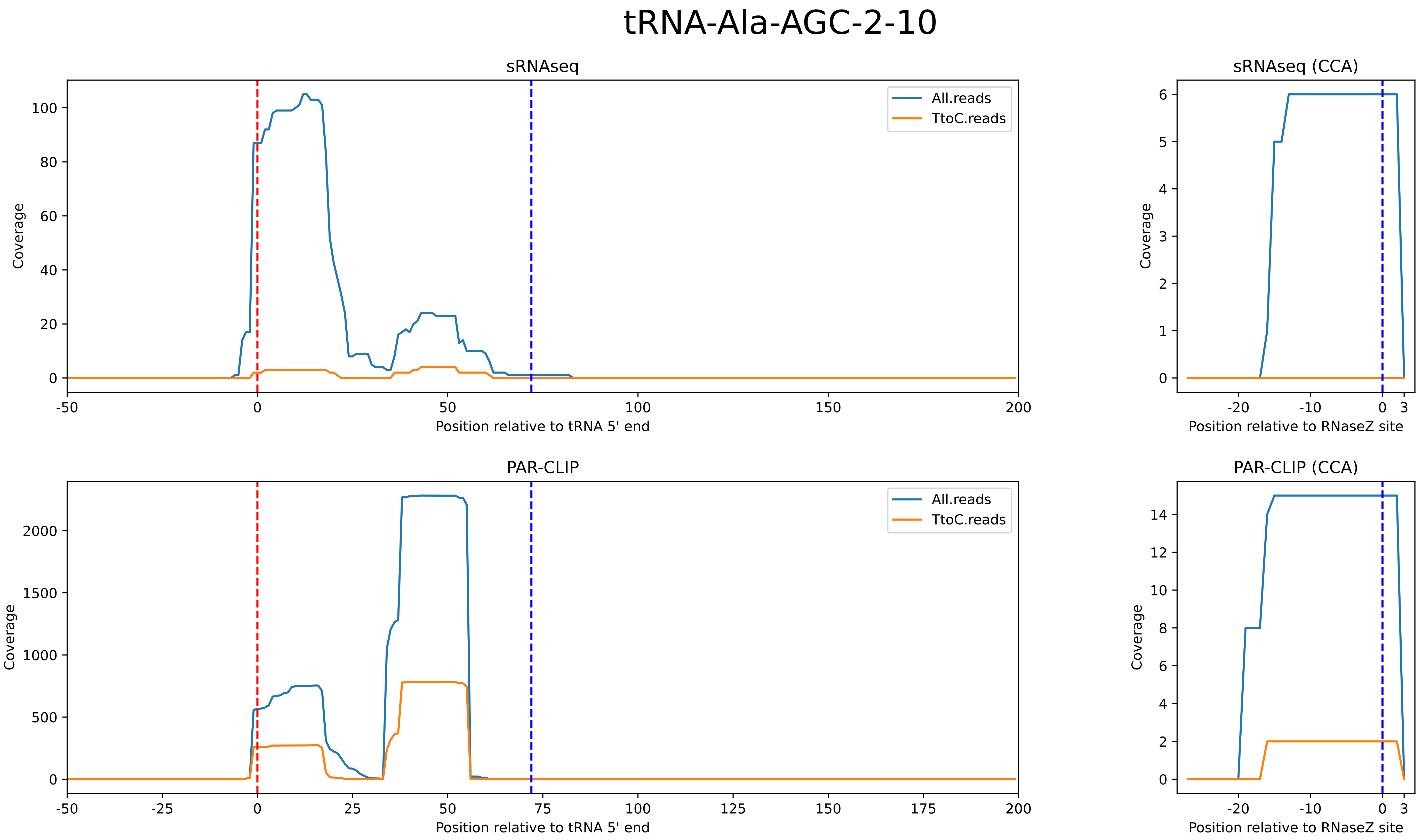

tRNA-Ala-AGC-2-10
sRNAseq
All.reads
TtoC.reads
Coverage
Position relative to tRNA 5' end
sRNAseq (CCA)
Position relative to RNaseZ site
PAR-CLIP
PAR-CLIP (CCA)

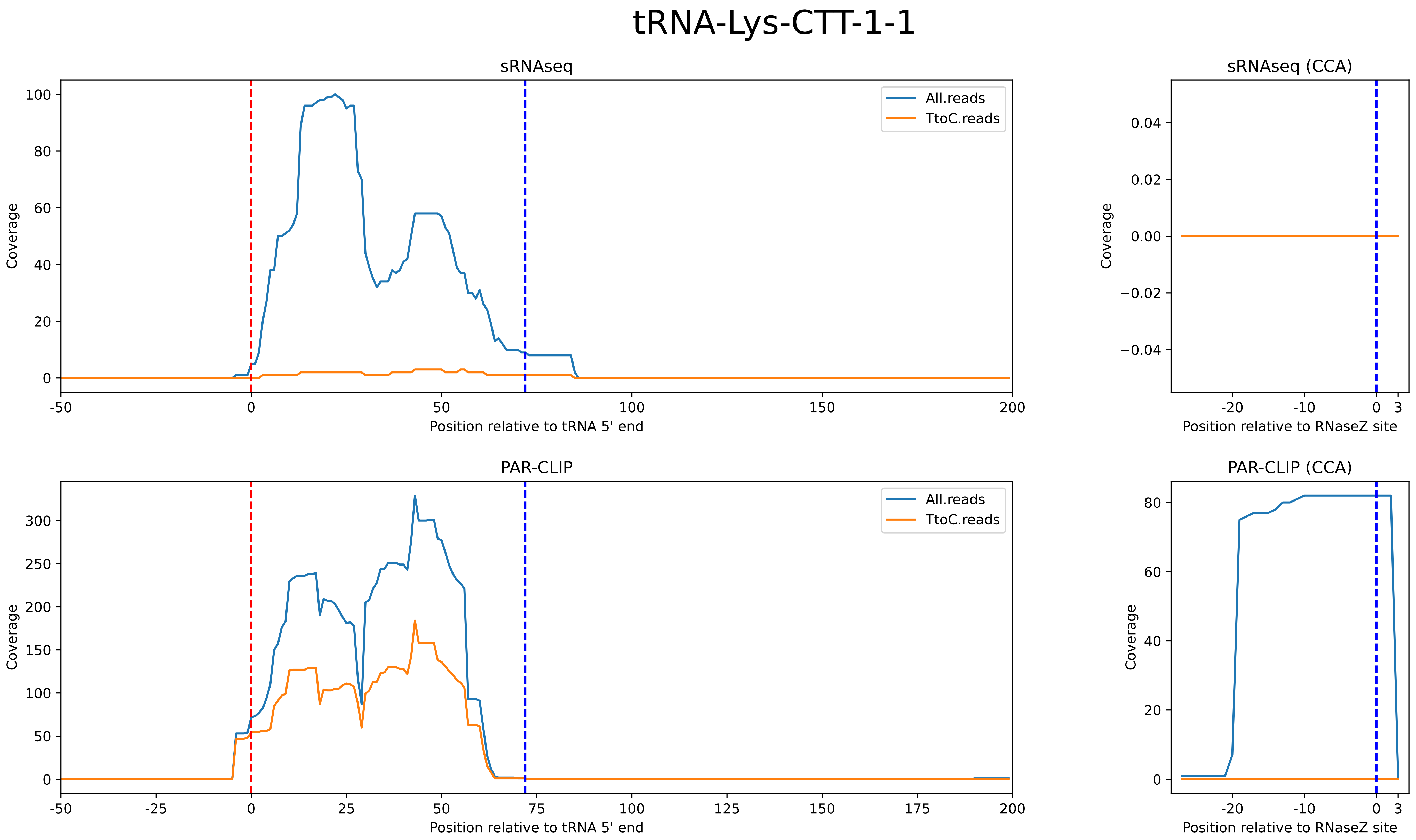
tRNA-Lys-CTT-1-1
sRNAseq
sRNAseq (CCA)
PAR-CLIP
PAR-CLIP (CCA)
All.reads
TtoC.reads
Coverage
Position relative to tRNA 5' end
Position relative to RNaseZ site

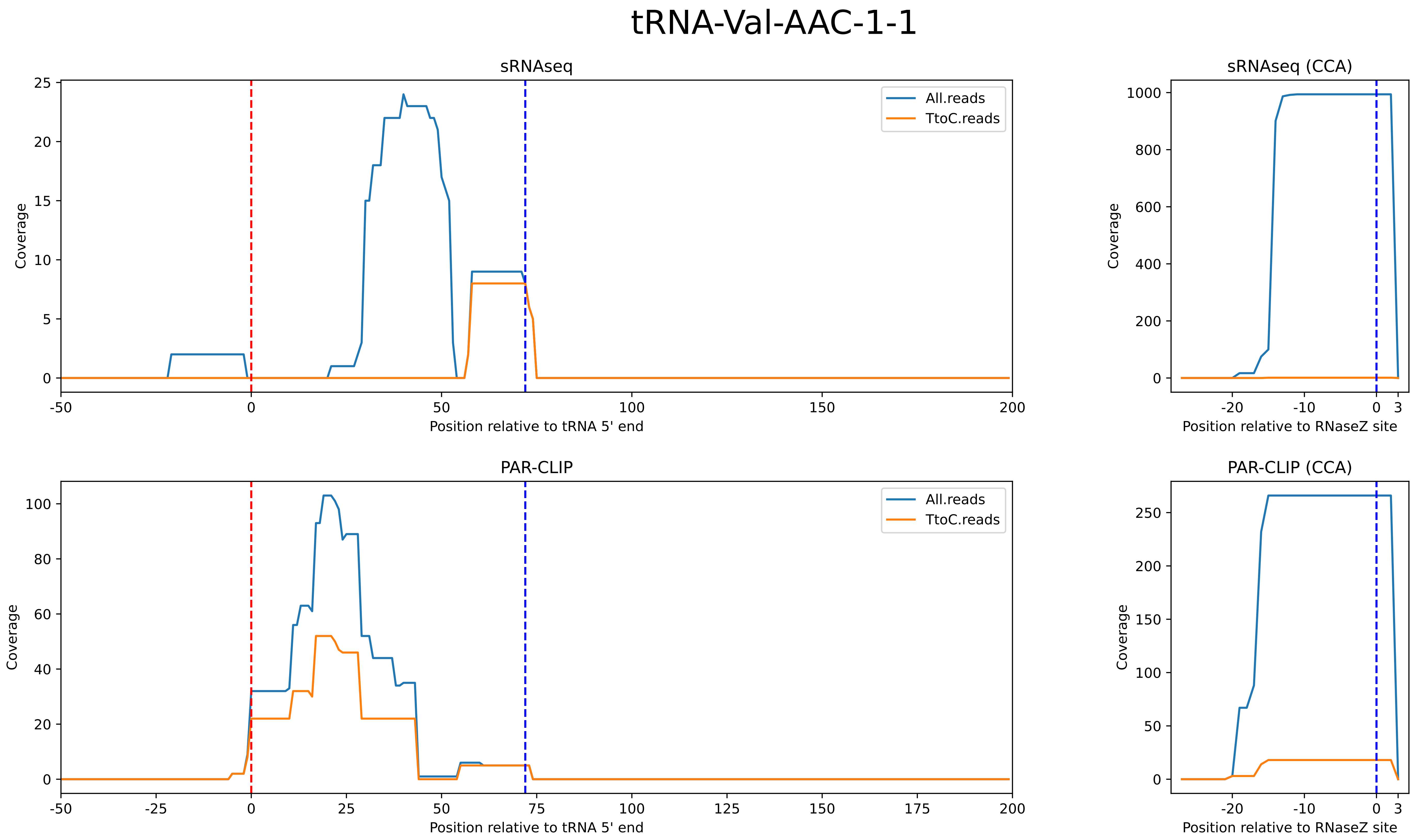

tRNA-Val-AAC-1-1
sRNAseq
All.reads
TtoC.reads
Coverage
Position relative to tRNA 5' end
sRNAseq (CCA)
Position relative to RNaseZ site
PAR-CLIP
PAR-CLIP (CCA)

# tRNA-Asp-GTC-1-8

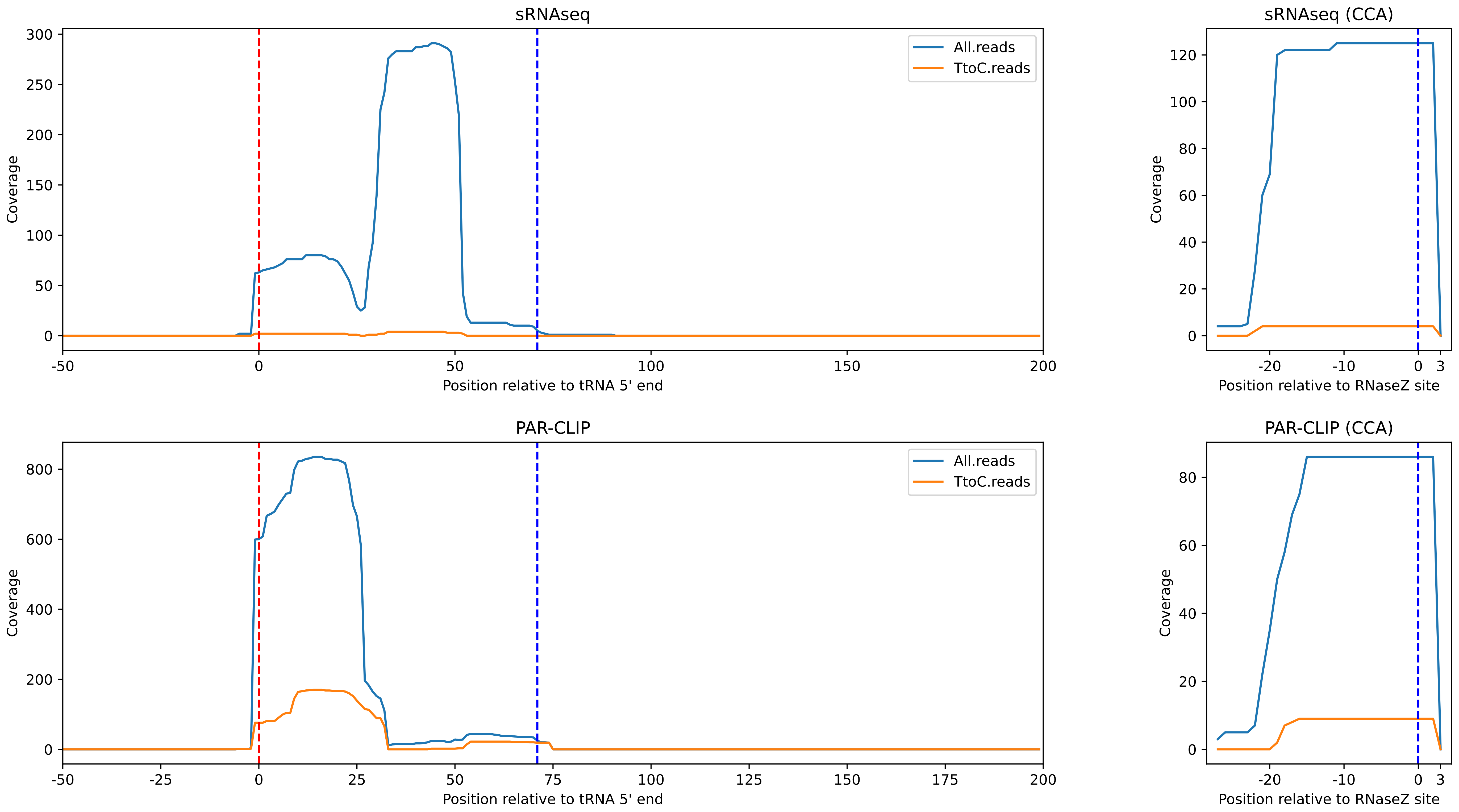

# tRNA-Gln-CTG-2-2

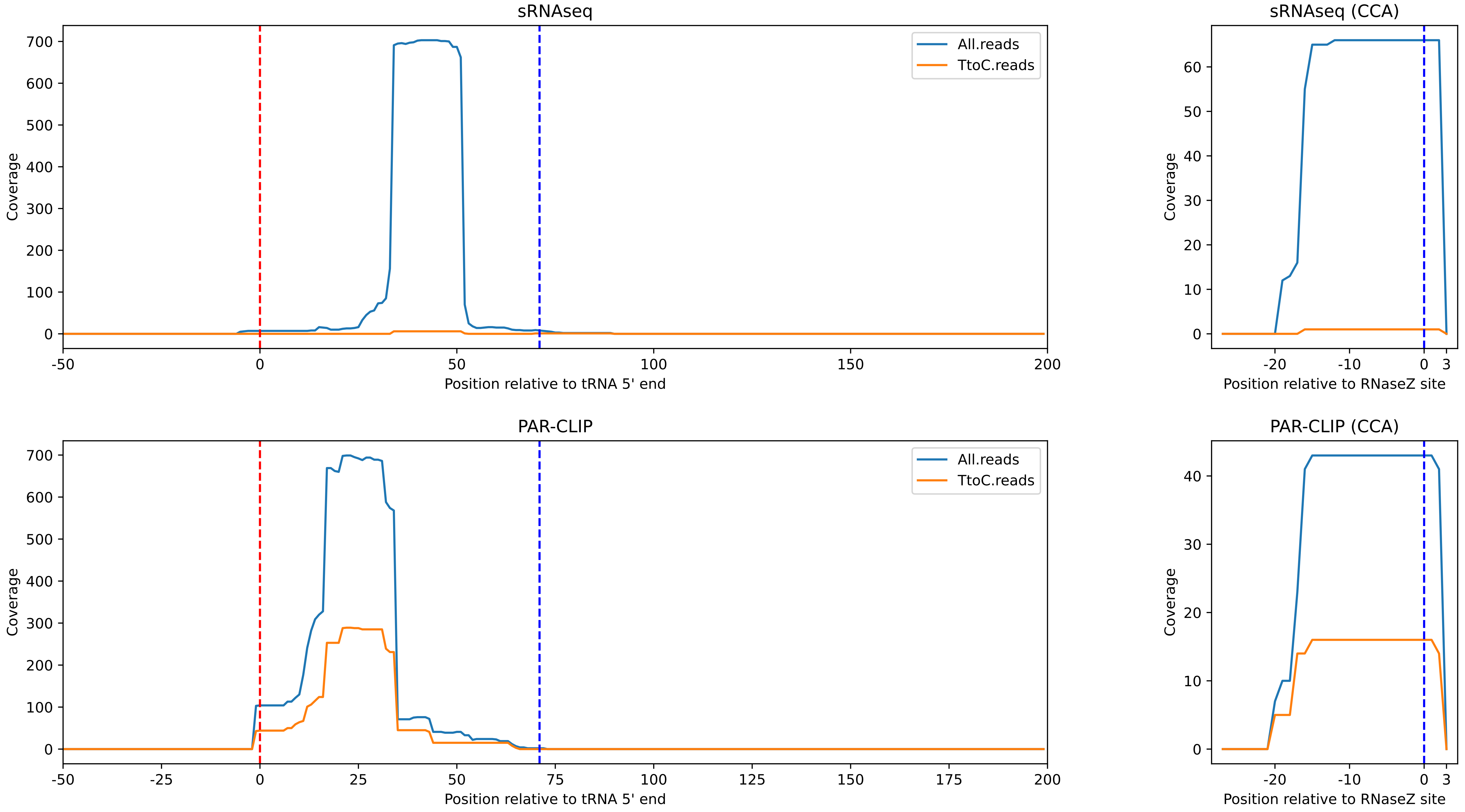

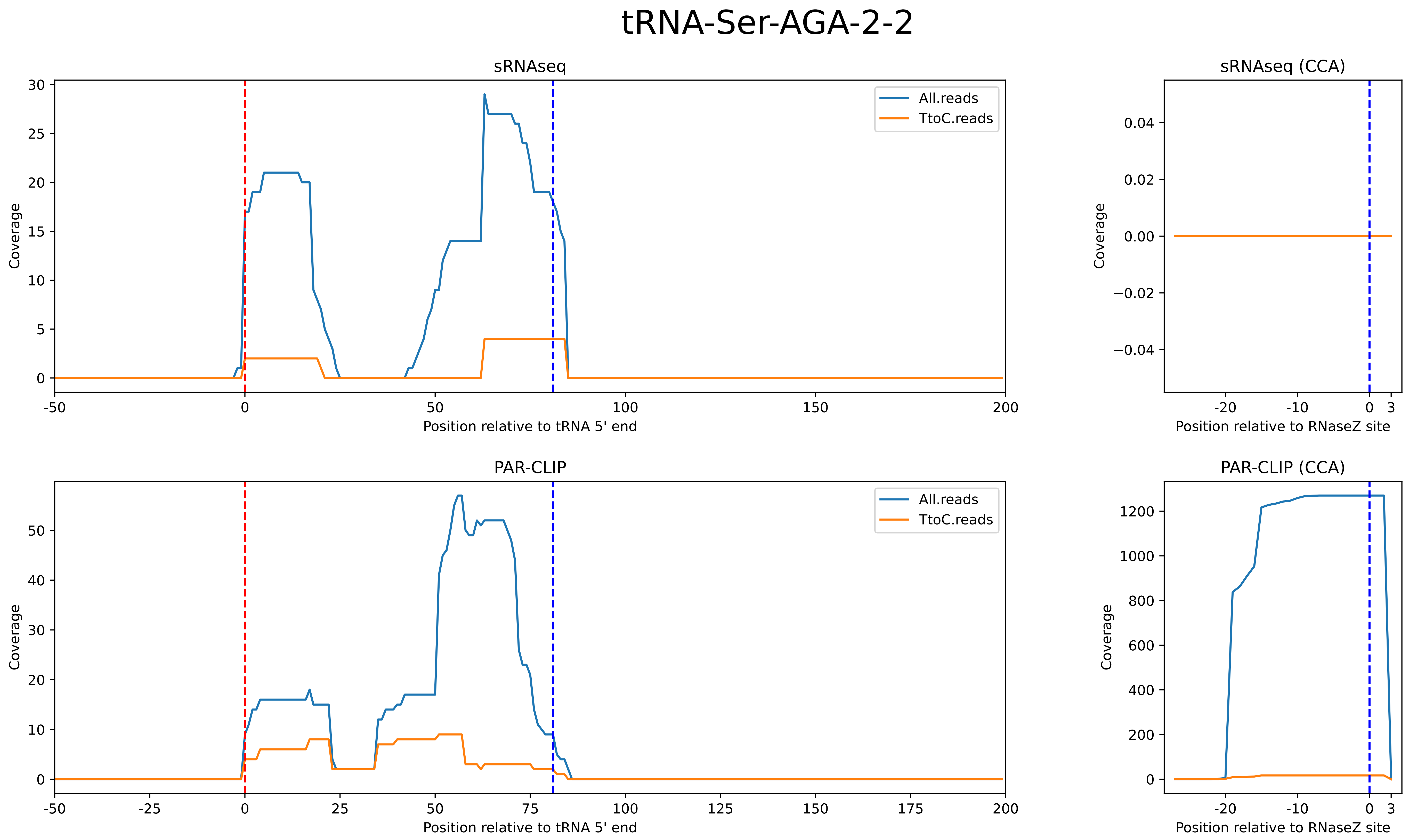

tRNA-Ser-AGA-2-2
sRNAseq
All.reads
TtoC.reads
Coverage
Position relative to tRNA 5' end
sRNAseq (CCA)
Position relative to RNaseZ site
PAR-CLIP
PAR-CLIP (CCA)

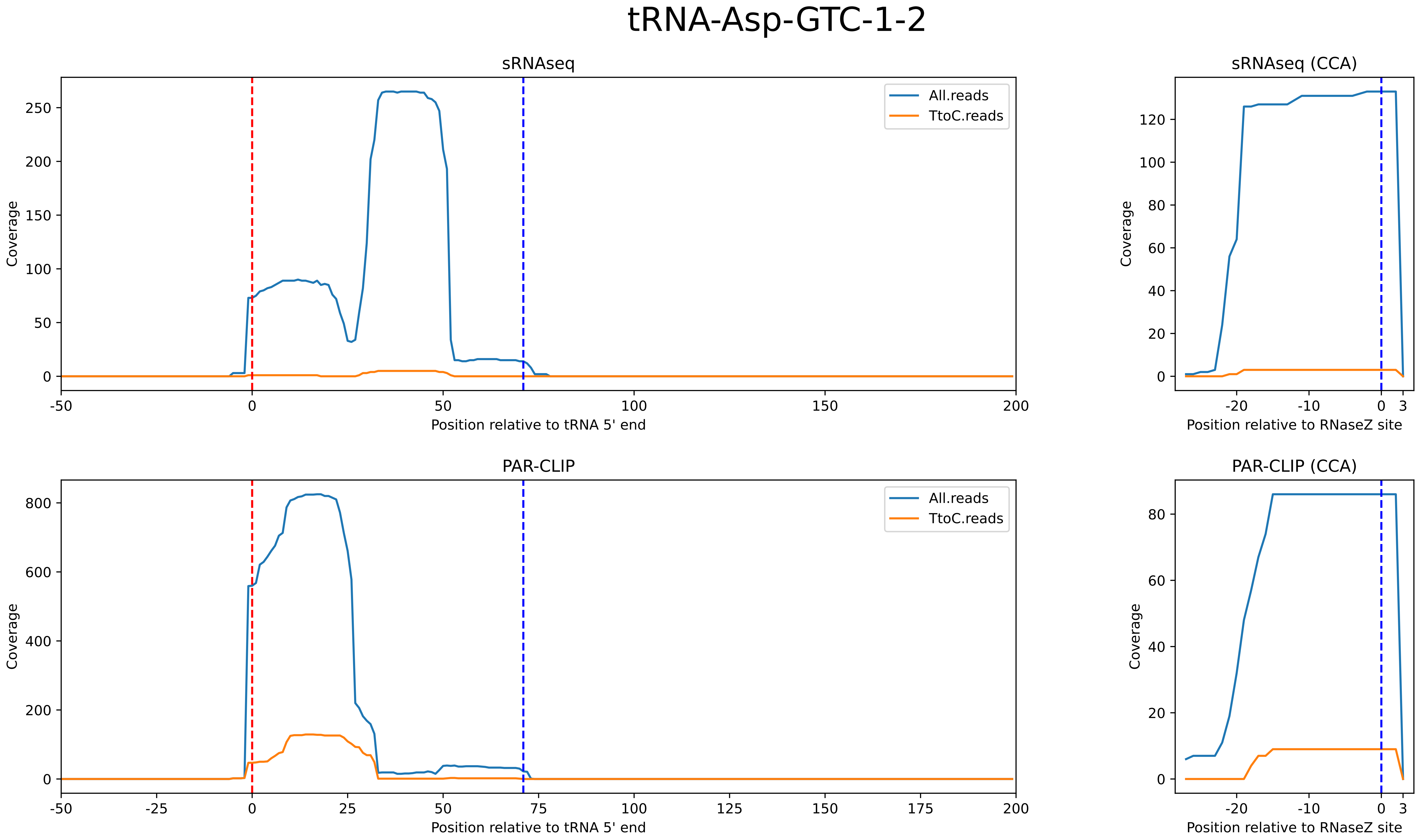

tRNA-Asp-GTC-1-2
sRNAseq
sRNAseq (CCA)
PAR-CLIP
PAR-CLIP (CCA)
All.reads
TtoC.reads
Coverage
Position relative to tRNA 5' end
Position relative to RNaseZ site

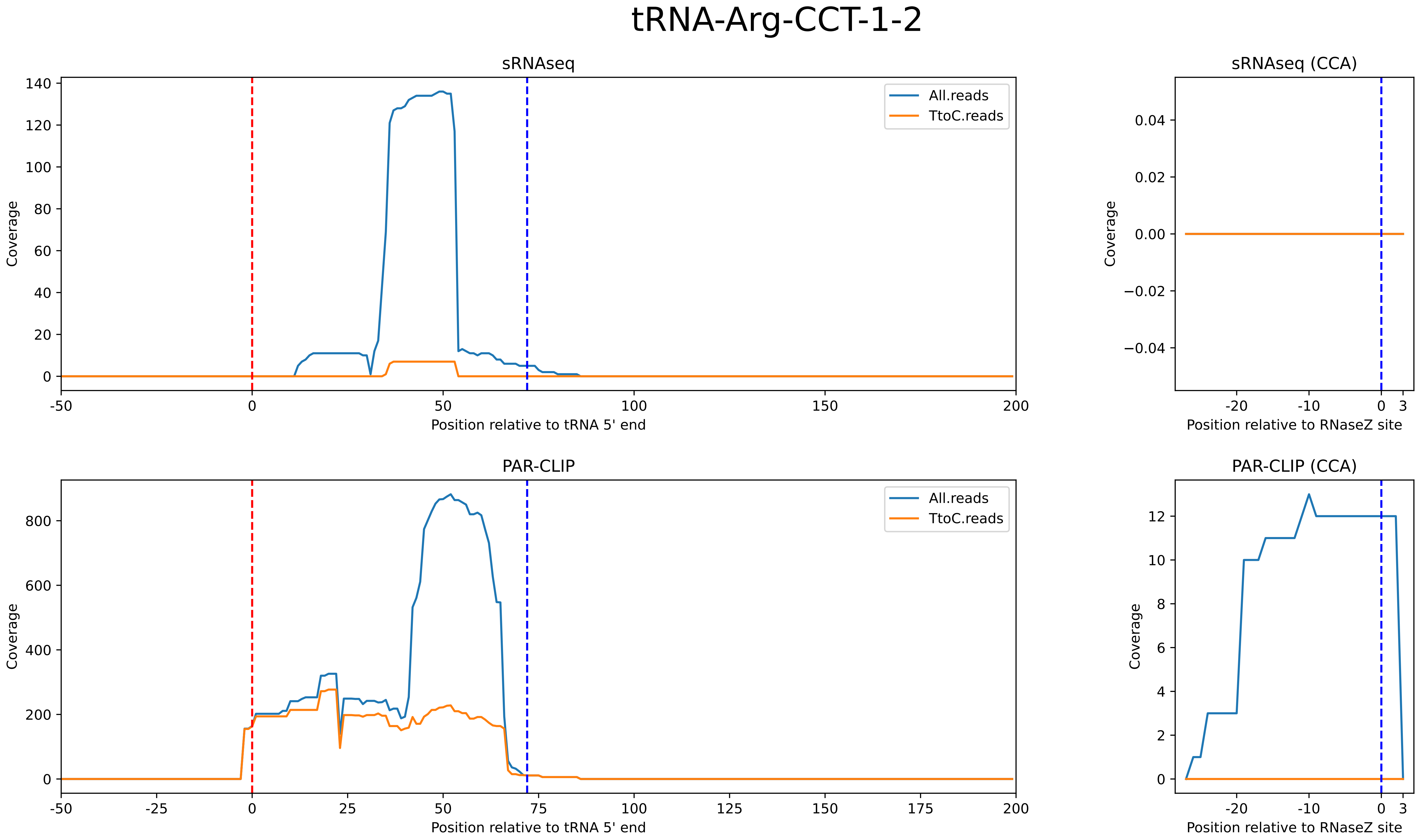
tRNA-Arg-CCT-1-2
sRNAseq
Coverage
Position relative to tRNA 5' end
All.reads
TtoC.reads
sRNAseq (CCA)
Position relative to RNaseZ site
PAR-CLIP
PAR-CLIP (CCA)

# tRNA-Lys-CTT-1-3

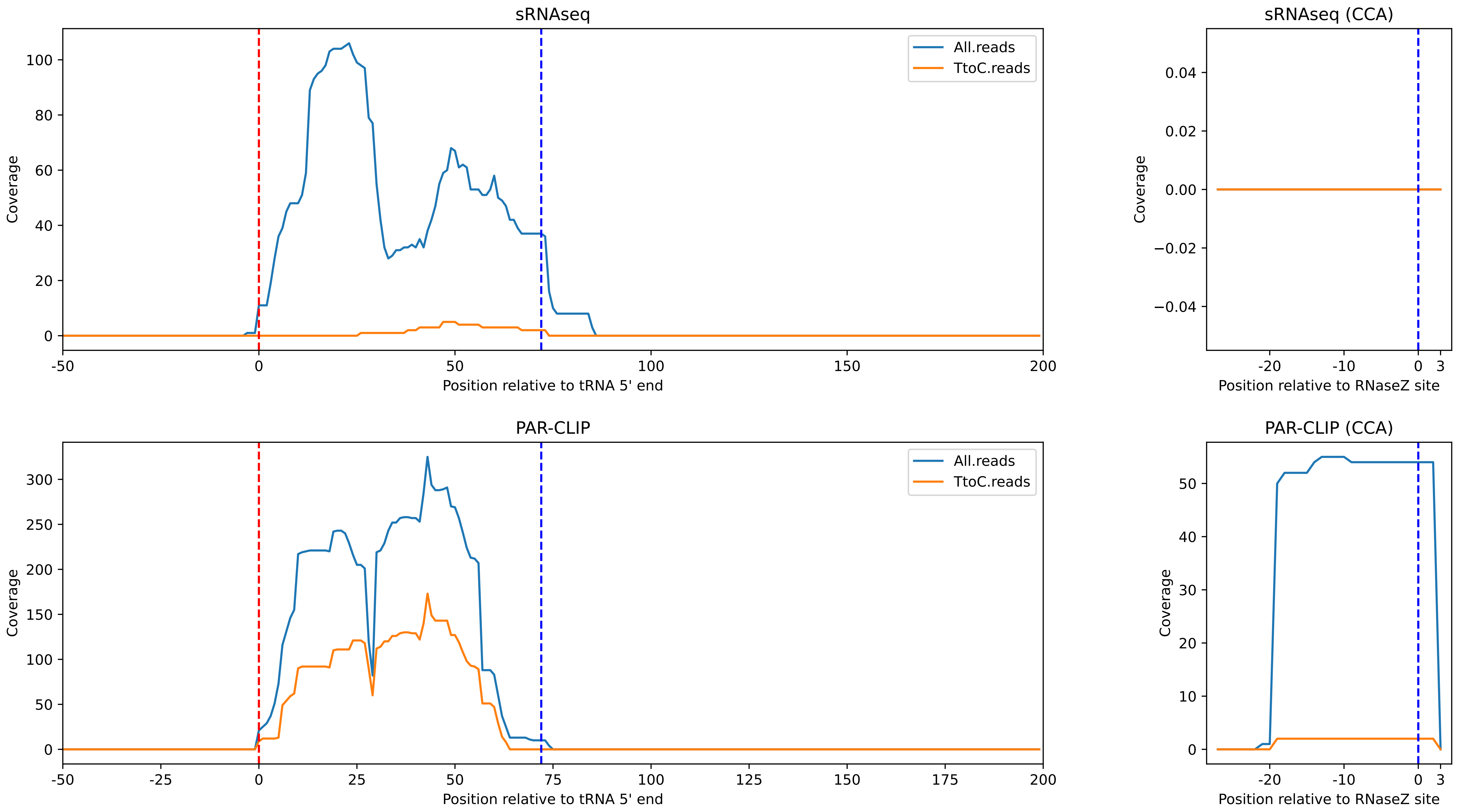

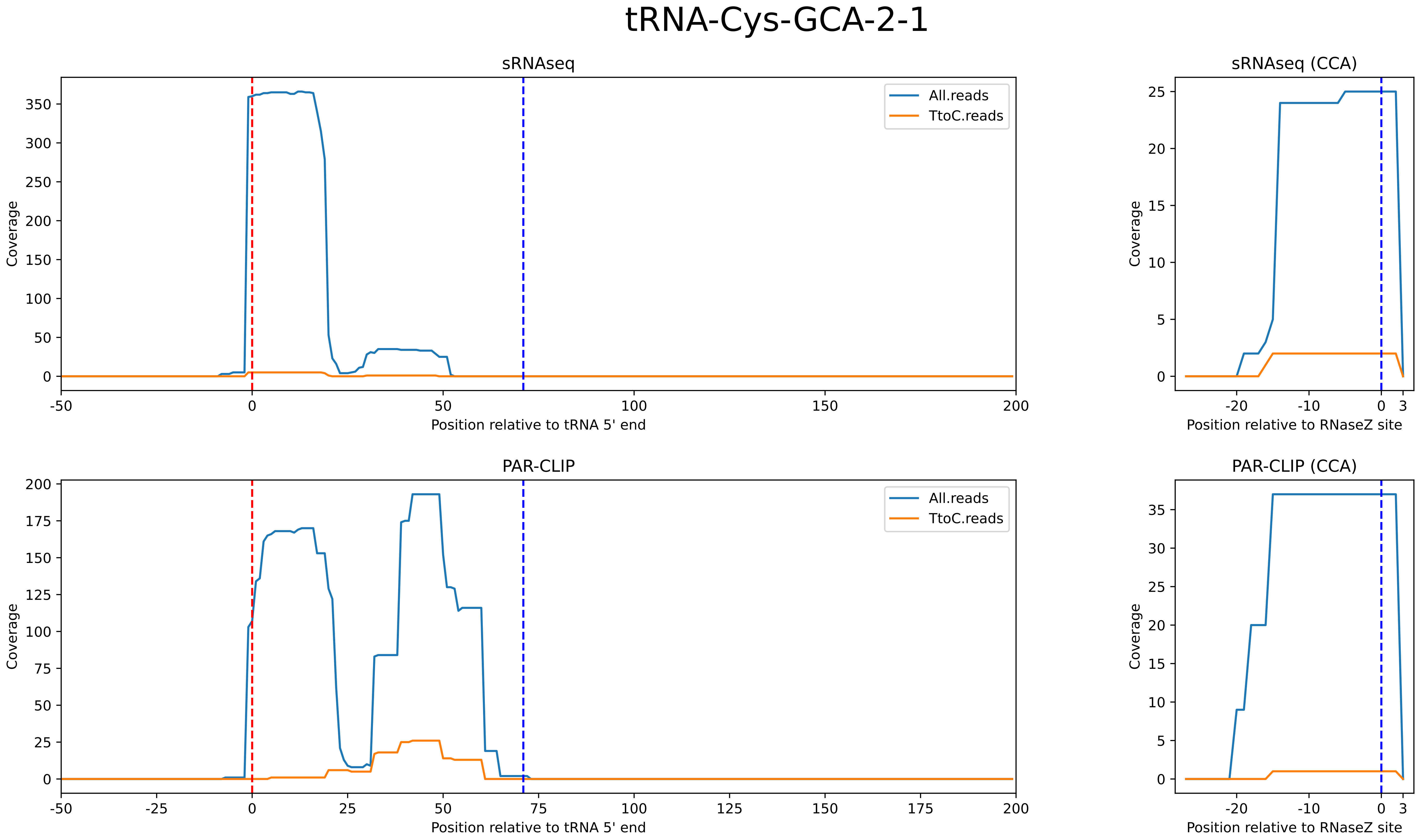
tRNA-Cys-GCA-2-1
sRNAseq
All.reads
TtoC.reads
Coverage
Position relative to tRNA 5' end
sRNAseq (CCA)
Position relative to RNaseZ site
PAR-CLIP
PAR-CLIP (CCA)

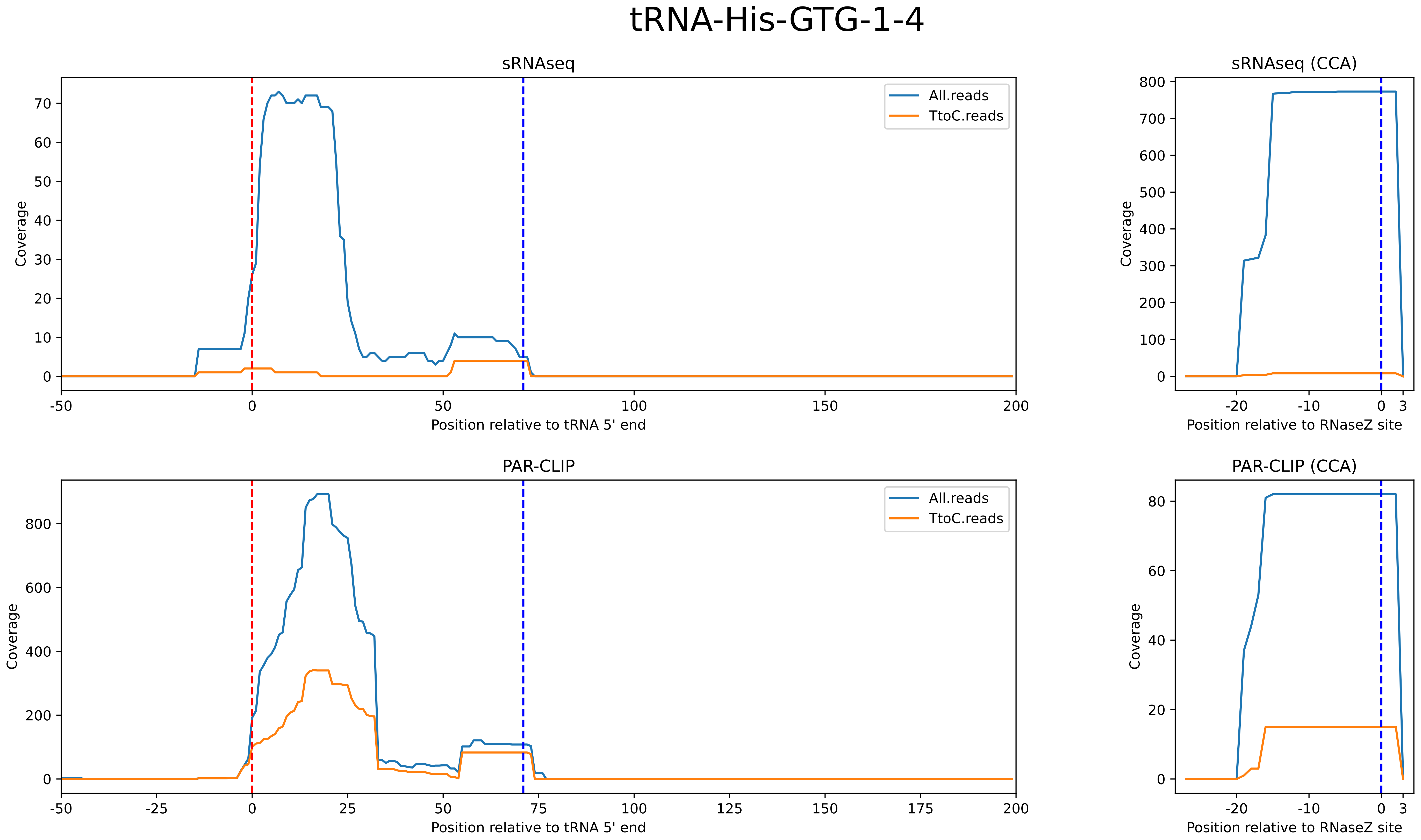

tRNA-His-GTG-1-4
sRNAseq
All.reads
TtoC.reads
Coverage
Position relative to tRNA 5' end
sRNAseq (CCA)
Position relative to RNaseZ site
PAR-CLIP
PAR-CLIP (CCA)

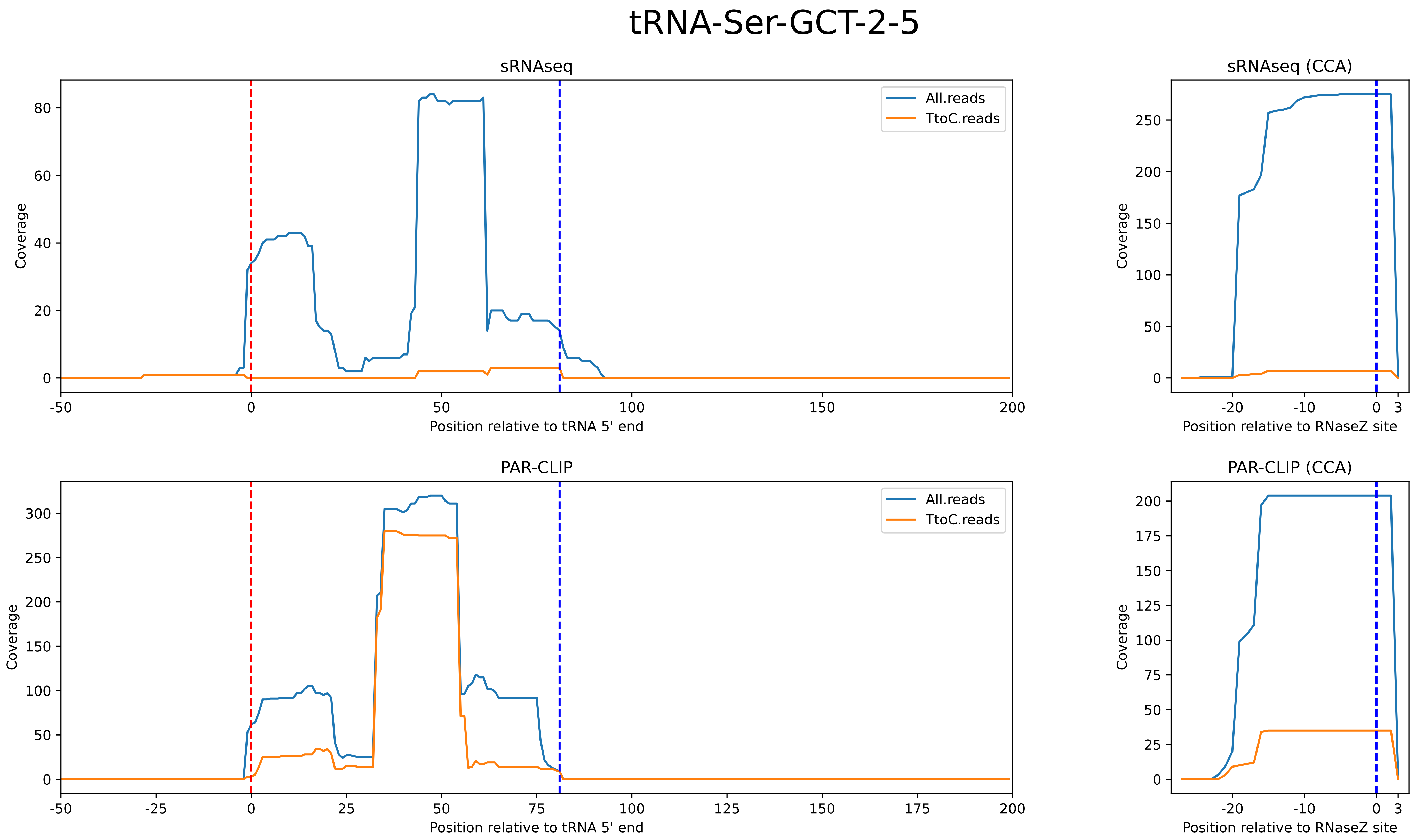
tRNA-Ser-GCT-2-5
sRNAseq
All.reads
TtoC.reads
Coverage
Position relative to tRNA 5' end
sRNAseq (CCA)
Position relative to RNaseZ site
PAR-CLIP
PAR-CLIP (CCA)

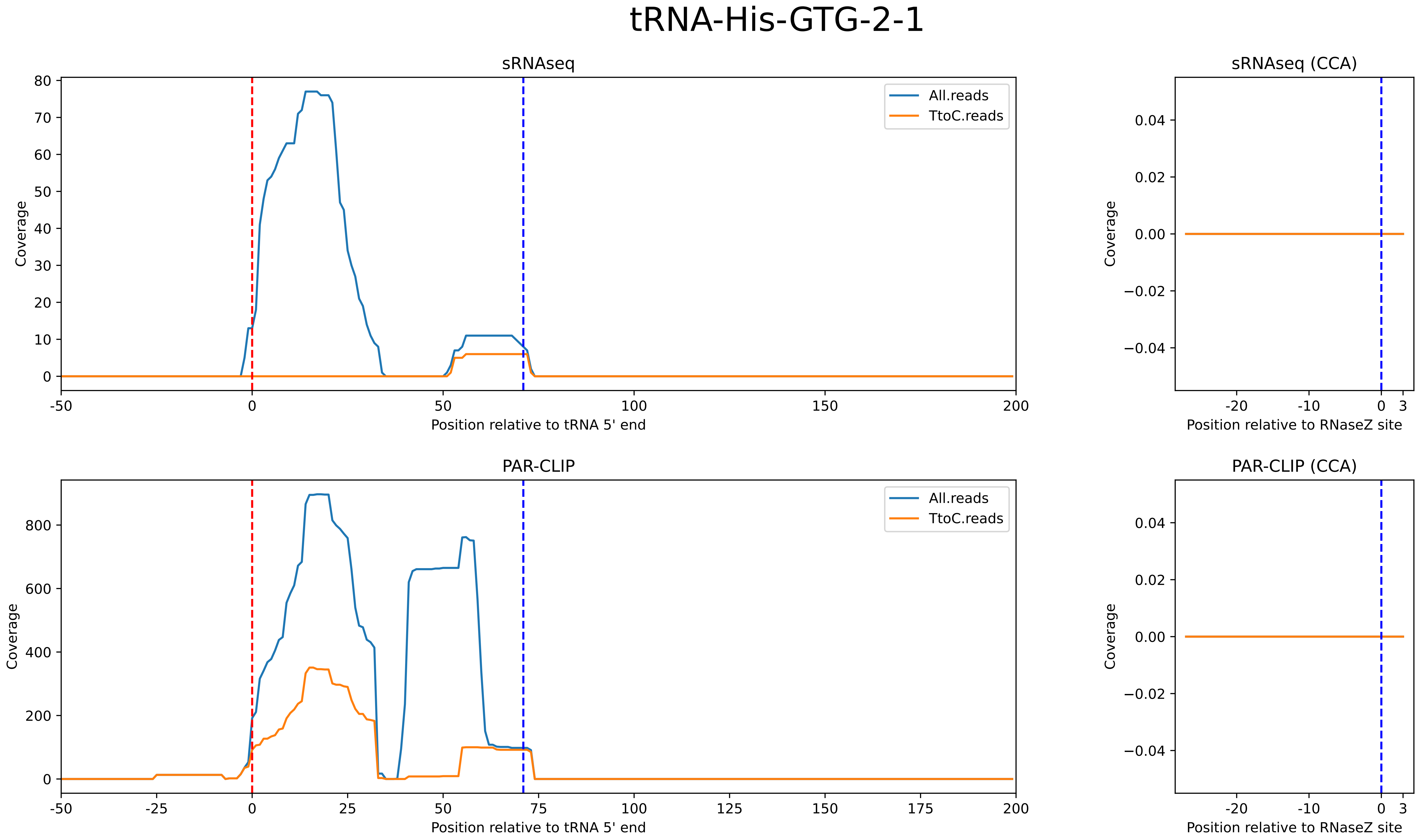
tRNA-His-GTG-2-1
sRNAseq
All.reads
TtoC.reads
Coverage
Position relative to tRNA 5' end
sRNAseq (CCA)
Position relative to RNaseZ site
PAR-CLIP
PAR-CLIP (CCA)

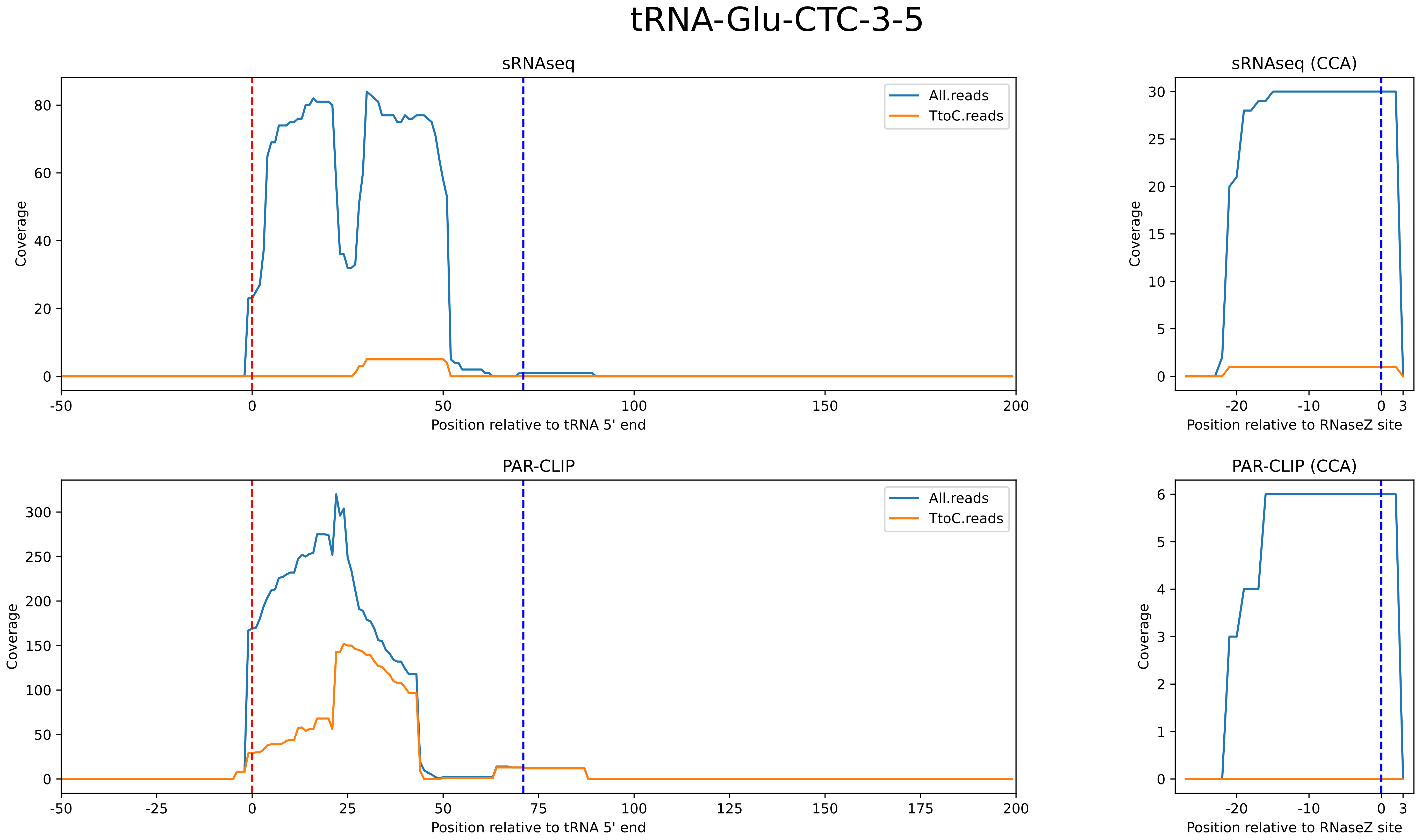

tRNA-Glu-CTC-3-5
sRNAseq
All.reads
TtoC.reads
Coverage
Position relative to tRNA 5' end
sRNAseq (CCA)
Position relative to RNaseZ site
PAR-CLIP
PAR-CLIP (CCA)

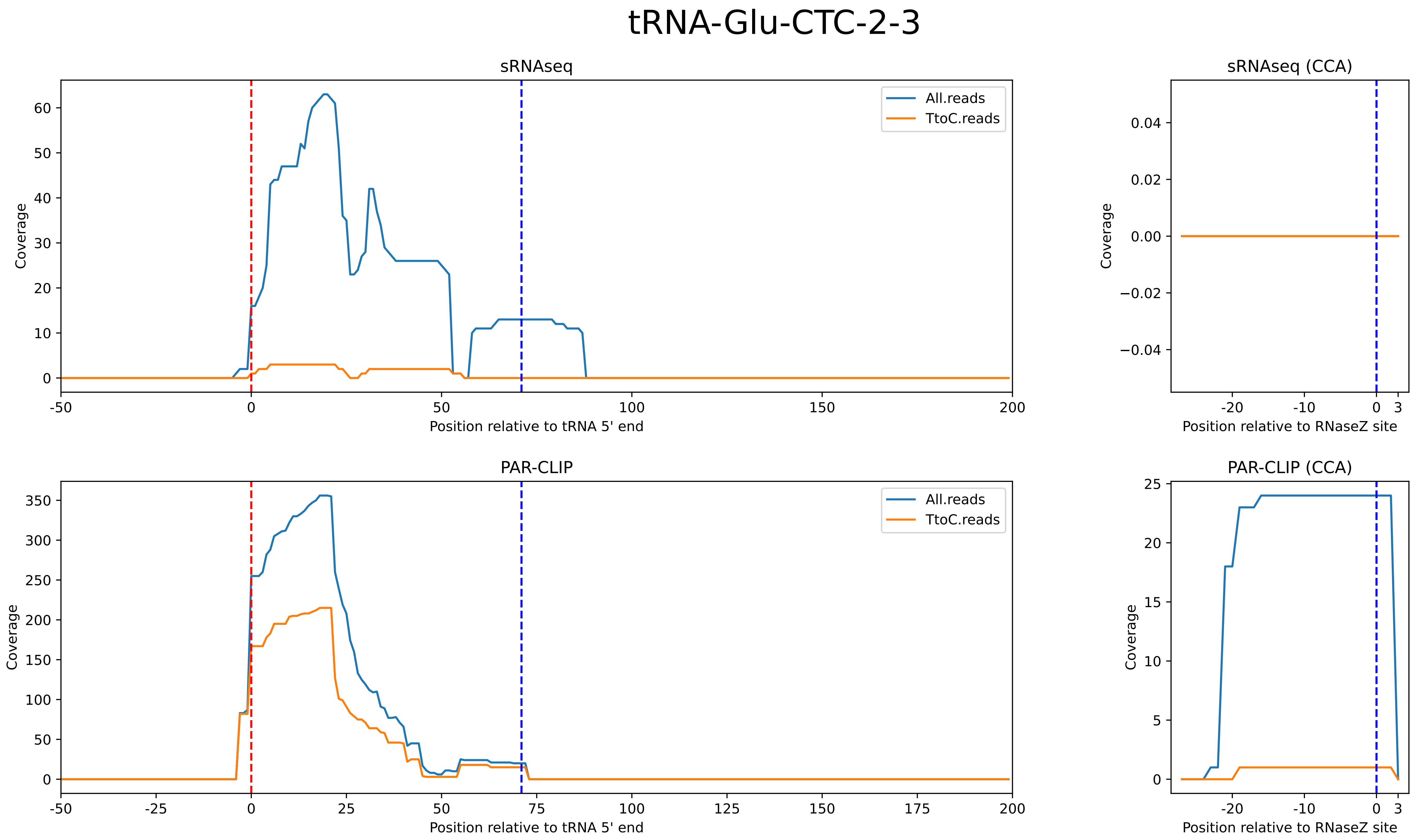
tRNA-Glu-CTC-2-3
sRNAseq
sRNAseq (CCA)
PAR-CLIP
PAR-CLIP (CCA)
All.reads
TtoC.reads
Coverage
Position relative to tRNA 5' end
Position relative to RNaseZ site

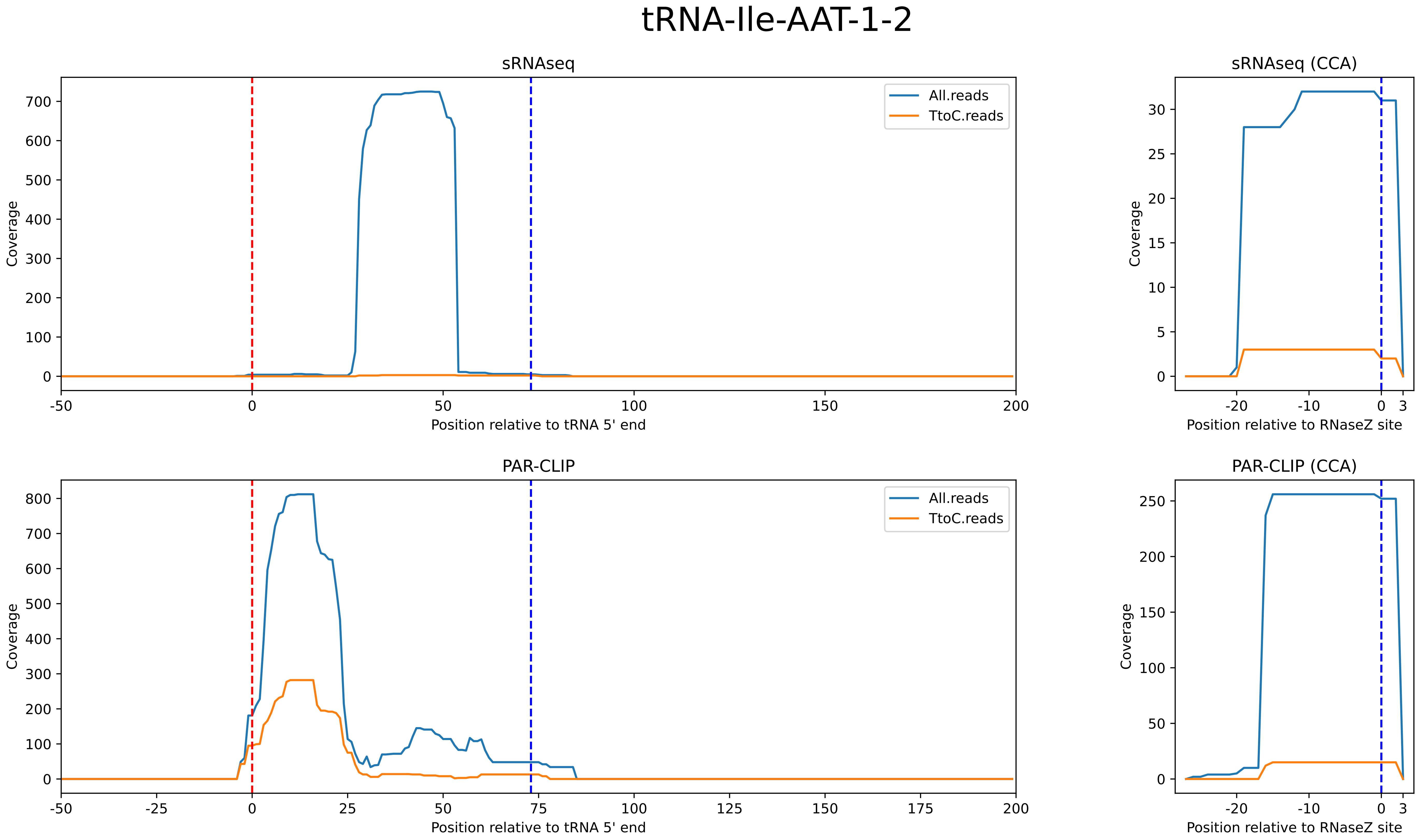
tRNA-Ile-AAT-1-2
sRNAseq
sRNAseq (CCA)
PAR-CLIP
PAR-CLIP (CCA)
All.reads
TtoC.reads
Coverage
Position relative to tRNA 5' end
Position relative to RNaseZ site

# tRNA-Glu-CTC-2-1

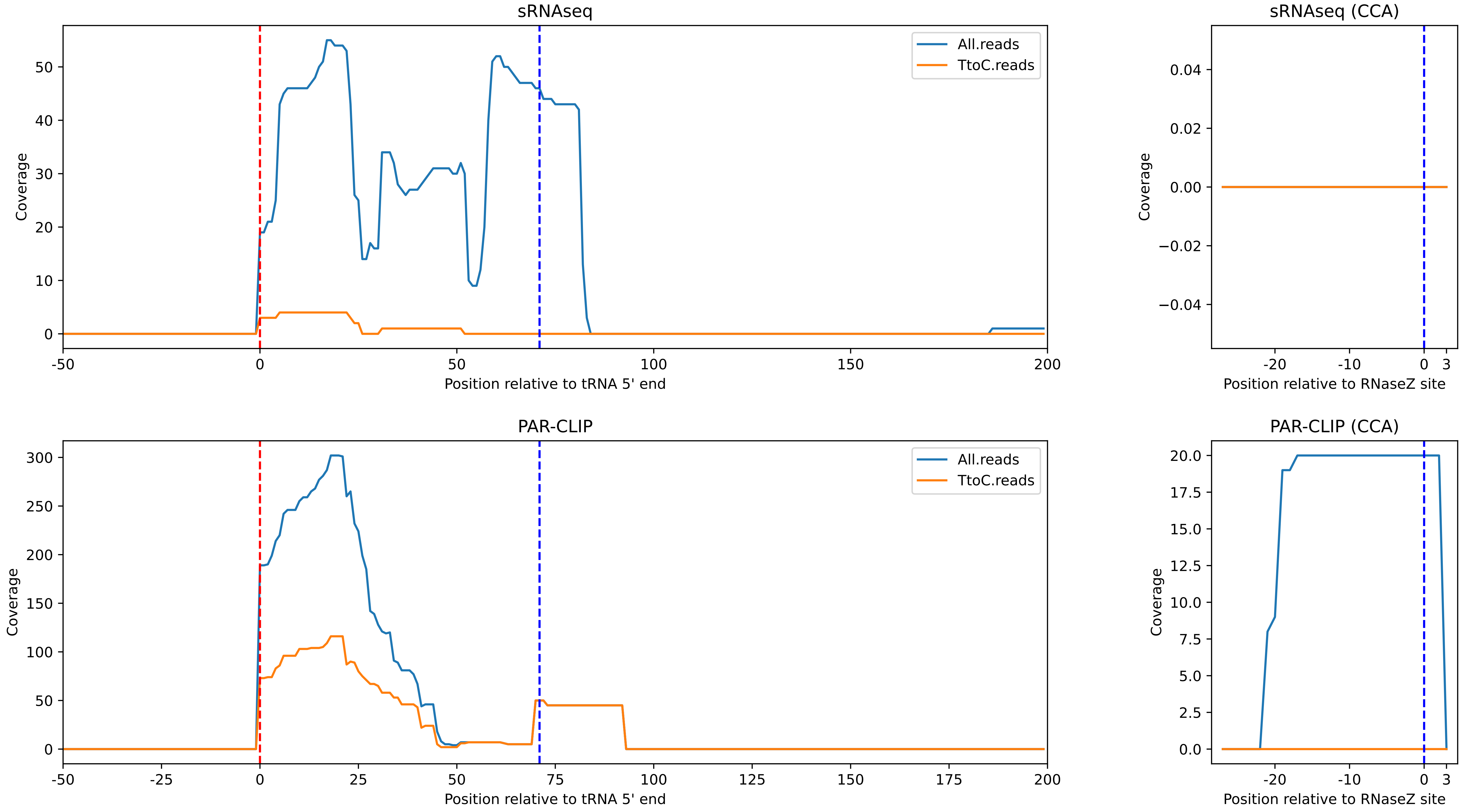

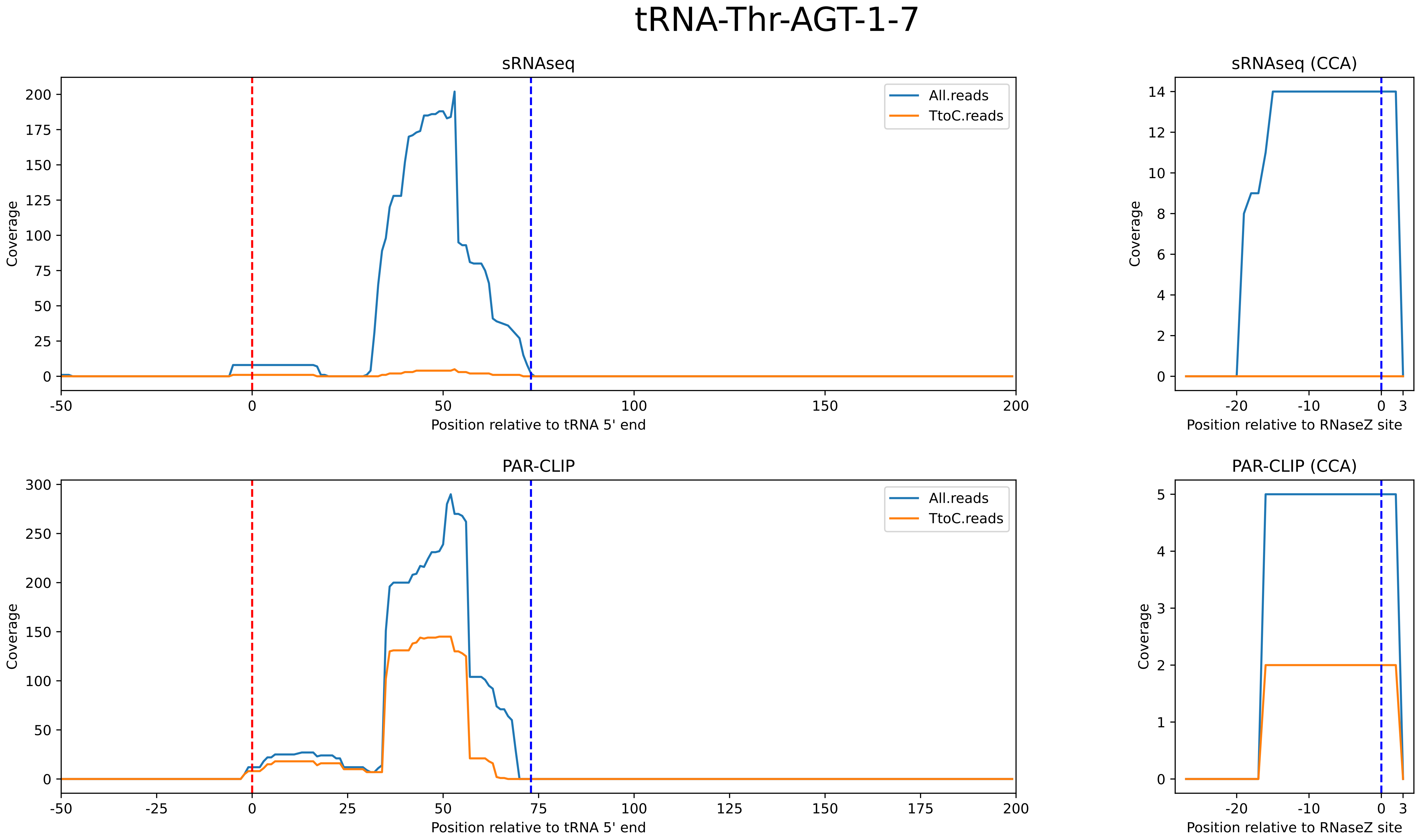
tRNA-Thr-AGT-1-7
sRNAseq
sRNAseq (CCA)
PAR-CLIP
PAR-CLIP (CCA)
All.reads
TtoC.reads
Coverage
Position relative to tRNA 5' end
Position relative to RNaseZ site

tRNA-Ala-TGC-2-1

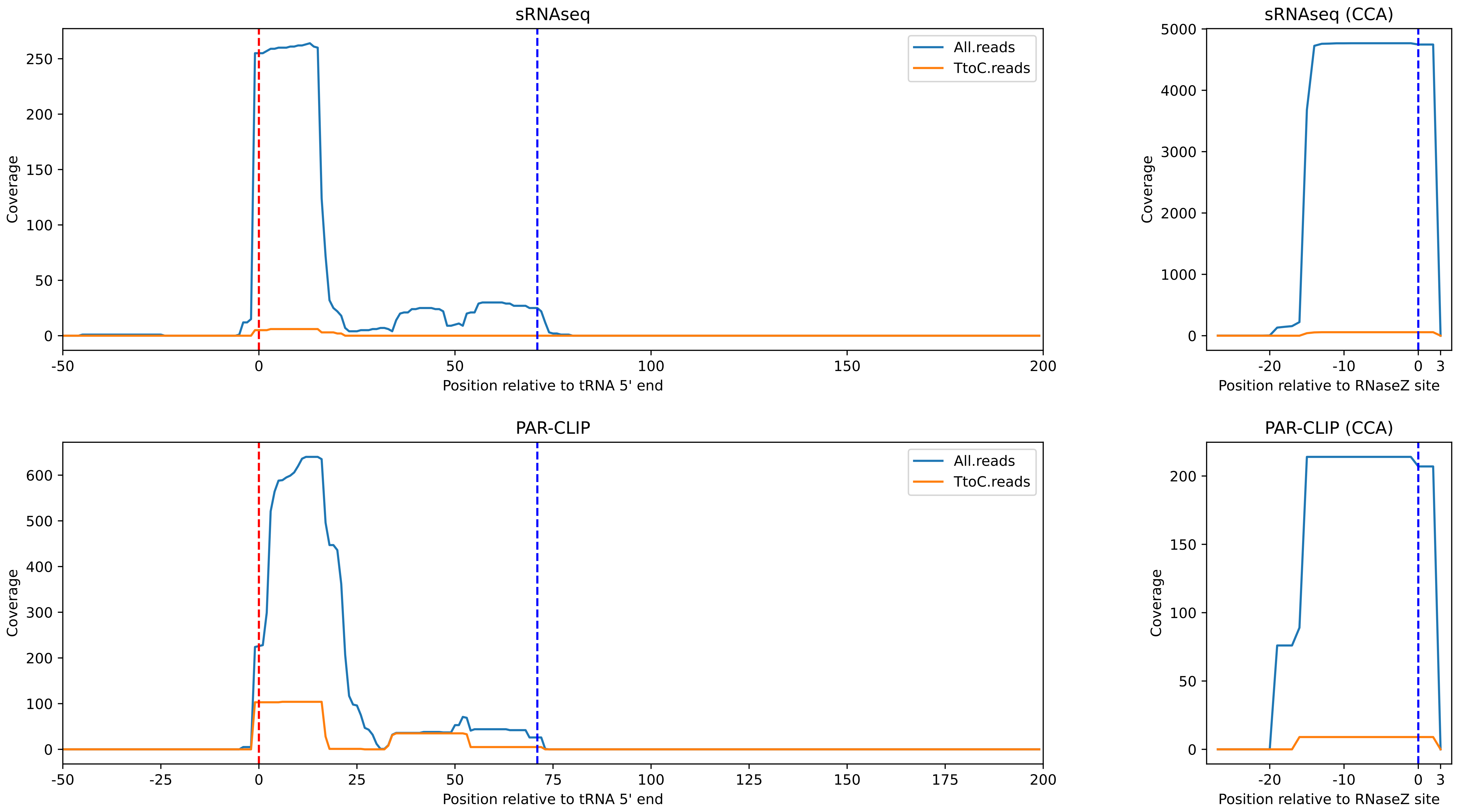

# tRNA-Met-CAT-1-3

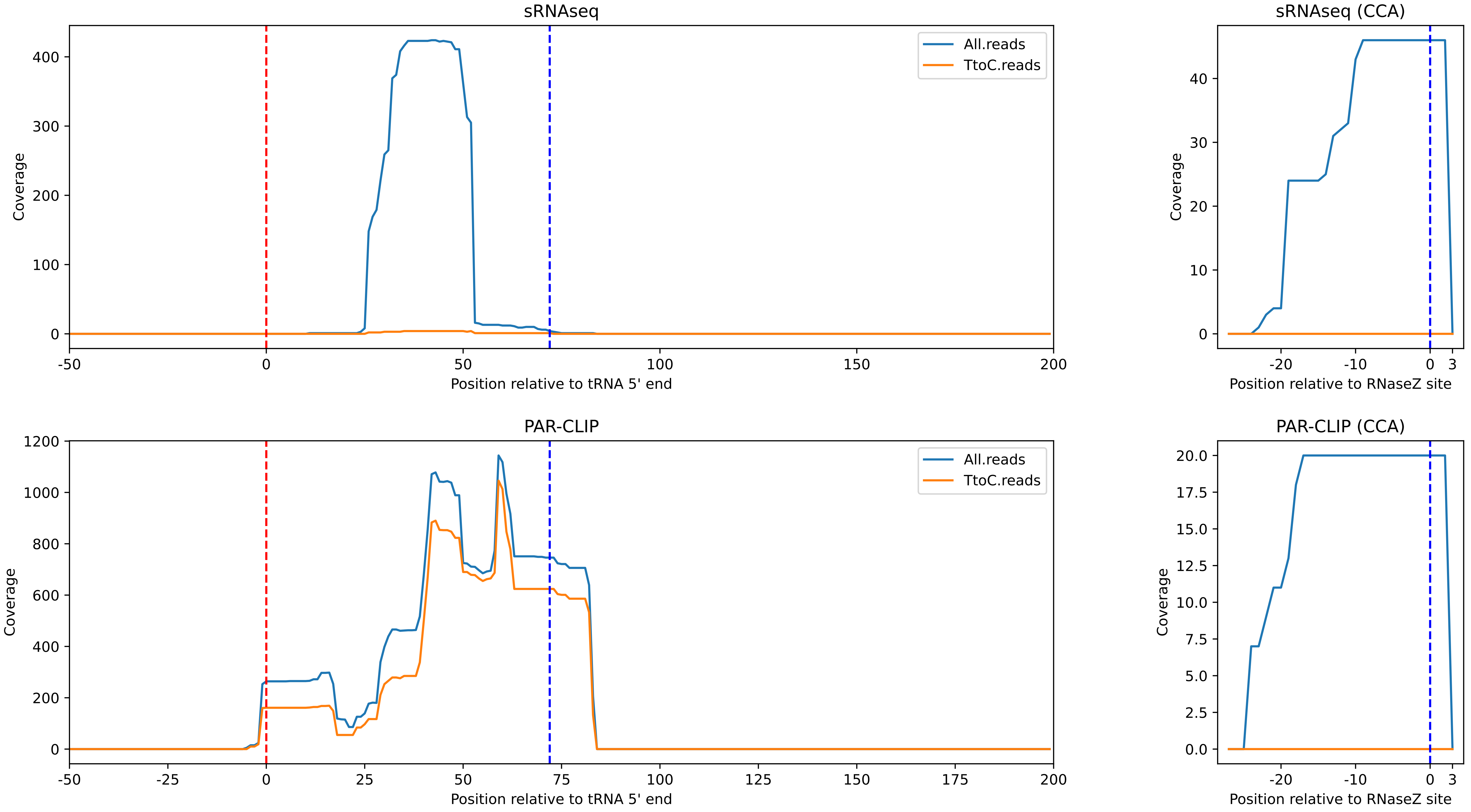

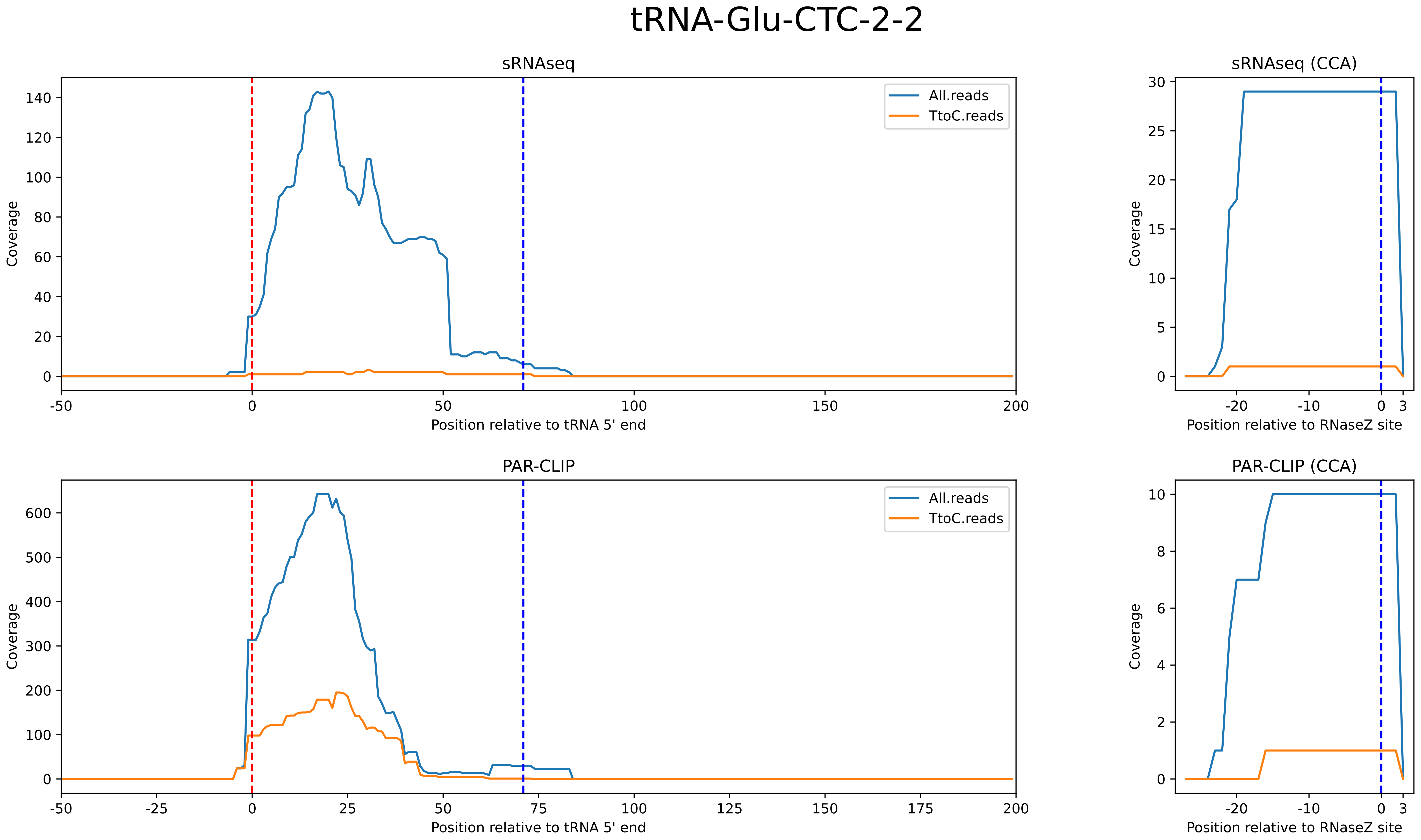

tRNA-Glu-CTC-2-2
sRNAseq
All.reads
TtoC.reads
Coverage
Position relative to tRNA 5' end
sRNAseq (CCA)
Position relative to RNaseZ site
PAR-CLIP
PAR-CLIP (CCA)

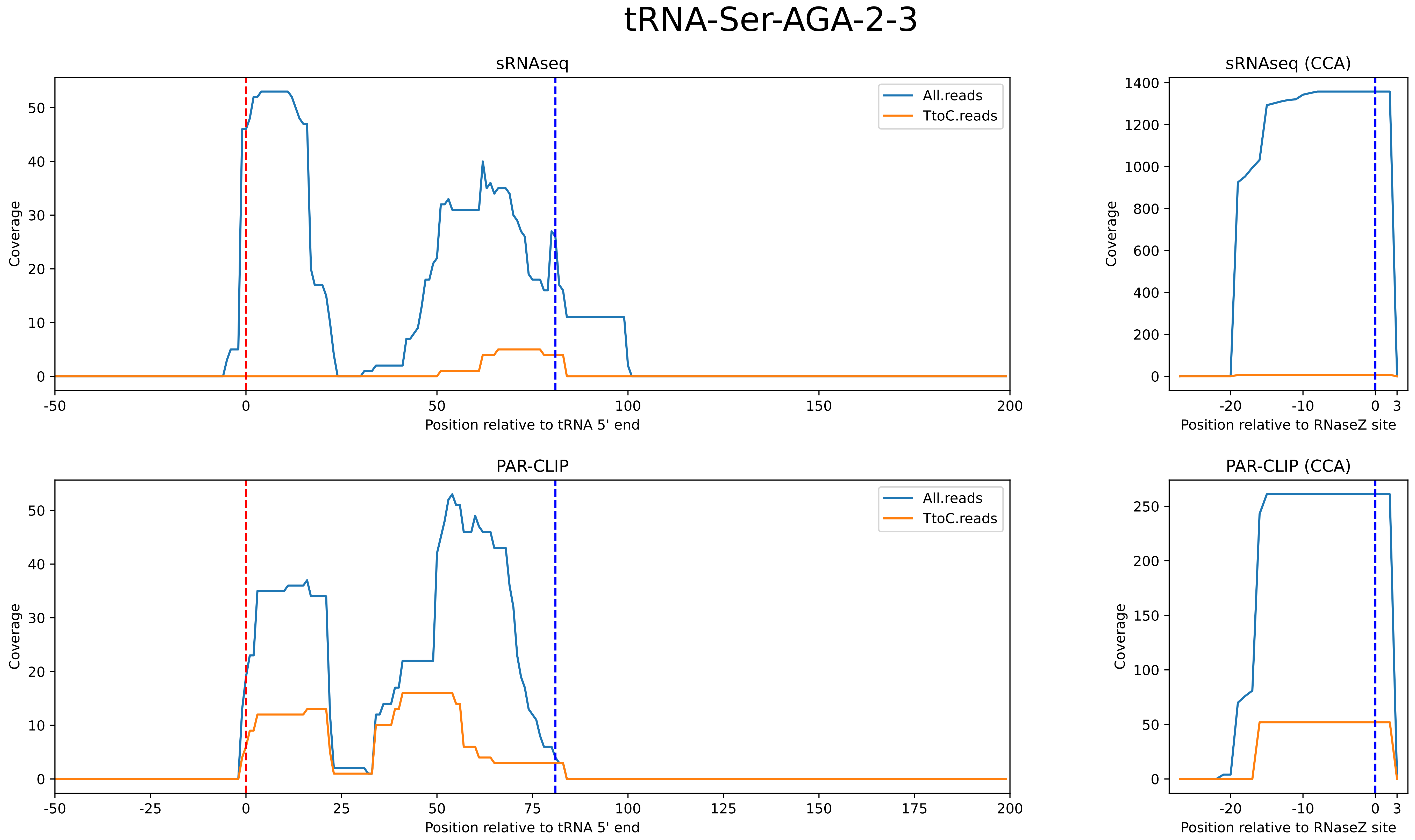

tRNA-Ser-AGA-2-3
sRNAseq
sRNAseq (CCA)
PAR-CLIP
PAR-CLIP (CCA)
All.reads
TtoC.reads
Coverage
Position relative to tRNA 5' end
Position relative to RNaseZ site

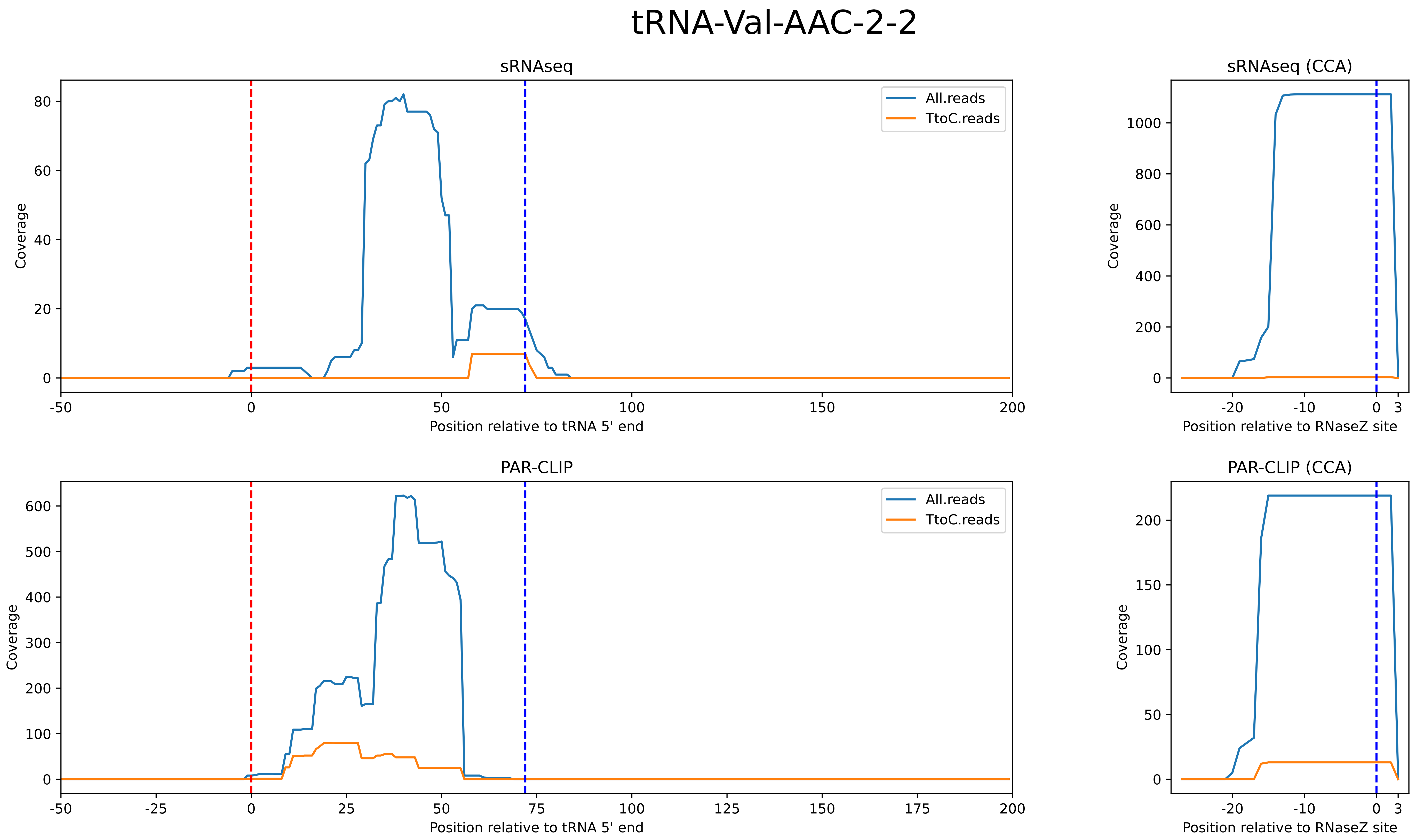
tRNA-Val-AAC-2-2
sRNAseq
All.reads
TtoC.reads
Coverage
Position relative to tRNA 5' end
sRNAseq (CCA)
Position relative to RNaseZ site
PAR-CLIP
PAR-CLIP (CCA)
-50
-25
0
25
50
75
100
125
150
175
200
-20
-10
3

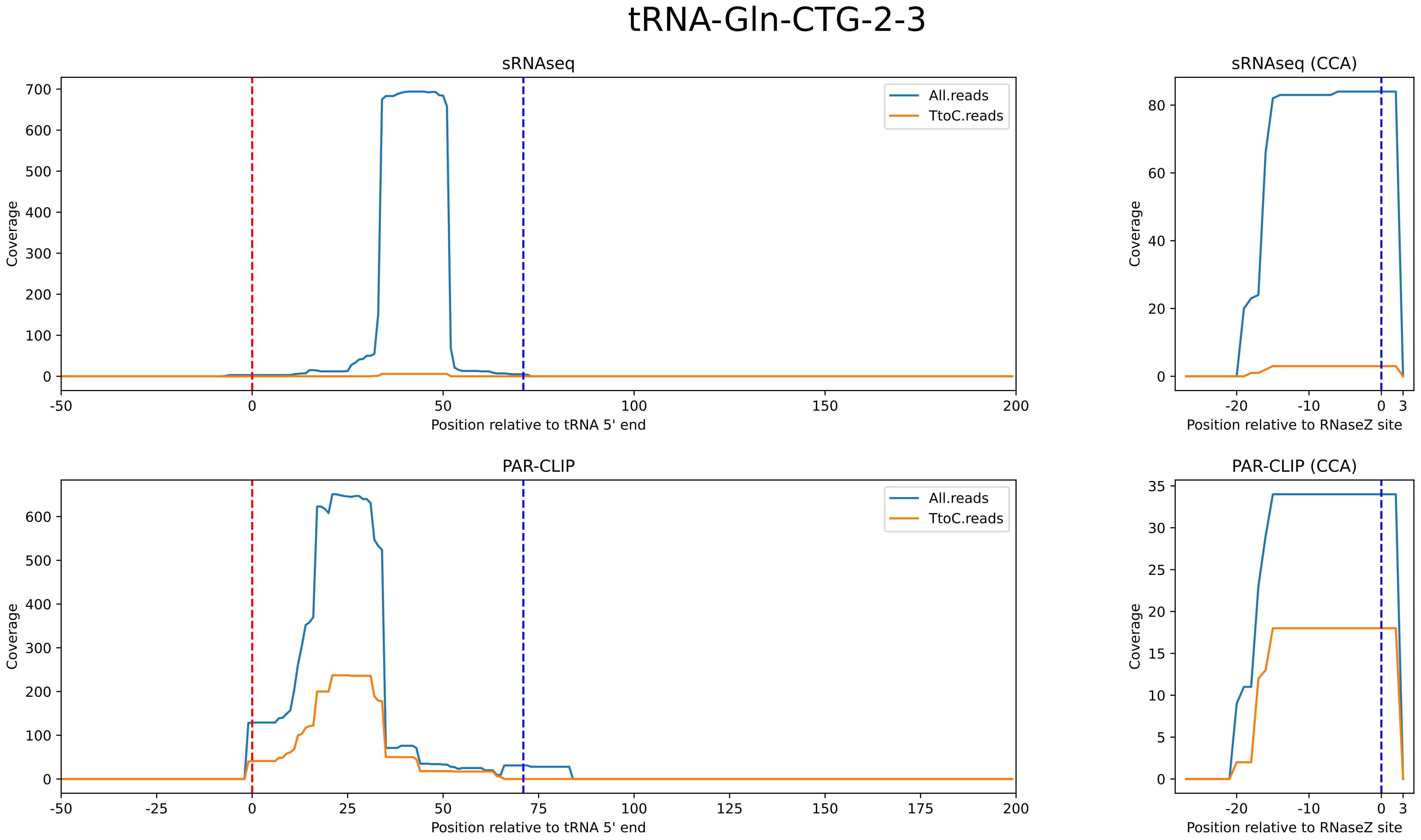

tRNA-Gln-CTG-2-3
sRNAseq
sRNAseq (CCA)
PAR-CLIP
PAR-CLIP (CCA)
All.reads
TtoC.reads
Coverage
Position relative to tRNA 5' end
Position relative to RNaseZ site

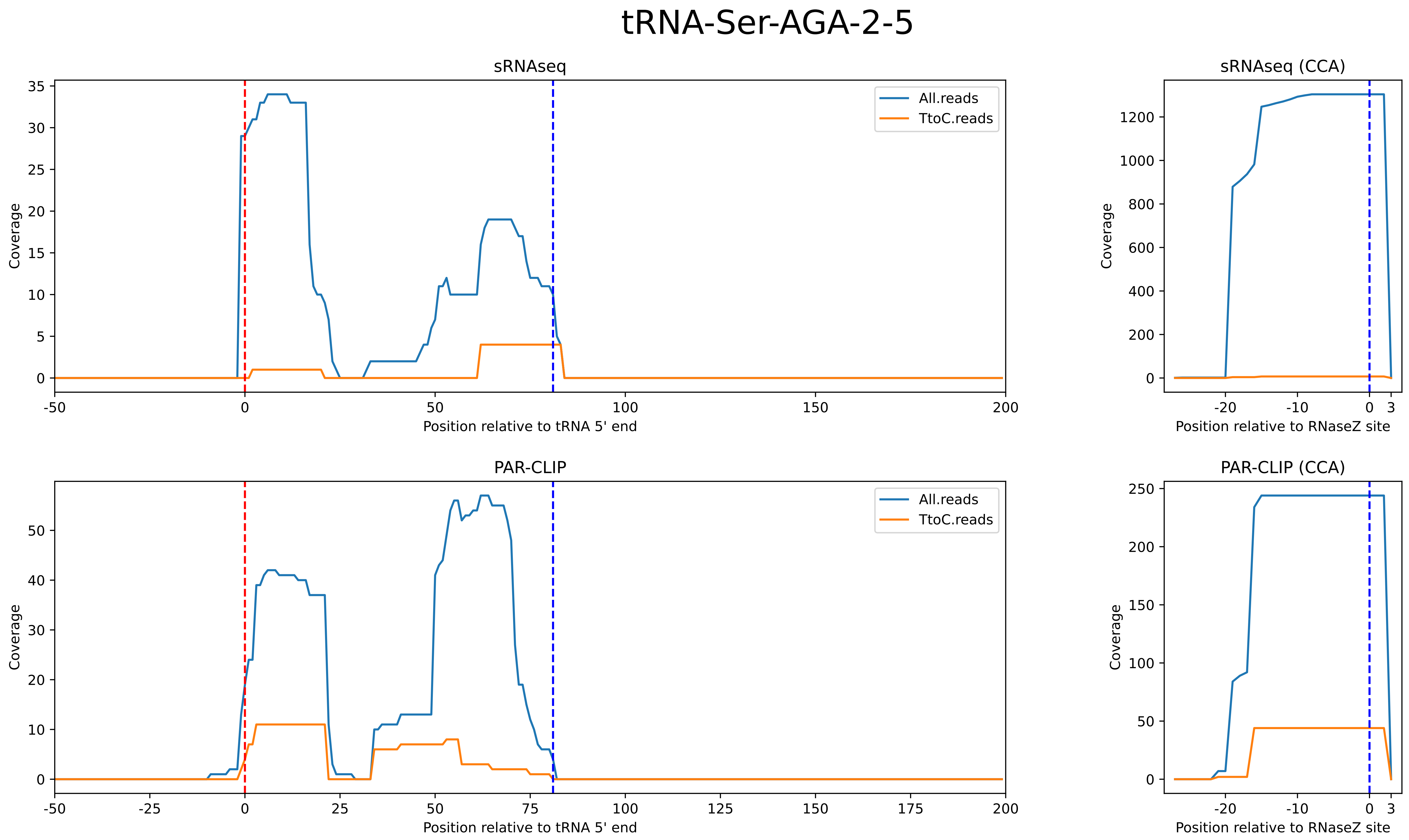
tRNA-Ser-AGA-2-5
sRNAseq
sRNAseq (CCA)
PAR-CLIP
PAR-CLIP (CCA)
All.reads
TtoC.reads
Coverage
Position relative to tRNA 5' end
Position relative to RNaseZ site

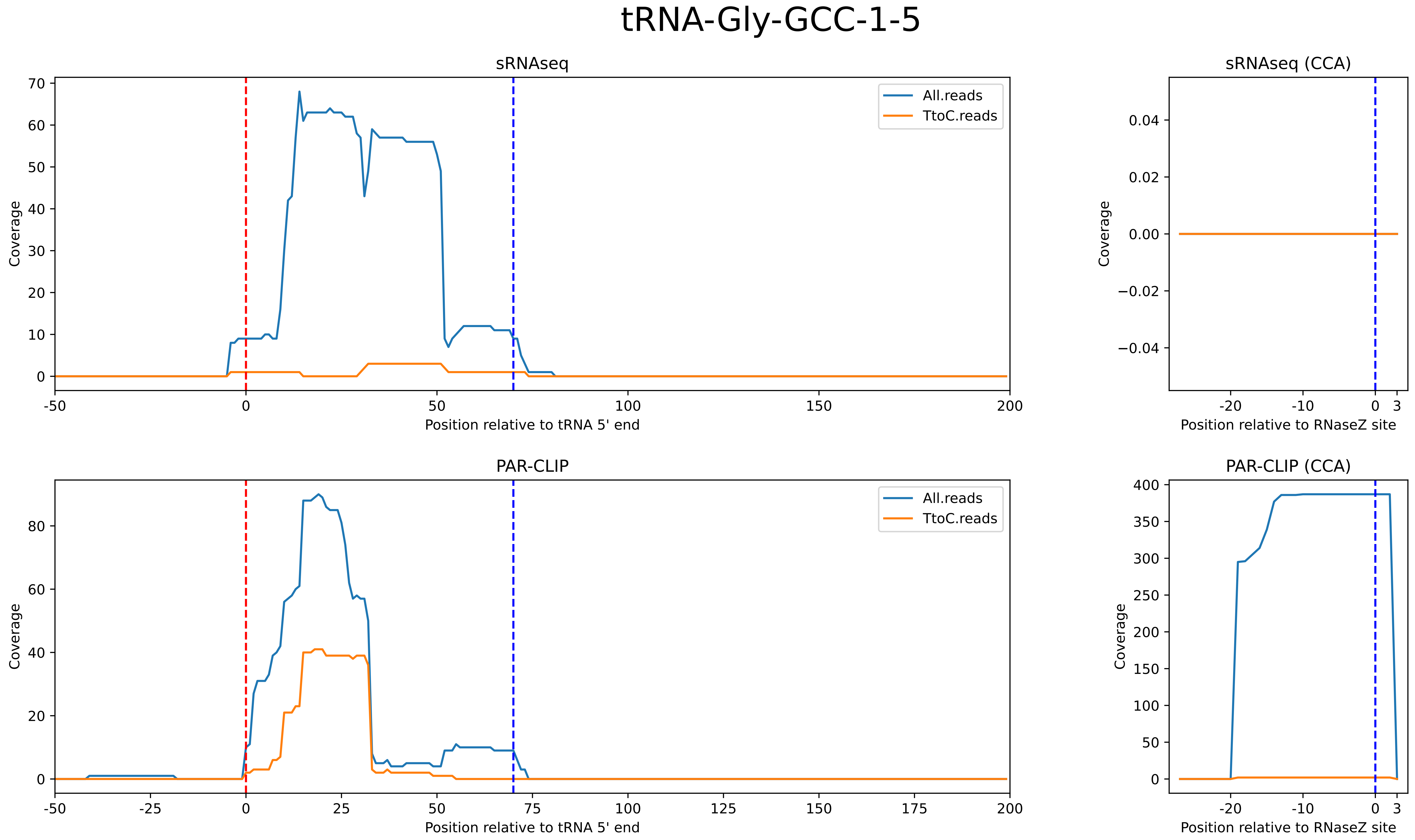

tRNA-Gly-GCC-1-5
sRNAseq
All.reads
TtoC.reads
Coverage
Position relative to tRNA 5' end
sRNAseq (CCA)
Position relative to RNaseZ site
PAR-CLIP
PAR-CLIP (CCA)

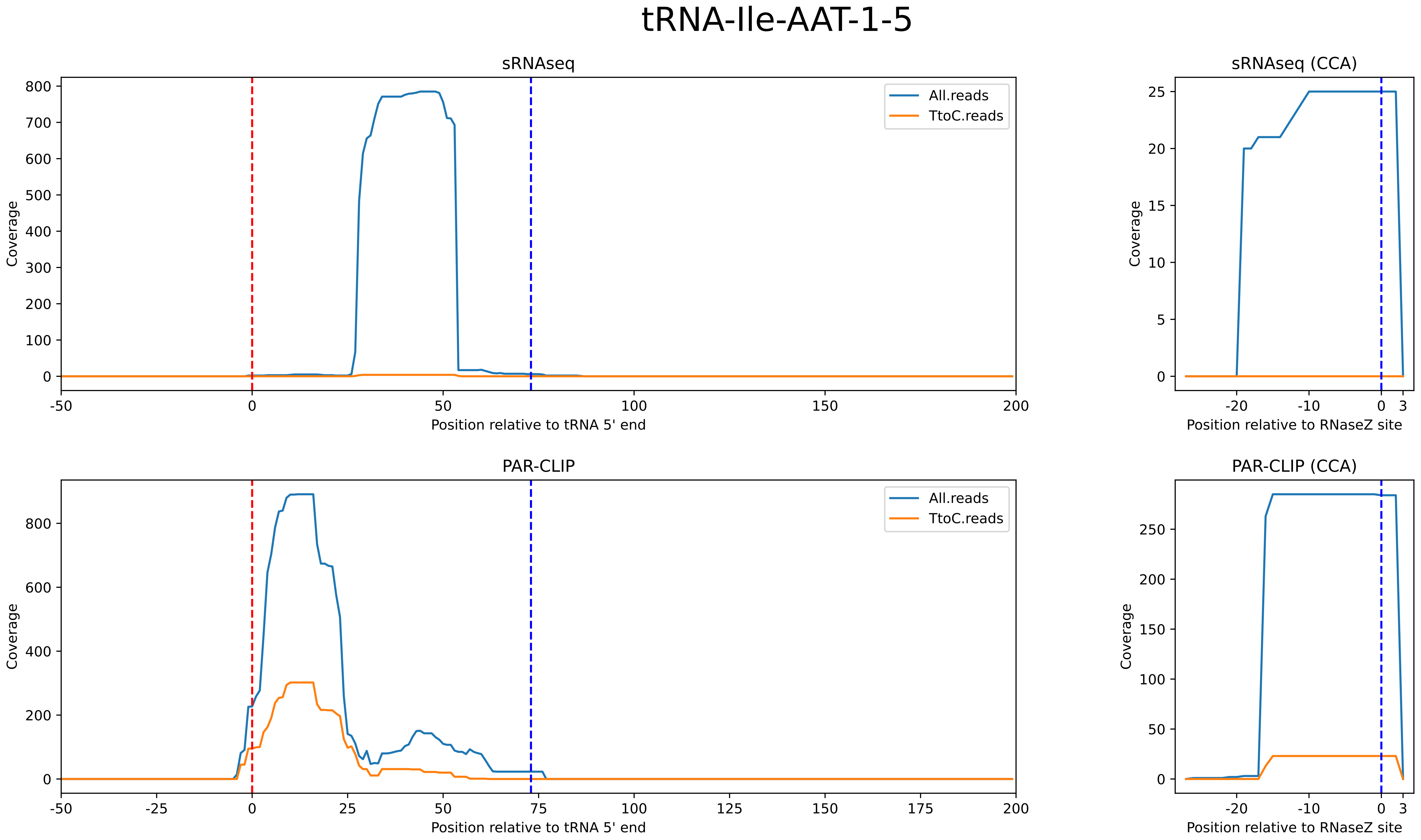

tRNA-Ile-AAT-1-5
sRNAseq
All.reads
TtoC.reads
Coverage
Position relative to tRNA 5' end
sRNAseq (CCA)
Position relative to RNaseZ site
PAR-CLIP
PAR-CLIP (CCA)

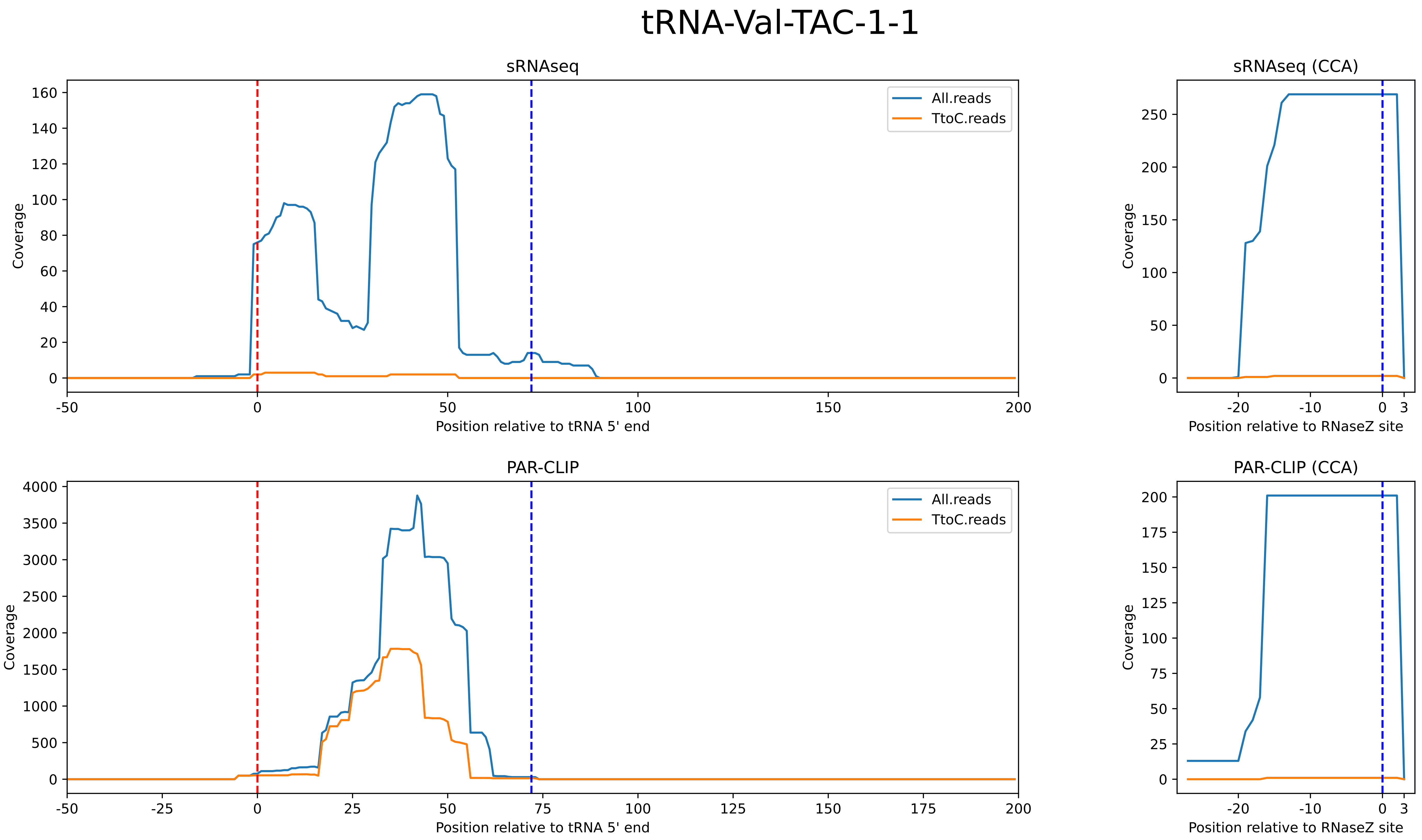

tRNA-Val-TAC-1-1
sRNAseq
All.reads
TtoC.reads
Coverage
Position relative to tRNA 5' end
sRNAseq (CCA)
Position relative to RNaseZ site
PAR-CLIP
PAR-CLIP (CCA)

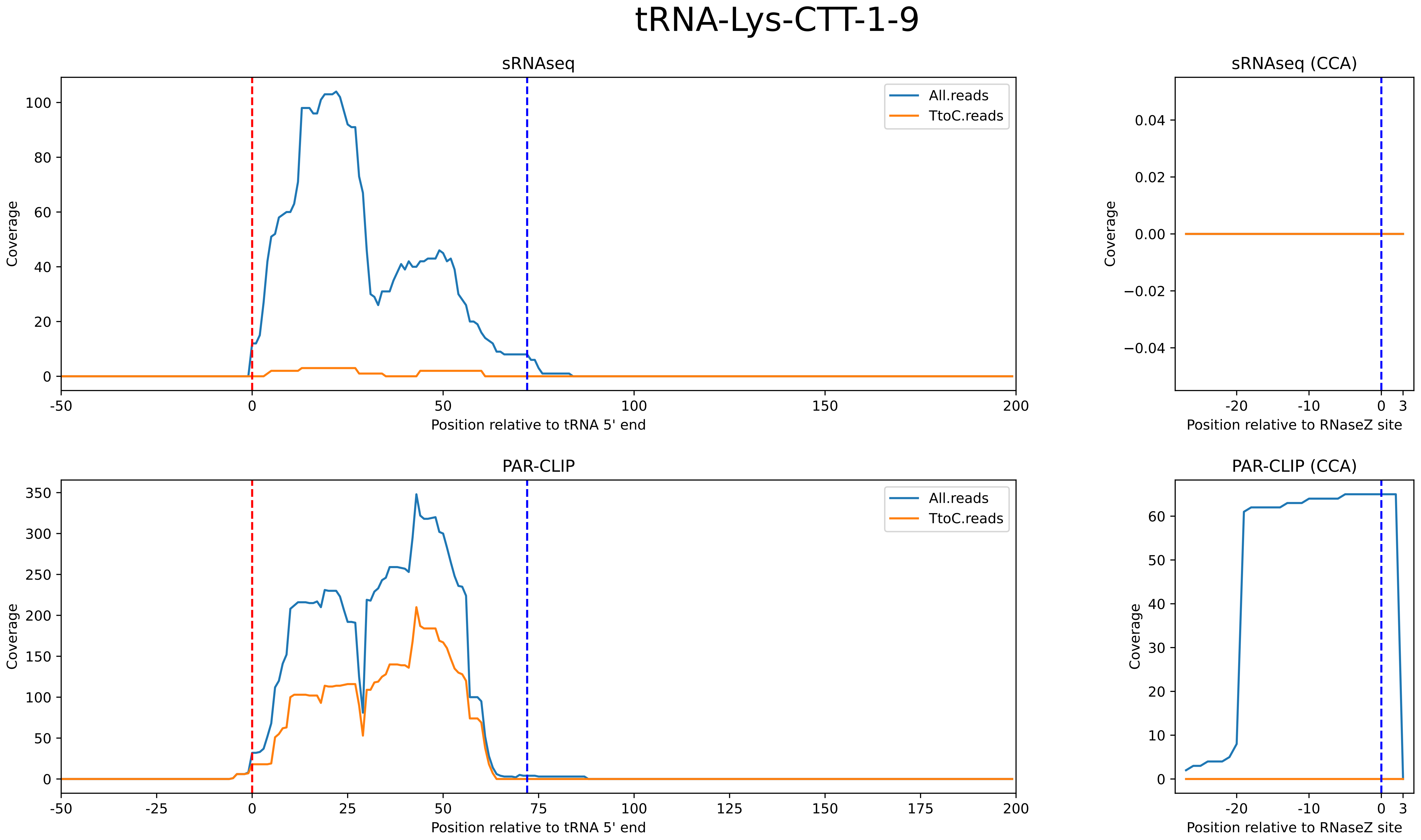

tRNA-Lys-CTT-1-9
sRNAseq
All.reads
TtoC.reads
Coverage
Position relative to tRNA 5' end
sRNAseq (CCA)
Position relative to RNaseZ site
PAR-CLIP
PAR-CLIP (CCA)

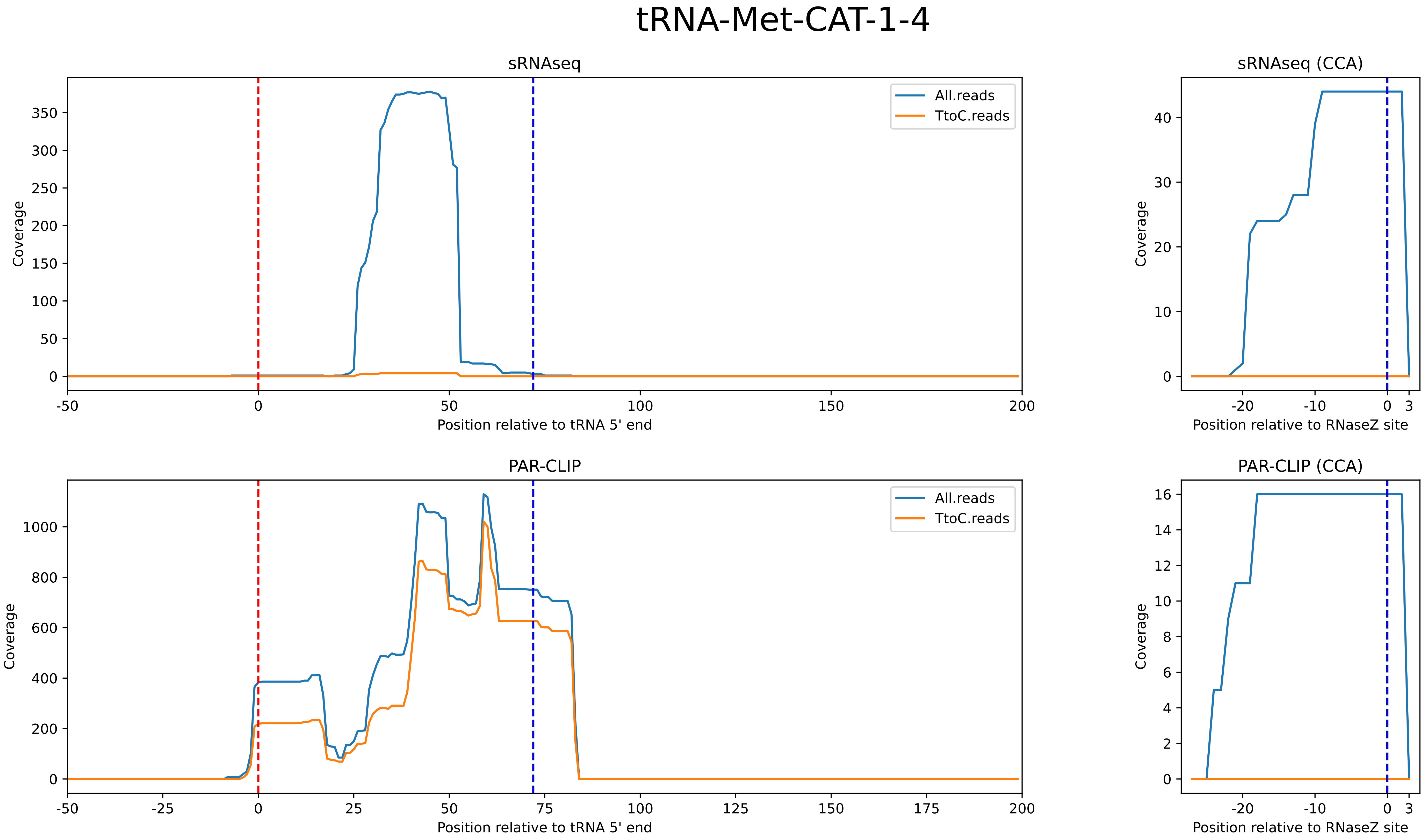

tRNA-Met-CAT-1-4
sRNAseq
All.reads
TtoC.reads
Coverage
Position relative to tRNA 5' end
sRNAseq (CCA)
Position relative to RNaseZ site
PAR-CLIP
PAR-CLIP (CCA)

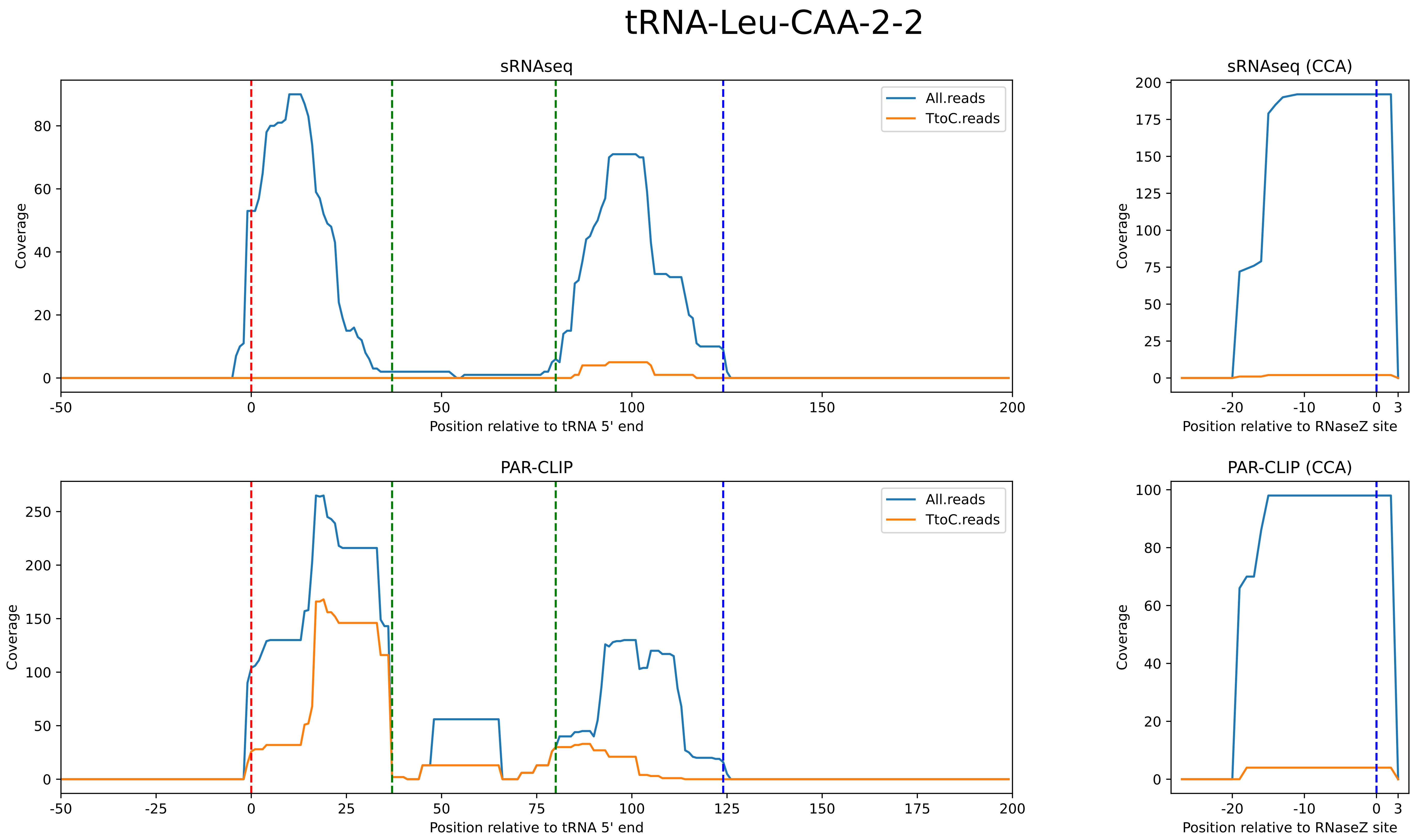

tRNA-Leu-CAA-2-2
sRNAseq
Coverage
Position relative to tRNA 5' end
All.reads
TtoC.reads
0
20
40
60
80
-50
0
50
100
150
200
sRNAseq (CCA)
Position relative to RNaseZ site
0
25
50
75
100
125
150
175
200
-20
-10
0
3
PAR-CLIP
0
50
100
150
200
250
-25
25
75
125
175
PAR-CLIP (CCA)
0
20
40
60
80
100

# tRNA-Gly-GCC-1-8

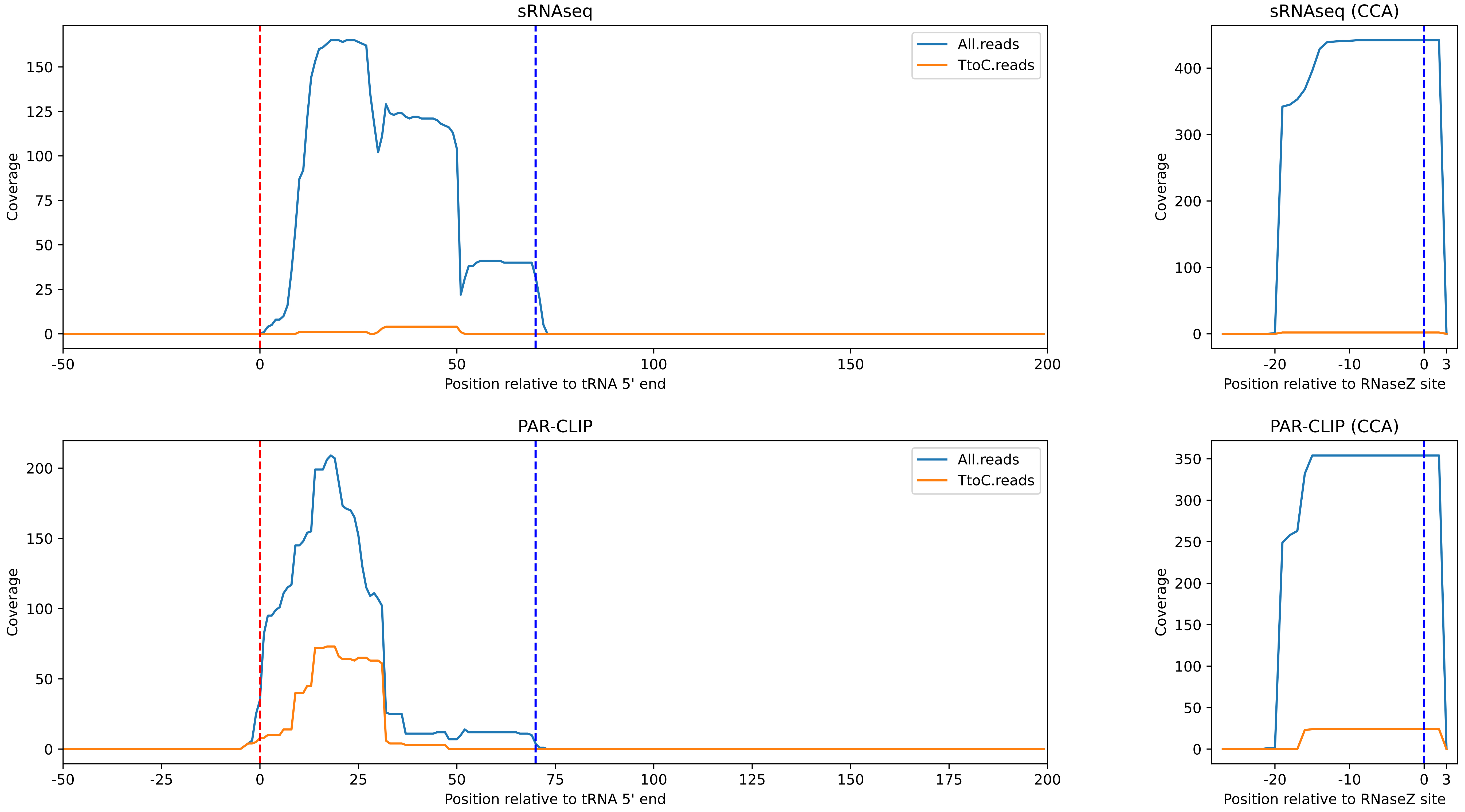

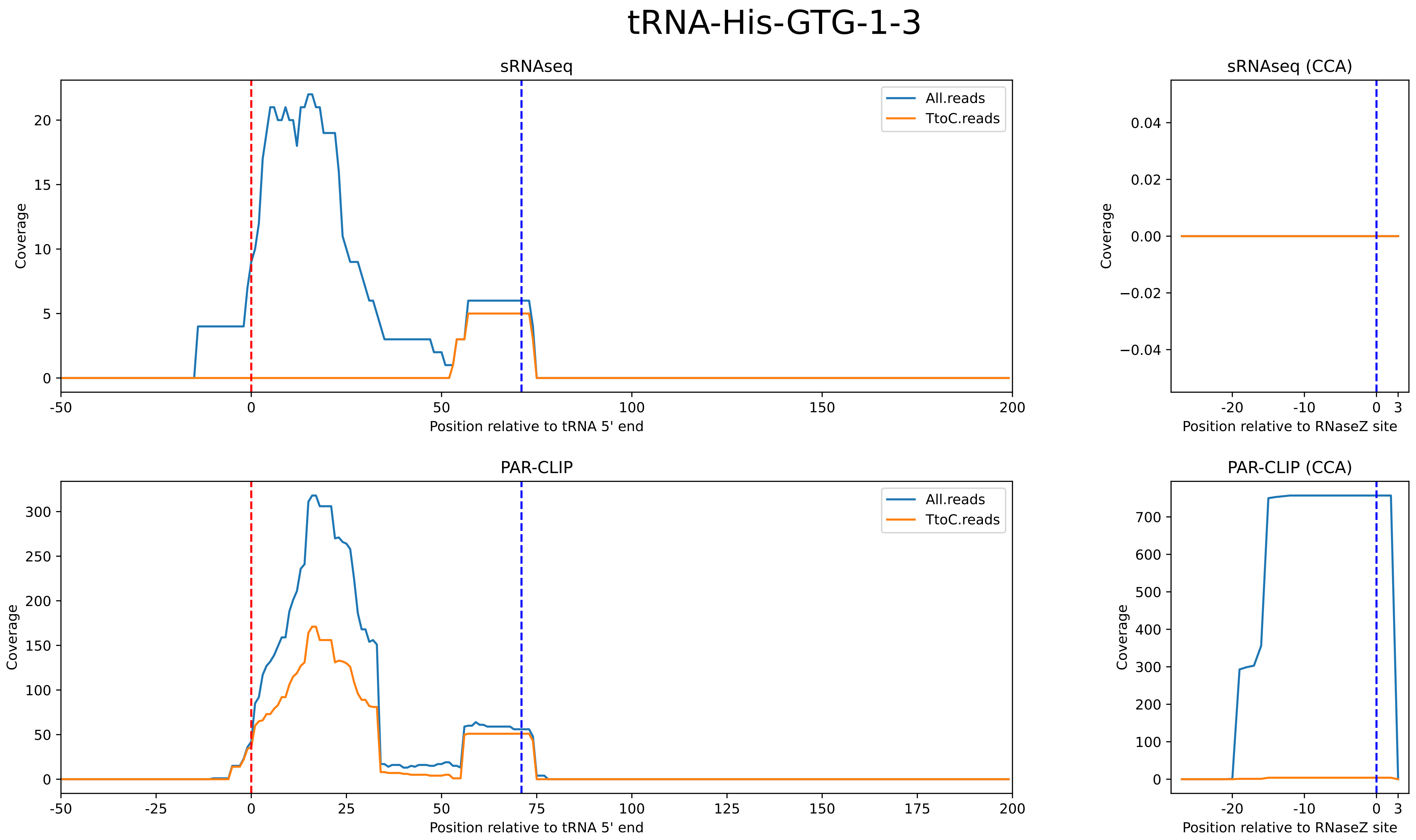

tRNA-His-GTG-1-3
sRNAseq
All.reads
TtoC.reads
Coverage
Position relative to tRNA 5' end
sRNAseq (CCA)
Position relative to RNaseZ site
PAR-CLIP
PAR-CLIP (CCA)

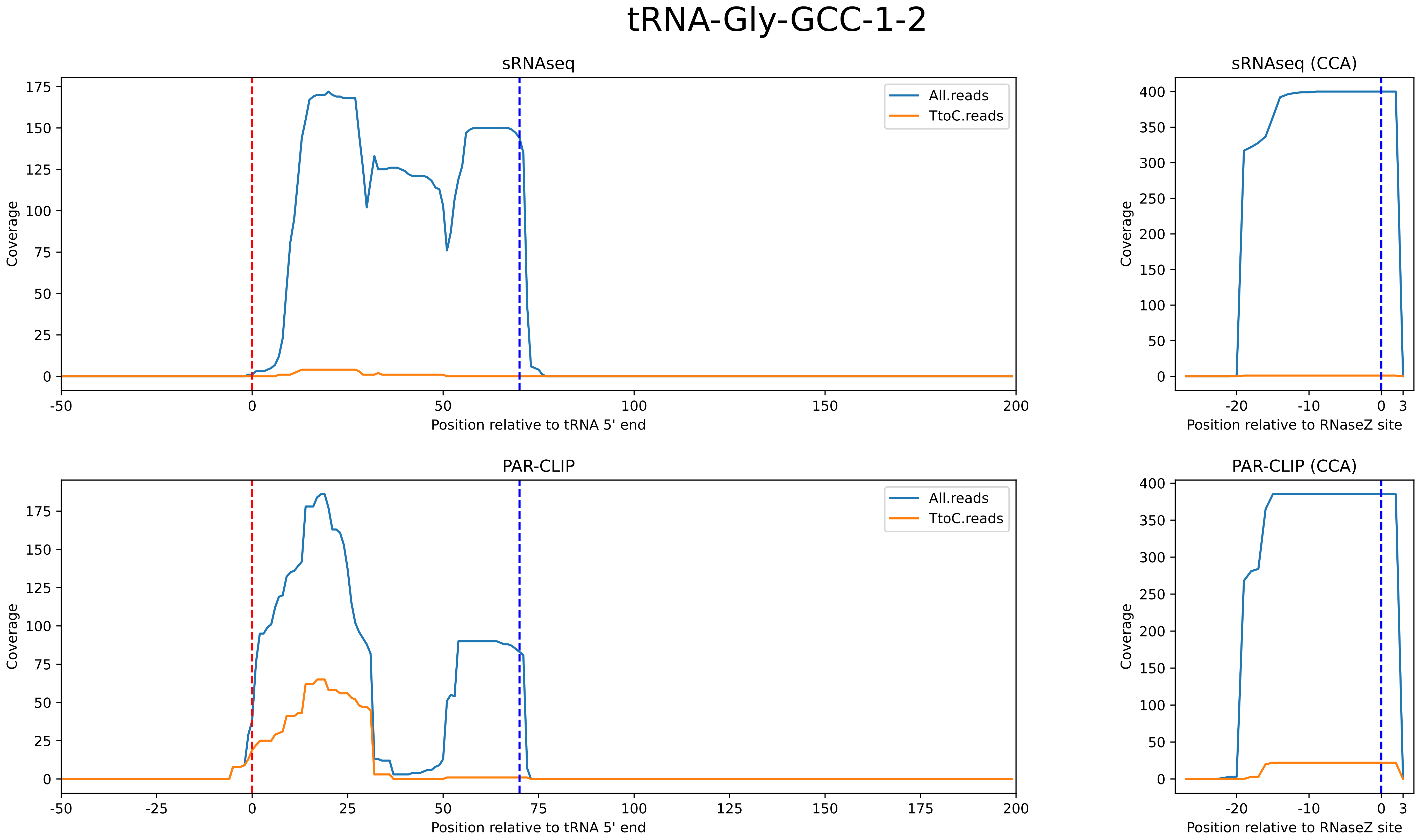

tRNA-Gly-GCC-1-2
sRNAseq
sRNAseq (CCA)
PAR-CLIP
PAR-CLIP (CCA)
All.reads
TtoC.reads
Coverage
Position relative to tRNA 5' end
Position relative to RNaseZ site

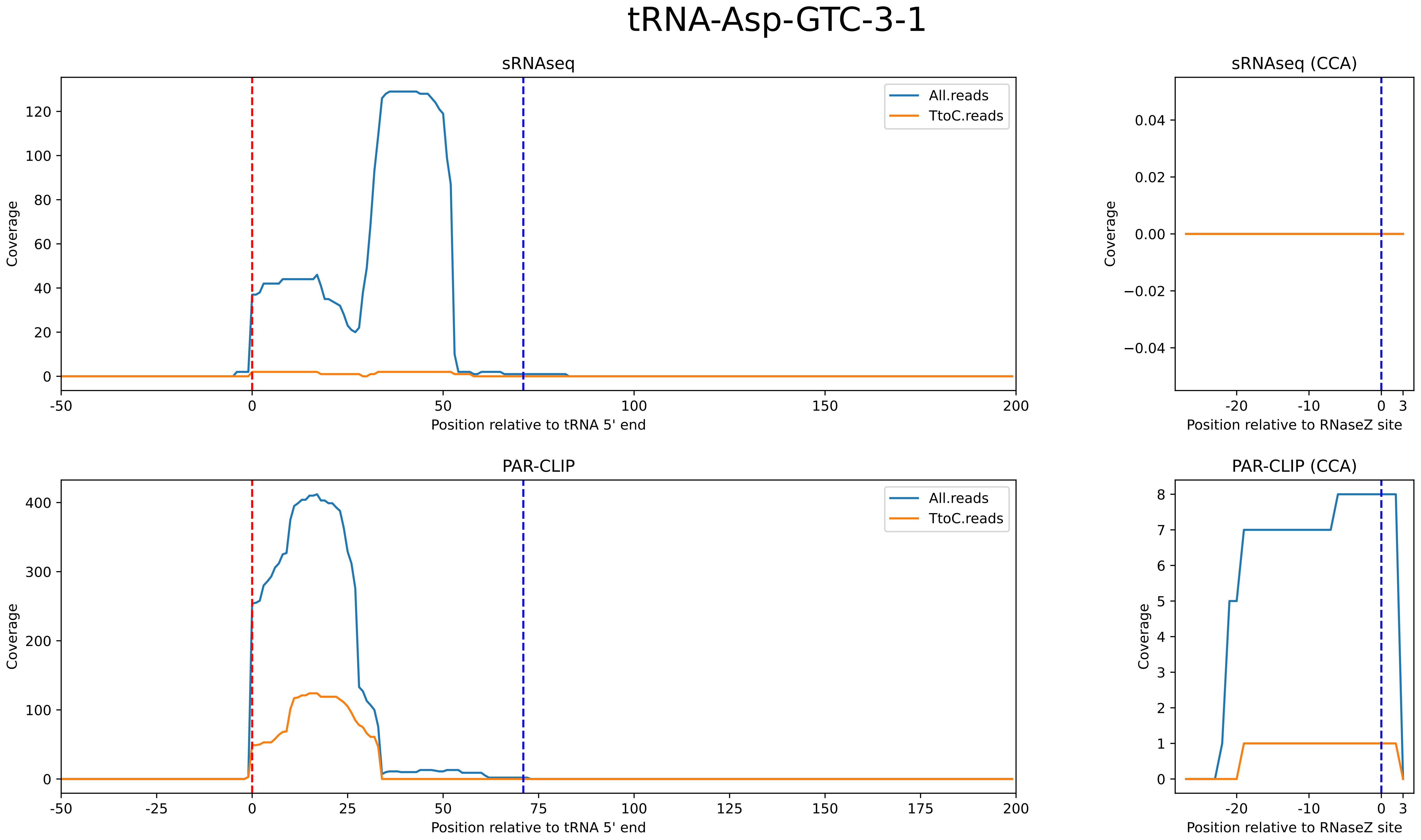

tRNA-Asp-GTC-3-1
sRNAseq
sRNAseq (CCA)
PAR-CLIP
PAR-CLIP (CCA)
All.reads
TtoC.reads
Coverage
Position relative to tRNA 5' end
Position relative to RNaseZ site

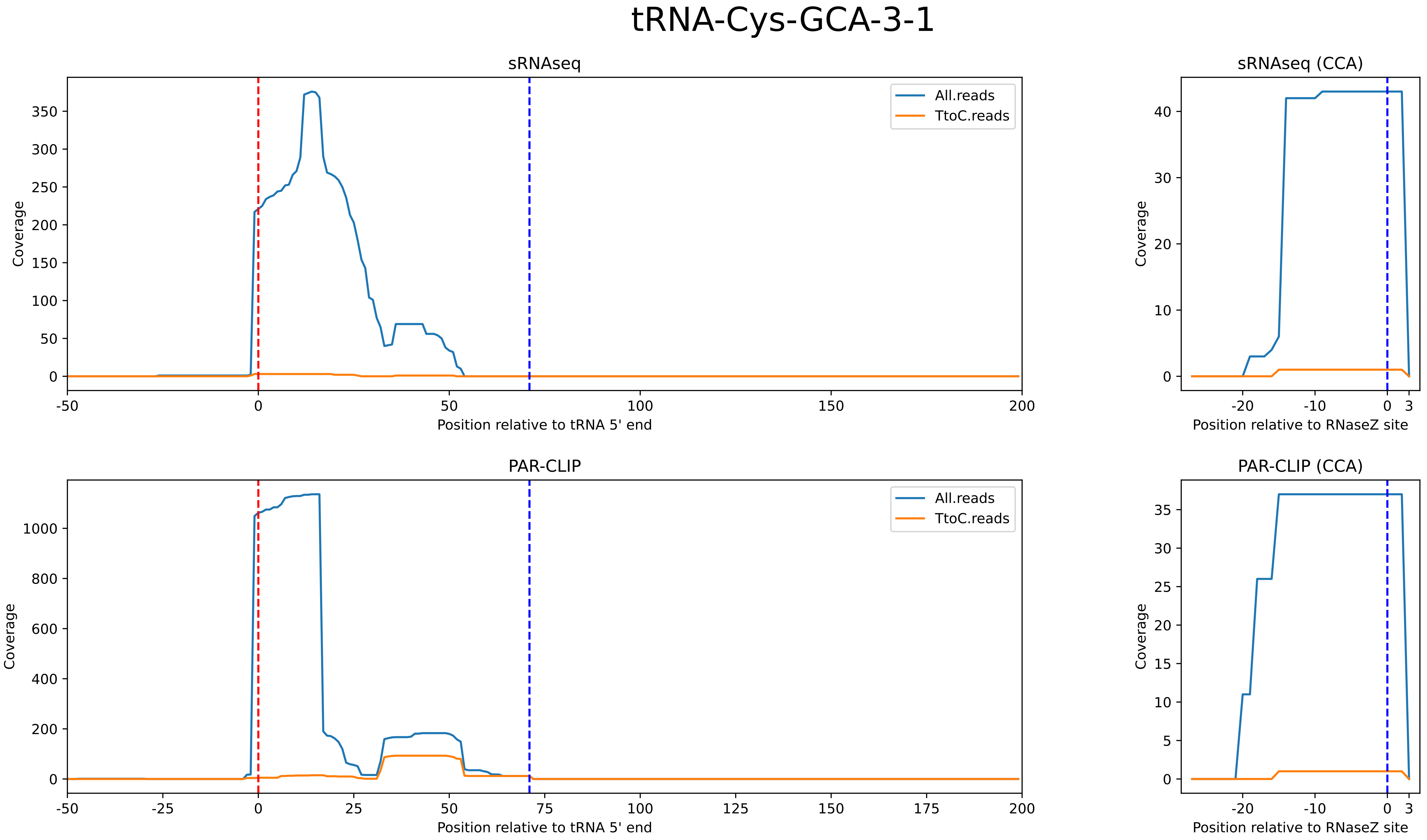

tRNA-Cys-GCA-3-1
sRNAseq
All.reads
TtoC.reads
Coverage
Position relative to tRNA 5' end
sRNAseq (CCA)
Position relative to RNaseZ site
PAR-CLIP
PAR-CLIP (CCA)

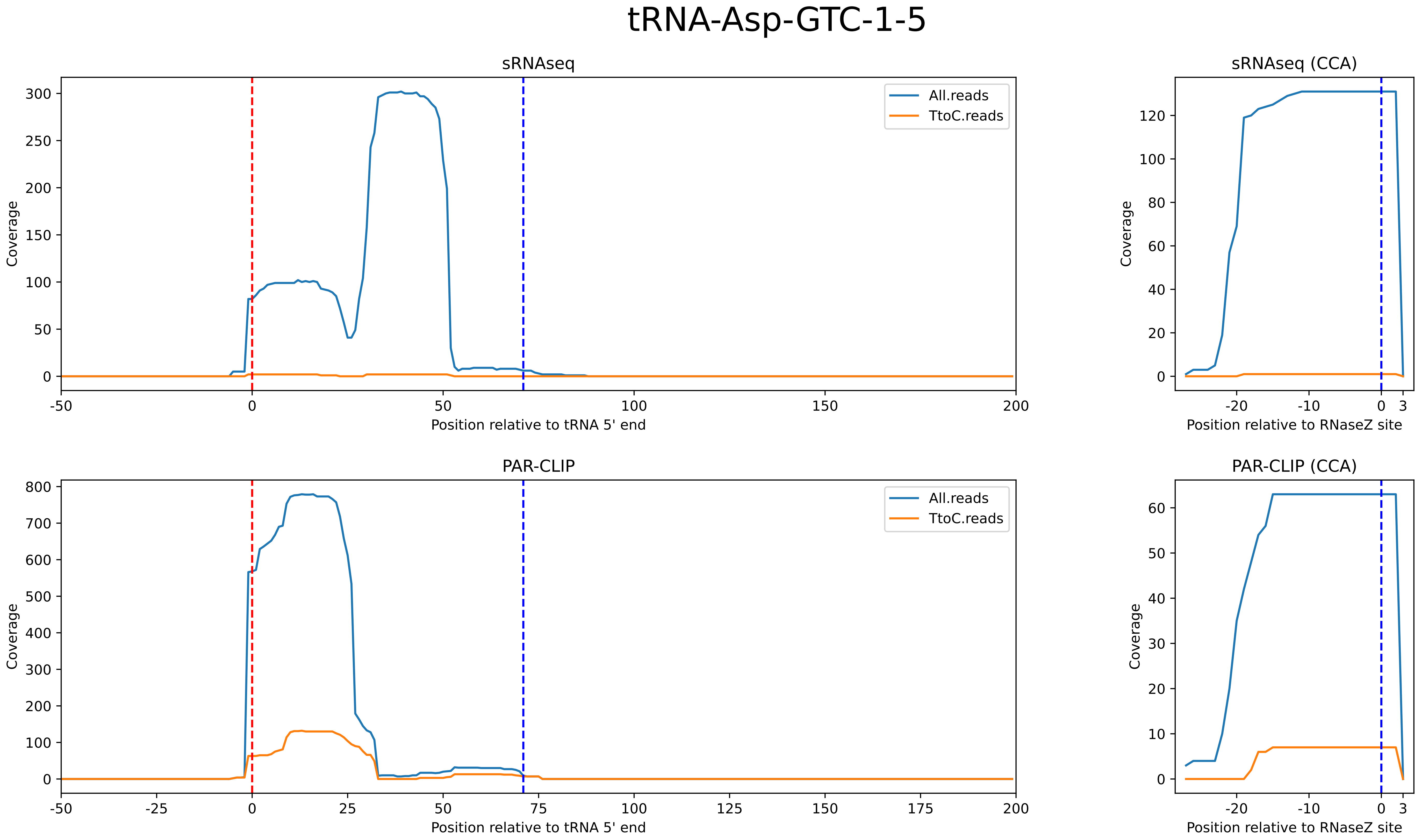
tRNA-Asp-GTC-1-5
sRNAseq
All.reads
TtoC.reads
Coverage
Position relative to tRNA 5' end
sRNAseq (CCA)
Position relative to RNaseZ site
PAR-CLIP
PAR-CLIP (CCA)

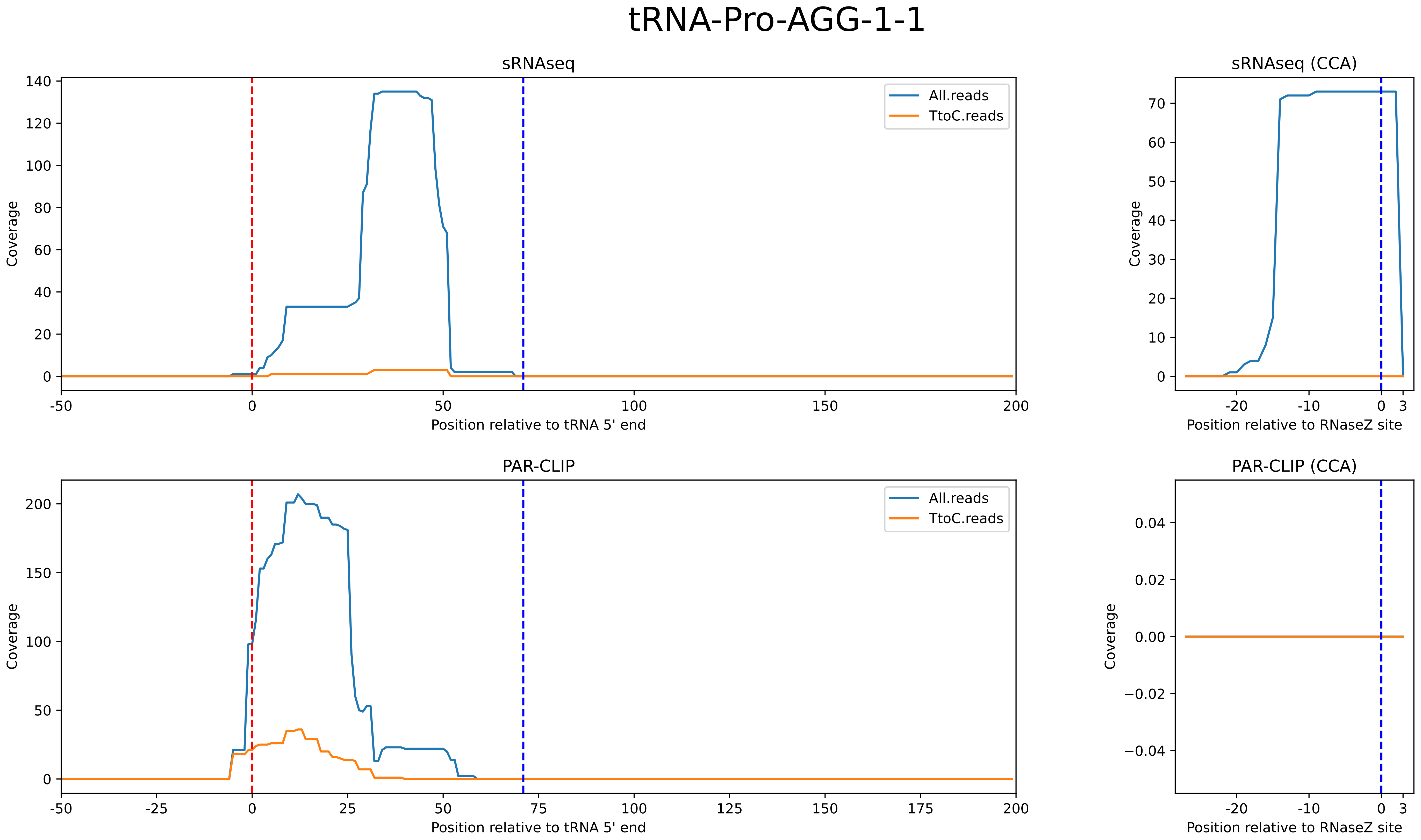

tRNA-Pro-AGG-1-1
sRNAseq
sRNAseq (CCA)
PAR-CLIP
PAR-CLIP (CCA)
All.reads
TtoC.reads
Coverage
Position relative to tRNA 5' end
Position relative to RNaseZ site

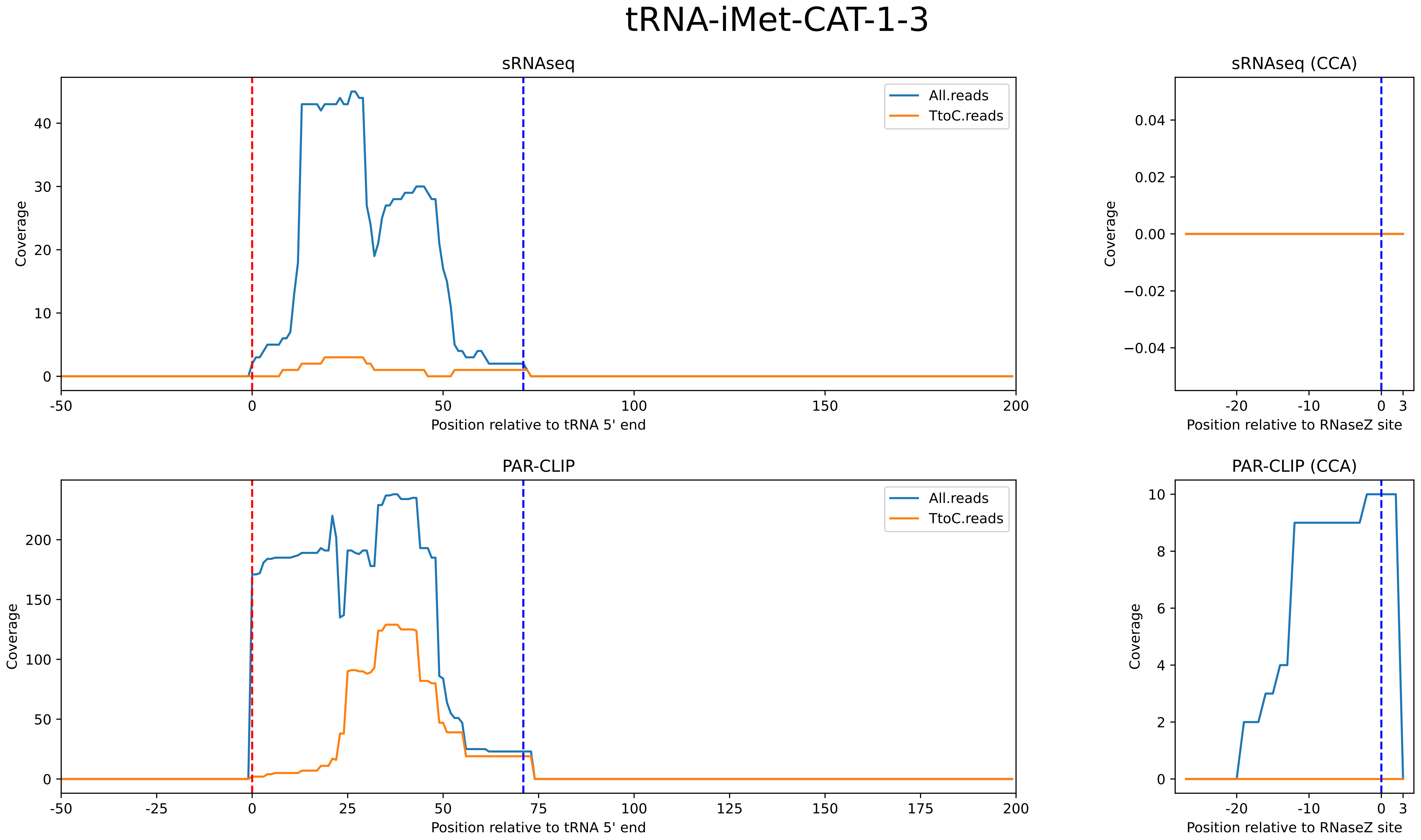

tRNA-iMet-CAT-1-3
sRNAseq
All.reads
TtoC.reads
Coverage
Position relative to tRNA 5' end
sRNAseq (CCA)
Position relative to RNaseZ site
PAR-CLIP
PAR-CLIP (CCA)

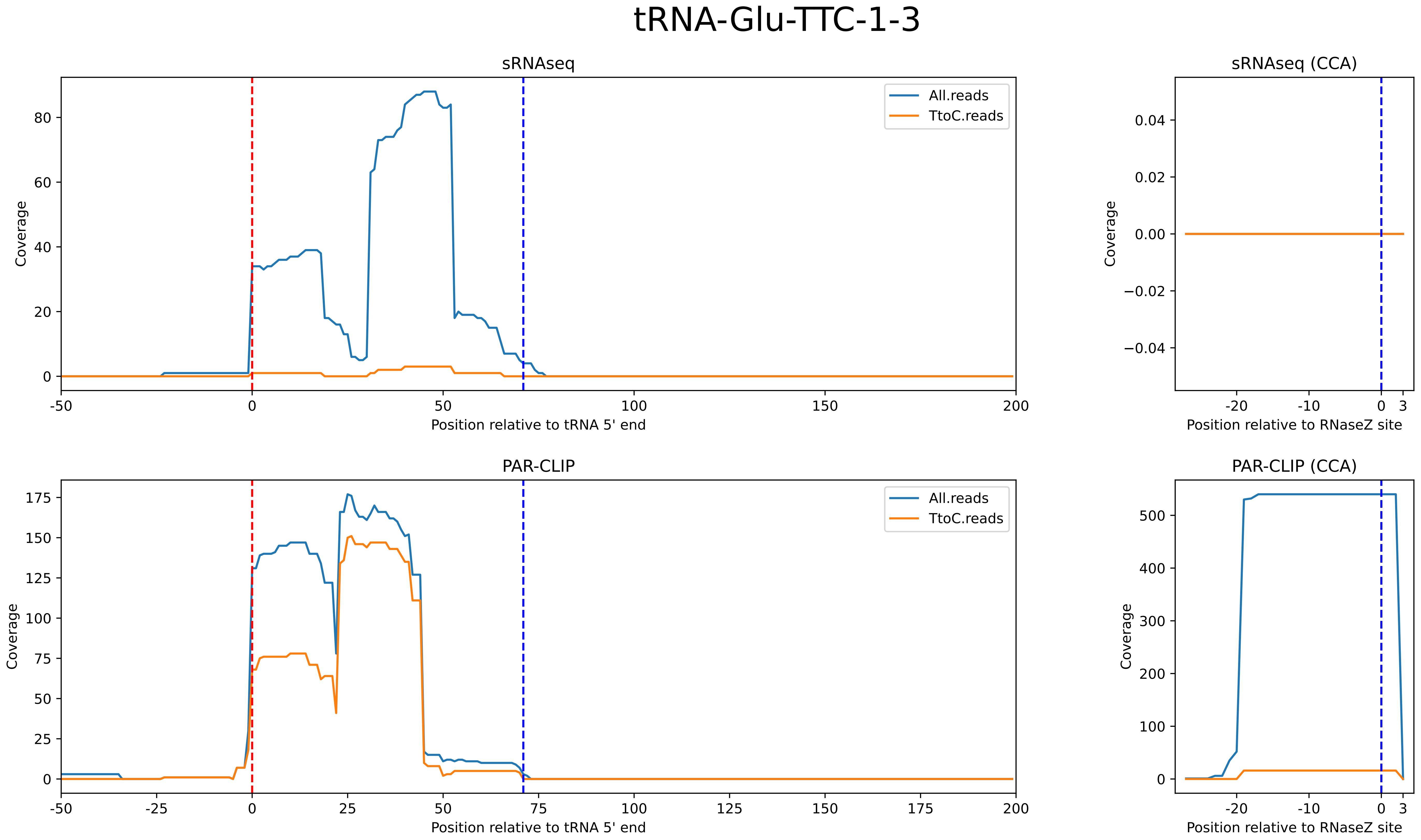

tRNA-Glu-TTC-1-3
sRNAseq
All.reads
TtoC.reads
Coverage
Position relative to tRNA 5' end
sRNAseq (CCA)
Position relative to RNaseZ site
PAR-CLIP
PAR-CLIP (CCA)

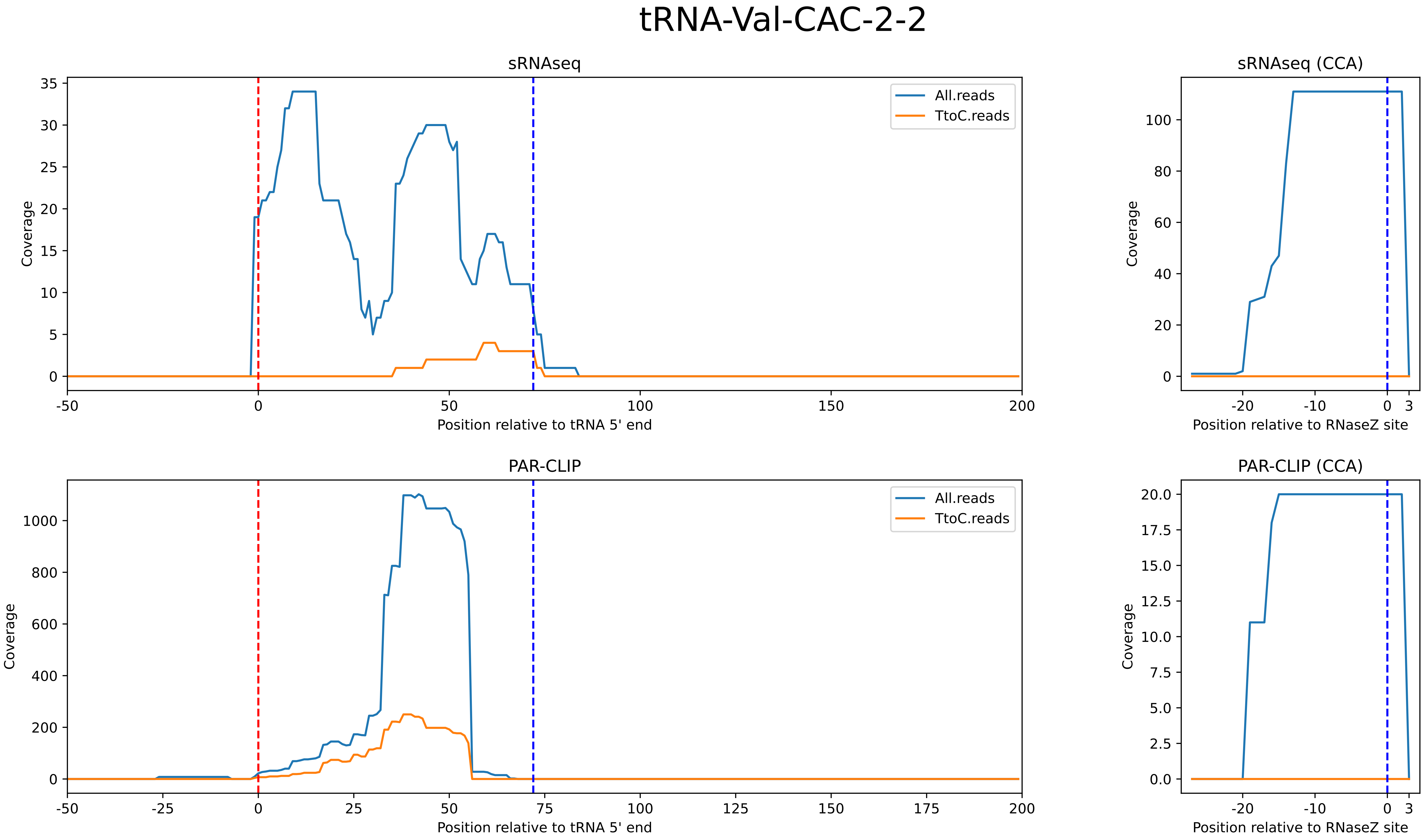

tRNA-Val-CAC-2-2
sRNAseq
sRNAseq (CCA)
PAR-CLIP
PAR-CLIP (CCA)
All.reads
TtoC.reads
Coverage
Position relative to tRNA 5' end
Position relative to RNaseZ site

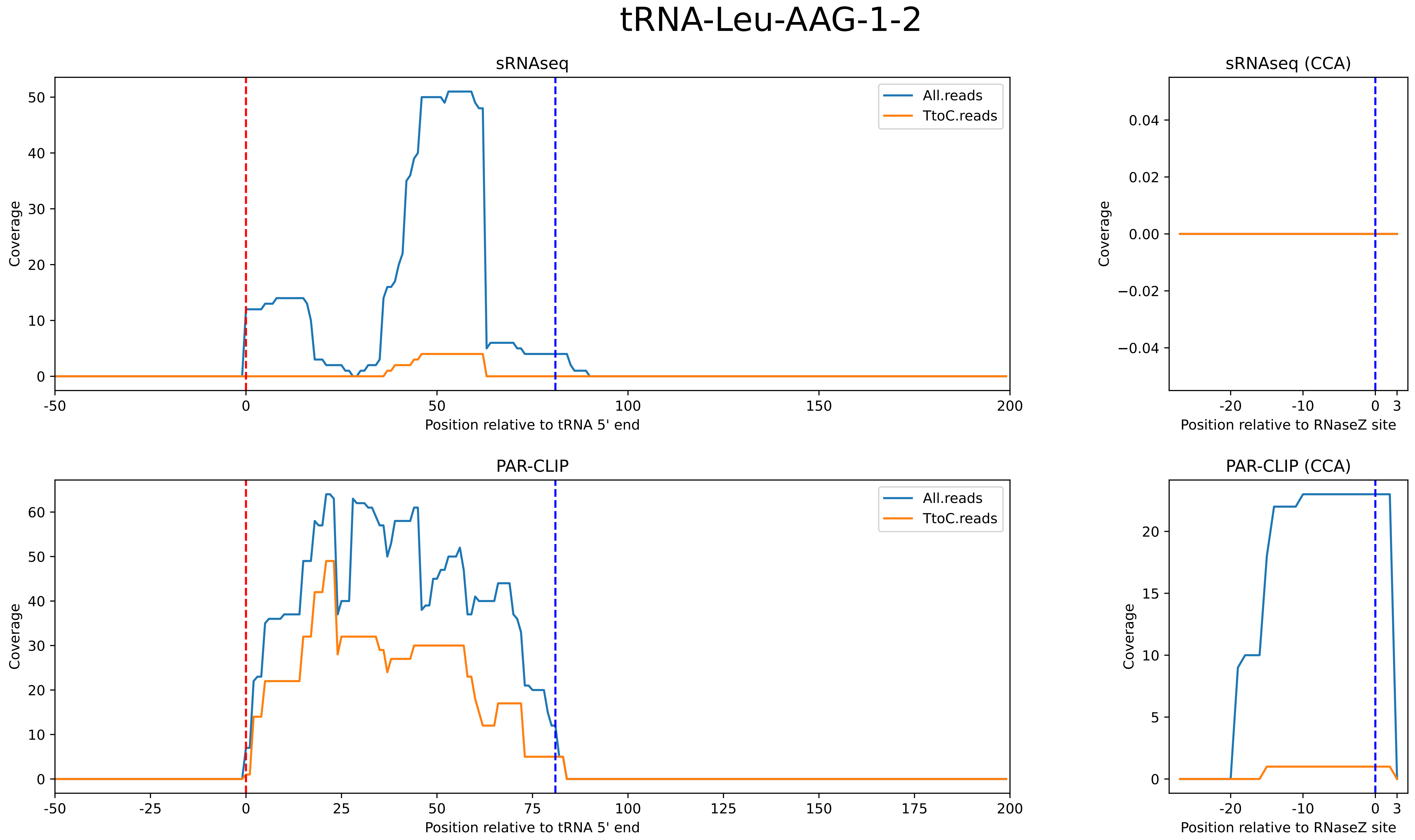

tRNA-Leu-AAG-1-2
sRNAseq
sRNAseq (CCA)
PAR-CLIP
PAR-CLIP (CCA)
All.reads
TtoC.reads
Coverage
Position relative to tRNA 5' end
Position relative to RNaseZ site

# tRNA-Leu-AAG-1-3

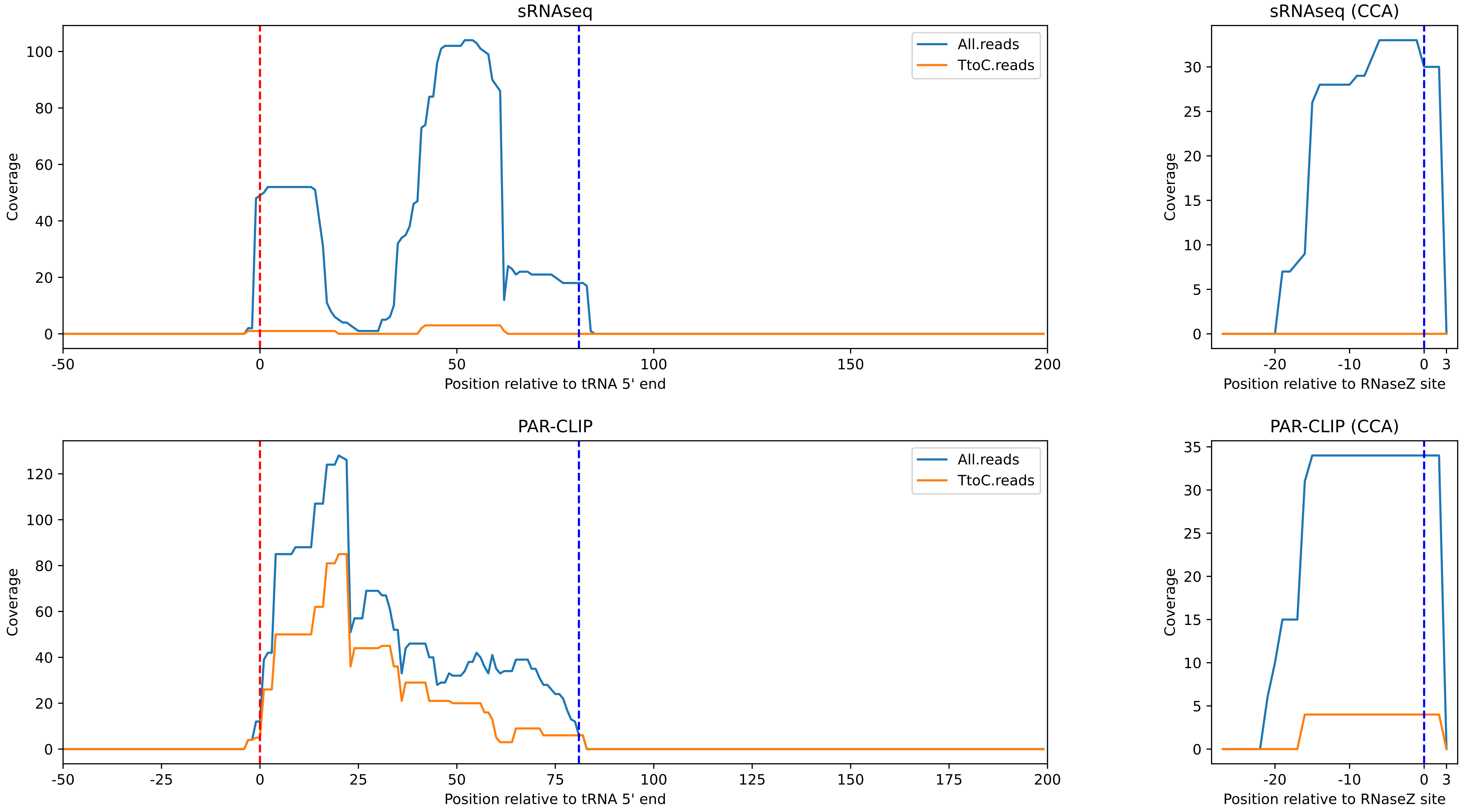

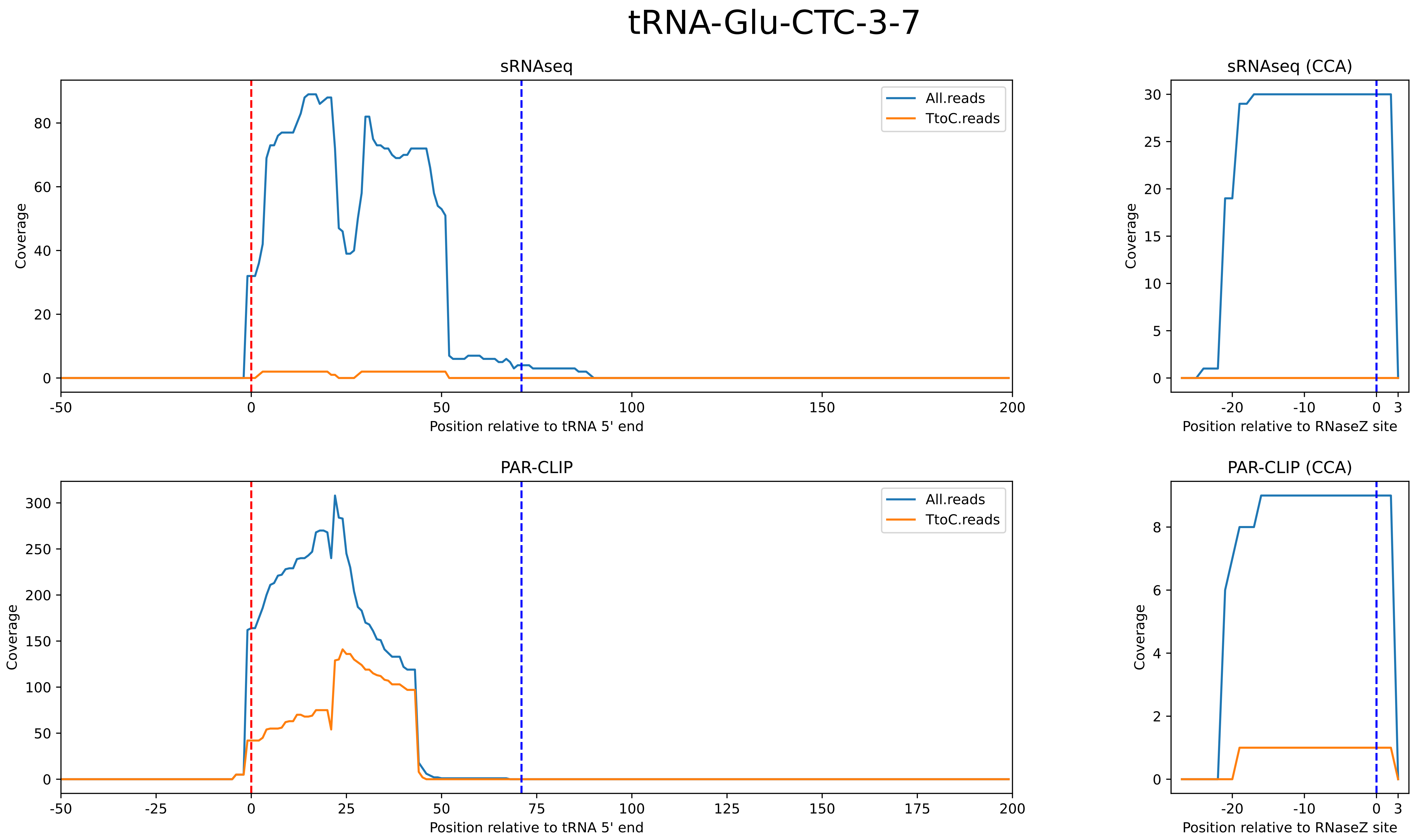
tRNA-Glu-CTC-3-7
sRNAseq
sRNAseq (CCA)
PAR-CLIP
PAR-CLIP (CCA)
All.reads
TtoC.reads
Coverage
Position relative to tRNA 5' end
Position relative to RNaseZ site

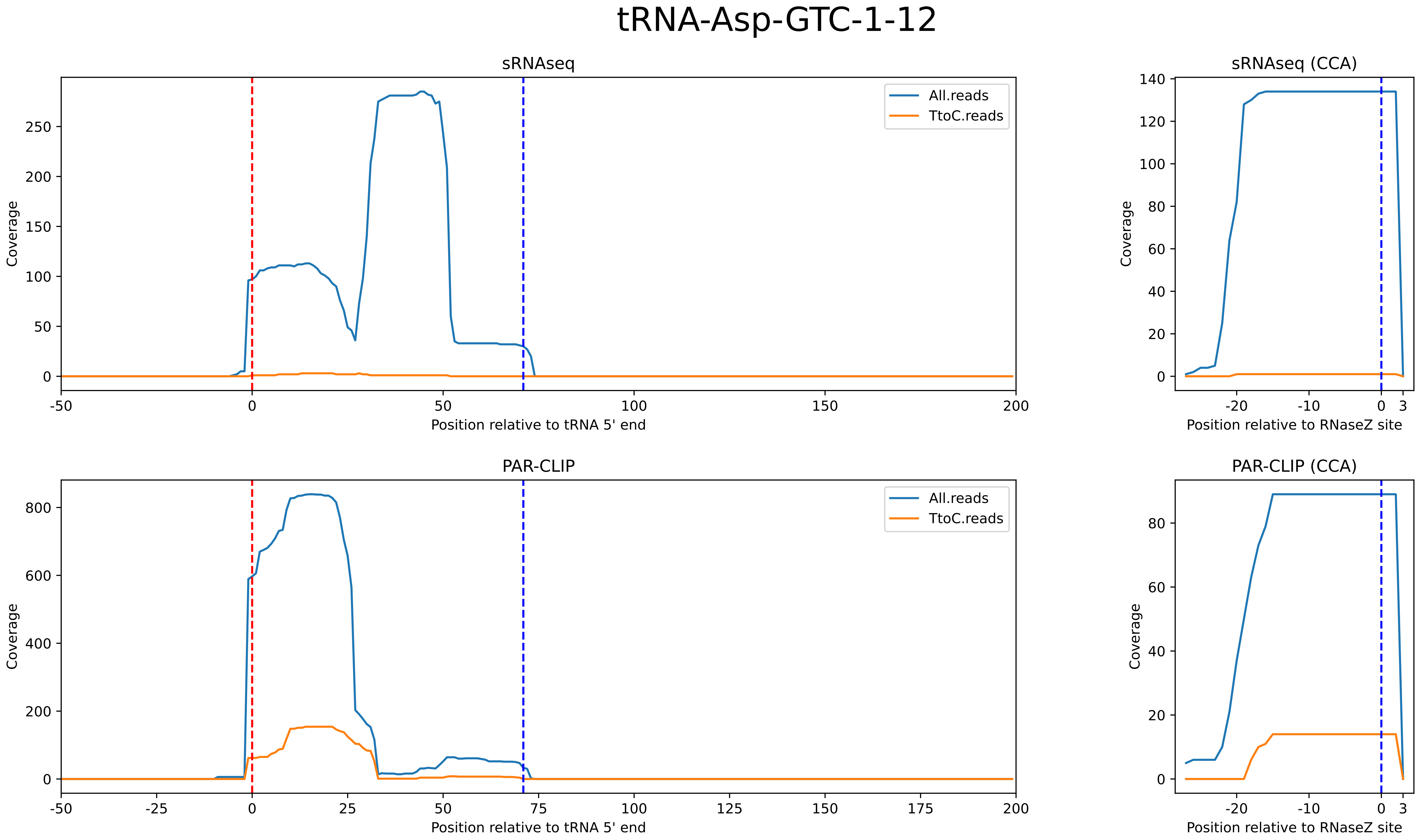

tRNA-Asp-GTC-1-12
sRNAseq
All.reads
TtoC.reads
Coverage
Position relative to tRNA 5' end
sRNAseq (CCA)
Position relative to RNaseZ site
PAR-CLIP
PAR-CLIP (CCA)

# tRNA-Thr-AGT-1-1

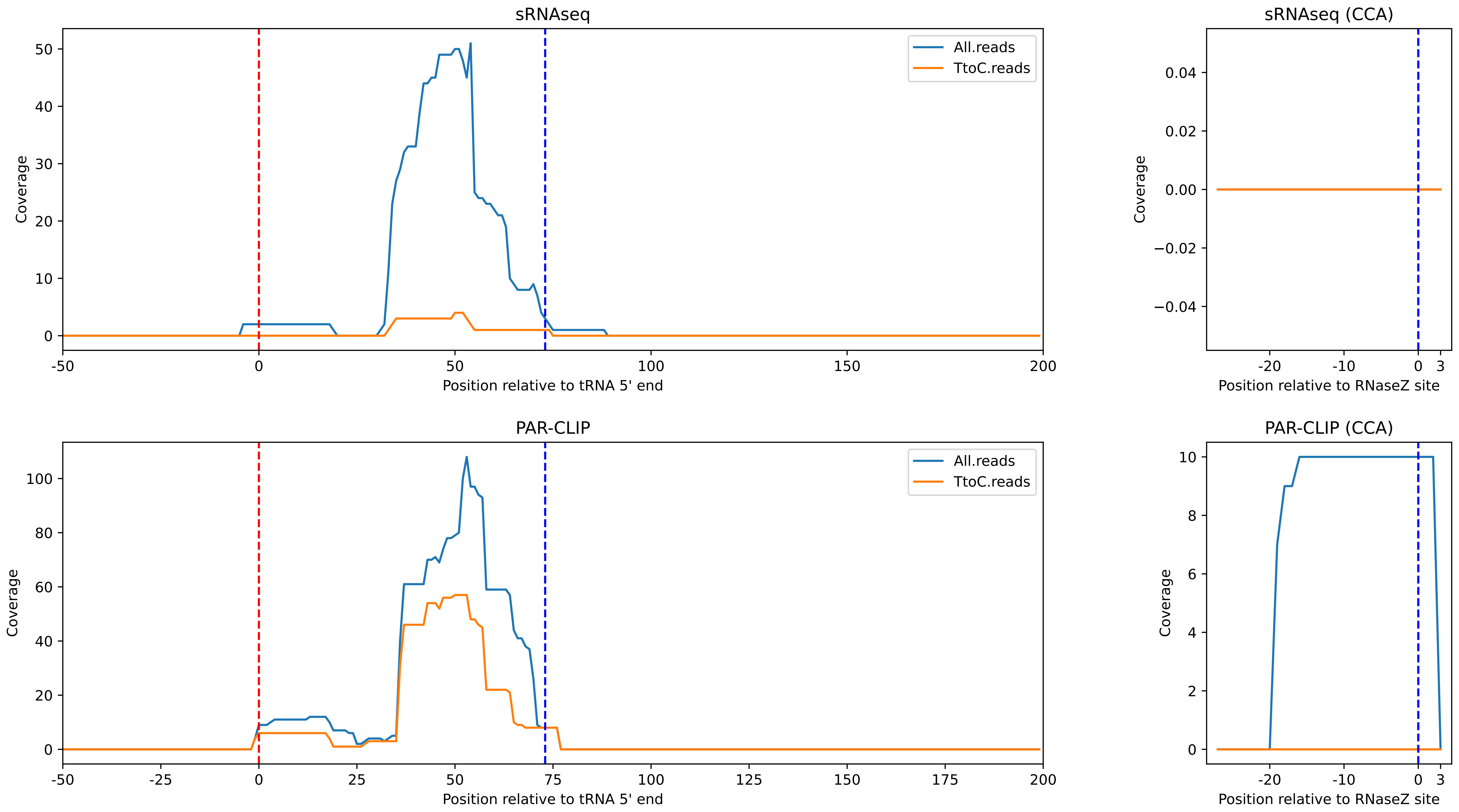

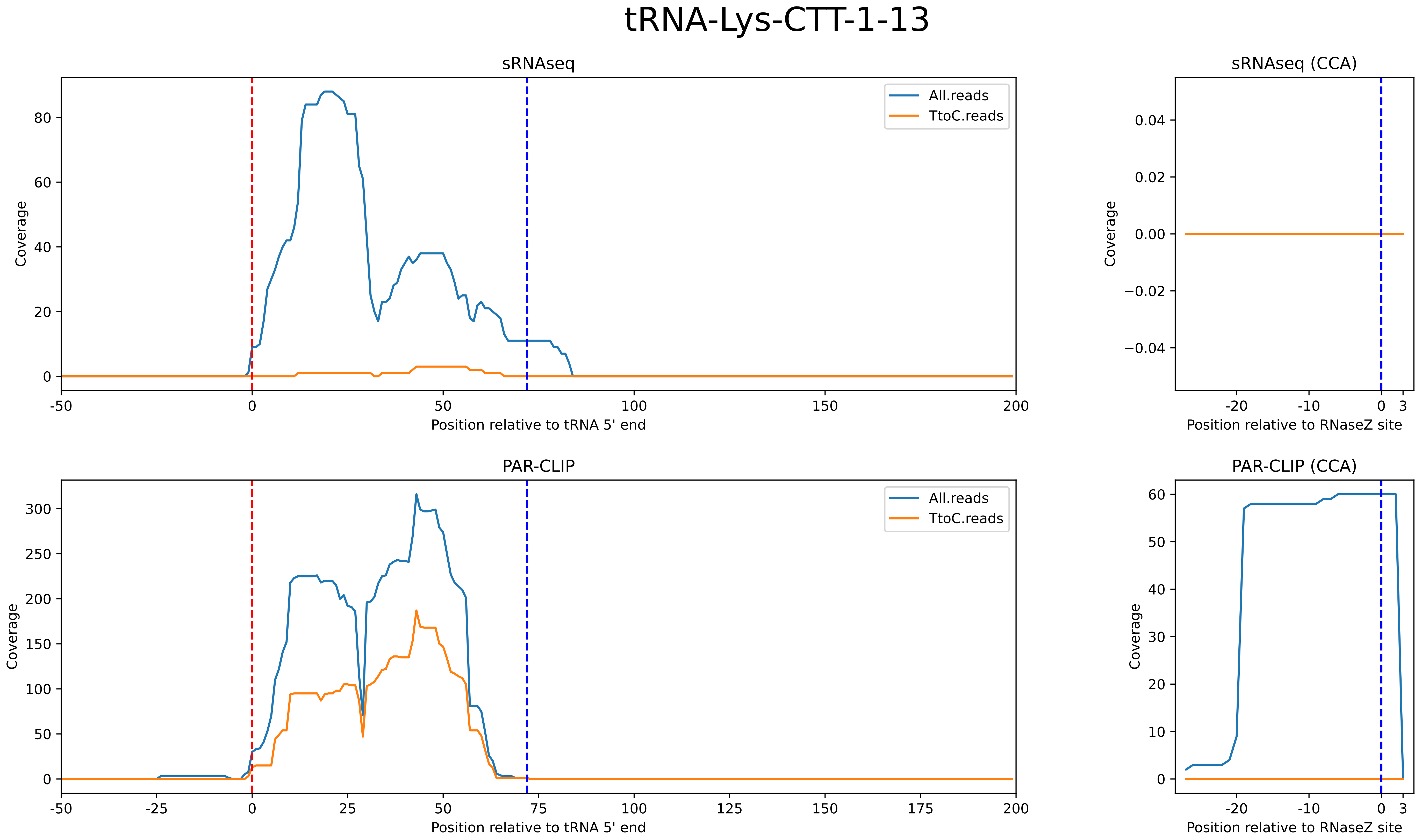

tRNA-Lys-CTT-1-13
sRNAseq
Coverage
0
20
40
60
80
-50
0
50
100
150
200
Position relative to tRNA 5' end
All.reads
TtoC.reads
sRNAseq (CCA)
Coverage
0.04
0.02
0.00
−0.02
−0.04
-20
-10
0
3
Position relative to RNaseZ site
PAR-CLIP
Coverage
0
50
100
150
200
250
300
-50
-25
0
25
50
75
100
125
150
175
200
Position relative to tRNA 5' end
All.reads
TtoC.reads
PAR-CLIP (CCA)
Coverage
0
10
20
30
40
50
60
-20
-10
0
3
Position relative to RNaseZ site

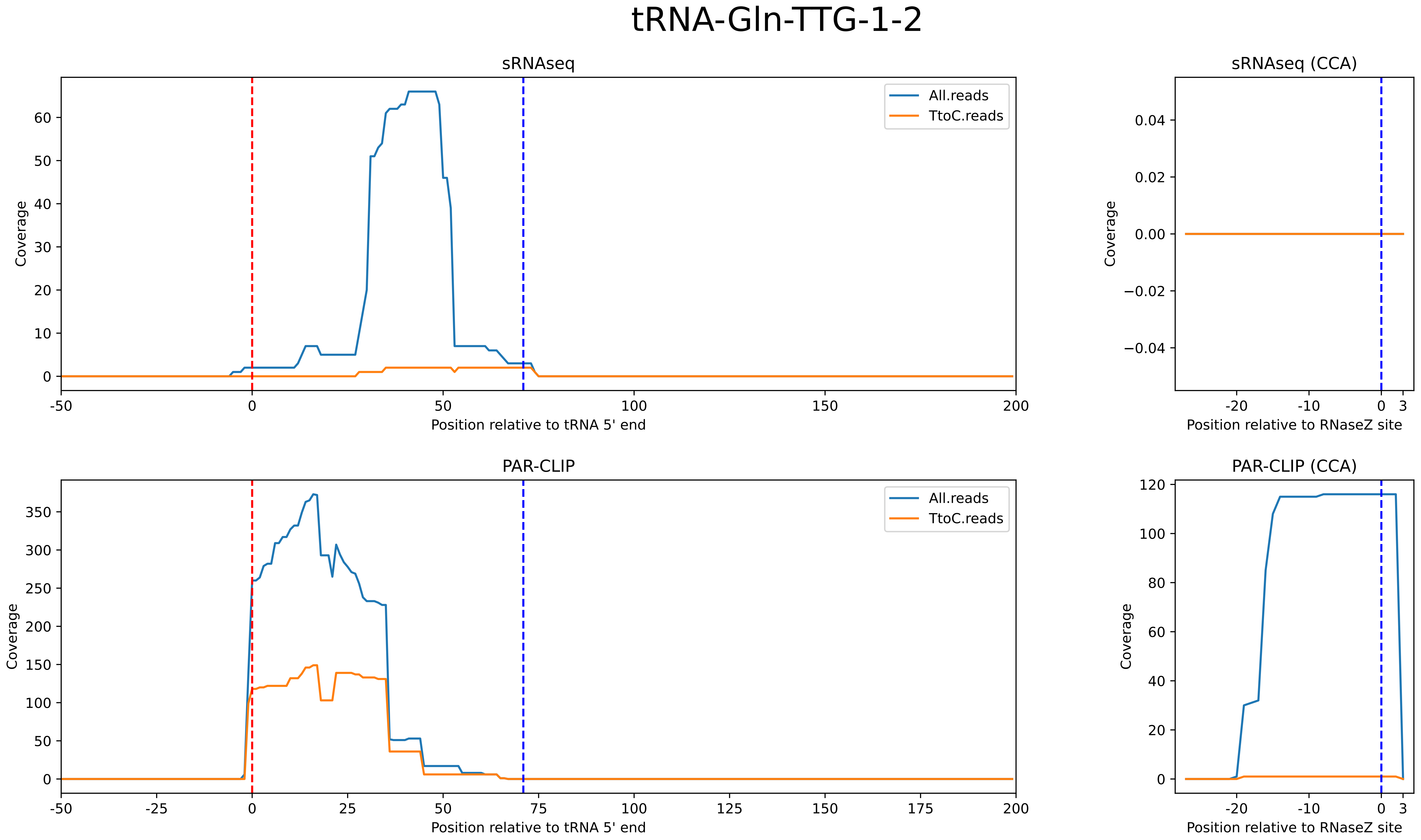

tRNA-Gln-TTG-1-2
sRNAseq
All.reads
TtoC.reads
Coverage
Position relative to tRNA 5' end
sRNAseq (CCA)
Position relative to RNaseZ site
PAR-CLIP
PAR-CLIP (CCA)

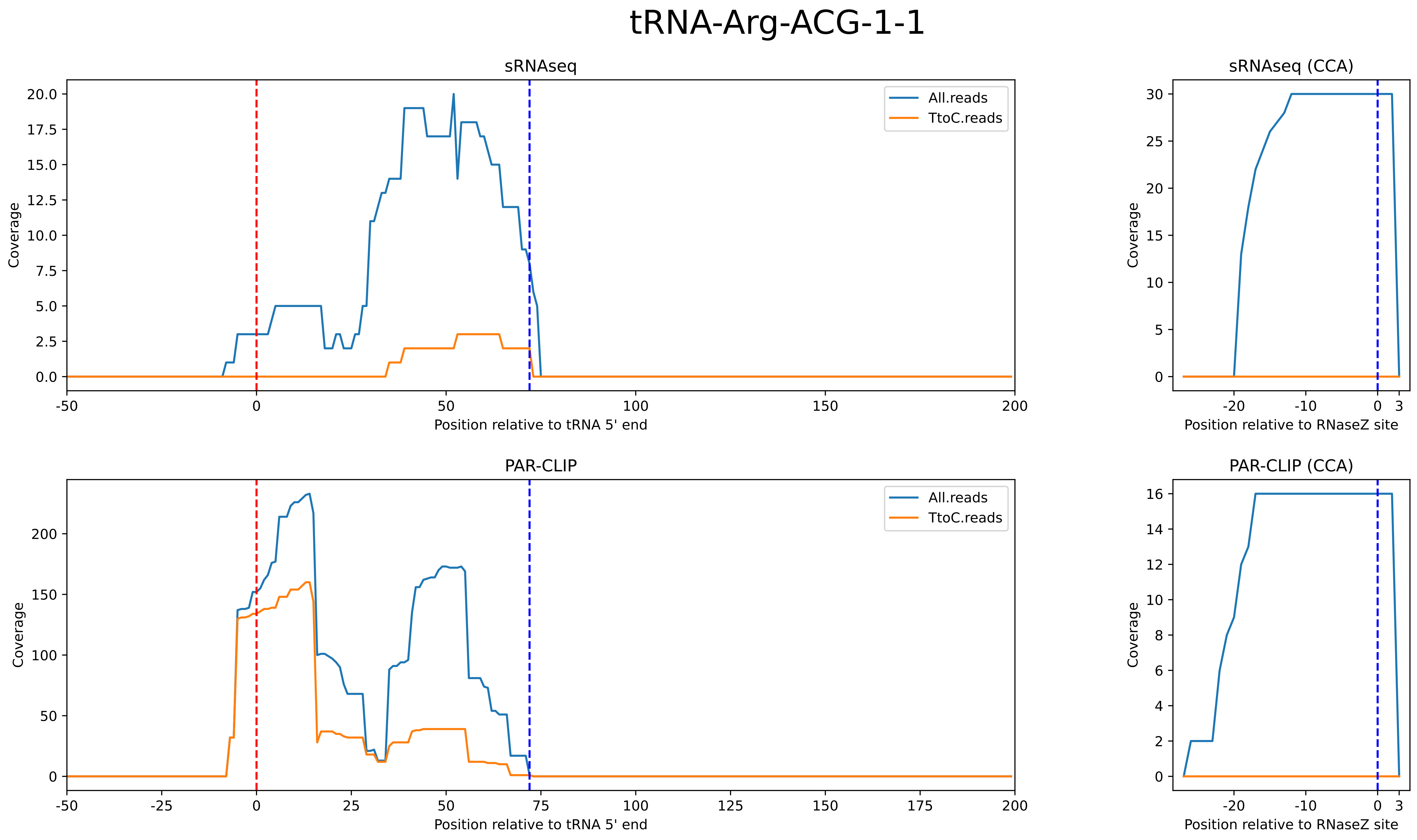

tRNA-Arg-ACG-1-1
sRNAseq
sRNAseq (CCA)
PAR-CLIP
PAR-CLIP (CCA)
All.reads
TtoC.reads
Coverage
Position relative to tRNA 5' end
Position relative to RNaseZ site

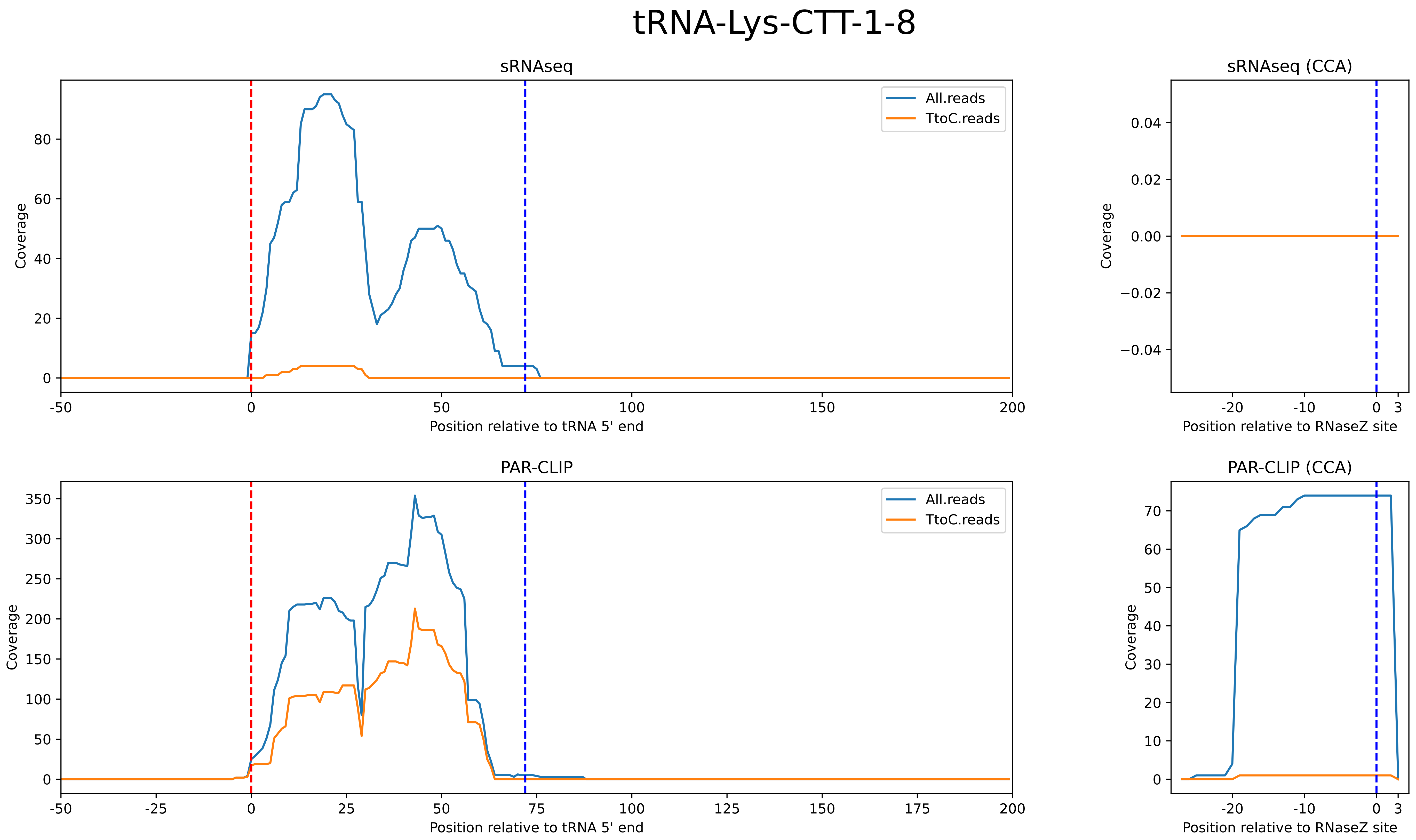

tRNA-Lys-CTT-1-8
sRNAseq
All.reads
TtoC.reads
Coverage
Position relative to tRNA 5' end
sRNAseq (CCA)
Position relative to RNaseZ site
PAR-CLIP
All.reads
TtoC.reads
Coverage
Position relative to tRNA 5' end
PAR-CLIP (CCA)
Coverage
Position relative to RNaseZ site

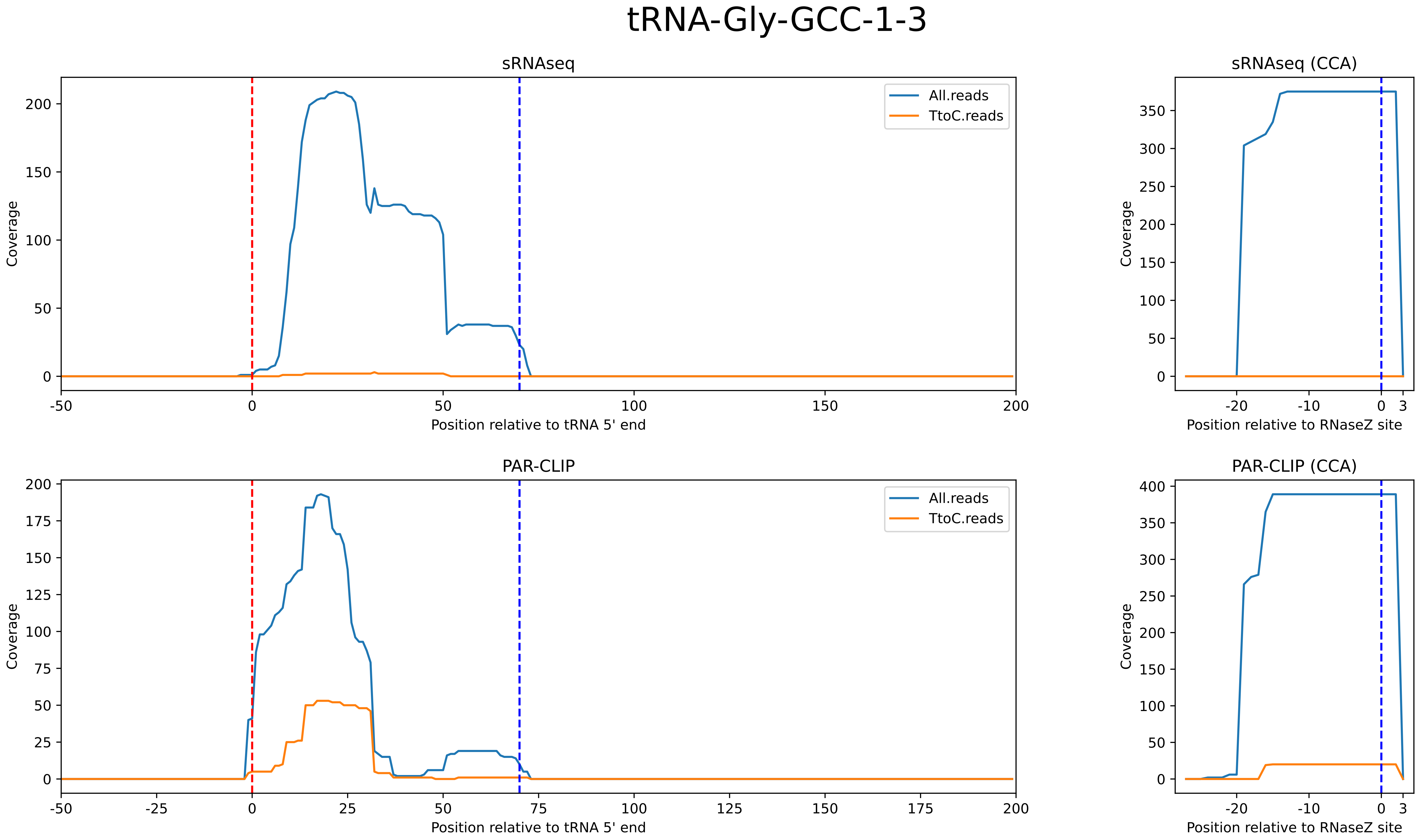
tRNA-Gly-GCC-1-3
sRNAseq
All.reads
TtoC.reads
Coverage
Position relative to tRNA 5' end
sRNAseq (CCA)
Position relative to RNaseZ site
PAR-CLIP
PAR-CLIP (CCA)

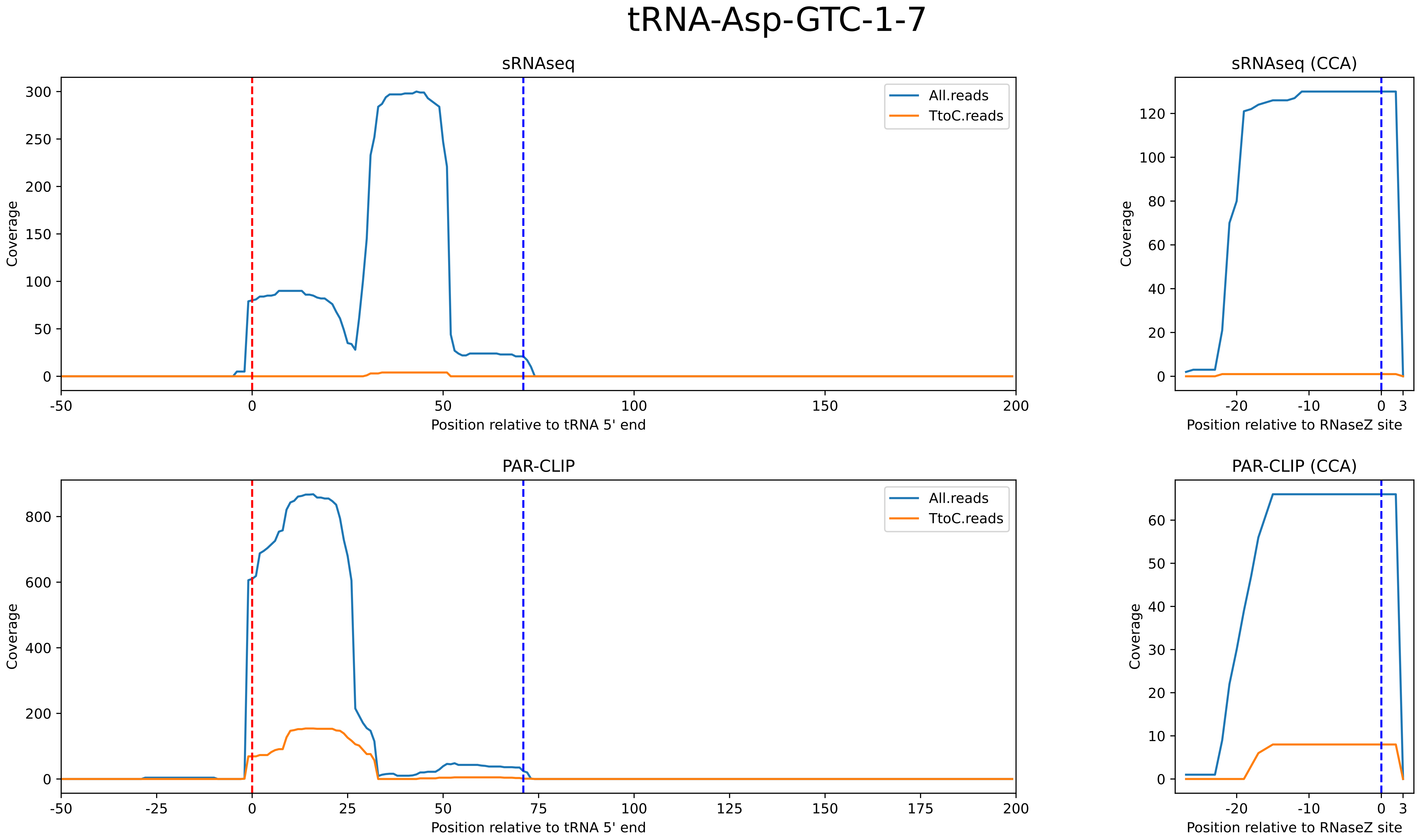
tRNA-Asp-GTC-1-7
sRNAseq
sRNAseq (CCA)
PAR-CLIP
PAR-CLIP (CCA)
All.reads
TtoC.reads
Coverage
Position relative to tRNA 5' end
Position relative to RNaseZ site

# tRNA-Glu-TTC-1-6

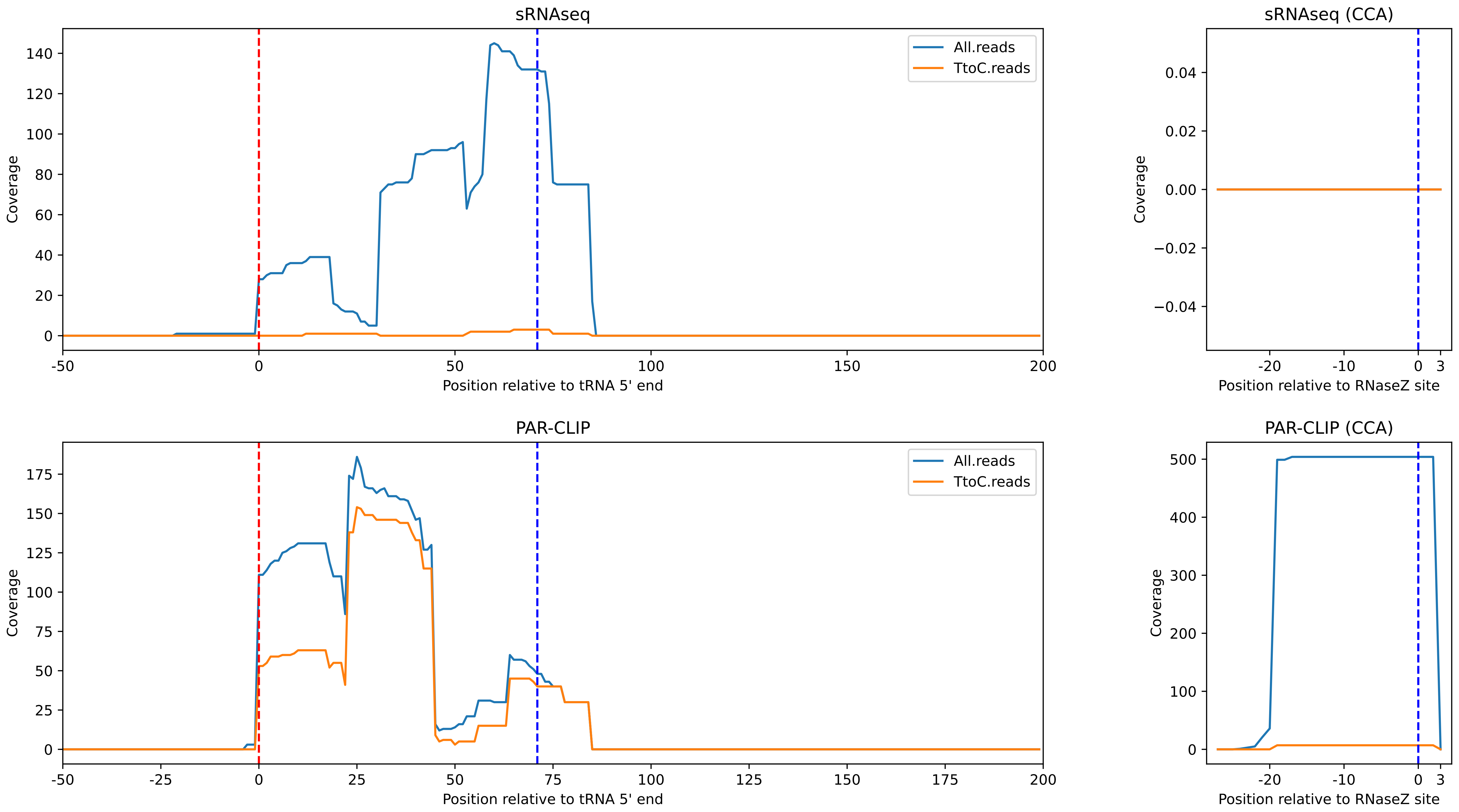

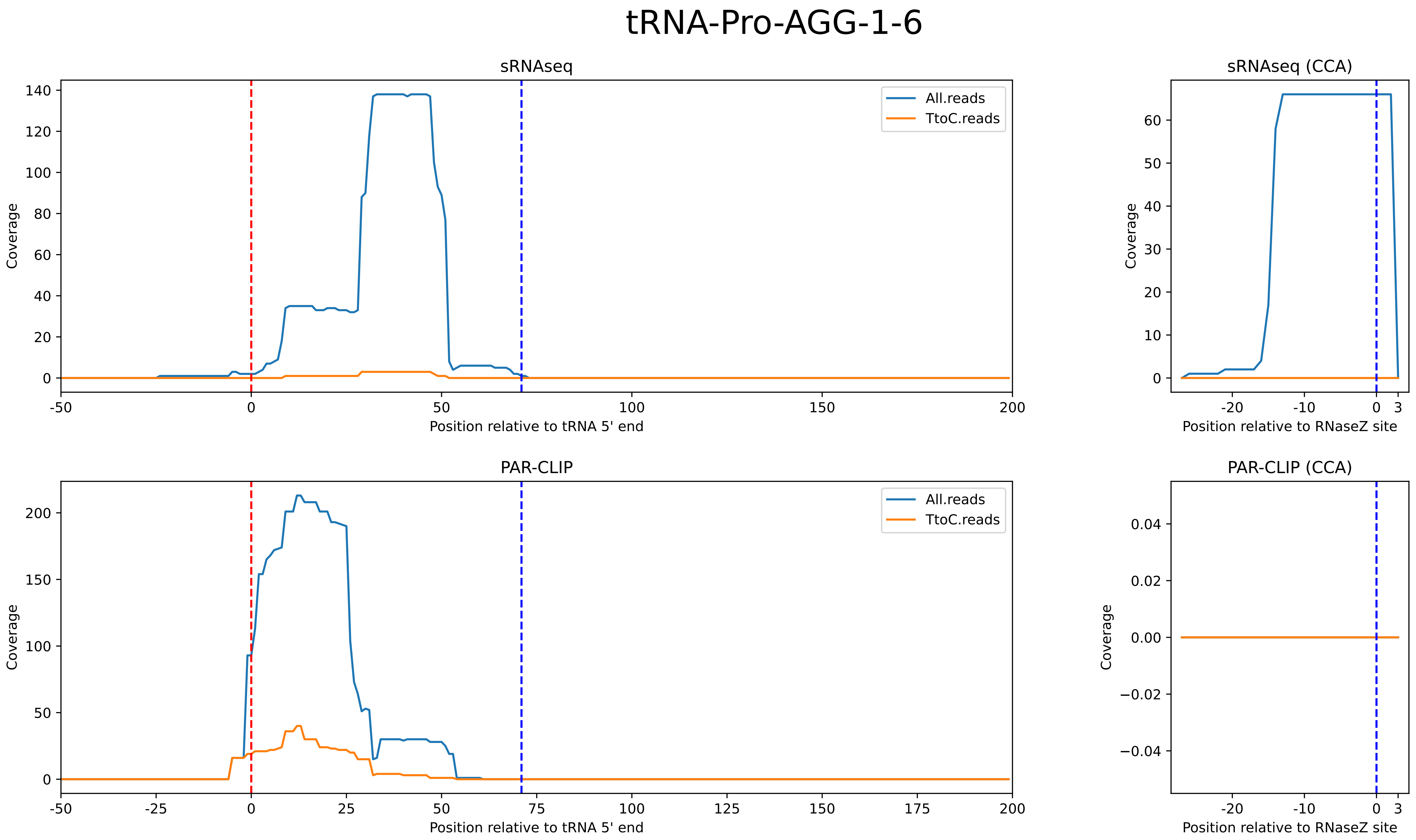

tRNA-Pro-AGG-1-6
sRNAseq
All.reads
TtoC.reads
Coverage
Position relative to tRNA 5' end
sRNAseq (CCA)
Position relative to RNaseZ site
PAR-CLIP
PAR-CLIP (CCA)

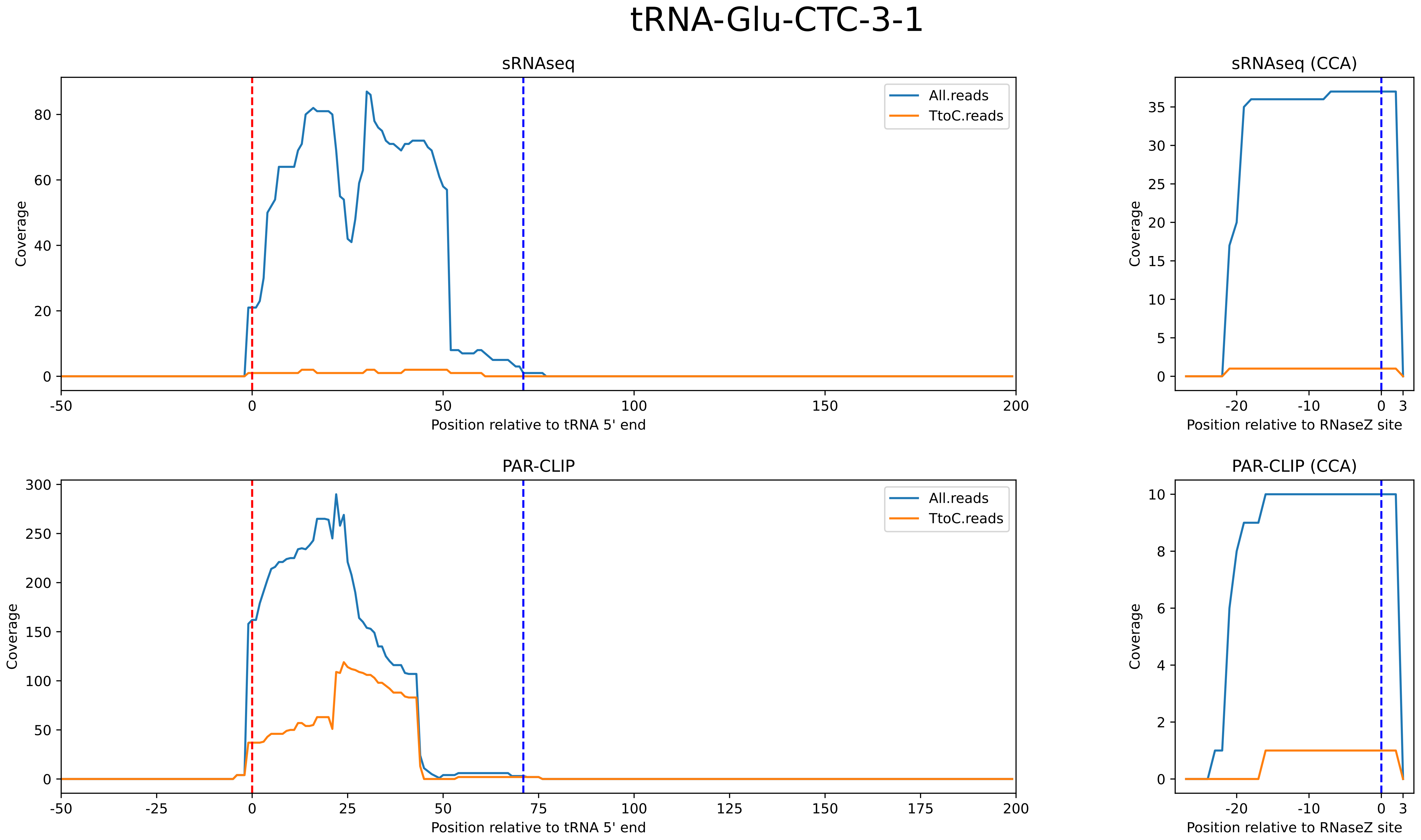
tRNA-Glu-CTC-3-1
sRNAseq
All.reads
TtoC.reads
Coverage
Position relative to tRNA 5' end
sRNAseq (CCA)
Position relative to RNaseZ site
PAR-CLIP
PAR-CLIP (CCA)

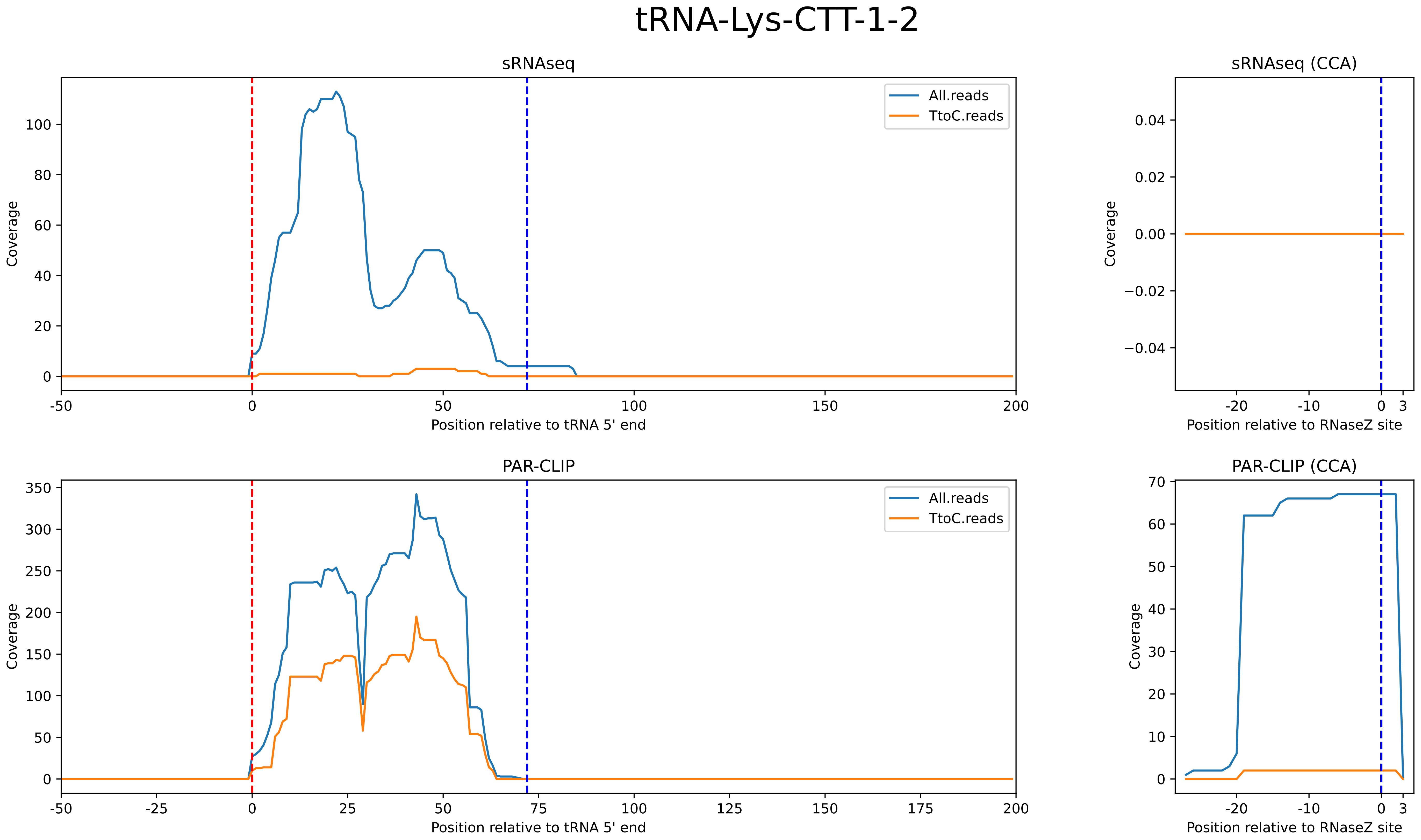
tRNA-Lys-CTT-1-2
sRNAseq
All.reads
TtoC.reads
Coverage
Position relative to tRNA 5' end
sRNAseq (CCA)
Position relative to RNaseZ site
PAR-CLIP
PAR-CLIP (CCA)

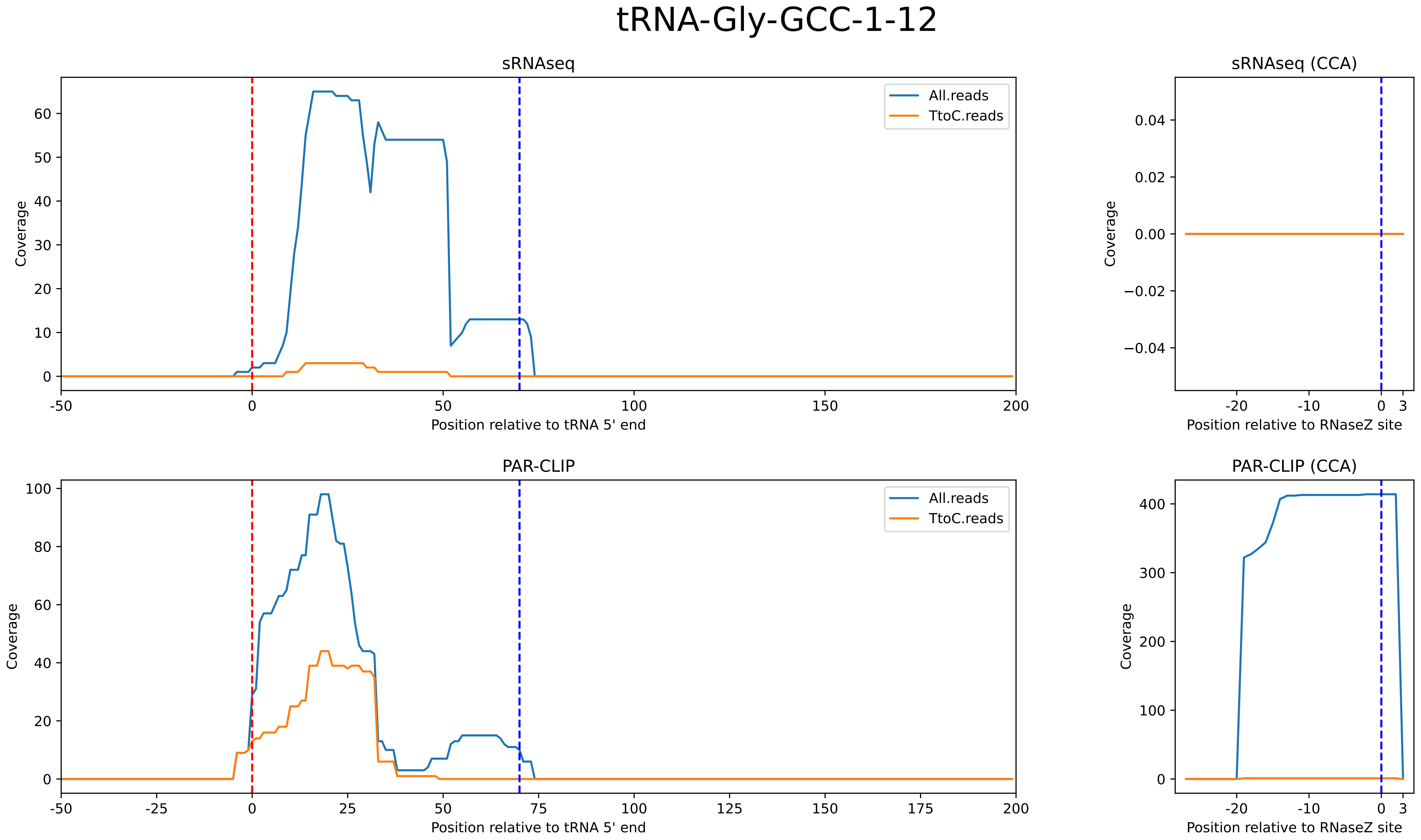

tRNA-Gly-GCC-1-12
sRNAseq
sRNAseq (CCA)
PAR-CLIP
PAR-CLIP (CCA)
All.reads
TtoC.reads
Coverage
Position relative to tRNA 5' end
Position relative to RNaseZ site

# tRNA-Thr-CGT-1-2

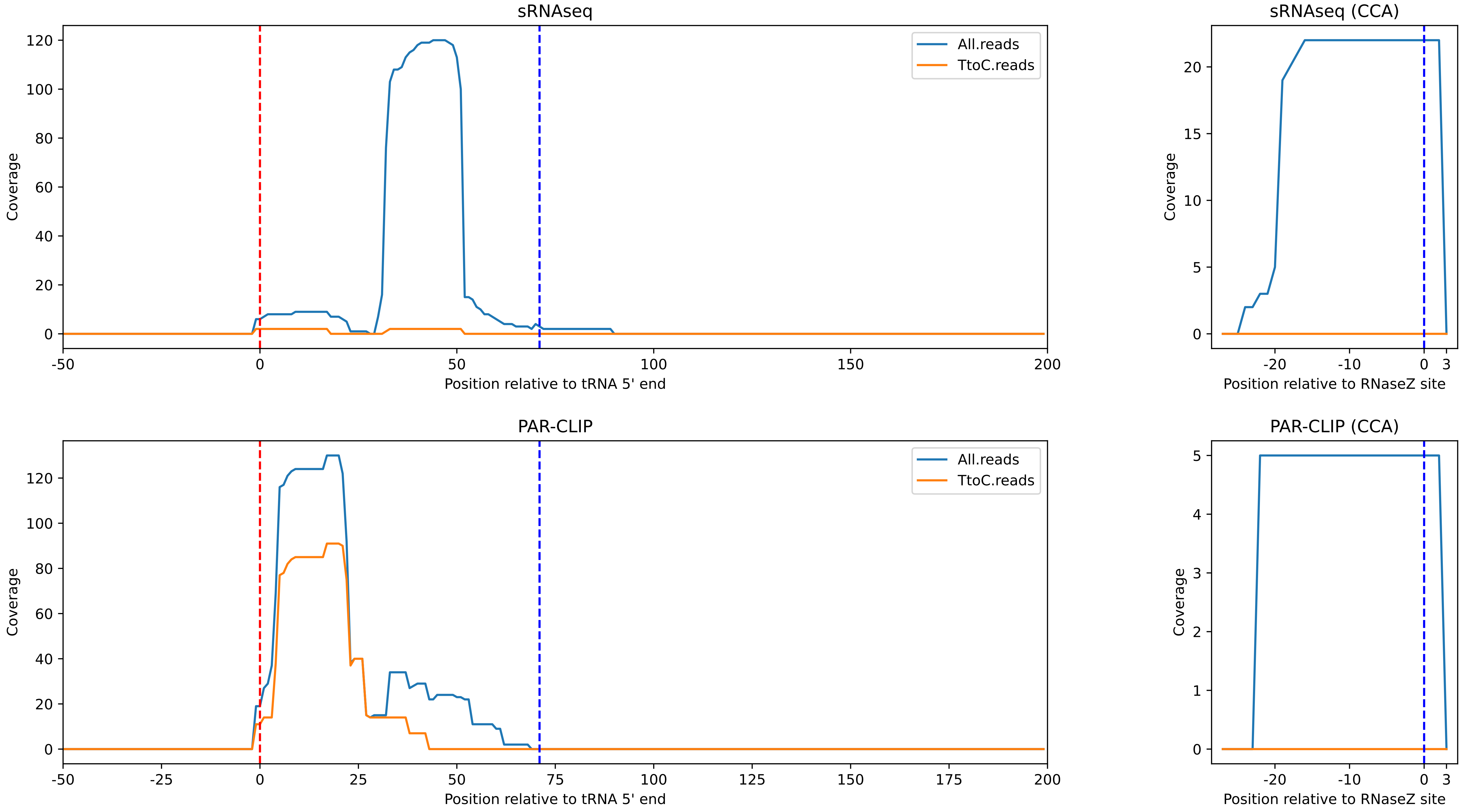

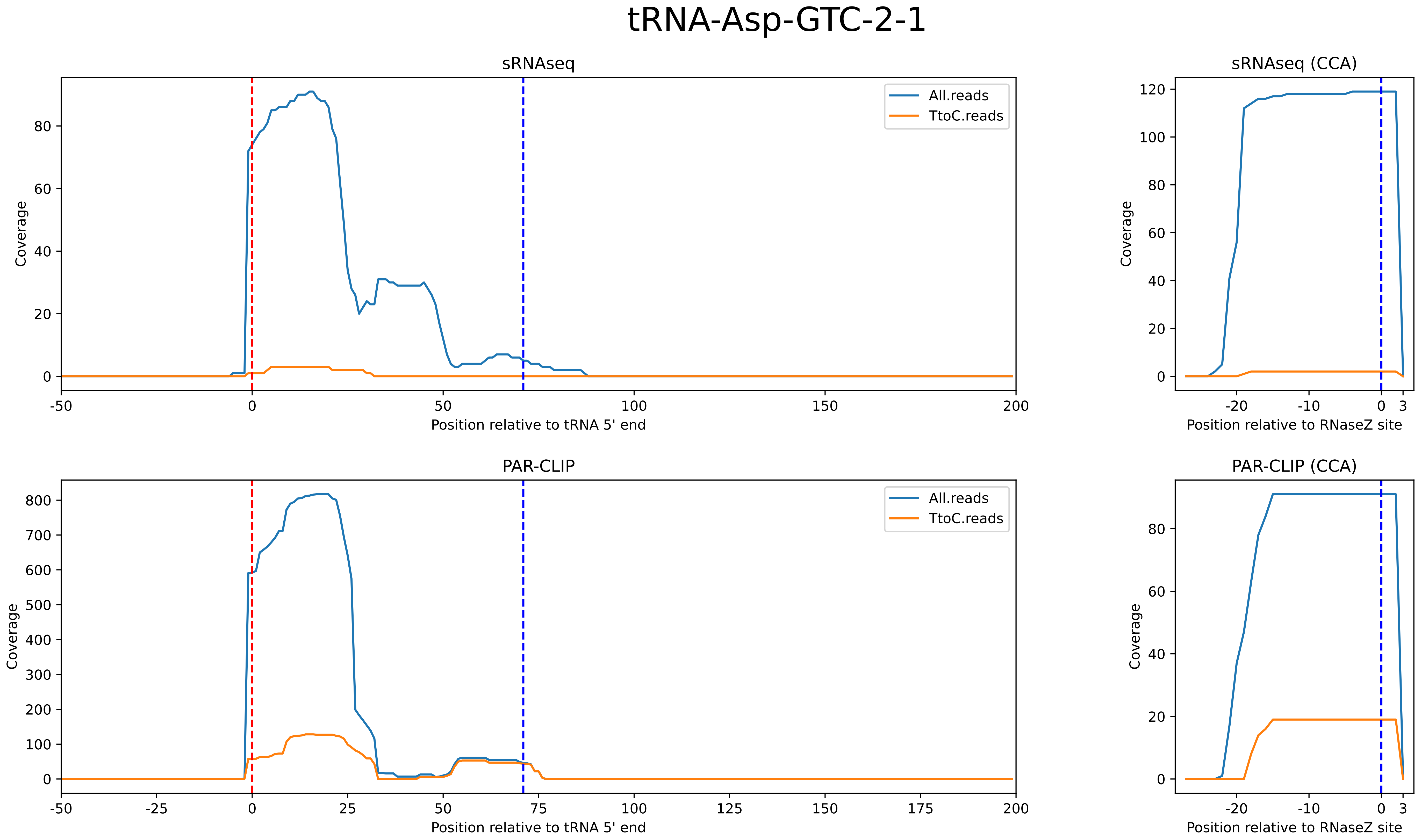

tRNA-Asp-GTC-2-1
sRNAseq
All.reads
TtoC.reads
Coverage
Position relative to tRNA 5' end
sRNAseq (CCA)
Position relative to RNaseZ site
PAR-CLIP
PAR-CLIP (CCA)

# tRNA-Gly-GCC-2-1

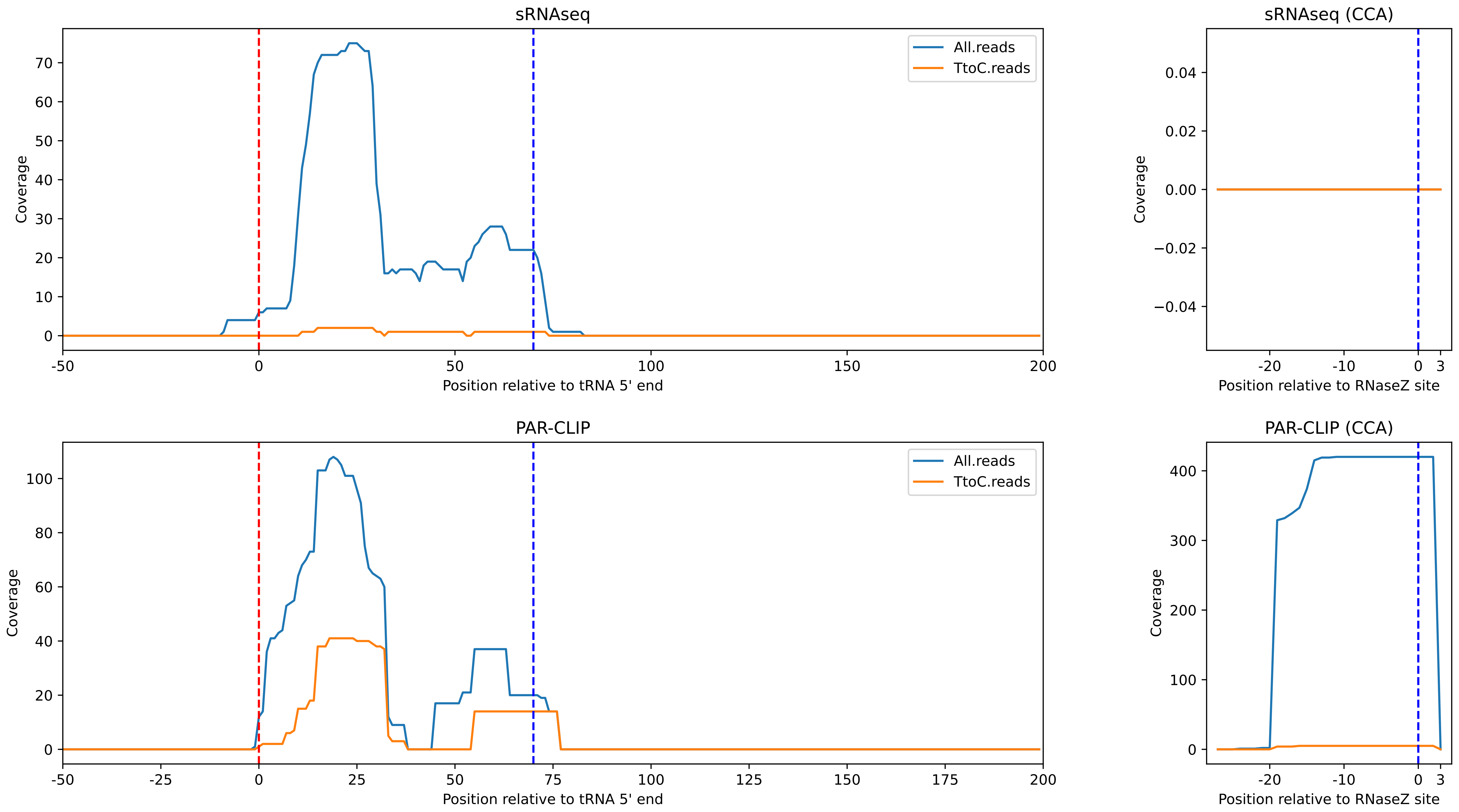

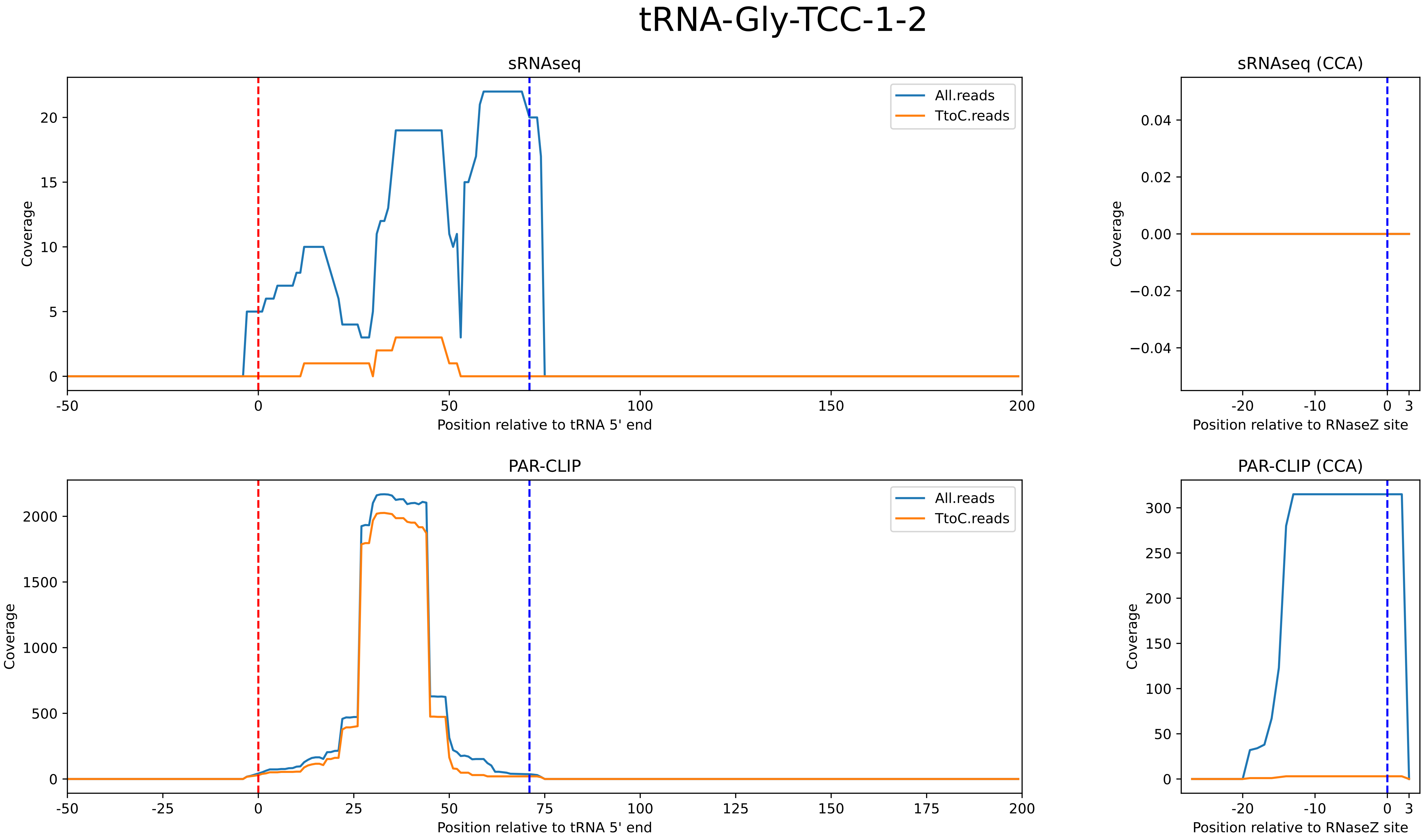

tRNA-Gly-TCC-1-2
sRNAseq
All.reads
TtoC.reads
Coverage
Position relative to tRNA 5' end
sRNAseq (CCA)
Position relative to RNaseZ site
PAR-CLIP
PAR-CLIP (CCA)

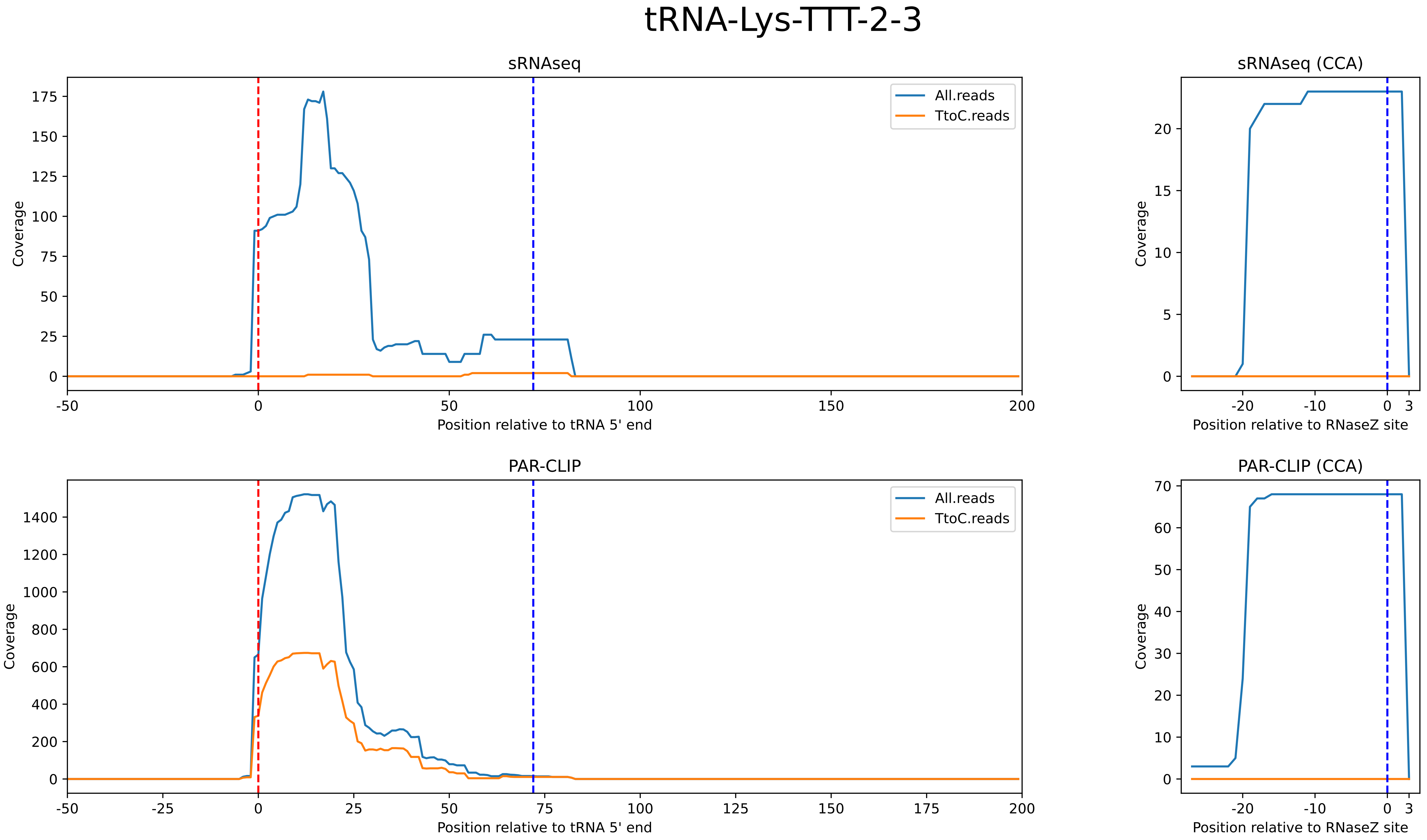

tRNA-Lys-TTT-2-3
sRNAseq
All.reads
TtoC.reads
Coverage
Position relative to tRNA 5' end
sRNAseq (CCA)
Position relative to RNaseZ site
PAR-CLIP
PAR-CLIP (CCA)

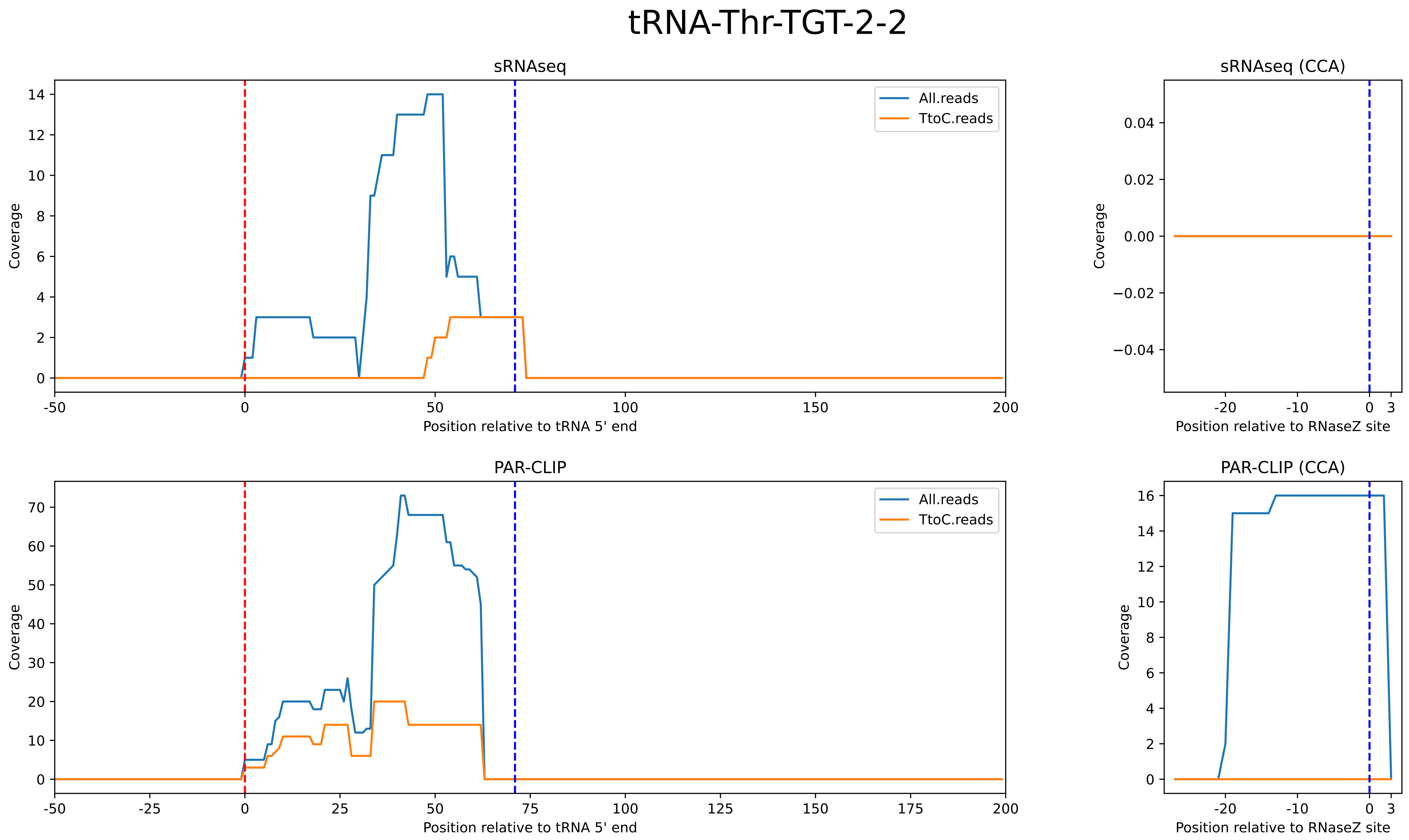
tRNA-Thr-TGT-2-2
sRNAseq
sRNAseq (CCA)
PAR-CLIP
PAR-CLIP (CCA)
All.reads
TtoC.reads
Coverage
Position relative to tRNA 5' end
Position relative to RNaseZ site

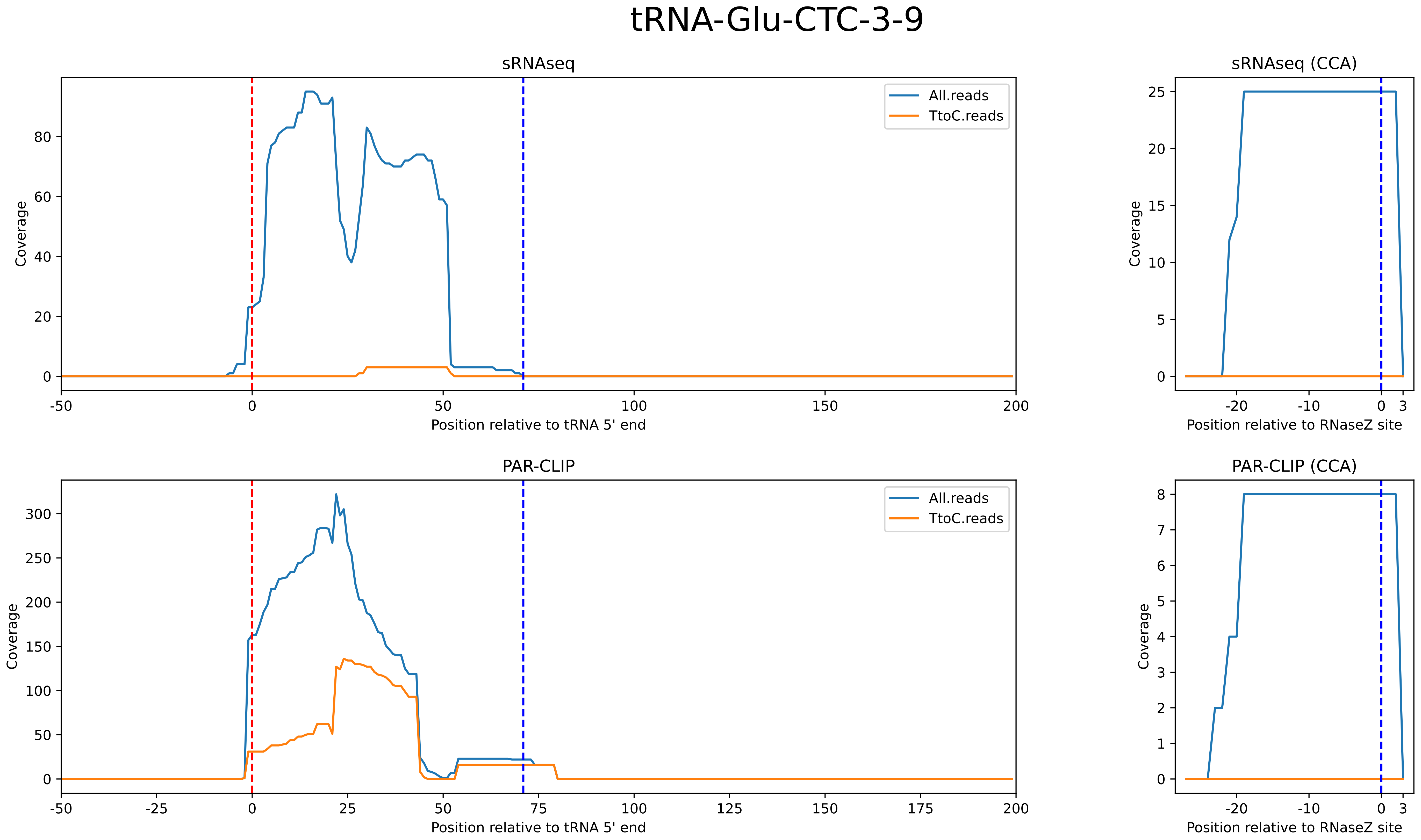

tRNA-Glu-CTC-3-9
sRNAseq
All.reads
TtoC.reads
Coverage
Position relative to tRNA 5' end
sRNAseq (CCA)
Position relative to RNaseZ site
PAR-CLIP
PAR-CLIP (CCA)

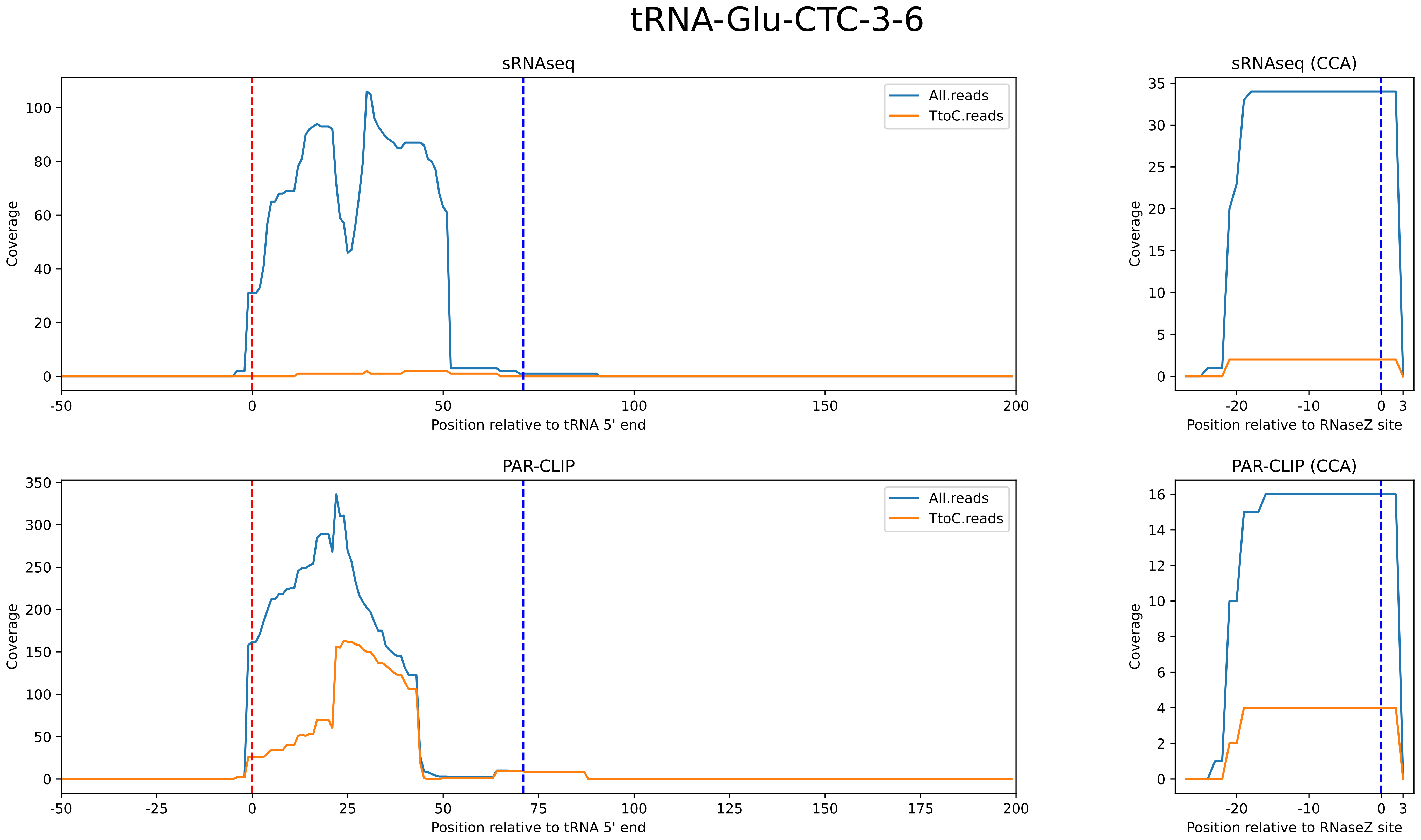
tRNA-Glu-CTC-3-6
sRNAseq
All.reads
TtoC.reads
Coverage
Position relative to tRNA 5' end
sRNAseq (CCA)
Position relative to RNaseZ site
PAR-CLIP
PAR-CLIP (CCA)

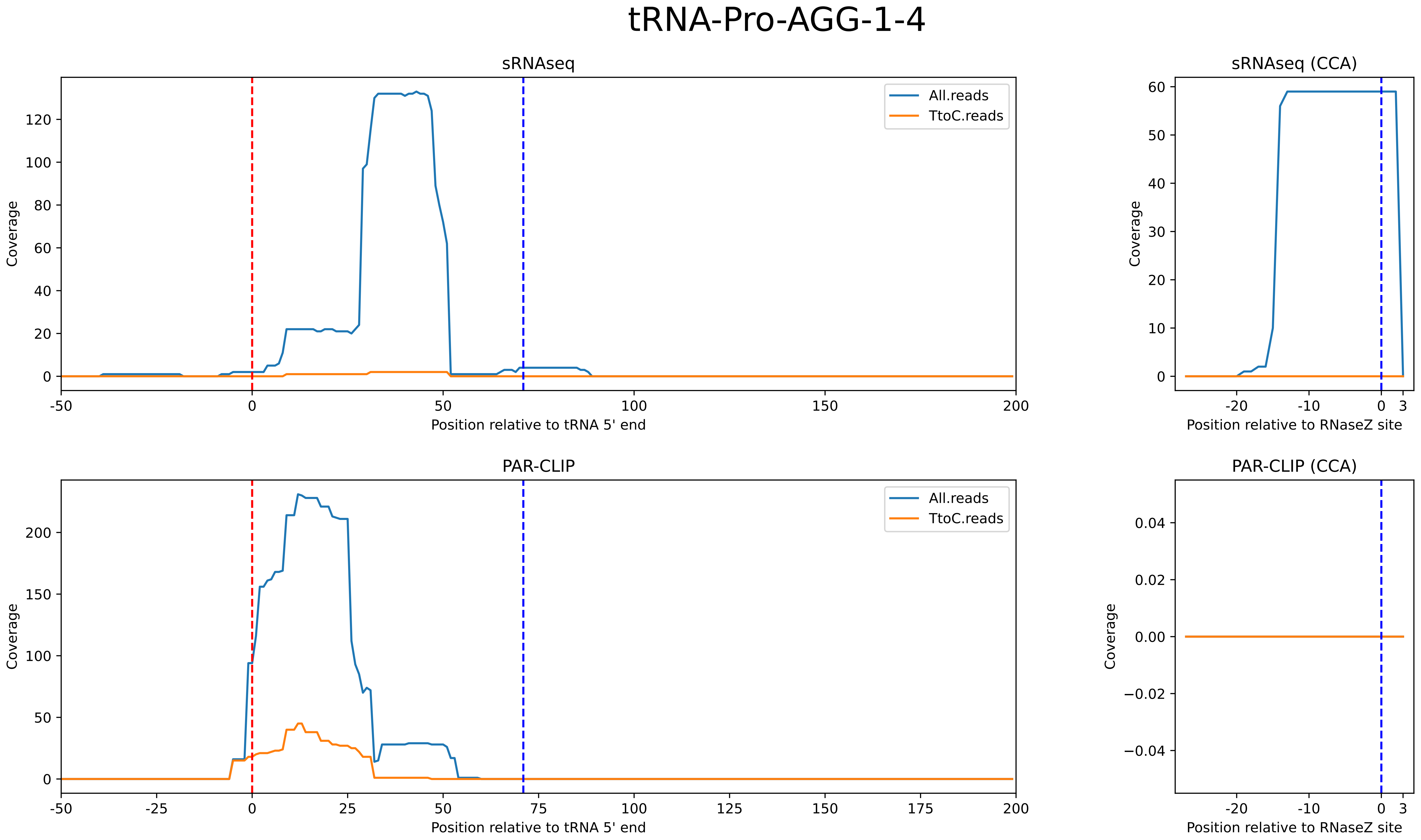
tRNA-Pro-AGG-1-4
sRNAseq
All.reads
TtoC.reads
Coverage
Position relative to tRNA 5' end
sRNAseq (CCA)
Position relative to RNaseZ site
PAR-CLIP
PAR-CLIP (CCA)

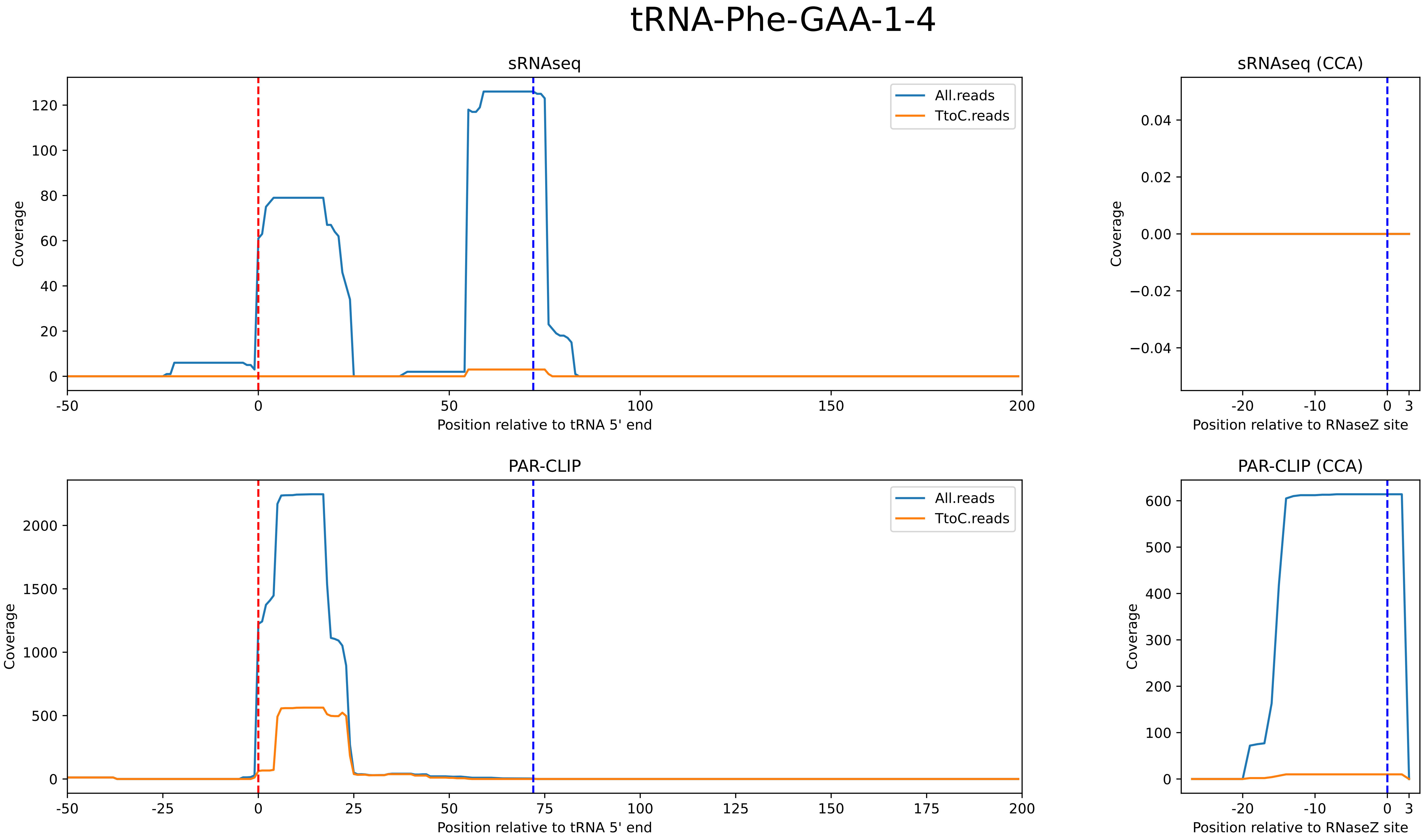
tRNA-Phe-GAA-1-4
sRNAseq
All.reads
TtoC.reads
Coverage
Position relative to tRNA 5' end
sRNAseq (CCA)
Position relative to RNaseZ site
PAR-CLIP
PAR-CLIP (CCA)

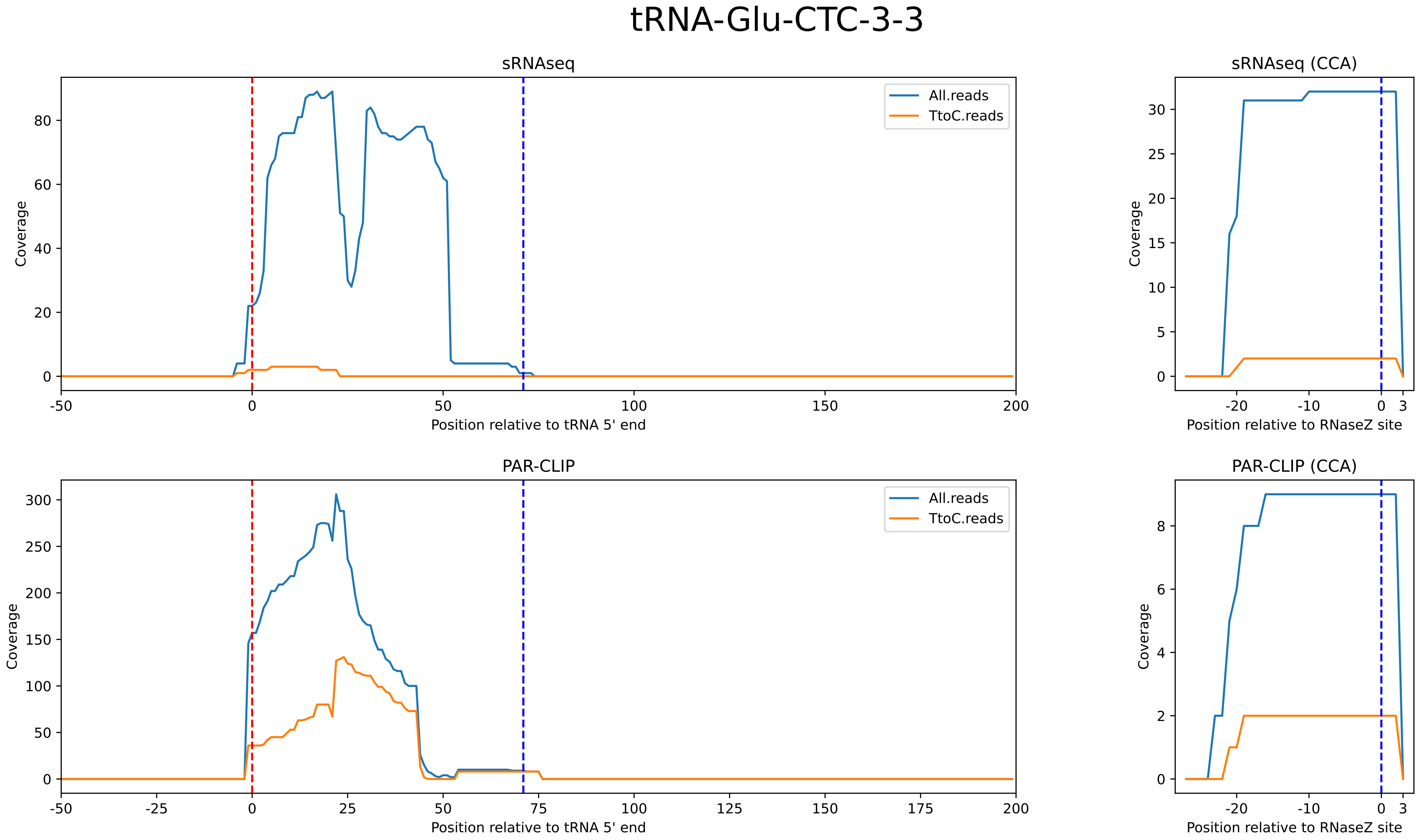
tRNA-Glu-CTC-3-3
sRNAseq
sRNAseq (CCA)
PAR-CLIP
PAR-CLIP (CCA)
All.reads
TtoC.reads
Coverage
Position relative to tRNA 5' end
Position relative to RNaseZ site

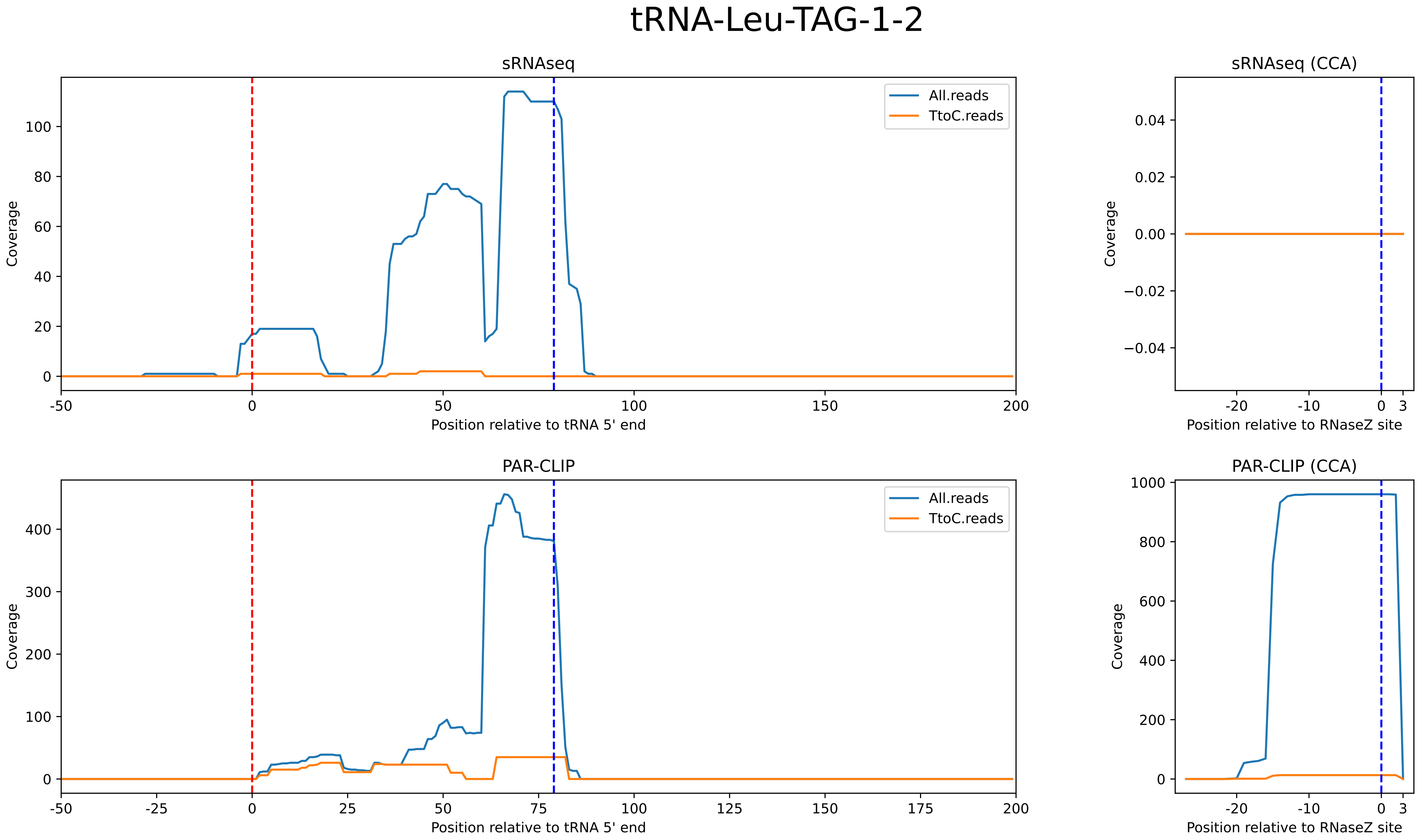

tRNA-Leu-TAG-1-2
sRNAseq
All.reads
TtoC.reads
Coverage
Position relative to tRNA 5' end
sRNAseq (CCA)
Position relative to RNaseZ site
PAR-CLIP
PAR-CLIP (CCA)

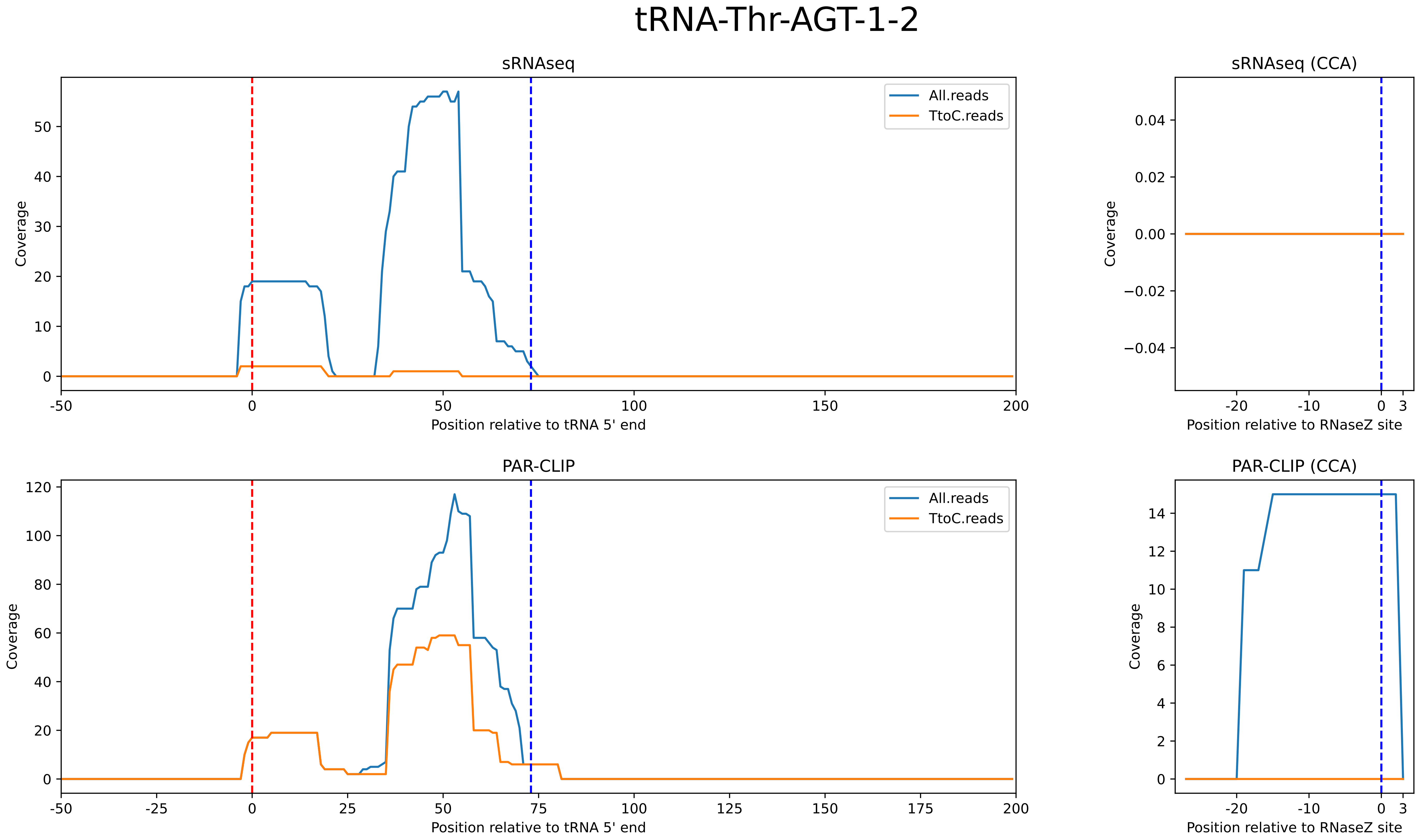

tRNA-Thr-AGT-1-2
sRNAseq
All.reads
TtoC.reads
Coverage
Position relative to tRNA 5' end
-50
0
50
100
150
200
sRNAseq (CCA)
0.04
0.02
0.00
−0.02
−0.04
-20
-10
0
3
Position relative to RNaseZ site
PAR-CLIP
120
100
80
60
40
20
-25
25
75
125
175
PAR-CLIP (CCA)
14
12
10
8
6
4
2

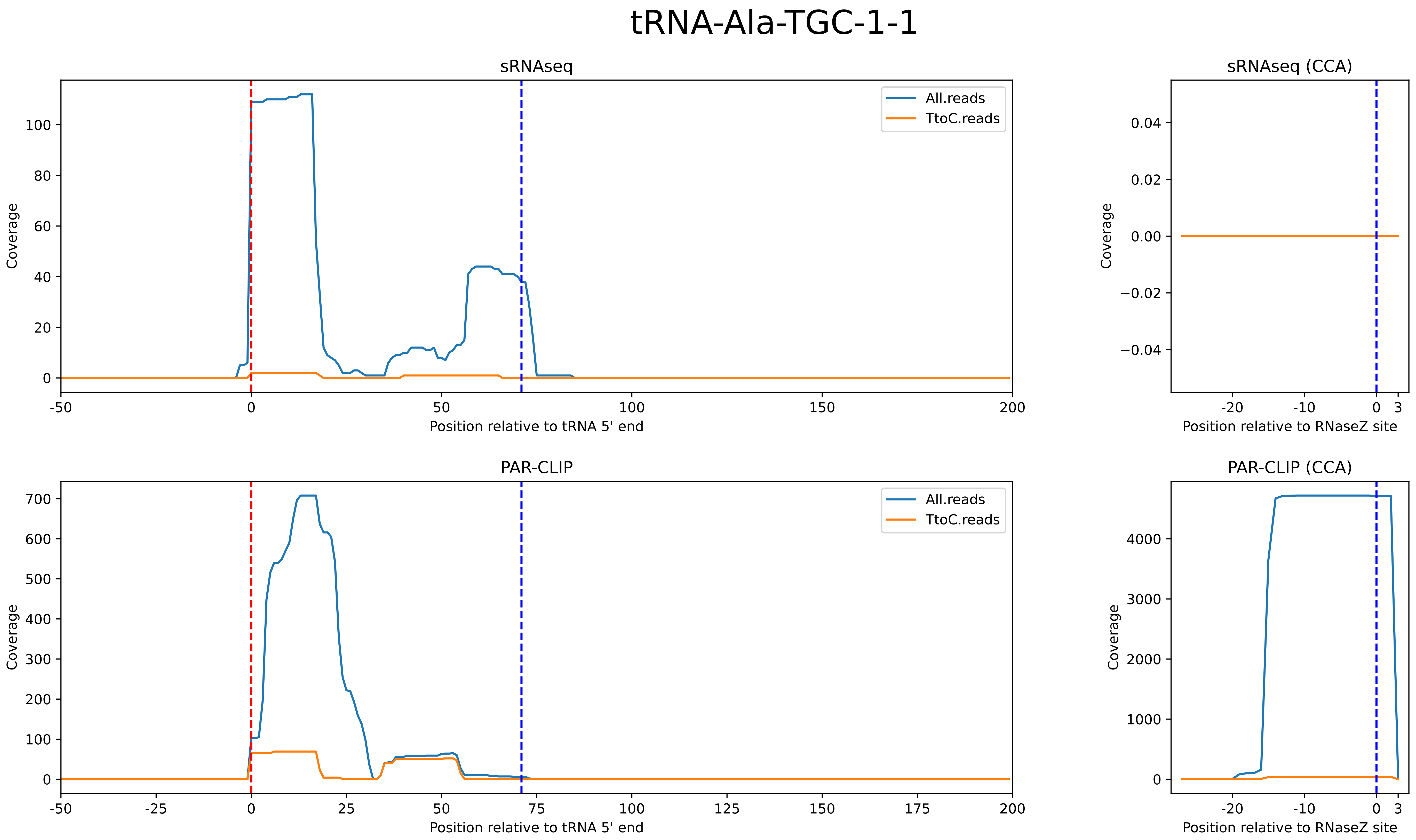
tRNA-Ala-TGC-1-1
sRNAseq
sRNAseq (CCA)
PAR-CLIP
PAR-CLIP (CCA)
All.reads
TtoC.reads
Coverage
Position relative to tRNA 5' end
Position relative to RNaseZ site

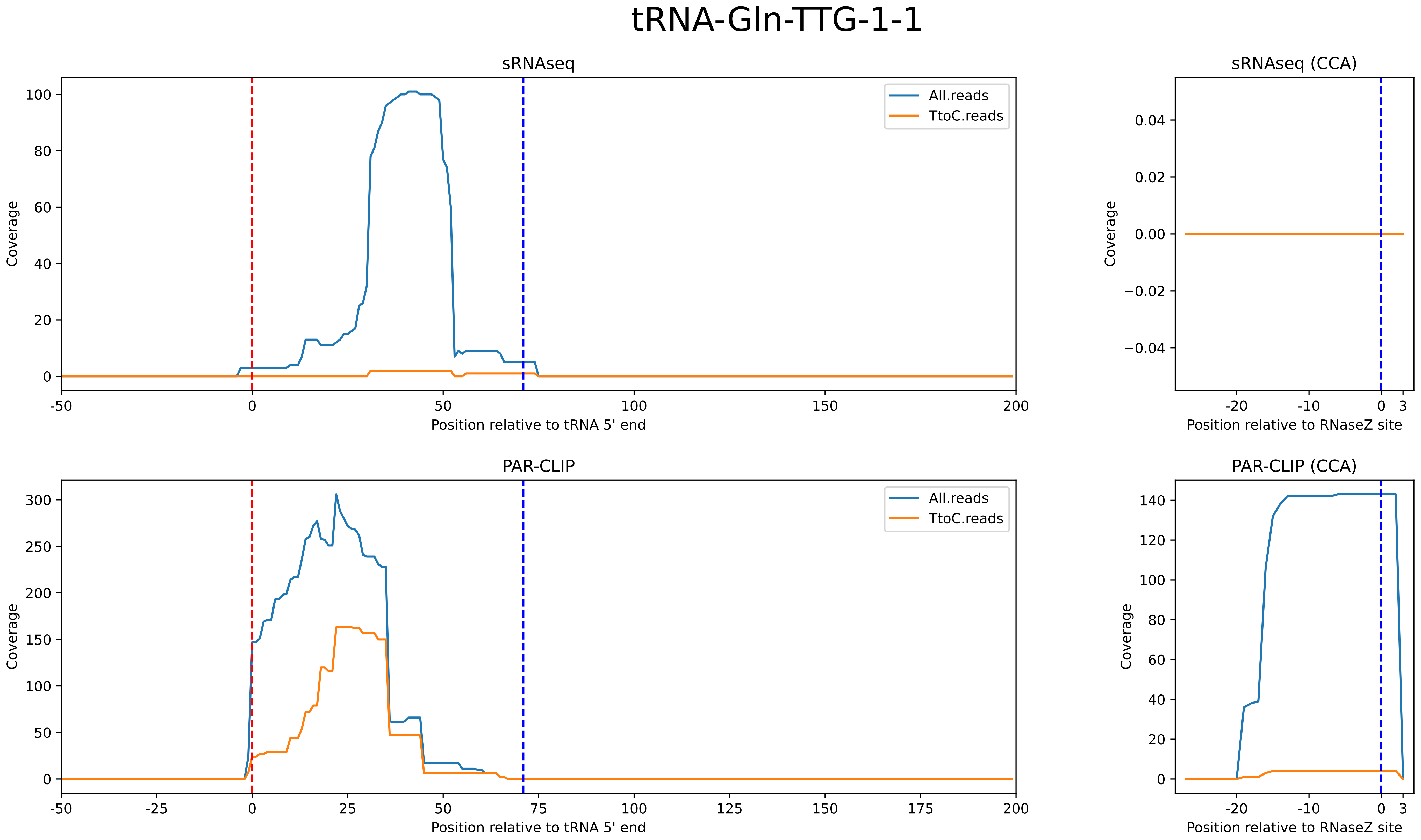
tRNA-Gln-TTG-1-1
sRNAseq
sRNAseq (CCA)
PAR-CLIP
PAR-CLIP (CCA)
All.reads
TtoC.reads
Coverage
Position relative to tRNA 5' end
Position relative to RNaseZ site

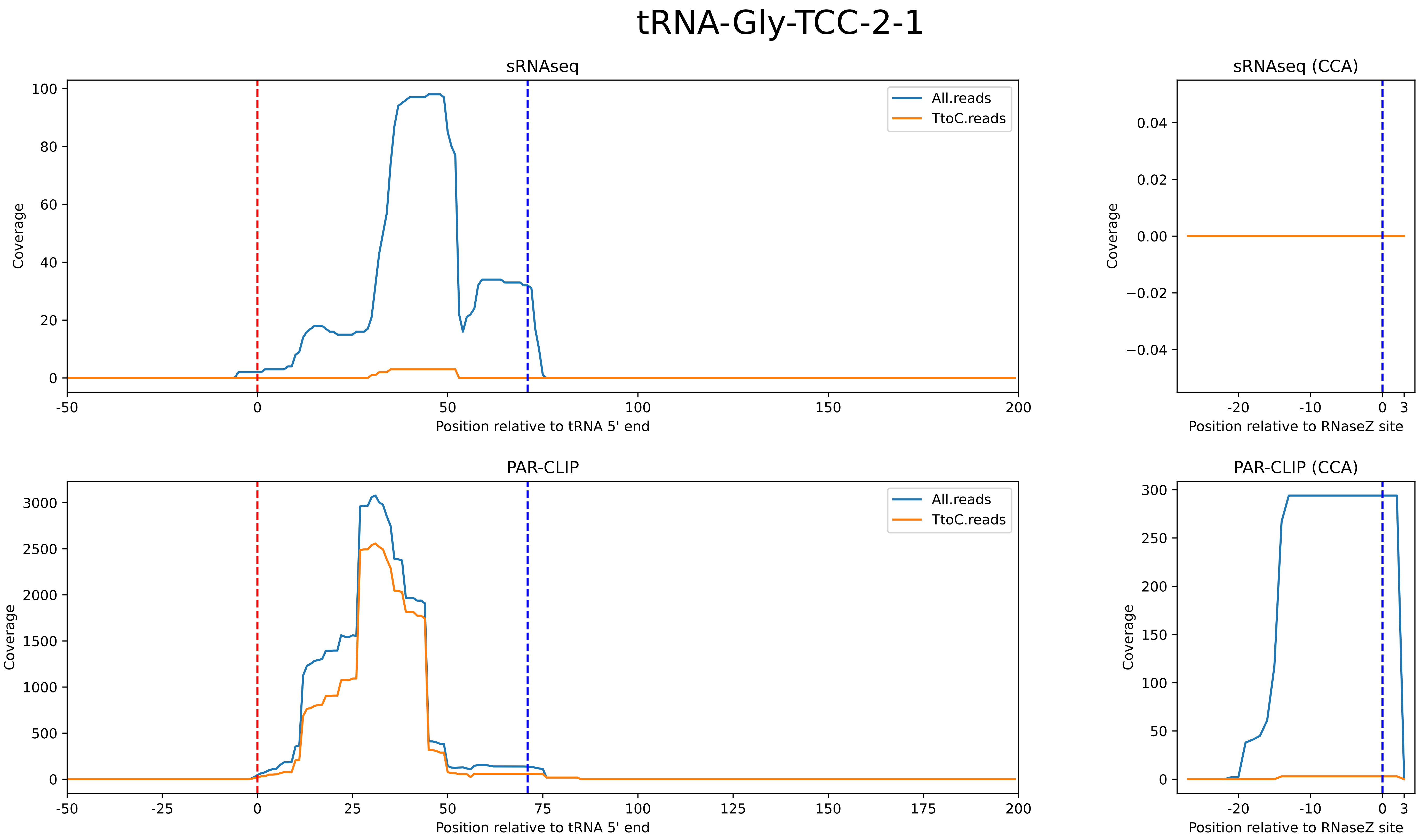

tRNA-Gly-TCC-2-1
sRNAseq
All.reads
TtoC.reads
Coverage
Position relative to tRNA 5' end
sRNAseq (CCA)
Position relative to RNaseZ site
PAR-CLIP
PAR-CLIP (CCA)

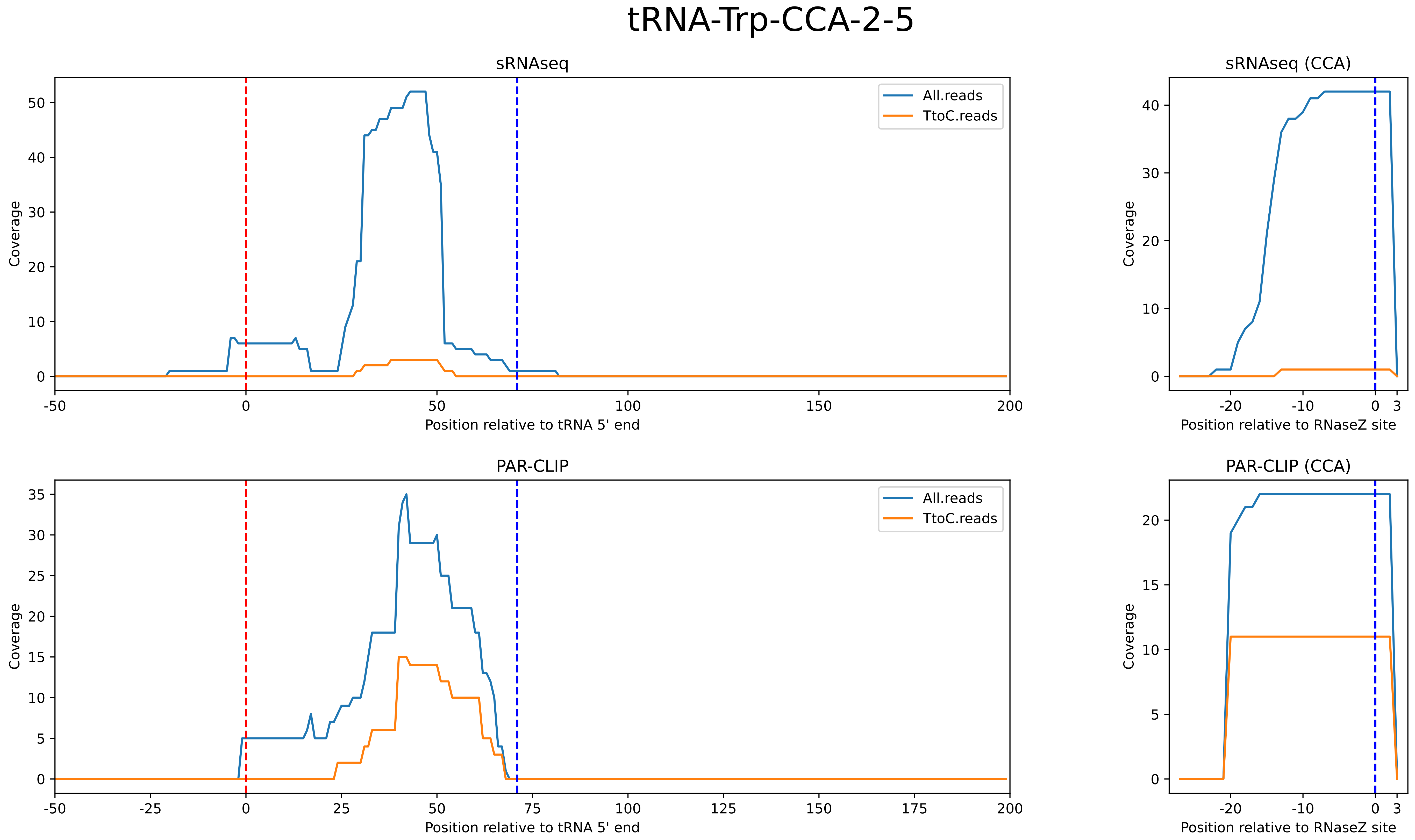

tRNA-Trp-CCA-2-5
sRNAseq
All.reads
TtoC.reads
Coverage
Position relative to tRNA 5' end
sRNAseq (CCA)
Position relative to RNaseZ site
PAR-CLIP
PAR-CLIP (CCA)

# tRNA-Ile-TAT-1-1

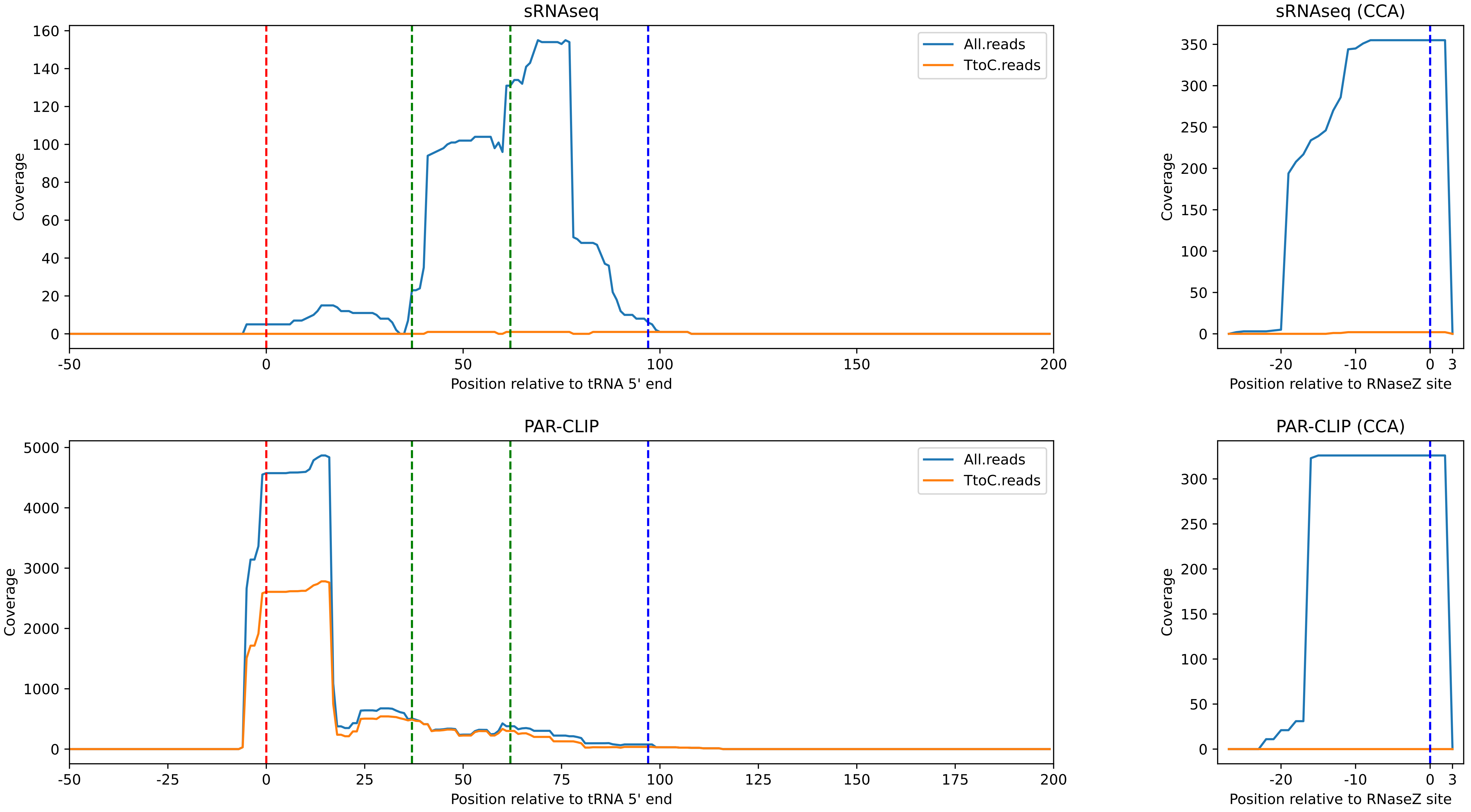

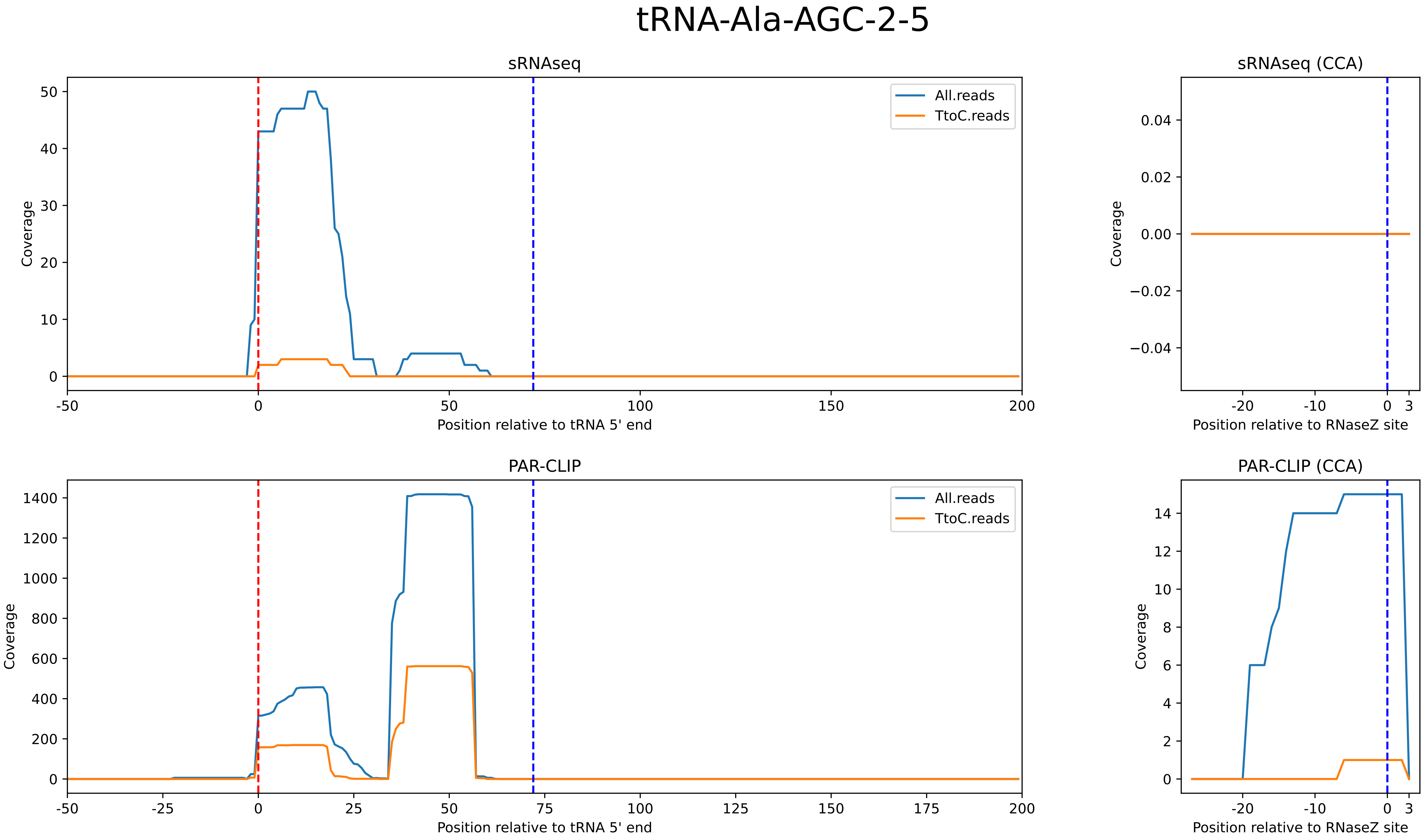

tRNA-Ala-AGC-2-5
sRNAseq
All.reads
TtoC.reads
Coverage
Position relative to tRNA 5' end
sRNAseq (CCA)
Position relative to RNaseZ site
PAR-CLIP
PAR-CLIP (CCA)

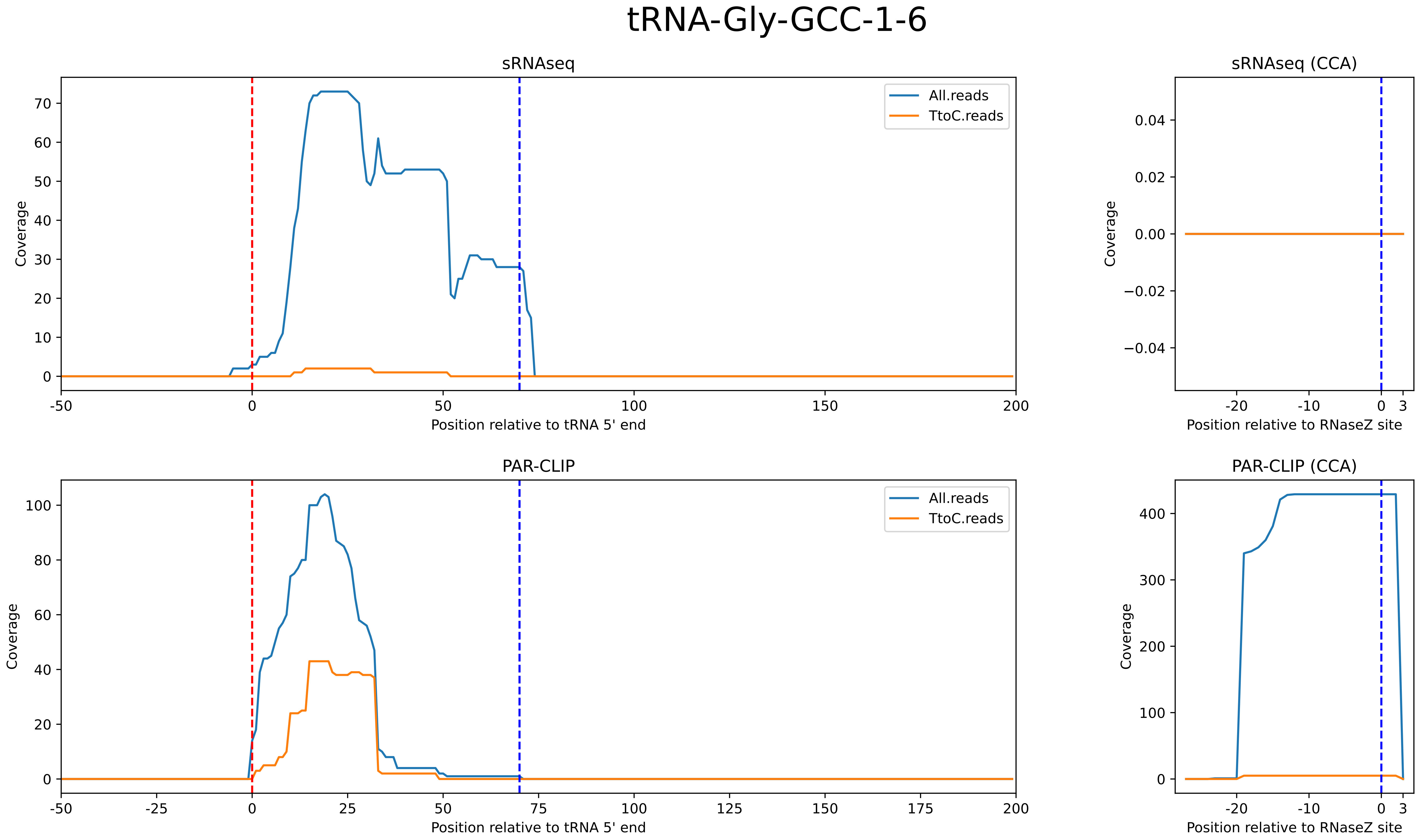
tRNA-Gly-GCC-1-6
sRNAseq
All.reads
TtoC.reads
Coverage
Position relative to tRNA 5' end
sRNAseq (CCA)
Position relative to RNaseZ site
PAR-CLIP
PAR-CLIP (CCA)

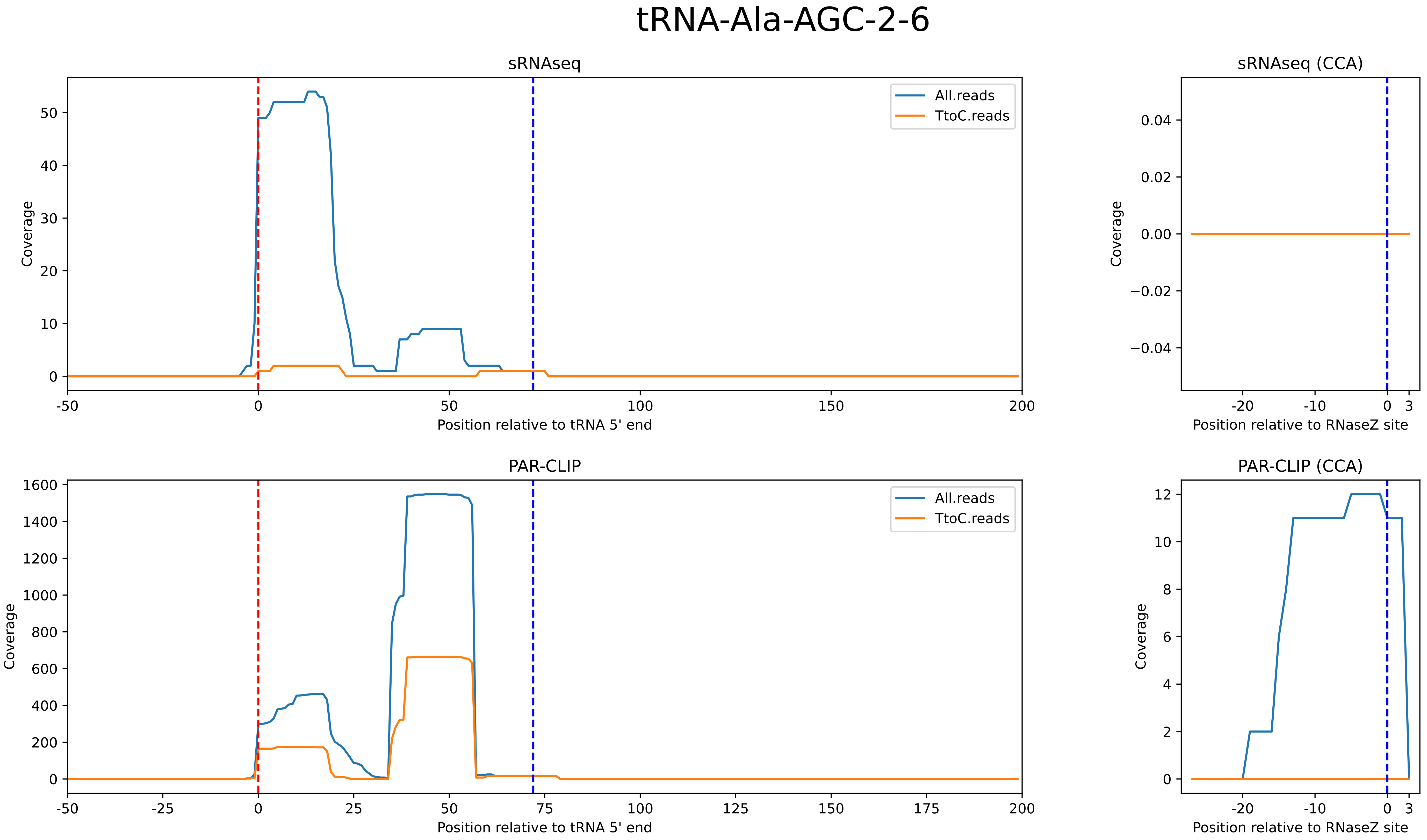
tRNA-Ala-AGC-2-6
sRNAseq
sRNAseq (CCA)
PAR-CLIP
PAR-CLIP (CCA)
All.reads
TtoC.reads
Coverage
Position relative to tRNA 5' end
Position relative to RNaseZ site

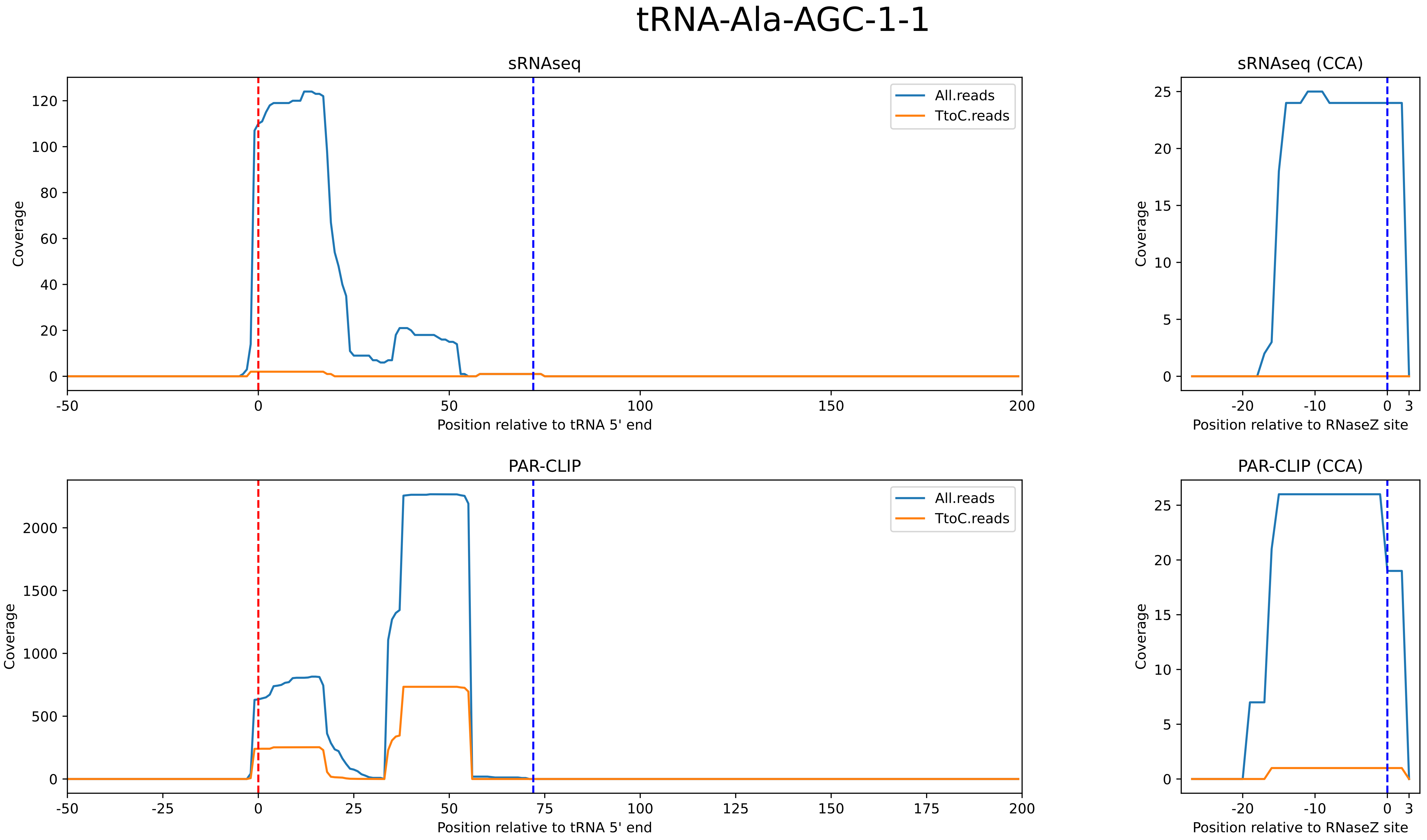
tRNA-Ala-AGC-1-1
sRNAseq
All.reads
TtoC.reads
Coverage
Position relative to tRNA 5' end
sRNAseq (CCA)
Position relative to RNaseZ site
PAR-CLIP
PAR-CLIP (CCA)

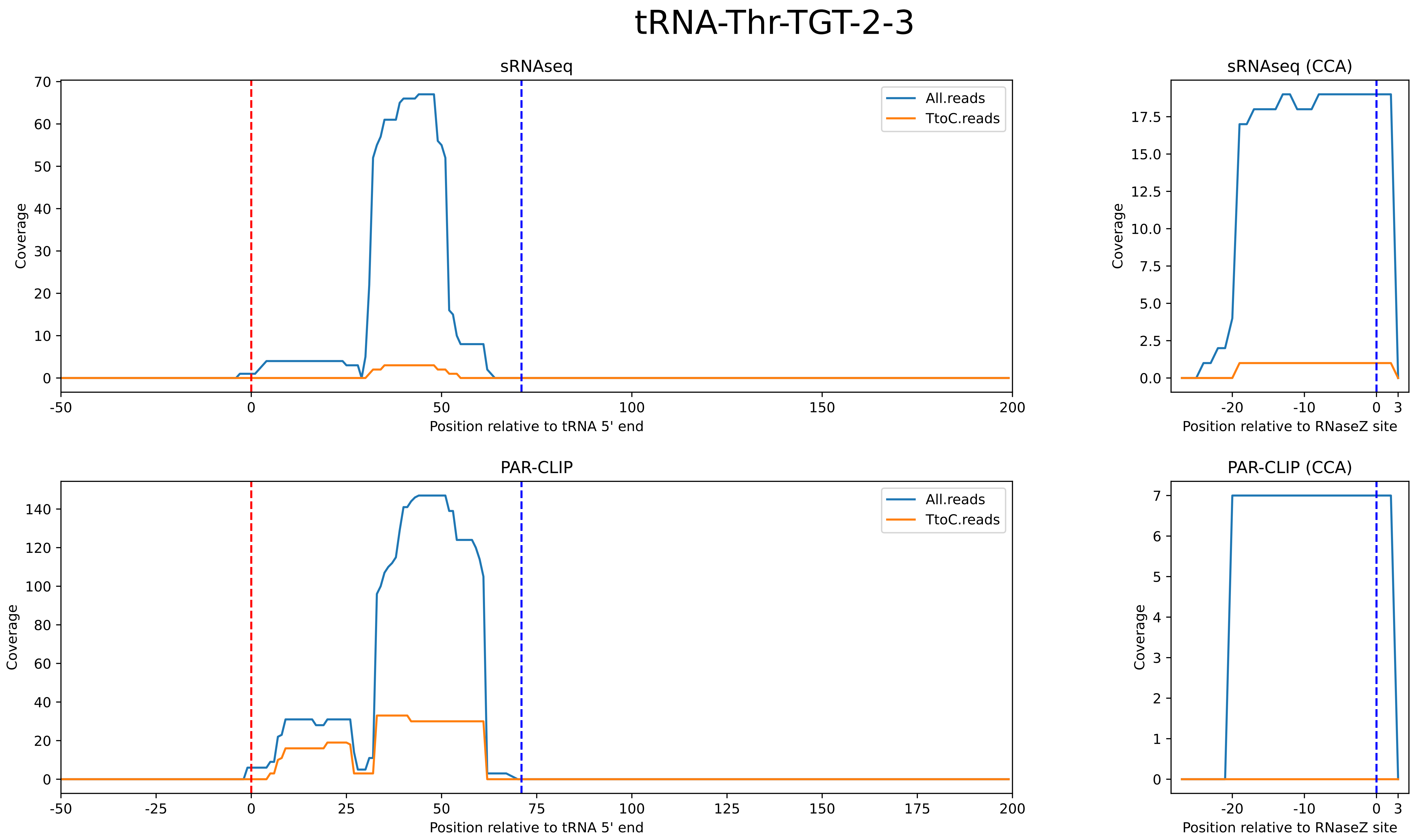
tRNA-Thr-TGT-2-3
sRNAseq
sRNAseq (CCA)
PAR-CLIP
PAR-CLIP (CCA)
All.reads
TtoC.reads
Coverage
Position relative to tRNA 5' end
Position relative to RNaseZ site

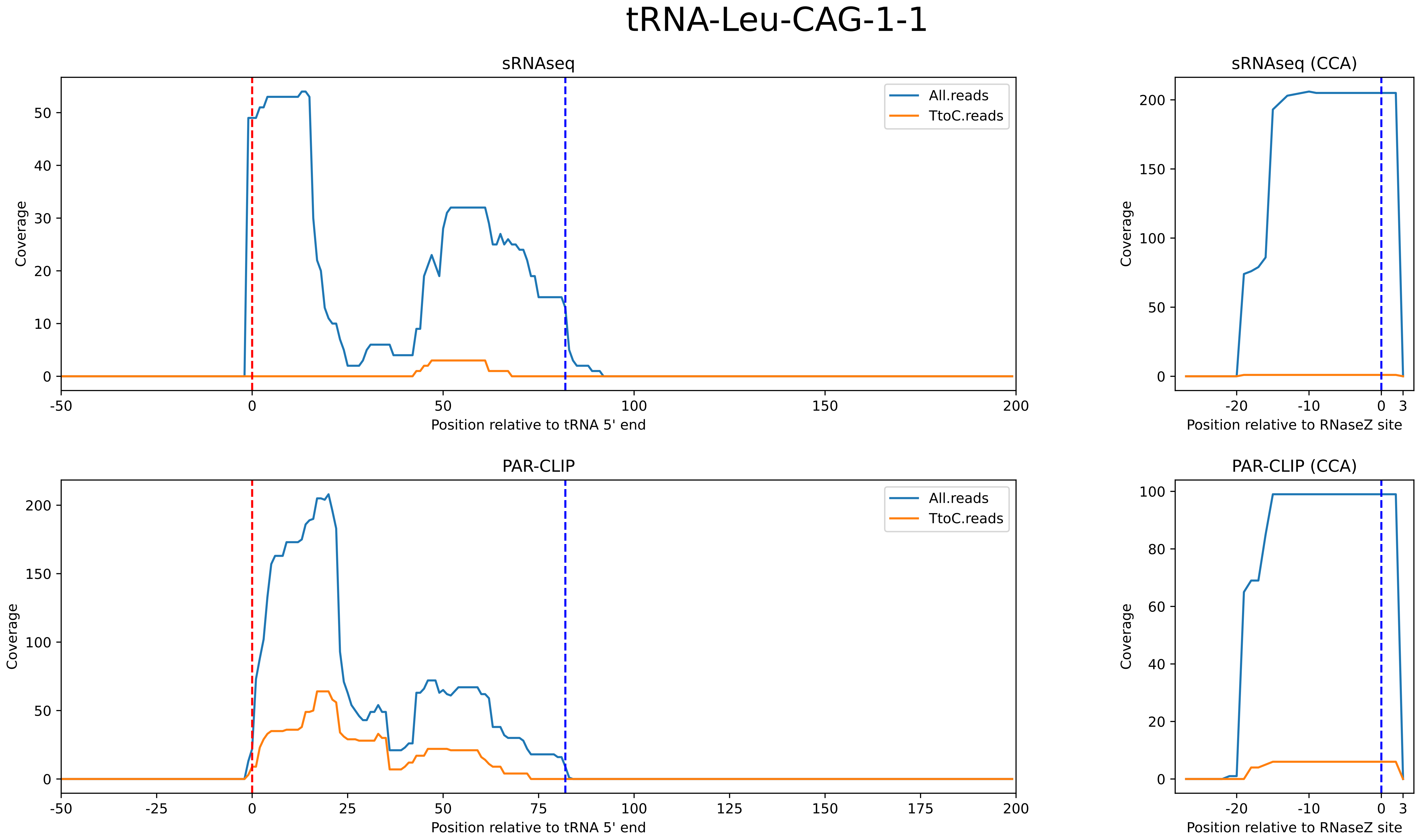
tRNA-Leu-CAG-1-1
sRNAseq
All.reads
TtoC.reads
Coverage
Position relative to tRNA 5' end
sRNAseq (CCA)
Position relative to RNaseZ site
PAR-CLIP
PAR-CLIP (CCA)

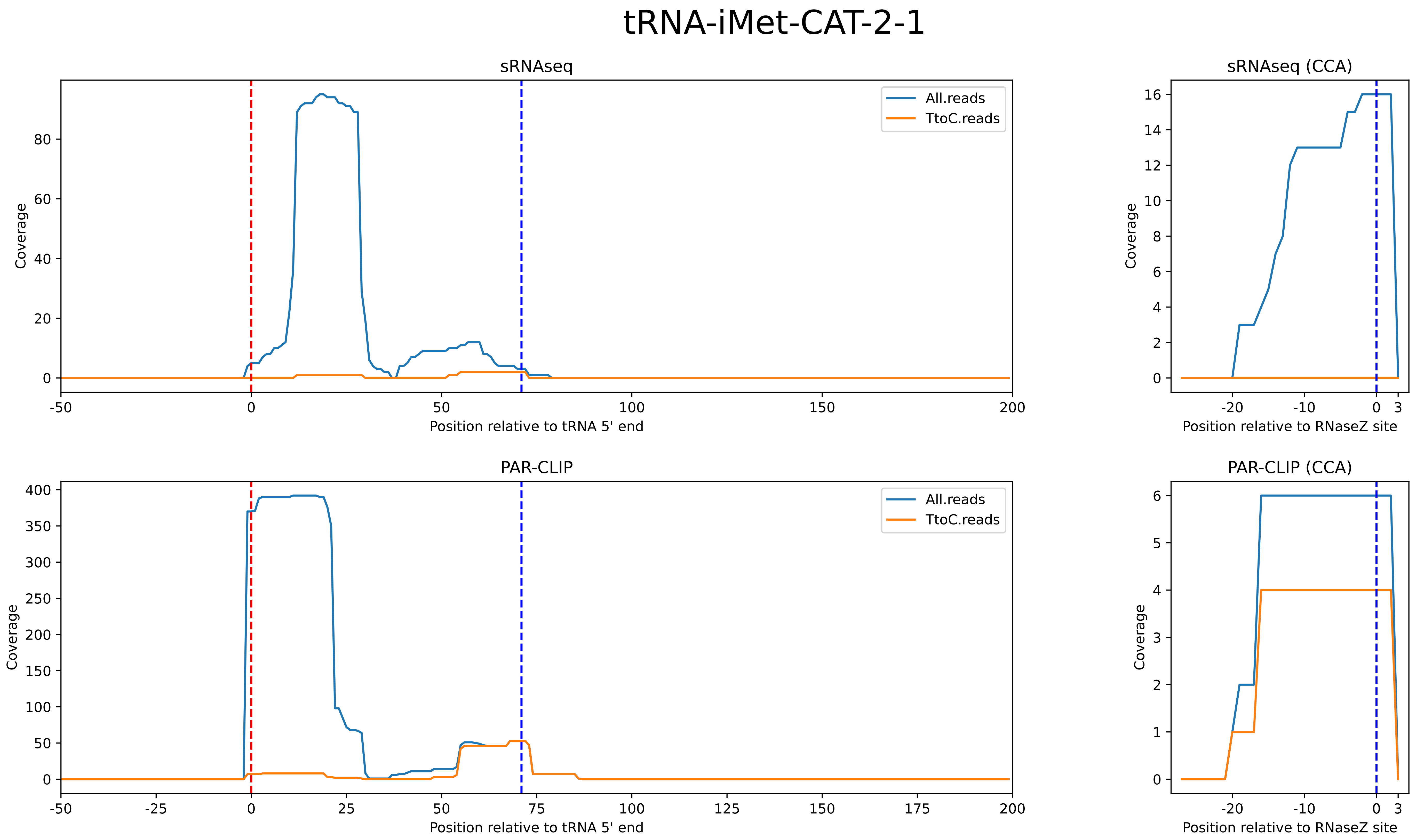

tRNA-iMet-CAT-2-1
sRNAseq
All.reads
TtoC.reads
Coverage
Position relative to tRNA 5' end
sRNAseq (CCA)
Position relative to RNaseZ site
PAR-CLIP
PAR-CLIP (CCA)

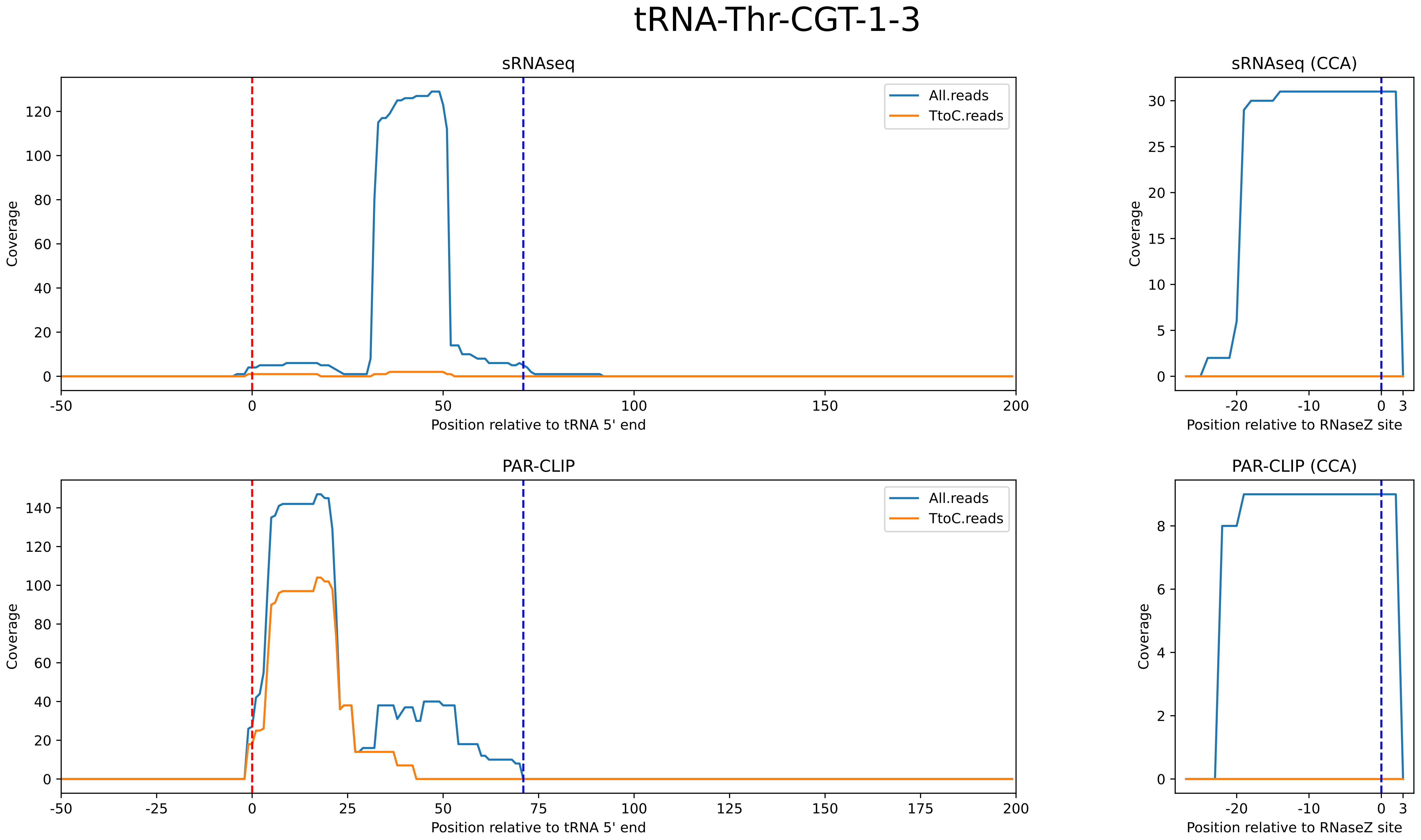

tRNA-Thr-CGT-1-3
sRNAseq
All.reads
TtoC.reads
Coverage
Position relative to tRNA 5' end
sRNAseq (CCA)
Position relative to RNaseZ site
PAR-CLIP
PAR-CLIP (CCA)

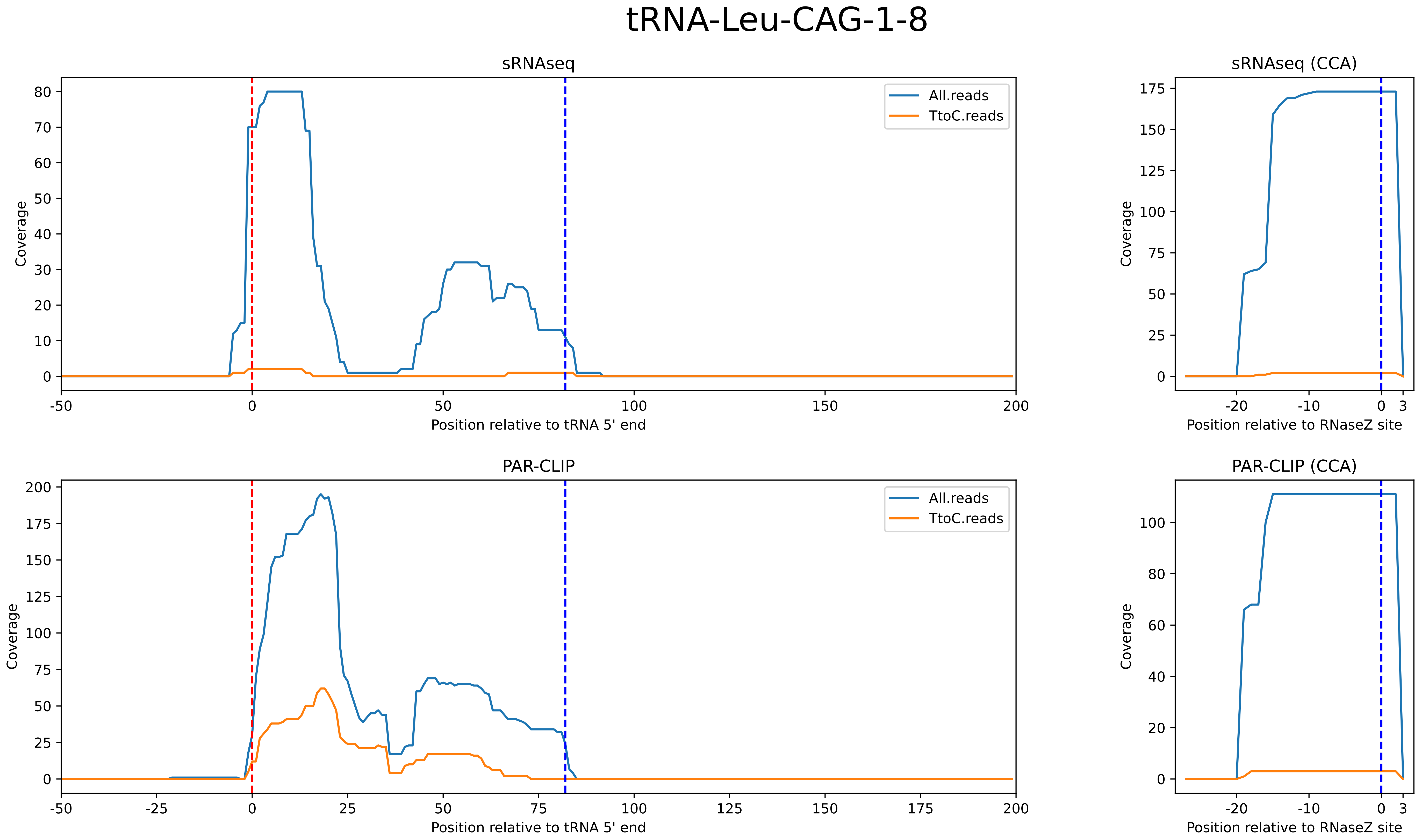
tRNA-Leu-CAG-1-8
sRNAseq
All.reads
TtoC.reads
Coverage
Position relative to tRNA 5' end
sRNAseq (CCA)
Position relative to RNaseZ site
PAR-CLIP
PAR-CLIP (CCA)

# tRNA-Phe-GAA-1-2

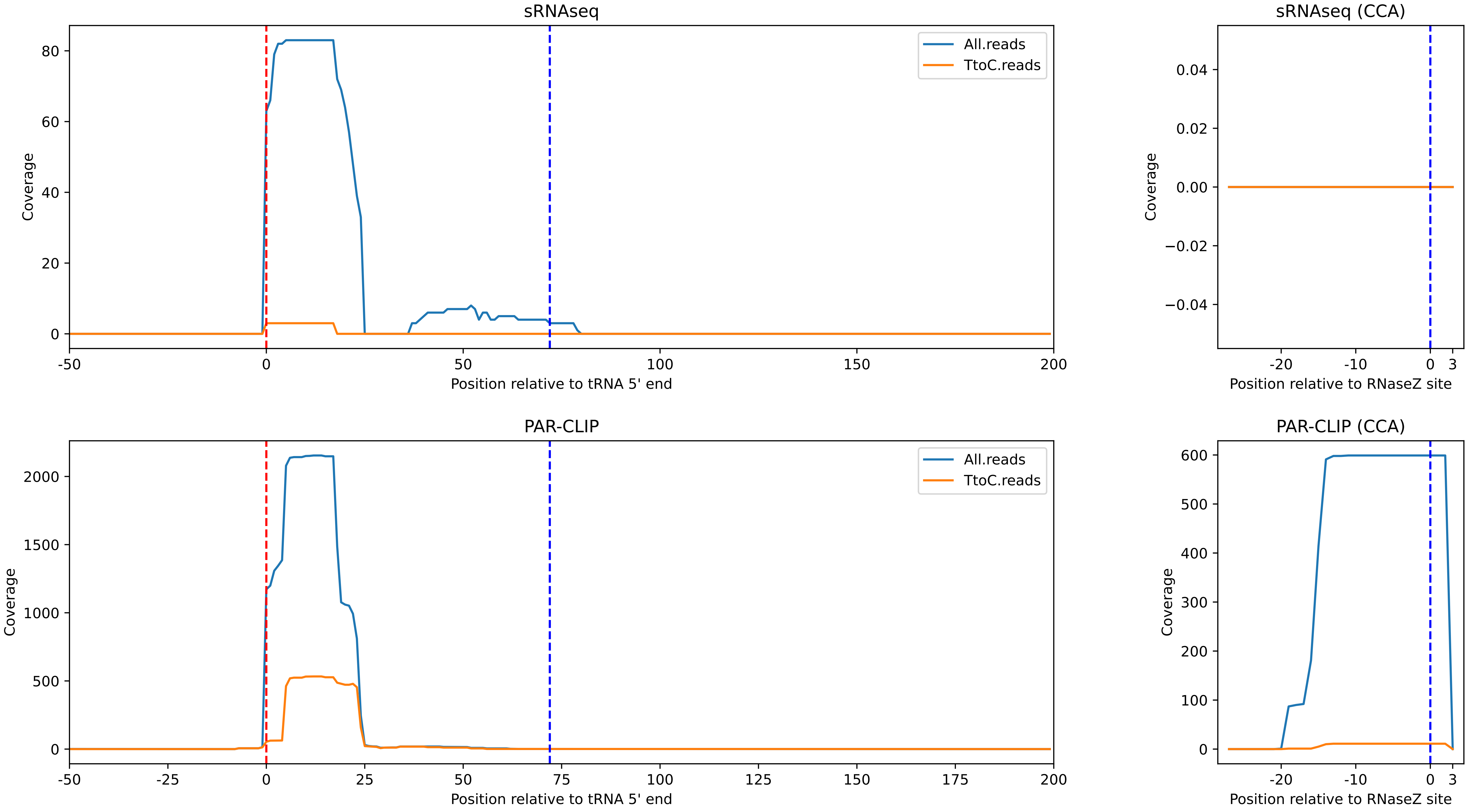

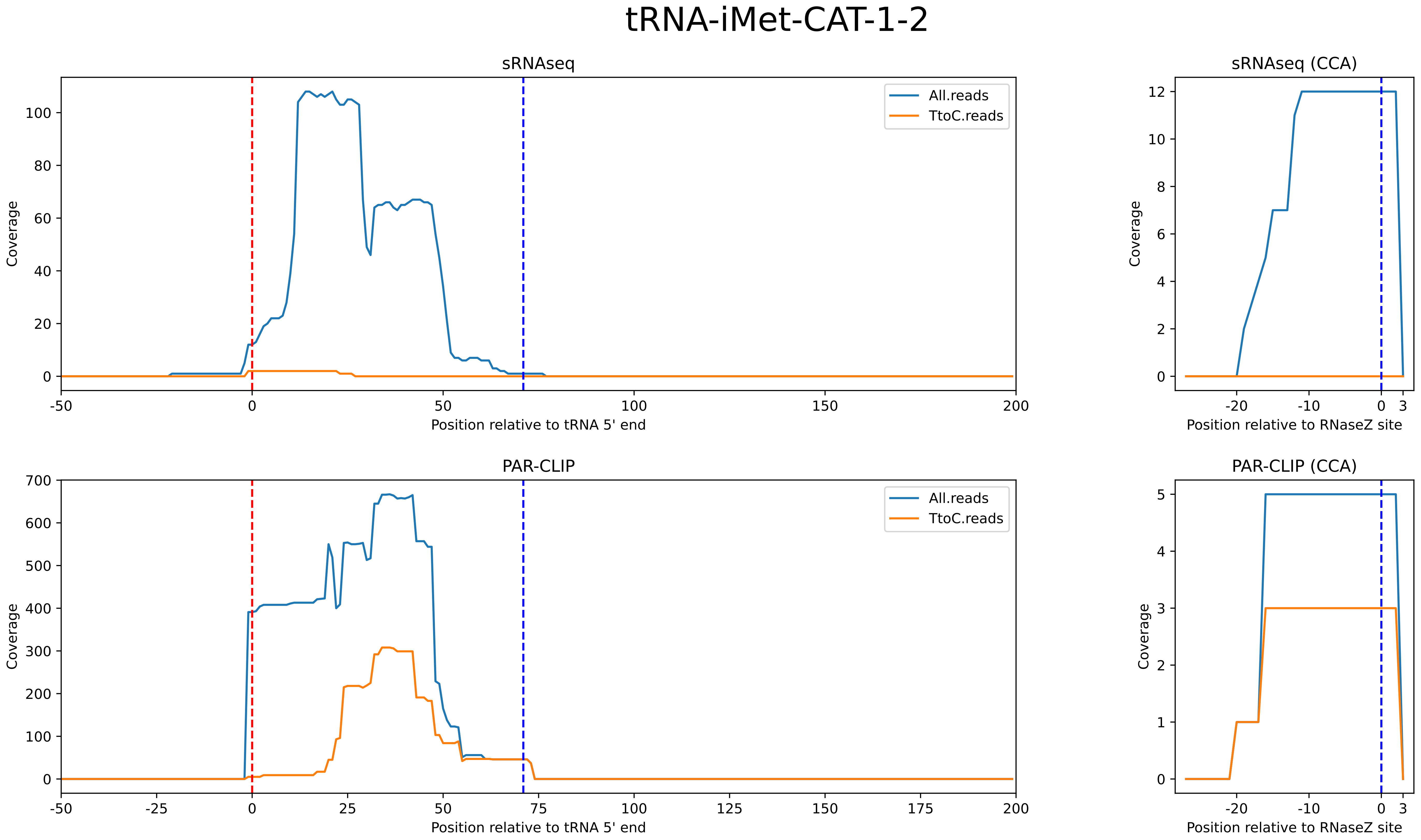
tRNA-iMet-CAT-1-2
sRNAseq
All.reads
TtoC.reads
Coverage
Position relative to tRNA 5' end
sRNAseq (CCA)
Position relative to RNaseZ site
PAR-CLIP
PAR-CLIP (CCA)

# tRNA-Ile-AAT-1-6

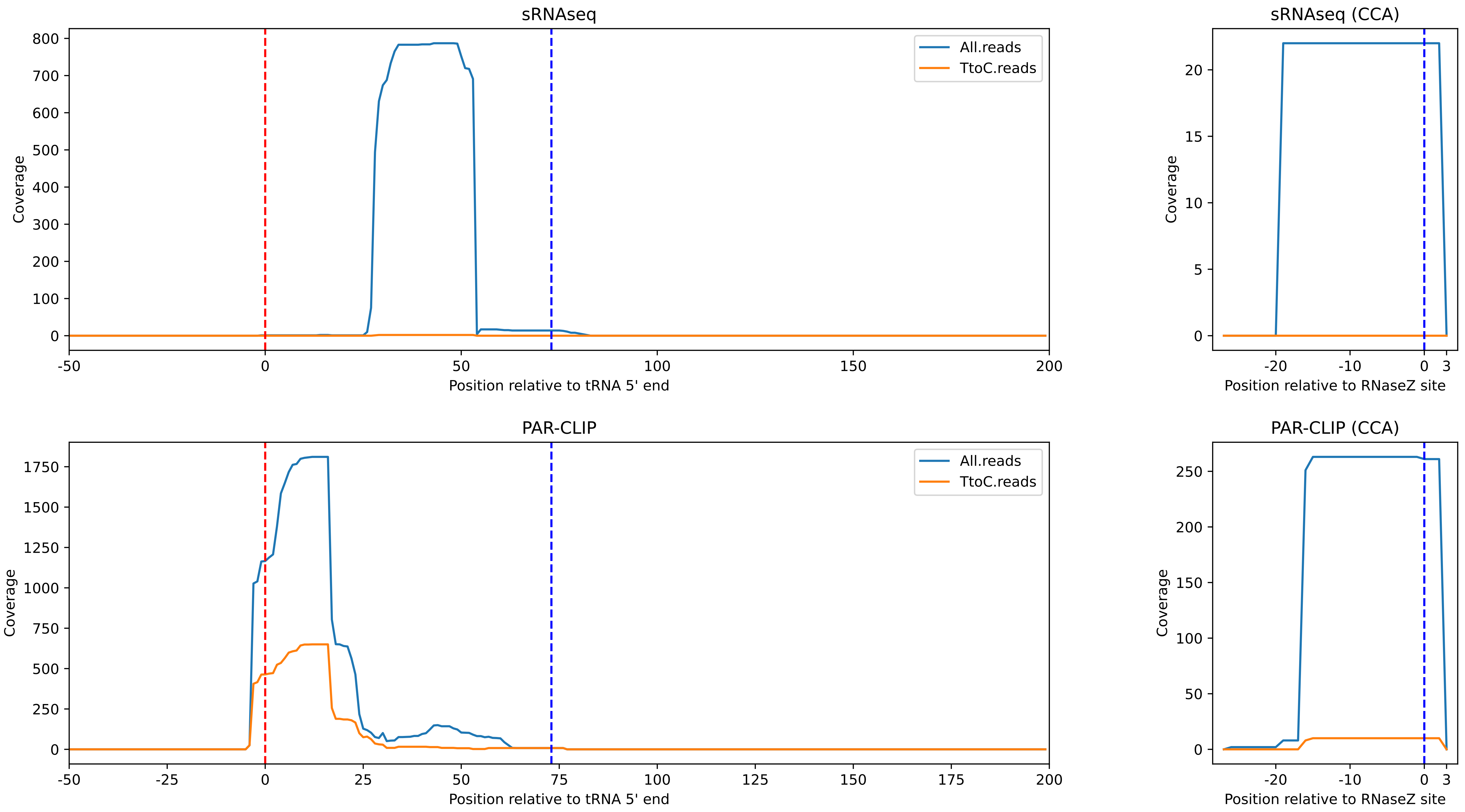

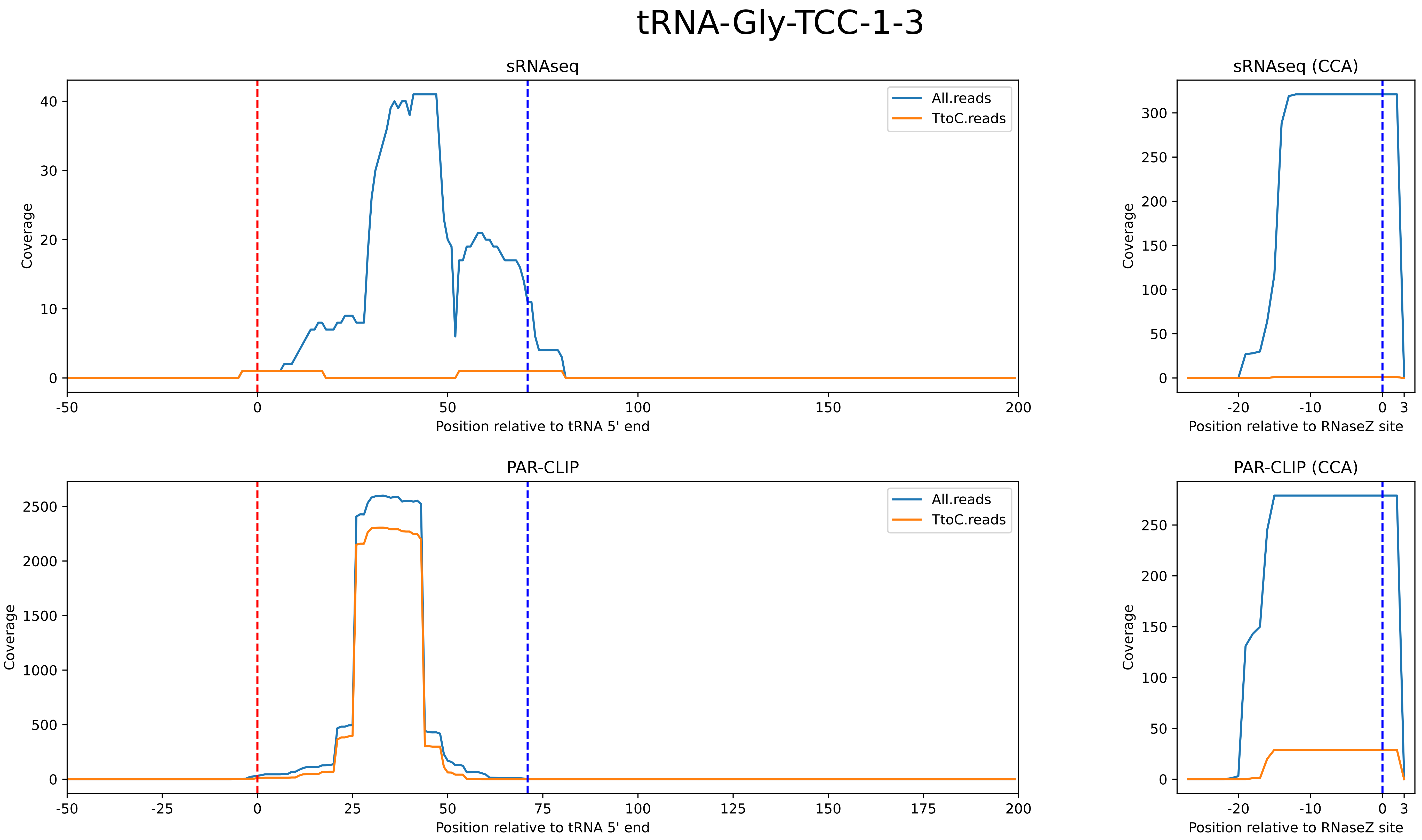
tRNA-Gly-TCC-1-3
sRNAseq
All.reads
TtoC.reads
Coverage
Position relative to tRNA 5' end
-50
0
50
100
150
200
0
10
20
30
40
sRNAseq (CCA)
Position relative to RNaseZ site
-20
-10
0
3
0
50
100
150
200
250
300
PAR-CLIP
-25
25
75
125
175
500
1000
1500
2000
2500
PAR-CLIP (CCA)

# tRNA-Glu-CTC-3-4

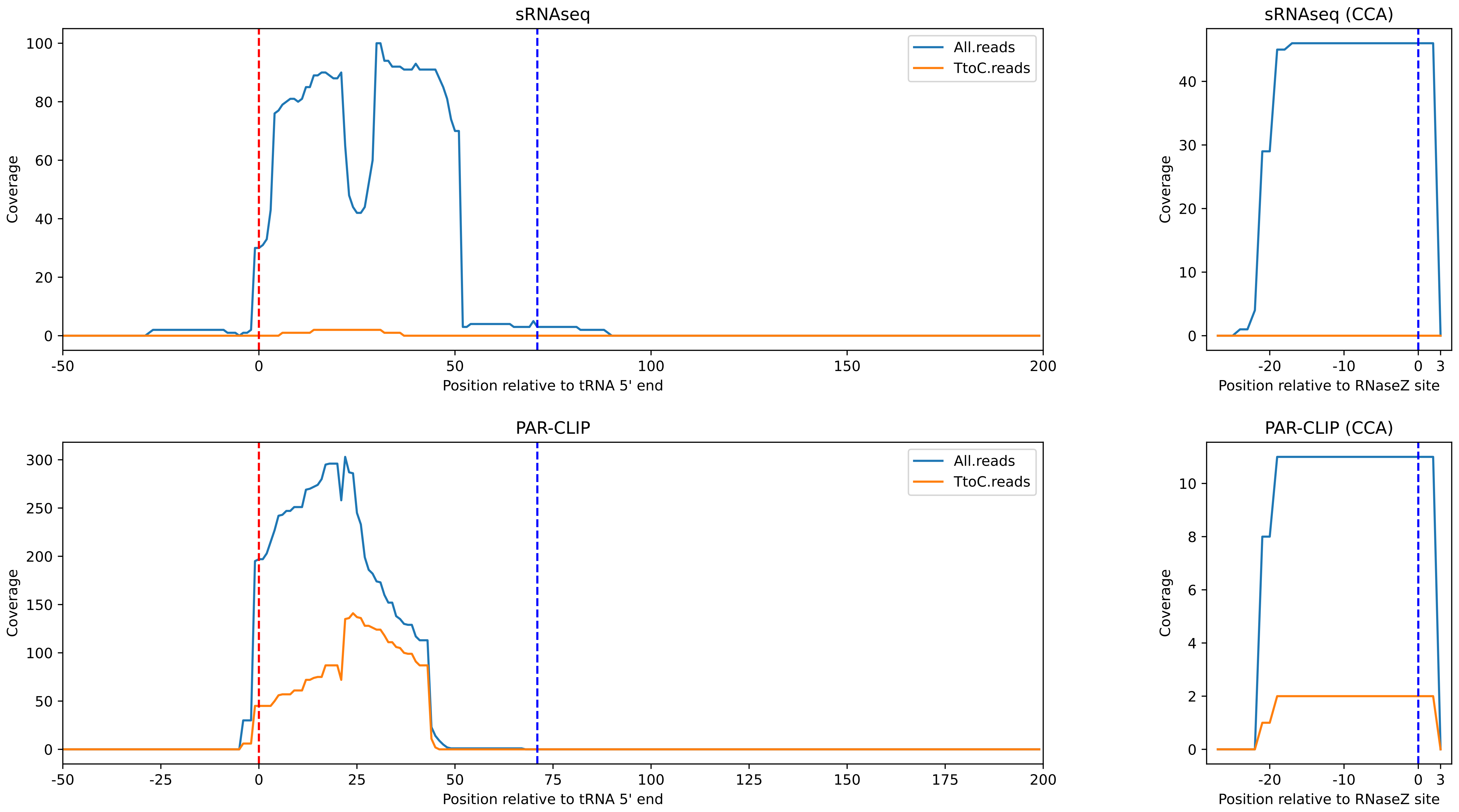

# tRNA-Leu-AAG-1-1

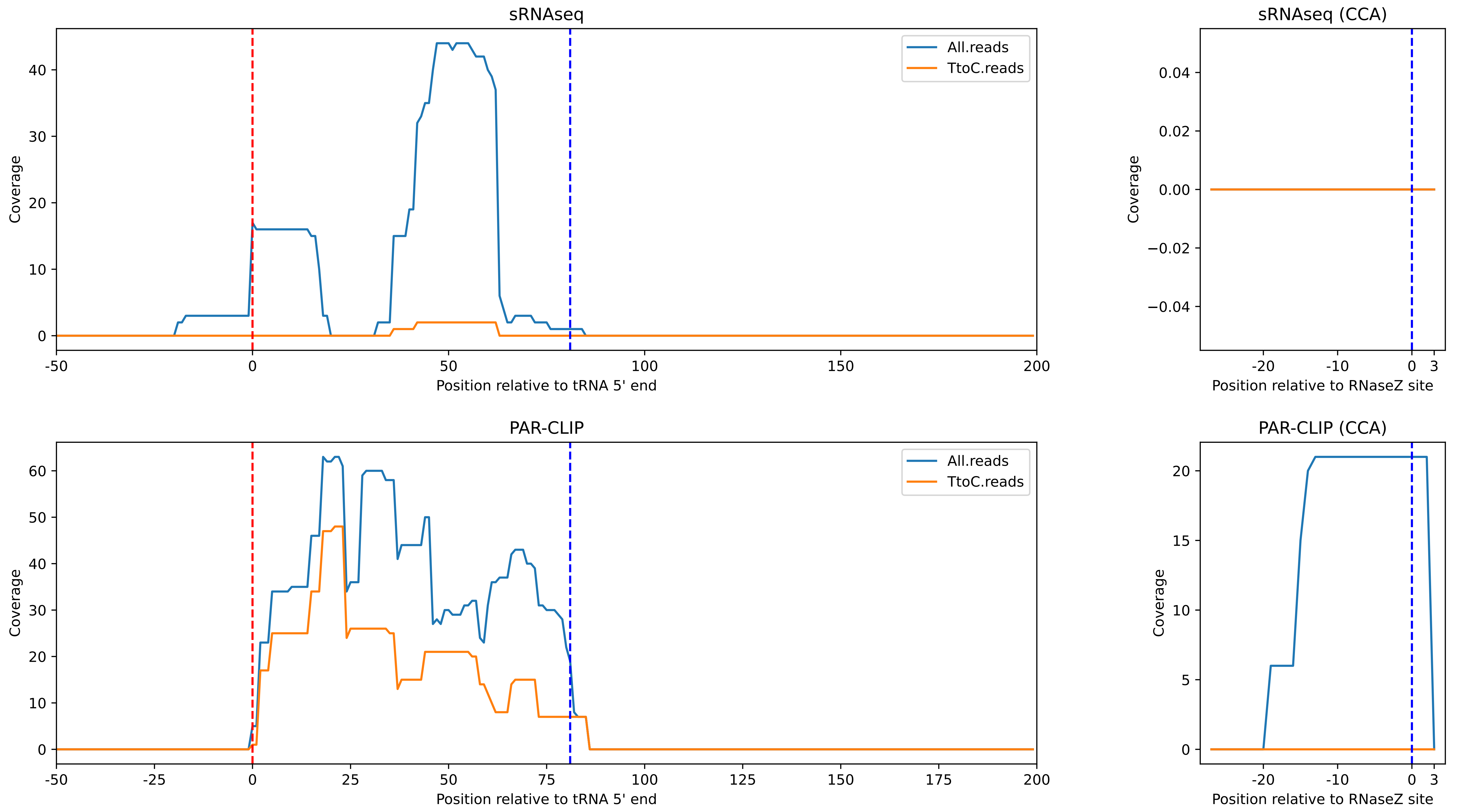

# tRNA-Phe-GAA-1-6

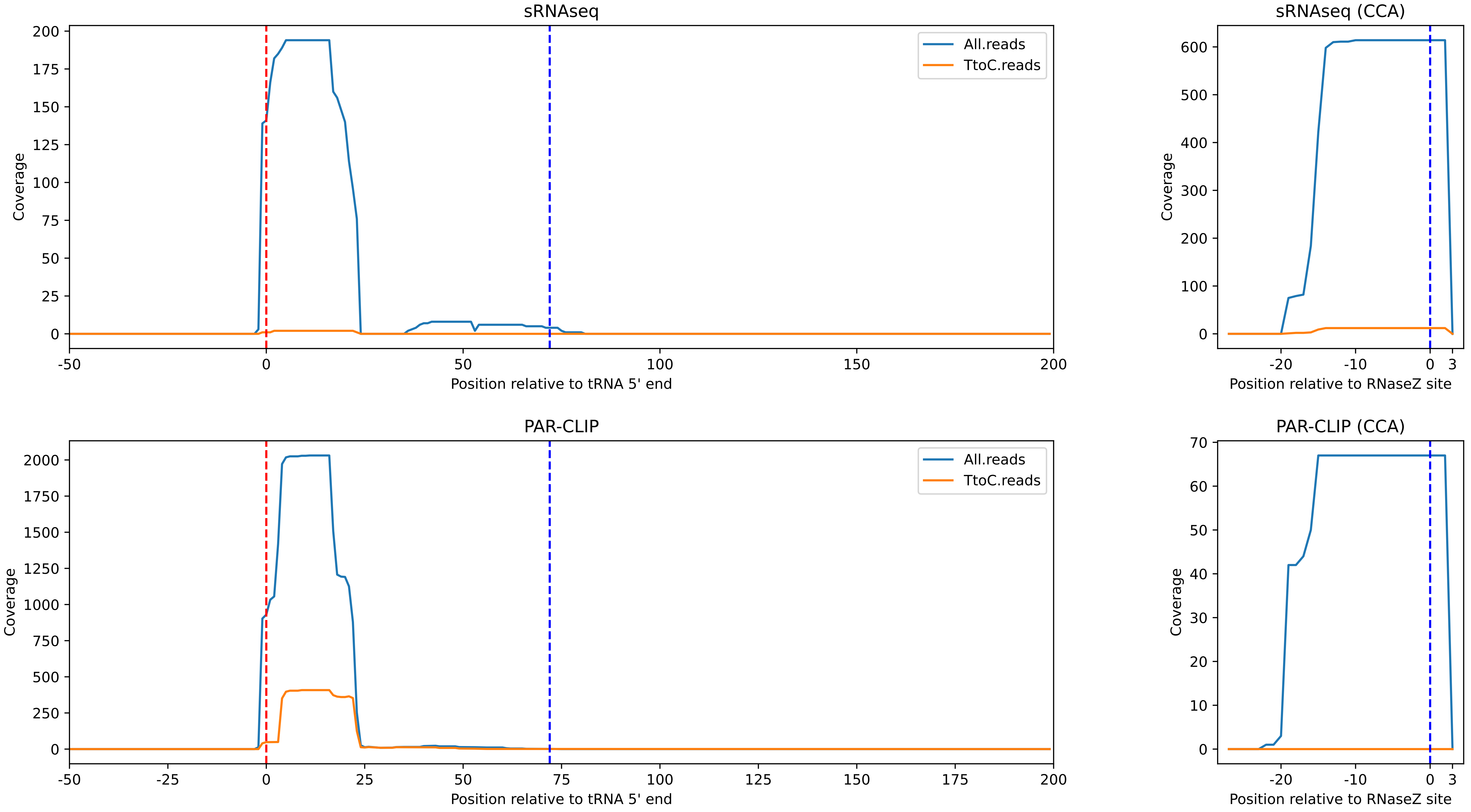

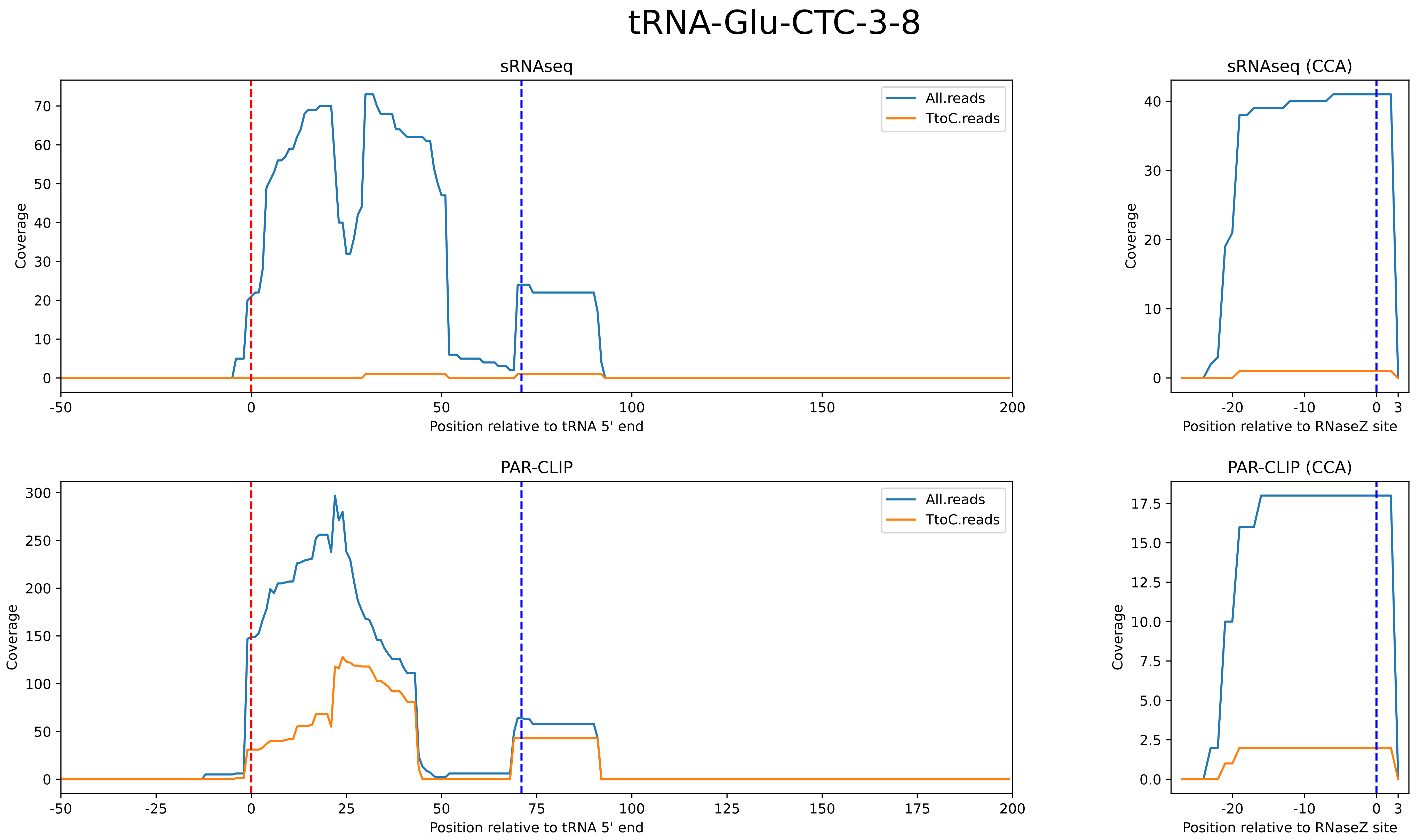
tRNA-Glu-CTC-3-8
sRNAseq
All.reads
TtoC.reads
Coverage
Position relative to tRNA 5' end
sRNAseq (CCA)
Position relative to RNaseZ site
PAR-CLIP
PAR-CLIP (CCA)

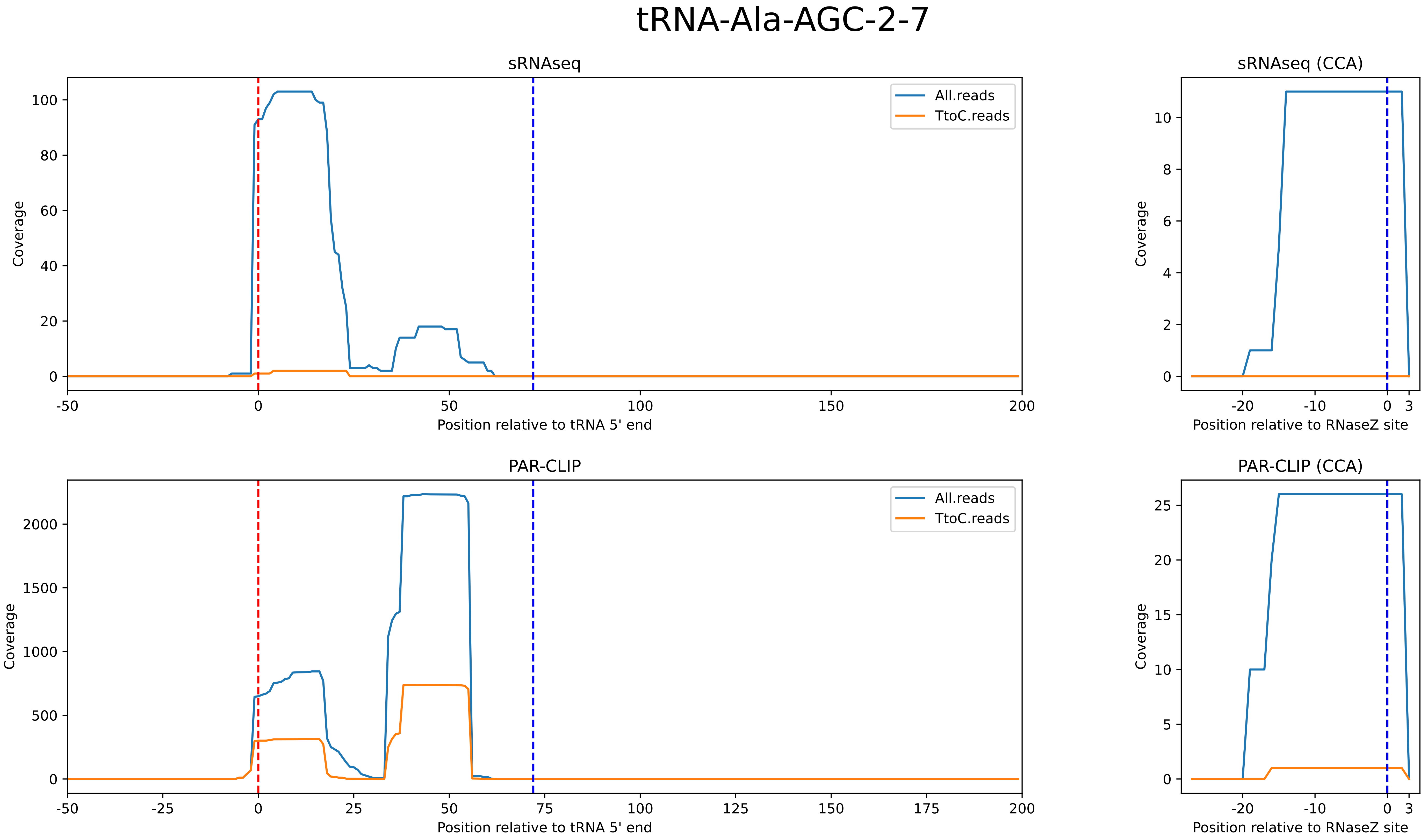
tRNA-Ala-AGC-2-7
sRNAseq
sRNAseq (CCA)
PAR-CLIP
PAR-CLIP (CCA)
All.reads
TtoC.reads
Coverage
Position relative to tRNA 5' end
Position relative to RNaseZ site

# tRNA-Glu-CTC-3-2

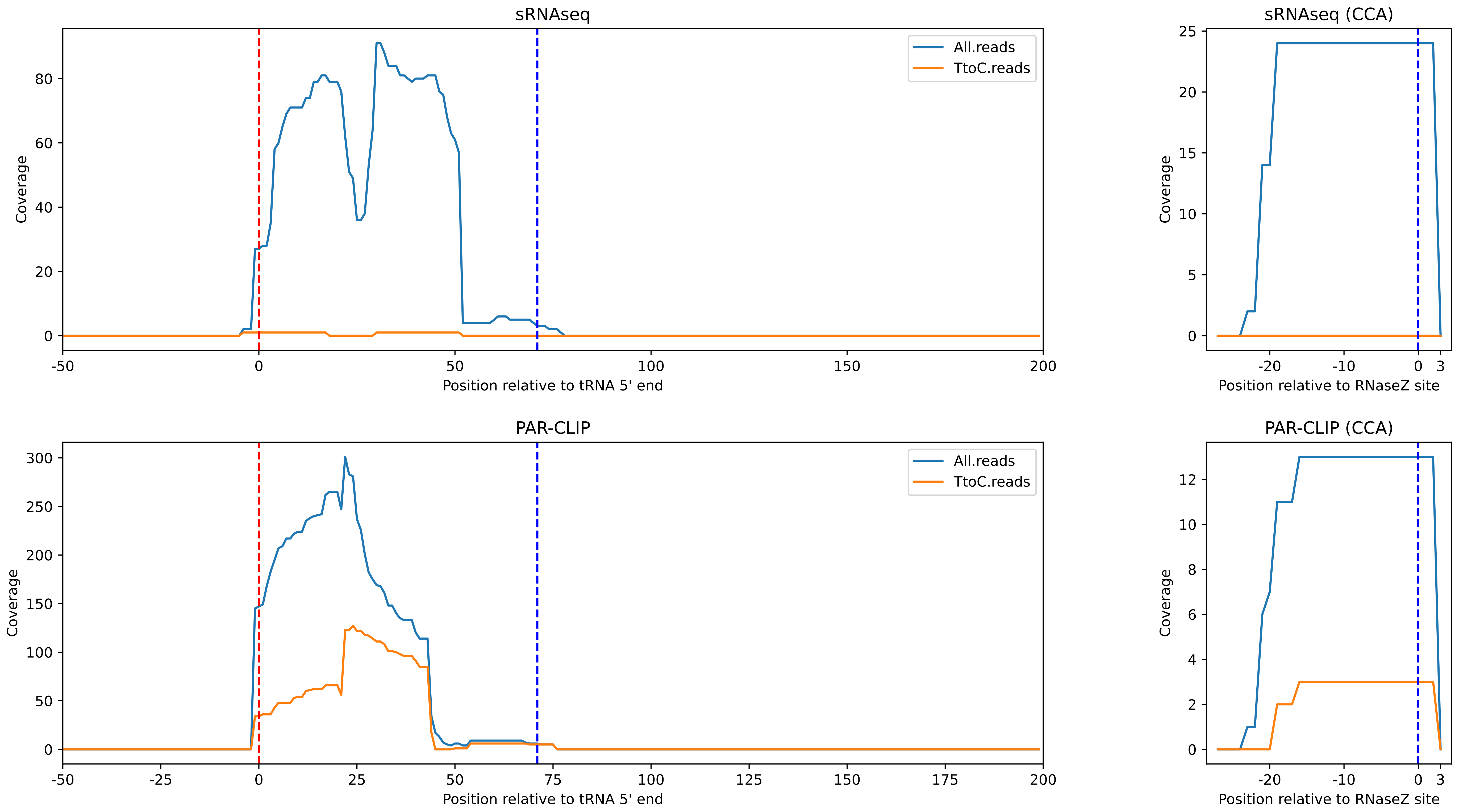

# tRNA-Ser-CGA-1-3

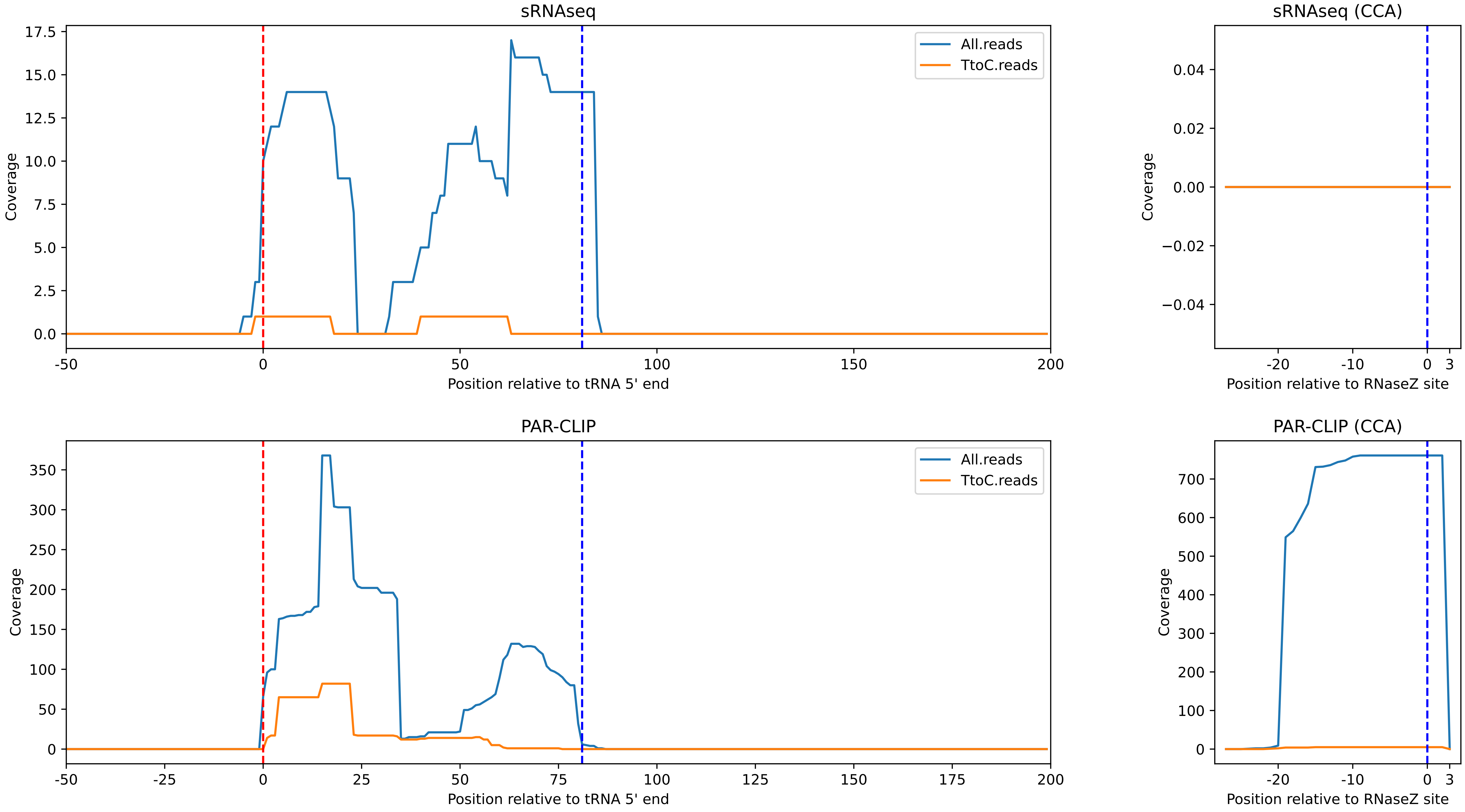

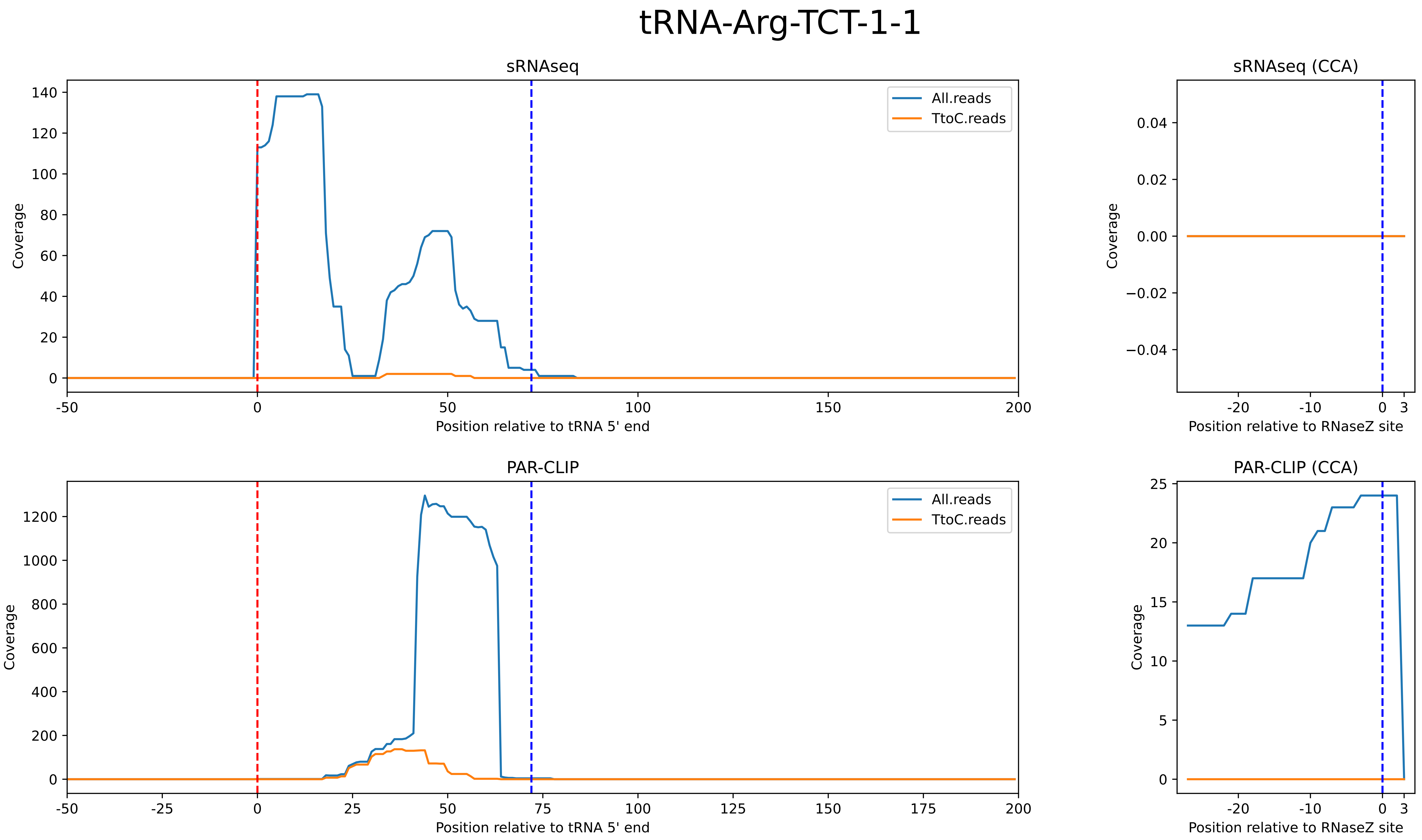

tRNA-Arg-TCT-1-1
sRNAseq
sRNAseq (CCA)
PAR-CLIP
PAR-CLIP (CCA)
All.reads
TtoC.reads
Coverage
Position relative to tRNA 5' end
Position relative to RNaseZ site

# tRNA-Ile-TAT-1-2

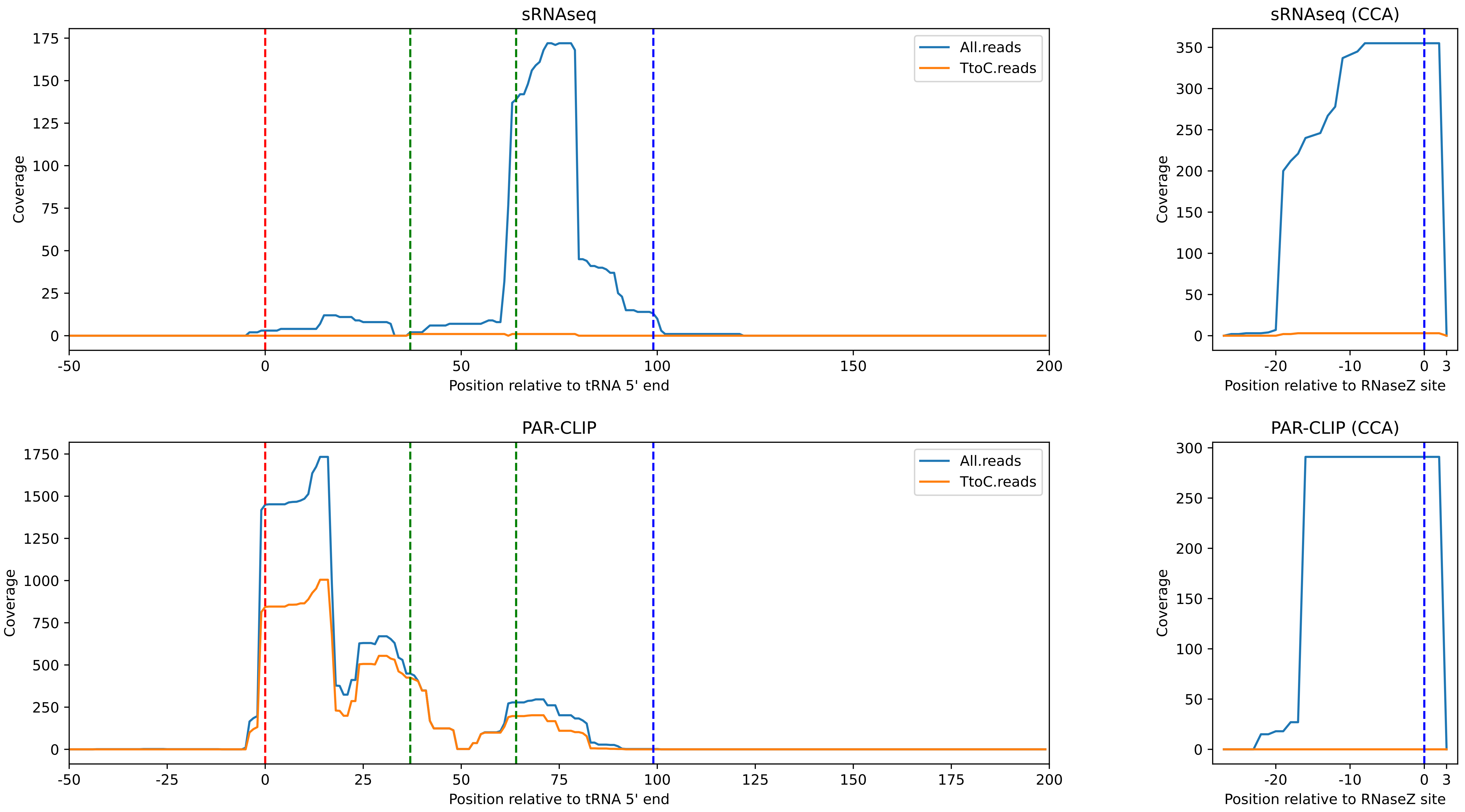

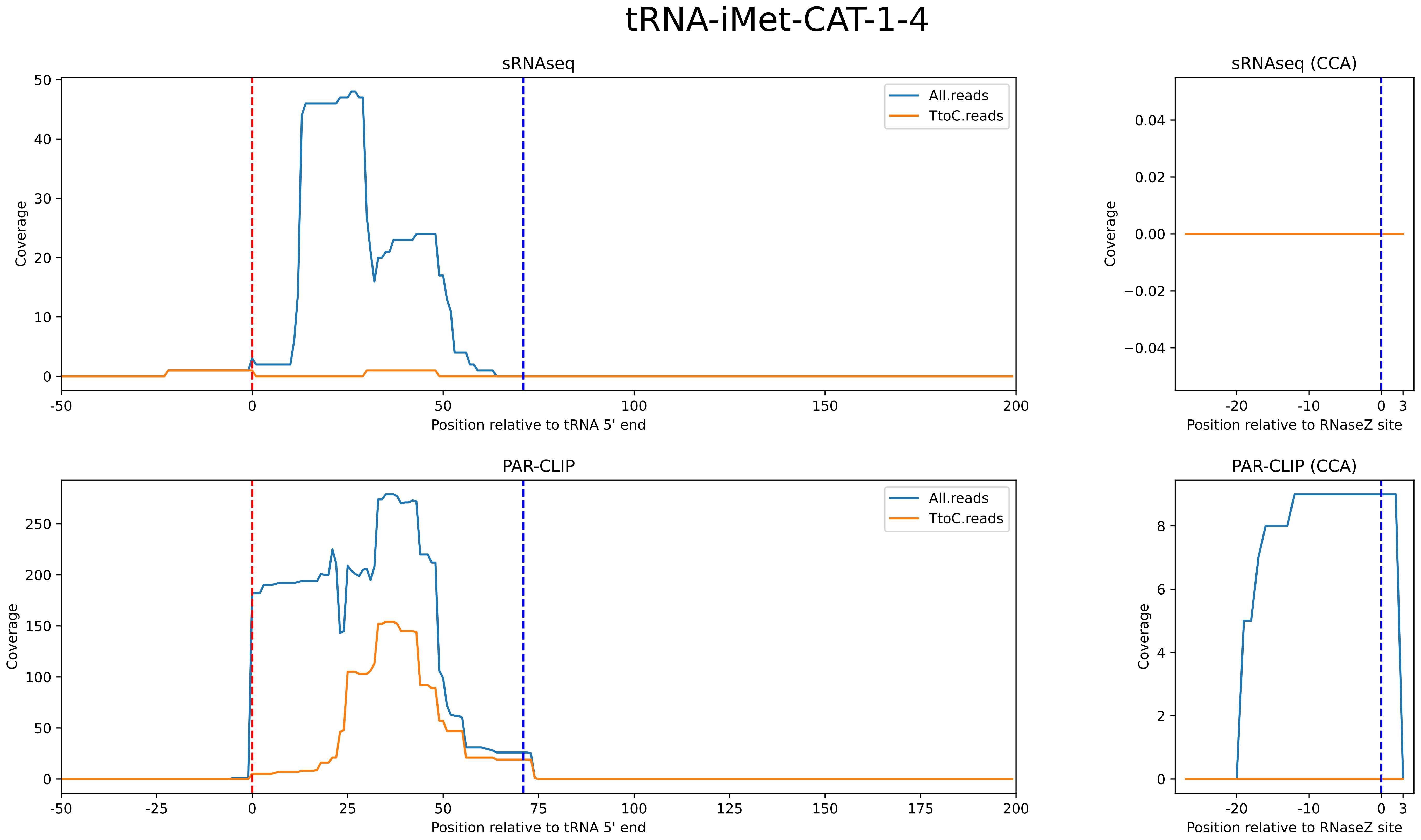
tRNA-iMet-CAT-1-4
sRNAseq
sRNAseq (CCA)
PAR-CLIP
PAR-CLIP (CCA)
All.reads
TtoC.reads
Coverage
Position relative to tRNA 5' end
Position relative to RNaseZ site

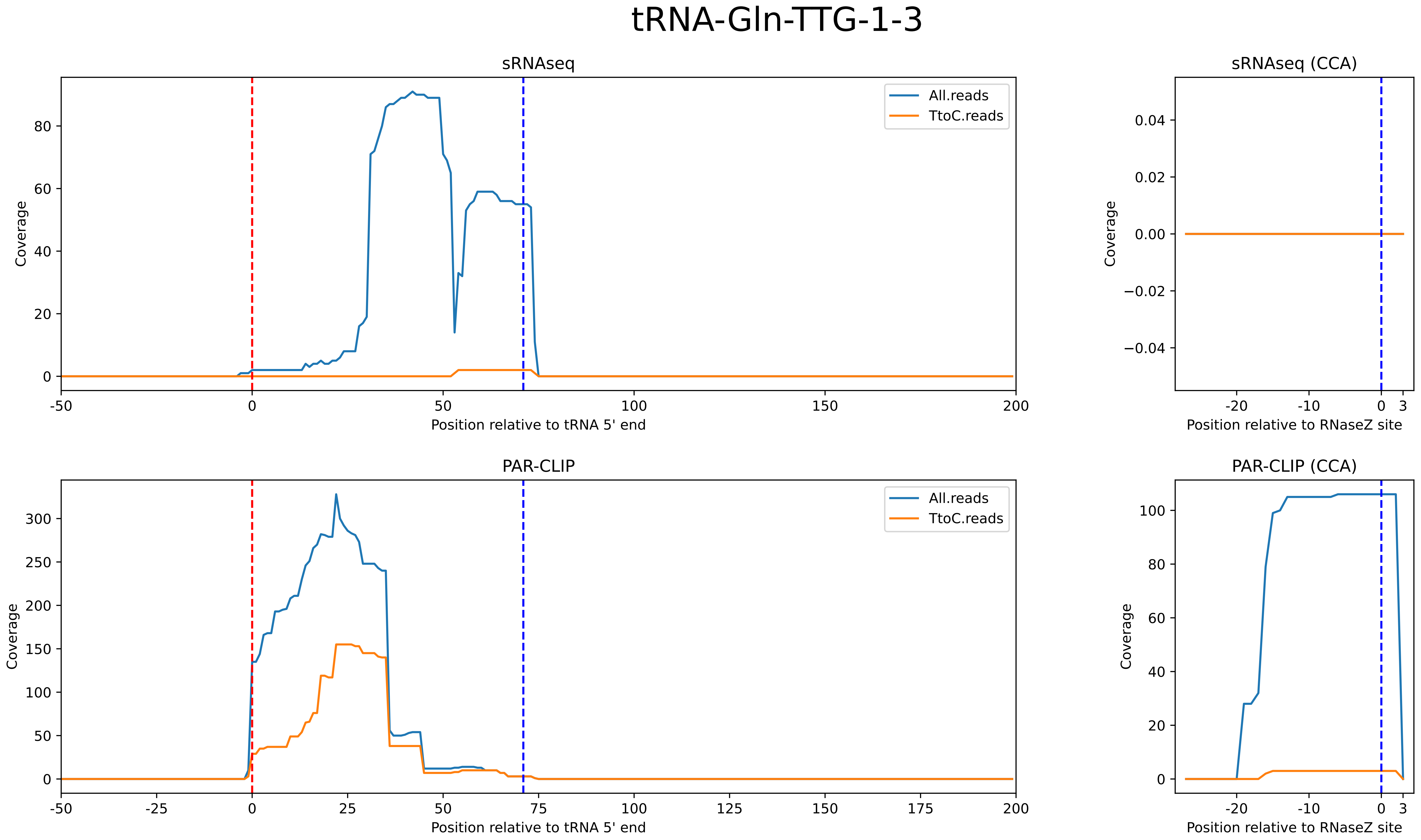

tRNA-Gln-TTG-1-3
sRNAseq
All.reads
TtoC.reads
Coverage
Position relative to tRNA 5' end
sRNAseq (CCA)
Position relative to RNaseZ site
PAR-CLIP
PAR-CLIP (CCA)

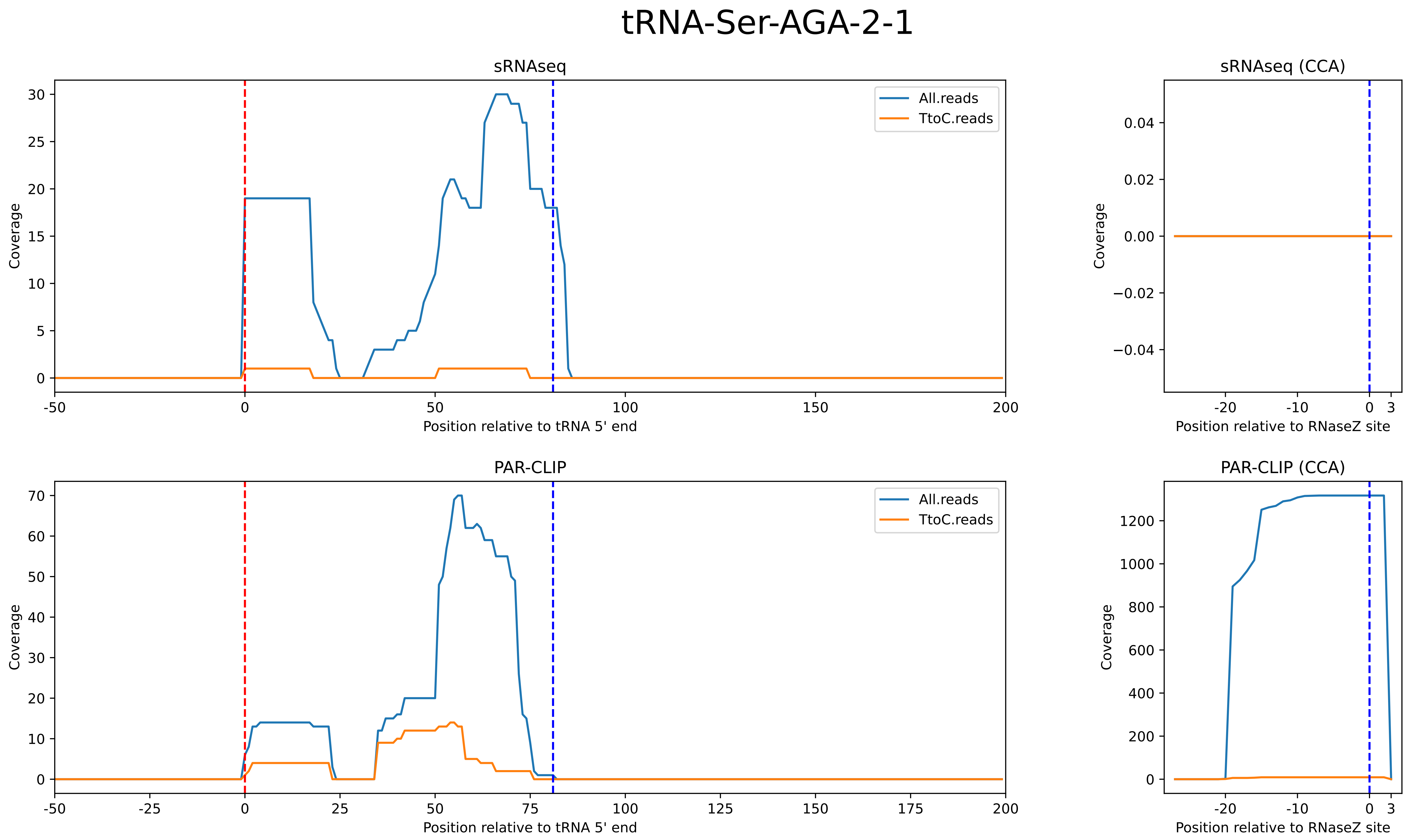
tRNA-Ser-AGA-2-1
sRNAseq
All.reads
TtoC.reads
Coverage
Position relative to tRNA 5' end
sRNAseq (CCA)
Position relative to RNaseZ site
PAR-CLIP
PAR-CLIP (CCA)
0
25
50
75
100
125
150
175
200
-50
-25
-20
-10
3
0.04
0.02
0.00
−0.02
−0.04
1200
1000
800
600
400
200
70
60
40
30
20
10
15
5

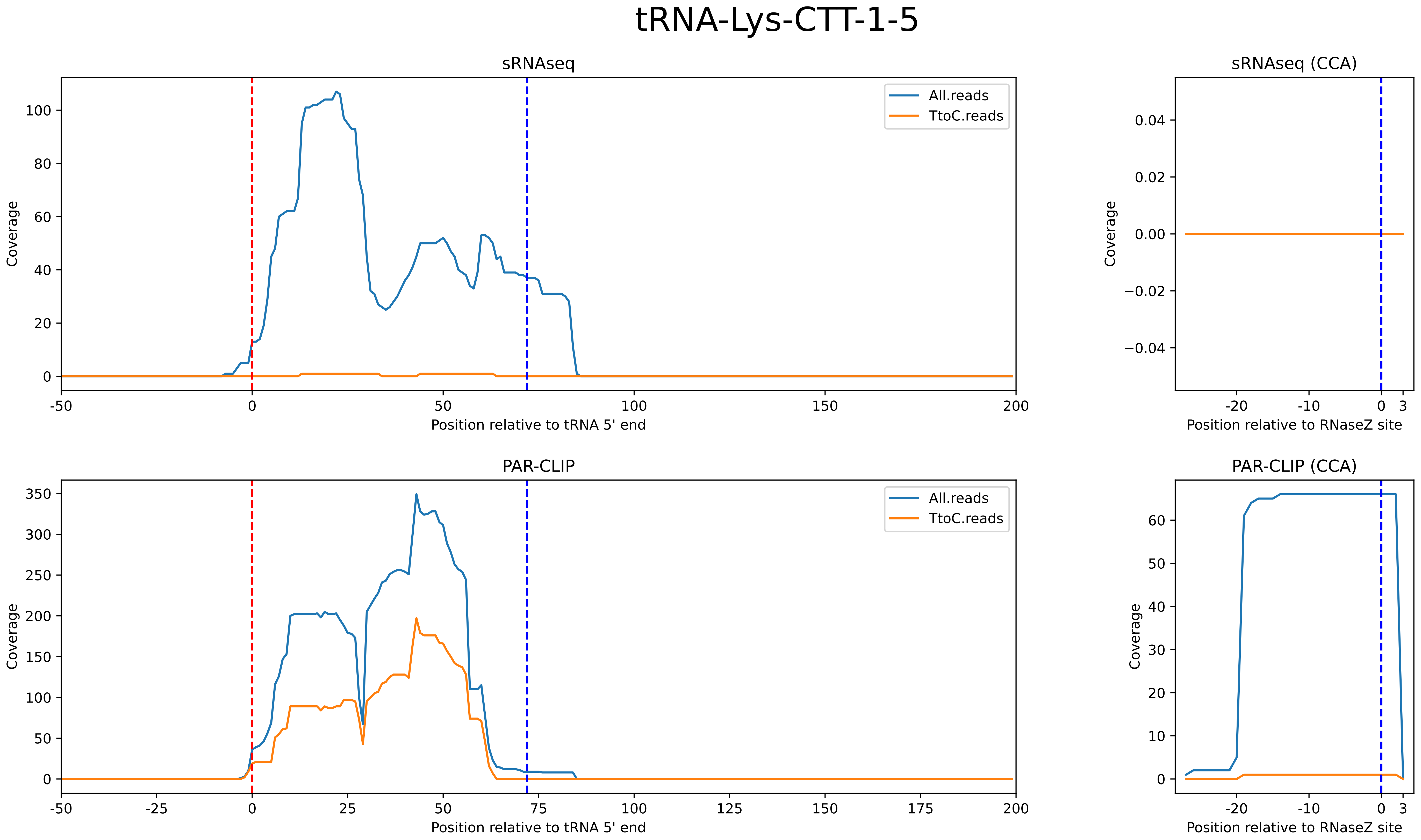

tRNA-Lys-CTT-1-5
sRNAseq
sRNAseq (CCA)
PAR-CLIP
PAR-CLIP (CCA)
All.reads
TtoC.reads
Coverage
Position relative to tRNA 5' end
Position relative to RNaseZ site

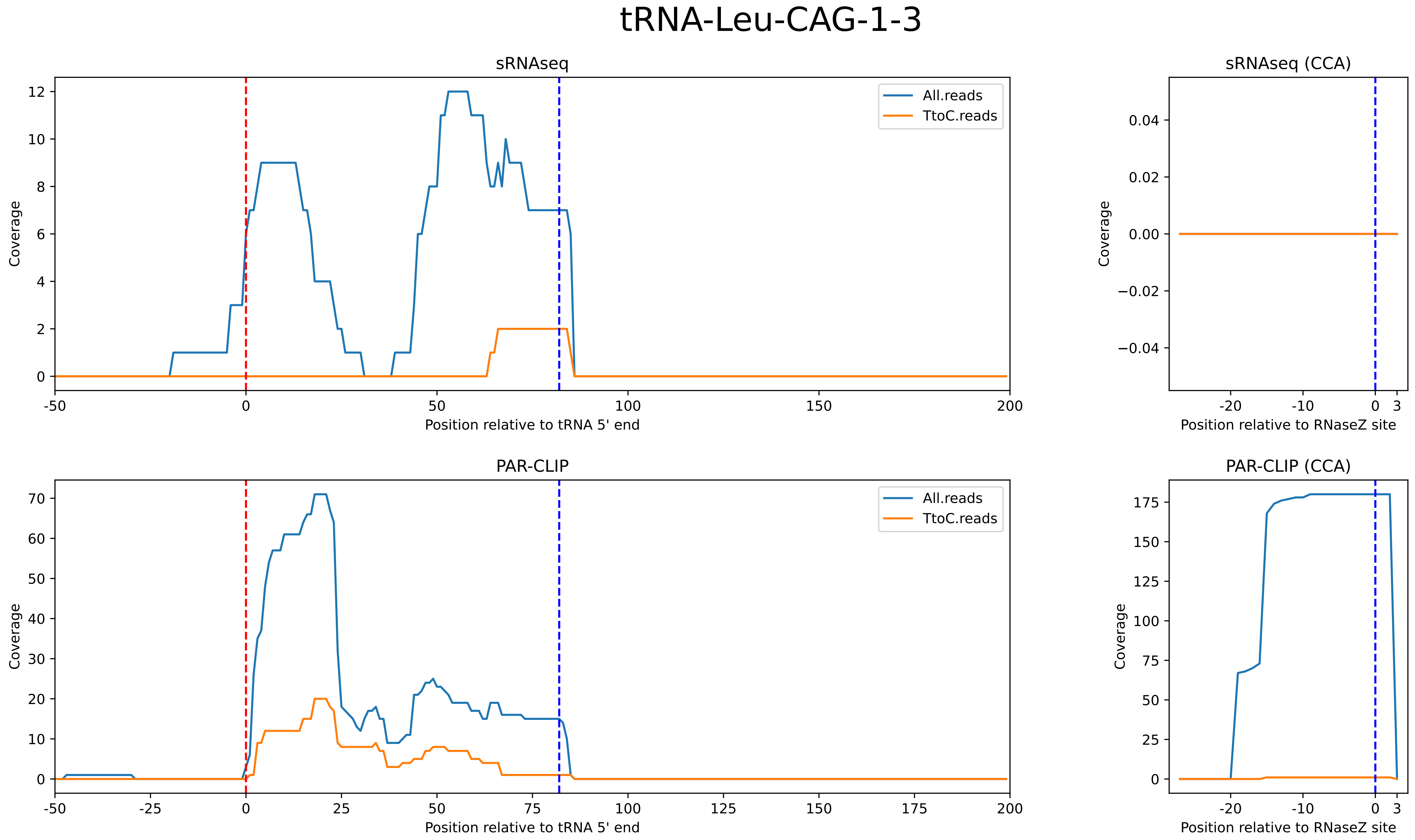

tRNA-Leu-CAG-1-3
sRNAseq
All.reads
TtoC.reads
Coverage
Position relative to tRNA 5' end
sRNAseq (CCA)
Position relative to RNaseZ site
PAR-CLIP
PAR-CLIP (CCA)

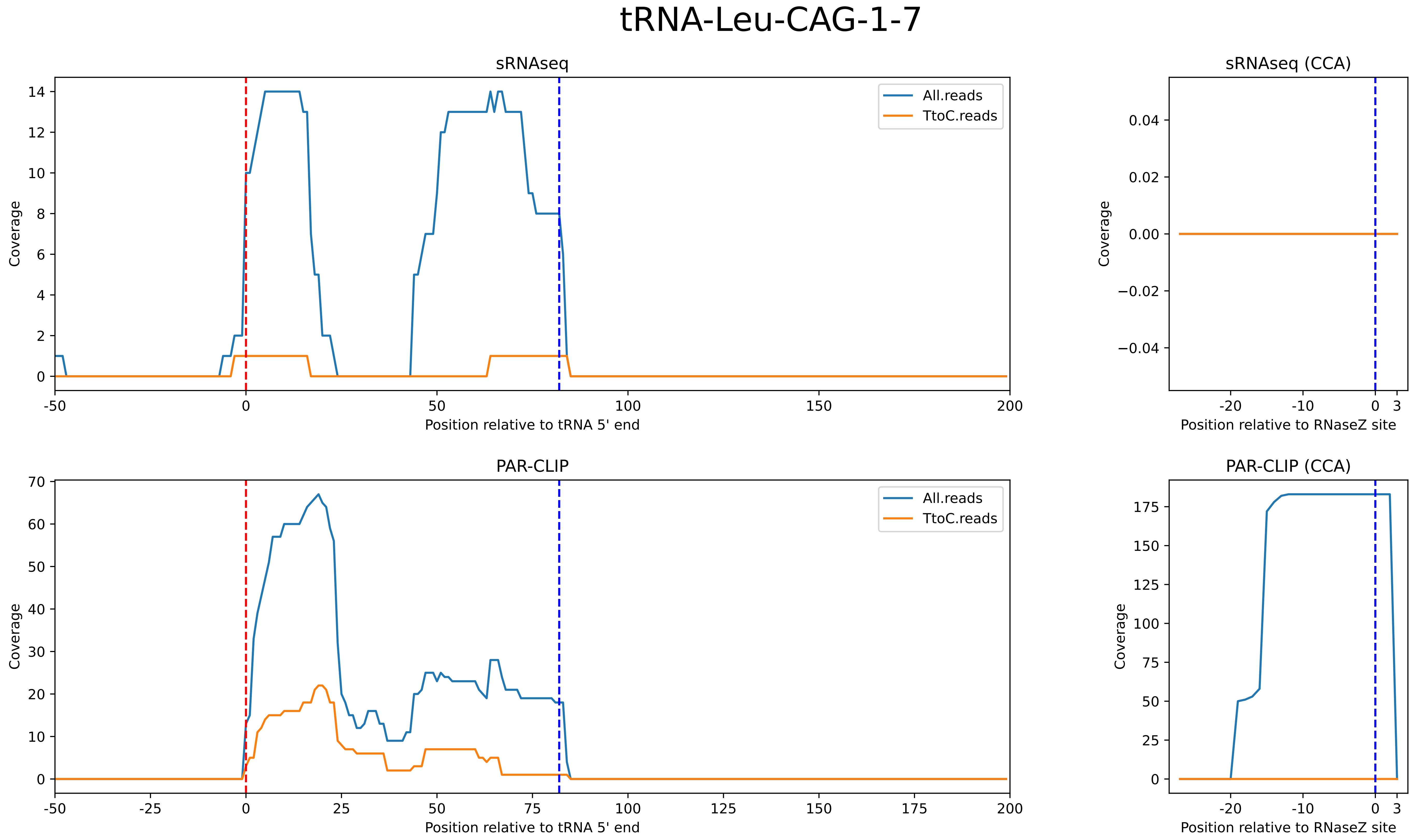

tRNA-Leu-CAG-1-7
sRNAseq
All.reads
TtoC.reads
Coverage
Position relative to tRNA 5' end
sRNAseq (CCA)
Position relative to RNaseZ site
PAR-CLIP
PAR-CLIP (CCA)

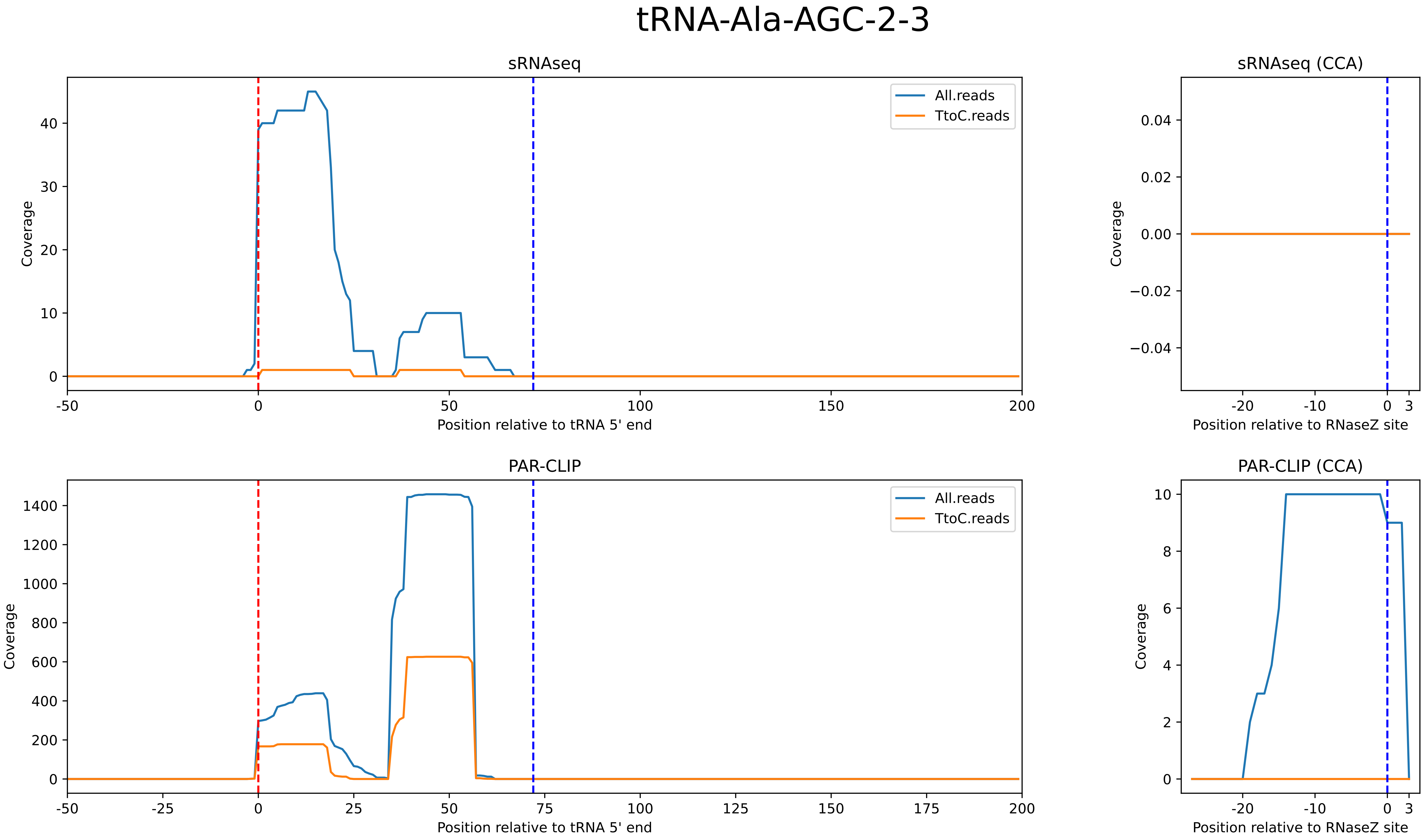

tRNA-Ala-AGC-2-3
sRNAseq
All.reads
TtoC.reads
Coverage
Position relative to tRNA 5' end
sRNAseq (CCA)
Position relative to RNaseZ site
PAR-CLIP
PAR-CLIP (CCA)

# tRNA-Asp-GTC-1-9

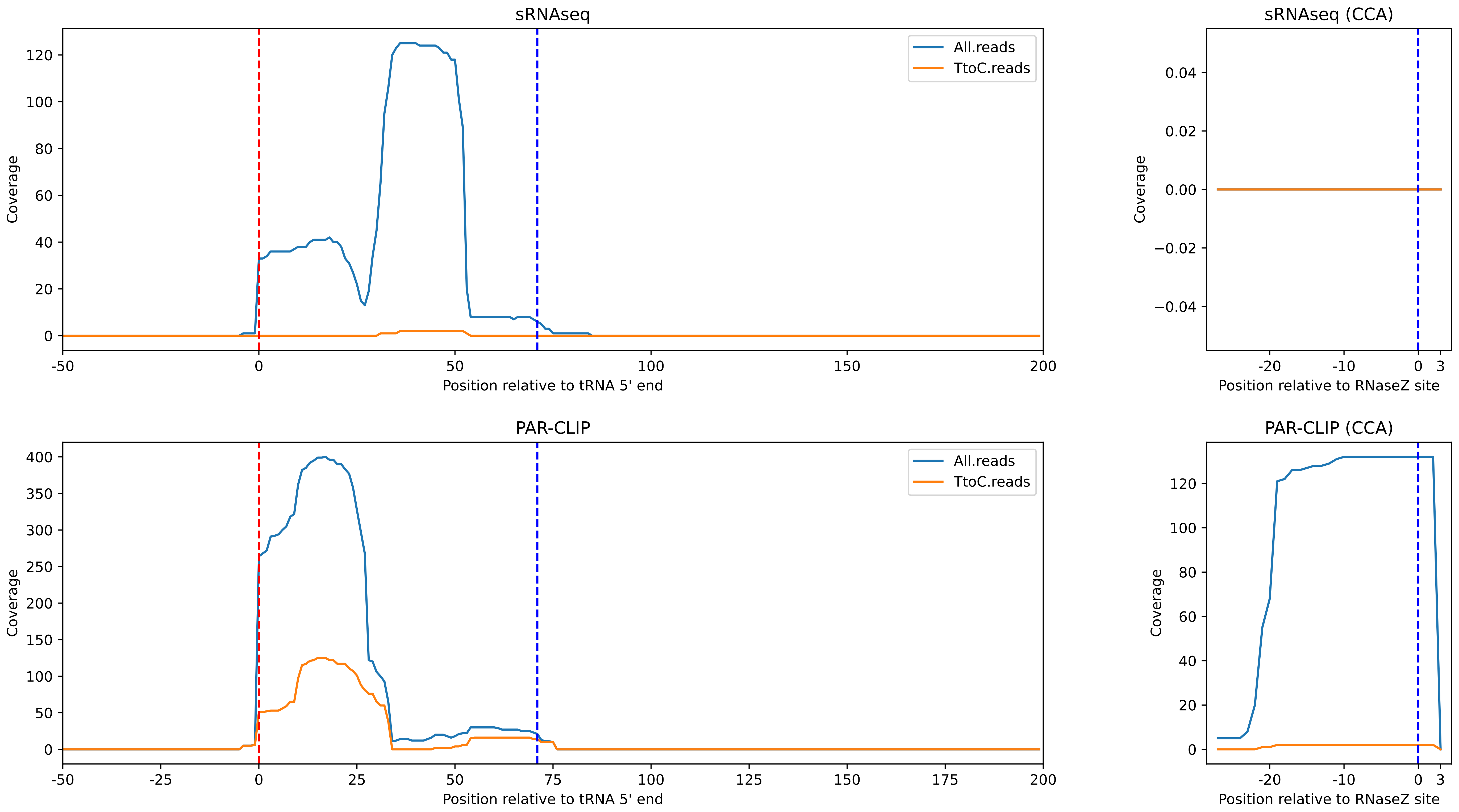

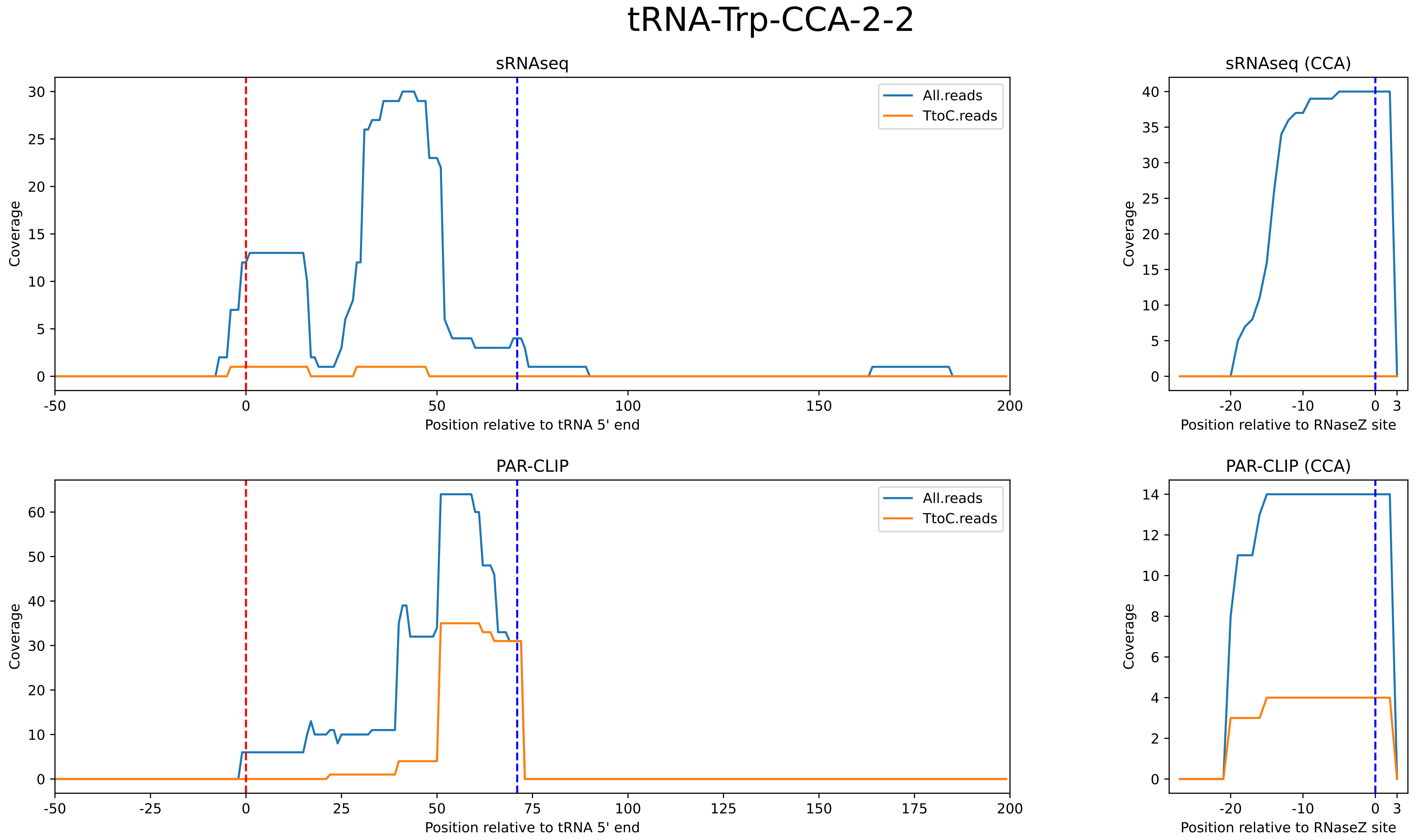
tRNA-Trp-CCA-2-2
sRNAseq
sRNAseq (CCA)
PAR-CLIP
PAR-CLIP (CCA)
All.reads
TtoC.reads
Coverage
Position relative to tRNA 5' end
Position relative to RNaseZ site

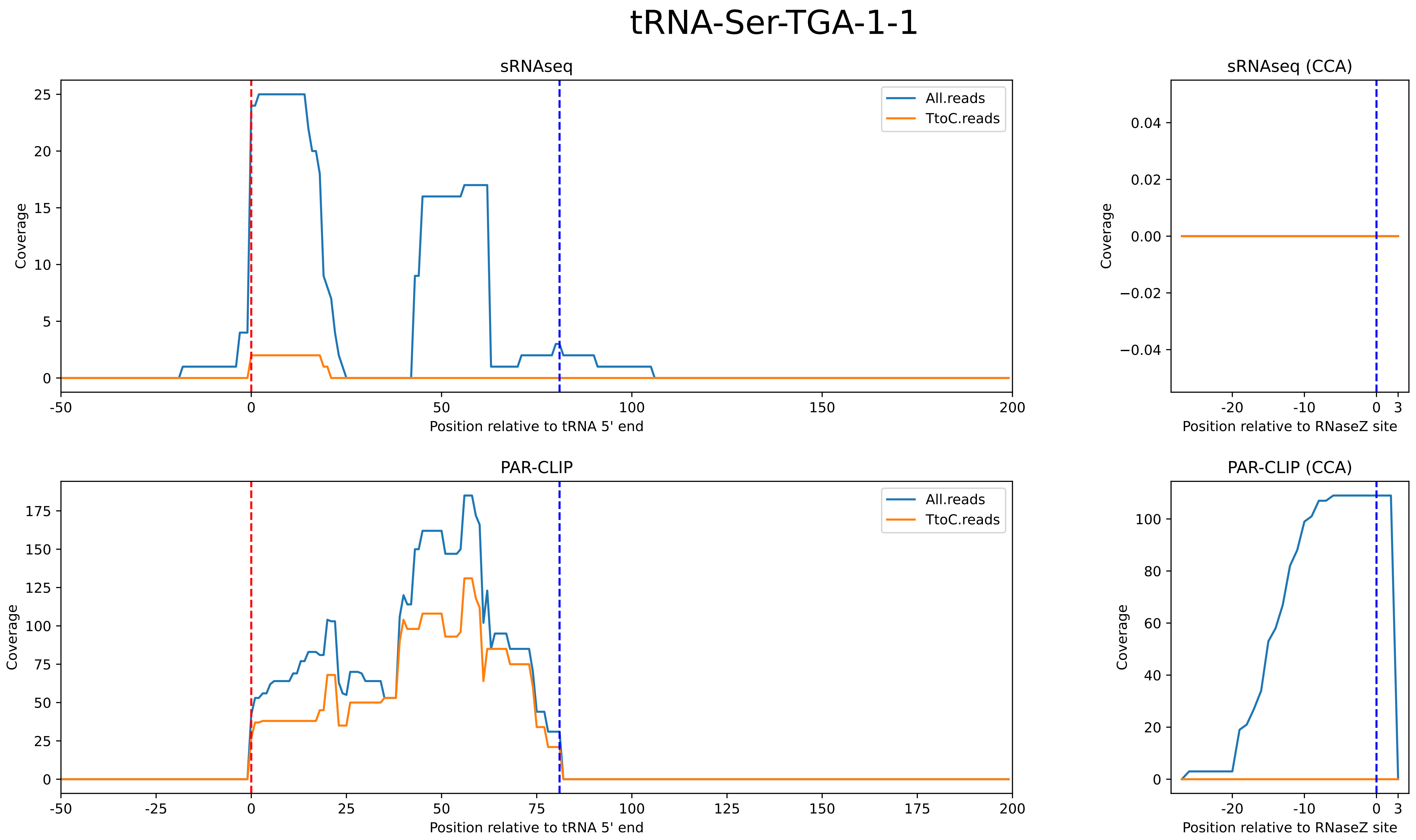

tRNA-Ser-TGA-1-1
sRNAseq
sRNAseq (CCA)
PAR-CLIP
PAR-CLIP (CCA)
All.reads
TtoC.reads
Coverage
Position relative to tRNA 5' end
Position relative to RNaseZ site

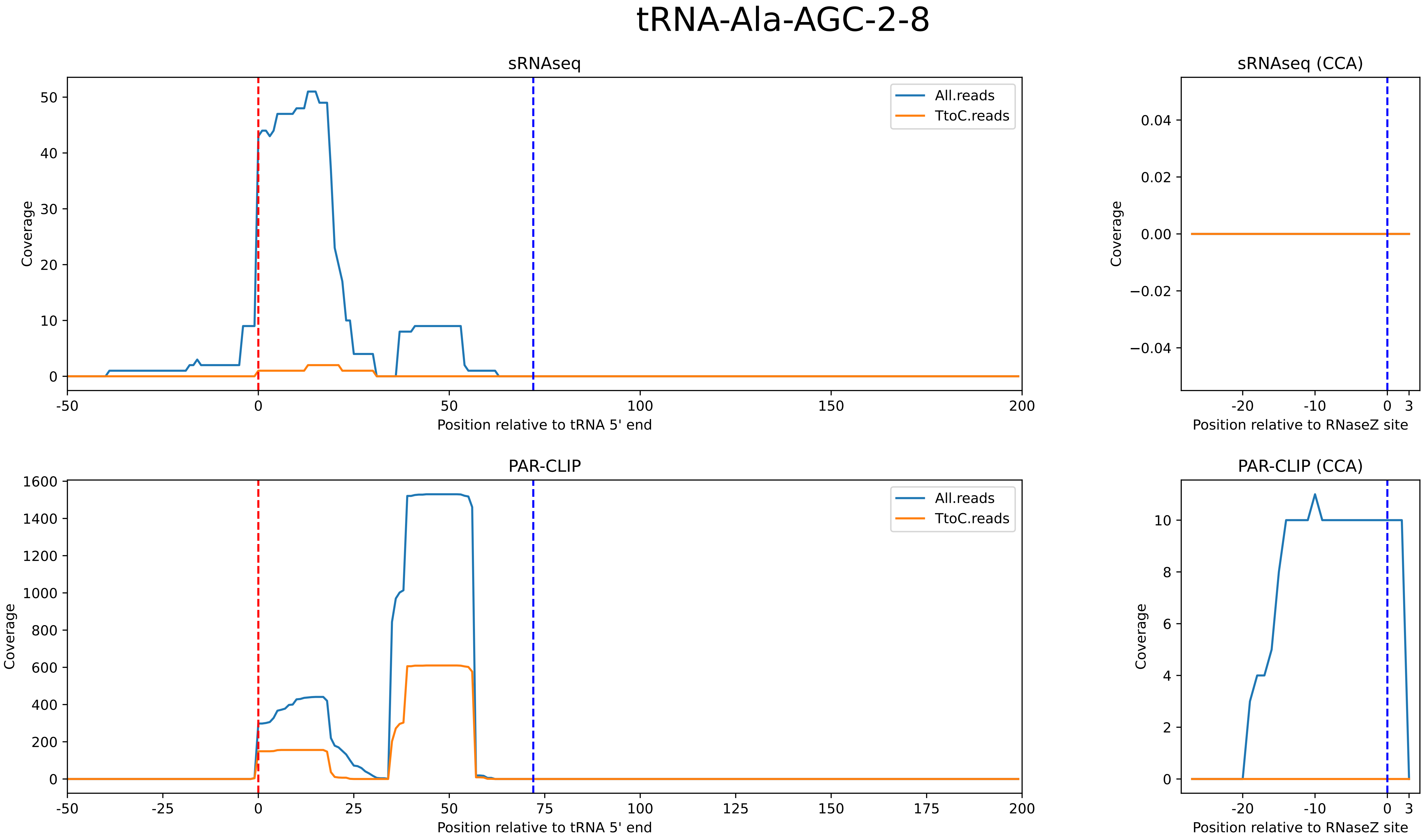

tRNA-Ala-AGC-2-8
sRNAseq
sRNAseq (CCA)
PAR-CLIP
PAR-CLIP (CCA)
All.reads
TtoC.reads
Coverage
Position relative to tRNA 5' end
Position relative to RNaseZ site

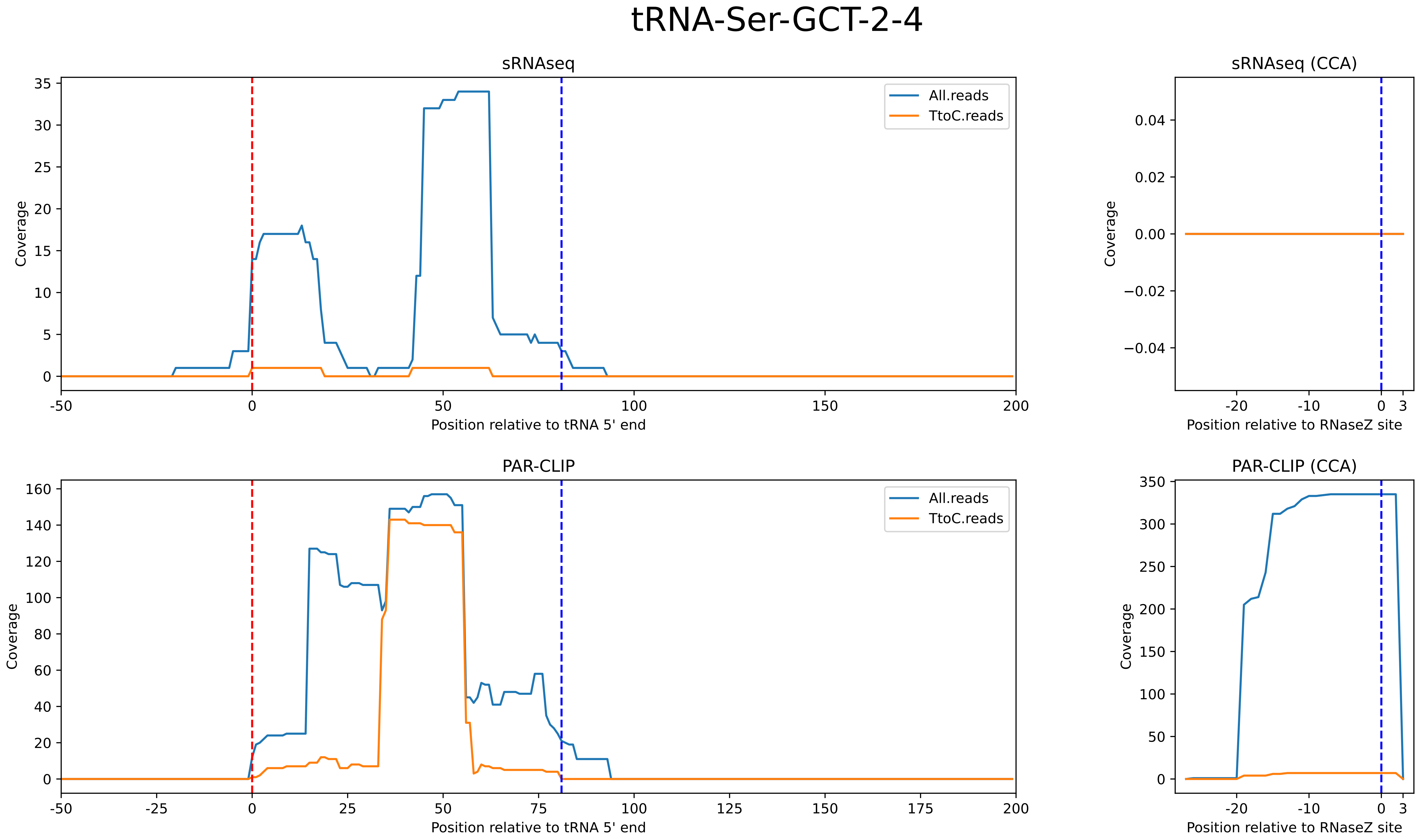

tRNA-Ser-GCT-2-4
sRNAseq
All.reads
TtoC.reads
Coverage
Position relative to tRNA 5' end
sRNAseq (CCA)
Position relative to RNaseZ site
PAR-CLIP
PAR-CLIP (CCA)

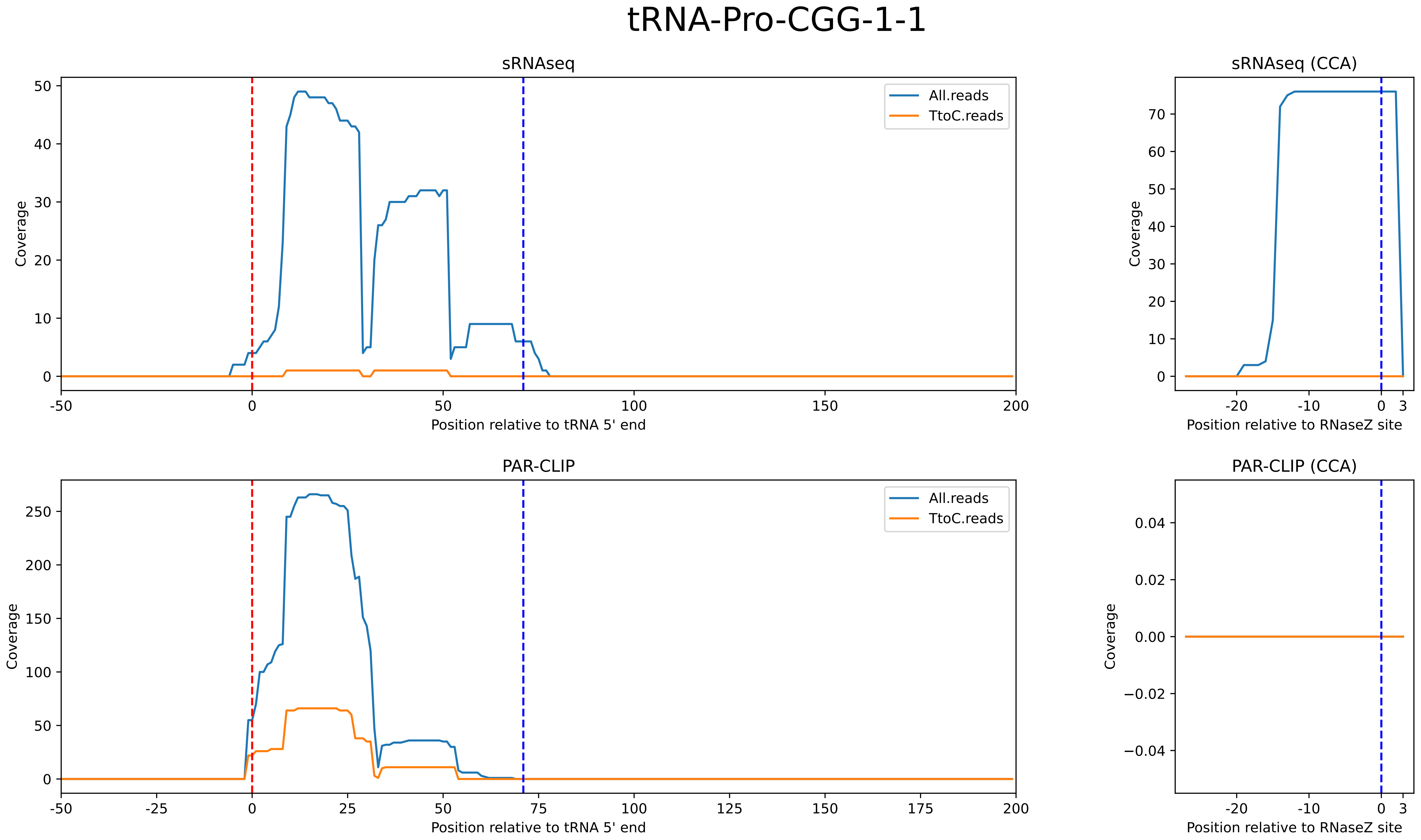
tRNA-Pro-CGG-1-1
sRNAseq
All.reads
TtoC.reads
Coverage
Position relative to tRNA 5' end
sRNAseq (CCA)
Position relative to RNaseZ site
PAR-CLIP
PAR-CLIP (CCA)

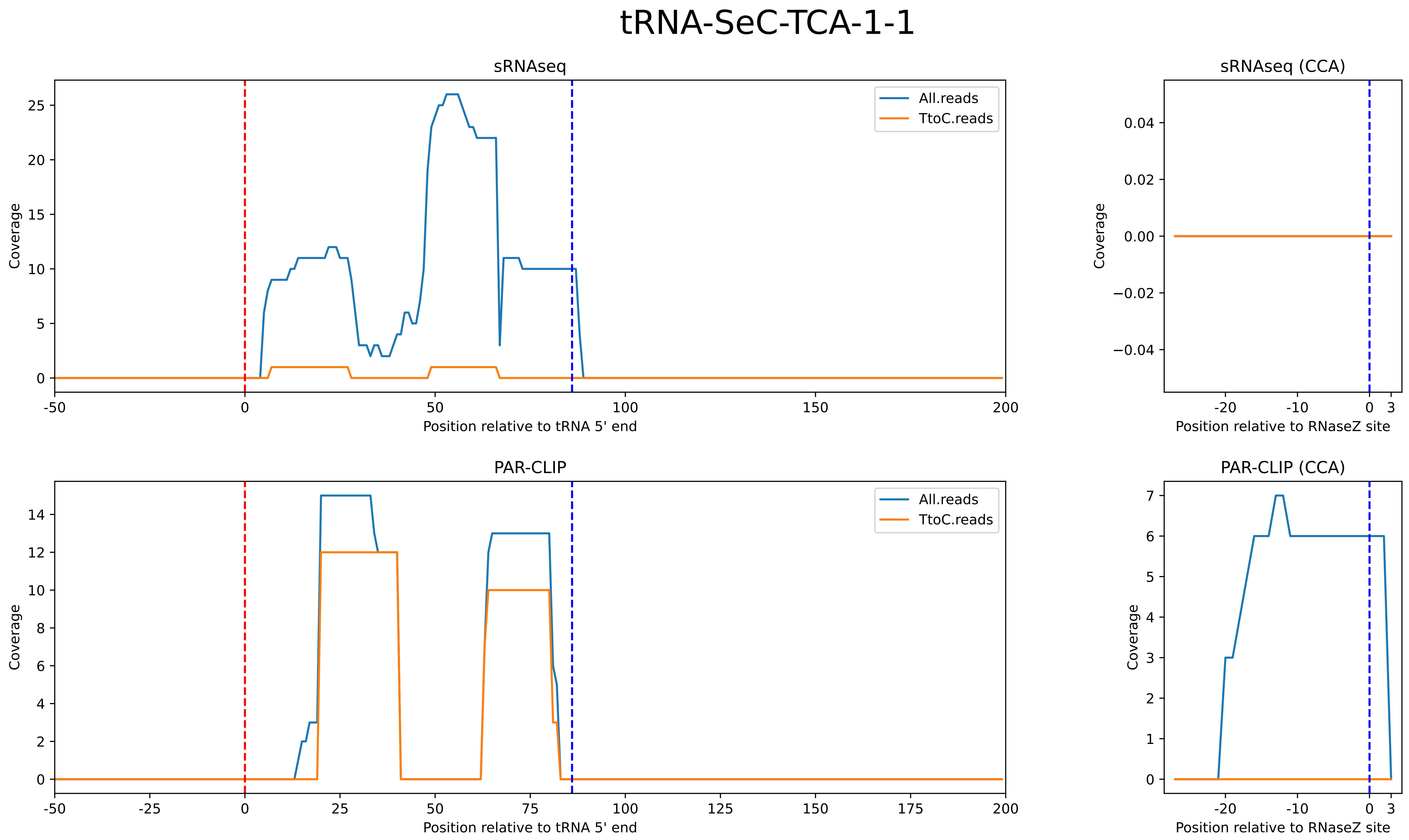
tRNA-SeC-TCA-1-1
sRNAseq
All.reads
TtoC.reads
Coverage
0
5
10
15
20
25
-50
0
50
100
150
200
Position relative to tRNA 5' end
sRNAseq (CCA)
0.04
0.02
0.00
−0.02
−0.04
-20
-10
0
3
Position relative to RNaseZ site
PAR-CLIP
All.reads
TtoC.reads
0
2
4
6
8
10
12
14
-50
-25
0
25
50
75
100
125
150
175
200
Position relative to tRNA 5' end
PAR-CLIP (CCA)
0
1
2
3
4
5
6
7
-20
-10
0
3
Position relative to RNaseZ site

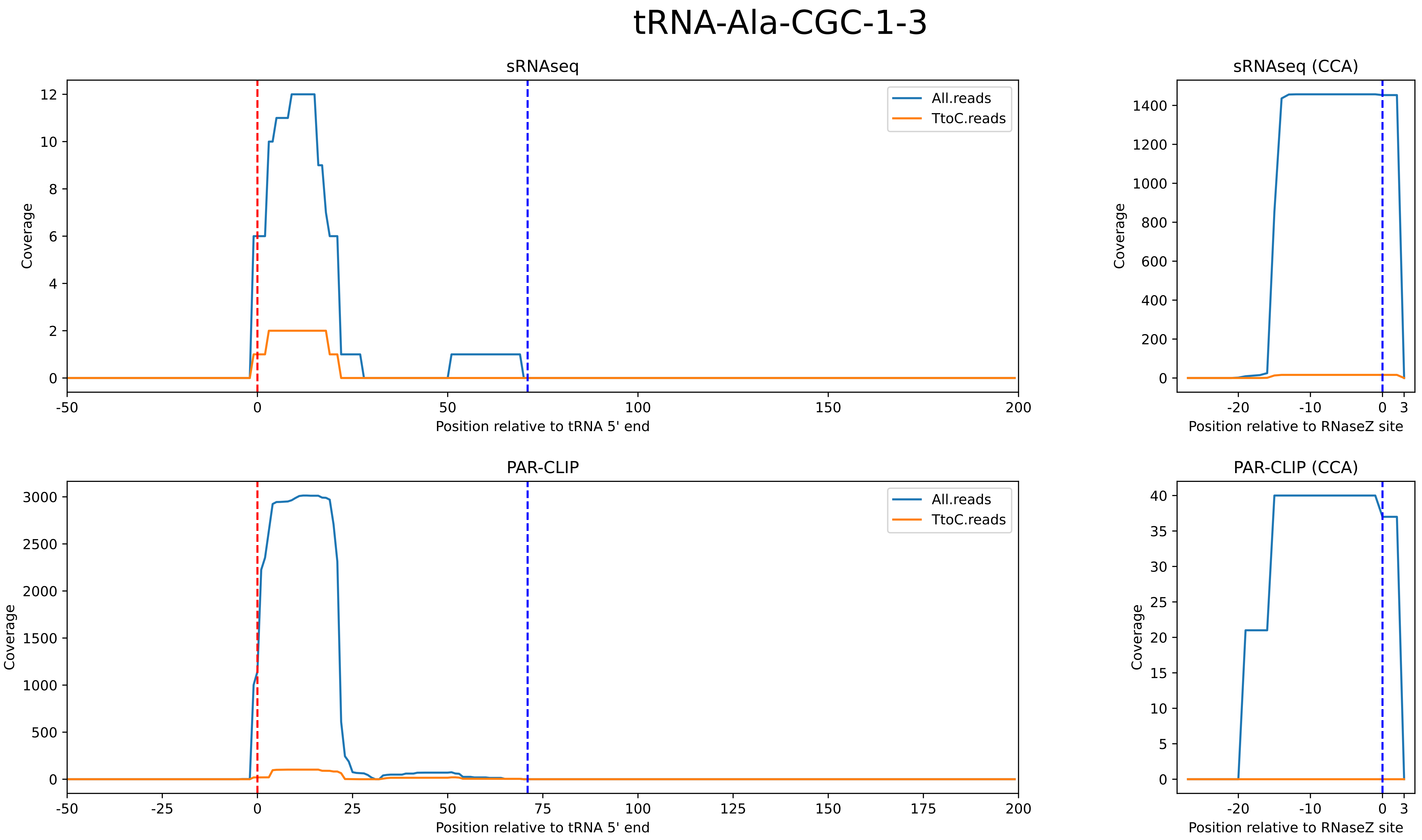

tRNA-Ala-CGC-1-3
sRNAseq
All.reads
TtoC.reads
Coverage
Position relative to tRNA 5' end
sRNAseq (CCA)
Position relative to RNaseZ site
PAR-CLIP
PAR-CLIP (CCA)

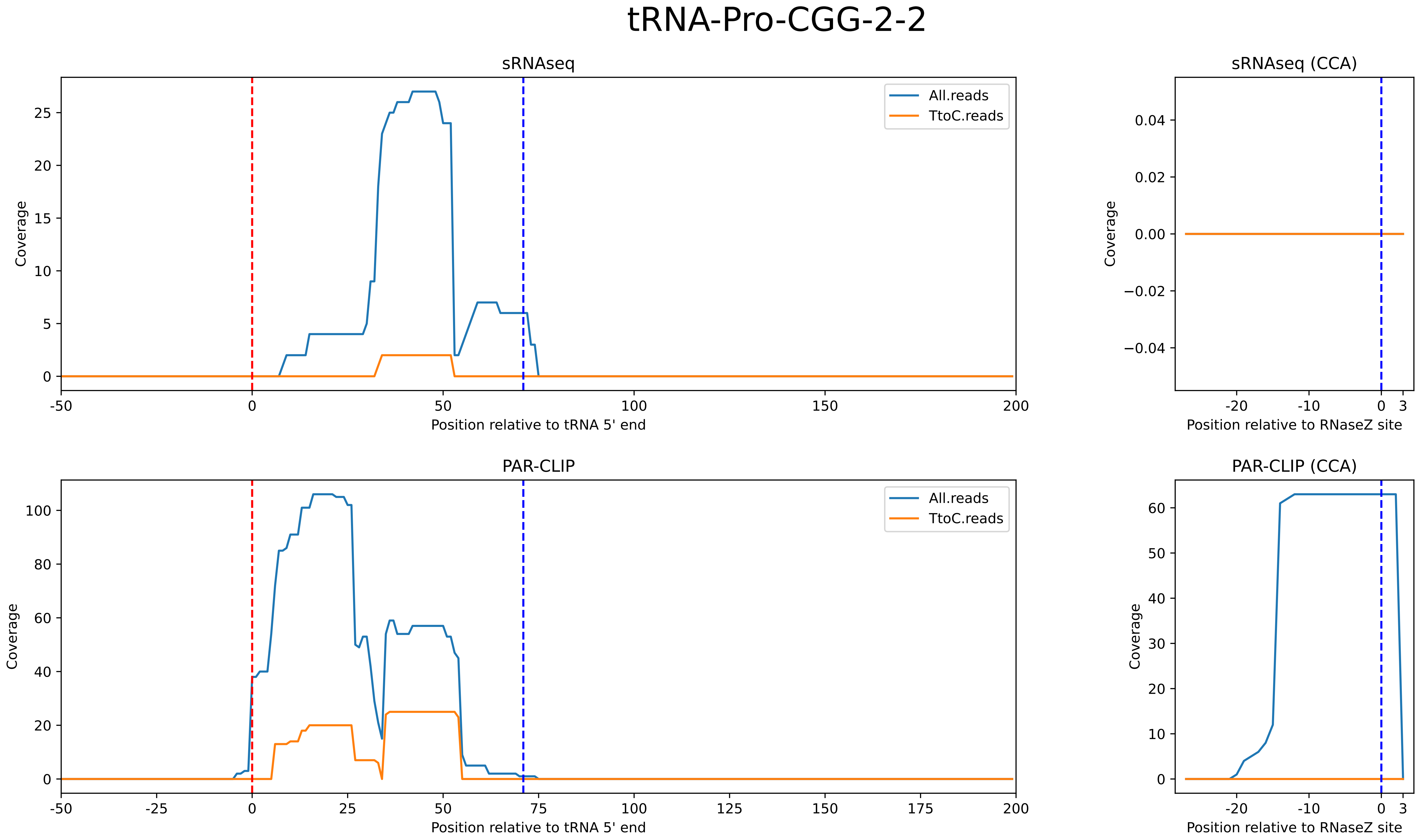

tRNA-Pro-CGG-2-2
sRNAseq
All.reads
TtoC.reads
Coverage
Position relative to tRNA 5' end
sRNAseq (CCA)
Position relative to RNaseZ site
PAR-CLIP
PAR-CLIP (CCA)

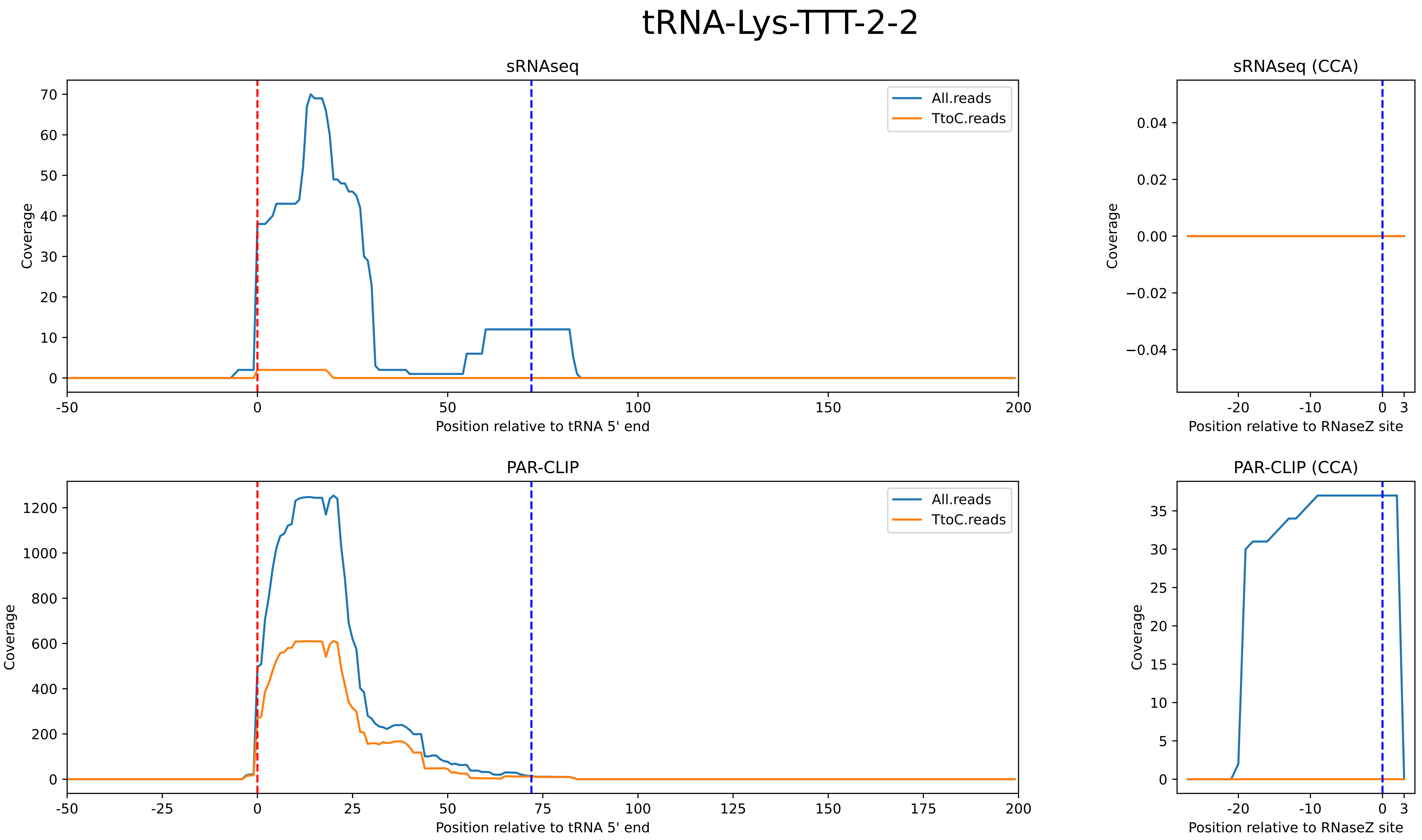

tRNA-Lys-TTT-2-2
sRNAseq
sRNAseq (CCA)
PAR-CLIP
PAR-CLIP (CCA)
All.reads
TtoC.reads
Coverage
Position relative to tRNA 5' end
Position relative to RNaseZ site

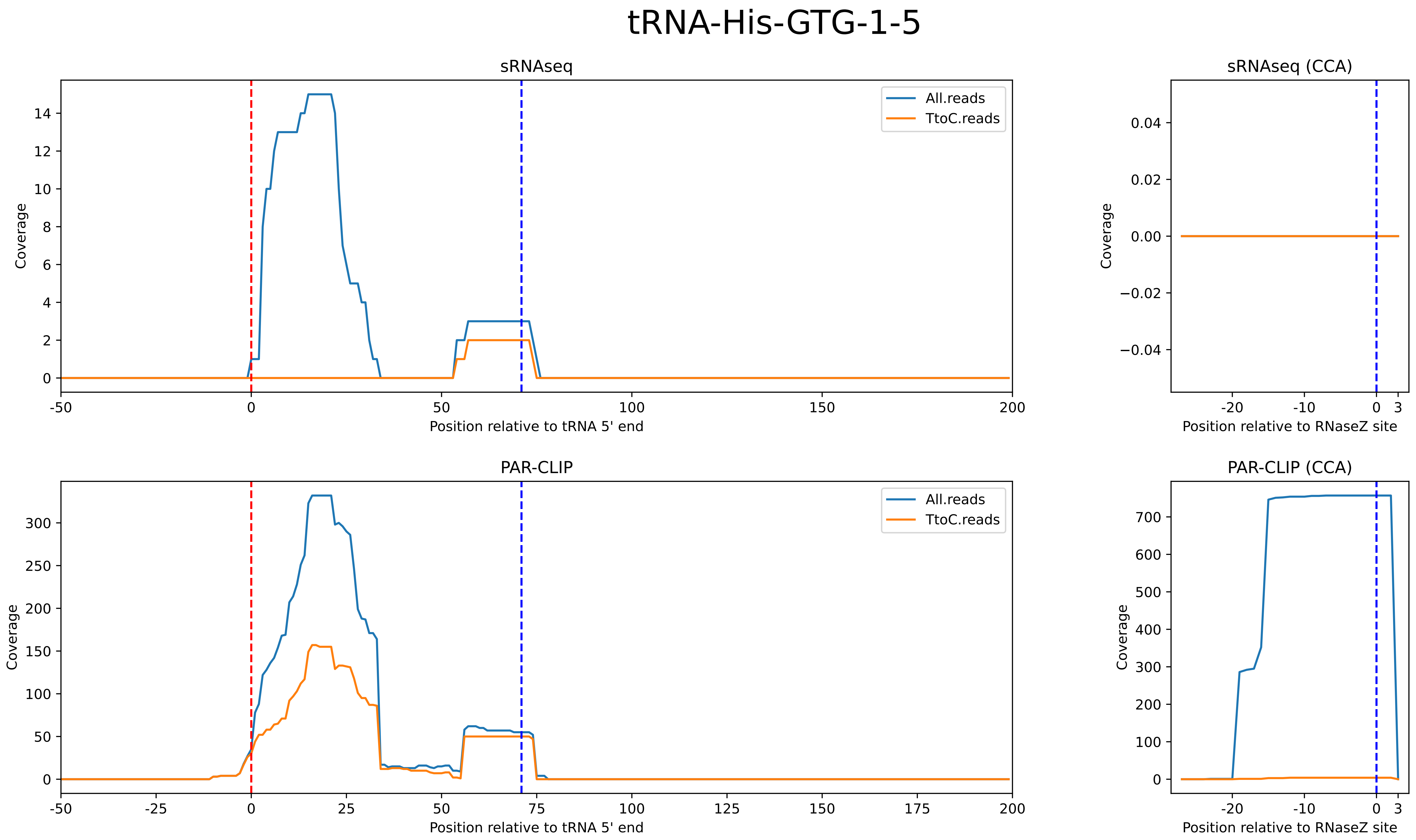
tRNA-His-GTG-1-5
sRNAseq
All.reads
TtoC.reads
Coverage
Position relative to tRNA 5' end
sRNAseq (CCA)
Position relative to RNaseZ site
PAR-CLIP
PAR-CLIP (CCA)

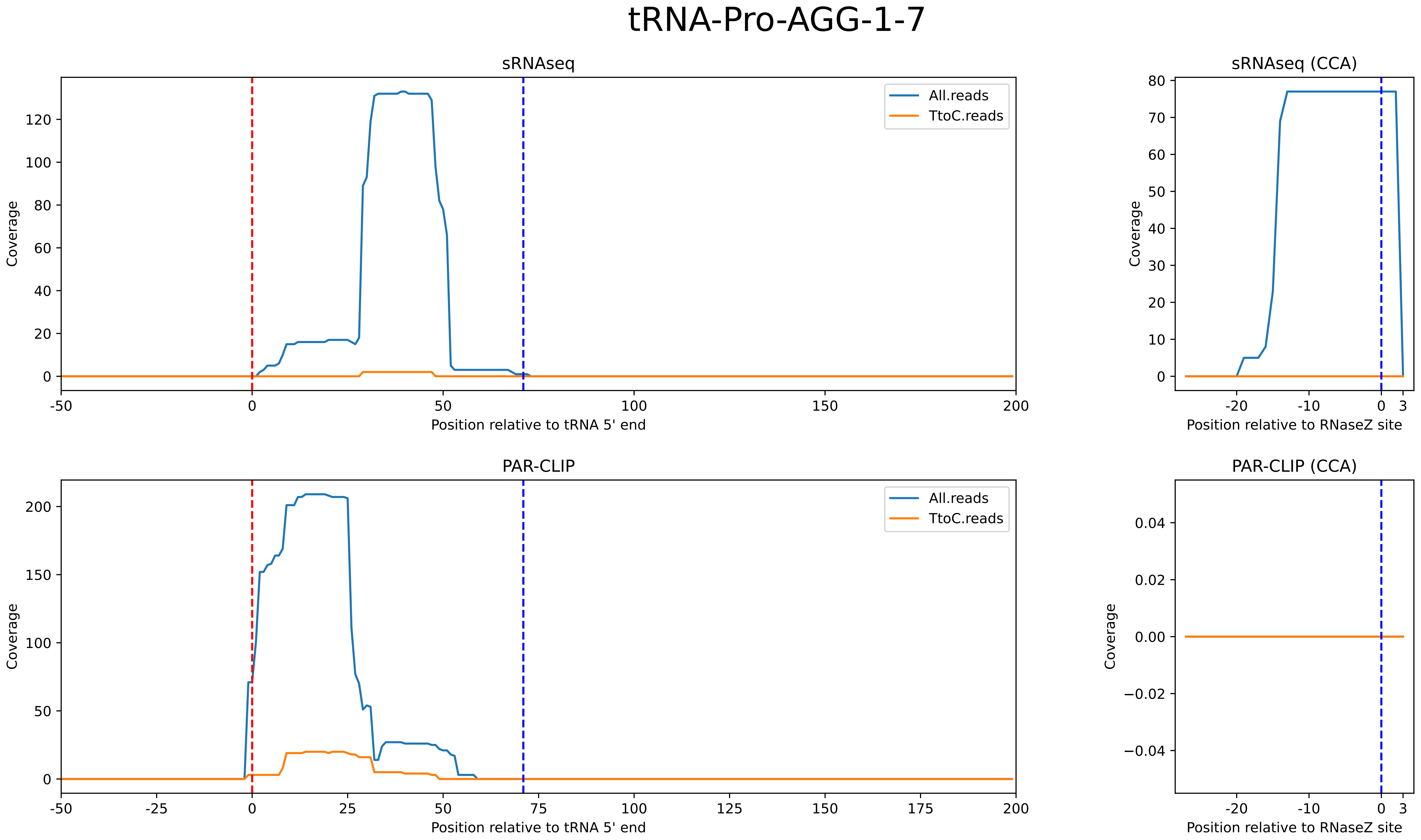

tRNA-Pro-AGG-1-7
sRNAseq
All.reads
TtoC.reads
Coverage
Position relative to tRNA 5' end
sRNAseq (CCA)
Position relative to RNaseZ site
PAR-CLIP
PAR-CLIP (CCA)

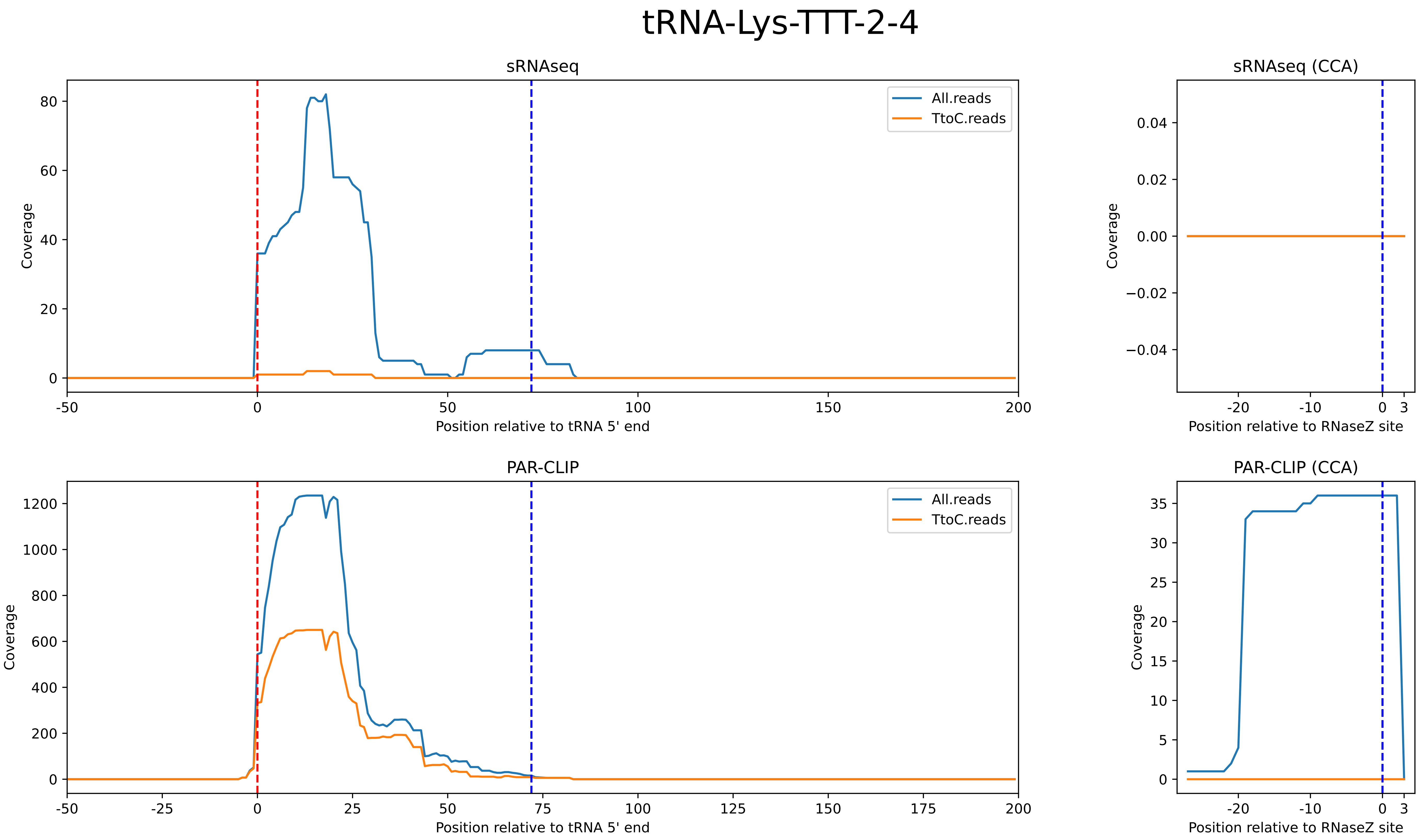
tRNA-Lys-TTT-2-4
sRNAseq
All.reads
TtoC.reads
Coverage
Position relative to tRNA 5' end
sRNAseq (CCA)
Position relative to RNaseZ site
PAR-CLIP
PAR-CLIP (CCA)

# tRNA-Thr-AGT-1-4

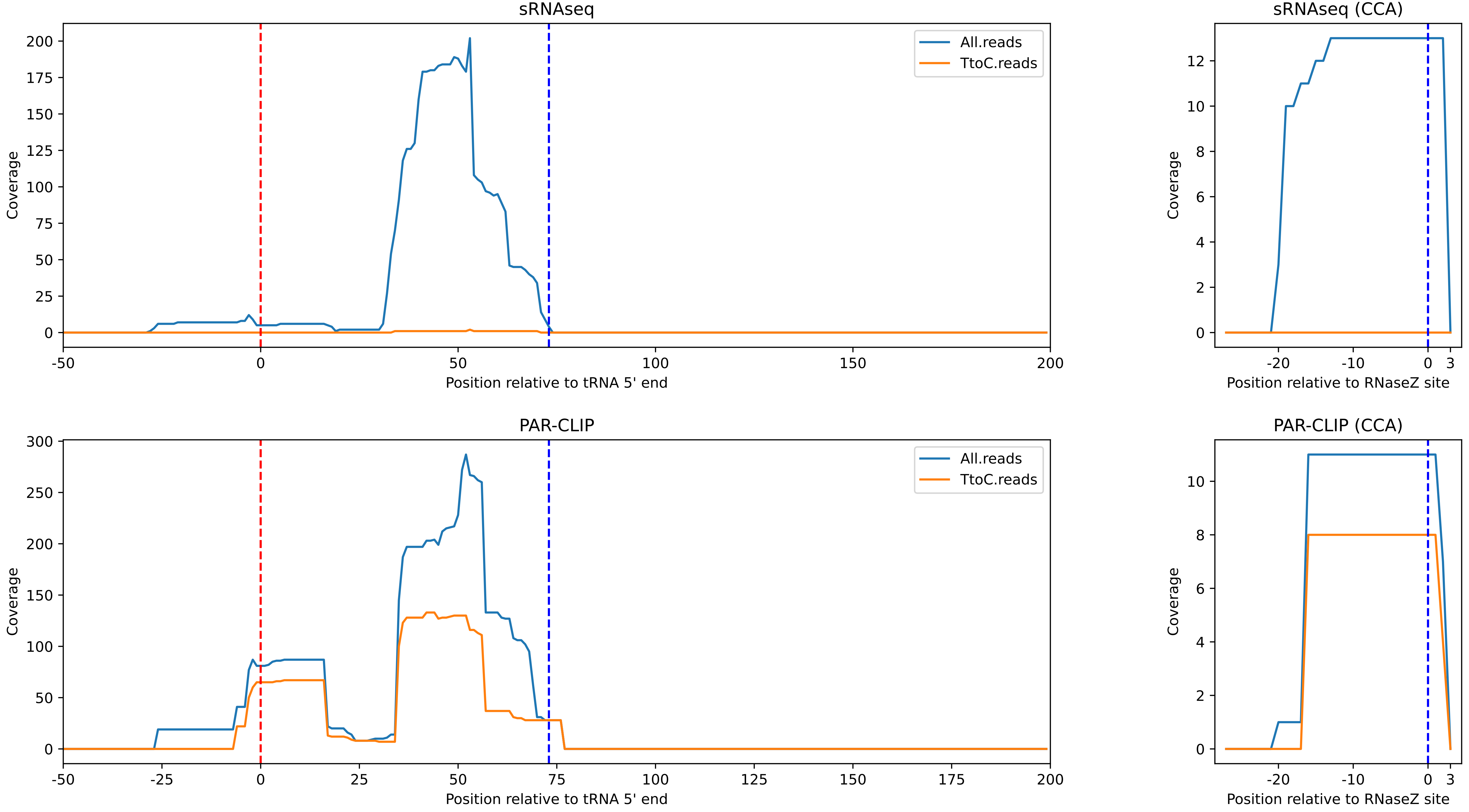

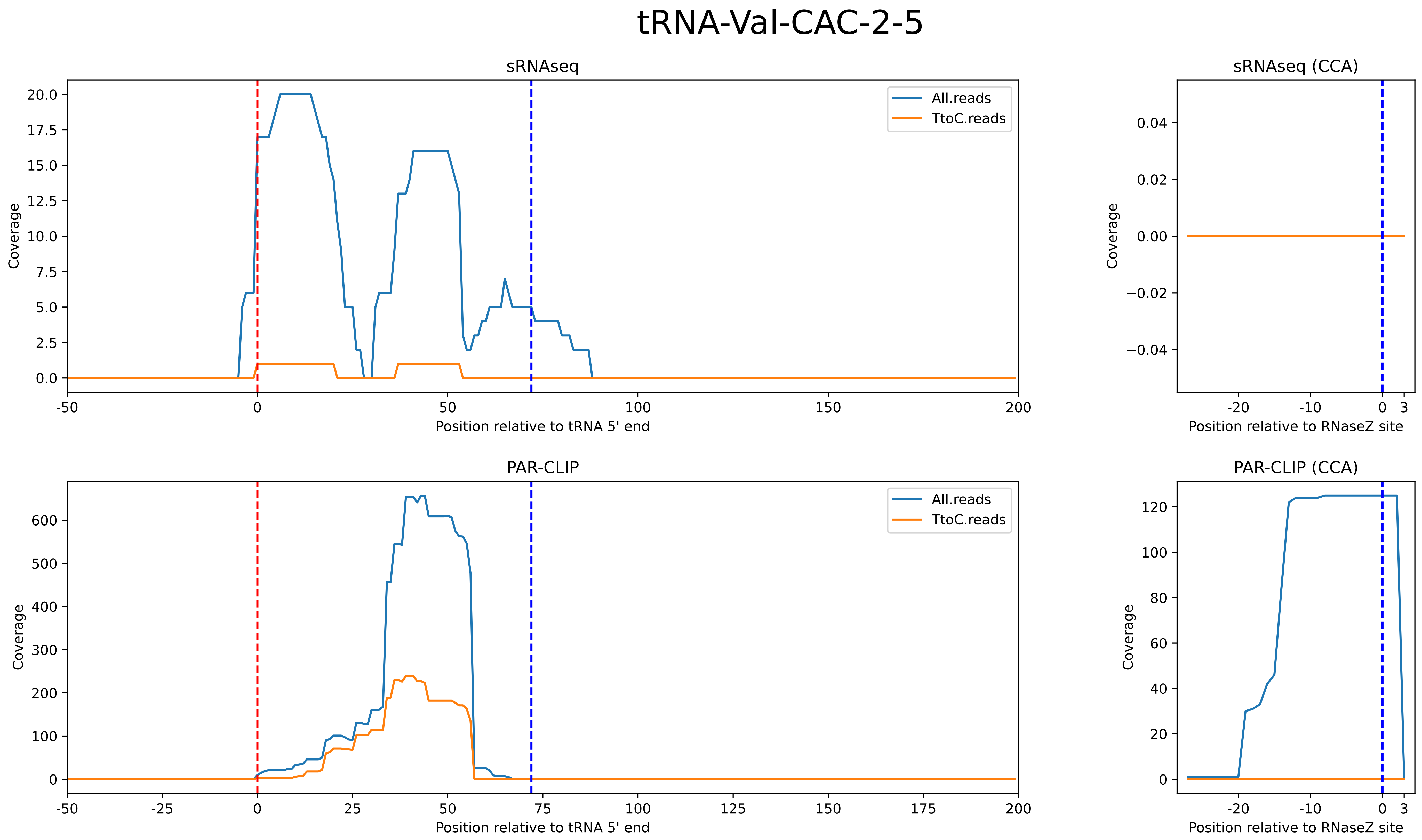

tRNA-Val-CAC-2-5
sRNAseq
sRNAseq (CCA)
PAR-CLIP
PAR-CLIP (CCA)
All.reads
TtoC.reads
Coverage
Position relative to tRNA 5' end
Position relative to RNaseZ site

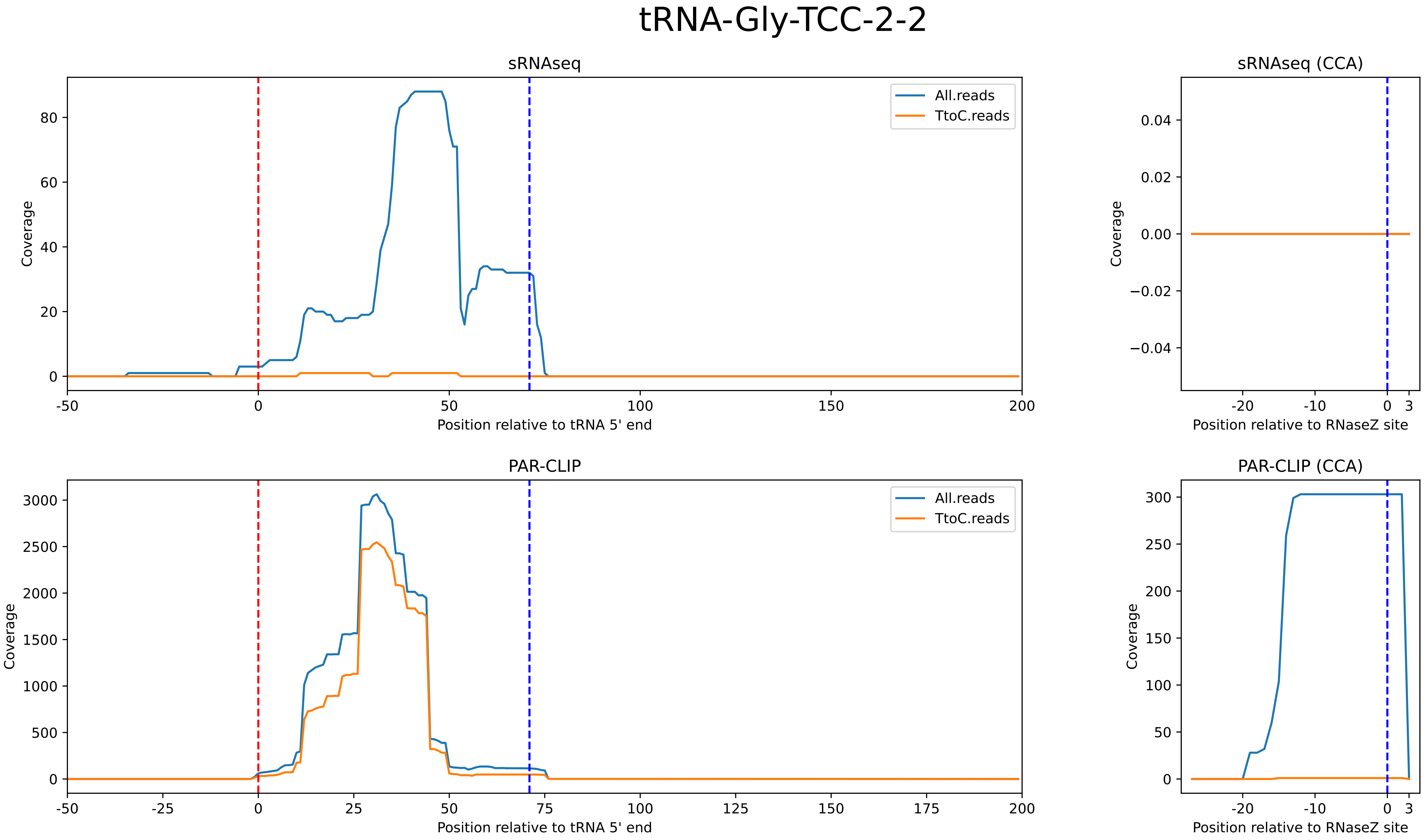

tRNA-Gly-TCC-2-2
sRNAseq
All.reads
TtoC.reads
Coverage
Position relative to tRNA 5' end
sRNAseq (CCA)
Position relative to RNaseZ site
PAR-CLIP
PAR-CLIP (CCA)

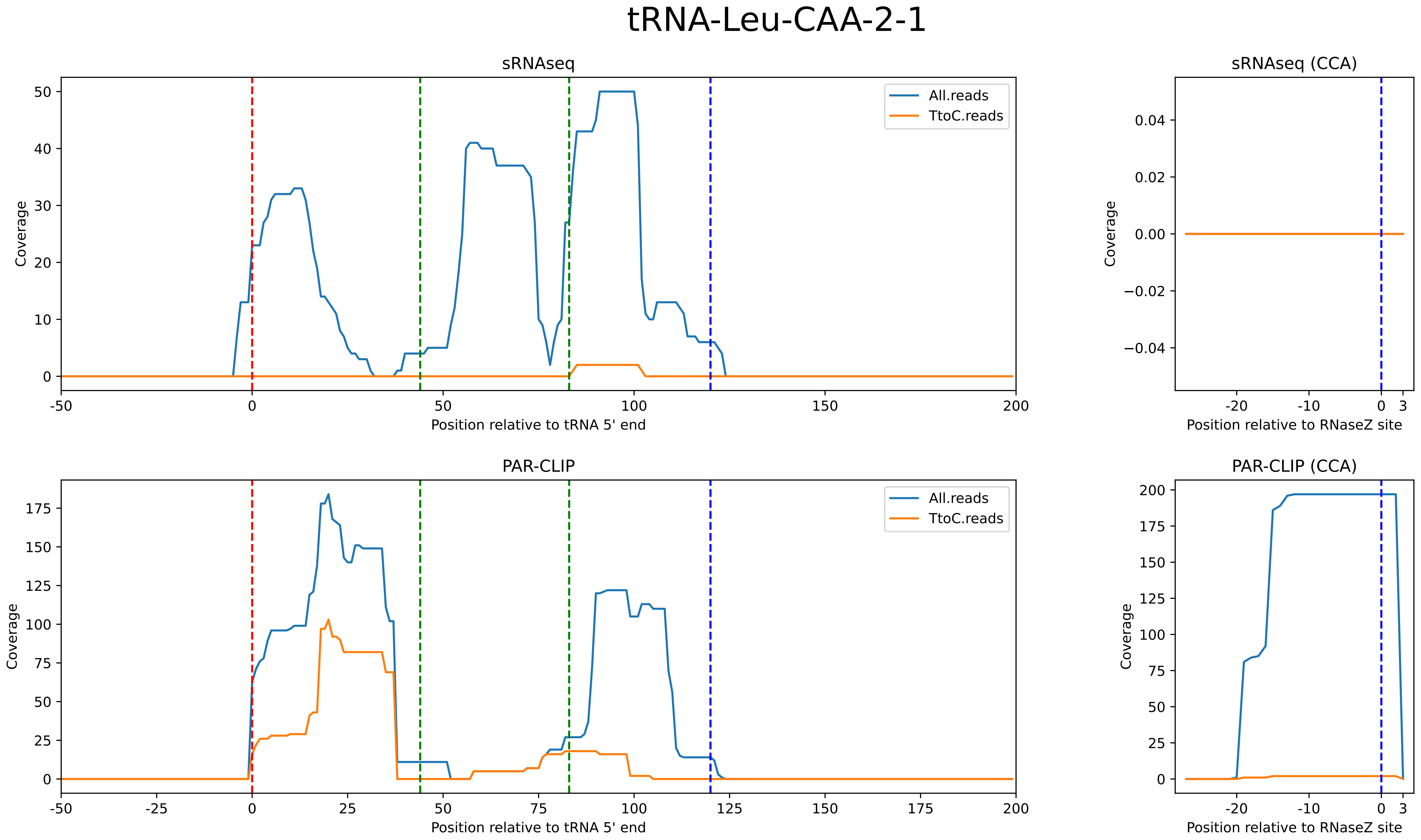

tRNA-Leu-CAA-2-1
sRNAseq
All.reads
TtoC.reads
Coverage
Position relative to tRNA 5' end
sRNAseq (CCA)
Position relative to RNaseZ site
PAR-CLIP
PAR-CLIP (CCA)

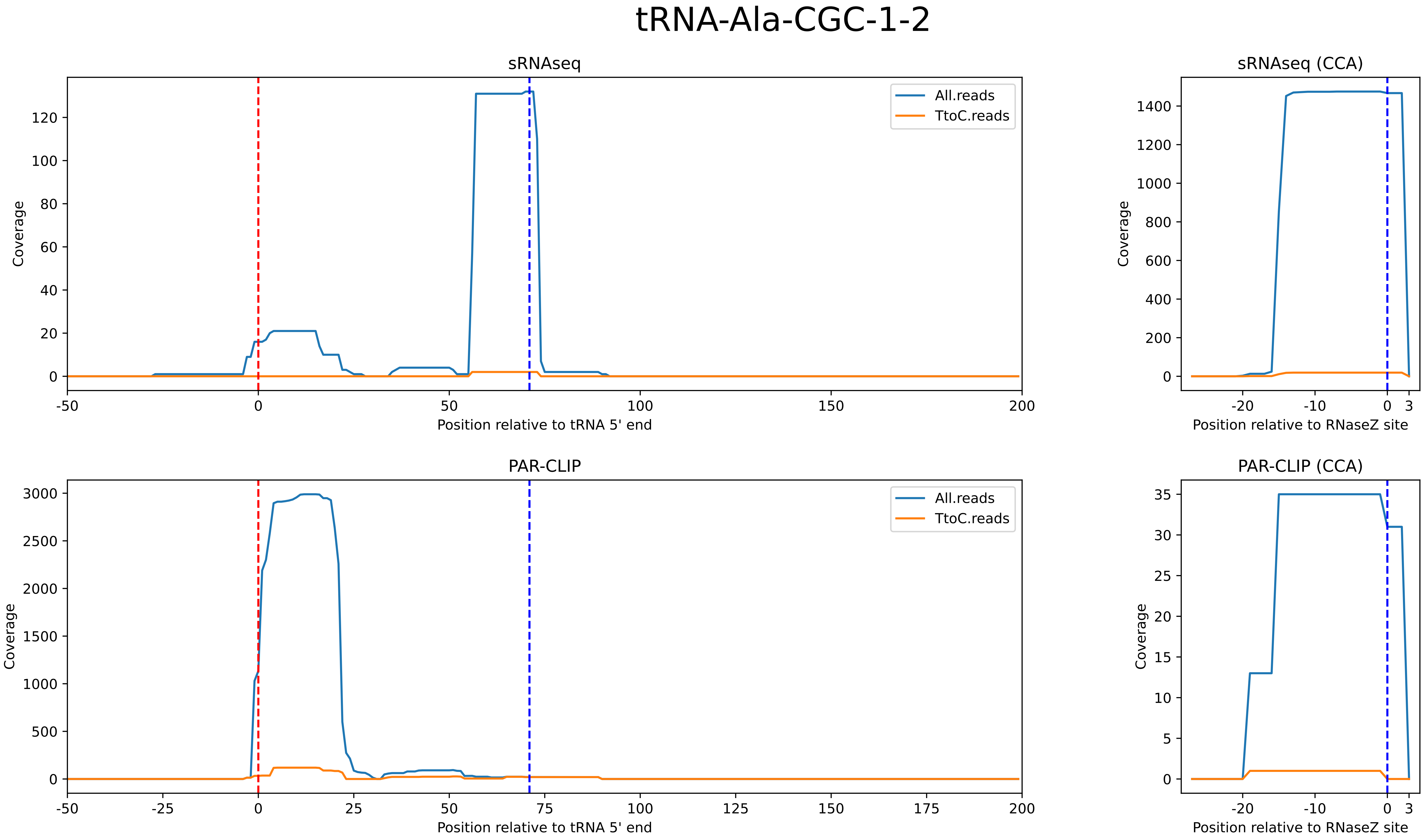
tRNA-Ala-CGC-1-2
sRNAseq
All.reads
TtoC.reads
Coverage
Position relative to tRNA 5' end
sRNAseq (CCA)
Position relative to RNaseZ site
PAR-CLIP
PAR-CLIP (CCA)

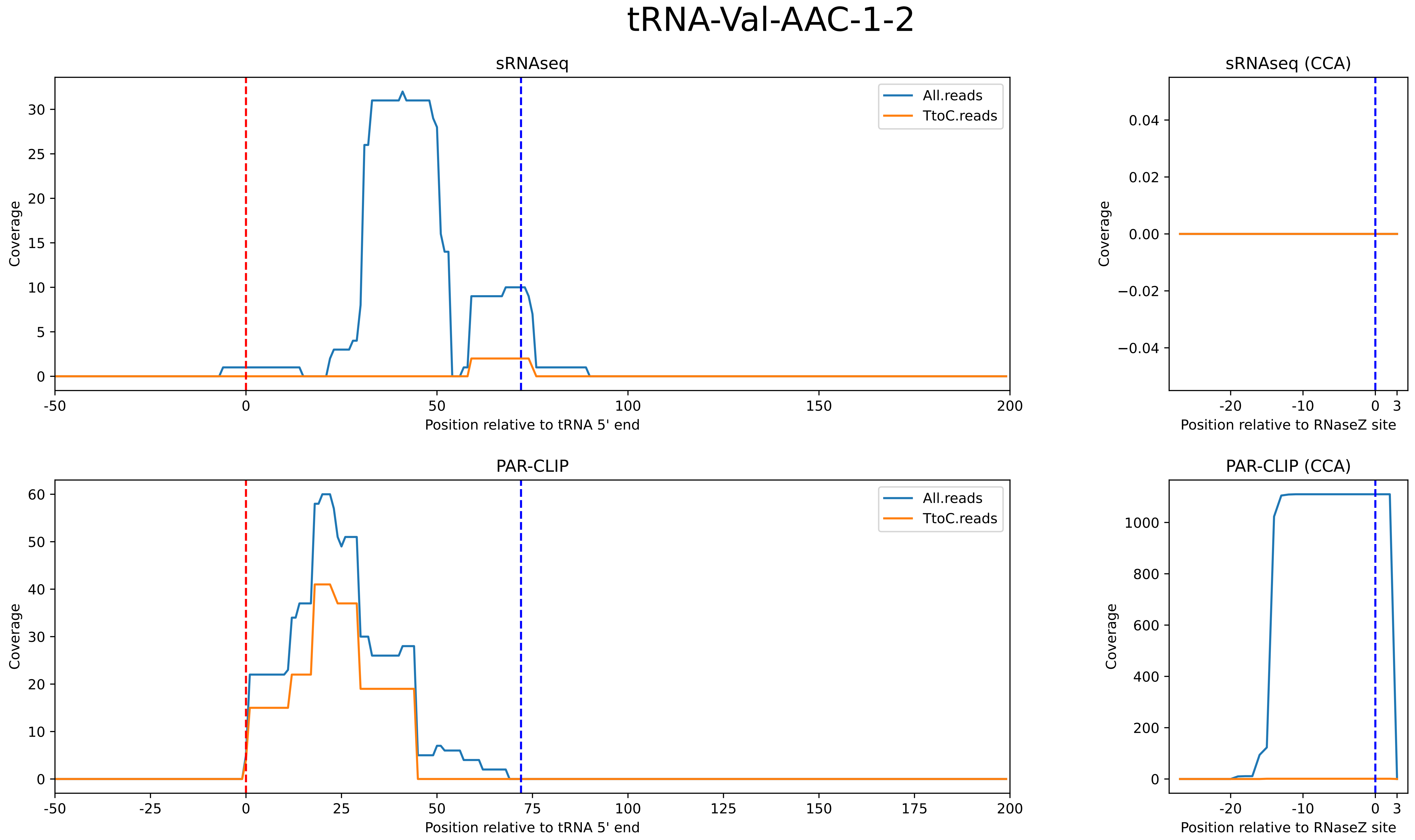
tRNA-Val-AAC-1-2
sRNAseq
All.reads
TtoC.reads
Coverage
Position relative to tRNA 5' end
sRNAseq (CCA)
Position relative to RNaseZ site
PAR-CLIP
PAR-CLIP (CCA)

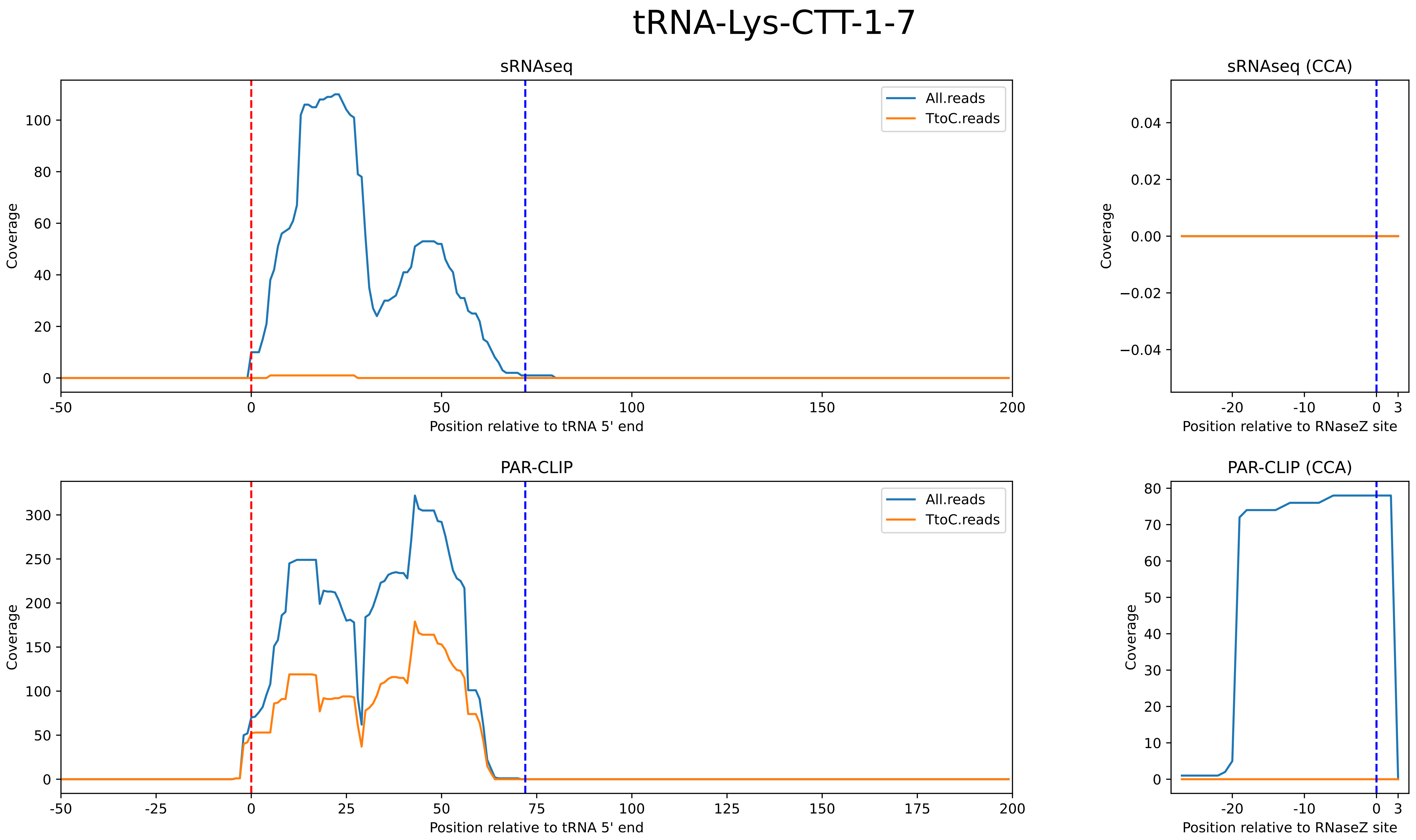
tRNA-Lys-CTT-1-7
sRNAseq
sRNAseq (CCA)
PAR-CLIP
PAR-CLIP (CCA)
All.reads
TtoC.reads
Coverage
Position relative to tRNA 5' end
Position relative to RNaseZ site

# tRNA-Thr-AGT-2-1

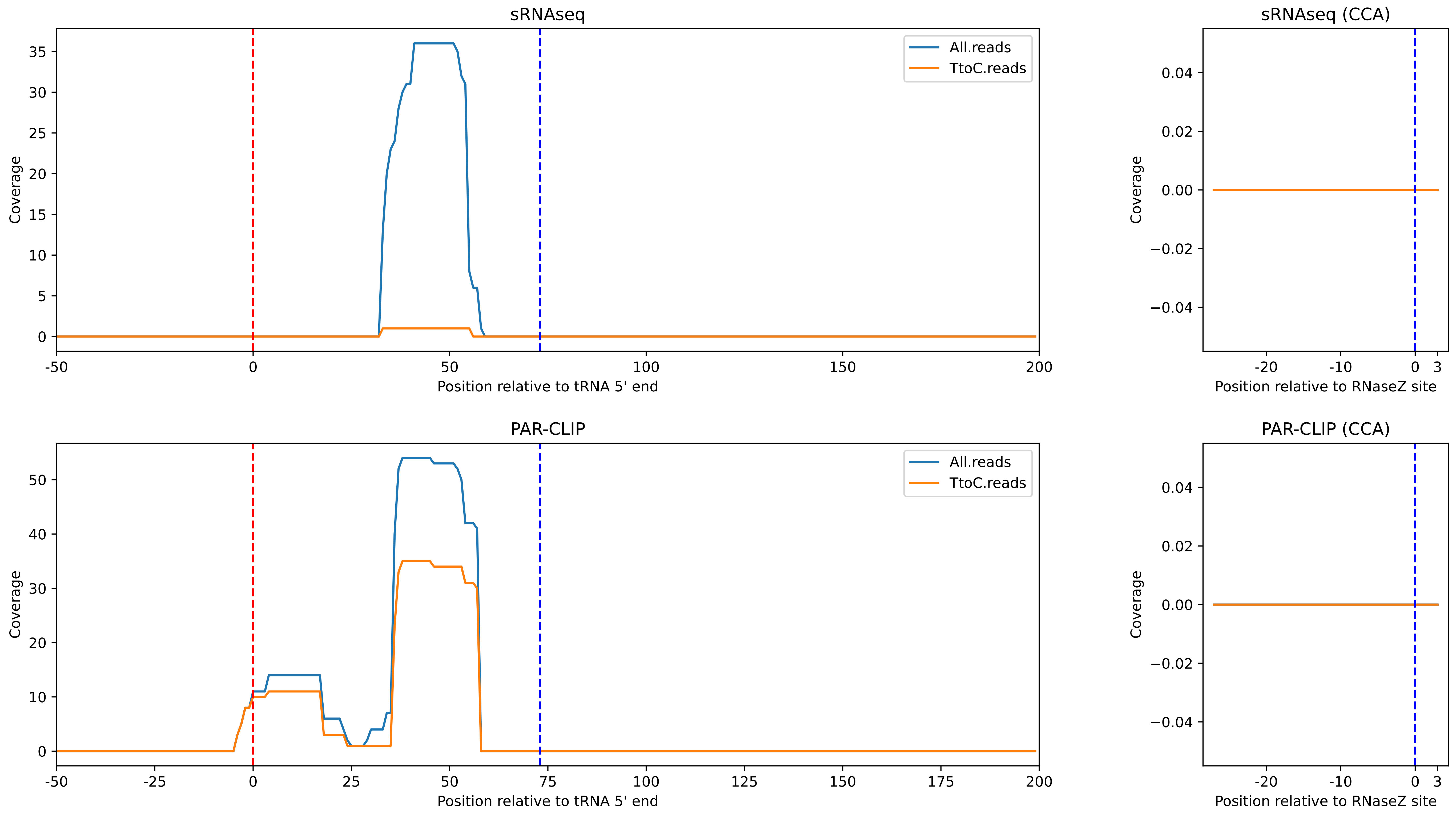

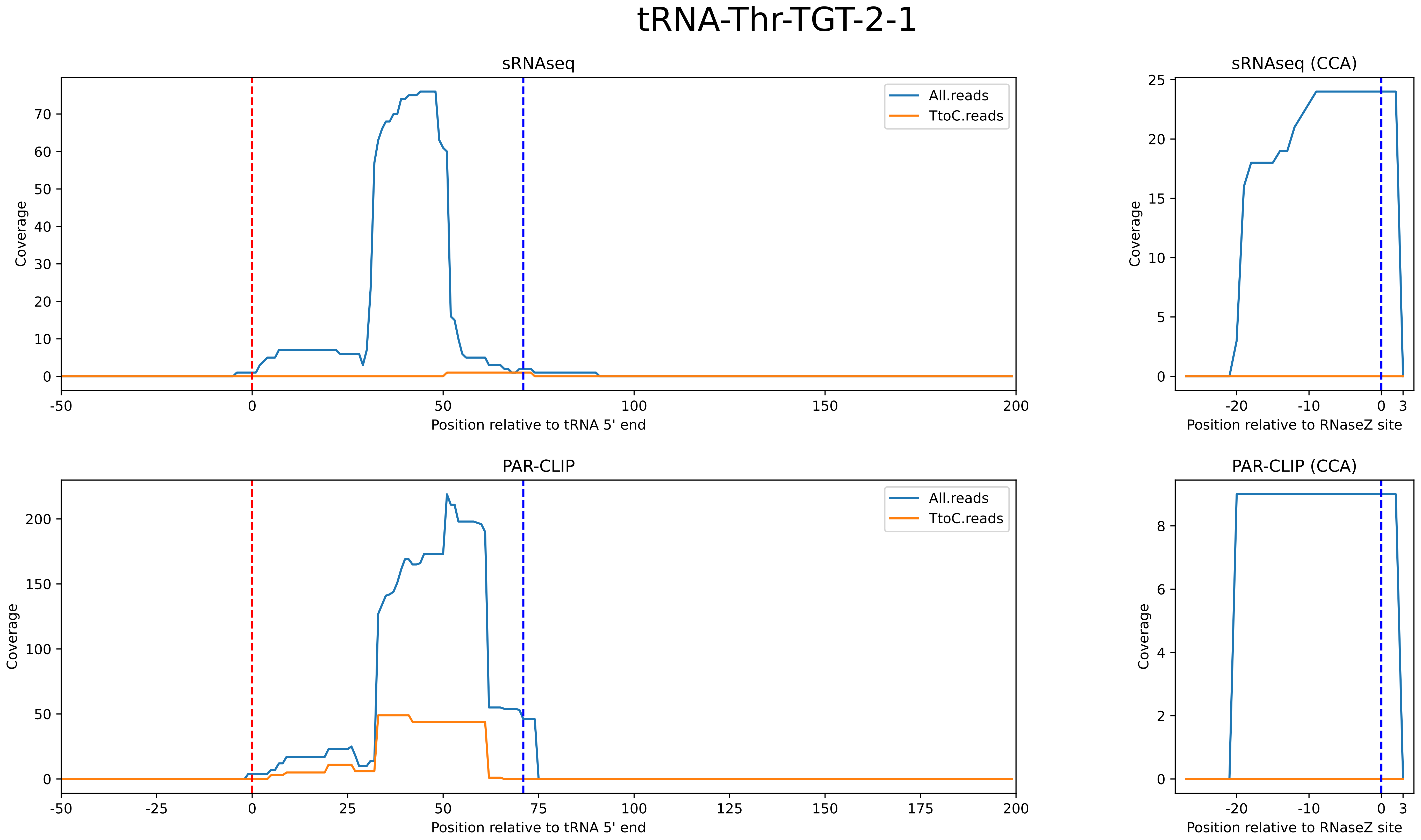
tRNA-Thr-TGT-2-1
sRNAseq
All.reads
TtoC.reads
Coverage
Position relative to tRNA 5' end
sRNAseq (CCA)
Coverage
Position relative to RNaseZ site
PAR-CLIP
All.reads
TtoC.reads
Coverage
Position relative to tRNA 5' end
PAR-CLIP (CCA)
Coverage
Position relative to RNaseZ site

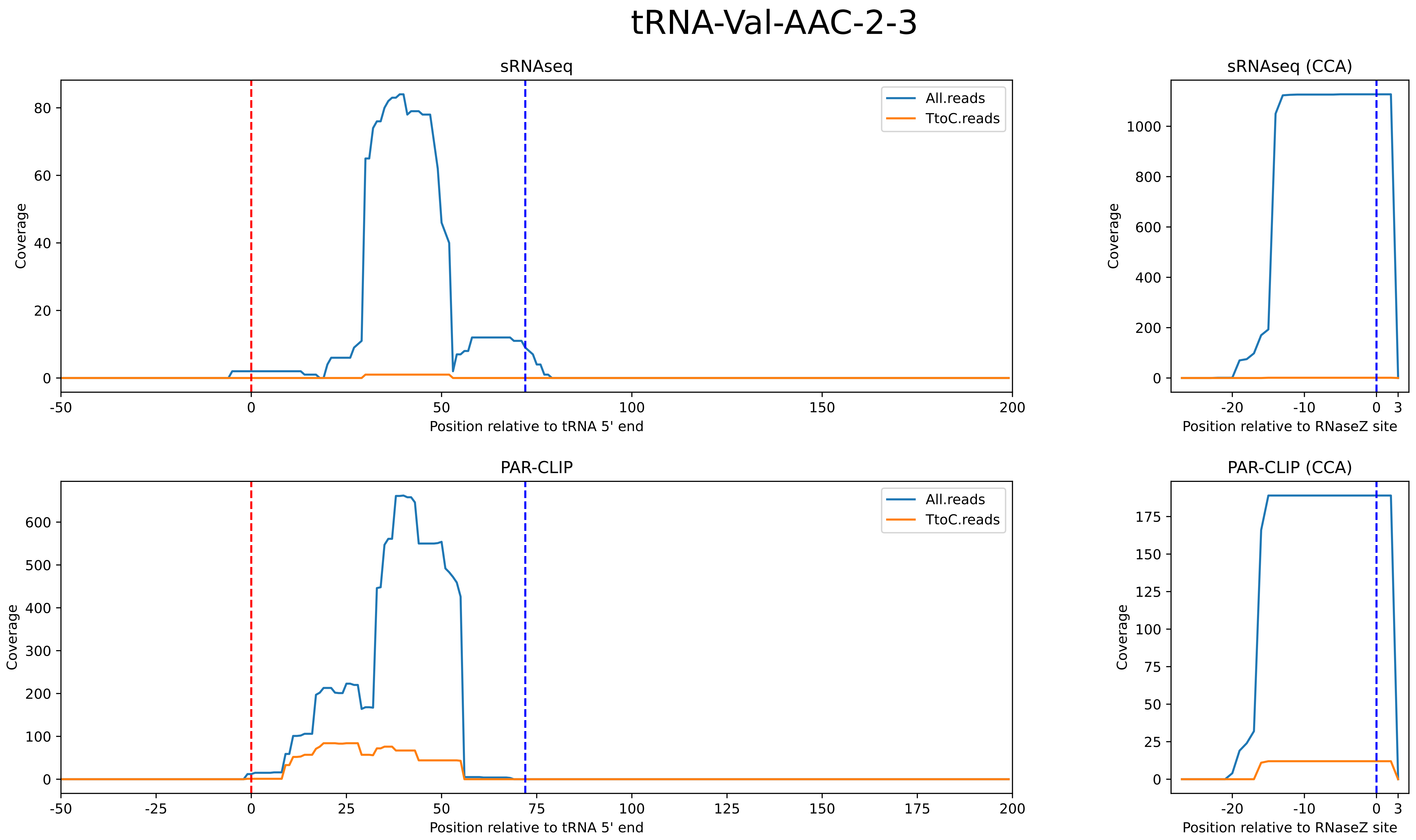

tRNA-Val-AAC-2-3
sRNAseq
sRNAseq (CCA)
PAR-CLIP
PAR-CLIP (CCA)
All.reads
TtoC.reads
Coverage
Position relative to tRNA 5' end
Position relative to RNaseZ site

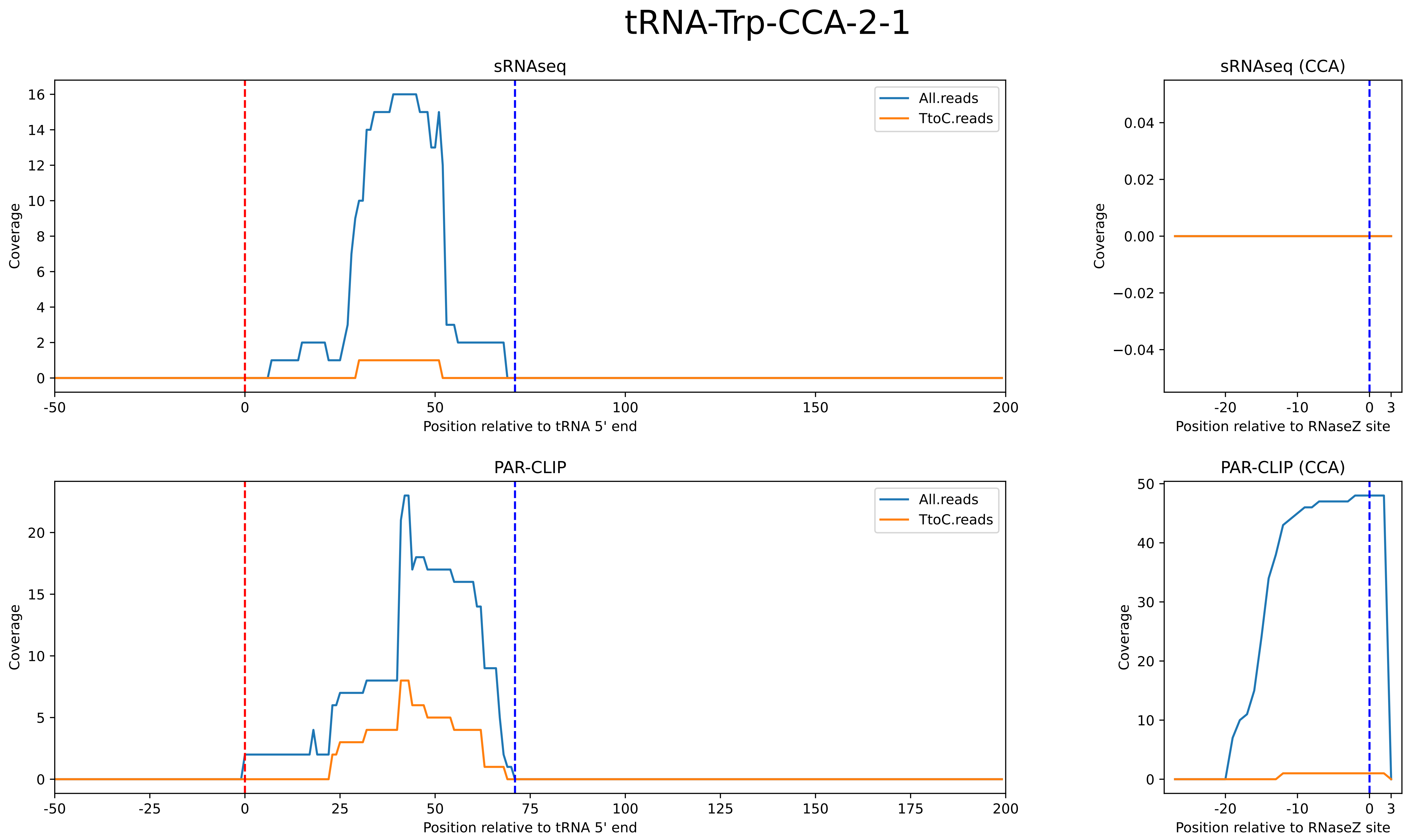
tRNA-Trp-CCA-2-1
sRNAseq
All.reads
TtoC.reads
Coverage
Position relative to tRNA 5' end
sRNAseq (CCA)
Position relative to RNaseZ site
PAR-CLIP
PAR-CLIP (CCA)

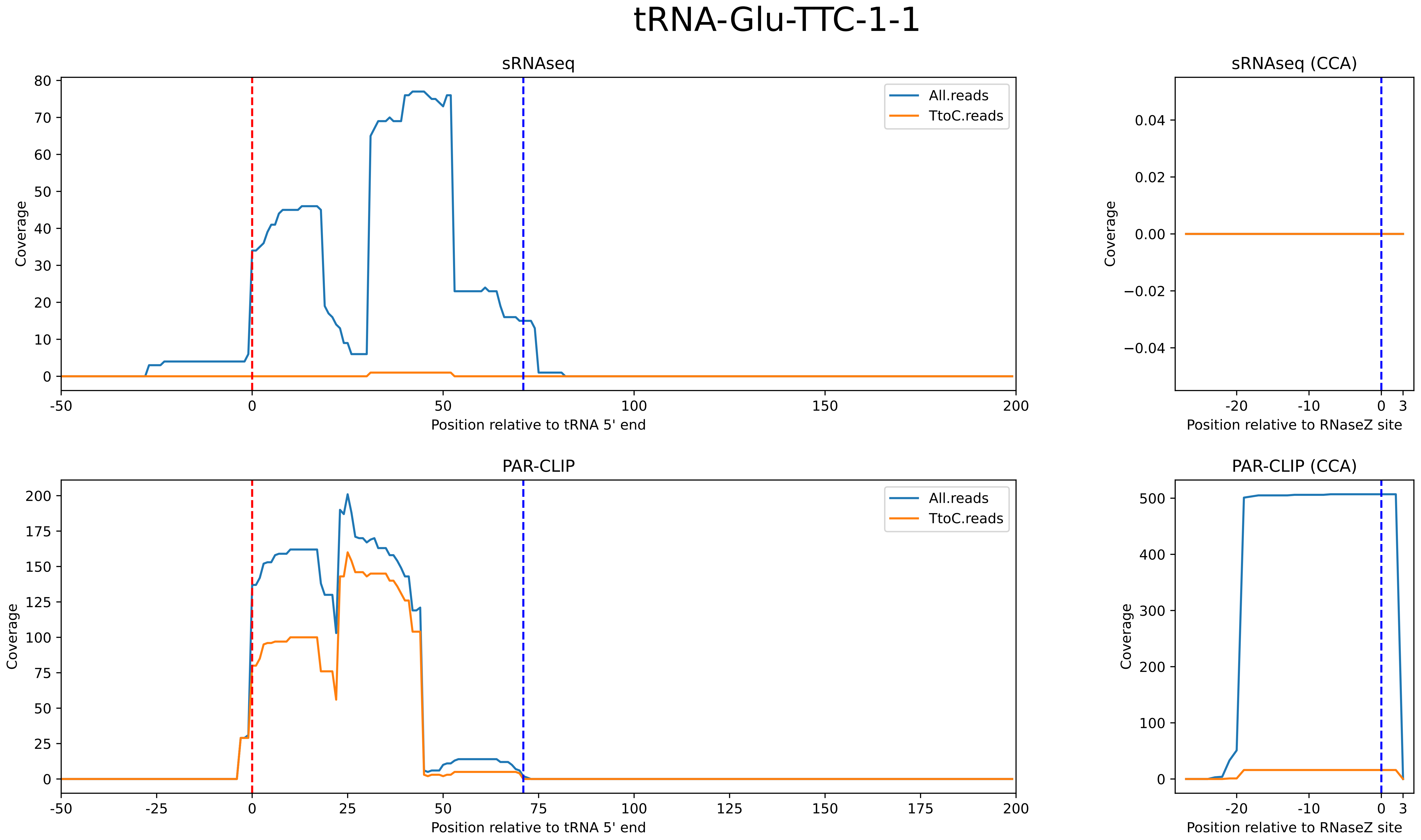
tRNA-Glu-TTC-1-1
sRNAseq
All.reads
TtoC.reads
Coverage
Position relative to tRNA 5' end
sRNAseq (CCA)
Position relative to RNaseZ site
PAR-CLIP
PAR-CLIP (CCA)

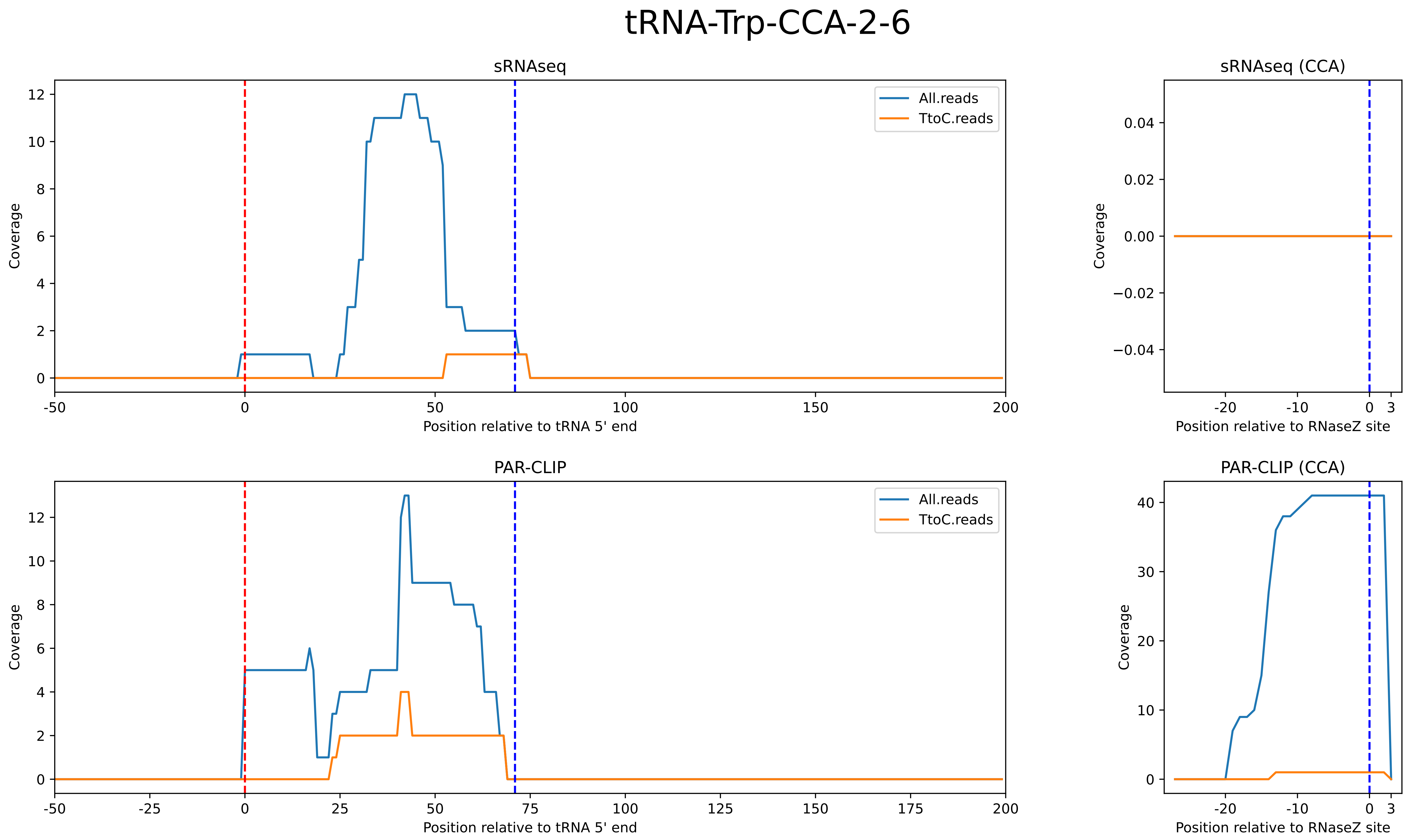

tRNA-Trp-CCA-2-6
sRNAseq
All.reads
TtoC.reads
Coverage
Position relative to tRNA 5' end
sRNAseq (CCA)
Position relative to RNaseZ site
PAR-CLIP
PAR-CLIP (CCA)

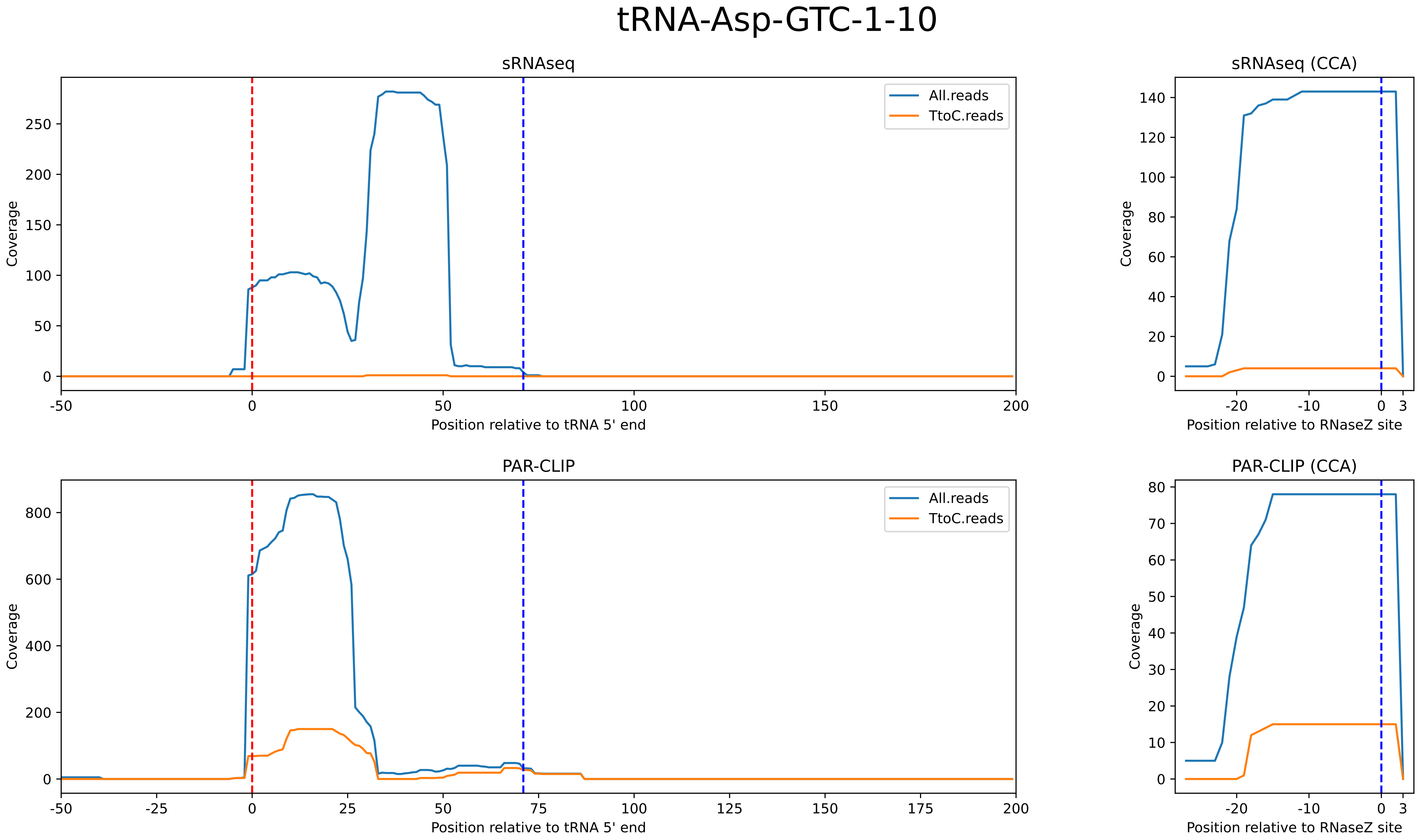
tRNA-Asp-GTC-1-10
sRNAseq
All.reads
TtoC.reads
Coverage
Position relative to tRNA 5' end
sRNAseq (CCA)
Position relative to RNaseZ site
PAR-CLIP
PAR-CLIP (CCA)

# tRNA-Val-CAC-2-4

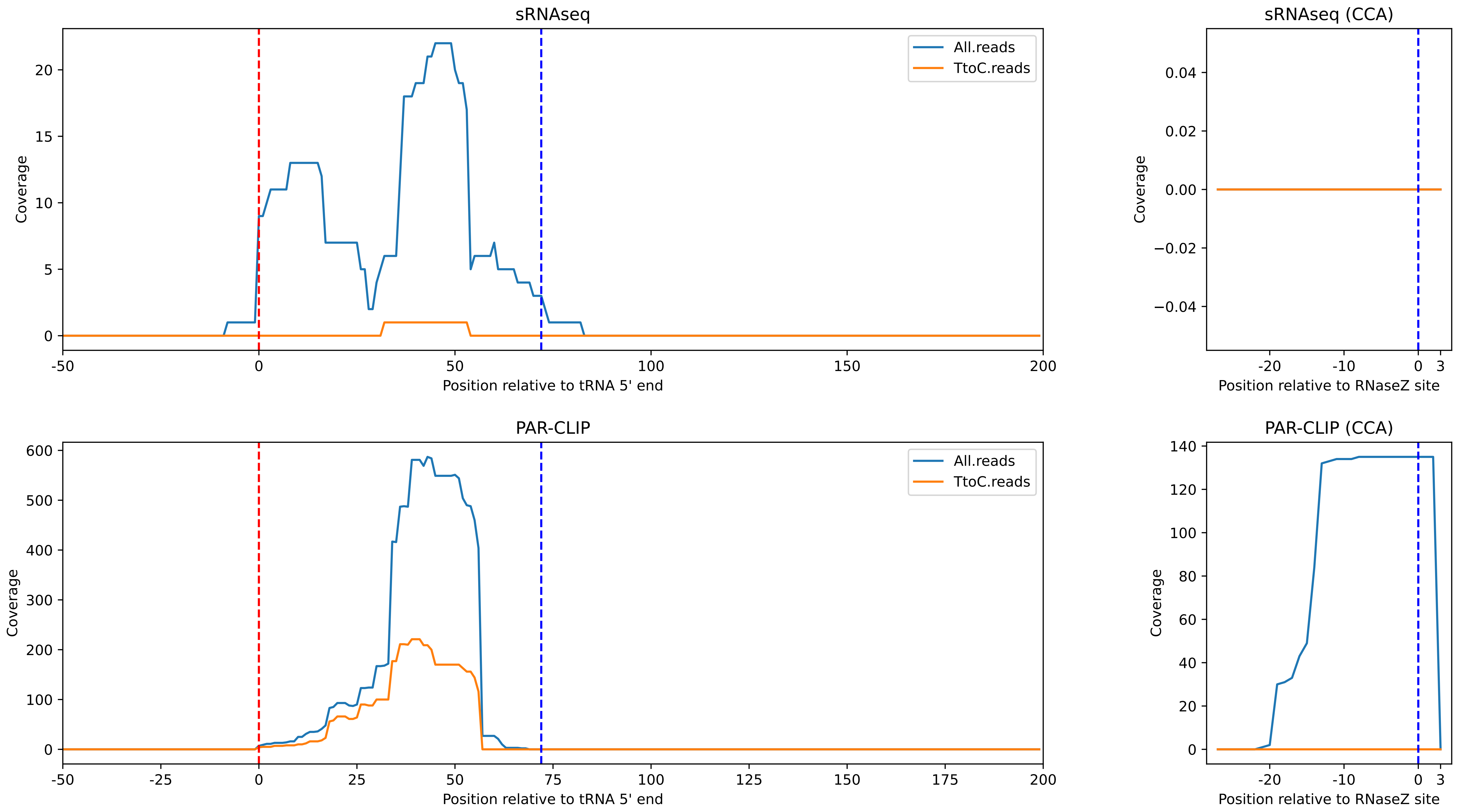

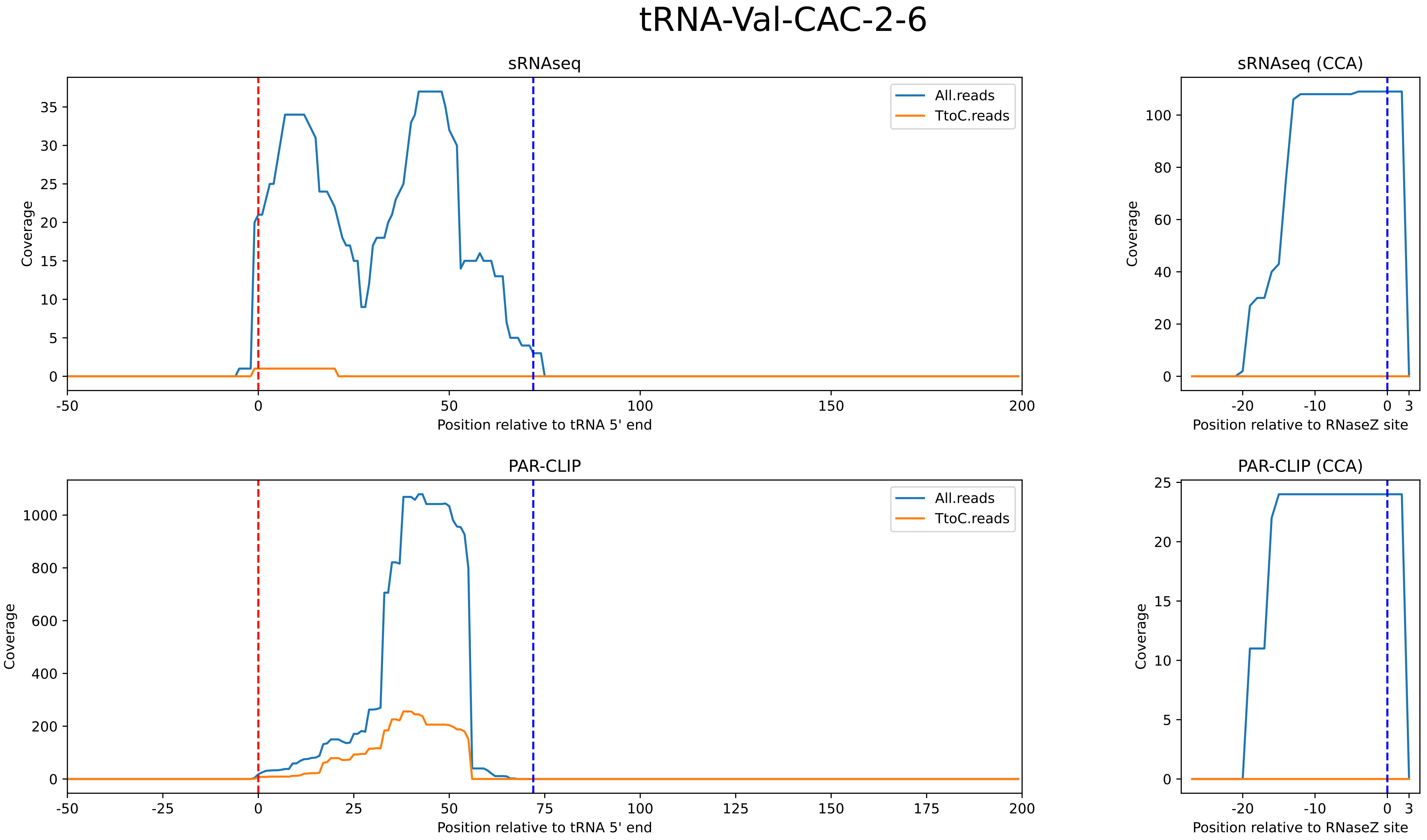
tRNA-Val-CAC-2-6
sRNAseq
All.reads
TtoC.reads
Coverage
Position relative to tRNA 5' end
sRNAseq (CCA)
Position relative to RNaseZ site
PAR-CLIP
PAR-CLIP (CCA)

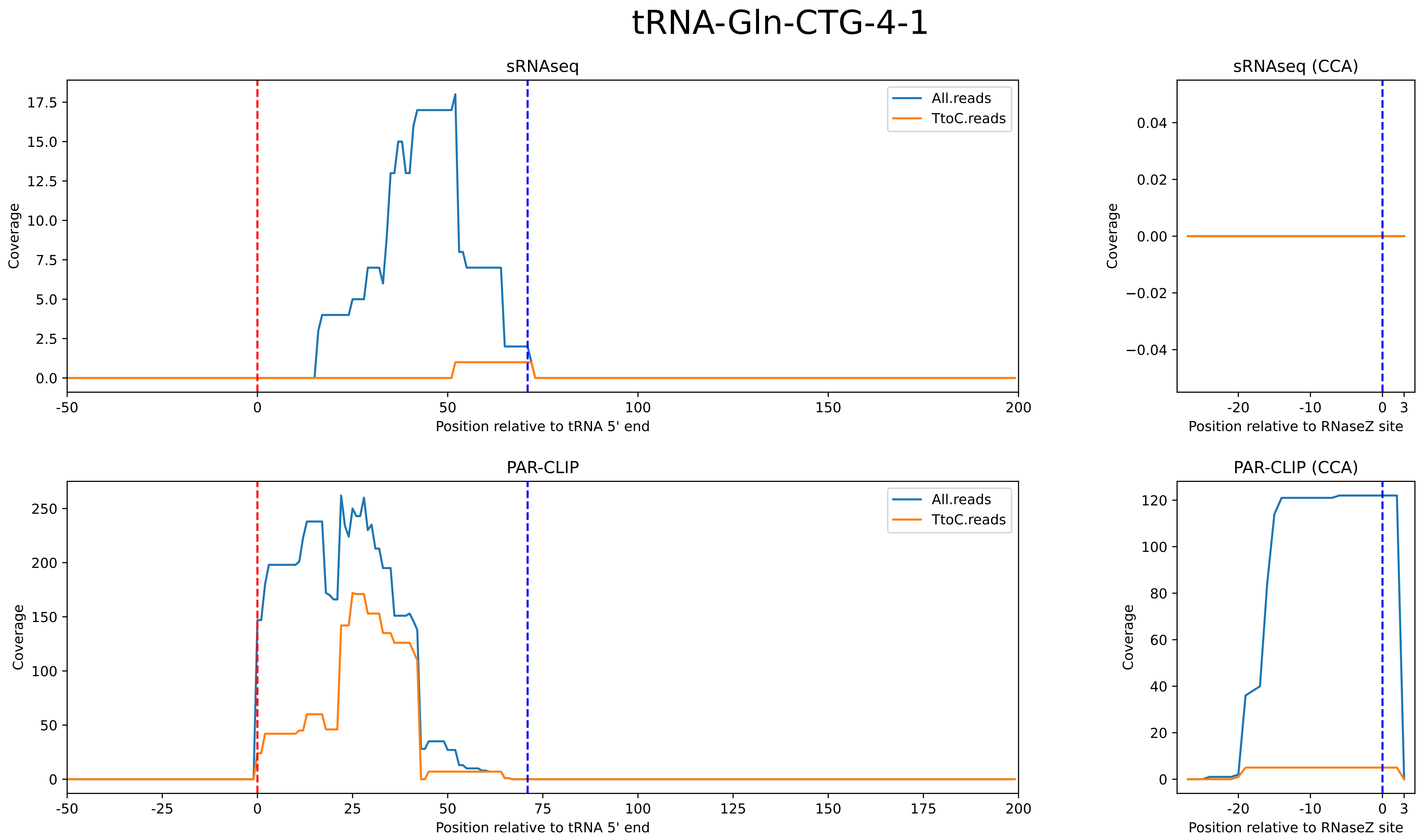
tRNA-Gln-CTG-4-1
sRNAseq
sRNAseq (CCA)
PAR-CLIP
PAR-CLIP (CCA)
All.reads
TtoC.reads
Coverage
Position relative to tRNA 5' end
Position relative to RNaseZ site
0.0
2.5
5.0
7.5
10.0
12.5
15.0
17.5
0.04
0.02
0.00
−0.02
−0.04
0
50
100
150
200
250
0
20
40
60
80
100
120
-50
-25
0
25
50
75
100
125
150
175
200
-20
-10
0
3

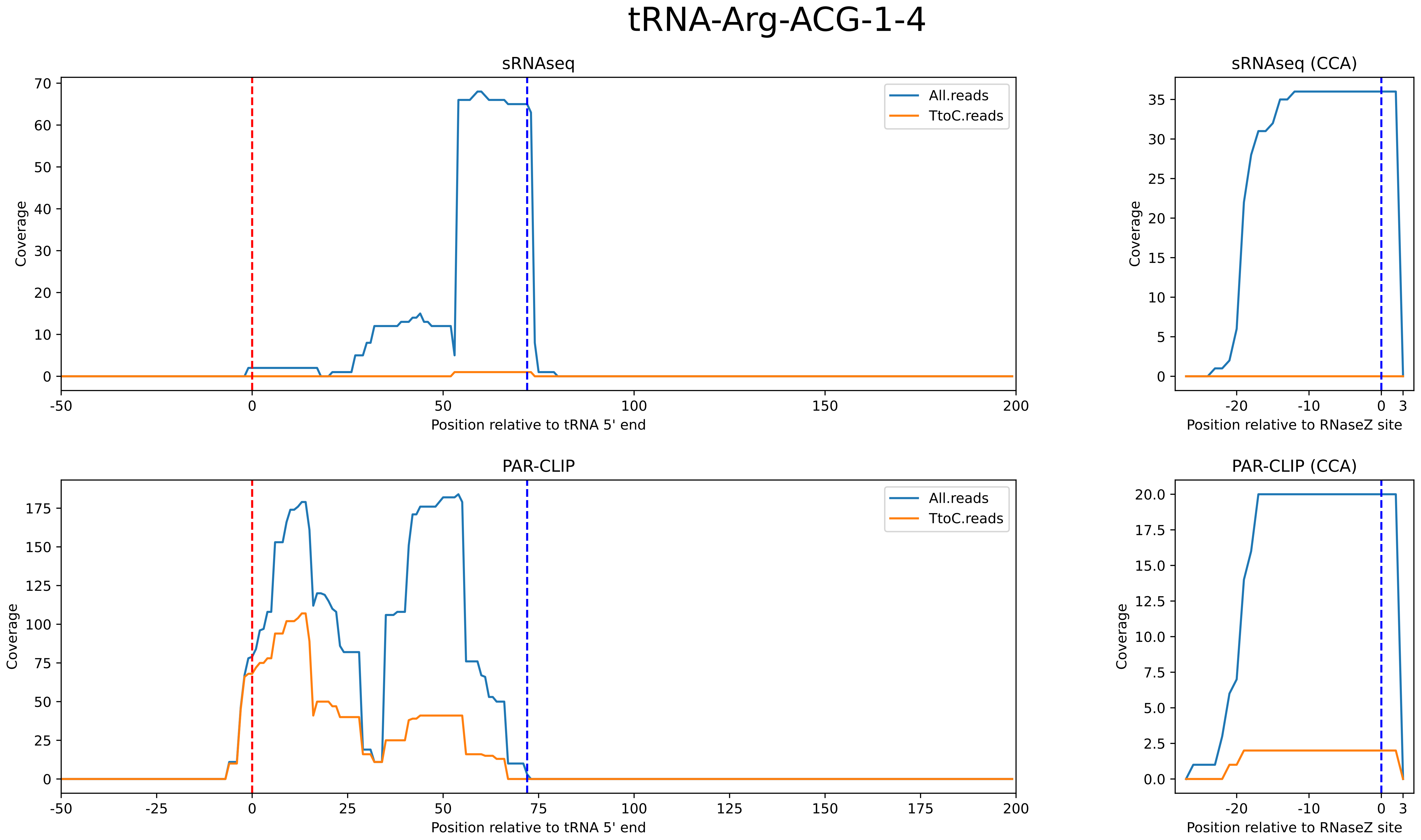

tRNA-Arg-ACG-1-4
sRNAseq
All.reads
TtoC.reads
Coverage
Position relative to tRNA 5' end
sRNAseq (CCA)
Position relative to RNaseZ site
PAR-CLIP
PAR-CLIP (CCA)

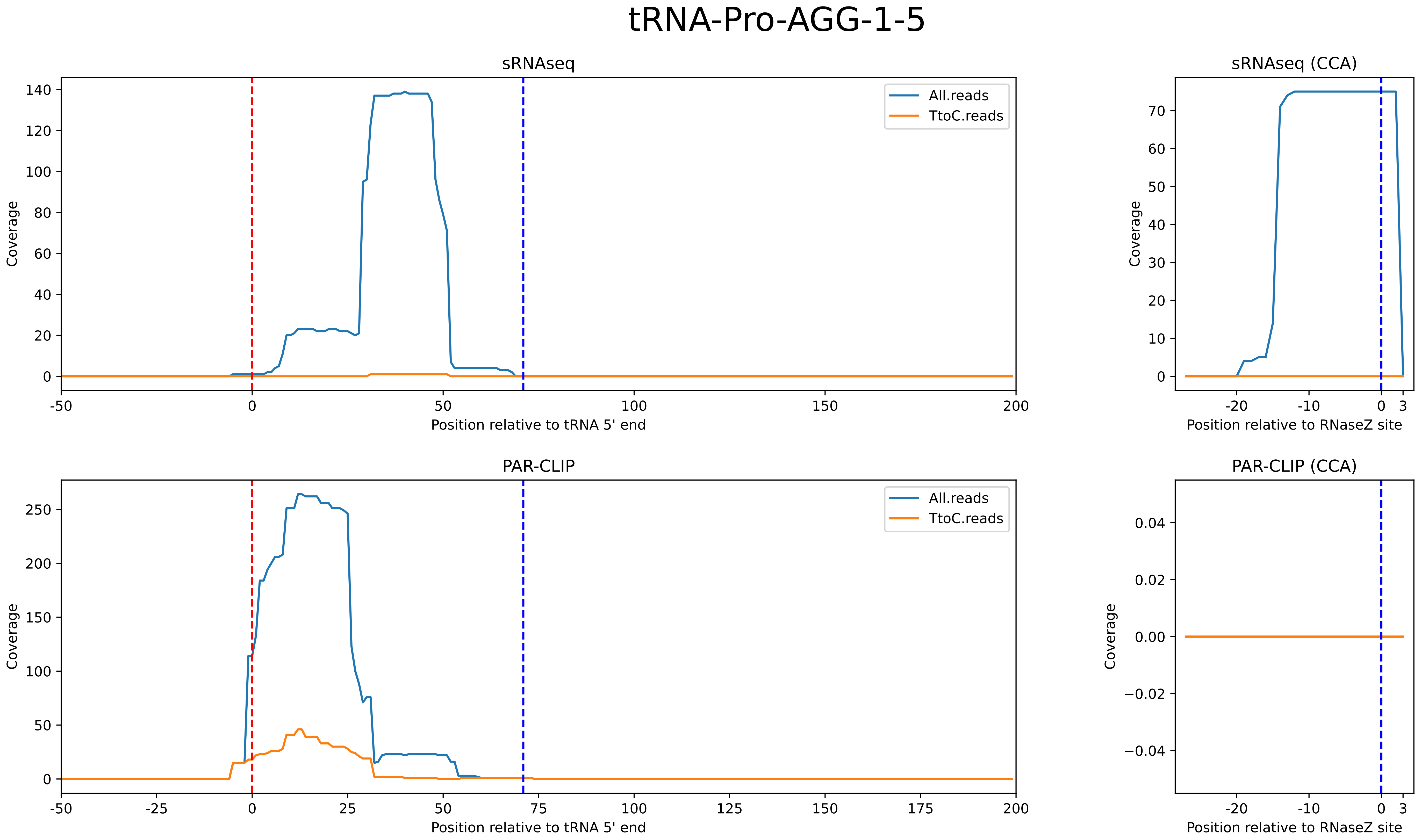
tRNA-Pro-AGG-1-5
sRNAseq
sRNAseq (CCA)
PAR-CLIP
PAR-CLIP (CCA)
All.reads
TtoC.reads
Coverage
Position relative to tRNA 5' end
Position relative to RNaseZ site

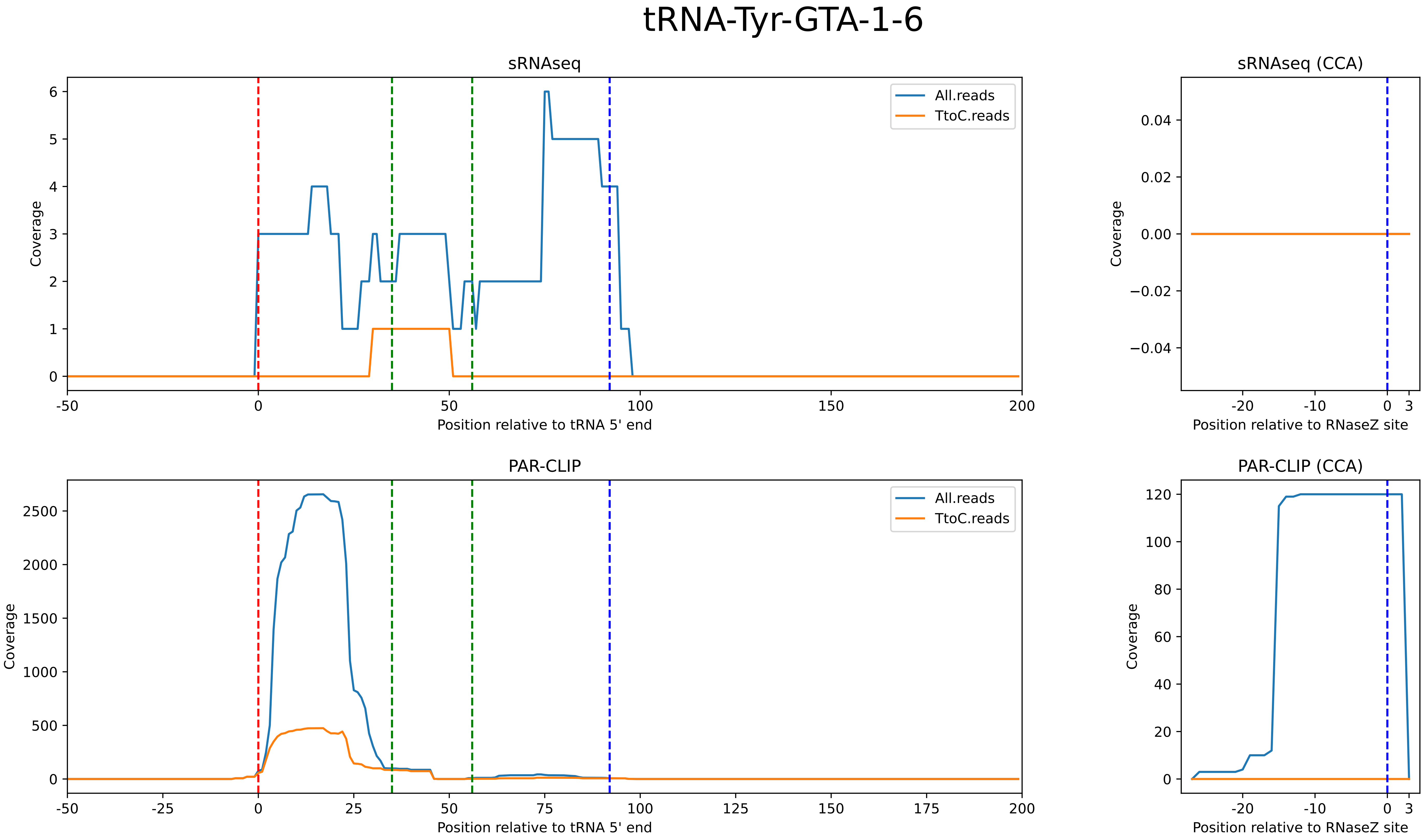

tRNA-Tyr-GTA-1-6
sRNAseq
All.reads
TtoC.reads
Coverage
Position relative to tRNA 5' end
sRNAseq (CCA)
Position relative to RNaseZ site
PAR-CLIP
All.reads
TtoC.reads
Coverage
Position relative to tRNA 5' end
PAR-CLIP (CCA)
Coverage
Position relative to RNaseZ site

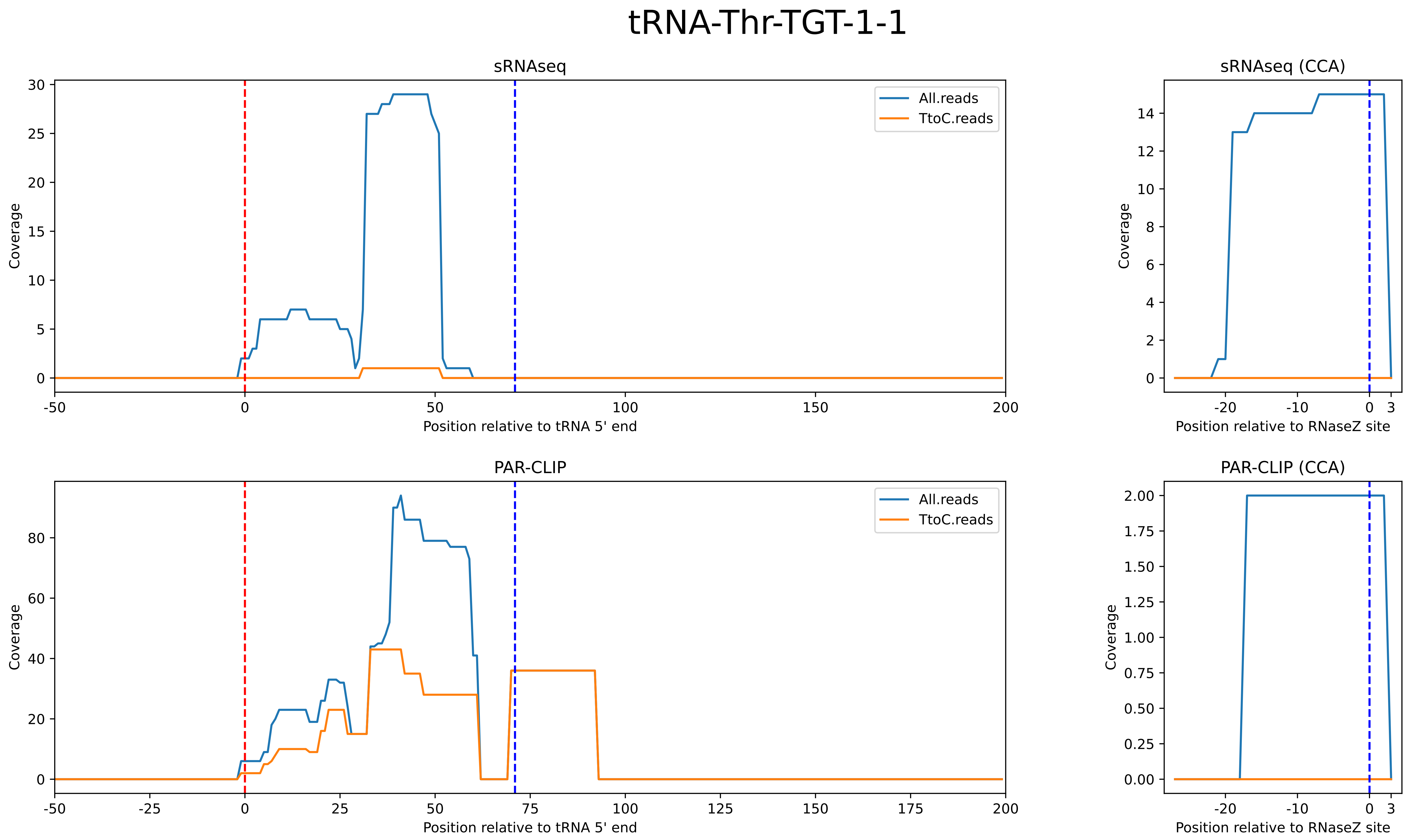

tRNA-Thr-TGT-1-1
sRNAseq
sRNAseq (CCA)
PAR-CLIP
PAR-CLIP (CCA)
All.reads
TtoC.reads
Coverage
Position relative to tRNA 5' end
Position relative to RNaseZ site

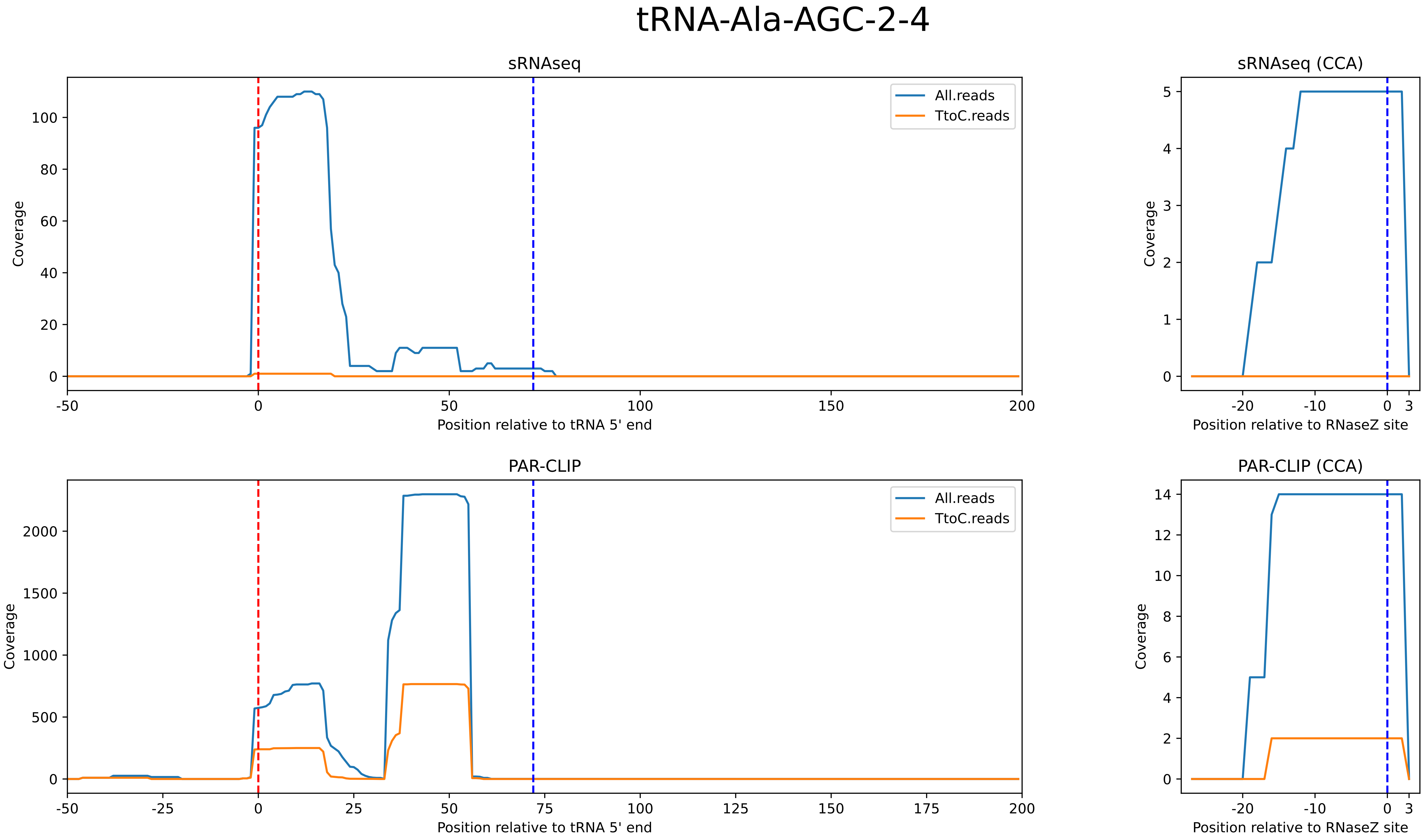
tRNA-Ala-AGC-2-4
sRNAseq
sRNAseq (CCA)
PAR-CLIP
PAR-CLIP (CCA)
All.reads
TtoC.reads
Coverage
Position relative to tRNA 5' end
Position relative to RNaseZ site

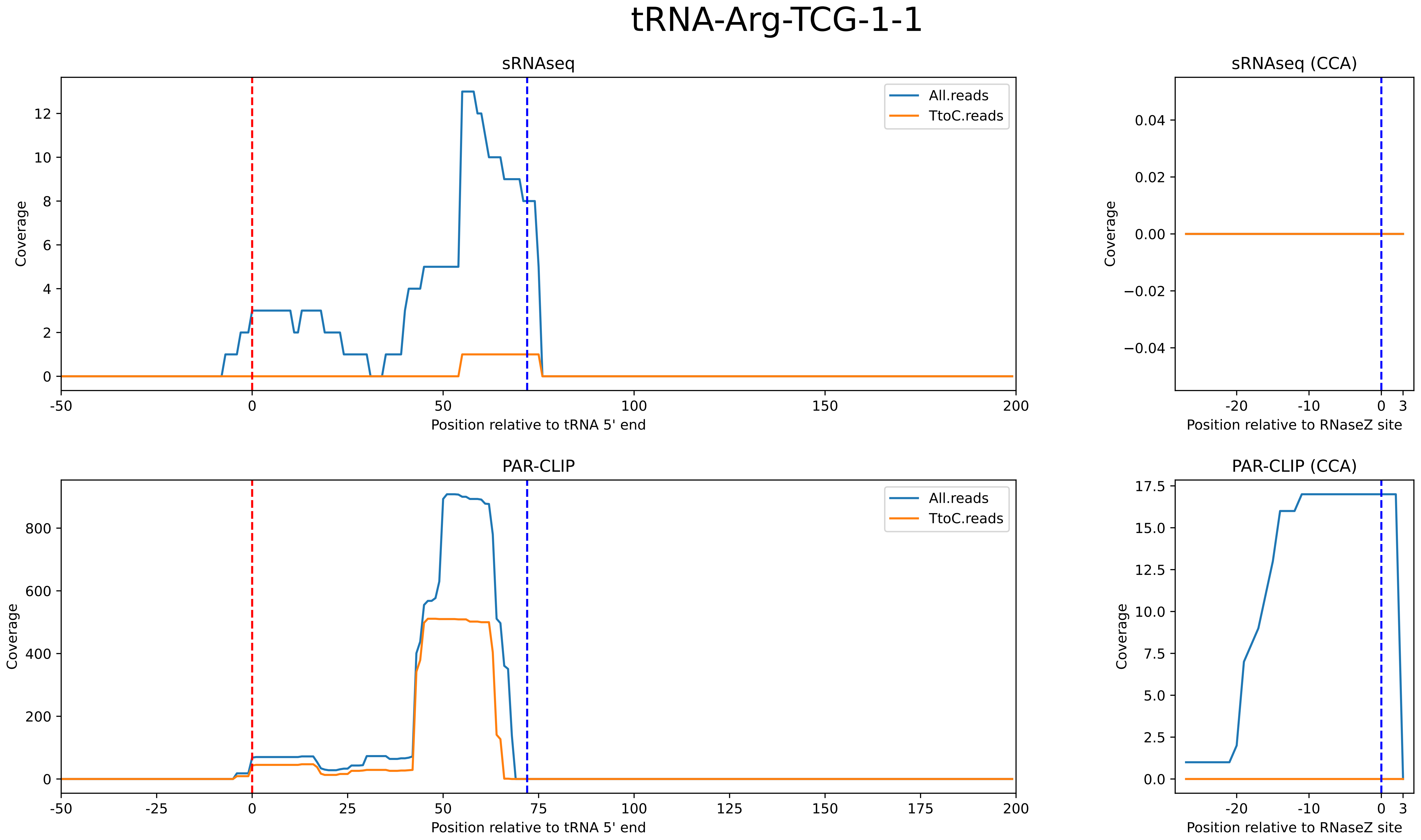
tRNA-Arg-TCG-1-1
sRNAseq
All.reads
TtoC.reads
Coverage
Position relative to tRNA 5' end
sRNAseq (CCA)
Position relative to RNaseZ site
PAR-CLIP
PAR-CLIP (CCA)

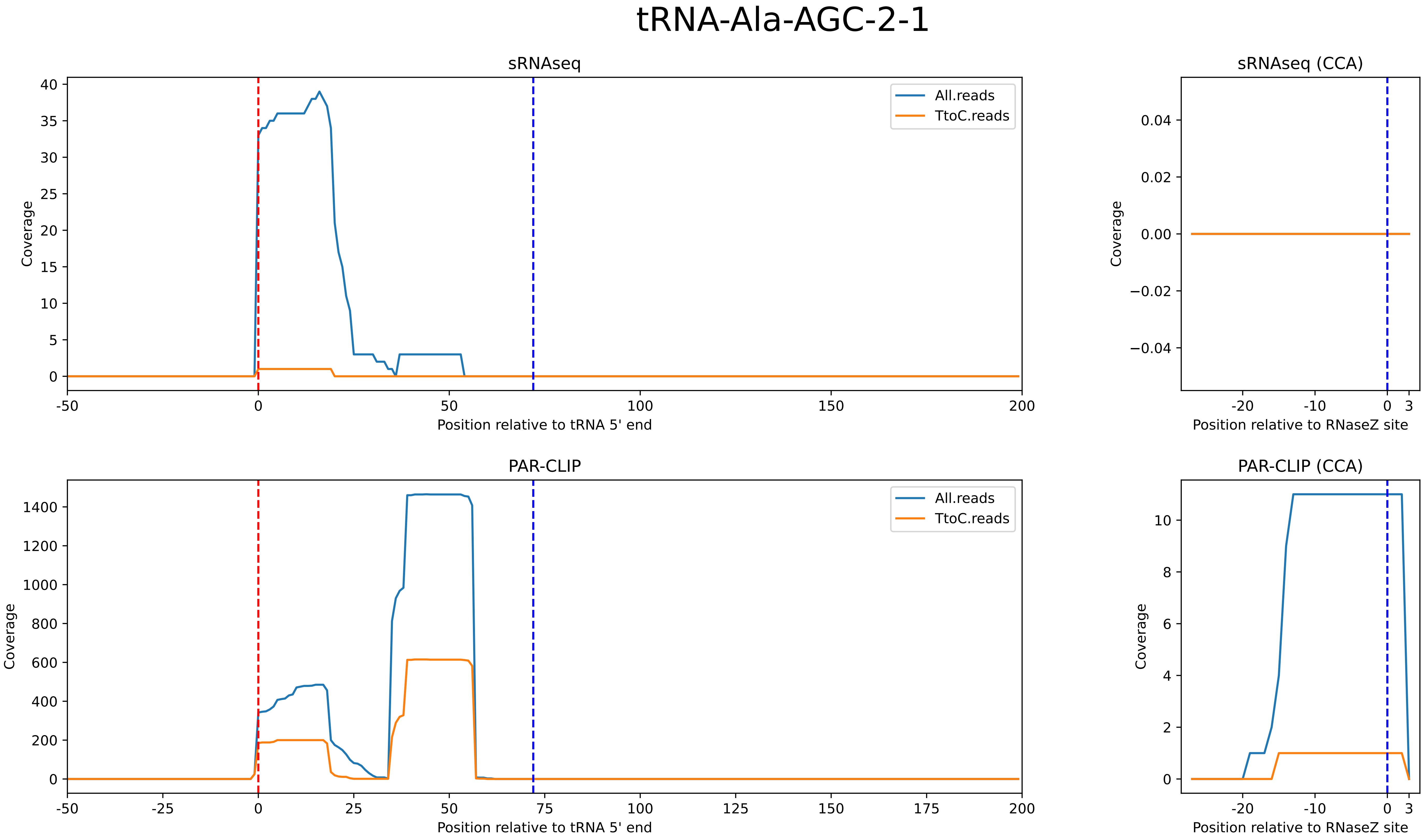

tRNA-Ala-AGC-2-1
sRNAseq
All.reads
TtoC.reads
Coverage
Position relative to tRNA 5' end
sRNAseq (CCA)
Position relative to RNaseZ site
PAR-CLIP
PAR-CLIP (CCA)

# tRNA-Thr-AGT-1-6

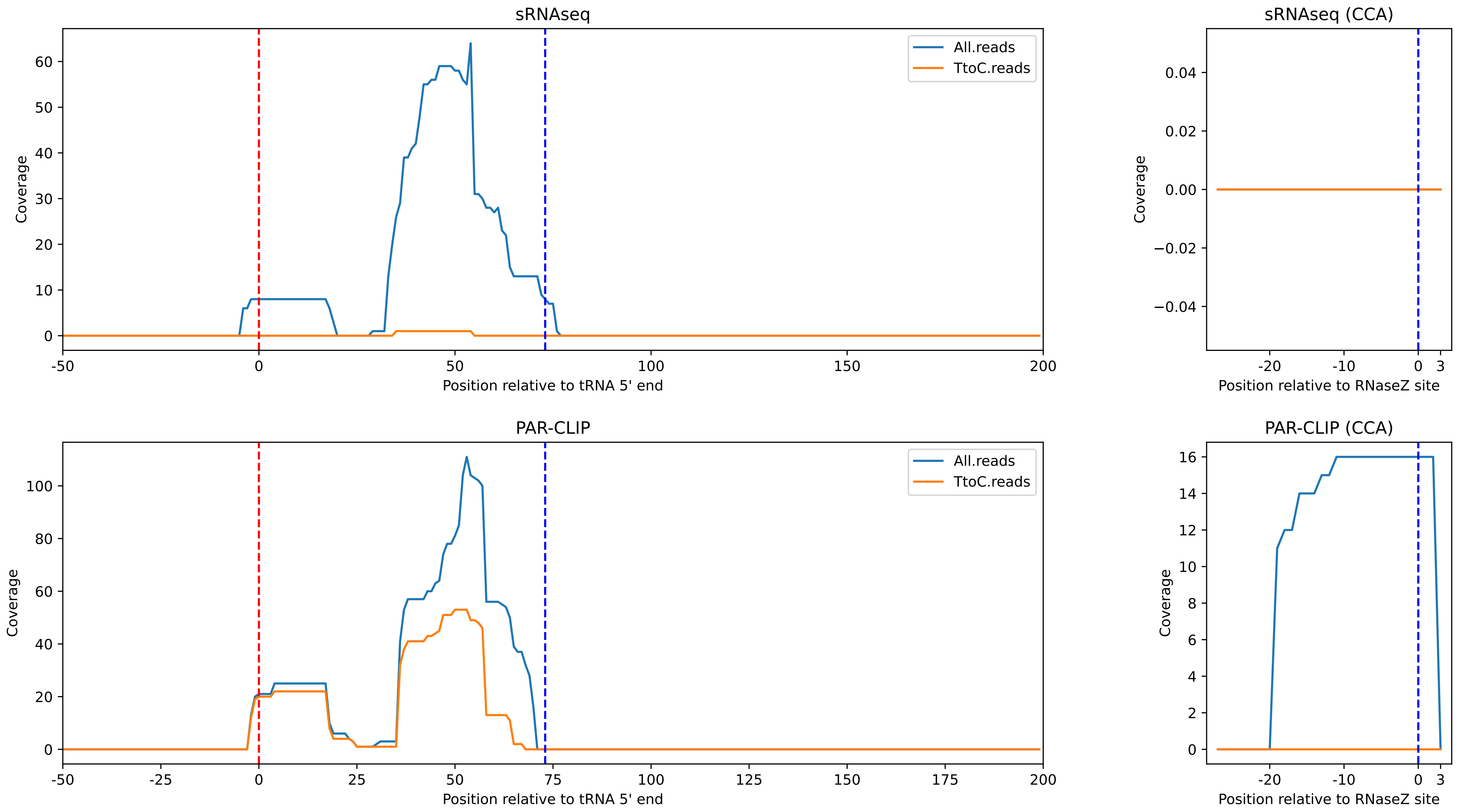

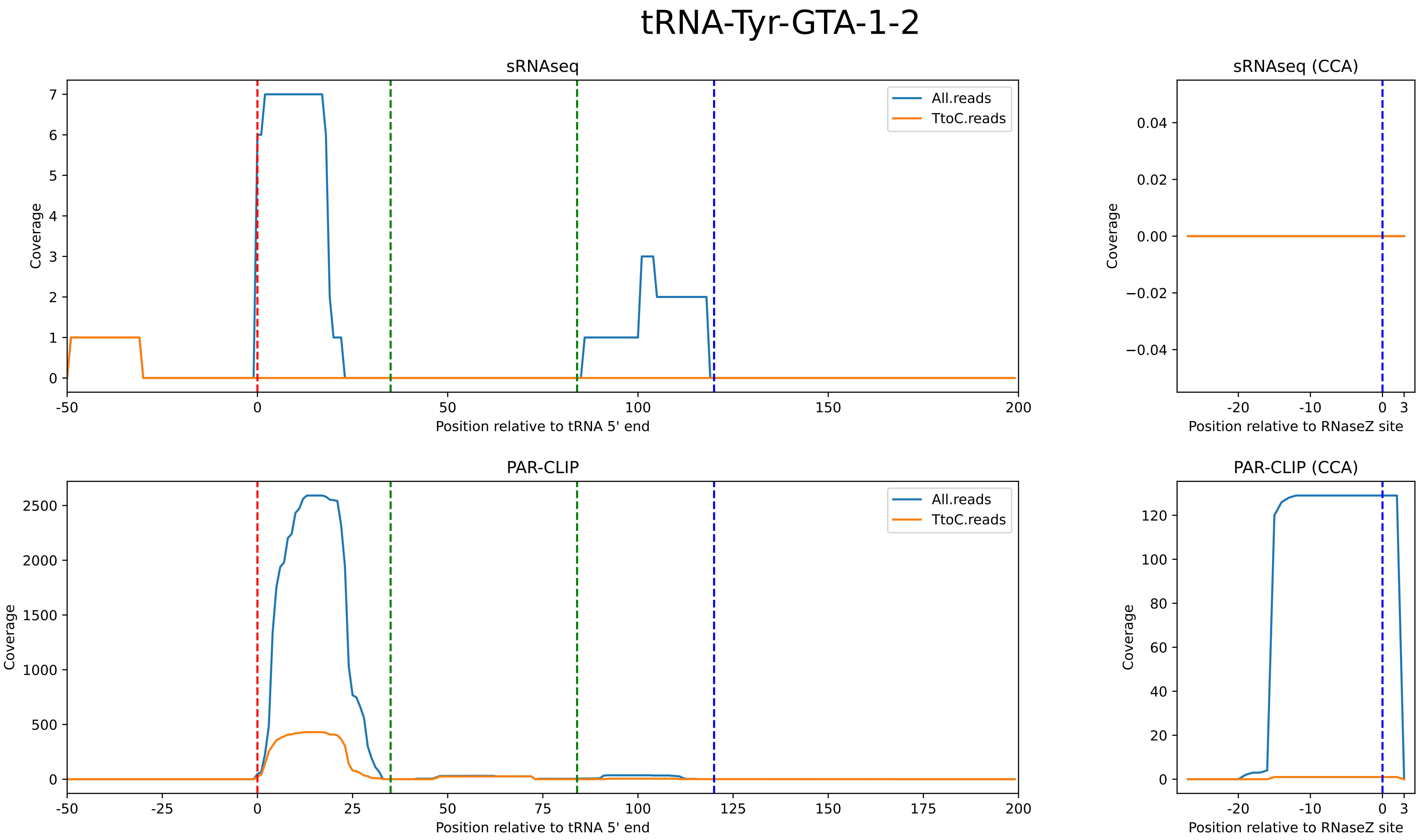
tRNA-Tyr-GTA-1-2
sRNAseq
All.reads
TtoC.reads
Coverage
Position relative to tRNA 5' end
-50
0
50
100
150
200
sRNAseq (CCA)
Position relative to RNaseZ site
PAR-CLIP
PAR-CLIP (CCA)
-25
25
75
125
175
-20
-10
3

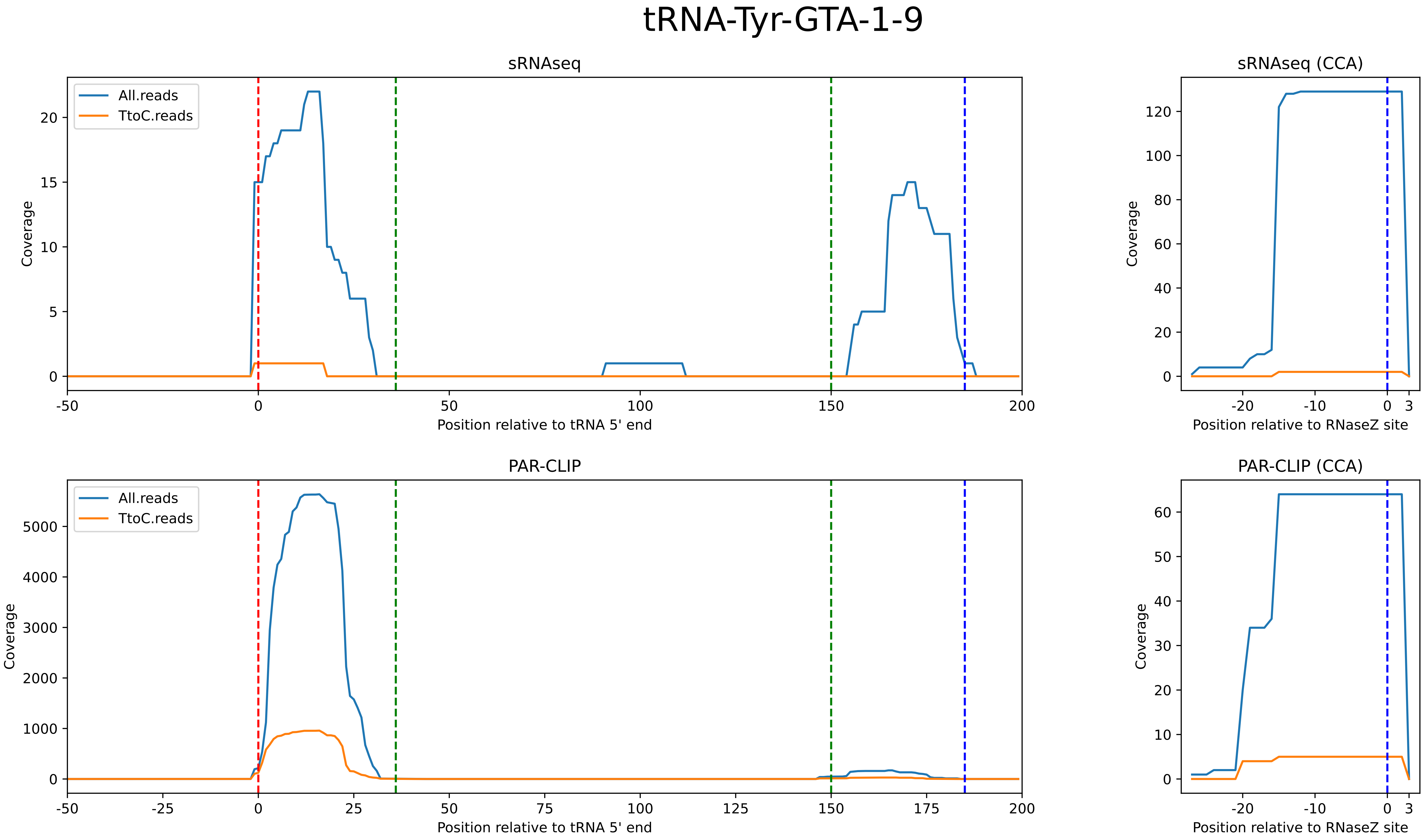

tRNA-Tyr-GTA-1-9
sRNAseq
All.reads
TtoC.reads
Coverage
Position relative to tRNA 5' end
sRNAseq (CCA)
Position relative to RNaseZ site
PAR-CLIP
PAR-CLIP (CCA)

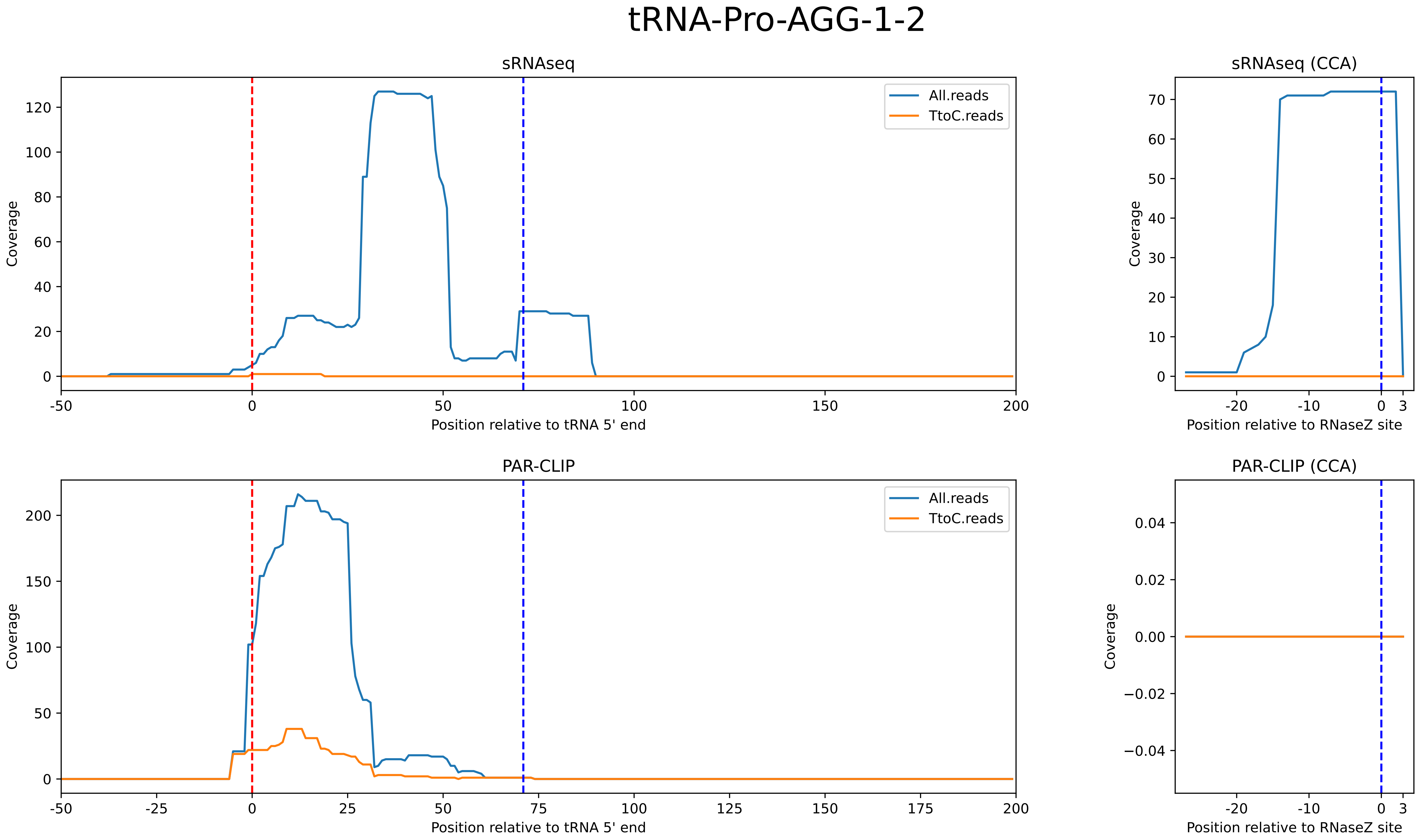
tRNA-Pro-AGG-1-2
sRNAseq
sRNAseq (CCA)
PAR-CLIP
PAR-CLIP (CCA)
All.reads
TtoC.reads
Coverage
Position relative to tRNA 5' end
Position relative to RNaseZ site

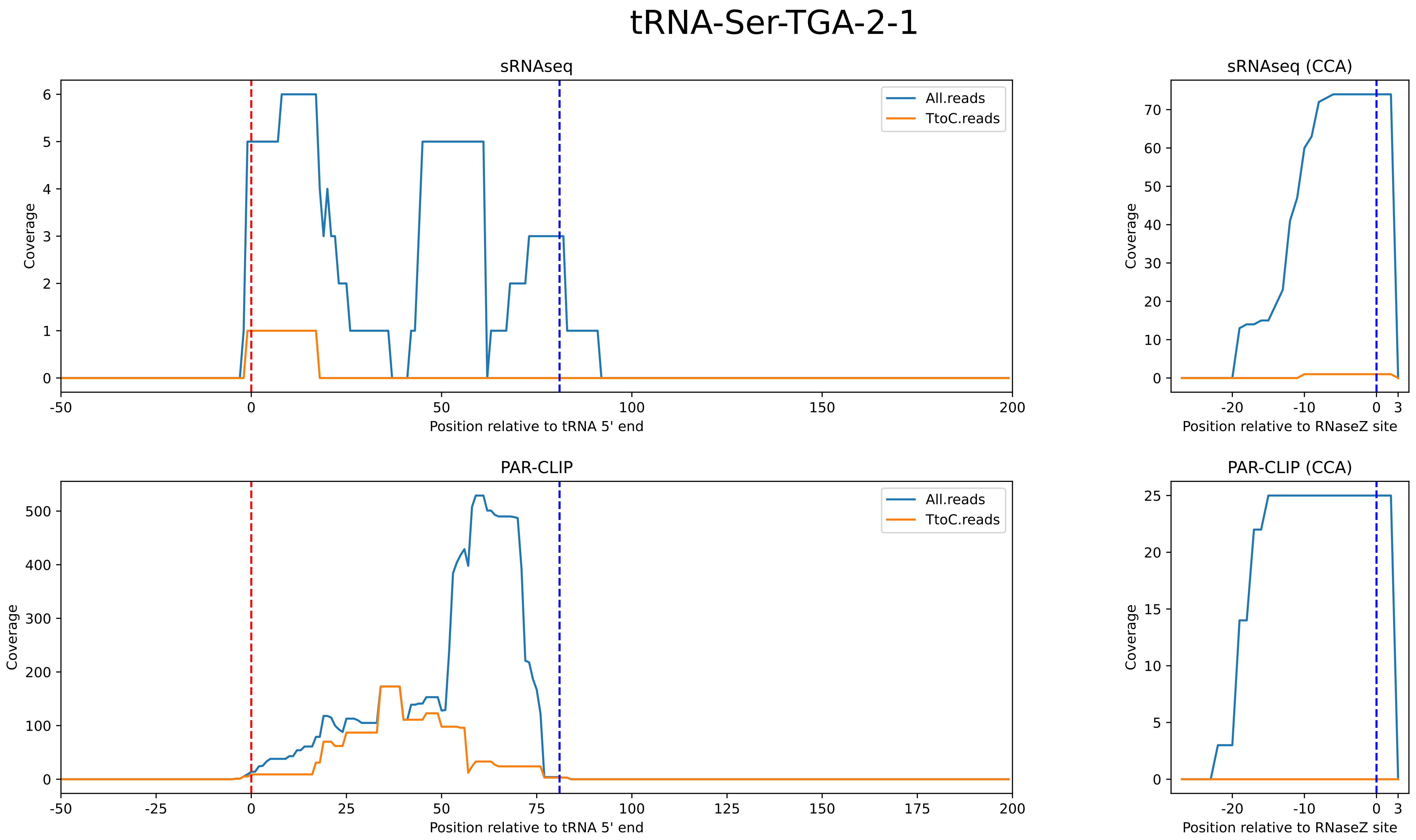
tRNA-Ser-TGA-2-1
sRNAseq
sRNAseq (CCA)
PAR-CLIP
PAR-CLIP (CCA)
All.reads
TtoC.reads
Coverage
Position relative to tRNA 5' end
Position relative to RNaseZ site

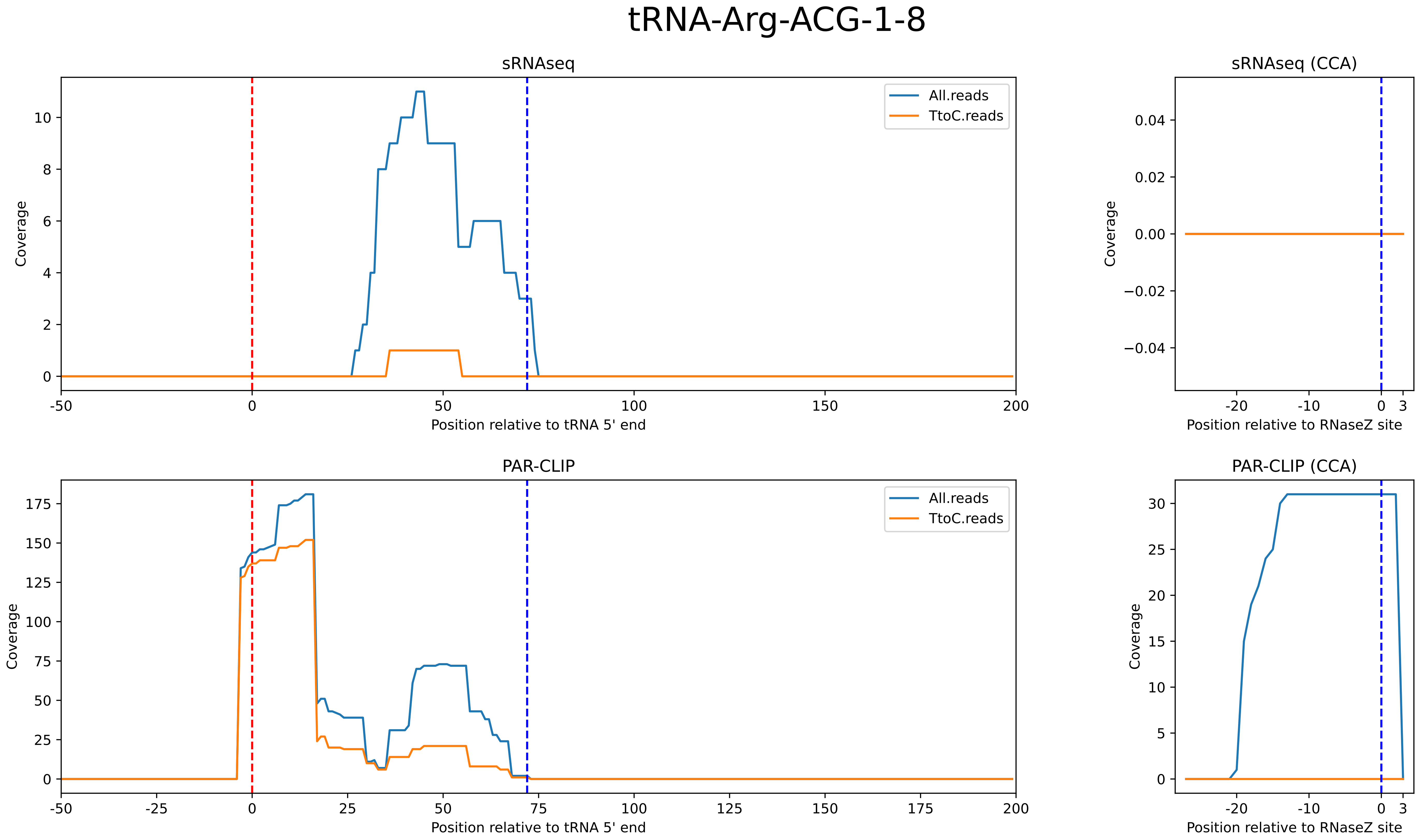

tRNA-Arg-ACG-1-8
sRNAseq
All.reads
TtoC.reads
Coverage
Position relative to tRNA 5' end
sRNAseq (CCA)
Position relative to RNaseZ site
PAR-CLIP
PAR-CLIP (CCA)

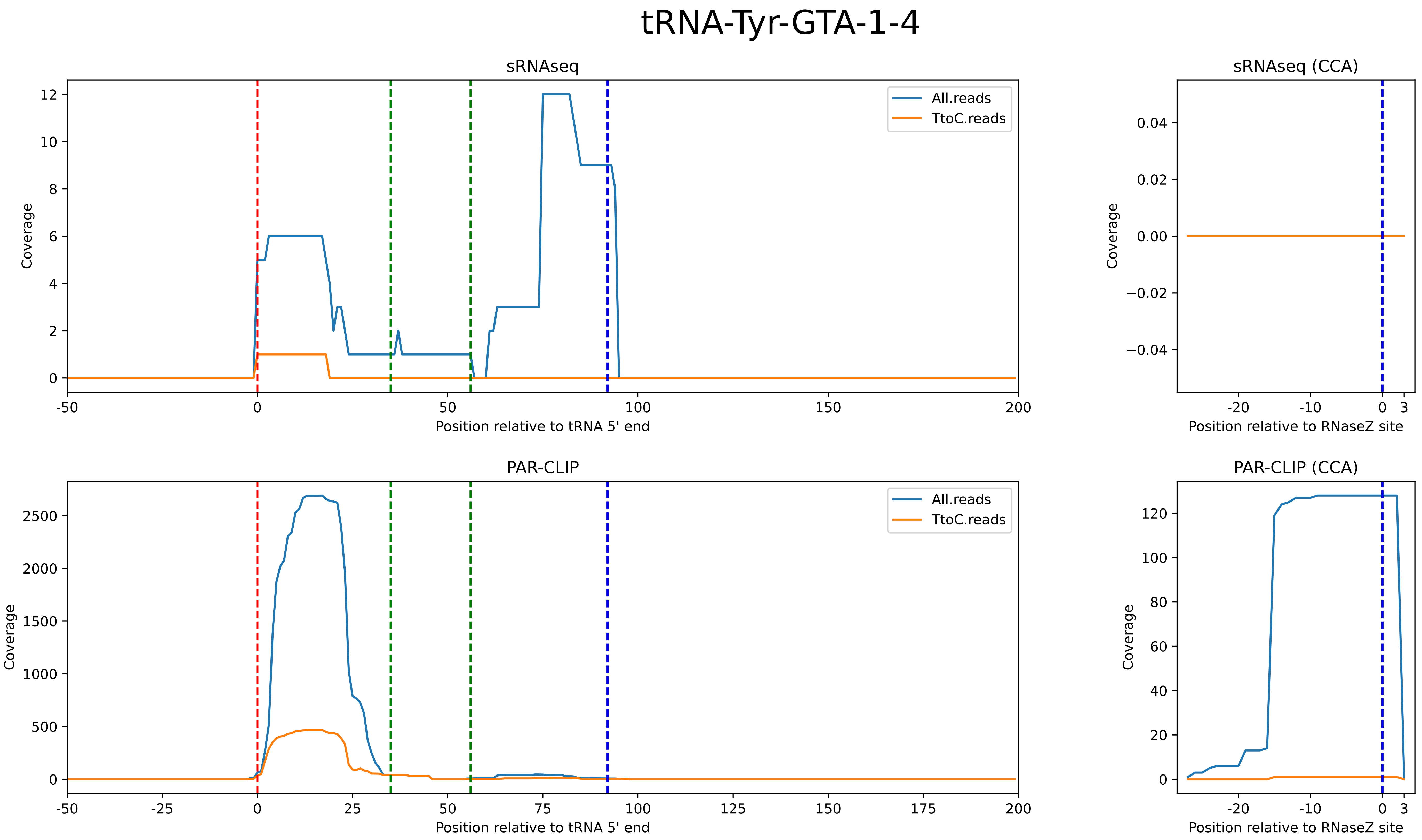
tRNA-Tyr-GTA-1-4
sRNAseq
sRNAseq (CCA)
PAR-CLIP
PAR-CLIP (CCA)
All.reads
TtoC.reads
Coverage
Position relative to tRNA 5' end
Position relative to RNaseZ site

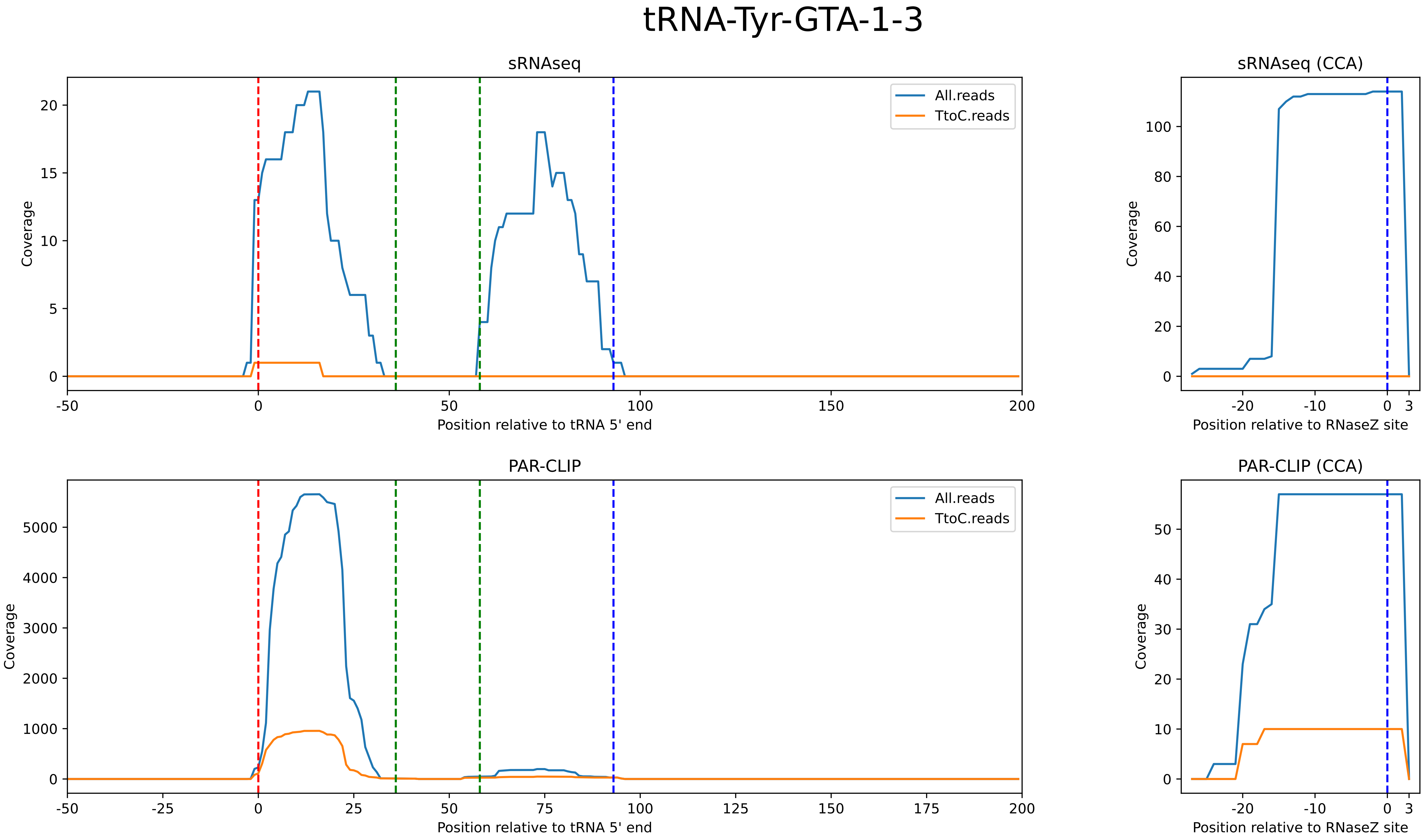
tRNA-Tyr-GTA-1-3
sRNAseq
sRNAseq (CCA)
PAR-CLIP
PAR-CLIP (CCA)
All.reads
TtoC.reads
Coverage
Position relative to tRNA 5' end
Position relative to RNaseZ site

# tRNA-Arg-ACG-1-3

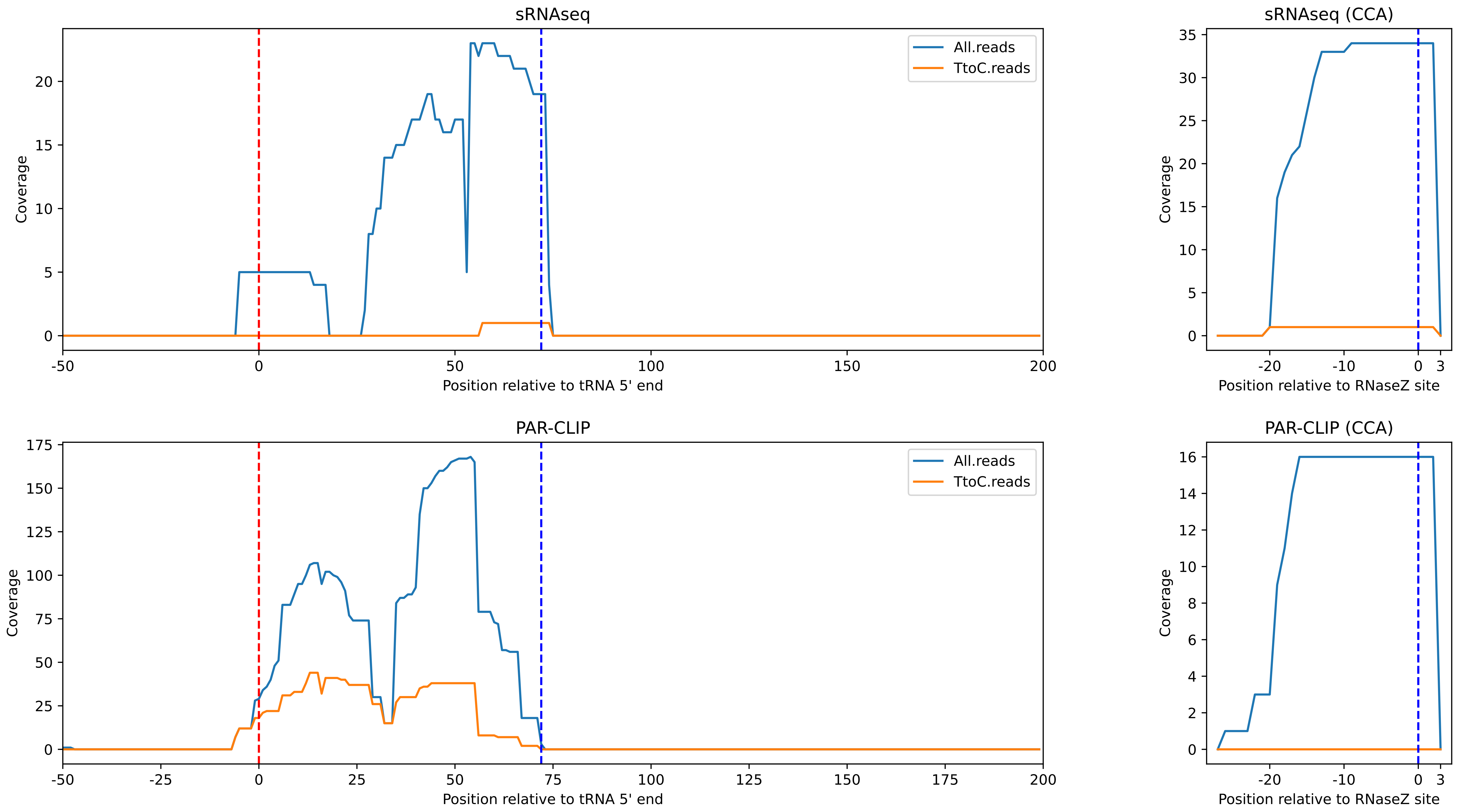

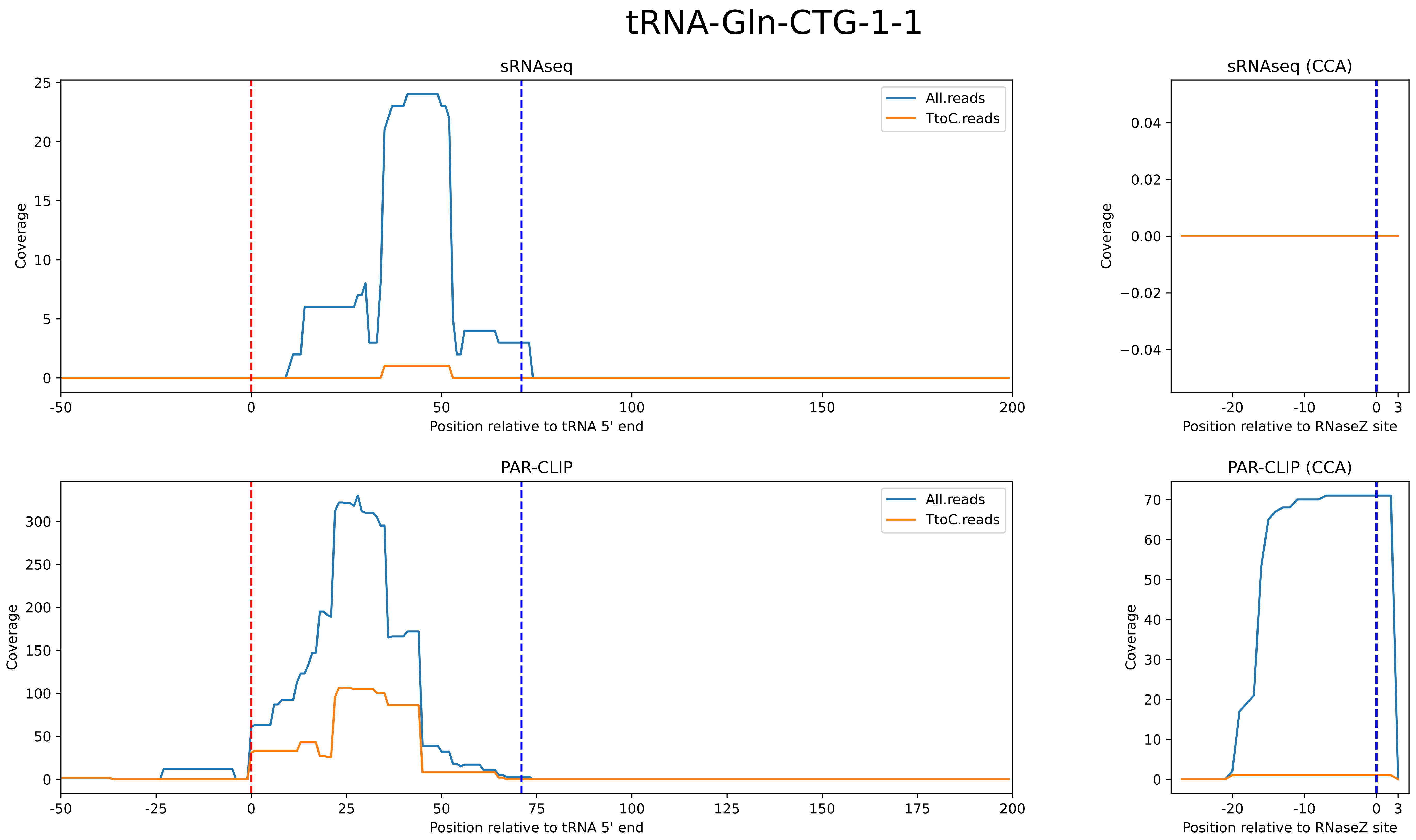
tRNA-Gln-CTG-1-1
sRNAseq
sRNAseq (CCA)
PAR-CLIP
PAR-CLIP (CCA)
All.reads
TtoC.reads
Coverage
Position relative to tRNA 5' end
Position relative to RNaseZ site

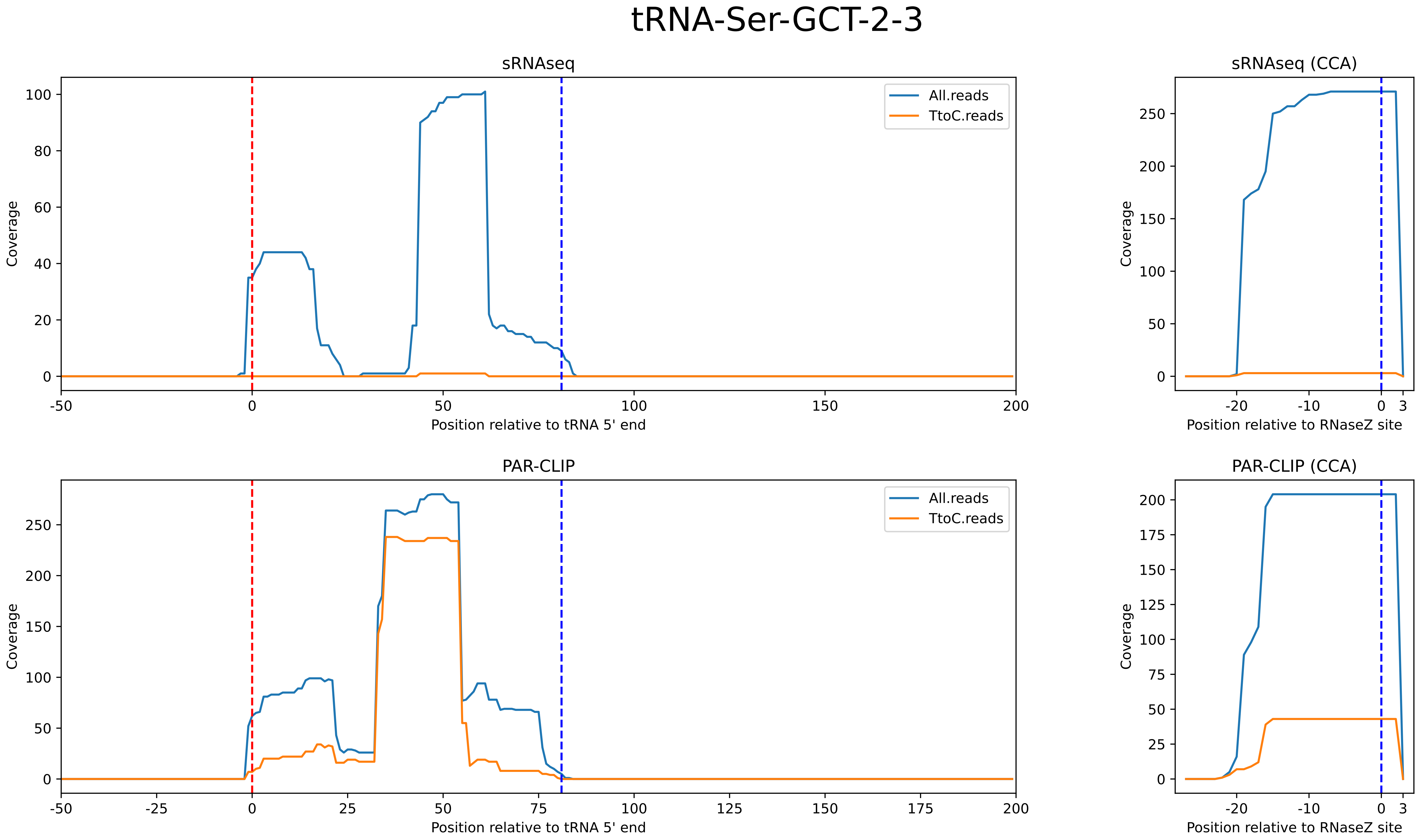

tRNA-Ser-GCT-2-3
sRNAseq
sRNAseq (CCA)
PAR-CLIP
PAR-CLIP (CCA)
All.reads
TtoC.reads
Coverage
Position relative to tRNA 5' end
Position relative to RNaseZ site

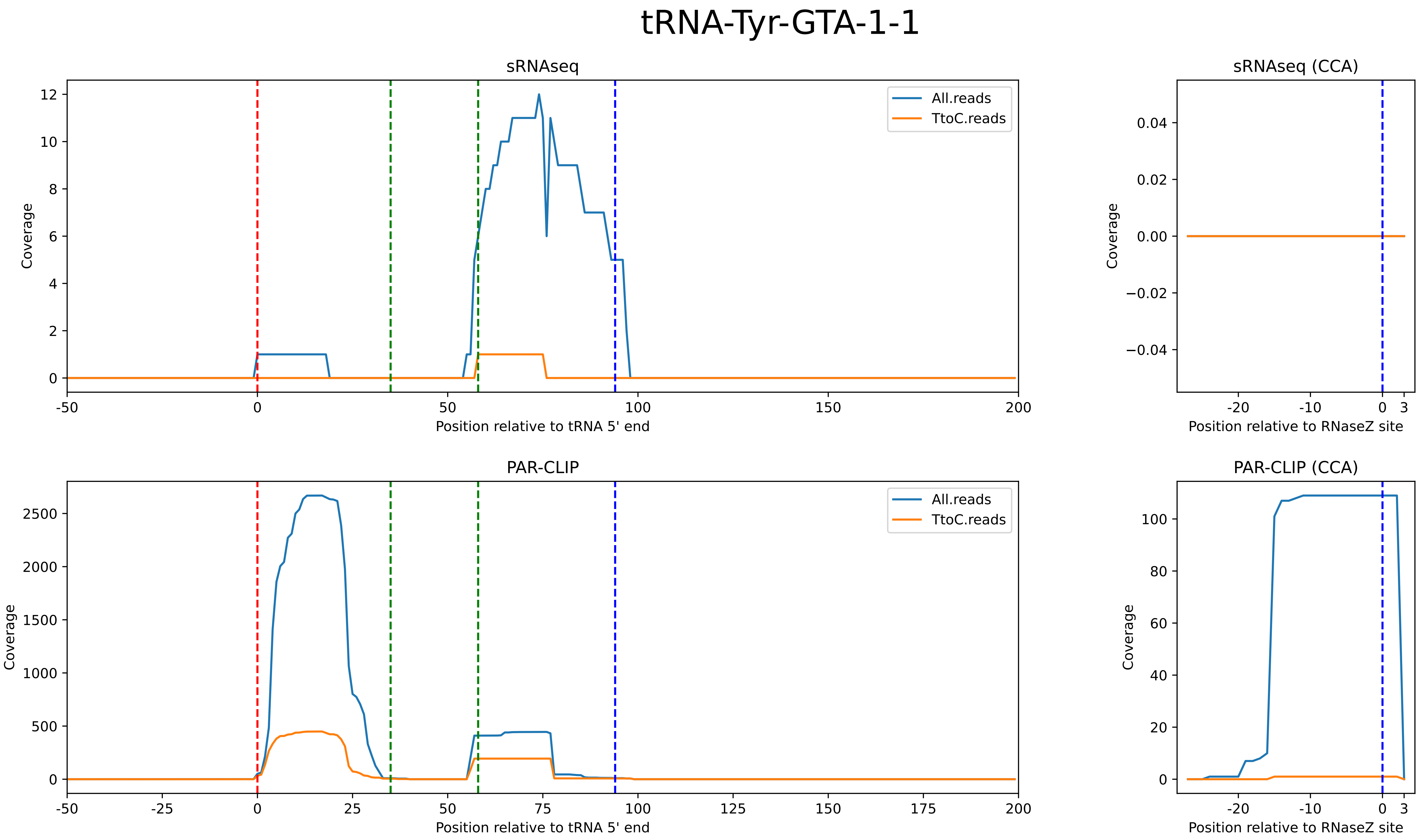
tRNA-Tyr-GTA-1-1
sRNAseq
All.reads
TtoC.reads
Coverage
Position relative to tRNA 5' end
sRNAseq (CCA)
Position relative to RNaseZ site
PAR-CLIP
PAR-CLIP (CCA)

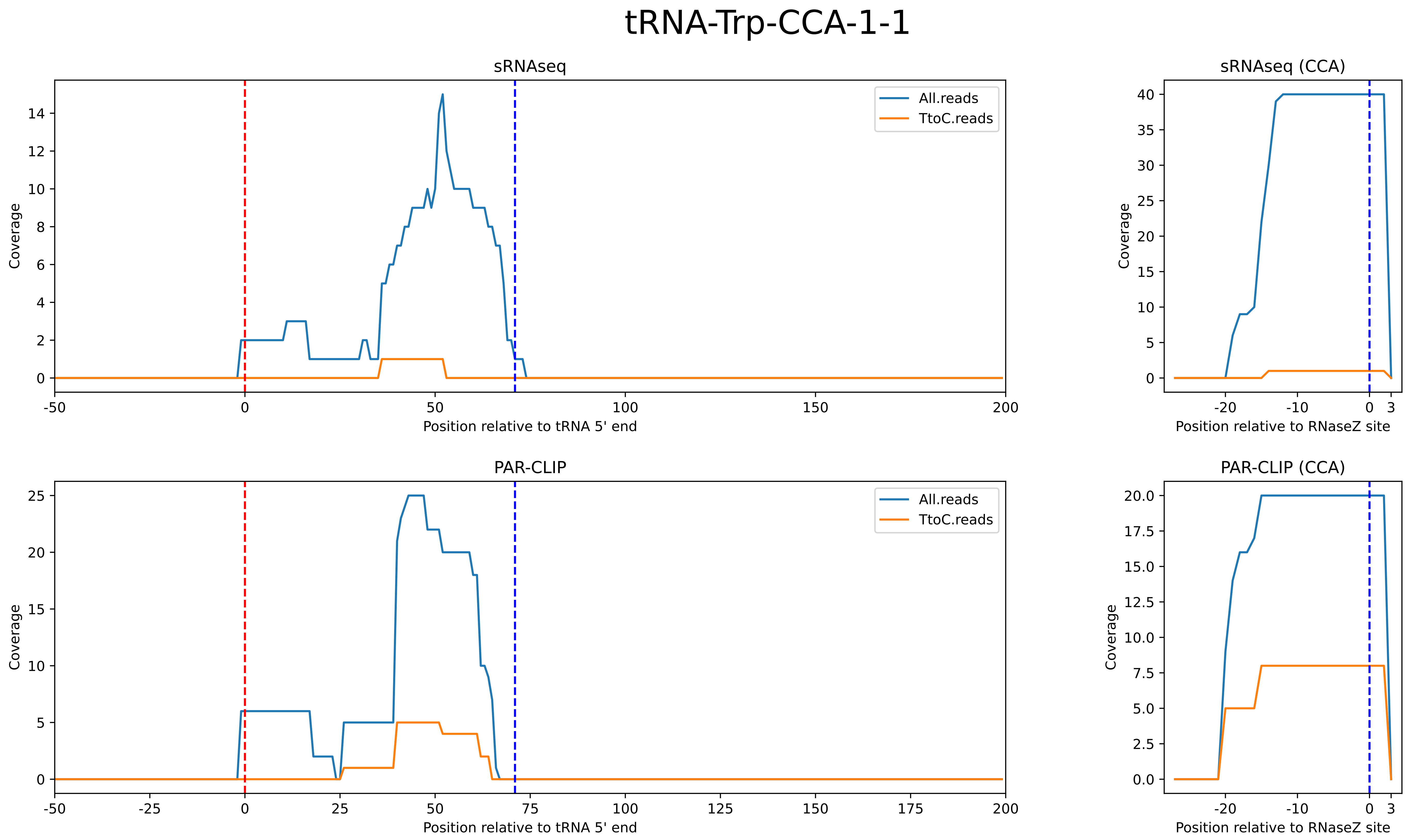
tRNA-Trp-CCA-1-1
sRNAseq
sRNAseq (CCA)
PAR-CLIP
PAR-CLIP (CCA)
All.reads
TtoC.reads
Coverage
Position relative to tRNA 5' end
Position relative to RNaseZ site

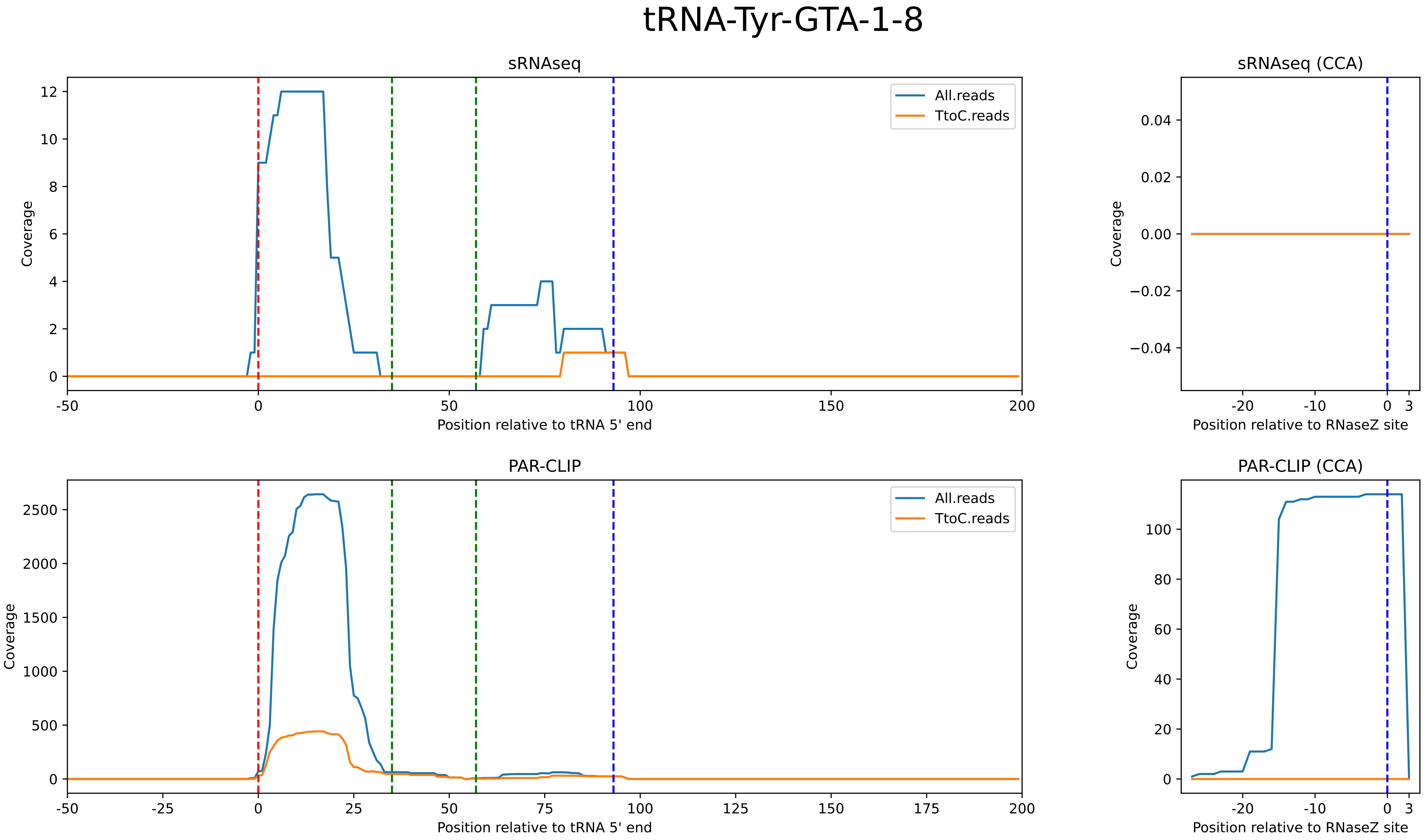

tRNA-Tyr-GTA-1-8
sRNAseq
All.reads
TtoC.reads
Coverage
Position relative to tRNA 5' end
sRNAseq (CCA)
Position relative to RNaseZ site
PAR-CLIP
PAR-CLIP (CCA)

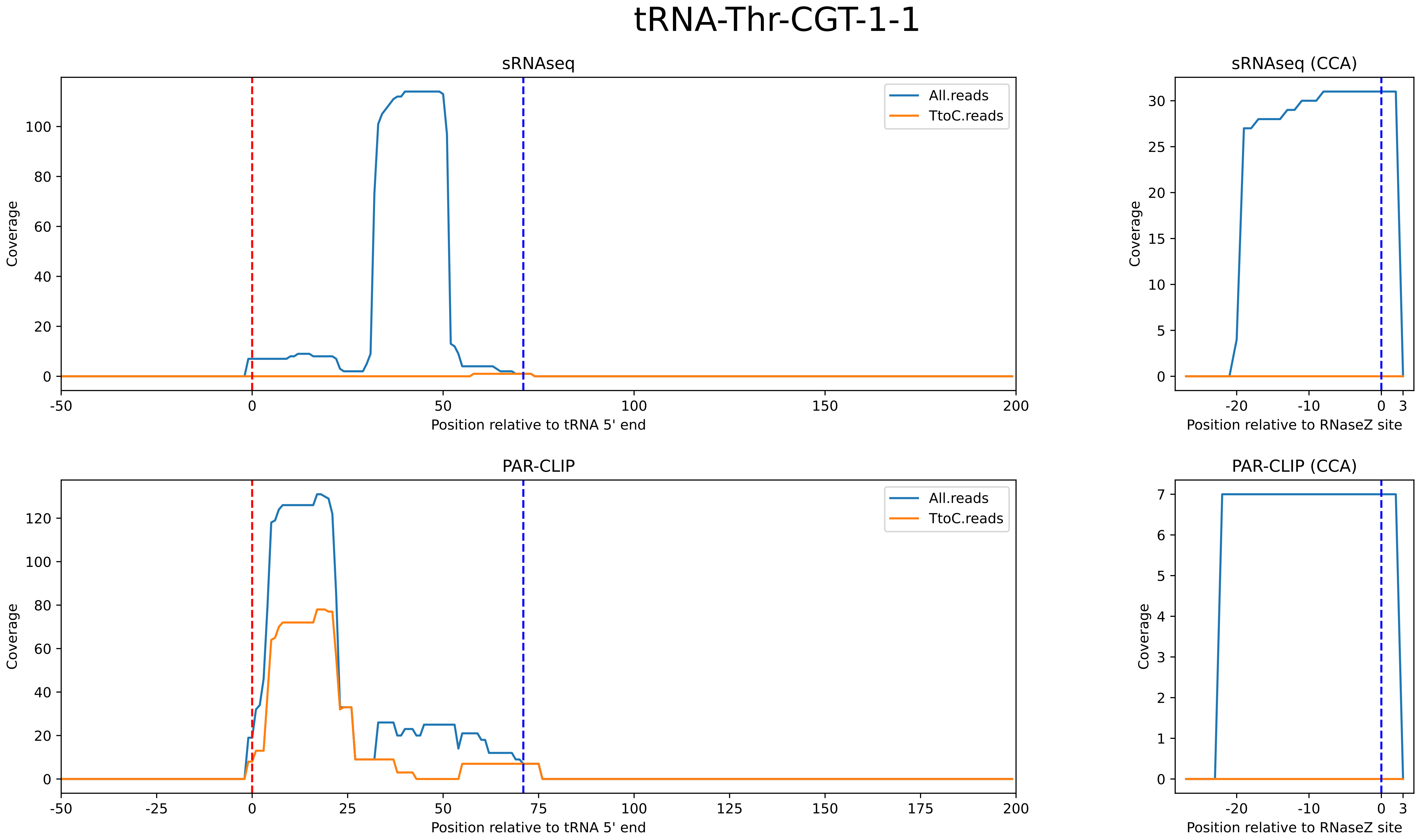

tRNA-Thr-CGT-1-1
sRNAseq
All.reads
TtoC.reads
Coverage
Position relative to tRNA 5' end
sRNAseq (CCA)
Position relative to RNaseZ site
PAR-CLIP
PAR-CLIP (CCA)

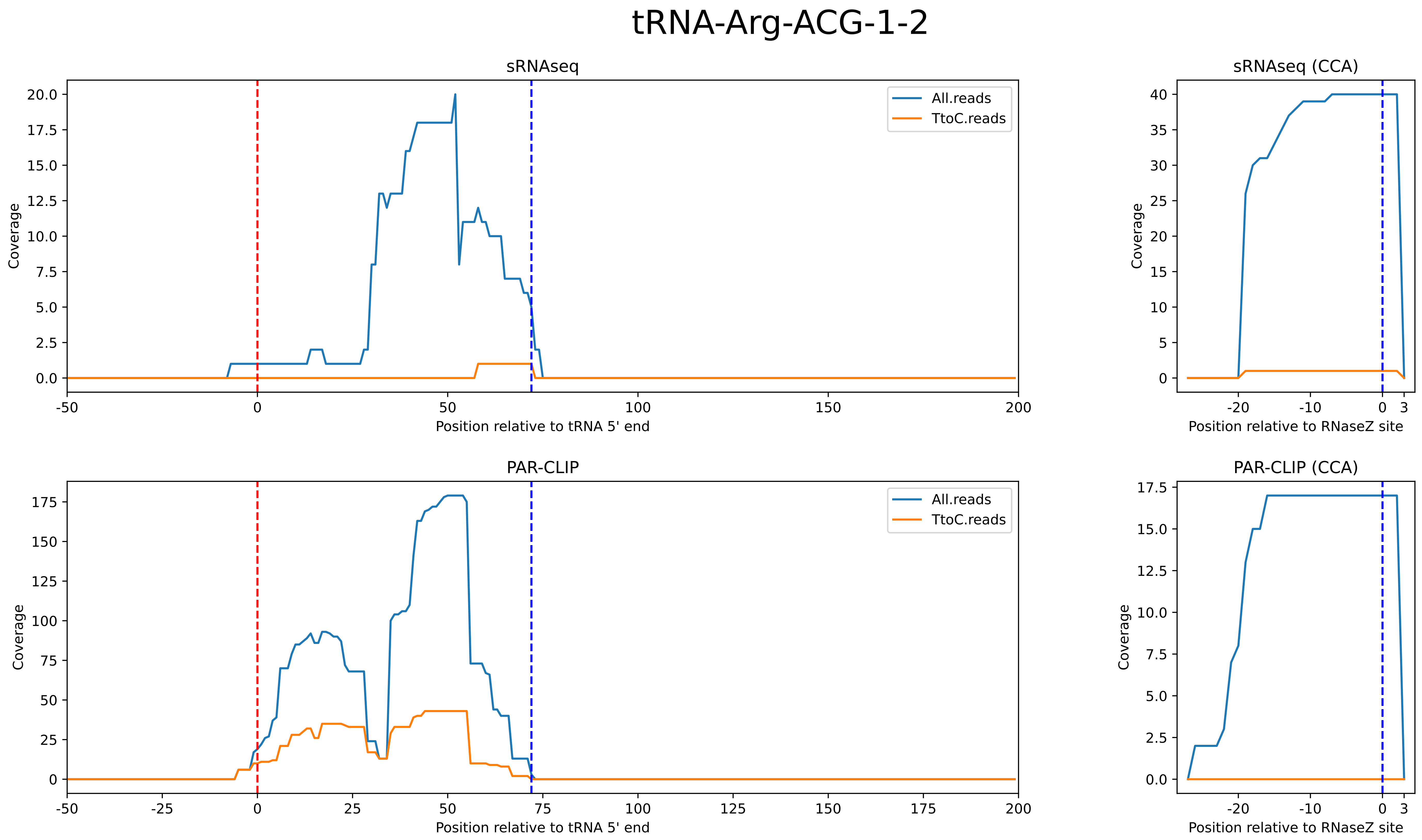
tRNA-Arg-ACG-1-2
sRNAseq
sRNAseq (CCA)
PAR-CLIP
PAR-CLIP (CCA)
All.reads
TtoC.reads
Coverage
Position relative to tRNA 5' end
Position relative to RNaseZ site

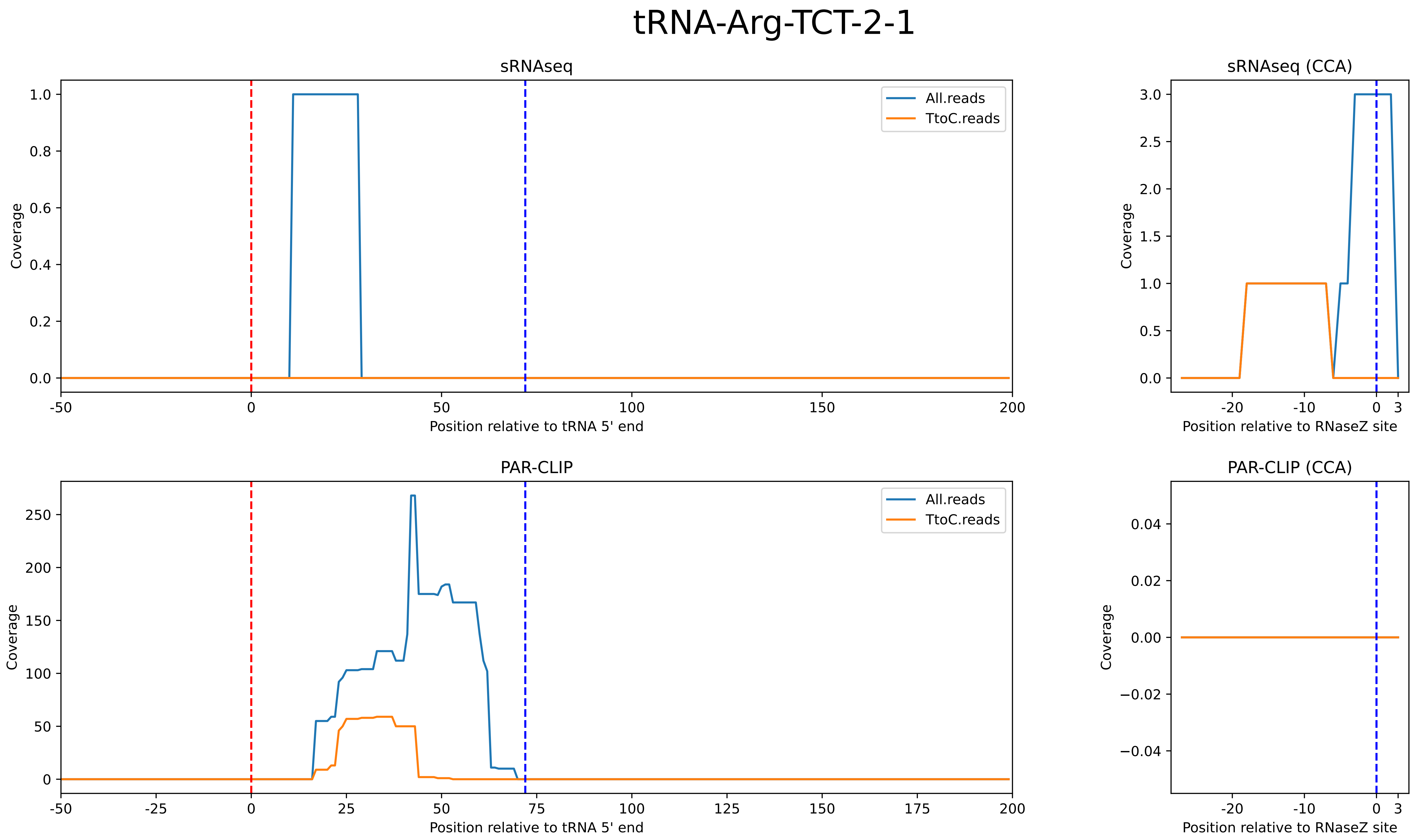

tRNA-Arg-TCT-2-1
sRNAseq
All.reads
TtoC.reads
Coverage
1.0
0.8
0.6
0.4
0.2
0.0
-50
0
50
100
150
200
Position relative to tRNA 5' end
sRNAseq (CCA)
3.0
2.5
2.0
1.5
1.0
0.5
0.0
-20
-10
0
3
Position relative to RNaseZ site
PAR-CLIP
250
200
150
100
50
0
-25
25
75
125
175
PAR-CLIP (CCA)
0.04
0.02
0.00
−0.02
−0.04

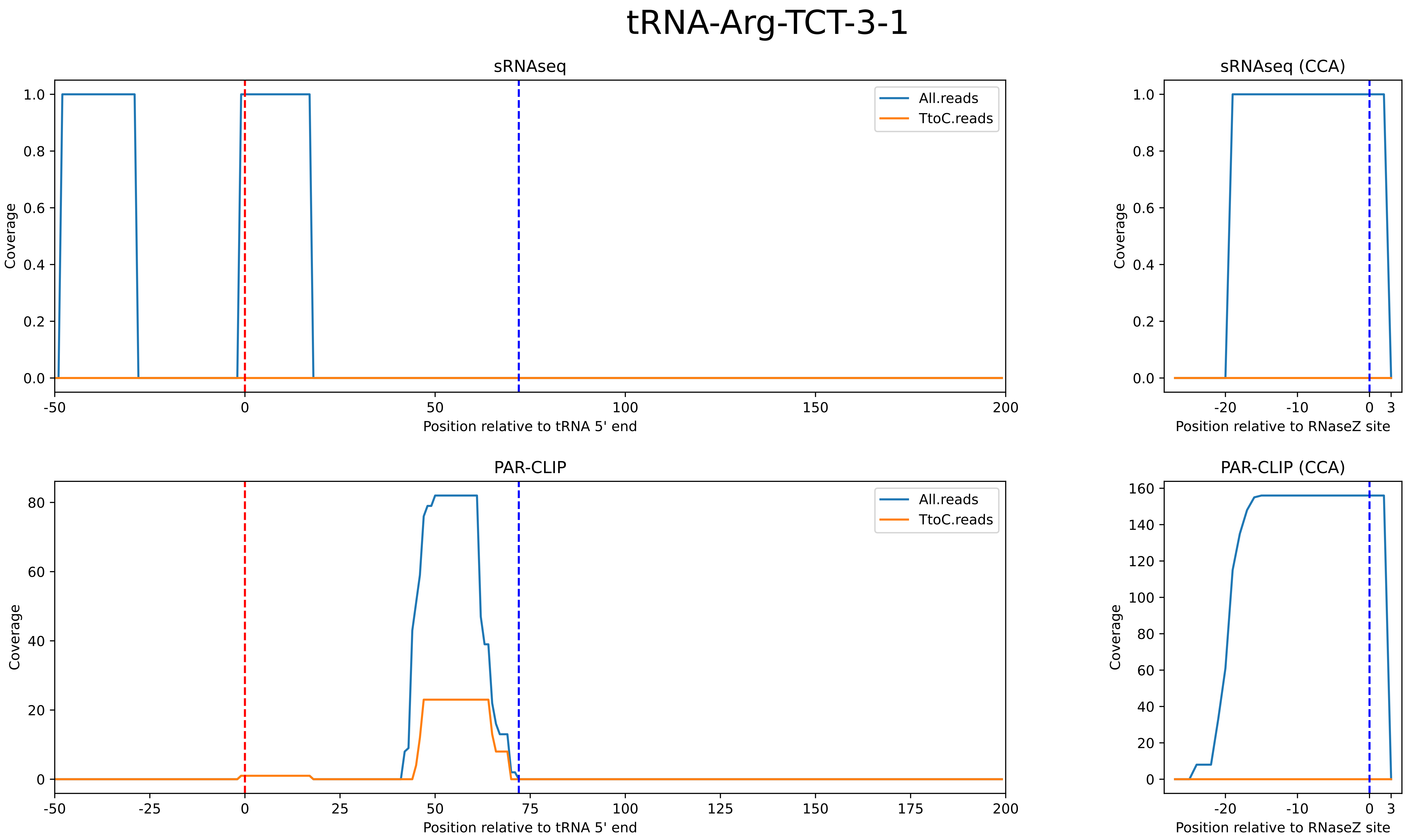

tRNA-Arg-TCT-3-1
sRNAseq
All.reads
TtoC.reads
Coverage
1.0
0.8
0.6
0.4
0.2
0.0
-50
0
50
100
150
200
Position relative to tRNA 5' end
sRNAseq (CCA)
-20
-10
0
3
Position relative to RNaseZ site
PAR-CLIP
80
60
40
20
0
-25
25
75
125
175
PAR-CLIP (CCA)
160
140
120
100

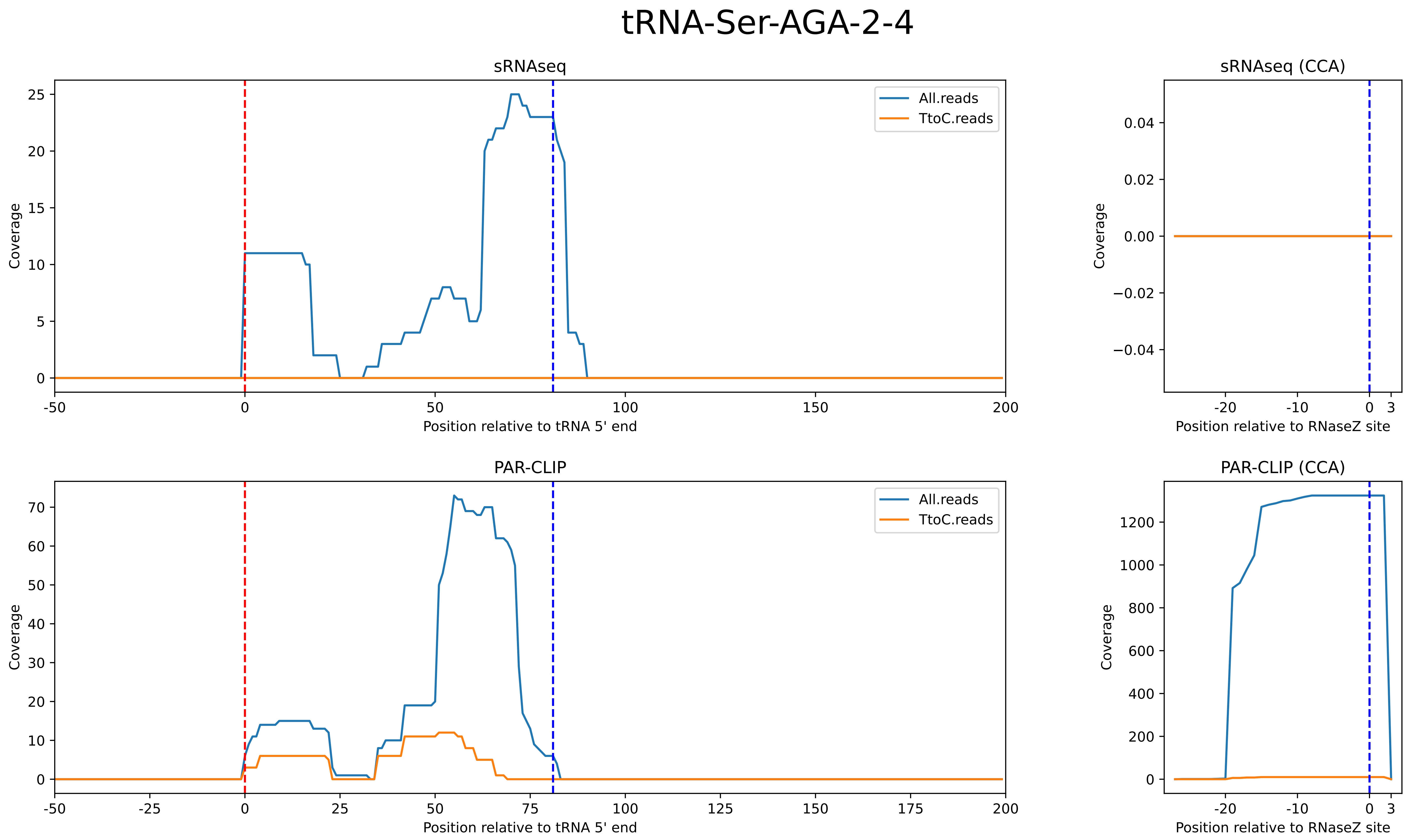

tRNA-Ser-AGA-2-4
sRNAseq
All.reads
TtoC.reads
Coverage
Position relative to tRNA 5' end
sRNAseq (CCA)
Position relative to RNaseZ site
PAR-CLIP
PAR-CLIP (CCA)

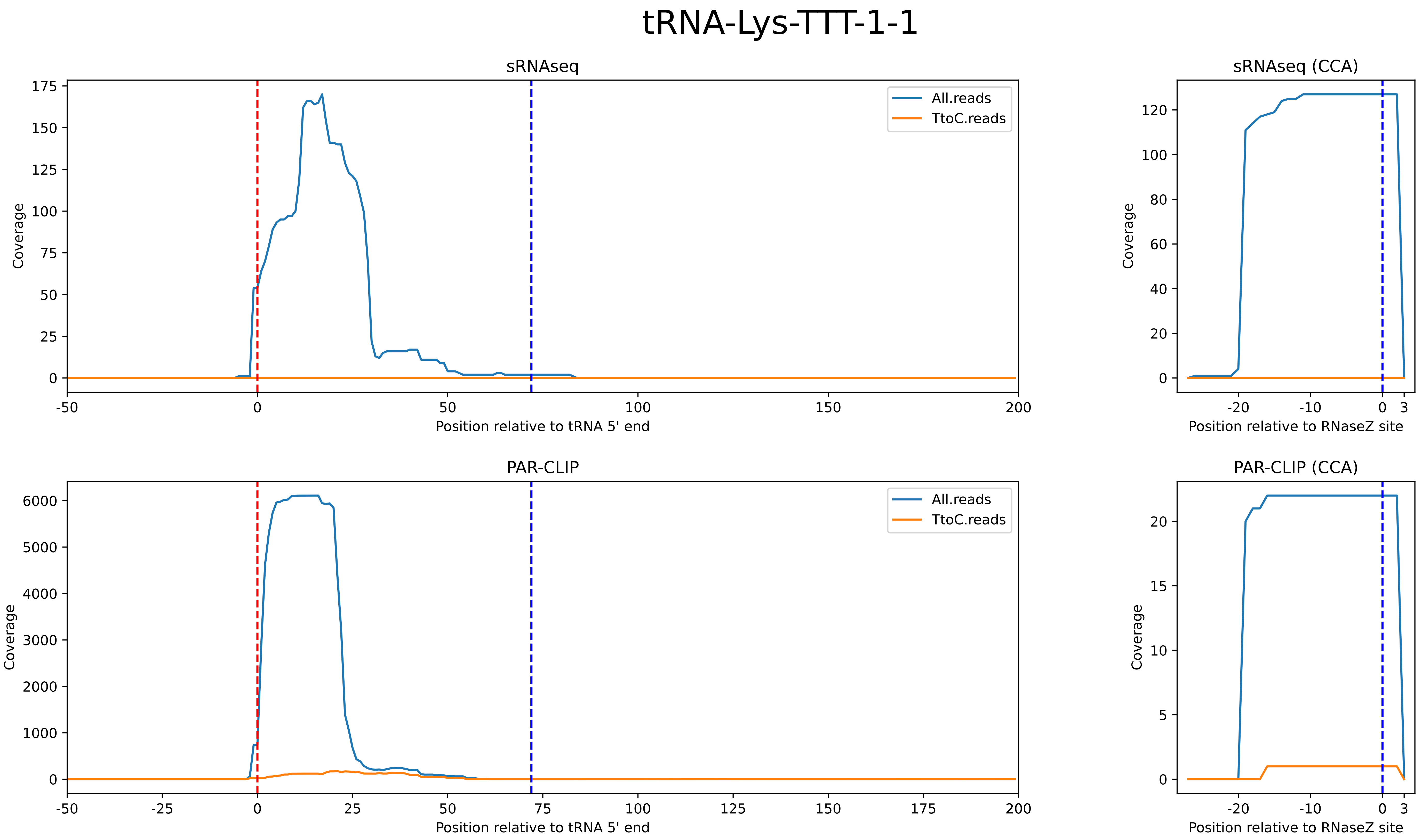
tRNA-Lys-TTT-1-1
sRNAseq
sRNAseq (CCA)
PAR-CLIP
PAR-CLIP (CCA)
All.reads
TtoC.reads
Coverage
Position relative to tRNA 5' end
Position relative to RNaseZ site

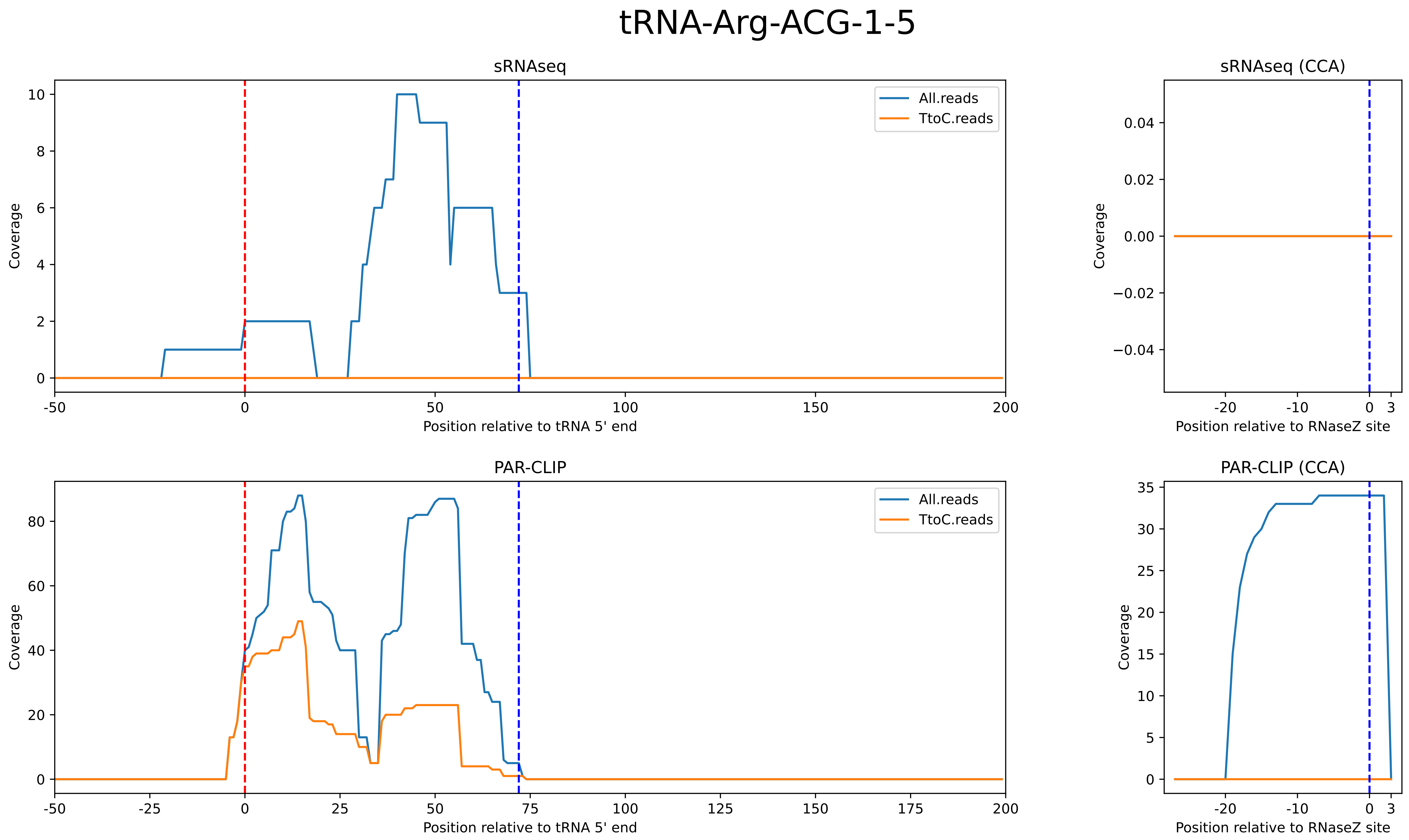

tRNA-Arg-ACG-1-5
sRNAseq
sRNAseq (CCA)
PAR-CLIP
PAR-CLIP (CCA)
All.reads
TtoC.reads
Coverage
Position relative to tRNA 5' end
Position relative to RNaseZ site

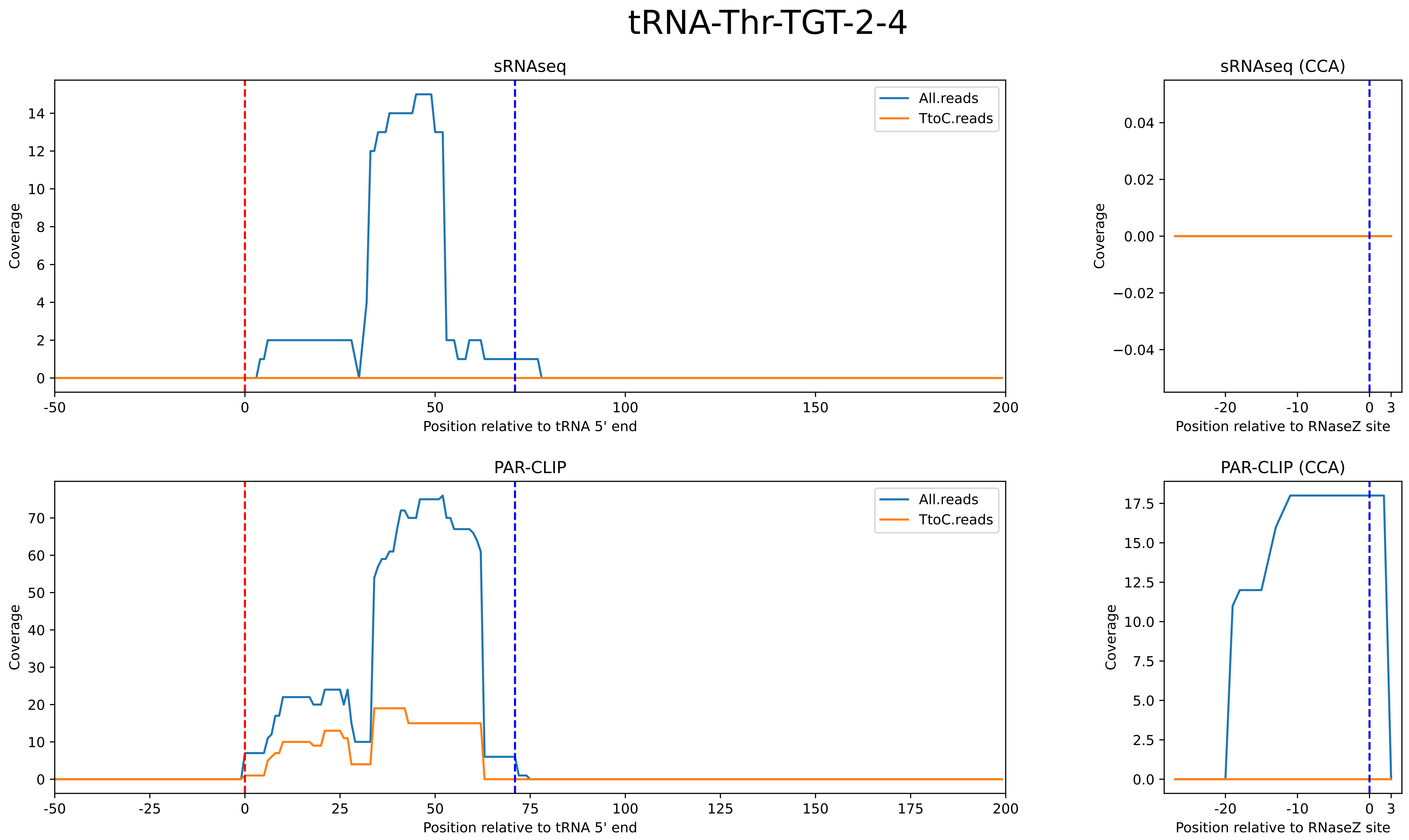
tRNA-Thr-TGT-2-4
sRNAseq
All.reads
TtoC.reads
Coverage
Position relative to tRNA 5' end
sRNAseq (CCA)
Position relative to RNaseZ site
PAR-CLIP
PAR-CLIP (CCA)

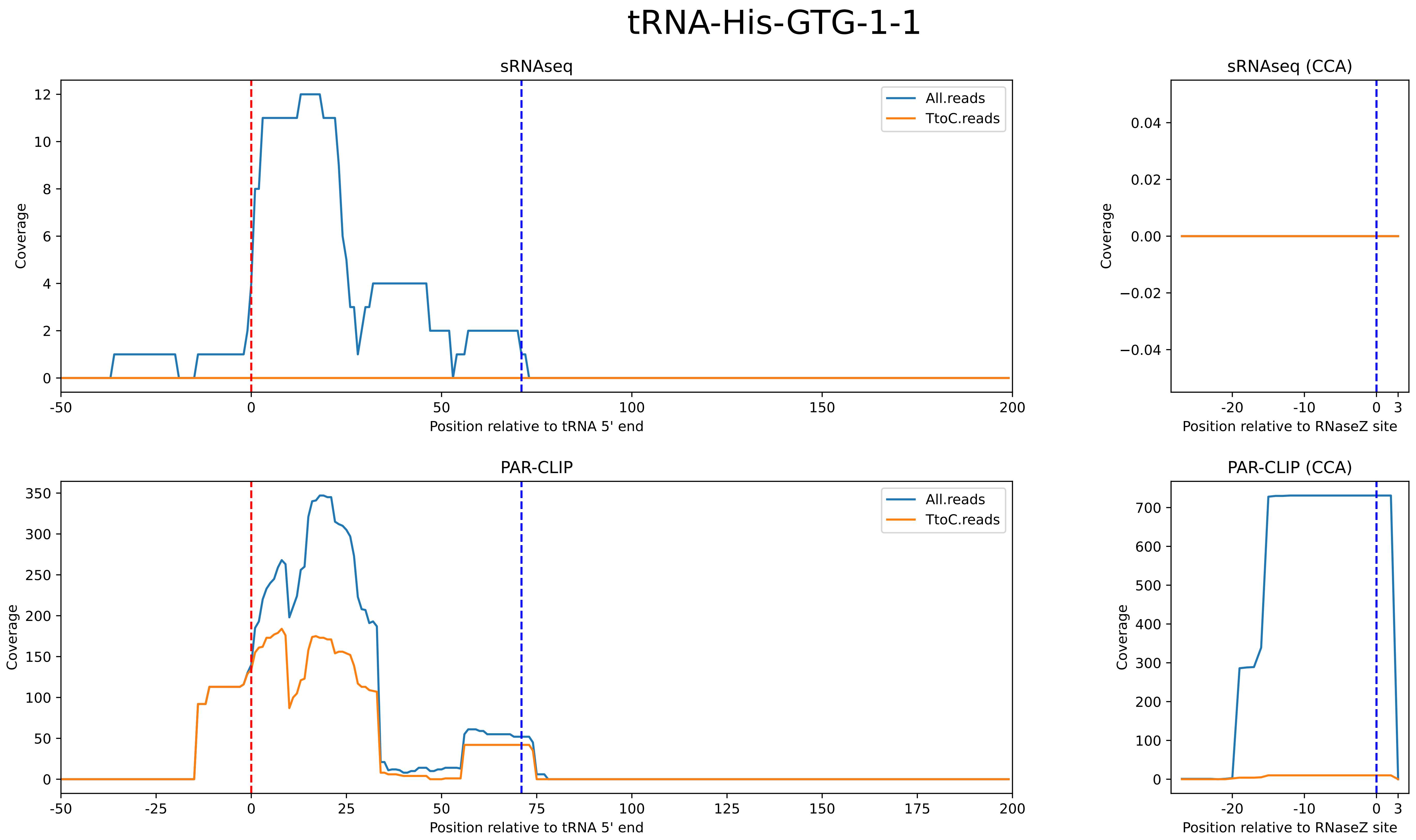

tRNA-His-GTG-1-1
sRNAseq
sRNAseq (CCA)
PAR-CLIP
PAR-CLIP (CCA)
All.reads
TtoC.reads
Coverage
Position relative to tRNA 5' end
Position relative to RNaseZ site

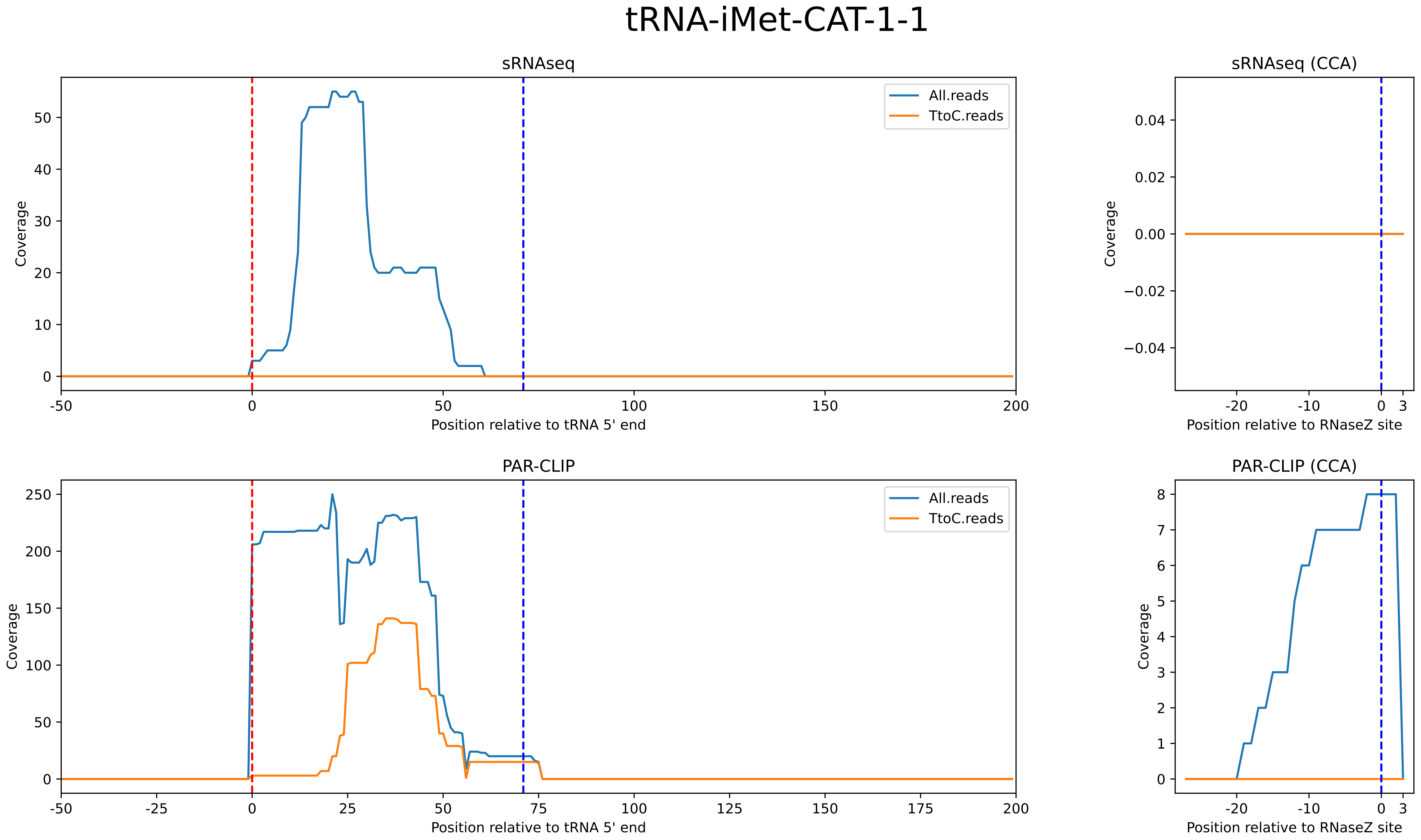

tRNA-iMet-CAT-1-1
sRNAseq
All.reads
TtoC.reads
Coverage
Position relative to tRNA 5' end
sRNAseq (CCA)
Position relative to RNaseZ site
PAR-CLIP
PAR-CLIP (CCA)

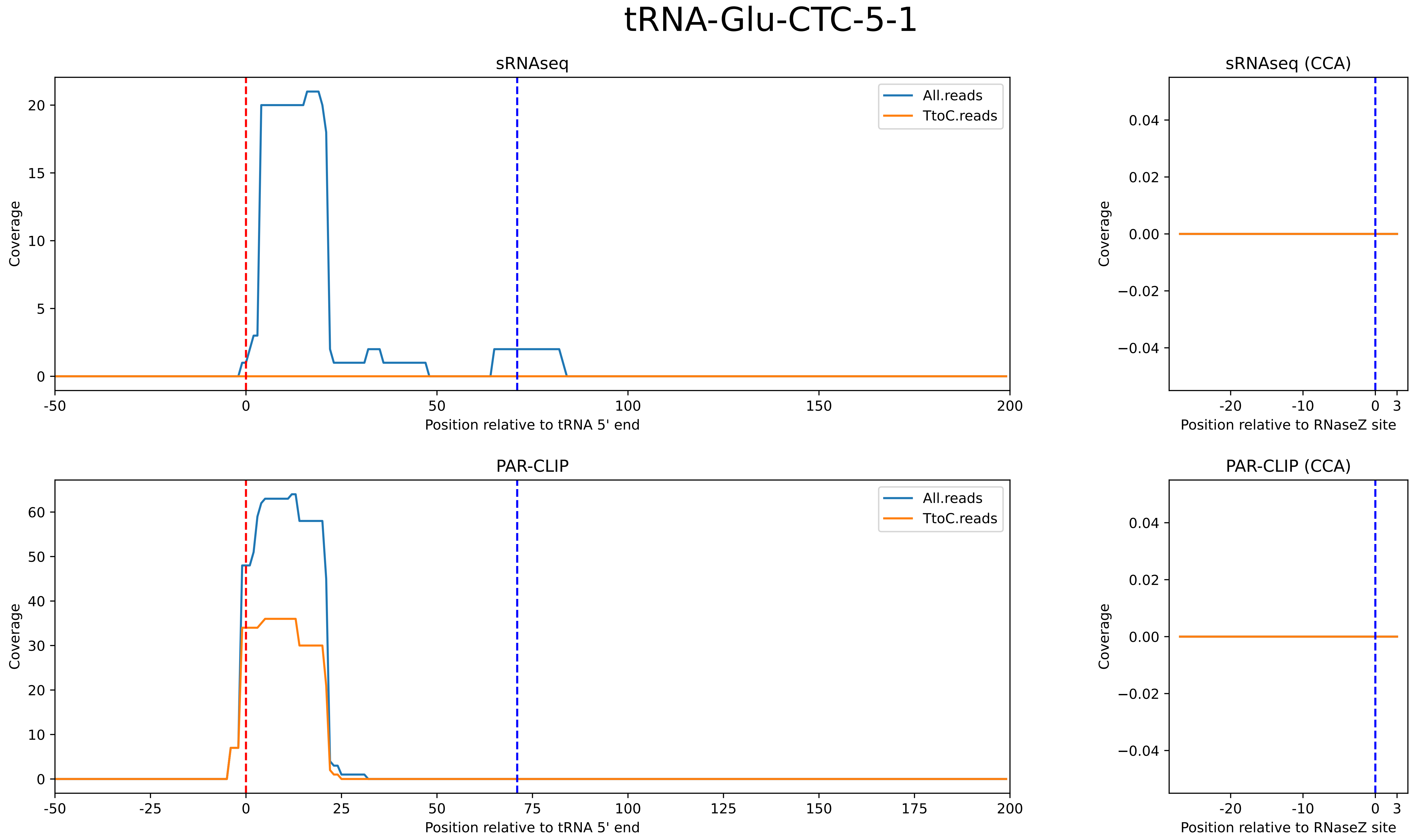

tRNA-Glu-CTC-5-1
sRNAseq
All.reads
TtoC.reads
Coverage
Position relative to tRNA 5' end
sRNAseq (CCA)
Position relative to RNaseZ site
PAR-CLIP
PAR-CLIP (CCA)

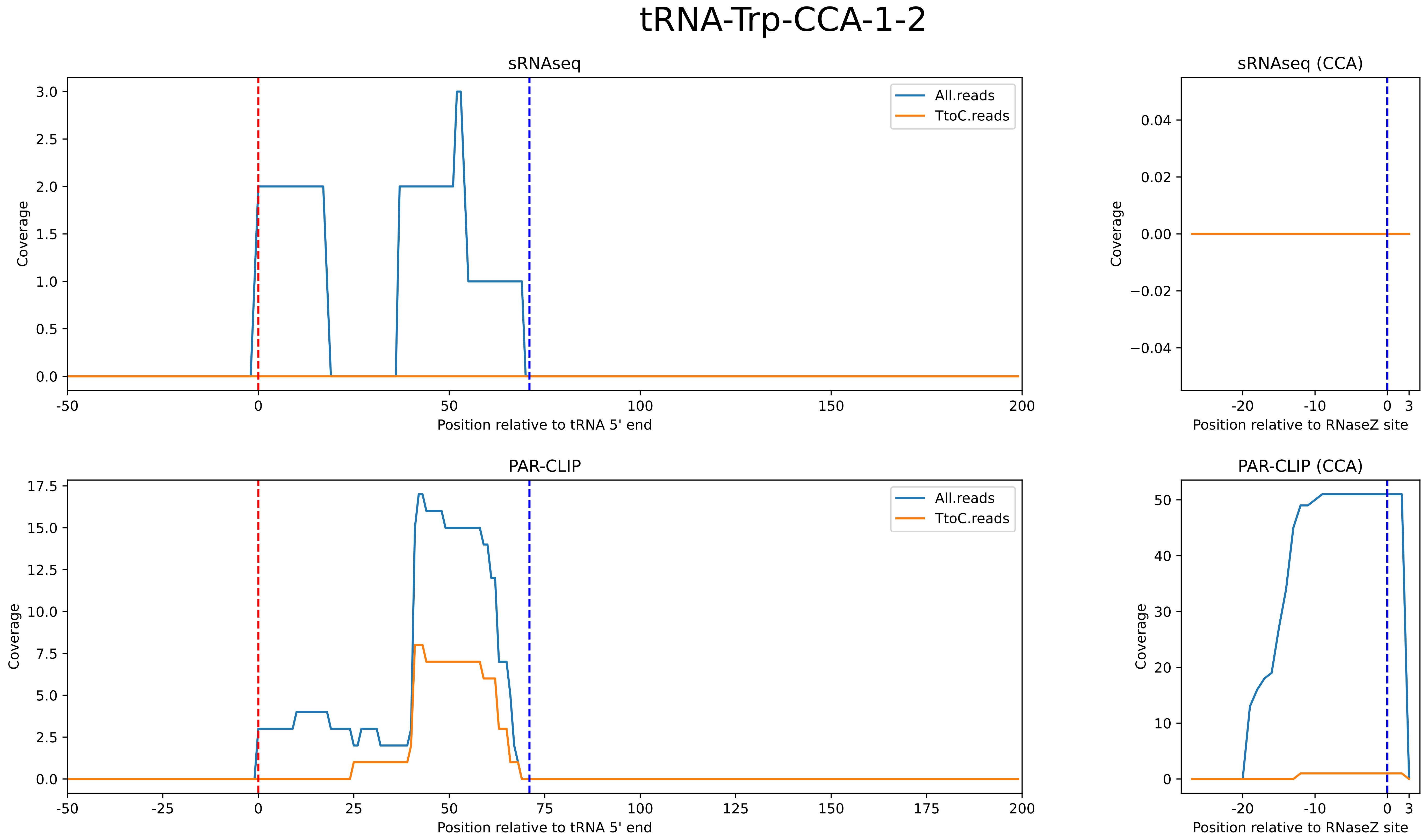
tRNA-Trp-CCA-1-2
sRNAseq
All.reads
TtoC.reads
Coverage
Position relative to tRNA 5' end
sRNAseq (CCA)
Position relative to RNaseZ site
PAR-CLIP
PAR-CLIP (CCA)

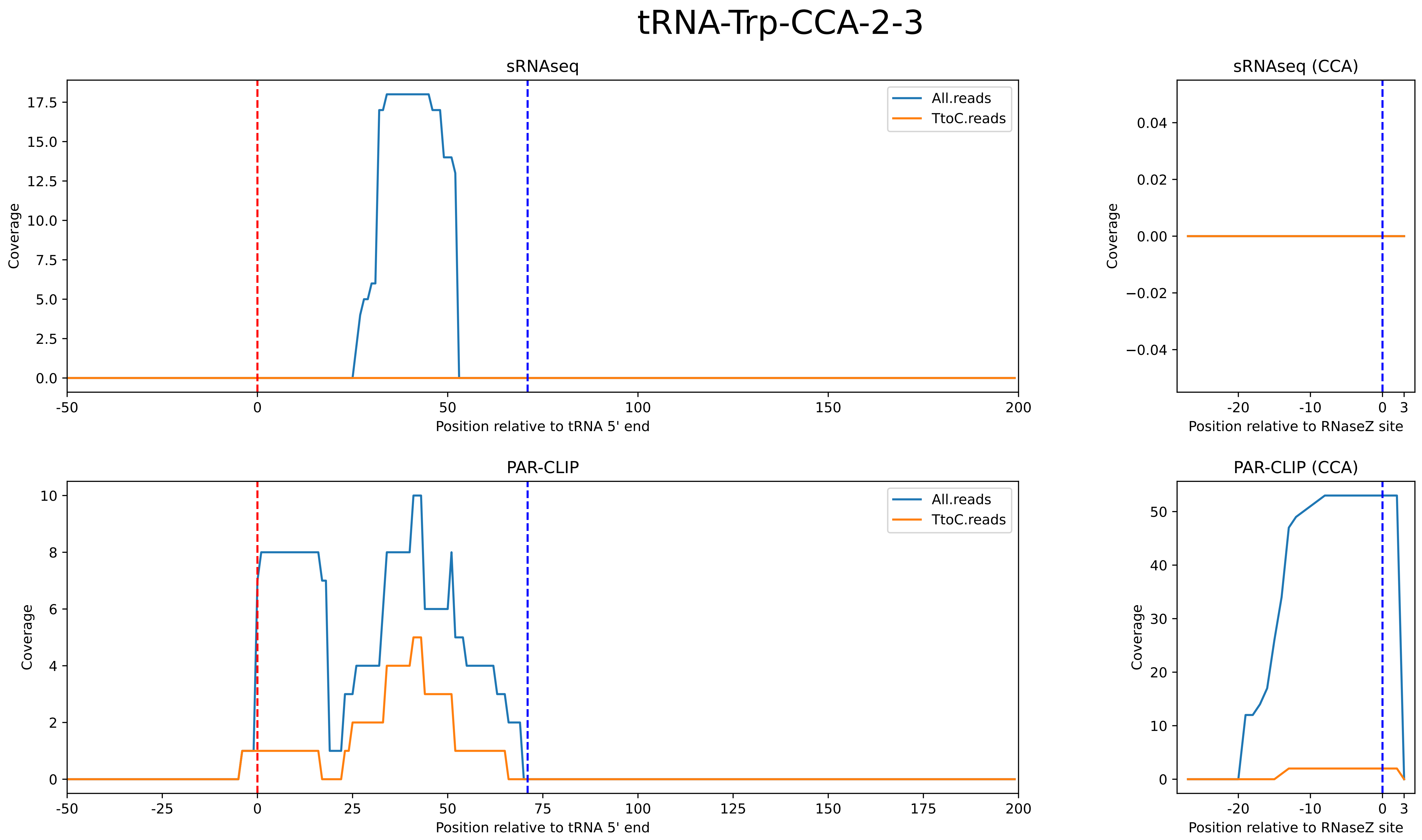

tRNA-Trp-CCA-2-3
sRNAseq
All.reads
TtoC.reads
Coverage
Position relative to tRNA 5' end
sRNAseq (CCA)
Position relative to RNaseZ site
PAR-CLIP
PAR-CLIP (CCA)

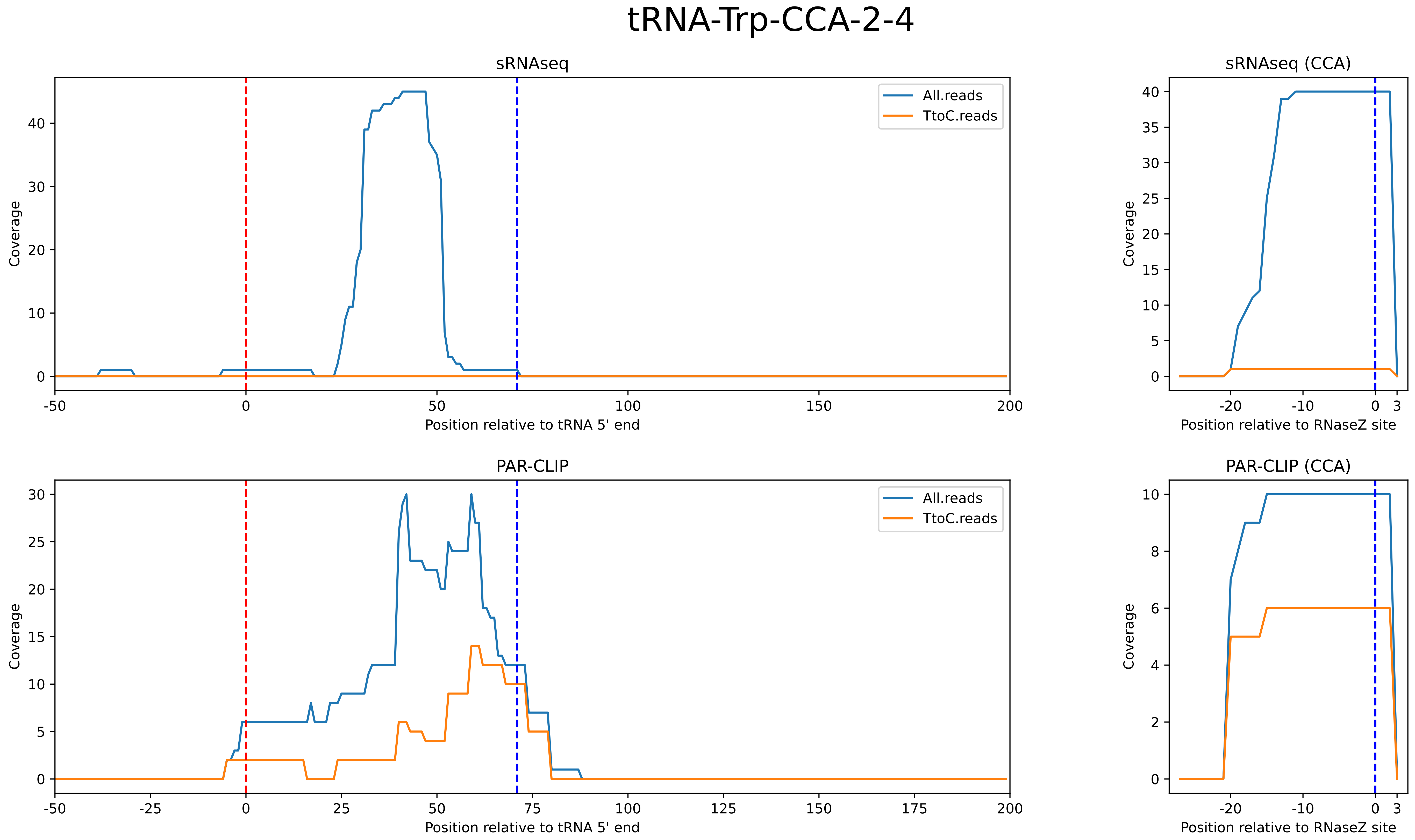
tRNA-Trp-CCA-2-4
sRNAseq
sRNAseq (CCA)
PAR-CLIP
PAR-CLIP (CCA)
All.reads
TtoC.reads
Coverage
Position relative to tRNA 5' end
Position relative to RNaseZ site

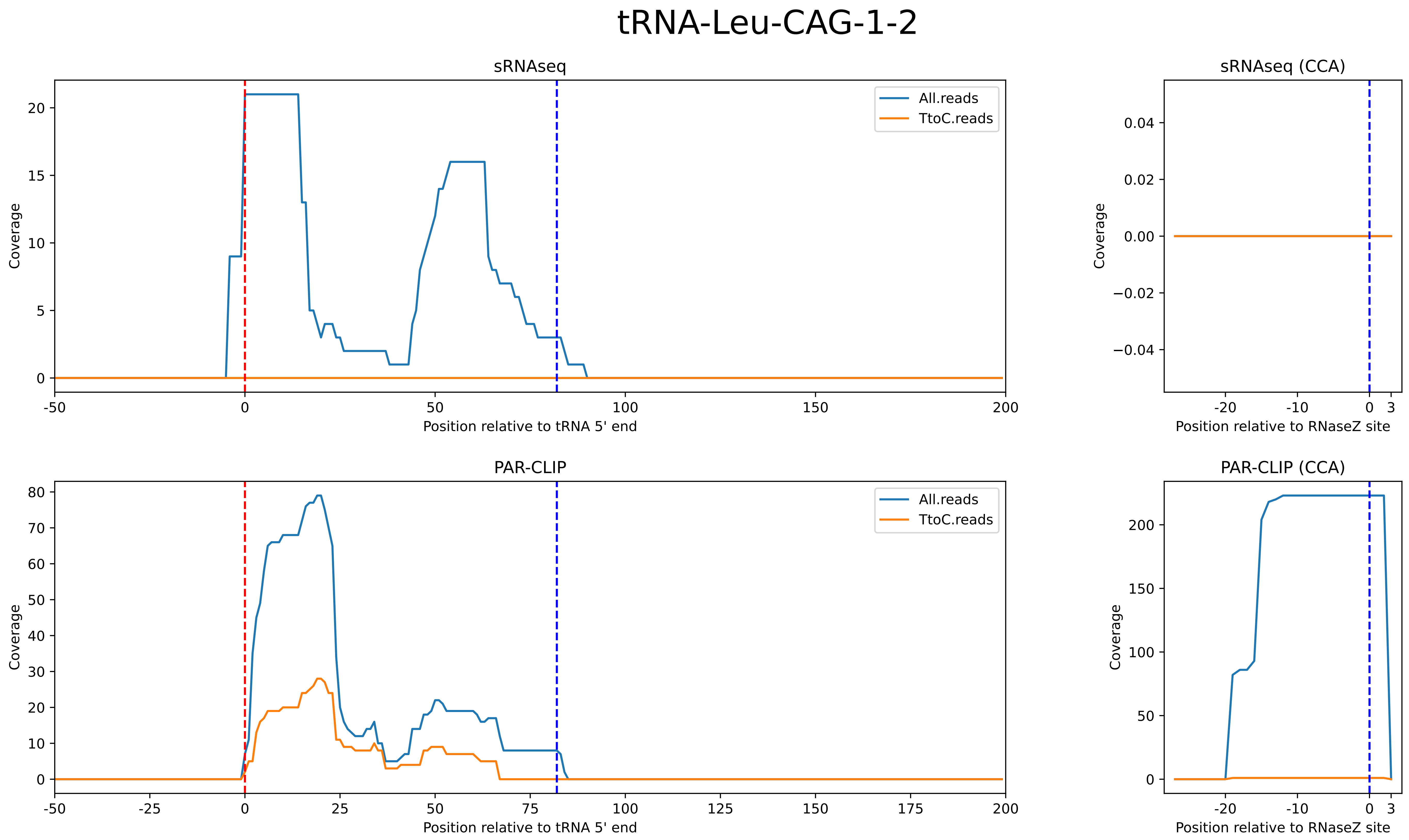

tRNA-Leu-CAG-1-2
sRNAseq
sRNAseq (CCA)
PAR-CLIP
PAR-CLIP (CCA)
All.reads
TtoC.reads
Coverage
Position relative to tRNA 5' end
Position relative to RNaseZ site

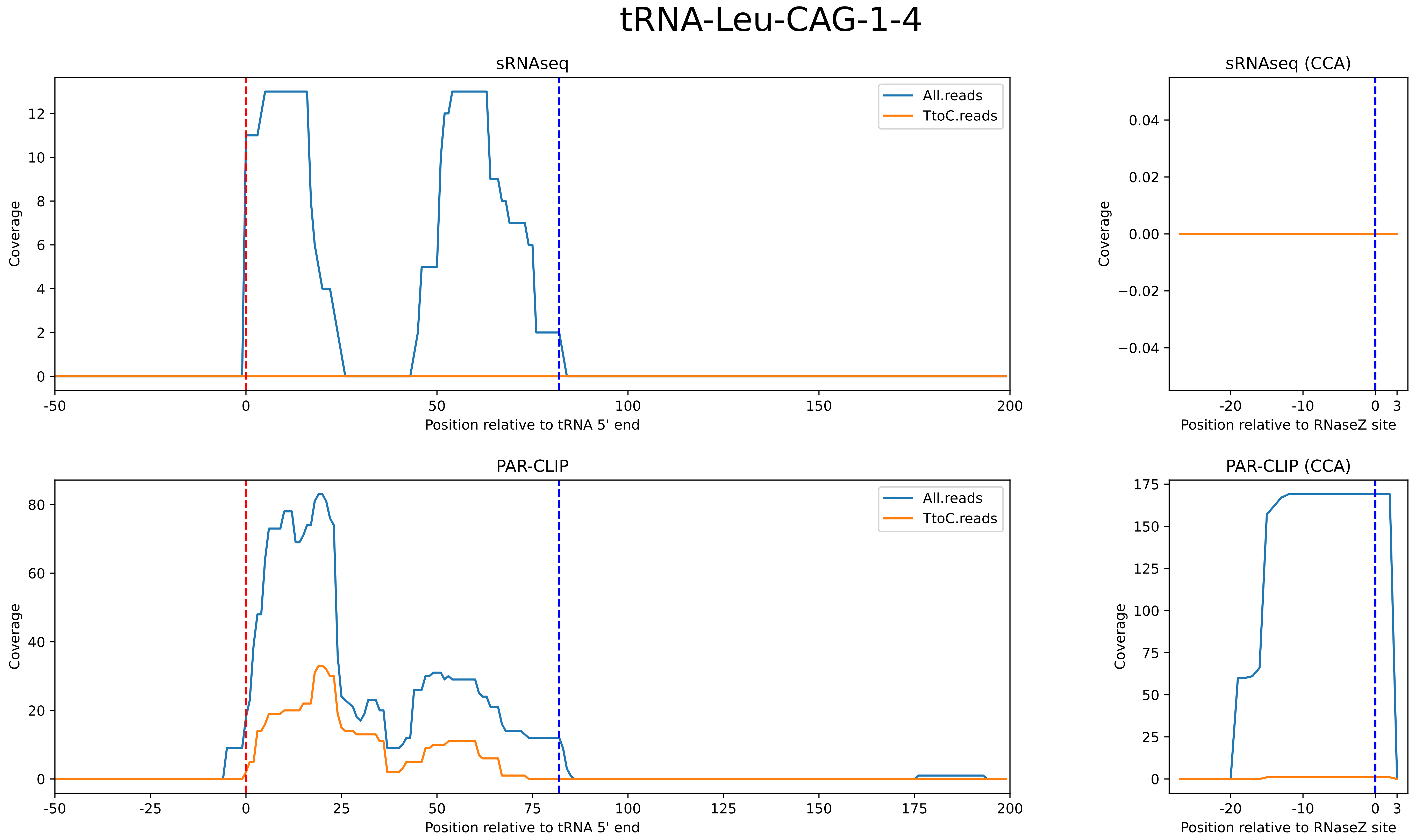
tRNA-Leu-CAG-1-4
sRNAseq
All.reads
TtoC.reads
Coverage
Position relative to tRNA 5' end
sRNAseq (CCA)
Position relative to RNaseZ site
PAR-CLIP
PAR-CLIP (CCA)

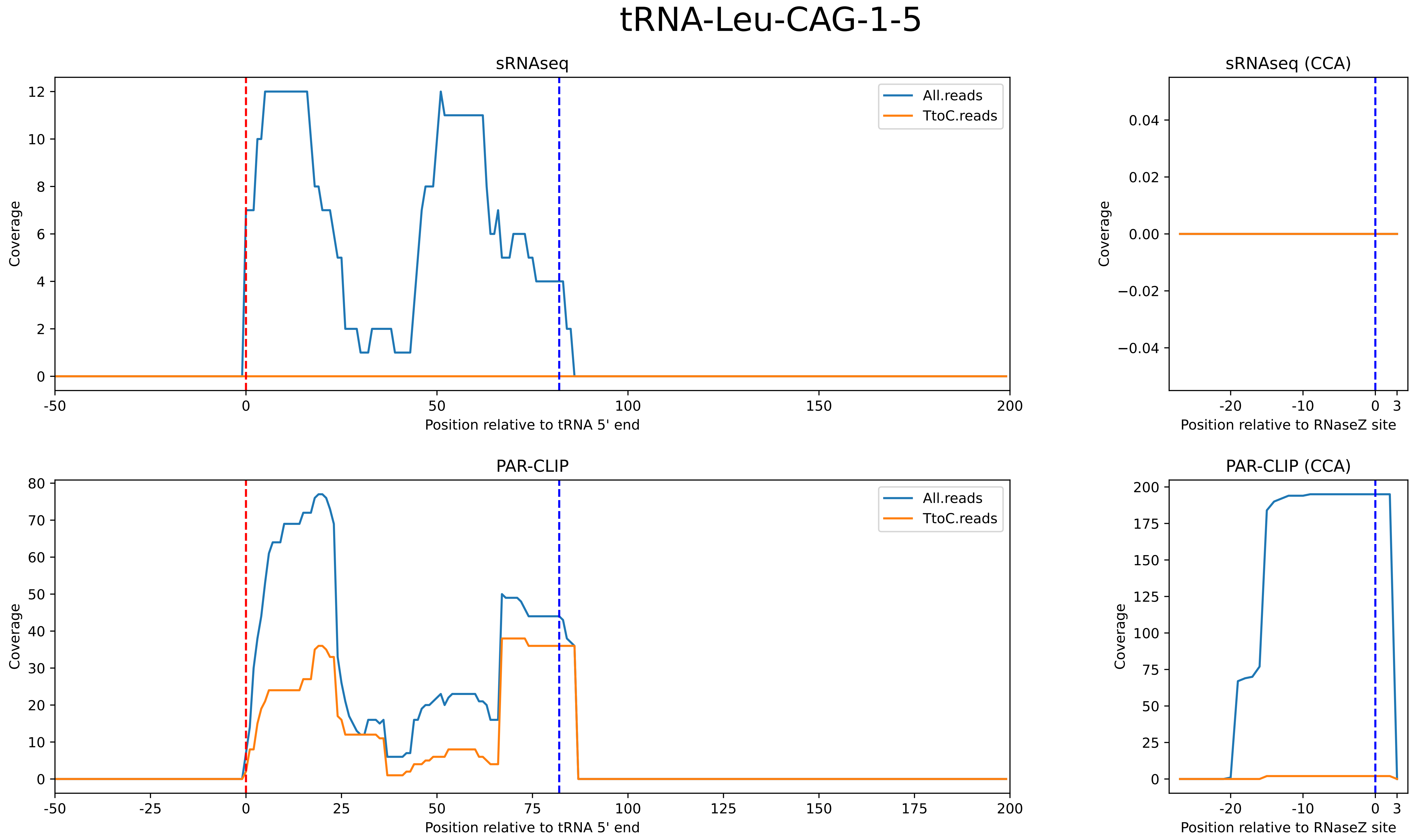
tRNA-Leu-CAG-1-5
sRNAseq
sRNAseq (CCA)
PAR-CLIP
PAR-CLIP (CCA)
All.reads
TtoC.reads
Coverage
Position relative to tRNA 5' end
Position relative to RNaseZ site

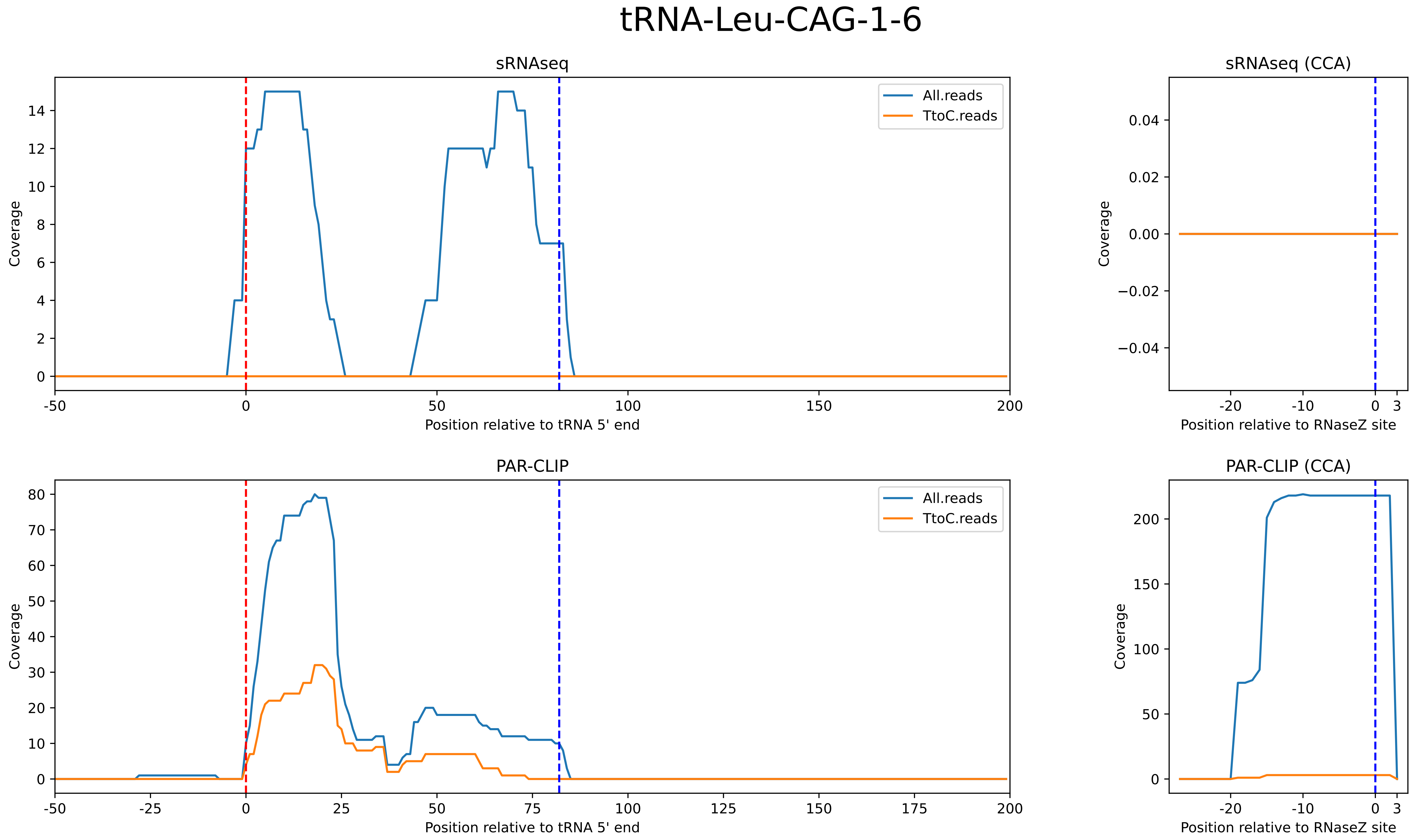

tRNA-Leu-CAG-1-6
sRNAseq
All.reads
TtoC.reads
Coverage
Position relative to tRNA 5' end
sRNAseq (CCA)
Position relative to RNaseZ site
PAR-CLIP
PAR-CLIP (CCA)

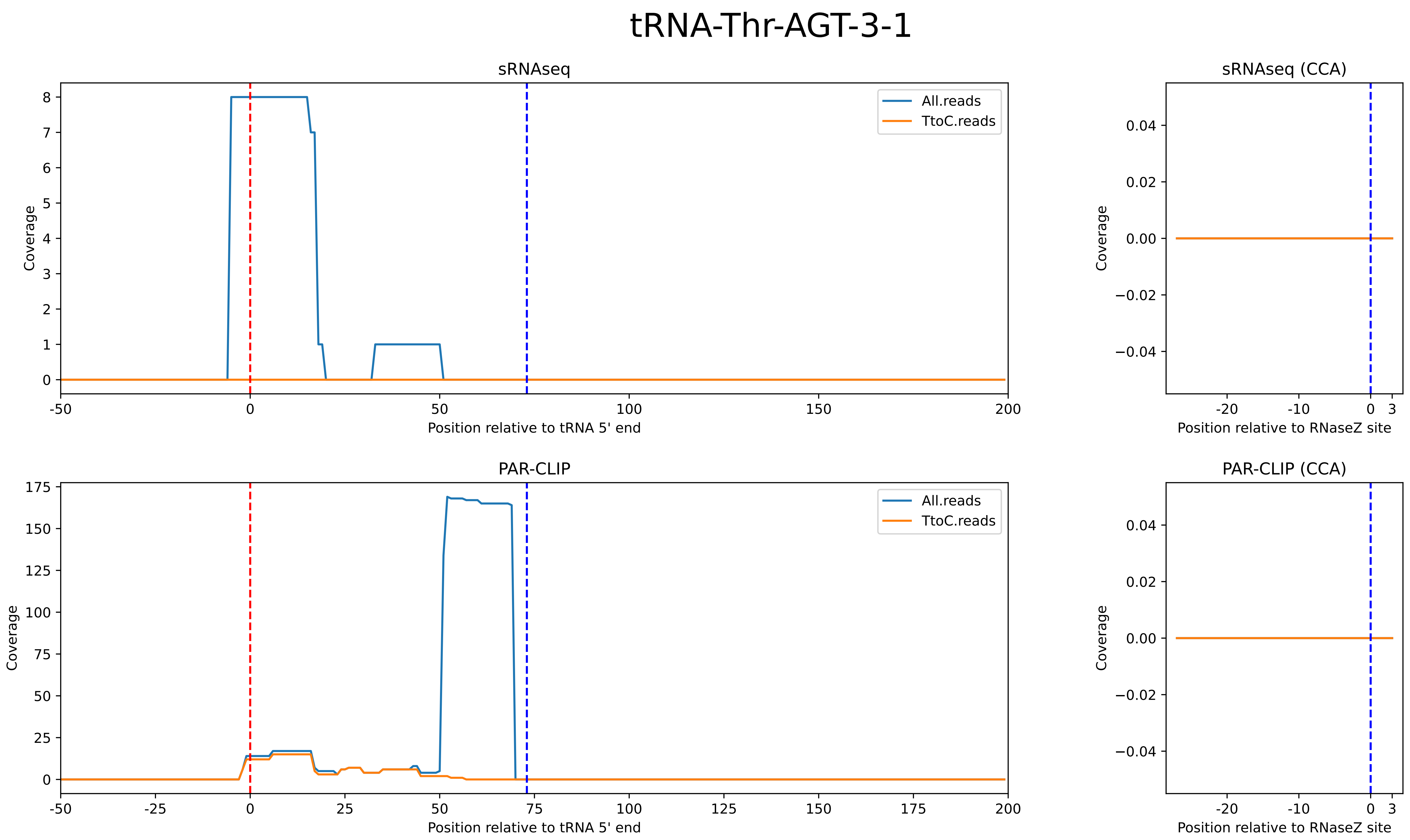
tRNA-Thr-AGT-3-1
sRNAseq
sRNAseq (CCA)
PAR-CLIP
PAR-CLIP (CCA)
All.reads
TtoC.reads
Coverage
Position relative to tRNA 5' end
Position relative to RNaseZ site

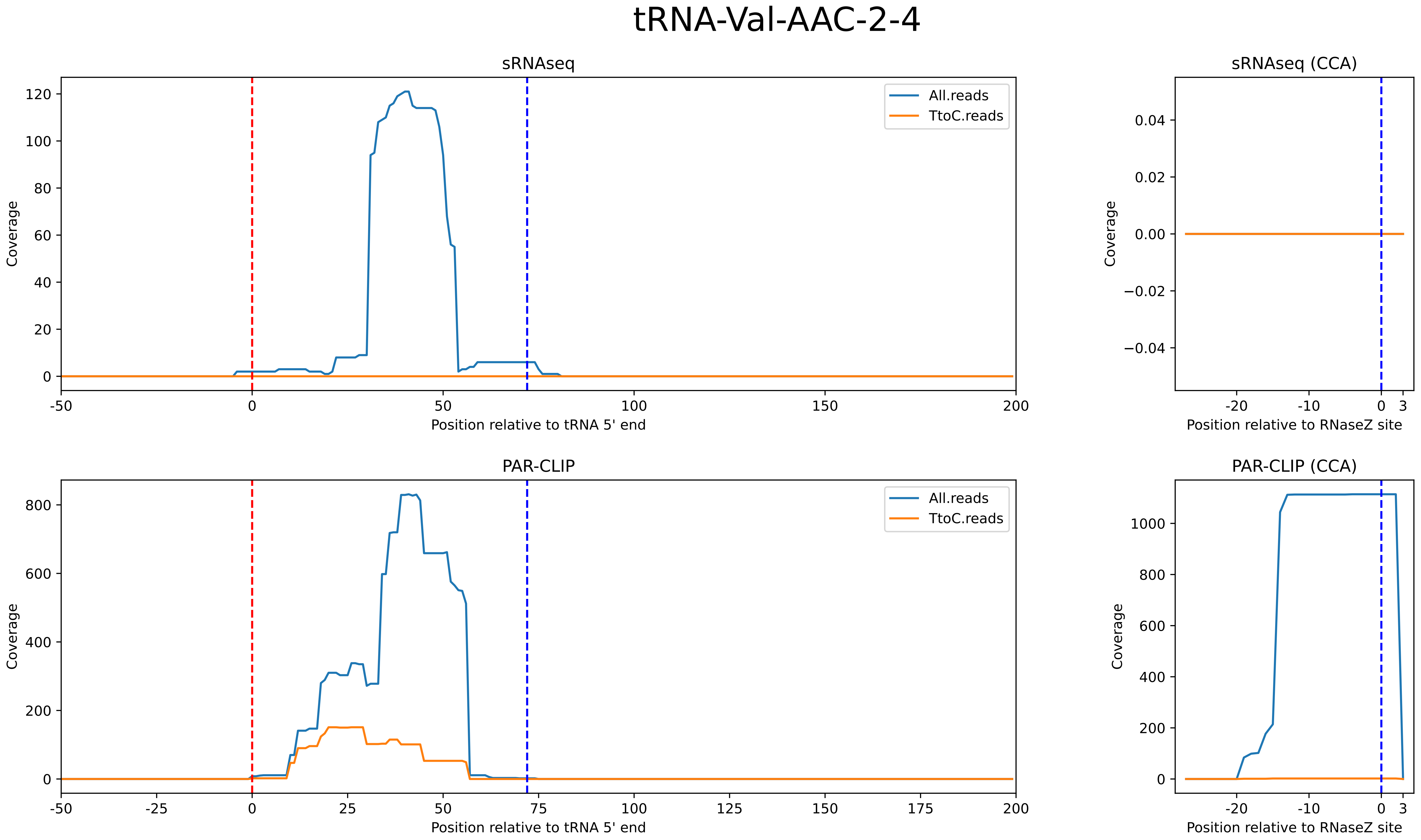

tRNA-Val-AAC-2-4
sRNAseq
All.reads
TtoC.reads
Coverage
Position relative to tRNA 5' end
sRNAseq (CCA)
Position relative to RNaseZ site
PAR-CLIP
PAR-CLIP (CCA)

# tRNA-iMet-CAT-1-5

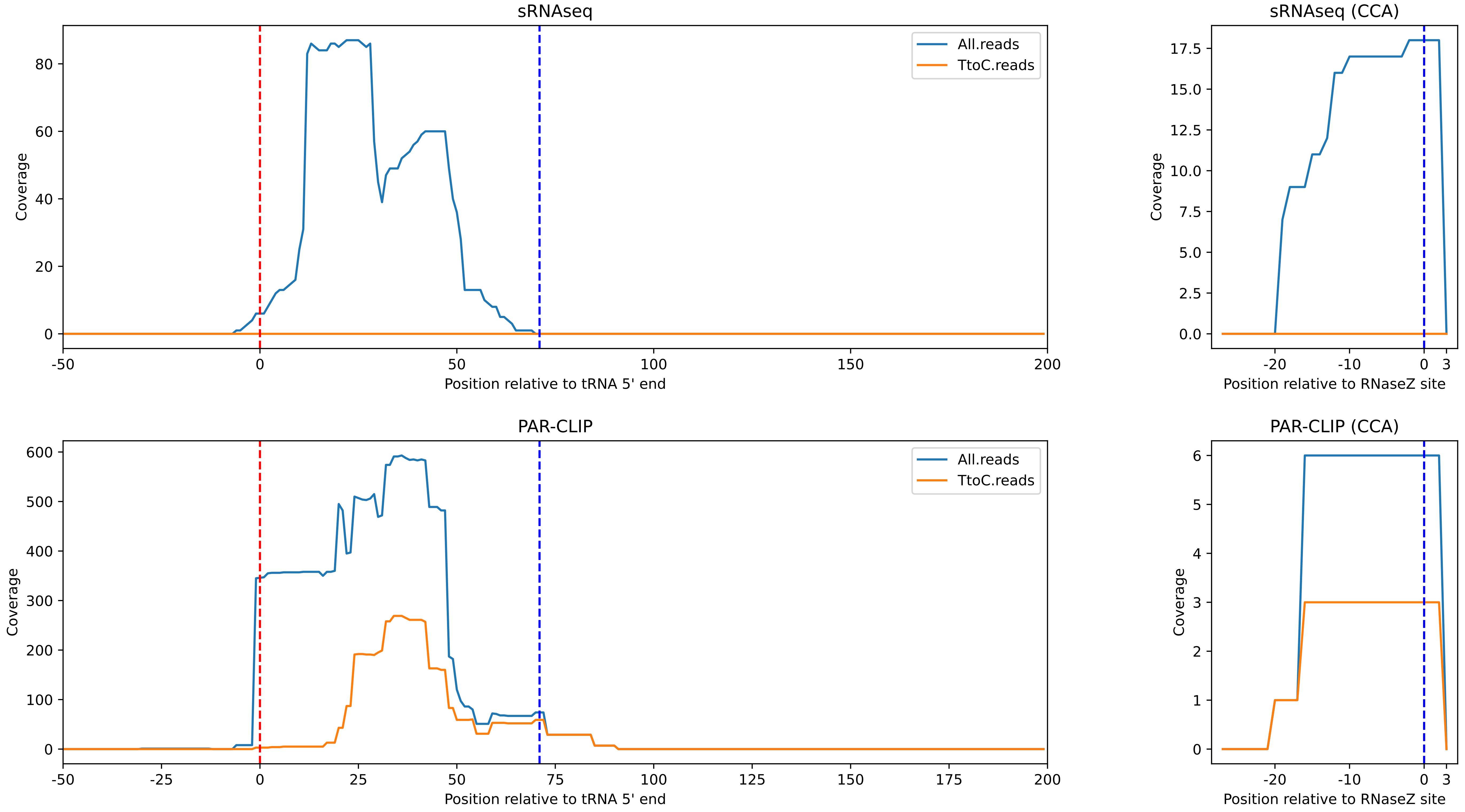

# tRNA-Pro-CGG-2-3

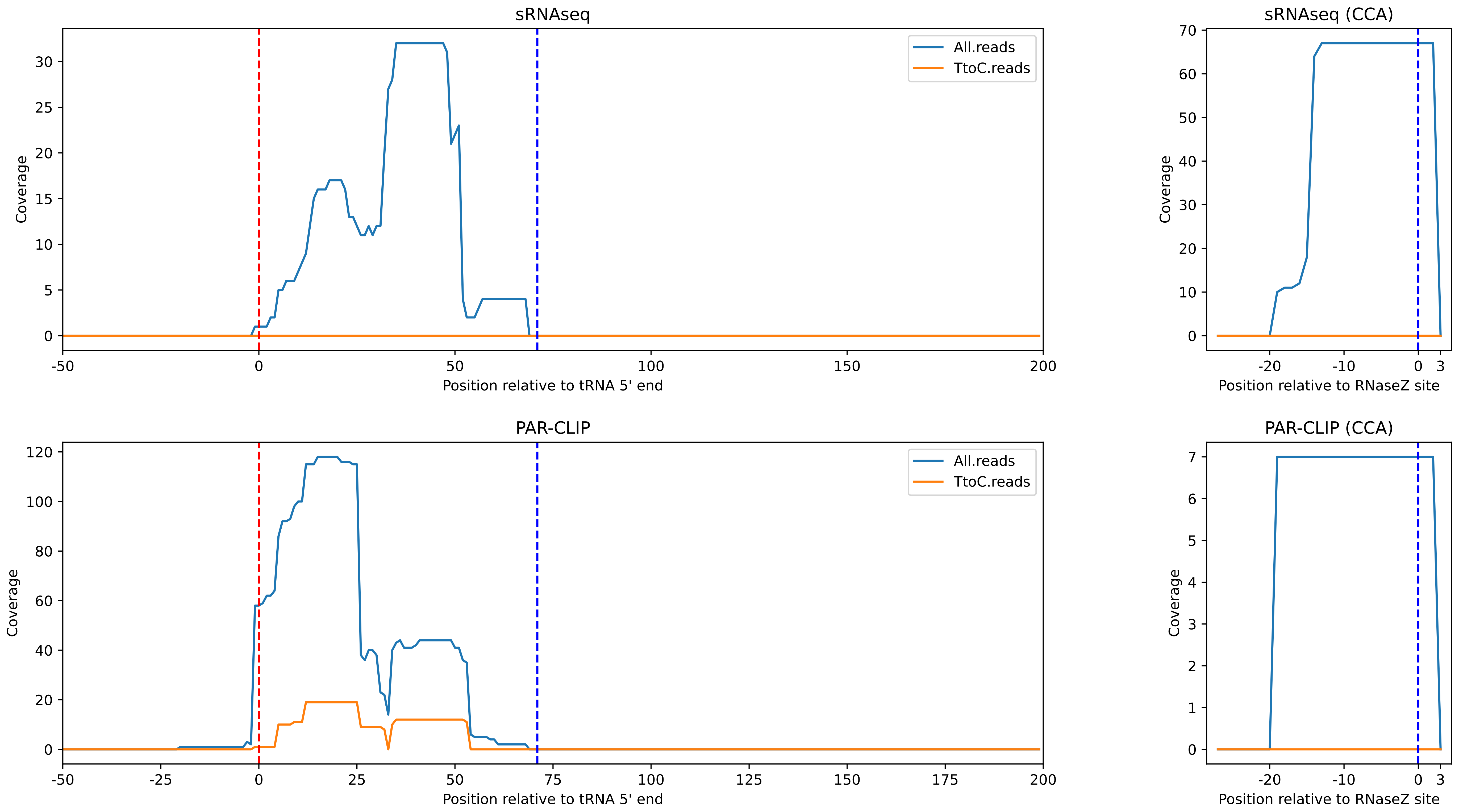

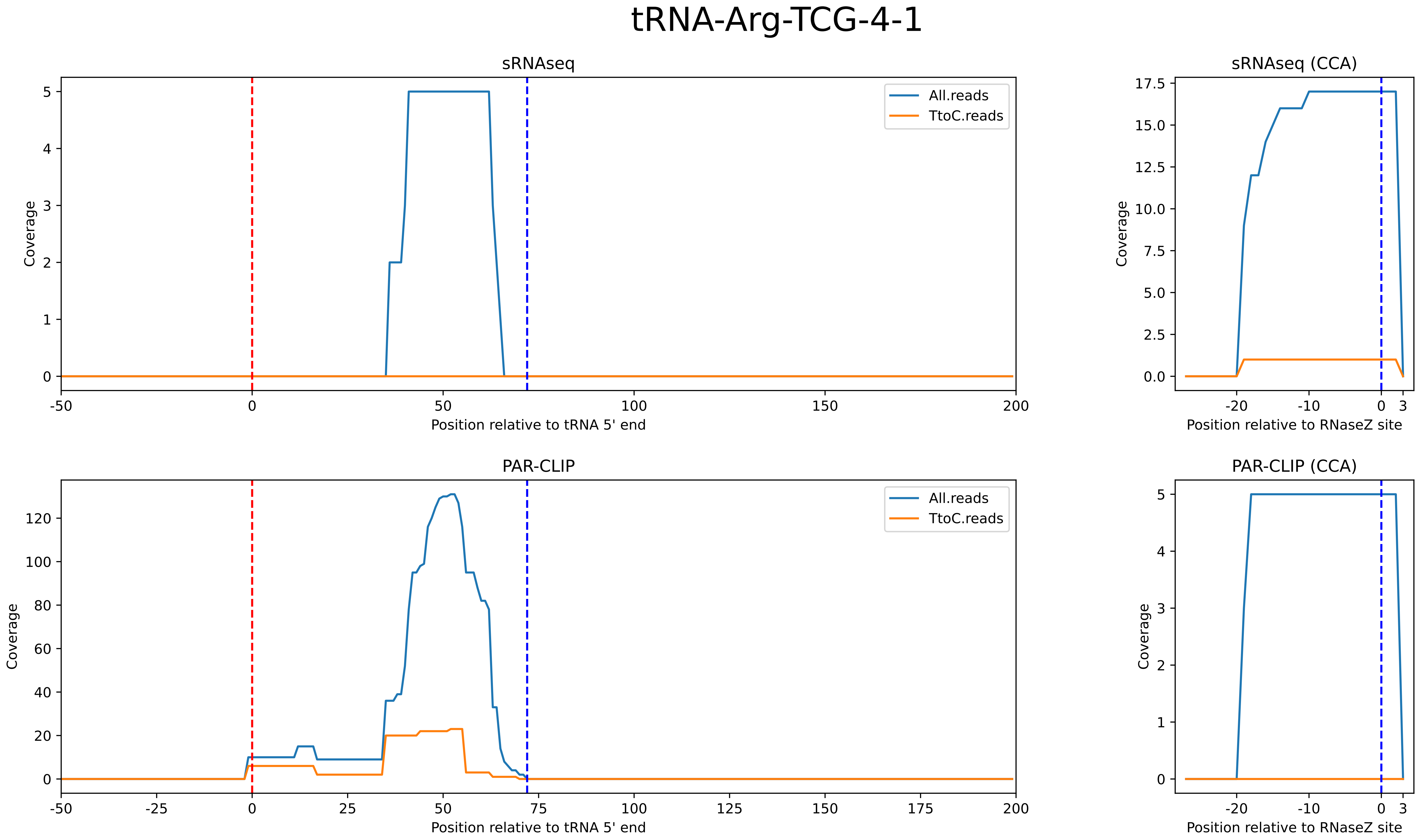
tRNA-Arg-TCG-4-1
sRNAseq
All.reads
TtoC.reads
Coverage
Position relative to tRNA 5' end
sRNAseq (CCA)
Position relative to RNaseZ site
PAR-CLIP
PAR-CLIP (CCA)

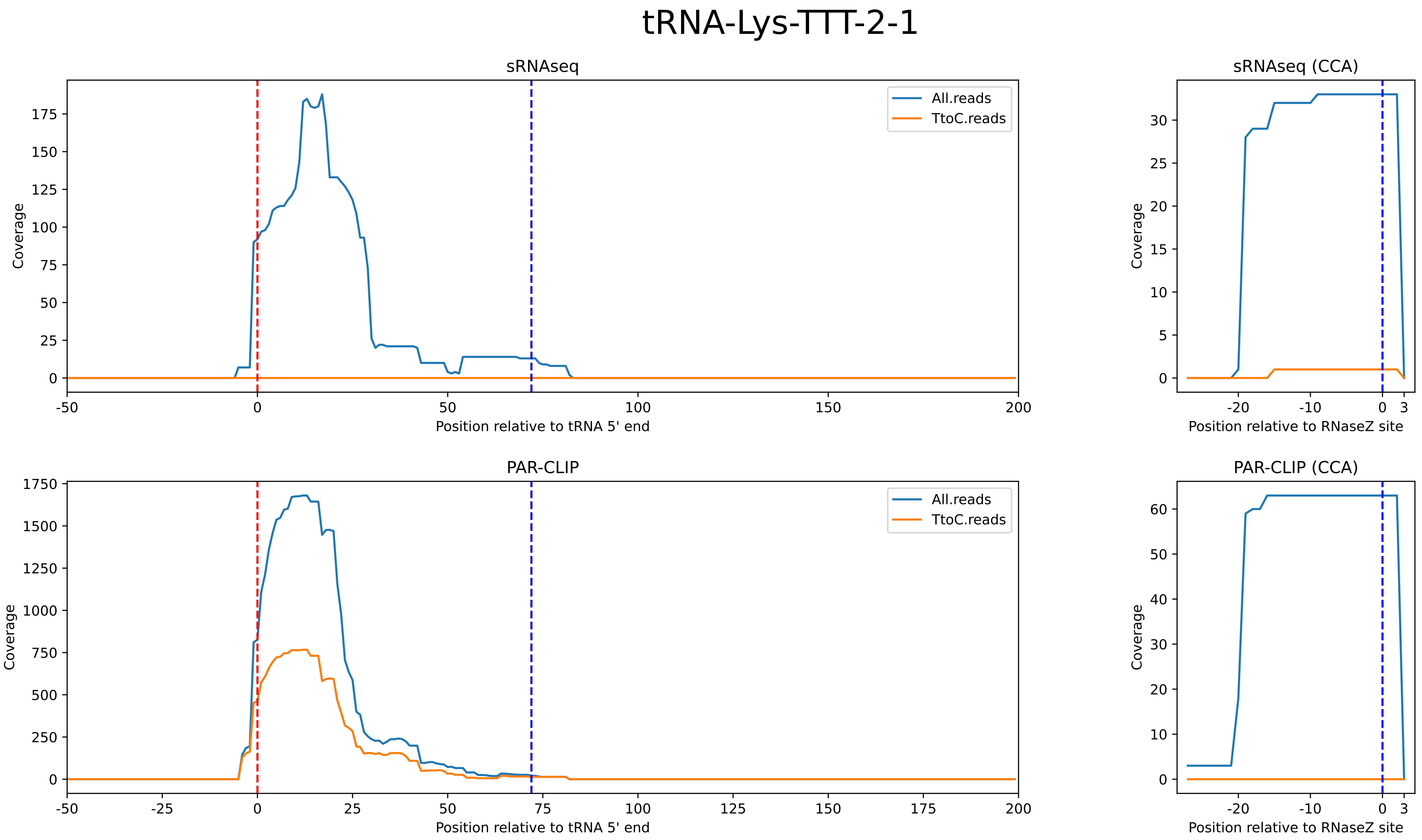
tRNA-Lys-TTT-2-1
sRNAseq
sRNAseq (CCA)
PAR-CLIP
PAR-CLIP (CCA)
All.reads
TtoC.reads
Coverage
Position relative to tRNA 5' end
Position relative to RNaseZ site
-50
-25
0
25
50
75
100
125
150
175
200
-20
-10
3
250
500
750
1000
1250
1500
1750
5
10
15
20
30
40
60

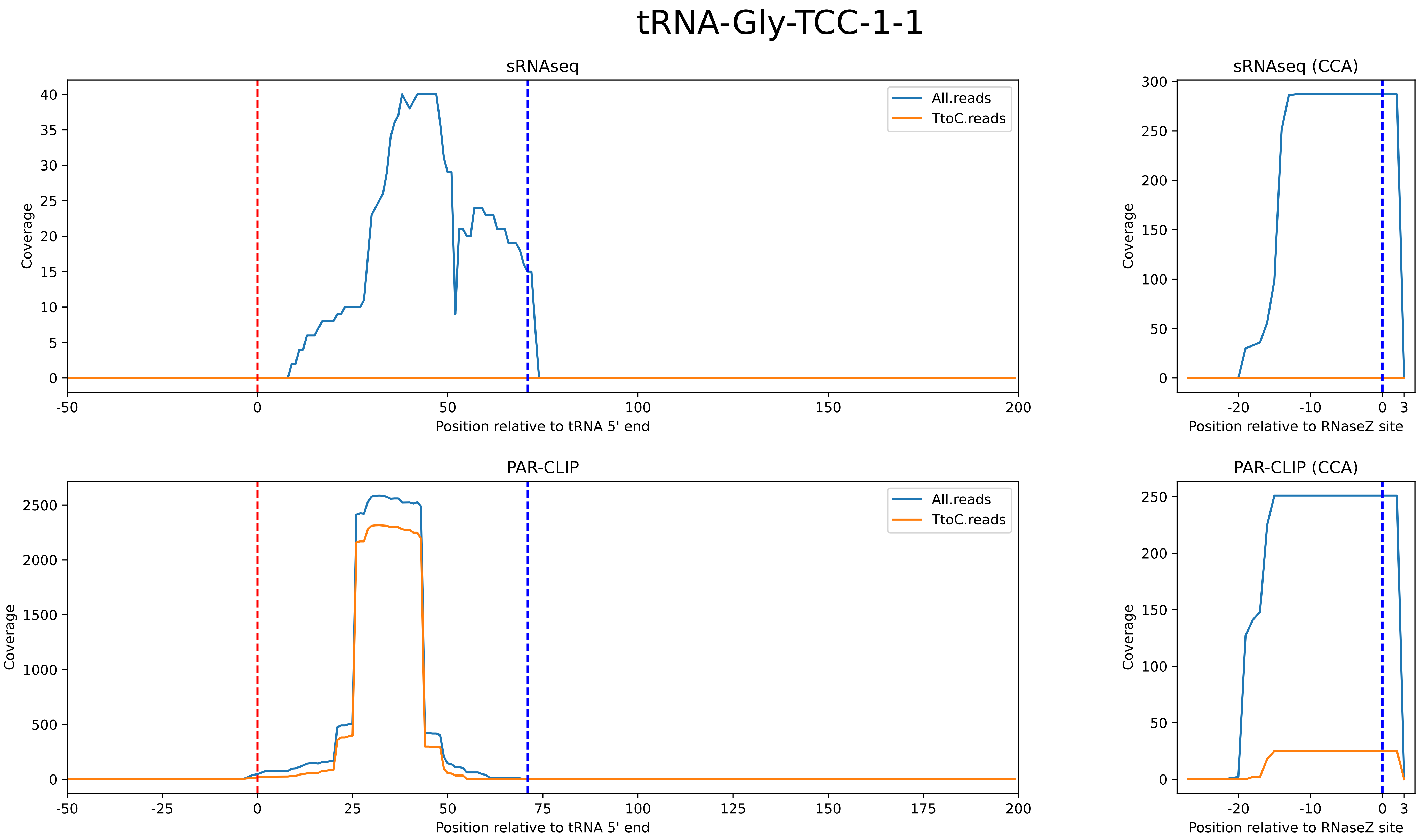

tRNA-Gly-TCC-1-1
sRNAseq
sRNAseq (CCA)
PAR-CLIP
PAR-CLIP (CCA)
All.reads
TtoC.reads
Coverage
Position relative to tRNA 5' end
Position relative to RNaseZ site

# tRNA-Val-CAC-2-1

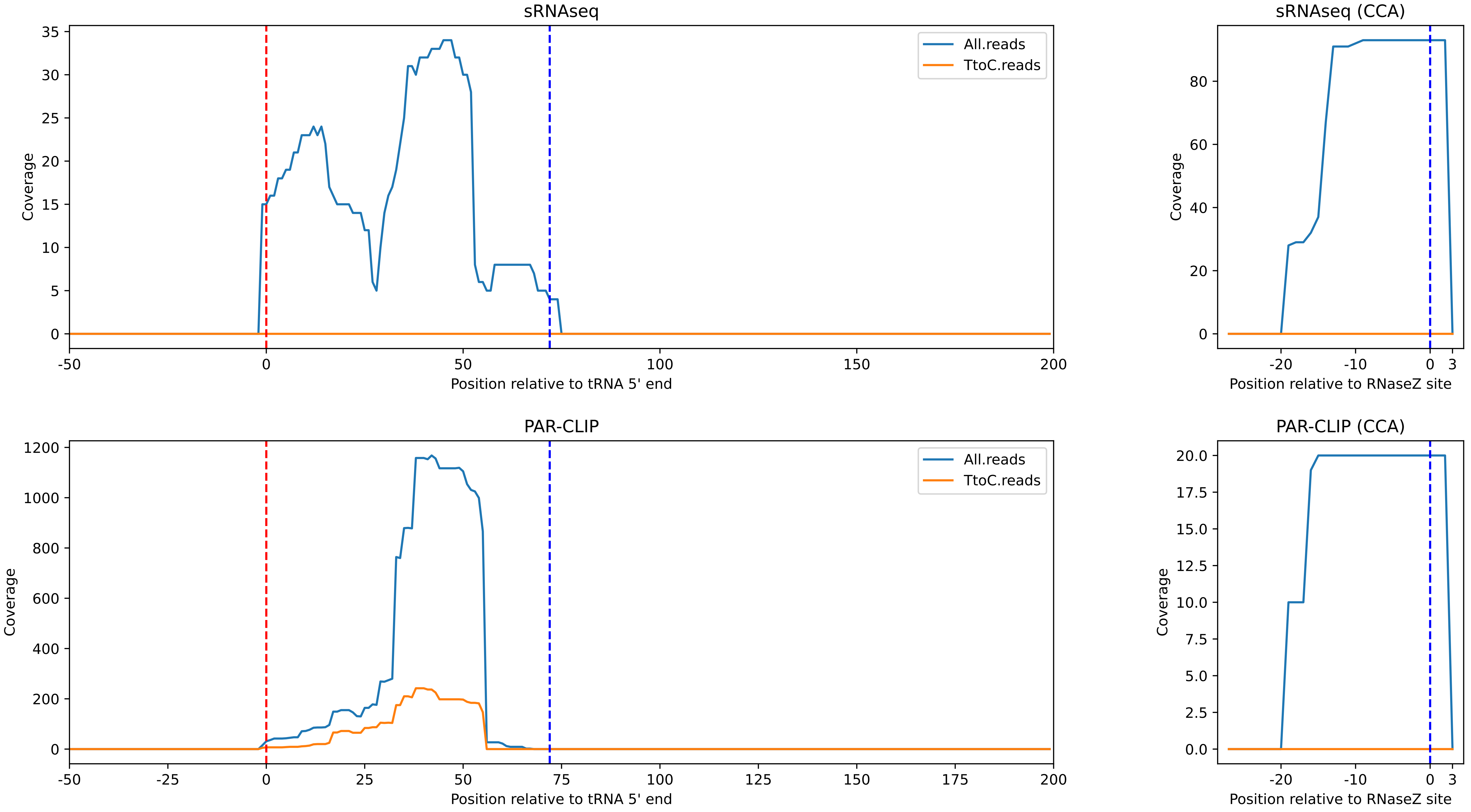

# tRNA-Val-CAC-2-3

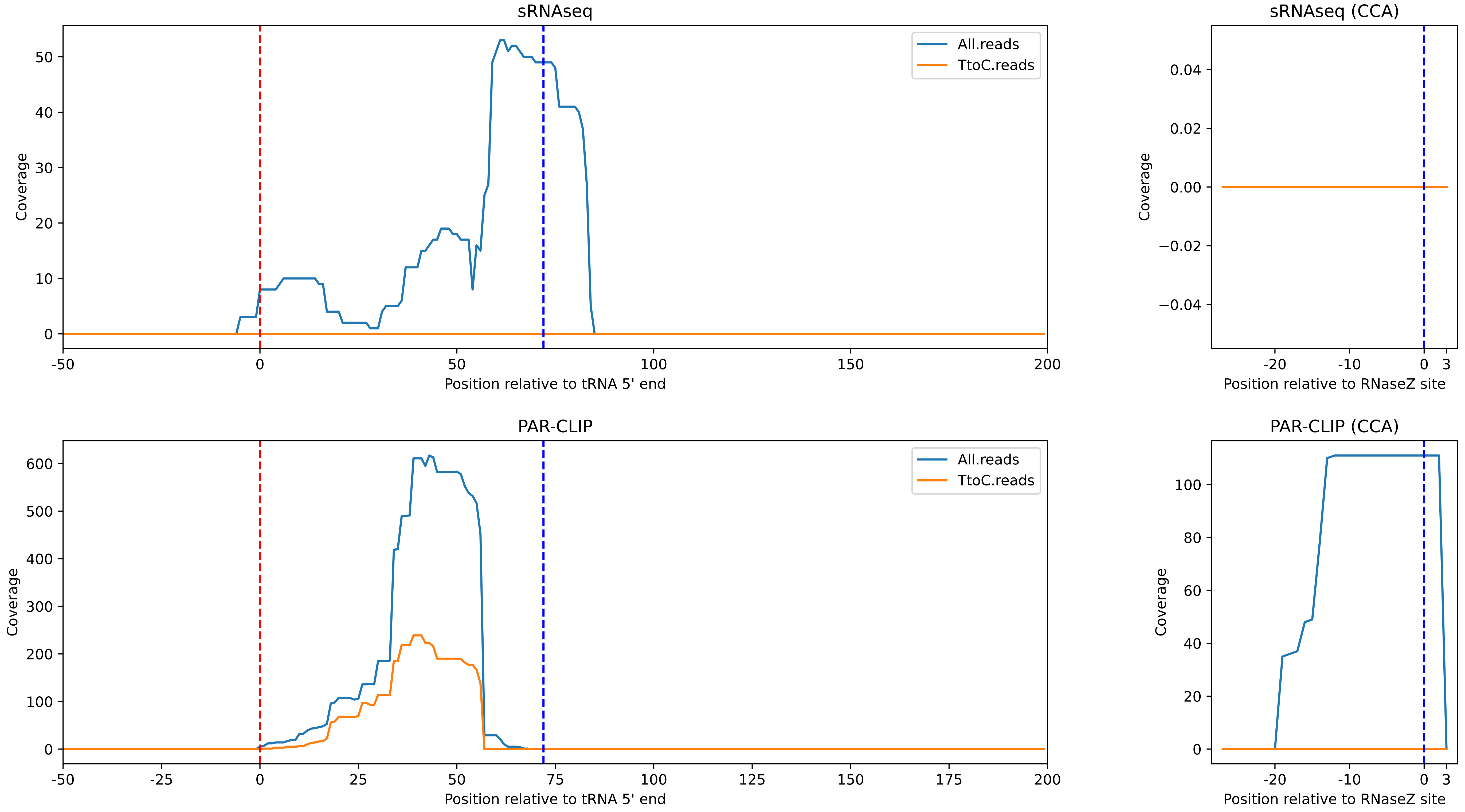

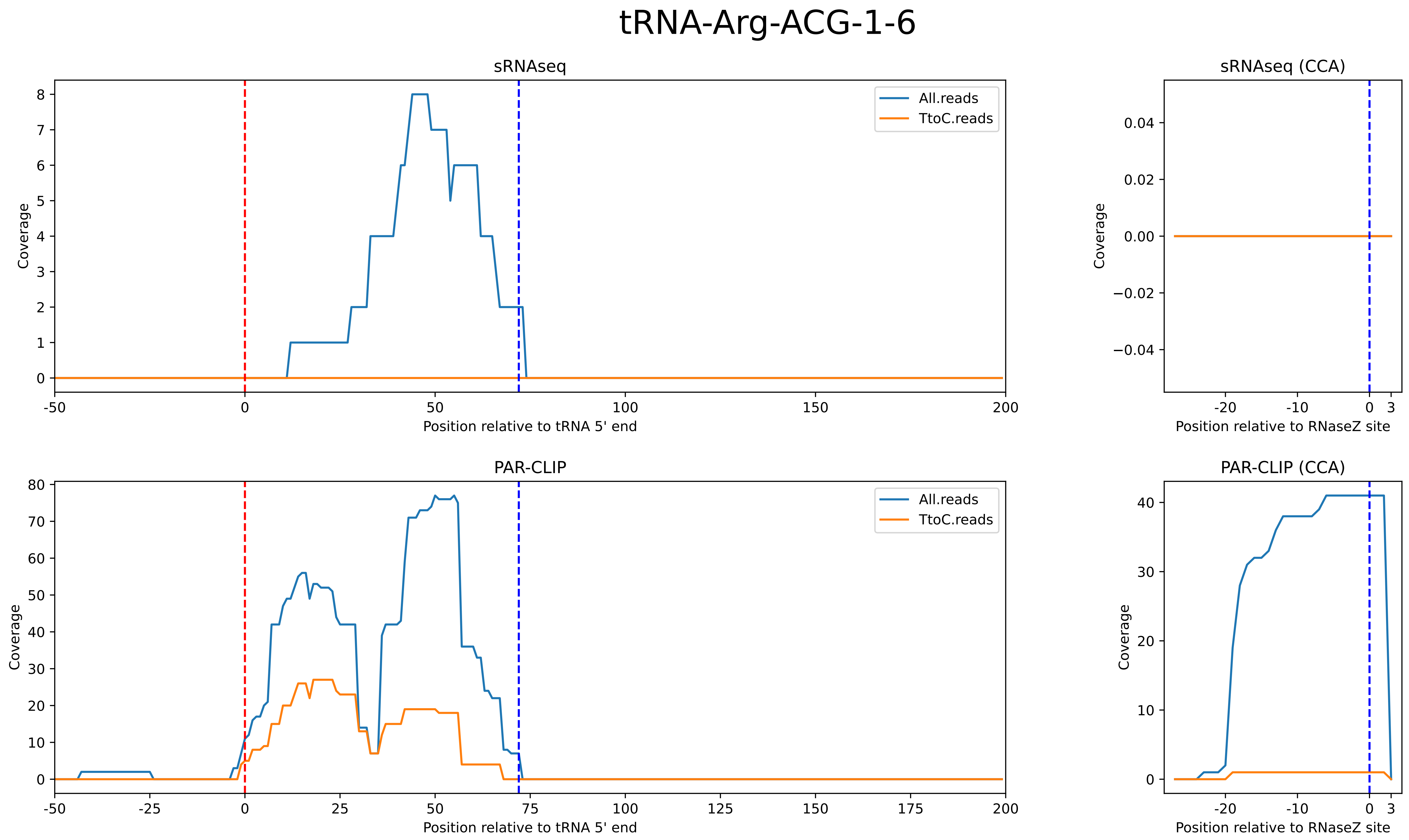

tRNA-Arg-ACG-1-6
sRNAseq
Coverage
Position relative to tRNA 5' end
All.reads
TtoC.reads
sRNAseq (CCA)
Coverage
Position relative to RNaseZ site
PAR-CLIP
Coverage
Position relative to tRNA 5' end
All.reads
TtoC.reads
PAR-CLIP (CCA)
Coverage
Position relative to RNaseZ site

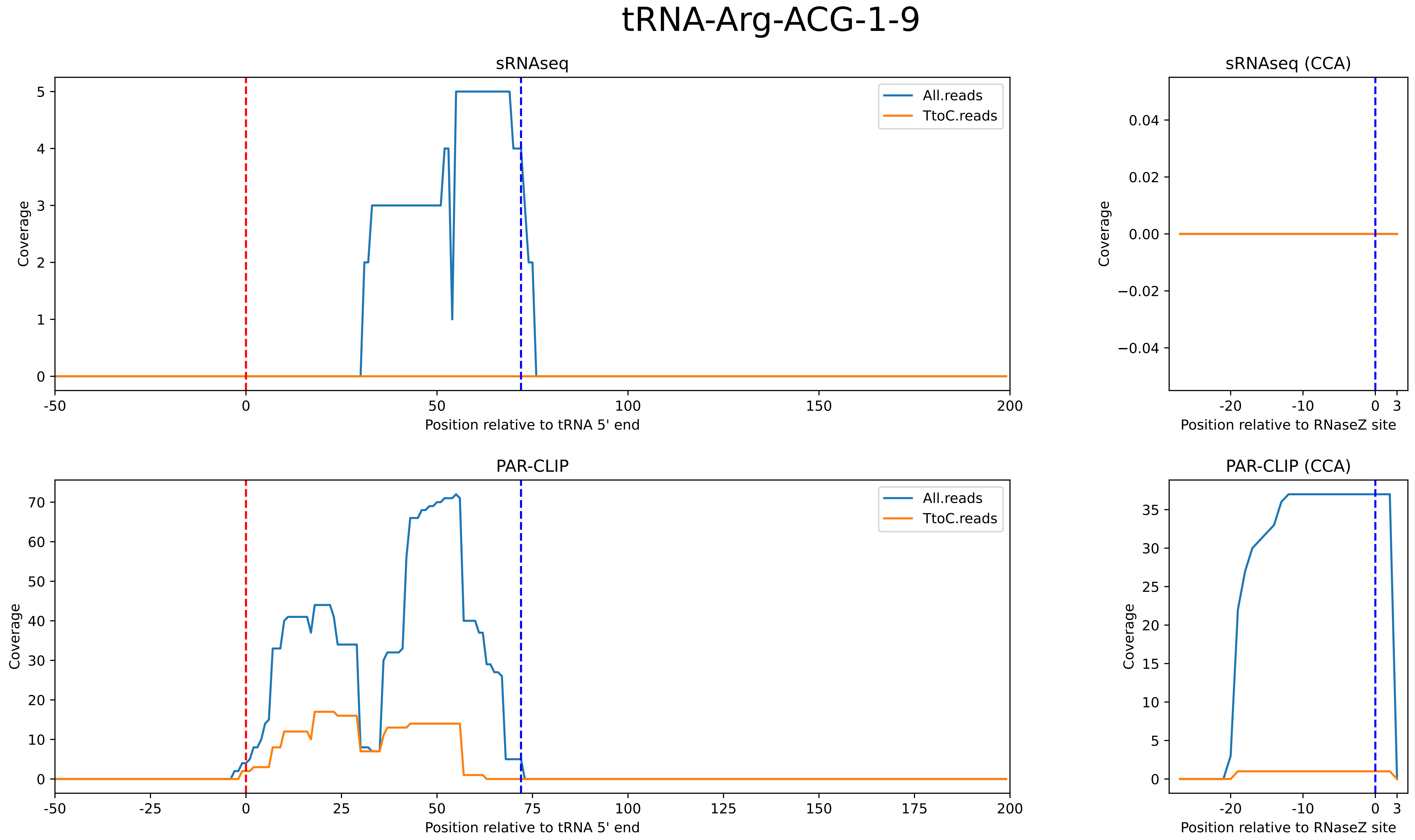

tRNA-Arg-ACG-1-9
sRNAseq
All.reads
TtoC.reads
Coverage
Position relative to tRNA 5' end
sRNAseq (CCA)
Position relative to RNaseZ site
PAR-CLIP
PAR-CLIP (CCA)

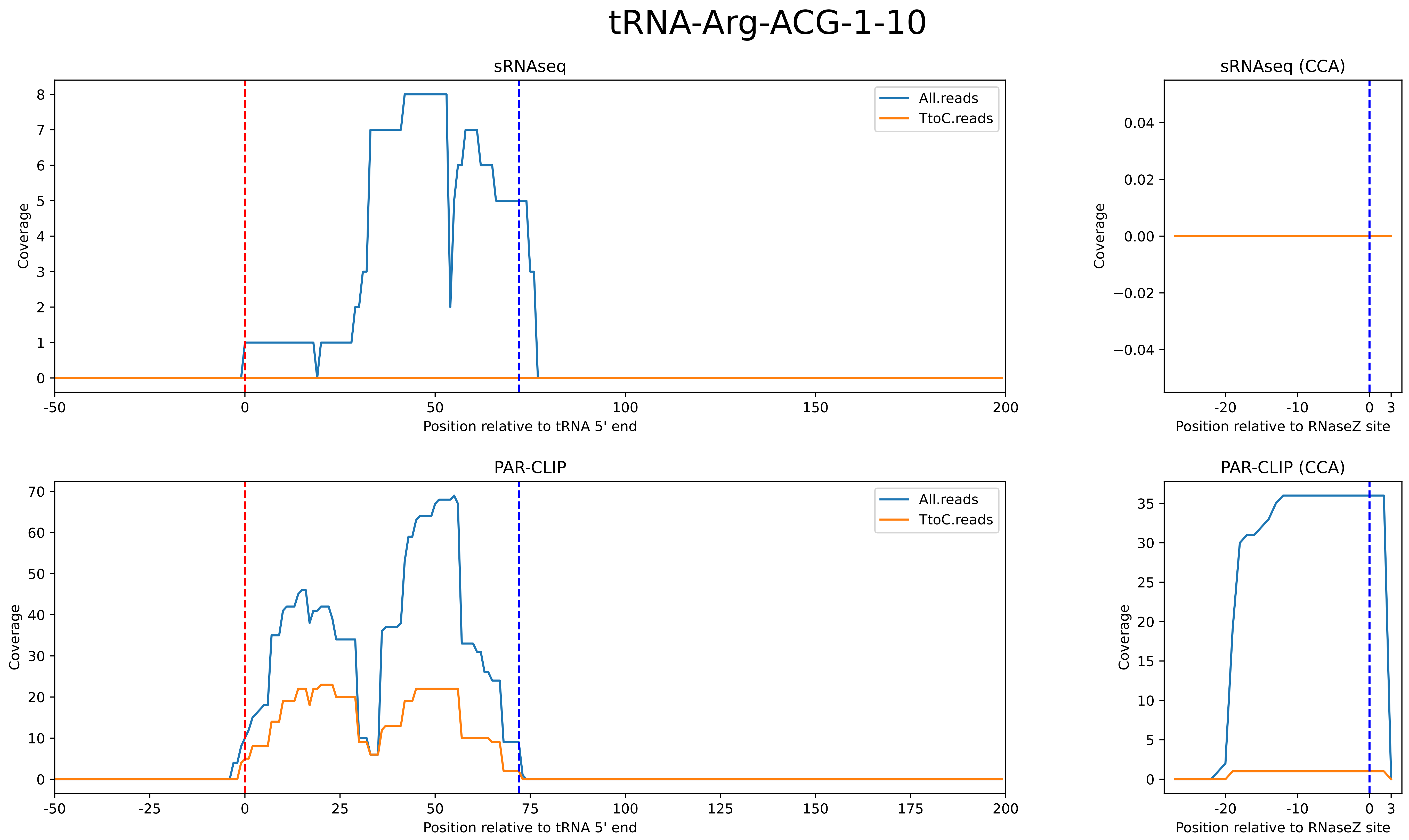

tRNA-Arg-ACG-1-10
sRNAseq
sRNAseq (CCA)
PAR-CLIP
PAR-CLIP (CCA)
All.reads
TtoC.reads
Coverage
Position relative to tRNA 5' end
Position relative to RNaseZ site

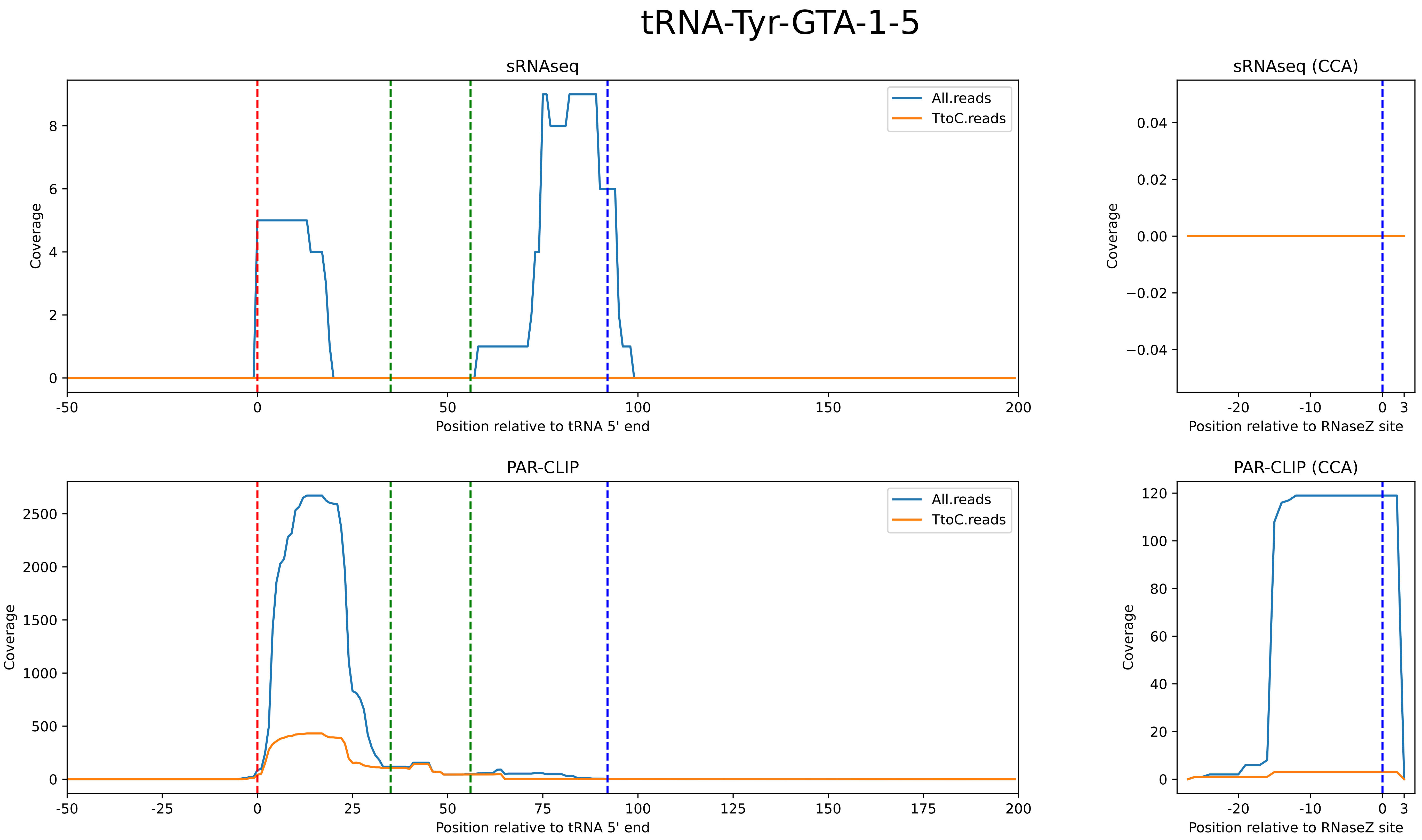

tRNA-Tyr-GTA-1-5
sRNAseq
All.reads
TtoC.reads
Coverage
Position relative to tRNA 5' end
sRNAseq (CCA)
Position relative to RNaseZ site
PAR-CLIP
PAR-CLIP (CCA)

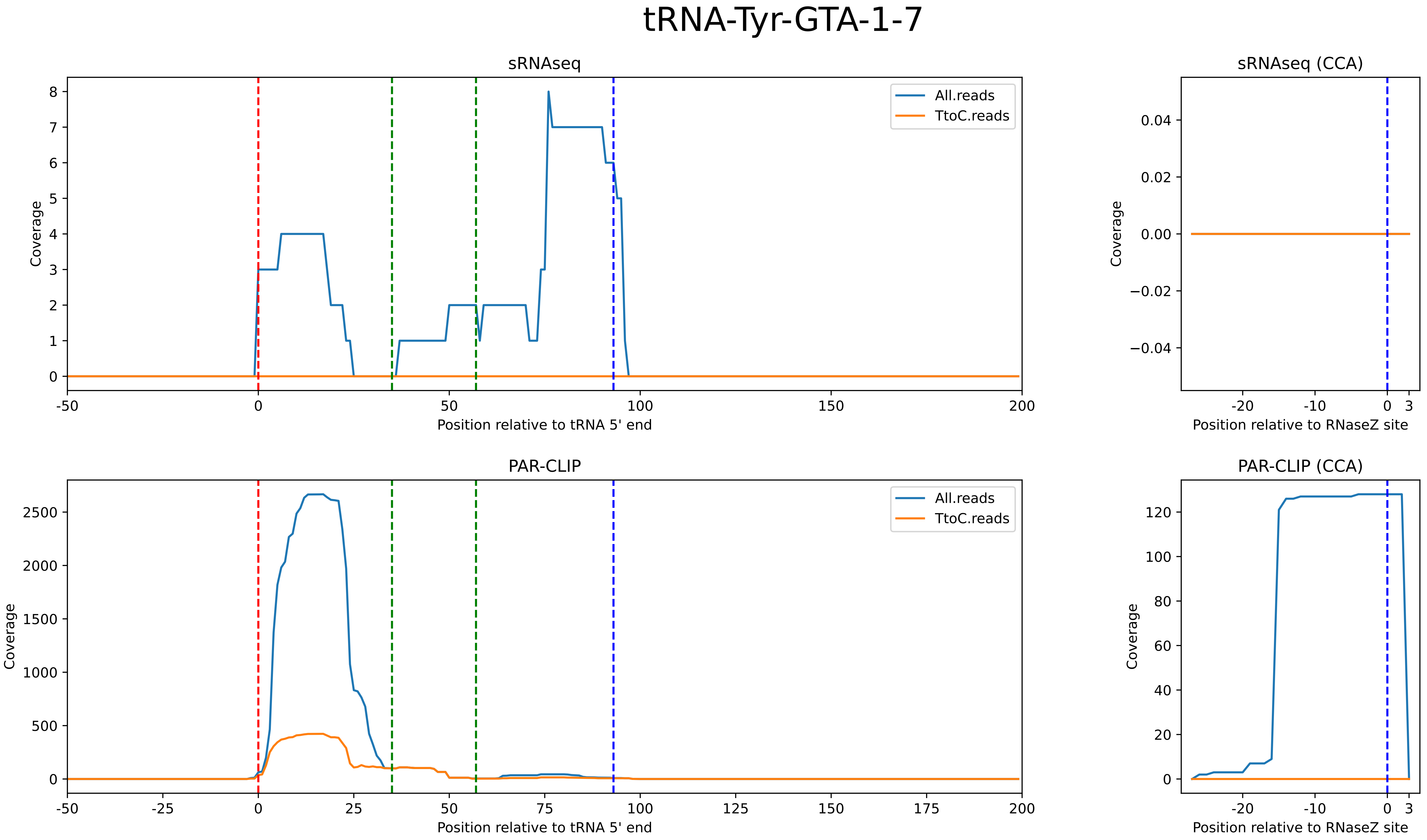
tRNA-Tyr-GTA-1-7
sRNAseq
All.reads
TtoC.reads
Coverage
Position relative to tRNA 5' end
sRNAseq (CCA)
Position relative to RNaseZ site
PAR-CLIP
PAR-CLIP (CCA)

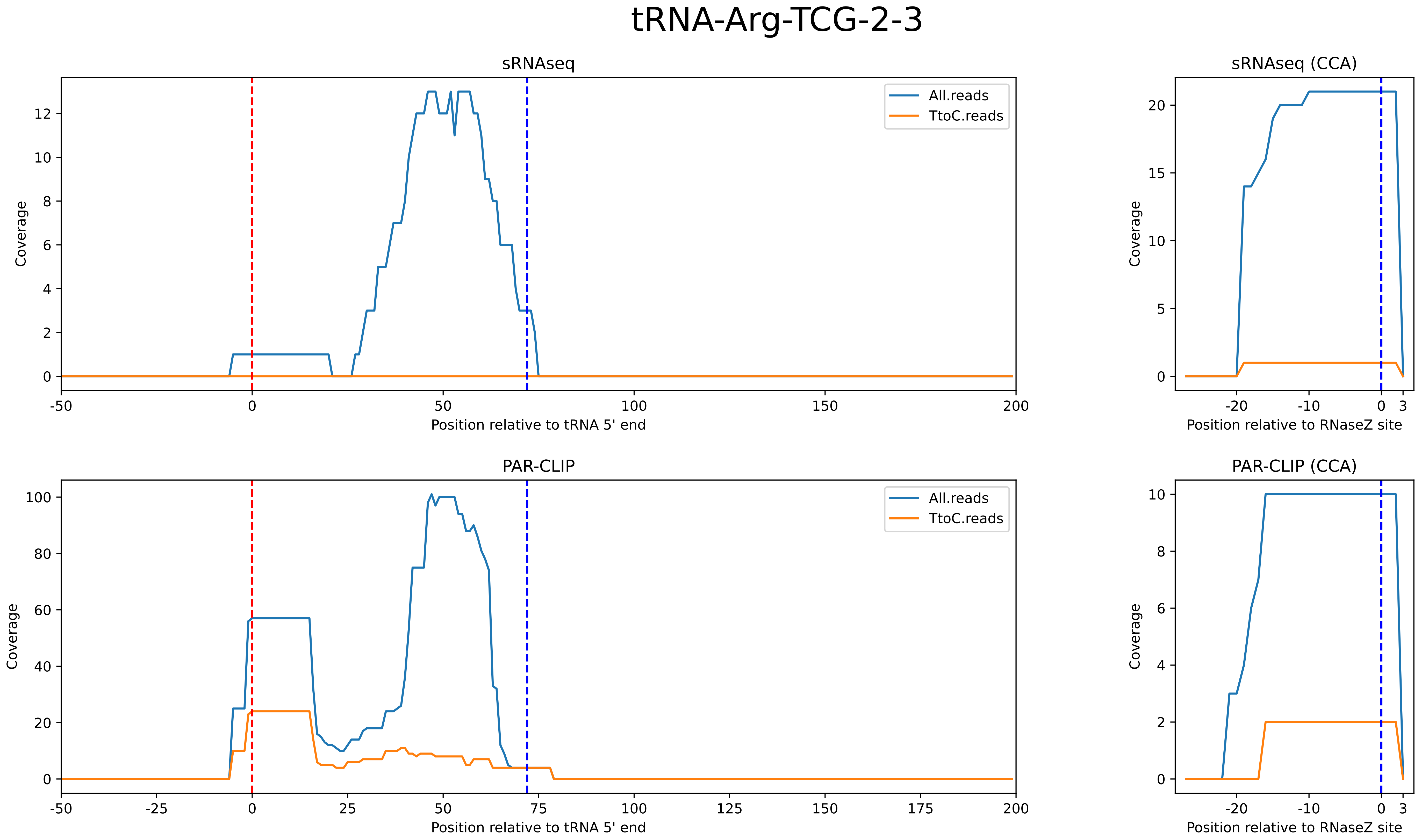

tRNA-Arg-TCG-2-3
sRNAseq
sRNAseq (CCA)
PAR-CLIP
PAR-CLIP (CCA)
All.reads
TtoC.reads
Coverage
Position relative to tRNA 5' end
Position relative to RNaseZ site

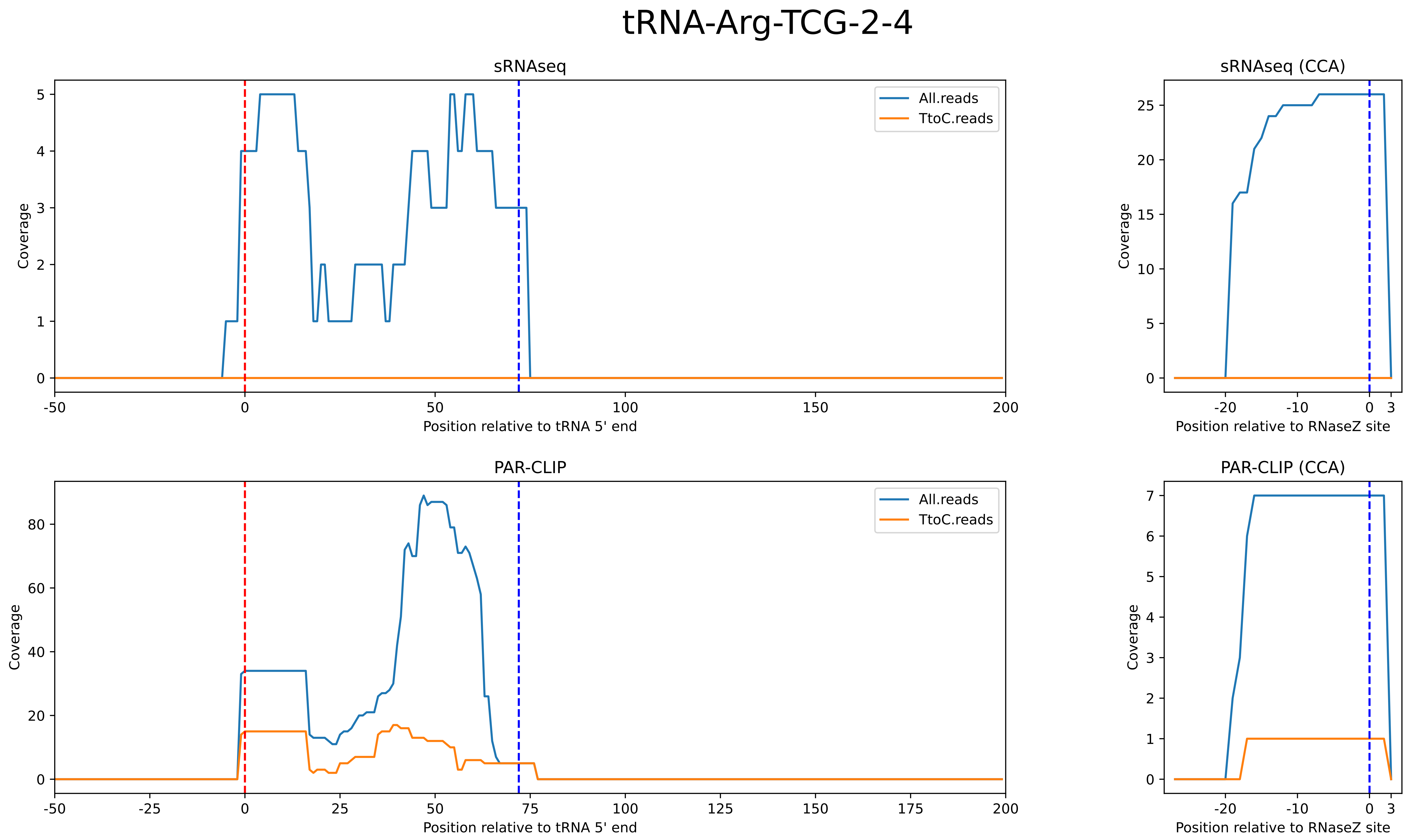

tRNA-Arg-TCG-2-4
sRNAseq
sRNAseq (CCA)
PAR-CLIP
PAR-CLIP (CCA)
All.reads
TtoC.reads
Coverage
Position relative to tRNA 5' end
Position relative to RNaseZ site

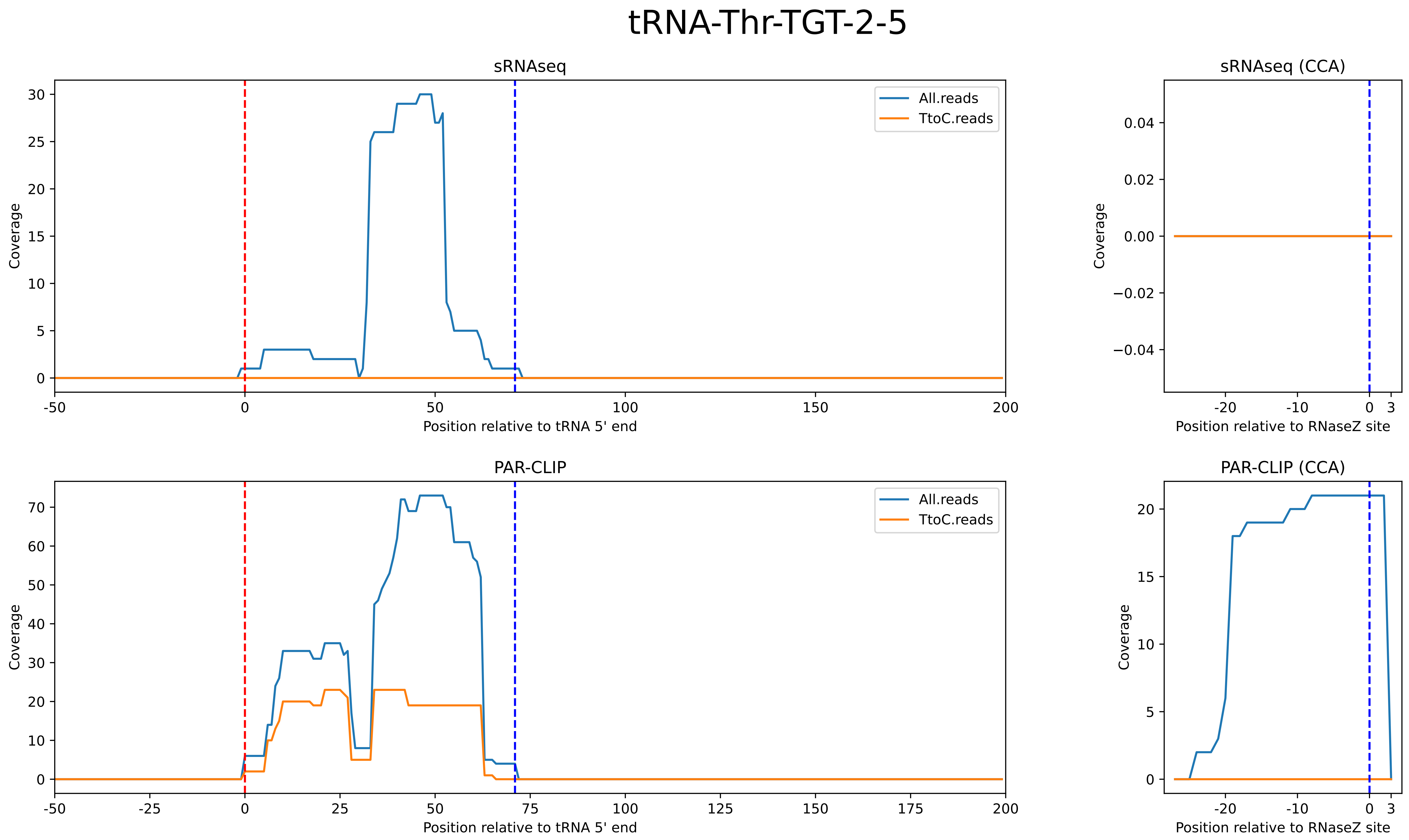

tRNA-Thr-TGT-2-5
sRNAseq
sRNAseq (CCA)
PAR-CLIP
PAR-CLIP (CCA)
All.reads
TtoC.reads
Coverage
Position relative to tRNA 5' end
Position relative to RNaseZ site

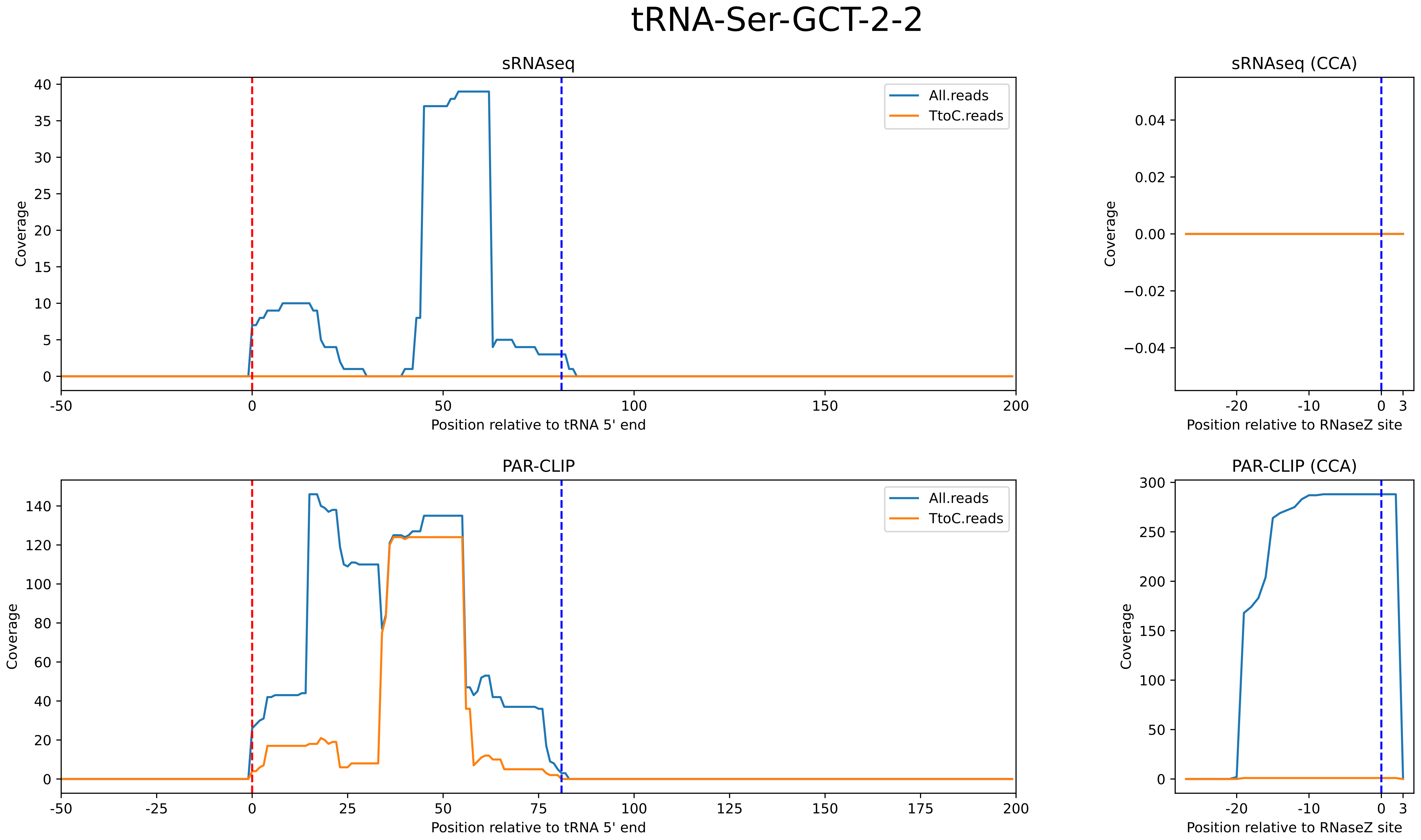
tRNA-Ser-GCT-2-2
sRNAseq
All.reads
TtoC.reads
Coverage
Position relative to tRNA 5' end
sRNAseq (CCA)
Position relative to RNaseZ site
PAR-CLIP
PAR-CLIP (CCA)

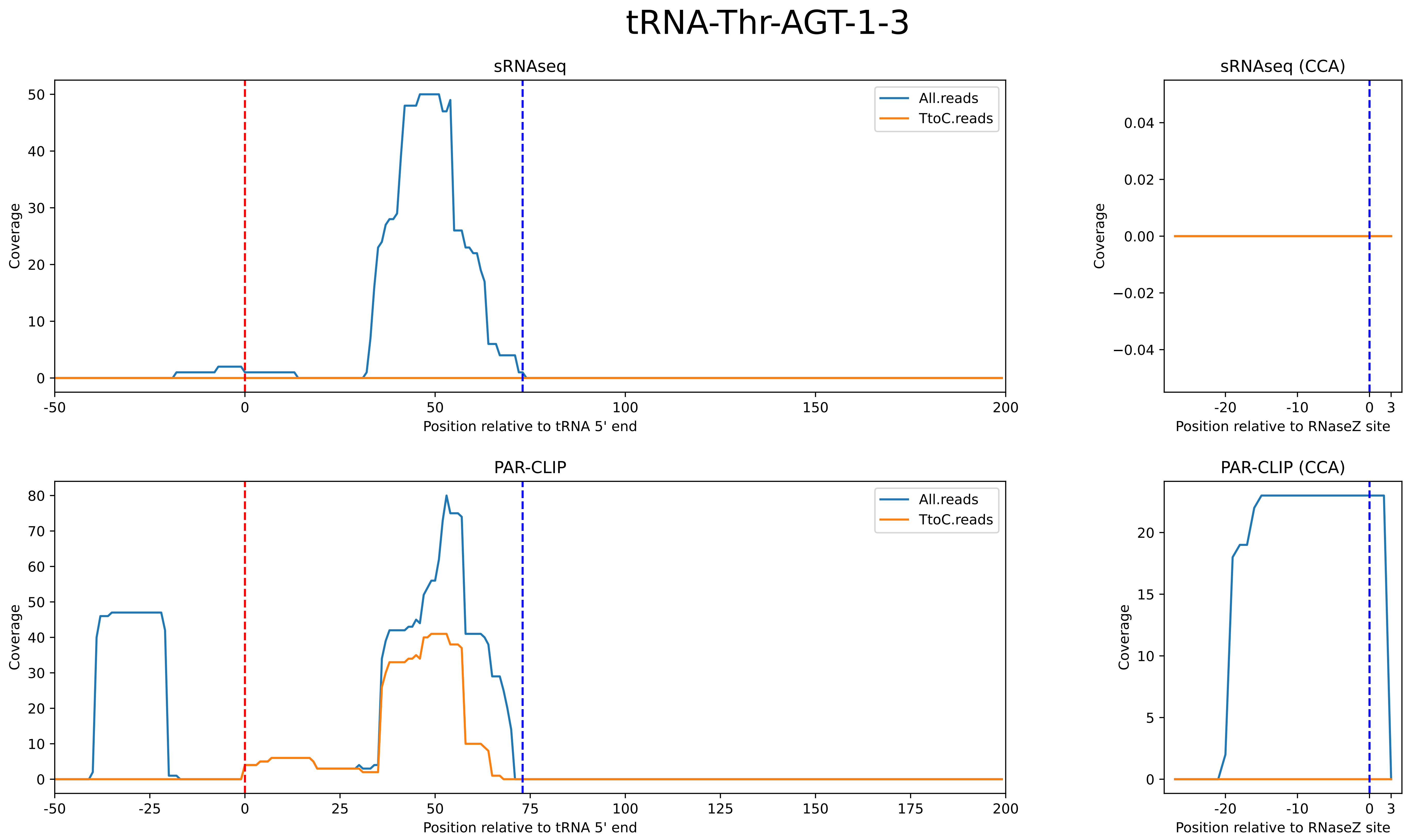

tRNA-Thr-AGT-1-3
sRNAseq
All.reads
TtoC.reads
Coverage
Position relative to tRNA 5' end
sRNAseq (CCA)
Position relative to RNaseZ site
PAR-CLIP
PAR-CLIP (CCA)

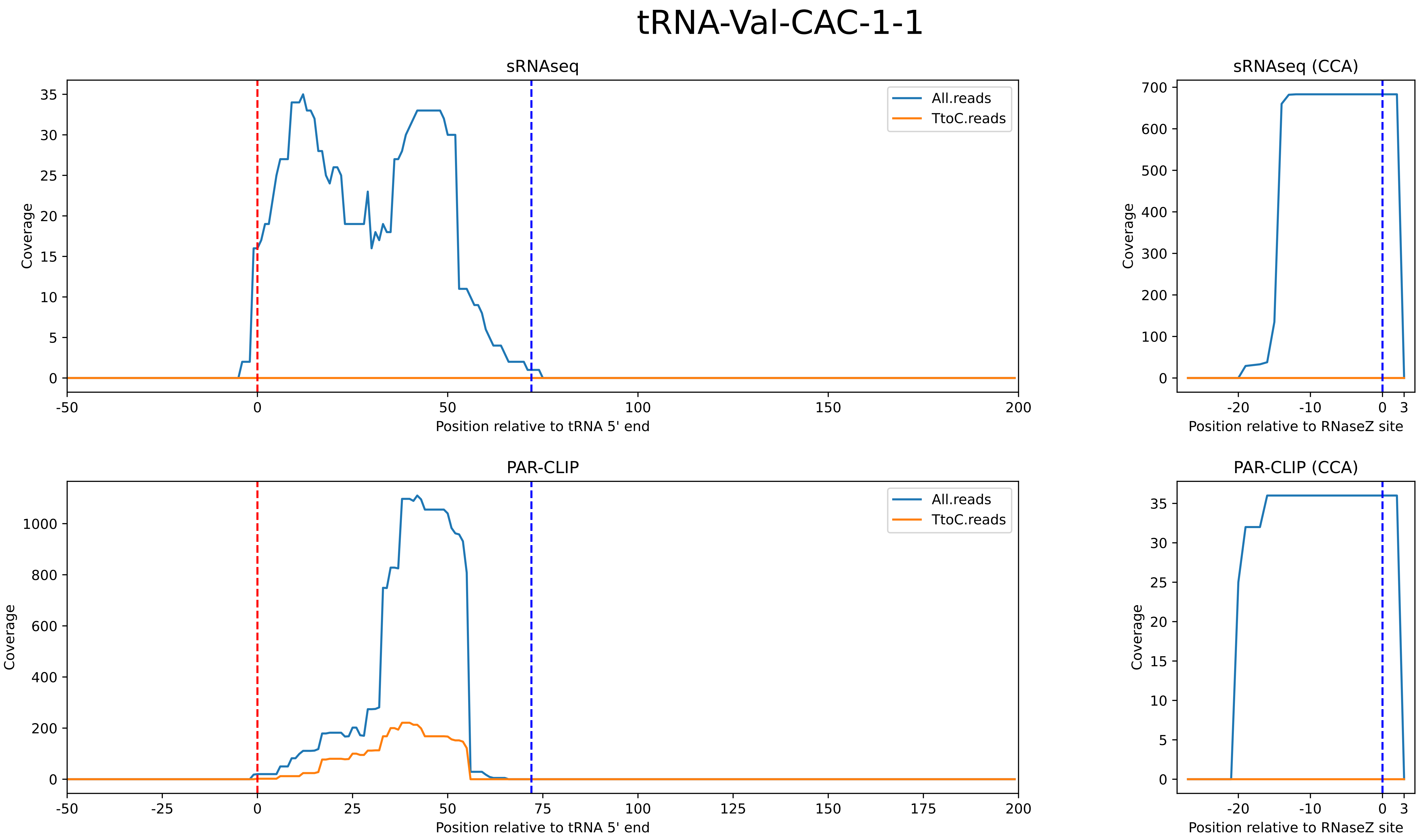

tRNA-Val-CAC-1-1
sRNAseq
All.reads
TtoC.reads
Coverage
Position relative to tRNA 5' end
sRNAseq (CCA)
Position relative to RNaseZ site
PAR-CLIP
PAR-CLIP (CCA)

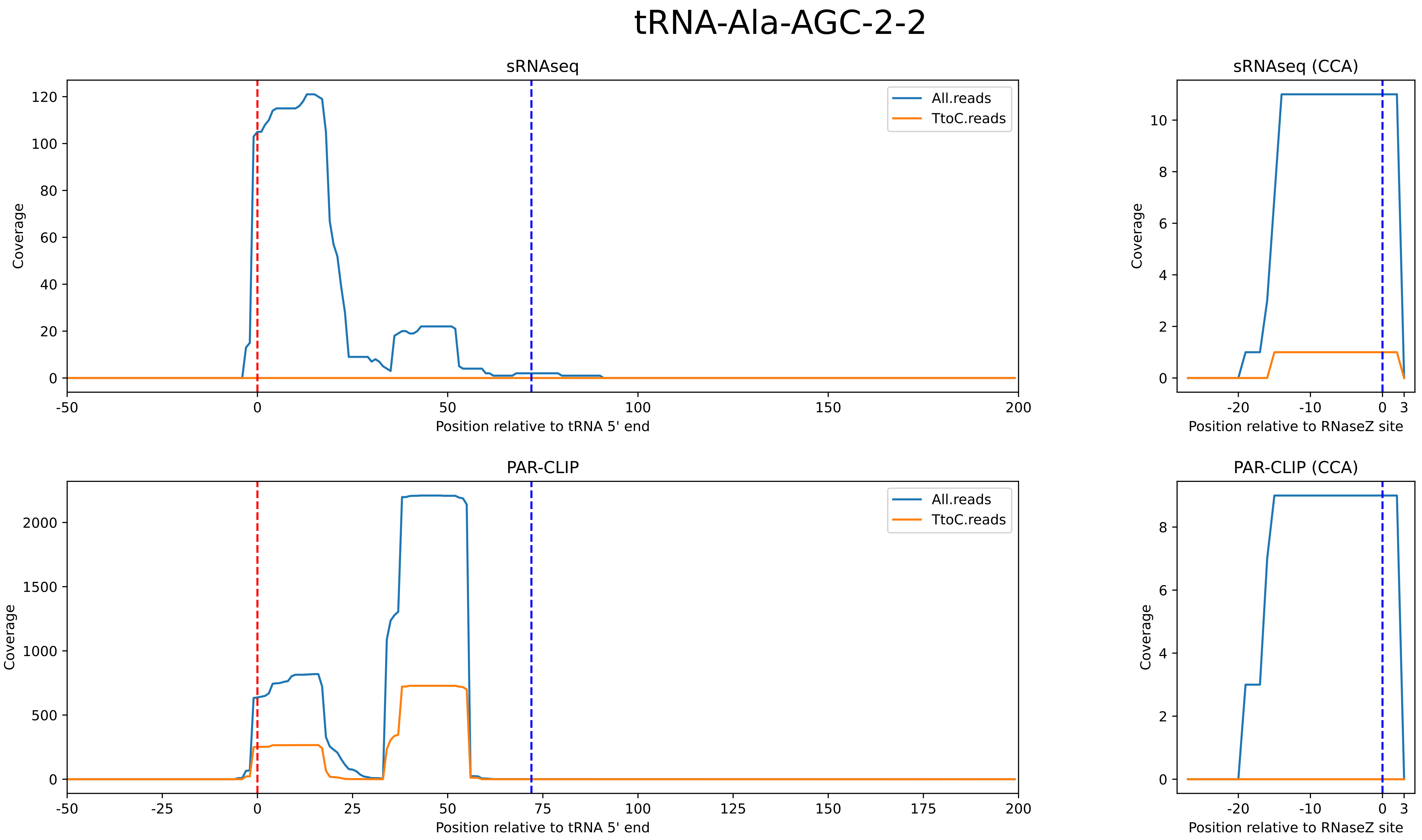

tRNA-Ala-AGC-2-2
sRNAseq
All.reads
TtoC.reads
Coverage
Position relative to tRNA 5' end
sRNAseq (CCA)
Position relative to RNaseZ site
PAR-CLIP
PAR-CLIP (CCA)

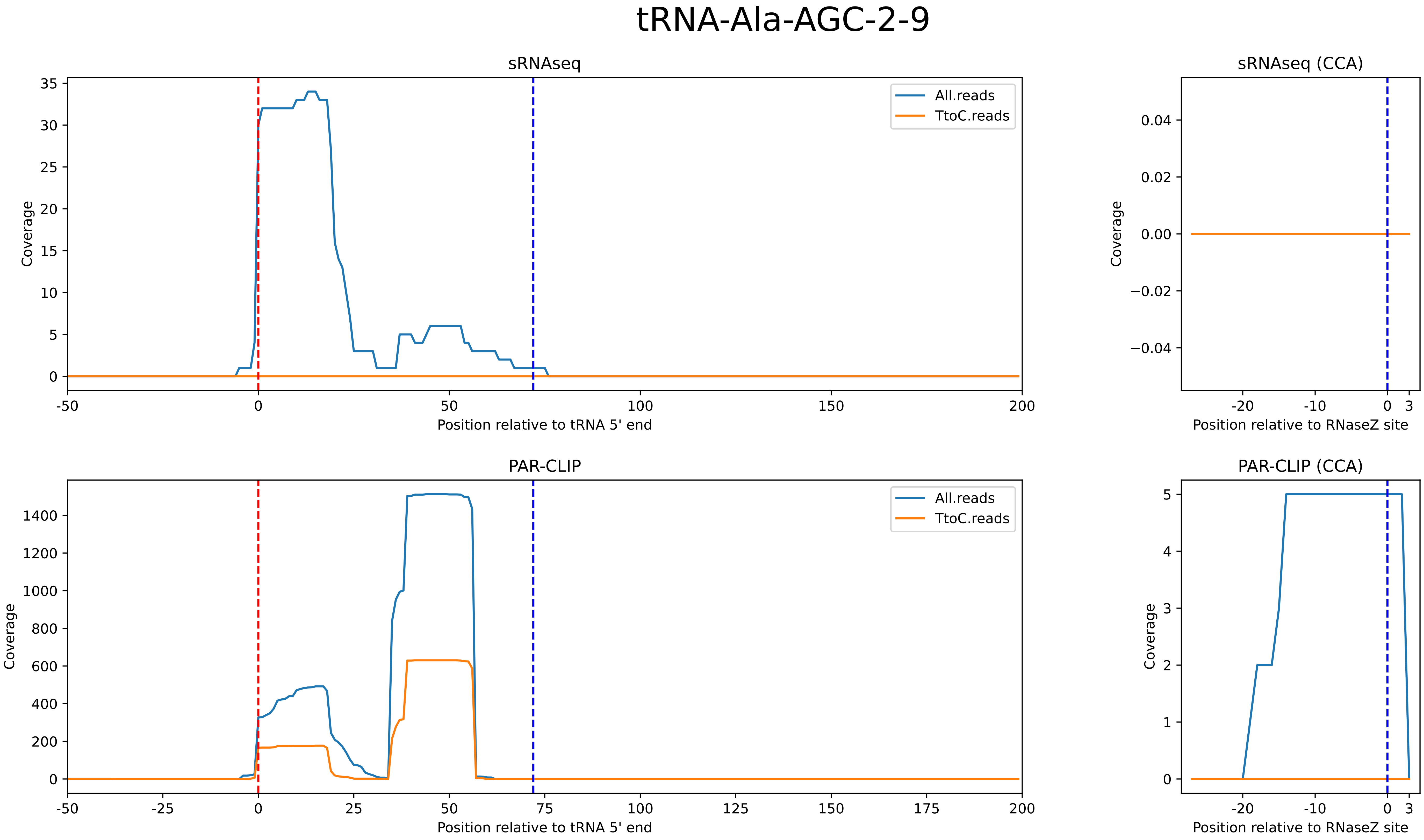

tRNA-Ala-AGC-2-9
sRNAseq
All.reads
TtoC.reads
Coverage
Position relative to tRNA 5' end
sRNAseq (CCA)
Position relative to RNaseZ site
PAR-CLIP
PAR-CLIP (CCA)

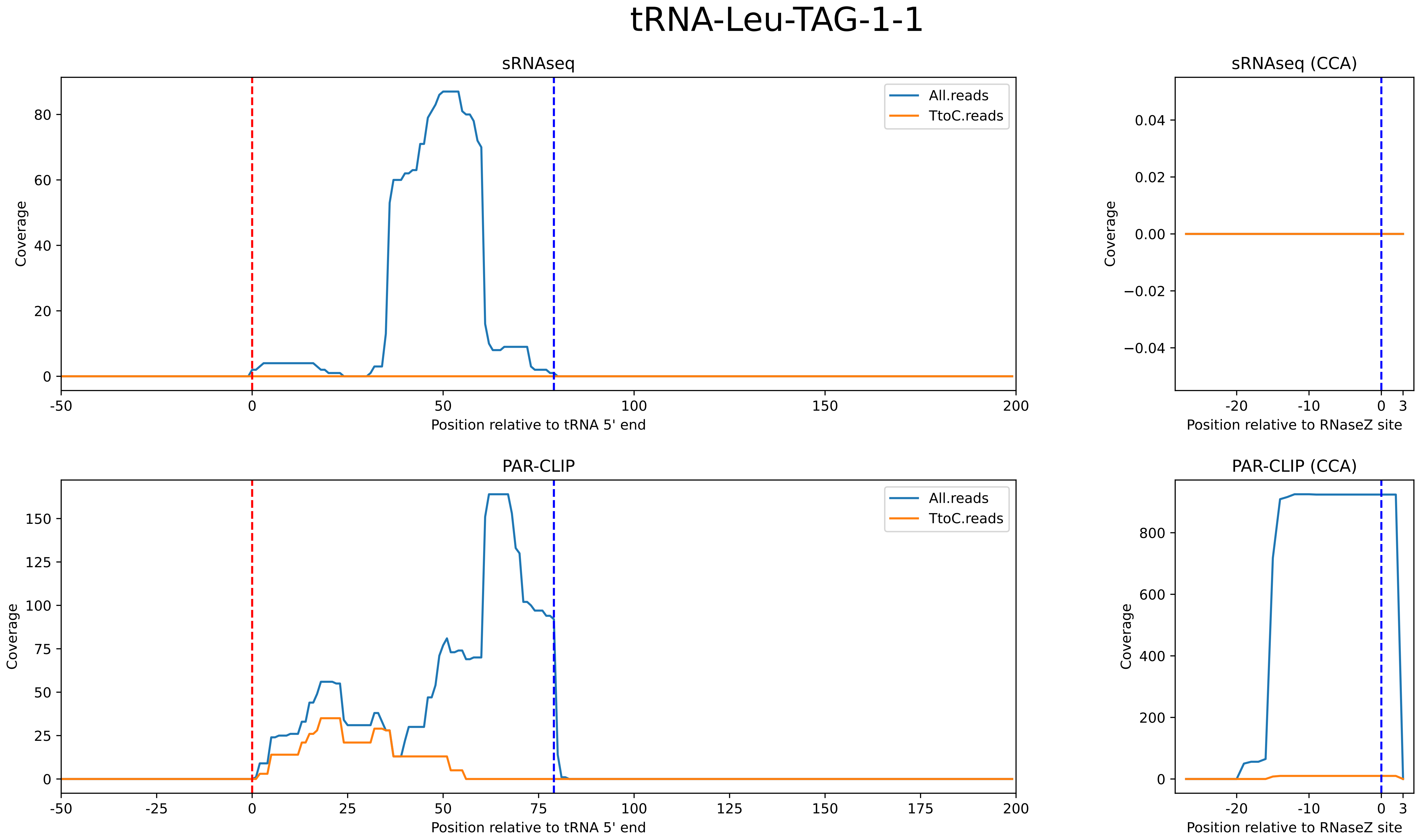

tRNA-Leu-TAG-1-1
sRNAseq
All.reads
TtoC.reads
Coverage
Position relative to tRNA 5' end
sRNAseq (CCA)
Position relative to RNaseZ site
PAR-CLIP
PAR-CLIP (CCA)

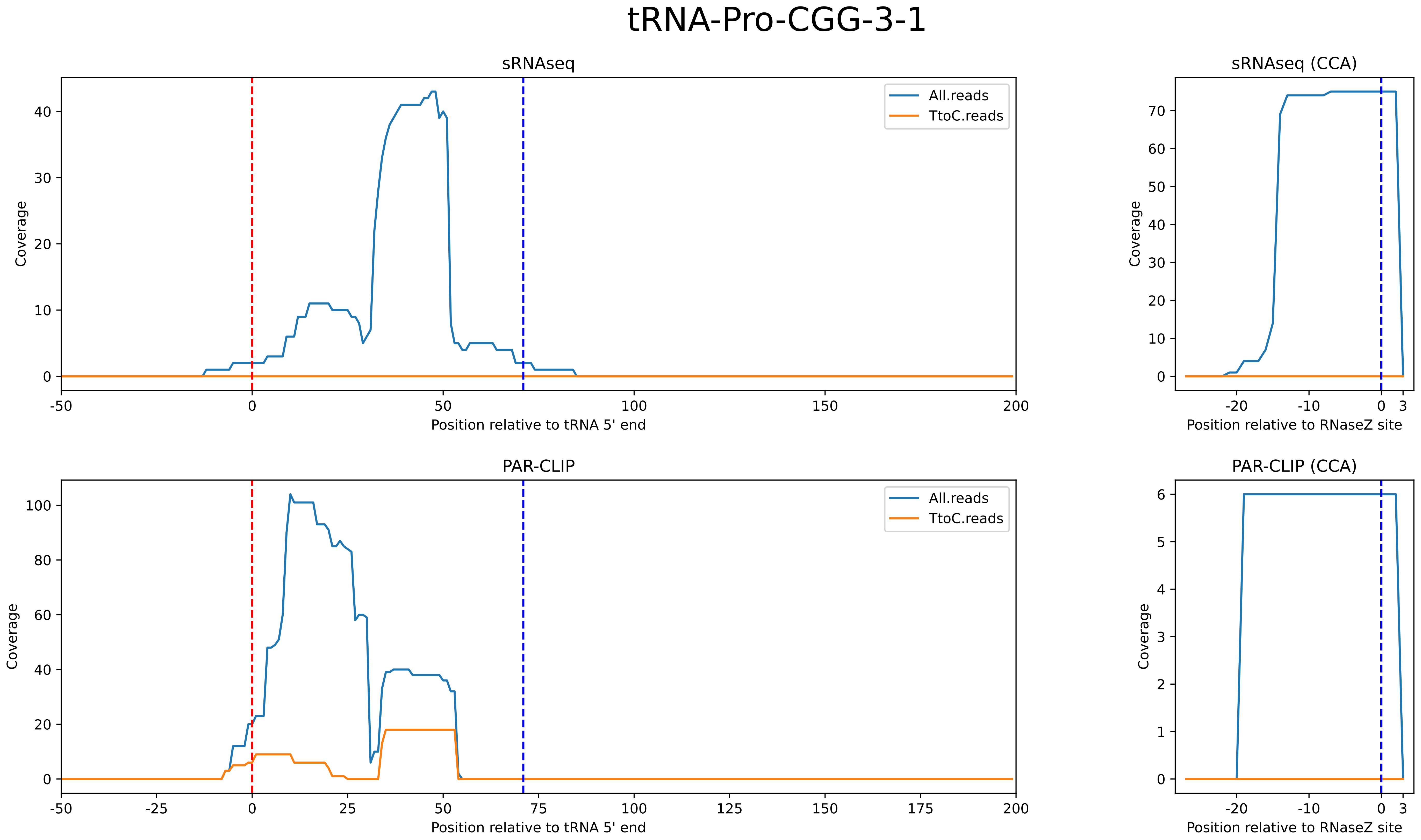
tRNA-Pro-CGG-3-1
sRNAseq
sRNAseq (CCA)
PAR-CLIP
PAR-CLIP (CCA)
All.reads
TtoC.reads
Coverage
Position relative to tRNA 5' end
Position relative to RNaseZ site

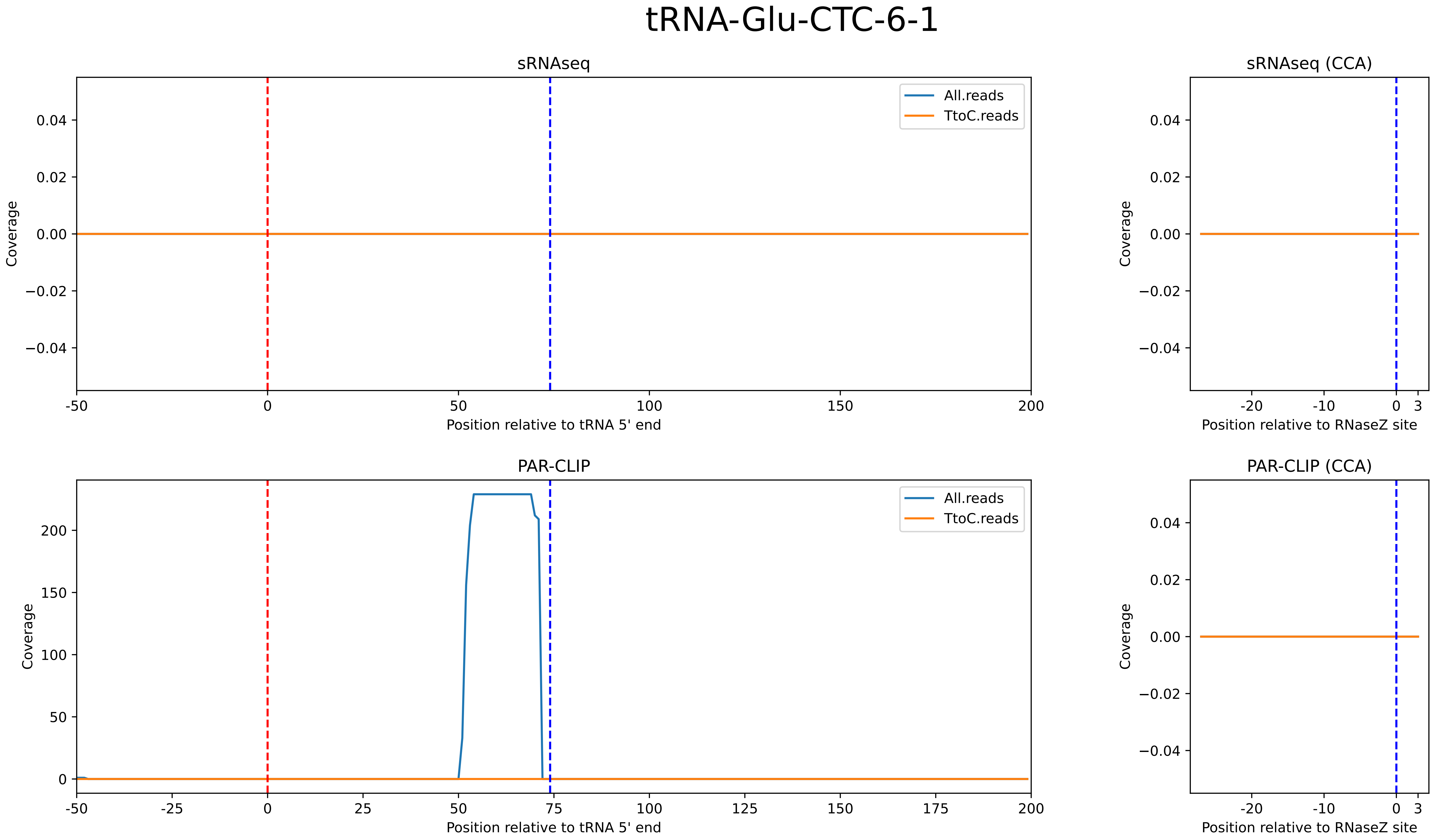
tRNA-Glu-CTC-6-1
sRNAseq
sRNAseq (CCA)
PAR-CLIP
PAR-CLIP (CCA)
All.reads
TtoC.reads
Coverage
Position relative to tRNA 5' end
Position relative to RNaseZ site

# tRNA-Arg-TCG-2-1

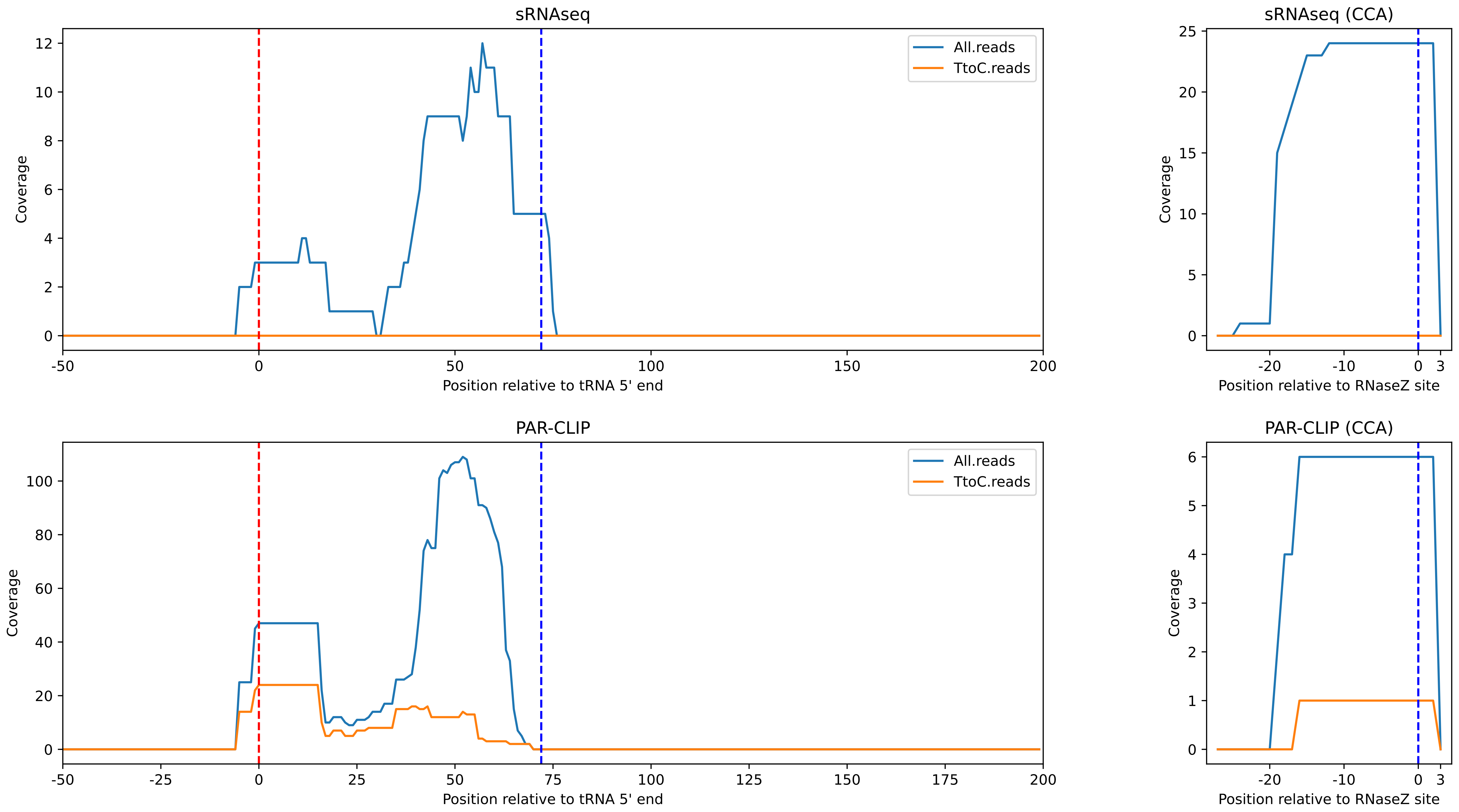

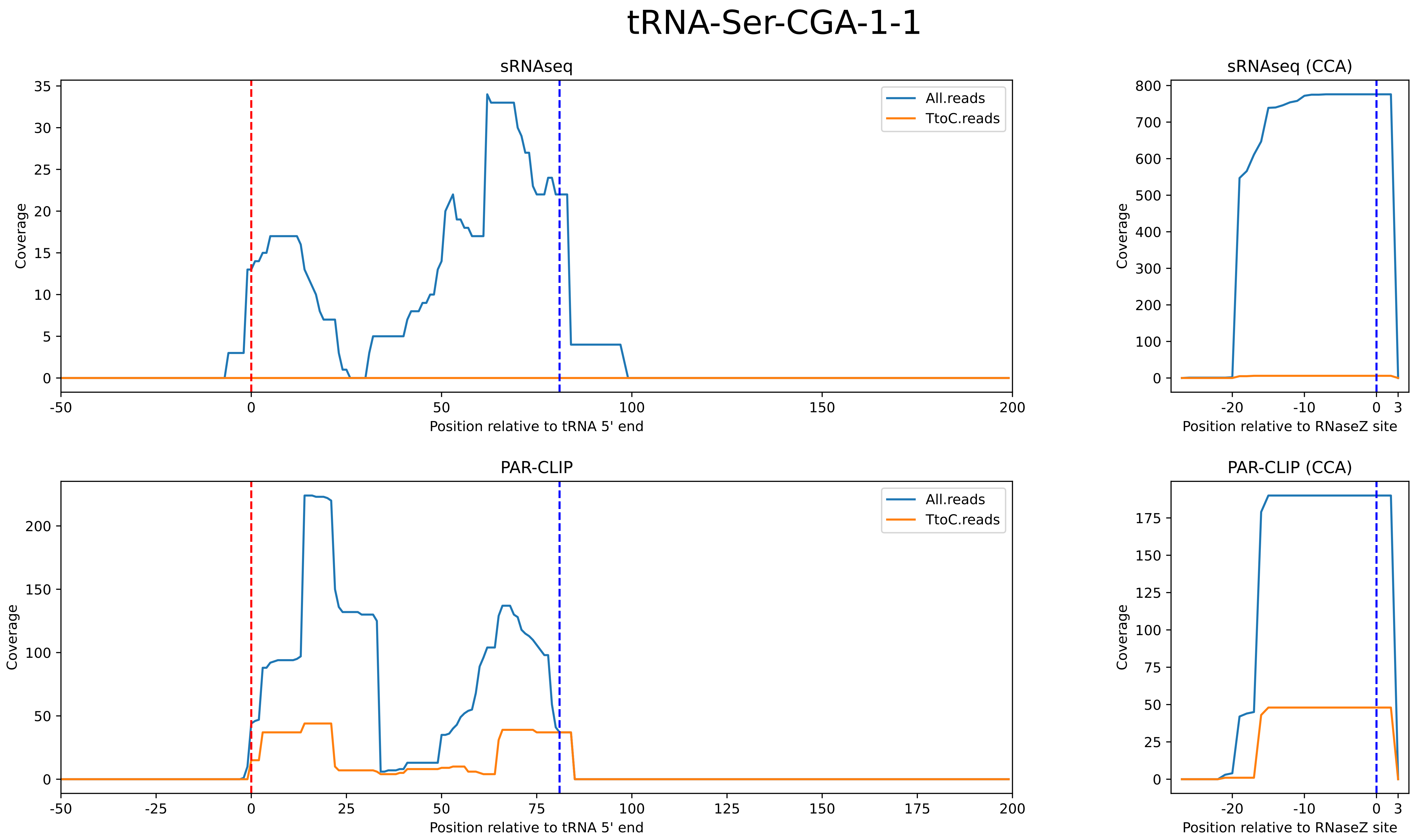
tRNA-Ser-CGA-1-1
sRNAseq
Coverage
Position relative to tRNA 5' end
All.reads
TtoC.reads
sRNAseq (CCA)
Position relative to RNaseZ site
PAR-CLIP
PAR-CLIP (CCA)

# tRNA-Ser-CGA-1-2

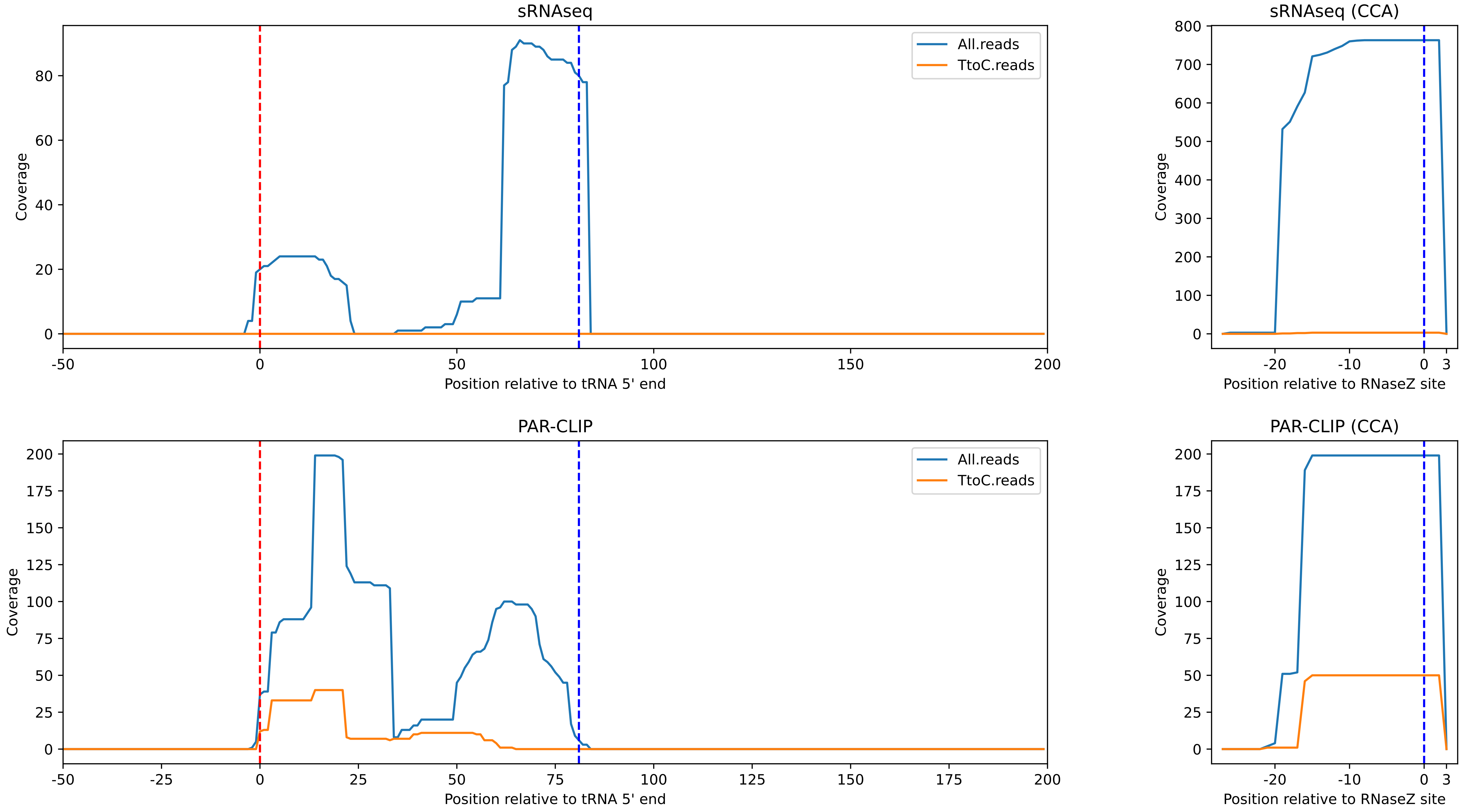

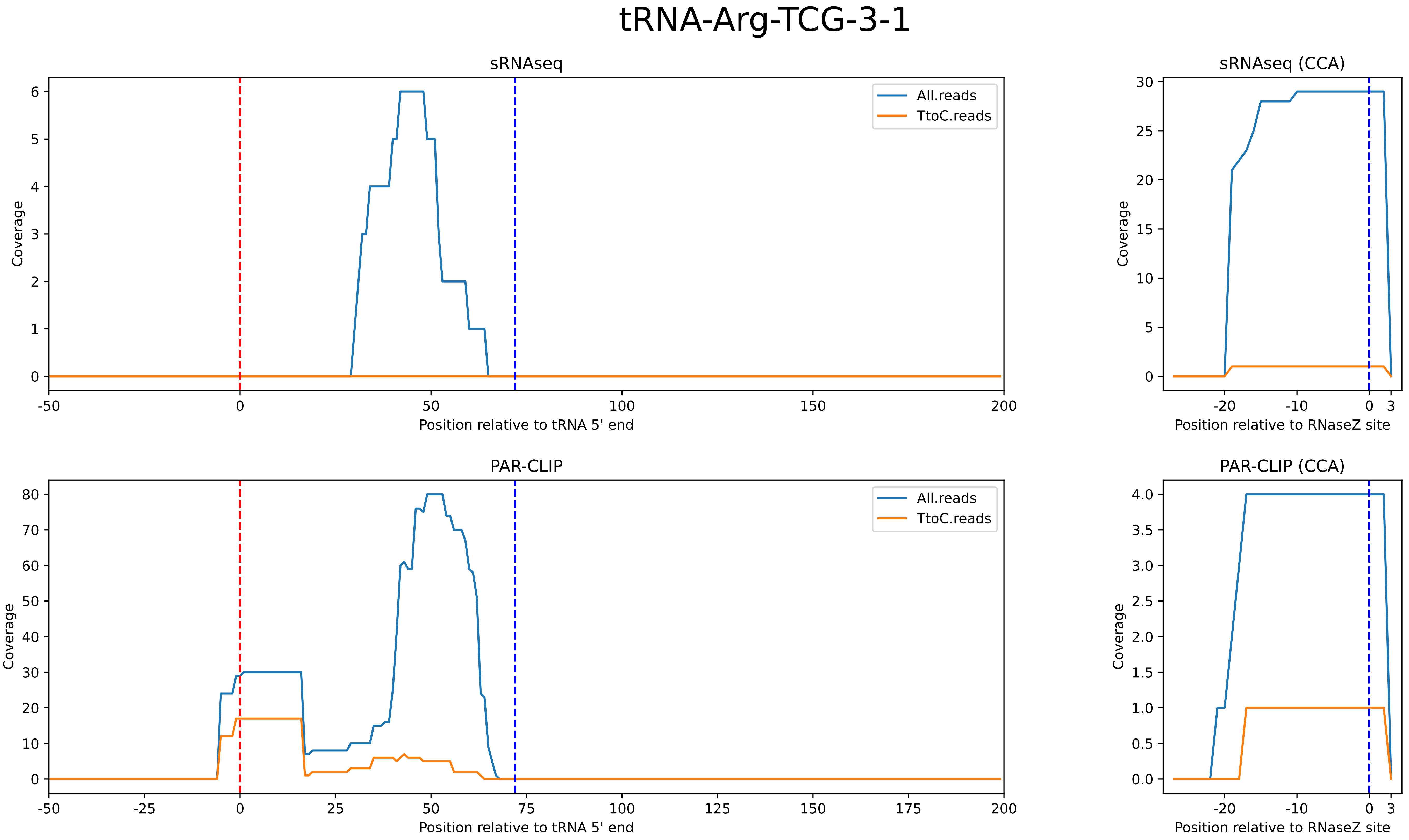
tRNA-Arg-TCG-3-1
sRNAseq
All.reads
TtoC.reads
Coverage
Position relative to tRNA 5' end
sRNAseq (CCA)
Position relative to RNaseZ site
PAR-CLIP
PAR-CLIP (CCA)

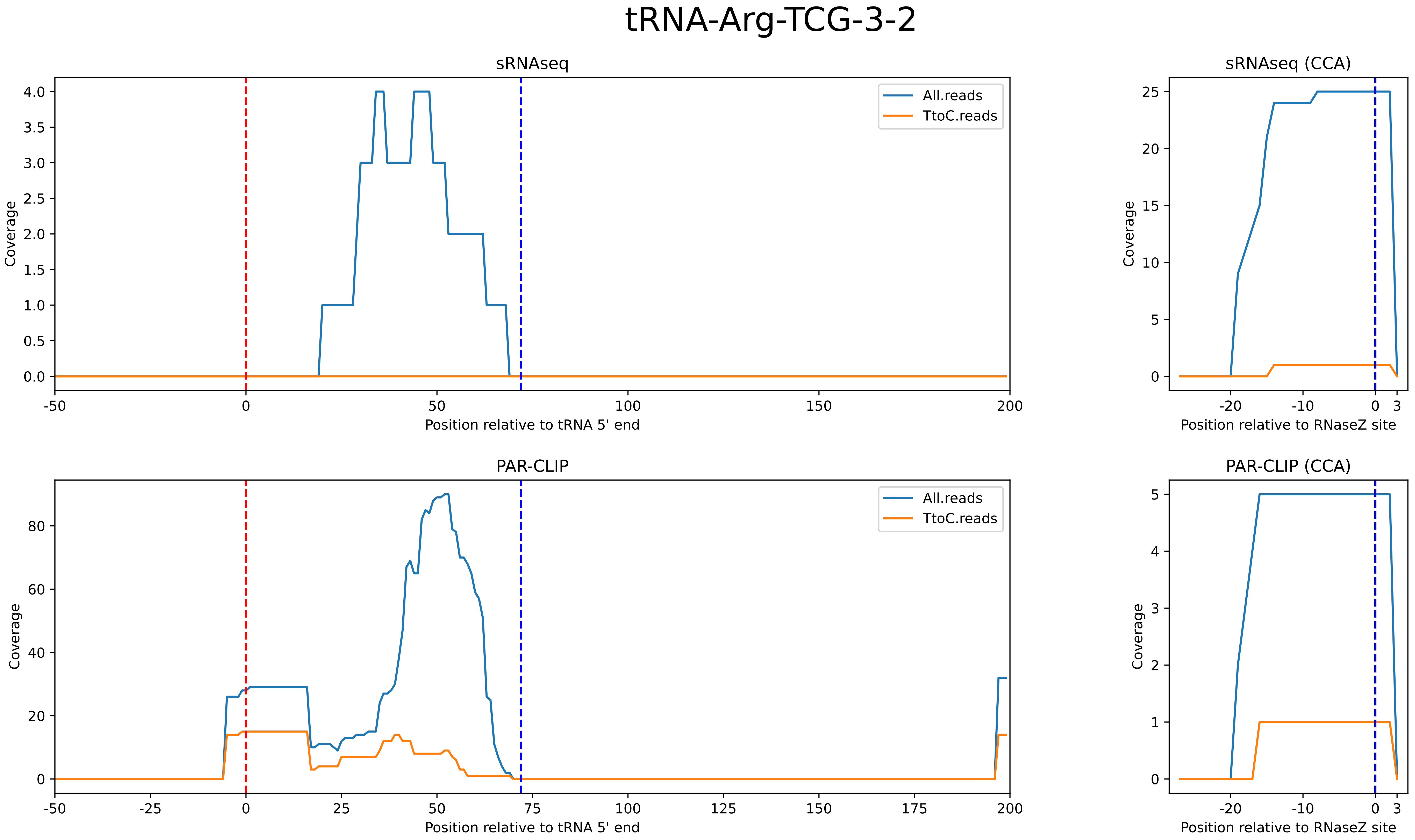
tRNA-Arg-TCG-3-2
sRNAseq
All.reads
TtoC.reads
Coverage
Position relative to tRNA 5' end
sRNAseq (CCA)
Position relative to RNaseZ site
PAR-CLIP
PAR-CLIP (CCA)

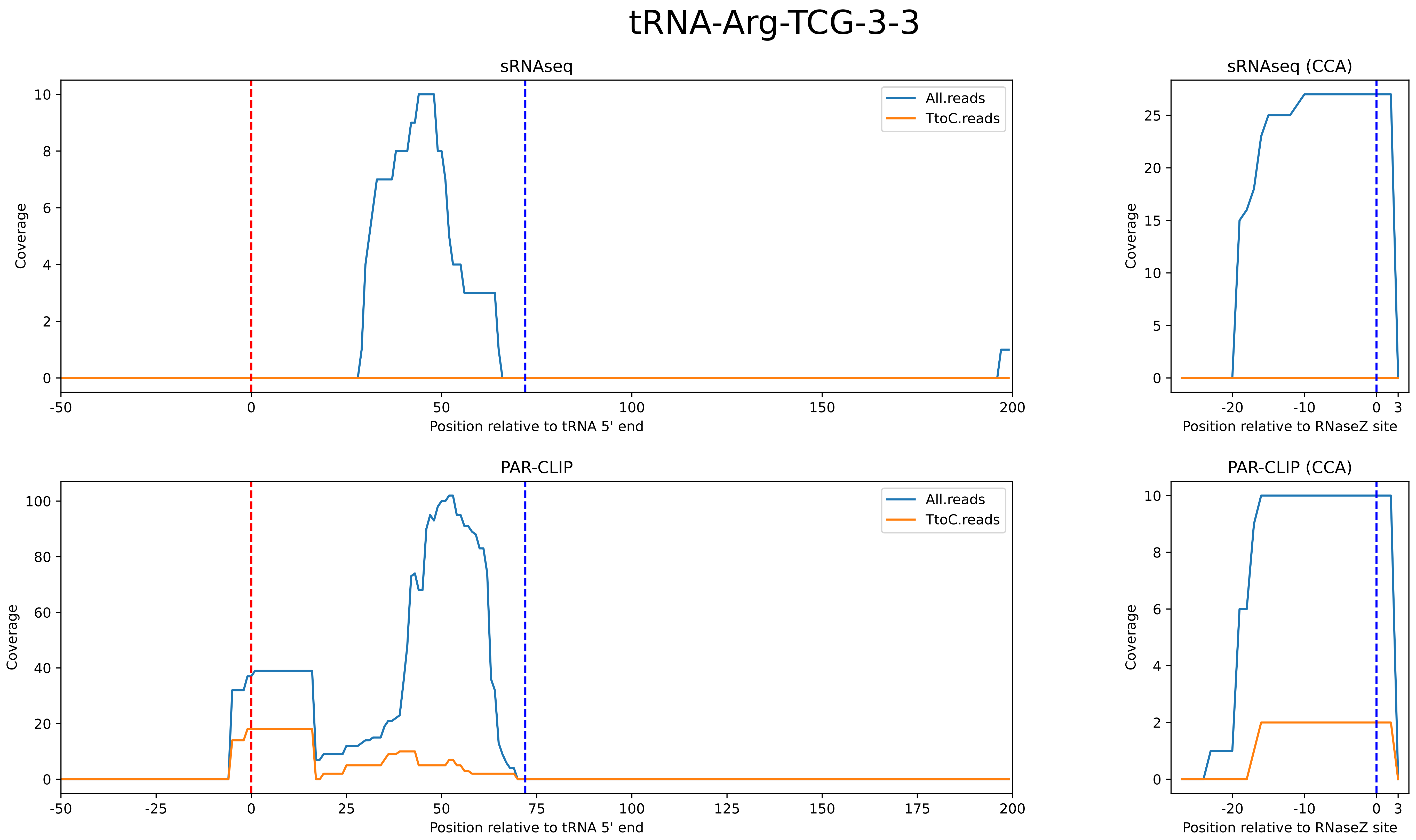

tRNA-Arg-TCG-3-3
sRNAseq
All.reads
TtoC.reads
Coverage
Position relative to tRNA 5' end
sRNAseq (CCA)
Position relative to RNaseZ site
PAR-CLIP
PAR-CLIP (CCA)

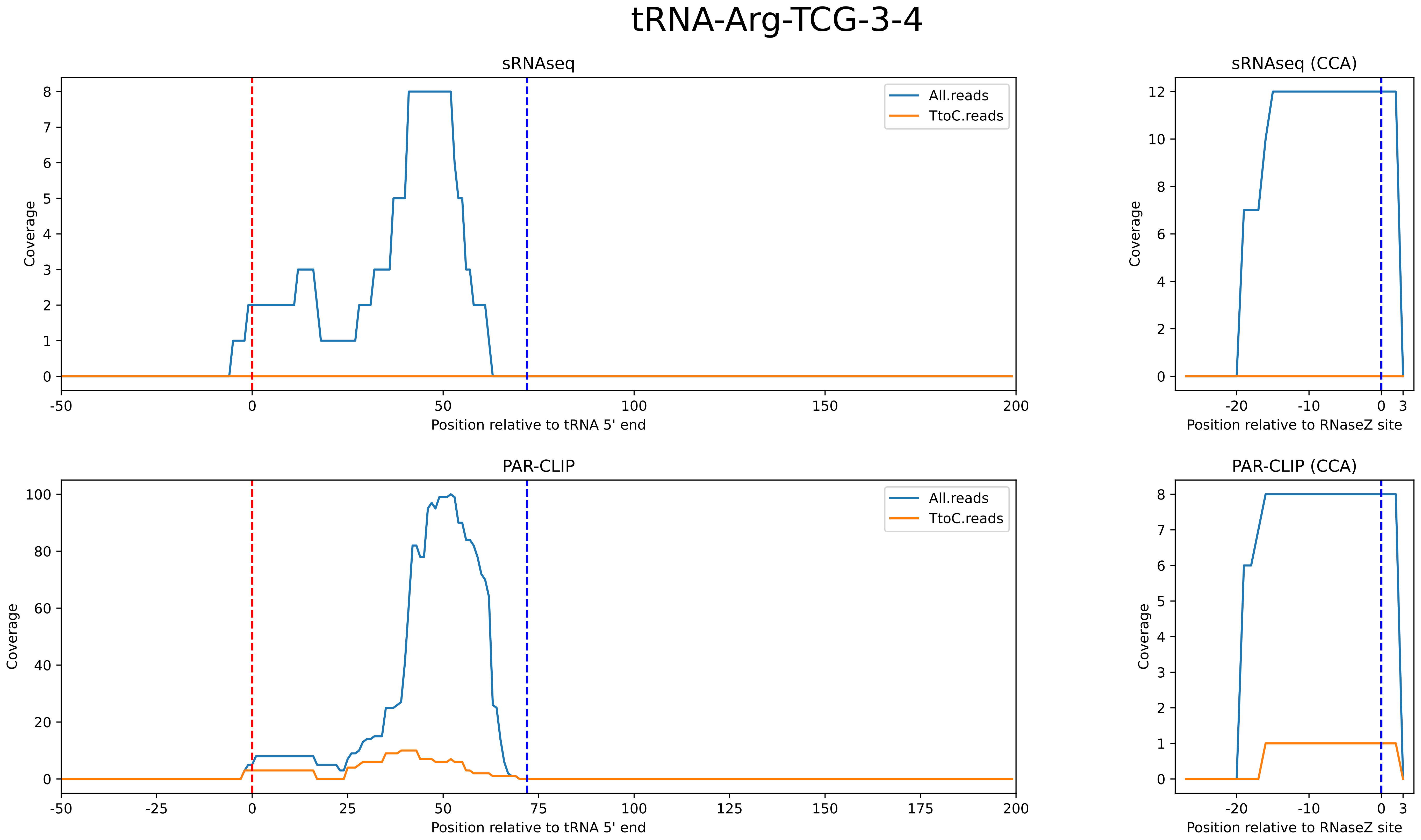
tRNA-Arg-TCG-3-4
sRNAseq
All.reads
TtoC.reads
Coverage
Position relative to tRNA 5' end
sRNAseq (CCA)
Position relative to RNaseZ site
PAR-CLIP
PAR-CLIP (CCA)

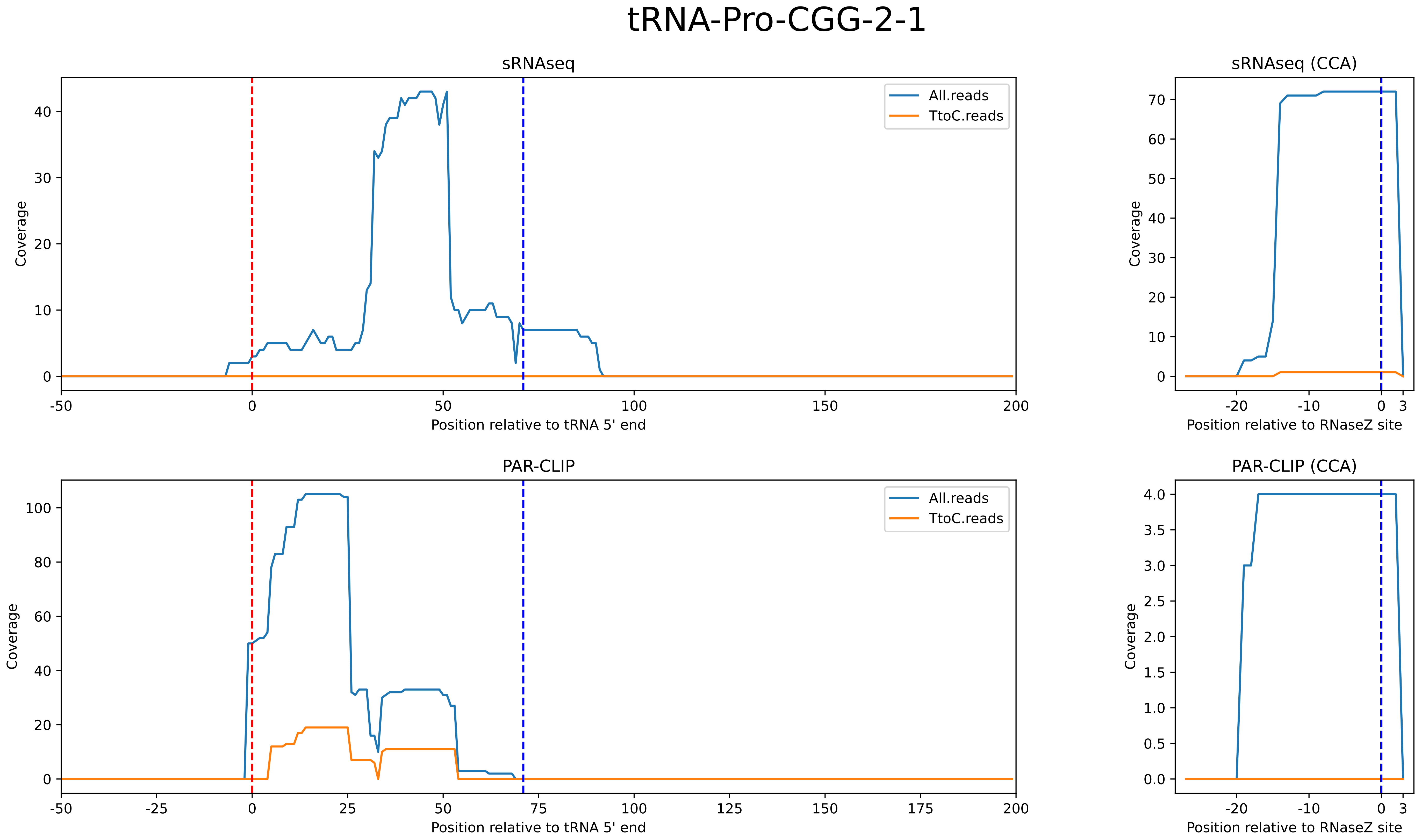
tRNA-Pro-CGG-2-1
sRNAseq
sRNAseq (CCA)
PAR-CLIP
PAR-CLIP (CCA)
All.reads
TtoC.reads
Coverage
Position relative to tRNA 5' end
Position relative to RNaseZ site

# tRNA-Arg-TCG-2-2

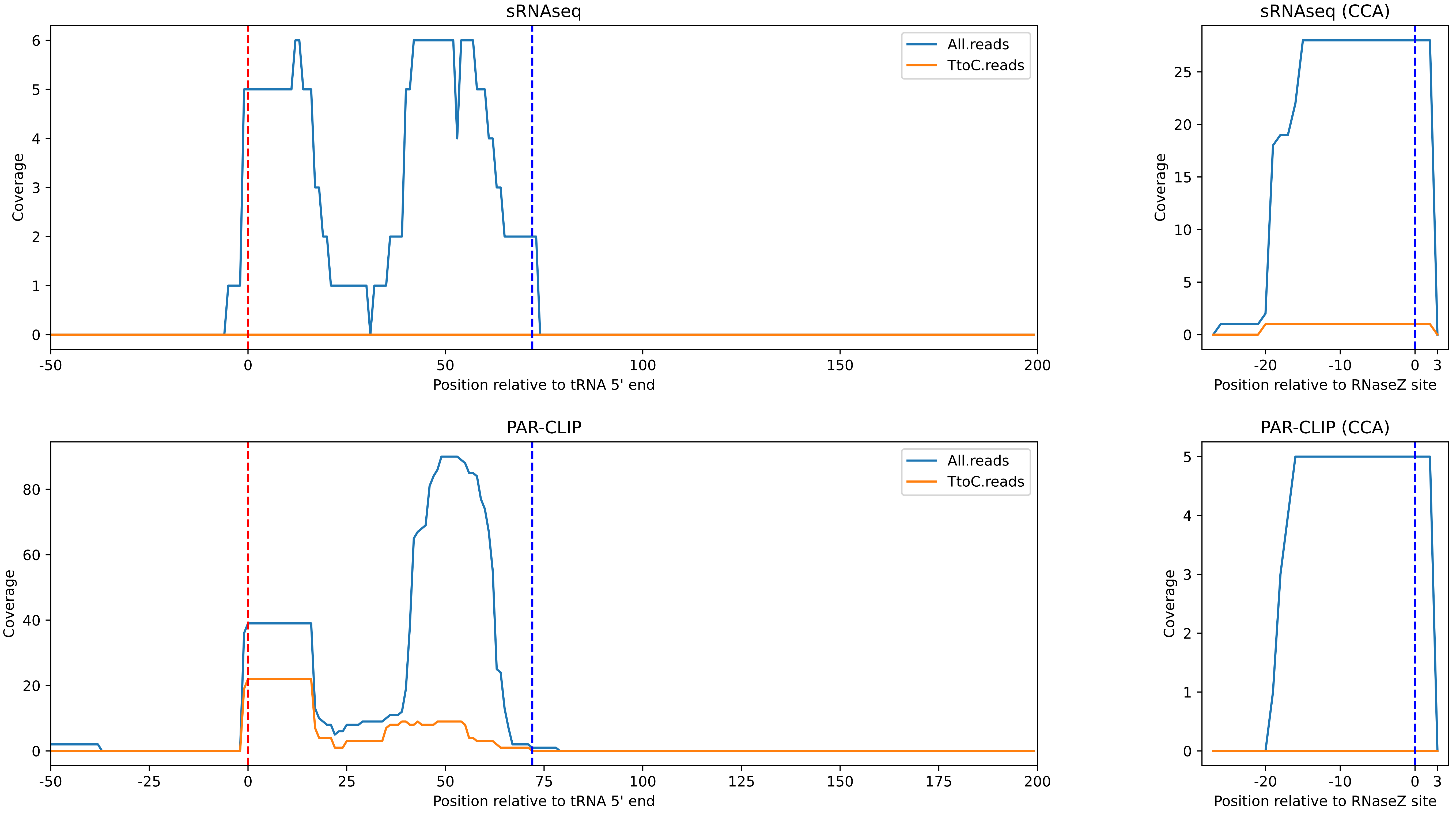

# tRNA-Tyr-GTA-2-1

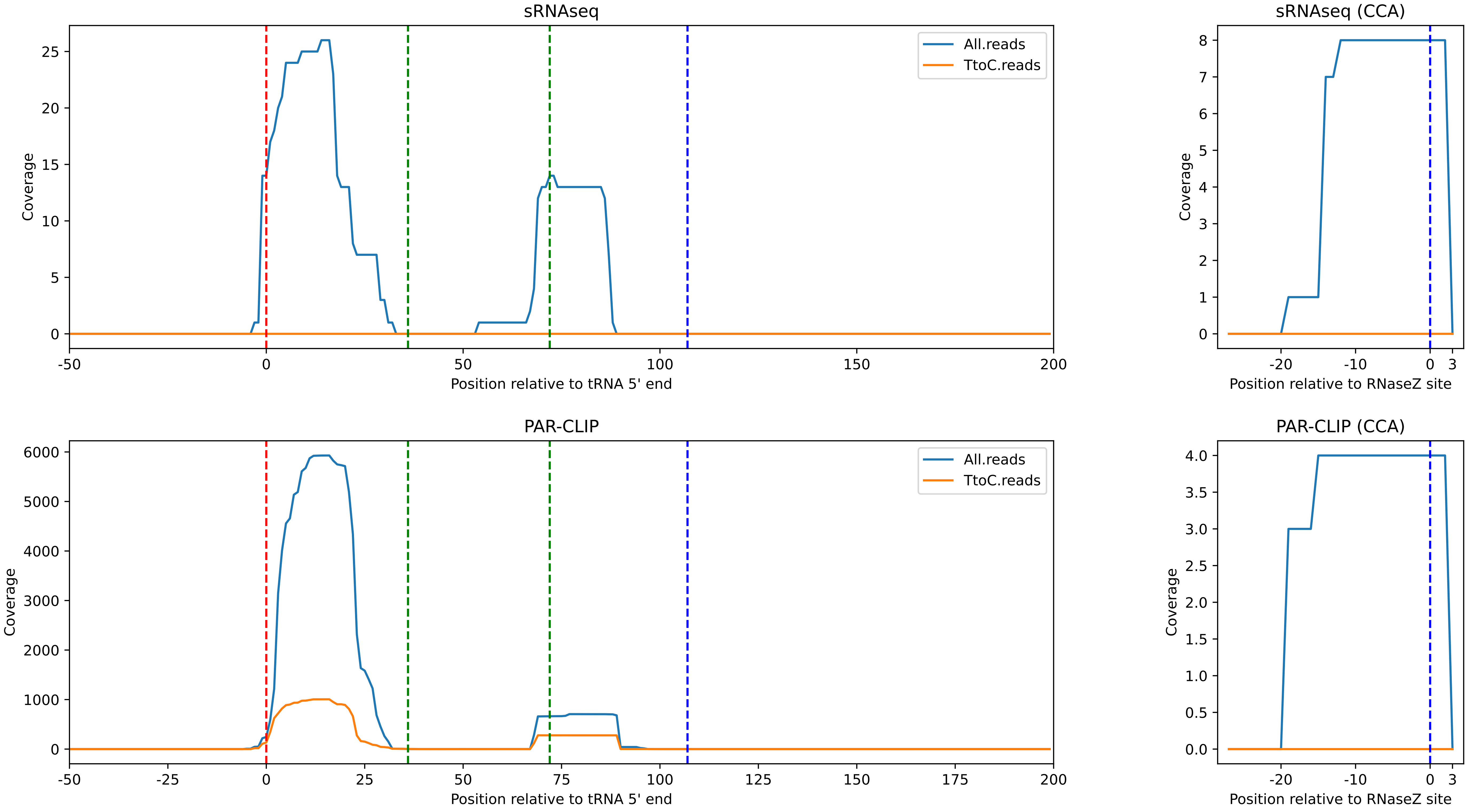

Supplement: Supplementary Material 2 [file mmc3.pdf]
