# Supplementary material for: Exportin-5 binding precedes 5′- and 3′-end processing of tRNA precursors in Drosophila
Source: J Biol Chem. 2024 Aug 2;300(9):107632. doi: 10.1016/j.jbc.2024.107632 (PMC11402290; doi:10.1016/j.jbc.2024.107632)
Supplement: UCSC Tracks [file mmc10.zip › UCSC_tracks/SupplementalDataLegend.docx]

**Supplemental data Legend**

**cluster.bed: PARalyzer cluster bed file**

**TtoC_merged.bw : T-to-C substitution bigwig file**

**D041exdm6.best.mm.forward_for_UCSC.bw & D041exdm6.best.mm.reverse_for_UCSC.bw: PAR-CLIP read density bigwig file**
