# Supplementary material for: Exportin-5 binding precedes 5′- and 3′-end processing of tRNA precursors in Drosophila
Source: J Biol Chem. 2024 Aug 2;300(9):107632. doi: 10.1016/j.jbc.2024.107632 (PMC11402290; doi:10.1016/j.jbc.2024.107632)
Supplement: Supplementary Figure S1 [file mmc11.pdf]

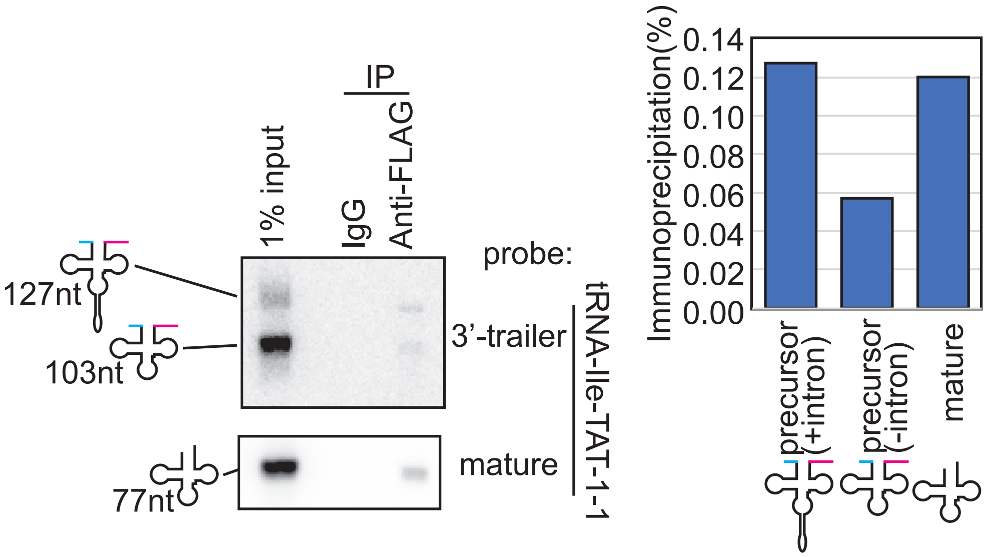

Supplementary Figure S1 Pre- and mature tRNA detection in FLAG-Exp5 immunoprecipitations.

The Exp5 complex was immunoprecipitated by a FLAG-antibody from S2-R+ cells expressing FLAG-tagged Exp5, and RNA was extracted from the input and IP samples. RNA was separated on 10% Acrylamide gel, and bands detected by the indicated probes. The relative band intensity in the IP lane compared to that in the input lane was used to calculate the IP percentage.
